# Supplementary material for: DMRforPairs: identifying Differentially Methylated Regions between unique samples using array based methylation profiles
Source: BMC Bioinformatics. 2014 May 15;15:141. doi: 10.1186/1471-2105-15-141 (PMC4046028; doi:10.1186/1471-2105-15-141)
Supplement: Additional file 2 — DMRforPairs output for the comparison of A431-MCF7 and NA17018-NA17105. Please start from the HTML files in each folder. Available via the BMC Bioinformatics website. [file 1471-2105-15-141-S2.zip › 1394847754114233_MOESM2_ESM/A431_MCF7/relevant.html]

DMRforPairs output generated on Tue Nov 19 17:07:20 2013. Identified regions were set to contain at least 4 probes with a maximum distance of 200 bp between individual probes (n=29404). Regions in which median methylation levels (M-values) between the samples differed at least |1.4| (n=9744) (=relevant) were tested for statistical significance (significant: p<0.05; multiple testing adjusted, n=2626). The folowing samples were studied: A431,MCF7. Chromosomal positions are indicated in bp. N indicates the number of probes in a region. ID indicates the region ID. Values in the columns per sample indicate median beta values. dM indicates the largest median difference (absolute) between any of the sample pairs. p indicates uncorrected p-value from Mann-Whitney U test (n=2) or Kruskal Wallis test (n>2). p.adj denotes the multiple testing corrected p-value (method:fdr). Gene symbols of overlapping transcripts are listed for the exact region and within a margin of 10000 bp of the region.

| Thumbnail | Chr | Start | End | Links | Length | n | ID | Class | A431 | MCF7 | Gene.Symbol | dM | p | p.adj |
| --- | --- | --- | --- | --- | --- | --- | --- | --- | --- | --- | --- | --- | --- | --- |
|  | 16 | 699218 | 699357 | PDFSTATSENSEMBLUCSC | 140 | 5 | 14972 | tss | 0.001474 | 0.989376 | FAM195A; WDR90; AL022341.3 (margin: FAM195A; WDR90; AL022341.1; AL022341.3; LA16c-349E10.1) | 17.96 | 7.94e-03 | 3.19e-02 |
|  | 16 | 699218 | 699500 | PDFSTATSENSEMBLUCSC | 283 | 6 | 15468 | island | 0.003816 | 0.987805 | FAM195A; WDR90; AL022341.3 (margin: FAM195A; WDR90; AL022341.1; AL022341.3; LA16c-349E10.1) | 16.64 | 2.16e-03 | 1.45e-02 |
|  | 4 | 18024261 | 18024445 | PDFSTATSENSEMBLUCSC | 185 | 4 | 30321 | tss;island | 0.211504 | 0.003079 | Not queried | 16.15 | 5.71e-02 | 1.00e-01 |
|  | 9 | 74764263 | 74764504 | PDFSTATSENSEMBLUCSC | 242 | 4 | 40893 | island | 0.001870 | 0.983691 | Not queried | 16.10 | 2.86e-02 | 6.12e-02 |
|  | 7 | 27153636 | 27153944 | PDFSTATSENSEMBLUCSC | 309 | 5 | 37489 | tss | 0.000914 | 0.984478 | HOXA-AS2; HOXA3 (margin: HOXA-AS2; HOXA2; HOXA3) | 15.97 | 7.94e-03 | 3.19e-02 |
|  | 15 | 90455635 | 90455799 | PDFSTATSENSEMBLUCSC | 165 | 4 | 13475 | gene;island | 0.001777 | 0.932153 | Not queried | 15.64 | 2.86e-02 | 6.12e-02 |
|  | 17 | 46682308 | 46682413 | PDFSTATSENSEMBLUCSC | 106 | 6 | 16657 | gene;island | 0.001299 | 0.985588 | HOXB-AS3; HOXB3; HOXB6 (margin: HOXB-AS3; HOXB3; HOXB5; HOXB6; HOXB7; HOXB8) | 15.62 | 2.16e-03 | 1.45e-02 |
|  | 17 | 46682390 | 46682413 | PDFSTATSENSEMBLUCSC | 24 | 4 | 17465 | tss | 0.003780 | 0.986245 | Not queried | 15.62 | 2.86e-02 | 6.12e-02 |
|  | 5 | 156351010 | 156351266 | PDFSTATSENSEMBLUCSC | 257 | 4 | 31872 | gene | 0.004408 | 0.594417 | Not queried | 15.34 | 2.86e-02 | 6.12e-02 |
|  | 21 | 26934197 | 26934455 | PDFSTATSENSEMBLUCSC | 259 | 4 | 26496 | tss | 0.002506 | 0.964432 | Not queried | 15.12 | 2.86e-02 | 6.12e-02 |
|  | 7 | 27153580 | 27153944 | PDFSTATSENSEMBLUCSC | 365 | 7 | 36930 | gene;island | 0.003833 | 0.984256 | HOXA-AS2; HOXA3 (margin: HOXA-AS2; HOXA2; HOXA3) | 15.03 | 5.83e-04 | 6.47e-03 |
|  | 7 | 33080496 | 33080615 | PDFSTATSENSEMBLUCSC | 120 | 4 | 36960 | gene | 0.003264 | 0.984846 | Not queried | 14.99 | 2.86e-02 | 6.12e-02 |
|  | 5 | 139040820 | 139041014 | PDFSTATSENSEMBLUCSC | 195 | 4 | 31778 | gene;island | 0.007190 | 0.987198 | Not queried | 14.57 | 2.86e-02 | 6.12e-02 |
|  | 19 | 52074293 | 52074501 | PDFSTATSENSEMBLUCSC | 209 | 8 | 20998 | tss;island | 0.005935 | 0.984765 | ZNF175 (margin: ZNF175; SIGLEC28P; SIGLEC29P) | 14.38 | 1.55e-04 | 2.71e-03 |
|  | 6 | 26045532 | 26045987 | PDFSTATSENSEMBLUCSC | 456 | 5 | 35512 | island | 0.001452 | 0.939584 | U91328.2; HIST1H3C; HIST1H2BB (margin: U91328.2; HIST1H3C; HIST1H2AB; HIST1H2BB; HIST1H1C) | 14.32 | 7.94e-03 | 3.19e-02 |
|  | 3 | 37902229 | 37902741 | PDFSTATSENSEMBLUCSC | 513 | 5 | 28223 | tss;island | 0.004721 | 0.965672 | CTDSPL; AC093415.2 (margin: CTDSPL; AC093415.2) | 14.32 | 7.94e-03 | 3.19e-02 |
|  | 22 | 21368291 | 21368765 | PDFSTATSENSEMBLUCSC | 475 | 8 | 26989 | tss;island | 0.003739 | 0.979388 | P2RX6; TUBA3FP (margin: THAP7-AS1; P2RX6; THAP7; TUBA3FP; AC002472.1) | 14.29 | 1.55e-04 | 2.71e-03 |
|  | 1 | 77332991 | 77333159 | PDFSTATSENSEMBLUCSC | 169 | 6 | 1703 | tss | 0.977517 | 0.006373 | ST6GALNAC5 (margin: ST6GALNAC5) | 14.24 | 2.16e-03 | 1.45e-02 |
|  | 1 | 90309205 | 90309605 | PDFSTATSENSEMBLUCSC | 401 | 5 | 622 | gene;island | 0.004863 | 0.984824 | RP11-302M6.4; LRRC8D (margin: RP11-302M6.4; LRRC8D) | 14.20 | 7.94e-03 | 3.19e-02 |
|  | 2 | 240196769 | 240197131 | PDFSTATSENSEMBLUCSC | 363 | 4 | 23112 | gene;island | 0.007898 | 0.985732 | Not queried | 14.15 | 2.86e-02 | 6.12e-02 |
|  | 15 | 41061984 | 41062273 | PDFSTATSENSEMBLUCSC | 290 | 6 | 13296 | gene | 0.001681 | 0.963081 | C15orf62; DNAJC17 (margin: RP11-532F12.6; GCHFR; C15orf62; DNAJC17) | 14.11 | 2.16e-03 | 1.45e-02 |
|  | 15 | 41061984 | 41062113 | PDFSTATSENSEMBLUCSC | 130 | 4 | 13595 | tss | 0.001394 | 0.940619 | Not queried | 14.11 | 2.86e-02 | 6.12e-02 |
|  | 6 | 27106988 | 27107756 | PDFSTATSENSEMBLUCSC | 769 | 11 | 33617 | gene;island | 0.007674 | 0.971868 | HIST1H4I; HIST1H2BK (margin: HIST1H2AG; HIST1H4I; HIST1H2AH; MIR3143; HIST1H2BJ; HIST1H2BK) | 13.93 | 2.84e-06 | 1.96e-04 |
|  | 7 | 99156126 | 99156387 | PDFSTATSENSEMBLUCSC | 262 | 4 | 37130 | gene | 0.005571 | 0.975440 | Not queried | 13.88 | 2.86e-02 | 6.12e-02 |
|  | 16 | 31483137 | 31483534 | PDFSTATSENSEMBLUCSC | 398 | 5 | 15212 | tss;island | 0.006349 | 0.981665 | TGFB1I1 (margin: ARMC5; TGFB1I1; SLC5A2) | 13.85 | 7.94e-03 | 3.19e-02 |
|  | 2 | 85640762 | 85641254 | PDFSTATSENSEMBLUCSC | 493 | 6 | 24478 | island | 0.001772 | 0.984150 | CAPG (margin: SH2D6; CAPG) | 13.84 | 2.16e-03 | 1.45e-02 |
|  | 4 | 40632723 | 40632860 | PDFSTATSENSEMBLUCSC | 138 | 4 | 30359 | tss;island | 0.970172 | 0.005905 | Not queried | 13.78 | 2.86e-02 | 6.12e-02 |
|  | 7 | 27195918 | 27196286 | PDFSTATSENSEMBLUCSC | 369 | 4 | 36937 | gene | 0.007221 | 0.985821 | Not queried | 13.70 | 2.86e-02 | 6.12e-02 |
|  | 12 | 6649733 | 6649994 | PDFSTATSENSEMBLUCSC | 262 | 4 | 9029 | gene;island | 0.005606 | 0.974390 | Not queried | 13.69 | 2.86e-02 | 6.12e-02 |
|  | 12 | 89744471 | 89744877 | PDFSTATSENSEMBLUCSC | 407 | 8 | 9238 | gene;island | 0.004694 | 0.967936 | DUSP6 (margin: DUSP6) | 13.69 | 1.55e-04 | 2.71e-03 |
|  | 19 | 48833410 | 48833729 | PDFSTATSENSEMBLUCSC | 320 | 4 | 20121 | gene;island | 0.974646 | 0.004663 | Not queried | 13.68 | 2.86e-02 | 6.12e-02 |
|  | 16 | 57836242 | 57836706 | PDFSTATSENSEMBLUCSC | 465 | 6 | 15969 | island | 0.008506 | 0.979331 | CTD-2600O9.1; KIFC3 (margin: CTD-2600O9.1; KIFC3) | 13.65 | 2.16e-03 | 1.45e-02 |
|  | 2 | 27603611 | 27604131 | PDFSTATSENSEMBLUCSC | 521 | 11 | 23267 | tss | 0.009595 | 0.975245 | ZNF513; PPM1G (margin: SNX17; EIF2B4; ZNF513; PPM1G; FTH1P3) | 13.63 | 2.84e-06 | 1.96e-04 |
|  | 10 | 17270300 | 17270601 | PDFSTATSENSEMBLUCSC | 302 | 5 | 4517 | gene | 0.007815 | 0.989157 | VIM; VIM-AS1 (margin: VIM; VIM-AS1; RP11-124N14.3) | 13.61 | 7.94e-03 | 3.19e-02 |
|  | 1 | 24307153 | 24307535 | PDFSTATSENSEMBLUCSC | 383 | 5 | 1354 | tss;island | 0.001968 | 0.971472 | SRSF10 (margin: SRSF10) | 13.60 | 7.94e-03 | 3.19e-02 |
|  | 2 | 105470558 | 105470794 | PDFSTATSENSEMBLUCSC | 237 | 4 | 23550 | tss;island | 0.933530 | 0.002311 | Not queried | 13.60 | 2.86e-02 | 6.12e-02 |
|  | 1 | 207224510 | 207224740 | PDFSTATSENSEMBLUCSC | 231 | 4 | 2217 | tss | 0.001017 | 0.927974 | Not queried | 13.58 | 2.86e-02 | 6.12e-02 |
|  | 4 | 40518024 | 40518143 | PDFSTATSENSEMBLUCSC | 120 | 4 | 30358 | tss | 0.010016 | 0.968447 | Not queried | 13.57 | 2.86e-02 | 6.12e-02 |
|  | 5 | 134366831 | 134367099 | PDFSTATSENSEMBLUCSC | 269 | 4 | 31757 | gene;island | 0.005708 | 0.985115 | Not queried | 13.44 | 2.86e-02 | 6.12e-02 |
|  | 4 | 40517938 | 40518143 | PDFSTATSENSEMBLUCSC | 206 | 5 | 29987 | gene | 0.011117 | 0.973169 | RBM47 (margin: RBM47) | 13.42 | 7.94e-03 | 3.19e-02 |
|  | 11 | 33037397 | 33037626 | PDFSTATSENSEMBLUCSC | 230 | 5 | 7230 | tss | 0.009358 | 0.989353 | DEPDC7 (margin: DEPDC7; Y\_RNA) | 13.35 | 7.94e-03 | 3.19e-02 |
|  | 2 | 29337946 | 29338258 | PDFSTATSENSEMBLUCSC | 313 | 9 | 23282 | tss | 0.005635 | 0.988847 | CLIP4 (margin: CLIP4) | 13.32 | 4.11e-05 | 1.17e-03 |
|  | 5 | 912383 | 912860 | PDFSTATSENSEMBLUCSC | 478 | 6 | 31516 | gene;island | 0.008493 | 0.984922 | TRIP13 (margin: TRIP13) | 13.29 | 2.16e-03 | 1.45e-02 |
|  | 6 | 106546704 | 106546754 | PDFSTATSENSEMBLUCSC | 51 | 4 | 35148 | tss | 0.002773 | 0.956460 | Not queried | 13.29 | 2.86e-02 | 6.12e-02 |
|  | 2 | 29337946 | 29338432 | PDFSTATSENSEMBLUCSC | 487 | 10 | 24198 | island | 0.008064 | 0.989186 | CLIP4 (margin: CLIP4) | 13.26 | 1.08e-05 | 4.82e-04 |
|  | 11 | 121986961 | 121987203 | PDFSTATSENSEMBLUCSC | 243 | 7 | 6948 | gene;tss | 0.981970 | 0.012363 | RP11-166D19.1; BLID (margin: RP11-166D19.1; BLID) | 13.05 | 5.83e-04 | 6.47e-03 |
|  | 7 | 27195918 | 27196555 | PDFSTATSENSEMBLUCSC | 638 | 11 | 38353 | island | 0.005620 | 0.979773 | HOXA-AS3; RP1-170O19.21; HOXA7 (margin: HOXA-AS3; RP1-170O19.21; HOXA-AS4; HOXA3; HOXA6; HOXA7; HOXA9; RP1-170O19.20) | 13.03 | 2.84e-06 | 1.96e-04 |
|  | 7 | 27196296 | 27196555 | PDFSTATSENSEMBLUCSC | 260 | 7 | 37494 | tss | 0.005620 | 0.979246 | HOXA-AS3; RP1-170O19.21; HOXA7 (margin: HOXA-AS3; RP1-170O19.21; HOXA-AS4; HOXA3; HOXA6; HOXA7; HOXA9; RP1-170O19.20) | 13.03 | 5.83e-04 | 6.47e-03 |
|  | 18 | 44526430 | 44526756 | PDFSTATSENSEMBLUCSC | 327 | 6 | 19260 | tss | 0.960910 | 0.002831 | KATNAL2 (margin: KATNAL2) | 13.03 | 2.16e-03 | 1.45e-02 |
|  | 13 | 88323266 | 88323607 | PDFSTATSENSEMBLUCSC | 342 | 5 | 11807 | island | 0.284860 | 0.002756 | SLITRK5; MIR4500HG (margin: SLITRK5; MIR4500HG) | 12.96 | 7.94e-03 | 3.19e-02 |
|  | 16 | 56659336 | 56659724 | PDFSTATSENSEMBLUCSC | 389 | 7 | 15949 | island | 0.007345 | 0.970085 | MT1E (margin: MT1L; MT1E; MT1M; MT1JP; AC026461.1) | 12.93 | 5.83e-04 | 6.47e-03 |
|  | 12 | 21809973 | 21810762 | PDFSTATSENSEMBLUCSC | 790 | 12 | 9080 | gene | 0.007273 | 0.976024 | LDHB (margin: RP11-59N23.3; LDHB) | 12.92 | 7.40e-07 | 7.21e-05 |
|  | 21 | 26934197 | 26934682 | PDFSTATSENSEMBLUCSC | 486 | 6 | 26615 | island | 0.005765 | 0.975697 | MIR155HG (margin: MIR155HG) | 12.87 | 2.16e-03 | 1.45e-02 |
|  | 7 | 27154262 | 27155039 | PDFSTATSENSEMBLUCSC | 778 | 14 | 37490 | tss | 0.003101 | 0.969552 | HOXA-AS2; HOXA3 (margin: HOXA-AS2; HOXA2; HOXA3) | 12.86 | 4.99e-08 | 9.34e-06 |
|  | 1 | 77332991 | 77333229 | PDFSTATSENSEMBLUCSC | 239 | 8 | 3393 | island | 0.980715 | 0.008635 | ST6GALNAC5 (margin: ST6GALNAC5) | 12.85 | 1.55e-04 | 2.71e-03 |
|  | 11 | 33037397 | 33037916 | PDFSTATSENSEMBLUCSC | 520 | 7 | 8185 | island | 0.011832 | 0.978505 | DEPDC7 (margin: DEPDC7; Y\_RNA) | 12.80 | 5.83e-04 | 6.47e-03 |
|  | 4 | 176987009 | 176987577 | PDFSTATSENSEMBLUCSC | 569 | 7 | 30186 | gene | 0.740243 | 0.004721 | WDR17 (margin: WDR17) | 12.79 | 5.83e-04 | 6.47e-03 |
|  | 16 | 474271 | 474528 | PDFSTATSENSEMBLUCSC | 258 | 4 | 14967 | tss;island | 0.001397 | 0.923472 | Not queried | 12.79 | 2.86e-02 | 6.12e-02 |
|  | 3 | 12330263 | 12330532 | PDFSTATSENSEMBLUCSC | 270 | 4 | 27672 | gene;island | 0.006339 | 0.942147 | Not queried | 12.76 | 2.86e-02 | 6.12e-02 |
|  | 12 | 51566731 | 51567112 | PDFSTATSENSEMBLUCSC | 382 | 9 | 9712 | tss | 0.011744 | 0.977694 | TFCP2 (margin: RNU6-199P; TFCP2) | 12.65 | 4.11e-05 | 1.17e-03 |
|  | 12 | 30948636 | 30948794 | PDFSTATSENSEMBLUCSC | 159 | 5 | 10308 | island | 0.007858 | 0.980801 | LINC00941 (margin: LINC00941) | 12.65 | 7.94e-03 | 3.19e-02 |
|  | 7 | 92672812 | 92673176 | PDFSTATSENSEMBLUCSC | 365 | 5 | 38665 | island | 0.008107 | 0.960080 | (margin: ) | 12.62 | 7.94e-03 | 3.19e-02 |
|  | 4 | 165877875 | 165878317 | PDFSTATSENSEMBLUCSC | 443 | 8 | 30171 | gene;island | 0.982038 | 0.020190 | RP11-366M4.8; FAM218A; TRIM61 (margin: RP11-366M4.8; FAM218A; TRIM61; RP11-366M4.11) | 12.61 | 1.55e-04 | 2.71e-03 |
|  | 16 | 56659336 | 56659552 | PDFSTATSENSEMBLUCSC | 217 | 4 | 15248 | tss | 0.011753 | 0.975973 | Not queried | 12.53 | 2.86e-02 | 6.12e-02 |
|  | 11 | 33037530 | 33037916 | PDFSTATSENSEMBLUCSC | 387 | 6 | 6572 | gene | 0.013750 | 0.983929 | DEPDC7 (margin: DEPDC7; Y\_RNA) | 12.52 | 2.16e-03 | 1.45e-02 |
|  | 14 | 75593812 | 75594058 | PDFSTATSENSEMBLUCSC | 247 | 9 | 12516 | tss | 0.006636 | 0.982689 | RP11-950C14.7; NEK9 (margin: RP11-950C14.7; NEK9; HIF1AP1; TMED10) | 12.52 | 4.11e-05 | 1.17e-03 |
|  | 6 | 138893673 | 138893718 | PDFSTATSENSEMBLUCSC | 46 | 5 | 35253 | tss | 0.985415 | 0.014239 | NHSL1 (margin: NHSL1) | 12.51 | 7.94e-03 | 3.19e-02 |
|  | 14 | 75593334 | 75594058 | PDFSTATSENSEMBLUCSC | 725 | 14 | 13013 | island | 0.006478 | 0.983746 | RP11-950C14.7; NEK9 (margin: RP11-950C14.7; NEK9; HIF1AP1; TMED10) | 12.50 | 4.99e-08 | 9.34e-06 |
|  | 14 | 75593334 | 75593738 | PDFSTATSENSEMBLUCSC | 405 | 5 | 12144 | gene | 0.005556 | 0.984694 | RP11-950C14.7; NEK9 (margin: RP11-950C14.7; NEK9; HIF1AP1; TMED10) | 12.48 | 7.94e-03 | 3.19e-02 |
|  | 18 | 44526430 | 44527026 | PDFSTATSENSEMBLUCSC | 597 | 8 | 19493 | island | 0.960910 | 0.003157 | KATNAL2 (margin: KATNAL2) | 12.45 | 1.55e-04 | 2.71e-03 |
|  | 6 | 125284203 | 125284359 | PDFSTATSENSEMBLUCSC | 157 | 6 | 35208 | tss;island | 0.010135 | 0.985424 | RNF217; RP11-510H23.1 (margin: RNF217; RP11-510H23.1) | 12.43 | 2.16e-03 | 1.45e-02 |
|  | 2 | 172378774 | 172379087 | PDFSTATSENSEMBLUCSC | 314 | 4 | 24854 | island | 0.010130 | 0.963241 | Not queried | 12.43 | 2.86e-02 | 6.12e-02 |
|  | 6 | 710835 | 711405 | PDFSTATSENSEMBLUCSC | 571 | 4 | 35364 | island | 0.010388 | 0.982338 | Not queried | 12.42 | 2.86e-02 | 6.12e-02 |
|  | 20 | 57581903 | 57582292 | PDFSTATSENSEMBLUCSC | 390 | 6 | 25478 | gene | 0.008945 | 0.982509 | CTSZ (margin: NELFCD; CTSZ) | 12.41 | 2.16e-03 | 1.45e-02 |
|  | 19 | 9649241 | 9649542 | PDFSTATSENSEMBLUCSC | 302 | 9 | 21500 | island | 0.004427 | 0.965967 | ZNF426 (margin: ZNF426) | 12.39 | 4.11e-05 | 1.17e-03 |
|  | 19 | 9649310 | 9649542 | PDFSTATSENSEMBLUCSC | 233 | 7 | 20426 | tss | 0.008980 | 0.965967 | ZNF426 (margin: ZNF426) | 12.38 | 5.83e-04 | 6.47e-03 |
|  | 14 | 69256677 | 69256977 | PDFSTATSENSEMBLUCSC | 301 | 5 | 12121 | gene;island | 0.010901 | 0.978651 | ZFP36L1 (margin: ZFP36L1) | 12.31 | 7.94e-03 | 3.19e-02 |
|  | 6 | 30652202 | 30652399 | PDFSTATSENSEMBLUCSC | 198 | 9 | 33802 | gene;island | 0.019670 | 0.982218 | PPP1R18 (margin: DHX16; PPP1R18; NRM) | 12.26 | 4.11e-05 | 1.17e-03 |
|  | 3 | 15311021 | 15311269 | PDFSTATSENSEMBLUCSC | 249 | 4 | 27690 | gene | 0.005477 | 0.962556 | Not queried | 12.24 | 2.86e-02 | 6.12e-02 |
|  | 3 | 49395923 | 49395991 | PDFSTATSENSEMBLUCSC | 69 | 4 | 28327 | tss | 0.008492 | 0.971158 | Not queried | 12.21 | 2.86e-02 | 6.12e-02 |
|  | 10 | 3149673 | 3149852 | PDFSTATSENSEMBLUCSC | 180 | 4 | 4472 | gene;island | 0.005934 | 0.983179 | Not queried | 12.21 | 2.86e-02 | 6.12e-02 |
|  | 19 | 50979561 | 50979637 | PDFSTATSENSEMBLUCSC | 77 | 4 | 20979 | tss | 0.010242 | 0.975583 | Not queried | 12.15 | 2.86e-02 | 6.12e-02 |
|  | 1 | 25566331 | 25566753 | PDFSTATSENSEMBLUCSC | 423 | 5 | 2868 | island | 0.002736 | 0.954348 | C1orf63 (margin: RP3-465N24.6; SYF2; C1orf63) | 12.14 | 7.94e-03 | 3.19e-02 |
|  | 10 | 3824387 | 3824687 | PDFSTATSENSEMBLUCSC | 301 | 4 | 4476 | gene;island | 0.004917 | 0.981779 | Not queried | 12.13 | 2.86e-02 | 6.12e-02 |
|  | 12 | 81331457 | 81331660 | PDFSTATSENSEMBLUCSC | 204 | 4 | 9227 | gene | 0.980413 | 0.012335 | Not queried | 12.12 | 2.86e-02 | 6.12e-02 |
|  | 1 | 207224090 | 207224740 | PDFSTATSENSEMBLUCSC | 651 | 9 | 4146 | island | 0.004897 | 0.954783 | PFKFB2; YOD1 (margin: PFKFB2; snoU13; YOD1) | 12.12 | 4.11e-05 | 1.17e-03 |
|  | 5 | 38846475 | 38846743 | PDFSTATSENSEMBLUCSC | 269 | 4 | 31597 | gene;island | 0.001315 | 0.730800 | Not queried | 12.05 | 2.86e-02 | 6.12e-02 |
|  | 2 | 27603547 | 27604131 | PDFSTATSENSEMBLUCSC | 585 | 12 | 24181 | island | 0.013656 | 0.973814 | ZNF513; PPM1G (margin: SNX17; EIF2B4; ZNF513; PPM1G; FTH1P3) | 12.04 | 7.40e-07 | 7.21e-05 |
|  | 13 | 49549440 | 49549665 | PDFSTATSENSEMBLUCSC | 226 | 4 | 11437 | tss;island | 0.545433 | 0.000725 | Not queried | 12.01 | 2.86e-02 | 6.12e-02 |
|  | 7 | 92466078 | 92466386 | PDFSTATSENSEMBLUCSC | 309 | 6 | 37711 | tss;island | 0.007206 | 0.964979 | AC002454.1; CDK6 (margin: AC002454.1; CDK6) | 11.98 | 2.16e-03 | 1.45e-02 |
|  | 17 | 79881468 | 79881543 | PDFSTATSENSEMBLUCSC | 76 | 5 | 16929 | gene;tss;island | 0.009827 | 0.987489 | RP11-498C9.12; MAFG (margin: RP11-498C9.12; MAFG-AS1; RP11-498C9.13; SIRT7; MAFG; PYCR1) | 11.97 | 7.94e-03 | 3.19e-02 |
|  | 19 | 1155030 | 1155225 | PDFSTATSENSEMBLUCSC | 196 | 4 | 19647 | gene;island | 0.005177 | 0.950577 | Not queried | 11.95 | 2.86e-02 | 6.12e-02 |
|  | 2 | 79220029 | 79220528 | PDFSTATSENSEMBLUCSC | 500 | 5 | 24460 | island | 0.936069 | 0.011013 | (margin: ) | 11.95 | 7.94e-03 | 3.19e-02 |
|  | 5 | 102201421 | 102201601 | PDFSTATSENSEMBLUCSC | 181 | 4 | 32989 | island | 0.004214 | 0.844028 | Not queried | 11.94 | 2.86e-02 | 6.12e-02 |
|  | 11 | 22850866 | 22850970 | PDFSTATSENSEMBLUCSC | 105 | 4 | 6540 | gene;island | 0.948203 | 0.005114 | Not queried | 11.90 | 2.86e-02 | 6.12e-02 |
|  | 8 | 145025059 | 145025178 | PDFSTATSENSEMBLUCSC | 120 | 5 | 39966 | tss | 0.007933 | 0.967393 | PLEC (margin: PLEC; MIR661) | 11.87 | 7.94e-03 | 3.19e-02 |
|  | 22 | 45608428 | 45608713 | PDFSTATSENSEMBLUCSC | 286 | 9 | 27219 | tss | 0.011787 | 0.979508 | KIAA0930 (margin: KIAA0930; MIR1249) | 11.85 | 4.11e-05 | 1.17e-03 |
|  | 10 | 91405265 | 91405300 | PDFSTATSENSEMBLUCSC | 36 | 4 | 5216 | tss;island | 0.741338 | 0.007029 | Not queried | 11.83 | 2.86e-02 | 6.12e-02 |
|  | 19 | 18385409 | 18385724 | PDFSTATSENSEMBLUCSC | 316 | 4 | 20615 | tss | 0.002919 | 0.921855 | Not queried | 11.82 | 2.86e-02 | 6.12e-02 |
|  | 11 | 6341842 | 6341908 | PDFSTATSENSEMBLUCSC | 67 | 7 | 7106 | tss | 0.016418 | 0.981032 | PRKCDBP (margin: PRKCDBP) | 11.81 | 5.83e-04 | 6.47e-03 |
|  | 20 | 57581903 | 57582371 | PDFSTATSENSEMBLUCSC | 469 | 7 | 26299 | island | 0.010674 | 0.982445 | CTSZ (margin: NELFCD; TUBB1; CTSZ) | 11.80 | 5.83e-04 | 6.47e-03 |
|  | 11 | 8832111 | 8832283 | PDFSTATSENSEMBLUCSC | 173 | 4 | 6499 | gene | 0.013468 | 0.977635 | Not queried | 11.80 | 2.86e-02 | 6.12e-02 |
|  | 5 | 11904015 | 11904127 | PDFSTATSENSEMBLUCSC | 113 | 6 | 32695 | island | 0.949890 | 0.016061 | CTNND2 (margin: CTNND2) | 11.78 | 2.16e-03 | 1.45e-02 |
|  | 3 | 48507354 | 48507618 | PDFSTATSENSEMBLUCSC | 265 | 6 | 27767 | gene | 0.000825 | 0.796395 | ATRIP; TREX1; SHISA5 (margin: ATRIP; TREX1; SHISA5) | 11.77 | 2.16e-03 | 1.45e-02 |
|  | 5 | 137225191 | 137225509 | PDFSTATSENSEMBLUCSC | 319 | 5 | 31763 | gene;island | 0.002456 | 0.960502 | MYOT; PKD2L2; RP11-381K20.2 (margin: MYOT; PKD2L2; RP11-381K20.2) | 11.76 | 7.94e-03 | 3.19e-02 |
|  | 22 | 36236649 | 36236760 | PDFSTATSENSEMBLUCSC | 112 | 4 | 26854 | gene;tss | 0.013271 | 0.769061 | Not queried | 11.76 | 2.86e-02 | 6.12e-02 |
|  | 3 | 50378407 | 50378664 | PDFSTATSENSEMBLUCSC | 258 | 9 | 28353 | tss | 0.010074 | 0.974616 | ZMYND10-AS1; RASSF1; ZMYND10 (margin: ZMYND10-AS1; CYB561D2; XXcos-LUCA11.5; RASSF1; ZMYND10; NPRL2) | 11.73 | 4.11e-05 | 1.17e-03 |
|  | 7 | 27154262 | 27155548 | PDFSTATSENSEMBLUCSC | 1287 | 18 | 36931 | gene;island | 0.005790 | 0.969552 | HOXA-AS2; HOXA3 (margin: HOXA-AS2; HOXA2; HOXA3) | 11.71 | 2.20e-10 | 1.16e-07 |
|  | 5 | 119799822 | 119799864 | PDFSTATSENSEMBLUCSC | 43 | 4 | 32240 | tss;island | 0.817131 | 0.003775 | Not queried | 11.71 | 2.86e-02 | 6.12e-02 |
|  | 15 | 79382548 | 79383167 | PDFSTATSENSEMBLUCSC | 620 | 7 | 13447 | gene;island | 0.944524 | 0.009283 | RASGRF1 (margin: RASGRF1) | 11.70 | 5.83e-04 | 6.47e-03 |
|  | 17 | 8056950 | 8057058 | PDFSTATSENSEMBLUCSC | 109 | 4 | 17110 | tss;island | 0.003997 | 0.866107 | Not queried | 11.67 | 2.86e-02 | 6.12e-02 |
|  | 7 | 24796981 | 24797486 | PDFSTATSENSEMBLUCSC | 506 | 5 | 36922 | gene | 0.008012 | 0.951322 | DFNA5 (margin: DFNA5) | 11.64 | 7.94e-03 | 3.19e-02 |
|  | 8 | 143781297 | 143781406 | PDFSTATSENSEMBLUCSC | 110 | 4 | 39933 | tss;island | 0.002420 | 0.794309 | Not queried | 11.64 | 2.86e-02 | 6.12e-02 |
|  | 2 | 176994142 | 176994448 | PDFSTATSENSEMBLUCSC | 307 | 5 | 23739 | tss | 0.012218 | 0.970804 | HOXD8; HOXD-AS2 (margin: HOXD10; HOXD9; HOXD8; HOXD3; HOXD-AS2) | 11.62 | 7.94e-03 | 3.19e-02 |
|  | 12 | 51566379 | 51567112 | PDFSTATSENSEMBLUCSC | 734 | 11 | 10422 | island | 0.011744 | 0.973162 | TFCP2 (margin: RNU6-199P; TFCP2) | 11.62 | 2.84e-06 | 1.96e-04 |
|  | 1 | 150254280 | 150254546 | PDFSTATSENSEMBLUCSC | 267 | 5 | 1905 | tss;island | 0.879954 | 0.005045 | C1orf54; C1orf51 (margin: C1orf54; C1orf51; MRPS21) | 11.61 | 7.94e-03 | 3.19e-02 |
|  | 6 | 28979210 | 28979498 | PDFSTATSENSEMBLUCSC | 289 | 12 | 35608 | island | 0.018409 | 0.977578 | (margin: ZNF311) | 11.61 | 7.40e-07 | 7.21e-05 |
|  | 19 | 37997675 | 37997831 | PDFSTATSENSEMBLUCSC | 157 | 8 | 20740 | tss | 0.899233 | 0.002090 | ZNF793; CTD-3064H18.1 (margin: ZNF793; CTD-3064H18.1; CTD-3064H18.2) | 11.60 | 1.55e-04 | 2.71e-03 |
|  | 6 | 134213992 | 134214307 | PDFSTATSENSEMBLUCSC | 316 | 4 | 34480 | gene;island | 0.967153 | 0.007315 | Not queried | 11.58 | 2.86e-02 | 6.12e-02 |
|  | 11 | 796361 | 796607 | PDFSTATSENSEMBLUCSC | 247 | 5 | 7028 | tss;island | 0.007354 | 0.958939 | SLC25A22 (margin: CEND1; SLC25A22; PIDD) | 11.58 | 7.94e-03 | 3.19e-02 |
|  | 11 | 6341717 | 6341908 | PDFSTATSENSEMBLUCSC | 192 | 8 | 8013 | island | 0.018490 | 0.980499 | PRKCDBP (margin: PRKCDBP) | 11.58 | 1.55e-04 | 2.71e-03 |
|  | 2 | 178257176 | 178257923 | PDFSTATSENSEMBLUCSC | 748 | 14 | 22943 | gene;tss;island | 0.011288 | 0.953897 | AGPS; NFE2L2; AC074286.1 (margin: AGPS; NFE2L2; AC074286.1) | 11.57 | 4.99e-08 | 9.34e-06 |
|  | 7 | 25219543 | 25220162 | PDFSTATSENSEMBLUCSC | 620 | 12 | 38322 | island | 0.011678 | 0.965690 | C7orf31 (margin: AC004129.7; C7orf31) | 11.56 | 7.40e-07 | 7.21e-05 |
|  | 19 | 41129881 | 41130041 | PDFSTATSENSEMBLUCSC | 161 | 4 | 20048 | gene;island | 0.965347 | 0.012452 | Not queried | 11.55 | 2.86e-02 | 6.12e-02 |
|  | 2 | 119605210 | 119605441 | PDFSTATSENSEMBLUCSC | 232 | 4 | 22842 | gene;island | 0.037500 | 0.975718 | Not queried | 11.55 | 2.86e-02 | 6.12e-02 |
|  | 1 | 38461540 | 38461896 | PDFSTATSENSEMBLUCSC | 357 | 4 | 3081 | island | 0.015567 | 0.976182 | Not queried | 11.55 | 2.86e-02 | 6.12e-02 |
|  | 22 | 50968250 | 50968343 | PDFSTATSENSEMBLUCSC | 94 | 5 | 26931 | gene | 0.012995 | 0.976905 | TYMP; ODF3B (margin: NCAPH2; SCO2; TYMP; ODF3B) | 11.54 | 7.94e-03 | 3.19e-02 |
|  | 22 | 50968297 | 50968516 | PDFSTATSENSEMBLUCSC | 220 | 5 | 27258 | tss | 0.012080 | 0.976905 | TYMP; ODF3B (margin: NCAPH2; SCO2; TYMP; ODF3B) | 11.54 | 7.94e-03 | 3.19e-02 |
|  | 6 | 105584551 | 105584780 | PDFSTATSENSEMBLUCSC | 230 | 5 | 35145 | tss;island | 0.013436 | 0.967087 | BVES-AS1; BVES (margin: BVES-AS1; BVES) | 11.54 | 7.94e-03 | 3.19e-02 |
|  | 3 | 11178593 | 11178758 | PDFSTATSENSEMBLUCSC | 166 | 5 | 28154 | tss | 0.004118 | 0.882237 | HRH1 (margin: HRH1) | 11.53 | 7.94e-03 | 3.19e-02 |
|  | 6 | 26017939 | 26018185 | PDFSTATSENSEMBLUCSC | 247 | 7 | 35504 | island | 0.006947 | 0.980631 | Not queried | 11.53 | 1.75e-02 | 5.79e-02 |
|  | 3 | 3885955 | 3886273 | PDFSTATSENSEMBLUCSC | 319 | 4 | 27640 | gene | 0.013403 | 0.806034 | Not queried | 11.52 | 2.86e-02 | 6.12e-02 |
|  | 19 | 15162515 | 15162971 | PDFSTATSENSEMBLUCSC | 457 | 4 | 20553 | tss | 0.014175 | 0.963997 | Not queried | 11.49 | 2.86e-02 | 6.12e-02 |
|  | 6 | 74160917 | 74161222 | PDFSTATSENSEMBLUCSC | 306 | 4 | 34389 | gene;island | 0.001597 | 0.785273 | Not queried | 11.46 | 2.86e-02 | 6.12e-02 |
|  | 22 | 45608345 | 45608713 | PDFSTATSENSEMBLUCSC | 369 | 10 | 26901 | gene | 0.010202 | 0.977688 | KIAA0930 (margin: KIAA0930; MIR1249) | 11.45 | 1.08e-05 | 4.82e-04 |
|  | 3 | 196065289 | 196065688 | PDFSTATSENSEMBLUCSC | 400 | 10 | 28812 | tss | 0.008387 | 0.963775 | TM4SF19 (margin: TM4SF19; RNU6-910P; UBXN7) | 11.45 | 1.08e-05 | 4.82e-04 |
|  | 22 | 50968250 | 50968516 | PDFSTATSENSEMBLUCSC | 267 | 6 | 27629 | island | 0.012538 | 0.975918 | TYMP; ODF3B (margin: NCAPH2; SCO2; TYMP; ODF3B) | 11.45 | 2.16e-03 | 1.45e-02 |
|  | 2 | 183942982 | 183943203 | PDFSTATSENSEMBLUCSC | 222 | 4 | 23769 | tss | 0.028148 | 0.000409 | Not queried | 11.45 | 2.00e-01 | 2.55e-01 |
|  | 14 | 23834860 | 23834995 | PDFSTATSENSEMBLUCSC | 136 | 6 | 12302 | tss | 0.012711 | 0.979647 | EFS (margin: RP11-124D2.3; IL25; CMTM5; EFS) | 11.43 | 2.16e-03 | 1.45e-02 |
|  | 2 | 202122309 | 202122669 | PDFSTATSENSEMBLUCSC | 361 | 6 | 23817 | tss | 0.004098 | 0.903978 | CASP8 (margin: CASP8) | 11.43 | 2.16e-03 | 1.45e-02 |
|  | 17 | 5404330 | 5404581 | PDFSTATSENSEMBLUCSC | 252 | 4 | 17900 | island | 0.943779 | 0.007345 | Not queried | 11.42 | 2.86e-02 | 6.12e-02 |
|  | 1 | 234743527 | 234743830 | PDFSTATSENSEMBLUCSC | 304 | 4 | 1087 | gene;island | 0.012562 | 0.948585 | Not queried | 11.42 | 2.86e-02 | 6.12e-02 |
|  | 7 | 96746798 | 96747063 | PDFSTATSENSEMBLUCSC | 266 | 4 | 37108 | gene;island | 0.011987 | 0.945926 | Not queried | 11.40 | 2.86e-02 | 6.12e-02 |
|  | 4 | 44728642 | 44728857 | PDFSTATSENSEMBLUCSC | 216 | 7 | 30370 | tss | 0.002233 | 0.560186 | GNPDA2 (margin: GNPDA2) | 11.39 | 5.83e-04 | 6.47e-03 |
|  | 2 | 220117599 | 220117945 | PDFSTATSENSEMBLUCSC | 347 | 7 | 23029 | gene;tss;island | 0.010294 | 0.976942 | TUBA4B; TUBA4A (margin: STK16; TUBA4B; GLB1L; TUBA4A) | 11.37 | 5.83e-04 | 6.47e-03 |
|  | 19 | 44302666 | 44303058 | PDFSTATSENSEMBLUCSC | 393 | 4 | 20079 | gene;island | 0.022808 | 0.983030 | Not queried | 11.36 | 2.86e-02 | 6.12e-02 |
|  | 6 | 28367279 | 28367898 | PDFSTATSENSEMBLUCSC | 620 | 9 | 35588 | island | 0.010310 | 0.940185 | ZSCAN12 (margin: RNU2-45P; ZSCAN12) | 11.34 | 4.11e-05 | 1.17e-03 |
|  | 1 | 21616619 | 21617100 | PDFSTATSENSEMBLUCSC | 482 | 5 | 295 | gene;island | 0.012588 | 0.976768 | ECE1 (margin: RP5-1071N3.1; ECE1) | 11.34 | 7.94e-03 | 3.19e-02 |
|  | 21 | 34398085 | 34398265 | PDFSTATSENSEMBLUCSC | 181 | 8 | 26642 | island | 0.938258 | 0.005250 | OLIG2; AP000282.2 (margin: OLIG2; AP000282.2) | 11.33 | 1.55e-04 | 2.71e-03 |
|  | 7 | 48129797 | 48129992 | PDFSTATSENSEMBLUCSC | 196 | 5 | 37006 | gene;island | 0.005170 | 0.925872 | UPP1 (margin: UPP1) | 11.32 | 7.94e-03 | 3.19e-02 |
|  | 4 | 165877875 | 165878091 | PDFSTATSENSEMBLUCSC | 217 | 5 | 30665 | tss | 0.976683 | 0.033206 | RP11-366M4.8; FAM218A; TRIM61 (margin: RP11-366M4.8; FAM218A; TRIM61; RP11-366M4.11) | 11.31 | 7.94e-03 | 3.19e-02 |
|  | 7 | 27209131 | 27209582 | PDFSTATSENSEMBLUCSC | 452 | 10 | 38358 | island | 0.014704 | 0.977453 | HOXA-AS4; HOXA9; RP1-170O19.20; MIR196B; HOXA10 (margin: RP1-170O19.21; HOXA-AS4; HOXA7; HOXA9; RP1-170O19.20; MIR196B; HOXA10; HOXA11) | 11.30 | 1.08e-05 | 4.82e-04 |
|  | 6 | 106546704 | 106546824 | PDFSTATSENSEMBLUCSC | 121 | 5 | 34434 | gene | 0.004456 | 0.965134 | PRDM1; RP1-134E15.3 (margin: PRDM1; RP1-134E15.3) | 11.29 | 7.94e-03 | 3.19e-02 |
|  | 5 | 140207460 | 140207636 | PDFSTATSENSEMBLUCSC | 177 | 5 | 32352 | tss | 0.957192 | 0.013723 | PCDHA1; PCDHA2; PCDHA3; PCDHA4; PCDHA5; PCDHA6 (margin: PCDHA1; PCDHA2; PCDHA3; PCDHA4; PCDHA5; PCDHA6; PCDHA7) | 11.29 | 7.94e-03 | 3.19e-02 |
|  | 21 | 42797847 | 42798386 | PDFSTATSENSEMBLUCSC | 540 | 6 | 26430 | gene;island | 0.018467 | 0.973560 | MX1 (margin: MX1) | 11.28 | 2.16e-03 | 1.45e-02 |
|  | 19 | 37997675 | 37998171 | PDFSTATSENSEMBLUCSC | 497 | 11 | 21957 | island | 0.884806 | 0.002573 | ZNF793; CTD-3064H18.1 (margin: ZNF793; CTD-3064H18.1; CTD-3064H18.2) | 11.28 | 2.84e-06 | 1.96e-04 |
|  | 1 | 209825672 | 209825943 | PDFSTATSENSEMBLUCSC | 272 | 7 | 2230 | tss | 0.004107 | 0.896473 | LAMB3 (margin: LAMB3; RP1-28O10.1) | 11.26 | 5.83e-04 | 6.47e-03 |
|  | 1 | 89664260 | 89664582 | PDFSTATSENSEMBLUCSC | 323 | 6 | 619 | gene | 0.018831 | 0.978538 | GBP4 (margin: GBP4) | 11.26 | 2.16e-03 | 1.45e-02 |
|  | 7 | 44152996 | 44153500 | PDFSTATSENSEMBLUCSC | 505 | 6 | 36990 | gene;island | 0.978531 | 0.016479 | AEBP1; POLD2 (margin: AEBP1; MIR4649; POLD2; RNA5SP230) | 11.24 | 2.16e-03 | 1.45e-02 |
|  | 14 | 27066404 | 27066771 | PDFSTATSENSEMBLUCSC | 368 | 4 | 12052 | gene;island | 0.896330 | 0.006895 | Not queried | 11.24 | 2.86e-02 | 6.12e-02 |
|  | 4 | 163085235 | 163085411 | PDFSTATSENSEMBLUCSC | 177 | 8 | 30658 | tss | 0.954803 | 0.006920 | FSTL5 (margin: FSTL5) | 11.24 | 1.55e-04 | 2.71e-03 |
|  | 20 | 6104215 | 6104274 | PDFSTATSENSEMBLUCSC | 60 | 4 | 25589 | tss | 0.006005 | 0.922701 | Not queried | 11.23 | 2.86e-02 | 6.12e-02 |
|  | 2 | 38892846 | 38892958 | PDFSTATSENSEMBLUCSC | 113 | 4 | 23312 | tss | 0.917546 | 0.006464 | Not queried | 11.23 | 2.86e-02 | 6.12e-02 |
|  | 3 | 192445388 | 192445594 | PDFSTATSENSEMBLUCSC | 207 | 7 | 28792 | tss | 0.916534 | 0.006587 | FGF12; RNU1-20P (margin: FGF12; RNU1-20P) | 11.22 | 5.83e-04 | 6.47e-03 |
|  | 3 | 50378191 | 50378664 | PDFSTATSENSEMBLUCSC | 474 | 11 | 29153 | island | 0.015913 | 0.974616 | ZMYND10-AS1; RASSF1; ZMYND10 (margin: ZMYND10-AS1; CYB561D2; XXcos-LUCA11.5; RASSF1; ZMYND10; NPRL2) | 11.21 | 2.84e-06 | 1.96e-04 |
|  | 18 | 28622474 | 28623159 | PDFSTATSENSEMBLUCSC | 686 | 13 | 19446 | island | 0.013858 | 0.969944 | DSC3 (margin: DSC3) | 11.21 | 1.92e-07 | 2.60e-05 |
|  | 6 | 27840957 | 27841423 | PDFSTATSENSEMBLUCSC | 467 | 11 | 34728 | tss;island | 0.036927 | 0.984328 | HIST1H3I; HIST1H4L (margin: HIST1H2AL; HIST1H2BPS2; HIST1H1B; HIST1H3I; HIST1H4L) | 11.21 | 2.84e-06 | 1.96e-04 |
|  | 6 | 27840957 | 27841230 | PDFSTATSENSEMBLUCSC | 274 | 5 | 33624 | gene | 0.047288 | 0.978076 | HIST1H3I; HIST1H4L (margin: HIST1H2AL; HIST1H2BPS2; HIST1H1B; HIST1H3I; HIST1H4L) | 11.21 | 7.94e-03 | 3.19e-02 |
|  | 15 | 84047906 | 84048308 | PDFSTATSENSEMBLUCSC | 403 | 5 | 14399 | island | 0.007696 | 0.965035 | RP11-382A20.4 (margin: RP11-382A20.4) | 11.19 | 7.94e-03 | 3.19e-02 |
|  | 18 | 28622811 | 28623159 | PDFSTATSENSEMBLUCSC | 349 | 10 | 19220 | tss | 0.015294 | 0.970967 | DSC3 (margin: DSC3) | 11.18 | 1.08e-05 | 4.82e-04 |
|  | 7 | 27209195 | 27209582 | PDFSTATSENSEMBLUCSC | 388 | 9 | 37497 | tss | 0.013288 | 0.977706 | HOXA-AS4; HOXA9; RP1-170O19.20; MIR196B; HOXA10 (margin: RP1-170O19.21; HOXA-AS4; HOXA7; HOXA9; RP1-170O19.20; MIR196B; HOXA10; HOXA11) | 11.18 | 4.11e-05 | 1.17e-03 |
|  | 5 | 140344290 | 140344745 | PDFSTATSENSEMBLUCSC | 456 | 4 | 31805 | gene;tss;island | 0.015587 | 0.973009 | Not queried | 11.15 | 1.14e-01 | 1.68e-01 |
|  | 11 | 74022348 | 74022642 | PDFSTATSENSEMBLUCSC | 295 | 4 | 6827 | gene | 0.696548 | 0.002887 | Not queried | 11.15 | 5.71e-02 | 1.00e-01 |
|  | 2 | 71503484 | 71503883 | PDFSTATSENSEMBLUCSC | 400 | 7 | 24396 | island | 0.788977 | 0.005315 | ZNF638 (margin: ZNF638) | 11.14 | 5.83e-04 | 6.47e-03 |
|  | 19 | 571107 | 571416 | PDFSTATSENSEMBLUCSC | 310 | 5 | 20274 | tss;island | 0.908118 | 0.009494 | BSG; AC009005.2 (margin: BSG; AC009005.2) | 11.11 | 7.94e-03 | 3.19e-02 |
|  | 6 | 28367544 | 28367898 | PDFSTATSENSEMBLUCSC | 355 | 6 | 34741 | tss | 0.013260 | 0.946440 | ZSCAN12 (margin: RNU2-45P; ZSCAN12) | 11.11 | 2.16e-03 | 1.45e-02 |
|  | 5 | 162931471 | 162931829 | PDFSTATSENSEMBLUCSC | 359 | 4 | 31882 | gene;tss;island | 0.004525 | 0.861060 | Not queried | 11.11 | 2.86e-02 | 6.12e-02 |
|  | 17 | 49008322 | 49008925 | PDFSTATSENSEMBLUCSC | 604 | 7 | 18563 | island | 0.018188 | 0.964140 | (margin: RP11-700H6.1) | 11.09 | 5.83e-04 | 6.47e-03 |
|  | 12 | 49393166 | 49393408 | PDFSTATSENSEMBLUCSC | 243 | 7 | 9680 | tss | 0.021603 | 0.984336 | RP11-386G11.5; DDN (margin: RP11-386G11.3; RP11-386G11.5; DDN; PRKAG1) | 11.09 | 5.83e-04 | 6.47e-03 |
|  | 12 | 49393027 | 49393408 | PDFSTATSENSEMBLUCSC | 382 | 8 | 10381 | island | 0.021250 | 0.908442 | RP11-386G11.5; DDN (margin: RP11-386G11.3; RP11-386G11.5; DDN; PRKAG1) | 11.08 | 1.55e-04 | 2.71e-03 |
|  | 17 | 19648846 | 19649293 | PDFSTATSENSEMBLUCSC | 448 | 10 | 17194 | tss | 0.012939 | 0.939134 | ALDH3A1 (margin: RP11-311F12.2; AC005722.4; ALDH3A1) | 11.05 | 1.08e-05 | 4.82e-04 |
|  | 2 | 208795620 | 208795859 | PDFSTATSENSEMBLUCSC | 240 | 4 | 22999 | gene;island | 0.972417 | 0.021811 | Not queried | 11.05 | 2.86e-02 | 6.12e-02 |
|  | 1 | 209825672 | 209825747 | PDFSTATSENSEMBLUCSC | 76 | 4 | 985 | gene | 0.004093 | 0.889062 | Not queried | 11.04 | 2.86e-02 | 6.12e-02 |
|  | 11 | 63655748 | 63656090 | PDFSTATSENSEMBLUCSC | 343 | 5 | 6655 | gene | 0.009720 | 0.927391 | MARK2 (margin: MARK2; RNU6-1306P) | 11.04 | 7.94e-03 | 3.19e-02 |
|  | 10 | 118031864 | 118032081 | PDFSTATSENSEMBLUCSC | 218 | 5 | 4773 | gene;tss;island | 0.817448 | 0.011891 | GFRA1 (margin: GFRA1) | 11.03 | 7.94e-03 | 3.19e-02 |
|  | 5 | 140207460 | 140208201 | PDFSTATSENSEMBLUCSC | 742 | 9 | 31797 | gene;island | 0.937397 | 0.013723 | PCDHA1; PCDHA2; PCDHA3; PCDHA4; PCDHA5; PCDHA6 (margin: PCDHA1; PCDHA2; PCDHA3; PCDHA4; PCDHA5; PCDHA6; PCDHA7) | 11.02 | 4.11e-05 | 1.17e-03 |
|  | 5 | 113697330 | 113697649 | PDFSTATSENSEMBLUCSC | 320 | 4 | 32221 | tss;island | 0.807927 | 0.001794 | Not queried | 11.01 | 2.86e-02 | 6.12e-02 |
|  | 8 | 67454546 | 67454892 | PDFSTATSENSEMBLUCSC | 347 | 5 | 40356 | island | 0.963815 | 0.013273 | (margin: ) | 10.99 | 7.94e-03 | 3.19e-02 |
|  | 7 | 87257356 | 87257786 | PDFSTATSENSEMBLUCSC | 431 | 7 | 37073 | gene;island | 0.668913 | 0.003613 | RUNDC3B; ABCB1 (margin: snoU13; RUNDC3B; ABCB1) | 10.99 | 5.83e-04 | 6.47e-03 |
|  | 7 | 87257356 | 87257673 | PDFSTATSENSEMBLUCSC | 318 | 5 | 37687 | tss | 0.858652 | 0.009259 | RUNDC3B; ABCB1 (margin: snoU13; RUNDC3B; ABCB1) | 10.99 | 7.94e-03 | 3.19e-02 |
|  | 19 | 57702917 | 57703364 | PDFSTATSENSEMBLUCSC | 448 | 6 | 20244 | gene | 0.007256 | 0.951395 | ZNF264 (margin: ZNF264) | 10.99 | 2.16e-03 | 1.45e-02 |
|  | 12 | 104609432 | 104609526 | PDFSTATSENSEMBLUCSC | 95 | 4 | 9958 | tss | 0.019891 | 0.965230 | Not queried | 10.98 | 2.86e-02 | 6.12e-02 |
|  | 5 | 179740743 | 179741120 | PDFSTATSENSEMBLUCSC | 378 | 4 | 31942 | gene;island | 0.942633 | 0.009284 | Not queried | 10.94 | 2.86e-02 | 6.12e-02 |
|  | 6 | 152011103 | 152011415 | PDFSTATSENSEMBLUCSC | 313 | 5 | 35302 | tss | 0.900167 | 0.009987 | ESR1 (margin: ESR1) | 10.93 | 7.94e-03 | 3.19e-02 |
|  | 1 | 95006322 | 95006977 | PDFSTATSENSEMBLUCSC | 656 | 6 | 643 | gene;island | 0.002054 | 0.775091 | F3 (margin: F3) | 10.91 | 8.66e-03 | 3.36e-02 |
|  | 6 | 33228341 | 33228498 | PDFSTATSENSEMBLUCSC | 158 | 4 | 34235 | gene | 0.033194 | 0.983867 | Not queried | 10.91 | 2.86e-02 | 6.12e-02 |
|  | 13 | 96296708 | 96297338 | PDFSTATSENSEMBLUCSC | 631 | 12 | 11827 | island | 0.021332 | 0.984614 | DZIP1 (margin: DZIP1; DNAJC3-AS1) | 10.89 | 1.48e-06 | 1.37e-04 |
|  | 13 | 96296979 | 96297338 | PDFSTATSENSEMBLUCSC | 360 | 10 | 11503 | tss | 0.033612 | 0.984614 | DZIP1 (margin: DZIP1; DNAJC3-AS1) | 10.89 | 2.17e-05 | 8.94e-04 |
|  | 19 | 1132256 | 1132537 | PDFSTATSENSEMBLUCSC | 282 | 8 | 19644 | gene | 0.019989 | 0.985408 | SBNO2 (margin: SBNO2) | 10.88 | 1.55e-04 | 2.71e-03 |
|  | 3 | 49395574 | 49395991 | PDFSTATSENSEMBLUCSC | 418 | 8 | 29116 | island | 0.012079 | 0.971158 | GPX1; RHOA (margin: GPX1; RHOA; RHOA-IT1) | 10.87 | 1.55e-04 | 2.71e-03 |
|  | 1 | 235805403 | 235805770 | PDFSTATSENSEMBLUCSC | 368 | 4 | 1091 | gene;island | 0.015000 | 0.962960 | Not queried | 10.87 | 2.86e-02 | 6.12e-02 |
|  | 17 | 4981403 | 4981610 | PDFSTATSENSEMBLUCSC | 208 | 5 | 17040 | tss;island | 0.908852 | 0.006288 | ZFP3; RP11-46I8.3 (margin: ZFP3; RP11-46I8.3) | 10.86 | 7.94e-03 | 3.19e-02 |
|  | 4 | 158141449 | 158141570 | PDFSTATSENSEMBLUCSC | 122 | 5 | 30654 | tss;island | 0.979948 | 0.029928 | GRIA2 (margin: GRIA2) | 10.84 | 7.94e-03 | 3.19e-02 |
|  | 5 | 114937535 | 114938459 | PDFSTATSENSEMBLUCSC | 925 | 11 | 31721 | gene;island | 0.018747 | 0.982651 | AC010226.4; TICAM2; TMED7-TICAM2 (margin: AC010226.4; TICAM2; TMED7-TICAM2; TMED7) | 10.83 | 2.84e-06 | 1.96e-04 |
|  | 12 | 125412894 | 125413164 | PDFSTATSENSEMBLUCSC | 271 | 4 | 10979 | island | 0.013773 | 0.973359 | Not queried | 10.82 | 2.86e-02 | 6.12e-02 |
|  | 7 | 25219840 | 25220162 | PDFSTATSENSEMBLUCSC | 323 | 9 | 37480 | tss | 0.011957 | 0.960595 | C7orf31 (margin: AC004129.7; C7orf31) | 10.82 | 4.11e-05 | 1.17e-03 |
|  | 3 | 48632625 | 48633144 | PDFSTATSENSEMBLUCSC | 520 | 8 | 28301 | tss | 0.011260 | 0.962315 | COL7A1 (margin: COL7A1; UQCRC1) | 10.81 | 1.55e-04 | 2.71e-03 |
|  | 14 | 23834710 | 23834995 | PDFSTATSENSEMBLUCSC | 286 | 8 | 12718 | island | 0.012988 | 0.974690 | EFS (margin: RP11-124D2.3; IL25; CMTM5; EFS) | 10.81 | 6.22e-04 | 6.71e-03 |
|  | 8 | 98290148 | 98290601 | PDFSTATSENSEMBLUCSC | 454 | 9 | 40476 | island | 0.964551 | 0.023812 | TSPYL5 (margin: TSPYL5) | 10.80 | 4.11e-05 | 1.17e-03 |
|  | 3 | 11610138 | 11610338 | PDFSTATSENSEMBLUCSC | 201 | 5 | 27668 | gene;island | 0.009809 | 0.975604 | VGLL4 (margin: ATG7; VGLL4) | 10.77 | 7.94e-03 | 3.19e-02 |
|  | 17 | 19651881 | 19652056 | PDFSTATSENSEMBLUCSC | 176 | 4 | 17195 | tss;island | 0.002403 | 0.658867 | Not queried | 10.76 | 2.86e-02 | 6.12e-02 |
|  | 13 | 67804285 | 67804744 | PDFSTATSENSEMBLUCSC | 460 | 9 | 11769 | island | 0.780391 | 0.004872 | PCDH9 (margin: PCDH9) | 10.76 | 4.11e-05 | 1.17e-03 |
|  | 13 | 67804509 | 67804744 | PDFSTATSENSEMBLUCSC | 236 | 7 | 11471 | tss | 0.794527 | 0.004872 | PCDH9 (margin: PCDH9) | 10.76 | 5.83e-04 | 6.47e-03 |
|  | 15 | 83952722 | 83953068 | PDFSTATSENSEMBLUCSC | 347 | 5 | 13459 | gene;island | 0.025098 | 0.979800 | RP11-382A20.4; BNC1 (margin: RP11-382A20.4; BNC1) | 10.76 | 7.94e-03 | 3.19e-02 |
|  | 1 | 223888869 | 223889141 | PDFSTATSENSEMBLUCSC | 273 | 4 | 2273 | tss | 0.007443 | 0.842060 | Not queried | 10.75 | 2.86e-02 | 6.12e-02 |
|  | 1 | 25256939 | 25257191 | PDFSTATSENSEMBLUCSC | 253 | 6 | 330 | gene;tss;island | 0.030781 | 0.937064 | RUNX3 (margin: RUNX3) | 10.74 | 2.16e-03 | 1.45e-02 |
|  | 2 | 128158438 | 128159058 | PDFSTATSENSEMBLUCSC | 621 | 6 | 24688 | island | 0.022968 | 0.943948 | AC068282.3 (margin: AC068282.3) | 10.74 | 2.16e-03 | 1.45e-02 |
|  | 17 | 19648718 | 19649293 | PDFSTATSENSEMBLUCSC | 576 | 11 | 16484 | gene;island | 0.013915 | 0.950760 | ALDH3A1 (margin: RP11-311F12.2; AC005722.4; ALDH3A1) | 10.72 | 2.84e-06 | 1.96e-04 |
|  | 2 | 131594925 | 131595321 | PDFSTATSENSEMBLUCSC | 397 | 4 | 24724 | island | 0.012951 | 0.954481 | Not queried | 10.71 | 2.86e-02 | 6.12e-02 |
|  | 10 | 6243791 | 6244010 | PDFSTATSENSEMBLUCSC | 220 | 4 | 4479 | gene;tss;island | 0.934695 | 0.008752 | Not queried | 10.70 | 2.86e-02 | 6.12e-02 |
|  | 21 | 34398085 | 34398226 | PDFSTATSENSEMBLUCSC | 142 | 6 | 26516 | tss | 0.791043 | 0.005250 | OLIG2; AP000282.2 (margin: OLIG2; AP000282.2) | 10.68 | 2.16e-03 | 1.45e-02 |
|  | 19 | 50979561 | 50979755 | PDFSTATSENSEMBLUCSC | 195 | 6 | 20177 | gene;island | 0.015864 | 0.965028 | EMC10; FAM71E1 (margin: MYBPC2; EMC10; FAM71E1; CTD-2545M3.2; CTD-2545M3.8) | 10.65 | 2.16e-03 | 1.45e-02 |
|  | 3 | 149374761 | 149375293 | PDFSTATSENSEMBLUCSC | 533 | 7 | 27992 | gene;island | 0.007860 | 0.973288 | WWTR1-AS1; WWTR1 (margin: WWTR1-AS1; WWTR1; WWTR1-IT1) | 10.63 | 5.83e-04 | 6.47e-03 |
|  | 7 | 92237896 | 92238364 | PDFSTATSENSEMBLUCSC | 469 | 6 | 37086 | gene | 0.975002 | 0.019117 | CDK6 (margin: CDK6) | 10.63 | 2.16e-03 | 1.45e-02 |
|  | 6 | 114177329 | 114177535 | PDFSTATSENSEMBLUCSC | 207 | 4 | 35184 | tss;island | 0.050505 | 0.981531 | Not queried | 10.62 | 2.86e-02 | 6.12e-02 |
|  | 19 | 17413767 | 17414399 | PDFSTATSENSEMBLUCSC | 633 | 9 | 21734 | island | 0.014257 | 0.975877 | MRPL34; ABHD8 (margin: MRPL34; DDA1; ABHD8; CTD-2278I10.4) | 10.61 | 4.11e-05 | 1.17e-03 |
|  | 8 | 49835988 | 49836230 | PDFSTATSENSEMBLUCSC | 243 | 4 | 40287 | island | 0.011391 | 0.943230 | Not queried | 10.61 | 2.86e-02 | 6.12e-02 |
|  | 3 | 158390329 | 158390821 | PDFSTATSENSEMBLUCSC | 493 | 10 | 28012 | gene | 0.957981 | 0.016181 | GFM1; LXN (margin: GFM1; LXN) | 10.61 | 1.08e-05 | 4.82e-04 |
|  | 16 | 4588826 | 4589127 | PDFSTATSENSEMBLUCSC | 302 | 7 | 15071 | tss | 0.008563 | 0.977908 | CDIP1 (margin: CDIP1) | 10.61 | 4.08e-03 | 2.47e-02 |
|  | 19 | 17414284 | 17414399 | PDFSTATSENSEMBLUCSC | 116 | 6 | 20585 | tss | 0.012743 | 0.949612 | MRPL34; ABHD8 (margin: MRPL34; DDA1; ABHD8; CTD-2278I10.4) | 10.61 | 2.16e-03 | 1.45e-02 |
|  | 17 | 32906394 | 32906622 | PDFSTATSENSEMBLUCSC | 229 | 4 | 17260 | tss | 0.565381 | 0.002567 | Not queried | 10.60 | 2.86e-02 | 6.12e-02 |
|  | 1 | 32041507 | 32042037 | PDFSTATSENSEMBLUCSC | 531 | 7 | 1448 | tss | 0.009848 | 0.948990 | TINAGL1; RP11-73M7.1 (margin: TINAGL1; RP11-73M7.1) | 10.59 | 5.83e-04 | 6.47e-03 |
|  | 17 | 40834743 | 40835003 | PDFSTATSENSEMBLUCSC | 261 | 4 | 16585 | gene;tss;island | 0.010785 | 0.903911 | Not queried | 10.59 | 2.86e-02 | 6.12e-02 |
|  | 13 | 20392406 | 20392706 | PDFSTATSENSEMBLUCSC | 301 | 5 | 11562 | island | 0.978530 | 0.029956 | ST6GALNAC4P1 (margin: ST6GALNAC4P1; ZMYM5) | 10.58 | 7.94e-03 | 3.19e-02 |
|  | 19 | 1168733 | 1169138 | PDFSTATSENSEMBLUCSC | 406 | 4 | 19648 | gene;island | 0.021771 | 0.931869 | Not queried | 10.58 | 2.86e-02 | 6.12e-02 |
|  | 19 | 40314862 | 40315143 | PDFSTATSENSEMBLUCSC | 282 | 5 | 22005 | island | 0.982789 | 0.050287 | DYRK1B (margin: DYRK1B; FBL) | 10.58 | 7.94e-03 | 3.19e-02 |
|  | 20 | 13976093 | 13976190 | PDFSTATSENSEMBLUCSC | 98 | 7 | 26012 | island | 0.855699 | 0.004673 | MACROD2; SEL1L2 (margin: MACROD2; RPS3P1; SEL1L2) | 10.56 | 5.83e-04 | 6.47e-03 |
|  | 16 | 12996046 | 12996587 | PDFSTATSENSEMBLUCSC | 542 | 6 | 14672 | gene;island | 0.927152 | 0.007908 | Not queried | 10.56 | 1.52e-02 | 5.36e-02 |
|  | 19 | 50832552 | 50832861 | PDFSTATSENSEMBLUCSC | 310 | 6 | 22304 | island | 0.848330 | 0.011421 | NR1H2; KCNC3 (margin: NR1H2; KCNC3; NAPSB) | 10.54 | 2.16e-03 | 1.45e-02 |
|  | 6 | 26018058 | 26018185 | PDFSTATSENSEMBLUCSC | 128 | 5 | 34677 | tss | 0.049410 | 0.981755 | Not queried | 10.52 | 9.52e-02 | 1.53e-01 |
|  | 20 | 48099248 | 48099479 | PDFSTATSENSEMBLUCSC | 232 | 7 | 25808 | tss | 0.898502 | 0.010212 | KCNB1 (margin: KCNB1) | 10.52 | 5.83e-04 | 6.47e-03 |
|  | 11 | 22851383 | 22851591 | PDFSTATSENSEMBLUCSC | 209 | 7 | 7204 | tss | 0.933412 | 0.004292 | RP11-17A1.3; SVIP (margin: RP11-17A1.3; SVIP) | 10.50 | 5.83e-04 | 6.47e-03 |
|  | 7 | 83277941 | 83278138 | PDFSTATSENSEMBLUCSC | 198 | 5 | 37068 | gene | 0.776585 | 0.002733 | SEMA3E (margin: SEMA3E) | 10.49 | 7.94e-03 | 3.19e-02 |
|  | 6 | 96463710 | 96464135 | PDFSTATSENSEMBLUCSC | 426 | 4 | 36333 | island | 0.942053 | 0.018117 | Not queried | 10.45 | 2.86e-02 | 6.12e-02 |
|  | 17 | 4872326 | 4872603 | PDFSTATSENSEMBLUCSC | 278 | 4 | 16361 | gene;tss;island | 0.024161 | 0.961627 | Not queried | 10.45 | 2.86e-02 | 6.12e-02 |
|  | 6 | 26044204 | 26044460 | PDFSTATSENSEMBLUCSC | 257 | 6 | 34683 | tss;island | 0.054543 | 0.985655 | U91328.2; HIST1H3C; HIST1H2BB (margin: U91328.2; HIST1H3C; HIST1H3B; HIST1H2AB; HIST1H2BB; HIST1H1C) | 10.44 | 2.16e-03 | 1.45e-02 |
|  | 3 | 197183271 | 197183662 | PDFSTATSENSEMBLUCSC | 392 | 6 | 29817 | island | 0.025600 | 0.960186 | AC128709.2; AC128709.3; AC128709.4 (margin: AC128709.2; AC128709.3; AC128709.4) | 10.43 | 2.16e-03 | 1.45e-02 |
|  | 12 | 65218069 | 65218413 | PDFSTATSENSEMBLUCSC | 345 | 4 | 10597 | island | 0.811890 | 0.008479 | Not queried | 10.39 | 2.86e-02 | 6.12e-02 |
|  | 17 | 72856049 | 72856181 | PDFSTATSENSEMBLUCSC | 133 | 4 | 17632 | tss | 0.079594 | 0.991046 | Not queried | 10.39 | 2.86e-02 | 6.12e-02 |
|  | 19 | 37407257 | 37407486 | PDFSTATSENSEMBLUCSC | 230 | 6 | 20006 | gene | 0.909618 | 0.006031 | ZNF568; ZNF829 (margin: ZNF345; ZNF568; ZNF829) | 10.39 | 2.16e-03 | 1.45e-02 |
|  | 2 | 85637673 | 85637851 | PDFSTATSENSEMBLUCSC | 179 | 4 | 24477 | island | 0.011856 | 0.914911 | Not queried | 10.39 | 2.86e-02 | 6.12e-02 |
|  | 1 | 51810823 | 51811185 | PDFSTATSENSEMBLUCSC | 363 | 5 | 1622 | tss | 0.030217 | 0.975769 | TTC39A (margin: RP11-275F13.1; TTC39A; EPS15) | 10.38 | 7.94e-03 | 3.19e-02 |
|  | 15 | 39871808 | 39872186 | PDFSTATSENSEMBLUCSC | 379 | 7 | 13576 | tss;island | 0.003617 | 0.770523 | THBS1 (margin: THBS1) | 10.37 | 5.83e-04 | 6.47e-03 |
|  | 8 | 10586678 | 10587055 | PDFSTATSENSEMBLUCSC | 378 | 7 | 39199 | gene;island | 0.011931 | 0.966725 | CTD-2135J3.3; SOX7 (margin: CTD-2135J3.3; SOX7) | 10.36 | 5.83e-04 | 6.47e-03 |
|  | 7 | 12443478 | 12443704 | PDFSTATSENSEMBLUCSC | 227 | 4 | 36895 | gene | 0.794406 | 0.003608 | Not queried | 10.34 | 2.86e-02 | 6.12e-02 |
|  | 3 | 53032638 | 53033167 | PDFSTATSENSEMBLUCSC | 530 | 6 | 27827 | gene;island | 0.029937 | 0.975782 | SFMBT1; RP11-894J14.5 (margin: SFMBT1; RP11-894J14.5) | 10.33 | 2.16e-03 | 1.45e-02 |
|  | 20 | 48099146 | 48099479 | PDFSTATSENSEMBLUCSC | 334 | 8 | 26234 | island | 0.909396 | 0.010603 | KCNB1 (margin: KCNB1) | 10.33 | 2.95e-03 | 1.88e-02 |
|  | 6 | 3752421 | 3752499 | PDFSTATSENSEMBLUCSC | 79 | 4 | 34600 | tss;island | 0.010079 | 0.524484 | Not queried | 10.31 | 2.86e-02 | 6.12e-02 |
|  | 2 | 100938799 | 100939045 | PDFSTATSENSEMBLUCSC | 247 | 4 | 22810 | gene;island | 0.772578 | 0.006100 | Not queried | 10.30 | 2.86e-02 | 6.12e-02 |
|  | 4 | 186456837 | 186457012 | PDFSTATSENSEMBLUCSC | 176 | 5 | 30702 | tss | 0.925963 | 0.011100 | PDLIM3 (margin: PDLIM3) | 10.28 | 7.94e-03 | 3.19e-02 |
|  | 4 | 175750507 | 175751001 | PDFSTATSENSEMBLUCSC | 495 | 10 | 30686 | tss | 0.847449 | 0.013931 | ADAM29; GLRA3 (margin: ADAM29; GLRA3) | 10.27 | 1.08e-05 | 4.82e-04 |
|  | 7 | 87848143 | 87848358 | PDFSTATSENSEMBLUCSC | 216 | 4 | 37076 | gene;island | 0.018973 | 0.950146 | Not queried | 10.27 | 2.86e-02 | 6.12e-02 |
|  | 19 | 10172712 | 10172899 | PDFSTATSENSEMBLUCSC | 188 | 4 | 19806 | gene;island | 0.604505 | 0.009269 | Not queried | 10.26 | 2.86e-02 | 6.12e-02 |
|  | 12 | 21809973 | 21811034 | PDFSTATSENSEMBLUCSC | 1062 | 21 | 10280 | island | 0.014872 | 0.965898 | LDHB (margin: RP11-59N23.3; LDHB) | 10.26 | 3.72e-12 | 5.17e-09 |
|  | 10 | 118032872 | 118033370 | PDFSTATSENSEMBLUCSC | 499 | 11 | 5382 | tss;island | 0.869783 | 0.006086 | GFRA1 (margin: GFRA1) | 10.26 | 2.84e-06 | 1.96e-04 |
|  | 10 | 118032872 | 118033115 | PDFSTATSENSEMBLUCSC | 244 | 7 | 4774 | gene | 0.941245 | 0.006086 | GFRA1 (margin: GFRA1) | 10.26 | 5.83e-04 | 6.47e-03 |
|  | 8 | 57233269 | 57233391 | PDFSTATSENSEMBLUCSC | 123 | 4 | 39713 | tss;island | 0.006008 | 0.680437 | Not queried | 10.24 | 2.86e-02 | 6.12e-02 |
|  | 11 | 22851321 | 22851591 | PDFSTATSENSEMBLUCSC | 271 | 8 | 8137 | island | 0.924659 | 0.009330 | RP11-17A1.3; SVIP (margin: RP11-17A1.3; SVIP) | 10.23 | 1.55e-04 | 2.71e-03 |
|  | 7 | 143582146 | 143582630 | PDFSTATSENSEMBLUCSC | 485 | 4 | 37256 | gene;island | 0.983661 | 0.064683 | Not queried | 10.23 | 2.86e-02 | 6.12e-02 |
|  | 17 | 74868561 | 74868725 | PDFSTATSENSEMBLUCSC | 165 | 4 | 16798 | gene;tss;island | 0.002158 | 0.701748 | Not queried | 10.22 | 2.86e-02 | 6.12e-02 |
|  | 19 | 59066570 | 59066700 | PDFSTATSENSEMBLUCSC | 131 | 7 | 21150 | tss;island | 0.002624 | 0.583028 | CHMP2A; UBE2M (margin: TRIM28; AC016629.8; RN7SL525P; CHMP2A; UBE2M; MZF1) | 10.22 | 5.83e-04 | 6.47e-03 |
|  | 8 | 58907018 | 58907091 | PDFSTATSENSEMBLUCSC | 74 | 4 | 39718 | tss | 0.848680 | 0.013007 | Not queried | 10.18 | 2.86e-02 | 6.12e-02 |
|  | 19 | 1510494 | 1510692 | PDFSTATSENSEMBLUCSC | 199 | 4 | 19657 | gene;island | 0.039922 | 0.988794 | Not queried | 10.16 | 2.86e-02 | 6.12e-02 |
|  | 18 | 32173084 | 32173227 | PDFSTATSENSEMBLUCSC | 144 | 4 | 19085 | gene;tss | 0.145869 | 0.991603 | Not queried | 10.16 | 2.86e-02 | 6.12e-02 |
|  | 2 | 202122309 | 202122832 | PDFSTATSENSEMBLUCSC | 524 | 8 | 22988 | gene | 0.002760 | 0.875523 | CASP8 (margin: CASP8) | 10.16 | 1.55e-04 | 2.71e-03 |
|  | 21 | 46890849 | 46891093 | PDFSTATSENSEMBLUCSC | 245 | 4 | 26469 | gene;island | 0.028392 | 0.974641 | Not queried | 10.16 | 2.86e-02 | 6.12e-02 |
|  | 19 | 3285973 | 3286244 | PDFSTATSENSEMBLUCSC | 272 | 4 | 19713 | gene;island | 0.947193 | 0.043228 | Not queried | 10.14 | 2.86e-02 | 6.12e-02 |
|  | 19 | 18335359 | 18335429 | PDFSTATSENSEMBLUCSC | 71 | 4 | 20614 | tss | 0.663565 | 0.002882 | Not queried | 10.14 | 2.86e-02 | 6.12e-02 |
|  | 17 | 72855943 | 72856181 | PDFSTATSENSEMBLUCSC | 239 | 5 | 18726 | island | 0.045745 | 0.990572 | GRIN2C (margin: GRIN2C; FDXR) | 10.14 | 7.94e-03 | 3.19e-02 |
|  | 11 | 66103823 | 66104174 | PDFSTATSENSEMBLUCSC | 352 | 7 | 8509 | island | 0.004602 | 0.830360 | RP11-867G23.12; RIN1; BRMS1 (margin: RP11-867G23.12; RP11-867G23.8; RIN1; BRMS1; B3GNT1) | 10.13 | 5.83e-04 | 6.47e-03 |
|  | 22 | 38201496 | 38201848 | PDFSTATSENSEMBLUCSC | 353 | 4 | 26864 | gene;island | 0.589294 | 0.005663 | Not queried | 10.13 | 2.86e-02 | 6.12e-02 |
|  | 1 | 85358968 | 85359134 | PDFSTATSENSEMBLUCSC | 167 | 4 | 1717 | tss;island | 0.172726 | 0.979049 | Not queried | 10.13 | 2.86e-02 | 6.12e-02 |
|  | 1 | 85527430 | 85527943 | PDFSTATSENSEMBLUCSC | 514 | 7 | 1721 | tss | 0.008497 | 0.975686 | WDR63 (margin: WDR63) | 10.13 | 5.83e-04 | 6.47e-03 |
|  | 3 | 13921803 | 13921889 | PDFSTATSENSEMBLUCSC | 87 | 4 | 28167 | tss;island | 0.021586 | 0.959795 | Not queried | 10.13 | 2.86e-02 | 6.12e-02 |
|  | 19 | 49223814 | 49224165 | PDFSTATSENSEMBLUCSC | 352 | 5 | 20925 | tss;island | 0.987176 | 0.034829 | MAMSTR; RASIP1 (margin: MAMSTR; RASIP1) | 10.10 | 7.94e-03 | 3.19e-02 |
|  | 8 | 38965122 | 38965492 | PDFSTATSENSEMBLUCSC | 371 | 4 | 39286 | gene | 0.988235 | 0.132704 | Not queried | 10.10 | 2.86e-02 | 6.12e-02 |
|  | 6 | 31025933 | 31026287 | PDFSTATSENSEMBLUCSC | 355 | 6 | 33849 | gene | 0.001543 | 0.145543 | HCG22 (margin: HCG22) | 10.09 | 2.16e-03 | 1.45e-02 |
|  | 11 | 10476494 | 10476662 | PDFSTATSENSEMBLUCSC | 169 | 5 | 6506 | gene;tss;island | 0.009191 | 0.897041 | AMPD3 (margin: AMPD3) | 10.09 | 7.94e-03 | 3.19e-02 |
|  | 8 | 145024637 | 145025178 | PDFSTATSENSEMBLUCSC | 542 | 9 | 39502 | gene;island | 0.018046 | 0.958787 | PLEC (margin: PLEC; MIR661) | 10.08 | 4.11e-05 | 1.17e-03 |
|  | 20 | 13976093 | 13976143 | PDFSTATSENSEMBLUCSC | 51 | 6 | 25605 | tss | 0.876097 | 0.005956 | MACROD2; SEL1L2 (margin: MACROD2; RPS3P1; SEL1L2) | 10.08 | 2.16e-03 | 1.45e-02 |
|  | 7 | 27205159 | 27205658 | PDFSTATSENSEMBLUCSC | 500 | 10 | 37496 | tss | 0.045967 | 0.966025 | HOXA9; RP1-170O19.20 (margin: HOXA-AS3; RP1-170O19.21; HOXA-AS4; HOXA7; HOXA9; RP1-170O19.20; MIR196B; HOXA10) | 10.07 | 1.08e-05 | 4.82e-04 |
|  | 15 | 74537819 | 74538030 | PDFSTATSENSEMBLUCSC | 212 | 4 | 13421 | gene;island | 0.906783 | 0.023738 | Not queried | 10.07 | 2.86e-02 | 6.12e-02 |
|  | 16 | 12897684 | 12897821 | PDFSTATSENSEMBLUCSC | 138 | 4 | 15715 | island | 0.489558 | 0.003028 | Not queried | 10.07 | 2.86e-02 | 6.12e-02 |
|  | 3 | 72704324 | 72704701 | PDFSTATSENSEMBLUCSC | 378 | 5 | 29281 | island | 0.938345 | 0.011033 | (margin: ) | 10.06 | 7.94e-03 | 3.19e-02 |
|  | 1 | 21877524 | 21877781 | PDFSTATSENSEMBLUCSC | 258 | 5 | 298 | gene;tss | 0.973677 | 0.025881 | ALPL (margin: ALPL) | 10.03 | 7.94e-03 | 3.19e-02 |
|  | 11 | 12030088 | 12030914 | PDFSTATSENSEMBLUCSC | 827 | 13 | 6511 | gene | 0.022862 | 0.971522 | DKK3 (margin: DKK3) | 10.03 | 1.92e-07 | 2.60e-05 |
|  | 6 | 121758653 | 121758980 | PDFSTATSENSEMBLUCSC | 328 | 4 | 34460 | gene | 0.100629 | 0.982367 | Not queried | 10.03 | 2.86e-02 | 6.12e-02 |
|  | 13 | 92050675 | 92050843 | PDFSTATSENSEMBLUCSC | 169 | 9 | 11496 | tss | 0.586370 | 0.007782 | GPC5 (margin: GPC5) | 10.00 | 4.11e-05 | 1.17e-03 |
|  | 7 | 25019747 | 25019943 | PDFSTATSENSEMBLUCSC | 197 | 5 | 38320 | island | 0.019260 | 0.958923 | OSBPL3 (margin: OSBPL3) | 9.98 | 7.94e-03 | 3.19e-02 |
|  | 7 | 134143906 | 134144288 | PDFSTATSENSEMBLUCSC | 383 | 11 | 37911 | tss | 0.024920 | 0.982342 | AKR1B1 (margin: AKR1B1) | 9.97 | 2.84e-06 | 1.96e-04 |
|  | 1 | 209605104 | 209605401 | PDFSTATSENSEMBLUCSC | 298 | 4 | 983 | gene;tss | 0.051630 | 0.976314 | Not queried | 9.97 | 2.86e-02 | 6.12e-02 |
|  | 7 | 99155673 | 99156387 | PDFSTATSENSEMBLUCSC | 715 | 13 | 37760 | tss;island | 0.007404 | 0.896113 | ZNF655; FAM200A (margin: ZNF655; FAM200A) | 9.97 | 1.92e-07 | 2.60e-05 |
|  | 1 | 51810337 | 51811185 | PDFSTATSENSEMBLUCSC | 849 | 8 | 3261 | island | 0.029659 | 0.975807 | TTC39A (margin: RP11-275F13.1; TTC39A; EPS15) | 9.97 | 1.55e-04 | 2.71e-03 |
|  | 6 | 132272342 | 132272790 | PDFSTATSENSEMBLUCSC | 449 | 6 | 36481 | island | 0.564627 | 0.006562 | RP11-69I8.3; CTGF (margin: RP11-69I8.3; CTGF) | 9.96 | 2.16e-03 | 1.45e-02 |
|  | 6 | 132272545 | 132272790 | PDFSTATSENSEMBLUCSC | 246 | 4 | 35225 | tss | 0.519628 | 0.006562 | Not queried | 9.96 | 2.86e-02 | 6.12e-02 |
|  | 1 | 228225533 | 228225886 | PDFSTATSENSEMBLUCSC | 354 | 5 | 1045 | gene;island | 0.047340 | 0.978439 | WNT3A (margin: WNT3A) | 9.96 | 7.94e-03 | 3.19e-02 |
|  | 5 | 135364827 | 135365012 | PDFSTATSENSEMBLUCSC | 186 | 4 | 31761 | gene;island | 0.008937 | 0.828079 | Not queried | 9.95 | 2.86e-02 | 6.12e-02 |
|  | 7 | 98739496 | 98739782 | PDFSTATSENSEMBLUCSC | 287 | 4 | 37123 | gene;island | 0.013142 | 0.946736 | Not queried | 9.95 | 2.86e-02 | 6.12e-02 |
|  | 7 | 99156634 | 99157059 | PDFSTATSENSEMBLUCSC | 426 | 4 | 37131 | gene;island | 0.011357 | 0.901108 | Not queried | 9.94 | 2.86e-02 | 6.12e-02 |
|  | 6 | 30070738 | 30071612 | PDFSTATSENSEMBLUCSC | 875 | 27 | 33720 | gene;island | 0.932903 | 0.010832 | TRIM31-AS1; TRIM31 (margin: TRIM31-AS1; TRIM31) | 9.94 | 1.95e-06 | 1.79e-04 |
|  | 19 | 11998686 | 11999148 | PDFSTATSENSEMBLUCSC | 463 | 7 | 19850 | gene | 0.088674 | 0.956262 | ZNF69 (margin: ZNF439; ZNF69) | 9.92 | 5.83e-04 | 6.47e-03 |
|  | 20 | 6103629 | 6104274 | PDFSTATSENSEMBLUCSC | 646 | 7 | 25992 | island | 0.009234 | 0.898500 | FERMT1 (margin: FERMT1) | 9.92 | 1.11e-02 | 4.14e-02 |
|  | 5 | 140220686 | 140220822 | PDFSTATSENSEMBLUCSC | 137 | 4 | 32354 | tss | 0.899014 | 0.008052 | Not queried | 9.92 | 2.86e-02 | 6.12e-02 |
|  | 12 | 21810791 | 21811034 | PDFSTATSENSEMBLUCSC | 244 | 9 | 9600 | tss | 0.018450 | 0.956870 | LDHB (margin: RP11-59N23.3; LDHB) | 9.90 | 4.11e-05 | 1.17e-03 |
|  | 19 | 52004992 | 52005188 | PDFSTATSENSEMBLUCSC | 197 | 6 | 20996 | tss | 0.009491 | 0.910986 | SIGLEC12 (margin: CEACAM18; SIGLEC27P; SIGLEC12; SIGLEC26P) | 9.89 | 2.16e-03 | 1.45e-02 |
|  | 16 | 4714647 | 4714815 | PDFSTATSENSEMBLUCSC | 169 | 4 | 14642 | gene | 0.093896 | 0.985609 | Not queried | 9.89 | 2.86e-02 | 6.12e-02 |
|  | 8 | 143763326 | 143763565 | PDFSTATSENSEMBLUCSC | 240 | 4 | 39466 | gene | 0.013196 | 0.911042 | Not queried | 9.89 | 2.86e-02 | 6.12e-02 |
|  | 6 | 28778091 | 28778676 | PDFSTATSENSEMBLUCSC | 586 | 9 | 35595 | island | 0.951181 | 0.012313 | (margin: ) | 9.89 | 4.11e-05 | 1.17e-03 |
|  | 16 | 56228442 | 56228901 | PDFSTATSENSEMBLUCSC | 460 | 9 | 15243 | tss | 0.892045 | 0.007110 | Not queried | 9.88 | 5.03e-02 | 9.87e-02 |
|  | 10 | 116164598 | 116164955 | PDFSTATSENSEMBLUCSC | 358 | 6 | 5377 | tss;island | 0.034999 | 0.973863 | AFAP1L2 (margin: AFAP1L2) | 9.86 | 2.16e-03 | 1.45e-02 |
|  | 2 | 219866424 | 219866628 | PDFSTATSENSEMBLUCSC | 205 | 6 | 25102 | island | 0.507048 | 0.007961 | AC097468.4; MIR375; CCDC108 (margin: AC097468.4; CRYBA2; MIR375; CCDC108) | 9.84 | 4.33e-03 | 2.52e-02 |
|  | 17 | 37123669 | 37123949 | PDFSTATSENSEMBLUCSC | 281 | 8 | 17302 | tss | 0.913609 | 0.017886 | FBXO47 (margin: FBXO47) | 9.84 | 1.55e-04 | 2.71e-03 |
|  | 11 | 117069780 | 117069966 | PDFSTATSENSEMBLUCSC | 187 | 7 | 7717 | tss | 0.028597 | 0.960869 | SIDT2; TAGLN (margin: SIDT2; TAGLN; PCSK7) | 9.83 | 5.83e-04 | 6.47e-03 |
|  | 13 | 29292888 | 29293454 | PDFSTATSENSEMBLUCSC | 567 | 11 | 11642 | island | 0.929417 | 0.016220 | SLC46A3 (margin: SLC46A3; CYP51P2) | 9.83 | 2.84e-06 | 1.96e-04 |
|  | 8 | 98290229 | 98290601 | PDFSTATSENSEMBLUCSC | 373 | 8 | 39829 | tss | 0.961593 | 0.025739 | TSPYL5 (margin: TSPYL5) | 9.83 | 1.55e-04 | 2.71e-03 |
|  | 1 | 13910555 | 13910796 | PDFSTATSENSEMBLUCSC | 242 | 6 | 235 | gene;tss;island | 0.049612 | 0.979461 | PDPN (margin: PDPN) | 9.80 | 2.16e-03 | 1.45e-02 |
|  | 9 | 140401382 | 140401576 | PDFSTATSENSEMBLUCSC | 195 | 4 | 40799 | gene;island | 0.029872 | 0.980685 | Not queried | 9.79 | 2.86e-02 | 6.12e-02 |
|  | 3 | 141087187 | 141087363 | PDFSTATSENSEMBLUCSC | 177 | 5 | 27973 | gene | 0.007575 | 0.812234 | ZBTB38; RP11-438D8.2 (margin: ZBTB38; RP11-438D8.2) | 9.78 | 7.94e-03 | 3.19e-02 |
|  | 16 | 3096477 | 3096662 | PDFSTATSENSEMBLUCSC | 186 | 5 | 15049 | tss | 0.033886 | 0.906769 | MMP25 (margin: RP11-473M20.5; MMP25; CCDC64B; RP11-473M20.7) | 9.77 | 7.94e-03 | 3.19e-02 |
|  | 1 | 42384310 | 42384474 | PDFSTATSENSEMBLUCSC | 165 | 4 | 463 | gene | 0.691826 | 0.021940 | Not queried | 9.77 | 2.86e-02 | 6.12e-02 |
|  | 9 | 36258825 | 36258971 | PDFSTATSENSEMBLUCSC | 147 | 4 | 40765 | gene;tss;island | 0.058951 | 0.984770 | Not queried | 9.77 | 2.86e-02 | 6.12e-02 |
|  | 12 | 63327142 | 63327235 | PDFSTATSENSEMBLUCSC | 94 | 4 | 9201 | gene;island | 0.877485 | 0.008056 | Not queried | 9.76 | 2.86e-02 | 6.12e-02 |
|  | 19 | 52408259 | 52408520 | PDFSTATSENSEMBLUCSC | 262 | 8 | 21000 | tss | 0.039673 | 0.971608 | ZNF649 (margin: CTC-429C10.2; ZNF649) | 9.76 | 1.09e-03 | 1.10e-02 |
|  | 5 | 121647546 | 121647642 | PDFSTATSENSEMBLUCSC | 97 | 4 | 32244 | tss;island | 0.045037 | 0.978118 | Not queried | 9.75 | 2.86e-02 | 6.12e-02 |
|  | 1 | 204183337 | 204183655 | PDFSTATSENSEMBLUCSC | 319 | 7 | 2197 | tss | 0.954737 | 0.011183 | GOLT1A (margin: GOLT1A; PLEKHA6) | 9.74 | 5.83e-04 | 6.47e-03 |
|  | 16 | 4588414 | 4589127 | PDFSTATSENSEMBLUCSC | 714 | 11 | 15671 | island | 0.019614 | 0.977908 | CDIP1 (margin: CDIP1) | 9.72 | 3.40e-05 | 1.17e-03 |
|  | 1 | 43613433 | 43613627 | PDFSTATSENSEMBLUCSC | 195 | 7 | 3158 | island | 0.913609 | 0.012452 | FAM183A (margin: FAM183A) | 9.72 | 5.83e-04 | 6.47e-03 |
|  | 19 | 18335182 | 18335429 | PDFSTATSENSEMBLUCSC | 248 | 5 | 19931 | gene;island | 0.637108 | 0.002962 | PDE4C (margin: AC068499.10; PDE4C) | 9.71 | 7.94e-03 | 3.19e-02 |
|  | 16 | 56228114 | 56228901 | PDFSTATSENSEMBLUCSC | 788 | 12 | 14756 | gene;island | 0.814362 | 0.009296 | Not queried | 9.71 | 1.73e-02 | 5.79e-02 |
|  | 5 | 89854293 | 89854979 | PDFSTATSENSEMBLUCSC | 687 | 11 | 32955 | island | 0.692951 | 0.011913 | GPR98 (margin: GPR98) | 9.70 | 2.84e-06 | 1.96e-04 |
|  | 5 | 89854632 | 89854979 | PDFSTATSENSEMBLUCSC | 348 | 5 | 31687 | gene | 0.918695 | 0.011913 | GPR98 (margin: GPR98) | 9.70 | 7.94e-03 | 3.19e-02 |
|  | 11 | 414951 | 415111 | PDFSTATSENSEMBLUCSC | 161 | 4 | 6373 | gene;island | 0.035298 | 0.959460 | Not queried | 9.70 | 2.86e-02 | 6.12e-02 |
|  | 19 | 52430190 | 52430632 | PDFSTATSENSEMBLUCSC | 443 | 7 | 21001 | tss | 0.001913 | 0.480264 | ZNF613 (margin: ZNF613) | 9.70 | 5.83e-04 | 6.47e-03 |
|  | 7 | 134143823 | 134144288 | PDFSTATSENSEMBLUCSC | 466 | 12 | 38935 | island | 0.024656 | 0.978145 | AKR1B1 (margin: AKR1B1) | 9.70 | 7.40e-07 | 7.21e-05 |
|  | 7 | 148637970 | 148638192 | PDFSTATSENSEMBLUCSC | 223 | 4 | 38986 | island | 0.800101 | 0.007461 | Not queried | 9.70 | 2.86e-02 | 6.12e-02 |
|  | 19 | 50832651 | 50832861 | PDFSTATSENSEMBLUCSC | 211 | 5 | 20974 | tss | 0.788778 | 0.016828 | NR1H2; KCNC3 (margin: NR1H2; KCNC3; NAPSB) | 9.69 | 7.94e-03 | 3.19e-02 |
|  | 14 | 103674159 | 103674342 | PDFSTATSENSEMBLUCSC | 184 | 4 | 13184 | island | 0.042567 | 0.963346 | Not queried | 9.67 | 2.86e-02 | 6.12e-02 |
|  | 21 | 42733729 | 42733894 | PDFSTATSENSEMBLUCSC | 166 | 5 | 26556 | tss | 0.022285 | 0.842877 | MX2 (margin: FAM3B; MX2) | 9.66 | 7.94e-03 | 3.19e-02 |
|  | 19 | 1132294 | 1132537 | PDFSTATSENSEMBLUCSC | 244 | 7 | 20293 | tss | 0.021376 | 0.982191 | SBNO2 (margin: SBNO2) | 9.66 | 5.83e-04 | 6.47e-03 |
|  | 4 | 175750109 | 175751001 | PDFSTATSENSEMBLUCSC | 893 | 13 | 31438 | island | 0.913758 | 0.011559 | ADAM29; GLRA3 (margin: ADAM29; GLRA3) | 9.66 | 1.92e-07 | 2.60e-05 |
|  | 19 | 34263905 | 34264111 | PDFSTATSENSEMBLUCSC | 207 | 4 | 19975 | gene;island | 0.019357 | 0.951554 | Not queried | 9.65 | 2.86e-02 | 6.12e-02 |
|  | 2 | 74153086 | 74153317 | PDFSTATSENSEMBLUCSC | 232 | 4 | 23447 | tss;island | 0.053175 | 0.977401 | Not queried | 9.65 | 2.86e-02 | 6.12e-02 |
|  | 11 | 13689411 | 13689686 | PDFSTATSENSEMBLUCSC | 276 | 5 | 7158 | tss;island | 0.411502 | 0.011516 | FAR1; FAR1-IT1 (margin: FAR1; FAR1-IT1) | 9.65 | 7.94e-03 | 3.19e-02 |
|  | 17 | 75315486 | 75315567 | PDFSTATSENSEMBLUCSC | 82 | 5 | 17696 | tss | 0.014663 | 0.851854 | SEPT9 (margin: SEPT9) | 9.64 | 7.94e-03 | 3.19e-02 |
|  | 12 | 106533840 | 106533903 | PDFSTATSENSEMBLUCSC | 64 | 5 | 9966 | tss | 0.058686 | 0.979669 | NUAK1 (margin: NUAK1) | 9.63 | 7.94e-03 | 3.19e-02 |
|  | 3 | 48632484 | 48633144 | PDFSTATSENSEMBLUCSC | 661 | 11 | 29090 | island | 0.013916 | 0.965343 | COL7A1 (margin: COL7A1; UQCRC1) | 9.63 | 2.84e-06 | 1.96e-04 |
|  | 7 | 101768610 | 101768874 | PDFSTATSENSEMBLUCSC | 265 | 4 | 37161 | gene | 0.011319 | 0.872686 | Not queried | 9.63 | 2.86e-02 | 6.12e-02 |
|  | 4 | 101111147 | 101111427 | PDFSTATSENSEMBLUCSC | 281 | 4 | 30070 | gene | 0.729836 | 0.011880 | Not queried | 9.62 | 2.86e-02 | 6.12e-02 |
|  | 11 | 124587993 | 124588484 | PDFSTATSENSEMBLUCSC | 492 | 5 | 8914 | island | 0.010895 | 0.966803 | (margin: ) | 9.61 | 7.94e-03 | 3.19e-02 |
|  | 7 | 38670804 | 38671001 | PDFSTATSENSEMBLUCSC | 198 | 6 | 36972 | gene;island | 0.725317 | 0.007844 | AMPH (margin: AMPH) | 9.60 | 2.16e-03 | 1.45e-02 |
|  | 1 | 50489654 | 50489954 | PDFSTATSENSEMBLUCSC | 301 | 8 | 1613 | tss | 0.835859 | 0.022435 | AGBL4 (margin: MTND2P29; AGBL4) | 9.60 | 1.55e-04 | 2.71e-03 |
|  | 15 | 45421578 | 45422095 | PDFSTATSENSEMBLUCSC | 518 | 11 | 13646 | tss;island | 0.121457 | 0.979476 | DUOX1; DUOXA1 (margin: DUOXA2; DUOX1; DUOXA1) | 9.60 | 2.84e-06 | 1.96e-04 |
|  | 2 | 176994142 | 176994764 | PDFSTATSENSEMBLUCSC | 623 | 9 | 24905 | island | 0.010562 | 0.932802 | HOXD8; HOXD-AS2 (margin: HOXD10; HOXD9; HOXD8; HOXD3; HOXD-AS2) | 9.59 | 2.76e-03 | 1.78e-02 |
|  | 8 | 17271067 | 17271119 | PDFSTATSENSEMBLUCSC | 53 | 4 | 39566 | tss;island | 0.886640 | 0.007375 | Not queried | 9.58 | 2.86e-02 | 6.12e-02 |
|  | 1 | 2986362 | 2986566 | PDFSTATSENSEMBLUCSC | 205 | 4 | 97 | gene;island | 0.378911 | 0.001254 | Not queried | 9.58 | 2.86e-02 | 6.12e-02 |
|  | 20 | 52199520 | 52199778 | PDFSTATSENSEMBLUCSC | 259 | 5 | 26263 | island | 0.004588 | 0.853325 | ZNF217 (margin: RP4-724E16.2; ZNF217) | 9.57 | 7.94e-03 | 3.19e-02 |
|  | 1 | 43613433 | 43613567 | PDFSTATSENSEMBLUCSC | 135 | 5 | 1556 | tss | 0.917510 | 0.029188 | FAM183A (margin: FAM183A) | 9.56 | 7.94e-03 | 3.19e-02 |
|  | 18 | 53257019 | 53257340 | PDFSTATSENSEMBLUCSC | 322 | 5 | 19286 | tss;island | 0.042610 | 0.963112 | TCF4 (margin: TCF4) | 9.56 | 7.94e-03 | 3.19e-02 |
|  | 2 | 219858132 | 219858271 | PDFSTATSENSEMBLUCSC | 140 | 6 | 23887 | tss | 0.047441 | 0.967599 | CRYBA2 (margin: AC097468.4; FEV; CRYBA2; MIR375; CCDC108) | 9.55 | 2.16e-03 | 1.45e-02 |
|  | 1 | 248100585 | 248100614 | PDFSTATSENSEMBLUCSC | 30 | 4 | 1129 | gene | 0.804205 | 0.011896 | Not queried | 9.54 | 2.86e-02 | 6.12e-02 |
|  | 1 | 24126017 | 24126230 | PDFSTATSENSEMBLUCSC | 214 | 5 | 320 | gene;island | 0.004854 | 0.685825 | GALE (margin: PITHD1; LYPLA2; GALE; HMGCL) | 9.53 | 7.94e-03 | 3.19e-02 |
|  | 11 | 66103386 | 66103604 | PDFSTATSENSEMBLUCSC | 219 | 4 | 6731 | gene;island | 0.025377 | 0.857443 | Not queried | 9.50 | 2.86e-02 | 6.12e-02 |
|  | 2 | 69664772 | 69664869 | PDFSTATSENSEMBLUCSC | 98 | 5 | 23406 | tss | 0.003137 | 0.577149 | NFU1 (margin: NFU1) | 9.49 | 7.94e-03 | 3.19e-02 |
|  | 18 | 13801010 | 13801271 | PDFSTATSENSEMBLUCSC | 262 | 4 | 19411 | island | 0.564323 | 0.006556 | Not queried | 9.48 | 2.86e-02 | 6.12e-02 |
|  | 1 | 2063799 | 2064216 | PDFSTATSENSEMBLUCSC | 418 | 5 | 63 | gene;island | 0.028678 | 0.975813 | PRKCZ (margin: PRKCZ; RP5-892K4.1) | 9.47 | 7.94e-03 | 3.19e-02 |
|  | 2 | 191045309 | 191045668 | PDFSTATSENSEMBLUCSC | 360 | 6 | 23783 | tss | 0.087126 | 0.982349 | C2orf88 (margin: C2orf88; HIBCH) | 9.45 | 2.16e-03 | 1.45e-02 |
|  | 1 | 3615390 | 3615762 | PDFSTATSENSEMBLUCSC | 373 | 5 | 139 | gene | 0.096132 | 0.985532 | TP73 (margin: TP73) | 9.44 | 7.94e-03 | 3.19e-02 |
|  | 13 | 29293196 | 29293454 | PDFSTATSENSEMBLUCSC | 259 | 8 | 11370 | tss | 0.925900 | 0.017420 | SLC46A3 (margin: SLC46A3; CYP51P2) | 9.42 | 1.55e-04 | 2.71e-03 |
|  | 22 | 19710880 | 19711051 | PDFSTATSENSEMBLUCSC | 172 | 6 | 26788 | gene;tss;island | 0.054825 | 0.984456 | SEPT5; GP1BB (margin: SEPT5; GP1BB) | 9.42 | 2.16e-03 | 1.45e-02 |
|  | 11 | 66104018 | 66104174 | PDFSTATSENSEMBLUCSC | 157 | 4 | 7465 | tss | 0.011575 | 0.843598 | Not queried | 9.42 | 2.86e-02 | 6.12e-02 |
|  | 5 | 113697861 | 113697946 | PDFSTATSENSEMBLUCSC | 86 | 4 | 32222 | tss | 0.825181 | 0.012146 | Not queried | 9.41 | 2.86e-02 | 6.12e-02 |
|  | 1 | 24152579 | 24152774 | PDFSTATSENSEMBLUCSC | 196 | 4 | 1350 | tss | 0.976206 | 0.077438 | Not queried | 9.41 | 2.86e-02 | 6.12e-02 |
|  | 7 | 138794512 | 138794752 | PDFSTATSENSEMBLUCSC | 241 | 7 | 37928 | tss | 0.005235 | 0.697692 | ZC3HAV1 (margin: ZC3HAV1) | 9.41 | 5.83e-04 | 6.47e-03 |
|  | 22 | 46481603 | 46481822 | PDFSTATSENSEMBLUCSC | 220 | 7 | 27228 | tss | 0.025801 | 0.961956 | FLJ27365 (margin: FLJ27365; MIR3619) | 9.36 | 5.83e-04 | 6.47e-03 |
|  | 3 | 193587490 | 193587939 | PDFSTATSENSEMBLUCSC | 450 | 4 | 29778 | island | 0.041739 | 0.950463 | Not queried | 9.35 | 2.86e-02 | 6.12e-02 |
|  | 5 | 101632286 | 101632559 | PDFSTATSENSEMBLUCSC | 274 | 9 | 32198 | tss | 0.967476 | 0.047995 | SLCO4C1 (margin: RN7SKP68; SLCO4C1) | 9.35 | 1.65e-04 | 2.80e-03 |
|  | 4 | 81118188 | 81118588 | PDFSTATSENSEMBLUCSC | 401 | 4 | 30033 | gene;tss;island | 0.945632 | 0.061405 | Not queried | 9.35 | 2.86e-02 | 6.12e-02 |
|  | 20 | 19915716 | 19915874 | PDFSTATSENSEMBLUCSC | 159 | 6 | 25369 | gene | 0.058450 | 0.976445 | RIN2 (margin: RIN2) | 9.35 | 2.16e-03 | 1.45e-02 |
|  | 1 | 190447232 | 190447640 | PDFSTATSENSEMBLUCSC | 409 | 5 | 2147 | tss | 0.460310 | 0.010099 | RP11-547I7.2; RP11-161I10.1; FAM5C (margin: RP11-547I7.2; RP11-161I10.1; FAM5C) | 9.35 | 7.94e-03 | 3.19e-02 |
|  | 3 | 32442928 | 32443496 | PDFSTATSENSEMBLUCSC | 569 | 6 | 27709 | gene;island | 0.128735 | 0.978788 | CMTM7 (margin: CMTM7) | 9.34 | 2.16e-03 | 1.45e-02 |
|  | 12 | 133022423 | 133022955 | PDFSTATSENSEMBLUCSC | 533 | 6 | 11058 | island | 0.174085 | 0.962201 | (margin: RP11-503G7.1) | 9.33 | 2.16e-03 | 1.45e-02 |
|  | 8 | 11665843 | 11666017 | PDFSTATSENSEMBLUCSC | 175 | 4 | 39207 | gene;island | 0.785944 | 0.016172 | Not queried | 9.33 | 2.86e-02 | 6.12e-02 |
|  | 12 | 112204716 | 112204829 | PDFSTATSENSEMBLUCSC | 114 | 4 | 9312 | gene;island | 0.025808 | 0.930630 | Not queried | 9.33 | 2.86e-02 | 6.12e-02 |
|  | 13 | 99630462 | 99630583 | PDFSTATSENSEMBLUCSC | 122 | 4 | 11203 | gene;tss | 0.046950 | 0.978530 | Not queried | 9.33 | 2.86e-02 | 6.12e-02 |
|  | 1 | 202183264 | 202183605 | PDFSTATSENSEMBLUCSC | 342 | 5 | 945 | gene;island | 0.046696 | 0.960675 | LGR6 (margin: LGR6) | 9.32 | 7.94e-03 | 3.19e-02 |
|  | 17 | 41477271 | 41477468 | PDFSTATSENSEMBLUCSC | 198 | 4 | 16597 | gene;island | 0.025214 | 0.937032 | Not queried | 9.31 | 2.86e-02 | 6.12e-02 |
|  | 19 | 37406932 | 37407486 | PDFSTATSENSEMBLUCSC | 555 | 14 | 20732 | tss;island | 0.825523 | 0.007232 | ZNF568; ZNF829 (margin: ZNF345; ZNF568; ZNF829) | 9.31 | 4.99e-08 | 9.34e-06 |
|  | 1 | 207224090 | 207224388 | PDFSTATSENSEMBLUCSC | 299 | 5 | 975 | gene | 0.007758 | 0.958089 | PFKFB2; YOD1 (margin: PFKFB2; snoU13; YOD1) | 9.29 | 7.94e-03 | 3.19e-02 |
|  | 4 | 91048416 | 91048584 | PDFSTATSENSEMBLUCSC | 169 | 6 | 30498 | tss;island | 0.628345 | 0.005865 | CCSER1 (margin: CCSER1) | 9.28 | 2.16e-03 | 1.45e-02 |
|  | 2 | 121104029 | 121104400 | PDFSTATSENSEMBLUCSC | 372 | 4 | 22849 | gene;island | 0.806658 | 0.005966 | Not queried | 9.28 | 2.86e-02 | 6.12e-02 |
|  | 1 | 7022041 | 7022284 | PDFSTATSENSEMBLUCSC | 244 | 4 | 183 | gene;island | 0.952907 | 0.035336 | Not queried | 9.28 | 2.86e-02 | 6.12e-02 |
|  | 1 | 162337134 | 162337375 | PDFSTATSENSEMBLUCSC | 242 | 4 | 859 | gene;island | 0.107483 | 0.990146 | Not queried | 9.27 | 2.86e-02 | 6.12e-02 |
|  | 7 | 138720786 | 138721065 | PDFSTATSENSEMBLUCSC | 280 | 9 | 37927 | tss;island | 0.030965 | 0.980251 | ZC3HAV1L (margin: ZC3HAV1L; ZC3HAV1) | 9.27 | 4.11e-05 | 1.17e-03 |
|  | 11 | 12030088 | 12031508 | PDFSTATSENSEMBLUCSC | 1421 | 21 | 8065 | island | 0.039606 | 0.971522 | DKK3 (margin: DKK3) | 9.26 | 2.72e-08 | 6.41e-06 |
|  | 17 | 46124684 | 46124821 | PDFSTATSENSEMBLUCSC | 138 | 4 | 17451 | tss;island | 0.904854 | 0.010388 | Not queried | 9.25 | 2.86e-02 | 6.12e-02 |
|  | 18 | 59560365 | 59560495 | PDFSTATSENSEMBLUCSC | 131 | 5 | 19301 | tss | 0.762207 | 0.005339 | RNF152 (margin: RNF152) | 9.25 | 7.94e-03 | 3.19e-02 |
|  | 5 | 101631990 | 101632559 | PDFSTATSENSEMBLUCSC | 570 | 12 | 32986 | island | 0.953598 | 0.030635 | SLCO4C1 (margin: RN7SKP68; SLCO4C1) | 9.24 | 2.96e-06 | 2.02e-04 |
|  | 11 | 34535357 | 34535579 | PDFSTATSENSEMBLUCSC | 223 | 7 | 7242 | tss | 0.959613 | 0.037742 | ELF5 (margin: ELF5) | 9.24 | 5.83e-04 | 6.47e-03 |
|  | 6 | 28092048 | 28092370 | PDFSTATSENSEMBLUCSC | 323 | 8 | 34732 | tss | 0.880292 | 0.018527 | ZSCAN16; RP1-265C24.9 (margin: RP1-265C24.5; ZSCAN16; RP1-265C24.9) | 9.24 | 1.55e-04 | 2.71e-03 |
|  | 4 | 146859802 | 146860017 | PDFSTATSENSEMBLUCSC | 216 | 4 | 30621 | tss;island | 0.009361 | 0.699793 | Not queried | 9.23 | 2.86e-02 | 6.12e-02 |
|  | 16 | 4588414 | 4588809 | PDFSTATSENSEMBLUCSC | 396 | 4 | 14641 | gene | 0.071580 | 0.972108 | Not queried | 9.21 | 2.86e-02 | 6.12e-02 |
|  | 12 | 6560689 | 6560851 | PDFSTATSENSEMBLUCSC | 163 | 5 | 9028 | gene | 0.015785 | 0.899048 | CD27; TAPBPL; CD27-AS1 (margin: CD27; TAPBPL; CD27-AS1; VAMP1) | 9.20 | 7.94e-03 | 3.19e-02 |
|  | 8 | 70746191 | 70746368 | PDFSTATSENSEMBLUCSC | 178 | 4 | 39332 | gene;tss;island | 0.451373 | 0.005105 | Not queried | 9.20 | 2.86e-02 | 6.12e-02 |
|  | 6 | 35732234 | 35732480 | PDFSTATSENSEMBLUCSC | 247 | 4 | 36075 | island | 0.749264 | 0.010799 | Not queried | 9.18 | 2.86e-02 | 6.12e-02 |
|  | 12 | 56325715 | 56325867 | PDFSTATSENSEMBLUCSC | 153 | 6 | 9169 | gene;island | 0.001860 | 0.458642 | DGKA; WIBG (margin: DGKA; WIBG) | 9.18 | 2.16e-03 | 1.45e-02 |
|  | 1 | 47655861 | 47656140 | PDFSTATSENSEMBLUCSC | 280 | 4 | 1606 | tss | 0.016277 | 0.896488 | Not queried | 9.17 | 2.86e-02 | 6.12e-02 |
|  | 6 | 39016262 | 39016479 | PDFSTATSENSEMBLUCSC | 218 | 5 | 34952 | tss | 0.027951 | 0.961741 | Not queried | 9.16 | 1.59e-02 | 5.36e-02 |
|  | 2 | 97171137 | 97171449 | PDFSTATSENSEMBLUCSC | 313 | 5 | 22781 | gene;island | 0.004061 | 0.697676 | NEURL3 (margin: NEURL3) | 9.16 | 7.94e-03 | 3.19e-02 |
|  | 17 | 75315486 | 75315868 | PDFSTATSENSEMBLUCSC | 383 | 8 | 16804 | gene | 0.015659 | 0.855331 | SEPT9 (margin: SEPT9) | 9.16 | 1.55e-04 | 2.71e-03 |
|  | 11 | 12030187 | 12031508 | PDFSTATSENSEMBLUCSC | 1322 | 20 | 7153 | tss | 0.035832 | 0.970982 | DKK3 (margin: DKK3) | 9.15 | 1.06e-07 | 1.81e-05 |
|  | 5 | 23507450 | 23507656 | PDFSTATSENSEMBLUCSC | 207 | 9 | 32001 | tss | 0.846754 | 0.018159 | PRDM9 (margin: PRDM9) | 9.13 | 4.11e-05 | 1.17e-03 |
|  | 6 | 132128915 | 132129103 | PDFSTATSENSEMBLUCSC | 189 | 9 | 35224 | tss;island | 0.608125 | 0.004505 | ENPP1 (margin: ENPP1; RP3-323K23.3) | 9.11 | 4.11e-05 | 1.17e-03 |
|  | 12 | 54388841 | 54389264 | PDFSTATSENSEMBLUCSC | 424 | 5 | 10488 | island | 0.024892 | 0.969435 | HOXC5; HOXC6; HOXC9; HOXC-AS2 (margin: HOXC10; HOXC5; HOXC6; MIR196A2; HOXC9; HOXC-AS3; HOXC-AS2; HOXC-AS1) | 9.11 | 7.94e-03 | 3.19e-02 |
|  | 8 | 38964784 | 38965492 | PDFSTATSENSEMBLUCSC | 709 | 11 | 40239 | island | 0.987796 | 0.084596 | ADAM32 (margin: ADAM9; ADAM32) | 9.11 | 1.98e-05 | 8.44e-04 |
|  | 8 | 38964784 | 38965026 | PDFSTATSENSEMBLUCSC | 243 | 7 | 39662 | tss | 0.987257 | 0.084596 | ADAM32 (margin: ADAM9; ADAM32) | 9.11 | 2.33e-03 | 1.52e-02 |
|  | 19 | 12267592 | 12267796 | PDFSTATSENSEMBLUCSC | 205 | 6 | 20490 | tss | 0.018638 | 0.906795 | ZNF625-ZNF20; ZNF625 (margin: ZNF136; ZNF625-ZNF20; ZNF625) | 9.11 | 2.16e-03 | 1.45e-02 |
|  | 4 | 184826215 | 184826479 | PDFSTATSENSEMBLUCSC | 265 | 9 | 30693 | tss;island | 0.874283 | 0.018242 | STOX2 (margin: STOX2) | 9.10 | 4.11e-05 | 1.17e-03 |
|  | 16 | 66638210 | 66638438 | PDFSTATSENSEMBLUCSC | 229 | 8 | 15283 | tss | 0.836301 | 0.009440 | CMTM3 (margin: CMTM3; CMTM4) | 9.10 | 1.55e-04 | 2.71e-03 |
|  | 6 | 28129313 | 28129498 | PDFSTATSENSEMBLUCSC | 186 | 7 | 34734 | tss | 0.038744 | 0.862817 | ZNF192P1 (margin: ZKSCAN8; ZNF192P1) | 9.09 | 5.83e-04 | 6.47e-03 |
|  | 5 | 1882775 | 1883011 | PDFSTATSENSEMBLUCSC | 237 | 8 | 32645 | island | 0.678166 | 0.027929 | CTD-2194D22.3; IRX4 (margin: CTD-2194D22.3; CTD-2194D22.4; IRX4) | 9.08 | 1.55e-04 | 2.71e-03 |
|  | 5 | 1882885 | 1883011 | PDFSTATSENSEMBLUCSC | 127 | 6 | 31974 | tss | 0.726794 | 0.032784 | CTD-2194D22.3; IRX4 (margin: CTD-2194D22.3; CTD-2194D22.4; IRX4) | 9.08 | 2.16e-03 | 1.45e-02 |
|  | 4 | 37455280 | 37455499 | PDFSTATSENSEMBLUCSC | 220 | 7 | 30341 | tss;island | 0.877526 | 0.026067 | C4orf19 (margin: KIAA1239; C4orf19) | 9.08 | 5.83e-04 | 6.47e-03 |
|  | 2 | 75788212 | 75788552 | PDFSTATSENSEMBLUCSC | 341 | 8 | 23469 | tss | 0.128480 | 0.985477 | EVA1A (margin: EVA1A) | 9.08 | 1.55e-04 | 2.71e-03 |
|  | 12 | 56325715 | 56325797 | PDFSTATSENSEMBLUCSC | 83 | 4 | 9775 | tss | 0.001860 | 0.424641 | Not queried | 9.07 | 2.86e-02 | 6.12e-02 |
|  | 6 | 32548321 | 32548627 | PDFSTATSENSEMBLUCSC | 307 | 4 | 34120 | gene;island | 0.024578 | 0.885483 | Not queried | 9.06 | 2.86e-02 | 6.12e-02 |
|  | 8 | 58907018 | 58907387 | PDFSTATSENSEMBLUCSC | 370 | 6 | 40321 | island | 0.843875 | 0.015844 | FAM110B (margin: RP11-1112C15.1; FAM110B) | 9.05 | 2.16e-03 | 1.45e-02 |
|  | 8 | 49293379 | 49293600 | PDFSTATSENSEMBLUCSC | 222 | 4 | 40282 | island | 0.044708 | 0.967290 | Not queried | 9.05 | 2.86e-02 | 6.12e-02 |
|  | 5 | 118965093 | 118965233 | PDFSTATSENSEMBLUCSC | 141 | 4 | 32239 | tss | 0.184450 | 0.973334 | Not queried | 9.05 | 2.86e-02 | 6.12e-02 |
|  | 10 | 118033581 | 118034031 | PDFSTATSENSEMBLUCSC | 451 | 4 | 5383 | tss;island | 0.778200 | 0.012974 | Not queried | 9.05 | 2.86e-02 | 6.12e-02 |
|  | 6 | 27841633 | 27841865 | PDFSTATSENSEMBLUCSC | 233 | 4 | 34729 | tss;island | 0.075409 | 0.982097 | Not queried | 9.03 | 2.86e-02 | 6.12e-02 |
|  | 2 | 75788038 | 75788552 | PDFSTATSENSEMBLUCSC | 515 | 9 | 22752 | gene;island | 0.133202 | 0.984694 | EVA1A (margin: EVA1A) | 9.03 | 4.11e-05 | 1.17e-03 |
|  | 2 | 219157083 | 219157702 | PDFSTATSENSEMBLUCSC | 620 | 11 | 23014 | gene;island | 0.010717 | 0.942961 | PNKD; TMBIM1 (margin: PNKD; TMBIM1) | 9.03 | 8.51e-05 | 2.07e-03 |
|  | 19 | 37701476 | 37701738 | PDFSTATSENSEMBLUCSC | 263 | 7 | 20735 | tss | 0.384546 | 0.002679 | CTC-454I21.3; ZNF585B (margin: ZNF383; CTC-454I21.3; ZNF585B) | 9.02 | 5.83e-04 | 6.47e-03 |
|  | 1 | 57110686 | 57110878 | PDFSTATSENSEMBLUCSC | 193 | 6 | 1652 | tss;island | 0.684087 | 0.008662 | PRKAA2; PPAP2B (margin: PRKAA2; PPAP2B) | 9.01 | 2.16e-03 | 1.45e-02 |
|  | 19 | 16178091 | 16178253 | PDFSTATSENSEMBLUCSC | 163 | 6 | 20566 | tss | 0.002484 | 0.607286 | CTD-2231E14.4; TPM4 (margin: CTD-2231E14.4; TPM4) | 9.01 | 2.16e-03 | 1.45e-02 |
|  | 12 | 106533667 | 106533903 | PDFSTATSENSEMBLUCSC | 237 | 6 | 10747 | island | 0.054368 | 0.972992 | NUAK1 (margin: NUAK1) | 9.00 | 2.16e-03 | 1.45e-02 |
|  | 6 | 11771473 | 11771840 | PDFSTATSENSEMBLUCSC | 368 | 4 | 33572 | gene | 0.029615 | 0.900774 | Not queried | 8.99 | 2.86e-02 | 6.12e-02 |
|  | 1 | 215256254 | 215256451 | PDFSTATSENSEMBLUCSC | 198 | 4 | 2253 | tss | 0.750750 | 0.008462 | Not queried | 8.99 | 2.86e-02 | 6.12e-02 |
|  | 5 | 118691415 | 118691794 | PDFSTATSENSEMBLUCSC | 380 | 6 | 31725 | gene;island | 0.003070 | 0.343675 | TNFAIP8 (margin: TNFAIP8) | 8.99 | 2.16e-03 | 1.45e-02 |
|  | 6 | 29855110 | 29855347 | PDFSTATSENSEMBLUCSC | 238 | 7 | 34756 | tss | 0.072403 | 0.966219 | HLA-H; HCG4P7 (margin: HLA-H; HLA-T; HCG4P7) | 8.98 | 5.83e-04 | 6.47e-03 |
|  | 1 | 2359927 | 2360074 | PDFSTATSENSEMBLUCSC | 148 | 4 | 2501 | island | 0.059530 | 0.958642 | Not queried | 8.96 | 2.86e-02 | 6.12e-02 |
|  | 19 | 20150159 | 20150505 | PDFSTATSENSEMBLUCSC | 347 | 7 | 20655 | tss;island | 0.665355 | 0.004091 | ZNF682 (margin: AC006539.3; ZNF682) | 8.96 | 5.83e-04 | 6.47e-03 |
|  | 17 | 33701321 | 33701529 | PDFSTATSENSEMBLUCSC | 209 | 4 | 17268 | tss;island | 0.112543 | 0.984104 | Not queried | 8.96 | 2.86e-02 | 6.12e-02 |
|  | 2 | 191045309 | 191045885 | PDFSTATSENSEMBLUCSC | 577 | 8 | 22960 | gene;island | 0.121313 | 0.978846 | C2orf88 (margin: C2orf88; HIBCH) | 8.95 | 1.55e-04 | 2.71e-03 |
|  | 2 | 69664562 | 69664869 | PDFSTATSENSEMBLUCSC | 308 | 8 | 24369 | island | 0.003569 | 0.519370 | NFU1 (margin: NFU1) | 8.94 | 1.55e-04 | 2.71e-03 |
|  | 2 | 219866437 | 219866628 | PDFSTATSENSEMBLUCSC | 192 | 5 | 23888 | tss | 0.432642 | 0.015110 | Not queried | 8.94 | 1.59e-02 | 5.36e-02 |
|  | 2 | 11679845 | 11680057 | PDFSTATSENSEMBLUCSC | 213 | 5 | 23191 | tss | 0.588034 | 0.009326 | GREB1; MIR4429 (margin: GREB1; MIR4429) | 8.94 | 7.94e-03 | 3.19e-02 |
|  | 2 | 238395764 | 238395947 | PDFSTATSENSEMBLUCSC | 184 | 5 | 25230 | island | 0.772013 | 0.008410 | MLPH (margin: MLPH) | 8.94 | 7.94e-03 | 3.19e-02 |
|  | 10 | 30723892 | 30724374 | PDFSTATSENSEMBLUCSC | 483 | 6 | 4550 | gene;island | 0.008516 | 0.820812 | MAP3K8 (margin: MAP3K8) | 8.93 | 2.16e-03 | 1.45e-02 |
|  | 12 | 51818636 | 51818768 | PDFSTATSENSEMBLUCSC | 133 | 4 | 9132 | gene | 0.450213 | 0.002534 | Not queried | 8.92 | 2.86e-02 | 6.12e-02 |
|  | 1 | 50489240 | 50489954 | PDFSTATSENSEMBLUCSC | 715 | 15 | 3249 | island | 0.891673 | 0.023798 | AGBL4 (margin: MTND2P29; AGBL4) | 8.92 | 5.80e-07 | 7.07e-05 |
|  | 10 | 43892790 | 43893015 | PDFSTATSENSEMBLUCSC | 226 | 8 | 4570 | gene;tss;island | 0.861726 | 0.015783 | HNRNPF (margin: HNRNPF) | 8.92 | 1.55e-04 | 2.71e-03 |
|  | 14 | 24457893 | 24458014 | PDFSTATSENSEMBLUCSC | 122 | 6 | 12308 | tss | 0.013160 | 0.737206 | DHRS4L2; DHRS4-AS1 (margin: DHRS4L2; DHRS4-AS1) | 8.92 | 2.16e-03 | 1.45e-02 |
|  | 17 | 41446167 | 41446521 | PDFSTATSENSEMBLUCSC | 355 | 5 | 18369 | island | 0.004838 | 0.531986 | LINC00910 (margin: LINC00910) | 8.91 | 7.94e-03 | 3.19e-02 |
|  | 1 | 3607097 | 3607222 | PDFSTATSENSEMBLUCSC | 126 | 4 | 1186 | tss | 0.098070 | 0.980860 | Not queried | 8.90 | 2.86e-02 | 6.12e-02 |
|  | 2 | 165812079 | 165812297 | PDFSTATSENSEMBLUCSC | 219 | 4 | 23694 | tss | 0.961590 | 0.036482 | Not queried | 8.89 | 1.14e-01 | 1.68e-01 |
|  | 6 | 10416337 | 10416531 | PDFSTATSENSEMBLUCSC | 195 | 4 | 33562 | gene;tss;island | 0.004692 | 0.623777 | Not queried | 8.88 | 2.86e-02 | 6.12e-02 |
|  | 6 | 166400871 | 166401018 | PDFSTATSENSEMBLUCSC | 148 | 4 | 35332 | tss | 0.414624 | 0.002382 | Not queried | 8.87 | 2.86e-02 | 6.12e-02 |
|  | 22 | 49297824 | 49297925 | PDFSTATSENSEMBLUCSC | 102 | 4 | 27601 | island | 0.079319 | 0.978141 | Not queried | 8.87 | 2.86e-02 | 6.12e-02 |
|  | 19 | 1401118 | 1401492 | PDFSTATSENSEMBLUCSC | 375 | 5 | 19654 | gene | 0.675243 | 0.004816 | GAMT (margin: NDUFS7; DAZAP1; AC005329.7; GAMT) | 8.87 | 7.94e-03 | 3.19e-02 |
|  | 13 | 88324432 | 88324711 | PDFSTATSENSEMBLUCSC | 280 | 4 | 11494 | tss | 0.870474 | 0.019563 | Not queried | 8.86 | 2.86e-02 | 6.12e-02 |
|  | 1 | 155043322 | 155043967 | PDFSTATSENSEMBLUCSC | 646 | 7 | 3806 | island | 0.031025 | 0.980523 | EFNA4; EFNA3 (margin: ADAM15; EFNA4; EFNA3) | 8.86 | 5.83e-04 | 6.47e-03 |
|  | 6 | 166400871 | 166401051 | PDFSTATSENSEMBLUCSC | 181 | 5 | 34541 | gene;island | 0.457473 | 0.003310 | PDE10A; LINC00473 (margin: PDE10A; LINC00473) | 8.86 | 7.94e-03 | 3.19e-02 |
|  | 5 | 89854293 | 89854539 | PDFSTATSENSEMBLUCSC | 247 | 6 | 32174 | tss | 0.635778 | 0.013226 | GPR98 (margin: GPR98) | 8.85 | 2.16e-03 | 1.45e-02 |
|  | 2 | 11679845 | 11680144 | PDFSTATSENSEMBLUCSC | 300 | 7 | 22571 | gene | 0.588034 | 0.028536 | GREB1; MIR4429 (margin: GREB1; MIR4429) | 8.85 | 5.83e-04 | 6.47e-03 |
|  | 12 | 79258313 | 79258438 | PDFSTATSENSEMBLUCSC | 126 | 4 | 9225 | gene;tss | 0.954512 | 0.041782 | Not queried | 8.83 | 2.86e-02 | 6.12e-02 |
|  | 19 | 16178091 | 16178570 | PDFSTATSENSEMBLUCSC | 480 | 9 | 21712 | island | 0.004067 | 0.669571 | CTD-2231E14.4; TPM4 (margin: CTD-2231E14.4; TPM4) | 8.83 | 4.11e-05 | 1.17e-03 |
|  | 10 | 27609163 | 27609333 | PDFSTATSENSEMBLUCSC | 171 | 4 | 5629 | island | 0.046912 | 0.919988 | Not queried | 8.83 | 2.86e-02 | 6.12e-02 |
|  | 6 | 31516015 | 31516059 | PDFSTATSENSEMBLUCSC | 45 | 4 | 33899 | gene;tss | 0.021728 | 0.902976 | Not queried | 8.82 | 2.86e-02 | 6.12e-02 |
|  | 17 | 42392436 | 42392697 | PDFSTATSENSEMBLUCSC | 262 | 4 | 16614 | gene;island | 0.552458 | 0.001933 | Not queried | 8.81 | 2.86e-02 | 6.12e-02 |
|  | 3 | 48700375 | 48700498 | PDFSTATSENSEMBLUCSC | 124 | 6 | 28305 | tss | 0.422085 | 0.003052 | RP11-148G20.1; CELSR3; NCKIPSD (margin: RP11-148G20.1; RP11-572O6.1; CELSR3; NCKIPSD) | 8.81 | 2.16e-03 | 1.45e-02 |
|  | 5 | 118690955 | 118691126 | PDFSTATSENSEMBLUCSC | 172 | 5 | 31724 | gene;tss;island | 0.004970 | 0.820874 | TNFAIP8 (margin: TNFAIP8) | 8.81 | 7.94e-03 | 3.19e-02 |
|  | 19 | 49127327 | 49127660 | PDFSTATSENSEMBLUCSC | 334 | 5 | 20132 | gene;island | 0.959783 | 0.017284 | SPHK2; AC022154.7 (margin: SPHK2; FAM83E; RPL18; AC022154.7; DBP) | 8.81 | 7.94e-03 | 3.19e-02 |
|  | 1 | 42384310 | 42384647 | PDFSTATSENSEMBLUCSC | 338 | 6 | 3142 | island | 0.604034 | 0.016815 | HIVEP3 (margin: HIVEP3) | 8.79 | 2.16e-03 | 1.45e-02 |
|  | 15 | 91498139 | 91498497 | PDFSTATSENSEMBLUCSC | 359 | 4 | 13481 | gene | 0.743268 | 0.014918 | Not queried | 8.79 | 2.86e-02 | 6.12e-02 |
|  | 13 | 84456127 | 84456486 | PDFSTATSENSEMBLUCSC | 360 | 6 | 11186 | gene | 0.966255 | 0.071352 | SLITRK1 (margin: SLITRK1) | 8.78 | 8.66e-03 | 3.36e-02 |
|  | 17 | 8027830 | 8027961 | PDFSTATSENSEMBLUCSC | 132 | 4 | 17108 | tss;island | 0.882005 | 0.013921 | Not queried | 8.78 | 2.86e-02 | 6.12e-02 |
|  | 10 | 17270087 | 17270601 | PDFSTATSENSEMBLUCSC | 515 | 10 | 5578 | island | 0.136589 | 0.983783 | VIM; VIM-AS1 (margin: VIM; VIM-AS1; RP11-124N14.3) | 8.78 | 1.08e-05 | 4.82e-04 |
|  | 19 | 38794554 | 38794664 | PDFSTATSENSEMBLUCSC | 111 | 4 | 20749 | tss | 0.003183 | 0.543103 | Not queried | 8.77 | 2.86e-02 | 6.12e-02 |
|  | 11 | 106889318 | 106889745 | PDFSTATSENSEMBLUCSC | 428 | 7 | 7659 | tss;island | 0.386764 | 0.004799 | GUCY1A2 (margin: GUCY1A2) | 8.76 | 1.17e-03 | 1.13e-02 |
|  | 11 | 87908631 | 87908938 | PDFSTATSENSEMBLUCSC | 308 | 8 | 7612 | tss | 0.102286 | 0.971219 | MIR3166; RAB38 (margin: MIR3166; RAB38) | 8.76 | 1.55e-04 | 2.71e-03 |
|  | 17 | 78161467 | 78161572 | PDFSTATSENSEMBLUCSC | 106 | 5 | 17722 | tss | 0.011574 | 0.864894 | CARD14 (margin: CARD14) | 8.76 | 7.94e-03 | 3.19e-02 |
|  | 1 | 153508511 | 153508713 | PDFSTATSENSEMBLUCSC | 203 | 6 | 760 | gene | 0.003945 | 0.606352 | BX470102.3; S100A6; S100A5 (margin: BX470102.3; S100A6; S100A5; S100A4; S100A3) | 8.75 | 2.16e-03 | 1.45e-02 |
|  | 3 | 157824148 | 157824283 | PDFSTATSENSEMBLUCSC | 136 | 5 | 28682 | tss;island | 0.336309 | 0.002194 | RSRC1; SHOX2 (margin: RSRC1; SHOX2) | 8.75 | 7.94e-03 | 3.19e-02 |
|  | 13 | 61989701 | 61990025 | PDFSTATSENSEMBLUCSC | 325 | 8 | 11470 | tss | 0.744357 | 0.009223 | Not queried | 8.74 | 1.48e-02 | 5.36e-02 |
|  | 5 | 140105626 | 140105729 | PDFSTATSENSEMBLUCSC | 104 | 6 | 32351 | tss | 0.001389 | 0.377814 | VTRNA1-3 (margin: VTRNA1-2; VTRNA1-3; RP11-515C16.7) | 8.73 | 2.16e-03 | 1.45e-02 |
|  | 22 | 46481603 | 46482023 | PDFSTATSENSEMBLUCSC | 421 | 10 | 27581 | island | 0.065337 | 0.964811 | FLJ27365 (margin: FLJ27365; MIR3619) | 8.73 | 1.08e-05 | 4.82e-04 |
|  | 2 | 219157356 | 219157702 | PDFSTATSENSEMBLUCSC | 347 | 6 | 23877 | tss | 0.013629 | 0.747018 | Not queried | 8.72 | 2.60e-02 | 6.12e-02 |
|  | 4 | 156297854 | 156298050 | PDFSTATSENSEMBLUCSC | 197 | 5 | 30158 | gene | 0.860690 | 0.015627 | AC097467.2; MAP9 (margin: AC097467.2; RP11-27G13.5; MAP9; RP11-27G13.3; RP11-27G13.4) | 8.72 | 7.94e-03 | 3.19e-02 |
|  | 3 | 50604613 | 50605269 | PDFSTATSENSEMBLUCSC | 657 | 8 | 29160 | island | 0.051019 | 0.964121 | HEMK1; C3orf18 (margin: HEMK1; C3orf18) | 8.70 | 1.09e-03 | 1.10e-02 |
|  | 17 | 9479910 | 9479965 | PDFSTATSENSEMBLUCSC | 56 | 7 | 17127 | tss;island | 0.387108 | 0.003701 | WDR16; STX8 (margin: WDR16; STX8) | 8.70 | 5.83e-04 | 6.47e-03 |
|  | 18 | 59560091 | 59560495 | PDFSTATSENSEMBLUCSC | 405 | 8 | 19547 | island | 0.787620 | 0.010956 | RNF152 (margin: RNF152) | 8.70 | 1.55e-04 | 2.71e-03 |
|  | 16 | 3096477 | 3096711 | PDFSTATSENSEMBLUCSC | 235 | 6 | 15635 | island | 0.057479 | 0.931068 | MMP25 (margin: RP11-473M20.5; MMP25; CCDC64B; RP11-473M20.7) | 8.69 | 2.16e-03 | 1.45e-02 |
|  | 6 | 34206400 | 34206683 | PDFSTATSENSEMBLUCSC | 284 | 4 | 34302 | gene;island | 0.010049 | 0.765712 | Not queried | 8.69 | 5.71e-02 | 1.00e-01 |
|  | 20 | 3220565 | 3220985 | PDFSTATSENSEMBLUCSC | 421 | 5 | 25969 | island | 0.040605 | 0.956441 | SLC4A11 (margin: SLC4A11; C20orf194) | 8.69 | 7.94e-03 | 3.19e-02 |
|  | 17 | 78161467 | 78161706 | PDFSTATSENSEMBLUCSC | 240 | 6 | 16859 | gene;island | 0.014000 | 0.878414 | CARD14 (margin: CARD14) | 8.69 | 2.16e-03 | 1.45e-02 |
|  | 6 | 30652647 | 30652907 | PDFSTATSENSEMBLUCSC | 261 | 5 | 33803 | gene;island | 0.083283 | 0.967976 | PPP1R18 (margin: DHX16; PPP1R18; NRM; RPL7P4) | 8.68 | 7.94e-03 | 3.19e-02 |
|  | 8 | 143868013 | 143868262 | PDFSTATSENSEMBLUCSC | 250 | 8 | 39939 | tss | 0.026861 | 0.884179 | RP11-706C16.8; LY6D (margin: RP11-706C16.8; LYNX1; LY6D) | 8.67 | 1.55e-04 | 2.71e-03 |
|  | 19 | 6740812 | 6741181 | PDFSTATSENSEMBLUCSC | 370 | 6 | 19771 | gene;island | 0.191525 | 0.968983 | TRIP10 (margin: TRIP10; C3; GPR108; CTD-3128G10.6; SH2D3A) | 8.66 | 2.16e-03 | 1.45e-02 |
|  | 20 | 44540794 | 44541153 | PDFSTATSENSEMBLUCSC | 360 | 5 | 25783 | tss | 0.192369 | 0.988378 | PLTP (margin: PLTP) | 8.66 | 7.94e-03 | 3.19e-02 |
|  | 15 | 45422325 | 45422553 | PDFSTATSENSEMBLUCSC | 229 | 4 | 13328 | gene;tss;island | 0.114533 | 0.979096 | Not queried | 8.66 | 2.86e-02 | 6.12e-02 |
|  | 12 | 104609432 | 104610064 | PDFSTATSENSEMBLUCSC | 633 | 8 | 10736 | island | 0.056908 | 0.982772 | TXNRD1 (margin: TXNRD1) | 8.66 | 1.55e-04 | 2.71e-03 |
|  | 12 | 71833017 | 71833193 | PDFSTATSENSEMBLUCSC | 177 | 4 | 9871 | tss;island | 0.710025 | 0.008791 | Not queried | 8.65 | 2.86e-02 | 6.12e-02 |
|  | 3 | 139258700 | 139258948 | PDFSTATSENSEMBLUCSC | 249 | 7 | 28633 | tss | 0.093914 | 0.974384 | RP11-319G6.1; RBP1 (margin: RP11-319G6.1; RBP1) | 8.65 | 5.83e-04 | 6.47e-03 |
|  | 20 | 44540777 | 44541153 | PDFSTATSENSEMBLUCSC | 377 | 6 | 26209 | island | 0.202694 | 0.988870 | PLTP (margin: PLTP) | 8.65 | 2.16e-03 | 1.45e-02 |
|  | 1 | 36184717 | 36184863 | PDFSTATSENSEMBLUCSC | 147 | 4 | 3043 | island | 0.543379 | 0.018011 | Not queried | 8.65 | 5.71e-02 | 1.00e-01 |
|  | 4 | 7436701 | 7437156 | PDFSTATSENSEMBLUCSC | 456 | 5 | 30292 | tss | 0.010148 | 0.805170 | Not queried | 8.65 | 2.22e-01 | 2.76e-01 |
|  | 7 | 107205042 | 107205392 | PDFSTATSENSEMBLUCSC | 351 | 5 | 37180 | gene;tss;island | 0.003187 | 0.673096 | DUS4L; COG5 (margin: DUS4L; COG5) | 8.64 | 7.94e-03 | 3.19e-02 |
|  | 12 | 6809887 | 6809939 | PDFSTATSENSEMBLUCSC | 53 | 4 | 9523 | tss | 0.346858 | 0.003307 | Not queried | 8.64 | 2.86e-02 | 6.12e-02 |
|  | 17 | 38599271 | 38599508 | PDFSTATSENSEMBLUCSC | 238 | 4 | 17334 | tss | 0.879795 | 0.033131 | Not queried | 8.63 | 2.86e-02 | 6.12e-02 |
|  | 17 | 7108305 | 7108792 | PDFSTATSENSEMBLUCSC | 488 | 4 | 16382 | gene;island | 0.010458 | 0.802690 | Not queried | 8.62 | 2.86e-02 | 6.12e-02 |
|  | 3 | 139258658 | 139258948 | PDFSTATSENSEMBLUCSC | 291 | 8 | 29539 | island | 0.093103 | 0.974421 | RP11-319G6.1; RBP1 (margin: RP11-319G6.1; RBP1) | 8.62 | 1.55e-04 | 2.71e-03 |
|  | 17 | 8771003 | 8771331 | PDFSTATSENSEMBLUCSC | 329 | 5 | 17123 | tss | 0.008925 | 0.540892 | PIK3R6 (margin: PIK3R6; PIK3R5) | 8.62 | 7.94e-03 | 3.19e-02 |
|  | 13 | 88324432 | 88324879 | PDFSTATSENSEMBLUCSC | 448 | 5 | 11809 | island | 0.867562 | 0.021160 | SLITRK5; MIR4500HG (margin: SLITRK5; MIR4500HG) | 8.61 | 7.94e-03 | 3.19e-02 |
|  | 19 | 38794554 | 38794845 | PDFSTATSENSEMBLUCSC | 292 | 6 | 20014 | gene | 0.003595 | 0.543103 | CTB-102L5.4; C19orf33; YIF1B (margin: SPINT2; CTB-102L5.4; Y\_RNA; C19orf33; YIF1B) | 8.60 | 2.16e-03 | 1.45e-02 |
|  | 4 | 175138997 | 175139240 | PDFSTATSENSEMBLUCSC | 244 | 4 | 31434 | island | 0.591675 | 0.003914 | Not queried | 8.60 | 2.86e-02 | 6.12e-02 |
|  | 8 | 49647579 | 49647833 | PDFSTATSENSEMBLUCSC | 255 | 6 | 39301 | gene | 0.079211 | 0.979170 | EFCAB1 (margin: EFCAB1) | 8.60 | 2.16e-03 | 1.45e-02 |
|  | 10 | 729479 | 729956 | PDFSTATSENSEMBLUCSC | 478 | 6 | 4451 | gene | 0.052854 | 0.978291 | DIP2C (margin: DIP2C) | 8.60 | 2.16e-03 | 1.45e-02 |
|  | 19 | 9434309 | 9434924 | PDFSTATSENSEMBLUCSC | 616 | 10 | 20422 | tss | 0.029063 | 0.800087 | ZNF559; ZNF177 (margin: ZNF559; ZNF177) | 8.60 | 1.08e-05 | 4.82e-04 |
|  | 14 | 100626084 | 100626205 | PDFSTATSENSEMBLUCSC | 122 | 6 | 12602 | tss;island | 0.827248 | 0.015392 | DEGS2 (margin: DEGS2) | 8.60 | 2.16e-03 | 1.45e-02 |
|  | 19 | 54371775 | 54372062 | PDFSTATSENSEMBLUCSC | 288 | 4 | 20208 | gene;tss;island | 0.474826 | 0.002641 | Not queried | 8.60 | 2.86e-02 | 6.12e-02 |
|  | 17 | 7530871 | 7531394 | PDFSTATSENSEMBLUCSC | 524 | 13 | 16406 | gene;tss;island | 0.003860 | 0.461972 | SHBG; SAT2 (margin: SHBG; SAT2) | 8.59 | 1.92e-07 | 2.60e-05 |
|  | 1 | 159046773 | 159047163 | PDFSTATSENSEMBLUCSC | 391 | 5 | 2028 | tss | 0.018595 | 0.877316 | AIM2 (margin: AIM2; RP11-520H16.4) | 8.58 | 7.94e-03 | 3.19e-02 |
|  | 14 | 24457893 | 24458345 | PDFSTATSENSEMBLUCSC | 453 | 10 | 12723 | island | 0.010836 | 0.720686 | DHRS4L2; DHRS4-AS1 (margin: DHRS4L2; DHRS4-AS1) | 8.58 | 1.08e-05 | 4.82e-04 |
|  | 19 | 38085706 | 38085810 | PDFSTATSENSEMBLUCSC | 105 | 6 | 20008 | gene;tss;island | 0.401397 | 0.001503 | ZNF540; ZNF571 (margin: ZNF571-AS1; ZNF540; ZNF571) | 8.57 | 2.16e-03 | 1.45e-02 |
|  | 16 | 765416 | 765670 | PDFSTATSENSEMBLUCSC | 255 | 4 | 14541 | gene;island | 0.529330 | 0.007591 | Not queried | 8.57 | 2.86e-02 | 6.12e-02 |
|  | 19 | 37569390 | 37569736 | PDFSTATSENSEMBLUCSC | 347 | 4 | 20007 | gene | 0.745607 | 0.032624 | Not queried | 8.55 | 2.86e-02 | 6.12e-02 |
|  | 16 | 69141250 | 69141478 | PDFSTATSENSEMBLUCSC | 229 | 7 | 14817 | gene;island | 0.181953 | 0.984359 | HAS3 (margin: HAS3; CHTF8) | 8.54 | 5.83e-04 | 6.47e-03 |
|  | 16 | 69141250 | 69141437 | PDFSTATSENSEMBLUCSC | 188 | 5 | 15326 | tss | 0.106971 | 0.977922 | HAS3 (margin: HAS3; CHTF8) | 8.54 | 7.94e-03 | 3.19e-02 |
|  | 8 | 67940640 | 67941178 | PDFSTATSENSEMBLUCSC | 539 | 10 | 40363 | island | 0.645954 | 0.007102 | PPP1R42 (margin: PPP1R42) | 8.54 | 1.08e-05 | 4.82e-04 |
|  | 22 | 45596948 | 45597035 | PDFSTATSENSEMBLUCSC | 88 | 7 | 27218 | tss | 0.140214 | 0.984315 | KIAA0930; MIR1249 (margin: KIAA0930; MIR1249) | 8.53 | 5.83e-04 | 6.47e-03 |
|  | 22 | 45596797 | 45597035 | PDFSTATSENSEMBLUCSC | 239 | 8 | 26899 | gene;island | 0.153226 | 0.983490 | KIAA0930; MIR1249 (margin: KIAA0930; MIR1249) | 8.53 | 1.55e-04 | 2.71e-03 |
|  | 4 | 47839876 | 47840162 | PDFSTATSENSEMBLUCSC | 287 | 6 | 31025 | island | 0.151099 | 0.973189 | CORIN; RP11-121C2.2 (margin: CORIN; RP11-121C2.2; NFXL1) | 8.52 | 2.16e-03 | 1.45e-02 |
|  | 1 | 50798944 | 50799316 | PDFSTATSENSEMBLUCSC | 373 | 4 | 3251 | island | 0.853546 | 0.019837 | Not queried | 8.51 | 2.86e-02 | 6.12e-02 |
|  | 19 | 2427708 | 2428122 | PDFSTATSENSEMBLUCSC | 415 | 5 | 21284 | island | 0.008211 | 0.513268 | TMPRSS9; TIMM13; LMNB2 (margin: TMPRSS9; TIMM13; LMNB2) | 8.51 | 7.94e-03 | 3.19e-02 |
|  | 5 | 42949542 | 42950152 | PDFSTATSENSEMBLUCSC | 611 | 6 | 32761 | island | 0.216285 | 0.985877 | (margin: ) | 8.51 | 2.16e-03 | 1.45e-02 |
|  | 19 | 31640642 | 31640711 | PDFSTATSENSEMBLUCSC | 70 | 5 | 20673 | tss | 0.178352 | 0.984016 | AC020952.1 (margin: AC020952.1) | 8.51 | 7.94e-03 | 3.19e-02 |
|  | 19 | 52097626 | 52097905 | PDFSTATSENSEMBLUCSC | 280 | 4 | 22343 | island | 0.003520 | 0.530575 | Not queried | 8.51 | 2.86e-02 | 6.12e-02 |
|  | 12 | 123215151 | 123215684 | PDFSTATSENSEMBLUCSC | 534 | 9 | 10086 | tss | 0.945788 | 0.019804 | HCAR1 (margin: HCAR1; RP11-324E6.9) | 8.50 | 4.11e-05 | 1.17e-03 |
|  | 4 | 187476326 | 187477065 | PDFSTATSENSEMBLUCSC | 740 | 11 | 31483 | island | 0.810564 | 0.010342 | RP11-215A19.2; MTNR1A (margin: RP11-215A19.2; MTNR1A) | 8.48 | 3.94e-04 | 5.86e-03 |
|  | 4 | 187476543 | 187477065 | PDFSTATSENSEMBLUCSC | 523 | 9 | 30707 | tss | 0.810564 | 0.010342 | RP11-215A19.2; MTNR1A (margin: RP11-215A19.2; MTNR1A) | 8.48 | 2.76e-03 | 1.78e-02 |
|  | 11 | 6342214 | 6342510 | PDFSTATSENSEMBLUCSC | 297 | 4 | 7107 | tss;island | 0.003810 | 0.518654 | Not queried | 8.48 | 2.86e-02 | 6.12e-02 |
|  | 16 | 29757344 | 29757461 | PDFSTATSENSEMBLUCSC | 118 | 4 | 15167 | tss | 0.043144 | 0.923600 | Not queried | 8.47 | 2.86e-02 | 6.12e-02 |
|  | 7 | 156332539 | 156332978 | PDFSTATSENSEMBLUCSC | 440 | 4 | 38015 | tss | 0.014498 | 0.805250 | Not queried | 8.46 | 2.86e-02 | 6.12e-02 |
|  | 12 | 107714673 | 107715146 | PDFSTATSENSEMBLUCSC | 474 | 7 | 9283 | gene;island | 0.076962 | 0.964106 | BTBD11 (margin: BTBD11) | 8.45 | 5.83e-04 | 6.47e-03 |
|  | 4 | 186456397 | 186457012 | PDFSTATSENSEMBLUCSC | 616 | 9 | 31477 | island | 0.836308 | 0.009316 | PDLIM3 (margin: PDLIM3) | 8.44 | 4.11e-05 | 1.17e-03 |
|  | 11 | 94278324 | 94278603 | PDFSTATSENSEMBLUCSC | 280 | 8 | 6867 | gene;island | 0.118704 | 0.979869 | PIWIL4; FUT4; RP11-867G2.8 (margin: PIWIL4; FUT4; RP11-867G2.8) | 8.43 | 1.55e-04 | 2.71e-03 |
|  | 17 | 9940004 | 9940321 | PDFSTATSENSEMBLUCSC | 318 | 4 | 16441 | gene | 0.010258 | 0.741770 | Not queried | 8.43 | 2.86e-02 | 6.12e-02 |
|  | 10 | 128994432 | 128994702 | PDFSTATSENSEMBLUCSC | 271 | 6 | 5434 | tss | 0.739762 | 0.007243 | DOCK1; FAM196A (margin: DOCK1; FAM196A) | 8.43 | 2.16e-03 | 1.45e-02 |
|  | 19 | 48896922 | 48897279 | PDFSTATSENSEMBLUCSC | 358 | 6 | 20914 | tss;island | 0.834172 | 0.008599 | GRIN2D (margin: GRIN2D; KDELR1) | 8.43 | 2.16e-03 | 1.45e-02 |
|  | 2 | 119603383 | 119603969 | PDFSTATSENSEMBLUCSC | 587 | 5 | 22841 | gene;island | 0.059361 | 0.976684 | EN1 (margin: RP11-19E11.1; EN1) | 8.43 | 7.94e-03 | 3.19e-02 |
|  | 6 | 76059450 | 76059773 | PDFSTATSENSEMBLUCSC | 324 | 6 | 34396 | gene | 0.741377 | 0.029618 | FILIP1 (margin: RP11-415D17.3; FILIP1) | 8.43 | 4.33e-03 | 2.52e-02 |
|  | 13 | 78272372 | 78272639 | PDFSTATSENSEMBLUCSC | 268 | 7 | 11789 | island | 0.656925 | 0.007208 | SLAIN1; MIR3665 (margin: SLAIN1; MIR3665) | 8.42 | 5.83e-04 | 6.47e-03 |
|  | 12 | 81471867 | 81472177 | PDFSTATSENSEMBLUCSC | 311 | 6 | 9228 | gene | 0.676889 | 0.007213 | ACSS3 (margin: ACSS3) | 8.42 | 2.16e-03 | 1.45e-02 |
|  | 5 | 63802106 | 63802412 | PDFSTATSENSEMBLUCSC | 307 | 5 | 32089 | tss | 0.906224 | 0.021361 | RGS7BP (margin: RGS7BP) | 8.42 | 7.94e-03 | 3.19e-02 |
|  | 3 | 72788183 | 72788482 | PDFSTATSENSEMBLUCSC | 300 | 4 | 29282 | island | 0.823777 | 0.030353 | Not queried | 8.41 | 2.86e-02 | 6.12e-02 |
|  | 1 | 57111099 | 57111511 | PDFSTATSENSEMBLUCSC | 413 | 8 | 553 | gene;island | 0.678122 | 0.009737 | PRKAA2; PPAP2B (margin: PRKAA2; PPAP2B) | 8.40 | 1.55e-04 | 2.71e-03 |
|  | 22 | 27053195 | 27053470 | PDFSTATSENSEMBLUCSC | 276 | 10 | 27037 | tss | 0.218240 | 0.988351 | MIAT (margin: MIAT; ISCA2P1; CTA-373H7.7) | 8.40 | 1.08e-05 | 4.82e-04 |
|  | 3 | 49395138 | 49395303 | PDFSTATSENSEMBLUCSC | 166 | 4 | 27780 | gene;island | 0.243055 | 0.967755 | Not queried | 8.39 | 2.86e-02 | 6.12e-02 |
|  | 19 | 9434309 | 9435206 | PDFSTATSENSEMBLUCSC | 898 | 13 | 21496 | island | 0.047853 | 0.872710 | ZNF559; ZNF177 (margin: ZNF559; ZNF177) | 8.39 | 1.92e-07 | 2.60e-05 |
|  | 8 | 120651190 | 120651398 | PDFSTATSENSEMBLUCSC | 209 | 4 | 39886 | tss | 0.062873 | 0.913937 | Not queried | 8.39 | 2.86e-02 | 6.12e-02 |
|  | 3 | 113251061 | 113251417 | PDFSTATSENSEMBLUCSC | 357 | 5 | 29366 | island | 0.109499 | 0.000906 | SIDT1 (margin: SIDT1) | 8.38 | 7.94e-03 | 3.19e-02 |
|  | 19 | 3136019 | 3136097 | PDFSTATSENSEMBLUCSC | 79 | 4 | 20340 | tss | 0.017877 | 0.824607 | Not queried | 8.37 | 2.86e-02 | 6.12e-02 |
|  | 2 | 88849457 | 88849830 | PDFSTATSENSEMBLUCSC | 374 | 4 | 24503 | island | 0.904798 | 0.029471 | Not queried | 8.37 | 2.86e-02 | 6.12e-02 |
|  | 1 | 55267006 | 55267293 | PDFSTATSENSEMBLUCSC | 288 | 7 | 1648 | tss | 0.009138 | 0.857175 | TTC22 (margin: RP11-67L3.2; C1orf177; TTC22) | 8.37 | 5.83e-04 | 6.47e-03 |
|  | 1 | 8271918 | 8272277 | PDFSTATSENSEMBLUCSC | 360 | 5 | 2634 | island | 0.008565 | 0.622882 | RP11-431K24.3 (margin: RP11-431K24.3; RNU1-7P; RP11-431K24.4) | 8.37 | 7.94e-03 | 3.19e-02 |
|  | 19 | 54177144 | 54177261 | PDFSTATSENSEMBLUCSC | 118 | 4 | 21034 | tss | 0.044697 | 0.919274 | Not queried | 8.36 | 5.71e-02 | 1.00e-01 |
|  | 6 | 39281421 | 39281885 | PDFSTATSENSEMBLUCSC | 465 | 5 | 34325 | gene;island | 0.090577 | 0.970292 | KCNK17; KCNK16 (margin: KCNK17; KCNK16) | 8.36 | 7.94e-03 | 3.19e-02 |
|  | 5 | 175108269 | 175108429 | PDFSTATSENSEMBLUCSC | 161 | 4 | 31907 | gene;tss | 0.013245 | 0.892671 | Not queried | 8.36 | 2.86e-02 | 6.12e-02 |
|  | 14 | 61116048 | 61116506 | PDFSTATSENSEMBLUCSC | 459 | 7 | 12907 | island | 0.004193 | 0.548357 | SIX1 (margin: RP11-1042B17.5; SIX1) | 8.35 | 5.83e-04 | 6.47e-03 |
|  | 14 | 61116211 | 61116506 | PDFSTATSENSEMBLUCSC | 296 | 5 | 12439 | tss | 0.004213 | 0.554423 | SIX1 (margin: RP11-1042B17.5; SIX1) | 8.35 | 7.94e-03 | 3.19e-02 |
|  | 11 | 133800685 | 133800913 | PDFSTATSENSEMBLUCSC | 229 | 4 | 6986 | gene;island | 0.084006 | 0.969427 | Not queried | 8.35 | 2.86e-02 | 6.12e-02 |
|  | 10 | 128994297 | 128994702 | PDFSTATSENSEMBLUCSC | 406 | 7 | 4818 | gene;island | 0.736439 | 0.008229 | DOCK1; FAM196A (margin: DOCK1; FAM196A) | 8.34 | 5.83e-04 | 6.47e-03 |
|  | 8 | 13424080 | 13424269 | PDFSTATSENSEMBLUCSC | 190 | 5 | 39561 | tss | 0.481329 | 0.004359 | C8orf48; RP11-145O15.3 (margin: C8orf48; RP11-145O15.3) | 8.34 | 7.94e-03 | 3.19e-02 |
|  | 5 | 138861832 | 138861941 | PDFSTATSENSEMBLUCSC | 110 | 4 | 31777 | gene | 0.004851 | 0.636394 | Not queried | 8.34 | 2.86e-02 | 6.12e-02 |
|  | 11 | 64014518 | 64014598 | PDFSTATSENSEMBLUCSC | 81 | 5 | 7390 | tss;island | 0.461853 | 0.013209 | RP11-783K16.5; PPP1R14B; RP11-783K16.13 (margin: VEGFB; FKBP2; RP11-783K16.5; PLCB3; PPP1R14B; RP11-783K16.13) | 8.34 | 7.94e-03 | 3.19e-02 |
|  | 22 | 36044151 | 36044328 | PDFSTATSENSEMBLUCSC | 178 | 4 | 27103 | tss | 0.167621 | 0.984358 | Not queried | 8.33 | 2.86e-02 | 6.12e-02 |
|  | 11 | 2170870 | 2171184 | PDFSTATSENSEMBLUCSC | 315 | 5 | 7059 | tss | 0.031741 | 0.753333 | IGF2-AS; IGF2; INS-IGF2 (margin: IGF2-AS; IGF2; INS-IGF2; INS) | 8.33 | 7.94e-03 | 3.19e-02 |
|  | 1 | 179557438 | 179558114 | PDFSTATSENSEMBLUCSC | 677 | 9 | 3999 | island | 0.099654 | 0.931889 | RP11-545A16.3; RP11-545A16.4 (margin: RNU5F-2P; RP11-545A16.3; RP11-545A16.4; TDRD5) | 8.31 | 2.76e-03 | 1.78e-02 |
|  | 22 | 27053195 | 27053934 | PDFSTATSENSEMBLUCSC | 740 | 15 | 27379 | island | 0.211227 | 0.988579 | MIAT (margin: MIAT; ISCA2P1; CTA-373H7.7) | 8.31 | 5.16e-08 | 9.48e-06 |
|  | 19 | 11531294 | 11531469 | PDFSTATSENSEMBLUCSC | 176 | 4 | 20472 | tss | 0.676051 | 0.022362 | Not queried | 8.31 | 2.86e-02 | 6.12e-02 |
|  | 18 | 55862577 | 55862872 | PDFSTATSENSEMBLUCSC | 296 | 7 | 19120 | gene;island | 0.115909 | 0.976980 | NEDD4L; RP11-718I15.1 (margin: NEDD4L; RP11-718I15.1) | 8.31 | 5.83e-04 | 6.47e-03 |
|  | 2 | 1417109 | 1417431 | PDFSTATSENSEMBLUCSC | 323 | 5 | 23157 | tss | 0.038387 | 0.942360 | Not queried | 8.29 | 3.17e-02 | 6.50e-02 |
|  | 11 | 67232269 | 67232511 | PDFSTATSENSEMBLUCSC | 243 | 4 | 6759 | gene;island | 0.804169 | 0.014115 | Not queried | 8.28 | 2.86e-02 | 6.12e-02 |
|  | 19 | 12267308 | 12267796 | PDFSTATSENSEMBLUCSC | 489 | 8 | 21595 | island | 0.025189 | 0.906795 | ZNF625-ZNF20; ZNF625 (margin: ZNF136; ZNF625-ZNF20; ZNF625) | 8.28 | 1.55e-04 | 2.71e-03 |
|  | 13 | 53425635 | 53426032 | PDFSTATSENSEMBLUCSC | 398 | 5 | 11764 | island | 0.386905 | 0.001581 | (margin: PCDH8) | 8.28 | 7.94e-03 | 3.19e-02 |
|  | 4 | 30721997 | 30722274 | PDFSTATSENSEMBLUCSC | 278 | 4 | 30970 | island | 0.004283 | 0.476841 | Not queried | 8.28 | 2.86e-02 | 6.12e-02 |
|  | 8 | 23021325 | 23021690 | PDFSTATSENSEMBLUCSC | 366 | 6 | 40146 | island | 0.136673 | 0.976092 | TNFRSF10D (margin: TNFRSF10D) | 8.27 | 2.16e-03 | 1.45e-02 |
|  | 12 | 6560689 | 6561007 | PDFSTATSENSEMBLUCSC | 319 | 7 | 9511 | tss | 0.015785 | 0.898030 | CD27; TAPBPL; CD27-AS1 (margin: CD27; TAPBPL; CD27-AS1; VAMP1) | 8.27 | 5.83e-04 | 6.47e-03 |
|  | 13 | 39358777 | 39358893 | PDFSTATSENSEMBLUCSC | 117 | 4 | 11140 | gene | 0.120448 | 0.977525 | Not queried | 8.27 | 2.86e-02 | 6.12e-02 |
|  | 7 | 43288635 | 43288889 | PDFSTATSENSEMBLUCSC | 255 | 4 | 36981 | gene;island | 0.016599 | 0.890493 | Not queried | 8.26 | 2.86e-02 | 6.12e-02 |
|  | 6 | 5996893 | 5997375 | PDFSTATSENSEMBLUCSC | 483 | 6 | 35404 | island | 0.023421 | 0.836363 | NRN1 (margin: NRN1) | 8.26 | 2.16e-03 | 1.45e-02 |
|  | 1 | 31158026 | 31158299 | PDFSTATSENSEMBLUCSC | 274 | 5 | 2964 | island | 0.875772 | 0.023554 | (margin: ) | 8.25 | 7.94e-03 | 3.19e-02 |
|  | 11 | 2170831 | 2171184 | PDFSTATSENSEMBLUCSC | 354 | 6 | 6449 | gene | 0.037514 | 0.749320 | IGF2-AS; IGF2; INS-IGF2 (margin: IGF2-AS; IGF2; INS-IGF2; INS) | 8.24 | 2.16e-03 | 1.45e-02 |
|  | 1 | 109941043 | 109941201 | PDFSTATSENSEMBLUCSC | 159 | 5 | 1802 | tss;island | 0.946062 | 0.037674 | SORT1; PSMA5 (margin: SORT1; PSMA5) | 8.24 | 7.94e-03 | 3.19e-02 |
|  | 19 | 37569236 | 37569736 | PDFSTATSENSEMBLUCSC | 501 | 11 | 21950 | island | 0.802375 | 0.006859 | ZNF420; CTD-2293H3.2 (margin: ZNF420; CTD-2293H3.2) | 8.23 | 2.84e-06 | 1.96e-04 |
|  | 19 | 37569236 | 37569352 | PDFSTATSENSEMBLUCSC | 117 | 7 | 20733 | tss | 0.802375 | 0.006859 | ZNF420; CTD-2293H3.2 (margin: ZNF420; CTD-2293H3.2) | 8.23 | 5.83e-04 | 6.47e-03 |
|  | 21 | 47401109 | 47401492 | PDFSTATSENSEMBLUCSC | 384 | 5 | 26598 | tss;island | 0.326367 | 0.009284 | Not queried | 8.23 | 3.17e-02 | 6.50e-02 |
|  | 20 | 43439387 | 43439688 | PDFSTATSENSEMBLUCSC | 302 | 5 | 25764 | tss;island | 0.735318 | 0.024146 | Not queried | 8.23 | 4.21e-01 | 4.59e-01 |
|  | 6 | 87647370 | 87647644 | PDFSTATSENSEMBLUCSC | 275 | 5 | 34408 | gene;island | 0.926751 | 0.015316 | HTR1E (margin: HTR1E) | 8.21 | 7.94e-03 | 3.19e-02 |
|  | 19 | 12476508 | 12476577 | PDFSTATSENSEMBLUCSC | 70 | 4 | 20494 | tss | 0.003232 | 0.402869 | Not queried | 8.21 | 2.86e-02 | 6.12e-02 |
|  | 8 | 61193715 | 61194292 | PDFSTATSENSEMBLUCSC | 578 | 11 | 40329 | island | 0.823630 | 0.013995 | CA8 (margin: CA8) | 8.21 | 2.84e-06 | 1.96e-04 |
|  | 8 | 644521 | 644878 | PDFSTATSENSEMBLUCSC | 358 | 5 | 39164 | gene | 0.626278 | 0.006045 | Not queried | 8.21 | 1.59e-02 | 5.36e-02 |
|  | 7 | 94537584 | 94537893 | PDFSTATSENSEMBLUCSC | 310 | 9 | 37094 | gene;island | 0.823709 | 0.011077 | PPP1R9A (margin: PPP1R9A) | 8.20 | 4.11e-05 | 1.17e-03 |
|  | 16 | 88943191 | 88943641 | PDFSTATSENSEMBLUCSC | 451 | 6 | 14920 | gene;island | 0.178678 | 0.972056 | CBFA2T3 (margin: RP11-830F9.5; PABPN1L; CBFA2T3) | 8.20 | 2.16e-03 | 1.45e-02 |
|  | 1 | 2222253 | 2222674 | PDFSTATSENSEMBLUCSC | 422 | 5 | 72 | gene;island | 0.114408 | 0.954061 | SKI (margin: SKI) | 8.20 | 7.94e-03 | 3.19e-02 |
|  | 17 | 78800574 | 78800806 | PDFSTATSENSEMBLUCSC | 233 | 5 | 16875 | gene;island | 0.109899 | 0.972621 | RPTOR (margin: RPTOR) | 8.19 | 7.94e-03 | 3.19e-02 |
|  | 10 | 117884825 | 117885028 | PDFSTATSENSEMBLUCSC | 204 | 4 | 4771 | gene;island | 0.179376 | 0.978074 | Not queried | 8.19 | 2.86e-02 | 6.12e-02 |
|  | 1 | 55266578 | 55267293 | PDFSTATSENSEMBLUCSC | 716 | 10 | 3302 | island | 0.019140 | 0.895996 | TTC22 (margin: RP11-67L3.2; C1orf177; TTC22) | 8.19 | 1.08e-05 | 4.82e-04 |
|  | 4 | 164253207 | 164253656 | PDFSTATSENSEMBLUCSC | 450 | 4 | 30167 | gene | 0.543658 | 0.005611 | Not queried | 8.18 | 2.86e-02 | 6.12e-02 |
|  | 6 | 49755009 | 49755190 | PDFSTATSENSEMBLUCSC | 182 | 4 | 35028 | tss | 0.022477 | 0.846515 | Not queried | 8.18 | 2.86e-02 | 6.12e-02 |
|  | 1 | 32714023 | 32714308 | PDFSTATSENSEMBLUCSC | 286 | 5 | 392 | gene;island | 0.871854 | 0.028445 | FAM167B (margin: FAM167B; LCK; MTMR9LP) | 8.18 | 7.94e-03 | 3.19e-02 |
|  | 14 | 95693784 | 95693981 | PDFSTATSENSEMBLUCSC | 198 | 4 | 12197 | gene | 0.950925 | 0.111533 | Not queried | 8.18 | 2.86e-02 | 6.12e-02 |
|  | 4 | 13546145 | 13546673 | PDFSTATSENSEMBLUCSC | 529 | 9 | 30304 | tss | 0.739958 | 0.017081 | NKX3-2; AC006445.8 (margin: NKX3-2; AC006445.8) | 8.18 | 7.82e-04 | 8.17e-03 |
|  | 19 | 5340582 | 5340832 | PDFSTATSENSEMBLUCSC | 251 | 4 | 21403 | island | 0.365576 | 0.006175 | Not queried | 8.17 | 2.86e-02 | 6.12e-02 |
|  | 1 | 82267923 | 82268281 | PDFSTATSENSEMBLUCSC | 359 | 5 | 597 | gene;island | 0.479268 | 0.007504 | LPHN2 (margin: LPHN2) | 8.17 | 7.94e-03 | 3.19e-02 |
|  | 3 | 53190593 | 53190763 | PDFSTATSENSEMBLUCSC | 171 | 4 | 29198 | island | 0.874551 | 0.028373 | Not queried | 8.17 | 2.86e-02 | 6.12e-02 |
|  | 13 | 43566401 | 43566642 | PDFSTATSENSEMBLUCSC | 242 | 9 | 11414 | tss | 0.085550 | 0.970030 | EPSTI1 (margin: EPSTI1) | 8.17 | 4.11e-05 | 1.17e-03 |
|  | 8 | 121824218 | 121824929 | PDFSTATSENSEMBLUCSC | 712 | 10 | 40565 | island | 0.726989 | 0.007600 | RP11-713M15.2; SNTB1 (margin: RP11-713M15.2; SNTB1) | 8.17 | 1.05e-03 | 1.08e-02 |
|  | 11 | 128392383 | 128392708 | PDFSTATSENSEMBLUCSC | 326 | 4 | 6965 | gene;tss;island | 0.007349 | 0.687912 | Not queried | 8.17 | 2.86e-02 | 6.12e-02 |
|  | 17 | 80806135 | 80806435 | PDFSTATSENSEMBLUCSC | 301 | 5 | 16960 | gene | 0.078442 | 0.968522 | TBCD (margin: TBCD; ZNF750) | 8.17 | 7.94e-03 | 3.19e-02 |
|  | 12 | 15374457 | 15374609 | PDFSTATSENSEMBLUCSC | 153 | 6 | 9590 | tss | 0.687846 | 0.012008 | RERG (margin: RERG) | 8.17 | 2.16e-03 | 1.45e-02 |
|  | 15 | 40697714 | 40698044 | PDFSTATSENSEMBLUCSC | 331 | 4 | 13292 | gene | 0.383874 | 0.002255 | Not queried | 8.16 | 2.86e-02 | 6.12e-02 |
|  | 16 | 88700622 | 88700916 | PDFSTATSENSEMBLUCSC | 295 | 4 | 16211 | island | 0.967711 | 0.082549 | Not queried | 8.15 | 2.86e-02 | 6.12e-02 |
|  | 1 | 20811132 | 20811408 | PDFSTATSENSEMBLUCSC | 277 | 4 | 288 | gene;island | 0.021400 | 0.862064 | Not queried | 8.14 | 2.86e-02 | 6.12e-02 |
|  | 6 | 29894619 | 29894908 | PDFSTATSENSEMBLUCSC | 290 | 11 | 34758 | tss | 0.038503 | 0.917549 | HLA-K; HCG4B (margin: HLA-K; HLA-U; HCG4B) | 8.14 | 5.67e-06 | 3.54e-04 |
|  | 6 | 28411279 | 28411423 | PDFSTATSENSEMBLUCSC | 145 | 6 | 34742 | tss | 0.100601 | 0.944974 | ZSCAN23 (margin: COX11P1; ZSCAN23) | 8.13 | 2.16e-03 | 1.45e-02 |
|  | 4 | 8582101 | 8582300 | PDFSTATSENSEMBLUCSC | 200 | 7 | 30922 | island | 0.657911 | 0.010474 | GPR78 (margin: GPR78) | 8.13 | 5.83e-04 | 6.47e-03 |
|  | 15 | 89438611 | 89438964 | PDFSTATSENSEMBLUCSC | 354 | 7 | 14423 | island | 0.107684 | 0.965608 | HAPLN3 (margin: HAPLN3; MFGE8) | 8.13 | 5.83e-04 | 6.47e-03 |
|  | 19 | 35645556 | 35645712 | PDFSTATSENSEMBLUCSC | 157 | 7 | 20701 | tss;island | 0.002698 | 0.594460 | FXYD7; FXYD5 (margin: FXYD1; CTD-2527I21.4; FXYD7; FXYD5) | 8.13 | 5.83e-04 | 6.47e-03 |
|  | 10 | 530635 | 531584 | PDFSTATSENSEMBLUCSC | 950 | 12 | 4441 | gene;island | 0.099031 | 0.974379 | DIP2C (margin: DIP2C) | 8.12 | 7.40e-07 | 7.21e-05 |
|  | 14 | 91580380 | 91580613 | PDFSTATSENSEMBLUCSC | 234 | 6 | 12176 | gene | 0.033793 | 0.892250 | C14orf159 (margin: C14orf159) | 8.12 | 2.16e-03 | 1.45e-02 |
|  | 6 | 33040535 | 33040914 | PDFSTATSENSEMBLUCSC | 380 | 5 | 34183 | gene | 0.126712 | 0.975667 | Not queried | 8.11 | 3.17e-02 | 6.50e-02 |
|  | 19 | 50194120 | 50194252 | PDFSTATSENSEMBLUCSC | 133 | 5 | 20961 | tss;island | 0.202623 | 0.977257 | PRMT1; ADM5; CPT1C; CTB-33G10.6 (margin: PRMT1; MIR5088; ADM5; CPT1C; CTB-33G10.6) | 8.11 | 7.94e-03 | 3.19e-02 |
|  | 4 | 186456397 | 186456682 | PDFSTATSENSEMBLUCSC | 286 | 4 | 30211 | gene | 0.565050 | 0.008499 | Not queried | 8.11 | 2.86e-02 | 6.12e-02 |
|  | 10 | 119302050 | 119302318 | PDFSTATSENSEMBLUCSC | 269 | 8 | 4785 | gene;island | 0.132328 | 0.979747 | EMX2; EMX2OS (margin: EMX2; EMX2OS) | 8.10 | 1.55e-04 | 2.71e-03 |
|  | 21 | 43188171 | 43188367 | PDFSTATSENSEMBLUCSC | 197 | 4 | 26560 | tss;island | 0.004435 | 0.462394 | Not queried | 8.10 | 2.86e-02 | 6.12e-02 |
|  | 2 | 620044 | 620310 | PDFSTATSENSEMBLUCSC | 267 | 4 | 24008 | island | 0.165146 | 0.964067 | Not queried | 8.10 | 5.71e-02 | 1.00e-01 |
|  | 8 | 61193970 | 61194292 | PDFSTATSENSEMBLUCSC | 323 | 9 | 39724 | tss | 0.823630 | 0.015638 | CA8 (margin: CA8) | 8.10 | 4.11e-05 | 1.17e-03 |
|  | 5 | 128796595 | 128796754 | PDFSTATSENSEMBLUCSC | 160 | 4 | 31741 | gene;island | 0.368480 | 0.004377 | Not queried | 8.10 | 2.86e-02 | 6.12e-02 |
|  | 11 | 87908558 | 87908938 | PDFSTATSENSEMBLUCSC | 381 | 9 | 8719 | island | 0.069801 | 0.964408 | MIR3166; RAB38 (margin: MIR3166; RAB38) | 8.09 | 4.11e-05 | 1.17e-03 |
|  | 1 | 153508511 | 153508875 | PDFSTATSENSEMBLUCSC | 365 | 10 | 3763 | island | 0.005899 | 0.606352 | BX470102.3; S100A6; S100A5 (margin: BX470102.3; S100A6; S100A5; S100A4; S100A3) | 8.08 | 1.08e-05 | 4.82e-04 |
|  | 1 | 3007701 | 3008033 | PDFSTATSENSEMBLUCSC | 333 | 4 | 98 | gene | 0.242998 | 0.982965 | Not queried | 8.08 | 2.86e-02 | 6.12e-02 |
|  | 7 | 93520074 | 93520566 | PDFSTATSENSEMBLUCSC | 493 | 13 | 37715 | tss | 0.839425 | 0.031040 | GNGT1; AC002076.10; TFPI2 (margin: GNGT1; AC002076.10; TFPI2) | 8.08 | 1.92e-07 | 2.60e-05 |
|  | 11 | 124311131 | 124311423 | PDFSTATSENSEMBLUCSC | 293 | 5 | 7782 | tss | 0.116300 | 0.978830 | OR8B8 (margin: OR8B8) | 8.08 | 7.94e-03 | 3.19e-02 |
|  | 2 | 220083306 | 220083951 | PDFSTATSENSEMBLUCSC | 646 | 10 | 25110 | island | 0.753240 | 0.010149 | ABCB6; ATG9A (margin: ZFAND2B; ANKZF1; ABCB6; ATG9A; AC068946.1) | 8.07 | 1.08e-05 | 4.82e-04 |
|  | 2 | 220083696 | 220083951 | PDFSTATSENSEMBLUCSC | 256 | 8 | 23895 | tss | 0.770464 | 0.010149 | ABCB6; ATG9A (margin: ZFAND2B; ANKZF1; ABCB6; ATG9A; AC068946.1) | 8.07 | 1.55e-04 | 2.71e-03 |
|  | 11 | 120530774 | 120530973 | PDFSTATSENSEMBLUCSC | 200 | 5 | 7764 | tss | 0.046716 | 0.935960 | GRIK4 (margin: GRIK4) | 8.06 | 7.94e-03 | 3.19e-02 |
|  | 4 | 184922382 | 184922646 | PDFSTATSENSEMBLUCSC | 265 | 5 | 30197 | gene | 0.211735 | 0.985779 | Not queried | 8.06 | 3.17e-02 | 6.50e-02 |
|  | 19 | 34112683 | 34113010 | PDFSTATSENSEMBLUCSC | 328 | 4 | 21879 | island | 0.737335 | 0.015480 | Not queried | 8.06 | 2.86e-02 | 6.12e-02 |
|  | 1 | 53308597 | 53308908 | PDFSTATSENSEMBLUCSC | 312 | 4 | 535 | gene;island | 0.780585 | 0.013724 | Not queried | 8.06 | 2.86e-02 | 6.12e-02 |
|  | 7 | 145812842 | 145813066 | PDFSTATSENSEMBLUCSC | 225 | 5 | 37958 | tss;island | 0.894397 | 0.031559 | Not queried | 8.05 | 1.51e-01 | 2.11e-01 |
|  | 16 | 604802 | 605347 | PDFSTATSENSEMBLUCSC | 546 | 6 | 15462 | island | 0.127057 | 0.972312 | CAPN15; LA16c-366D1.3 (margin: CAPN15; MIR3176; C16orf11; PIGQ; NHLRC4; LA16c-366D1.3) | 8.05 | 2.16e-03 | 1.45e-02 |
|  | 15 | 99789622 | 99789855 | PDFSTATSENSEMBLUCSC | 234 | 5 | 14503 | island | 0.003573 | 0.426038 | LRRC28; TTC23 (margin: LRRC28; AC022819.1; HSP90B2P; TTC23) | 8.05 | 7.94e-03 | 3.19e-02 |
|  | 19 | 57610536 | 57610870 | PDFSTATSENSEMBLUCSC | 335 | 4 | 22456 | island | 0.957023 | 0.087846 | Not queried | 8.04 | 5.71e-02 | 1.00e-01 |
|  | 11 | 101000436 | 101000717 | PDFSTATSENSEMBLUCSC | 282 | 6 | 8755 | island | 0.312186 | 0.013126 | PGR (margin: PGR) | 8.03 | 2.16e-03 | 1.45e-02 |
|  | 19 | 50706387 | 50706874 | PDFSTATSENSEMBLUCSC | 488 | 7 | 20973 | tss;island | 0.918338 | 0.023526 | MYH14 (margin: MYH14) | 8.03 | 5.83e-04 | 6.47e-03 |
|  | 7 | 94537716 | 94537893 | PDFSTATSENSEMBLUCSC | 178 | 8 | 37723 | tss | 0.817289 | 0.010912 | PPP1R9A (margin: PPP1R9A) | 8.03 | 1.55e-04 | 2.71e-03 |
|  | 19 | 39523309 | 39523626 | PDFSTATSENSEMBLUCSC | 318 | 5 | 20764 | tss | 0.580014 | 0.005356 | Not queried | 8.03 | 1.59e-02 | 5.36e-02 |
|  | 6 | 146285286 | 146285836 | PDFSTATSENSEMBLUCSC | 551 | 11 | 35274 | tss;island | 0.003789 | 0.402435 | SHPRH (margin: SHPRH) | 8.03 | 8.51e-05 | 2.07e-03 |
|  | 5 | 140220686 | 140221106 | PDFSTATSENSEMBLUCSC | 421 | 6 | 31799 | gene;island | 0.841240 | 0.018679 | PCDHA1; PCDHA2; PCDHA3; PCDHA4; PCDHA5; PCDHA6; PCDHA7; PCDHA8 (margin: PCDHA1; PCDHA2; PCDHA3; PCDHA4; PCDHA5; PCDHA6; PCDHA7; PCDHA8; PCDHA9) | 8.02 | 2.16e-03 | 1.45e-02 |
|  | 11 | 818752 | 818917 | PDFSTATSENSEMBLUCSC | 166 | 5 | 7884 | island | 0.017277 | 0.544300 | PNPLA2 (margin: RPLP2; SNORA52; PNPLA2; EFCAB4A; PIDD; AP006621.8) | 8.02 | 7.94e-03 | 3.19e-02 |
|  | 16 | 67918484 | 67918965 | PDFSTATSENSEMBLUCSC | 482 | 8 | 16033 | island | 0.902547 | 0.031086 | EDC4; CTC-479C5.10; NRN1L (margin: EDC4; AC040162.1; CTC-479C5.10; NRN1L; PSKH1) | 8.02 | 1.86e-03 | 1.45e-02 |
|  | 1 | 3607097 | 3607425 | PDFSTATSENSEMBLUCSC | 329 | 7 | 138 | gene;island | 0.130280 | 0.979487 | TP73 (margin: TP73) | 8.01 | 5.83e-04 | 6.47e-03 |
|  | 7 | 27204663 | 27205658 | PDFSTATSENSEMBLUCSC | 996 | 17 | 38356 | island | 0.085212 | 0.964733 | HOXA9; RP1-170O19.20 (margin: HOXA-AS3; RP1-170O19.21; HOXA-AS4; HOXA7; HOXA9; RP1-170O19.20; MIR196B; HOXA10) | 8.01 | 8.57e-10 | 3.90e-07 |
|  | 2 | 97760606 | 97760749 | PDFSTATSENSEMBLUCSC | 144 | 7 | 23527 | tss;island | 0.008109 | 0.588898 | FAHD2B (margin: AC018892.9; RN7SL313P; FAHD2B) | 8.01 | 5.83e-04 | 6.47e-03 |
|  | 6 | 44265644 | 44265696 | PDFSTATSENSEMBLUCSC | 53 | 4 | 35011 | tss;island | 0.837790 | 0.023293 | Not queried | 8.00 | 2.86e-02 | 6.12e-02 |
|  | 8 | 110703888 | 110704289 | PDFSTATSENSEMBLUCSC | 402 | 6 | 40541 | island | 0.681649 | 0.008420 | SYBU (margin: SYBU) | 7.99 | 2.16e-03 | 1.45e-02 |
|  | 21 | 34638126 | 34638662 | PDFSTATSENSEMBLUCSC | 537 | 7 | 26519 | tss;island | 0.878568 | 0.020458 | Not queried | 7.98 | 2.59e-01 | 3.16e-01 |
|  | 7 | 45961772 | 45962236 | PDFSTATSENSEMBLUCSC | 465 | 13 | 37588 | tss;island | 0.840260 | 0.031424 | IGFBP3 (margin: IGFBP3) | 7.98 | 1.92e-07 | 2.60e-05 |
|  | 1 | 35220224 | 35220710 | PDFSTATSENSEMBLUCSC | 487 | 6 | 1478 | tss | 0.080881 | 0.961439 | GJB5; SMIM12 (margin: GJB5; GJB4; SMIM12; RP1-34M23.5) | 7.98 | 2.16e-03 | 1.45e-02 |
|  | 19 | 11531294 | 11531519 | PDFSTATSENSEMBLUCSC | 226 | 5 | 19840 | gene;island | 0.816227 | 0.026545 | RGL3; CCDC151 (margin: Y\_RNA; RGL3; CCDC151) | 7.97 | 7.94e-03 | 3.19e-02 |
|  | 10 | 44070149 | 44070200 | PDFSTATSENSEMBLUCSC | 52 | 4 | 5052 | tss | 0.807696 | 0.015111 | Not queried | 7.96 | 2.86e-02 | 6.12e-02 |
|  | 2 | 227700458 | 227700568 | PDFSTATSENSEMBLUCSC | 111 | 6 | 23928 | tss;island | 0.731950 | 0.021903 | RHBDD1 (margin: RHBDD1) | 7.96 | 2.16e-03 | 1.45e-02 |
|  | 5 | 77805886 | 77806312 | PDFSTATSENSEMBLUCSC | 427 | 4 | 31657 | gene;island | 0.970887 | 0.275052 | Not queried | 7.96 | 1.14e-01 | 1.68e-01 |
|  | 1 | 183155366 | 183155644 | PDFSTATSENSEMBLUCSC | 279 | 4 | 914 | gene | 0.073438 | 0.930400 | Not queried | 7.95 | 2.86e-02 | 6.12e-02 |
|  | 12 | 7282081 | 7282931 | PDFSTATSENSEMBLUCSC | 851 | 14 | 9546 | tss | 0.661019 | 0.010001 | CLSTN3; RBP5; RP11-273B20.1 (margin: C1RL-AS1; RNU6-485P; CLSTN3; RBP5; RP11-273B20.1) | 7.95 | 1.86e-05 | 7.98e-04 |
|  | 11 | 1471410 | 1471699 | PDFSTATSENSEMBLUCSC | 290 | 6 | 6420 | gene;island | 0.145169 | 0.968587 | BRSK2 (margin: BRSK2) | 7.94 | 2.16e-03 | 1.45e-02 |
|  | 15 | 99789622 | 99789777 | PDFSTATSENSEMBLUCSC | 156 | 4 | 13502 | gene | 0.004473 | 0.455974 | Not queried | 7.94 | 2.86e-02 | 6.12e-02 |
|  | 5 | 128796079 | 128796266 | PDFSTATSENSEMBLUCSC | 188 | 5 | 33074 | island | 0.567654 | 0.010943 | ADAMTS19; ADAMTS19-AS1 (margin: ADAMTS19; ADAMTS19-AS1) | 7.93 | 7.94e-03 | 3.19e-02 |
|  | 3 | 187456175 | 187456579 | PDFSTATSENSEMBLUCSC | 405 | 5 | 28078 | gene;island | 0.156983 | 0.988083 | BCL6 (margin: RP11-211G3.3; RP11-211G3.2; BCL6) | 7.92 | 7.94e-03 | 3.19e-02 |
|  | 1 | 12226913 | 12226961 | PDFSTATSENSEMBLUCSC | 49 | 4 | 1258 | tss | 0.193826 | 0.974516 | Not queried | 7.91 | 2.86e-02 | 6.12e-02 |
|  | 1 | 171810105 | 171810570 | PDFSTATSENSEMBLUCSC | 466 | 8 | 2093 | tss;island | 0.039977 | 0.833515 | DNM3 (margin: DNM3) | 7.90 | 1.55e-04 | 2.71e-03 |
|  | 7 | 138794401 | 138794752 | PDFSTATSENSEMBLUCSC | 352 | 9 | 38949 | island | 0.005235 | 0.674601 | ZC3HAV1 (margin: ZC3HAV1) | 7.90 | 4.11e-05 | 1.17e-03 |
|  | 3 | 48700269 | 48700498 | PDFSTATSENSEMBLUCSC | 230 | 8 | 29095 | island | 0.422085 | 0.005861 | RP11-148G20.1; CELSR3; NCKIPSD (margin: RP11-148G20.1; RP11-572O6.1; CELSR3; NCKIPSD) | 7.90 | 1.55e-04 | 2.71e-03 |
|  | 2 | 219857793 | 219858271 | PDFSTATSENSEMBLUCSC | 479 | 9 | 25100 | island | 0.253318 | 0.974547 | CRYBA2 (margin: AC097468.4; FEV; CRYBA2; MIR375; CCDC108) | 7.90 | 8.23e-05 | 2.06e-03 |
|  | 2 | 237080267 | 237080535 | PDFSTATSENSEMBLUCSC | 269 | 4 | 25226 | island | 0.298461 | 0.985265 | Not queried | 7.90 | 2.86e-02 | 6.12e-02 |
|  | 6 | 50810682 | 50810993 | PDFSTATSENSEMBLUCSC | 312 | 4 | 34367 | gene;island | 0.688417 | 0.015113 | Not queried | 7.89 | 2.86e-02 | 6.12e-02 |
|  | 19 | 1395365 | 1395646 | PDFSTATSENSEMBLUCSC | 282 | 4 | 21221 | island | 0.969283 | 0.087763 | Not queried | 7.89 | 2.86e-02 | 6.12e-02 |
|  | 2 | 176994498 | 176994764 | PDFSTATSENSEMBLUCSC | 267 | 4 | 22938 | gene | 0.006869 | 0.202359 | Not queried | 7.89 | 2.00e-01 | 2.55e-01 |
|  | 16 | 81526954 | 81527504 | PDFSTATSENSEMBLUCSC | 551 | 7 | 14854 | gene | 0.016507 | 0.868232 | CMIP (margin: CMIP) | 7.89 | 5.83e-04 | 6.47e-03 |
|  | 3 | 38071166 | 38071578 | PDFSTATSENSEMBLUCSC | 413 | 5 | 28224 | tss;island | 0.148634 | 0.986851 | Not queried | 7.88 | 1.59e-02 | 5.36e-02 |
|  | 13 | 99135543 | 99135804 | PDFSTATSENSEMBLUCSC | 262 | 6 | 11201 | gene | 0.116801 | 0.932018 | STK24 (margin: STK24) | 7.88 | 2.16e-03 | 1.45e-02 |
|  | 1 | 179560700 | 179561111 | PDFSTATSENSEMBLUCSC | 412 | 6 | 4000 | island | 0.631001 | 0.009640 | RP11-545A16.4; TDRD5 (margin: RP11-545A16.3; RP11-545A16.4; TDRD5) | 7.87 | 2.16e-03 | 1.45e-02 |
|  | 8 | 22926785 | 22926911 | PDFSTATSENSEMBLUCSC | 127 | 7 | 39595 | tss | 0.015330 | 0.496727 | RP11-875O11.2; TNFRSF10B; RP11-875O11.3 (margin: RP11-875O11.2; TNFRSF10B; RP11-875O11.3) | 7.87 | 1.17e-03 | 1.13e-02 |
|  | 10 | 98946673 | 98946943 | PDFSTATSENSEMBLUCSC | 271 | 4 | 5258 | tss;island | 0.129449 | 0.968908 | Not queried | 7.86 | 2.86e-02 | 6.12e-02 |
|  | 17 | 6899522 | 6899888 | PDFSTATSENSEMBLUCSC | 367 | 5 | 16380 | gene | 0.240198 | 0.977947 | ALOX12; AC027763.2; RP11-589P10.7; RP11-589P10.5 (margin: ALOX12; AC027763.2; RP11-589P10.7; RP11-589P10.5) | 7.86 | 7.94e-03 | 3.19e-02 |
|  | 5 | 57878188 | 57878526 | PDFSTATSENSEMBLUCSC | 339 | 7 | 32077 | tss;island | 0.785558 | 0.012728 | RAB3C (margin: RAB3C) | 7.85 | 5.83e-04 | 6.47e-03 |
|  | 3 | 46448753 | 46449100 | PDFSTATSENSEMBLUCSC | 348 | 5 | 27748 | gene | 0.021420 | 0.861580 | ACKR5; RP11-24F11.2 (margin: ACKR5; RP11-24F11.2) | 7.85 | 7.94e-03 | 3.19e-02 |
|  | 13 | 78272372 | 78272443 | PDFSTATSENSEMBLUCSC | 72 | 6 | 11486 | tss | 0.656605 | 0.007721 | SLAIN1; MIR3665 (margin: SLAIN1; MIR3665) | 7.84 | 2.16e-03 | 1.45e-02 |
|  | 11 | 120008025 | 120008607 | PDFSTATSENSEMBLUCSC | 583 | 6 | 6943 | gene;island | 0.062986 | 0.928554 | TRIM29 (margin: TRIM29) | 7.83 | 2.16e-03 | 1.45e-02 |
|  | 18 | 32398024 | 32398287 | PDFSTATSENSEMBLUCSC | 264 | 4 | 19086 | gene;tss | 0.333285 | 0.987138 | Not queried | 7.83 | 5.71e-02 | 1.00e-01 |
|  | 10 | 77163316 | 77163401 | PDFSTATSENSEMBLUCSC | 86 | 6 | 4643 | gene;tss;island | 0.009462 | 0.568389 | ZNF503-AS2; ZNF503 (margin: ZNF503-AS2; RP11-399K21.11; ZNF503) | 7.82 | 2.16e-03 | 1.45e-02 |
|  | 2 | 228029433 | 228029856 | PDFSTATSENSEMBLUCSC | 424 | 8 | 23058 | gene;tss;island | 0.750292 | 0.014867 | COL4A3; COL4A4 (margin: COL4A3; COL4A4) | 7.81 | 1.55e-04 | 2.71e-03 |
|  | 6 | 31275551 | 31275881 | PDFSTATSENSEMBLUCSC | 331 | 11 | 35762 | island | 0.689049 | 0.014808 | XXbac-BPG248L24.10 (margin: XXbac-BPG248L24.13; XXbac-BPG248L24.10) | 7.81 | 2.84e-06 | 1.96e-04 |
|  | 22 | 30901532 | 30901886 | PDFSTATSENSEMBLUCSC | 355 | 7 | 27413 | island | 0.052521 | 0.916292 | SEC14L4 (margin: SEC14L4) | 7.80 | 5.83e-04 | 6.47e-03 |
|  | 12 | 25538177 | 25538380 | PDFSTATSENSEMBLUCSC | 204 | 4 | 10292 | island | 0.954747 | 0.100114 | Not queried | 7.80 | 2.86e-02 | 6.12e-02 |
|  | 13 | 37006063 | 37006340 | PDFSTATSENSEMBLUCSC | 278 | 7 | 11132 | gene;tss;island | 0.785718 | 0.019720 | CCNA1 (margin: CCNA1) | 7.80 | 5.83e-04 | 6.47e-03 |
|  | 9 | 80912058 | 80912260 | PDFSTATSENSEMBLUCSC | 203 | 4 | 40768 | gene;island | 0.032623 | 0.534617 | Not queried | 7.80 | 2.86e-02 | 6.12e-02 |
|  | 1 | 204182957 | 204183116 | PDFSTATSENSEMBLUCSC | 160 | 4 | 958 | gene | 0.830768 | 0.023058 | Not queried | 7.79 | 2.86e-02 | 6.12e-02 |
|  | 1 | 61542546 | 61542689 | PDFSTATSENSEMBLUCSC | 144 | 5 | 1661 | tss;island | 0.675588 | 0.009963 | NFIA (margin: NFIA; AC096534.1) | 7.79 | 7.94e-03 | 3.19e-02 |
|  | 5 | 175224563 | 175224721 | PDFSTATSENSEMBLUCSC | 159 | 4 | 31908 | gene;island | 0.767202 | 0.017873 | Not queried | 7.79 | 2.86e-02 | 6.12e-02 |
|  | 3 | 69915189 | 69915449 | PDFSTATSENSEMBLUCSC | 261 | 4 | 27856 | gene | 0.019766 | 0.863707 | Not queried | 7.78 | 2.86e-02 | 6.12e-02 |
|  | 17 | 30592997 | 30593034 | PDFSTATSENSEMBLUCSC | 38 | 5 | 17253 | tss;island | 0.657925 | 0.008674 | RHBDL3 (margin: RHBDL3) | 7.78 | 7.94e-03 | 3.19e-02 |
|  | 1 | 3663902 | 3664084 | PDFSTATSENSEMBLUCSC | 183 | 4 | 1187 | tss | 0.092310 | 0.934966 | Not queried | 7.78 | 2.86e-02 | 6.12e-02 |
|  | 1 | 248100183 | 248100614 | PDFSTATSENSEMBLUCSC | 432 | 10 | 4422 | island | 0.757900 | 0.018502 | OR2L13; OR2X1P; RP11-438H8.8 (margin: OR2AJ1; OR2L13; OR2X1P; OR2L8; RP11-438H8.8) | 7.78 | 7.58e-05 | 1.98e-03 |
|  | 12 | 15373987 | 15374609 | PDFSTATSENSEMBLUCSC | 623 | 9 | 10268 | island | 0.748996 | 0.012201 | RERG (margin: RERG) | 7.78 | 4.11e-05 | 1.17e-03 |
|  | 6 | 32301073 | 32301513 | PDFSTATSENSEMBLUCSC | 441 | 4 | 34103 | gene | 0.209174 | 0.970054 | Not queried | 7.78 | 2.86e-02 | 6.12e-02 |
|  | 8 | 38831148 | 38831857 | PDFSTATSENSEMBLUCSC | 710 | 7 | 40237 | island | 0.932871 | 0.065260 | Not queried | 7.78 | 1.75e-02 | 5.79e-02 |
|  | 6 | 46138725 | 46139019 | PDFSTATSENSEMBLUCSC | 295 | 10 | 35019 | tss | 0.532603 | 0.007367 | ENPP5 (margin: ENPP5) | 7.77 | 1.08e-05 | 4.82e-04 |
|  | 5 | 131347439 | 131347773 | PDFSTATSENSEMBLUCSC | 335 | 5 | 32266 | tss | 0.630365 | 0.020212 | AC034228.2; ACSL6 (margin: AC034228.3; AC034228.2; ACSL6) | 7.76 | 7.94e-03 | 3.19e-02 |
|  | 1 | 215256254 | 215256978 | PDFSTATSENSEMBLUCSC | 725 | 9 | 1009 | gene;island | 0.688374 | 0.019739 | KCNK2 (margin: KCNK2) | 7.76 | 7.82e-04 | 8.17e-03 |
|  | 2 | 214148856 | 214149035 | PDFSTATSENSEMBLUCSC | 180 | 7 | 23859 | tss | 0.240302 | 0.976442 | SPAG16; AC079610.2 (margin: SPAG16; RP11-105N14.2; AC079610.2) | 7.75 | 5.83e-04 | 6.47e-03 |
|  | 17 | 14204310 | 14204593 | PDFSTATSENSEMBLUCSC | 284 | 6 | 18034 | island | 0.510526 | 0.005011 | HS3ST3B1 (margin: HS3ST3B1; RP11-214O1.2; RP11-214O1.1) | 7.75 | 2.16e-03 | 1.45e-02 |
|  | 16 | 1146277 | 1146545 | PDFSTATSENSEMBLUCSC | 269 | 4 | 14988 | tss | 0.302182 | 0.984988 | Not queried | 7.75 | 2.86e-02 | 6.12e-02 |
|  | 14 | 91580129 | 91580613 | PDFSTATSENSEMBLUCSC | 485 | 9 | 12558 | tss | 0.034577 | 0.905840 | C14orf159 (margin: C14orf159) | 7.74 | 4.11e-05 | 1.17e-03 |
|  | 4 | 176986621 | 176987577 | PDFSTATSENSEMBLUCSC | 957 | 14 | 31440 | island | 0.612494 | 0.008049 | WDR17 (margin: WDR17) | 7.74 | 3.49e-07 | 4.42e-05 |
|  | 13 | 78271874 | 78271894 | PDFSTATSENSEMBLUCSC | 21 | 4 | 11485 | tss;island | 0.680316 | 0.009725 | Not queried | 7.74 | 2.86e-02 | 6.12e-02 |
|  | 9 | 114557067 | 114557553 | PDFSTATSENSEMBLUCSC | 487 | 4 | 40929 | island | 0.121344 | 0.971110 | Not queried | 7.74 | 5.71e-02 | 1.00e-01 |
|  | 16 | 67918484 | 67918754 | PDFSTATSENSEMBLUCSC | 271 | 5 | 15315 | tss | 0.914013 | 0.047918 | Not queried | 7.74 | 3.17e-02 | 6.50e-02 |
|  | 6 | 152128258 | 152129036 | PDFSTATSENSEMBLUCSC | 779 | 20 | 34514 | gene;island | 0.487199 | 0.007051 | ESR1 (margin: ESR1; RP3-443C4.2) | 7.73 | 5.80e-11 | 4.35e-08 |
|  | 6 | 152128258 | 152128805 | PDFSTATSENSEMBLUCSC | 548 | 18 | 35305 | tss | 0.487199 | 0.008777 | ESR1 (margin: ESR1; RP3-443C4.2) | 7.73 | 8.82e-10 | 3.90e-07 |
|  | 7 | 65878734 | 65879115 | PDFSTATSENSEMBLUCSC | 382 | 4 | 38534 | island | 0.829186 | 0.032688 | Not queried | 7.73 | 2.86e-02 | 6.12e-02 |
|  | 4 | 8582101 | 8582287 | PDFSTATSENSEMBLUCSC | 187 | 6 | 30298 | tss | 0.652664 | 0.011809 | GPR78 (margin: GPR78) | 7.73 | 2.16e-03 | 1.45e-02 |
|  | 1 | 23751369 | 23751761 | PDFSTATSENSEMBLUCSC | 393 | 5 | 1341 | tss | 0.885921 | 0.027362 | TCEA3 (margin: TCEA3; ASAP3) | 7.73 | 7.94e-03 | 3.19e-02 |
|  | 2 | 242212422 | 242212528 | PDFSTATSENSEMBLUCSC | 107 | 5 | 23133 | gene;tss;island | 0.037830 | 0.925862 | HDLBP (margin: HDLBP) | 7.73 | 7.94e-03 | 3.19e-02 |
|  | 7 | 28996196 | 28996639 | PDFSTATSENSEMBLUCSC | 444 | 5 | 36947 | gene;island | 0.643632 | 0.018173 | AC005013.5; AC005013.1; TRIL (margin: AC005013.5; AC005013.1; TRIL) | 7.73 | 7.94e-03 | 3.19e-02 |
|  | 1 | 76262648 | 76262984 | PDFSTATSENSEMBLUCSC | 337 | 4 | 590 | gene | 0.271065 | 0.984402 | Not queried | 7.73 | 2.86e-02 | 6.12e-02 |
|  | 19 | 18118666 | 18119069 | PDFSTATSENSEMBLUCSC | 404 | 6 | 19926 | gene;island | 0.171320 | 0.979980 | ARRDC2 (margin: CTB-52I2.8; ARRDC2; CTB-52I2.5) | 7.73 | 2.16e-03 | 1.45e-02 |
|  | 10 | 116391467 | 116391843 | PDFSTATSENSEMBLUCSC | 377 | 5 | 4769 | gene;island | 0.141263 | 0.972966 | ABLIM1 (margin: ABLIM1) | 7.72 | 7.94e-03 | 3.19e-02 |
|  | 10 | 131412507 | 131412764 | PDFSTATSENSEMBLUCSC | 258 | 4 | 4828 | gene | 0.214503 | 0.975969 | Not queried | 7.72 | 2.86e-02 | 6.12e-02 |
|  | 4 | 101111147 | 101111927 | PDFSTATSENSEMBLUCSC | 781 | 9 | 31203 | island | 0.742314 | 0.017194 | RP11-15B17.1; RP11-588P8.1; DDIT4L (margin: RP11-15B17.1; RP11-588P8.1; DDIT4L) | 7.72 | 5.64e-03 | 3.18e-02 |
|  | 3 | 50604613 | 50605156 | PDFSTATSENSEMBLUCSC | 544 | 6 | 27812 | gene | 0.051019 | 0.977475 | HEMK1; C3orf18 (margin: HEMK1; C3orf18) | 7.72 | 8.66e-03 | 3.36e-02 |
|  | 12 | 108733253 | 108733370 | PDFSTATSENSEMBLUCSC | 118 | 6 | 9980 | tss | 0.077269 | 0.925810 | CMKLR1 (margin: CMKLR1) | 7.71 | 2.16e-03 | 1.45e-02 |
|  | 3 | 191048361 | 191048439 | PDFSTATSENSEMBLUCSC | 79 | 4 | 28789 | tss | 0.005729 | 0.350331 | Not queried | 7.71 | 2.86e-02 | 6.12e-02 |
|  | 5 | 73937299 | 73937330 | PDFSTATSENSEMBLUCSC | 32 | 4 | 32124 | tss | 0.310210 | 0.003581 | Not queried | 7.71 | 2.86e-02 | 6.12e-02 |
|  | 2 | 160654866 | 160655081 | PDFSTATSENSEMBLUCSC | 216 | 4 | 23682 | tss;island | 0.856196 | 0.078602 | Not queried | 7.71 | 2.86e-02 | 6.12e-02 |
|  | 19 | 50831762 | 50832037 | PDFSTATSENSEMBLUCSC | 276 | 5 | 20173 | gene;island | 0.941142 | 0.053825 | NR1H2; KCNC3 (margin: NR1H2; KCNC3; NAPSB) | 7.71 | 7.94e-03 | 3.19e-02 |
|  | 11 | 119993722 | 119994008 | PDFSTATSENSEMBLUCSC | 287 | 7 | 6942 | gene | 0.052219 | 0.943752 | TRIM29 (margin: TRIM29) | 7.71 | 5.83e-04 | 6.47e-03 |
|  | 1 | 40420503 | 40421002 | PDFSTATSENSEMBLUCSC | 500 | 13 | 3111 | island | 0.016041 | 0.536977 | MFSD2A (margin: MFSD2A; RP3-342P20.2; Y\_RNA) | 7.70 | 2.28e-04 | 3.75e-03 |
|  | 1 | 40420503 | 40420692 | PDFSTATSENSEMBLUCSC | 190 | 9 | 1531 | tss | 0.006049 | 0.543333 | MFSD2A (margin: MFSD2A; RP3-342P20.2; Y\_RNA) | 7.70 | 7.77e-03 | 3.19e-02 |
|  | 16 | 751312 | 751603 | PDFSTATSENSEMBLUCSC | 292 | 4 | 14540 | gene;island | 0.078586 | 0.920617 | Not queried | 7.70 | 2.86e-02 | 6.12e-02 |
|  | 14 | 24458099 | 24458345 | PDFSTATSENSEMBLUCSC | 247 | 4 | 12034 | gene | 0.010505 | 0.635548 | Not queried | 7.70 | 2.86e-02 | 6.12e-02 |
|  | 4 | 7940375 | 7940579 | PDFSTATSENSEMBLUCSC | 205 | 4 | 29943 | gene;island | 0.008531 | 0.555510 | Not queried | 7.70 | 2.86e-02 | 6.12e-02 |
|  | 1 | 155107802 | 155108104 | PDFSTATSENSEMBLUCSC | 303 | 4 | 1981 | tss;island | 0.003640 | 0.014624 | Not queried | 7.69 | 3.43e-01 | 3.84e-01 |
|  | 20 | 37230326 | 37230484 | PDFSTATSENSEMBLUCSC | 159 | 5 | 25737 | tss | 0.032872 | 0.874856 | ARHGAP40 (margin: ARHGAP40) | 7.69 | 7.94e-03 | 3.19e-02 |
|  | 1 | 50489240 | 50489596 | PDFSTATSENSEMBLUCSC | 357 | 7 | 522 | gene | 0.891673 | 0.032619 | AGBL4 (margin: MTND2P29; AGBL4) | 7.69 | 4.08e-03 | 2.47e-02 |
|  | 21 | 43786663 | 43786997 | PDFSTATSENSEMBLUCSC | 335 | 5 | 26567 | tss | 0.672652 | 0.016623 | TFF1 (margin: TFF1; TMPRSS3) | 7.68 | 7.94e-03 | 3.19e-02 |
|  | 19 | 11494980 | 11495295 | PDFSTATSENSEMBLUCSC | 316 | 4 | 21566 | island | 0.899277 | 0.121359 | Not queried | 7.68 | 2.86e-02 | 6.12e-02 |
|  | 7 | 5111488 | 5111669 | PDFSTATSENSEMBLUCSC | 182 | 8 | 37420 | tss;island | 0.204464 | 0.974606 | RBAK (margin: RBAK) | 7.68 | 1.55e-04 | 2.71e-03 |
|  | 14 | 37051693 | 37051949 | PDFSTATSENSEMBLUCSC | 257 | 7 | 12787 | island | 0.006821 | 0.558471 | NKX2-8 (margin: RPL29P3; NKX2-8; RN7SKP257) | 7.68 | 5.83e-04 | 6.47e-03 |
|  | 11 | 111383889 | 111384223 | PDFSTATSENSEMBLUCSC | 335 | 4 | 7686 | tss;island | 0.090146 | 0.881994 | Not queried | 7.67 | 2.86e-02 | 6.12e-02 |
|  | 6 | 30653167 | 30653799 | PDFSTATSENSEMBLUCSC | 633 | 12 | 33804 | gene;island | 0.045732 | 0.899116 | PPP1R18 (margin: PPP1R18; NRM; RPL7P4) | 7.67 | 7.40e-07 | 7.21e-05 |
|  | 12 | 95009858 | 95010113 | PDFSTATSENSEMBLUCSC | 256 | 5 | 9250 | gene | 0.008302 | 0.726044 | Not queried | 7.67 | 3.17e-02 | 6.50e-02 |
|  | 20 | 62284488 | 62284780 | PDFSTATSENSEMBLUCSC | 293 | 4 | 26362 | island | 0.217401 | 0.002724 | Not queried | 7.67 | 2.86e-02 | 6.12e-02 |
|  | 22 | 21369411 | 21369777 | PDFSTATSENSEMBLUCSC | 367 | 4 | 26990 | tss;island | 0.076383 | 0.778550 | Not queried | 7.66 | 2.86e-02 | 6.12e-02 |
|  | 3 | 131753858 | 131754273 | PDFSTATSENSEMBLUCSC | 416 | 6 | 28596 | tss;island | 0.402378 | 0.012064 | Not queried | 7.66 | 6.49e-02 | 1.11e-01 |
|  | 7 | 75932840 | 75932977 | PDFSTATSENSEMBLUCSC | 138 | 6 | 37053 | gene;island | 0.397926 | 0.003831 | HSPB1 (margin: HSPB1) | 7.64 | 2.16e-03 | 1.45e-02 |
|  | 12 | 121975121 | 121975669 | PDFSTATSENSEMBLUCSC | 549 | 5 | 9369 | gene;island | 0.824954 | 0.008835 | KDM2B (margin: KDM2B) | 7.64 | 7.94e-03 | 3.19e-02 |
|  | 7 | 87563462 | 87563649 | PDFSTATSENSEMBLUCSC | 188 | 6 | 37689 | tss | 0.490881 | 0.005335 | ADAM22 (margin: ADAM22) | 7.64 | 2.16e-03 | 1.45e-02 |
|  | 12 | 101603835 | 101603996 | PDFSTATSENSEMBLUCSC | 162 | 4 | 9267 | gene | 0.592742 | 0.012182 | Not queried | 7.64 | 2.86e-02 | 6.12e-02 |
|  | 7 | 38468944 | 38469273 | PDFSTATSENSEMBLUCSC | 330 | 5 | 36971 | gene | 0.018347 | 0.851583 | AMPH (margin: AMPH) | 7.63 | 7.94e-03 | 3.19e-02 |
|  | 8 | 110704026 | 110704289 | PDFSTATSENSEMBLUCSC | 264 | 5 | 39874 | tss | 0.641600 | 0.010868 | SYBU (margin: SYBU) | 7.63 | 7.94e-03 | 3.19e-02 |
|  | 20 | 1206682 | 1206758 | PDFSTATSENSEMBLUCSC | 77 | 6 | 25535 | tss | 0.163230 | 0.003784 | RAD21L1 (margin: RAD21L1) | 7.62 | 2.16e-03 | 1.45e-02 |
|  | 14 | 23305548 | 23305957 | PDFSTATSENSEMBLUCSC | 410 | 6 | 12703 | island | 0.041398 | 0.890281 | MRPL52; MMP14 (margin: MRPL52; MMP14; SLC7A7) | 7.62 | 2.16e-03 | 1.45e-02 |
|  | 17 | 38465281 | 38465422 | PDFSTATSENSEMBLUCSC | 142 | 5 | 17329 | tss | 0.559284 | 0.005766 | RARA (margin: CDC6; CTD-2267D19.6; RARA) | 7.61 | 7.94e-03 | 3.19e-02 |
|  | 19 | 19281140 | 19281559 | PDFSTATSENSEMBLUCSC | 420 | 9 | 20636 | tss | 0.518657 | 0.005905 | MEF2BNB-MEF2B; MEF2B (margin: MEF2BNB-MEF2B; MEF2B; MEF2BNB) | 7.61 | 2.76e-03 | 1.78e-02 |
|  | 19 | 54210147 | 54210518 | PDFSTATSENSEMBLUCSC | 372 | 4 | 21035 | tss | 0.055226 | 0.903967 | Not queried | 7.59 | 5.71e-02 | 1.00e-01 |
|  | 2 | 74730507 | 74730948 | PDFSTATSENSEMBLUCSC | 442 | 9 | 23461 | tss | 0.024516 | 0.584881 | RP11-523H20.3; LBX2-AS1; LBX2; PCGF1 (margin: TTC31; RP11-523H20.3; LBX2-AS1; TLX2; LBX2; AC005041.17; PCGF1) | 7.59 | 4.11e-05 | 1.17e-03 |
|  | 3 | 191048308 | 191048439 | PDFSTATSENSEMBLUCSC | 132 | 5 | 28084 | gene;island | 0.001429 | 0.324409 | CCDC50; UTS2B (margin: CCDC50; UTS2B) | 7.59 | 7.94e-03 | 3.19e-02 |
|  | 4 | 44728525 | 44728857 | PDFSTATSENSEMBLUCSC | 333 | 9 | 31018 | island | 0.005683 | 0.527410 | GNPDA2 (margin: GNPDA2) | 7.58 | 4.11e-05 | 1.17e-03 |
|  | 8 | 38831148 | 38831598 | PDFSTATSENSEMBLUCSC | 451 | 4 | 39660 | tss | 0.924795 | 0.221405 | Not queried | 7.58 | 2.00e-01 | 2.55e-01 |
|  | 19 | 54372324 | 54373043 | PDFSTATSENSEMBLUCSC | 720 | 13 | 20209 | gene;island | 0.636065 | 0.006806 | MYADM; AC008753.6 (margin: AC008440.10; MYADM; PRKCG; AC008753.6; AC008440.5) | 7.57 | 1.92e-07 | 2.60e-05 |
|  | 19 | 54372324 | 54372713 | PDFSTATSENSEMBLUCSC | 390 | 9 | 21043 | tss | 0.617231 | 0.006806 | MYADM; AC008753.6 (margin: AC008440.10; MYADM; PRKCG; AC008753.6; AC008440.5) | 7.57 | 4.11e-05 | 1.17e-03 |
|  | 4 | 96470053 | 96470349 | PDFSTATSENSEMBLUCSC | 297 | 7 | 31191 | island | 0.640816 | 0.007889 | RP11-710C12.1; UNC5C (margin: RP11-710C12.1; UNC5C) | 7.57 | 5.83e-04 | 6.47e-03 |
|  | 4 | 96470237 | 96470349 | PDFSTATSENSEMBLUCSC | 113 | 5 | 30505 | tss | 0.792742 | 0.007889 | RP11-710C12.1; UNC5C (margin: RP11-710C12.1; UNC5C) | 7.57 | 7.94e-03 | 3.19e-02 |
|  | 17 | 21279561 | 21279696 | PDFSTATSENSEMBLUCSC | 136 | 5 | 17203 | tss | 0.455491 | 0.985082 | KCNJ12 (margin: KCNJ12) | 7.56 | 7.94e-03 | 3.19e-02 |
|  | 17 | 693137 | 693388 | PDFSTATSENSEMBLUCSC | 252 | 4 | 16295 | gene | 0.130459 | 0.956058 | Not queried | 7.56 | 2.86e-02 | 6.12e-02 |
|  | 17 | 7197878 | 7198087 | PDFSTATSENSEMBLUCSC | 210 | 7 | 17072 | tss | 0.683375 | 0.004111 | YBX2 (margin: SLC2A4; RP1-4G17.2; YBX2) | 7.56 | 2.33e-03 | 1.52e-02 |
|  | 8 | 22560922 | 22561238 | PDFSTATSENSEMBLUCSC | 317 | 5 | 40142 | island | 0.002856 | 0.375158 | RP11-459E5.1 (margin: RP11-459E5.1; EGR3; PEBP4) | 7.56 | 7.94e-03 | 3.19e-02 |
|  | 6 | 106958303 | 106958645 | PDFSTATSENSEMBLUCSC | 343 | 5 | 35150 | tss;island | 0.873249 | 0.013001 | AIM1 (margin: AIM1) | 7.55 | 7.94e-03 | 3.19e-02 |
|  | 6 | 3848634 | 3849577 | PDFSTATSENSEMBLUCSC | 944 | 26 | 34602 | tss | 0.007421 | 0.498059 | RP11-420L9.4; FAM50B (margin: RP11-420L9.4; FAM50B) | 7.55 | 4.03e-15 | 1.31e-11 |
|  | 7 | 43797839 | 43798207 | PDFSTATSENSEMBLUCSC | 369 | 7 | 37558 | tss;island | 0.563564 | 0.008304 | BLVRA (margin: BLVRA; AC005189.6) | 7.55 | 4.08e-03 | 2.47e-02 |
|  | 4 | 3486039 | 3486380 | PDFSTATSENSEMBLUCSC | 342 | 4 | 29908 | gene;island | 0.004899 | 0.534056 | Not queried | 7.54 | 2.86e-02 | 6.12e-02 |
|  | 15 | 93199018 | 93199180 | PDFSTATSENSEMBLUCSC | 163 | 6 | 14473 | island | 0.439713 | 0.008437 | FAM174B (margin: FAM174B) | 7.54 | 2.16e-03 | 1.45e-02 |
|  | 11 | 118401536 | 118401801 | PDFSTATSENSEMBLUCSC | 266 | 4 | 7734 | tss | 0.307914 | 0.983165 | Not queried | 7.54 | 2.86e-02 | 6.12e-02 |
|  | 6 | 26189333 | 26189597 | PDFSTATSENSEMBLUCSC | 265 | 6 | 34692 | tss | 0.002518 | 0.259360 | HIST1H4D (margin: HIST1H2BE; HIST1H1PS1; HIST1H2BF; HIST1H4D; HIST1H3D; HIST1H2AD) | 7.54 | 2.16e-03 | 1.45e-02 |
|  | 19 | 36132263 | 36132627 | PDFSTATSENSEMBLUCSC | 365 | 6 | 20710 | tss;island | 0.212847 | 0.943455 | ETV2 (margin: RBM42; ETV2; COX6B1) | 7.54 | 8.66e-03 | 3.36e-02 |
|  | 3 | 71802420 | 71802608 | PDFSTATSENSEMBLUCSC | 189 | 4 | 27859 | gene;tss;island | 0.893977 | 0.044735 | Not queried | 7.54 | 2.00e-01 | 2.55e-01 |
|  | 18 | 20714217 | 20714496 | PDFSTATSENSEMBLUCSC | 280 | 6 | 19203 | tss;island | 0.494748 | 0.005530 | CABLES1; AC105247.1 (margin: CABLES1; AC105247.1) | 7.54 | 2.16e-03 | 1.45e-02 |
|  | 1 | 179560700 | 179560983 | PDFSTATSENSEMBLUCSC | 284 | 5 | 2119 | tss | 0.545865 | 0.011234 | RP11-545A16.4; TDRD5 (margin: RP11-545A16.3; RP11-545A16.4; TDRD5) | 7.53 | 7.94e-03 | 3.19e-02 |
|  | 11 | 119020099 | 119020168 | PDFSTATSENSEMBLUCSC | 70 | 6 | 6937 | gene;tss;island | 0.245700 | 0.005085 | ABCG4 (margin: ABCG4; AP002956.1) | 7.53 | 2.16e-03 | 1.45e-02 |
|  | 12 | 113415883 | 113416268 | PDFSTATSENSEMBLUCSC | 386 | 5 | 10024 | tss | 0.024073 | 0.948489 | Not queried | 7.53 | 9.52e-02 | 1.53e-01 |
|  | 5 | 98104747 | 98105150 | PDFSTATSENSEMBLUCSC | 404 | 5 | 32981 | island | 0.229708 | 0.004054 | Not queried | 7.52 | 1.59e-02 | 5.36e-02 |
|  | 16 | 53468112 | 53468205 | PDFSTATSENSEMBLUCSC | 94 | 4 | 15235 | tss | 0.219088 | 0.004335 | Not queried | 7.52 | 3.43e-01 | 3.84e-01 |
|  | 16 | 89267679 | 89268040 | PDFSTATSENSEMBLUCSC | 362 | 4 | 16246 | island | 0.815057 | 0.031517 | Not queried | 7.52 | 2.86e-02 | 6.12e-02 |
|  | 12 | 66123127 | 66123496 | PDFSTATSENSEMBLUCSC | 370 | 4 | 10600 | island | 0.025177 | 0.800347 | Not queried | 7.52 | 2.86e-02 | 6.12e-02 |
|  | 11 | 118401536 | 118401878 | PDFSTATSENSEMBLUCSC | 343 | 5 | 8858 | island | 0.410006 | 0.978223 | TTC36; TMEM25; RP11-770J1.3 (margin: KMT2A; TTC36; TMEM25; RP11-770J1.3) | 7.52 | 7.94e-03 | 3.19e-02 |
|  | 10 | 52833902 | 52834189 | PDFSTATSENSEMBLUCSC | 288 | 7 | 4590 | gene;tss;island | 0.777780 | 0.013817 | PRKG1 (margin: PRKG1; RP11-96B5.3) | 7.51 | 5.83e-04 | 6.47e-03 |
|  | 5 | 67583972 | 67584380 | PDFSTATSENSEMBLUCSC | 409 | 8 | 31637 | gene;island | 0.637094 | 0.009804 | PIK3R1 (margin: PIK3R1) | 7.51 | 1.55e-04 | 2.71e-03 |
|  | 5 | 67583972 | 67584222 | PDFSTATSENSEMBLUCSC | 251 | 6 | 32103 | tss | 0.637094 | 0.009804 | PIK3R1 (margin: PIK3R1) | 7.51 | 2.16e-03 | 1.45e-02 |
|  | 7 | 122526824 | 122526940 | PDFSTATSENSEMBLUCSC | 117 | 6 | 37867 | tss | 0.479181 | 0.007492 | CADPS2 (margin: CADPS2) | 7.50 | 2.16e-03 | 1.45e-02 |
|  | 6 | 29893926 | 29894341 | PDFSTATSENSEMBLUCSC | 416 | 16 | 34757 | tss;island | 0.060563 | 0.909336 | HLA-K; HCG4B (margin: HLA-K; HLA-U; HCG4B) | 7.50 | 6.65e-09 | 2.09e-06 |
|  | 1 | 207038460 | 207038704 | PDFSTATSENSEMBLUCSC | 245 | 4 | 2216 | tss | 0.363597 | 0.004456 | Not queried | 7.50 | 2.00e-01 | 2.55e-01 |
|  | 18 | 70211774 | 70211974 | PDFSTATSENSEMBLUCSC | 201 | 6 | 19318 | tss;island | 0.667853 | 0.014394 | CBLN2 (margin: CBLN2) | 7.50 | 2.16e-03 | 1.45e-02 |
|  | 7 | 37959686 | 37959966 | PDFSTATSENSEMBLUCSC | 281 | 4 | 37543 | tss;island | 0.060875 | 0.875862 | Not queried | 7.50 | 2.86e-02 | 6.12e-02 |
|  | 11 | 98891492 | 98891820 | PDFSTATSENSEMBLUCSC | 329 | 9 | 7640 | tss;island | 0.807820 | 0.019823 | CNTN5 (margin: CNTN5) | 7.50 | 4.11e-05 | 1.17e-03 |
|  | 11 | 63439115 | 63439313 | PDFSTATSENSEMBLUCSC | 199 | 7 | 7377 | tss | 0.805798 | 0.015898 | ATL3 (margin: RTN3; ATL3) | 7.50 | 5.83e-04 | 6.47e-03 |
|  | 3 | 158390510 | 158390821 | PDFSTATSENSEMBLUCSC | 312 | 7 | 28686 | tss | 0.967124 | 0.030208 | GFM1; LXN (margin: GFM1; LXN) | 7.49 | 5.83e-04 | 6.47e-03 |
|  | 17 | 46713842 | 46714169 | PDFSTATSENSEMBLUCSC | 328 | 4 | 18494 | island | 0.972454 | 0.146427 | Not queried | 7.49 | 2.86e-02 | 6.12e-02 |
|  | 3 | 10206254 | 10206731 | PDFSTATSENSEMBLUCSC | 478 | 10 | 28874 | island | 0.011123 | 0.629617 | IRAK2 (margin: IRAK2) | 7.49 | 1.08e-05 | 4.82e-04 |
|  | 17 | 406249 | 406501 | PDFSTATSENSEMBLUCSC | 253 | 4 | 17796 | island | 0.195381 | 0.971665 | Not queried | 7.48 | 2.86e-02 | 6.12e-02 |
|  | 3 | 73674074 | 73674702 | PDFSTATSENSEMBLUCSC | 629 | 12 | 28446 | tss | 0.687794 | 0.010838 | PDZRN3-AS1; PDZRN3 (margin: PDZRN3-AS1; PDZRN3) | 7.48 | 7.40e-07 | 7.21e-05 |
|  | 6 | 46097392 | 46097784 | PDFSTATSENSEMBLUCSC | 393 | 9 | 36184 | island | 0.555134 | 0.004956 | ENPP4; RP1-8B1.4 (margin: ENPP4; RP1-8B1.4) | 7.48 | 4.11e-05 | 1.17e-03 |
|  | 12 | 7167584 | 7167900 | PDFSTATSENSEMBLUCSC | 317 | 4 | 9543 | tss | 0.137563 | 0.950416 | Not queried | 7.48 | 1.14e-01 | 1.68e-01 |
|  | 5 | 156886816 | 156887129 | PDFSTATSENSEMBLUCSC | 314 | 7 | 33342 | island | 0.151132 | 0.933745 | NIPAL4; CTB-109A12.1; ADAM19 (margin: NIPAL4; CTB-109A12.1; ADAM19) | 7.47 | 5.83e-04 | 6.47e-03 |
|  | 6 | 46097392 | 46097695 | PDFSTATSENSEMBLUCSC | 304 | 8 | 35018 | tss | 0.547611 | 0.005192 | ENPP4; RP1-8B1.4 (margin: ENPP4; RP1-8B1.4) | 7.47 | 1.55e-04 | 2.71e-03 |
|  | 12 | 131303016 | 131303247 | PDFSTATSENSEMBLUCSC | 232 | 4 | 9418 | gene;island | 0.588329 | 0.009899 | Not queried | 7.47 | 2.86e-02 | 6.12e-02 |
|  | 11 | 1463541 | 1463662 | PDFSTATSENSEMBLUCSC | 122 | 4 | 6419 | gene;island | 0.011709 | 0.689861 | Not queried | 7.47 | 2.86e-02 | 6.12e-02 |
|  | 5 | 140345461 | 140346403 | PDFSTATSENSEMBLUCSC | 943 | 14 | 31806 | gene;island | 0.654195 | 0.017370 | PCDHA1; PCDHA2; PCDHA3; PCDHA4; PCDHA5; PCDHA6; PCDHA7; PCDHA8; PCDHA9; PCDHA10; PCDHA11; PCDHA12; PCDHA13; PCDHAC1; PCDHAC2 (margin: PCDHA1; PCDHA2; PCDHA3; PCDHA4; PCDHA5; PCDHA6; PCDHA7; PCDHA8; PCDHA9; PCDHA10; PCDHA11; PCDHA12; PCDHA13; PCDHAC1; PCDHAC2) | 7.47 | 6.74e-03 | 3.19e-02 |
|  | 17 | 4613992 | 4614422 | PDFSTATSENSEMBLUCSC | 431 | 5 | 16356 | gene;island | 0.496924 | 0.006775 | Not queried | 7.47 | 9.52e-02 | 1.53e-01 |
|  | 13 | 92050675 | 92051154 | PDFSTATSENSEMBLUCSC | 480 | 11 | 11814 | island | 0.586370 | 0.007782 | GPC5 (margin: GPC5) | 7.47 | 5.67e-06 | 3.54e-04 |
|  | 3 | 43020791 | 43020952 | PDFSTATSENSEMBLUCSC | 162 | 4 | 27737 | gene | 0.310496 | 0.002610 | Not queried | 7.47 | 2.86e-02 | 6.12e-02 |
|  | 22 | 45405621 | 45405768 | PDFSTATSENSEMBLUCSC | 148 | 4 | 26898 | gene | 0.526464 | 0.010334 | Not queried | 7.46 | 2.86e-02 | 6.12e-02 |
|  | 12 | 98850691 | 98851021 | PDFSTATSENSEMBLUCSC | 331 | 6 | 10703 | island | 0.260487 | 0.976988 | SLC9A7P1 (margin: SLC9A7P1) | 7.46 | 2.16e-03 | 1.45e-02 |
|  | 16 | 711015 | 711235 | PDFSTATSENSEMBLUCSC | 221 | 4 | 14535 | gene;island | 0.569783 | 0.012232 | Not queried | 7.45 | 5.71e-02 | 1.00e-01 |
|  | 4 | 144480206 | 144480561 | PDFSTATSENSEMBLUCSC | 356 | 11 | 30614 | tss | 0.978698 | 0.244793 | SMARCA5; GUSBP5 (margin: SMARCA5; GUSBP5; RP11-481K16.2) | 7.45 | 2.84e-06 | 1.96e-04 |
|  | 4 | 172734266 | 172734514 | PDFSTATSENSEMBLUCSC | 249 | 6 | 30678 | tss;island | 0.712600 | 0.012023 | GALNTL6 (margin: GALNTL6) | 7.44 | 4.33e-03 | 2.52e-02 |
|  | 11 | 121970496 | 121970768 | PDFSTATSENSEMBLUCSC | 273 | 6 | 6946 | gene | 0.971004 | 0.179433 | RP11-166D19.1; MIR125B1 (margin: RP11-166D19.1; MIR125B1) | 7.43 | 2.16e-03 | 1.45e-02 |
|  | 11 | 125366238 | 125366590 | PDFSTATSENSEMBLUCSC | 353 | 9 | 7791 | tss;island | 0.277824 | 0.979808 | AP000708.1; FEZ1 (margin: AP000708.1; FEZ1) | 7.43 | 1.06e-02 | 4.01e-02 |
|  | 3 | 46448496 | 46448963 | PDFSTATSENSEMBLUCSC | 468 | 8 | 28274 | tss | 0.040052 | 0.864856 | ACKR5; RP11-24F11.2 (margin: ACKR5; RP11-24F11.2) | 7.43 | 1.55e-04 | 2.71e-03 |
|  | 10 | 63422511 | 63422841 | PDFSTATSENSEMBLUCSC | 331 | 8 | 5769 | island | 0.692062 | 0.021229 | C10orf107 (margin: C10orf107) | 7.43 | 1.55e-04 | 2.71e-03 |
|  | 1 | 197881327 | 197881540 | PDFSTATSENSEMBLUCSC | 214 | 5 | 2157 | tss;island | 0.035328 | 0.956617 | LHX9 (margin: C1orf53; LHX9) | 7.41 | 7.94e-03 | 3.19e-02 |
|  | 5 | 140248564 | 140249080 | PDFSTATSENSEMBLUCSC | 517 | 5 | 31801 | gene;island | 0.575850 | 0.008211 | Not queried | 7.41 | 3.17e-02 | 6.50e-02 |
|  | 12 | 15114703 | 15114904 | PDFSTATSENSEMBLUCSC | 202 | 4 | 9589 | tss | 0.185747 | 0.984248 | Not queried | 7.41 | 5.71e-02 | 1.00e-01 |
|  | 15 | 57210107 | 57210406 | PDFSTATSENSEMBLUCSC | 300 | 4 | 13362 | gene;tss;island | 0.109660 | 0.809698 | Not queried | 7.41 | 2.86e-02 | 6.12e-02 |
|  | 7 | 8473234 | 8473578 | PDFSTATSENSEMBLUCSC | 345 | 6 | 37439 | tss;island | 0.778704 | 0.025441 | NXPH1 (margin: NXPH1) | 7.41 | 2.16e-03 | 1.45e-02 |
|  | 17 | 39216457 | 39216687 | PDFSTATSENSEMBLUCSC | 231 | 4 | 17337 | tss | 0.257701 | 0.977789 | Not queried | 7.40 | 2.86e-02 | 6.12e-02 |
|  | 15 | 78423938 | 78424152 | PDFSTATSENSEMBLUCSC | 215 | 6 | 13829 | tss | 0.522790 | 0.016524 | IDH3A; CIB2 (margin: IDH3A; RP11-285A1.1; CIB2) | 7.39 | 2.16e-03 | 1.45e-02 |
|  | 15 | 83951663 | 83952019 | PDFSTATSENSEMBLUCSC | 357 | 4 | 13458 | gene;island | 0.074909 | 0.895493 | Not queried | 7.39 | 2.86e-02 | 6.12e-02 |
|  | 7 | 193229 | 193732 | PDFSTATSENSEMBLUCSC | 504 | 5 | 36701 | gene;island | 0.017854 | 0.682162 | FAM20C; AC093627.12 (margin: FAM20C; AC093627.12) | 7.39 | 7.94e-03 | 3.19e-02 |
|  | 10 | 135340445 | 135340850 | PDFSTATSENSEMBLUCSC | 406 | 7 | 5465 | tss | 0.041826 | 0.892464 | CYP2E1; SPRN; AL161645.2 (margin: RP11-108K14.4; CYP2E1; SPRN; AL161645.2) | 7.39 | 5.83e-04 | 6.47e-03 |
|  | 4 | 55991782 | 55991943 | PDFSTATSENSEMBLUCSC | 162 | 6 | 30402 | tss | 0.643531 | 0.024479 | KDR (margin: KDR) | 7.39 | 2.16e-03 | 1.45e-02 |
|  | 19 | 29455596 | 29455913 | PDFSTATSENSEMBLUCSC | 318 | 4 | 20668 | tss | 0.030038 | 0.772435 | Not queried | 7.39 | 2.00e-01 | 2.55e-01 |
|  | 21 | 40984760 | 40984886 | PDFSTATSENSEMBLUCSC | 127 | 5 | 26552 | tss | 0.835622 | 0.021726 | B3GALT5; C21orf88 (margin: B3GALT5; C21orf88) | 7.39 | 7.94e-03 | 3.19e-02 |
|  | 5 | 42424356 | 42424604 | PDFSTATSENSEMBLUCSC | 249 | 5 | 31601 | gene;island | 0.707137 | 0.009842 | GHR (margin: GHR) | 7.38 | 7.94e-03 | 3.19e-02 |
|  | 16 | 54318044 | 54318314 | PDFSTATSENSEMBLUCSC | 271 | 4 | 14751 | gene;island | 0.893315 | 0.031754 | Not queried | 7.38 | 2.86e-02 | 6.12e-02 |
|  | 10 | 38645346 | 38645450 | PDFSTATSENSEMBLUCSC | 105 | 4 | 4560 | gene;island | 0.457571 | 0.004137 | Not queried | 7.38 | 2.86e-02 | 6.12e-02 |
|  | 2 | 214148856 | 214149200 | PDFSTATSENSEMBLUCSC | 345 | 9 | 25064 | island | 0.252636 | 0.968467 | SPAG16; AC079610.2 (margin: SPAG16; RP11-105N14.2; AC079610.2) | 7.38 | 4.11e-05 | 1.17e-03 |
|  | 11 | 24518149 | 24518448 | PDFSTATSENSEMBLUCSC | 300 | 9 | 7205 | tss;island | 0.678467 | 0.031119 | LUZP2 (margin: LUZP2) | 7.37 | 4.11e-05 | 1.17e-03 |
|  | 2 | 217560272 | 217560403 | PDFSTATSENSEMBLUCSC | 132 | 5 | 23871 | tss;island | 0.563550 | 0.009585 | AC007563.5; IGFBP5 (margin: AC007563.5; IGFBP5) | 7.37 | 7.94e-03 | 3.19e-02 |
|  | 14 | 90042201 | 90042364 | PDFSTATSENSEMBLUCSC | 164 | 4 | 12171 | gene;tss | 0.128284 | 0.970497 | Not queried | 7.37 | 2.86e-02 | 6.12e-02 |
|  | 5 | 35617687 | 35618055 | PDFSTATSENSEMBLUCSC | 369 | 10 | 32728 | island | 0.507961 | 0.006058 | SPEF2 (margin: SPEF2) | 7.36 | 1.50e-03 | 1.41e-02 |
|  | 5 | 35617687 | 35617969 | PDFSTATSENSEMBLUCSC | 283 | 8 | 32015 | tss | 0.510631 | 0.006665 | SPEF2 (margin: SPEF2) | 7.36 | 1.04e-02 | 3.96e-02 |
|  | 6 | 168417574 | 168417743 | PDFSTATSENSEMBLUCSC | 170 | 4 | 35345 | tss | 0.228778 | 0.989783 | Not queried | 7.36 | 2.86e-02 | 6.12e-02 |
|  | 12 | 100750473 | 100750811 | PDFSTATSENSEMBLUCSC | 339 | 6 | 9941 | tss | 0.114190 | 0.933134 | SLC17A8 (margin: SLC17A8) | 7.36 | 2.16e-03 | 1.45e-02 |
|  | 20 | 24929607 | 24929822 | PDFSTATSENSEMBLUCSC | 216 | 5 | 25643 | tss | 0.234285 | 0.865448 | Not queried | 7.35 | 5.56e-02 | 1.00e-01 |
|  | 10 | 134901199 | 134901305 | PDFSTATSENSEMBLUCSC | 107 | 7 | 5452 | tss | 0.867132 | 0.042019 | GPR123; RP13-439H18.4; AL445199.1 (margin: GPR123; RP13-439H18.4; AL445199.1) | 7.35 | 5.83e-04 | 6.47e-03 |
|  | 5 | 115910467 | 115910732 | PDFSTATSENSEMBLUCSC | 266 | 5 | 33032 | island | 0.436631 | 0.006489 | CTB-118N6.2; SEMA6A (margin: CTB-118N6.1; CTB-118N6.2; SEMA6A) | 7.35 | 7.94e-03 | 3.19e-02 |
|  | 16 | 87958145 | 87958493 | PDFSTATSENSEMBLUCSC | 349 | 4 | 14895 | gene | 0.283577 | 0.985765 | Not queried | 7.34 | 2.86e-02 | 6.12e-02 |
|  | 8 | 121824330 | 121824929 | PDFSTATSENSEMBLUCSC | 600 | 9 | 39891 | tss | 0.726566 | 0.007639 | RP11-713M15.2; SNTB1 (margin: RP11-713M15.2; SNTB1) | 7.34 | 3.99e-03 | 2.47e-02 |
|  | 2 | 177043147 | 177043501 | PDFSTATSENSEMBLUCSC | 355 | 4 | 24913 | island | 0.007948 | 0.628178 | Not queried | 7.34 | 2.86e-02 | 6.12e-02 |
|  | 19 | 50194120 | 50194228 | PDFSTATSENSEMBLUCSC | 109 | 4 | 20162 | gene | 0.233812 | 0.974322 | Not queried | 7.34 | 2.86e-02 | 6.12e-02 |
|  | 11 | 121970613 | 121970768 | PDFSTATSENSEMBLUCSC | 156 | 5 | 7769 | tss | 0.986994 | 0.305700 | RP11-166D19.1; MIR125B1 (margin: RP11-166D19.1; MIR125B1) | 7.33 | 7.94e-03 | 3.19e-02 |
|  | 5 | 134824531 | 134824947 | PDFSTATSENSEMBLUCSC | 417 | 6 | 33136 | island | 0.516441 | 0.015087 | (margin: ) | 7.33 | 2.16e-03 | 1.45e-02 |
|  | 20 | 62037503 | 62037758 | PDFSTATSENSEMBLUCSC | 256 | 4 | 26352 | island | 0.084440 | 0.948969 | Not queried | 7.32 | 2.86e-02 | 6.12e-02 |
|  | 8 | 70747067 | 70747646 | PDFSTATSENSEMBLUCSC | 580 | 7 | 40372 | island | 0.394780 | 0.004073 | RP11-159H10.3; SLCO5A1 (margin: RP11-159H10.3; SLCO5A1) | 7.32 | 1.17e-03 | 1.13e-02 |
|  | 11 | 120008944 | 120009135 | PDFSTATSENSEMBLUCSC | 192 | 5 | 7758 | tss | 0.067310 | 0.316430 | Not queried | 7.32 | 9.52e-02 | 1.53e-01 |
|  | 15 | 62358751 | 62359139 | PDFSTATSENSEMBLUCSC | 389 | 6 | 13715 | tss | 0.533029 | 0.018068 | Not queried | 7.31 | 6.49e-02 | 1.11e-01 |
|  | 2 | 219849133 | 219849307 | PDFSTATSENSEMBLUCSC | 175 | 4 | 23023 | gene;island | 0.264461 | 0.970331 | Not queried | 7.31 | 2.86e-02 | 6.12e-02 |
|  | 4 | 94749865 | 94750061 | PDFSTATSENSEMBLUCSC | 197 | 6 | 30501 | tss | 0.560383 | 0.008257 | ATOH1 (margin: RNA5SP164; ATOH1) | 7.31 | 2.16e-03 | 1.45e-02 |
|  | 11 | 92931250 | 92931416 | PDFSTATSENSEMBLUCSC | 167 | 6 | 7618 | tss;island | 0.395396 | 0.006289 | SLC36A4 (margin: SLC36A4) | 7.31 | 2.16e-03 | 1.45e-02 |
|  | 10 | 94833520 | 94833632 | PDFSTATSENSEMBLUCSC | 113 | 6 | 5235 | tss | 0.467561 | 0.006944 | CYP26A1 (margin: CYP26C1; CYP26A1) | 7.31 | 2.16e-03 | 1.45e-02 |
|  | 10 | 63422511 | 63422668 | PDFSTATSENSEMBLUCSC | 158 | 7 | 5098 | tss | 0.667549 | 0.026702 | C10orf107 (margin: C10orf107) | 7.30 | 5.83e-04 | 6.47e-03 |
|  | 4 | 54457737 | 54458246 | PDFSTATSENSEMBLUCSC | 510 | 6 | 30397 | tss | 0.383187 | 0.003479 | Not queried | 7.30 | 1.80e-01 | 2.45e-01 |
|  | 10 | 73533035 | 73533561 | PDFSTATSENSEMBLUCSC | 527 | 10 | 4624 | gene;island | 0.006436 | 0.547136 | CDH23; C10orf54 (margin: CDH23; C10orf54) | 7.30 | 1.08e-05 | 4.82e-04 |
|  | 1 | 117664976 | 117665186 | PDFSTATSENSEMBLUCSC | 211 | 4 | 1869 | tss;island | 0.932906 | 0.087905 | Not queried | 7.30 | 2.86e-02 | 6.12e-02 |
|  | 17 | 46681111 | 46681401 | PDFSTATSENSEMBLUCSC | 291 | 6 | 16656 | gene | 0.003590 | 0.628672 | HOXB-AS3; HOXB3; HOXB6 (margin: HOXB-AS3; HOXB3; HOXB5; HOXB6; HOXB7; HOXB8) | 7.30 | 4.33e-03 | 2.52e-02 |
|  | 6 | 31096861 | 31097078 | PDFSTATSENSEMBLUCSC | 218 | 5 | 33853 | gene | 0.839512 | 0.003845 | Not queried | 7.29 | 1.59e-02 | 5.36e-02 |
|  | 4 | 91156040 | 91156290 | PDFSTATSENSEMBLUCSC | 251 | 4 | 30060 | gene | 0.072729 | 0.968660 | Not queried | 7.29 | 5.71e-02 | 1.00e-01 |
|  | 6 | 39902288 | 39902693 | PDFSTATSENSEMBLUCSC | 406 | 9 | 36113 | island | 0.690766 | 0.015526 | Not queried | 7.29 | 4.00e-02 | 8.03e-02 |
|  | 6 | 170494301 | 170494475 | PDFSTATSENSEMBLUCSC | 175 | 4 | 36681 | island | 0.008996 | 0.547292 | Not queried | 7.29 | 2.86e-02 | 6.12e-02 |
|  | 16 | 56998544 | 56998712 | PDFSTATSENSEMBLUCSC | 169 | 4 | 14764 | gene | 0.709882 | 0.012984 | Not queried | 7.29 | 5.71e-02 | 1.00e-01 |
|  | 7 | 120497998 | 120498369 | PDFSTATSENSEMBLUCSC | 372 | 5 | 38863 | island | 0.434233 | 0.006446 | TSPAN12 (margin: TSPAN12) | 7.29 | 7.94e-03 | 3.19e-02 |
|  | 7 | 95025611 | 95026248 | PDFSTATSENSEMBLUCSC | 638 | 20 | 38678 | island | 0.162573 | 0.971275 | PON1; PON3 (margin: PON1; PON3; PON2) | 7.29 | 1.45e-11 | 1.57e-08 |
|  | 4 | 7436693 | 7437156 | PDFSTATSENSEMBLUCSC | 464 | 6 | 29937 | gene | 0.006284 | 0.610361 | Not queried | 7.28 | 1.32e-01 | 1.89e-01 |
|  | 2 | 12856626 | 12857067 | PDFSTATSENSEMBLUCSC | 442 | 10 | 24085 | island | 0.186230 | 0.003452 | RP11-333O1.1; TRIB2 (margin: RP11-333O1.1; TRIB2) | 7.28 | 1.08e-05 | 4.82e-04 |
|  | 1 | 220921216 | 220921670 | PDFSTATSENSEMBLUCSC | 455 | 8 | 2262 | tss | 0.546819 | 0.009086 | MARC2 (margin: MARC2) | 7.28 | 1.55e-04 | 2.71e-03 |
|  | 3 | 182970675 | 182971069 | PDFSTATSENSEMBLUCSC | 395 | 11 | 28049 | gene;island | 0.006621 | 0.382088 | B3GNT5; MCF2L2 (margin: B3GNT5; MCF2L2; RNA5SP151) | 7.28 | 1.28e-02 | 4.76e-02 |
|  | 2 | 1497815 | 1498138 | PDFSTATSENSEMBLUCSC | 324 | 5 | 22531 | gene;island | 0.113098 | 0.958536 | TPO (margin: TPO) | 7.27 | 7.94e-03 | 3.19e-02 |
|  | 6 | 29429909 | 29430506 | PDFSTATSENSEMBLUCSC | 598 | 6 | 33658 | gene | 0.003507 | 0.470864 | OR2H1; UBDP1 (margin: OR2H1; OR11A1; UBDP1) | 7.27 | 2.16e-03 | 1.45e-02 |
|  | 6 | 50818685 | 50819236 | PDFSTATSENSEMBLUCSC | 552 | 4 | 36200 | island | 0.266374 | 0.972384 | Not queried | 7.27 | 2.86e-02 | 6.12e-02 |
|  | 1 | 154539974 | 154540159 | PDFSTATSENSEMBLUCSC | 186 | 4 | 1967 | tss | 0.182501 | 0.959925 | Not queried | 7.27 | 2.86e-02 | 6.12e-02 |
|  | 4 | 55991683 | 55991943 | PDFSTATSENSEMBLUCSC | 261 | 8 | 31057 | island | 0.720908 | 0.024979 | KDR (margin: KDR) | 7.27 | 1.55e-04 | 2.71e-03 |
|  | 11 | 133938676 | 133939064 | PDFSTATSENSEMBLUCSC | 389 | 6 | 8974 | island | 0.218973 | 0.979688 | JAM3 (margin: JAM3) | 7.27 | 2.16e-03 | 1.45e-02 |
|  | 11 | 133938676 | 133938810 | PDFSTATSENSEMBLUCSC | 135 | 4 | 7827 | tss | 0.258573 | 0.979688 | Not queried | 7.27 | 2.86e-02 | 6.12e-02 |
|  | 14 | 103989208 | 103989789 | PDFSTATSENSEMBLUCSC | 582 | 9 | 12649 | tss;island | 0.074624 | 0.979670 | CKB; RP11-600F24.7 (margin: TRMT61A; CKB; RP11-600F24.7) | 7.26 | 4.11e-05 | 1.17e-03 |
|  | 8 | 17434749 | 17435088 | PDFSTATSENSEMBLUCSC | 340 | 5 | 39217 | gene;island | 0.432513 | 0.005639 | PDGFRL (margin: SLC7A2; PDGFRL) | 7.26 | 7.94e-03 | 3.19e-02 |
|  | 17 | 75471193 | 75471308 | PDFSTATSENSEMBLUCSC | 116 | 4 | 17699 | tss | 0.303943 | 0.981956 | Not queried | 7.26 | 2.86e-02 | 6.12e-02 |
|  | 8 | 142517341 | 142517553 | PDFSTATSENSEMBLUCSC | 213 | 5 | 39930 | tss | 0.027156 | 0.882560 | MROH5 (margin: AC138647.1; MROH5) | 7.25 | 7.94e-03 | 3.19e-02 |
|  | 10 | 75670653 | 75670903 | PDFSTATSENSEMBLUCSC | 251 | 8 | 4638 | gene;island | 0.181772 | 0.971353 | PLAU; C10orf55 (margin: PLAU; C10orf55) | 7.25 | 1.55e-04 | 2.71e-03 |
|  | 1 | 41889271 | 41889625 | PDFSTATSENSEMBLUCSC | 355 | 4 | 3139 | island | 0.862203 | 0.063379 | Not queried | 7.25 | 2.86e-02 | 6.12e-02 |
|  | 7 | 86850357 | 86850688 | PDFSTATSENSEMBLUCSC | 332 | 5 | 38633 | island | 0.495105 | 0.006404 | Not queried | 7.25 | 3.17e-02 | 6.50e-02 |
|  | 15 | 62358751 | 62359275 | PDFSTATSENSEMBLUCSC | 525 | 7 | 14198 | island | 0.560908 | 0.018455 | Not queried | 7.25 | 2.62e-02 | 6.12e-02 |
|  | 10 | 44880542 | 44880819 | PDFSTATSENSEMBLUCSC | 278 | 7 | 5055 | tss;island | 0.814968 | 0.028098 | AL137026.1; CXCL12 (margin: AL137026.1; CXCL12) | 7.25 | 5.83e-04 | 6.47e-03 |
|  | 19 | 12175107 | 12175540 | PDFSTATSENSEMBLUCSC | 434 | 7 | 20488 | tss | 0.174627 | 0.933873 | ZNF844 (margin: ZNF844; RNA5SP466; CTD-2006C1.10; ZNF878) | 7.25 | 5.83e-04 | 6.47e-03 |
|  | 2 | 74753281 | 74753358 | PDFSTATSENSEMBLUCSC | 78 | 4 | 22747 | gene;island | 0.085947 | 0.879355 | Not queried | 7.25 | 2.86e-02 | 6.12e-02 |
|  | 11 | 133800146 | 133800473 | PDFSTATSENSEMBLUCSC | 328 | 4 | 6985 | gene;island | 0.171266 | 0.985737 | Not queried | 7.25 | 2.86e-02 | 6.12e-02 |
|  | 4 | 13546111 | 13546673 | PDFSTATSENSEMBLUCSC | 563 | 10 | 30933 | island | 0.676668 | 0.018335 | NKX3-2; AC006445.8 (margin: NKX3-2; AC006445.8) | 7.24 | 3.25e-04 | 4.91e-03 |
|  | 11 | 8832817 | 8833095 | PDFSTATSENSEMBLUCSC | 279 | 6 | 6500 | gene;tss | 0.111682 | 0.946756 | RP11-318C2.1; ST5 (margin: RP11-318C2.1; ST5) | 7.24 | 2.16e-03 | 1.45e-02 |
|  | 17 | 43922122 | 43922244 | PDFSTATSENSEMBLUCSC | 123 | 4 | 17432 | tss | 0.045442 | 0.848530 | Not queried | 7.24 | 2.86e-02 | 6.12e-02 |
|  | 21 | 45622096 | 45622493 | PDFSTATSENSEMBLUCSC | 398 | 5 | 26731 | island | 0.921074 | 0.072315 | AP001057.1 (margin: AP001057.1; AP001058.3) | 7.24 | 7.94e-03 | 3.19e-02 |
|  | 7 | 150413564 | 150413801 | PDFSTATSENSEMBLUCSC | 238 | 4 | 39012 | island | 0.013508 | 0.710288 | Not queried | 7.23 | 2.86e-02 | 6.12e-02 |
|  | 11 | 6440690 | 6440968 | PDFSTATSENSEMBLUCSC | 279 | 10 | 7110 | tss;island | 0.578195 | 0.018045 | APBB1 (margin: APBB1; HPX) | 7.22 | 3.25e-04 | 4.91e-03 |
|  | 10 | 134901199 | 134901689 | PDFSTATSENSEMBLUCSC | 491 | 9 | 6332 | island | 0.866980 | 0.042019 | GPR123; RP13-439H18.4; AL445199.1 (margin: GPR123; RP13-439H18.4; AL445199.1) | 7.22 | 4.11e-05 | 1.17e-03 |
|  | 19 | 20162642 | 20163138 | PDFSTATSENSEMBLUCSC | 497 | 7 | 21833 | island | 0.311244 | 0.003944 | (margin: AC006539.4) | 7.21 | 5.83e-04 | 6.47e-03 |
|  | 14 | 105044984 | 105045347 | PDFSTATSENSEMBLUCSC | 364 | 4 | 12658 | tss;island | 0.046992 | 0.785534 | Not queried | 7.21 | 2.86e-02 | 6.12e-02 |
|  | 1 | 17381800 | 17382130 | PDFSTATSENSEMBLUCSC | 331 | 4 | 1290 | tss;island | 0.122715 | 0.959489 | Not queried | 7.21 | 5.71e-02 | 1.00e-01 |
|  | 12 | 99548329 | 99548550 | PDFSTATSENSEMBLUCSC | 222 | 5 | 9262 | gene | 0.118350 | 0.931670 | ANKS1B (margin: ANKS1B) | 7.21 | 7.94e-03 | 3.19e-02 |
|  | 13 | 43566114 | 43566642 | PDFSTATSENSEMBLUCSC | 529 | 12 | 11698 | island | 0.127893 | 0.967059 | EPSTI1 (margin: EPSTI1) | 7.21 | 7.40e-07 | 7.21e-05 |
|  | 11 | 6440456 | 6440482 | PDFSTATSENSEMBLUCSC | 27 | 5 | 7109 | tss | 0.589523 | 0.011723 | APBB1 (margin: APBB1; HPX) | 7.21 | 7.94e-03 | 3.19e-02 |
|  | 20 | 20433062 | 20433328 | PDFSTATSENSEMBLUCSC | 267 | 4 | 25373 | gene;island | 0.235161 | 0.972393 | Not queried | 7.20 | 5.71e-02 | 1.00e-01 |
|  | 13 | 88323940 | 88324193 | PDFSTATSENSEMBLUCSC | 254 | 4 | 11493 | tss;island | 0.758001 | 0.024337 | Not queried | 7.20 | 2.86e-02 | 6.12e-02 |
|  | 7 | 98099806 | 98100419 | PDFSTATSENSEMBLUCSC | 614 | 7 | 38713 | island | 0.885376 | 0.031724 | (margin: AC074121.4) | 7.20 | 4.08e-03 | 2.47e-02 |
|  | 2 | 142889283 | 142889392 | PDFSTATSENSEMBLUCSC | 110 | 4 | 23653 | tss;island | 0.349046 | 0.012864 | Not queried | 7.20 | 2.86e-02 | 6.12e-02 |
|  | 7 | 155326713 | 155326803 | PDFSTATSENSEMBLUCSC | 91 | 4 | 38012 | tss;island | 0.020474 | 0.747684 | Not queried | 7.19 | 2.86e-02 | 6.12e-02 |
|  | 1 | 121260494 | 121261146 | PDFSTATSENSEMBLUCSC | 653 | 7 | 3668 | island | 0.028271 | 0.732992 | EMBP1 (margin: EMBP1; RP11-344P13.4) | 7.19 | 4.08e-03 | 2.47e-02 |
|  | 1 | 121260494 | 121260900 | PDFSTATSENSEMBLUCSC | 407 | 5 | 1879 | tss | 0.020804 | 0.732992 | Not queried | 7.19 | 3.17e-02 | 6.50e-02 |
|  | 4 | 77172617 | 77172841 | PDFSTATSENSEMBLUCSC | 225 | 6 | 30447 | tss | 0.104825 | 0.002015 | FAM47E; FAM47E-STBD1 (margin: FAM47E; FAM47E-STBD1) | 7.19 | 4.33e-03 | 2.52e-02 |
|  | 6 | 39902296 | 39902693 | PDFSTATSENSEMBLUCSC | 398 | 8 | 34960 | tss | 0.671991 | 0.016838 | Not queried | 7.19 | 8.30e-02 | 1.40e-01 |
|  | 11 | 122850490 | 122850741 | PDFSTATSENSEMBLUCSC | 252 | 4 | 6949 | gene;island | 0.942926 | 0.209417 | Not queried | 7.19 | 2.86e-02 | 6.12e-02 |
|  | 17 | 39969264 | 39969397 | PDFSTATSENSEMBLUCSC | 134 | 4 | 16572 | gene;tss;island | 0.797285 | 0.028228 | Not queried | 7.19 | 2.86e-02 | 6.12e-02 |
|  | 7 | 5647658 | 5647920 | PDFSTATSENSEMBLUCSC | 263 | 4 | 38234 | island | 0.116213 | 0.942979 | Not queried | 7.19 | 2.86e-02 | 6.12e-02 |
|  | 4 | 144480206 | 144480740 | PDFSTATSENSEMBLUCSC | 535 | 12 | 31337 | island | 0.969658 | 0.244241 | SMARCA5; GUSBP5 (margin: SMARCA5; GUSBP5; RP11-481K16.2) | 7.18 | 7.40e-07 | 7.21e-05 |
|  | 10 | 50341989 | 50342145 | PDFSTATSENSEMBLUCSC | 157 | 4 | 5730 | island | 0.009579 | 0.593901 | Not queried | 7.18 | 2.86e-02 | 6.12e-02 |
|  | 5 | 112073348 | 112073769 | PDFSTATSENSEMBLUCSC | 422 | 15 | 31713 | gene | 0.006453 | 0.436353 | APC (margin: APC) | 7.18 | 1.29e-08 | 3.49e-06 |
|  | 5 | 112073348 | 112073544 | PDFSTATSENSEMBLUCSC | 197 | 11 | 32214 | tss | 0.006453 | 0.439442 | APC (margin: APC) | 7.18 | 2.84e-06 | 1.96e-04 |
|  | 5 | 150400001 | 150400672 | PDFSTATSENSEMBLUCSC | 672 | 6 | 31866 | gene | 0.892062 | 0.076013 | Not queried | 7.18 | 2.60e-02 | 6.12e-02 |
|  | 7 | 95025736 | 95026248 | PDFSTATSENSEMBLUCSC | 513 | 19 | 37726 | tss | 0.169020 | 0.970806 | PON1; PON3 (margin: PON1; PON3; PON2) | 7.17 | 5.66e-11 | 4.35e-08 |
|  | 19 | 7827560 | 7827894 | PDFSTATSENSEMBLUCSC | 335 | 4 | 20406 | tss | 0.274692 | 0.973774 | Not queried | 7.17 | 2.86e-02 | 6.12e-02 |
|  | 10 | 97515222 | 97515398 | PDFSTATSENSEMBLUCSC | 177 | 4 | 4689 | gene;tss | 0.130079 | 0.956813 | Not queried | 7.17 | 2.86e-02 | 6.12e-02 |
|  | 10 | 6622279 | 6622566 | PDFSTATSENSEMBLUCSC | 288 | 8 | 4935 | tss;island | 0.012952 | 0.847074 | PRKCQ-AS1; PRKCQ (margin: PRKCQ-AS1; RP11-5N23.3; PRKCQ) | 7.17 | 6.22e-04 | 6.71e-03 |
|  | 11 | 35441012 | 35441311 | PDFSTATSENSEMBLUCSC | 300 | 6 | 8207 | island | 0.602157 | 0.016611 | RP4-683L5.1; SLC1A2 (margin: RP4-683L5.1; SLC1A2) | 7.17 | 4.33e-03 | 2.52e-02 |
|  | 9 | 841340 | 841636 | PDFSTATSENSEMBLUCSC | 297 | 5 | 40801 | tss;island | 0.183238 | 0.972307 | DMRT1 (margin: DMRT1) | 7.16 | 7.94e-03 | 3.19e-02 |
|  | 7 | 45026259 | 45026583 | PDFSTATSENSEMBLUCSC | 325 | 8 | 37576 | tss;island | 0.009017 | 0.510605 | SNHG15 (margin: MYO1G; SNHG15) | 7.16 | 1.55e-04 | 2.71e-03 |
|  | 12 | 54366760 | 54367008 | PDFSTATSENSEMBLUCSC | 249 | 6 | 10484 | island | 0.004694 | 0.387656 | HOXC11; HOTAIR (margin: HOXC11; HOXC10; HOTAIR; HOXC-AS3) | 7.16 | 2.16e-03 | 1.45e-02 |
|  | 12 | 54366760 | 54366875 | PDFSTATSENSEMBLUCSC | 116 | 4 | 9749 | tss | 0.004694 | 0.422591 | Not queried | 7.16 | 2.86e-02 | 6.12e-02 |
|  | 15 | 45421578 | 45422054 | PDFSTATSENSEMBLUCSC | 477 | 8 | 13327 | gene | 0.179751 | 0.938979 | DUOX1; DUOXA1 (margin: DUOXA2; DUOX1; DUOXA1) | 7.16 | 1.55e-04 | 2.71e-03 |
|  | 7 | 50633725 | 50634252 | PDFSTATSENSEMBLUCSC | 528 | 6 | 37595 | tss | 0.050865 | 0.840452 | DDC (margin: DDC) | 7.15 | 2.16e-03 | 1.45e-02 |
|  | 4 | 118006405 | 118006832 | PDFSTATSENSEMBLUCSC | 428 | 9 | 31264 | island | 0.512939 | 0.007954 | TRAM1L1 (margin: TRAM1L1) | 7.14 | 4.11e-05 | 1.17e-03 |
|  | 10 | 94833520 | 94833658 | PDFSTATSENSEMBLUCSC | 139 | 7 | 4680 | gene;island | 0.469482 | 0.007278 | CYP26A1 (margin: CYP26C1; CYP26A1) | 7.14 | 5.83e-04 | 6.47e-03 |
|  | 14 | 27067638 | 27068135 | PDFSTATSENSEMBLUCSC | 498 | 5 | 12334 | tss;island | 0.944505 | 0.091814 | Not queried | 7.13 | 3.17e-02 | 6.50e-02 |
|  | 5 | 16508920 | 16509123 | PDFSTATSENSEMBLUCSC | 204 | 4 | 31567 | gene | 0.022197 | 0.623388 | Not queried | 7.13 | 2.86e-02 | 6.12e-02 |
|  | 19 | 16830613 | 16830749 | PDFSTATSENSEMBLUCSC | 137 | 4 | 20575 | tss | 0.014028 | 0.491361 | Not queried | 7.13 | 2.86e-02 | 6.12e-02 |
|  | 4 | 190731443 | 190731885 | PDFSTATSENSEMBLUCSC | 443 | 5 | 31493 | island | 0.286342 | 0.989105 | Not queried | 7.12 | 3.17e-02 | 6.50e-02 |
|  | 15 | 48470205 | 48470516 | PDFSTATSENSEMBLUCSC | 312 | 4 | 13336 | gene | 0.851102 | 0.024578 | Not queried | 7.12 | 2.86e-02 | 6.12e-02 |
|  | 2 | 1879820 | 1880131 | PDFSTATSENSEMBLUCSC | 312 | 4 | 22539 | gene | 0.276802 | 0.963712 | Not queried | 7.12 | 2.86e-02 | 6.12e-02 |
|  | 8 | 12990213 | 12991196 | PDFSTATSENSEMBLUCSC | 984 | 12 | 39212 | gene;island | 0.391183 | 0.005200 | DLC1 (margin: DLC1) | 7.12 | 7.40e-07 | 7.21e-05 |
|  | 14 | 54413218 | 54413931 | PDFSTATSENSEMBLUCSC | 714 | 6 | 12852 | island | 0.771558 | 0.023964 | Not queried | 7.12 | 2.60e-02 | 6.12e-02 |
|  | 4 | 48491941 | 48492344 | PDFSTATSENSEMBLUCSC | 404 | 8 | 31033 | island | 0.141045 | 0.955433 | SLC10A4; ZAR1 (margin: SLC10A4; ZAR1; FRYL) | 7.12 | 1.55e-04 | 2.71e-03 |
|  | 20 | 45523098 | 45523294 | PDFSTATSENSEMBLUCSC | 197 | 5 | 26222 | island | 0.312438 | 0.003545 | EYA2 (margin: EYA2; RP11-323C15.2) | 7.12 | 7.94e-03 | 3.19e-02 |
|  | 8 | 80803196 | 80803526 | PDFSTATSENSEMBLUCSC | 331 | 5 | 40410 | island | 0.291396 | 0.004024 | (margin: ) | 7.11 | 7.94e-03 | 3.19e-02 |
|  | 8 | 63998640 | 63998778 | PDFSTATSENSEMBLUCSC | 139 | 6 | 39731 | tss | 0.526332 | 0.009706 | TTPA (margin: TTPA) | 7.11 | 2.16e-03 | 1.45e-02 |
|  | 6 | 33172817 | 33173581 | PDFSTATSENSEMBLUCSC | 765 | 17 | 34228 | gene;island | 0.007682 | 0.387922 | SLC39A7; HSD17B8 (margin: RNY4P10; SLC39A7; HSD17B8; MIR219-1; RING1; ZNF70P1; RXRB) | 7.11 | 1.71e-09 | 6.42e-07 |
|  | 11 | 26353484 | 26353811 | PDFSTATSENSEMBLUCSC | 328 | 8 | 8140 | island | 0.616941 | 0.020281 | ANO3 (margin: ANO3) | 7.11 | 1.55e-04 | 2.71e-03 |
|  | 7 | 1491787 | 1492018 | PDFSTATSENSEMBLUCSC | 232 | 5 | 36753 | gene;island | 0.149291 | 0.969127 | MICALL2 (margin: AC102953.4; MICALL2) | 7.11 | 7.94e-03 | 3.19e-02 |
|  | 4 | 96470887 | 96471143 | PDFSTATSENSEMBLUCSC | 257 | 4 | 30506 | tss;island | 0.690041 | 0.023341 | Not queried | 7.11 | 2.86e-02 | 6.12e-02 |
|  | 16 | 58059105 | 58059154 | PDFSTATSENSEMBLUCSC | 50 | 5 | 15269 | tss | 0.179183 | 0.003014 | MMP15 (margin: USB1; MMP15) | 7.11 | 7.94e-03 | 3.19e-02 |
|  | 13 | 93878969 | 93879042 | PDFSTATSENSEMBLUCSC | 74 | 4 | 11497 | tss;island | 0.316319 | 0.008931 | Not queried | 7.10 | 5.71e-02 | 1.00e-01 |
|  | 5 | 87971794 | 87972265 | PDFSTATSENSEMBLUCSC | 472 | 7 | 31679 | gene;island | 0.459419 | 0.008097 | CTC-467M3.1; LINC00461 (margin: CTC-467M3.1; LINC00461) | 7.10 | 5.83e-04 | 6.47e-03 |
|  | 10 | 97666894 | 97667244 | PDFSTATSENSEMBLUCSC | 351 | 6 | 5250 | tss;island | 0.474981 | 0.006395 | RP11-248J23.7; C10orf131; ENTPD1-AS1 (margin: RP11-248J23.7; C10orf131; ENTPD1-AS1) | 7.10 | 2.16e-03 | 1.45e-02 |
|  | 12 | 34175262 | 34175567 | PDFSTATSENSEMBLUCSC | 306 | 5 | 9094 | gene | 0.002575 | 0.214019 | Not queried | 7.09 | 1.59e-02 | 5.36e-02 |
|  | 12 | 52400530 | 52400667 | PDFSTATSENSEMBLUCSC | 138 | 4 | 9722 | tss;island | 0.340461 | 0.981769 | Not queried | 7.08 | 2.86e-02 | 6.12e-02 |
|  | 12 | 51818350 | 51818768 | PDFSTATSENSEMBLUCSC | 419 | 7 | 10427 | island | 0.466417 | 0.008697 | SLC4A8; RP11-607P23.1 (margin: SLC4A8; RP11-607P23.1) | 7.08 | 1.17e-03 | 1.13e-02 |
|  | 12 | 6442873 | 6443045 | PDFSTATSENSEMBLUCSC | 173 | 4 | 9026 | gene | 0.232545 | 0.978441 | Not queried | 7.07 | 2.86e-02 | 6.12e-02 |
|  | 1 | 40420800 | 40421002 | PDFSTATSENSEMBLUCSC | 203 | 4 | 449 | gene | 0.018113 | 0.515649 | Not queried | 7.07 | 2.86e-02 | 6.12e-02 |
|  | 5 | 147443067 | 147443347 | PDFSTATSENSEMBLUCSC | 281 | 4 | 32410 | tss | 0.085861 | 0.930969 | Not queried | 7.07 | 2.86e-02 | 6.12e-02 |
|  | 3 | 187457128 | 187457732 | PDFSTATSENSEMBLUCSC | 605 | 7 | 28079 | gene;island | 0.087885 | 0.867960 | BCL6 (margin: RP11-211G3.3; RP11-211G3.2; BCL6) | 7.07 | 5.83e-04 | 6.47e-03 |
|  | 12 | 116996773 | 116997095 | PDFSTATSENSEMBLUCSC | 323 | 8 | 10038 | tss | 0.161729 | 0.969115 | MAP1LC3B2 (margin: RP11-809C9.2; MAP1LC3B2) | 7.06 | 1.09e-03 | 1.10e-02 |
|  | 19 | 54410030 | 54410305 | PDFSTATSENSEMBLUCSC | 276 | 4 | 20211 | gene;island | 0.513490 | 0.004030 | Not queried | 7.06 | 2.86e-02 | 6.12e-02 |
|  | 1 | 63249197 | 63249213 | PDFSTATSENSEMBLUCSC | 17 | 4 | 1666 | tss | 0.315848 | 0.004214 | Not queried | 7.05 | 2.86e-02 | 6.12e-02 |
|  | 4 | 164264796 | 164265012 | PDFSTATSENSEMBLUCSC | 217 | 7 | 30661 | tss;island | 0.680610 | 0.022560 | NPY5R; NPY1R (margin: NPY5R; NPY1R) | 7.05 | 5.83e-04 | 6.47e-03 |
|  | 4 | 48491941 | 48492304 | PDFSTATSENSEMBLUCSC | 364 | 7 | 30383 | tss | 0.172506 | 0.946471 | SLC10A4; ZAR1 (margin: SLC10A4; ZAR1; FRYL) | 7.04 | 5.83e-04 | 6.47e-03 |
|  | 19 | 37288291 | 37288793 | PDFSTATSENSEMBLUCSC | 503 | 6 | 21946 | island | 0.673186 | 0.018297 | CTD-2162K18.5 (margin: CTD-2162K18.5) | 7.04 | 2.16e-03 | 1.45e-02 |
|  | 17 | 76976091 | 76976357 | PDFSTATSENSEMBLUCSC | 267 | 5 | 17709 | tss | 0.086381 | 0.943434 | Not queried | 7.04 | 3.17e-02 | 6.50e-02 |
|  | 4 | 6202379 | 6202609 | PDFSTATSENSEMBLUCSC | 231 | 6 | 30278 | tss;island | 0.623207 | 0.013714 | RP11-586D19.1; RP11-586D19.2; JAKMIP1 (margin: RP11-586D19.1; RP11-586D19.2; JAKMIP1) | 7.04 | 2.16e-03 | 1.45e-02 |
|  | 5 | 156886816 | 156887005 | PDFSTATSENSEMBLUCSC | 190 | 6 | 32456 | tss | 0.163736 | 0.951439 | NIPAL4; CTB-109A12.1; ADAM19 (margin: NIPAL4; CTB-109A12.1; ADAM19) | 7.04 | 2.16e-03 | 1.45e-02 |
|  | 6 | 147827800 | 147828073 | PDFSTATSENSEMBLUCSC | 274 | 4 | 36548 | island | 0.162236 | 0.944442 | Not queried | 7.04 | 2.86e-02 | 6.12e-02 |
|  | 11 | 120195717 | 120195905 | PDFSTATSENSEMBLUCSC | 189 | 5 | 7762 | tss;island | 0.852304 | 0.044329 | TMEM136 (margin: POU2F3; TMEM136; ARHGEF12) | 7.04 | 7.94e-03 | 3.19e-02 |
|  | 17 | 21454528 | 21454896 | PDFSTATSENSEMBLUCSC | 369 | 4 | 16491 | gene | 0.009688 | 0.503583 | Not queried | 7.03 | 2.86e-02 | 6.12e-02 |
|  | 19 | 50004152 | 50004625 | PDFSTATSENSEMBLUCSC | 474 | 6 | 20952 | tss | 0.281142 | 0.936057 | RPS11; hsa-mir-150; MIR150; CTD-3148I10.13 (margin: RPL13A; SNORD32A; SNORD33; SNORD34; SNORD35A; RPS11; SNORD35B; hsa-mir-150; FCGRT; MIR150; CTD-3148I10.13) | 7.03 | 2.16e-03 | 1.45e-02 |
|  | 2 | 210636350 | 210636560 | PDFSTATSENSEMBLUCSC | 211 | 5 | 23852 | tss | 0.825957 | 0.022004 | UNC80 (margin: UNC80) | 7.03 | 7.94e-03 | 3.19e-02 |
|  | 13 | 113776873 | 113776964 | PDFSTATSENSEMBLUCSC | 92 | 4 | 11957 | island | 0.247083 | 0.972170 | Not queried | 7.03 | 2.86e-02 | 6.12e-02 |
|  | 5 | 115910592 | 115910732 | PDFSTATSENSEMBLUCSC | 141 | 4 | 32233 | tss | 0.456507 | 0.007753 | Not queried | 7.02 | 2.86e-02 | 6.12e-02 |
|  | 15 | 66947392 | 66947617 | PDFSTATSENSEMBLUCSC | 226 | 4 | 14245 | island | 0.946461 | 0.105753 | Not queried | 7.02 | 2.86e-02 | 6.12e-02 |
|  | 10 | 18629497 | 18629602 | PDFSTATSENSEMBLUCSC | 106 | 4 | 4974 | tss | 0.190985 | 0.959524 | Not queried | 7.02 | 2.86e-02 | 6.12e-02 |
|  | 1 | 220921216 | 220922217 | PDFSTATSENSEMBLUCSC | 1002 | 14 | 4219 | island | 0.534819 | 0.010064 | MARC2 (margin: MARC2) | 7.01 | 4.99e-08 | 9.34e-06 |
|  | 6 | 33538979 | 33539394 | PDFSTATSENSEMBLUCSC | 416 | 5 | 36041 | island | 0.869745 | 0.066346 | BAK1 (margin: BAK1) | 7.01 | 7.94e-03 | 3.19e-02 |
|  | 13 | 23734299 | 23734512 | PDFSTATSENSEMBLUCSC | 214 | 4 | 11596 | island | 0.505708 | 0.010404 | Not queried | 7.01 | 2.86e-02 | 6.12e-02 |
|  | 16 | 53468112 | 53468350 | PDFSTATSENSEMBLUCSC | 239 | 5 | 15925 | island | 0.018294 | 0.004797 | Not queried | 7.00 | 2.22e-01 | 2.76e-01 |
|  | 11 | 2919689 | 2919808 | PDFSTATSENSEMBLUCSC | 120 | 4 | 6468 | gene;tss;island | 0.114435 | 0.909422 | Not queried | 7.00 | 5.71e-02 | 1.00e-01 |
|  | 22 | 45405621 | 45405919 | PDFSTATSENSEMBLUCSC | 299 | 9 | 27216 | tss;island | 0.487334 | 0.009462 | PHF21B (margin: PHF21B; RP4-753M9.1) | 7.00 | 2.88e-04 | 4.58e-03 |
|  | 5 | 121648313 | 121648479 | PDFSTATSENSEMBLUCSC | 167 | 4 | 31727 | gene;island | 0.119722 | 0.964617 | Not queried | 7.00 | 2.86e-02 | 6.12e-02 |
|  | 17 | 8869099 | 8869285 | PDFSTATSENSEMBLUCSC | 187 | 6 | 17124 | tss;island | 0.777673 | 0.023190 | CTB-41I6.1; CTB-41I6.2; PIK3R5 (margin: CTB-41I6.1; CTB-41I6.2; PIK3R5) | 7.00 | 2.16e-03 | 1.45e-02 |
|  | 10 | 133949683 | 133950036 | PDFSTATSENSEMBLUCSC | 354 | 4 | 4852 | gene;island | 0.146904 | 0.854213 | Not queried | 7.00 | 2.86e-02 | 6.12e-02 |
|  | 5 | 140261653 | 140261810 | PDFSTATSENSEMBLUCSC | 158 | 6 | 32356 | tss | 0.981093 | 0.294043 | PCDHA1; PCDHA2; PCDHA3; PCDHA4; PCDHA5; PCDHA6; PCDHA7; PCDHA8; PCDHA9; PCDHA10; PCDHA11; PCDHA12; PCDHA13 (margin: PCDHA1; PCDHA2; PCDHA3; PCDHA4; PCDHA5; PCDHA6; PCDHA7; PCDHA8; PCDHA9; PCDHA10; PCDHA11; PCDHA12; PCDHA13) | 7.00 | 8.66e-03 | 3.36e-02 |
|  | 6 | 151186411 | 151186614 | PDFSTATSENSEMBLUCSC | 204 | 5 | 35297 | tss | 0.094617 | 0.002454 | Not queried | 6.99 | 4.21e-01 | 4.59e-01 |
|  | 17 | 38599271 | 38599763 | PDFSTATSENSEMBLUCSC | 493 | 6 | 18306 | island | 0.827302 | 0.022852 | IGFBP4 (margin: IGFBP4; RP11-58O9.1) | 6.99 | 2.16e-03 | 1.45e-02 |
|  | 17 | 78237389 | 78237543 | PDFSTATSENSEMBLUCSC | 155 | 4 | 16864 | gene;island | 0.151598 | 0.966284 | Not queried | 6.99 | 2.86e-02 | 6.12e-02 |
|  | 15 | 40697419 | 40698044 | PDFSTATSENSEMBLUCSC | 626 | 7 | 14037 | island | 0.346119 | 0.006987 | Not queried | 6.98 | 2.62e-02 | 6.12e-02 |
|  | 3 | 186649165 | 186649855 | PDFSTATSENSEMBLUCSC | 691 | 6 | 28076 | gene;island | 0.637317 | 0.014181 | ST6GAL1; AC007690.1 (margin: ST6GAL1; AC007690.1; RP11-42D20.1) | 6.98 | 2.16e-03 | 1.45e-02 |
|  | 16 | 67450370 | 67450810 | PDFSTATSENSEMBLUCSC | 441 | 6 | 15302 | tss;island | 0.180101 | 0.936257 | Not queried | 6.98 | 1.52e-02 | 5.36e-02 |
|  | 10 | 73533407 | 73533561 | PDFSTATSENSEMBLUCSC | 155 | 7 | 5145 | tss | 0.008946 | 0.573310 | CDH23; C10orf54 (margin: CDH23; C10orf54) | 6.98 | 5.83e-04 | 6.47e-03 |
|  | 5 | 73937141 | 73937330 | PDFSTATSENSEMBLUCSC | 190 | 5 | 32878 | island | 0.292345 | 0.003646 | Not queried | 6.98 | 3.17e-02 | 6.50e-02 |
|  | 6 | 40345376 | 40345751 | PDFSTATSENSEMBLUCSC | 376 | 4 | 34961 | tss | 0.265451 | 0.964634 | Not queried | 6.98 | 2.86e-02 | 6.12e-02 |
|  | 6 | 74104388 | 74104600 | PDFSTATSENSEMBLUCSC | 213 | 4 | 34388 | gene;island | 0.987524 | 0.384459 | Not queried | 6.97 | 2.86e-02 | 6.12e-02 |
|  | 5 | 154091976 | 154092426 | PDFSTATSENSEMBLUCSC | 451 | 6 | 32450 | tss | 0.630488 | 0.012511 | LARP1 (margin: LARP1; RN7SL439P; Metazoa\_SRP) | 6.97 | 2.16e-03 | 1.45e-02 |
|  | 5 | 140261653 | 140261930 | PDFSTATSENSEMBLUCSC | 278 | 7 | 31803 | gene;island | 0.979313 | 0.309877 | Not queried | 6.97 | 1.75e-02 | 5.79e-02 |
|  | 19 | 18063041 | 18063313 | PDFSTATSENSEMBLUCSC | 273 | 4 | 19925 | gene;island | 0.555135 | 0.023125 | Not queried | 6.97 | 2.86e-02 | 6.12e-02 |
|  | 12 | 52845930 | 52846088 | PDFSTATSENSEMBLUCSC | 159 | 4 | 9728 | tss | 0.189236 | 0.956476 | Not queried | 6.97 | 2.86e-02 | 6.12e-02 |
|  | 5 | 122426096 | 122426400 | PDFSTATSENSEMBLUCSC | 305 | 4 | 31729 | gene;island | 0.795984 | 0.042409 | Not queried | 6.97 | 2.86e-02 | 6.12e-02 |
|  | 22 | 38851884 | 38852154 | PDFSTATSENSEMBLUCSC | 271 | 4 | 27140 | tss;island | 0.553633 | 0.015895 | Not queried | 6.96 | 5.71e-02 | 1.00e-01 |
|  | 1 | 29101849 | 29102153 | PDFSTATSENSEMBLUCSC | 305 | 5 | 2952 | island | 0.194740 | 0.973939 | (margin: YTHDF2) | 6.96 | 7.94e-03 | 3.19e-02 |
|  | 2 | 241721922 | 241722281 | PDFSTATSENSEMBLUCSC | 360 | 4 | 23125 | gene;island | 0.275604 | 0.969753 | Not queried | 6.96 | 2.86e-02 | 6.12e-02 |
|  | 4 | 94749865 | 94750205 | PDFSTATSENSEMBLUCSC | 341 | 7 | 31186 | island | 0.541368 | 0.009574 | ATOH1 (margin: RNA5SP164; ATOH1) | 6.96 | 5.83e-04 | 6.47e-03 |
|  | 11 | 34535085 | 34535281 | PDFSTATSENSEMBLUCSC | 197 | 4 | 6581 | gene | 0.938586 | 0.242154 | Not queried | 6.96 | 2.86e-02 | 6.12e-02 |
|  | 8 | 67940823 | 67941178 | PDFSTATSENSEMBLUCSC | 356 | 7 | 39749 | tss | 0.624074 | 0.011857 | PPP1R42 (margin: PPP1R42) | 6.96 | 5.83e-04 | 6.47e-03 |
|  | 6 | 80657412 | 80657555 | PDFSTATSENSEMBLUCSC | 144 | 7 | 35083 | tss;island | 0.579808 | 0.011203 | ELOVL4 (margin: ELOVL4; GAPDHP63) | 6.95 | 5.83e-04 | 6.47e-03 |
|  | 12 | 123319216 | 123319366 | PDFSTATSENSEMBLUCSC | 151 | 4 | 10089 | tss;island | 0.620060 | 0.013265 | Not queried | 6.95 | 2.86e-02 | 6.12e-02 |
|  | 2 | 70995349 | 70995607 | PDFSTATSENSEMBLUCSC | 259 | 8 | 23420 | tss;island | 0.359977 | 0.985026 | ADD2 (margin: ADD2; FIGLA) | 6.95 | 1.55e-04 | 2.71e-03 |
|  | 22 | 38506712 | 38506781 | PDFSTATSENSEMBLUCSC | 70 | 4 | 27131 | tss | 0.018130 | 0.692891 | Not queried | 6.95 | 2.86e-02 | 6.12e-02 |
|  | 8 | 75896975 | 75897310 | PDFSTATSENSEMBLUCSC | 336 | 4 | 39339 | gene | 0.436059 | 0.008201 | Not queried | 6.94 | 2.86e-02 | 6.12e-02 |
|  | 11 | 133907114 | 133907497 | PDFSTATSENSEMBLUCSC | 384 | 4 | 6988 | gene;island | 0.306986 | 0.004485 | Not queried | 6.94 | 5.71e-02 | 1.00e-01 |
|  | 16 | 54968727 | 54969146 | PDFSTATSENSEMBLUCSC | 420 | 6 | 15932 | island | 0.899024 | 0.089233 | Not queried | 6.94 | 2.60e-02 | 6.12e-02 |
|  | 7 | 37488162 | 37488438 | PDFSTATSENSEMBLUCSC | 277 | 4 | 36968 | gene | 0.886184 | 0.052820 | Not queried | 6.94 | 2.86e-02 | 6.12e-02 |
|  | 4 | 5712581 | 5713100 | PDFSTATSENSEMBLUCSC | 520 | 9 | 30880 | island | 0.022459 | 0.670293 | EVC; EVC2 (margin: EVC; EVC2) | 6.94 | 8.23e-05 | 2.06e-03 |
|  | 7 | 23287222 | 23287560 | PDFSTATSENSEMBLUCSC | 339 | 4 | 36914 | gene;island | 0.366650 | 0.986192 | Not queried | 6.93 | 2.86e-02 | 6.12e-02 |
|  | 3 | 44902933 | 44903330 | PDFSTATSENSEMBLUCSC | 398 | 8 | 28264 | tss | 0.020158 | 0.510301 | KIF15; TMEM42; MIR564 (margin: KIF15; TMEM42; MIR564) | 6.93 | 1.55e-04 | 2.71e-03 |
|  | 1 | 67520192 | 67520352 | PDFSTATSENSEMBLUCSC | 161 | 4 | 1682 | tss;island | 0.350050 | 0.010645 | Not queried | 6.92 | 2.86e-02 | 6.12e-02 |
|  | 5 | 150399781 | 150400672 | PDFSTATSENSEMBLUCSC | 892 | 9 | 33315 | island | 0.956282 | 0.106810 | GPX3 (margin: GPX3; TNIP1) | 6.92 | 1.85e-03 | 1.45e-02 |
|  | 1 | 143913409 | 143913569 | PDFSTATSENSEMBLUCSC | 161 | 4 | 1880 | tss;island | 0.007323 | 0.420702 | Not queried | 6.92 | 2.86e-02 | 6.12e-02 |
|  | 5 | 1008828 | 1009204 | PDFSTATSENSEMBLUCSC | 377 | 5 | 32600 | island | 0.411998 | 0.010622 | NKD2 (margin: AC116351.2; RP11-43F13.4; NKD2; RP11-43F13.3) | 6.91 | 7.94e-03 | 3.19e-02 |
|  | 1 | 183154981 | 183155154 | PDFSTATSENSEMBLUCSC | 174 | 5 | 2134 | tss | 0.092016 | 0.915549 | LAMC2 (margin: LAMC2) | 6.91 | 7.94e-03 | 3.19e-02 |
|  | 10 | 133793442 | 133793734 | PDFSTATSENSEMBLUCSC | 293 | 5 | 4847 | gene;island | 0.982381 | 0.224085 | BNIP3 (margin: BNIP3) | 6.91 | 7.94e-03 | 3.19e-02 |
|  | 1 | 24194307 | 24194573 | PDFSTATSENSEMBLUCSC | 267 | 5 | 323 | gene;island | 0.355572 | 0.009865 | Not queried | 6.90 | 3.17e-02 | 6.50e-02 |
|  | 7 | 86850357 | 86850525 | PDFSTATSENSEMBLUCSC | 169 | 4 | 37684 | tss | 0.343267 | 0.004311 | Not queried | 6.90 | 2.86e-02 | 6.12e-02 |
|  | 1 | 3163341 | 3163710 | PDFSTATSENSEMBLUCSC | 370 | 4 | 113 | gene;island | 0.071225 | 0.970414 | Not queried | 6.90 | 1.14e-01 | 1.68e-01 |
|  | 17 | 9929528 | 9929867 | PDFSTATSENSEMBLUCSC | 340 | 5 | 16440 | gene | 0.015714 | 0.685017 | GAS7 (margin: GAS7) | 6.90 | 7.94e-03 | 3.19e-02 |
|  | 19 | 12175107 | 12175719 | PDFSTATSENSEMBLUCSC | 613 | 10 | 21591 | island | 0.293095 | 0.937219 | ZNF844 (margin: ZNF844; RNA5SP466; CTD-2006C1.10; ZNF878) | 6.89 | 1.08e-05 | 4.82e-04 |
|  | 3 | 73673893 | 73674702 | PDFSTATSENSEMBLUCSC | 810 | 15 | 29286 | island | 0.642287 | 0.010218 | PDZRN3-AS1; PDZRN3 (margin: PDZRN3-AS1; PDZRN3) | 6.89 | 1.29e-08 | 3.49e-06 |
|  | 10 | 86001241 | 86001560 | PDFSTATSENSEMBLUCSC | 320 | 7 | 5194 | tss | 0.024339 | 0.916705 | LRIT1 (margin: RGR; LRIT1) | 6.89 | 4.08e-03 | 2.47e-02 |
|  | 7 | 24796981 | 24797884 | PDFSTATSENSEMBLUCSC | 904 | 12 | 38318 | island | 0.245013 | 0.965274 | DFNA5 (margin: DFNA5) | 6.88 | 3.64e-03 | 2.29e-02 |
|  | 11 | 62477052 | 62477624 | PDFSTATSENSEMBLUCSC | 573 | 9 | 7361 | tss | 0.410492 | 0.988075 | GNG3; BSCL2; RP11-831H9.16 (margin: GNG3; BSCL2; RP11-831H9.16; HNRNPUL2) | 6.88 | 4.11e-05 | 1.17e-03 |
|  | 16 | 30572739 | 30573013 | PDFSTATSENSEMBLUCSC | 275 | 5 | 15836 | island | 0.445333 | 0.989192 | (margin: AC002310.7; AC002310.13; ZNF764; ZNF688) | 6.88 | 7.94e-03 | 3.19e-02 |
|  | 6 | 166666838 | 166667648 | PDFSTATSENSEMBLUCSC | 811 | 7 | 36628 | island | 0.287847 | 0.969182 | Not queried | 6.88 | 1.75e-02 | 5.79e-02 |
|  | 6 | 166419479 | 166419943 | PDFSTATSENSEMBLUCSC | 465 | 6 | 36625 | island | 0.304983 | 0.004398 | (margin: ) | 6.88 | 4.33e-03 | 2.52e-02 |
|  | 10 | 131265470 | 131265796 | PDFSTATSENSEMBLUCSC | 327 | 4 | 4823 | gene | 0.271264 | 0.004495 | Not queried | 6.87 | 2.86e-02 | 6.12e-02 |
|  | 6 | 4282075 | 4282346 | PDFSTATSENSEMBLUCSC | 272 | 4 | 35394 | island | 0.084029 | 0.909710 | Not queried | 6.87 | 2.86e-02 | 6.12e-02 |
|  | 3 | 38664022 | 38664451 | PDFSTATSENSEMBLUCSC | 430 | 4 | 27722 | gene | 0.146508 | 0.943008 | Not queried | 6.87 | 5.71e-02 | 1.00e-01 |
|  | 6 | 150346721 | 150347053 | PDFSTATSENSEMBLUCSC | 333 | 10 | 35294 | tss | 0.014398 | 0.685007 | RAET1L (margin: RAET1M; RAET1L) | 6.87 | 2.17e-05 | 8.94e-04 |
|  | 6 | 26188969 | 26189597 | PDFSTATSENSEMBLUCSC | 629 | 10 | 35522 | island | 0.003306 | 0.279103 | HIST1H4D (margin: HIST1H2BE; HIST1H1PS1; HIST1H2BF; HIST1H4D; HIST1H3D; HIST1H2AD) | 6.87 | 1.08e-05 | 4.82e-04 |
|  | 14 | 75535757 | 75536561 | PDFSTATSENSEMBLUCSC | 805 | 9 | 13012 | island | 0.008778 | 0.498084 | ZC2HC1C; ACYP1 (margin: ZC2HC1C; ACYP1) | 6.87 | 4.11e-05 | 1.17e-03 |
|  | 22 | 37640314 | 37640514 | PDFSTATSENSEMBLUCSC | 201 | 5 | 27115 | tss | 0.025419 | 0.747675 | RAC2 (margin: RAC2) | 6.87 | 7.94e-03 | 3.19e-02 |
|  | 1 | 247275326 | 247275542 | PDFSTATSENSEMBLUCSC | 217 | 5 | 1124 | gene | 0.571079 | 0.009801 | C1orf229 (margin: FGFR3P6; ZNF669; C1orf229; ZNF124) | 6.87 | 7.94e-03 | 3.19e-02 |
|  | 13 | 114780772 | 114780992 | PDFSTATSENSEMBLUCSC | 221 | 4 | 11304 | gene;island | 0.170166 | 0.935994 | Not queried | 6.87 | 2.86e-02 | 6.12e-02 |
|  | 6 | 108485043 | 108485182 | PDFSTATSENSEMBLUCSC | 140 | 4 | 36395 | island | 0.136404 | 0.852090 | Not queried | 6.87 | 2.86e-02 | 6.12e-02 |
|  | 5 | 174151043 | 174151567 | PDFSTATSENSEMBLUCSC | 525 | 7 | 32505 | tss | 0.355032 | 0.003599 | MSX2 (margin: MSX2) | 6.86 | 1.17e-03 | 1.13e-02 |
|  | 16 | 66638210 | 66638702 | PDFSTATSENSEMBLUCSC | 493 | 10 | 14782 | gene;island | 0.731925 | 0.009440 | CMTM3 (margin: CMTM3; CMTM4) | 6.86 | 1.05e-03 | 1.08e-02 |
|  | 12 | 66583029 | 66583431 | PDFSTATSENSEMBLUCSC | 403 | 5 | 9207 | gene;island | 0.249094 | 0.984925 | IRAK3 (margin: IRAK3) | 6.86 | 7.94e-03 | 3.19e-02 |
|  | 6 | 32371488 | 32371538 | PDFSTATSENSEMBLUCSC | 51 | 4 | 34109 | gene | 0.061631 | 0.845348 | Not queried | 6.86 | 1.14e-01 | 1.68e-01 |
|  | 4 | 57976431 | 57976944 | PDFSTATSENSEMBLUCSC | 514 | 9 | 31076 | island | 0.013286 | 0.637867 | RP11-12A1.1; IGFBP7 (margin: RP11-12A1.1; IGFBP7; snoU13) | 6.85 | 4.11e-05 | 1.17e-03 |
|  | 10 | 75670653 | 75670793 | PDFSTATSENSEMBLUCSC | 141 | 7 | 5166 | tss | 0.208011 | 0.960350 | PLAU; C10orf55 (margin: PLAU; C10orf55) | 6.85 | 5.83e-04 | 6.47e-03 |
|  | 4 | 93226245 | 93226625 | PDFSTATSENSEMBLUCSC | 381 | 5 | 30061 | gene;island | 0.863815 | 0.058550 | GRID2; RP11-9B6.1 (margin: GRID2; RP11-9B6.1) | 6.84 | 7.94e-03 | 3.19e-02 |
|  | 3 | 132378605 | 132379275 | PDFSTATSENSEMBLUCSC | 671 | 9 | 27950 | gene;tss;island | 0.005477 | 0.401725 | UBA5; ACAD11; NPHP3 (margin: HSPA8P19; UBA5; ACAD11; NPHP3) | 6.84 | 4.11e-05 | 1.17e-03 |
|  | 1 | 78354053 | 78354406 | PDFSTATSENSEMBLUCSC | 354 | 4 | 3400 | island | 0.468879 | 0.985661 | Not queried | 6.84 | 2.86e-02 | 6.12e-02 |
|  | 7 | 87563462 | 87563931 | PDFSTATSENSEMBLUCSC | 470 | 11 | 38639 | island | 0.494824 | 0.010785 | ADAM22 (margin: ADAM22) | 6.84 | 5.67e-06 | 3.54e-04 |
|  | 12 | 89746101 | 89746771 | PDFSTATSENSEMBLUCSC | 671 | 10 | 10664 | island | 0.002736 | 0.231467 | DUSP6 (margin: DUSP6) | 6.84 | 1.08e-05 | 4.82e-04 |
|  | 20 | 36793705 | 36794002 | PDFSTATSENSEMBLUCSC | 298 | 10 | 25731 | tss | 0.638531 | 0.011227 | TGM2 (margin: TGM2) | 6.84 | 1.08e-05 | 4.82e-04 |
|  | 11 | 113906981 | 113907379 | PDFSTATSENSEMBLUCSC | 399 | 4 | 8821 | island | 0.629374 | 0.015923 | Not queried | 6.84 | 2.86e-02 | 6.12e-02 |
|  | 15 | 91497863 | 91498497 | PDFSTATSENSEMBLUCSC | 635 | 10 | 14464 | island | 0.762567 | 0.026799 | UNC45A; RCCD1; AC068831.6 (margin: UNC45A; RCCD1; PRC1-AS1; AC068831.6; PRC1) | 6.83 | 1.08e-05 | 4.82e-04 |
|  | 1 | 155035008 | 155035530 | PDFSTATSENSEMBLUCSC | 523 | 5 | 1978 | tss;island | 0.921286 | 0.055797 | ADAM15; EFNA4; EFNA3 (margin: DCST1; ADAM15; EFNA4; EFNA3) | 6.83 | 7.94e-03 | 3.19e-02 |
|  | 11 | 62476891 | 62477624 | PDFSTATSENSEMBLUCSC | 734 | 10 | 8355 | island | 0.388633 | 0.986732 | GNG3; BSCL2; RP11-831H9.16 (margin: GNG3; BSCL2; RP11-831H9.16; HNRNPUL2) | 6.83 | 4.33e-05 | 1.22e-03 |
|  | 10 | 35484768 | 35484972 | PDFSTATSENSEMBLUCSC | 205 | 4 | 4558 | gene | 0.011346 | 0.398793 | Not queried | 6.83 | 5.71e-02 | 1.00e-01 |
|  | 3 | 181444368 | 181445156 | PDFSTATSENSEMBLUCSC | 789 | 9 | 28045 | gene;island | 0.169176 | 0.971031 | SOX2-OT (margin: SOX2-OT) | 6.83 | 8.23e-05 | 2.06e-03 |
|  | 19 | 54219381 | 54219607 | PDFSTATSENSEMBLUCSC | 227 | 4 | 21037 | tss | 0.138967 | 0.944461 | Not queried | 6.83 | 2.86e-02 | 6.12e-02 |
|  | 13 | 20805380 | 20805679 | PDFSTATSENSEMBLUCSC | 300 | 8 | 11330 | tss | 0.152697 | 0.927213 | GJB6 (margin: GJB6) | 6.83 | 1.55e-04 | 2.71e-03 |
|  | 14 | 101405587 | 101405842 | PDFSTATSENSEMBLUCSC | 256 | 5 | 12616 | tss | 0.195033 | 0.894583 | Not queried | 6.82 | 3.17e-02 | 6.50e-02 |
|  | 2 | 130940427 | 130940706 | PDFSTATSENSEMBLUCSC | 280 | 5 | 22866 | gene;tss;island | 0.037022 | 0.505899 | Not queried | 6.82 | 1.59e-02 | 5.36e-02 |
|  | 11 | 128761146 | 128761211 | PDFSTATSENSEMBLUCSC | 66 | 6 | 7807 | tss;island | 0.023015 | 0.640883 | KCNJ5 (margin: KCNJ5; RP11-740D6.3; C11orf45) | 6.82 | 2.16e-03 | 1.45e-02 |
|  | 3 | 36422198 | 36422406 | PDFSTATSENSEMBLUCSC | 209 | 4 | 27714 | gene | 0.014268 | 0.600984 | Not queried | 6.82 | 2.86e-02 | 6.12e-02 |
|  | 5 | 158689508 | 158689727 | PDFSTATSENSEMBLUCSC | 220 | 4 | 32461 | tss;island | 0.343054 | 0.981265 | Not queried | 6.82 | 2.86e-02 | 6.12e-02 |
|  | 3 | 182970675 | 182971025 | PDFSTATSENSEMBLUCSC | 351 | 10 | 28744 | tss | 0.007019 | 0.386655 | Not queried | 6.81 | 2.32e-02 | 6.12e-02 |
|  | 11 | 121971092 | 121971247 | PDFSTATSENSEMBLUCSC | 156 | 4 | 6947 | gene;tss | 0.980709 | 0.484394 | Not queried | 6.81 | 1.14e-01 | 1.68e-01 |
|  | 1 | 27189270 | 27189440 | PDFSTATSENSEMBLUCSC | 171 | 4 | 1399 | tss;island | 0.003717 | 0.103571 | Not queried | 6.81 | 1.14e-01 | 1.68e-01 |
|  | 1 | 151689979 | 151690075 | PDFSTATSENSEMBLUCSC | 97 | 4 | 1930 | tss | 0.956831 | 0.215896 | Not queried | 6.81 | 2.86e-02 | 6.12e-02 |
|  | 18 | 44099053 | 44099340 | PDFSTATSENSEMBLUCSC | 288 | 4 | 19257 | tss | 0.032234 | 0.749684 | Not queried | 6.81 | 3.43e-01 | 3.84e-01 |
|  | 7 | 56160717 | 56161020 | PDFSTATSENSEMBLUCSC | 304 | 7 | 37609 | tss | 0.714170 | 0.015311 | PHKG1 (margin: snoU13; PHKG1; CHCHD2) | 6.81 | 5.83e-04 | 6.47e-03 |
|  | 2 | 219861012 | 219861150 | PDFSTATSENSEMBLUCSC | 139 | 5 | 25101 | island | 0.397121 | 0.005859 | Not queried | 6.81 | 6.90e-01 | 7.01e-01 |
|  | 3 | 9594151 | 9594385 | PDFSTATSENSEMBLUCSC | 235 | 4 | 27653 | gene;island | 0.965531 | 0.057029 | Not queried | 6.81 | 5.71e-02 | 1.00e-01 |
|  | 12 | 11422897 | 11423120 | PDFSTATSENSEMBLUCSC | 224 | 4 | 9566 | tss | 0.185472 | 0.893824 | Not queried | 6.81 | 1.14e-01 | 1.68e-01 |
|  | 6 | 34433676 | 34433789 | PDFSTATSENSEMBLUCSC | 114 | 7 | 34919 | tss;island | 0.698268 | 0.008613 | PACSIN1 (margin: PACSIN1; BX255972.1) | 6.80 | 5.83e-04 | 6.47e-03 |
|  | 6 | 29855110 | 29855636 | PDFSTATSENSEMBLUCSC | 527 | 16 | 35639 | island | 0.040919 | 0.661666 | HLA-H; HCG4P7 (margin: HLA-H; HLA-T; HCG4P7) | 6.80 | 2.23e-07 | 2.90e-05 |
|  | 5 | 43020161 | 43020716 | PDFSTATSENSEMBLUCSC | 556 | 7 | 32765 | island | 0.004369 | 0.423874 | CTD-2035E11.3; CTD-2201E18.3 (margin: CTD-2035E11.3; CTD-2201E18.3) | 6.80 | 5.83e-04 | 6.47e-03 |
|  | 13 | 111522755 | 111522985 | PDFSTATSENSEMBLUCSC | 231 | 5 | 11540 | tss | 0.005884 | 0.468077 | LINC00346 (margin: LINC00346; ANKRD10) | 6.80 | 7.94e-03 | 3.19e-02 |
|  | 17 | 39968362 | 39968871 | PDFSTATSENSEMBLUCSC | 510 | 14 | 17345 | tss;island | 0.509380 | 0.007789 | FKBP10; LEPREL4 (margin: FKBP10; LEPREL4) | 6.79 | 1.99e-07 | 2.63e-05 |
|  | 1 | 150208519 | 150208726 | PDFSTATSENSEMBLUCSC | 208 | 8 | 1903 | tss;island | 0.004783 | 0.316037 | RNU2-17P; ANP32E (margin: RNU2-17P; ANP32E) | 6.79 | 1.55e-04 | 2.71e-03 |
|  | 8 | 8820844 | 8821258 | PDFSTATSENSEMBLUCSC | 415 | 5 | 40059 | island | 0.620522 | 0.018267 | RP11-62H7.2; SNORA70 (margin: RP11-62H7.2; SNORA70) | 6.79 | 7.94e-03 | 3.19e-02 |
|  | 18 | 18822579 | 18823249 | PDFSTATSENSEMBLUCSC | 671 | 7 | 19066 | gene;island | 0.927544 | 0.116218 | GREB1L; RP11-699A5.2 (margin: GREB1L; RP11-699A5.2) | 6.79 | 5.83e-04 | 6.47e-03 |
|  | 20 | 25039658 | 25039812 | PDFSTATSENSEMBLUCSC | 155 | 5 | 25644 | tss;island | 0.792545 | 0.125721 | ACSS1 (margin: ACSS1; VSX1) | 6.79 | 7.94e-03 | 3.19e-02 |
|  | 4 | 571371 | 571737 | PDFSTATSENSEMBLUCSC | 367 | 4 | 30721 | island | 0.197857 | 0.945877 | Not queried | 6.78 | 2.86e-02 | 6.12e-02 |
|  | 17 | 45056626 | 45056976 | PDFSTATSENSEMBLUCSC | 351 | 8 | 17438 | tss;island | 0.495075 | 0.008322 | GOSR2; RP11-156P1.2; LRRC37A17P; RPRML (margin: GOSR2; RP11-156P1.2; MIR5089; LRRC37A17P; RPRML; RP11-156P1.3) | 6.78 | 6.22e-04 | 6.71e-03 |
|  | 1 | 18691778 | 18692019 | PDFSTATSENSEMBLUCSC | 242 | 5 | 271 | gene;island | 0.053513 | 0.974561 | IGSF21 (margin: IGSF21) | 6.78 | 7.94e-03 | 3.19e-02 |
|  | 4 | 41936852 | 41937034 | PDFSTATSENSEMBLUCSC | 183 | 4 | 30364 | tss | 0.007955 | 0.481959 | Not queried | 6.78 | 2.86e-02 | 6.12e-02 |
|  | 19 | 18717742 | 18717783 | PDFSTATSENSEMBLUCSC | 42 | 4 | 20624 | tss | 0.208630 | 0.007186 | Not queried | 6.77 | 5.71e-02 | 1.00e-01 |
|  | 17 | 32905966 | 32906622 | PDFSTATSENSEMBLUCSC | 657 | 7 | 18201 | island | 0.494155 | 0.008877 | Not queried | 6.77 | 1.28e-01 | 1.87e-01 |
|  | 12 | 108523395 | 108523472 | PDFSTATSENSEMBLUCSC | 78 | 5 | 9979 | tss | 0.783637 | 0.032147 | WSCD2 (margin: WSCD2) | 6.77 | 7.94e-03 | 3.19e-02 |
|  | 5 | 140345461 | 140346309 | PDFSTATSENSEMBLUCSC | 849 | 12 | 32359 | tss | 0.579571 | 0.017370 | Not queried | 6.77 | 2.84e-02 | 6.12e-02 |
|  | 6 | 41620951 | 41621322 | PDFSTATSENSEMBLUCSC | 372 | 6 | 34331 | gene;island | 0.862050 | 0.047630 | MDFI (margin: MDFI) | 6.77 | 2.16e-03 | 1.45e-02 |
|  | 6 | 31126599 | 31127379 | PDFSTATSENSEMBLUCSC | 781 | 21 | 34800 | tss | 0.007658 | 0.682361 | TCF19; CCHCR1 (margin: TCF19; CCHCR1; POU5F1) | 6.77 | 4.34e-08 | 9.34e-06 |
|  | 12 | 5541220 | 5541403 | PDFSTATSENSEMBLUCSC | 184 | 6 | 10182 | island | 0.077432 | 0.870977 | NTF3 (margin: NTF3) | 6.76 | 2.16e-03 | 1.45e-02 |
|  | 10 | 118765099 | 118765335 | PDFSTATSENSEMBLUCSC | 237 | 8 | 5387 | tss;island | 0.584996 | 0.014700 | KIAA1598 (margin: KIAA1598) | 6.76 | 1.55e-04 | 2.71e-03 |
|  | 17 | 40346279 | 40346486 | PDFSTATSENSEMBLUCSC | 208 | 5 | 16578 | gene | 0.069590 | 0.772510 | GHDC (margin: HCRT; GHDC; STAT5B) | 6.76 | 7.94e-03 | 3.19e-02 |
|  | 20 | 32307885 | 32308529 | PDFSTATSENSEMBLUCSC | 645 | 11 | 26102 | island | 0.513200 | 0.006186 | PXMP4 (margin: ZNF341; PXMP4; RP4-553F4.2) | 6.76 | 3.18e-03 | 2.01e-02 |
|  | 20 | 32308170 | 32308529 | PDFSTATSENSEMBLUCSC | 360 | 7 | 25685 | tss | 0.442620 | 0.006008 | Not queried | 6.76 | 7.28e-02 | 1.23e-01 |
|  | 7 | 130126153 | 130126637 | PDFSTATSENSEMBLUCSC | 485 | 6 | 37223 | gene | 0.412190 | 0.005814 | MEST; RP11-2E11.5 (margin: MEST; MIR335; RP11-2E11.5; hsa-mir-335) | 6.76 | 8.66e-03 | 3.36e-02 |
|  | 17 | 79107083 | 79107259 | PDFSTATSENSEMBLUCSC | 177 | 4 | 16895 | gene;island | 0.194345 | 0.938043 | Not queried | 6.76 | 2.86e-02 | 6.12e-02 |
|  | 16 | 8806966 | 8807043 | PDFSTATSENSEMBLUCSC | 78 | 5 | 14653 | gene | 0.075806 | 0.896051 | ABAT (margin: ABAT; RNU7-63P) | 6.76 | 7.94e-03 | 3.19e-02 |
|  | 3 | 186857284 | 186857593 | PDFSTATSENSEMBLUCSC | 310 | 7 | 28778 | tss;island | 0.004173 | 0.401838 | RPL39L (margin: RPL39L) | 6.75 | 5.83e-04 | 6.47e-03 |
|  | 1 | 12226913 | 12227131 | PDFSTATSENSEMBLUCSC | 219 | 5 | 2699 | island | 0.306616 | 0.970181 | TNFRSF1B (margin: TNFRSF1B) | 6.75 | 7.94e-03 | 3.19e-02 |
|  | 11 | 122852412 | 122852698 | PDFSTATSENSEMBLUCSC | 287 | 8 | 7775 | tss | 0.917900 | 0.161289 | BSX (margin: BSX) | 6.75 | 1.55e-04 | 2.71e-03 |
|  | 4 | 186435443 | 186435667 | PDFSTATSENSEMBLUCSC | 225 | 6 | 30210 | gene | 0.976491 | 0.284488 | PDLIM3 (margin: PDLIM3) | 6.75 | 2.16e-03 | 1.45e-02 |
|  | 19 | 18118666 | 18118880 | PDFSTATSENSEMBLUCSC | 215 | 4 | 20608 | tss | 0.213314 | 0.956814 | Not queried | 6.75 | 2.86e-02 | 6.12e-02 |
|  | 1 | 211306986 | 211307408 | PDFSTATSENSEMBLUCSC | 423 | 5 | 993 | gene;island | 0.426752 | 0.006266 | KCNH1; KCNH1-IT1 (margin: KCNH1; KCNH1-IT1) | 6.75 | 7.94e-03 | 3.19e-02 |
|  | 1 | 210110998 | 210111162 | PDFSTATSENSEMBLUCSC | 165 | 5 | 2235 | tss;island | 0.336794 | 0.009198 | SYT14 (margin: SYT14) | 6.74 | 7.94e-03 | 3.19e-02 |
|  | 1 | 39874802 | 39875069 | PDFSTATSENSEMBLUCSC | 268 | 12 | 1521 | tss | 0.701414 | 0.019579 | MACF1; KIAA0754 (margin: MACF1; KIAA0754) | 6.74 | 2.22e-05 | 9.05e-04 |
|  | 7 | 93519855 | 93520566 | PDFSTATSENSEMBLUCSC | 712 | 19 | 38669 | island | 0.833285 | 0.039170 | GNGT1; AC002076.10; TFPI2 (margin: GNGT1; AC002076.10; TFPI2) | 6.74 | 2.26e-10 | 1.16e-07 |
|  | 6 | 3848634 | 3849818 | PDFSTATSENSEMBLUCSC | 1185 | 31 | 35389 | island | 0.009897 | 0.496856 | RP11-420L9.4; FAM50B (margin: RP11-420L9.4; FAM50B) | 6.74 | 2.03e-11 | 1.98e-08 |
|  | 14 | 75535757 | 75536217 | PDFSTATSENSEMBLUCSC | 461 | 6 | 12515 | tss | 0.010809 | 0.558942 | ZC2HC1C; ACYP1 (margin: ZC2HC1C; ACYP1) | 6.74 | 2.16e-03 | 1.45e-02 |
|  | 17 | 9929698 | 9929867 | PDFSTATSENSEMBLUCSC | 170 | 4 | 17133 | tss | 0.012237 | 0.562388 | Not queried | 6.74 | 2.86e-02 | 6.12e-02 |
|  | 17 | 33787402 | 33787739 | PDFSTATSENSEMBLUCSC | 338 | 4 | 18215 | island | 0.153017 | 0.949397 | Not queried | 6.73 | 2.86e-02 | 6.12e-02 |
|  | 6 | 27782909 | 27783331 | PDFSTATSENSEMBLUCSC | 423 | 5 | 34721 | tss;island | 0.146570 | 0.955043 | Not queried | 6.73 | 1.59e-02 | 5.36e-02 |
|  | 13 | 113104883 | 113105341 | PDFSTATSENSEMBLUCSC | 459 | 5 | 11920 | island | 0.472716 | 0.898778 | Not queried | 6.73 | 3.17e-02 | 6.50e-02 |
|  | 10 | 25464008 | 25464259 | PDFSTATSENSEMBLUCSC | 252 | 8 | 4991 | tss | 0.728679 | 0.026978 | GPR158; GPR158-AS1 (margin: GPR158; GPR158-AS1) | 6.73 | 1.55e-04 | 2.71e-03 |
|  | 1 | 92949447 | 92950836 | PDFSTATSENSEMBLUCSC | 1390 | 29 | 1751 | tss | 0.243322 | 0.971923 | GFI1 (margin: GFI1) | 6.73 | 1.05e-08 | 3.21e-06 |
|  | 10 | 135340445 | 135341025 | PDFSTATSENSEMBLUCSC | 581 | 9 | 6367 | island | 0.169381 | 0.892464 | CYP2E1; SPRN; AL161645.2 (margin: RP11-108K14.4; CYP2E1; SPRN; AL161645.2) | 6.73 | 4.11e-05 | 1.17e-03 |
|  | 19 | 50031246 | 50031560 | PDFSTATSENSEMBLUCSC | 315 | 4 | 20157 | gene;island | 0.363882 | 0.979871 | Not queried | 6.73 | 2.86e-02 | 6.12e-02 |
|  | 8 | 1764878 | 1765477 | PDFSTATSENSEMBLUCSC | 600 | 12 | 40038 | island | 0.377028 | 0.985968 | MIR596 (margin: MIR596; ARHGEF10) | 6.72 | 7.40e-07 | 7.21e-05 |
|  | 8 | 1764878 | 1765387 | PDFSTATSENSEMBLUCSC | 510 | 10 | 39535 | tss | 0.377028 | 0.985968 | MIR596 (margin: MIR596; ARHGEF10) | 6.72 | 1.08e-05 | 4.82e-04 |
|  | 15 | 91497863 | 91498080 | PDFSTATSENSEMBLUCSC | 218 | 6 | 13908 | tss | 0.783728 | 0.037068 | UNC45A; RCCD1; AC068831.6 (margin: UNC45A; RCCD1; PRC1-AS1; AC068831.6; PRC1) | 6.72 | 2.16e-03 | 1.45e-02 |
|  | 12 | 34174976 | 34175567 | PDFSTATSENSEMBLUCSC | 592 | 7 | 10320 | island | 0.002575 | 0.104530 | Not queried | 6.72 | 7.28e-02 | 1.23e-01 |
|  | 4 | 142558042 | 142558451 | PDFSTATSENSEMBLUCSC | 410 | 4 | 30140 | gene;island | 0.010714 | 0.428916 | Not queried | 6.72 | 2.86e-02 | 6.12e-02 |
|  | 19 | 3700613 | 3700773 | PDFSTATSENSEMBLUCSC | 161 | 4 | 20350 | tss;island | 0.004564 | 0.003908 | Not queried | 6.72 | 6.86e-01 | 6.99e-01 |
|  | 1 | 244213358 | 244213618 | PDFSTATSENSEMBLUCSC | 261 | 5 | 2354 | tss;island | 0.941729 | 0.170774 | ZBTB18 (margin: RP11-278H7.1; ZBTB18) | 6.72 | 7.94e-03 | 3.19e-02 |
|  | 8 | 144814911 | 144815275 | PDFSTATSENSEMBLUCSC | 365 | 4 | 39490 | gene;island | 0.008237 | 0.149824 | Not queried | 6.72 | 2.86e-02 | 6.12e-02 |
|  | 15 | 68260324 | 68260709 | PDFSTATSENSEMBLUCSC | 386 | 5 | 14255 | island | 0.244956 | 0.946960 | Not queried | 6.72 | 3.17e-02 | 6.50e-02 |
|  | 16 | 56623312 | 56623549 | PDFSTATSENSEMBLUCSC | 238 | 4 | 14762 | gene | 0.363462 | 0.983763 | Not queried | 6.71 | 2.86e-02 | 6.12e-02 |
|  | 11 | 73020351 | 73020729 | PDFSTATSENSEMBLUCSC | 379 | 5 | 6821 | gene;island | 0.011069 | 0.614347 | Not queried | 6.70 | 9.52e-02 | 1.53e-01 |
|  | 1 | 156675322 | 156675562 | PDFSTATSENSEMBLUCSC | 241 | 7 | 3863 | island | 0.650819 | 0.020391 | CRABP2 (margin: CRABP2; RP11-66D17.5) | 6.70 | 5.83e-04 | 6.47e-03 |
|  | 3 | 53700141 | 53700276 | PDFSTATSENSEMBLUCSC | 136 | 4 | 27830 | gene | 0.387679 | 0.975015 | Not queried | 6.70 | 2.86e-02 | 6.12e-02 |
|  | 19 | 37329090 | 37329859 | PDFSTATSENSEMBLUCSC | 770 | 15 | 21947 | island | 0.593467 | 0.008594 | ZNF790 (margin: CTD-2162K18.5; ZNF345; ZNF790) | 6.70 | 1.29e-08 | 3.49e-06 |
|  | 20 | 44746392 | 44747006 | PDFSTATSENSEMBLUCSC | 615 | 11 | 26215 | island | 0.129910 | 0.934471 | CD40 (margin: CD40) | 6.70 | 2.84e-06 | 1.96e-04 |
|  | 20 | 44746392 | 44746902 | PDFSTATSENSEMBLUCSC | 511 | 9 | 25789 | tss | 0.129910 | 0.934471 | CD40 (margin: CD40) | 6.70 | 4.11e-05 | 1.17e-03 |
|  | 10 | 95462915 | 95463124 | PDFSTATSENSEMBLUCSC | 210 | 4 | 5241 | tss;island | 0.628638 | 0.010132 | Not queried | 6.70 | 1.14e-01 | 1.68e-01 |
|  | 19 | 58661689 | 58661962 | PDFSTATSENSEMBLUCSC | 274 | 4 | 20257 | gene;island | 0.304466 | 0.003800 | Not queried | 6.69 | 2.86e-02 | 6.12e-02 |
|  | 17 | 46671131 | 46671635 | PDFSTATSENSEMBLUCSC | 505 | 9 | 17464 | tss | 0.493760 | 0.013263 | HOXB-AS3; HOXB3; HOXB5; HOXB6 (margin: HOXB-AS3; HOXB3; MIR10A; HOXB5; HOXB6) | 6.69 | 1.23e-03 | 1.18e-02 |
|  | 17 | 19437691 | 19437929 | PDFSTATSENSEMBLUCSC | 239 | 4 | 16481 | gene;island | 0.032779 | 0.636870 | Not queried | 6.69 | 1.14e-01 | 1.68e-01 |
|  | 10 | 57390536 | 57391055 | PDFSTATSENSEMBLUCSC | 520 | 4 | 5755 | island | 0.844519 | 0.103729 | Not queried | 6.68 | 2.86e-02 | 6.12e-02 |
|  | 16 | 21170927 | 21171239 | PDFSTATSENSEMBLUCSC | 313 | 5 | 14688 | gene;tss;island | 0.136289 | 0.002012 | TMEM159; DNAH3 (margin: TMEM159; DNAH3) | 6.68 | 7.94e-03 | 3.19e-02 |
|  | 13 | 113759771 | 113760286 | PDFSTATSENSEMBLUCSC | 516 | 6 | 11956 | island | 0.559401 | 0.019443 | F7 (margin: MCF2L; F7; AL137002.1) | 6.68 | 2.16e-03 | 1.45e-02 |
|  | 15 | 81666512 | 81666847 | PDFSTATSENSEMBLUCSC | 336 | 4 | 13854 | tss | 0.262253 | 0.951225 | Not queried | 6.68 | 5.71e-02 | 1.00e-01 |
|  | 16 | 31159558 | 31159920 | PDFSTATSENSEMBLUCSC | 363 | 5 | 14733 | gene;island | 0.783503 | 0.123001 | PRSS36 (margin: PRSS36) | 6.68 | 7.94e-03 | 3.19e-02 |
|  | 13 | 36050844 | 36051073 | PDFSTATSENSEMBLUCSC | 230 | 7 | 11390 | tss | 0.074358 | 0.881816 | NBEA; MAB21L1 (margin: NBEA; MAB21L1) | 6.68 | 5.83e-04 | 6.47e-03 |
|  | 10 | 88295210 | 88295591 | PDFSTATSENSEMBLUCSC | 382 | 4 | 5905 | island | 0.013965 | 0.545100 | Not queried | 6.67 | 2.86e-02 | 6.12e-02 |
|  | 6 | 41472382 | 41472657 | PDFSTATSENSEMBLUCSC | 276 | 4 | 36123 | island | 0.241212 | 0.809969 | Not queried | 6.67 | 2.86e-02 | 6.12e-02 |
|  | 17 | 43972806 | 43973522 | PDFSTATSENSEMBLUCSC | 717 | 8 | 16640 | gene;tss;island | 0.646577 | 0.016691 | Not queried | 6.67 | 1.05e-01 | 1.66e-01 |
|  | 5 | 94620929 | 94621055 | PDFSTATSENSEMBLUCSC | 127 | 4 | 32182 | tss;island | 0.612725 | 0.025824 | Not queried | 6.67 | 2.86e-02 | 6.12e-02 |
|  | 7 | 100769903 | 100770286 | PDFSTATSENSEMBLUCSC | 384 | 5 | 37798 | tss | 0.004803 | 0.401164 | SERPINE1 (margin: SERPINE1) | 6.67 | 7.94e-03 | 3.19e-02 |
|  | 1 | 10056837 | 10057168 | PDFSTATSENSEMBLUCSC | 332 | 7 | 1233 | tss | 0.361523 | 0.982024 | RBP7 (margin: NMNAT1; RBP7) | 6.67 | 5.83e-04 | 6.47e-03 |
|  | 21 | 45232531 | 45232601 | PDFSTATSENSEMBLUCSC | 71 | 4 | 26579 | tss | 0.015615 | 0.659336 | Not queried | 6.66 | 2.86e-02 | 6.12e-02 |
|  | 3 | 142838682 | 142839022 | PDFSTATSENSEMBLUCSC | 341 | 4 | 27979 | gene;island | 0.332733 | 0.982562 | Not queried | 6.66 | 2.86e-02 | 6.12e-02 |
|  | 8 | 110986685 | 110987019 | PDFSTATSENSEMBLUCSC | 335 | 5 | 40542 | island | 0.522314 | 0.027212 | RP11-696P8.2; KCNV1 (margin: RP11-696P8.2; KCNV1) | 6.66 | 7.94e-03 | 3.19e-02 |
|  | 6 | 27463112 | 27463347 | PDFSTATSENSEMBLUCSC | 236 | 4 | 35558 | island | 0.007432 | 0.425154 | Not queried | 6.66 | 2.86e-02 | 6.12e-02 |
|  | 2 | 47499521 | 47499812 | PDFSTATSENSEMBLUCSC | 292 | 4 | 24281 | island | 0.593539 | 0.020362 | Not queried | 6.66 | 2.86e-02 | 6.12e-02 |
|  | 14 | 92980026 | 92980103 | PDFSTATSENSEMBLUCSC | 78 | 4 | 12567 | tss | 0.012388 | 0.515492 | Not queried | 6.66 | 2.86e-02 | 6.12e-02 |
|  | 17 | 6899085 | 6899888 | PDFSTATSENSEMBLUCSC | 804 | 14 | 17913 | island | 0.305123 | 0.979368 | ALOX12; AC027763.2; RP11-589P10.7; RP11-589P10.5 (margin: ALOX12; AC027763.2; RP11-589P10.7; RP11-589P10.5) | 6.66 | 4.99e-08 | 9.34e-06 |
|  | 1 | 10854205 | 10854560 | PDFSTATSENSEMBLUCSC | 356 | 5 | 217 | gene;island | 0.350352 | 0.006745 | CASZ1 (margin: CASZ1) | 6.66 | 7.94e-03 | 3.19e-02 |
|  | 4 | 48484997 | 48485328 | PDFSTATSENSEMBLUCSC | 332 | 8 | 30382 | tss;island | 0.206271 | 0.977545 | SLC10A4 (margin: SLC10A4; ZAR1) | 6.66 | 2.95e-03 | 1.88e-02 |
|  | 18 | 35145983 | 35146770 | PDFSTATSENSEMBLUCSC | 788 | 21 | 19477 | island | 0.717615 | 0.008448 | CELF4 (margin: CELF4) | 6.65 | 3.40e-09 | 1.10e-06 |
|  | 2 | 74730266 | 74730948 | PDFSTATSENSEMBLUCSC | 683 | 11 | 22745 | gene;island | 0.020739 | 0.557663 | RP11-523H20.3; LBX2-AS1; LBX2; PCGF1 (margin: TTC31; RP11-523H20.3; LBX2-AS1; TLX2; LBX2; AC005041.17; PCGF1) | 6.65 | 2.84e-06 | 1.96e-04 |
|  | 12 | 101603835 | 101604258 | PDFSTATSENSEMBLUCSC | 424 | 8 | 10717 | island | 0.701681 | 0.019187 | SLC5A8 (margin: SLC5A8; RNU6-768P) | 6.65 | 1.55e-04 | 2.71e-03 |
|  | 19 | 57701880 | 57703364 | PDFSTATSENSEMBLUCSC | 1485 | 25 | 22460 | island | 0.033015 | 0.854410 | Not queried | 6.65 | 5.94e-02 | 1.04e-01 |
|  | 1 | 109939800 | 109940036 | PDFSTATSENSEMBLUCSC | 237 | 4 | 667 | gene;island | 0.571609 | 0.018185 | Not queried | 6.65 | 5.71e-02 | 1.00e-01 |
|  | 8 | 49647579 | 49648363 | PDFSTATSENSEMBLUCSC | 785 | 11 | 40284 | island | 0.139527 | 0.971559 | EFCAB1 (margin: EFCAB1) | 6.65 | 2.75e-04 | 4.46e-03 |
|  | 1 | 156675388 | 156675562 | PDFSTATSENSEMBLUCSC | 175 | 6 | 2020 | tss | 0.632415 | 0.021955 | CRABP2 (margin: CRABP2; RP11-66D17.5) | 6.65 | 2.16e-03 | 1.45e-02 |
|  | 1 | 1356565 | 1356902 | PDFSTATSENSEMBLUCSC | 338 | 4 | 2437 | island | 0.123512 | 0.955458 | Not queried | 6.65 | 2.86e-02 | 6.12e-02 |
|  | 19 | 11877661 | 11878036 | PDFSTATSENSEMBLUCSC | 376 | 7 | 21583 | island | 0.012621 | 0.552066 | ZNF441 (margin: ZNF441; CTC-499B15.1) | 6.64 | 5.83e-04 | 6.47e-03 |
|  | 15 | 41805868 | 41806234 | PDFSTATSENSEMBLUCSC | 367 | 5 | 14063 | island | 0.178321 | 0.937749 | LTK (margin: ITPKA; LTK; RPAP1) | 6.64 | 7.94e-03 | 3.19e-02 |
|  | 19 | 37406932 | 37407152 | PDFSTATSENSEMBLUCSC | 221 | 6 | 20005 | gene | 0.482800 | 0.008364 | ZNF568; ZNF829 (margin: ZNF345; ZNF568; ZNF829) | 6.64 | 2.16e-03 | 1.45e-02 |
|  | 18 | 12287173 | 12287561 | PDFSTATSENSEMBLUCSC | 389 | 4 | 19402 | island | 0.171023 | 0.925650 | Not queried | 6.64 | 2.86e-02 | 6.12e-02 |
|  | 19 | 19280969 | 19281559 | PDFSTATSENSEMBLUCSC | 591 | 11 | 19947 | gene;island | 0.518657 | 0.012012 | MEF2BNB-MEF2B; MEF2B (margin: MEF2BNB-MEF2B; MEF2B; MEF2BNB) | 6.64 | 3.94e-04 | 5.86e-03 |
|  | 15 | 63674108 | 63674276 | PDFSTATSENSEMBLUCSC | 169 | 6 | 13722 | tss;island | 0.488175 | 0.019645 | CA12 (margin: RP11-321G12.1; CA12) | 6.64 | 2.16e-03 | 1.45e-02 |
|  | 1 | 13840381 | 13840712 | PDFSTATSENSEMBLUCSC | 332 | 7 | 2703 | island | 0.424862 | 0.968490 | RP4-597A16.2; LRRC38 (margin: RP4-597A16.2; LRRC38) | 6.63 | 5.83e-04 | 6.47e-03 |
|  | 1 | 1141617 | 1141991 | PDFSTATSENSEMBLUCSC | 375 | 5 | 20 | gene | 0.667637 | 0.018728 | TNFRSF18 (margin: TTLL10; TNFRSF18; TNFRSF4; SDF4) | 6.63 | 7.94e-03 | 3.19e-02 |
|  | 10 | 126106425 | 126106614 | PDFSTATSENSEMBLUCSC | 190 | 4 | 4805 | gene;island | 0.055023 | 0.795134 | Not queried | 6.63 | 2.86e-02 | 6.12e-02 |
|  | 6 | 52009019 | 52009126 | PDFSTATSENSEMBLUCSC | 108 | 4 | 35029 | tss | 0.140698 | 0.886007 | Not queried | 6.63 | 2.86e-02 | 6.12e-02 |
|  | 2 | 74229594 | 74229995 | PDFSTATSENSEMBLUCSC | 402 | 4 | 24423 | island | 0.599781 | 0.017276 | Not queried | 6.63 | 2.86e-02 | 6.12e-02 |
|  | 14 | 36003074 | 36003211 | PDFSTATSENSEMBLUCSC | 138 | 6 | 12354 | tss | 0.523529 | 0.013804 | INSM2 (margin: INSM2; RALGAPA1) | 6.63 | 2.16e-03 | 1.45e-02 |
|  | 12 | 96336486 | 96336545 | PDFSTATSENSEMBLUCSC | 60 | 4 | 9928 | tss;island | 0.004859 | 0.303172 | Not queried | 6.63 | 2.86e-02 | 6.12e-02 |
|  | 18 | 43652172 | 43652594 | PDFSTATSENSEMBLUCSC | 423 | 10 | 19484 | island | 0.744952 | 0.047956 | PSTPIP2 (margin: PSTPIP2; Y\_RNA; ATP5A1) | 6.62 | 1.08e-05 | 4.82e-04 |
|  | 6 | 151646540 | 151646651 | PDFSTATSENSEMBLUCSC | 112 | 5 | 35299 | tss | 0.515729 | 0.012495 | AKAP12 (margin: AKAP12; RN7SKP268) | 6.62 | 7.94e-03 | 3.19e-02 |
|  | 18 | 43652255 | 43652594 | PDFSTATSENSEMBLUCSC | 340 | 8 | 19251 | tss | 0.897404 | 0.079279 | PSTPIP2 (margin: PSTPIP2; Y\_RNA; ATP5A1) | 6.62 | 1.55e-04 | 2.71e-03 |
|  | 17 | 14204310 | 14204485 | PDFSTATSENSEMBLUCSC | 176 | 5 | 17146 | tss | 0.482895 | 0.005378 | HS3ST3B1 (margin: HS3ST3B1; RP11-214O1.2; RP11-214O1.1) | 6.62 | 7.94e-03 | 3.19e-02 |
|  | 16 | 1146194 | 1146545 | PDFSTATSENSEMBLUCSC | 352 | 5 | 15516 | island | 0.280982 | 0.982434 | C1QTNF8 (margin: C1QTNF8; LA16c-349E11.1) | 6.62 | 7.94e-03 | 3.19e-02 |
|  | 6 | 55444061 | 55444488 | PDFSTATSENSEMBLUCSC | 428 | 7 | 35043 | tss | 0.680507 | 0.052985 | HMGCLL1 (margin: HMGCLL1) | 6.62 | 1.17e-03 | 1.13e-02 |
|  | 7 | 12443478 | 12444290 | PDFSTATSENSEMBLUCSC | 813 | 11 | 38266 | island | 0.705256 | 0.066293 | VWDE (margin: VWDE) | 6.62 | 4.10e-03 | 2.48e-02 |
|  | 4 | 6114498 | 6114699 | PDFSTATSENSEMBLUCSC | 202 | 4 | 29926 | gene;island | 0.211807 | 0.915129 | Not queried | 6.62 | 2.86e-02 | 6.12e-02 |
|  | 19 | 39522418 | 39523626 | PDFSTATSENSEMBLUCSC | 1209 | 11 | 21986 | island | 0.494865 | 0.006166 | CTB-189B5.3; FBXO27 (margin: CTB-189B5.3; FBXO27) | 6.62 | 3.94e-04 | 5.86e-03 |
|  | 1 | 47915553 | 47915951 | PDFSTATSENSEMBLUCSC | 399 | 6 | 3241 | island | 0.903153 | 0.206862 | Not queried | 6.62 | 6.49e-02 | 1.11e-01 |
|  | 19 | 6502101 | 6502304 | PDFSTATSENSEMBLUCSC | 204 | 4 | 19765 | gene | 0.720100 | 0.035078 | Not queried | 6.62 | 2.86e-02 | 6.12e-02 |
|  | 6 | 36355488 | 36355825 | PDFSTATSENSEMBLUCSC | 338 | 6 | 34936 | tss | 0.114272 | 0.965613 | RP1-50J22.4; ETV7 (margin: RP1-50J22.4; ETV7; PXT1) | 6.61 | 8.66e-03 | 3.36e-02 |
|  | 7 | 37488162 | 37489005 | PDFSTATSENSEMBLUCSC | 844 | 15 | 38416 | island | 0.858881 | 0.052875 | ELMO1 (margin: ELMO1) | 6.61 | 1.55e-07 | 2.60e-05 |
|  | 7 | 37488564 | 37489005 | PDFSTATSENSEMBLUCSC | 442 | 11 | 37541 | tss | 0.843908 | 0.052875 | ELMO1 (margin: ELMO1) | 6.61 | 3.40e-05 | 1.17e-03 |
|  | 2 | 136875144 | 136875349 | PDFSTATSENSEMBLUCSC | 206 | 5 | 22886 | gene;island | 0.528429 | 0.020206 | CXCR4 (margin: CXCR4) | 6.60 | 7.94e-03 | 3.19e-02 |
|  | 15 | 35087506 | 35087773 | PDFSTATSENSEMBLUCSC | 268 | 5 | 13281 | gene | 0.132278 | 0.949119 | RP11-814P5.1; ACTC1 (margin: RP11-814P5.1; ACTC1) | 6.60 | 7.94e-03 | 3.19e-02 |
|  | 16 | 58548873 | 58549320 | PDFSTATSENSEMBLUCSC | 448 | 8 | 15275 | tss;island | 0.327962 | 0.013806 | NDRG4; SETD6 (margin: NDRG4; SETD6; CNOT1) | 6.60 | 1.09e-03 | 1.10e-02 |
|  | 1 | 92949337 | 92950836 | PDFSTATSENSEMBLUCSC | 1500 | 31 | 635 | gene;island | 0.245164 | 0.974474 | GFI1 (margin: GFI1) | 6.59 | 2.50e-09 | 8.71e-07 |
|  | 19 | 54023732 | 54024435 | PDFSTATSENSEMBLUCSC | 704 | 12 | 22380 | island | 0.727632 | 0.005698 | ZNF331 (margin: CTD-2224J9.8; ZNF331) | 6.59 | 1.11e-03 | 1.12e-02 |
|  | 19 | 54023732 | 54024110 | PDFSTATSENSEMBLUCSC | 379 | 8 | 21030 | tss | 0.861365 | 0.024017 | ZNF331 (margin: CTD-2224J9.8; ZNF331) | 6.59 | 1.04e-02 | 3.96e-02 |
|  | 6 | 29894619 | 29895260 | PDFSTATSENSEMBLUCSC | 642 | 25 | 35642 | island | 0.080932 | 0.920289 | HLA-K; HCG4B (margin: HLA-K; HLA-U; HCG4B) | 6.59 | 1.56e-09 | 6.31e-07 |
|  | 11 | 6440279 | 6440482 | PDFSTATSENSEMBLUCSC | 204 | 6 | 6490 | gene;island | 0.581947 | 0.017068 | APBB1 (margin: APBB1; HPX) | 6.59 | 2.16e-03 | 1.45e-02 |
|  | 7 | 3341524 | 3342048 | PDFSTATSENSEMBLUCSC | 525 | 6 | 36823 | gene;island | 0.547954 | 0.034528 | SDK1; AC073316.1 (margin: SDK1; AC073316.1) | 6.59 | 2.16e-03 | 1.45e-02 |
|  | 6 | 146136083 | 146136749 | PDFSTATSENSEMBLUCSC | 667 | 10 | 35273 | tss;island | 0.011085 | 0.503427 | RP11-545I5.3; FBXO30 (margin: RP11-545I5.3; FBXO30) | 6.59 | 2.09e-03 | 1.45e-02 |
|  | 10 | 17270087 | 17270250 | PDFSTATSENSEMBLUCSC | 164 | 5 | 4969 | tss | 0.323860 | 0.967403 | VIM; VIM-AS1 (margin: VIM; VIM-AS1; RP11-124N14.3) | 6.58 | 7.94e-03 | 3.19e-02 |
|  | 11 | 128563483 | 128563716 | PDFSTATSENSEMBLUCSC | 234 | 6 | 7803 | tss | 0.281283 | 0.836003 | Not queried | 6.58 | 2.60e-02 | 6.12e-02 |
|  | 6 | 88875236 | 88875844 | PDFSTATSENSEMBLUCSC | 609 | 8 | 36315 | island | 0.506668 | 0.026711 | CNR1 (margin: CNR1) | 6.58 | 1.55e-04 | 2.71e-03 |
|  | 8 | 52722023 | 52722237 | PDFSTATSENSEMBLUCSC | 215 | 5 | 39695 | tss | 0.906344 | 0.086865 | Not queried | 6.58 | 1.59e-02 | 5.36e-02 |
|  | 18 | 35146001 | 35146770 | PDFSTATSENSEMBLUCSC | 770 | 20 | 19246 | tss | 0.730509 | 0.007968 | CELF4 (margin: CELF4) | 6.58 | 1.33e-08 | 3.50e-06 |
|  | 15 | 79724739 | 79724802 | PDFSTATSENSEMBLUCSC | 64 | 4 | 13845 | tss;island | 0.575332 | 0.028818 | Not queried | 6.58 | 2.86e-02 | 6.12e-02 |
|  | 5 | 1008828 | 1009091 | PDFSTATSENSEMBLUCSC | 264 | 4 | 31965 | tss | 0.338653 | 0.011599 | Not queried | 6.58 | 2.86e-02 | 6.12e-02 |
|  | 17 | 32690255 | 32690732 | PDFSTATSENSEMBLUCSC | 478 | 5 | 17259 | tss | 0.012368 | 0.543769 | Not queried | 6.57 | 9.52e-02 | 1.53e-01 |
|  | 19 | 2095364 | 2095643 | PDFSTATSENSEMBLUCSC | 280 | 4 | 19679 | gene;island | 0.423651 | 0.975457 | Not queried | 6.57 | 2.86e-02 | 6.12e-02 |
|  | 17 | 75471193 | 75471491 | PDFSTATSENSEMBLUCSC | 299 | 6 | 16811 | gene | 0.347778 | 0.979823 | SEPT9 (margin: SEPT9; RP11-75C10.7; RP11-75C10.9) | 6.57 | 2.16e-03 | 1.45e-02 |
|  | 5 | 91988 | 92385 | PDFSTATSENSEMBLUCSC | 398 | 5 | 32571 | island | 0.107656 | 0.918667 | CTD-2231H16.1 (margin: CTD-2231H16.1) | 6.57 | 7.94e-03 | 3.19e-02 |
|  | 7 | 50800426 | 50800620 | PDFSTATSENSEMBLUCSC | 195 | 4 | 37013 | gene;tss | 0.211052 | 0.956958 | Not queried | 6.57 | 2.86e-02 | 6.12e-02 |
|  | 2 | 8818142 | 8818497 | PDFSTATSENSEMBLUCSC | 356 | 5 | 24048 | island | 0.286482 | 0.005503 | Not queried | 6.57 | 1.59e-02 | 5.36e-02 |
|  | 14 | 79744991 | 79745592 | PDFSTATSENSEMBLUCSC | 602 | 7 | 12159 | gene;tss;island | 0.602879 | 0.012765 | NRXN3 (margin: NRXN3) | 6.57 | 4.08e-03 | 2.47e-02 |
|  | 12 | 54763081 | 54763433 | PDFSTATSENSEMBLUCSC | 353 | 4 | 9165 | gene;island | 0.923901 | 0.182527 | Not queried | 6.56 | 2.86e-02 | 6.12e-02 |
|  | 10 | 98945775 | 98946238 | PDFSTATSENSEMBLUCSC | 464 | 10 | 5257 | tss | 0.854337 | 0.024803 | Not queried | 6.56 | 3.55e-02 | 7.24e-02 |
|  | 15 | 79576060 | 79576298 | PDFSTATSENSEMBLUCSC | 239 | 4 | 13448 | gene;island | 0.545524 | 0.013747 | Not queried | 6.56 | 5.71e-02 | 1.00e-01 |
|  | 12 | 101604027 | 101604258 | PDFSTATSENSEMBLUCSC | 232 | 4 | 9943 | tss | 0.706108 | 0.025149 | Not queried | 6.55 | 2.86e-02 | 6.12e-02 |
|  | 5 | 128301230 | 128301488 | PDFSTATSENSEMBLUCSC | 259 | 4 | 31740 | gene | 0.538457 | 0.044875 | Not queried | 6.55 | 5.71e-02 | 1.00e-01 |
|  | 2 | 228582758 | 228582820 | PDFSTATSENSEMBLUCSC | 63 | 5 | 23931 | tss | 0.156773 | 0.001916 | SLC19A3 (margin: SLC19A3) | 6.55 | 7.94e-03 | 3.19e-02 |
|  | 20 | 45523098 | 45523187 | PDFSTATSENSEMBLUCSC | 90 | 4 | 25798 | tss | 0.287835 | 0.004803 | Not queried | 6.55 | 2.86e-02 | 6.12e-02 |
|  | 1 | 65775467 | 65775772 | PDFSTATSENSEMBLUCSC | 306 | 5 | 570 | gene;island | 0.619651 | 0.010243 | DNAJC6 (margin: DNAJC6; RP5-1044H5.1; RP5-1044H5.2) | 6.55 | 7.94e-03 | 3.19e-02 |
|  | 10 | 20105179 | 20105641 | PDFSTATSENSEMBLUCSC | 463 | 5 | 5588 | island | 0.668752 | 0.006702 | Not queried | 6.55 | 3.17e-02 | 6.50e-02 |
|  | 3 | 21792248 | 21792684 | PDFSTATSENSEMBLUCSC | 437 | 4 | 27700 | gene | 0.955596 | 0.222052 | Not queried | 6.54 | 1.14e-01 | 1.68e-01 |
|  | 1 | 203598573 | 203599089 | PDFSTATSENSEMBLUCSC | 517 | 6 | 953 | gene;island | 0.262317 | 0.981518 | ATP2B4 (margin: ATP2B4) | 6.54 | 2.16e-03 | 1.45e-02 |
|  | 13 | 113776873 | 113777109 | PDFSTATSENSEMBLUCSC | 237 | 8 | 11548 | tss | 0.115509 | 0.933082 | F7; F10 (margin: F7; F10; KARSP2; F10-AS1) | 6.54 | 1.55e-04 | 2.71e-03 |
|  | 2 | 31456964 | 31457061 | PDFSTATSENSEMBLUCSC | 98 | 5 | 22638 | gene | 0.090967 | 0.004553 | EHD3; CAPN14 (margin: RNA5SP90; EHD3; CAPN14) | 6.53 | 7.94e-03 | 3.19e-02 |
|  | 4 | 81124484 | 81124767 | PDFSTATSENSEMBLUCSC | 284 | 4 | 30035 | gene;island | 0.335068 | 0.977972 | Not queried | 6.53 | 2.86e-02 | 6.12e-02 |
|  | 5 | 172662315 | 172662463 | PDFSTATSENSEMBLUCSC | 149 | 4 | 32502 | tss | 0.096154 | 0.893537 | Not queried | 6.53 | 2.86e-02 | 6.12e-02 |
|  | 3 | 50283852 | 50284305 | PDFSTATSENSEMBLUCSC | 454 | 4 | 27797 | gene;tss | 0.043893 | 0.672487 | Not queried | 6.53 | 2.86e-02 | 6.12e-02 |
|  | 20 | 37230326 | 37230741 | PDFSTATSENSEMBLUCSC | 416 | 7 | 26158 | island | 0.037851 | 0.835436 | ARHGAP40 (margin: ARHGAP40) | 6.53 | 5.83e-04 | 6.47e-03 |
|  | 20 | 9819697 | 9819820 | PDFSTATSENSEMBLUCSC | 124 | 6 | 25593 | tss;island | 0.309722 | 0.006363 | PAK7 (margin: PAK7) | 6.53 | 2.16e-03 | 1.45e-02 |
|  | 2 | 210636350 | 210636844 | PDFSTATSENSEMBLUCSC | 495 | 8 | 25053 | island | 0.754735 | 0.036160 | UNC80 (margin: UNC80) | 6.53 | 3.11e-04 | 4.77e-03 |
|  | 13 | 20805094 | 20805679 | PDFSTATSENSEMBLUCSC | 586 | 10 | 11096 | gene;island | 0.152697 | 0.927213 | GJB6 (margin: GJB6) | 6.52 | 1.08e-05 | 4.82e-04 |
|  | 19 | 54024182 | 54024435 | PDFSTATSENSEMBLUCSC | 254 | 4 | 20204 | gene | 0.333428 | 0.001588 | Not queried | 6.52 | 2.86e-02 | 6.12e-02 |
|  | 10 | 116581112 | 116581358 | PDFSTATSENSEMBLUCSC | 247 | 6 | 5378 | tss;island | 0.233004 | 0.004568 | Not queried | 6.52 | 4.11e-02 | 8.14e-02 |
|  | 13 | 36920660 | 36921174 | PDFSTATSENSEMBLUCSC | 515 | 12 | 11394 | tss | 0.223460 | 0.005104 | SPG20OS; SPG20 (margin: SPG20OS; AL139377.1; SPG20) | 6.52 | 3.64e-03 | 2.29e-02 |
|  | 1 | 49242616 | 49242934 | PDFSTATSENSEMBLUCSC | 319 | 5 | 1612 | tss | 0.738194 | 0.015883 | AGBL4; BEND5 (margin: AGBL4; BEND5) | 6.52 | 7.94e-03 | 3.19e-02 |
|  | 10 | 103070221 | 103070503 | PDFSTATSENSEMBLUCSC | 283 | 4 | 6067 | island | 0.786899 | 0.113933 | Not queried | 6.52 | 2.86e-02 | 6.12e-02 |
|  | 19 | 36360396 | 36360605 | PDFSTATSENSEMBLUCSC | 210 | 4 | 19994 | gene;island | 0.170581 | 0.011656 | Not queried | 6.52 | 5.71e-02 | 1.00e-01 |
|  | 3 | 48885189 | 48885665 | PDFSTATSENSEMBLUCSC | 477 | 10 | 29098 | island | 0.005880 | 0.386344 | PRKAR2A-AS1; PRKAR2A (margin: PRKAR2A-AS1; PRKAR2A; SLC25A20) | 6.52 | 2.06e-04 | 3.42e-03 |
|  | 8 | 122651840 | 122652162 | PDFSTATSENSEMBLUCSC | 323 | 4 | 39414 | gene;island | 0.020066 | 0.529299 | Not queried | 6.52 | 2.86e-02 | 6.12e-02 |
|  | 16 | 29296614 | 29296797 | PDFSTATSENSEMBLUCSC | 184 | 4 | 15801 | island | 0.549133 | 0.017397 | Not queried | 6.52 | 2.86e-02 | 6.12e-02 |
|  | 1 | 2461820 | 2461929 | PDFSTATSENSEMBLUCSC | 110 | 6 | 1175 | tss;island | 0.557598 | 0.989228 | HES5 (margin: PANK4; HES5) | 6.52 | 2.16e-03 | 1.45e-02 |
|  | 3 | 89156676 | 89157002 | PDFSTATSENSEMBLUCSC | 327 | 4 | 27868 | gene | 0.469475 | 0.007523 | Not queried | 6.51 | 2.86e-02 | 6.12e-02 |
|  | 1 | 85464000 | 85464261 | PDFSTATSENSEMBLUCSC | 262 | 4 | 1719 | tss;island | 0.360845 | 0.980733 | Not queried | 6.51 | 2.86e-02 | 6.12e-02 |
|  | 6 | 16238916 | 16239221 | PDFSTATSENSEMBLUCSC | 306 | 4 | 33584 | gene;island | 0.557119 | 0.009248 | Not queried | 6.51 | 2.86e-02 | 6.12e-02 |
|  | 21 | 39288661 | 39289266 | PDFSTATSENSEMBLUCSC | 606 | 8 | 26681 | island | 0.536400 | 0.030395 | KCNJ6 (margin: KCNJ6) | 6.51 | 3.11e-04 | 4.77e-03 |
|  | 1 | 177140126 | 177140609 | PDFSTATSENSEMBLUCSC | 484 | 7 | 2108 | tss | 0.957226 | 0.077755 | FAM5B (margin: FAM5B; ASTN1) | 6.51 | 6.99e-03 | 3.19e-02 |
|  | 3 | 161089871 | 161091175 | PDFSTATSENSEMBLUCSC | 1305 | 14 | 28698 | tss | 0.529137 | 0.014096 | SPTSSB (margin: SPTSSB) | 6.51 | 2.53e-05 | 1.02e-03 |
|  | 1 | 23751134 | 23751761 | PDFSTATSENSEMBLUCSC | 628 | 9 | 2835 | island | 0.700462 | 0.031916 | TCEA3 (margin: TCEA3; ASAP3) | 6.51 | 4.11e-05 | 1.17e-03 |
|  | 10 | 44069847 | 44070200 | PDFSTATSENSEMBLUCSC | 354 | 7 | 5700 | island | 0.480529 | 0.010686 | ZNF239 (margin: ZNF239) | 6.50 | 5.83e-04 | 6.47e-03 |
|  | 11 | 120080945 | 120081259 | PDFSTATSENSEMBLUCSC | 315 | 4 | 7759 | tss;island | 0.082943 | 0.873826 | Not queried | 6.50 | 5.71e-02 | 1.00e-01 |
|  | 10 | 44101496 | 44101845 | PDFSTATSENSEMBLUCSC | 350 | 9 | 5053 | tss | 0.784494 | 0.021595 | Not queried | 6.50 | 2.44e-02 | 6.12e-02 |
|  | 2 | 197457277 | 197457430 | PDFSTATSENSEMBLUCSC | 154 | 6 | 24975 | island | 0.594782 | 0.016642 | HECW2 (margin: HECW2) | 6.50 | 2.16e-03 | 1.45e-02 |
|  | 1 | 11751616 | 11751781 | PDFSTATSENSEMBLUCSC | 166 | 4 | 225 | gene;tss;island | 0.527397 | 0.020963 | Not queried | 6.50 | 2.86e-02 | 6.12e-02 |
|  | 2 | 223916502 | 223916952 | PDFSTATSENSEMBLUCSC | 451 | 7 | 25157 | island | 0.266617 | 0.981798 | KCNE4 (margin: KCNE4) | 6.50 | 2.33e-03 | 1.52e-02 |
|  | 2 | 223916502 | 223916820 | PDFSTATSENSEMBLUCSC | 319 | 5 | 23921 | tss | 0.373658 | 0.981798 | Not queried | 6.50 | 1.59e-02 | 5.36e-02 |
|  | 2 | 170220773 | 170221103 | PDFSTATSENSEMBLUCSC | 331 | 4 | 24838 | island | 0.534225 | 0.005307 | Not queried | 6.50 | 5.71e-02 | 1.00e-01 |
|  | 11 | 122852284 | 122852698 | PDFSTATSENSEMBLUCSC | 415 | 9 | 8904 | island | 0.936536 | 0.171709 | BSX (margin: BSX) | 6.50 | 2.88e-04 | 4.58e-03 |
|  | 3 | 49941376 | 49941613 | PDFSTATSENSEMBLUCSC | 238 | 7 | 28339 | tss;island | 0.011266 | 0.453635 | CTD-2330K9.2; CTD-2330K9.3; MST1R (margin: CTD-2330K9.2; CTD-2330K9.3; MST1R; MON1A) | 6.50 | 5.83e-04 | 6.47e-03 |
|  | 3 | 128336483 | 128337044 | PDFSTATSENSEMBLUCSC | 562 | 8 | 29456 | island | 0.543072 | 0.015749 | RPN1 (margin: RPN1) | 6.50 | 1.55e-04 | 2.71e-03 |
|  | 16 | 66969401 | 66969506 | PDFSTATSENSEMBLUCSC | 106 | 4 | 14786 | gene;tss;island | 0.035735 | 0.826148 | Not queried | 6.49 | 2.86e-02 | 6.12e-02 |
|  | 8 | 17433926 | 17434541 | PDFSTATSENSEMBLUCSC | 616 | 9 | 39567 | tss;island | 0.559855 | 0.011597 | PDGFRL (margin: SLC7A2; PDGFRL) | 6.49 | 1.23e-03 | 1.18e-02 |
|  | 3 | 49395574 | 49395745 | PDFSTATSENSEMBLUCSC | 172 | 4 | 27781 | gene | 0.091698 | 0.936949 | Not queried | 6.49 | 2.86e-02 | 6.12e-02 |
|  | 6 | 31126599 | 31127527 | PDFSTATSENSEMBLUCSC | 929 | 22 | 33860 | gene;island | 0.009629 | 0.701390 | TCF19; CCHCR1 (margin: TCF19; CCHCR1; POU5F1) | 6.49 | 1.96e-07 | 2.62e-05 |
|  | 5 | 113697861 | 113698506 | PDFSTATSENSEMBLUCSC | 646 | 8 | 33017 | island | 0.918513 | 0.045542 | KCNN2 (margin: KCNN2) | 6.48 | 6.22e-04 | 6.71e-03 |
|  | 16 | 90114063 | 90114307 | PDFSTATSENSEMBLUCSC | 245 | 6 | 15437 | tss | 0.476131 | 0.015025 | URAHP (margin: GAS8; URAHP; PRDM7) | 6.48 | 2.16e-03 | 1.45e-02 |
|  | 16 | 90113893 | 90114173 | PDFSTATSENSEMBLUCSC | 281 | 4 | 14956 | gene | 0.604223 | 0.015025 | Not queried | 6.48 | 2.86e-02 | 6.12e-02 |
|  | 19 | 37064156 | 37064886 | PDFSTATSENSEMBLUCSC | 731 | 13 | 20003 | gene;island | 0.009136 | 0.396197 | AC092295.7; ZNF529 (margin: AC092295.7; ZNF529) | 6.48 | 1.92e-07 | 2.60e-05 |
|  | 16 | 1251787 | 1252140 | PDFSTATSENSEMBLUCSC | 354 | 4 | 14560 | gene;island | 0.393944 | 0.986045 | Not queried | 6.48 | 2.86e-02 | 6.12e-02 |
|  | 19 | 54668230 | 54668604 | PDFSTATSENSEMBLUCSC | 375 | 5 | 20218 | gene;island | 0.969556 | 0.394043 | TMC4 (margin: CNOT3; LENG1; TMC4; MBOAT7) | 6.48 | 7.94e-03 | 3.19e-02 |
|  | 12 | 64784070 | 64784252 | PDFSTATSENSEMBLUCSC | 183 | 4 | 9203 | gene;island | 0.358566 | 0.980417 | Not queried | 6.48 | 2.86e-02 | 6.12e-02 |
|  | 2 | 223160102 | 223160569 | PDFSTATSENSEMBLUCSC | 468 | 5 | 23045 | gene;island | 0.458132 | 0.984497 | PAX3 (margin: CCDC140; PAX3) | 6.48 | 7.94e-03 | 3.19e-02 |
|  | 20 | 32307885 | 32308119 | PDFSTATSENSEMBLUCSC | 235 | 4 | 25394 | gene | 0.527867 | 0.016180 | Not queried | 6.47 | 2.86e-02 | 6.12e-02 |
|  | 4 | 26585218 | 26585505 | PDFSTATSENSEMBLUCSC | 288 | 6 | 30338 | tss | 0.137125 | 0.002431 | Not queried | 6.47 | 2.60e-02 | 6.12e-02 |
|  | 20 | 170364 | 170641 | PDFSTATSENSEMBLUCSC | 278 | 5 | 25521 | tss | 0.192470 | 0.952277 | DEFB128 (margin: DEFB128; AL360078.1) | 6.47 | 7.94e-03 | 3.19e-02 |
|  | 1 | 1141617 | 1142405 | PDFSTATSENSEMBLUCSC | 789 | 9 | 2414 | island | 0.627937 | 0.032601 | TNFRSF18 (margin: TTLL10; TNFRSF18; TNFRSF4; SDF4) | 6.47 | 1.65e-04 | 2.80e-03 |
|  | 2 | 17721431 | 17722068 | PDFSTATSENSEMBLUCSC | 638 | 11 | 24094 | island | 0.032765 | 0.624398 | VSNL1 (margin: VSNL1) | 6.46 | 2.84e-06 | 1.96e-04 |
|  | 1 | 177140126 | 177140812 | PDFSTATSENSEMBLUCSC | 687 | 9 | 3983 | island | 0.881297 | 0.197926 | Not queried | 6.46 | 2.44e-02 | 6.12e-02 |
|  | 1 | 39874802 | 39875238 | PDFSTATSENSEMBLUCSC | 437 | 13 | 446 | gene;island | 0.749083 | 0.019585 | MACF1; KIAA0754 (margin: MACF1; KIAA0754) | 6.46 | 1.29e-05 | 5.58e-04 |
|  | 15 | 24123232 | 24123421 | PDFSTATSENSEMBLUCSC | 190 | 4 | 13949 | island | 0.389939 | 0.013803 | Not queried | 6.46 | 2.86e-02 | 6.12e-02 |
|  | 10 | 77871958 | 77872458 | PDFSTATSENSEMBLUCSC | 501 | 5 | 4646 | gene;island | 0.823891 | 0.011326 | C10orf11 (margin: C10orf11) | 6.46 | 7.94e-03 | 3.19e-02 |
|  | 12 | 81471311 | 81472177 | PDFSTATSENSEMBLUCSC | 867 | 13 | 10650 | island | 0.536744 | 0.011255 | ACSS3 (margin: ACSS3) | 6.46 | 1.92e-07 | 2.60e-05 |
|  | 16 | 86370962 | 86371442 | PDFSTATSENSEMBLUCSC | 481 | 6 | 14883 | gene | 0.085211 | 0.901485 | LINC00917 (margin: RP11-158I3.3; LINC00917) | 6.46 | 2.16e-03 | 1.45e-02 |
|  | 3 | 47620439 | 47620545 | PDFSTATSENSEMBLUCSC | 107 | 6 | 28288 | tss;island | 0.761374 | 0.025960 | CSPG5 (margin: CSPG5; SMARCC1) | 6.45 | 2.16e-03 | 1.45e-02 |
|  | 15 | 60296138 | 60296273 | PDFSTATSENSEMBLUCSC | 136 | 4 | 13708 | tss | 0.621428 | 0.012177 | Not queried | 6.45 | 2.86e-02 | 6.12e-02 |
|  | 12 | 64784483 | 64784686 | PDFSTATSENSEMBLUCSC | 204 | 5 | 9841 | tss;island | 0.379663 | 0.977796 | RPS11P6; C12orf56 (margin: RPS11P6; C12orf56) | 6.45 | 7.94e-03 | 3.19e-02 |
|  | 6 | 33160250 | 33160604 | PDFSTATSENSEMBLUCSC | 355 | 9 | 34889 | tss | 0.282673 | 0.947236 | COL11A2; RXRB (margin: RNY4P10; SLC39A7; HSD17B8; COL11A2; RXRB) | 6.45 | 4.11e-05 | 1.17e-03 |
|  | 5 | 52083947 | 52084055 | PDFSTATSENSEMBLUCSC | 109 | 6 | 31610 | gene | 0.012348 | 0.421759 | ITGA1; PELO; CTD-2288O8.1 (margin: ITGA1; PELO; CTD-2288O8.1) | 6.45 | 2.16e-03 | 1.45e-02 |
|  | 1 | 201508965 | 201509415 | PDFSTATSENSEMBLUCSC | 451 | 5 | 4078 | island | 0.910190 | 0.045115 | (margin: RP11-134G8.7; RPS10P7) | 6.44 | 7.94e-03 | 3.19e-02 |
|  | 11 | 93754094 | 93754347 | PDFSTATSENSEMBLUCSC | 254 | 4 | 7627 | tss | 0.131032 | 0.960757 | Not queried | 6.44 | 2.86e-02 | 6.12e-02 |
|  | 6 | 29427451 | 29427915 | PDFSTATSENSEMBLUCSC | 465 | 5 | 33657 | gene | 0.006874 | 0.547106 | Not queried | 6.44 | 9.52e-02 | 1.53e-01 |
|  | 17 | 5403805 | 5404090 | PDFSTATSENSEMBLUCSC | 286 | 4 | 16369 | gene;island | 0.832249 | 0.085549 | Not queried | 6.44 | 2.86e-02 | 6.12e-02 |
|  | 8 | 41504078 | 41504331 | PDFSTATSENSEMBLUCSC | 254 | 4 | 39288 | gene;island | 0.479558 | 0.981437 | Not queried | 6.44 | 2.86e-02 | 6.12e-02 |
|  | 10 | 17271006 | 17271303 | PDFSTATSENSEMBLUCSC | 298 | 5 | 4518 | gene;island | 0.433903 | 0.984517 | Not queried | 6.43 | 3.17e-02 | 6.50e-02 |
|  | 19 | 18771079 | 18771327 | PDFSTATSENSEMBLUCSC | 249 | 4 | 19940 | gene;island | 0.206995 | 0.956946 | Not queried | 6.43 | 2.86e-02 | 6.12e-02 |
|  | 3 | 44770851 | 44771316 | PDFSTATSENSEMBLUCSC | 466 | 11 | 29041 | island | 0.273772 | 0.929794 | ZNF501 (margin: ZNF502; ZNF501; KIAA1143) | 6.43 | 5.67e-06 | 3.54e-04 |
|  | 3 | 44770851 | 44771113 | PDFSTATSENSEMBLUCSC | 263 | 9 | 28261 | tss | 0.321675 | 0.976770 | ZNF501 (margin: ZNF502; ZNF501; KIAA1143) | 6.43 | 8.23e-05 | 2.06e-03 |
|  | 8 | 144660590 | 144660772 | PDFSTATSENSEMBLUCSC | 183 | 5 | 39953 | tss | 0.550473 | 0.990780 | RP11-661A12.9; RP11-661A12.7; NAPRT1; EEF1D (margin: RP11-661A12.9; RP11-661A12.7; MROH6; NAPRT1; EEF1D) | 6.43 | 7.94e-03 | 3.19e-02 |
|  | 4 | 25090198 | 25090665 | PDFSTATSENSEMBLUCSC | 468 | 6 | 30958 | island | 0.331168 | 0.972069 | (margin: ) | 6.42 | 2.16e-03 | 1.45e-02 |
|  | 1 | 37500195 | 37500508 | PDFSTATSENSEMBLUCSC | 314 | 5 | 1499 | tss;island | 0.891274 | 0.055756 | GRIK3 (margin: GRIK3) | 6.42 | 7.94e-03 | 3.19e-02 |
|  | 2 | 237086398 | 237086782 | PDFSTATSENSEMBLUCSC | 385 | 4 | 25227 | island | 0.351171 | 0.981949 | Not queried | 6.41 | 2.86e-02 | 6.12e-02 |
|  | 19 | 827715 | 827821 | PDFSTATSENSEMBLUCSC | 107 | 4 | 20280 | tss | 0.222433 | 0.960935 | Not queried | 6.41 | 2.86e-02 | 6.12e-02 |
|  | 5 | 180230767 | 180231194 | PDFSTATSENSEMBLUCSC | 428 | 9 | 31947 | gene;tss;island | 0.136342 | 0.972816 | MGAT1 (margin: MGAT1) | 6.41 | 4.11e-05 | 1.17e-03 |
|  | 8 | 89339404 | 89339622 | PDFSTATSENSEMBLUCSC | 219 | 4 | 39356 | gene | 0.231850 | 0.009233 | Not queried | 6.40 | 1.14e-01 | 1.68e-01 |
|  | 3 | 56502021 | 56502293 | PDFSTATSENSEMBLUCSC | 273 | 5 | 27840 | gene;island | 0.384032 | 0.010285 | ERC2 (margin: ERC2) | 6.40 | 7.94e-03 | 3.19e-02 |
|  | 15 | 52587751 | 52588279 | PDFSTATSENSEMBLUCSC | 529 | 8 | 14149 | island | 0.476469 | 0.011951 | MYO5C (margin: MYO5C; MYO5A) | 6.40 | 1.55e-04 | 2.71e-03 |
|  | 6 | 152126736 | 152126938 | PDFSTATSENSEMBLUCSC | 203 | 5 | 34512 | gene;island | 0.132970 | 0.866096 | ESR1 (margin: ESR1; RP3-443C4.2) | 6.40 | 7.94e-03 | 3.19e-02 |
|  | 2 | 162930666 | 162931003 | PDFSTATSENSEMBLUCSC | 338 | 4 | 22911 | gene | 0.344428 | 0.010690 | Not queried | 6.39 | 2.00e-01 | 2.55e-01 |
|  | 20 | 45179226 | 45179413 | PDFSTATSENSEMBLUCSC | 188 | 5 | 25793 | tss | 0.056548 | 0.921750 | Not queried | 6.39 | 1.59e-02 | 5.36e-02 |
|  | 19 | 37959744 | 37960107 | PDFSTATSENSEMBLUCSC | 364 | 8 | 21956 | island | 0.505132 | 0.011363 | ZNF570; ZNF569 (margin: ZNF570; ZNF569) | 6.39 | 6.99e-03 | 3.19e-02 |
|  | 6 | 42946962 | 42947317 | PDFSTATSENSEMBLUCSC | 356 | 9 | 36146 | island | 0.014483 | 0.635762 | PEX6 (margin: PPP2R5D; PEX6) | 6.39 | 4.11e-05 | 1.17e-03 |
|  | 5 | 63802106 | 63802993 | PDFSTATSENSEMBLUCSC | 888 | 10 | 32829 | island | 0.660904 | 0.049289 | Not queried | 6.39 | 1.85e-02 | 6.12e-02 |
|  | 7 | 27204663 | 27205114 | PDFSTATSENSEMBLUCSC | 452 | 7 | 36938 | gene | 0.293685 | 0.951591 | HOXA9; RP1-170O19.20 (margin: HOXA-AS3; RP1-170O19.21; HOXA-AS4; HOXA7; HOXA9; RP1-170O19.20; MIR196B; HOXA10) | 6.38 | 5.83e-04 | 6.47e-03 |
|  | 10 | 88718164 | 88718393 | PDFSTATSENSEMBLUCSC | 230 | 4 | 5201 | tss | 0.047892 | 0.528624 | Not queried | 6.38 | 2.86e-02 | 6.12e-02 |
|  | 6 | 151814892 | 151814994 | PDFSTATSENSEMBLUCSC | 103 | 5 | 35301 | tss;island | 0.564496 | 0.019981 | CCDC170 (margin: CCDC170) | 6.38 | 7.94e-03 | 3.19e-02 |
|  | 4 | 122302226 | 122302421 | PDFSTATSENSEMBLUCSC | 196 | 7 | 30570 | tss;island | 0.315206 | 0.978108 | QRFPR (margin: QRFPR; RP11-364P2.2) | 6.38 | 5.83e-04 | 6.47e-03 |
|  | 6 | 55443757 | 55444488 | PDFSTATSENSEMBLUCSC | 732 | 10 | 36218 | island | 0.649865 | 0.037714 | HMGCLL1 (margin: HMGCLL1) | 6.38 | 4.33e-05 | 1.22e-03 |
|  | 2 | 12856626 | 12856970 | PDFSTATSENSEMBLUCSC | 345 | 9 | 23194 | tss | 0.180360 | 0.004155 | RP11-333O1.1; TRIB2 (margin: RP11-333O1.1; TRIB2) | 6.38 | 4.11e-05 | 1.17e-03 |
|  | 4 | 170913337 | 170913676 | PDFSTATSENSEMBLUCSC | 340 | 5 | 30177 | gene | 0.481659 | 0.978187 | Not queried | 6.38 | 3.17e-02 | 6.50e-02 |
|  | 2 | 228582688 | 228582820 | PDFSTATSENSEMBLUCSC | 133 | 6 | 25168 | island | 0.309152 | 0.001938 | SLC19A3 (margin: SLC19A3) | 6.38 | 2.16e-03 | 1.45e-02 |
|  | 19 | 8115260 | 8115480 | PDFSTATSENSEMBLUCSC | 221 | 4 | 21474 | island | 0.196545 | 0.950464 | Not queried | 6.37 | 2.86e-02 | 6.12e-02 |
|  | 15 | 79383385 | 79383980 | PDFSTATSENSEMBLUCSC | 596 | 14 | 13842 | tss;island | 0.540917 | 0.008829 | RASGRF1 (margin: RASGRF1) | 6.37 | 4.99e-08 | 9.34e-06 |
|  | 6 | 71122912 | 71123290 | PDFSTATSENSEMBLUCSC | 379 | 8 | 36238 | island | 0.011426 | 0.508432 | FAM135A; RP11-462G2.2 (margin: FAM135A; RP11-462G2.2) | 6.37 | 1.55e-04 | 2.71e-03 |
|  | 4 | 681086 | 681509 | PDFSTATSENSEMBLUCSC | 424 | 4 | 29832 | gene;island | 0.107733 | 0.782375 | Not queried | 6.37 | 2.86e-02 | 6.12e-02 |
|  | 10 | 112258394 | 112258680 | PDFSTATSENSEMBLUCSC | 287 | 4 | 4754 | gene;island | 0.017099 | 0.555277 | Not queried | 6.37 | 2.86e-02 | 6.12e-02 |
|  | 2 | 170335723 | 170335894 | PDFSTATSENSEMBLUCSC | 172 | 5 | 23702 | tss;island | 0.557555 | 0.021626 | Not queried | 6.37 | 9.52e-02 | 1.53e-01 |
|  | 17 | 6899085 | 6899380 | PDFSTATSENSEMBLUCSC | 296 | 9 | 17056 | tss | 0.325649 | 0.980788 | ALOX12; AC027763.2; RP11-589P10.7; RP11-589P10.5 (margin: ALOX12; AC027763.2; RP11-589P10.7; RP11-589P10.5) | 6.36 | 4.11e-05 | 1.17e-03 |
|  | 20 | 749264 | 749620 | PDFSTATSENSEMBLUCSC | 357 | 6 | 25529 | tss | 0.013551 | 0.627063 | SLC52A3 (margin: SLC52A3) | 6.36 | 2.16e-03 | 1.45e-02 |
|  | 1 | 77333749 | 77334256 | PDFSTATSENSEMBLUCSC | 508 | 7 | 592 | gene;island | 0.754421 | 0.322444 | Not queried | 6.36 | 2.59e-01 | 3.16e-01 |
|  | 6 | 33172442 | 33172572 | PDFSTATSENSEMBLUCSC | 131 | 6 | 34227 | gene | 0.010851 | 0.455195 | SLC39A7; HSD17B8 (margin: RNY4P10; SLC39A7; HSD17B8; MIR219-1; RING1; ZNF70P1; RXRB) | 6.36 | 2.16e-03 | 1.45e-02 |
|  | 11 | 63438586 | 63439313 | PDFSTATSENSEMBLUCSC | 728 | 13 | 8371 | island | 0.723416 | 0.013477 | ATL3 (margin: RTN3; ATL3) | 6.36 | 1.30e-04 | 2.71e-03 |
|  | 20 | 31870775 | 31870935 | PDFSTATSENSEMBLUCSC | 161 | 4 | 25681 | tss | 0.027122 | 0.696052 | Not queried | 6.36 | 2.86e-02 | 6.12e-02 |
|  | 4 | 113970617 | 113970935 | PDFSTATSENSEMBLUCSC | 319 | 6 | 30103 | gene | 0.041525 | 0.823644 | ANK2; RP11-650J17.1 (margin: ANK2; RP11-650J17.1) | 6.36 | 4.33e-03 | 2.52e-02 |
|  | 1 | 166039444 | 166039918 | PDFSTATSENSEMBLUCSC | 475 | 5 | 867 | gene | 0.031010 | 0.722946 | Not queried | 6.35 | 3.17e-02 | 6.50e-02 |
|  | 5 | 2751822 | 2751872 | PDFSTATSENSEMBLUCSC | 51 | 4 | 31975 | tss;island | 0.259002 | 0.005147 | Not queried | 6.35 | 2.86e-02 | 6.12e-02 |
|  | 7 | 132261287 | 132261418 | PDFSTATSENSEMBLUCSC | 132 | 4 | 37230 | gene;island | 0.771043 | 0.070029 | Not queried | 6.35 | 2.86e-02 | 6.12e-02 |
|  | 8 | 67344553 | 67344665 | PDFSTATSENSEMBLUCSC | 113 | 6 | 39742 | tss | 0.413016 | 0.981792 | RRS1; ADHFE1 (margin: RRS1; ADHFE1; RP11-346I3.4) | 6.34 | 2.16e-03 | 1.45e-02 |
|  | 13 | 114880761 | 114881194 | PDFSTATSENSEMBLUCSC | 434 | 6 | 11316 | gene;island | 0.296170 | 0.970036 | RASA3 (margin: RASA3; RASA3-IT1) | 6.34 | 2.16e-03 | 1.45e-02 |
|  | 1 | 99729757 | 99729807 | PDFSTATSENSEMBLUCSC | 51 | 6 | 1773 | tss;island | 0.794715 | 0.067753 | LPPR4 (margin: LPPR4) | 6.34 | 2.16e-03 | 1.45e-02 |
|  | 6 | 26550783 | 26551010 | PDFSTATSENSEMBLUCSC | 228 | 4 | 35540 | island | 0.017125 | 0.461687 | Not queried | 6.33 | 2.86e-02 | 6.12e-02 |
|  | 11 | 111847692 | 111848400 | PDFSTATSENSEMBLUCSC | 709 | 10 | 6903 | gene;island | 0.702735 | 0.030183 | DIXDC1 (margin: DIXDC1) | 6.33 | 8.93e-03 | 3.45e-02 |
|  | 6 | 43044771 | 43045187 | PDFSTATSENSEMBLUCSC | 417 | 4 | 34342 | gene;island | 0.008481 | 0.435573 | Not queried | 6.33 | 2.86e-02 | 6.12e-02 |
|  | 15 | 83953690 | 83954395 | PDFSTATSENSEMBLUCSC | 706 | 18 | 13865 | tss;island | 0.122262 | 0.926995 | RP11-382A20.4; BNC1 (margin: RP11-382A20.4; BNC1) | 6.33 | 2.20e-10 | 1.16e-07 |
|  | 14 | 95236539 | 95236615 | PDFSTATSENSEMBLUCSC | 77 | 4 | 12585 | tss;island | 0.446348 | 0.010773 | Not queried | 6.33 | 2.86e-02 | 6.12e-02 |
|  | 6 | 32710933 | 32711102 | PDFSTATSENSEMBLUCSC | 170 | 4 | 34128 | gene | 0.127681 | 0.950331 | Not queried | 6.33 | 1.14e-01 | 1.68e-01 |
|  | 4 | 90031538 | 90031969 | PDFSTATSENSEMBLUCSC | 432 | 4 | 31177 | island | 0.868006 | 0.117612 | Not queried | 6.33 | 1.14e-01 | 1.68e-01 |
|  | 7 | 12610355 | 12610833 | PDFSTATSENSEMBLUCSC | 479 | 6 | 36896 | gene | 0.684784 | 0.046006 | SCIN; AC005281.2 (margin: SCIN; AC005281.2) | 6.32 | 2.16e-03 | 1.45e-02 |
|  | 2 | 43903470 | 43904011 | PDFSTATSENSEMBLUCSC | 542 | 8 | 22660 | gene;tss | 0.207571 | 0.004602 | PLEKHH2; AC011242.6 (margin: PLEKHH2; AC011242.6; AC011242.5) | 6.32 | 6.99e-03 | 3.19e-02 |
|  | 5 | 134210707 | 134210839 | PDFSTATSENSEMBLUCSC | 133 | 4 | 31754 | gene;island | 0.004680 | 0.219605 | Not queried | 6.32 | 2.86e-02 | 6.12e-02 |
|  | 5 | 139927007 | 139927242 | PDFSTATSENSEMBLUCSC | 236 | 8 | 32342 | tss | 0.010983 | 0.458789 | ANKHD1; ANKHD1-EIF4EBP3; EIF4EBP3; SRA1 (margin: ANKHD1; ANKHD1-EIF4EBP3; EIF4EBP3; SRA1; APBB3) | 6.32 | 1.55e-04 | 2.71e-03 |
|  | 6 | 32050905 | 32051108 | PDFSTATSENSEMBLUCSC | 204 | 5 | 34037 | gene;island | 0.024513 | 0.656600 | TNXB (margin: TNXB; RNA5SP206) | 6.31 | 7.94e-03 | 3.19e-02 |
|  | 17 | 42733527 | 42733729 | PDFSTATSENSEMBLUCSC | 203 | 8 | 17412 | tss;island | 0.920764 | 0.221093 | C17orf104 (margin: RP11-1072C15.3; C17orf104; RP11-1072C15.2) | 6.31 | 1.55e-04 | 2.71e-03 |
|  | 7 | 134576049 | 134576386 | PDFSTATSENSEMBLUCSC | 338 | 6 | 37233 | gene | 0.011827 | 0.474737 | CALD1 (margin: CALD1) | 6.31 | 2.16e-03 | 1.45e-02 |
|  | 17 | 77776428 | 77776716 | PDFSTATSENSEMBLUCSC | 289 | 4 | 18843 | island | 0.357273 | 0.975415 | Not queried | 6.31 | 2.86e-02 | 6.12e-02 |
|  | 3 | 44753865 | 44754131 | PDFSTATSENSEMBLUCSC | 267 | 8 | 28260 | tss | 0.395013 | 0.976595 | ZNF502; SOCS5P3 (margin: ZNF502; SOCS5P3) | 6.31 | 1.86e-03 | 1.45e-02 |
|  | 8 | 67344553 | 67344720 | PDFSTATSENSEMBLUCSC | 168 | 7 | 40354 | island | 0.435886 | 0.984524 | RRS1; ADHFE1 (margin: RRS1; ADHFE1; RP11-346I3.4) | 6.30 | 5.83e-04 | 6.47e-03 |
|  | 12 | 24715864 | 24716204 | PDFSTATSENSEMBLUCSC | 341 | 4 | 9606 | tss;island | 0.513088 | 0.014652 | Not queried | 6.30 | 2.86e-02 | 6.12e-02 |
|  | 8 | 108510286 | 108510343 | PDFSTATSENSEMBLUCSC | 58 | 4 | 39865 | tss | 0.904093 | 0.112200 | Not queried | 6.30 | 2.86e-02 | 6.12e-02 |
|  | 10 | 97667581 | 97667923 | PDFSTATSENSEMBLUCSC | 343 | 6 | 5969 | island | 0.546975 | 0.019100 | RP11-248J23.7; C10orf131; ENTPD1-AS1 (margin: RP11-248J23.7; C10orf131; ENTPD1-AS1) | 6.30 | 2.16e-03 | 1.45e-02 |
|  | 10 | 97667581 | 97667677 | PDFSTATSENSEMBLUCSC | 97 | 4 | 5251 | tss | 0.581677 | 0.019100 | Not queried | 6.30 | 2.86e-02 | 6.12e-02 |
|  | 3 | 48885314 | 48885665 | PDFSTATSENSEMBLUCSC | 352 | 9 | 28308 | tss | 0.005971 | 0.373760 | PRKAR2A-AS1; PRKAR2A (margin: PRKAR2A-AS1; PRKAR2A; SLC25A20) | 6.30 | 7.82e-04 | 8.17e-03 |
|  | 2 | 12858298 | 12858655 | PDFSTATSENSEMBLUCSC | 358 | 5 | 22577 | gene;island | 0.225473 | 0.832890 | RP11-333O1.1; TRIB2 (margin: RP11-333O1.1; TRIB2) | 6.29 | 7.94e-03 | 3.19e-02 |
|  | 11 | 66824138 | 66824219 | PDFSTATSENSEMBLUCSC | 82 | 5 | 7483 | tss;island | 0.008882 | 0.371961 | RHOD (margin: SYT12; RHOD) | 6.29 | 7.94e-03 | 3.19e-02 |
|  | 13 | 70682525 | 70682771 | PDFSTATSENSEMBLUCSC | 247 | 5 | 11176 | gene;island | 0.130157 | 0.980361 | ATXN8OS; KLHL1 (margin: ATXN8OS; KLHL1) | 6.29 | 7.94e-03 | 3.19e-02 |
|  | 19 | 34111400 | 34111733 | PDFSTATSENSEMBLUCSC | 334 | 5 | 20686 | tss;island | 0.411950 | 0.979887 | CHST8 (margin: CHST8) | 6.29 | 7.94e-03 | 3.19e-02 |
|  | 18 | 47792917 | 47793175 | PDFSTATSENSEMBLUCSC | 259 | 6 | 19274 | tss | 0.281794 | 0.007149 | CCDC11; MBD1 (margin: CCDC11; MBD1) | 6.29 | 2.16e-03 | 1.45e-02 |
|  | 13 | 36050788 | 36051073 | PDFSTATSENSEMBLUCSC | 286 | 8 | 11127 | gene;island | 0.138508 | 0.902072 | NBEA; MAB21L1 (margin: NBEA; MAB21L1) | 6.29 | 1.55e-04 | 2.71e-03 |
|  | 1 | 107684059 | 107684425 | PDFSTATSENSEMBLUCSC | 367 | 4 | 659 | gene;island | 0.503182 | 0.011244 | Not queried | 6.28 | 2.86e-02 | 6.12e-02 |
|  | 8 | 145018010 | 145018300 | PDFSTATSENSEMBLUCSC | 291 | 5 | 39499 | gene;tss;island | 0.547107 | 0.018419 | PLEC; MIR661 (margin: PLEC; MIR661) | 6.28 | 7.94e-03 | 3.19e-02 |
|  | 7 | 12609907 | 12610833 | PDFSTATSENSEMBLUCSC | 927 | 15 | 38267 | island | 0.431974 | 0.009856 | SCIN; AC005281.2 (margin: SCIN; AC005281.2) | 6.28 | 3.87e-07 | 4.83e-05 |
|  | 7 | 12609907 | 12610196 | PDFSTATSENSEMBLUCSC | 290 | 9 | 37445 | tss | 0.228348 | 0.007662 | SCIN; AC005281.2 (margin: SCIN; AC005281.2) | 6.28 | 4.11e-05 | 1.17e-03 |
|  | 4 | 96468962 | 96469634 | PDFSTATSENSEMBLUCSC | 673 | 9 | 30063 | gene;island | 0.669987 | 0.010880 | RP11-710C12.1; UNC5C (margin: RP11-710C12.1; UNC5C) | 6.27 | 1.65e-04 | 2.80e-03 |
|  | 20 | 62680970 | 62681428 | PDFSTATSENSEMBLUCSC | 459 | 11 | 26380 | island | 0.815018 | 0.013399 | TCEA2; ZNF512B; SOX18 (margin: LINC00176; TCEA2; RP13-152O15.5; ZNF512B; SOX18) | 6.27 | 1.28e-04 | 2.71e-03 |
|  | 20 | 62680986 | 62681428 | PDFSTATSENSEMBLUCSC | 443 | 9 | 25909 | tss | 0.818539 | 0.013399 | TCEA2; ZNF512B; SOX18 (margin: LINC00176; TCEA2; RP13-152O15.5; ZNF512B; SOX18) | 6.27 | 7.82e-04 | 8.17e-03 |
|  | 10 | 112256517 | 112256825 | PDFSTATSENSEMBLUCSC | 309 | 4 | 5356 | tss;island | 0.034962 | 0.754468 | Not queried | 6.27 | 2.86e-02 | 6.12e-02 |
|  | 12 | 45269519 | 45269790 | PDFSTATSENSEMBLUCSC | 272 | 5 | 9651 | tss | 0.601295 | 0.019498 | NELL2 (margin: NELL2; Y\_RNA) | 6.27 | 7.94e-03 | 3.19e-02 |
|  | 4 | 74964856 | 74965278 | PDFSTATSENSEMBLUCSC | 423 | 11 | 31102 | island | 0.011064 | 0.363721 | CXCL2 (margin: CXCL2) | 6.27 | 2.84e-06 | 1.96e-04 |
|  | 4 | 74965068 | 74965278 | PDFSTATSENSEMBLUCSC | 211 | 9 | 30434 | tss | 0.011064 | 0.350260 | CXCL2 (margin: CXCL2) | 6.27 | 4.11e-05 | 1.17e-03 |
|  | 2 | 70994802 | 70995134 | PDFSTATSENSEMBLUCSC | 333 | 4 | 22725 | gene;island | 0.063351 | 0.860000 | Not queried | 6.27 | 2.86e-02 | 6.12e-02 |
|  | 3 | 168863860 | 168864313 | PDFSTATSENSEMBLUCSC | 454 | 7 | 28020 | gene;island | 0.009253 | 0.248934 | MECOM (margin: MECOM) | 6.27 | 5.83e-04 | 6.47e-03 |
|  | 5 | 140602851 | 140603034 | PDFSTATSENSEMBLUCSC | 184 | 4 | 32367 | tss;island | 0.005546 | 0.262907 | Not queried | 6.26 | 2.86e-02 | 6.12e-02 |
|  | 11 | 65779192 | 65779343 | PDFSTATSENSEMBLUCSC | 152 | 6 | 7456 | tss | 0.237062 | 0.950759 | CST6 (margin: BANF1; CST6; EIF1AD; CATSPER1) | 6.26 | 2.16e-03 | 1.45e-02 |
|  | 19 | 18496984 | 18497201 | PDFSTATSENSEMBLUCSC | 218 | 4 | 19934 | gene | 0.021011 | 0.634013 | Not queried | 6.26 | 2.00e-01 | 2.55e-01 |
|  | 2 | 228028601 | 228028776 | PDFSTATSENSEMBLUCSC | 176 | 4 | 23057 | gene;tss;island | 0.332318 | 0.006036 | Not queried | 6.26 | 5.71e-02 | 1.00e-01 |
|  | 2 | 189156874 | 189157566 | PDFSTATSENSEMBLUCSC | 693 | 11 | 24953 | island | 0.009169 | 0.441112 | GULP1 (margin: GULP1; MIR561; AC068718.1) | 6.26 | 8.51e-05 | 2.07e-03 |
|  | 2 | 189156874 | 189157361 | PDFSTATSENSEMBLUCSC | 488 | 9 | 23777 | tss | 0.009169 | 0.461662 | GULP1 (margin: GULP1; MIR561; AC068718.1) | 6.26 | 7.82e-04 | 8.17e-03 |
|  | 5 | 100238977 | 100239071 | PDFSTATSENSEMBLUCSC | 95 | 6 | 32196 | tss | 0.107932 | 0.004465 | ST8SIA4 (margin: ST8SIA4) | 6.26 | 2.16e-03 | 1.45e-02 |
|  | 3 | 138665654 | 138666473 | PDFSTATSENSEMBLUCSC | 820 | 13 | 28626 | tss;island | 0.445298 | 0.983005 | C3orf72; FOXL2 (margin: C3orf72; RP11-548O1.3; FOXL2) | 6.25 | 7.69e-07 | 7.42e-05 |
|  | 2 | 178937517 | 178937882 | PDFSTATSENSEMBLUCSC | 366 | 8 | 23751 | tss | 0.241980 | 0.011030 | PDE11A (margin: PDE11A) | 6.25 | 4.66e-03 | 2.68e-02 |
|  | 21 | 39288720 | 39289266 | PDFSTATSENSEMBLUCSC | 547 | 7 | 26544 | tss | 0.575433 | 0.033411 | KCNJ6 (margin: KCNJ6) | 6.25 | 1.17e-03 | 1.13e-02 |
|  | 16 | 30022548 | 30022725 | PDFSTATSENSEMBLUCSC | 178 | 7 | 15175 | tss;island | 0.279049 | 0.002162 | DOC2A (margin: INO80E; C16orf92; DOC2A) | 6.25 | 1.17e-03 | 1.13e-02 |
|  | 15 | 40268421 | 40268777 | PDFSTATSENSEMBLUCSC | 357 | 4 | 13286 | gene;island | 0.691962 | 0.027632 | Not queried | 6.25 | 2.00e-01 | 2.55e-01 |
|  | 1 | 239549577 | 239549853 | PDFSTATSENSEMBLUCSC | 277 | 6 | 4370 | island | 0.786841 | 0.016996 | CHRM3 (margin: CHRM3) | 6.25 | 2.16e-03 | 1.45e-02 |
|  | 6 | 168435914 | 168436099 | PDFSTATSENSEMBLUCSC | 186 | 4 | 34558 | gene;island | 0.868309 | 0.085299 | Not queried | 6.24 | 5.71e-02 | 1.00e-01 |
|  | 18 | 44099028 | 44099340 | PDFSTATSENSEMBLUCSC | 313 | 5 | 19103 | gene | 0.050106 | 0.700953 | Not queried | 6.24 | 1.51e-01 | 2.11e-01 |
|  | 7 | 100463416 | 100463759 | PDFSTATSENSEMBLUCSC | 344 | 4 | 37148 | gene;island | 0.049159 | 0.755116 | Not queried | 6.24 | 2.86e-02 | 6.12e-02 |
|  | 1 | 3059517 | 3059820 | PDFSTATSENSEMBLUCSC | 304 | 4 | 104 | gene;island | 0.249965 | 0.972655 | Not queried | 6.24 | 2.86e-02 | 6.12e-02 |
|  | 10 | 119304583 | 119304719 | PDFSTATSENSEMBLUCSC | 137 | 5 | 5392 | tss | 0.442560 | 0.984314 | EMX2; EMX2OS (margin: EMX2; EMX2OS) | 6.24 | 7.94e-03 | 3.19e-02 |
|  | 19 | 57149436 | 57149813 | PDFSTATSENSEMBLUCSC | 378 | 5 | 22451 | island | 0.852907 | 0.257036 | Not queried | 6.24 | 3.10e-01 | 3.66e-01 |
|  | 19 | 54224374 | 54224709 | PDFSTATSENSEMBLUCSC | 336 | 4 | 21038 | tss | 0.066416 | 0.967305 | Not queried | 6.24 | 5.71e-02 | 1.00e-01 |
|  | 12 | 65672405 | 65672531 | PDFSTATSENSEMBLUCSC | 127 | 4 | 10599 | island | 0.008929 | 0.376284 | Not queried | 6.23 | 2.86e-02 | 6.12e-02 |
|  | 19 | 37959744 | 37959961 | PDFSTATSENSEMBLUCSC | 218 | 7 | 20739 | tss | 0.538243 | 0.013255 | Not queried | 6.23 | 1.75e-02 | 5.79e-02 |
|  | 6 | 291687 | 291909 | PDFSTATSENSEMBLUCSC | 223 | 4 | 34585 | tss;island | 0.007615 | 0.388765 | Not queried | 6.23 | 2.86e-02 | 6.12e-02 |
|  | 8 | 49231559 | 49231786 | PDFSTATSENSEMBLUCSC | 228 | 4 | 40280 | island | 0.007867 | 0.167879 | Not queried | 6.23 | 2.86e-02 | 6.12e-02 |
|  | 2 | 154335014 | 154335250 | PDFSTATSENSEMBLUCSC | 237 | 4 | 22899 | gene | 0.412896 | 0.027492 | Not queried | 6.23 | 2.86e-02 | 6.12e-02 |
|  | 1 | 82268588 | 82268801 | PDFSTATSENSEMBLUCSC | 214 | 4 | 598 | gene;island | 0.487875 | 0.024127 | Not queried | 6.23 | 2.86e-02 | 6.12e-02 |
|  | 19 | 37329330 | 37329859 | PDFSTATSENSEMBLUCSC | 530 | 13 | 20730 | tss | 0.558811 | 0.011420 | ZNF790 (margin: CTD-2162K18.5; ZNF345; ZNF790) | 6.22 | 1.92e-07 | 2.60e-05 |
|  | 8 | 144654887 | 144655030 | PDFSTATSENSEMBLUCSC | 144 | 4 | 40672 | island | 0.107611 | 0.913290 | Not queried | 6.22 | 2.86e-02 | 6.12e-02 |
|  | 22 | 49376354 | 49376628 | PDFSTATSENSEMBLUCSC | 275 | 4 | 27602 | island | 0.036200 | 0.858214 | Not queried | 6.22 | 2.00e-01 | 2.55e-01 |
|  | 1 | 17308032 | 17308303 | PDFSTATSENSEMBLUCSC | 272 | 5 | 1288 | tss;island | 0.156133 | 0.950767 | RP1-37C10.3; MFAP2 (margin: CROCC; RP1-37C10.3; MFAP2; ATP13A2) | 6.22 | 7.94e-03 | 3.19e-02 |
|  | 12 | 125223502 | 125223679 | PDFSTATSENSEMBLUCSC | 178 | 4 | 10976 | island | 0.172318 | 0.803249 | Not queried | 6.22 | 2.86e-02 | 6.12e-02 |
|  | 3 | 138665654 | 138665957 | PDFSTATSENSEMBLUCSC | 304 | 4 | 27966 | gene | 0.242754 | 0.979615 | Not queried | 6.22 | 2.86e-02 | 6.12e-02 |
|  | 12 | 6809792 | 6809939 | PDFSTATSENSEMBLUCSC | 148 | 6 | 10206 | island | 0.433403 | 0.048832 | Not queried | 6.22 | 4.11e-02 | 8.14e-02 |
|  | 15 | 60289239 | 60289532 | PDFSTATSENSEMBLUCSC | 294 | 4 | 14187 | island | 0.316452 | 0.872513 | Not queried | 6.21 | 2.86e-02 | 6.12e-02 |
|  | 8 | 63997977 | 63998778 | PDFSTATSENSEMBLUCSC | 802 | 11 | 40337 | island | 0.447284 | 0.022318 | TTPA (margin: TTPA) | 6.21 | 8.51e-05 | 2.07e-03 |
|  | 17 | 42907937 | 42908410 | PDFSTATSENSEMBLUCSC | 474 | 5 | 17416 | tss;island | 0.314644 | 0.980501 | Not queried | 6.21 | 1.59e-02 | 5.36e-02 |
|  | 17 | 46670933 | 46671635 | PDFSTATSENSEMBLUCSC | 703 | 13 | 16655 | gene;island | 0.548156 | 0.029049 | HOXB-AS3; HOXB3; HOXB5; HOXB6 (margin: HOXB-AS3; HOXB3; MIR10A; HOXB5; HOXB6) | 6.21 | 2.28e-04 | 3.75e-03 |
|  | 3 | 161089712 | 161091175 | PDFSTATSENSEMBLUCSC | 1464 | 15 | 29638 | island | 0.487654 | 0.015737 | SPTSSB (margin: SPTSSB) | 6.21 | 8.82e-06 | 4.82e-04 |
|  | 2 | 201450323 | 201450743 | PDFSTATSENSEMBLUCSC | 421 | 11 | 25003 | island | 0.413673 | 0.980182 | SGOL2; AOX1 (margin: SGOL2; AOX1) | 6.20 | 2.84e-06 | 1.96e-04 |
|  | 2 | 201450323 | 201450690 | PDFSTATSENSEMBLUCSC | 368 | 9 | 23810 | tss | 0.457338 | 0.984032 | SGOL2; AOX1 (margin: SGOL2; AOX1) | 6.20 | 4.11e-05 | 1.17e-03 |
|  | 11 | 71163428 | 71164189 | PDFSTATSENSEMBLUCSC | 762 | 11 | 7531 | tss | 0.222654 | 0.003882 | Not queried | 6.20 | 1.01e-01 | 1.62e-01 |
|  | 14 | 60974384 | 60974525 | PDFSTATSENSEMBLUCSC | 142 | 5 | 12904 | island | 0.206321 | 0.970574 | Not queried | 6.20 | 9.52e-02 | 1.53e-01 |
|  | 12 | 81331457 | 81331845 | PDFSTATSENSEMBLUCSC | 389 | 7 | 10649 | island | 0.971457 | 0.223871 | ACSS3; LIN7A; MIR618 (margin: ACSS3; LIN7A; MIR618) | 6.20 | 6.99e-03 | 3.19e-02 |
|  | 5 | 131592959 | 131593413 | PDFSTATSENSEMBLUCSC | 455 | 10 | 33086 | island | 0.277509 | 0.957158 | PDLIM4; P4HA2 (margin: PDLIM4; P4HA2) | 6.19 | 1.30e-04 | 2.71e-03 |
|  | 3 | 186490656 | 186490915 | PDFSTATSENSEMBLUCSC | 260 | 5 | 29752 | island | 0.681611 | 0.025317 | Not queried | 6.19 | 1.59e-02 | 5.36e-02 |
|  | 11 | 2172019 | 2172168 | PDFSTATSENSEMBLUCSC | 150 | 4 | 6450 | gene;tss | 0.019235 | 0.524214 | Not queried | 6.19 | 2.86e-02 | 6.12e-02 |
|  | 6 | 39895360 | 39895717 | PDFSTATSENSEMBLUCSC | 358 | 5 | 34326 | gene | 0.063668 | 0.936333 | Not queried | 6.18 | 1.59e-02 | 5.36e-02 |
|  | 10 | 94833042 | 94833214 | PDFSTATSENSEMBLUCSC | 173 | 6 | 5234 | tss;island | 0.865288 | 0.050848 | CYP26A1 (margin: CYP26C1; CYP26A1) | 6.18 | 2.16e-03 | 1.45e-02 |
|  | 19 | 4769531 | 4769690 | PDFSTATSENSEMBLUCSC | 160 | 7 | 19739 | gene;tss | 0.407219 | 0.005552 | MIR7-3HG; MIR7-3 (margin: MIR7-3HG; MIR7-3) | 6.18 | 5.83e-04 | 6.47e-03 |
|  | 10 | 23460245 | 23460646 | PDFSTATSENSEMBLUCSC | 402 | 4 | 5607 | island | 0.960927 | 0.277394 | Not queried | 6.18 | 1.14e-01 | 1.68e-01 |
|  | 6 | 11538528 | 11538767 | PDFSTATSENSEMBLUCSC | 240 | 4 | 33571 | gene;island | 0.554839 | 0.015035 | Not queried | 6.18 | 2.86e-02 | 6.12e-02 |
|  | 16 | 4421445 | 4421654 | PDFSTATSENSEMBLUCSC | 210 | 5 | 14638 | gene;tss;island | 0.838493 | 0.115543 | VASN; CORO7-PAM16; CORO7 (margin: VASN; CORO7-PAM16; CORO7) | 6.18 | 7.94e-03 | 3.19e-02 |
|  | 13 | 113106260 | 113106473 | PDFSTATSENSEMBLUCSC | 214 | 4 | 11921 | island | 0.325391 | 0.952085 | Not queried | 6.18 | 2.86e-02 | 6.12e-02 |
|  | 19 | 54850444 | 54850645 | PDFSTATSENSEMBLUCSC | 202 | 4 | 21059 | tss | 0.051111 | 0.809861 | Not queried | 6.18 | 3.43e-01 | 3.84e-01 |
|  | 12 | 2800755 | 2801158 | PDFSTATSENSEMBLUCSC | 404 | 5 | 9007 | gene;island | 0.068173 | 0.959282 | CACNA1C; CACNA1C-AS1 (margin: CACNA1C; CACNA1C-AS1) | 6.18 | 7.94e-03 | 3.19e-02 |
|  | 22 | 37252282 | 37252652 | PDFSTATSENSEMBLUCSC | 371 | 4 | 27460 | island | 0.180673 | 0.017800 | Not queried | 6.17 | 4.86e-01 | 5.11e-01 |
|  | 7 | 194719 | 194861 | PDFSTATSENSEMBLUCSC | 143 | 4 | 36702 | gene;island | 0.118439 | 0.903919 | Not queried | 6.17 | 2.86e-02 | 6.12e-02 |
|  | 11 | 69468789 | 69469175 | PDFSTATSENSEMBLUCSC | 387 | 6 | 6782 | gene | 0.932932 | 0.316453 | CCND1; ORAOV1 (margin: CCND1; ORAOV1) | 6.17 | 2.16e-03 | 1.45e-02 |
|  | 10 | 8101307 | 8101566 | PDFSTATSENSEMBLUCSC | 260 | 4 | 4490 | gene;island | 0.949520 | 0.250557 | Not queried | 6.17 | 2.86e-02 | 6.12e-02 |
|  | 5 | 148521075 | 148521226 | PDFSTATSENSEMBLUCSC | 152 | 4 | 31855 | gene | 0.013823 | 0.323339 | Not queried | 6.17 | 2.86e-02 | 6.12e-02 |
|  | 10 | 127262562 | 127262851 | PDFSTATSENSEMBLUCSC | 290 | 4 | 5428 | tss | 0.133525 | 0.905833 | Not queried | 6.16 | 2.86e-02 | 6.12e-02 |
|  | 17 | 15244970 | 15245237 | PDFSTATSENSEMBLUCSC | 268 | 7 | 17150 | tss | 0.349483 | 0.980780 | TEKT3 (margin: TEKT3) | 6.16 | 5.83e-04 | 6.47e-03 |
|  | 2 | 158731577 | 158731796 | PDFSTATSENSEMBLUCSC | 220 | 4 | 22904 | gene | 0.000915 | 0.190108 | Not queried | 6.16 | 2.86e-02 | 6.12e-02 |
|  | 19 | 45996372 | 45996644 | PDFSTATSENSEMBLUCSC | 273 | 6 | 20099 | gene;island | 0.587822 | 0.030170 | PPM1N; RTN2 (margin: PPM1N; RTN2) | 6.16 | 2.16e-03 | 1.45e-02 |
|  | 10 | 99531765 | 99531934 | PDFSTATSENSEMBLUCSC | 170 | 6 | 5272 | tss | 0.325937 | 0.970258 | SFRP5 (margin: ZFYVE27; SFRP5) | 6.16 | 8.66e-03 | 3.36e-02 |
|  | 11 | 1029029 | 1029336 | PDFSTATSENSEMBLUCSC | 308 | 5 | 6400 | gene;island | 0.374158 | 0.966743 | MUC6 (margin: MUC6) | 6.16 | 7.94e-03 | 3.19e-02 |
|  | 17 | 26645200 | 26645523 | PDFSTATSENSEMBLUCSC | 324 | 4 | 17207 | tss;island | 0.886787 | 0.056729 | Not queried | 6.15 | 5.71e-02 | 1.00e-01 |
|  | 17 | 40346279 | 40346798 | PDFSTATSENSEMBLUCSC | 520 | 8 | 17358 | tss | 0.054685 | 0.714715 | GHDC (margin: HCRT; GHDC; STAT5B) | 6.15 | 3.11e-04 | 4.77e-03 |
|  | 10 | 118084109 | 118084587 | PDFSTATSENSEMBLUCSC | 479 | 6 | 4775 | gene | 0.087577 | 0.912214 | CCDC172 (margin: CCDC172) | 6.15 | 4.33e-03 | 2.52e-02 |
|  | 1 | 220921855 | 220922217 | PDFSTATSENSEMBLUCSC | 363 | 6 | 1020 | gene | 0.456907 | 0.011596 | MARC2 (margin: MARC2) | 6.14 | 2.16e-03 | 1.45e-02 |
|  | 17 | 14213447 | 14213798 | PDFSTATSENSEMBLUCSC | 352 | 4 | 16452 | gene;island | 0.408737 | 0.981301 | Not queried | 6.14 | 2.86e-02 | 6.12e-02 |
|  | 17 | 71161157 | 71161502 | PDFSTATSENSEMBLUCSC | 346 | 7 | 18699 | island | 0.694264 | 0.031428 | RP11-143K11.5; SSTR2; POLR3KP2 (margin: RP11-143K11.5; SSTR2; RP11-143K11.1; POLR3KP2) | 6.13 | 5.83e-04 | 6.47e-03 |
|  | 3 | 10206254 | 10206537 | PDFSTATSENSEMBLUCSC | 284 | 7 | 28145 | tss | 0.028970 | 0.673477 | IRAK2 (margin: IRAK2) | 6.13 | 5.83e-04 | 6.47e-03 |
|  | 7 | 1607146 | 1607868 | PDFSTATSENSEMBLUCSC | 723 | 7 | 36762 | gene;island | 0.340174 | 0.960540 | PSMG3-AS1; PSMG3 (margin: PSMG3-AS1; TMEM184A; PSMG3) | 6.13 | 1.17e-03 | 1.13e-02 |
|  | 11 | 128563483 | 128563903 | PDFSTATSENSEMBLUCSC | 421 | 7 | 6966 | gene;island | 0.367918 | 0.945303 | Not queried | 6.13 | 2.62e-02 | 6.12e-02 |
|  | 22 | 42310846 | 42311374 | PDFSTATSENSEMBLUCSC | 529 | 7 | 27532 | island | 0.806686 | 0.027708 | Not queried | 6.13 | 3.79e-02 | 7.66e-02 |
|  | 2 | 178937477 | 178937882 | PDFSTATSENSEMBLUCSC | 406 | 9 | 22944 | gene;island | 0.262155 | 0.011359 | PDE11A (margin: PDE11A) | 6.13 | 2.76e-03 | 1.78e-02 |
|  | 6 | 36355343 | 36355825 | PDFSTATSENSEMBLUCSC | 483 | 7 | 36081 | island | 0.129236 | 0.957947 | RP1-50J22.4; ETV7 (margin: RP1-50J22.4; ETV7; PXT1) | 6.13 | 2.33e-03 | 1.52e-02 |
|  | 6 | 29910755 | 29911558 | PDFSTATSENSEMBLUCSC | 804 | 22 | 33702 | gene;island | 0.052525 | 0.818446 | HLA-A; HCG4P5 (margin: HLA-U; HLA-A; HCG4P5) | 6.13 | 2.17e-08 | 5.43e-06 |
|  | 6 | 42947051 | 42947317 | PDFSTATSENSEMBLUCSC | 267 | 8 | 34986 | tss | 0.019655 | 0.617764 | PEX6 (margin: PPP2R5D; PEX6) | 6.13 | 1.55e-04 | 2.71e-03 |
|  | 5 | 2038743 | 2038989 | PDFSTATSENSEMBLUCSC | 247 | 4 | 32651 | island | 0.469595 | 0.021283 | Not queried | 6.13 | 2.86e-02 | 6.12e-02 |
|  | 4 | 113970617 | 113970745 | PDFSTATSENSEMBLUCSC | 129 | 4 | 30555 | tss | 0.119083 | 0.762257 | Not queried | 6.13 | 5.71e-02 | 1.00e-01 |
|  | 1 | 6659205 | 6659504 | PDFSTATSENSEMBLUCSC | 300 | 5 | 181 | gene;island | 0.431185 | 0.978370 | KLHL21 (margin: ZBTB48; KLHL21) | 6.13 | 7.94e-03 | 3.19e-02 |
|  | 8 | 26724744 | 26725033 | PDFSTATSENSEMBLUCSC | 290 | 5 | 40177 | island | 0.231974 | 0.008826 | Not queried | 6.12 | 3.17e-02 | 6.50e-02 |
|  | 5 | 78985425 | 78985592 | PDFSTATSENSEMBLUCSC | 168 | 9 | 32147 | tss | 0.544246 | 0.021568 | CMYA5 (margin: PAPD4; CMYA5) | 6.12 | 4.11e-05 | 1.17e-03 |
|  | 15 | 52588020 | 52588279 | PDFSTATSENSEMBLUCSC | 260 | 6 | 13679 | tss | 0.449042 | 0.012765 | MYO5C (margin: MYO5C; MYO5A) | 6.12 | 2.16e-03 | 1.45e-02 |
|  | 17 | 21454528 | 21455116 | PDFSTATSENSEMBLUCSC | 589 | 6 | 18125 | island | 0.015745 | 0.460394 | RP11-822E23.3; C17orf51 (margin: RP11-822E23.2; RP11-822E23.3; C17orf51) | 6.12 | 2.16e-03 | 1.45e-02 |
|  | 6 | 50808627 | 50808813 | PDFSTATSENSEMBLUCSC | 187 | 4 | 34366 | gene;island | 0.440650 | 0.978798 | Not queried | 6.12 | 2.86e-02 | 6.12e-02 |
|  | 7 | 28319343 | 28319676 | PDFSTATSENSEMBLUCSC | 334 | 6 | 37504 | tss | 0.105150 | 0.923947 | AC005017.2 (margin: AC005017.2) | 6.12 | 4.33e-03 | 2.52e-02 |
|  | 1 | 204120607 | 204120951 | PDFSTATSENSEMBLUCSC | 345 | 5 | 956 | gene;island | 0.863620 | 0.021578 | ETNK2 (margin: RP11-74C13.3; ETNK2; REN) | 6.12 | 7.94e-03 | 3.19e-02 |
|  | 4 | 7194841 | 7195206 | PDFSTATSENSEMBLUCSC | 366 | 5 | 29935 | gene;island | 0.002403 | 0.193058 | SORCS2 (margin: SORCS2) | 6.12 | 7.94e-03 | 3.19e-02 |
|  | 3 | 193776047 | 193776240 | PDFSTATSENSEMBLUCSC | 194 | 4 | 29780 | island | 0.402069 | 0.970359 | Not queried | 6.12 | 2.86e-02 | 6.12e-02 |
|  | 6 | 31744339 | 31744962 | PDFSTATSENSEMBLUCSC | 624 | 14 | 33954 | gene | 0.010125 | 0.615516 | VWA7; VARS; Y\_RNA (margin: MSH5; MSH5-SAPCD1; SAPCD1; SAPCD1-AS1; VWA7; VARS; Y\_RNA) | 6.11 | 7.94e-04 | 8.28e-03 |
|  | 14 | 89878562 | 89878733 | PDFSTATSENSEMBLUCSC | 172 | 6 | 12170 | gene;island | 0.163694 | 0.923881 | FOXN3; RP11-33N16.3 (margin: RP11-33N16.2; FOXN3-AS1; FOXN3; RP11-33N16.3) | 6.11 | 2.16e-03 | 1.45e-02 |
|  | 11 | 12398874 | 12399176 | PDFSTATSENSEMBLUCSC | 303 | 8 | 8066 | island | 0.012764 | 0.470699 | Not queried | 6.11 | 4.99e-02 | 9.81e-02 |
|  | 1 | 161697052 | 161697281 | PDFSTATSENSEMBLUCSC | 230 | 4 | 855 | gene;island | 0.005641 | 0.435775 | Not queried | 6.11 | 2.86e-02 | 6.12e-02 |
|  | 8 | 17013409 | 17013756 | PDFSTATSENSEMBLUCSC | 348 | 4 | 39564 | tss;island | 0.561060 | 0.022220 | Not queried | 6.11 | 2.86e-02 | 6.12e-02 |
|  | 7 | 27207996 | 27208285 | PDFSTATSENSEMBLUCSC | 290 | 4 | 38357 | island | 0.232640 | 0.945094 | Not queried | 6.11 | 2.86e-02 | 6.12e-02 |
|  | 4 | 118006747 | 118006832 | PDFSTATSENSEMBLUCSC | 86 | 6 | 30559 | tss | 0.518175 | 0.022554 | TRAM1L1 (margin: TRAM1L1) | 6.10 | 2.16e-03 | 1.45e-02 |
|  | 12 | 16758692 | 16759029 | PDFSTATSENSEMBLUCSC | 338 | 6 | 9072 | gene | 0.637440 | 0.049020 | MGST1; LMO3 (margin: MGST1; LMO3) | 6.10 | 2.16e-03 | 1.45e-02 |
|  | 16 | 58533743 | 58534157 | PDFSTATSENSEMBLUCSC | 415 | 4 | 14773 | gene;island | 0.745341 | 0.090821 | Not queried | 6.10 | 5.71e-02 | 1.00e-01 |
|  | 8 | 119963842 | 119964248 | PDFSTATSENSEMBLUCSC | 407 | 5 | 39406 | gene | 0.373087 | 0.002835 | Not queried | 6.10 | 1.59e-02 | 5.36e-02 |
|  | 2 | 27805536 | 27805844 | PDFSTATSENSEMBLUCSC | 309 | 7 | 23273 | tss | 0.012305 | 0.422875 | Not queried | 6.10 | 1.75e-02 | 5.79e-02 |
|  | 1 | 240776162 | 240776316 | PDFSTATSENSEMBLUCSC | 155 | 4 | 2348 | tss | 0.411614 | 0.978334 | Not queried | 6.10 | 5.71e-02 | 1.00e-01 |
|  | 2 | 38300537 | 38300885 | PDFSTATSENSEMBLUCSC | 349 | 4 | 22650 | gene;island | 0.577575 | 0.039078 | Not queried | 6.10 | 2.86e-02 | 6.12e-02 |
|  | 6 | 157745216 | 157745382 | PDFSTATSENSEMBLUCSC | 167 | 4 | 36591 | island | 0.536428 | 0.019159 | Not queried | 6.09 | 2.86e-02 | 6.12e-02 |
|  | 11 | 4629597 | 4629912 | PDFSTATSENSEMBLUCSC | 316 | 6 | 7103 | tss | 0.058294 | 0.348002 | Not queried | 6.09 | 1.32e-01 | 1.89e-01 |
|  | 15 | 78631512 | 78631652 | PDFSTATSENSEMBLUCSC | 141 | 4 | 13833 | tss;island | 0.352001 | 0.952378 | Not queried | 6.09 | 2.86e-02 | 6.12e-02 |
|  | 1 | 9884565 | 9884732 | PDFSTATSENSEMBLUCSC | 168 | 8 | 1230 | tss;island | 0.183542 | 0.007948 | CLSTN1 (margin: CLSTN1) | 6.09 | 1.55e-04 | 2.71e-03 |
|  | 8 | 143851287 | 143851427 | PDFSTATSENSEMBLUCSC | 141 | 4 | 39467 | gene;tss | 0.046552 | 0.804198 | Not queried | 6.09 | 2.86e-02 | 6.12e-02 |
|  | 13 | 98869706 | 98870144 | PDFSTATSENSEMBLUCSC | 439 | 5 | 11198 | gene | 0.396489 | 0.986032 | Not queried | 6.09 | 5.56e-02 | 1.00e-01 |
|  | 5 | 142065671 | 142065819 | PDFSTATSENSEMBLUCSC | 149 | 5 | 32398 | tss | 0.031688 | 0.508175 | FGF1 (margin: FGF1) | 6.09 | 7.94e-03 | 3.19e-02 |
|  | 16 | 68482518 | 68483194 | PDFSTATSENSEMBLUCSC | 677 | 9 | 15322 | tss;island | 0.870023 | 0.039648 | SMPD3 (margin: SMPD3) | 6.09 | 8.23e-05 | 2.06e-03 |
|  | 17 | 41791038 | 41791475 | PDFSTATSENSEMBLUCSC | 438 | 5 | 18374 | island | 0.502205 | 0.014899 | (margin: CTC-501O10.1; RP11-209M4.1) | 6.09 | 7.94e-03 | 3.19e-02 |
|  | 18 | 19752292 | 19752414 | PDFSTATSENSEMBLUCSC | 123 | 4 | 19068 | gene;island | 0.489614 | 0.984977 | Not queried | 6.08 | 2.86e-02 | 6.12e-02 |
|  | 10 | 44101496 | 44102227 | PDFSTATSENSEMBLUCSC | 732 | 12 | 5701 | island | 0.575818 | 0.016390 | Not queried | 6.08 | 6.84e-02 | 1.17e-01 |
|  | 6 | 111408745 | 111408765 | PDFSTATSENSEMBLUCSC | 21 | 5 | 35175 | tss | 0.467771 | 0.013681 | SLC16A10 (margin: SLC16A10; RNU6-960P) | 6.08 | 7.94e-03 | 3.19e-02 |
|  | 19 | 18717638 | 18717783 | PDFSTATSENSEMBLUCSC | 146 | 6 | 21787 | island | 0.084521 | 0.002036 | Not queried | 6.08 | 1.52e-02 | 5.36e-02 |
|  | 8 | 11559039 | 11559488 | PDFSTATSENSEMBLUCSC | 450 | 4 | 40085 | island | 0.452733 | 0.977976 | Not queried | 6.08 | 2.00e-01 | 2.55e-01 |
|  | 3 | 190580589 | 190580644 | PDFSTATSENSEMBLUCSC | 56 | 4 | 28787 | tss | 0.369549 | 0.012015 | Not queried | 6.08 | 2.86e-02 | 6.12e-02 |
|  | 22 | 27053559 | 27053934 | PDFSTATSENSEMBLUCSC | 376 | 5 | 26826 | gene | 0.148714 | 0.988579 | MIAT (margin: MIAT; CTA-373H7.7) | 6.08 | 7.94e-03 | 3.19e-02 |
|  | 1 | 10056837 | 10057312 | PDFSTATSENSEMBLUCSC | 476 | 9 | 2662 | island | 0.474104 | 0.982024 | RBP7 (margin: NMNAT1; RBP7) | 6.08 | 4.11e-05 | 1.17e-03 |
|  | 7 | 123484898 | 123485052 | PDFSTATSENSEMBLUCSC | 155 | 4 | 37871 | tss | 0.229059 | 0.912302 | Not queried | 6.08 | 2.86e-02 | 6.12e-02 |
|  | 13 | 72441404 | 72441711 | PDFSTATSENSEMBLUCSC | 308 | 5 | 11472 | tss;island | 0.666538 | 0.046586 | DACH1 (margin: DACH1) | 6.08 | 7.94e-03 | 3.19e-02 |
|  | 5 | 57878745 | 57878919 | PDFSTATSENSEMBLUCSC | 175 | 5 | 32078 | tss | 0.563468 | 0.027230 | RAB3C (margin: RAB3C) | 6.08 | 7.94e-03 | 3.19e-02 |
|  | 11 | 70917159 | 70917533 | PDFSTATSENSEMBLUCSC | 375 | 4 | 6800 | gene | 0.945821 | 0.270310 | Not queried | 6.07 | 2.86e-02 | 6.12e-02 |
|  | 5 | 139927007 | 139927271 | PDFSTATSENSEMBLUCSC | 265 | 10 | 31785 | gene;island | 0.016379 | 0.474184 | ANKHD1; ANKHD1-EIF4EBP3; EIF4EBP3; SRA1 (margin: ANKHD1; ANKHD1-EIF4EBP3; EIF4EBP3; SRA1; APBB3) | 6.07 | 1.08e-05 | 4.82e-04 |
|  | 8 | 144660395 | 144660772 | PDFSTATSENSEMBLUCSC | 378 | 6 | 40673 | island | 0.546836 | 0.990304 | RP11-661A12.9; RP11-661A12.7; NAPRT1; EEF1D (margin: RP11-661A12.9; RP11-661A12.7; MROH6; NAPRT1; EEF1D) | 6.07 | 2.16e-03 | 1.45e-02 |
|  | 1 | 84326462 | 84326856 | PDFSTATSENSEMBLUCSC | 395 | 8 | 3406 | island | 0.506696 | 0.012575 | RP11-475O6.1 (margin: RP11-475O6.1; TTLL7) | 6.07 | 1.55e-04 | 2.71e-03 |
|  | 17 | 20811860 | 20812310 | PDFSTATSENSEMBLUCSC | 451 | 5 | 18113 | island | 0.011897 | 0.443634 | RP11-344E13.3 (margin: RP11-344E13.3; RNASEH1P1) | 6.07 | 7.94e-03 | 3.19e-02 |
|  | 6 | 152125861 | 152126441 | PDFSTATSENSEMBLUCSC | 581 | 8 | 34511 | gene;tss;island | 0.590549 | 0.991160 | ESR1 (margin: ESR1; RP3-443C4.2) | 6.07 | 1.55e-04 | 2.71e-03 |
|  | 17 | 42402738 | 42403152 | PDFSTATSENSEMBLUCSC | 415 | 4 | 17408 | tss;island | 0.015232 | 0.381163 | Not queried | 6.07 | 2.86e-02 | 6.12e-02 |
|  | 19 | 11877661 | 11877792 | PDFSTATSENSEMBLUCSC | 132 | 4 | 20481 | tss | 0.018002 | 0.535520 | Not queried | 6.07 | 2.86e-02 | 6.12e-02 |
|  | 7 | 1460673 | 1460944 | PDFSTATSENSEMBLUCSC | 272 | 4 | 38104 | island | 0.427314 | 0.984901 | Not queried | 6.06 | 2.86e-02 | 6.12e-02 |
|  | 6 | 26043546 | 26043990 | PDFSTATSENSEMBLUCSC | 445 | 5 | 35510 | island | 0.163920 | 0.936430 | U91328.2; HIST1H3C; HIST1H2BB (margin: U91328.2; HIST1H3C; HIST1H3B; HIST1H2AB; HIST1H2BB; HIST1H1C) | 6.06 | 7.94e-03 | 3.19e-02 |
|  | 3 | 138666118 | 138666473 | PDFSTATSENSEMBLUCSC | 356 | 4 | 27967 | gene | 0.512174 | 0.986453 | Not queried | 6.06 | 1.14e-01 | 1.68e-01 |
|  | 2 | 177001256 | 177001426 | PDFSTATSENSEMBLUCSC | 171 | 4 | 24906 | island | 0.423450 | 0.978561 | Not queried | 6.06 | 2.86e-02 | 6.12e-02 |
|  | 2 | 65593761 | 65594021 | PDFSTATSENSEMBLUCSC | 261 | 4 | 22709 | gene | 0.001281 | 0.076296 | Not queried | 6.06 | 5.71e-02 | 1.00e-01 |
|  | 7 | 123563992 | 123564379 | PDFSTATSENSEMBLUCSC | 388 | 4 | 37872 | tss | 0.061690 | 0.896077 | Not queried | 6.06 | 2.86e-02 | 6.12e-02 |
|  | 18 | 78004045 | 78004474 | PDFSTATSENSEMBLUCSC | 430 | 4 | 19147 | gene;island | 0.236407 | 0.965587 | Not queried | 6.06 | 2.00e-01 | 2.55e-01 |
|  | 10 | 54073047 | 54073642 | PDFSTATSENSEMBLUCSC | 596 | 8 | 5086 | tss;island | 0.356181 | 0.013192 | DKK1; PRKG1-AS1 (margin: DKK1; PRKG1-AS1) | 6.06 | 1.55e-04 | 2.71e-03 |
|  | 6 | 146864752 | 146864885 | PDFSTATSENSEMBLUCSC | 134 | 4 | 36544 | island | 0.025329 | 0.508396 | Not queried | 6.06 | 2.86e-02 | 6.12e-02 |
|  | 17 | 75524913 | 75525151 | PDFSTATSENSEMBLUCSC | 239 | 4 | 18811 | island | 0.289939 | 0.931643 | Not queried | 6.05 | 2.86e-02 | 6.12e-02 |
|  | 6 | 111408745 | 111408840 | PDFSTATSENSEMBLUCSC | 96 | 6 | 36418 | island | 0.470901 | 0.013862 | SLC16A10 (margin: SLC16A10; RNU6-960P) | 6.05 | 2.16e-03 | 1.45e-02 |
|  | 14 | 37051863 | 37051949 | PDFSTATSENSEMBLUCSC | 87 | 4 | 12359 | tss | 0.044710 | 0.529740 | Not queried | 6.05 | 2.86e-02 | 6.12e-02 |
|  | 6 | 5085404 | 5085713 | PDFSTATSENSEMBLUCSC | 310 | 5 | 34608 | tss;island | 0.016225 | 0.530779 | PPP1R3G (margin: PPP1R3G) | 6.04 | 7.94e-03 | 3.19e-02 |
|  | 6 | 39016262 | 39016776 | PDFSTATSENSEMBLUCSC | 515 | 7 | 36105 | island | 0.471043 | 0.961741 | GLP1R (margin: GLP1R; RP1-202I21.3) | 6.04 | 2.33e-03 | 1.52e-02 |
|  | 17 | 6659164 | 6659562 | PDFSTATSENSEMBLUCSC | 399 | 7 | 16377 | gene | 0.182400 | 0.893293 | XAF1 (margin: RPL23AP73; XAF1; CTC-281F24.2) | 6.04 | 2.33e-03 | 1.52e-02 |
|  | 15 | 76351361 | 76351713 | PDFSTATSENSEMBLUCSC | 353 | 4 | 13820 | tss;island | 0.332340 | 0.020722 | Not queried | 6.04 | 2.86e-02 | 6.12e-02 |
|  | 2 | 33359198 | 33359688 | PDFSTATSENSEMBLUCSC | 491 | 6 | 22641 | gene | 0.109078 | 0.918688 | LTBP1 (margin: LTBP1) | 6.04 | 2.16e-03 | 1.45e-02 |
|  | 1 | 115632236 | 115632727 | PDFSTATSENSEMBLUCSC | 492 | 8 | 1857 | tss | 0.013920 | 0.513893 | TSPAN2 (margin: RP4-666F24.3; TSPAN2) | 6.04 | 1.55e-04 | 2.71e-03 |
|  | 8 | 89340069 | 89340480 | PDFSTATSENSEMBLUCSC | 412 | 5 | 39802 | tss;island | 0.690120 | 0.068353 | RP11-586K2.1; MMP16 (margin: RP11-586K2.1; MMP16) | 6.04 | 7.94e-03 | 3.19e-02 |
|  | 4 | 142054254 | 142054446 | PDFSTATSENSEMBLUCSC | 193 | 4 | 30139 | gene;island | 0.512212 | 0.017767 | Not queried | 6.04 | 2.86e-02 | 6.12e-02 |
|  | 16 | 31538556 | 31538946 | PDFSTATSENSEMBLUCSC | 391 | 4 | 15214 | tss | 0.073613 | 0.813350 | Not queried | 6.03 | 5.71e-02 | 1.00e-01 |
|  | 11 | 1328419 | 1328622 | PDFSTATSENSEMBLUCSC | 204 | 4 | 6415 | gene;island | 0.193386 | 0.946824 | Not queried | 6.03 | 2.86e-02 | 6.12e-02 |
|  | 15 | 32321637 | 32321944 | PDFSTATSENSEMBLUCSC | 308 | 4 | 13551 | tss;island | 0.424776 | 0.027240 | Not queried | 6.03 | 1.14e-01 | 1.68e-01 |
|  | 1 | 3331080 | 3331278 | PDFSTATSENSEMBLUCSC | 199 | 4 | 124 | gene;island | 0.151769 | 0.943557 | Not queried | 6.02 | 2.86e-02 | 6.12e-02 |
|  | 11 | 35639492 | 35639686 | PDFSTATSENSEMBLUCSC | 195 | 6 | 7247 | tss;island | 0.018668 | 0.601576 | FJX1 (margin: FJX1) | 6.02 | 4.33e-03 | 2.52e-02 |
|  | 6 | 30124214 | 30124299 | PDFSTATSENSEMBLUCSC | 86 | 4 | 33731 | gene | 0.072611 | 0.832947 | Not queried | 6.02 | 2.86e-02 | 6.12e-02 |
|  | 17 | 16255560 | 16255884 | PDFSTATSENSEMBLUCSC | 325 | 4 | 16458 | gene;island | 0.037278 | 0.634985 | Not queried | 6.01 | 2.86e-02 | 6.12e-02 |
|  | 6 | 32165877 | 32165917 | PDFSTATSENSEMBLUCSC | 41 | 4 | 34084 | gene;island | 0.378217 | 0.908996 | Not queried | 6.01 | 5.71e-02 | 1.00e-01 |
|  | 12 | 133286734 | 133286970 | PDFSTATSENSEMBLUCSC | 237 | 4 | 10133 | tss;island | 0.279042 | 0.004724 | Not queried | 6.01 | 2.86e-02 | 6.12e-02 |
|  | 4 | 57547347 | 57547699 | PDFSTATSENSEMBLUCSC | 353 | 4 | 30011 | gene | 0.213734 | 0.005804 | Not queried | 6.01 | 5.71e-02 | 1.00e-01 |
|  | 5 | 88179235 | 88179539 | PDFSTATSENSEMBLUCSC | 305 | 6 | 31682 | gene;island | 0.331203 | 0.009086 | MEF2C-AS1; MEF2C (margin: MEF2C-AS1; MEF2C) | 6.01 | 2.16e-03 | 1.45e-02 |
|  | 14 | 54430382 | 54430831 | PDFSTATSENSEMBLUCSC | 450 | 4 | 12854 | island | 0.565653 | 0.042517 | Not queried | 6.01 | 3.43e-01 | 3.84e-01 |
|  | 1 | 8002302 | 8002698 | PDFSTATSENSEMBLUCSC | 397 | 5 | 2629 | island | 0.848814 | 0.068610 | RP5-892F13.2; TNFRSF9 (margin: RP5-892F13.2; PARK7; TNFRSF9) | 6.01 | 7.94e-03 | 3.19e-02 |
|  | 6 | 88875236 | 88875651 | PDFSTATSENSEMBLUCSC | 416 | 5 | 34413 | gene | 0.506050 | 0.073838 | CNR1 (margin: CNR1) | 6.01 | 7.94e-03 | 3.19e-02 |
|  | 5 | 114938148 | 114938459 | PDFSTATSENSEMBLUCSC | 312 | 5 | 32228 | tss | 0.488307 | 0.980304 | AC010226.4; TICAM2; TMED7-TICAM2 (margin: AC010226.4; TICAM2; TMED7-TICAM2; TMED7) | 6.01 | 7.94e-03 | 3.19e-02 |
|  | 8 | 143535437 | 143535891 | PDFSTATSENSEMBLUCSC | 455 | 5 | 40634 | island | 0.384072 | 0.973533 | BAI1 (margin: BAI1) | 6.00 | 7.94e-03 | 3.19e-02 |
|  | 3 | 44753865 | 44754587 | PDFSTATSENSEMBLUCSC | 723 | 12 | 29040 | island | 0.395013 | 0.976294 | ZNF502; SOCS5P3 (margin: ZNF502; SOCS5P3) | 6.00 | 6.56e-04 | 7.02e-03 |
|  | 13 | 28493913 | 28494177 | PDFSTATSENSEMBLUCSC | 265 | 8 | 11633 | island | 0.392318 | 0.943998 | PDX1; PDX1-AS1 (margin: PDX1; PDX1-AS1) | 6.00 | 1.55e-04 | 2.71e-03 |
|  | 13 | 28493913 | 28494161 | PDFSTATSENSEMBLUCSC | 249 | 6 | 11365 | tss | 0.487884 | 0.943998 | PDX1; PDX1-AS1 (margin: PDX1; PDX1-AS1) | 6.00 | 2.16e-03 | 1.45e-02 |
|  | 4 | 114681544 | 114681880 | PDFSTATSENSEMBLUCSC | 337 | 4 | 30105 | gene;island | 0.010410 | 0.513822 | Not queried | 6.00 | 2.86e-02 | 6.12e-02 |
|  | 17 | 29886672 | 29886890 | PDFSTATSENSEMBLUCSC | 219 | 4 | 17248 | tss | 0.618761 | 0.025518 | Not queried | 6.00 | 2.86e-02 | 6.12e-02 |
|  | 6 | 33084420 | 33085063 | PDFSTATSENSEMBLUCSC | 644 | 20 | 34188 | gene;island | 0.090258 | 0.948950 | HLA-DPB2 (margin: HLA-DPB2; COL11A2P1) | 5.99 | 9.25e-07 | 8.75e-05 |
|  | 6 | 33226591 | 33227015 | PDFSTATSENSEMBLUCSC | 425 | 6 | 34234 | gene | 0.359641 | 0.939509 | Not queried | 5.99 | 2.60e-02 | 6.12e-02 |
|  | 10 | 119293800 | 119294332 | PDFSTATSENSEMBLUCSC | 533 | 5 | 4782 | gene;island | 0.954787 | 0.340131 | Not queried | 5.99 | 1.59e-02 | 5.36e-02 |
|  | 20 | 656446 | 656743 | PDFSTATSENSEMBLUCSC | 298 | 4 | 25323 | gene | 0.537668 | 0.019821 | Not queried | 5.98 | 5.71e-02 | 1.00e-01 |
|  | 15 | 41952235 | 41952491 | PDFSTATSENSEMBLUCSC | 257 | 6 | 13609 | tss | 0.530867 | 0.018091 | Not queried | 5.98 | 6.49e-02 | 1.11e-01 |
|  | 6 | 33095664 | 33096501 | PDFSTATSENSEMBLUCSC | 838 | 9 | 34192 | gene | 0.021820 | 0.596136 | HLA-DPB2 (margin: HLA-DPB2; HLA-DPA3) | 5.98 | 2.76e-03 | 1.78e-02 |
|  | 17 | 19647973 | 19648414 | PDFSTATSENSEMBLUCSC | 442 | 4 | 16483 | gene;island | 0.461370 | 0.980792 | Not queried | 5.98 | 2.86e-02 | 6.12e-02 |
|  | 11 | 826837 | 827509 | PDFSTATSENSEMBLUCSC | 673 | 6 | 7031 | tss | 0.496828 | 0.018847 | PNPLA2; EFCAB4A; AP006621.8 (margin: PNPLA2; EFCAB4A; CD151; AP006621.8; POLR2L) | 5.98 | 2.16e-03 | 1.45e-02 |
|  | 12 | 6486598 | 6486709 | PDFSTATSENSEMBLUCSC | 112 | 4 | 9510 | tss | 0.218239 | 0.942218 | Not queried | 5.98 | 2.86e-02 | 6.12e-02 |
|  | 4 | 155664941 | 155665249 | PDFSTATSENSEMBLUCSC | 309 | 7 | 31387 | island | 0.895922 | 0.095788 | LRAT (margin: LRAT; RP11-21G20.3) | 5.98 | 5.83e-04 | 6.47e-03 |
|  | 6 | 27342376 | 27342738 | PDFSTATSENSEMBLUCSC | 363 | 5 | 33619 | gene;island | 0.008442 | 0.349678 | ZNF391 (margin: ZNF391; ZNF204P) | 5.98 | 7.94e-03 | 3.19e-02 |
|  | 19 | 13915583 | 13915877 | PDFSTATSENSEMBLUCSC | 295 | 6 | 19883 | gene;island | 0.977923 | 0.437804 | ZSWIM4 (margin: ZSWIM4; CTD-3252C9.2; RN7SL619P) | 5.98 | 2.16e-03 | 1.45e-02 |
|  | 10 | 49893336 | 49893463 | PDFSTATSENSEMBLUCSC | 128 | 6 | 5069 | tss | 0.312916 | 0.932955 | WDFY4 (margin: WDFY4) | 5.98 | 2.16e-03 | 1.45e-02 |
|  | 7 | 4872926 | 4873549 | PDFSTATSENSEMBLUCSC | 624 | 6 | 36860 | gene;island | 0.267219 | 0.958084 | RADIL (margin: RADIL) | 5.98 | 2.16e-03 | 1.45e-02 |
|  | 2 | 74010290 | 74010621 | PDFSTATSENSEMBLUCSC | 332 | 4 | 23445 | tss;island | 0.099337 | 0.877788 | Not queried | 5.97 | 1.14e-01 | 1.68e-01 |
|  | 11 | 72982929 | 72983097 | PDFSTATSENSEMBLUCSC | 169 | 4 | 6820 | gene;tss | 0.775901 | 0.177122 | Not queried | 5.97 | 5.71e-02 | 1.00e-01 |
|  | 7 | 157352978 | 157353191 | PDFSTATSENSEMBLUCSC | 214 | 5 | 37326 | gene;island | 0.062639 | 0.719889 | Not queried | 5.97 | 3.17e-02 | 6.50e-02 |
|  | 11 | 70963994 | 70964256 | PDFSTATSENSEMBLUCSC | 263 | 4 | 8617 | island | 0.505508 | 0.013492 | Not queried | 5.97 | 2.86e-02 | 6.12e-02 |
|  | 12 | 104609609 | 104610064 | PDFSTATSENSEMBLUCSC | 456 | 4 | 9271 | gene | 0.579520 | 0.987648 | Not queried | 5.97 | 2.86e-02 | 6.12e-02 |
|  | 19 | 51529846 | 51529999 | PDFSTATSENSEMBLUCSC | 154 | 4 | 20189 | gene | 0.020986 | 0.560118 | Not queried | 5.97 | 2.86e-02 | 6.12e-02 |
|  | 14 | 101335129 | 101335386 | PDFSTATSENSEMBLUCSC | 258 | 5 | 12609 | tss | 0.055990 | 0.822506 | Not queried | 5.97 | 5.56e-02 | 1.00e-01 |
|  | 3 | 36422059 | 36422406 | PDFSTATSENSEMBLUCSC | 348 | 7 | 28978 | island | 0.041633 | 0.619133 | STAC (margin: STAC) | 5.97 | 5.83e-04 | 6.47e-03 |
|  | 1 | 17445660 | 17446162 | PDFSTATSENSEMBLUCSC | 503 | 8 | 2757 | island | 0.436290 | 0.014641 | PADI2 (margin: PADI2) | 5.97 | 1.55e-04 | 2.71e-03 |
|  | 5 | 174178587 | 174178884 | PDFSTATSENSEMBLUCSC | 298 | 4 | 33418 | island | 0.591511 | 0.012195 | Not queried | 5.97 | 5.71e-02 | 1.00e-01 |
|  | 19 | 37663410 | 37663856 | PDFSTATSENSEMBLUCSC | 447 | 12 | 20734 | tss | 0.409742 | 0.010275 | CTC-454I21.3; ZNF585A (margin: CTC-454I21.3; ZNF585A; ZNF585B) | 5.97 | 7.40e-07 | 7.21e-05 |
|  | 11 | 65194933 | 65195039 | PDFSTATSENSEMBLUCSC | 107 | 4 | 8456 | island | 0.010905 | 0.383993 | Not queried | 5.97 | 2.86e-02 | 6.12e-02 |
|  | 14 | 94759646 | 94759873 | PDFSTATSENSEMBLUCSC | 228 | 4 | 12583 | tss | 0.012439 | 0.403823 | Not queried | 5.96 | 3.43e-01 | 3.84e-01 |
|  | 6 | 24911204 | 24911615 | PDFSTATSENSEMBLUCSC | 412 | 10 | 34672 | tss | 0.662098 | 0.055435 | FAM65B (margin: FAM65B) | 5.96 | 2.17e-05 | 8.94e-04 |
|  | 15 | 78556423 | 78556534 | PDFSTATSENSEMBLUCSC | 112 | 4 | 13831 | tss;island | 0.161088 | 0.913428 | Not queried | 5.96 | 2.86e-02 | 6.12e-02 |
|  | 10 | 43846281 | 43846705 | PDFSTATSENSEMBLUCSC | 425 | 5 | 5694 | island | 0.962561 | 0.331306 | Not queried | 5.96 | 3.17e-02 | 6.50e-02 |
|  | 19 | 57337294 | 57337648 | PDFSTATSENSEMBLUCSC | 355 | 4 | 20240 | gene;tss | 0.003586 | 0.084055 | Not queried | 5.96 | 1.14e-01 | 1.68e-01 |
|  | 1 | 248020632 | 248020812 | PDFSTATSENSEMBLUCSC | 181 | 5 | 1128 | gene | 0.471304 | 0.980558 | TRIM58 (margin: TRIM58; OR2W3) | 5.96 | 7.94e-03 | 3.19e-02 |
|  | 10 | 118030848 | 118031256 | PDFSTATSENSEMBLUCSC | 409 | 4 | 4772 | gene;island | 0.887982 | 0.060398 | Not queried | 5.96 | 2.86e-02 | 6.12e-02 |
|  | 3 | 123603306 | 123603618 | PDFSTATSENSEMBLUCSC | 313 | 6 | 28547 | tss;island | 0.108916 | 0.003896 | Not queried | 5.95 | 4.11e-02 | 8.14e-02 |
|  | 19 | 3481788 | 3481985 | PDFSTATSENSEMBLUCSC | 198 | 4 | 20343 | tss;island | 0.162037 | 0.901901 | Not queried | 5.95 | 2.86e-02 | 6.12e-02 |
|  | 1 | 47697948 | 47698126 | PDFSTATSENSEMBLUCSC | 179 | 4 | 3233 | island | 0.512384 | 0.979869 | Not queried | 5.95 | 2.86e-02 | 6.12e-02 |
|  | 19 | 1675848 | 1676136 | PDFSTATSENSEMBLUCSC | 289 | 4 | 21237 | island | 0.528646 | 0.018100 | Not queried | 5.95 | 2.86e-02 | 6.12e-02 |
|  | 12 | 89746328 | 89746771 | PDFSTATSENSEMBLUCSC | 444 | 8 | 9906 | tss | 0.002736 | 0.199021 | DUSP6 (margin: DUSP6) | 5.95 | 1.55e-04 | 2.71e-03 |
|  | 14 | 92980026 | 92980204 | PDFSTATSENSEMBLUCSC | 179 | 5 | 13089 | island | 0.018268 | 0.513562 | RIN3 (margin: RIN3) | 5.95 | 7.94e-03 | 3.19e-02 |
|  | 1 | 68299388 | 68299511 | PDFSTATSENSEMBLUCSC | 124 | 5 | 1685 | tss;island | 0.326230 | 0.007830 | Not queried | 5.95 | 1.59e-02 | 5.36e-02 |
|  | 10 | 29698152 | 29698470 | PDFSTATSENSEMBLUCSC | 319 | 8 | 5008 | tss;island | 0.008323 | 0.335828 | PTCHD3P1 (margin: PTCHD3P1) | 5.95 | 6.99e-03 | 3.19e-02 |
|  | 2 | 207139066 | 207139471 | PDFSTATSENSEMBLUCSC | 406 | 9 | 23835 | tss;island | 0.010014 | 0.425622 | ZDBF2 (margin: ZDBF2) | 5.94 | 4.11e-05 | 1.17e-03 |
|  | 3 | 113251979 | 113252350 | PDFSTATSENSEMBLUCSC | 372 | 5 | 27898 | gene;island | 0.472188 | 0.017230 | SIDT1 (margin: SIDT1) | 5.94 | 7.94e-03 | 3.19e-02 |
|  | 10 | 124894943 | 124895500 | PDFSTATSENSEMBLUCSC | 558 | 9 | 5415 | tss | 0.516571 | 0.986226 | HMX3 (margin: HMX3) | 5.94 | 1.65e-04 | 2.80e-03 |
|  | 3 | 182971430 | 182971663 | PDFSTATSENSEMBLUCSC | 234 | 4 | 28050 | gene;island | 0.010491 | 0.393845 | Not queried | 5.94 | 2.86e-02 | 6.12e-02 |
|  | 2 | 121493807 | 121494043 | PDFSTATSENSEMBLUCSC | 237 | 5 | 24669 | island | 0.017600 | 0.451545 | GLI2 (margin: GLI2) | 5.94 | 7.94e-03 | 3.19e-02 |
|  | 11 | 79151719 | 79152112 | PDFSTATSENSEMBLUCSC | 394 | 7 | 7593 | tss | 0.630142 | 0.030519 | TENM4 (margin: TENM4) | 5.93 | 5.83e-04 | 6.47e-03 |
|  | 17 | 56232260 | 56232499 | PDFSTATSENSEMBLUCSC | 240 | 4 | 17532 | tss | 0.014022 | 0.682383 | Not queried | 5.93 | 2.86e-02 | 6.12e-02 |
|  | 3 | 61235829 | 61236016 | PDFSTATSENSEMBLUCSC | 188 | 4 | 27844 | gene;island | 0.654623 | 0.041877 | Not queried | 5.93 | 2.86e-02 | 6.12e-02 |
|  | 4 | 147558089 | 147558544 | PDFSTATSENSEMBLUCSC | 456 | 4 | 31351 | island | 0.925126 | 0.127419 | Not queried | 5.93 | 2.00e-01 | 2.55e-01 |
|  | 15 | 93199037 | 93199180 | PDFSTATSENSEMBLUCSC | 144 | 5 | 13912 | tss | 0.409325 | 0.015216 | FAM174B (margin: FAM174B) | 5.93 | 7.94e-03 | 3.19e-02 |
|  | 13 | 35515865 | 35516223 | PDFSTATSENSEMBLUCSC | 359 | 4 | 11389 | tss | 0.279878 | 0.005842 | Not queried | 5.93 | 2.86e-02 | 6.12e-02 |
|  | 2 | 149634369 | 149634726 | PDFSTATSENSEMBLUCSC | 358 | 5 | 22894 | gene;island | 0.518422 | 0.017532 | KIF5C; AC105402.4 (margin: KIF5C; AC105402.4; AC105402.2; AC105402.3) | 5.93 | 7.94e-03 | 3.19e-02 |
|  | 2 | 182521874 | 182521926 | PDFSTATSENSEMBLUCSC | 53 | 6 | 23762 | tss;island | 0.459963 | 0.016813 | CERKL (margin: CERKL) | 5.93 | 2.16e-03 | 1.45e-02 |
|  | 17 | 47840643 | 47841033 | PDFSTATSENSEMBLUCSC | 391 | 5 | 16675 | gene;island | 0.324220 | 0.003879 | Not queried | 5.92 | 1.59e-02 | 5.36e-02 |
|  | 2 | 136875897 | 136876188 | PDFSTATSENSEMBLUCSC | 292 | 7 | 23649 | tss;island | 0.589683 | 0.008708 | CXCR4 (margin: CXCR4) | 5.92 | 5.83e-04 | 6.47e-03 |
|  | 5 | 43037123 | 43037666 | PDFSTATSENSEMBLUCSC | 544 | 6 | 32766 | island | 0.509784 | 0.984734 | CTD-2201E18.3; ANXA2R (margin: CTD-2035E11.4; AC025171.1; CTD-2201E18.3; ANXA2R) | 5.92 | 2.16e-03 | 1.45e-02 |
|  | 4 | 10459128 | 10459435 | PDFSTATSENSEMBLUCSC | 308 | 9 | 30300 | tss;island | 0.209543 | 0.915958 | ZNF518B (margin: ZNF518B) | 5.92 | 4.11e-05 | 1.17e-03 |
|  | 17 | 7486551 | 7486961 | PDFSTATSENSEMBLUCSC | 411 | 9 | 17090 | tss | 0.826787 | 0.025556 | Not queried | 5.92 | 2.44e-02 | 6.12e-02 |
|  | 4 | 53617076 | 53617448 | PDFSTATSENSEMBLUCSC | 373 | 5 | 31045 | island | 0.311470 | 0.740989 | ERVMER34-1; AC104066.1 (margin: SNORA26; ERVMER34-1; AC104066.1) | 5.92 | 7.94e-03 | 3.19e-02 |
|  | 1 | 26737005 | 26737470 | PDFSTATSENSEMBLUCSC | 466 | 8 | 2895 | island | 0.279963 | 0.974743 | LIN28A (margin: LIN28A) | 5.91 | 1.55e-04 | 2.71e-03 |
|  | 2 | 10830636 | 10830764 | PDFSTATSENSEMBLUCSC | 129 | 5 | 23183 | tss;island | 0.932419 | 0.050049 | RN7SL832P; NOL10 (margin: RN7SL832P; NOL10) | 5.91 | 7.94e-03 | 3.19e-02 |
|  | 2 | 202097062 | 202097173 | PDFSTATSENSEMBLUCSC | 112 | 4 | 23816 | tss | 0.000832 | 0.038683 | Not queried | 5.91 | 2.86e-02 | 6.12e-02 |
|  | 7 | 157291868 | 157292182 | PDFSTATSENSEMBLUCSC | 315 | 4 | 39104 | island | 0.605827 | 0.979256 | Not queried | 5.91 | 2.86e-02 | 6.12e-02 |
|  | 17 | 2699021 | 2699718 | PDFSTATSENSEMBLUCSC | 698 | 10 | 17009 | tss | 0.308136 | 0.960232 | RAP1GAP2 (margin: RAP1GAP2) | 5.91 | 4.87e-04 | 6.47e-03 |
|  | 17 | 15244887 | 15245237 | PDFSTATSENSEMBLUCSC | 351 | 8 | 18040 | island | 0.435952 | 0.977951 | TEKT3 (margin: TEKT3) | 5.90 | 1.55e-04 | 2.71e-03 |
|  | 12 | 8995557 | 8995965 | PDFSTATSENSEMBLUCSC | 409 | 5 | 9050 | gene | 0.418807 | 0.975110 | Not queried | 5.90 | 9.52e-02 | 1.53e-01 |
|  | 7 | 29519297 | 29519450 | PDFSTATSENSEMBLUCSC | 154 | 5 | 37511 | tss | 0.181840 | 0.927205 | CHN2 (margin: CHN2) | 5.90 | 7.94e-03 | 3.19e-02 |
|  | 13 | 93879303 | 93879769 | PDFSTATSENSEMBLUCSC | 467 | 5 | 11190 | gene;island | 0.912796 | 0.087961 | GPC6 (margin: GPC6) | 5.90 | 7.94e-03 | 3.19e-02 |
|  | 2 | 161126295 | 161126857 | PDFSTATSENSEMBLUCSC | 563 | 7 | 24814 | island | 0.013273 | 0.445791 | ITGB6; RBMS1 (margin: ITGB6; RBMS1) | 5.90 | 5.83e-04 | 6.47e-03 |
|  | 6 | 29342522 | 29342824 | PDFSTATSENSEMBLUCSC | 303 | 8 | 33653 | gene | 0.035507 | 0.537811 | Not queried | 5.90 | 1.05e-01 | 1.66e-01 |
|  | 1 | 207627372 | 207627601 | PDFSTATSENSEMBLUCSC | 230 | 5 | 2222 | tss;island | 0.197666 | 0.005754 | Not queried | 5.90 | 1.59e-02 | 5.36e-02 |
|  | 14 | 101525784 | 101526140 | PDFSTATSENSEMBLUCSC | 357 | 6 | 12630 | tss | 0.072769 | 0.882319 | MIR154; MIR496 (margin: MIR381HG; MIR889; MIR544A; MIR655; AL132709.3; MIR487A; MIR134; MIR485; MIR323B; MIR154; MIR496; MIR377; MIR541; MIR409; MIR412; MIR369; MIR410; MIR656; MEG9; MIR382) | 5.90 | 2.16e-03 | 1.45e-02 |
|  | 19 | 11998457 | 11999148 | PDFSTATSENSEMBLUCSC | 692 | 14 | 21586 | island | 0.170670 | 0.937151 | ZNF69 (margin: ZNF439; ZNF69) | 5.90 | 4.99e-08 | 9.34e-06 |
|  | 6 | 39290342 | 39290877 | PDFSTATSENSEMBLUCSC | 536 | 7 | 34956 | tss | 0.026917 | 0.733122 | KCNK16 (margin: KCNK17; KCNK16; KIF6) | 5.89 | 4.08e-03 | 2.47e-02 |
|  | 12 | 26986758 | 26987164 | PDFSTATSENSEMBLUCSC | 407 | 11 | 9615 | tss;island | 0.808322 | 0.098829 | ITPR2 (margin: ITPR2) | 5.89 | 5.53e-04 | 6.47e-03 |
|  | 1 | 870791 | 871057 | PDFSTATSENSEMBLUCSC | 267 | 5 | 2 | gene;island | 0.255959 | 0.973119 | SAMD11 (margin: SAMD11; AL645608.1; NOC2L) | 5.89 | 7.94e-03 | 3.19e-02 |
|  | 4 | 141347993 | 141348737 | PDFSTATSENSEMBLUCSC | 745 | 9 | 30138 | gene | 0.334080 | 0.006771 | CLGN (margin: CLGN) | 5.89 | 3.99e-03 | 2.47e-02 |
|  | 12 | 5541220 | 5541270 | PDFSTATSENSEMBLUCSC | 51 | 4 | 9504 | tss | 0.062700 | 0.774116 | Not queried | 5.89 | 2.86e-02 | 6.12e-02 |
|  | 2 | 11485384 | 11485561 | PDFSTATSENSEMBLUCSC | 178 | 4 | 23189 | tss;island | 0.849425 | 0.098333 | Not queried | 5.89 | 2.86e-02 | 6.12e-02 |
|  | 18 | 37421319 | 37421988 | PDFSTATSENSEMBLUCSC | 670 | 5 | 19479 | island | 0.628367 | 0.028092 | RP11-636O21.1 (margin: RP11-636O21.1) | 5.89 | 7.94e-03 | 3.19e-02 |
|  | 7 | 94953956 | 94954202 | PDFSTATSENSEMBLUCSC | 247 | 4 | 37725 | tss | 0.459190 | 0.978775 | Not queried | 5.89 | 2.86e-02 | 6.12e-02 |
|  | 12 | 52545884 | 52546408 | PDFSTATSENSEMBLUCSC | 525 | 5 | 10436 | island | 0.028730 | 0.590420 | (margin: ) | 5.88 | 7.94e-03 | 3.19e-02 |
|  | 2 | 75426726 | 75427699 | PDFSTATSENSEMBLUCSC | 974 | 12 | 23468 | tss;island | 0.387107 | 0.013947 | TACR1 (margin: RP11-355F16.1; TACR1) | 5.88 | 7.40e-07 | 7.21e-05 |
|  | 22 | 44576869 | 44577240 | PDFSTATSENSEMBLUCSC | 372 | 8 | 27211 | tss | 0.226517 | 0.944394 | PARVG (margin: PARVB; PARVG) | 5.88 | 1.55e-04 | 2.71e-03 |
|  | 1 | 76262302 | 76262984 | PDFSTATSENSEMBLUCSC | 683 | 8 | 3390 | island | 0.384534 | 0.980276 | RABGGTB; MSH4 (margin: ACADM; RABGGTB; SNORD45C; SNORD45A; SNORD45B; MSH4) | 5.88 | 1.55e-04 | 2.71e-03 |
|  | 12 | 7260546 | 7260888 | PDFSTATSENSEMBLUCSC | 343 | 8 | 9044 | gene;tss | 0.019868 | 0.901568 | C1RL-AS1; C1RL (margin: C1RL-AS1; RNU6-485P; C1RL) | 5.88 | 1.55e-04 | 2.71e-03 |
|  | 2 | 113992762 | 113993313 | PDFSTATSENSEMBLUCSC | 552 | 8 | 22834 | gene;tss;island | 0.986977 | 0.563694 | AC016683.6; PAX8 (margin: AC016683.6; RP11-65I12.1; PAX8) | 5.88 | 1.55e-04 | 2.71e-03 |
|  | 12 | 12223661 | 12223989 | PDFSTATSENSEMBLUCSC | 329 | 4 | 9567 | tss | 0.083817 | 0.003581 | Not queried | 5.88 | 1.14e-01 | 1.68e-01 |
|  | 7 | 93204046 | 93204478 | PDFSTATSENSEMBLUCSC | 433 | 9 | 37713 | tss;island | 0.777357 | 0.075143 | CALCR (margin: CALCR) | 5.87 | 4.11e-05 | 1.17e-03 |
|  | 3 | 62304470 | 62304779 | PDFSTATSENSEMBLUCSC | 310 | 5 | 28424 | tss;island | 0.943505 | 0.239458 | C3orf14; PTPRG-AS1 (margin: C3orf14; PTPRG-AS1) | 5.87 | 7.94e-03 | 3.19e-02 |
|  | 20 | 2276435 | 2276571 | PDFSTATSENSEMBLUCSC | 137 | 4 | 25542 | tss | 0.303470 | 0.928396 | Not queried | 5.87 | 2.86e-02 | 6.12e-02 |
|  | 14 | 90168304 | 90168492 | PDFSTATSENSEMBLUCSC | 189 | 4 | 13067 | island | 0.914635 | 0.228948 | Not queried | 5.87 | 2.86e-02 | 6.12e-02 |
|  | 11 | 19736150 | 19736333 | PDFSTATSENSEMBLUCSC | 184 | 5 | 6535 | gene;tss;island | 0.292789 | 0.974156 | NAV2; RP11-359E10.1 (margin: NAV2; RP11-359E10.1) | 5.87 | 7.94e-03 | 3.19e-02 |
|  | 1 | 186649153 | 186649530 | PDFSTATSENSEMBLUCSC | 378 | 6 | 925 | gene | 0.157196 | 0.003652 | PTGS2 (margin: PTGS2) | 5.87 | 4.33e-03 | 2.52e-02 |
|  | 19 | 4531638 | 4531961 | PDFSTATSENSEMBLUCSC | 324 | 4 | 19735 | gene;island | 0.490238 | 0.970222 | Not queried | 5.87 | 2.86e-02 | 6.12e-02 |
|  | 11 | 4629034 | 4629912 | PDFSTATSENSEMBLUCSC | 879 | 10 | 8007 | island | 0.017314 | 0.378816 | TRIM68 (margin: TRIM68) | 5.87 | 8.93e-03 | 3.45e-02 |
|  | 11 | 4629034 | 4629432 | PDFSTATSENSEMBLUCSC | 399 | 4 | 6484 | gene | 0.017314 | 0.400915 | Not queried | 5.87 | 2.86e-02 | 6.12e-02 |
|  | 11 | 120008836 | 120009135 | PDFSTATSENSEMBLUCSC | 300 | 6 | 8888 | island | 0.046607 | 0.203284 | Not queried | 5.87 | 9.31e-02 | 1.53e-01 |
|  | 1 | 248020331 | 248020812 | PDFSTATSENSEMBLUCSC | 482 | 9 | 4421 | island | 0.465363 | 0.976431 | TRIM58 (margin: TRIM58; OR2W3) | 5.87 | 4.11e-05 | 1.17e-03 |
|  | 10 | 102289529 | 102289885 | PDFSTATSENSEMBLUCSC | 357 | 5 | 6027 | island | 0.013803 | 0.395162 | HIF1AN; SEC31B; NDUFB8 (margin: HIF1AN; SEC31B; NDUFB8) | 5.87 | 7.94e-03 | 3.19e-02 |
|  | 11 | 2884118 | 2884460 | PDFSTATSENSEMBLUCSC | 343 | 5 | 7975 | island | 0.549021 | 0.986063 | KCNQ1-AS1 (margin: KCNQ1DN; KCNQ1-AS1) | 5.87 | 7.94e-03 | 3.19e-02 |
|  | 6 | 29855401 | 29855636 | PDFSTATSENSEMBLUCSC | 236 | 9 | 33699 | gene | 0.008569 | 0.534975 | HLA-H; HCG4P7 (margin: HLA-H; HLA-T; HCG4P7) | 5.87 | 1.65e-04 | 2.80e-03 |
|  | 1 | 3689151 | 3689201 | PDFSTATSENSEMBLUCSC | 51 | 4 | 1189 | tss;island | 0.093615 | 0.003681 | Not queried | 5.86 | 5.71e-02 | 1.00e-01 |
|  | 1 | 32860290 | 32860685 | PDFSTATSENSEMBLUCSC | 396 | 4 | 1460 | tss;island | 0.344383 | 0.009219 | Not queried | 5.86 | 5.71e-02 | 1.00e-01 |
|  | 4 | 119809845 | 119809975 | PDFSTATSENSEMBLUCSC | 131 | 4 | 30564 | tss | 0.582340 | 0.026706 | Not queried | 5.86 | 2.86e-02 | 6.12e-02 |
|  | 17 | 21279561 | 21279874 | PDFSTATSENSEMBLUCSC | 314 | 6 | 18118 | island | 0.547441 | 0.984027 | KCNJ12 (margin: KCNJ12) | 5.86 | 2.16e-03 | 1.45e-02 |
|  | 5 | 180018495 | 180018735 | PDFSTATSENSEMBLUCSC | 241 | 6 | 32560 | tss | 0.371413 | 0.968255 | SCGB3A1 (margin: AC122714.1; SCGB3A1; FLT4) | 5.86 | 2.16e-03 | 1.45e-02 |
|  | 7 | 39649224 | 39649443 | PDFSTATSENSEMBLUCSC | 220 | 5 | 36974 | gene;island | 0.364593 | 0.970401 | YAE1D1; AC004837.3; AC004837.4 (margin: YAE1D1; AC004837.3; AC004837.4) | 5.85 | 7.94e-03 | 3.19e-02 |
|  | 5 | 100238655 | 100239071 | PDFSTATSENSEMBLUCSC | 417 | 8 | 32984 | island | 0.113773 | 0.005165 | ST8SIA4 (margin: ST8SIA4) | 5.85 | 1.55e-04 | 2.71e-03 |
|  | 5 | 100238655 | 100238983 | PDFSTATSENSEMBLUCSC | 329 | 4 | 31702 | gene | 0.078008 | 0.001844 | Not queried | 5.85 | 2.86e-02 | 6.12e-02 |
|  | 5 | 75379270 | 75379515 | PDFSTATSENSEMBLUCSC | 246 | 4 | 32888 | island | 0.666170 | 0.036585 | Not queried | 5.85 | 2.86e-02 | 6.12e-02 |
|  | 3 | 190580422 | 190580644 | PDFSTATSENSEMBLUCSC | 223 | 5 | 29770 | island | 0.397620 | 0.017517 | Not queried | 5.85 | 9.52e-02 | 1.53e-01 |
|  | 2 | 176968584 | 176968920 | PDFSTATSENSEMBLUCSC | 337 | 4 | 24895 | island | 0.168178 | 0.917030 | Not queried | 5.85 | 2.86e-02 | 6.12e-02 |
|  | 5 | 131592959 | 131593287 | PDFSTATSENSEMBLUCSC | 329 | 9 | 32268 | tss | 0.223401 | 0.950462 | PDLIM4; P4HA2 (margin: PDLIM4; P4HA2) | 5.85 | 4.94e-04 | 6.47e-03 |
|  | 6 | 30431563 | 30431860 | PDFSTATSENSEMBLUCSC | 298 | 15 | 35682 | island | 0.239452 | 0.945388 | (margin: TMPOP1; SUCLA2P1) | 5.85 | 1.29e-08 | 3.49e-06 |
|  | 18 | 24445134 | 24445318 | PDFSTATSENSEMBLUCSC | 185 | 4 | 19082 | gene;island | 0.024717 | 0.523455 | Not queried | 5.84 | 2.86e-02 | 6.12e-02 |
|  | 2 | 197457393 | 197457430 | PDFSTATSENSEMBLUCSC | 38 | 5 | 23794 | tss | 0.570079 | 0.018206 | HECW2 (margin: HECW2) | 5.84 | 7.94e-03 | 3.19e-02 |
|  | 12 | 54422127 | 54422488 | PDFSTATSENSEMBLUCSC | 362 | 5 | 9159 | gene;island | 0.891134 | 0.332585 | HOXC5; HOXC6; HOXC4 (margin: HOXC5; HOXC6; HOXC4; AC012531.25; MIR615) | 5.84 | 7.94e-03 | 3.19e-02 |
|  | 14 | 101509144 | 101509391 | PDFSTATSENSEMBLUCSC | 248 | 4 | 12626 | tss | 0.379991 | 0.967576 | Not queried | 5.84 | 2.86e-02 | 6.12e-02 |
|  | 2 | 179278332 | 179278855 | PDFSTATSENSEMBLUCSC | 524 | 6 | 22945 | gene;island | 0.013720 | 0.350643 | AC009948.5 (margin: RP11-65L3.1; AC009948.5) | 5.84 | 2.16e-03 | 1.45e-02 |
|  | 8 | 11059029 | 11059303 | PDFSTATSENSEMBLUCSC | 275 | 8 | 39549 | tss;island | 0.093767 | 0.703286 | AF131215.8; XKR6 (margin: AF131215.8; XKR6) | 5.84 | 6.99e-03 | 3.19e-02 |
|  | 16 | 86600309 | 86600499 | PDFSTATSENSEMBLUCSC | 191 | 4 | 15404 | tss;island | 0.310746 | 0.963963 | Not queried | 5.84 | 2.86e-02 | 6.12e-02 |
|  | 3 | 96533511 | 96534005 | PDFSTATSENSEMBLUCSC | 495 | 6 | 27871 | gene;island | 0.920773 | 0.252637 | EPHA6 (margin: EPHA6) | 5.84 | 2.16e-03 | 1.45e-02 |
|  | 3 | 55517496 | 55517853 | PDFSTATSENSEMBLUCSC | 358 | 4 | 27838 | gene;island | 0.501389 | 0.978696 | Not queried | 5.83 | 2.86e-02 | 6.12e-02 |
|  | 17 | 71161160 | 71161502 | PDFSTATSENSEMBLUCSC | 343 | 6 | 16745 | gene | 0.588200 | 0.020480 | RP11-143K11.5; SSTR2; POLR3KP2 (margin: RP11-143K11.5; SSTR2; RP11-143K11.1; POLR3KP2) | 5.83 | 2.16e-03 | 1.45e-02 |
|  | 11 | 10472001 | 10472161 | PDFSTATSENSEMBLUCSC | 161 | 7 | 7144 | tss;island | 0.026589 | 0.576345 | AMPD3 (margin: AMPD3) | 5.83 | 5.83e-04 | 6.47e-03 |
|  | 7 | 154684327 | 154684562 | PDFSTATSENSEMBLUCSC | 236 | 4 | 37304 | gene;island | 0.279060 | 0.972801 | Not queried | 5.83 | 5.71e-02 | 1.00e-01 |
|  | 5 | 140536175 | 140536496 | PDFSTATSENSEMBLUCSC | 322 | 4 | 31810 | gene;island | 0.524568 | 0.981968 | Not queried | 5.83 | 2.86e-02 | 6.12e-02 |
|  | 17 | 45772333 | 45772523 | PDFSTATSENSEMBLUCSC | 191 | 5 | 17443 | tss | 0.251027 | 0.972468 | TBKBP1 (margin: KPNB1; TBKBP1) | 5.83 | 7.94e-03 | 3.19e-02 |
|  | 13 | 36920644 | 36921174 | PDFSTATSENSEMBLUCSC | 531 | 13 | 11131 | gene;island | 0.117488 | 0.005981 | SPG20OS; SPG20 (margin: SPG20OS; AL139377.1; SPG20) | 5.83 | 1.91e-03 | 1.45e-02 |
|  | 14 | 89628141 | 89628422 | PDFSTATSENSEMBLUCSC | 282 | 4 | 12169 | gene;island | 0.440296 | 0.978890 | Not queried | 5.83 | 2.86e-02 | 6.12e-02 |
|  | 7 | 8301971 | 8302301 | PDFSTATSENSEMBLUCSC | 331 | 8 | 37438 | tss;island | 0.657986 | 0.040590 | AC007009.1; AC007128.1; ICA1 (margin: AC007009.1; AC007128.1; ICA1) | 5.83 | 1.55e-04 | 2.71e-03 |
|  | 2 | 191207820 | 191208232 | PDFSTATSENSEMBLUCSC | 413 | 5 | 24961 | island | 0.044663 | 0.842378 | Not queried | 5.83 | 9.52e-02 | 1.53e-01 |
|  | 8 | 144653855 | 144653975 | PDFSTATSENSEMBLUCSC | 121 | 4 | 39484 | gene;island | 0.424006 | 0.971171 | Not queried | 5.83 | 2.86e-02 | 6.12e-02 |
|  | 2 | 5836111 | 5836366 | PDFSTATSENSEMBLUCSC | 256 | 5 | 22558 | gene;island | 0.948911 | 0.287835 | SOX11; AC010729.1 (margin: SOX11; AC010729.1; AC107057.2; AC108025.2) | 5.83 | 7.94e-03 | 3.19e-02 |
|  | 10 | 124894943 | 124895886 | PDFSTATSENSEMBLUCSC | 944 | 13 | 6215 | island | 0.540449 | 0.984133 | HMX3 (margin: HMX3; HMX2) | 5.82 | 2.67e-05 | 1.07e-03 |
|  | 5 | 10307544 | 10307831 | PDFSTATSENSEMBLUCSC | 288 | 4 | 31560 | gene;island | 0.010647 | 0.368063 | Not queried | 5.82 | 2.86e-02 | 6.12e-02 |
|  | 17 | 7197775 | 7198087 | PDFSTATSENSEMBLUCSC | 313 | 9 | 17933 | island | 0.691478 | 0.008007 | YBX2 (margin: SLC2A4; RP1-4G17.2; YBX2) | 5.82 | 1.23e-03 | 1.18e-02 |
|  | 7 | 50849639 | 50849931 | PDFSTATSENSEMBLUCSC | 293 | 4 | 37015 | gene;island | 0.984045 | 0.567008 | Not queried | 5.82 | 2.86e-02 | 6.12e-02 |
|  | 2 | 27805536 | 27806117 | PDFSTATSENSEMBLUCSC | 582 | 11 | 24189 | island | 0.012305 | 0.379741 | C2orf16; ZNF512; RP11-158I13.2; AC074091.1 (margin: C2orf16; ZNF512; RP11-158I13.2; AC074091.1) | 5.82 | 3.94e-04 | 5.86e-03 |
|  | 6 | 83903419 | 83904091 | PDFSTATSENSEMBLUCSC | 673 | 8 | 34402 | gene;tss;island | 0.434121 | 0.011292 | RWDD2A; PGM3 (margin: RWDD2A; PGM3) | 5.82 | 1.04e-02 | 3.96e-02 |
|  | 17 | 7608697 | 7608859 | PDFSTATSENSEMBLUCSC | 163 | 4 | 16412 | gene;island | 0.480275 | 0.024733 | Not queried | 5.82 | 2.86e-02 | 6.12e-02 |
|  | 11 | 65405268 | 65405525 | PDFSTATSENSEMBLUCSC | 258 | 9 | 7437 | tss | 0.016034 | 0.521291 | PCNXL3; MIR4690; SIPA1 (margin: PCNXL3; MIR4690; SIPA1; MIR4489) | 5.82 | 3.99e-03 | 2.47e-02 |
|  | 11 | 62314820 | 62315119 | PDFSTATSENSEMBLUCSC | 300 | 4 | 7345 | tss;island | 0.039558 | 0.727139 | Not queried | 5.81 | 2.86e-02 | 6.12e-02 |
|  | 7 | 27187372 | 27187691 | PDFSTATSENSEMBLUCSC | 320 | 8 | 37493 | tss | 0.397123 | 0.977278 | HOXA-AS3; HOXA3; HOXA6 (margin: HOXA-AS3; RP1-170O19.21; HOXA3; HOXA5; HOXA6; HOXA7) | 5.81 | 1.55e-04 | 2.71e-03 |
|  | 3 | 44902933 | 44903730 | PDFSTATSENSEMBLUCSC | 798 | 13 | 29044 | island | 0.021293 | 0.490498 | KIF15; TMEM42; MIR564 (margin: KIF15; TMEM42; MIR564) | 5.81 | 1.92e-07 | 2.60e-05 |
|  | 3 | 49837348 | 49837689 | PDFSTATSENSEMBLUCSC | 342 | 4 | 28336 | tss;island | 0.275106 | 0.941116 | Not queried | 5.81 | 5.71e-02 | 1.00e-01 |
|  | 14 | 105147537 | 105147781 | PDFSTATSENSEMBLUCSC | 245 | 4 | 13213 | island | 0.048452 | 0.600620 | Not queried | 5.80 | 2.86e-02 | 6.12e-02 |
|  | 20 | 656446 | 656861 | PDFSTATSENSEMBLUCSC | 416 | 5 | 25927 | island | 0.456820 | 0.027914 | Not queried | 5.80 | 3.17e-02 | 6.50e-02 |
|  | 4 | 155664941 | 155665106 | PDFSTATSENSEMBLUCSC | 166 | 6 | 30647 | tss | 0.885266 | 0.107460 | LRAT (margin: LRAT; RP11-21G20.3) | 5.80 | 2.16e-03 | 1.45e-02 |
|  | 2 | 131513608 | 131513927 | PDFSTATSENSEMBLUCSC | 320 | 6 | 22867 | gene;island | 0.470951 | 0.029929 | AMER3; AC140481.8 (margin: AMER3; AC140481.8) | 5.80 | 2.16e-03 | 1.45e-02 |
|  | 15 | 99644973 | 99645231 | PDFSTATSENSEMBLUCSC | 259 | 7 | 13923 | tss;island | 0.014917 | 0.375982 | SYNM; RP11-654A16.3 (margin: SYNM; RP11-654A16.3) | 5.80 | 5.83e-04 | 6.47e-03 |
|  | 11 | 65547778 | 65548028 | PDFSTATSENSEMBLUCSC | 251 | 7 | 8478 | island | 0.016321 | 0.567325 | AP5B1; AP001266.1 (margin: RP11-770G2.2; OVOL1; AP5B1; AP001266.1; RP11-770G2.4) | 5.80 | 5.83e-04 | 6.47e-03 |
|  | 6 | 110736772 | 110737053 | PDFSTATSENSEMBLUCSC | 282 | 5 | 35169 | tss | 0.120860 | 0.883547 | DDO (margin: DDO; SLC22A16) | 5.80 | 7.94e-03 | 3.19e-02 |
|  | 6 | 31759728 | 31760825 | PDFSTATSENSEMBLUCSC | 1098 | 20 | 33957 | gene;island | 0.190099 | 0.941294 | VARS (margin: VARS; LSM2) | 5.80 | 2.00e-06 | 1.82e-04 |
|  | 20 | 36793608 | 36794002 | PDFSTATSENSEMBLUCSC | 395 | 12 | 26152 | island | 0.682876 | 0.025361 | TGM2 (margin: TGM2) | 5.79 | 7.40e-07 | 7.21e-05 |
|  | 9 | 135037134 | 135037343 | PDFSTATSENSEMBLUCSC | 210 | 4 | 40963 | island | 0.730549 | 0.070389 | Not queried | 5.79 | 2.86e-02 | 6.12e-02 |
|  | 17 | 1958851 | 1959132 | PDFSTATSENSEMBLUCSC | 282 | 6 | 16327 | gene;tss;island | 0.208448 | 0.962238 | HIC1 (margin: HIC1; RP11-667K14.3; MIR132; MIR212; SMG6) | 5.79 | 2.16e-03 | 1.45e-02 |
|  | 7 | 112726396 | 112726869 | PDFSTATSENSEMBLUCSC | 474 | 10 | 37191 | gene;tss | 0.365289 | 0.955694 | GPR85 (margin: GPR85) | 5.79 | 2.17e-05 | 8.94e-04 |
|  | 1 | 239550177 | 239550578 | PDFSTATSENSEMBLUCSC | 402 | 6 | 4371 | island | 0.780983 | 0.100971 | CHRM3 (margin: CHRM3) | 5.79 | 2.16e-03 | 1.45e-02 |
|  | 16 | 87894334 | 87894690 | PDFSTATSENSEMBLUCSC | 357 | 4 | 14893 | gene;island | 0.118183 | 0.737915 | Not queried | 5.79 | 5.71e-02 | 1.00e-01 |
|  | 14 | 33403060 | 33403226 | PDFSTATSENSEMBLUCSC | 167 | 4 | 12771 | island | 0.474474 | 0.019367 | Not queried | 5.78 | 2.86e-02 | 6.12e-02 |
|  | 4 | 148402399 | 148402899 | PDFSTATSENSEMBLUCSC | 501 | 5 | 30150 | gene;island | 0.499104 | 0.976841 | EDNRA (margin: EDNRA) | 5.78 | 7.94e-03 | 3.19e-02 |
|  | 2 | 152954970 | 152955206 | PDFSTATSENSEMBLUCSC | 237 | 4 | 22898 | gene;island | 0.513339 | 0.018133 | Not queried | 5.78 | 2.86e-02 | 6.12e-02 |
|  | 21 | 46424715 | 46424976 | PDFSTATSENSEMBLUCSC | 262 | 6 | 26591 | tss;island | 0.024753 | 0.572840 | LINC00162 (margin: LINC00163; AP001505.9; LINC00162) | 5.78 | 2.16e-03 | 1.45e-02 |
|  | 10 | 119292062 | 119292587 | PDFSTATSENSEMBLUCSC | 526 | 8 | 4780 | gene;island | 0.339663 | 0.959142 | Not queried | 5.77 | 2.07e-02 | 6.12e-02 |
|  | 6 | 32181764 | 32182326 | PDFSTATSENSEMBLUCSC | 563 | 6 | 34093 | gene | 0.066994 | 0.874716 | Not queried | 5.77 | 4.11e-02 | 8.14e-02 |
|  | 11 | 79151611 | 79152112 | PDFSTATSENSEMBLUCSC | 502 | 8 | 8697 | island | 0.568076 | 0.033910 | TENM4 (margin: TENM4) | 5.77 | 3.11e-04 | 4.77e-03 |
|  | 7 | 76026853 | 76027101 | PDFSTATSENSEMBLUCSC | 249 | 4 | 37056 | gene;island | 0.141843 | 0.007838 | Not queried | 5.77 | 2.86e-02 | 6.12e-02 |
|  | 17 | 37879654 | 37879858 | PDFSTATSENSEMBLUCSC | 205 | 4 | 16554 | gene;island | 0.977226 | 0.481837 | Not queried | 5.77 | 2.86e-02 | 6.12e-02 |
|  | 12 | 48206783 | 48207063 | PDFSTATSENSEMBLUCSC | 281 | 4 | 9106 | gene;island | 0.438575 | 0.977363 | Not queried | 5.76 | 2.86e-02 | 6.12e-02 |
|  | 14 | 104583632 | 104584279 | PDFSTATSENSEMBLUCSC | 648 | 6 | 13202 | island | 0.726304 | 0.035761 | MIR203 (margin: ASPG; MIR203) | 5.76 | 2.16e-03 | 1.45e-02 |
|  | 9 | 116861288 | 116861532 | PDFSTATSENSEMBLUCSC | 245 | 4 | 40932 | island | 0.858827 | 0.160326 | Not queried | 5.76 | 2.86e-02 | 6.12e-02 |
|  | 2 | 128180488 | 128180959 | PDFSTATSENSEMBLUCSC | 472 | 4 | 22857 | gene;island | 0.159367 | 0.880561 | Not queried | 5.76 | 1.14e-01 | 1.68e-01 |
|  | 4 | 5712581 | 5712884 | PDFSTATSENSEMBLUCSC | 304 | 6 | 30276 | tss | 0.031829 | 0.630970 | EVC; EVC2 (margin: EVC; EVC2) | 5.76 | 4.33e-03 | 2.52e-02 |
|  | 17 | 29886672 | 29887049 | PDFSTATSENSEMBLUCSC | 378 | 5 | 18188 | island | 0.612420 | 0.029355 | MIR193A (margin: MIR193A; AC003101.1; RNU6ATAC7P) | 5.76 | 7.94e-03 | 3.19e-02 |
|  | 11 | 57519282 | 57519659 | PDFSTATSENSEMBLUCSC | 378 | 8 | 7310 | tss;island | 0.834741 | 0.076773 | TMX2-CTNND1; RP11-691N7.6; CTNND1; BTBD18 (margin: TMX2; TMX2-CTNND1; C11orf31; RP11-691N7.6; CTNND1; BTBD18) | 5.76 | 6.99e-03 | 3.19e-02 |
|  | 10 | 131682251 | 131682509 | PDFSTATSENSEMBLUCSC | 259 | 5 | 4833 | gene | 0.012560 | 0.520478 | Not queried | 5.76 | 5.56e-02 | 1.00e-01 |
|  | 10 | 88730324 | 88730599 | PDFSTATSENSEMBLUCSC | 276 | 4 | 4664 | gene;island | 0.034869 | 0.687791 | Not queried | 5.76 | 2.86e-02 | 6.12e-02 |
|  | 15 | 72489416 | 72489828 | PDFSTATSENSEMBLUCSC | 413 | 4 | 13408 | gene;island | 0.329989 | 0.963135 | Not queried | 5.75 | 2.86e-02 | 6.12e-02 |
|  | 22 | 18593294 | 18593609 | PDFSTATSENSEMBLUCSC | 316 | 7 | 27284 | island | 0.513269 | 0.016596 | PEX26; TUBA8 (margin: PEX26; XXbac-B476C20.10; TUBA8; ARL2BPP10) | 5.75 | 5.83e-04 | 6.47e-03 |
|  | 11 | 12398874 | 12399109 | PDFSTATSENSEMBLUCSC | 236 | 7 | 7154 | tss | 0.016169 | 0.480489 | Not queried | 5.75 | 9.73e-02 | 1.55e-01 |
|  | 19 | 39998018 | 39998290 | PDFSTATSENSEMBLUCSC | 273 | 4 | 20028 | gene;island | 0.436178 | 0.966214 | Not queried | 5.75 | 2.86e-02 | 6.12e-02 |
|  | 19 | 409357 | 409679 | PDFSTATSENSEMBLUCSC | 323 | 4 | 20271 | tss;island | 0.221751 | 0.009017 | Not queried | 5.75 | 2.86e-02 | 6.12e-02 |
|  | 11 | 132582488 | 132582808 | PDFSTATSENSEMBLUCSC | 321 | 4 | 6980 | gene | 0.896249 | 0.159714 | Not queried | 5.75 | 2.00e-01 | 2.55e-01 |
|  | 6 | 30509366 | 30509642 | PDFSTATSENSEMBLUCSC | 277 | 9 | 33772 | gene | 0.029676 | 0.008681 | Not queried | 5.75 | 9.39e-02 | 1.53e-01 |
|  | 18 | 47792708 | 47793175 | PDFSTATSENSEMBLUCSC | 468 | 8 | 19508 | island | 0.281794 | 0.019063 | CCDC11; MBD1 (margin: CCDC11; MBD1) | 5.75 | 1.55e-04 | 2.71e-03 |
|  | 16 | 1110844 | 1111220 | PDFSTATSENSEMBLUCSC | 377 | 4 | 15512 | island | 0.246250 | 0.929266 | Not queried | 5.75 | 2.86e-02 | 6.12e-02 |
|  | 10 | 35930436 | 35930534 | PDFSTATSENSEMBLUCSC | 99 | 6 | 5029 | tss;island | 0.364800 | 0.008761 | FZD8; MIR4683 (margin: FZD8; MIR4683) | 5.74 | 2.16e-03 | 1.45e-02 |
|  | 2 | 27718173 | 27718498 | PDFSTATSENSEMBLUCSC | 326 | 5 | 23272 | tss;island | 0.466730 | 0.075524 | Not queried | 5.74 | 5.56e-02 | 1.00e-01 |
|  | 13 | 97646639 | 97646811 | PDFSTATSENSEMBLUCSC | 173 | 7 | 11506 | tss;island | 0.340892 | 0.013738 | OXGR1 (margin: LINC00359; OXGR1) | 5.74 | 5.83e-04 | 6.47e-03 |
|  | 7 | 31232532 | 31232861 | PDFSTATSENSEMBLUCSC | 330 | 6 | 38391 | island | 0.469134 | 0.008210 | (margin: ) | 5.74 | 2.16e-03 | 1.45e-02 |
|  | 22 | 44576869 | 44577265 | PDFSTATSENSEMBLUCSC | 397 | 9 | 26891 | gene | 0.246176 | 0.945574 | PARVG (margin: PARVB; PARVG) | 5.74 | 1.65e-04 | 2.80e-03 |
|  | 4 | 21305805 | 21306051 | PDFSTATSENSEMBLUCSC | 247 | 4 | 29965 | gene;tss | 0.775586 | 0.062325 | Not queried | 5.74 | 3.43e-01 | 3.84e-01 |
|  | 6 | 151646540 | 151647133 | PDFSTATSENSEMBLUCSC | 594 | 9 | 34509 | gene;island | 0.483203 | 0.020852 | AKAP12 (margin: AKAP12; RN7SKP268) | 5.74 | 2.76e-03 | 1.78e-02 |
|  | 2 | 74725040 | 74725369 | PDFSTATSENSEMBLUCSC | 330 | 4 | 22743 | gene;island | 0.477308 | 0.976776 | Not queried | 5.73 | 2.86e-02 | 6.12e-02 |
|  | 13 | 39260309 | 39260633 | PDFSTATSENSEMBLUCSC | 325 | 4 | 11402 | tss;island | 0.387894 | 0.007001 | Not queried | 5.73 | 5.71e-02 | 1.00e-01 |
|  | 2 | 119532041 | 119532574 | PDFSTATSENSEMBLUCSC | 534 | 7 | 24643 | island | 0.008394 | 0.296952 | (margin: ) | 5.73 | 1.17e-03 | 1.13e-02 |
|  | 16 | 433439 | 433825 | PDFSTATSENSEMBLUCSC | 387 | 5 | 14530 | gene;island | 0.563754 | 0.023778 | Z97634.5; TMEM8A (margin: Z97634.5; Z97634.3; TMEM8A) | 5.73 | 7.94e-03 | 3.19e-02 |
|  | 1 | 247712110 | 247712383 | PDFSTATSENSEMBLUCSC | 274 | 6 | 2370 | tss | 0.212938 | 0.737641 | Not queried | 5.73 | 4.11e-02 | 8.14e-02 |
|  | 11 | 2542384 | 2542862 | PDFSTATSENSEMBLUCSC | 479 | 4 | 6461 | gene | 0.445225 | 0.967720 | Not queried | 5.73 | 2.86e-02 | 6.12e-02 |
|  | 13 | 61988802 | 61989203 | PDFSTATSENSEMBLUCSC | 402 | 4 | 11174 | gene | 0.605488 | 0.044499 | Not queried | 5.73 | 5.71e-02 | 1.00e-01 |
|  | 11 | 1431735 | 1432205 | PDFSTATSENSEMBLUCSC | 471 | 4 | 6418 | gene;island | 0.062127 | 0.870609 | Not queried | 5.73 | 1.14e-01 | 1.68e-01 |
|  | 6 | 31799311 | 31799575 | PDFSTATSENSEMBLUCSC | 265 | 4 | 35849 | island | 0.239903 | 0.862844 | Not queried | 5.72 | 2.86e-02 | 6.12e-02 |
|  | 4 | 110223830 | 110224288 | PDFSTATSENSEMBLUCSC | 459 | 10 | 30542 | tss | 0.765990 | 0.059767 | AC004051.2; COL25A1 (margin: AC004051.2; COL25A1) | 5.72 | 1.50e-03 | 1.41e-02 |
|  | 6 | 108487063 | 108487183 | PDFSTATSENSEMBLUCSC | 121 | 6 | 35158 | tss;island | 0.381778 | 0.969383 | NR2E1; OSTM1 (margin: OSTM1-AS1; NR2E1; OSTM1) | 5.72 | 2.16e-03 | 1.45e-02 |
|  | 20 | 43029468 | 43029895 | PDFSTATSENSEMBLUCSC | 428 | 5 | 25759 | tss | 0.534753 | 0.889968 | HNF4A (margin: HNF4A; MIR3646; RP5-881L22.4; RP5-881L22.6) | 5.72 | 7.94e-03 | 3.19e-02 |
|  | 2 | 111875749 | 111876008 | PDFSTATSENSEMBLUCSC | 260 | 5 | 24611 | island | 0.242861 | 0.947520 | ACOXL; BCL2L11 (margin: ACOXL; BCL2L11; AC096670.3) | 5.72 | 7.94e-03 | 3.19e-02 |
|  | 1 | 49242359 | 49242934 | PDFSTATSENSEMBLUCSC | 576 | 8 | 520 | gene;island | 0.786971 | 0.053277 | AGBL4; BEND5 (margin: AGBL4; BEND5) | 5.72 | 2.95e-03 | 1.88e-02 |
|  | 17 | 80797881 | 80798078 | PDFSTATSENSEMBLUCSC | 198 | 8 | 16959 | gene | 0.368091 | 0.012338 | TBCD; ZNF750 (margin: TBCD; ZNF750) | 5.72 | 1.55e-04 | 2.71e-03 |
|  | 11 | 61335071 | 61335269 | PDFSTATSENSEMBLUCSC | 199 | 4 | 6631 | gene;island | 0.652765 | 0.038131 | Not queried | 5.72 | 2.86e-02 | 6.12e-02 |
|  | 6 | 32546614 | 32546743 | PDFSTATSENSEMBLUCSC | 130 | 4 | 34119 | gene | 0.158457 | 0.903302 | Not queried | 5.71 | 2.00e-01 | 2.55e-01 |
|  | 11 | 67806337 | 67806668 | PDFSTATSENSEMBLUCSC | 332 | 8 | 8564 | island | 0.009860 | 0.412339 | TCIRG1 (margin: ALDH3B1; NDUFS8; MIR4691; TCIRG1; RP11-802E16.3; RP5-901A4.1) | 5.71 | 1.55e-04 | 2.71e-03 |
|  | 11 | 67806337 | 67806437 | PDFSTATSENSEMBLUCSC | 101 | 6 | 7505 | tss | 0.009860 | 0.373438 | TCIRG1 (margin: ALDH3B1; NDUFS8; MIR4691; TCIRG1; RP11-802E16.3; RP5-901A4.1) | 5.71 | 2.16e-03 | 1.45e-02 |
|  | 7 | 150549175 | 150549560 | PDFSTATSENSEMBLUCSC | 386 | 5 | 37983 | tss | 0.039005 | 0.586068 | AOC1 (margin: AOC1) | 5.71 | 7.94e-03 | 3.19e-02 |
|  | 1 | 24513865 | 24514013 | PDFSTATSENSEMBLUCSC | 149 | 4 | 1356 | tss | 0.016287 | 0.440810 | Not queried | 5.71 | 2.86e-02 | 6.12e-02 |
|  | 5 | 49962507 | 49962729 | PDFSTATSENSEMBLUCSC | 223 | 5 | 32056 | tss;island | 0.013067 | 0.563956 | Not queried | 5.71 | 5.56e-02 | 1.00e-01 |
|  | 10 | 81679675 | 81679852 | PDFSTATSENSEMBLUCSC | 178 | 4 | 5184 | tss | 0.286001 | 0.802290 | Not queried | 5.71 | 1.14e-01 | 1.68e-01 |
|  | 1 | 6684860 | 6685162 | PDFSTATSENSEMBLUCSC | 303 | 7 | 1214 | tss;island | 0.006178 | 0.244805 | Not queried | 5.71 | 1.28e-01 | 1.87e-01 |
|  | 3 | 136751342 | 136751795 | PDFSTATSENSEMBLUCSC | 454 | 5 | 29511 | island | 0.527658 | 0.070571 | Not queried | 5.71 | 2.22e-01 | 2.76e-01 |
|  | 22 | 38071001 | 38071188 | PDFSTATSENSEMBLUCSC | 188 | 4 | 27120 | tss;island | 0.015692 | 0.426827 | Not queried | 5.71 | 2.86e-02 | 6.12e-02 |
|  | 12 | 113587240 | 113587690 | PDFSTATSENSEMBLUCSC | 451 | 6 | 10827 | island | 0.103513 | 0.610108 | Not queried | 5.70 | 6.49e-02 | 1.11e-01 |
|  | 10 | 119304379 | 119304719 | PDFSTATSENSEMBLUCSC | 341 | 7 | 4786 | gene;island | 0.458746 | 0.984314 | EMX2; EMX2OS (margin: EMX2; EMX2OS) | 5.70 | 5.83e-04 | 6.47e-03 |
|  | 12 | 8123380 | 8123627 | PDFSTATSENSEMBLUCSC | 248 | 4 | 10230 | island | 0.108548 | 0.004715 | Not queried | 5.70 | 2.86e-02 | 6.12e-02 |
|  | 6 | 31237013 | 31237199 | PDFSTATSENSEMBLUCSC | 187 | 4 | 33864 | gene;island | 0.925879 | 0.117375 | Not queried | 5.70 | 5.71e-02 | 1.00e-01 |
|  | 3 | 53880717 | 53880872 | PDFSTATSENSEMBLUCSC | 156 | 4 | 27832 | gene;tss;island | 0.773893 | 0.117042 | Not queried | 5.70 | 2.86e-02 | 6.12e-02 |
|  | 15 | 60296138 | 60296469 | PDFSTATSENSEMBLUCSC | 332 | 6 | 14189 | island | 0.621428 | 0.027204 | FOXB1 (margin: FOXB1) | 5.70 | 2.16e-03 | 1.45e-02 |
|  | 15 | 90039409 | 90039794 | PDFSTATSENSEMBLUCSC | 386 | 4 | 13470 | gene | 0.573691 | 0.033523 | Not queried | 5.70 | 2.86e-02 | 6.12e-02 |
|  | 10 | 180188 | 180411 | PDFSTATSENSEMBLUCSC | 224 | 7 | 4909 | tss | 0.024241 | 0.007479 | Not queried | 5.70 | 9.73e-02 | 1.55e-01 |
|  | 19 | 42082102 | 42082485 | PDFSTATSENSEMBLUCSC | 384 | 4 | 20813 | tss | 0.008207 | 0.395838 | Not queried | 5.70 | 1.14e-01 | 1.68e-01 |
|  | 1 | 156338639 | 156338826 | PDFSTATSENSEMBLUCSC | 188 | 4 | 2015 | tss | 0.875347 | 0.159688 | Not queried | 5.69 | 2.86e-02 | 6.12e-02 |
|  | 6 | 100062488 | 100062855 | PDFSTATSENSEMBLUCSC | 368 | 5 | 34430 | gene;island | 0.356666 | 0.960362 | PRDM13 (margin: PRDM13) | 5.69 | 7.94e-03 | 3.19e-02 |
|  | 11 | 1037640 | 1037923 | PDFSTATSENSEMBLUCSC | 284 | 5 | 7037 | tss;island | 0.665481 | 0.976247 | MUC6 (margin: RP13-870H17.3; MUC6) | 5.69 | 7.94e-03 | 3.19e-02 |
|  | 19 | 12476027 | 12476577 | PDFSTATSENSEMBLUCSC | 551 | 8 | 21600 | island | 0.017804 | 0.437119 | ZNF442 (margin: CTD-3105H18.5; ZNF442) | 5.69 | 1.04e-02 | 3.96e-02 |
|  | 8 | 41166990 | 41167278 | PDFSTATSENSEMBLUCSC | 289 | 6 | 39667 | tss;island | 0.599309 | 0.986692 | SFRP1 (margin: RNU6-895P; SFRP1) | 5.69 | 2.16e-03 | 1.45e-02 |
|  | 13 | 113676726 | 113677325 | PDFSTATSENSEMBLUCSC | 600 | 6 | 11269 | gene;island | 0.331854 | 0.961549 | MCF2L (margin: MCF2L) | 5.68 | 2.16e-03 | 1.45e-02 |
|  | 1 | 60539362 | 60539675 | PDFSTATSENSEMBLUCSC | 314 | 6 | 3327 | island | 0.636488 | 0.982084 | C1orf87 (margin: C1orf87) | 5.68 | 4.33e-03 | 2.52e-02 |
|  | 1 | 60539559 | 60539675 | PDFSTATSENSEMBLUCSC | 117 | 4 | 1660 | tss | 0.636488 | 0.982084 | Not queried | 5.68 | 2.86e-02 | 6.12e-02 |
|  | 10 | 22624663 | 22625015 | PDFSTATSENSEMBLUCSC | 353 | 4 | 5601 | island | 0.634029 | 0.988619 | Not queried | 5.68 | 2.86e-02 | 6.12e-02 |
|  | 10 | 18549378 | 18549536 | PDFSTATSENSEMBLUCSC | 159 | 5 | 4973 | tss | 0.486116 | 0.975565 | Not queried | 5.68 | 5.56e-02 | 1.00e-01 |
|  | 6 | 149806235 | 149806732 | PDFSTATSENSEMBLUCSC | 498 | 7 | 35283 | tss | 0.163840 | 0.906720 | ZC3H12D (margin: ZC3H12D; RP1-281H8.3) | 5.68 | 1.11e-02 | 4.14e-02 |
|  | 7 | 149570987 | 149571373 | PDFSTATSENSEMBLUCSC | 387 | 11 | 37973 | tss | 0.503624 | 0.014069 | ATP6V0E2; ATP6V0E2-AS1 (margin: ZNF862; ATP6V0E2; ATP6V0E2-AS1) | 5.67 | 2.84e-06 | 1.96e-04 |
|  | 17 | 47073810 | 47073994 | PDFSTATSENSEMBLUCSC | 185 | 4 | 17479 | tss;island | 0.539664 | 0.019194 | Not queried | 5.67 | 2.86e-02 | 6.12e-02 |
|  | 6 | 119399925 | 119400114 | PDFSTATSENSEMBLUCSC | 190 | 4 | 34458 | gene;tss;island | 0.353625 | 0.013298 | Not queried | 5.67 | 2.86e-02 | 6.12e-02 |
|  | 1 | 34175169 | 34175343 | PDFSTATSENSEMBLUCSC | 175 | 4 | 407 | gene | 0.191228 | 0.690044 | Not queried | 5.67 | 2.86e-02 | 6.12e-02 |
|  | 15 | 48470205 | 48470973 | PDFSTATSENSEMBLUCSC | 769 | 10 | 14119 | island | 0.926747 | 0.025624 | MYEF2 (margin: MYEF2; RP11-605F22.2) | 5.67 | 3.89e-03 | 2.43e-02 |
|  | 1 | 151810887 | 151810904 | PDFSTATSENSEMBLUCSC | 18 | 4 | 755 | gene;tss;island | 0.538328 | 0.981965 | Not queried | 5.67 | 2.86e-02 | 6.12e-02 |
|  | 14 | 55034288 | 55034666 | PDFSTATSENSEMBLUCSC | 379 | 8 | 12859 | island | 0.004207 | 0.230822 | SAMD4A (margin: SAMD4A) | 5.67 | 1.55e-04 | 2.71e-03 |
|  | 5 | 175200401 | 175200576 | PDFSTATSENSEMBLUCSC | 176 | 4 | 33420 | island | 0.251219 | 0.921720 | Not queried | 5.67 | 2.86e-02 | 6.12e-02 |
|  | 10 | 33269417 | 33269812 | PDFSTATSENSEMBLUCSC | 396 | 5 | 5659 | island | 0.009779 | 0.248114 | Not queried | 5.67 | 1.59e-02 | 5.36e-02 |
|  | 5 | 142065539 | 142065819 | PDFSTATSENSEMBLUCSC | 281 | 6 | 31843 | gene | 0.036642 | 0.577866 | FGF1 (margin: FGF1) | 5.67 | 2.16e-03 | 1.45e-02 |
|  | 19 | 19976569 | 19976679 | PDFSTATSENSEMBLUCSC | 111 | 6 | 20654 | tss | 0.684678 | 0.020332 | ZNF253 (margin: CTC-559E9.12; ZNF253; CTC-559E9.10) | 5.67 | 2.16e-03 | 1.45e-02 |
|  | 2 | 176971820 | 176972068 | PDFSTATSENSEMBLUCSC | 249 | 7 | 23734 | tss | 0.017044 | 0.384279 | HOXD11; AC009336.1; HOXD10 (margin: HOXD13; HOXD12; HOXD11; AC009336.1; HOXD10) | 5.67 | 5.83e-04 | 6.47e-03 |
|  | 4 | 4861618 | 4862240 | PDFSTATSENSEMBLUCSC | 623 | 6 | 29918 | gene;island | 0.019370 | 0.672059 | Not queried | 5.67 | 1.52e-02 | 5.36e-02 |
|  | 6 | 166401464 | 166401700 | PDFSTATSENSEMBLUCSC | 237 | 6 | 34542 | gene;island | 0.294165 | 0.003217 | PDE10A; LINC00473 (margin: PDE10A; LINC00473) | 5.66 | 2.16e-03 | 1.45e-02 |
|  | 19 | 54392916 | 54393394 | PDFSTATSENSEMBLUCSC | 479 | 5 | 20210 | gene;island | 0.547210 | 0.022555 | Not queried | 5.66 | 1.59e-02 | 5.36e-02 |
|  | 1 | 201476290 | 201476371 | PDFSTATSENSEMBLUCSC | 82 | 5 | 939 | gene | 0.017048 | 0.405215 | RP11-134G8.7; CSRP1 (margin: RP11-134G8.7; RPS10P7; CSRP1) | 5.66 | 7.94e-03 | 3.19e-02 |
|  | 1 | 26737005 | 26737262 | PDFSTATSENSEMBLUCSC | 258 | 5 | 1392 | tss | 0.182421 | 0.972274 | LIN28A (margin: LIN28A) | 5.66 | 7.94e-03 | 3.19e-02 |
|  | 7 | 96634480 | 96634914 | PDFSTATSENSEMBLUCSC | 435 | 5 | 37102 | gene;tss;island | 0.203229 | 0.974410 | Not queried | 5.66 | 1.59e-02 | 5.36e-02 |
|  | 17 | 54671073 | 54671575 | PDFSTATSENSEMBLUCSC | 503 | 5 | 16702 | gene | 0.496751 | 0.018319 | NOG (margin: NOG) | 5.66 | 7.94e-03 | 3.19e-02 |
|  | 17 | 33416834 | 33417113 | PDFSTATSENSEMBLUCSC | 280 | 5 | 17264 | tss;island | 0.056238 | 0.841096 | RFFL; RAD51L3-RFFL (margin: RNU6-840P; RFFL; RAD51L3-RFFL; Vault; RAD51D) | 5.66 | 7.94e-03 | 3.19e-02 |
|  | 6 | 41215806 | 41216090 | PDFSTATSENSEMBLUCSC | 285 | 4 | 34968 | tss | 0.030590 | 0.642007 | Not queried | 5.66 | 2.86e-02 | 6.12e-02 |
|  | 19 | 40324746 | 40325128 | PDFSTATSENSEMBLUCSC | 383 | 11 | 22006 | island | 0.437254 | 0.010936 | DYRK1B; FBL (margin: DYRK1B; FBL) | 5.65 | 1.04e-02 | 3.96e-02 |
|  | 22 | 19511967 | 19512320 | PDFSTATSENSEMBLUCSC | 354 | 5 | 26787 | gene;island | 0.323388 | 0.972269 | Not queried | 5.65 | 1.59e-02 | 5.36e-02 |
|  | 11 | 57195025 | 57195540 | PDFSTATSENSEMBLUCSC | 516 | 5 | 7300 | tss;island | 0.265189 | 0.910227 | Not queried | 5.65 | 1.51e-01 | 2.11e-01 |
|  | 5 | 14405632 | 14406021 | PDFSTATSENSEMBLUCSC | 390 | 5 | 31563 | gene | 0.545394 | 0.985757 | TRIO (margin: TRIO) | 5.65 | 7.94e-03 | 3.19e-02 |
|  | 4 | 57976556 | 57976944 | PDFSTATSENSEMBLUCSC | 389 | 8 | 30416 | tss | 0.146426 | 0.649772 | RP11-12A1.1; IGFBP7 (margin: RP11-12A1.1; IGFBP7; snoU13) | 5.64 | 1.55e-04 | 2.71e-03 |
|  | 19 | 51522454 | 51522921 | PDFSTATSENSEMBLUCSC | 468 | 7 | 20188 | gene;island | 0.013525 | 0.205710 | KLK10 (margin: CTB-147C22.9; CTC-518B2.12; CTC-518B2.9; KLK9; KLK10; KLK11; KLK12) | 5.64 | 6.99e-03 | 3.19e-02 |
|  | 5 | 178801332 | 178801812 | PDFSTATSENSEMBLUCSC | 481 | 4 | 33484 | island | 0.105261 | 0.820180 | Not queried | 5.64 | 2.86e-02 | 6.12e-02 |
|  | 10 | 100996579 | 100997023 | PDFSTATSENSEMBLUCSC | 445 | 4 | 5279 | tss;island | 0.336953 | 0.980796 | Not queried | 5.64 | 2.86e-02 | 6.12e-02 |
|  | 16 | 49316845 | 49317169 | PDFSTATSENSEMBLUCSC | 325 | 4 | 15226 | tss | 0.712574 | 0.199761 | Not queried | 5.64 | 5.71e-02 | 1.00e-01 |
|  | 5 | 128300967 | 128301488 | PDFSTATSENSEMBLUCSC | 522 | 10 | 33072 | island | 0.358096 | 0.012953 | SLC27A6 (margin: SLC27A6) | 5.64 | 3.25e-04 | 4.91e-03 |
|  | 17 | 32907636 | 32907731 | PDFSTATSENSEMBLUCSC | 96 | 4 | 17261 | tss | 0.878494 | 0.260641 | Not queried | 5.64 | 1.14e-01 | 1.68e-01 |
|  | 16 | 82661421 | 82661725 | PDFSTATSENSEMBLUCSC | 305 | 4 | 14859 | gene;island | 0.012887 | 0.473833 | Not queried | 5.64 | 2.86e-02 | 6.12e-02 |
|  | 6 | 43211208 | 43211491 | PDFSTATSENSEMBLUCSC | 284 | 5 | 36158 | island | 0.800057 | 0.988217 | TTBK1 (margin: TTBK1) | 5.64 | 7.94e-03 | 3.19e-02 |
|  | 3 | 9993731 | 9994061 | PDFSTATSENSEMBLUCSC | 331 | 5 | 27657 | gene | 0.415501 | 0.008481 | PRRT3-AS1; PRRT3 (margin: CRELD1; PRRT3-AS1; RP11-1020A11.2; PRRT3; EMC3) | 5.64 | 7.94e-03 | 3.19e-02 |
|  | 16 | 10970801 | 10971250 | PDFSTATSENSEMBLUCSC | 450 | 5 | 15697 | island | 0.017922 | 0.413516 | CIITA; RP11-876N24.2 (margin: CIITA; RP11-876N24.2) | 5.64 | 7.94e-03 | 3.19e-02 |
|  | 1 | 25943790 | 25943891 | PDFSTATSENSEMBLUCSC | 102 | 5 | 1373 | tss;island | 0.491869 | 0.016764 | MAN1C1 (margin: MAN1C1) | 5.64 | 7.94e-03 | 3.19e-02 |
|  | 17 | 40715222 | 40715281 | PDFSTATSENSEMBLUCSC | 60 | 5 | 16580 | gene;island | 0.021112 | 0.518758 | RP11-400F19.8; COASY (margin: RP11-400F19.8; HSD17B1; COASY; MLX; RP11-400F19.6; PSMC3IP) | 5.63 | 7.94e-03 | 3.19e-02 |
|  | 22 | 45072455 | 45072724 | PDFSTATSENSEMBLUCSC | 270 | 4 | 26892 | gene | 0.054630 | 0.625437 | Not queried | 5.63 | 2.86e-02 | 6.12e-02 |
|  | 21 | 43638893 | 43639122 | PDFSTATSENSEMBLUCSC | 230 | 9 | 26437 | gene;tss;island | 0.475864 | 0.017986 | ABCG1 (margin: ABCG1; RNA5SP492) | 5.63 | 1.65e-04 | 2.80e-03 |
|  | 10 | 134859687 | 134860210 | PDFSTATSENSEMBLUCSC | 524 | 7 | 6329 | island | 0.312062 | 0.831134 | (margin: ) | 5.63 | 2.33e-03 | 1.52e-02 |
|  | 20 | 57765815 | 57766071 | PDFSTATSENSEMBLUCSC | 257 | 4 | 25859 | tss;island | 0.369925 | 0.937521 | Not queried | 5.63 | 2.86e-02 | 6.12e-02 |
|  | 4 | 90228685 | 90229039 | PDFSTATSENSEMBLUCSC | 355 | 5 | 30056 | gene;island | 0.623390 | 0.032931 | Not queried | 5.63 | 3.17e-02 | 6.50e-02 |
|  | 5 | 45696455 | 45696592 | PDFSTATSENSEMBLUCSC | 138 | 4 | 32054 | tss;island | 0.873910 | 0.144826 | Not queried | 5.62 | 2.86e-02 | 6.12e-02 |
|  | 5 | 150284302 | 150284796 | PDFSTATSENSEMBLUCSC | 495 | 9 | 33313 | island | 0.292191 | 0.900417 | ZNF300 (margin: IRGM; ZNF300) | 5.62 | 1.65e-04 | 2.80e-03 |
|  | 7 | 90225761 | 90226043 | PDFSTATSENSEMBLUCSC | 283 | 4 | 38649 | island | 0.425479 | 0.027778 | Not queried | 5.62 | 2.00e-01 | 2.55e-01 |
|  | 5 | 178322737 | 178322924 | PDFSTATSENSEMBLUCSC | 188 | 6 | 33475 | island | 0.015562 | 0.471193 | Not queried | 5.62 | 6.49e-02 | 1.11e-01 |
|  | 15 | 41952235 | 41952827 | PDFSTATSENSEMBLUCSC | 593 | 8 | 14066 | island | 0.523476 | 0.019538 | MGA (margin: MGA) | 5.62 | 1.04e-02 | 3.96e-02 |
|  | 1 | 202679089 | 202679191 | PDFSTATSENSEMBLUCSC | 103 | 4 | 947 | gene;island | 0.249768 | 0.949926 | Not queried | 5.62 | 2.86e-02 | 6.12e-02 |
|  | 2 | 16082022 | 16082215 | PDFSTATSENSEMBLUCSC | 194 | 4 | 23198 | tss | 0.452296 | 0.970907 | Not queried | 5.62 | 2.86e-02 | 6.12e-02 |
|  | 13 | 113698408 | 113698688 | PDFSTATSENSEMBLUCSC | 281 | 6 | 11270 | gene;island | 0.172284 | 0.822952 | MCF2L (margin: MCF2L) | 5.62 | 8.66e-03 | 3.36e-02 |
|  | 6 | 166418799 | 166419138 | PDFSTATSENSEMBLUCSC | 340 | 4 | 36624 | island | 0.230913 | 0.960537 | Not queried | 5.61 | 2.86e-02 | 6.12e-02 |
|  | 5 | 150284416 | 150284796 | PDFSTATSENSEMBLUCSC | 381 | 8 | 32435 | tss | 0.332093 | 0.924752 | ZNF300 (margin: IRGM; ZNF300) | 5.61 | 6.22e-04 | 6.71e-03 |
|  | 7 | 50800837 | 50801423 | PDFSTATSENSEMBLUCSC | 587 | 8 | 37014 | gene;tss | 0.158946 | 0.937695 | GRB10 (margin: GRB10) | 5.61 | 4.66e-03 | 2.68e-02 |
|  | 11 | 134176598 | 134176888 | PDFSTATSENSEMBLUCSC | 291 | 5 | 6996 | gene | 0.119438 | 0.929676 | Not queried | 5.61 | 1.51e-01 | 2.11e-01 |
|  | 8 | 103750821 | 103751006 | PDFSTATSENSEMBLUCSC | 186 | 4 | 40513 | island | 0.003746 | 0.137447 | Not queried | 5.61 | 2.86e-02 | 6.12e-02 |
|  | 19 | 407758 | 408233 | PDFSTATSENSEMBLUCSC | 476 | 4 | 19625 | gene;island | 0.531603 | 0.978525 | Not queried | 5.61 | 2.86e-02 | 6.12e-02 |
|  | 5 | 175792454 | 175792510 | PDFSTATSENSEMBLUCSC | 57 | 5 | 33425 | island | 0.530436 | 0.985608 | ARL10 (margin: RP11-843P14.2; RP11-843P14.1; ARL10; MIR1271; KIAA1191) | 5.60 | 7.94e-03 | 3.19e-02 |
|  | 10 | 103603563 | 103604019 | PDFSTATSENSEMBLUCSC | 457 | 7 | 6074 | island | 0.940615 | 0.215024 | KCNIP2; C10orf76 (margin: KCNIP2; C10orf76) | 5.60 | 5.83e-04 | 6.47e-03 |
|  | 19 | 7767075 | 7767348 | PDFSTATSENSEMBLUCSC | 274 | 5 | 20405 | tss | 0.311366 | 0.917696 | FCER2 (margin: FCER2) | 5.60 | 7.94e-03 | 3.19e-02 |
|  | 11 | 101000566 | 101000717 | PDFSTATSENSEMBLUCSC | 152 | 5 | 7642 | tss | 0.444469 | 0.015088 | PGR (margin: PGR) | 5.60 | 7.94e-03 | 3.19e-02 |
|  | 11 | 123524633 | 123525237 | PDFSTATSENSEMBLUCSC | 605 | 5 | 6951 | gene;island | 0.317182 | 0.958896 | Not queried | 5.59 | 3.17e-02 | 6.50e-02 |
|  | 6 | 50813551 | 50814089 | PDFSTATSENSEMBLUCSC | 539 | 5 | 34368 | gene;island | 0.206636 | 0.986261 | TFAP2B (margin: TFAP2B; RPS17P5) | 5.59 | 7.94e-03 | 3.19e-02 |
|  | 19 | 9545862 | 9546157 | PDFSTATSENSEMBLUCSC | 296 | 4 | 19801 | gene | 0.018919 | 0.330510 | Not queried | 5.59 | 2.86e-02 | 6.12e-02 |
|  | 4 | 154125208 | 154125594 | PDFSTATSENSEMBLUCSC | 387 | 6 | 30640 | tss | 0.878636 | 0.276944 | TRIM2 (margin: TRIM2) | 5.59 | 4.33e-03 | 2.52e-02 |
|  | 2 | 3699195 | 3699353 | PDFSTATSENSEMBLUCSC | 159 | 4 | 24039 | island | 0.675772 | 0.055262 | Not queried | 5.59 | 2.86e-02 | 6.12e-02 |
|  | 16 | 1227293 | 1227475 | PDFSTATSENSEMBLUCSC | 183 | 4 | 14559 | gene;island | 0.264430 | 0.948565 | Not queried | 5.59 | 2.86e-02 | 6.12e-02 |
|  | 20 | 30457834 | 30458267 | PDFSTATSENSEMBLUCSC | 434 | 9 | 25384 | gene;tss;island | 0.522422 | 0.020716 | TTLL9; DUSP15 (margin: TTLL9; DUSP15) | 5.59 | 4.11e-05 | 1.17e-03 |
|  | 15 | 86313834 | 86313973 | PDFSTATSENSEMBLUCSC | 140 | 7 | 13463 | gene;tss;island | 0.452417 | 0.978538 | RP11-158M2.6; KLHL25; MIR1276 (margin: RP11-158M2.6; KLHL25; MIR1276) | 5.59 | 5.83e-04 | 6.47e-03 |
|  | 6 | 118973021 | 118973353 | PDFSTATSENSEMBLUCSC | 333 | 8 | 35200 | tss | 0.529956 | 0.034077 | CEP85L (margin: CEP85L) | 5.59 | 6.99e-03 | 3.19e-02 |
|  | 3 | 43020564 | 43020952 | PDFSTATSENSEMBLUCSC | 389 | 6 | 29025 | island | 0.349274 | 0.023147 | Not queried | 5.58 | 6.49e-02 | 1.11e-01 |
|  | 8 | 144640306 | 144640403 | PDFSTATSENSEMBLUCSC | 98 | 6 | 39952 | tss | 0.032462 | 0.611651 | GSDMD (margin: GSDMD; RP11-661A12.5; MROH6) | 5.58 | 2.16e-03 | 1.45e-02 |
|  | 19 | 40324850 | 40325128 | PDFSTATSENSEMBLUCSC | 279 | 8 | 20778 | tss | 0.451214 | 0.015920 | Not queried | 5.58 | 1.05e-01 | 1.66e-01 |
|  | 6 | 32410690 | 32411185 | PDFSTATSENSEMBLUCSC | 496 | 6 | 34114 | gene | 0.031762 | 0.604327 | HLA-DRA (margin: HLA-DRA) | 5.58 | 2.16e-03 | 1.45e-02 |
|  | 6 | 33140660 | 33141305 | PDFSTATSENSEMBLUCSC | 646 | 9 | 34201 | gene | 0.029186 | 0.611682 | COL11A2 (margin: COL11A2) | 5.58 | 1.23e-03 | 1.18e-02 |
|  | 20 | 1206682 | 1206830 | PDFSTATSENSEMBLUCSC | 149 | 8 | 25934 | island | 0.232500 | 0.006555 | RAD21L1 (margin: RAD21L1) | 5.58 | 1.09e-03 | 1.10e-02 |
|  | 10 | 135090098 | 135090381 | PDFSTATSENSEMBLUCSC | 284 | 4 | 4894 | gene | 0.314781 | 0.958757 | Not queried | 5.58 | 2.86e-02 | 6.12e-02 |
|  | 11 | 2019436 | 2020560 | PDFSTATSENSEMBLUCSC | 1125 | 36 | 7055 | tss;island | 0.601164 | 0.031832 | H19 (margin: AC051649.6; MRPL23-AS1; H19) | 5.57 | 4.70e-13 | 9.15e-10 |
|  | 12 | 85673860 | 85674022 | PDFSTATSENSEMBLUCSC | 163 | 6 | 9901 | tss | 0.561979 | 0.983125 | ALX1 (margin: ALX1) | 5.57 | 2.16e-03 | 1.45e-02 |
|  | 12 | 111126960 | 111127139 | PDFSTATSENSEMBLUCSC | 180 | 4 | 10008 | tss | 0.071262 | 0.865386 | Not queried | 5.57 | 1.14e-01 | 1.68e-01 |
|  | 13 | 113633379 | 113633600 | PDFSTATSENSEMBLUCSC | 222 | 4 | 11546 | tss | 0.114897 | 0.882872 | Not queried | 5.57 | 2.86e-02 | 6.12e-02 |
|  | 4 | 183061830 | 183062004 | PDFSTATSENSEMBLUCSC | 175 | 5 | 30188 | gene;island | 0.609397 | 0.981871 | AC108142.1 (margin: TENM3; RP11-402C9.1; AC108142.1) | 5.57 | 7.94e-03 | 3.19e-02 |
|  | 1 | 3319264 | 3319509 | PDFSTATSENSEMBLUCSC | 246 | 4 | 123 | gene;island | 0.128896 | 0.891015 | Not queried | 5.57 | 5.71e-02 | 1.00e-01 |
|  | 8 | 10273666 | 10273890 | PDFSTATSENSEMBLUCSC | 225 | 4 | 39196 | gene | 0.380739 | 0.966459 | Not queried | 5.57 | 2.86e-02 | 6.12e-02 |
|  | 16 | 55513415 | 55513660 | PDFSTATSENSEMBLUCSC | 246 | 4 | 14752 | gene;island | 0.026222 | 0.526373 | Not queried | 5.57 | 2.86e-02 | 6.12e-02 |
|  | 19 | 37064219 | 37064886 | PDFSTATSENSEMBLUCSC | 668 | 10 | 20727 | tss | 0.014667 | 0.410684 | AC092295.7; ZNF529 (margin: AC092295.7; ZNF529) | 5.57 | 1.08e-05 | 4.82e-04 |
|  | 7 | 94284258 | 94285004 | PDFSTATSENSEMBLUCSC | 747 | 27 | 37092 | gene;tss;island | 0.810916 | 0.082725 | PEG10; SGCE (margin: PEG10; SGCE) | 5.56 | 2.94e-11 | 2.60e-08 |
|  | 7 | 29519297 | 29519656 | PDFSTATSENSEMBLUCSC | 360 | 8 | 36949 | gene | 0.231188 | 0.907870 | CHN2 (margin: CHN2) | 5.56 | 3.11e-04 | 4.77e-03 |
|  | 14 | 105714833 | 105715025 | PDFSTATSENSEMBLUCSC | 193 | 4 | 12259 | gene;tss;island | 0.034778 | 0.424164 | Not queried | 5.56 | 2.86e-02 | 6.12e-02 |
|  | 19 | 49340489 | 49340765 | PDFSTATSENSEMBLUCSC | 277 | 4 | 20138 | gene;tss;island | 0.985702 | 0.534181 | Not queried | 5.56 | 2.86e-02 | 6.12e-02 |
|  | 6 | 6588693 | 6588931 | PDFSTATSENSEMBLUCSC | 239 | 4 | 34610 | tss | 0.141130 | 0.892032 | Not queried | 5.56 | 1.14e-01 | 1.68e-01 |
|  | 15 | 42186830 | 42187179 | PDFSTATSENSEMBLUCSC | 350 | 4 | 13612 | tss | 0.101805 | 0.821411 | Not queried | 5.56 | 1.14e-01 | 1.68e-01 |
|  | 4 | 54424476 | 54424787 | PDFSTATSENSEMBLUCSC | 312 | 4 | 30396 | tss | 0.527458 | 0.967305 | Not queried | 5.56 | 2.00e-01 | 2.55e-01 |
|  | 4 | 41867415 | 41867562 | PDFSTATSENSEMBLUCSC | 148 | 5 | 31005 | island | 0.455622 | 0.985565 | (margin: LINC00682) | 5.56 | 7.94e-03 | 3.19e-02 |
|  | 10 | 180188 | 180755 | PDFSTATSENSEMBLUCSC | 568 | 12 | 5468 | island | 0.038165 | 0.003573 | ZMYND11 (margin: ZMYND11) | 5.56 | 8.29e-03 | 3.33e-02 |
|  | 11 | 1320329 | 1320634 | PDFSTATSENSEMBLUCSC | 306 | 4 | 6413 | gene;island | 0.008714 | 0.280771 | Not queried | 5.55 | 2.00e-01 | 2.55e-01 |
|  | 7 | 24797102 | 24797884 | PDFSTATSENSEMBLUCSC | 783 | 11 | 37478 | tss | 0.392099 | 0.967413 | DFNA5 (margin: DFNA5) | 5.55 | 8.33e-03 | 3.34e-02 |
|  | 10 | 131265059 | 131265796 | PDFSTATSENSEMBLUCSC | 738 | 15 | 6258 | island | 0.406370 | 0.007612 | MGMT (margin: MGMT) | 5.55 | 4.40e-05 | 1.23e-03 |
|  | 6 | 11044877 | 11044974 | PDFSTATSENSEMBLUCSC | 98 | 4 | 34634 | tss;island | 0.968326 | 0.527188 | Not queried | 5.55 | 1.14e-01 | 1.68e-01 |
|  | 19 | 9545862 | 9546377 | PDFSTATSENSEMBLUCSC | 516 | 12 | 21498 | island | 0.013176 | 0.385001 | ZNF266 (margin: ZNF266) | 5.55 | 7.40e-07 | 7.21e-05 |
|  | 19 | 9546252 | 9546377 | PDFSTATSENSEMBLUCSC | 126 | 8 | 20424 | tss | 0.013176 | 0.385001 | ZNF266 (margin: ZNF266) | 5.55 | 1.55e-04 | 2.71e-03 |
|  | 10 | 92631135 | 92631706 | PDFSTATSENSEMBLUCSC | 572 | 12 | 5219 | tss | 0.084240 | 0.008324 | Not queried | 5.55 | 8.87e-02 | 1.49e-01 |
|  | 17 | 16256937 | 16257223 | PDFSTATSENSEMBLUCSC | 287 | 9 | 17156 | tss;island | 0.171990 | 0.936737 | CENPV (margin: PIGL; CENPV) | 5.55 | 8.23e-05 | 2.06e-03 |
|  | 3 | 170746029 | 170746265 | PDFSTATSENSEMBLUCSC | 237 | 5 | 28717 | tss | 0.330673 | 0.974284 | SLC2A2 (margin: SLC2A2) | 5.54 | 7.94e-03 | 3.19e-02 |
|  | 19 | 45146828 | 45147078 | PDFSTATSENSEMBLUCSC | 251 | 7 | 20851 | tss;island | 0.009621 | 0.405884 | Not queried | 5.54 | 1.75e-02 | 5.79e-02 |
|  | 11 | 134336336 | 134336476 | PDFSTATSENSEMBLUCSC | 141 | 4 | 8988 | island | 0.225137 | 0.923029 | Not queried | 5.54 | 2.86e-02 | 6.12e-02 |
|  | 17 | 79608890 | 79609346 | PDFSTATSENSEMBLUCSC | 457 | 7 | 17743 | tss | 0.090606 | 0.970898 | Not queried | 5.54 | 1.75e-02 | 5.79e-02 |
|  | 14 | 101488345 | 101488488 | PDFSTATSENSEMBLUCSC | 144 | 4 | 12618 | tss | 0.038468 | 0.777815 | Not queried | 5.54 | 2.86e-02 | 6.12e-02 |
|  | 3 | 134083080 | 134083286 | PDFSTATSENSEMBLUCSC | 207 | 4 | 27954 | gene;island | 0.017531 | 0.431731 | Not queried | 5.54 | 2.86e-02 | 6.12e-02 |
|  | 16 | 89066587 | 89067014 | PDFSTATSENSEMBLUCSC | 428 | 4 | 16234 | island | 0.327208 | 0.943550 | Not queried | 5.53 | 5.71e-02 | 1.00e-01 |
|  | 7 | 101005832 | 101006089 | PDFSTATSENSEMBLUCSC | 258 | 9 | 37802 | tss;island | 0.583787 | 0.988591 | COL26A1 (margin: COL26A1) | 5.53 | 2.88e-04 | 4.58e-03 |
|  | 12 | 678853 | 679287 | PDFSTATSENSEMBLUCSC | 435 | 4 | 8996 | gene;island | 0.012664 | 0.390528 | Not queried | 5.53 | 2.00e-01 | 2.55e-01 |
|  | 15 | 89438795 | 89438964 | PDFSTATSENSEMBLUCSC | 170 | 4 | 13882 | tss | 0.205237 | 0.915618 | Not queried | 5.53 | 2.86e-02 | 6.12e-02 |
|  | 2 | 27805893 | 27806117 | PDFSTATSENSEMBLUCSC | 225 | 4 | 22631 | gene | 0.015565 | 0.276515 | Not queried | 5.53 | 2.86e-02 | 6.12e-02 |
|  | 11 | 112832117 | 112832249 | PDFSTATSENSEMBLUCSC | 133 | 4 | 6907 | gene | 0.466898 | 0.020311 | Not queried | 5.53 | 2.86e-02 | 6.12e-02 |
|  | 2 | 219848258 | 219848580 | PDFSTATSENSEMBLUCSC | 323 | 5 | 23022 | gene;island | 0.473489 | 0.973430 | Not queried | 5.53 | 5.56e-02 | 1.00e-01 |
|  | 3 | 170746029 | 170746291 | PDFSTATSENSEMBLUCSC | 263 | 6 | 29661 | island | 0.379421 | 0.972852 | SLC2A2 (margin: SLC2A2) | 5.53 | 2.16e-03 | 1.45e-02 |
|  | 3 | 184243086 | 184243411 | PDFSTATSENSEMBLUCSC | 326 | 4 | 29729 | island | 0.353231 | 0.964655 | Not queried | 5.53 | 2.86e-02 | 6.12e-02 |
|  | 1 | 3044741 | 3045166 | PDFSTATSENSEMBLUCSC | 426 | 6 | 101 | gene | 0.308099 | 0.968146 | PRDM16 (margin: PRDM16; RP1-163G9.2) | 5.53 | 2.16e-03 | 1.45e-02 |
|  | 9 | 34457129 | 34457500 | PDFSTATSENSEMBLUCSC | 372 | 4 | 40761 | gene;island | 0.334110 | 0.936417 | Not queried | 5.53 | 2.86e-02 | 6.12e-02 |
|  | 7 | 55000889 | 55001207 | PDFSTATSENSEMBLUCSC | 319 | 4 | 38502 | island | 0.170127 | 0.003865 | Not queried | 5.53 | 1.14e-01 | 1.68e-01 |
|  | 11 | 65547881 | 65548028 | PDFSTATSENSEMBLUCSC | 148 | 6 | 7440 | tss | 0.018850 | 0.531819 | AP5B1; AP001266.1 (margin: RP11-770G2.2; OVOL1; AP5B1; AP001266.1; RP11-770G2.4) | 5.52 | 2.16e-03 | 1.45e-02 |
|  | 5 | 10563358 | 10563856 | PDFSTATSENSEMBLUCSC | 499 | 4 | 31990 | tss;island | 0.142650 | 0.662246 | Not queried | 5.52 | 1.14e-01 | 1.68e-01 |
|  | 10 | 86001154 | 86001418 | PDFSTATSENSEMBLUCSC | 265 | 6 | 5896 | island | 0.080456 | 0.797022 | LRIT1 (margin: RGR; LRIT1) | 5.52 | 8.66e-03 | 3.36e-02 |
|  | 10 | 135090098 | 135090565 | PDFSTATSENSEMBLUCSC | 468 | 5 | 6350 | island | 0.321387 | 0.958079 | ADAM8 (margin: ADAM8; TUBGCP2) | 5.52 | 7.94e-03 | 3.19e-02 |
|  | 5 | 175792454 | 175792498 | PDFSTATSENSEMBLUCSC | 45 | 4 | 32510 | tss | 0.513619 | 0.977906 | Not queried | 5.52 | 2.86e-02 | 6.12e-02 |
|  | 17 | 12568499 | 12568761 | PDFSTATSENSEMBLUCSC | 263 | 4 | 17140 | tss;island | 0.093804 | 0.876167 | Not queried | 5.51 | 2.86e-02 | 6.12e-02 |
|  | 14 | 36003074 | 36003528 | PDFSTATSENSEMBLUCSC | 455 | 10 | 12781 | island | 0.452536 | 0.013810 | INSM2 (margin: INSM2; RALGAPA1) | 5.51 | 1.08e-05 | 4.82e-04 |
|  | 16 | 82044738 | 82045078 | PDFSTATSENSEMBLUCSC | 341 | 5 | 14857 | gene | 0.034422 | 0.651977 | SDR42E1 (margin: SDR42E1) | 5.51 | 7.94e-03 | 3.19e-02 |
|  | 11 | 69468789 | 69469403 | PDFSTATSENSEMBLUCSC | 615 | 9 | 8593 | island | 0.895978 | 0.303387 | CCND1; ORAOV1 (margin: CCND1; ORAOV1) | 5.51 | 8.23e-05 | 2.06e-03 |
|  | 7 | 149570931 | 149571373 | PDFSTATSENSEMBLUCSC | 443 | 12 | 37269 | gene;island | 0.503133 | 0.017461 | ATP6V0E2; ATP6V0E2-AS1 (margin: ZNF862; ATP6V0E2; ATP6V0E2-AS1) | 5.51 | 7.40e-07 | 7.21e-05 |
|  | 5 | 87969157 | 87969330 | PDFSTATSENSEMBLUCSC | 174 | 4 | 31678 | gene;tss;island | 0.258432 | 0.941259 | Not queried | 5.50 | 2.86e-02 | 6.12e-02 |
|  | 19 | 47287778 | 47288263 | PDFSTATSENSEMBLUCSC | 486 | 7 | 20114 | gene;island | 0.000726 | 0.037782 | Not queried | 5.50 | 1.75e-02 | 5.79e-02 |
|  | 18 | 60987770 | 60988155 | PDFSTATSENSEMBLUCSC | 386 | 11 | 19554 | island | 0.202685 | 0.008352 | BCL2 (margin: BCL2; RP11-28F1.2; KDSR) | 5.50 | 6.63e-03 | 3.19e-02 |
|  | 2 | 152955498 | 152955783 | PDFSTATSENSEMBLUCSC | 286 | 5 | 23662 | tss;island | 0.541982 | 0.025987 | AC079790.2; CACNB4 (margin: AC079790.2; CACNB4) | 5.50 | 7.94e-03 | 3.19e-02 |
|  | 7 | 156802150 | 156802502 | PDFSTATSENSEMBLUCSC | 353 | 7 | 37317 | gene;tss;island | 0.671111 | 0.036629 | RP5-1121A15.1; MNX1 (margin: RP5-1121A15.4; RP5-1121A15.1; MNX1) | 5.50 | 5.83e-04 | 6.47e-03 |
|  | 14 | 69951211 | 69951495 | PDFSTATSENSEMBLUCSC | 285 | 7 | 12967 | island | 0.527078 | 0.018484 | PLEKHD1 (margin: PLEKHD1) | 5.50 | 5.83e-04 | 6.47e-03 |
|  | 1 | 32083737 | 32083990 | PDFSTATSENSEMBLUCSC | 254 | 4 | 382 | gene;island | 0.144941 | 0.019893 | Not queried | 5.50 | 2.00e-01 | 2.55e-01 |
|  | 15 | 83378637 | 83378895 | PDFSTATSENSEMBLUCSC | 259 | 8 | 13859 | tss | 0.136691 | 0.006341 | AP3B2; AC105339.1 (margin: AP3B2; AC105339.1) | 5.49 | 6.22e-04 | 6.71e-03 |
|  | 17 | 72889708 | 72890001 | PDFSTATSENSEMBLUCSC | 294 | 8 | 17634 | tss | 0.468382 | 0.954638 | FADS6 (margin: FADS6) | 5.49 | 6.22e-04 | 6.71e-03 |
|  | 20 | 55841869 | 55842071 | PDFSTATSENSEMBLUCSC | 203 | 8 | 25840 | tss;island | 0.863226 | 0.075758 | RP4-813D12.3; BMP7 (margin: RP4-813D12.3; BMP7) | 5.49 | 3.11e-04 | 4.77e-03 |
|  | 8 | 19540210 | 19540734 | PDFSTATSENSEMBLUCSC | 525 | 8 | 39572 | tss | 0.137751 | 0.892700 | RP11-1105O14.1; CSGALNACT1 (margin: RP11-1105O14.1; CSGALNACT1) | 5.49 | 6.99e-03 | 3.19e-02 |
|  | 11 | 124805912 | 124806173 | PDFSTATSENSEMBLUCSC | 262 | 4 | 6956 | gene | 0.035889 | 0.551990 | Not queried | 5.49 | 2.86e-02 | 6.12e-02 |
|  | 10 | 18549378 | 18549778 | PDFSTATSENSEMBLUCSC | 401 | 7 | 4521 | gene | 0.486116 | 0.963709 | CACNB2; RP11-109I13.2 (margin: CACNB2; RP11-109I13.2) | 5.49 | 6.99e-03 | 3.19e-02 |
|  | 5 | 476497 | 476842 | PDFSTATSENSEMBLUCSC | 346 | 4 | 31505 | gene;island | 0.376006 | 0.964312 | Not queried | 5.49 | 2.86e-02 | 6.12e-02 |
|  | 20 | 1974738 | 1974924 | PDFSTATSENSEMBLUCSC | 187 | 4 | 25540 | tss | 0.034960 | 0.463977 | Not queried | 5.49 | 2.86e-02 | 6.12e-02 |
|  | 8 | 145654565 | 145654854 | PDFSTATSENSEMBLUCSC | 290 | 5 | 39523 | gene;tss;island | 0.493492 | 0.980491 | VPS28; TONSL (margin: AC084125.4; VPS28; TONSL) | 5.49 | 7.94e-03 | 3.19e-02 |
|  | 12 | 71552188 | 71552569 | PDFSTATSENSEMBLUCSC | 382 | 4 | 9870 | tss | 0.083053 | 0.730225 | Not queried | 5.48 | 1.14e-01 | 1.68e-01 |
|  | 14 | 105041382 | 105041708 | PDFSTATSENSEMBLUCSC | 327 | 5 | 13207 | island | 0.117341 | 0.892549 | Not queried | 5.48 | 1.59e-02 | 5.36e-02 |
|  | 7 | 151553471 | 151553868 | PDFSTATSENSEMBLUCSC | 398 | 6 | 37297 | gene | 0.881688 | 0.030597 | PRKAG2 (margin: PRKAG2) | 5.48 | 8.66e-03 | 3.36e-02 |
|  | 7 | 1407330 | 1407571 | PDFSTATSENSEMBLUCSC | 242 | 6 | 38100 | island | 0.224746 | 0.931273 | Not queried | 5.48 | 1.32e-01 | 1.89e-01 |
|  | 1 | 6546574 | 6546839 | PDFSTATSENSEMBLUCSC | 266 | 5 | 176 | gene;tss;island | 0.617785 | 0.037988 | PLEKHG5 (margin: PLEKHG5) | 5.47 | 7.94e-03 | 3.19e-02 |
|  | 1 | 244624521 | 244624883 | PDFSTATSENSEMBLUCSC | 363 | 6 | 4394 | island | 0.418953 | 0.011095 | C1orf101 (margin: C1orf101; ADSS) | 5.47 | 2.16e-03 | 1.45e-02 |
|  | 13 | 113759771 | 113760101 | PDFSTATSENSEMBLUCSC | 331 | 5 | 11547 | tss | 0.539466 | 0.030111 | F7 (margin: MCF2L; F7; AL137002.1) | 5.47 | 7.94e-03 | 3.19e-02 |
|  | 2 | 70351227 | 70351524 | PDFSTATSENSEMBLUCSC | 298 | 4 | 22721 | gene;island | 0.600992 | 0.027638 | Not queried | 5.47 | 2.86e-02 | 6.12e-02 |
|  | 11 | 615945 | 616112 | PDFSTATSENSEMBLUCSC | 168 | 6 | 7020 | tss;island | 0.479860 | 0.011663 | IRF7; CDHR5 (margin: PHRF1; IRF7; CDHR5; SCT) | 5.47 | 4.33e-03 | 2.52e-02 |
|  | 2 | 174219209 | 174219534 | PDFSTATSENSEMBLUCSC | 326 | 5 | 23722 | tss;island | 0.012701 | 0.448256 | CDCA7; AC092573.2 (margin: CDCA7; AC092573.2) | 5.47 | 7.94e-03 | 3.19e-02 |
|  | 8 | 1642380 | 1642625 | PDFSTATSENSEMBLUCSC | 246 | 4 | 39172 | gene;island | 0.577777 | 0.978308 | Not queried | 5.47 | 5.71e-02 | 1.00e-01 |
|  | 6 | 6007259 | 6007769 | PDFSTATSENSEMBLUCSC | 511 | 6 | 35407 | island | 0.401251 | 0.976119 | NRN1 (margin: NRN1) | 5.47 | 2.16e-03 | 1.45e-02 |
|  | 8 | 133493139 | 133493200 | PDFSTATSENSEMBLUCSC | 62 | 4 | 39918 | tss;island | 0.441022 | 0.018585 | Not queried | 5.46 | 2.86e-02 | 6.12e-02 |
|  | 5 | 57878745 | 57879417 | PDFSTATSENSEMBLUCSC | 673 | 12 | 32817 | island | 0.681921 | 0.027506 | RAB3C (margin: RAB3C) | 5.46 | 2.01e-04 | 3.38e-03 |
|  | 12 | 111471192 | 111471489 | PDFSTATSENSEMBLUCSC | 298 | 4 | 10013 | tss;island | 0.504432 | 0.043061 | Not queried | 5.46 | 5.71e-02 | 1.00e-01 |
|  | 11 | 123301490 | 123301758 | PDFSTATSENSEMBLUCSC | 269 | 4 | 8908 | island | 0.438061 | 0.976297 | Not queried | 5.46 | 2.86e-02 | 6.12e-02 |
|  | 7 | 51096557 | 51096971 | PDFSTATSENSEMBLUCSC | 415 | 6 | 37016 | gene | 0.328526 | 0.980123 | Not queried | 5.46 | 2.60e-02 | 6.12e-02 |
|  | 8 | 75896645 | 75897310 | PDFSTATSENSEMBLUCSC | 666 | 9 | 40400 | island | 0.418945 | 0.013349 | CRISPLD1 (margin: CRISPLD1) | 5.46 | 4.11e-05 | 1.17e-03 |
|  | 2 | 121199631 | 121199920 | PDFSTATSENSEMBLUCSC | 290 | 4 | 24666 | island | 0.569901 | 0.032056 | Not queried | 5.46 | 2.86e-02 | 6.12e-02 |
|  | 2 | 30454146 | 30454643 | PDFSTATSENSEMBLUCSC | 498 | 8 | 24201 | island | 0.211085 | 0.715719 | LBH (margin: LBH) | 5.46 | 6.22e-04 | 6.71e-03 |
|  | 2 | 30454146 | 30454363 | PDFSTATSENSEMBLUCSC | 218 | 6 | 23285 | tss | 0.213201 | 0.715719 | LBH (margin: LBH) | 5.46 | 4.33e-03 | 2.52e-02 |
|  | 2 | 160761085 | 160761622 | PDFSTATSENSEMBLUCSC | 538 | 8 | 24812 | island | 0.357431 | 0.961751 | LY75; LY75-CD302 (margin: LY75; LY75-CD302) | 5.46 | 1.55e-04 | 2.71e-03 |
|  | 2 | 133428502 | 133429299 | PDFSTATSENSEMBLUCSC | 798 | 9 | 23640 | tss | 0.008623 | 0.132120 | LYPD1; NCKAP5 (margin: AC010974.3; LYPD1; NCKAP5) | 5.46 | 7.77e-03 | 3.19e-02 |
|  | 16 | 88966229 | 88966509 | PDFSTATSENSEMBLUCSC | 281 | 5 | 14921 | gene;island | 0.448193 | 0.989755 | Not queried | 5.45 | 1.59e-02 | 5.36e-02 |
|  | 3 | 9993731 | 9994280 | PDFSTATSENSEMBLUCSC | 550 | 9 | 28868 | island | 0.381345 | 0.009662 | PRRT3-AS1; PRRT3 (margin: CRELD1; PRRT3-AS1; RP11-1020A11.2; PRRT3; EMC3) | 5.45 | 4.11e-05 | 1.17e-03 |
|  | 14 | 65439236 | 65439362 | PDFSTATSENSEMBLUCSC | 127 | 4 | 12464 | tss;island | 0.539483 | 0.016977 | Not queried | 5.45 | 2.86e-02 | 6.12e-02 |
|  | 2 | 135476601 | 135477056 | PDFSTATSENSEMBLUCSC | 456 | 8 | 23642 | tss;island | 0.666534 | 0.053036 | TMEM163 (margin: TMEM163) | 5.45 | 1.04e-02 | 3.96e-02 |
|  | 15 | 98196022 | 98196333 | PDFSTATSENSEMBLUCSC | 312 | 5 | 14492 | island | 0.635794 | 0.075410 | Not queried | 5.45 | 9.52e-02 | 1.53e-01 |
|  | 2 | 12857398 | 12857811 | PDFSTATSENSEMBLUCSC | 414 | 4 | 22576 | gene;island | 0.225035 | 0.007340 | Not queried | 5.45 | 5.71e-02 | 1.00e-01 |
|  | 8 | 75896645 | 75896836 | PDFSTATSENSEMBLUCSC | 192 | 5 | 39775 | tss | 0.380480 | 0.019352 | CRISPLD1 (margin: CRISPLD1) | 5.45 | 7.94e-03 | 3.19e-02 |
|  | 2 | 242805838 | 242806119 | PDFSTATSENSEMBLUCSC | 282 | 5 | 25311 | island | 0.499163 | 0.976368 | (margin: CXXC11; PDCD1) | 5.45 | 7.94e-03 | 3.19e-02 |
|  | 12 | 113229245 | 113229534 | PDFSTATSENSEMBLUCSC | 290 | 7 | 10023 | tss | 0.846347 | 0.020576 | Not queried | 5.45 | 2.62e-02 | 6.12e-02 |
|  | 5 | 3962310 | 3962477 | PDFSTATSENSEMBLUCSC | 168 | 4 | 32671 | island | 0.867534 | 0.197283 | Not queried | 5.44 | 1.14e-01 | 1.68e-01 |
|  | 17 | 43922122 | 43922423 | PDFSTATSENSEMBLUCSC | 302 | 6 | 16638 | gene | 0.091335 | 0.828997 | SPPL2C; MAPT-AS1 (margin: CRHR1; SPPL2C; MAPT-AS1) | 5.44 | 2.16e-03 | 1.45e-02 |
|  | 2 | 20866107 | 20866500 | PDFSTATSENSEMBLUCSC | 394 | 9 | 24115 | island | 0.294031 | 0.983572 | GDF7 (margin: GDF7; AC012065.7; RP11-130L8.1) | 5.44 | 4.11e-05 | 1.17e-03 |
|  | 7 | 27194614 | 27195036 | PDFSTATSENSEMBLUCSC | 423 | 5 | 36936 | gene;island | 0.556553 | 0.957782 | Not queried | 5.44 | 3.17e-02 | 6.50e-02 |
|  | 16 | 215410 | 215960 | PDFSTATSENSEMBLUCSC | 551 | 7 | 14962 | tss | 0.544603 | 0.981374 | HBM; HBZP1 (margin: HBZ; HBM; HBZP1; Z84721.4; HBA2; HBA1) | 5.44 | 5.83e-04 | 6.47e-03 |
|  | 1 | 3147891 | 3148005 | PDFSTATSENSEMBLUCSC | 115 | 4 | 110 | gene;island | 0.474282 | 0.981394 | Not queried | 5.44 | 5.71e-02 | 1.00e-01 |
|  | 2 | 1544321 | 1544474 | PDFSTATSENSEMBLUCSC | 154 | 4 | 22533 | gene;island | 0.507832 | 0.962421 | Not queried | 5.43 | 2.86e-02 | 6.12e-02 |
|  | 19 | 55013549 | 55013954 | PDFSTATSENSEMBLUCSC | 406 | 6 | 21063 | tss | 0.153621 | 0.933992 | Not queried | 5.43 | 2.60e-02 | 6.12e-02 |
|  | 12 | 59989535 | 59989742 | PDFSTATSENSEMBLUCSC | 208 | 4 | 10580 | island | 0.222933 | 0.009409 | Not queried | 5.43 | 2.86e-02 | 6.12e-02 |
|  | 17 | 35078659 | 35079061 | PDFSTATSENSEMBLUCSC | 403 | 4 | 18233 | island | 0.479808 | 0.975946 | Not queried | 5.43 | 2.86e-02 | 6.12e-02 |
|  | 8 | 62200380 | 62200518 | PDFSTATSENSEMBLUCSC | 139 | 4 | 39727 | tss | 0.576647 | 0.160457 | Not queried | 5.43 | 3.43e-01 | 3.84e-01 |
|  | 1 | 91172710 | 91173030 | PDFSTATSENSEMBLUCSC | 321 | 5 | 3455 | island | 0.805338 | 0.021251 | Not queried | 5.43 | 9.52e-02 | 1.53e-01 |
|  | 6 | 112375186 | 112375333 | PDFSTATSENSEMBLUCSC | 148 | 6 | 35180 | tss | 0.152571 | 0.927626 | WISP3 (margin: WISP3) | 5.43 | 2.16e-03 | 1.45e-02 |
|  | 6 | 31148332 | 31148748 | PDFSTATSENSEMBLUCSC | 417 | 16 | 35754 | island | 0.973725 | 0.506941 | POU5F1 (margin: POU5F1; PSORS1C3) | 5.43 | 3.33e-09 | 1.10e-06 |
|  | 10 | 26504950 | 26505156 | PDFSTATSENSEMBLUCSC | 207 | 5 | 4993 | tss | 0.649864 | 0.033092 | GAD2 (margin: MYO3A; GAD2) | 5.43 | 7.94e-03 | 3.19e-02 |
|  | 2 | 113735377 | 113735503 | PDFSTATSENSEMBLUCSC | 127 | 4 | 23581 | tss | 0.133304 | 0.818621 | Not queried | 5.43 | 2.86e-02 | 6.12e-02 |
|  | 22 | 18593294 | 18593441 | PDFSTATSENSEMBLUCSC | 148 | 6 | 26948 | tss | 0.551085 | 0.017932 | PEX26; TUBA8 (margin: PEX26; XXbac-B476C20.10; TUBA8; ARL2BPP10) | 5.42 | 2.16e-03 | 1.45e-02 |
|  | 5 | 178322737 | 178322856 | PDFSTATSENSEMBLUCSC | 120 | 5 | 32544 | tss | 0.021141 | 0.461609 | Not queried | 5.42 | 1.51e-01 | 2.11e-01 |
|  | 8 | 60031823 | 60031944 | PDFSTATSENSEMBLUCSC | 122 | 6 | 39722 | tss;island | 0.284781 | 0.925134 | RP11-25K19.1; TOX (margin: RP11-25K19.1; TOX) | 5.42 | 2.16e-03 | 1.45e-02 |
|  | 6 | 33153052 | 33153499 | PDFSTATSENSEMBLUCSC | 448 | 7 | 34211 | gene | 0.186968 | 0.957079 | Not queried | 5.42 | 3.79e-02 | 7.66e-02 |
|  | 10 | 180465 | 180755 | PDFSTATSENSEMBLUCSC | 291 | 5 | 4430 | gene | 0.112421 | 0.003007 | Not queried | 5.42 | 9.52e-02 | 1.53e-01 |
|  | 2 | 176971820 | 176972113 | PDFSTATSENSEMBLUCSC | 294 | 8 | 24896 | island | 0.020965 | 0.473734 | HOXD11; AC009336.1; HOXD10 (margin: HOXD13; HOXD12; HOXD11; AC009336.1; HOXD10) | 5.41 | 1.55e-04 | 2.71e-03 |
|  | 3 | 61547104 | 61547287 | PDFSTATSENSEMBLUCSC | 184 | 7 | 29243 | island | 0.431322 | 0.019209 | PTPRG (margin: PTPRG) | 5.41 | 5.83e-04 | 6.47e-03 |
|  | 11 | 35160330 | 35160892 | PDFSTATSENSEMBLUCSC | 563 | 8 | 8205 | island | 0.008193 | 0.335136 | CD44; RP4-607I7.1 (margin: CD44; AL356215.1; RP4-607I7.1) | 5.41 | 1.09e-03 | 1.10e-02 |
|  | 14 | 55595666 | 55595958 | PDFSTATSENSEMBLUCSC | 293 | 5 | 12864 | island | 0.013638 | 0.428839 | LGALS3 (margin: LGALS3) | 5.41 | 7.94e-03 | 3.19e-02 |
|  | 17 | 38821155 | 38821403 | PDFSTATSENSEMBLUCSC | 249 | 4 | 16566 | gene | 0.544131 | 0.980207 | Not queried | 5.41 | 2.86e-02 | 6.12e-02 |
|  | 2 | 88752056 | 88752322 | PDFSTATSENSEMBLUCSC | 267 | 5 | 23500 | tss;island | 0.549623 | 0.021960 | Not queried | 5.41 | 1.59e-02 | 5.36e-02 |
|  | 5 | 176057740 | 176057996 | PDFSTATSENSEMBLUCSC | 257 | 5 | 31911 | gene;tss;island | 0.879414 | 0.232535 | Not queried | 5.41 | 9.52e-02 | 1.53e-01 |
|  | 5 | 172661803 | 172662463 | PDFSTATSENSEMBLUCSC | 661 | 8 | 33407 | island | 0.279337 | 0.934953 | NKX2-5 (margin: RPL7AP33; NKX2-5) | 5.41 | 4.66e-03 | 2.68e-02 |
|  | 17 | 7452289 | 7452644 | PDFSTATSENSEMBLUCSC | 356 | 5 | 17955 | island | 0.219343 | 0.945058 | TNFSF12; TNFSF12-TNFSF13 (margin: TNFSF12; TNFSF12-TNFSF13; TNFSF13; Y\_RNA) | 5.41 | 7.94e-03 | 3.19e-02 |
|  | 6 | 32026605 | 32027023 | PDFSTATSENSEMBLUCSC | 419 | 7 | 34023 | gene | 0.301706 | 0.945754 | Not queried | 5.41 | 2.59e-01 | 3.16e-01 |
|  | 17 | 71640253 | 71640369 | PDFSTATSENSEMBLUCSC | 117 | 6 | 17618 | tss | 0.419848 | 0.016548 | RP11-277J6.2; SDK2 (margin: RP11-277J6.2; SDK2) | 5.40 | 4.33e-03 | 2.52e-02 |
|  | 22 | 37212787 | 37213130 | PDFSTATSENSEMBLUCSC | 344 | 4 | 26860 | gene;island | 0.612351 | 0.019228 | Not queried | 5.40 | 2.86e-02 | 6.12e-02 |
|  | 10 | 125853676 | 125853893 | PDFSTATSENSEMBLUCSC | 218 | 4 | 6225 | island | 0.876870 | 0.141891 | Not queried | 5.40 | 2.86e-02 | 6.12e-02 |
|  | 10 | 99531645 | 99531934 | PDFSTATSENSEMBLUCSC | 290 | 8 | 5996 | island | 0.223994 | 0.945905 | SFRP5 (margin: ZFYVE27; SFRP5) | 5.39 | 2.95e-03 | 1.88e-02 |
|  | 12 | 120799597 | 120800066 | PDFSTATSENSEMBLUCSC | 470 | 4 | 9351 | gene;island | 0.648307 | 0.965862 | Not queried | 5.39 | 2.86e-02 | 6.12e-02 |
|  | 5 | 1930801 | 1931781 | PDFSTATSENSEMBLUCSC | 981 | 8 | 32646 | island | 0.428493 | 0.938438 | (margin: RP11-259O2.1) | 5.39 | 1.86e-03 | 1.45e-02 |
|  | 5 | 178986131 | 178986412 | PDFSTATSENSEMBLUCSC | 282 | 4 | 31932 | gene;tss;island | 0.205992 | 0.007863 | Not queried | 5.39 | 2.86e-02 | 6.12e-02 |
|  | 4 | 13530094 | 13530470 | PDFSTATSENSEMBLUCSC | 377 | 4 | 30930 | island | 0.423420 | 0.965986 | Not queried | 5.38 | 1.14e-01 | 1.68e-01 |
|  | 4 | 90757351 | 90757814 | PDFSTATSENSEMBLUCSC | 464 | 7 | 30057 | gene;island | 0.264788 | 0.962328 | RP11-67M1.1; SNCA (margin: RP11-67M1.1; SNCA) | 5.38 | 5.83e-04 | 6.47e-03 |
|  | 6 | 142409939 | 142410235 | PDFSTATSENSEMBLUCSC | 297 | 8 | 35260 | tss | 0.721184 | 0.037017 | Not queried | 5.38 | 3.79e-02 | 7.66e-02 |
|  | 2 | 20865651 | 20865847 | PDFSTATSENSEMBLUCSC | 197 | 4 | 23214 | tss;island | 0.648575 | 0.975942 | Not queried | 5.38 | 2.86e-02 | 6.12e-02 |
|  | 12 | 52431177 | 52431508 | PDFSTATSENSEMBLUCSC | 332 | 5 | 10434 | island | 0.759173 | 0.031809 | Not queried | 5.38 | 1.59e-02 | 5.36e-02 |
|  | 6 | 31431312 | 31431573 | PDFSTATSENSEMBLUCSC | 262 | 4 | 33877 | gene | 0.252286 | 0.928478 | Not queried | 5.38 | 5.71e-02 | 1.00e-01 |
|  | 1 | 2846836 | 2847220 | PDFSTATSENSEMBLUCSC | 385 | 5 | 2521 | island | 0.364526 | 0.949802 | Not queried | 5.38 | 1.59e-02 | 5.36e-02 |
|  | 6 | 71122912 | 71123041 | PDFSTATSENSEMBLUCSC | 130 | 5 | 35057 | tss | 0.016466 | 0.489987 | FAM135A; RP11-462G2.2 (margin: FAM135A; RP11-462G2.2) | 5.38 | 7.94e-03 | 3.19e-02 |
|  | 21 | 22369802 | 22370444 | PDFSTATSENSEMBLUCSC | 643 | 6 | 26495 | tss;island | 0.817345 | 0.279340 | NCAM2 (margin: NCAM2) | 5.38 | 2.16e-03 | 1.45e-02 |
|  | 16 | 67312928 | 67313043 | PDFSTATSENSEMBLUCSC | 116 | 7 | 14797 | gene;tss;island | 0.372309 | 0.034200 | PLEKHG4 (margin: SLC9A5; PLEKHG4; KCTD19) | 5.38 | 4.08e-03 | 2.47e-02 |
|  | 3 | 192958383 | 192958968 | PDFSTATSENSEMBLUCSC | 586 | 7 | 28794 | tss;island | 0.413652 | 0.972772 | HRASLS (margin: HRASLS) | 5.38 | 5.83e-04 | 6.47e-03 |
|  | 11 | 119455041 | 119455905 | PDFSTATSENSEMBLUCSC | 865 | 8 | 8885 | island | 0.012682 | 0.381139 | (margin: ) | 5.38 | 1.55e-04 | 2.71e-03 |
|  | 15 | 75917792 | 75918163 | PDFSTATSENSEMBLUCSC | 372 | 4 | 13431 | gene;island | 0.033785 | 0.576161 | Not queried | 5.38 | 2.86e-02 | 6.12e-02 |
|  | 17 | 45772333 | 45772800 | PDFSTATSENSEMBLUCSC | 468 | 7 | 18457 | island | 0.345098 | 0.972495 | TBKBP1 (margin: KPNB1; TBKBP1) | 5.38 | 1.17e-03 | 1.13e-02 |
|  | 6 | 29601398 | 29601705 | PDFSTATSENSEMBLUCSC | 308 | 6 | 34750 | tss;island | 0.940755 | 0.227902 | GABBR1 (margin: GABBR1; SUMO2P1) | 5.38 | 2.16e-03 | 1.45e-02 |
|  | 19 | 2041905 | 2042088 | PDFSTATSENSEMBLUCSC | 184 | 5 | 19676 | gene;island | 0.814789 | 0.113597 | Not queried | 5.37 | 3.17e-02 | 6.50e-02 |
|  | 20 | 47896769 | 47897451 | PDFSTATSENSEMBLUCSC | 683 | 7 | 25447 | gene;island | 0.009393 | 0.381828 | ZFAS1; SNORD12C; SNORD12B; SNORD12; ZNFX1 (margin: ZFAS1; SNORD12C; SNORD12B; SNORD12; ZNFX1) | 5.37 | 5.83e-04 | 6.47e-03 |
|  | 20 | 47896769 | 47897207 | PDFSTATSENSEMBLUCSC | 439 | 5 | 25807 | tss | 0.011800 | 0.381828 | ZFAS1; SNORD12C; SNORD12B; SNORD12; ZNFX1 (margin: ZFAS1; SNORD12C; SNORD12B; SNORD12; ZNFX1) | 5.37 | 7.94e-03 | 3.19e-02 |
|  | 17 | 54670875 | 54671575 | PDFSTATSENSEMBLUCSC | 701 | 6 | 18577 | island | 0.485699 | 0.024918 | NOG (margin: NOG) | 5.37 | 2.16e-03 | 1.45e-02 |
|  | 1 | 226411715 | 226411954 | PDFSTATSENSEMBLUCSC | 240 | 4 | 1032 | gene;island | 0.526982 | 0.979808 | Not queried | 5.37 | 2.86e-02 | 6.12e-02 |
|  | 19 | 40724308 | 40724388 | PDFSTATSENSEMBLUCSC | 81 | 5 | 20783 | tss;island | 0.308146 | 0.955682 | TTC9B (margin: MAP3K10; TTC9B; CNTD2; AKT2) | 5.37 | 7.94e-03 | 3.19e-02 |
|  | 3 | 61546275 | 61546526 | PDFSTATSENSEMBLUCSC | 252 | 4 | 28422 | tss;island | 0.223658 | 0.017494 | Not queried | 5.37 | 2.86e-02 | 6.12e-02 |
|  | 6 | 32525813 | 32526027 | PDFSTATSENSEMBLUCSC | 215 | 5 | 34117 | gene | 0.136934 | 0.760662 | HLA-DRB6 (margin: RNU1-61P; HLA-DRB6) | 5.37 | 7.94e-03 | 3.19e-02 |
|  | 12 | 111126844 | 111127139 | PDFSTATSENSEMBLUCSC | 296 | 5 | 9303 | gene;island | 0.077213 | 0.845415 | Not queried | 5.37 | 5.56e-02 | 1.00e-01 |
|  | 11 | 133827056 | 133827256 | PDFSTATSENSEMBLUCSC | 201 | 5 | 7826 | tss;island | 0.346179 | 0.010533 | IGSF9B; AP000911.1 (margin: IGSF9B; AP000911.1) | 5.37 | 7.94e-03 | 3.19e-02 |
|  | 11 | 46366643 | 46366884 | PDFSTATSENSEMBLUCSC | 242 | 6 | 7276 | tss | 0.169284 | 0.901455 | DGKZ (margin: DGKZ) | 5.37 | 2.16e-03 | 1.45e-02 |
|  | 19 | 58695006 | 58695164 | PDFSTATSENSEMBLUCSC | 159 | 4 | 20258 | gene;island | 0.027740 | 0.519126 | Not queried | 5.37 | 2.86e-02 | 6.12e-02 |
|  | 11 | 65360123 | 65360509 | PDFSTATSENSEMBLUCSC | 387 | 5 | 8467 | island | 0.140551 | 0.918716 | Not queried | 5.36 | 9.52e-02 | 1.53e-01 |
|  | 7 | 23509546 | 23509770 | PDFSTATSENSEMBLUCSC | 225 | 4 | 36915 | gene;island | 0.036493 | 0.551860 | Not queried | 5.36 | 1.14e-01 | 1.68e-01 |
|  | 1 | 34629098 | 34629559 | PDFSTATSENSEMBLUCSC | 462 | 5 | 408 | gene;island | 0.297024 | 0.959235 | Not queried | 5.36 | 1.59e-02 | 5.36e-02 |
|  | 12 | 113587240 | 113587581 | PDFSTATSENSEMBLUCSC | 342 | 5 | 10026 | tss | 0.190699 | 0.727223 | Not queried | 5.36 | 1.51e-01 | 2.11e-01 |
|  | 17 | 1810459 | 1810853 | PDFSTATSENSEMBLUCSC | 395 | 5 | 17832 | island | 0.643304 | 0.986509 | Not queried | 5.36 | 3.17e-02 | 6.50e-02 |
|  | 18 | 44259787 | 44260091 | PDFSTATSENSEMBLUCSC | 305 | 4 | 19104 | gene;island | 0.376586 | 0.972530 | Not queried | 5.35 | 5.71e-02 | 1.00e-01 |
|  | 12 | 4923404 | 4923529 | PDFSTATSENSEMBLUCSC | 126 | 4 | 9018 | gene;island | 0.189324 | 0.950790 | Not queried | 5.35 | 5.71e-02 | 1.00e-01 |
|  | 6 | 71666682 | 71666858 | PDFSTATSENSEMBLUCSC | 177 | 5 | 36242 | island | 0.446170 | 0.969011 | B3GAT2 (margin: B3GAT2) | 5.35 | 7.94e-03 | 3.19e-02 |
|  | 7 | 87104724 | 87105416 | PDFSTATSENSEMBLUCSC | 693 | 8 | 38635 | island | 0.471797 | 0.976440 | ABCB4 (margin: ABCB4) | 5.35 | 1.55e-04 | 2.71e-03 |
|  | 2 | 14772312 | 14772734 | PDFSTATSENSEMBLUCSC | 423 | 7 | 23195 | tss;island | 0.367170 | 0.015302 | Not queried | 5.35 | 2.62e-02 | 6.12e-02 |
|  | 3 | 49236800 | 49236918 | PDFSTATSENSEMBLUCSC | 119 | 5 | 28324 | tss | 0.328873 | 0.960796 | Not queried | 5.35 | 9.52e-02 | 1.53e-01 |
|  | 12 | 132293329 | 132293702 | PDFSTATSENSEMBLUCSC | 374 | 5 | 11015 | island | 0.524169 | 0.984082 | (margin: SFSWAP; RNA5SP378; RNU6-1017P) | 5.35 | 7.94e-03 | 3.19e-02 |
|  | 5 | 63461216 | 63461930 | PDFSTATSENSEMBLUCSC | 715 | 10 | 32828 | island | 0.665439 | 0.039022 | RNF180 (margin: RNF180) | 5.34 | 2.06e-04 | 3.42e-03 |
|  | 3 | 79817125 | 79817333 | PDFSTATSENSEMBLUCSC | 209 | 4 | 28448 | tss | 0.147765 | 0.880409 | Not queried | 5.34 | 2.86e-02 | 6.12e-02 |
|  | 3 | 187870621 | 187871034 | PDFSTATSENSEMBLUCSC | 414 | 7 | 28780 | tss;island | 0.195855 | 0.002931 | LPP; LPP-AS2 (margin: RP11-430L16.1; LPP; LPP-AS2) | 5.34 | 5.83e-04 | 6.47e-03 |
|  | 16 | 49316845 | 49317262 | PDFSTATSENSEMBLUCSC | 418 | 5 | 15907 | island | 0.767440 | 0.083553 | Not queried | 5.34 | 1.59e-02 | 5.36e-02 |
|  | 11 | 67183078 | 67183616 | PDFSTATSENSEMBLUCSC | 539 | 8 | 8545 | island | 0.503873 | 0.022044 | Not queried | 5.34 | 1.30e-01 | 1.89e-01 |
|  | 12 | 67072951 | 67073062 | PDFSTATSENSEMBLUCSC | 112 | 4 | 9850 | tss | 0.308709 | 0.979745 | Not queried | 5.34 | 2.86e-02 | 6.12e-02 |
|  | 17 | 27045043 | 27045302 | PDFSTATSENSEMBLUCSC | 260 | 6 | 17218 | tss;island | 0.008018 | 0.338559 | RPL23A; RAB34 (margin: RPL23A; SNORD42B; SNORD4A; SNORD42A; SNORD4B; NEK8; AC010761.14; PROCA1; RAB34; AC010761.8; TLCD1) | 5.34 | 2.16e-03 | 1.45e-02 |
|  | 2 | 160761262 | 160761622 | PDFSTATSENSEMBLUCSC | 361 | 6 | 23683 | tss | 0.357431 | 0.960949 | LY75; LY75-CD302 (margin: LY75; LY75-CD302) | 5.33 | 2.16e-03 | 1.45e-02 |
|  | 4 | 3471816 | 3472246 | PDFSTATSENSEMBLUCSC | 431 | 4 | 29906 | gene;island | 0.421125 | 0.941592 | Not queried | 5.33 | 5.71e-02 | 1.00e-01 |
|  | 1 | 205424972 | 205425241 | PDFSTATSENSEMBLUCSC | 270 | 6 | 4127 | island | 0.065263 | 0.747642 | Not queried | 5.33 | 9.31e-02 | 1.53e-01 |
|  | 15 | 40799212 | 40799450 | PDFSTATSENSEMBLUCSC | 239 | 4 | 14039 | island | 0.416316 | 0.016745 | Not queried | 5.33 | 2.86e-02 | 6.12e-02 |
|  | 2 | 131513608 | 131513735 | PDFSTATSENSEMBLUCSC | 128 | 5 | 23630 | tss | 0.424751 | 0.025977 | AMER3; AC140481.8 (margin: AMER3; AC140481.8) | 5.33 | 7.94e-03 | 3.19e-02 |
|  | 19 | 58446745 | 58446988 | PDFSTATSENSEMBLUCSC | 244 | 8 | 21129 | tss | 0.952230 | 0.361115 | ZNF418 (margin: ZNF418; ZNF256) | 5.33 | 1.55e-04 | 2.71e-03 |
|  | 1 | 115631930 | 115632727 | PDFSTATSENSEMBLUCSC | 798 | 10 | 3628 | island | 0.015525 | 0.513893 | TSPAN2 (margin: RP4-666F24.3; TSPAN2) | 5.32 | 1.08e-05 | 4.82e-04 |
|  | 8 | 143609379 | 143609637 | PDFSTATSENSEMBLUCSC | 259 | 4 | 39465 | gene;island | 0.654795 | 0.986766 | Not queried | 5.32 | 2.86e-02 | 6.12e-02 |
|  | 6 | 32522400 | 32522905 | PDFSTATSENSEMBLUCSC | 506 | 6 | 34116 | gene | 0.070519 | 0.539646 | Not queried | 5.32 | 1.52e-02 | 5.36e-02 |
|  | 17 | 32909042 | 32909375 | PDFSTATSENSEMBLUCSC | 334 | 4 | 16516 | gene;island | 0.302839 | 0.909662 | Not queried | 5.32 | 2.86e-02 | 6.12e-02 |
|  | 10 | 18629497 | 18629677 | PDFSTATSENSEMBLUCSC | 181 | 6 | 4522 | gene | 0.440669 | 0.959524 | CACNB2 (margin: CACNB2) | 5.32 | 2.16e-03 | 1.45e-02 |
|  | 17 | 72352688 | 72352968 | PDFSTATSENSEMBLUCSC | 281 | 4 | 16753 | gene;island | 0.498593 | 0.979005 | Not queried | 5.32 | 2.86e-02 | 6.12e-02 |
|  | 7 | 89783435 | 89783680 | PDFSTATSENSEMBLUCSC | 246 | 6 | 37695 | tss | 0.486059 | 0.018391 | Not queried | 5.32 | 4.11e-02 | 8.14e-02 |
|  | 5 | 88179379 | 88179539 | PDFSTATSENSEMBLUCSC | 161 | 4 | 32169 | tss | 0.192156 | 0.012545 | Not queried | 5.32 | 2.86e-02 | 6.12e-02 |
|  | 19 | 10627706 | 10628183 | PDFSTATSENSEMBLUCSC | 478 | 4 | 19814 | gene;island | 0.023408 | 0.457504 | Not queried | 5.32 | 2.86e-02 | 6.12e-02 |
|  | 19 | 37825307 | 37825446 | PDFSTATSENSEMBLUCSC | 140 | 6 | 20736 | tss;island | 0.883959 | 0.465372 | HKR1 (margin: HKR1; CTD-3220F14.2) | 5.32 | 2.16e-03 | 1.45e-02 |
|  | 6 | 42110740 | 42111005 | PDFSTATSENSEMBLUCSC | 266 | 5 | 34978 | tss;island | 0.019851 | 0.292652 | Not queried | 5.32 | 1.51e-01 | 2.11e-01 |
|  | 6 | 71666803 | 71666858 | PDFSTATSENSEMBLUCSC | 56 | 4 | 35060 | tss | 0.434896 | 0.960597 | Not queried | 5.32 | 2.86e-02 | 6.12e-02 |
|  | 13 | 20767498 | 20768560 | PDFSTATSENSEMBLUCSC | 1063 | 13 | 11329 | tss;island | 0.238638 | 0.959495 | GJB2 (margin: LINC00556; GJB2) | 5.32 | 5.77e-06 | 3.56e-04 |
|  | 5 | 59189649 | 59189934 | PDFSTATSENSEMBLUCSC | 286 | 8 | 32079 | tss | 0.726217 | 0.054985 | PDE4D (margin: PDE4D) | 5.31 | 1.55e-04 | 2.71e-03 |
|  | 2 | 162930666 | 162931175 | PDFSTATSENSEMBLUCSC | 510 | 5 | 24825 | island | 0.356066 | 0.016224 | Not queried | 5.31 | 2.22e-01 | 2.76e-01 |
|  | 10 | 7409524 | 7409783 | PDFSTATSENSEMBLUCSC | 260 | 5 | 4481 | gene;island | 0.662286 | 0.983266 | SFMBT2 (margin: SFMBT2) | 5.31 | 7.94e-03 | 3.19e-02 |
|  | 11 | 64937570 | 64937697 | PDFSTATSENSEMBLUCSC | 128 | 6 | 7419 | tss | 0.935295 | 0.243261 | SPDYC (margin: SPDYC; CAPN1; AP003068.17; AP003068.18; PGAM1P8; AP003068.23) | 5.31 | 2.16e-03 | 1.45e-02 |
|  | 8 | 110656096 | 110656442 | PDFSTATSENSEMBLUCSC | 347 | 5 | 39399 | gene;tss;island | 0.008485 | 0.178205 | Not queried | 5.31 | 5.56e-02 | 1.00e-01 |
|  | 4 | 140201065 | 140201433 | PDFSTATSENSEMBLUCSC | 369 | 4 | 30131 | gene | 0.160060 | 0.917450 | Not queried | 5.31 | 2.86e-02 | 6.12e-02 |
|  | 11 | 65405268 | 65405903 | PDFSTATSENSEMBLUCSC | 636 | 12 | 8472 | island | 0.029097 | 0.538585 | PCNXL3; MIR4690; SIPA1 (margin: PCNXL3; MIR4690; SIPA1; MIR4489) | 5.31 | 4.96e-04 | 6.47e-03 |
|  | 1 | 225616198 | 225616668 | PDFSTATSENSEMBLUCSC | 471 | 4 | 2279 | tss;island | 0.002348 | 0.015651 | Not queried | 5.31 | 2.86e-02 | 6.12e-02 |
|  | 7 | 48019388 | 48019713 | PDFSTATSENSEMBLUCSC | 326 | 4 | 37590 | tss | 0.801801 | 0.109507 | Not queried | 5.31 | 2.00e-01 | 2.55e-01 |
|  | 17 | 71434003 | 71434142 | PDFSTATSENSEMBLUCSC | 140 | 4 | 16747 | gene;island | 0.076423 | 0.761241 | Not queried | 5.31 | 2.00e-01 | 2.55e-01 |
|  | 9 | 129375812 | 129376185 | PDFSTATSENSEMBLUCSC | 374 | 4 | 40838 | tss;island | 0.379545 | 0.013871 | Not queried | 5.30 | 2.86e-02 | 6.12e-02 |
|  | 1 | 18967220 | 18967570 | PDFSTATSENSEMBLUCSC | 351 | 4 | 274 | gene;island | 0.815937 | 0.051667 | Not queried | 5.30 | 5.71e-02 | 1.00e-01 |
|  | 3 | 133748115 | 133748809 | PDFSTATSENSEMBLUCSC | 695 | 7 | 27953 | gene;island | 0.429992 | 0.018811 | Not queried | 5.30 | 1.75e-02 | 5.79e-02 |
|  | 2 | 109745210 | 109745841 | PDFSTATSENSEMBLUCSC | 632 | 8 | 22822 | gene;tss;island | 0.268846 | 0.921915 | SH3RF3; SH3RF3-AS1 (margin: SH3RF3; SH3RF3-AS1) | 5.30 | 1.09e-03 | 1.10e-02 |
|  | 5 | 54281437 | 54281733 | PDFSTATSENSEMBLUCSC | 297 | 4 | 32062 | tss | 0.016914 | 0.428328 | Not queried | 5.30 | 2.86e-02 | 6.12e-02 |
|  | 12 | 45269318 | 45269790 | PDFSTATSENSEMBLUCSC | 473 | 7 | 9100 | gene;island | 0.289309 | 0.020361 | NELL2 (margin: NELL2; Y\_RNA) | 5.30 | 1.11e-02 | 4.14e-02 |
|  | 4 | 8271196 | 8271507 | PDFSTATSENSEMBLUCSC | 312 | 7 | 30917 | island | 0.451292 | 0.964515 | HTRA3 (margin: HTRA3) | 5.30 | 5.83e-04 | 6.47e-03 |
|  | 11 | 128775513 | 128775750 | PDFSTATSENSEMBLUCSC | 238 | 7 | 6969 | gene | 0.007703 | 0.158108 | KCNJ5; C11orf45 (margin: KCNJ5; C11orf45) | 5.30 | 5.83e-04 | 6.47e-03 |
|  | 20 | 30639705 | 30640041 | PDFSTATSENSEMBLUCSC | 337 | 8 | 25671 | tss | 0.355977 | 0.947828 | RNA5SP482; HCK (margin: RNA5SP482; HCK) | 5.30 | 1.09e-03 | 1.10e-02 |
|  | 15 | 65341703 | 65341995 | PDFSTATSENSEMBLUCSC | 293 | 5 | 13378 | gene | 0.240295 | 0.005140 | SLC51B (margin: SLC51B; RASL12) | 5.29 | 7.94e-03 | 3.19e-02 |
|  | 6 | 166401536 | 166401700 | PDFSTATSENSEMBLUCSC | 165 | 5 | 35333 | tss | 0.159742 | 0.003473 | PDE10A; LINC00473 (margin: PDE10A; LINC00473) | 5.29 | 7.94e-03 | 3.19e-02 |
|  | 1 | 156051324 | 156051681 | PDFSTATSENSEMBLUCSC | 358 | 4 | 813 | gene | 0.707019 | 0.151042 | Not queried | 5.29 | 5.71e-02 | 1.00e-01 |
|  | 7 | 27199726 | 27199956 | PDFSTATSENSEMBLUCSC | 231 | 5 | 38355 | island | 0.698292 | 0.989795 | RP1-170O19.21 (margin: HOXA-AS3; RP1-170O19.21; HOXA-AS4; HOXA3; HOXA6; HOXA7; HOXA9; RP1-170O19.20; MIR196B; HOXA10) | 5.29 | 7.94e-03 | 3.19e-02 |
|  | 15 | 31175309 | 31175522 | PDFSTATSENSEMBLUCSC | 214 | 4 | 13979 | island | 0.413075 | 0.967645 | Not queried | 5.29 | 2.86e-02 | 6.12e-02 |
|  | 6 | 127664674 | 127664950 | PDFSTATSENSEMBLUCSC | 277 | 6 | 35216 | tss;island | 0.027107 | 0.464196 | ECHDC1 (margin: YWHAZP4; ECHDC1) | 5.29 | 2.16e-03 | 1.45e-02 |
|  | 7 | 130125200 | 130126637 | PDFSTATSENSEMBLUCSC | 1438 | 18 | 38921 | island | 0.512617 | 0.020161 | MEST; RP11-2E11.5 (margin: MEST; MIR335; RP11-2E11.5; hsa-mir-335) | 5.29 | 2.11e-04 | 3.49e-03 |
|  | 15 | 29406026 | 29406153 | PDFSTATSENSEMBLUCSC | 128 | 4 | 13267 | gene;island | 0.483564 | 0.955517 | Not queried | 5.29 | 2.86e-02 | 6.12e-02 |
|  | 16 | 960911 | 961353 | PDFSTATSENSEMBLUCSC | 443 | 4 | 14551 | gene;island | 0.571700 | 0.984376 | Not queried | 5.29 | 2.86e-02 | 6.12e-02 |
|  | 17 | 41176744 | 41177097 | PDFSTATSENSEMBLUCSC | 354 | 4 | 17382 | tss;island | 0.512442 | 0.026525 | Not queried | 5.29 | 2.86e-02 | 6.12e-02 |
|  | 19 | 8669959 | 8670193 | PDFSTATSENSEMBLUCSC | 235 | 4 | 19797 | gene;island | 0.444901 | 0.975707 | Not queried | 5.29 | 2.86e-02 | 6.12e-02 |
|  | 7 | 148394842 | 148395181 | PDFSTATSENSEMBLUCSC | 340 | 4 | 37960 | tss;island | 0.284607 | 0.857713 | Not queried | 5.28 | 1.14e-01 | 1.68e-01 |
|  | 11 | 3239953 | 3240399 | PDFSTATSENSEMBLUCSC | 447 | 6 | 6478 | gene;island | 0.046947 | 0.794759 | MRGPRG-AS1; MRGPRG (margin: MRGPRG-AS1; AC109309.4; MRGPRG; MRGPRE) | 5.28 | 2.16e-03 | 1.45e-02 |
|  | 17 | 7341436 | 7341641 | PDFSTATSENSEMBLUCSC | 206 | 4 | 17083 | tss;island | 0.212610 | 0.940031 | Not queried | 5.28 | 2.86e-02 | 6.12e-02 |
|  | 19 | 418899 | 419190 | PDFSTATSENSEMBLUCSC | 292 | 5 | 19626 | gene;island | 0.440726 | 0.971018 | SHC2 (margin: C2CD4C; SHC2) | 5.28 | 7.94e-03 | 3.19e-02 |
|  | 17 | 33775295 | 33775779 | PDFSTATSENSEMBLUCSC | 485 | 5 | 16520 | gene | 0.314494 | 0.966579 | SLFN13 (margin: SLFN13; RP11-1094M14.7) | 5.28 | 7.94e-03 | 3.19e-02 |
|  | 21 | 47918542 | 47918727 | PDFSTATSENSEMBLUCSC | 186 | 4 | 26480 | gene;island | 0.548727 | 0.985727 | Not queried | 5.27 | 1.14e-01 | 1.68e-01 |
|  | 4 | 77355817 | 77356250 | PDFSTATSENSEMBLUCSC | 434 | 5 | 30449 | tss | 0.281378 | 0.012750 | SHROOM3 (margin: SHROOM3) | 5.27 | 7.94e-03 | 3.19e-02 |
|  | 13 | 80915061 | 80915130 | PDFSTATSENSEMBLUCSC | 70 | 4 | 11801 | island | 0.405809 | 0.018823 | Not queried | 5.27 | 2.86e-02 | 6.12e-02 |
|  | 4 | 185940923 | 185941304 | PDFSTATSENSEMBLUCSC | 382 | 5 | 30207 | gene;island | 0.397556 | 0.951253 | HELT (margin: HELT) | 5.27 | 7.94e-03 | 3.19e-02 |
|  | 8 | 24813866 | 24814137 | PDFSTATSENSEMBLUCSC | 272 | 4 | 40162 | island | 0.531986 | 0.971489 | Not queried | 5.27 | 2.86e-02 | 6.12e-02 |
|  | 2 | 103235258 | 103235720 | PDFSTATSENSEMBLUCSC | 463 | 5 | 23549 | tss;island | 0.738229 | 0.009197 | Not queried | 5.27 | 3.17e-02 | 6.50e-02 |
|  | 5 | 180230113 | 180230146 | PDFSTATSENSEMBLUCSC | 34 | 4 | 32561 | tss | 0.048833 | 0.648475 | Not queried | 5.27 | 2.86e-02 | 6.12e-02 |
|  | 6 | 72595944 | 72596566 | PDFSTATSENSEMBLUCSC | 623 | 8 | 35062 | tss | 0.636975 | 0.228353 | Not queried | 5.27 | 2.07e-02 | 6.12e-02 |
|  | 14 | 76451771 | 76452033 | PDFSTATSENSEMBLUCSC | 263 | 4 | 12523 | tss | 0.002222 | 0.045431 | Not queried | 5.27 | 2.00e-01 | 2.55e-01 |
|  | 11 | 108408950 | 108408981 | PDFSTATSENSEMBLUCSC | 32 | 4 | 7673 | tss | 0.611901 | 0.987295 | Not queried | 5.27 | 5.71e-02 | 1.00e-01 |
|  | 1 | 17446027 | 17446162 | PDFSTATSENSEMBLUCSC | 136 | 5 | 1291 | tss | 0.434895 | 0.020577 | PADI2 (margin: PADI2) | 5.27 | 7.94e-03 | 3.19e-02 |
|  | 15 | 64443650 | 64444055 | PDFSTATSENSEMBLUCSC | 406 | 10 | 14212 | island | 0.506168 | 0.019056 | SNX22 (margin: SNX1; SNX22; PPIB) | 5.26 | 8.93e-03 | 3.45e-02 |
|  | 2 | 154333481 | 154334077 | PDFSTATSENSEMBLUCSC | 597 | 9 | 24791 | island | 0.245991 | 0.005185 | AC012501.2; RPRM (margin: AC012501.2; RPRM) | 5.26 | 8.23e-05 | 2.06e-03 |
|  | 11 | 826837 | 827613 | PDFSTATSENSEMBLUCSC | 777 | 7 | 7885 | island | 0.434926 | 0.019704 | PNPLA2; EFCAB4A; AP006621.8 (margin: PNPLA2; EFCAB4A; CD151; AP006621.8; POLR2L) | 5.26 | 5.83e-04 | 6.47e-03 |
|  | 20 | 57462798 | 57464129 | PDFSTATSENSEMBLUCSC | 1332 | 37 | 25473 | gene;tss;island | 0.577175 | 0.978901 | GNAS; RP1-309F20.3 (margin: GNAS; RP1-309F20.3; RP1-309F20.4) | 5.26 | 1.34e-17 | 6.55e-14 |
|  | 15 | 78423530 | 78424152 | PDFSTATSENSEMBLUCSC | 623 | 9 | 14345 | island | 0.547779 | 0.032416 | Not queried | 5.26 | 1.88e-02 | 6.12e-02 |
|  | 15 | 100890907 | 100890996 | PDFSTATSENSEMBLUCSC | 90 | 4 | 13928 | tss;island | 0.090910 | 0.546429 | Not queried | 5.26 | 2.00e-01 | 2.55e-01 |
|  | 20 | 61924240 | 61924423 | PDFSTATSENSEMBLUCSC | 184 | 4 | 25895 | tss;island | 0.199261 | 0.871263 | Not queried | 5.25 | 2.86e-02 | 6.12e-02 |
|  | 6 | 27782909 | 27783226 | PDFSTATSENSEMBLUCSC | 318 | 4 | 33620 | gene | 0.158889 | 0.793880 | Not queried | 5.25 | 5.71e-02 | 1.00e-01 |
|  | 4 | 5021084 | 5021328 | PDFSTATSENSEMBLUCSC | 245 | 8 | 30875 | island | 0.385327 | 0.974312 | CYTL1 (margin: CYTL1) | 5.25 | 3.11e-04 | 4.77e-03 |
|  | 13 | 96742905 | 96743170 | PDFSTATSENSEMBLUCSC | 266 | 4 | 11831 | island | 0.431484 | 0.024507 | Not queried | 5.25 | 2.86e-02 | 6.12e-02 |
|  | 1 | 205011906 | 205012139 | PDFSTATSENSEMBLUCSC | 234 | 4 | 2200 | tss | 0.190472 | 0.842795 | Not queried | 5.25 | 2.86e-02 | 6.12e-02 |
|  | 12 | 48592270 | 48592818 | PDFSTATSENSEMBLUCSC | 549 | 5 | 10367 | island | 0.352573 | 0.947188 | Not queried | 5.25 | 5.56e-02 | 1.00e-01 |
|  | 12 | 114838567 | 114839007 | PDFSTATSENSEMBLUCSC | 441 | 5 | 9324 | gene;island | 0.313676 | 0.968563 | TBX5 (margin: TBX5-AS1; TBX5) | 5.24 | 7.94e-03 | 3.19e-02 |
|  | 12 | 97300410 | 97300582 | PDFSTATSENSEMBLUCSC | 173 | 4 | 9932 | tss;island | 0.059347 | 0.695121 | Not queried | 5.24 | 2.86e-02 | 6.12e-02 |
|  | 1 | 151763548 | 151764147 | PDFSTATSENSEMBLUCSC | 600 | 6 | 1932 | tss;island | 0.328046 | 0.009567 | Not queried | 5.24 | 1.52e-02 | 5.36e-02 |
|  | 5 | 134073430 | 134073579 | PDFSTATSENSEMBLUCSC | 150 | 5 | 32296 | tss;island | 0.772581 | 0.083759 | Not queried | 5.24 | 3.17e-02 | 6.50e-02 |
|  | 8 | 81397601 | 81397876 | PDFSTATSENSEMBLUCSC | 276 | 5 | 39782 | tss;island | 0.048556 | 0.003771 | ZBTB10; Y\_RNA (margin: ZBTB10; Y\_RNA) | 5.24 | 7.94e-03 | 3.19e-02 |
|  | 21 | 42879998 | 42880107 | PDFSTATSENSEMBLUCSC | 110 | 4 | 26558 | tss;island | 0.361176 | 0.016582 | Not queried | 5.24 | 2.86e-02 | 6.12e-02 |
|  | 4 | 981485 | 981630 | PDFSTATSENSEMBLUCSC | 146 | 4 | 29853 | gene;island | 0.020689 | 0.334234 | Not queried | 5.24 | 2.86e-02 | 6.12e-02 |
|  | 5 | 126409007 | 126409573 | PDFSTATSENSEMBLUCSC | 567 | 12 | 33065 | island | 0.550185 | 0.031769 | Not queried | 5.24 | 2.05e-02 | 6.12e-02 |
|  | 6 | 26043546 | 26043820 | PDFSTATSENSEMBLUCSC | 275 | 4 | 33599 | gene | 0.178357 | 0.870634 | Not queried | 5.24 | 2.86e-02 | 6.12e-02 |
|  | 17 | 72667005 | 72667394 | PDFSTATSENSEMBLUCSC | 390 | 5 | 18721 | island | 0.943335 | 0.195762 | Not queried | 5.24 | 1.51e-01 | 2.11e-01 |
|  | 15 | 45314789 | 45314943 | PDFSTATSENSEMBLUCSC | 155 | 4 | 13643 | tss;island | 0.655373 | 0.067405 | Not queried | 5.24 | 2.86e-02 | 6.12e-02 |
|  | 1 | 214360574 | 214360753 | PDFSTATSENSEMBLUCSC | 180 | 4 | 4197 | island | 0.575388 | 0.955065 | Not queried | 5.24 | 1.14e-01 | 1.68e-01 |
|  | 6 | 150311305 | 150312368 | PDFSTATSENSEMBLUCSC | 1064 | 9 | 36564 | island | 0.184427 | 0.962426 | (margin: RP11-472G23.3; BTF3P10; RAET1K) | 5.24 | 4.11e-05 | 1.17e-03 |
|  | 8 | 24772137 | 24772435 | PDFSTATSENSEMBLUCSC | 299 | 7 | 39605 | tss | 0.667441 | 0.981603 | NEFM; GS1-72M22.1 (margin: NEFM; RP11-624C23.1; GS1-72M22.1) | 5.23 | 5.83e-04 | 6.47e-03 |
|  | 11 | 3240068 | 3240399 | PDFSTATSENSEMBLUCSC | 332 | 5 | 7093 | tss | 0.053077 | 0.678098 | MRGPRG-AS1; MRGPRG (margin: MRGPRG-AS1; AC109309.4; MRGPRG; MRGPRE) | 5.23 | 7.94e-03 | 3.19e-02 |
|  | 11 | 46408262 | 46408584 | PDFSTATSENSEMBLUCSC | 323 | 4 | 7278 | tss;island | 0.401654 | 0.953711 | Not queried | 5.23 | 5.71e-02 | 1.00e-01 |
|  | 19 | 474994 | 475344 | PDFSTATSENSEMBLUCSC | 351 | 7 | 20272 | tss | 0.552802 | 0.973316 | Not queried | 5.23 | 1.75e-02 | 5.79e-02 |
|  | 12 | 54133422 | 54133730 | PDFSTATSENSEMBLUCSC | 309 | 5 | 10474 | island | 0.661846 | 0.976949 | Not queried | 5.23 | 5.56e-02 | 1.00e-01 |
|  | 4 | 165304259 | 165304540 | PDFSTATSENSEMBLUCSC | 282 | 8 | 31404 | island | 0.287069 | 0.952757 | MARCH1 (margin: MARCH1) | 5.23 | 3.11e-04 | 4.77e-03 |
|  | 4 | 165304443 | 165304540 | PDFSTATSENSEMBLUCSC | 98 | 6 | 30663 | tss | 0.318970 | 0.952757 | MARCH1 (margin: MARCH1) | 5.23 | 2.16e-03 | 1.45e-02 |
|  | 19 | 22817176 | 22817496 | PDFSTATSENSEMBLUCSC | 321 | 4 | 19965 | gene | 0.483883 | 0.091587 | Not queried | 5.23 | 2.86e-02 | 6.12e-02 |
|  | 3 | 55521351 | 55521529 | PDFSTATSENSEMBLUCSC | 179 | 5 | 28402 | tss;island | 0.234347 | 0.008240 | WNT5A-AS1; WNT5A (margin: WNT5A-AS1; WNT5A; RP11-875H7.5) | 5.23 | 7.94e-03 | 3.19e-02 |
|  | 5 | 59189375 | 59189934 | PDFSTATSENSEMBLUCSC | 560 | 10 | 31625 | gene;island | 0.726217 | 0.069314 | PDE4D (margin: PDE4D) | 5.23 | 3.25e-04 | 4.91e-03 |
|  | 5 | 180230022 | 180230146 | PDFSTATSENSEMBLUCSC | 125 | 5 | 31946 | gene;island | 0.047511 | 0.646353 | Not queried | 5.23 | 5.56e-02 | 1.00e-01 |
|  | 11 | 1660843 | 1661201 | PDFSTATSENSEMBLUCSC | 359 | 4 | 6426 | gene | 0.060849 | 0.709396 | Not queried | 5.23 | 2.86e-02 | 6.12e-02 |
|  | 17 | 80797948 | 80798078 | PDFSTATSENSEMBLUCSC | 131 | 7 | 17781 | tss | 0.336913 | 0.015865 | TBCD; ZNF750 (margin: TBCD; ZNF750) | 5.23 | 5.83e-04 | 6.47e-03 |
|  | 14 | 94255361 | 94255513 | PDFSTATSENSEMBLUCSC | 153 | 4 | 12576 | tss;island | 0.254282 | 0.013235 | Not queried | 5.23 | 2.86e-02 | 6.12e-02 |
|  | 11 | 46366643 | 46367100 | PDFSTATSENSEMBLUCSC | 458 | 8 | 6598 | gene;island | 0.185126 | 0.901455 | DGKZ (margin: DGKZ) | 5.22 | 1.55e-04 | 2.71e-03 |
|  | 1 | 23751134 | 23751210 | PDFSTATSENSEMBLUCSC | 77 | 4 | 313 | gene | 0.668065 | 0.043896 | Not queried | 5.22 | 2.86e-02 | 6.12e-02 |
|  | 7 | 99149191 | 99149627 | PDFSTATSENSEMBLUCSC | 437 | 5 | 37129 | gene | 0.047253 | 0.654433 | Not queried | 5.22 | 9.52e-02 | 1.53e-01 |
|  | 7 | 94953653 | 94954202 | PDFSTATSENSEMBLUCSC | 550 | 9 | 38677 | island | 0.491455 | 0.977226 | PON1 (margin: PON1) | 5.22 | 4.11e-05 | 1.17e-03 |
|  | 18 | 53255401 | 53255894 | PDFSTATSENSEMBLUCSC | 494 | 4 | 19527 | island | 0.050448 | 0.579228 | Not queried | 5.22 | 2.86e-02 | 6.12e-02 |
|  | 10 | 99530944 | 99531416 | PDFSTATSENSEMBLUCSC | 473 | 5 | 4700 | gene;island | 0.420866 | 0.969401 | Not queried | 5.22 | 5.56e-02 | 1.00e-01 |
|  | 4 | 48988015 | 48988253 | PDFSTATSENSEMBLUCSC | 239 | 6 | 30387 | tss | 0.432403 | 0.958524 | Not queried | 5.22 | 1.52e-02 | 5.36e-02 |
|  | 6 | 33043072 | 33043220 | PDFSTATSENSEMBLUCSC | 149 | 4 | 34888 | tss | 0.046103 | 0.703955 | Not queried | 5.22 | 5.71e-02 | 1.00e-01 |
|  | 3 | 145968620 | 145968726 | PDFSTATSENSEMBLUCSC | 107 | 6 | 27983 | gene;island | 0.171166 | 0.007655 | PLSCR4 (margin: PLSCR4) | 5.22 | 8.66e-03 | 3.36e-02 |
|  | 7 | 98972104 | 98972197 | PDFSTATSENSEMBLUCSC | 94 | 6 | 37752 | tss;island | 0.442184 | 0.030038 | ARPC1A; ARPC1B (margin: ARPC1A; ARPC1B) | 5.22 | 2.16e-03 | 1.45e-02 |
|  | 12 | 9227173 | 9227320 | PDFSTATSENSEMBLUCSC | 148 | 5 | 9051 | gene | 0.044872 | 0.490089 | A2M (margin: A2M-AS1; LINC00612; A2M) | 5.22 | 7.94e-03 | 3.19e-02 |
|  | 18 | 63417605 | 63417783 | PDFSTATSENSEMBLUCSC | 179 | 4 | 19122 | gene;tss;island | 0.778743 | 0.102891 | Not queried | 5.22 | 2.86e-02 | 6.12e-02 |
|  | 19 | 54584636 | 54585167 | PDFSTATSENSEMBLUCSC | 532 | 4 | 21048 | tss | 0.193323 | 0.878606 | Not queried | 5.22 | 2.86e-02 | 6.12e-02 |
|  | 5 | 181726 | 182028 | PDFSTATSENSEMBLUCSC | 303 | 4 | 31496 | gene;island | 0.356387 | 0.974049 | Not queried | 5.22 | 2.86e-02 | 6.12e-02 |
|  | 2 | 106015594 | 106015869 | PDFSTATSENSEMBLUCSC | 276 | 6 | 23555 | tss | 0.636350 | 0.079987 | Not queried | 5.21 | 1.32e-01 | 1.89e-01 |
|  | 5 | 177099102 | 177099378 | PDFSTATSENSEMBLUCSC | 277 | 4 | 33461 | island | 0.303999 | 0.017772 | Not queried | 5.21 | 2.86e-02 | 6.12e-02 |
|  | 5 | 134827136 | 134827820 | PDFSTATSENSEMBLUCSC | 685 | 6 | 33137 | island | 0.919573 | 0.181944 | Not queried | 5.21 | 1.80e-01 | 2.45e-01 |
|  | 8 | 143545214 | 143545574 | PDFSTATSENSEMBLUCSC | 361 | 5 | 40635 | island | 0.220458 | 0.878272 | Not queried | 5.21 | 1.59e-02 | 5.36e-02 |
|  | 11 | 6947353 | 6947647 | PDFSTATSENSEMBLUCSC | 295 | 9 | 7118 | tss | 0.235941 | 0.739429 | Not queried | 5.21 | 1.88e-02 | 6.12e-02 |
|  | 19 | 39989234 | 39989592 | PDFSTATSENSEMBLUCSC | 359 | 8 | 21999 | island | 0.070824 | 0.610580 | DLL3 (margin: TIMM50; DLL3) | 5.21 | 6.22e-04 | 6.71e-03 |
|  | 22 | 45809596 | 45810043 | PDFSTATSENSEMBLUCSC | 448 | 8 | 26904 | gene | 0.017408 | 0.288738 | RIBC2; SMC1B (margin: RIBC2; SMC1B) | 5.21 | 3.11e-04 | 4.77e-03 |
|  | 15 | 65203834 | 65204146 | PDFSTATSENSEMBLUCSC | 313 | 6 | 14222 | island | 0.409145 | 0.019701 | AC069368.3; ANKDD1A (margin: AC069368.3; ANKDD1A; RN7SL348P) | 5.21 | 2.16e-03 | 1.45e-02 |
|  | 10 | 5566543 | 5566908 | PDFSTATSENSEMBLUCSC | 366 | 6 | 4926 | tss;island | 0.197042 | 0.886065 | CALML3; CALML3-AS1; RP11-116G8.5 (margin: CALML3; CALML3-AS1; RP11-116G8.5) | 5.21 | 2.16e-03 | 1.45e-02 |
|  | 6 | 117198428 | 117198743 | PDFSTATSENSEMBLUCSC | 316 | 4 | 34453 | gene | 0.581754 | 0.030773 | Not queried | 5.20 | 2.86e-02 | 6.12e-02 |
|  | 4 | 154125208 | 154125720 | PDFSTATSENSEMBLUCSC | 513 | 9 | 30152 | gene | 0.970704 | 0.366064 | TRIM2 (margin: TRIM2) | 5.20 | 7.82e-04 | 8.17e-03 |
|  | 7 | 135433610 | 135433790 | PDFSTATSENSEMBLUCSC | 181 | 6 | 37921 | tss | 0.974190 | 0.558042 | Not queried | 5.20 | 1.52e-02 | 5.36e-02 |
|  | 3 | 184971652 | 184971844 | PDFSTATSENSEMBLUCSC | 193 | 4 | 28068 | gene;island | 0.024803 | 0.419923 | Not queried | 5.20 | 2.86e-02 | 6.12e-02 |
|  | 19 | 53662261 | 53662723 | PDFSTATSENSEMBLUCSC | 463 | 9 | 22369 | island | 0.892812 | 0.281867 | ZNF347; ZNF665 (margin: CTD-2245F17.2; ZNF347; ZNF665) | 5.20 | 8.23e-05 | 2.06e-03 |
|  | 12 | 15114233 | 15114494 | PDFSTATSENSEMBLUCSC | 262 | 4 | 9068 | gene | 0.040515 | 0.601882 | Not queried | 5.19 | 2.86e-02 | 6.12e-02 |
|  | 1 | 201915757 | 201915999 | PDFSTATSENSEMBLUCSC | 243 | 7 | 2170 | tss | 0.453973 | 0.021186 | LMOD1 (margin: TIMM17A; LMOD1) | 5.19 | 1.17e-03 | 1.13e-02 |
|  | 11 | 843897 | 844400 | PDFSTATSENSEMBLUCSC | 504 | 8 | 7033 | tss | 0.676259 | 0.056119 | TSPAN4; POLR2L (margin: EFCAB4A; CD151; TSPAN4; AP006621.8; POLR2L) | 5.19 | 1.55e-04 | 2.71e-03 |
|  | 4 | 1537622 | 1537799 | PDFSTATSENSEMBLUCSC | 178 | 4 | 30790 | island | 0.284811 | 0.919805 | Not queried | 5.19 | 3.43e-01 | 3.84e-01 |
|  | 16 | 68481813 | 68482277 | PDFSTATSENSEMBLUCSC | 465 | 5 | 14816 | gene;island | 0.647321 | 0.048241 | SMPD3 (margin: SMPD3) | 5.19 | 7.94e-03 | 3.19e-02 |
|  | 10 | 95517382 | 95517562 | PDFSTATSENSEMBLUCSC | 181 | 5 | 5242 | tss | 0.579254 | 0.946187 | Not queried | 5.19 | 9.52e-02 | 1.53e-01 |
|  | 6 | 125684510 | 125684960 | PDFSTATSENSEMBLUCSC | 451 | 8 | 36460 | island | 0.427742 | 0.023079 | (margin: RP11-735G4.1) | 5.19 | 1.55e-04 | 2.71e-03 |
|  | 3 | 173113376 | 173113576 | PDFSTATSENSEMBLUCSC | 201 | 4 | 29670 | island | 0.314624 | 0.019265 | Not queried | 5.19 | 2.86e-02 | 6.12e-02 |
|  | 14 | 69951211 | 69951422 | PDFSTATSENSEMBLUCSC | 212 | 6 | 12481 | tss | 0.480368 | 0.024134 | PLEKHD1 (margin: PLEKHD1) | 5.19 | 2.16e-03 | 1.45e-02 |
|  | 5 | 172661803 | 172662130 | PDFSTATSENSEMBLUCSC | 328 | 4 | 31904 | gene | 0.455808 | 0.964252 | Not queried | 5.19 | 1.14e-01 | 1.68e-01 |
|  | 19 | 15580310 | 15580445 | PDFSTATSENSEMBLUCSC | 136 | 4 | 19908 | gene;island | 0.814966 | 0.985123 | Not queried | 5.19 | 1.14e-01 | 1.68e-01 |
|  | 1 | 184005360 | 184005990 | PDFSTATSENSEMBLUCSC | 631 | 5 | 920 | gene;island | 0.313433 | 0.981122 | Not queried | 5.19 | 3.17e-02 | 6.50e-02 |
|  | 11 | 26353484 | 26353630 | PDFSTATSENSEMBLUCSC | 147 | 5 | 7206 | tss | 0.594606 | 0.025228 | ANO3 (margin: ANO3) | 5.19 | 7.94e-03 | 3.19e-02 |
|  | 1 | 111746873 | 111747324 | PDFSTATSENSEMBLUCSC | 452 | 8 | 3589 | island | 0.440277 | 0.025789 | CHI3L2; DENND2D (margin: CHI3L2; DENND2D) | 5.18 | 1.55e-04 | 2.71e-03 |
|  | 5 | 92918829 | 92918943 | PDFSTATSENSEMBLUCSC | 115 | 6 | 32176 | tss;island | 0.302238 | 0.011722 | Not queried | 5.18 | 1.80e-01 | 2.45e-01 |
|  | 12 | 85306323 | 85306574 | PDFSTATSENSEMBLUCSC | 252 | 4 | 9232 | gene | 0.417982 | 0.959607 | Not queried | 5.18 | 2.86e-02 | 6.12e-02 |
|  | 10 | 131354782 | 131355302 | PDFSTATSENSEMBLUCSC | 521 | 9 | 4826 | gene | 0.712452 | 0.985817 | MGMT (margin: MGMT) | 5.18 | 4.11e-05 | 1.17e-03 |
|  | 14 | 101532886 | 101533127 | PDFSTATSENSEMBLUCSC | 242 | 4 | 13154 | island | 0.265437 | 0.824915 | Not queried | 5.18 | 2.86e-02 | 6.12e-02 |
|  | 15 | 73661642 | 73662002 | PDFSTATSENSEMBLUCSC | 361 | 6 | 13781 | tss | 0.413589 | 0.960301 | HCN4 (margin: HCN4) | 5.18 | 4.33e-03 | 2.52e-02 |
|  | 14 | 105511982 | 105512306 | PDFSTATSENSEMBLUCSC | 325 | 5 | 13228 | island | 0.462706 | 0.013975 | Not queried | 5.17 | 1.51e-01 | 2.11e-01 |
|  | 20 | 50419082 | 50419348 | PDFSTATSENSEMBLUCSC | 267 | 7 | 25826 | tss | 0.972177 | 0.523511 | SALL4 (margin: SALL4) | 5.17 | 5.83e-04 | 6.47e-03 |
|  | 1 | 76262302 | 76262594 | PDFSTATSENSEMBLUCSC | 293 | 4 | 1701 | tss | 0.384534 | 0.958062 | Not queried | 5.17 | 2.86e-02 | 6.12e-02 |
|  | 19 | 14359671 | 14359902 | PDFSTATSENSEMBLUCSC | 232 | 4 | 21677 | island | 0.234076 | 0.008525 | Not queried | 5.17 | 2.86e-02 | 6.12e-02 |
|  | 1 | 19615303 | 19615702 | PDFSTATSENSEMBLUCSC | 400 | 11 | 1302 | tss;island | 0.609638 | 0.977349 | AKR7A3 (margin: RP4-657E11.10; AKR7A3) | 5.17 | 8.51e-05 | 2.07e-03 |
|  | 7 | 33944213 | 33944420 | PDFSTATSENSEMBLUCSC | 208 | 4 | 37533 | tss;island | 0.351216 | 0.938290 | Not queried | 5.17 | 2.86e-02 | 6.12e-02 |
|  | 12 | 108523395 | 108523597 | PDFSTATSENSEMBLUCSC | 203 | 6 | 10767 | island | 0.795360 | 0.034562 | WSCD2 (margin: WSCD2) | 5.17 | 8.66e-03 | 3.36e-02 |
|  | 18 | 43913565 | 43913755 | PDFSTATSENSEMBLUCSC | 191 | 5 | 19256 | tss;island | 0.337040 | 0.011847 | RNF165 (margin: RNF165) | 5.17 | 7.94e-03 | 3.19e-02 |
|  | 19 | 2702863 | 2703086 | PDFSTATSENSEMBLUCSC | 224 | 10 | 20329 | tss;island | 0.742810 | 0.008144 | GNG7 (margin: GNG7; DIRAS1) | 5.16 | 8.93e-03 | 3.45e-02 |
|  | 7 | 155174991 | 155175340 | PDFSTATSENSEMBLUCSC | 350 | 4 | 39065 | island | 0.551080 | 0.977300 | Not queried | 5.16 | 2.00e-01 | 2.55e-01 |
|  | 16 | 30905695 | 30905875 | PDFSTATSENSEMBLUCSC | 181 | 5 | 15197 | tss;island | 0.723482 | 0.041021 | Not queried | 5.16 | 1.59e-02 | 5.36e-02 |
|  | 13 | 27335082 | 27335542 | PDFSTATSENSEMBLUCSC | 461 | 9 | 11356 | tss;island | 0.351129 | 0.955254 | GPR12 (margin: GPR12) | 5.16 | 4.11e-05 | 1.17e-03 |
|  | 8 | 30890108 | 30890632 | PDFSTATSENSEMBLUCSC | 525 | 11 | 39271 | gene;tss | 0.553844 | 0.028674 | WRN; PURG (margin: WRN; PURG) | 5.16 | 1.28e-02 | 4.76e-02 |
|  | 3 | 46933782 | 46933972 | PDFSTATSENSEMBLUCSC | 191 | 4 | 27757 | gene;island | 0.623426 | 0.052196 | Not queried | 5.16 | 2.86e-02 | 6.12e-02 |
|  | 19 | 1753444 | 1753640 | PDFSTATSENSEMBLUCSC | 197 | 6 | 20311 | tss;island | 0.214113 | 0.742492 | Not queried | 5.16 | 6.49e-02 | 1.11e-01 |
|  | 15 | 79296377 | 79296585 | PDFSTATSENSEMBLUCSC | 209 | 4 | 13444 | gene | 0.599789 | 0.974396 | Not queried | 5.16 | 2.86e-02 | 6.12e-02 |
|  | 6 | 126070013 | 126070499 | PDFSTATSENSEMBLUCSC | 487 | 7 | 35211 | tss;island | 0.016100 | 0.184108 | Not queried | 5.16 | 9.73e-02 | 1.55e-01 |
|  | 5 | 65891878 | 65892109 | PDFSTATSENSEMBLUCSC | 232 | 7 | 32101 | tss | 0.316111 | 0.009449 | MAST4 (margin: MAST4) | 5.16 | 1.17e-03 | 1.13e-02 |
|  | 11 | 46940434 | 46940613 | PDFSTATSENSEMBLUCSC | 180 | 4 | 7282 | tss;island | 0.360216 | 0.746644 | Not queried | 5.16 | 3.43e-01 | 3.84e-01 |
|  | 19 | 7852051 | 7852238 | PDFSTATSENSEMBLUCSC | 188 | 6 | 20407 | tss;island | 0.169891 | 0.931435 | CLEC4GP1 (margin: CLEC4GP1) | 5.16 | 2.16e-03 | 1.45e-02 |
|  | 16 | 89044523 | 89044882 | PDFSTATSENSEMBLUCSC | 360 | 5 | 15418 | tss | 0.052894 | 0.710303 | CBFA2T3 (margin: CTD-2555A7.1; CBFA2T3) | 5.16 | 7.94e-03 | 3.19e-02 |
|  | 8 | 120428418 | 120428692 | PDFSTATSENSEMBLUCSC | 275 | 4 | 40558 | island | 0.020988 | 0.428166 | Not queried | 5.15 | 2.86e-02 | 6.12e-02 |
|  | 1 | 240775075 | 240775401 | PDFSTATSENSEMBLUCSC | 327 | 4 | 1107 | gene | 0.494937 | 0.964848 | Not queried | 5.15 | 2.86e-02 | 6.12e-02 |
|  | 15 | 89920824 | 89921258 | PDFSTATSENSEMBLUCSC | 435 | 10 | 13887 | tss;island | 0.918596 | 0.147375 | Not queried | 5.15 | 8.92e-02 | 1.50e-01 |
|  | 7 | 96654152 | 96654198 | PDFSTATSENSEMBLUCSC | 47 | 4 | 37735 | tss | 0.596755 | 0.044994 | Not queried | 5.15 | 2.86e-02 | 6.12e-02 |
|  | 6 | 124124875 | 124125371 | PDFSTATSENSEMBLUCSC | 497 | 6 | 36455 | island | 0.577329 | 0.046300 | NKAIN2 (margin: NKAIN2) | 5.15 | 8.66e-03 | 3.36e-02 |
|  | 13 | 44361019 | 44361220 | PDFSTATSENSEMBLUCSC | 202 | 5 | 11699 | island | 0.113119 | 0.006501 | RP11-168P13.1; ENOX1 (margin: RP11-168P13.1; ENOX1) | 5.15 | 7.94e-03 | 3.19e-02 |
|  | 3 | 168864101 | 168864313 | PDFSTATSENSEMBLUCSC | 213 | 5 | 28706 | tss | 0.009253 | 0.248934 | MECOM (margin: MECOM) | 5.15 | 7.94e-03 | 3.19e-02 |
|  | 5 | 68787696 | 68788117 | PDFSTATSENSEMBLUCSC | 422 | 8 | 32111 | tss | 0.359692 | 0.022828 | Not queried | 5.15 | 1.48e-02 | 5.36e-02 |
|  | 2 | 33359198 | 33359550 | PDFSTATSENSEMBLUCSC | 353 | 5 | 23298 | tss | 0.127685 | 0.916879 | LTBP1 (margin: LTBP1) | 5.15 | 7.94e-03 | 3.19e-02 |
|  | 1 | 77747875 | 77748221 | PDFSTATSENSEMBLUCSC | 347 | 6 | 1705 | tss | 0.459902 | 0.020755 | AK5 (margin: AK5) | 5.15 | 4.33e-03 | 2.52e-02 |
|  | 20 | 1246703 | 1247058 | PDFSTATSENSEMBLUCSC | 356 | 8 | 25935 | island | 0.317867 | 0.929687 | RAD21L1; SNPH (margin: RAD21L1; SNPH) | 5.15 | 1.55e-04 | 2.71e-03 |
|  | 20 | 1246703 | 1246934 | PDFSTATSENSEMBLUCSC | 232 | 6 | 25536 | tss | 0.429381 | 0.935216 | RAD21L1; SNPH (margin: RAD21L1; SNPH) | 5.15 | 2.16e-03 | 1.45e-02 |
|  | 13 | 29394754 | 29394960 | PDFSTATSENSEMBLUCSC | 207 | 4 | 11644 | island | 0.563355 | 0.047633 | Not queried | 5.15 | 2.86e-02 | 6.12e-02 |
|  | 5 | 100239919 | 100240223 | PDFSTATSENSEMBLUCSC | 305 | 4 | 32197 | tss;island | 0.529388 | 0.976502 | Not queried | 5.15 | 2.86e-02 | 6.12e-02 |
|  | 19 | 55314879 | 55315034 | PDFSTATSENSEMBLUCSC | 156 | 4 | 21065 | tss | 0.284346 | 0.858010 | Not queried | 5.15 | 5.71e-02 | 1.00e-01 |
|  | 13 | 22245333 | 22245730 | PDFSTATSENSEMBLUCSC | 398 | 5 | 11100 | gene | 0.220183 | 0.018572 | Not queried | 5.15 | 3.17e-02 | 6.50e-02 |
|  | 2 | 20866107 | 20866414 | PDFSTATSENSEMBLUCSC | 308 | 8 | 23215 | tss | 0.419811 | 0.983403 | GDF7 (margin: GDF7; AC012065.7; RP11-130L8.1) | 5.14 | 1.55e-04 | 2.71e-03 |
|  | 1 | 1238436 | 1238797 | PDFSTATSENSEMBLUCSC | 362 | 5 | 27 | gene;island | 0.311613 | 0.960958 | ACAP3 (margin: SCNN1D; PUSL1; ACAP3; CPSF3L) | 5.14 | 7.94e-03 | 3.19e-02 |
|  | 9 | 90112515 | 90112711 | PDFSTATSENSEMBLUCSC | 197 | 4 | 40819 | tss;island | 0.075537 | 0.840711 | Not queried | 5.14 | 2.86e-02 | 6.12e-02 |
|  | 1 | 236559257 | 236559675 | PDFSTATSENSEMBLUCSC | 419 | 7 | 1097 | gene;island | 0.027803 | 0.597609 | EDARADD (margin: EDARADD) | 5.14 | 6.99e-03 | 3.19e-02 |
|  | 10 | 128077307 | 128077376 | PDFSTATSENSEMBLUCSC | 70 | 6 | 5433 | tss;island | 0.587569 | 0.979567 | ADAM12 (margin: ADAM12) | 5.14 | 2.16e-03 | 1.45e-02 |
|  | 21 | 46493156 | 46493436 | PDFSTATSENSEMBLUCSC | 281 | 5 | 26592 | tss;island | 0.856554 | 0.151464 | ADARB1; SSR4P1; AP001579.1 (margin: ADARB1; SSR4P1; AP001579.1) | 5.14 | 7.94e-03 | 3.19e-02 |
|  | 2 | 25391505 | 25391911 | PDFSTATSENSEMBLUCSC | 407 | 11 | 24139 | island | 0.503666 | 0.967084 | POMC (margin: RP11-509E16.1; POMC) | 5.14 | 2.84e-06 | 1.96e-04 |
|  | 4 | 108852496 | 108852651 | PDFSTATSENSEMBLUCSC | 156 | 7 | 30535 | tss;island | 0.227892 | 0.011491 | CYP2U1; RP11-286E11.1 (margin: CYP2U1; RP11-286E11.1; RP11-286E11.2) | 5.14 | 5.83e-04 | 6.47e-03 |
|  | 14 | 24837072 | 24837184 | PDFSTATSENSEMBLUCSC | 113 | 4 | 12049 | gene;tss;island | 0.364491 | 0.020150 | Not queried | 5.13 | 2.86e-02 | 6.12e-02 |
|  | 6 | 74161911 | 74162142 | PDFSTATSENSEMBLUCSC | 232 | 7 | 36256 | island | 0.023750 | 0.412442 | MB21D1 (margin: MTO1; RNU6-975P; MB21D1) | 5.13 | 5.83e-04 | 6.47e-03 |
|  | 3 | 44903463 | 44903730 | PDFSTATSENSEMBLUCSC | 268 | 5 | 27745 | gene | 0.021293 | 0.428484 | KIF15; TMEM42; MIR564 (margin: KIF15; TMEM42; MIR564) | 5.13 | 7.94e-03 | 3.19e-02 |
|  | 8 | 85094742 | 85094923 | PDFSTATSENSEMBLUCSC | 182 | 4 | 39787 | tss;island | 0.855963 | 0.106231 | Not queried | 5.13 | 2.86e-02 | 6.12e-02 |
|  | 1 | 153508755 | 153508875 | PDFSTATSENSEMBLUCSC | 121 | 4 | 1942 | tss | 0.038660 | 0.599646 | Not queried | 5.13 | 2.86e-02 | 6.12e-02 |
|  | 8 | 33457483 | 33457822 | PDFSTATSENSEMBLUCSC | 340 | 6 | 39642 | tss | 0.394121 | 0.965104 | DUSP26 (margin: RP11-317N12.1; DUSP26) | 5.13 | 2.16e-03 | 1.45e-02 |
|  | 17 | 80185943 | 80186336 | PDFSTATSENSEMBLUCSC | 394 | 6 | 17769 | tss;island | 0.471860 | 0.974856 | SLC16A3 (margin: RP13-516M14.2; SLC16A3; CSNK1D) | 5.12 | 8.66e-03 | 3.36e-02 |
|  | 12 | 12502846 | 12503544 | PDFSTATSENSEMBLUCSC | 699 | 10 | 10246 | island | 0.387336 | 0.019286 | MANSC1 (margin: RP11-757G14.1; LOH12CR1; MANSC1; LOH12CR2) | 5.12 | 2.17e-05 | 8.94e-04 |
|  | 12 | 12503186 | 12503544 | PDFSTATSENSEMBLUCSC | 359 | 8 | 9569 | tss | 0.365928 | 0.019286 | MANSC1 (margin: RP11-757G14.1; LOH12CR1; MANSC1; LOH12CR2) | 5.12 | 1.55e-04 | 2.71e-03 |
|  | 2 | 242751796 | 242752080 | PDFSTATSENSEMBLUCSC | 285 | 4 | 23146 | gene;island | 0.210522 | 0.893517 | Not queried | 5.12 | 3.43e-01 | 3.84e-01 |
|  | 4 | 176986621 | 176986950 | PDFSTATSENSEMBLUCSC | 330 | 7 | 30687 | tss | 0.493372 | 0.010285 | WDR17 (margin: WDR17) | 5.12 | 2.33e-03 | 1.52e-02 |
|  | 8 | 70378380 | 70378760 | PDFSTATSENSEMBLUCSC | 381 | 6 | 39752 | tss | 0.039381 | 0.567749 | SULF1 (margin: SULF1) | 5.12 | 8.66e-03 | 3.36e-02 |
|  | 1 | 18972919 | 18973303 | PDFSTATSENSEMBLUCSC | 385 | 5 | 275 | gene;island | 0.207721 | 0.903599 | Not queried | 5.12 | 5.56e-02 | 1.00e-01 |
|  | 4 | 980604 | 980676 | PDFSTATSENSEMBLUCSC | 73 | 5 | 29852 | gene;tss;island | 0.246837 | 0.011616 | IDUA; DGKQ; SLC26A1 (margin: IDUA; DGKQ; SLC26A1) | 5.12 | 7.94e-03 | 3.19e-02 |
|  | 20 | 30639705 | 30640256 | PDFSTATSENSEMBLUCSC | 552 | 11 | 26089 | island | 0.374674 | 0.945993 | RNA5SP482; HCK (margin: RNA5SP482; HCK) | 5.12 | 5.39e-05 | 1.47e-03 |
|  | 2 | 10861364 | 10861710 | PDFSTATSENSEMBLUCSC | 347 | 8 | 23184 | tss;island | 0.578129 | 0.038452 | Not queried | 5.11 | 2.79e-01 | 3.38e-01 |
|  | 17 | 80291982 | 80292252 | PDFSTATSENSEMBLUCSC | 271 | 4 | 17773 | tss;island | 0.433170 | 0.908272 | Not queried | 5.11 | 1.14e-01 | 1.68e-01 |
|  | 1 | 153651886 | 153652247 | PDFSTATSENSEMBLUCSC | 362 | 4 | 765 | gene;island | 0.465943 | 0.971808 | Not queried | 5.11 | 2.86e-02 | 6.12e-02 |
|  | 16 | 126848 | 127088 | PDFSTATSENSEMBLUCSC | 241 | 5 | 14958 | tss;island | 0.031226 | 0.613298 | Not queried | 5.11 | 2.22e-01 | 2.76e-01 |
|  | 1 | 18956762 | 18957023 | PDFSTATSENSEMBLUCSC | 262 | 4 | 1295 | tss;island | 0.535119 | 0.976998 | Not queried | 5.11 | 2.86e-02 | 6.12e-02 |
|  | 19 | 52490101 | 52490339 | PDFSTATSENSEMBLUCSC | 239 | 9 | 21002 | tss | 0.518917 | 0.014081 | ZNF350 (margin: HCCAT3; ZNF350; ZNF615) | 5.11 | 4.11e-05 | 1.17e-03 |
|  | 5 | 153857824 | 153858102 | PDFSTATSENSEMBLUCSC | 279 | 4 | 32448 | tss | 0.350618 | 0.954138 | Not queried | 5.11 | 2.86e-02 | 6.12e-02 |
|  | 19 | 12624679 | 12624832 | PDFSTATSENSEMBLUCSC | 154 | 6 | 20498 | tss | 0.579203 | 0.982915 | ZNF709 (margin: CTD-3105H18.8; CTD-3105H18.10; CTD-3105H18.9; CTD-3105H18.11; PPIAP20; ZNF709; ZNF564; CTD-2192J16.20) | 5.11 | 2.16e-03 | 1.45e-02 |
|  | 10 | 52834498 | 52835078 | PDFSTATSENSEMBLUCSC | 581 | 5 | 4591 | gene;island | 0.543458 | 0.014095 | Not queried | 5.10 | 5.56e-02 | 1.00e-01 |
|  | 6 | 99281500 | 99281977 | PDFSTATSENSEMBLUCSC | 478 | 5 | 35131 | tss;island | 0.469362 | 0.966429 | Not queried | 5.10 | 9.52e-02 | 1.53e-01 |
|  | 6 | 25992047 | 25992401 | PDFSTATSENSEMBLUCSC | 355 | 4 | 35503 | island | 0.291890 | 0.019617 | Not queried | 5.10 | 1.14e-01 | 1.68e-01 |
|  | 17 | 32907636 | 32907997 | PDFSTATSENSEMBLUCSC | 362 | 7 | 18202 | island | 0.573578 | 0.185917 | TMEM132E; C17orf102 (margin: TMEM132E; C17orf102) | 5.10 | 1.11e-02 | 4.14e-02 |
|  | 5 | 65891878 | 65892187 | PDFSTATSENSEMBLUCSC | 310 | 8 | 32843 | island | 0.285027 | 0.015871 | MAST4 (margin: MAST4) | 5.10 | 6.22e-04 | 6.71e-03 |
|  | 7 | 30810852 | 30810882 | PDFSTATSENSEMBLUCSC | 31 | 5 | 37521 | tss;island | 0.601242 | 0.044448 | INMT-FAM188B; FAM188B (margin: INMT-FAM188B; FAM188B) | 5.10 | 7.94e-03 | 3.19e-02 |
|  | 15 | 75470777 | 75471218 | PDFSTATSENSEMBLUCSC | 442 | 6 | 14325 | island | 0.967945 | 0.643665 | (margin: RPL36AP45) | 5.10 | 2.16e-03 | 1.45e-02 |
|  | 11 | 123525662 | 123525714 | PDFSTATSENSEMBLUCSC | 53 | 4 | 7779 | tss;island | 0.478380 | 0.967063 | Not queried | 5.09 | 2.86e-02 | 6.12e-02 |
|  | 17 | 77478569 | 77478927 | PDFSTATSENSEMBLUCSC | 359 | 5 | 17714 | tss | 0.012887 | 0.304013 | Not queried | 5.09 | 5.56e-02 | 1.00e-01 |
|  | 2 | 207307490 | 207307732 | PDFSTATSENSEMBLUCSC | 243 | 4 | 23836 | tss;island | 0.441978 | 0.929281 | Not queried | 5.09 | 2.86e-02 | 6.12e-02 |
|  | 6 | 158093883 | 158094060 | PDFSTATSENSEMBLUCSC | 178 | 4 | 34525 | gene;island | 0.582635 | 0.973890 | Not queried | 5.09 | 2.86e-02 | 6.12e-02 |
|  | 5 | 7850070 | 7850438 | PDFSTATSENSEMBLUCSC | 369 | 4 | 31556 | gene;island | 0.333997 | 0.870878 | Not queried | 5.09 | 1.14e-01 | 1.68e-01 |
|  | 7 | 154862021 | 154862157 | PDFSTATSENSEMBLUCSC | 137 | 4 | 38009 | tss;island | 0.693138 | 0.175711 | Not queried | 5.08 | 1.14e-01 | 1.68e-01 |
|  | 1 | 247275326 | 247276096 | PDFSTATSENSEMBLUCSC | 771 | 10 | 4414 | island | 0.694229 | 0.049019 | C1orf229 (margin: FGFR3P6; ZNF669; C1orf229; ZNF124) | 5.08 | 5.20e-03 | 2.96e-02 |
|  | 7 | 51384371 | 51384676 | PDFSTATSENSEMBLUCSC | 306 | 7 | 38495 | island | 0.374087 | 0.017654 | COBL (margin: COBL) | 5.08 | 5.83e-04 | 6.47e-03 |
|  | 3 | 10857688 | 10857800 | PDFSTATSENSEMBLUCSC | 113 | 8 | 28153 | tss;island | 0.410025 | 0.960746 | SLC6A11 (margin: SLC6A11) | 5.08 | 1.55e-04 | 2.71e-03 |
|  | 14 | 102026230 | 102026555 | PDFSTATSENSEMBLUCSC | 326 | 10 | 12635 | tss | 0.517699 | 0.974135 | DIO3; DIO3OS (margin: DIO3; DIO3OS) | 5.08 | 1.08e-05 | 4.82e-04 |
|  | 22 | 50524374 | 50524691 | PDFSTATSENSEMBLUCSC | 318 | 5 | 27245 | tss;island | 0.645094 | 0.981709 | Not queried | 5.08 | 5.56e-02 | 1.00e-01 |
|  | 12 | 121570677 | 121571068 | PDFSTATSENSEMBLUCSC | 392 | 4 | 9362 | gene | 0.163598 | 0.866505 | Not queried | 5.08 | 3.43e-01 | 3.84e-01 |
|  | 10 | 133938604 | 133938970 | PDFSTATSENSEMBLUCSC | 367 | 6 | 4849 | gene;island | 0.497038 | 0.956080 | Not queried | 5.08 | 6.49e-02 | 1.11e-01 |
|  | 4 | 93224895 | 93225466 | PDFSTATSENSEMBLUCSC | 572 | 7 | 30499 | tss | 0.525546 | 0.025623 | GRID2; RP11-9B6.1 (margin: GRID2; RP11-9B6.1) | 5.08 | 2.33e-03 | 1.52e-02 |
|  | 6 | 166139905 | 166140706 | PDFSTATSENSEMBLUCSC | 802 | 8 | 36621 | island | 0.263519 | 0.948228 | PDE10A (margin: PDE10A; RNU6-730P) | 5.08 | 1.55e-04 | 2.71e-03 |
|  | 6 | 49681299 | 49681391 | PDFSTATSENSEMBLUCSC | 93 | 5 | 35027 | tss | 0.585130 | 0.982850 | CRISP2 (margin: CRISP2) | 5.07 | 7.94e-03 | 3.19e-02 |
|  | 2 | 154335014 | 154335386 | PDFSTATSENSEMBLUCSC | 373 | 9 | 24792 | island | 0.115355 | 0.011100 | AC012501.2; RPRM (margin: AC012501.2; RPRM) | 5.07 | 4.11e-05 | 1.17e-03 |
|  | 6 | 32365491 | 32366096 | PDFSTATSENSEMBLUCSC | 606 | 7 | 34107 | gene | 0.445272 | 0.971855 | BTNL2 (margin: HCG23; BTNL2) | 5.07 | 6.99e-03 | 3.19e-02 |
|  | 13 | 114774837 | 114775133 | PDFSTATSENSEMBLUCSC | 297 | 5 | 11303 | gene;island | 0.648366 | 0.986697 | Not queried | 5.07 | 9.52e-02 | 1.53e-01 |
|  | 20 | 3776212 | 3776483 | PDFSTATSENSEMBLUCSC | 272 | 8 | 25977 | island | 0.632599 | 0.050435 | CDC25B (margin: CDC25B; CENPB) | 5.07 | 3.11e-04 | 4.77e-03 |
|  | 19 | 3932951 | 3933195 | PDFSTATSENSEMBLUCSC | 245 | 5 | 21354 | island | 0.122736 | 0.675741 | NMRK2 (margin: ATCAY; NMRK2) | 5.07 | 7.94e-03 | 3.19e-02 |
|  | 7 | 122526408 | 122526940 | PDFSTATSENSEMBLUCSC | 533 | 9 | 38875 | island | 0.453150 | 0.020894 | CADPS2 (margin: CADPS2) | 5.07 | 4.11e-05 | 1.17e-03 |
|  | 12 | 107711872 | 107712194 | PDFSTATSENSEMBLUCSC | 323 | 5 | 9975 | tss | 0.136036 | 0.002522 | Not queried | 5.07 | 1.59e-02 | 5.36e-02 |
|  | 6 | 46702683 | 46703422 | PDFSTATSENSEMBLUCSC | 740 | 12 | 34360 | gene | 0.443560 | 0.966600 | PLA2G7 (margin: ANKRD66; PLA2G7) | 5.07 | 2.22e-05 | 9.05e-04 |
|  | 8 | 144640306 | 144640755 | PDFSTATSENSEMBLUCSC | 450 | 9 | 39483 | gene;island | 0.041254 | 0.602544 | GSDMD (margin: GSDMD; RP11-661A12.5; MROH6) | 5.07 | 4.11e-05 | 1.17e-03 |
|  | 22 | 25423531 | 25424194 | PDFSTATSENSEMBLUCSC | 664 | 6 | 27368 | island | 0.453674 | 0.973365 | Not queried | 5.06 | 4.11e-02 | 8.14e-02 |
|  | 5 | 68787696 | 68788173 | PDFSTATSENSEMBLUCSC | 478 | 9 | 32854 | island | 0.338818 | 0.015133 | OCLN; RP11-241G9.3; GUSBP3 (margin: OCLN; RP11-241G9.3; GUSBP3) | 5.06 | 1.06e-02 | 4.01e-02 |
|  | 11 | 35441107 | 35441311 | PDFSTATSENSEMBLUCSC | 205 | 4 | 7245 | tss | 0.444449 | 0.041249 | Not queried | 5.06 | 5.71e-02 | 1.00e-01 |
|  | 4 | 26585218 | 26585903 | PDFSTATSENSEMBLUCSC | 686 | 11 | 30968 | island | 0.023070 | 0.003820 | Not queried | 5.06 | 2.81e-02 | 6.12e-02 |
|  | 19 | 45720771 | 45721076 | PDFSTATSENSEMBLUCSC | 306 | 4 | 20089 | gene;island | 0.471864 | 0.969908 | Not queried | 5.06 | 2.00e-01 | 2.55e-01 |
|  | 19 | 2607726 | 2607903 | PDFSTATSENSEMBLUCSC | 178 | 4 | 19696 | gene;island | 0.303376 | 0.011982 | Not queried | 5.06 | 5.71e-02 | 1.00e-01 |
|  | 15 | 92936678 | 92937030 | PDFSTATSENSEMBLUCSC | 353 | 4 | 13911 | tss;island | 0.481447 | 0.978723 | Not queried | 5.06 | 2.86e-02 | 6.12e-02 |
|  | 6 | 134638742 | 134639020 | PDFSTATSENSEMBLUCSC | 279 | 5 | 34483 | gene | 0.424915 | 0.958450 | SGK1 (margin: SGK1) | 5.06 | 7.94e-03 | 3.19e-02 |
|  | 6 | 29527870 | 29528119 | PDFSTATSENSEMBLUCSC | 250 | 4 | 34748 | tss | 0.200403 | 0.867892 | Not queried | 5.06 | 1.14e-01 | 1.68e-01 |
|  | 8 | 143509263 | 143509556 | PDFSTATSENSEMBLUCSC | 294 | 4 | 40632 | island | 0.421181 | 0.958267 | Not queried | 5.06 | 2.86e-02 | 6.12e-02 |
|  | 20 | 50418952 | 50419348 | PDFSTATSENSEMBLUCSC | 397 | 9 | 26257 | island | 0.983176 | 0.560413 | SALL4 (margin: SALL4) | 5.06 | 8.23e-05 | 2.06e-03 |
|  | 5 | 57878953 | 57879417 | PDFSTATSENSEMBLUCSC | 465 | 7 | 31623 | gene | 0.835931 | 0.065268 | Not queried | 5.05 | 2.62e-02 | 6.12e-02 |
|  | 4 | 20255061 | 20255343 | PDFSTATSENSEMBLUCSC | 283 | 5 | 30953 | island | 0.799443 | 0.970024 | SLIT2 (margin: SLIT2) | 5.05 | 7.94e-03 | 3.19e-02 |
|  | 2 | 242716388 | 242716801 | PDFSTATSENSEMBLUCSC | 414 | 4 | 23145 | gene | 0.764692 | 0.988199 | Not queried | 5.05 | 2.86e-02 | 6.12e-02 |
|  | 5 | 38258623 | 38258884 | PDFSTATSENSEMBLUCSC | 262 | 4 | 31593 | gene | 0.464989 | 0.032871 | Not queried | 5.05 | 2.86e-02 | 6.12e-02 |
|  | 10 | 50534403 | 50534703 | PDFSTATSENSEMBLUCSC | 301 | 4 | 4584 | gene;island | 0.731037 | 0.987331 | Not queried | 5.05 | 5.71e-02 | 1.00e-01 |
|  | 6 | 32732585 | 32732806 | PDFSTATSENSEMBLUCSC | 222 | 4 | 34873 | tss;island | 0.163258 | 0.831343 | Not queried | 5.05 | 1.14e-01 | 1.68e-01 |
|  | 15 | 60296809 | 60296996 | PDFSTATSENSEMBLUCSC | 188 | 4 | 13368 | gene;island | 0.386871 | 0.937027 | Not queried | 5.05 | 2.86e-02 | 6.12e-02 |
|  | 10 | 134910271 | 134910540 | PDFSTATSENSEMBLUCSC | 270 | 4 | 4884 | gene;island | 0.250833 | 0.728671 | Not queried | 5.05 | 1.14e-01 | 1.68e-01 |
|  | 22 | 21400059 | 21400184 | PDFSTATSENSEMBLUCSC | 126 | 5 | 26993 | tss;island | 0.439903 | 0.017089 | AC002472.11; AC002472.13; P2RX6P (margin: AC002472.11; AC002472.13; MIR649; P2RX6P) | 5.05 | 7.94e-03 | 3.19e-02 |
|  | 14 | 24809297 | 24809759 | PDFSTATSENSEMBLUCSC | 463 | 4 | 12329 | tss | 0.236776 | 0.820258 | Not queried | 5.04 | 2.86e-02 | 6.12e-02 |
|  | 20 | 44098223 | 44098387 | PDFSTATSENSEMBLUCSC | 165 | 6 | 25776 | tss | 0.016406 | 0.389711 | WFDC2; AL031663.1 (margin: WFDC2; AL031663.1) | 5.04 | 2.16e-03 | 1.45e-02 |
|  | 9 | 72288069 | 72288179 | PDFSTATSENSEMBLUCSC | 111 | 4 | 40814 | tss;island | 0.458900 | 0.058271 | Not queried | 5.04 | 2.86e-02 | 6.12e-02 |
|  | 19 | 51225848 | 51226557 | PDFSTATSENSEMBLUCSC | 710 | 6 | 20984 | tss;island | 0.522912 | 0.960149 | CLEC11A (margin: CLEC11A; SHANK1) | 5.04 | 2.16e-03 | 1.45e-02 |
|  | 7 | 27186993 | 27187691 | PDFSTATSENSEMBLUCSC | 699 | 11 | 38349 | island | 0.454791 | 0.975381 | HOXA-AS3; HOXA3; HOXA6 (margin: HOXA-AS3; RP1-170O19.21; HOXA3; HOXA5; HOXA6; HOXA7) | 5.04 | 8.51e-05 | 2.07e-03 |
|  | 7 | 1022471 | 1022806 | PDFSTATSENSEMBLUCSC | 336 | 6 | 37392 | tss | 0.489814 | 0.971559 | CYP2W1 (margin: CYP2W1; COX19) | 5.04 | 2.16e-03 | 1.45e-02 |
|  | 6 | 10383526 | 10383804 | PDFSTATSENSEMBLUCSC | 279 | 4 | 35422 | island | 0.020811 | 0.396660 | Not queried | 5.04 | 2.86e-02 | 6.12e-02 |
|  | 15 | 72977851 | 72978371 | PDFSTATSENSEMBLUCSC | 521 | 5 | 13410 | gene;tss;island | 0.003354 | 0.002076 | Not queried | 5.04 | 4.21e-01 | 4.59e-01 |
|  | 3 | 183948058 | 183948322 | PDFSTATSENSEMBLUCSC | 265 | 6 | 29716 | island | 0.521744 | 0.974431 | Not queried | 5.04 | 6.49e-02 | 1.11e-01 |
|  | 6 | 13014871 | 13015221 | PDFSTATSENSEMBLUCSC | 351 | 5 | 33574 | gene;island | 0.226338 | 0.023793 | Not queried | 5.03 | 3.17e-02 | 6.50e-02 |
|  | 8 | 79427993 | 79428325 | PDFSTATSENSEMBLUCSC | 333 | 4 | 39778 | tss | 0.160937 | 0.800831 | Not queried | 5.03 | 2.86e-02 | 6.12e-02 |
|  | 5 | 146889536 | 146889701 | PDFSTATSENSEMBLUCSC | 166 | 4 | 33289 | island | 0.441579 | 0.949959 | Not queried | 5.03 | 2.86e-02 | 6.12e-02 |
|  | 22 | 46770063 | 46770440 | PDFSTATSENSEMBLUCSC | 378 | 4 | 26913 | gene;island | 0.450364 | 0.984196 | Not queried | 5.03 | 1.14e-01 | 1.68e-01 |
|  | 11 | 128775679 | 128775750 | PDFSTATSENSEMBLUCSC | 72 | 6 | 7808 | tss | 0.006616 | 0.127950 | KCNJ5; C11orf45 (margin: KCNJ5; C11orf45) | 5.03 | 2.16e-03 | 1.45e-02 |
|  | 19 | 51141227 | 51141413 | PDFSTATSENSEMBLUCSC | 187 | 4 | 20180 | gene;island | 0.029374 | 0.585961 | Not queried | 5.03 | 2.86e-02 | 6.12e-02 |
|  | 1 | 236228598 | 236228837 | PDFSTATSENSEMBLUCSC | 240 | 6 | 2339 | tss;island | 0.737854 | 0.982001 | NID1 (margin: Y\_RNA; NID1) | 5.02 | 2.16e-03 | 1.45e-02 |
|  | 11 | 133804526 | 133805057 | PDFSTATSENSEMBLUCSC | 532 | 6 | 6987 | gene | 0.365800 | 0.954423 | Not queried | 5.02 | 4.11e-02 | 8.14e-02 |
|  | 5 | 145717010 | 145717283 | PDFSTATSENSEMBLUCSC | 274 | 4 | 33280 | island | 0.174814 | 0.816181 | Not queried | 5.02 | 2.86e-02 | 6.12e-02 |
|  | 20 | 32899771 | 32899839 | PDFSTATSENSEMBLUCSC | 69 | 5 | 25690 | tss | 0.485204 | 0.071950 | Not queried | 5.02 | 3.17e-02 | 6.50e-02 |
|  | 6 | 75912410 | 75912711 | PDFSTATSENSEMBLUCSC | 302 | 4 | 34394 | gene;island | 0.318592 | 0.945987 | Not queried | 5.02 | 2.86e-02 | 6.12e-02 |
|  | 7 | 35077704 | 35077807 | PDFSTATSENSEMBLUCSC | 104 | 7 | 37534 | tss | 0.198062 | 0.005963 | DPY19L1 (margin: DPY19L1) | 5.02 | 1.17e-03 | 1.13e-02 |
|  | 1 | 29450569 | 29450836 | PDFSTATSENSEMBLUCSC | 268 | 6 | 1439 | tss;island | 0.015922 | 0.419294 | Not queried | 5.02 | 2.60e-02 | 6.12e-02 |
|  | 5 | 34043333 | 34043509 | PDFSTATSENSEMBLUCSC | 177 | 4 | 32011 | tss | 0.367473 | 0.912275 | Not queried | 5.02 | 1.14e-01 | 1.68e-01 |
|  | 20 | 61150472 | 61150637 | PDFSTATSENSEMBLUCSC | 166 | 4 | 25489 | gene;tss;island | 0.271139 | 0.921608 | Not queried | 5.02 | 2.86e-02 | 6.12e-02 |
|  | 8 | 12990898 | 12991196 | PDFSTATSENSEMBLUCSC | 299 | 7 | 39560 | tss | 0.162281 | 0.006757 | DLC1 (margin: DLC1) | 5.01 | 5.83e-04 | 6.47e-03 |
|  | 19 | 37341052 | 37341373 | PDFSTATSENSEMBLUCSC | 322 | 8 | 21948 | island | 0.390930 | 0.019365 | ZNF345; ZNF790 (margin: ZNF345; ZNF790; Y\_RNA) | 5.01 | 6.22e-04 | 6.71e-03 |
|  | 22 | 43253517 | 43253559 | PDFSTATSENSEMBLUCSC | 43 | 5 | 27198 | tss;island | 0.913136 | 0.456486 | ARFGAP3; PACSIN2 (margin: ARFGAP3; PACSIN2; AL049758.2) | 5.01 | 7.94e-03 | 3.19e-02 |
|  | 2 | 238767990 | 238768104 | PDFSTATSENSEMBLUCSC | 115 | 4 | 23967 | tss;island | 0.577705 | 0.046529 | Not queried | 5.01 | 2.86e-02 | 6.12e-02 |
|  | 7 | 87563717 | 87563931 | PDFSTATSENSEMBLUCSC | 215 | 5 | 37075 | gene | 0.591988 | 0.039971 | Not queried | 5.01 | 1.59e-02 | 5.36e-02 |
|  | 8 | 28560467 | 28560723 | PDFSTATSENSEMBLUCSC | 257 | 5 | 39262 | gene;island | 0.354185 | 0.963616 | EXTL3; EXTL3-AS1 (margin: EXTL3; EXTL3-AS1) | 5.01 | 7.94e-03 | 3.19e-02 |
|  | 11 | 57267101 | 57267244 | PDFSTATSENSEMBLUCSC | 144 | 4 | 6614 | gene;island | 0.030415 | 0.501531 | Not queried | 5.01 | 2.86e-02 | 6.12e-02 |
|  | 3 | 49236800 | 49236946 | PDFSTATSENSEMBLUCSC | 147 | 6 | 27778 | gene;island | 0.337116 | 0.964139 | Not queried | 5.01 | 2.60e-02 | 6.12e-02 |
|  | 6 | 32549139 | 32549631 | PDFSTATSENSEMBLUCSC | 493 | 5 | 34121 | gene;island | 0.212986 | 0.855769 | Not queried | 5.00 | 9.52e-02 | 1.53e-01 |
|  | 6 | 33141678 | 33142006 | PDFSTATSENSEMBLUCSC | 329 | 7 | 34202 | gene | 0.097925 | 0.734363 | Not queried | 5.00 | 1.75e-02 | 5.79e-02 |
|  | 5 | 180018465 | 180018735 | PDFSTATSENSEMBLUCSC | 271 | 7 | 33504 | island | 0.381737 | 0.976943 | SCGB3A1 (margin: AC122714.1; SCGB3A1; FLT4) | 5.00 | 5.83e-04 | 6.47e-03 |
|  | 17 | 72350354 | 72350710 | PDFSTATSENSEMBLUCSC | 357 | 4 | 16752 | gene;island | 0.215907 | 0.907077 | Not queried | 5.00 | 2.86e-02 | 6.12e-02 |
|  | 10 | 101290958 | 101291397 | PDFSTATSENSEMBLUCSC | 440 | 4 | 6011 | island | 0.473671 | 0.961989 | Not queried | 5.00 | 2.00e-01 | 2.55e-01 |
|  | 16 | 86337662 | 86337823 | PDFSTATSENSEMBLUCSC | 162 | 5 | 16168 | island | 0.375804 | 0.960567 | Not queried | 5.00 | 5.56e-02 | 1.00e-01 |
|  | 19 | 2650727 | 2650863 | PDFSTATSENSEMBLUCSC | 137 | 4 | 19699 | gene;island | 0.511282 | 0.977672 | Not queried | 5.00 | 2.86e-02 | 6.12e-02 |
|  | 6 | 149805995 | 149806131 | PDFSTATSENSEMBLUCSC | 137 | 4 | 34506 | gene | 0.527485 | 0.973512 | Not queried | 5.00 | 2.86e-02 | 6.12e-02 |
|  | 19 | 7735239 | 7735510 | PDFSTATSENSEMBLUCSC | 272 | 4 | 21461 | island | 0.240250 | 0.922039 | Not queried | 5.00 | 2.86e-02 | 6.12e-02 |
|  | 6 | 52858837 | 52859107 | PDFSTATSENSEMBLUCSC | 271 | 4 | 34370 | gene;island | 0.492688 | 0.975763 | Not queried | 5.00 | 2.86e-02 | 6.12e-02 |
|  | 7 | 87105022 | 87105416 | PDFSTATSENSEMBLUCSC | 395 | 5 | 37686 | tss | 0.400445 | 0.960192 | ABCB4 (margin: ABCB4) | 5.00 | 7.94e-03 | 3.19e-02 |
|  | 14 | 101489415 | 101489781 | PDFSTATSENSEMBLUCSC | 367 | 7 | 12619 | tss | 0.147084 | 0.859835 | Not queried | 5.00 | 3.79e-02 | 7.66e-02 |
|  | 20 | 50179151 | 50179192 | PDFSTATSENSEMBLUCSC | 42 | 4 | 26256 | island | 0.360878 | 0.014867 | Not queried | 5.00 | 2.86e-02 | 6.12e-02 |
|  | 4 | 95678703 | 95678939 | PDFSTATSENSEMBLUCSC | 237 | 5 | 30504 | tss;island | 0.214372 | 0.021672 | BMPR1B; RP11-168E14.1 (margin: BMPR1B; RP11-168E14.1) | 5.00 | 7.94e-03 | 3.19e-02 |
|  | 14 | 101530343 | 101530856 | PDFSTATSENSEMBLUCSC | 514 | 6 | 12633 | tss;island | 0.144352 | 0.848671 | Not queried | 5.00 | 1.52e-02 | 5.36e-02 |
|  | 8 | 24772137 | 24772513 | PDFSTATSENSEMBLUCSC | 377 | 8 | 39246 | gene;island | 0.673926 | 0.982568 | NEFM; GS1-72M22.1 (margin: NEFM; RP11-624C23.1; GS1-72M22.1) | 5.00 | 1.55e-04 | 2.71e-03 |
|  | 10 | 122216135 | 122216378 | PDFSTATSENSEMBLUCSC | 244 | 7 | 5403 | tss;island | 0.279159 | 0.898233 | PPAPDC1A (margin: PPAPDC1A; AC073587.1) | 4.99 | 1.17e-03 | 1.13e-02 |
|  | 10 | 106113175 | 106113447 | PDFSTATSENSEMBLUCSC | 273 | 5 | 5349 | tss | 0.587124 | 0.006710 | Not queried | 4.99 | 3.17e-02 | 6.50e-02 |
|  | 19 | 39989234 | 39989542 | PDFSTATSENSEMBLUCSC | 309 | 6 | 20774 | tss | 0.087637 | 0.668656 | DLL3 (margin: TIMM50; DLL3) | 4.99 | 8.66e-03 | 3.36e-02 |
|  | 7 | 155252701 | 155252944 | PDFSTATSENSEMBLUCSC | 244 | 4 | 37308 | gene;island | 0.645991 | 0.976337 | Not queried | 4.99 | 2.86e-02 | 6.12e-02 |
|  | 11 | 2182457 | 2182795 | PDFSTATSENSEMBLUCSC | 339 | 8 | 7061 | tss | 0.212626 | 0.938469 | INS-IGF2; INS (margin: MIR4686; IGF2; INS-IGF2; INS; TH) | 4.99 | 3.11e-04 | 4.77e-03 |
|  | 5 | 83679941 | 83680326 | PDFSTATSENSEMBLUCSC | 386 | 4 | 31672 | gene;island | 0.446981 | 0.029381 | Not queried | 4.99 | 1.14e-01 | 1.68e-01 |
|  | 4 | 4858340 | 4858814 | PDFSTATSENSEMBLUCSC | 475 | 5 | 30865 | island | 0.466723 | 0.022460 | Not queried | 4.99 | 9.52e-02 | 1.53e-01 |
|  | 1 | 43919949 | 43920264 | PDFSTATSENSEMBLUCSC | 316 | 8 | 1563 | tss | 0.440478 | 0.944286 | SZT2; HYI-AS1; HYI (margin: SZT2; HYI-AS1; SZT2-AS1; HYI) | 4.99 | 4.66e-03 | 2.68e-02 |
|  | 12 | 132259591 | 132259908 | PDFSTATSENSEMBLUCSC | 318 | 4 | 9427 | gene | 0.535804 | 0.981311 | Not queried | 4.99 | 5.71e-02 | 1.00e-01 |
|  | 5 | 114515626 | 114515936 | PDFSTATSENSEMBLUCSC | 311 | 4 | 31718 | gene;island | 0.640488 | 0.981853 | Not queried | 4.99 | 2.86e-02 | 6.12e-02 |
|  | 4 | 175138377 | 175138785 | PDFSTATSENSEMBLUCSC | 409 | 4 | 31433 | island | 0.500140 | 0.016144 | Not queried | 4.99 | 2.86e-02 | 6.12e-02 |
|  | 12 | 111284527 | 111284806 | PDFSTATSENSEMBLUCSC | 280 | 8 | 10011 | tss | 0.324939 | 0.926879 | RP1-74B13.2; CCDC63 (margin: RP1-74B13.2; CCDC63; RPL29P25) | 4.98 | 1.55e-04 | 2.71e-03 |
|  | 17 | 46810540 | 46810934 | PDFSTATSENSEMBLUCSC | 395 | 6 | 18505 | island | 0.268936 | 0.976042 | Not queried | 4.98 | 9.31e-02 | 1.53e-01 |
|  | 11 | 18727552 | 18727949 | PDFSTATSENSEMBLUCSC | 398 | 4 | 6532 | gene;island | 0.423607 | 0.974693 | Not queried | 4.98 | 2.00e-01 | 2.55e-01 |
|  | 5 | 140624869 | 140625130 | PDFSTATSENSEMBLUCSC | 262 | 7 | 32369 | tss | 0.428592 | 0.962029 | PCDHB15 (margin: PCDHB18; PCDHB19P; PCDHB15) | 4.98 | 5.83e-04 | 6.47e-03 |
|  | 11 | 14666436 | 14666839 | PDFSTATSENSEMBLUCSC | 404 | 4 | 6518 | gene;island | 0.306628 | 0.011803 | Not queried | 4.98 | 2.86e-02 | 6.12e-02 |
|  | 5 | 174151043 | 174151779 | PDFSTATSENSEMBLUCSC | 737 | 9 | 33416 | island | 0.355032 | 0.006026 | MSX2 (margin: MSX2) | 4.98 | 2.88e-04 | 4.58e-03 |
|  | 2 | 79739863 | 79739940 | PDFSTATSENSEMBLUCSC | 78 | 4 | 23472 | tss;island | 0.888851 | 0.156103 | Not queried | 4.98 | 2.86e-02 | 6.12e-02 |
|  | 12 | 57630107 | 57630456 | PDFSTATSENSEMBLUCSC | 350 | 4 | 9191 | gene;island | 0.566802 | 0.975379 | Not queried | 4.98 | 2.86e-02 | 6.12e-02 |
|  | 2 | 97652129 | 97652491 | PDFSTATSENSEMBLUCSC | 363 | 5 | 24544 | island | 0.409352 | 0.964038 | FAM178B (margin: FAM178B; snoU13) | 4.97 | 7.94e-03 | 3.19e-02 |
|  | 11 | 61355119 | 61355505 | PDFSTATSENSEMBLUCSC | 387 | 4 | 8329 | island | 0.017959 | 0.518732 | Not queried | 4.97 | 2.86e-02 | 6.12e-02 |
|  | 2 | 56150475 | 56150675 | PDFSTATSENSEMBLUCSC | 201 | 4 | 23365 | tss | 0.026248 | 0.361536 | Not queried | 4.97 | 5.71e-02 | 1.00e-01 |
|  | 6 | 167535764 | 167536184 | PDFSTATSENSEMBLUCSC | 421 | 7 | 34556 | gene;tss | 0.522759 | 0.952910 | CCR6 (margin: CCR6) | 4.97 | 2.33e-03 | 1.52e-02 |
|  | 20 | 44098223 | 44098409 | PDFSTATSENSEMBLUCSC | 187 | 7 | 26200 | island | 0.019264 | 0.398475 | WFDC2; AL031663.1 (margin: WFDC2; AL031663.1) | 4.97 | 5.83e-04 | 6.47e-03 |
|  | 17 | 42083193 | 42083306 | PDFSTATSENSEMBLUCSC | 114 | 4 | 16601 | gene;tss;island | 0.645987 | 0.978240 | Not queried | 4.97 | 2.86e-02 | 6.12e-02 |
|  | 14 | 101438430 | 101438663 | PDFSTATSENSEMBLUCSC | 234 | 4 | 12617 | tss | 0.610932 | 0.962201 | Not queried | 4.97 | 2.86e-02 | 6.12e-02 |
|  | 8 | 40755165 | 40755559 | PDFSTATSENSEMBLUCSC | 395 | 7 | 40240 | island | 0.494995 | 0.017408 | ZMAT4; RP11-465K16.1 (margin: ZMAT4; RP11-465K16.1) | 4.97 | 5.83e-04 | 6.47e-03 |
|  | 10 | 90967612 | 90968091 | PDFSTATSENSEMBLUCSC | 480 | 6 | 5212 | tss;island | 0.494427 | 0.973242 | Not queried | 4.97 | 2.60e-02 | 6.12e-02 |
|  | 4 | 52917271 | 52917567 | PDFSTATSENSEMBLUCSC | 297 | 9 | 30390 | tss | 0.017628 | 0.352360 | SPATA18 (margin: RP11-535C7.1; SPATA18) | 4.97 | 3.99e-03 | 2.47e-02 |
|  | 11 | 843897 | 844686 | PDFSTATSENSEMBLUCSC | 790 | 11 | 6393 | gene;island | 0.450766 | 0.067105 | TSPAN4; POLR2L (margin: EFCAB4A; CD151; TSPAN4; AP006621.8; POLR2L) | 4.97 | 5.39e-05 | 1.47e-03 |
|  | 2 | 223164459 | 223164925 | PDFSTATSENSEMBLUCSC | 467 | 8 | 23049 | gene;tss;island | 0.696724 | 0.980626 | CCDC140; PAX3 (margin: CCDC140; PAX3) | 4.97 | 6.22e-04 | 6.71e-03 |
|  | 18 | 48086162 | 48086680 | PDFSTATSENSEMBLUCSC | 519 | 7 | 19514 | island | 0.371146 | 0.029344 | MAPK4 (margin: MAPK4) | 4.97 | 2.33e-03 | 1.52e-02 |
|  | 20 | 24451769 | 24452131 | PDFSTATSENSEMBLUCSC | 363 | 6 | 25376 | gene;island | 0.448634 | 0.957220 | Not queried | 4.97 | 1.52e-02 | 5.36e-02 |
|  | 6 | 32811495 | 32812708 | PDFSTATSENSEMBLUCSC | 1214 | 44 | 34151 | gene;island | 0.007943 | 0.334668 | XXbac-BPG246D15.8; PSMB9; PSMB8; TAP1 (margin: XXbac-BPG246D15.8; PSMB9; TAP2; PSMB8; TAP1) | 4.97 | 1.30e-09 | 5.51e-07 |
|  | 6 | 134638742 | 134639482 | PDFSTATSENSEMBLUCSC | 741 | 10 | 36495 | island | 0.425329 | 0.951037 | SGK1 (margin: SGK1) | 4.97 | 1.08e-05 | 4.82e-04 |
|  | 11 | 2481857 | 2482929 | PDFSTATSENSEMBLUCSC | 1073 | 17 | 6460 | gene | 0.476189 | 0.954665 | KCNQ1 (margin: KCNQ1) | 4.97 | 4.35e-07 | 5.37e-05 |
|  | 3 | 112359488 | 112359652 | PDFSTATSENSEMBLUCSC | 165 | 6 | 27896 | gene | 0.008889 | 0.196253 | CCDC80 (margin: CCDC80) | 4.97 | 2.16e-03 | 1.45e-02 |
|  | 14 | 101505891 | 101506579 | PDFSTATSENSEMBLUCSC | 689 | 11 | 12625 | tss | 0.322106 | 0.958752 | AL132709.2; MIR376C; MIR654; MIR376A1; MIR300 (margin: MIR494; MIR1193; MIR543; MIR495; AL132709.2; MIR376C; MIR654; MIR376A1; MIR300; MIR1185-1; MIR1185-2; MIR381HG; MIR381; MIR487B; MIR539; MIR889; MIR544A; MIR655; AL132709.3) | 4.97 | 5.24e-03 | 2.98e-02 |
|  | 16 | 1922149 | 1922577 | PDFSTATSENSEMBLUCSC | 429 | 8 | 15008 | tss;island | 0.671429 | 0.194213 | Not queried | 4.97 | 2.81e-02 | 6.12e-02 |
|  | 4 | 40858965 | 40859344 | PDFSTATSENSEMBLUCSC | 380 | 9 | 29990 | gene;island | 0.975364 | 0.598613 | APBB2 (margin: APBB2; snoU13) | 4.97 | 4.11e-05 | 1.17e-03 |
|  | 1 | 236686564 | 236686804 | PDFSTATSENSEMBLUCSC | 241 | 5 | 1098 | gene;tss;island | 0.474351 | 0.013877 | LGALS8; LGALS8-AS1 (margin: LGALS8; LGALS8-AS1) | 4.96 | 7.94e-03 | 3.19e-02 |
|  | 14 | 55034288 | 55034616 | PDFSTATSENSEMBLUCSC | 329 | 6 | 12409 | tss | 0.004361 | 0.163211 | SAMD4A (margin: SAMD4A) | 4.96 | 2.16e-03 | 1.45e-02 |
|  | 3 | 56502612 | 56502814 | PDFSTATSENSEMBLUCSC | 203 | 5 | 28404 | tss;island | 0.331423 | 0.015684 | Not queried | 4.96 | 1.59e-02 | 5.36e-02 |
|  | 17 | 37382161 | 37382699 | PDFSTATSENSEMBLUCSC | 539 | 8 | 17306 | tss;island | 0.613784 | 0.021012 | Not queried | 4.96 | 2.81e-02 | 6.12e-02 |
|  | 8 | 98880860 | 98881351 | PDFSTATSENSEMBLUCSC | 492 | 9 | 40480 | island | 0.019364 | 0.490478 | Not queried | 4.96 | 3.15e-02 | 6.50e-02 |
|  | 19 | 1401118 | 1401781 | PDFSTATSENSEMBLUCSC | 664 | 9 | 21222 | island | 0.703484 | 0.046754 | Not queried | 4.96 | 1.88e-02 | 6.12e-02 |
|  | 13 | 33589962 | 33590495 | PDFSTATSENSEMBLUCSC | 534 | 11 | 11385 | tss;island | 0.485445 | 0.977110 | KL (margin: KL) | 4.96 | 3.40e-05 | 1.17e-03 |
|  | 11 | 108408907 | 108408981 | PDFSTATSENSEMBLUCSC | 75 | 5 | 6889 | gene | 0.693649 | 0.984097 | Not queried | 4.95 | 1.59e-02 | 5.36e-02 |
|  | 20 | 57464970 | 57465175 | PDFSTATSENSEMBLUCSC | 206 | 7 | 25474 | gene;tss;island | 0.573327 | 0.969520 | GNAS; RP1-309F20.3 (margin: GNAS; RP1-309F20.3) | 4.95 | 1.17e-03 | 1.13e-02 |
|  | 6 | 94129234 | 94129697 | PDFSTATSENSEMBLUCSC | 464 | 10 | 36331 | island | 0.865728 | 0.077628 | EPHA7 (margin: EPHA7) | 4.95 | 1.50e-03 | 1.41e-02 |
|  | 3 | 61547104 | 61547211 | PDFSTATSENSEMBLUCSC | 108 | 5 | 28423 | tss | 0.131795 | 0.019209 | PTPRG (margin: PTPRG) | 4.95 | 7.94e-03 | 3.19e-02 |
|  | 8 | 8859686 | 8860266 | PDFSTATSENSEMBLUCSC | 581 | 7 | 39542 | tss | 0.773504 | 0.020979 | Not queried | 4.95 | 4.56e-01 | 4.96e-01 |
|  | 2 | 17721431 | 17721803 | PDFSTATSENSEMBLUCSC | 373 | 8 | 23200 | tss | 0.071875 | 0.637873 | VSNL1 (margin: VSNL1) | 4.95 | 1.55e-04 | 2.71e-03 |
|  | 6 | 152702330 | 152702660 | PDFSTATSENSEMBLUCSC | 331 | 6 | 34516 | gene | 0.340864 | 0.920874 | SYNE1-AS1; SYNE1 (margin: SYNE1-AS1; SYNE1) | 4.95 | 2.16e-03 | 1.45e-02 |
|  | 11 | 72433396 | 72433619 | PDFSTATSENSEMBLUCSC | 224 | 4 | 6815 | gene | 0.027969 | 0.611451 | Not queried | 4.95 | 2.86e-02 | 6.12e-02 |
|  | 11 | 6947353 | 6947773 | PDFSTATSENSEMBLUCSC | 421 | 11 | 8023 | island | 0.314585 | 0.801923 | ZNF215 (margin: OR2D3; ZNF215) | 4.95 | 6.63e-03 | 3.19e-02 |
|  | 15 | 73611079 | 73611614 | PDFSTATSENSEMBLUCSC | 536 | 6 | 14287 | island | 0.096203 | 0.872733 | Not queried | 4.95 | 2.60e-02 | 6.12e-02 |
|  | 4 | 618114 | 618549 | PDFSTATSENSEMBLUCSC | 436 | 5 | 30225 | tss;island | 0.183082 | 0.896676 | Not queried | 4.95 | 2.22e-01 | 2.76e-01 |
|  | 7 | 4850075 | 4850345 | PDFSTATSENSEMBLUCSC | 271 | 4 | 36858 | gene;island | 0.522052 | 0.971990 | Not queried | 4.94 | 2.86e-02 | 6.12e-02 |
|  | 1 | 3239916 | 3240227 | PDFSTATSENSEMBLUCSC | 312 | 4 | 120 | gene;island | 0.221772 | 0.940325 | Not queried | 4.94 | 2.86e-02 | 6.12e-02 |
|  | 15 | 83348985 | 83349864 | PDFSTATSENSEMBLUCSC | 880 | 7 | 13454 | gene;island | 0.560507 | 0.066622 | RP11-752G15.3; AP3B2 (margin: RP11-752G15.3; AP3B2; RP11-752G15.4) | 4.94 | 6.99e-03 | 3.19e-02 |
|  | 12 | 103352235 | 103352454 | PDFSTATSENSEMBLUCSC | 220 | 5 | 9270 | gene;island | 0.957604 | 0.423874 | Not queried | 4.94 | 5.56e-02 | 1.00e-01 |
|  | 11 | 65779192 | 65779847 | PDFSTATSENSEMBLUCSC | 656 | 10 | 8495 | island | 0.439260 | 0.958825 | CST6 (margin: BANF1; CST6; EIF1AD; CATSPER1) | 4.94 | 5.20e-03 | 2.96e-02 |
|  | 1 | 21835424 | 21835703 | PDFSTATSENSEMBLUCSC | 280 | 7 | 1323 | tss;island | 0.552015 | 0.011326 | Not queried | 4.94 | 1.75e-02 | 5.79e-02 |
|  | 16 | 215410 | 216244 | PDFSTATSENSEMBLUCSC | 835 | 9 | 15443 | island | 0.673505 | 0.981374 | HBM; HBZP1 (margin: HBZ; HBM; HBZP1; Z84721.4; HBA2; HBA1) | 4.94 | 8.23e-05 | 2.06e-03 |
|  | 7 | 94953653 | 94953877 | PDFSTATSENSEMBLUCSC | 225 | 5 | 37096 | gene | 0.491455 | 0.962794 | PON1 (margin: PON1) | 4.94 | 7.94e-03 | 3.19e-02 |
|  | 8 | 39441968 | 39442089 | PDFSTATSENSEMBLUCSC | 122 | 5 | 39664 | tss | 0.684996 | 0.982794 | ADAM18 (margin: CTD-2024D23.1; ADAM18) | 4.94 | 7.94e-03 | 3.19e-02 |
|  | 6 | 166876490 | 166877038 | PDFSTATSENSEMBLUCSC | 549 | 8 | 34545 | gene | 0.434865 | 0.976870 | RPS6KA2; RPS6KA2-IT1 (margin: RPS6KA2; RPS6KA2-IT1) | 4.93 | 2.95e-03 | 1.88e-02 |
|  | 1 | 43919477 | 43920264 | PDFSTATSENSEMBLUCSC | 788 | 15 | 3167 | island | 0.099307 | 0.204098 | Not queried | 4.93 | 6.13e-02 | 1.07e-01 |
|  | 5 | 137474700 | 137475115 | PDFSTATSENSEMBLUCSC | 416 | 5 | 31764 | gene | 0.943178 | 0.354188 | NME5; BRD8 (margin: NME5; BRD8) | 4.93 | 7.94e-03 | 3.19e-02 |
|  | 13 | 88328251 | 88328471 | PDFSTATSENSEMBLUCSC | 221 | 4 | 11187 | gene;island | 0.782334 | 0.984064 | Not queried | 4.93 | 5.71e-02 | 1.00e-01 |
|  | 15 | 90456194 | 90456686 | PDFSTATSENSEMBLUCSC | 493 | 6 | 14445 | island | 0.288377 | 0.582302 | Not queried | 4.93 | 3.10e-01 | 3.66e-01 |
|  | 16 | 891260 | 891400 | PDFSTATSENSEMBLUCSC | 141 | 4 | 15493 | island | 0.123439 | 0.814044 | Not queried | 4.92 | 5.71e-02 | 1.00e-01 |
|  | 5 | 149669414 | 149669813 | PDFSTATSENSEMBLUCSC | 400 | 5 | 32427 | tss | 0.850101 | 0.258092 | CAMK2A (margin: CAMK2A; ARSI) | 4.92 | 7.94e-03 | 3.19e-02 |
|  | 1 | 2885085 | 2885244 | PDFSTATSENSEMBLUCSC | 160 | 4 | 2523 | island | 0.292902 | 0.964320 | Not queried | 4.92 | 5.71e-02 | 1.00e-01 |
|  | 2 | 906044 | 906427 | PDFSTATSENSEMBLUCSC | 384 | 4 | 23156 | tss | 0.361940 | 0.950771 | Not queried | 4.92 | 2.86e-02 | 6.12e-02 |
|  | 12 | 75728104 | 75728458 | PDFSTATSENSEMBLUCSC | 355 | 5 | 9880 | tss | 0.027242 | 0.001966 | GLIPR1L1; CAPS2 (margin: GLIPR1L1; CAPS2) | 4.92 | 7.94e-03 | 3.19e-02 |
|  | 6 | 29596840 | 29597113 | PDFSTATSENSEMBLUCSC | 274 | 5 | 33679 | gene;tss;island | 0.789930 | 0.097733 | Not queried | 4.92 | 3.17e-02 | 6.50e-02 |
|  | 4 | 77819534 | 77819792 | PDFSTATSENSEMBLUCSC | 259 | 4 | 30450 | tss;island | 0.367303 | 0.012995 | Not queried | 4.92 | 2.00e-01 | 2.55e-01 |
|  | 17 | 25676201 | 25676574 | PDFSTATSENSEMBLUCSC | 374 | 4 | 18134 | island | 0.449849 | 0.960620 | Not queried | 4.92 | 2.86e-02 | 6.12e-02 |
|  | 12 | 107711872 | 107712210 | PDFSTATSENSEMBLUCSC | 339 | 6 | 10761 | island | 0.143342 | 0.004099 | BTBD11 (margin: BTBD11) | 4.92 | 8.66e-03 | 3.36e-02 |
|  | 5 | 1445549 | 1445593 | PDFSTATSENSEMBLUCSC | 45 | 4 | 31970 | tss | 0.342208 | 0.923049 | Not queried | 4.91 | 2.86e-02 | 6.12e-02 |
|  | 8 | 98880860 | 98881265 | PDFSTATSENSEMBLUCSC | 406 | 8 | 39831 | tss | 0.023645 | 0.419854 | Not queried | 4.91 | 6.50e-02 | 1.11e-01 |
|  | 3 | 128720242 | 128720433 | PDFSTATSENSEMBLUCSC | 192 | 5 | 28575 | tss;island | 0.560640 | 0.944336 | EFCC1; KIAA1257 (margin: EFCC1; KIAA1257) | 4.91 | 7.94e-03 | 3.19e-02 |
|  | 5 | 142781498 | 142781736 | PDFSTATSENSEMBLUCSC | 239 | 4 | 31845 | gene;island | 0.018006 | 0.186103 | Not queried | 4.91 | 2.86e-02 | 6.12e-02 |
|  | 17 | 40713676 | 40714406 | PDFSTATSENSEMBLUCSC | 731 | 11 | 18340 | island | 0.019914 | 0.373920 | RP11-400F19.8; COASY (margin: RP11-400F19.8; HSD17B1; COASY; MLX; RP11-400F19.6; PSMC3IP) | 4.91 | 3.94e-04 | 5.86e-03 |
|  | 2 | 66810341 | 66810606 | PDFSTATSENSEMBLUCSC | 266 | 4 | 24359 | island | 0.831402 | 0.298453 | Not queried | 4.91 | 2.86e-02 | 6.12e-02 |
|  | 14 | 97058777 | 97059467 | PDFSTATSENSEMBLUCSC | 691 | 8 | 13119 | island | 0.656276 | 0.116143 | RP11-433J8.1 (margin: RP11-433J8.1) | 4.91 | 1.04e-02 | 3.96e-02 |
|  | 8 | 143869138 | 143869473 | PDFSTATSENSEMBLUCSC | 336 | 5 | 39940 | tss | 0.113818 | 0.880452 | RP11-706C16.8; LY6D (margin: RP11-706C16.8; LYNX1; LY6D) | 4.91 | 7.94e-03 | 3.19e-02 |
|  | 10 | 133956824 | 133957095 | PDFSTATSENSEMBLUCSC | 272 | 4 | 4856 | gene;island | 0.483537 | 0.979000 | Not queried | 4.91 | 5.71e-02 | 1.00e-01 |
|  | 1 | 221068730 | 221068935 | PDFSTATSENSEMBLUCSC | 206 | 4 | 4226 | island | 0.458415 | 0.974468 | Not queried | 4.91 | 5.71e-02 | 1.00e-01 |
|  | 5 | 140624869 | 140625389 | PDFSTATSENSEMBLUCSC | 521 | 10 | 33228 | island | 0.429667 | 0.958687 | PCDHB15 (margin: PCDHB18; PCDHB19P; PCDHB15) | 4.91 | 1.08e-05 | 4.82e-04 |
|  | 7 | 20817859 | 20818226 | PDFSTATSENSEMBLUCSC | 368 | 4 | 38291 | island | 0.823251 | 0.205788 | Not queried | 4.91 | 5.71e-02 | 1.00e-01 |
|  | 6 | 26188969 | 26189234 | PDFSTATSENSEMBLUCSC | 266 | 4 | 33604 | gene | 0.019571 | 0.349802 | Not queried | 4.91 | 2.86e-02 | 6.12e-02 |
|  | 1 | 156475001 | 156475319 | PDFSTATSENSEMBLUCSC | 319 | 4 | 3858 | island | 0.028830 | 0.456306 | Not queried | 4.90 | 2.86e-02 | 6.12e-02 |
|  | 5 | 140614369 | 140614765 | PDFSTATSENSEMBLUCSC | 397 | 4 | 31815 | gene;island | 0.429838 | 0.930969 | Not queried | 4.90 | 2.86e-02 | 6.12e-02 |
|  | 12 | 2761364 | 2761549 | PDFSTATSENSEMBLUCSC | 186 | 4 | 9004 | gene | 0.258993 | 0.865882 | Not queried | 4.90 | 2.86e-02 | 6.12e-02 |
|  | 14 | 101359793 | 101360236 | PDFSTATSENSEMBLUCSC | 444 | 11 | 12615 | tss | 0.395317 | 0.959555 | AL117190.3; MEG8 (margin: MIR433; MIR127; MIR432; MIR136; AL117190.3; MEG8; AL117190.1; RTL1) | 4.90 | 1.40e-03 | 1.33e-02 |
|  | 6 | 31093846 | 31094185 | PDFSTATSENSEMBLUCSC | 340 | 4 | 33851 | gene | 0.065698 | 0.775901 | Not queried | 4.90 | 5.71e-02 | 1.00e-01 |
|  | 17 | 17910235 | 17910425 | PDFSTATSENSEMBLUCSC | 191 | 4 | 16470 | gene;island | 0.932455 | 0.257662 | Not queried | 4.90 | 2.86e-02 | 6.12e-02 |
|  | 10 | 22726610 | 22726885 | PDFSTATSENSEMBLUCSC | 276 | 6 | 5605 | island | 0.008728 | 0.246909 | SPAG6 (margin: SPAG6) | 4.90 | 2.16e-03 | 1.45e-02 |
|  | 7 | 157669880 | 157670171 | PDFSTATSENSEMBLUCSC | 292 | 4 | 37349 | gene;island | 0.018596 | 0.403414 | Not queried | 4.90 | 2.86e-02 | 6.12e-02 |
|  | 13 | 113832355 | 113832595 | PDFSTATSENSEMBLUCSC | 241 | 4 | 11279 | gene | 0.381906 | 0.940830 | Not queried | 4.90 | 1.14e-01 | 1.68e-01 |
|  | 19 | 863054 | 863244 | PDFSTATSENSEMBLUCSC | 191 | 4 | 19635 | gene;island | 0.582223 | 0.974534 | Not queried | 4.90 | 2.86e-02 | 6.12e-02 |
|  | 1 | 236227410 | 236227686 | PDFSTATSENSEMBLUCSC | 277 | 6 | 1096 | gene;island | 0.219113 | 0.970203 | NID1 (margin: Y\_RNA; NID1) | 4.90 | 4.33e-03 | 2.52e-02 |
|  | 4 | 73434591 | 73434856 | PDFSTATSENSEMBLUCSC | 266 | 4 | 30427 | tss | 0.067101 | 0.623627 | Not queried | 4.90 | 2.86e-02 | 6.12e-02 |
|  | 5 | 76936361 | 76936793 | PDFSTATSENSEMBLUCSC | 433 | 4 | 32903 | island | 0.628979 | 0.985082 | Not queried | 4.90 | 2.86e-02 | 6.12e-02 |
|  | 6 | 6588693 | 6589075 | PDFSTATSENSEMBLUCSC | 383 | 5 | 33548 | gene | 0.155713 | 0.954648 | Not queried | 4.90 | 5.56e-02 | 1.00e-01 |
|  | 1 | 24882153 | 24882451 | PDFSTATSENSEMBLUCSC | 299 | 5 | 1361 | tss | 0.625320 | 0.057989 | Not queried | 4.89 | 1.59e-02 | 5.36e-02 |
|  | 16 | 87739377 | 87739453 | PDFSTATSENSEMBLUCSC | 77 | 4 | 15407 | tss;island | 0.954620 | 0.474269 | Not queried | 4.89 | 2.86e-02 | 6.12e-02 |
|  | 1 | 6085799 | 6086275 | PDFSTATSENSEMBLUCSC | 477 | 7 | 1196 | tss | 0.027194 | 0.510438 | KCNAB2 (margin: KCNAB2) | 4.89 | 5.83e-04 | 6.47e-03 |
|  | 6 | 17282284 | 17282859 | PDFSTATSENSEMBLUCSC | 576 | 10 | 33587 | gene;tss;island | 0.903507 | 0.487784 | RBM24 (margin: RBM24) | 4.89 | 3.25e-04 | 4.91e-03 |
|  | 10 | 102090297 | 102090589 | PDFSTATSENSEMBLUCSC | 293 | 5 | 5291 | tss | 0.174052 | 0.855414 | PKD2L1 (margin: PKD2L1; RP11-34D15.2) | 4.89 | 7.94e-03 | 3.19e-02 |
|  | 12 | 85430123 | 85430336 | PDFSTATSENSEMBLUCSC | 214 | 7 | 9233 | gene | 0.020907 | 0.001935 | Not queried | 4.89 | 1.75e-02 | 5.79e-02 |
|  | 5 | 176237221 | 176237381 | PDFSTATSENSEMBLUCSC | 161 | 4 | 32518 | tss;island | 0.125462 | 0.005387 | Not queried | 4.89 | 2.86e-02 | 6.12e-02 |
|  | 1 | 71513494 | 71513789 | PDFSTATSENSEMBLUCSC | 296 | 6 | 1693 | tss;island | 0.430896 | 0.035175 | Not queried | 4.89 | 2.60e-02 | 6.12e-02 |
|  | 4 | 46391414 | 46391929 | PDFSTATSENSEMBLUCSC | 516 | 11 | 30372 | tss | 0.910914 | 0.150960 | RP11-436F23.1; GABRA2 (margin: RP11-436F23.1; GABRA2) | 4.89 | 2.45e-03 | 1.59e-02 |
|  | 20 | 21685834 | 21686728 | PDFSTATSENSEMBLUCSC | 895 | 14 | 26042 | island | 0.614267 | 0.973968 | PAX1 (margin: PAX1) | 4.88 | 3.34e-06 | 2.26e-04 |
|  | 20 | 21685834 | 21686293 | PDFSTATSENSEMBLUCSC | 460 | 10 | 25631 | tss | 0.645460 | 0.981050 | PAX1 (margin: PAX1) | 4.88 | 1.30e-04 | 2.71e-03 |
|  | 12 | 131561080 | 131561312 | PDFSTATSENSEMBLUCSC | 233 | 4 | 9423 | gene | 0.244137 | 0.775264 | Not queried | 4.88 | 2.86e-02 | 6.12e-02 |
|  | 14 | 102554826 | 102554977 | PDFSTATSENSEMBLUCSC | 152 | 4 | 12226 | gene;tss;island | 0.965862 | 0.529847 | Not queried | 4.88 | 2.86e-02 | 6.12e-02 |
|  | 8 | 97247865 | 97248129 | PDFSTATSENSEMBLUCSC | 265 | 6 | 39825 | tss | 0.015536 | 0.353544 | KB-1043D8.6; UQCRB (margin: KB-1043D8.6; UQCRB; MTERFD1) | 4.88 | 8.66e-03 | 3.36e-02 |
|  | 1 | 31846397 | 31846873 | PDFSTATSENSEMBLUCSC | 477 | 5 | 1446 | tss | 0.375931 | 0.957897 | FABP3 (margin: ZCCHC17; FABP3) | 4.88 | 7.94e-03 | 3.19e-02 |
|  | 11 | 62369394 | 62369870 | PDFSTATSENSEMBLUCSC | 477 | 7 | 7348 | tss;island | 0.008402 | 0.064983 | Not queried | 4.88 | 3.79e-02 | 7.66e-02 |
|  | 11 | 45687263 | 45687494 | PDFSTATSENSEMBLUCSC | 232 | 7 | 7265 | tss;island | 0.256939 | 0.013035 | CHST1 (margin: CHST1) | 4.88 | 1.17e-03 | 1.13e-02 |
|  | 8 | 97505593 | 97505868 | PDFSTATSENSEMBLUCSC | 276 | 7 | 39827 | tss;island | 0.549832 | 0.032320 | SDC2 (margin: SDC2) | 4.88 | 2.33e-03 | 1.52e-02 |
|  | 1 | 224804053 | 224804396 | PDFSTATSENSEMBLUCSC | 344 | 7 | 4244 | island | 0.569051 | 0.028501 | CNIH3; RP11-100E13.1 (margin: CNIH3; RP11-100E13.1) | 4.88 | 1.17e-03 | 1.13e-02 |
|  | 19 | 35068555 | 35068788 | PDFSTATSENSEMBLUCSC | 234 | 4 | 21888 | island | 0.639436 | 0.976902 | Not queried | 4.88 | 1.14e-01 | 1.68e-01 |
|  | 16 | 2334163 | 2334346 | PDFSTATSENSEMBLUCSC | 184 | 4 | 14604 | gene;island | 0.425250 | 0.958822 | Not queried | 4.87 | 2.86e-02 | 6.12e-02 |
|  | 14 | 105045877 | 105045992 | PDFSTATSENSEMBLUCSC | 116 | 4 | 12659 | tss | 0.034244 | 0.654623 | Not queried | 4.87 | 2.86e-02 | 6.12e-02 |
|  | 3 | 46875226 | 46875436 | PDFSTATSENSEMBLUCSC | 211 | 4 | 27755 | gene;island | 0.423694 | 0.951881 | Not queried | 4.87 | 2.86e-02 | 6.12e-02 |
|  | 7 | 25893749 | 25894005 | PDFSTATSENSEMBLUCSC | 257 | 4 | 38323 | island | 0.593158 | 0.157299 | Not queried | 4.87 | 3.43e-01 | 3.84e-01 |
|  | 6 | 31685392 | 31685575 | PDFSTATSENSEMBLUCSC | 184 | 7 | 33940 | gene | 0.607218 | 0.027866 | LY6G6F; MEGT1; LY6G6D; C6orf25; LY6G6C (margin: LY6G6F; MEGT1; LY6G6D; C6orf25; XXbac-BPG32J3.20; LY6G6E; LY6G6C; DDAH2) | 4.87 | 5.83e-04 | 6.47e-03 |
|  | 4 | 4387697 | 4387917 | PDFSTATSENSEMBLUCSC | 221 | 6 | 30267 | tss;island | 0.660379 | 0.964272 | NSG1 (margin: NSG1) | 4.87 | 2.16e-03 | 1.45e-02 |
|  | 8 | 89339404 | 89339819 | PDFSTATSENSEMBLUCSC | 416 | 5 | 40441 | island | 0.302145 | 0.017680 | Not queried | 4.87 | 3.17e-02 | 6.50e-02 |
|  | 10 | 132909113 | 132909470 | PDFSTATSENSEMBLUCSC | 358 | 5 | 4840 | gene | 0.251516 | 0.919686 | TCERG1L (margin: TCERG1L) | 4.87 | 7.94e-03 | 3.19e-02 |
|  | 7 | 63774078 | 63774504 | PDFSTATSENSEMBLUCSC | 427 | 5 | 37023 | gene | 0.213308 | 0.006274 | Not queried | 4.87 | 3.17e-02 | 6.50e-02 |
|  | 17 | 72363447 | 72363634 | PDFSTATSENSEMBLUCSC | 188 | 4 | 17625 | tss | 0.078225 | 0.826515 | Not queried | 4.87 | 2.86e-02 | 6.12e-02 |
|  | 4 | 52917271 | 52917660 | PDFSTATSENSEMBLUCSC | 390 | 10 | 31040 | island | 0.018575 | 0.371187 | SPATA18 (margin: RP11-535C7.1; SPATA18) | 4.86 | 8.93e-03 | 3.45e-02 |
|  | 10 | 135150485 | 135150517 | PDFSTATSENSEMBLUCSC | 33 | 4 | 5461 | tss | 0.045391 | 0.699036 | Not queried | 4.86 | 2.86e-02 | 6.12e-02 |
|  | 8 | 26371551 | 26371569 | PDFSTATSENSEMBLUCSC | 19 | 4 | 39610 | tss | 0.297542 | 0.026548 | Not queried | 4.86 | 2.86e-02 | 6.12e-02 |
|  | 2 | 145275197 | 145275441 | PDFSTATSENSEMBLUCSC | 245 | 5 | 22890 | gene;island | 0.003127 | 0.101742 | ZEB2-AS1; ZEB2 (margin: ZEB2-AS1; ZEB2) | 4.86 | 7.94e-03 | 3.19e-02 |
|  | 4 | 25032472 | 25032564 | PDFSTATSENSEMBLUCSC | 93 | 5 | 30330 | tss | 0.406595 | 0.008616 | Not queried | 4.86 | 1.59e-02 | 5.36e-02 |
|  | 6 | 33041218 | 33041343 | PDFSTATSENSEMBLUCSC | 126 | 5 | 34184 | gene | 0.391908 | 0.900867 | Not queried | 4.86 | 5.56e-02 | 1.00e-01 |
|  | 2 | 119599459 | 119600002 | PDFSTATSENSEMBLUCSC | 544 | 6 | 24644 | island | 0.636856 | 0.979329 | EN1 (margin: RP11-19E11.1; EN1) | 4.86 | 2.16e-03 | 1.45e-02 |
|  | 1 | 44870946 | 44871634 | PDFSTATSENSEMBLUCSC | 689 | 8 | 3181 | island | 0.219642 | 0.009174 | RNF220 (margin: RNF220) | 4.86 | 6.22e-04 | 6.71e-03 |
|  | 5 | 140729429 | 140730092 | PDFSTATSENSEMBLUCSC | 664 | 9 | 31819 | gene;island | 0.207061 | 0.003246 | Not queried | 4.86 | 3.15e-02 | 6.50e-02 |
|  | 5 | 140729429 | 140729813 | PDFSTATSENSEMBLUCSC | 385 | 7 | 32374 | tss | 0.021277 | 0.003246 | Not queried | 4.86 | 7.28e-02 | 1.23e-01 |
|  | 17 | 46703854 | 46704004 | PDFSTATSENSEMBLUCSC | 151 | 6 | 17468 | tss;island | 0.008080 | 0.131316 | HOXB7; HOXB9 (margin: HOXB-AS4; HOXB7; HOXB8; HOXB9; MIR196A1) | 4.86 | 4.33e-03 | 2.52e-02 |
|  | 5 | 1268644 | 1269309 | PDFSTATSENSEMBLUCSC | 666 | 8 | 31531 | gene;island | 0.401198 | 0.977865 | TERT (margin: TERT) | 4.86 | 2.95e-03 | 1.88e-02 |
|  | 12 | 115130855 | 115131549 | PDFSTATSENSEMBLUCSC | 695 | 8 | 10849 | island | 0.549805 | 0.971863 | (margin: TBX3) | 4.86 | 6.99e-03 | 3.19e-02 |
|  | 12 | 114847438 | 114847641 | PDFSTATSENSEMBLUCSC | 204 | 4 | 10035 | tss;island | 0.287407 | 0.954737 | Not queried | 4.86 | 5.71e-02 | 1.00e-01 |
|  | 1 | 248100183 | 248100427 | PDFSTATSENSEMBLUCSC | 245 | 6 | 2372 | tss | 0.715596 | 0.057948 | Not queried | 4.85 | 1.52e-02 | 5.36e-02 |
|  | 15 | 26026152 | 26026414 | PDFSTATSENSEMBLUCSC | 263 | 6 | 13260 | gene | 0.435256 | 0.927717 | Not queried | 4.85 | 2.60e-02 | 6.12e-02 |
|  | 6 | 33144795 | 33145182 | PDFSTATSENSEMBLUCSC | 388 | 5 | 34205 | gene | 0.195683 | 0.886962 | Not queried | 4.85 | 9.52e-02 | 1.53e-01 |
|  | 1 | 182584068 | 182584578 | PDFSTATSENSEMBLUCSC | 511 | 6 | 4024 | island | 0.661253 | 0.059513 | RP11-317P15.4 (margin: RP11-317P15.4; RGS16) | 4.85 | 2.16e-03 | 1.45e-02 |
|  | 19 | 4534986 | 4535582 | PDFSTATSENSEMBLUCSC | 597 | 11 | 21379 | island | 0.143006 | 0.009940 | PLIN5; CTB-50L17.14; LRG1 (margin: RN7SL121P; PLIN5; CTB-50L17.14; LRG1; SEMA6B) | 4.85 | 7.65e-04 | 8.13e-03 |
|  | 13 | 100636183 | 100636299 | PDFSTATSENSEMBLUCSC | 117 | 4 | 11209 | gene;island | 0.413473 | 0.034422 | Not queried | 4.84 | 2.86e-02 | 6.12e-02 |
|  | 5 | 153857789 | 153858102 | PDFSTATSENSEMBLUCSC | 314 | 6 | 33329 | island | 0.401517 | 0.950425 | HAND1 (margin: HAND1; CTB-158E9.1) | 4.84 | 4.33e-03 | 2.52e-02 |
|  | 7 | 64734093 | 64734749 | PDFSTATSENSEMBLUCSC | 657 | 7 | 38527 | island | 0.275941 | 0.013319 | (margin: AC092685.1) | 4.84 | 1.17e-03 | 1.13e-02 |
|  | 10 | 133947518 | 133947916 | PDFSTATSENSEMBLUCSC | 399 | 4 | 4850 | gene;island | 0.488756 | 0.967801 | Not queried | 4.84 | 2.86e-02 | 6.12e-02 |
|  | 17 | 27045043 | 27045176 | PDFSTATSENSEMBLUCSC | 134 | 5 | 16500 | gene | 0.009760 | 0.319314 | RPL23A; RAB34 (margin: RPL23A; SNORD42B; SNORD4A; SNORD42A; SNORD4B; NEK8; AC010761.14; PROCA1; RAB34; AC010761.8; TLCD1) | 4.84 | 7.94e-03 | 3.19e-02 |
|  | 14 | 102026086 | 102026555 | PDFSTATSENSEMBLUCSC | 470 | 11 | 13158 | island | 0.518049 | 0.977254 | DIO3; DIO3OS (margin: DIO3; DIO3OS) | 4.84 | 5.67e-06 | 3.54e-04 |
|  | 19 | 15560076 | 15560314 | PDFSTATSENSEMBLUCSC | 239 | 8 | 19907 | gene;tss;island | 0.520131 | 0.034922 | Not queried | 4.84 | 3.79e-02 | 7.66e-02 |
|  | 19 | 52511285 | 52511900 | PDFSTATSENSEMBLUCSC | 616 | 12 | 22346 | island | 0.009672 | 0.290116 | ZNF615 (margin: ZNF615; ZNF614) | 4.84 | 3.64e-03 | 2.29e-02 |
|  | 19 | 52511502 | 52511900 | PDFSTATSENSEMBLUCSC | 399 | 10 | 21003 | tss | 0.009672 | 0.302971 | ZNF615 (margin: ZNF615; ZNF614) | 4.84 | 6.84e-03 | 3.19e-02 |
|  | 19 | 15051936 | 15052282 | PDFSTATSENSEMBLUCSC | 347 | 4 | 20550 | tss | 0.418280 | 0.937206 | Not queried | 4.84 | 5.71e-02 | 1.00e-01 |
|  | 8 | 99076526 | 99076837 | PDFSTATSENSEMBLUCSC | 312 | 6 | 40483 | island | 0.610609 | 0.965373 | C8orf47 (margin: C8orf47) | 4.84 | 2.16e-03 | 1.45e-02 |
|  | 9 | 138670609 | 138670828 | PDFSTATSENSEMBLUCSC | 220 | 4 | 40792 | gene;island | 0.382950 | 0.960601 | Not queried | 4.83 | 1.14e-01 | 1.68e-01 |
|  | 6 | 167276275 | 167276650 | PDFSTATSENSEMBLUCSC | 376 | 5 | 35339 | tss;island | 0.541495 | 0.039916 | Not queried | 4.83 | 3.17e-02 | 6.50e-02 |
|  | 11 | 2721207 | 2722713 | PDFSTATSENSEMBLUCSC | 1507 | 41 | 6466 | gene;island | 0.467848 | 0.027733 | KCNQ1; KCNQ1OT1 (margin: KCNQ1; KCNQ1OT1) | 4.83 | 3.24e-05 | 1.17e-03 |
|  | 1 | 41444651 | 41444871 | PDFSTATSENSEMBLUCSC | 221 | 9 | 1543 | tss;island | 0.955140 | 0.464707 | Not queried | 4.83 | 2.44e-02 | 6.12e-02 |
|  | 7 | 150945869 | 150946199 | PDFSTATSENSEMBLUCSC | 331 | 5 | 37289 | gene;tss;island | 0.358613 | 0.012071 | RP5-1070G24.2; SMARCD3 (margin: CHPF2; MIR671; RP5-1070G24.2; SMARCD3) | 4.83 | 7.94e-03 | 3.19e-02 |
|  | 10 | 134000009 | 134000410 | PDFSTATSENSEMBLUCSC | 402 | 9 | 5445 | tss;island | 0.538608 | 0.012571 | Not queried | 4.83 | 2.44e-02 | 6.12e-02 |
|  | 11 | 67350153 | 67350663 | PDFSTATSENSEMBLUCSC | 511 | 7 | 7497 | tss;island | 0.696911 | 0.208297 | Not queried | 4.83 | 3.79e-02 | 7.66e-02 |
|  | 6 | 39282164 | 39282532 | PDFSTATSENSEMBLUCSC | 369 | 5 | 36109 | island | 0.511229 | 0.959918 | Not queried | 4.83 | 1.59e-02 | 5.36e-02 |
|  | 5 | 167956306 | 167956425 | PDFSTATSENSEMBLUCSC | 120 | 4 | 32478 | tss | 0.714197 | 0.983773 | Not queried | 4.83 | 2.86e-02 | 6.12e-02 |
|  | 6 | 74162051 | 74162142 | PDFSTATSENSEMBLUCSC | 92 | 6 | 35070 | tss | 0.032792 | 0.404815 | MB21D1 (margin: MTO1; RNU6-975P; MB21D1) | 4.83 | 2.16e-03 | 1.45e-02 |
|  | 17 | 43974804 | 43975063 | PDFSTATSENSEMBLUCSC | 260 | 5 | 16641 | gene;island | 0.739027 | 0.978466 | Not queried | 4.82 | 1.59e-02 | 5.36e-02 |
|  | 2 | 19558368 | 19558569 | PDFSTATSENSEMBLUCSC | 202 | 5 | 24102 | island | 0.428487 | 0.955676 | OSR1 (margin: MIR4757; AC092594.1; OSR1) | 4.82 | 7.94e-03 | 3.19e-02 |
|  | 4 | 175443243 | 175443867 | PDFSTATSENSEMBLUCSC | 625 | 6 | 30184 | gene;island | 0.244689 | 0.006125 | HPGD; RP11-440I14.2 (margin: HPGD; RP11-440I14.2) | 4.82 | 2.16e-03 | 1.45e-02 |
|  | 8 | 22926478 | 22926911 | PDFSTATSENSEMBLUCSC | 434 | 10 | 40144 | island | 0.010998 | 0.381637 | RP11-875O11.2; TNFRSF10B; RP11-875O11.3 (margin: RP11-875O11.2; TNFRSF10B; RP11-875O11.3) | 4.82 | 3.89e-03 | 2.43e-02 |
|  | 2 | 122042939 | 122043074 | PDFSTATSENSEMBLUCSC | 136 | 5 | 23601 | tss;island | 0.055975 | 0.660437 | TFCP2L1 (margin: TFCP2L1) | 4.82 | 7.94e-03 | 3.19e-02 |
|  | 8 | 88884985 | 88885254 | PDFSTATSENSEMBLUCSC | 270 | 4 | 39355 | gene;island | 0.241120 | 0.793421 | Not queried | 4.82 | 1.14e-01 | 1.68e-01 |
|  | 2 | 154336045 | 154336401 | PDFSTATSENSEMBLUCSC | 357 | 5 | 23669 | tss;island | 0.133465 | 0.866415 | Not queried | 4.82 | 1.51e-01 | 2.11e-01 |
|  | 2 | 164593221 | 164593498 | PDFSTATSENSEMBLUCSC | 278 | 4 | 23691 | tss;island | 0.563411 | 0.077666 | Not queried | 4.82 | 2.86e-02 | 6.12e-02 |
|  | 22 | 30901707 | 30901886 | PDFSTATSENSEMBLUCSC | 180 | 4 | 27064 | tss | 0.257882 | 0.888236 | Not queried | 4.82 | 2.86e-02 | 6.12e-02 |
|  | 7 | 2563478 | 2563909 | PDFSTATSENSEMBLUCSC | 432 | 5 | 36804 | gene;island | 0.010983 | 0.347450 | Not queried | 4.82 | 1.59e-02 | 5.36e-02 |
|  | 7 | 96635336 | 96635868 | PDFSTATSENSEMBLUCSC | 533 | 5 | 37103 | gene;island | 0.413214 | 0.947431 | Not queried | 4.82 | 3.17e-02 | 6.50e-02 |
|  | 14 | 94393497 | 94393816 | PDFSTATSENSEMBLUCSC | 320 | 5 | 12193 | gene;tss | 0.480590 | 0.963541 | Not queried | 4.82 | 3.17e-02 | 6.50e-02 |
|  | 4 | 185938720 | 185938989 | PDFSTATSENSEMBLUCSC | 270 | 4 | 30698 | tss;island | 0.251135 | 0.963236 | Not queried | 4.82 | 5.71e-02 | 1.00e-01 |
|  | 20 | 825050 | 825268 | PDFSTATSENSEMBLUCSC | 219 | 5 | 25530 | tss | 0.307883 | 0.012493 | FAM110A; RP11-276A18.2 (margin: FAM110A; RP11-276A18.2; RPS10L) | 4.81 | 7.94e-03 | 3.19e-02 |
|  | 1 | 24513473 | 24514013 | PDFSTATSENSEMBLUCSC | 541 | 8 | 2856 | island | 0.027792 | 0.500168 | IFNLR1 (margin: IFNLR1) | 4.81 | 1.55e-04 | 2.71e-03 |
|  | 12 | 48152204 | 48152585 | PDFSTATSENSEMBLUCSC | 382 | 6 | 9105 | gene;tss;island | 0.188660 | 0.830921 | Not queried | 4.81 | 6.49e-02 | 1.11e-01 |
|  | 7 | 158800869 | 158800986 | PDFSTATSENSEMBLUCSC | 118 | 4 | 38025 | tss;island | 0.258133 | 0.904787 | Not queried | 4.81 | 2.86e-02 | 6.12e-02 |
|  | 12 | 72332386 | 72332624 | PDFSTATSENSEMBLUCSC | 239 | 4 | 9876 | tss | 0.581527 | 0.121272 | Not queried | 4.81 | 2.86e-02 | 6.12e-02 |
|  | 10 | 56561096 | 56561213 | PDFSTATSENSEMBLUCSC | 118 | 4 | 5087 | tss | 0.597302 | 0.062313 | Not queried | 4.81 | 2.86e-02 | 6.12e-02 |
|  | 17 | 47307954 | 47308460 | PDFSTATSENSEMBLUCSC | 507 | 7 | 18521 | island | 0.287656 | 0.007478 | PHOSPHO1 (margin: ABI3; PHOSPHO1) | 4.81 | 4.08e-03 | 2.47e-02 |
|  | 3 | 138067350 | 138067409 | PDFSTATSENSEMBLUCSC | 60 | 4 | 28622 | tss | 0.007703 | 0.001421 | Not queried | 4.81 | 2.86e-02 | 6.12e-02 |
|  | 3 | 196756621 | 196756875 | PDFSTATSENSEMBLUCSC | 255 | 4 | 29815 | island | 0.320751 | 0.009835 | Not queried | 4.81 | 1.14e-01 | 1.68e-01 |
|  | 19 | 37263704 | 37263997 | PDFSTATSENSEMBLUCSC | 294 | 4 | 21945 | island | 0.350624 | 0.018485 | Not queried | 4.81 | 2.86e-02 | 6.12e-02 |
|  | 10 | 77155376 | 77155697 | PDFSTATSENSEMBLUCSC | 322 | 4 | 5866 | island | 0.092872 | 0.618306 | Not queried | 4.81 | 2.86e-02 | 6.12e-02 |
|  | 13 | 79183424 | 79184101 | PDFSTATSENSEMBLUCSC | 678 | 6 | 11796 | island | 0.567422 | 0.971021 | RNF219-AS1 (margin: RNF219-AS1; RP11-52L5.6; POU4F1; RNF219) | 4.81 | 2.16e-03 | 1.45e-02 |
|  | 19 | 36347276 | 36347698 | PDFSTATSENSEMBLUCSC | 423 | 6 | 20716 | tss;island | 0.057667 | 0.656609 | KIRREL2; NPHS1 (margin: KIRREL2; APLP1; NPHS1) | 4.81 | 8.66e-03 | 3.36e-02 |
|  | 10 | 61122307 | 61122570 | PDFSTATSENSEMBLUCSC | 264 | 9 | 4596 | gene | 0.142931 | 0.006156 | FAM13C (margin: FAM13C) | 4.80 | 2.76e-03 | 1.78e-02 |
|  | 15 | 92937992 | 92938295 | PDFSTATSENSEMBLUCSC | 304 | 4 | 13486 | gene;island | 0.367106 | 0.892812 | Not queried | 4.80 | 2.86e-02 | 6.12e-02 |
|  | 1 | 247578859 | 247579312 | PDFSTATSENSEMBLUCSC | 454 | 4 | 2368 | tss | 0.212536 | 0.839635 | Not queried | 4.80 | 2.86e-02 | 6.12e-02 |
|  | 3 | 9994105 | 9994280 | PDFSTATSENSEMBLUCSC | 176 | 4 | 28138 | tss | 0.376372 | 0.050786 | Not queried | 4.80 | 2.86e-02 | 6.12e-02 |
|  | 10 | 131265059 | 131265435 | PDFSTATSENSEMBLUCSC | 377 | 11 | 5440 | tss | 0.495232 | 0.018989 | MGMT (margin: MGMT) | 4.80 | 3.18e-03 | 2.01e-02 |
|  | 11 | 1274018 | 1274269 | PDFSTATSENSEMBLUCSC | 252 | 4 | 6406 | gene;island | 0.231887 | 0.886595 | Not queried | 4.80 | 2.86e-02 | 6.12e-02 |
|  | 7 | 51384519 | 51384676 | PDFSTATSENSEMBLUCSC | 158 | 6 | 37599 | tss | 0.338481 | 0.023004 | COBL (margin: COBL) | 4.79 | 2.16e-03 | 1.45e-02 |
|  | 3 | 148415443 | 148415747 | PDFSTATSENSEMBLUCSC | 305 | 5 | 29577 | island | 0.547104 | 0.079380 | Not queried | 4.79 | 9.52e-02 | 1.53e-01 |
|  | 19 | 54024649 | 54024834 | PDFSTATSENSEMBLUCSC | 186 | 4 | 20205 | gene;island | 0.522894 | 0.032553 | Not queried | 4.79 | 5.71e-02 | 1.00e-01 |
|  | 13 | 113660246 | 113660495 | PDFSTATSENSEMBLUCSC | 250 | 4 | 11267 | gene;island | 0.663368 | 0.981090 | Not queried | 4.79 | 5.71e-02 | 1.00e-01 |
|  | 10 | 116852687 | 116852865 | PDFSTATSENSEMBLUCSC | 179 | 4 | 5380 | tss;island | 0.449220 | 0.026897 | Not queried | 4.79 | 2.86e-02 | 6.12e-02 |
|  | 2 | 25475583 | 25475916 | PDFSTATSENSEMBLUCSC | 334 | 4 | 22603 | gene;tss;island | 0.965851 | 0.461361 | Not queried | 4.79 | 2.86e-02 | 6.12e-02 |
|  | 19 | 35085512 | 35085677 | PDFSTATSENSEMBLUCSC | 166 | 4 | 20693 | tss | 0.515716 | 0.923995 | Not queried | 4.79 | 2.86e-02 | 6.12e-02 |
|  | 19 | 14183018 | 14183212 | PDFSTATSENSEMBLUCSC | 195 | 4 | 20536 | tss;island | 0.633336 | 0.048985 | Not queried | 4.79 | 2.86e-02 | 6.12e-02 |
|  | 17 | 74137399 | 74137522 | PDFSTATSENSEMBLUCSC | 124 | 5 | 17672 | tss | 0.087422 | 0.001948 | Not queried | 4.79 | 3.17e-02 | 6.50e-02 |
|  | 2 | 242754333 | 242754756 | PDFSTATSENSEMBLUCSC | 424 | 5 | 23147 | gene;island | 0.482622 | 0.938353 | Not queried | 4.78 | 5.56e-02 | 1.00e-01 |
|  | 1 | 40105664 | 40105764 | PDFSTATSENSEMBLUCSC | 101 | 5 | 1524 | tss;island | 0.813202 | 0.137318 | HEYL (margin: RP1-144F13.3; HEYL) | 4.78 | 7.94e-03 | 3.19e-02 |
|  | 1 | 1897585 | 1897959 | PDFSTATSENSEMBLUCSC | 375 | 4 | 51 | gene;island | 0.254471 | 0.881600 | Not queried | 4.78 | 2.86e-02 | 6.12e-02 |
|  | 7 | 74020711 | 74021054 | PDFSTATSENSEMBLUCSC | 344 | 5 | 38580 | island | 0.778451 | 0.091797 | (margin: GTF2IRD1) | 4.78 | 7.94e-03 | 3.19e-02 |
|  | 11 | 64981081 | 64981316 | PDFSTATSENSEMBLUCSC | 236 | 5 | 7421 | tss | 0.614964 | 0.954006 | Not queried | 4.78 | 1.59e-02 | 5.36e-02 |
|  | 19 | 50861774 | 50862121 | PDFSTATSENSEMBLUCSC | 348 | 5 | 20174 | gene;island | 0.575607 | 0.971541 | NR1H2; NAPSA (margin: NR1H2; NAPSA; CTB-191K22.6) | 4.78 | 7.94e-03 | 3.19e-02 |
|  | 20 | 825050 | 825798 | PDFSTATSENSEMBLUCSC | 749 | 10 | 25324 | gene;island | 0.421758 | 0.016535 | Not queried | 4.78 | 6.30e-02 | 1.10e-01 |
|  | 8 | 67874178 | 67874366 | PDFSTATSENSEMBLUCSC | 189 | 4 | 40362 | island | 0.683746 | 0.980254 | Not queried | 4.78 | 2.86e-02 | 6.12e-02 |
|  | 20 | 43374239 | 43374406 | PDFSTATSENSEMBLUCSC | 168 | 8 | 25763 | tss;island | 0.418571 | 0.044646 | KCNK15; RP11-445H22.4 (margin: KCNK15; RP11-445H22.4; RIMS4) | 4.78 | 3.11e-04 | 4.77e-03 |
|  | 15 | 83378614 | 83378895 | PDFSTATSENSEMBLUCSC | 282 | 9 | 14385 | island | 0.101681 | 0.007237 | AP3B2; AC105339.1 (margin: AP3B2; AC105339.1) | 4.78 | 4.94e-04 | 6.47e-03 |
|  | 4 | 187475669 | 187475891 | PDFSTATSENSEMBLUCSC | 223 | 4 | 30215 | gene;island | 0.348200 | 0.023849 | Not queried | 4.78 | 5.71e-02 | 1.00e-01 |
|  | 3 | 54121761 | 54122146 | PDFSTATSENSEMBLUCSC | 386 | 6 | 29209 | island | 0.400883 | 0.961718 | Not queried | 4.78 | 1.52e-02 | 5.36e-02 |
|  | 11 | 2444462 | 2445442 | PDFSTATSENSEMBLUCSC | 981 | 17 | 7072 | tss;island | 0.281698 | 0.881357 | TRPM5 (margin: TRPM5) | 4.78 | 1.67e-07 | 2.60e-05 |
|  | 6 | 32372791 | 32373089 | PDFSTATSENSEMBLUCSC | 299 | 5 | 34111 | gene | 0.310354 | 0.696461 | Not queried | 4.78 | 1.59e-02 | 5.36e-02 |
|  | 18 | 580188 | 580334 | PDFSTATSENSEMBLUCSC | 147 | 6 | 19151 | tss | 0.466927 | 0.963835 | CETN1 (margin: CETN1; RN7SKP146) | 4.78 | 4.33e-03 | 2.52e-02 |
|  | 8 | 22014425 | 22014724 | PDFSTATSENSEMBLUCSC | 300 | 8 | 39582 | tss;island | 0.462715 | 0.046217 | SFTPC; LGI3 (margin: SFTPC; BMP1; LGI3) | 4.78 | 1.55e-04 | 2.71e-03 |
|  | 2 | 84743142 | 84743573 | PDFSTATSENSEMBLUCSC | 432 | 9 | 23475 | tss | 0.584738 | 0.029368 | Not queried | 4.77 | 4.00e-02 | 8.03e-02 |
|  | 3 | 46853850 | 46854311 | PDFSTATSENSEMBLUCSC | 462 | 7 | 29061 | island | 0.361665 | 0.966916 | PRSS50; PRSS45; PRSS44 (margin: PRSS50; PRSS45; PRSS44) | 4.77 | 1.17e-03 | 1.13e-02 |
|  | 1 | 198828157 | 198828249 | PDFSTATSENSEMBLUCSC | 93 | 4 | 2159 | tss | 0.661354 | 0.983984 | Not queried | 4.77 | 2.86e-02 | 6.12e-02 |
|  | 2 | 97215789 | 97216131 | PDFSTATSENSEMBLUCSC | 343 | 4 | 22782 | gene;island | 0.482164 | 0.041152 | Not queried | 4.77 | 2.86e-02 | 6.12e-02 |
|  | 7 | 140773422 | 140774161 | PDFSTATSENSEMBLUCSC | 740 | 6 | 38967 | island | 0.700704 | 0.049542 | TMEM178B (margin: TMEM178B) | 4.77 | 2.16e-03 | 1.45e-02 |
|  | 16 | 30124803 | 30125403 | PDFSTATSENSEMBLUCSC | 601 | 8 | 15180 | tss | 0.098766 | 0.779381 | GDPD3; MAPK3 (margin: RP11-455F5.3; RP11-455F5.4; GDPD3; MAPK3) | 4.77 | 6.99e-03 | 3.19e-02 |
|  | 8 | 1992965 | 1993125 | PDFSTATSENSEMBLUCSC | 161 | 4 | 39537 | tss | 0.012843 | 0.202370 | Not queried | 4.77 | 5.71e-02 | 1.00e-01 |
|  | 6 | 127441060 | 127441199 | PDFSTATSENSEMBLUCSC | 140 | 4 | 34469 | gene;island | 0.360805 | 0.908062 | Not queried | 4.77 | 2.86e-02 | 6.12e-02 |
|  | 17 | 25798741 | 25799018 | PDFSTATSENSEMBLUCSC | 278 | 5 | 17205 | tss | 0.140731 | 0.819446 | KSR1 (margin: KSR1; RP11-720N19.2) | 4.77 | 7.94e-03 | 3.19e-02 |
|  | 6 | 94129412 | 94129697 | PDFSTATSENSEMBLUCSC | 286 | 8 | 35125 | tss | 0.814441 | 0.127732 | EPHA7 (margin: EPHA7) | 4.77 | 1.04e-02 | 3.96e-02 |
|  | 3 | 134369919 | 134370241 | PDFSTATSENSEMBLUCSC | 323 | 9 | 28607 | tss | 0.274664 | 0.896400 | EPHB1; KY (margin: EPHB1; KY) | 4.76 | 4.11e-05 | 1.17e-03 |
|  | 6 | 168426063 | 168426413 | PDFSTATSENSEMBLUCSC | 351 | 5 | 34557 | gene | 0.510988 | 0.965963 | Not queried | 4.76 | 5.56e-02 | 1.00e-01 |
|  | 13 | 100547192 | 100547367 | PDFSTATSENSEMBLUCSC | 176 | 5 | 11207 | gene;island | 0.627685 | 0.978577 | Not queried | 4.76 | 3.17e-02 | 6.50e-02 |
|  | 5 | 141293785 | 141294040 | PDFSTATSENSEMBLUCSC | 256 | 4 | 33262 | island | 0.470029 | 0.938905 | Not queried | 4.76 | 2.86e-02 | 6.12e-02 |
|  | 22 | 37215611 | 37215656 | PDFSTATSENSEMBLUCSC | 46 | 4 | 27112 | tss;island | 0.294269 | 0.869626 | Not queried | 4.76 | 2.86e-02 | 6.12e-02 |
|  | 4 | 619162 | 619314 | PDFSTATSENSEMBLUCSC | 153 | 4 | 30226 | tss | 0.180026 | 0.816801 | Not queried | 4.76 | 2.86e-02 | 6.12e-02 |
|  | 5 | 72746710 | 72746990 | PDFSTATSENSEMBLUCSC | 281 | 4 | 32874 | island | 0.527144 | 0.075329 | Not queried | 4.76 | 3.43e-01 | 3.84e-01 |
|  | 11 | 120973085 | 120973274 | PDFSTATSENSEMBLUCSC | 190 | 4 | 7766 | tss | 0.139059 | 0.796112 | Not queried | 4.76 | 2.00e-01 | 2.55e-01 |
|  | 13 | 113121632 | 113121958 | PDFSTATSENSEMBLUCSC | 327 | 4 | 11924 | island | 0.510449 | 0.935739 | Not queried | 4.76 | 2.86e-02 | 6.12e-02 |
|  | 13 | 74708078 | 74708194 | PDFSTATSENSEMBLUCSC | 117 | 6 | 11477 | tss | 0.116825 | 0.006948 | KLF12 (margin: KLF12) | 4.76 | 2.16e-03 | 1.45e-02 |
|  | 4 | 141489963 | 141490428 | PDFSTATSENSEMBLUCSC | 466 | 10 | 30608 | tss | 0.624971 | 0.978035 | UCP1 (margin: RN7SL152P; UCP1) | 4.75 | 1.08e-05 | 4.82e-04 |
|  | 10 | 31609882 | 31610093 | PDFSTATSENSEMBLUCSC | 212 | 8 | 4554 | gene;island | 0.049984 | 0.642516 | ZEB1; ZEB1-AS1 (margin: ZEB1; ZEB1-AS1) | 4.75 | 1.55e-04 | 2.71e-03 |
|  | 6 | 27840105 | 27840257 | PDFSTATSENSEMBLUCSC | 153 | 6 | 34727 | tss | 0.014174 | 0.000516 | HIST1H3I; HIST1H4L (margin: HIST1H2AL; HIST1H2BPS2; HIST1H1B; HIST1H3I; HIST1H4L) | 4.75 | 2.16e-03 | 1.45e-02 |
|  | 1 | 12251718 | 12251934 | PDFSTATSENSEMBLUCSC | 217 | 4 | 232 | gene;island | 0.489634 | 0.978834 | Not queried | 4.75 | 5.71e-02 | 1.00e-01 |
|  | 11 | 58869663 | 58870075 | PDFSTATSENSEMBLUCSC | 413 | 5 | 8301 | island | 0.972173 | 0.501543 | Not queried | 4.75 | 4.21e-01 | 4.59e-01 |
|  | 14 | 104094619 | 104095156 | PDFSTATSENSEMBLUCSC | 538 | 5 | 12652 | tss;island | 0.008228 | 0.214421 | Not queried | 4.75 | 1.51e-01 | 2.11e-01 |
|  | 2 | 145147129 | 145147558 | PDFSTATSENSEMBLUCSC | 430 | 5 | 22889 | gene;island | 0.338227 | 0.821686 | ZEB2 (margin: ZEB2) | 4.75 | 7.94e-03 | 3.19e-02 |
|  | 12 | 56075367 | 56075511 | PDFSTATSENSEMBLUCSC | 145 | 4 | 9166 | gene | 0.405482 | 0.021943 | Not queried | 4.75 | 2.86e-02 | 6.12e-02 |
|  | 8 | 102380960 | 102381107 | PDFSTATSENSEMBLUCSC | 148 | 4 | 39849 | tss | 0.171142 | 0.894536 | Not queried | 4.75 | 1.14e-01 | 1.68e-01 |
|  | 11 | 2721243 | 2722713 | PDFSTATSENSEMBLUCSC | 1471 | 40 | 7077 | tss | 0.477564 | 0.027740 | KCNQ1; KCNQ1OT1 (margin: KCNQ1; KCNQ1OT1) | 4.75 | 5.98e-05 | 1.62e-03 |
|  | 14 | 104195181 | 104195666 | PDFSTATSENSEMBLUCSC | 486 | 5 | 12243 | gene;island | 0.960675 | 0.590539 | ZFYVE21 (margin: ZFYVE21; Y\_RNA; PPP1R13B) | 4.75 | 7.94e-03 | 3.19e-02 |
|  | 6 | 52859607 | 52859823 | PDFSTATSENSEMBLUCSC | 217 | 4 | 34371 | gene;island | 0.015724 | 0.291433 | Not queried | 4.74 | 2.86e-02 | 6.12e-02 |
|  | 11 | 133804526 | 133804886 | PDFSTATSENSEMBLUCSC | 361 | 4 | 8971 | island | 0.477834 | 0.954423 | Not queried | 4.74 | 1.14e-01 | 1.68e-01 |
|  | 11 | 115530932 | 115531299 | PDFSTATSENSEMBLUCSC | 368 | 5 | 8830 | island | 0.867874 | 0.986832 | Not queried | 4.74 | 3.17e-02 | 6.50e-02 |
|  | 12 | 2224410 | 2224755 | PDFSTATSENSEMBLUCSC | 346 | 4 | 9002 | gene;island | 0.311476 | 0.917483 | Not queried | 4.74 | 1.14e-01 | 1.68e-01 |
|  | 19 | 1307393 | 1307668 | PDFSTATSENSEMBLUCSC | 276 | 4 | 21213 | island | 0.633108 | 0.976982 | Not queried | 4.74 | 2.86e-02 | 6.12e-02 |
|  | 21 | 34444104 | 34444382 | PDFSTATSENSEMBLUCSC | 279 | 4 | 26402 | gene;island | 0.581057 | 0.977391 | Not queried | 4.74 | 2.86e-02 | 6.12e-02 |
|  | 6 | 167263025 | 167263454 | PDFSTATSENSEMBLUCSC | 430 | 6 | 34555 | gene | 0.308054 | 0.906135 | RPS6KA2 (margin: RPS6KA2; RP11-514O12.4) | 4.73 | 2.16e-03 | 1.45e-02 |
|  | 14 | 100069535 | 100069840 | PDFSTATSENSEMBLUCSC | 306 | 4 | 12204 | gene;island | 0.642260 | 0.969614 | Not queried | 4.73 | 5.71e-02 | 1.00e-01 |
|  | 10 | 97200911 | 97201172 | PDFSTATSENSEMBLUCSC | 262 | 4 | 4688 | gene | 0.449790 | 0.942793 | Not queried | 4.73 | 2.86e-02 | 6.12e-02 |
|  | 12 | 12848977 | 12849748 | PDFSTATSENSEMBLUCSC | 772 | 11 | 10250 | island | 0.074570 | 0.008318 | GPR19 (margin: RP11-180M15.3; GPR19) | 4.73 | 5.53e-04 | 6.47e-03 |
|  | 1 | 206680459 | 206681158 | PDFSTATSENSEMBLUCSC | 700 | 8 | 4140 | island | 0.151715 | 0.799167 | Not queried | 4.73 | 2.81e-02 | 6.12e-02 |
|  | 13 | 111972854 | 111972997 | PDFSTATSENSEMBLUCSC | 144 | 6 | 11542 | tss | 0.583758 | 0.953972 | TEX29 (margin: TEX29) | 4.73 | 2.16e-03 | 1.45e-02 |
|  | 10 | 57389676 | 57390047 | PDFSTATSENSEMBLUCSC | 372 | 4 | 5754 | island | 0.889844 | 0.307772 | Not queried | 4.73 | 5.71e-02 | 1.00e-01 |
|  | 10 | 50323706 | 50323832 | PDFSTATSENSEMBLUCSC | 127 | 5 | 5070 | tss;island | 0.435328 | 0.951864 | VSTM4 (margin: FAM170B-AS1; VSTM4) | 4.73 | 7.94e-03 | 3.19e-02 |
|  | 22 | 50492970 | 50493212 | PDFSTATSENSEMBLUCSC | 243 | 5 | 27611 | island | 0.701834 | 0.982871 | Not queried | 4.73 | 3.17e-02 | 6.50e-02 |
|  | 8 | 49468684 | 49468988 | PDFSTATSENSEMBLUCSC | 305 | 6 | 40283 | island | 0.552412 | 0.969892 | RP11-770E5.1; RP11-567J20.2 (margin: RP11-770E5.1; RP11-567J20.2) | 4.72 | 2.16e-03 | 1.45e-02 |
|  | 6 | 36645500 | 36645886 | PDFSTATSENSEMBLUCSC | 387 | 5 | 34940 | tss;island | 0.265222 | 0.772500 | Not queried | 4.72 | 1.59e-02 | 5.36e-02 |
|  | 6 | 43276478 | 43276781 | PDFSTATSENSEMBLUCSC | 304 | 9 | 36161 | island | 0.419961 | 0.962723 | RP11-480N24.3; CRIP3; ZNF318 (margin: SLC22A7; RP11-480N24.3; CRIP3; ZNF318) | 4.72 | 4.11e-05 | 1.17e-03 |
|  | 11 | 14913752 | 14914012 | PDFSTATSENSEMBLUCSC | 261 | 8 | 7166 | tss | 0.149965 | 0.005475 | CYP2R1 (margin: CYP2R1) | 4.72 | 1.09e-03 | 1.10e-02 |
|  | 22 | 25960673 | 25960712 | PDFSTATSENSEMBLUCSC | 40 | 4 | 27031 | tss;island | 0.141644 | 0.010958 | Not queried | 4.72 | 2.86e-02 | 6.12e-02 |
|  | 1 | 3143682 | 3144209 | PDFSTATSENSEMBLUCSC | 528 | 5 | 109 | gene | 0.219199 | 0.839469 | Not queried | 4.72 | 3.17e-02 | 6.50e-02 |
|  | 11 | 17555063 | 17555319 | PDFSTATSENSEMBLUCSC | 257 | 4 | 6524 | gene | 0.472155 | 0.962084 | Not queried | 4.72 | 2.86e-02 | 6.12e-02 |
|  | 5 | 133451237 | 133451474 | PDFSTATSENSEMBLUCSC | 238 | 7 | 32287 | tss | 0.090258 | 0.814876 | TCF7 (margin: TCF7) | 4.72 | 1.17e-03 | 1.13e-02 |
|  | 10 | 26504950 | 26505503 | PDFSTATSENSEMBLUCSC | 554 | 9 | 5617 | island | 0.873031 | 0.093050 | Not queried | 4.72 | 2.58e-01 | 3.16e-01 |
|  | 19 | 7741454 | 7741838 | PDFSTATSENSEMBLUCSC | 385 | 5 | 20404 | tss | 0.055868 | 0.831206 | C19orf59; CTD-3214H19.16 (margin: CTD-3214H19.12; RETN; C19orf59; CTD-3214H19.16; TRAPPC5; FCER2) | 4.71 | 7.94e-03 | 3.19e-02 |
|  | 19 | 7741602 | 7741955 | PDFSTATSENSEMBLUCSC | 354 | 5 | 21462 | island | 0.055868 | 0.908037 | C19orf59; CTD-3214H19.16 (margin: CTD-3214H19.12; RETN; C19orf59; CTD-3214H19.16; TRAPPC5; FCER2) | 4.71 | 7.94e-03 | 3.19e-02 |
|  | 2 | 233497957 | 233498164 | PDFSTATSENSEMBLUCSC | 208 | 6 | 23953 | tss | 0.378354 | 0.024925 | EFHD1 (margin: EFHD1) | 4.71 | 2.16e-03 | 1.45e-02 |
|  | 4 | 55524145 | 55524400 | PDFSTATSENSEMBLUCSC | 256 | 4 | 30005 | gene;island | 0.674113 | 0.987221 | Not queried | 4.71 | 2.86e-02 | 6.12e-02 |
|  | 4 | 62382932 | 62383240 | PDFSTATSENSEMBLUCSC | 309 | 4 | 30012 | gene;island | 0.744892 | 0.101402 | Not queried | 4.71 | 2.86e-02 | 6.12e-02 |
|  | 7 | 128828120 | 128828765 | PDFSTATSENSEMBLUCSC | 646 | 9 | 38907 | island | 0.537283 | 0.968194 | SMO (margin: SMO) | 4.71 | 2.88e-04 | 4.58e-03 |
|  | 12 | 118407009 | 118407487 | PDFSTATSENSEMBLUCSC | 479 | 6 | 10045 | tss;island | 0.432803 | 0.027135 | KSR2 (margin: KSR2) | 4.71 | 8.66e-03 | 3.36e-02 |
|  | 11 | 94134446 | 94135029 | PDFSTATSENSEMBLUCSC | 584 | 10 | 8737 | island | 0.593387 | 0.981610 | GPR83 (margin: GPR83) | 4.71 | 1.05e-03 | 1.08e-02 |
|  | 20 | 30605656 | 30606095 | PDFSTATSENSEMBLUCSC | 440 | 5 | 25387 | gene;island | 0.368351 | 0.024602 | CCM2L (margin: RNA5SP481; CCM2L; RP1-310O13.7) | 4.71 | 7.94e-03 | 3.19e-02 |
|  | 3 | 118663470 | 118663697 | PDFSTATSENSEMBLUCSC | 228 | 4 | 27903 | gene | 0.094396 | 0.677194 | Not queried | 4.71 | 5.71e-02 | 1.00e-01 |
|  | 1 | 2776972 | 2777244 | PDFSTATSENSEMBLUCSC | 273 | 4 | 2519 | island | 0.138463 | 0.812642 | Not queried | 4.71 | 2.86e-02 | 6.12e-02 |
|  | 13 | 20806477 | 20806813 | PDFSTATSENSEMBLUCSC | 337 | 8 | 11331 | tss;island | 0.556864 | 0.984305 | GJB6 (margin: GJB6) | 4.71 | 1.55e-04 | 2.71e-03 |
|  | 2 | 233497957 | 233498223 | PDFSTATSENSEMBLUCSC | 267 | 7 | 23068 | gene;island | 0.369572 | 0.026663 | EFHD1 (margin: EFHD1) | 4.70 | 5.83e-04 | 6.47e-03 |
|  | 10 | 44198369 | 44198617 | PDFSTATSENSEMBLUCSC | 249 | 4 | 5703 | island | 0.341941 | 0.976712 | Not queried | 4.70 | 5.71e-02 | 1.00e-01 |
|  | 11 | 63258509 | 63258803 | PDFSTATSENSEMBLUCSC | 295 | 8 | 8369 | island | 0.557900 | 0.966761 | HRASLS5 (margin: HRASLS5) | 4.70 | 6.99e-03 | 3.19e-02 |
|  | 11 | 63258712 | 63258803 | PDFSTATSENSEMBLUCSC | 92 | 6 | 7375 | tss | 0.542762 | 0.966761 | Not queried | 4.70 | 4.11e-02 | 8.14e-02 |
|  | 16 | 89225495 | 89225922 | PDFSTATSENSEMBLUCSC | 428 | 6 | 16244 | island | 0.620175 | 0.970603 | Not queried | 4.70 | 1.52e-02 | 5.36e-02 |
|  | 15 | 90039409 | 90039913 | PDFSTATSENSEMBLUCSC | 505 | 10 | 14437 | island | 0.594427 | 0.056155 | RHCG (margin: RHCG; LINC00928) | 4.70 | 1.08e-05 | 4.82e-04 |
|  | 15 | 90039805 | 90039913 | PDFSTATSENSEMBLUCSC | 109 | 6 | 13888 | tss | 0.617174 | 0.058457 | RHCG (margin: RHCG; LINC00928) | 4.70 | 2.16e-03 | 1.45e-02 |
|  | 7 | 32982914 | 32983012 | PDFSTATSENSEMBLUCSC | 99 | 4 | 37528 | tss;island | 0.018934 | 0.259195 | Not queried | 4.70 | 2.86e-02 | 6.12e-02 |
|  | 5 | 1257034 | 1257221 | PDFSTATSENSEMBLUCSC | 188 | 5 | 31530 | gene;island | 0.493279 | 0.964162 | TERT (margin: SLC6A18; TERT) | 4.70 | 7.94e-03 | 3.19e-02 |
|  | 1 | 20878760 | 20879029 | PDFSTATSENSEMBLUCSC | 270 | 5 | 2795 | island | 0.709544 | 0.983762 | FAM43B (margin: FAM43B) | 4.70 | 7.94e-03 | 3.19e-02 |
|  | 6 | 32977983 | 32978775 | PDFSTATSENSEMBLUCSC | 793 | 16 | 34887 | tss;island | 0.461174 | 0.941066 | Not queried | 4.70 | 2.60e-02 | 6.12e-02 |
|  | 1 | 245753302 | 245753635 | PDFSTATSENSEMBLUCSC | 334 | 4 | 1118 | gene;island | 0.391781 | 0.924121 | Not queried | 4.70 | 2.00e-01 | 2.55e-01 |
|  | 21 | 42218932 | 42219299 | PDFSTATSENSEMBLUCSC | 368 | 8 | 26686 | island | 0.591361 | 0.986267 | DSCAM (margin: DSCAM) | 4.70 | 2.95e-03 | 1.88e-02 |
|  | 2 | 1926366 | 1926907 | PDFSTATSENSEMBLUCSC | 542 | 6 | 22541 | gene | 0.094606 | 0.649657 | MYT1L (margin: MYT1L) | 4.70 | 8.66e-03 | 3.36e-02 |
|  | 2 | 628881 | 629121 | PDFSTATSENSEMBLUCSC | 241 | 5 | 24009 | island | 0.098198 | 0.015110 | Not queried | 4.70 | 3.10e-01 | 3.66e-01 |
|  | 15 | 90792609 | 90792717 | PDFSTATSENSEMBLUCSC | 109 | 4 | 13898 | tss | 0.387415 | 0.010934 | Not queried | 4.70 | 5.71e-02 | 1.00e-01 |
|  | 1 | 161067636 | 161068387 | PDFSTATSENSEMBLUCSC | 752 | 12 | 3898 | island | 0.318415 | 0.006583 | KLHDC9; PFDN2 (margin: RP11-544M22.8; KLHDC9; PVRL4; PFDN2) | 4.70 | 5.56e-03 | 3.16e-02 |
|  | 8 | 15397729 | 15398333 | PDFSTATSENSEMBLUCSC | 605 | 7 | 39213 | gene | 0.225315 | 0.005372 | Not queried | 4.70 | 1.75e-02 | 5.79e-02 |
|  | 17 | 62777648 | 62777777 | PDFSTATSENSEMBLUCSC | 130 | 6 | 16734 | gene;tss;island | 0.060473 | 0.609135 | hsa-mir-6080; PLEKHM1P (margin: hsa-mir-6080; RP11-927P21.4; PLEKHM1P) | 4.70 | 2.16e-03 | 1.45e-02 |
|  | 7 | 4254699 | 4255011 | PDFSTATSENSEMBLUCSC | 313 | 4 | 36835 | gene | 0.312501 | 0.917934 | Not queried | 4.70 | 2.86e-02 | 6.12e-02 |
|  | 8 | 74205882 | 74205946 | PDFSTATSENSEMBLUCSC | 65 | 4 | 39768 | tss;island | 0.007875 | 0.000597 | Not queried | 4.69 | 8.86e-01 | 8.89e-01 |
|  | 18 | 580188 | 580435 | PDFSTATSENSEMBLUCSC | 248 | 7 | 19347 | island | 0.480626 | 0.963675 | CETN1 (margin: CETN1; RN7SKP146) | 4.69 | 2.33e-03 | 1.52e-02 |
|  | 5 | 128300967 | 128301185 | PDFSTATSENSEMBLUCSC | 219 | 6 | 32260 | tss | 0.155037 | 0.011585 | SLC27A6 (margin: SLC27A6) | 4.69 | 2.16e-03 | 1.45e-02 |
|  | 1 | 43389643 | 43389888 | PDFSTATSENSEMBLUCSC | 246 | 5 | 3154 | island | 0.449313 | 0.030480 | SLC2A1 (margin: SLC2A1) | 4.69 | 7.94e-03 | 3.19e-02 |
|  | 6 | 1608396 | 1608667 | PDFSTATSENSEMBLUCSC | 272 | 4 | 35373 | island | 0.949167 | 0.390169 | Not queried | 4.69 | 2.86e-02 | 6.12e-02 |
|  | 13 | 113633379 | 113634042 | PDFSTATSENSEMBLUCSC | 664 | 8 | 11263 | gene;island | 0.208671 | 0.896625 | MCF2L (margin: MCF2L; MCF2L-AS1) | 4.69 | 6.22e-04 | 6.71e-03 |
|  | 6 | 31913759 | 31914305 | PDFSTATSENSEMBLUCSC | 547 | 9 | 33995 | gene | 0.179012 | 0.845800 | Not queried | 4.68 | 1.36e-01 | 1.94e-01 |
|  | 6 | 24911001 | 24911615 | PDFSTATSENSEMBLUCSC | 615 | 12 | 35497 | island | 0.522011 | 0.055435 | FAM65B (margin: FAM65B) | 4.68 | 3.33e-05 | 1.17e-03 |
|  | 4 | 141489799 | 141490428 | PDFSTATSENSEMBLUCSC | 630 | 12 | 31327 | island | 0.624971 | 0.977642 | UCP1 (margin: RN7SL152P; UCP1) | 4.68 | 7.40e-07 | 7.21e-05 |
|  | 17 | 64188403 | 64188644 | PDFSTATSENSEMBLUCSC | 242 | 4 | 17595 | tss;island | 0.023921 | 0.344727 | Not queried | 4.68 | 2.00e-01 | 2.55e-01 |
|  | 4 | 8271196 | 8271414 | PDFSTATSENSEMBLUCSC | 219 | 6 | 30296 | tss | 0.430225 | 0.946298 | HTRA3 (margin: HTRA3) | 4.68 | 2.16e-03 | 1.45e-02 |
|  | 13 | 74707860 | 74708194 | PDFSTATSENSEMBLUCSC | 335 | 8 | 11779 | island | 0.143541 | 0.006948 | KLF12 (margin: KLF12) | 4.68 | 4.66e-03 | 2.68e-02 |
|  | 22 | 31536476 | 31536784 | PDFSTATSENSEMBLUCSC | 309 | 5 | 27075 | tss | 0.601958 | 0.134791 | Not queried | 4.68 | 1.59e-02 | 5.36e-02 |
|  | 1 | 197880384 | 197880923 | PDFSTATSENSEMBLUCSC | 540 | 6 | 2156 | tss;island | 0.418197 | 0.897991 | LHX9 (margin: C1orf53; LHX9) | 4.68 | 2.16e-03 | 1.45e-02 |
|  | 4 | 141347993 | 141349096 | PDFSTATSENSEMBLUCSC | 1104 | 16 | 31325 | island | 0.175649 | 0.006070 | CLGN (margin: CLGN) | 4.68 | 3.92e-04 | 5.86e-03 |
|  | 7 | 1250038 | 1251125 | PDFSTATSENSEMBLUCSC | 1088 | 17 | 38089 | island | 0.112095 | 0.773256 | (margin: ) | 4.68 | 1.21e-05 | 5.36e-04 |
|  | 3 | 120626881 | 120627170 | PDFSTATSENSEMBLUCSC | 290 | 6 | 29392 | island | 0.616596 | 0.046476 | STXBP5L (margin: STXBP5L) | 4.67 | 2.16e-03 | 1.45e-02 |
|  | 6 | 36237982 | 36238230 | PDFSTATSENSEMBLUCSC | 249 | 7 | 34935 | tss | 0.032196 | 0.459046 | PNPLA1 (margin: PNPLA1) | 4.67 | 5.83e-04 | 6.47e-03 |
|  | 11 | 66034681 | 66035276 | PDFSTATSENSEMBLUCSC | 596 | 17 | 6727 | gene | 0.480074 | 0.050703 | KLC2; RAB1B; RP11-867G23.1; RP11-867G23.2 (margin: KLC2; RAB1B; CNIH2; RP11-755F10.3; RP11-867G23.1; RP11-867G23.2; RP11-867G23.3; RP11-867G23.4) | 4.67 | 2.52e-04 | 4.14e-03 |
|  | 4 | 3475041 | 3475372 | PDFSTATSENSEMBLUCSC | 332 | 6 | 29907 | gene;island | 0.346135 | 0.942381 | Not queried | 4.67 | 2.40e-01 | 2.95e-01 |
|  | 1 | 149144820 | 149145243 | PDFSTATSENSEMBLUCSC | 424 | 5 | 3704 | island | 0.228465 | 0.893290 | RNU1-114P (margin: RNU1-114P) | 4.67 | 7.94e-03 | 3.19e-02 |
|  | 6 | 116937920 | 116938100 | PDFSTATSENSEMBLUCSC | 181 | 4 | 34452 | gene | 0.397709 | 0.040831 | Not queried | 4.67 | 2.86e-02 | 6.12e-02 |
|  | 1 | 68515788 | 68516453 | PDFSTATSENSEMBLUCSC | 666 | 11 | 574 | gene | 0.674010 | 0.978243 | GNG12-AS1; DIRAS3; ARL5AP3 (margin: GNG12-AS1; RP4-609E1.2; AL157407.1; DIRAS3; ARL5AP3) | 4.67 | 1.04e-03 | 1.08e-02 |
|  | 11 | 101454626 | 101454996 | PDFSTATSENSEMBLUCSC | 371 | 8 | 8756 | island | 0.596327 | 0.093083 | Not queried | 4.67 | 8.30e-02 | 1.40e-01 |
|  | 21 | 45705175 | 45705742 | PDFSTATSENSEMBLUCSC | 568 | 10 | 26582 | tss;island | 0.569780 | 0.984970 | AIRE (margin: AIRE) | 4.67 | 1.05e-03 | 1.08e-02 |
|  | 1 | 27817084 | 27817404 | PDFSTATSENSEMBLUCSC | 321 | 4 | 1414 | tss;island | 0.009577 | 0.410004 | Not queried | 4.67 | 1.14e-01 | 1.68e-01 |
|  | 4 | 46391259 | 46391929 | PDFSTATSENSEMBLUCSC | 671 | 13 | 29995 | gene;island | 0.910914 | 0.215073 | RP11-436F23.1; GABRA2 (margin: RP11-436F23.1; GABRA2) | 4.67 | 1.91e-03 | 1.45e-02 |
|  | 11 | 22214333 | 22214683 | PDFSTATSENSEMBLUCSC | 351 | 5 | 7201 | tss | 0.702834 | 0.086185 | Not queried | 4.67 | 1.59e-02 | 5.36e-02 |
|  | 8 | 35092687 | 35092876 | PDFSTATSENSEMBLUCSC | 190 | 4 | 39643 | tss;island | 0.927979 | 0.259406 | Not queried | 4.66 | 5.71e-02 | 1.00e-01 |
|  | 17 | 72580901 | 72581014 | PDFSTATSENSEMBLUCSC | 114 | 4 | 17628 | tss | 0.175580 | 0.881672 | Not queried | 4.66 | 5.71e-02 | 1.00e-01 |
|  | 11 | 46354562 | 46354635 | PDFSTATSENSEMBLUCSC | 74 | 6 | 7275 | tss | 0.771264 | 0.987943 | DGKZ (margin: CREB3L1; DGKZ) | 4.66 | 2.16e-03 | 1.45e-02 |
|  | 6 | 24877462 | 24877729 | PDFSTATSENSEMBLUCSC | 268 | 4 | 33594 | gene | 0.180265 | 0.855502 | Not queried | 4.66 | 2.86e-02 | 6.12e-02 |
|  | 6 | 43276570 | 43276781 | PDFSTATSENSEMBLUCSC | 212 | 8 | 34996 | tss | 0.405866 | 0.955916 | RP11-480N24.3; CRIP3; ZNF318 (margin: SLC22A7; RP11-480N24.3; CRIP3; ZNF318) | 4.66 | 1.55e-04 | 2.71e-03 |
|  | 1 | 100111713 | 100111949 | PDFSTATSENSEMBLUCSC | 237 | 4 | 648 | gene | 0.002838 | 0.132837 | Not queried | 4.66 | 2.86e-02 | 6.12e-02 |
|  | 10 | 123356205 | 123356336 | PDFSTATSENSEMBLUCSC | 132 | 4 | 5405 | tss | 0.478228 | 0.931252 | Not queried | 4.66 | 2.00e-01 | 2.55e-01 |
|  | 15 | 41056021 | 41056252 | PDFSTATSENSEMBLUCSC | 232 | 5 | 13594 | tss | 0.200461 | 0.011103 | GCHFR (margin: RP11-532F12.6; GCHFR; C15orf62; RMDN3; DNAJC17) | 4.66 | 7.94e-03 | 3.19e-02 |
|  | 13 | 100497453 | 100497789 | PDFSTATSENSEMBLUCSC | 337 | 4 | 11206 | gene | 0.757520 | 0.980077 | Not queried | 4.66 | 2.86e-02 | 6.12e-02 |
|  | 10 | 115998843 | 115999174 | PDFSTATSENSEMBLUCSC | 332 | 6 | 6152 | island | 0.151883 | 0.009628 | VWA2 (margin: TDRD1; VWA2; CTB-1144G6.5) | 4.66 | 8.66e-03 | 3.36e-02 |
|  | 10 | 115998843 | 115999009 | PDFSTATSENSEMBLUCSC | 167 | 4 | 5376 | tss | 0.114793 | 0.009482 | Not queried | 4.66 | 5.71e-02 | 1.00e-01 |
|  | 1 | 20878760 | 20878928 | PDFSTATSENSEMBLUCSC | 169 | 4 | 1315 | tss | 0.757062 | 0.985898 | Not queried | 4.66 | 2.86e-02 | 6.12e-02 |
|  | 17 | 40805728 | 40805956 | PDFSTATSENSEMBLUCSC | 229 | 5 | 18346 | island | 0.919291 | 0.314589 | (margin: ATP5LP7; HMGB3P27; TUBG2) | 4.66 | 7.94e-03 | 3.19e-02 |
|  | 11 | 118550443 | 118550644 | PDFSTATSENSEMBLUCSC | 202 | 4 | 7737 | tss | 0.195436 | 0.836141 | Not queried | 4.65 | 2.86e-02 | 6.12e-02 |
|  | 2 | 33953195 | 33953437 | PDFSTATSENSEMBLUCSC | 243 | 4 | 24216 | island | 0.287377 | 0.951617 | Not queried | 4.65 | 5.71e-02 | 1.00e-01 |
|  | 10 | 114135847 | 114136073 | PDFSTATSENSEMBLUCSC | 227 | 6 | 4762 | gene | 0.157526 | 0.794079 | ACSL5 (margin: ACSL5) | 4.65 | 2.16e-03 | 1.45e-02 |
|  | 6 | 21666391 | 21666666 | PDFSTATSENSEMBLUCSC | 276 | 5 | 34664 | tss | 0.280060 | 0.917560 | LINC00340 (margin: LINC00340) | 4.65 | 7.94e-03 | 3.19e-02 |
|  | 10 | 134977981 | 134978147 | PDFSTATSENSEMBLUCSC | 167 | 5 | 4887 | gene;island | 0.484765 | 0.963840 | KNDC1 (margin: KNDC1) | 4.65 | 7.94e-03 | 3.19e-02 |
|  | 14 | 90167459 | 90167917 | PDFSTATSENSEMBLUCSC | 459 | 5 | 13066 | island | 0.455687 | 0.042763 | Not queried | 4.65 | 9.52e-02 | 1.53e-01 |
|  | 6 | 150463775 | 150464265 | PDFSTATSENSEMBLUCSC | 491 | 8 | 36568 | island | 0.040524 | 0.550569 | PPP1R14C (margin: PPP1R14C) | 4.65 | 1.86e-03 | 1.45e-02 |
|  | 10 | 123357993 | 123358317 | PDFSTATSENSEMBLUCSC | 325 | 6 | 5406 | tss;island | 0.043592 | 0.486375 | FGFR2 (margin: FGFR2) | 4.64 | 2.16e-03 | 1.45e-02 |
|  | 20 | 61809557 | 61809932 | PDFSTATSENSEMBLUCSC | 376 | 9 | 26343 | island | 0.957203 | 0.473031 | Not queried | 4.64 | 1.88e-02 | 6.12e-02 |
|  | 8 | 15397637 | 15398333 | PDFSTATSENSEMBLUCSC | 697 | 8 | 40099 | island | 0.314539 | 0.009705 | TUSC3 (margin: TUSC3) | 4.64 | 1.04e-02 | 3.96e-02 |
|  | 17 | 72889323 | 72890001 | PDFSTATSENSEMBLUCSC | 679 | 11 | 18728 | island | 0.513969 | 0.953032 | FADS6 (margin: FADS6) | 4.64 | 8.51e-05 | 2.07e-03 |
|  | 6 | 69344406 | 69344702 | PDFSTATSENSEMBLUCSC | 297 | 5 | 35055 | tss;island | 0.428933 | 0.034555 | Not queried | 4.64 | 1.59e-02 | 5.36e-02 |
|  | 17 | 73874443 | 73874790 | PDFSTATSENSEMBLUCSC | 348 | 7 | 18765 | island | 0.012213 | 0.389366 | RP11-552F3.9; TRIM47; TRIM65 (margin: RP11-552F3.9; TRIM47; TRIM65) | 4.64 | 2.33e-03 | 1.52e-02 |
|  | 5 | 71404443 | 71404897 | PDFSTATSENSEMBLUCSC | 455 | 4 | 31640 | gene;island | 0.519950 | 0.963579 | Not queried | 4.64 | 2.86e-02 | 6.12e-02 |
|  | 2 | 134785392 | 134785775 | PDFSTATSENSEMBLUCSC | 384 | 5 | 24748 | island | 0.341059 | 0.953679 | Not queried | 4.64 | 1.59e-02 | 5.36e-02 |
|  | 4 | 156297854 | 156298427 | PDFSTATSENSEMBLUCSC | 574 | 12 | 31390 | island | 0.736511 | 0.039893 | AC097467.2; MAP9 (margin: AC097467.2; RP11-27G13.5; MAP9; RP11-27G13.3; RP11-27G13.4) | 4.64 | 2.32e-03 | 1.52e-02 |
|  | 10 | 26855907 | 26856128 | PDFSTATSENSEMBLUCSC | 222 | 4 | 4537 | gene;island | 0.557531 | 0.975374 | Not queried | 4.64 | 2.86e-02 | 6.12e-02 |
|  | 10 | 125651526 | 125651726 | PDFSTATSENSEMBLUCSC | 201 | 6 | 5420 | tss | 0.517902 | 0.962387 | Not queried | 4.64 | 1.52e-02 | 5.36e-02 |
|  | 14 | 36003367 | 36003528 | PDFSTATSENSEMBLUCSC | 162 | 4 | 12065 | gene | 0.264672 | 0.013810 | Not queried | 4.63 | 2.86e-02 | 6.12e-02 |
|  | 7 | 82792053 | 82792411 | PDFSTATSENSEMBLUCSC | 359 | 9 | 38625 | island | 0.148853 | 0.010428 | PCLO (margin: PCLO) | 4.63 | 1.65e-04 | 2.80e-03 |
|  | 10 | 131843517 | 131843798 | PDFSTATSENSEMBLUCSC | 282 | 4 | 6270 | island | 0.373826 | 0.033362 | Not queried | 4.63 | 2.86e-02 | 6.12e-02 |
|  | 10 | 129533998 | 129534411 | PDFSTATSENSEMBLUCSC | 414 | 5 | 6248 | island | 0.453016 | 0.893488 | FOXI2; AL391005.1 (margin: FOXI2; AL391005.1) | 4.63 | 7.94e-03 | 3.19e-02 |
|  | 6 | 168197410 | 168198163 | PDFSTATSENSEMBLUCSC | 754 | 9 | 36648 | island | 0.573375 | 0.976432 | AL009178.1; C6orf123 (margin: AL009178.1; C6orf123) | 4.63 | 7.82e-04 | 8.17e-03 |
|  | 6 | 168197625 | 168198163 | PDFSTATSENSEMBLUCSC | 539 | 7 | 35343 | tss | 0.573375 | 0.963023 | AL009178.1; C6orf123 (margin: AL009178.1; C6orf123) | 4.63 | 6.99e-03 | 3.19e-02 |
|  | 5 | 87956769 | 87957260 | PDFSTATSENSEMBLUCSC | 492 | 5 | 31675 | gene;island | 0.305375 | 0.012434 | Not queried | 4.63 | 3.17e-02 | 6.50e-02 |
|  | 2 | 192015279 | 192015918 | PDFSTATSENSEMBLUCSC | 640 | 10 | 22962 | gene | 0.294069 | 0.016357 | STAT4 (margin: STAT4) | 4.63 | 1.08e-05 | 4.82e-04 |
|  | 6 | 10886946 | 10887047 | PDFSTATSENSEMBLUCSC | 102 | 7 | 34633 | tss | 0.351650 | 0.971862 | RP11-637O19.3; SYCP2L (margin: RP11-637O19.3; SYCP2L; RP11-637O19.2; GCM2) | 4.63 | 1.17e-03 | 1.13e-02 |
|  | 14 | 23540729 | 23540892 | PDFSTATSENSEMBLUCSC | 164 | 4 | 12024 | gene | 0.122364 | 0.454293 | Not queried | 4.63 | 5.71e-02 | 1.00e-01 |
|  | 6 | 30226874 | 30227326 | PDFSTATSENSEMBLUCSC | 453 | 10 | 34766 | tss | 0.553470 | 0.057815 | Not queried | 4.62 | 1.85e-02 | 6.12e-02 |
|  | 5 | 140305947 | 140306249 | PDFSTATSENSEMBLUCSC | 303 | 8 | 32357 | tss | 0.957368 | 0.507255 | PCDHA1; PCDHA2; PCDHA3; PCDHA4; PCDHA5; PCDHA6; PCDHA7; PCDHA8; PCDHA9; PCDHA10; PCDHA11; PCDHA12; PCDHA13; PCDHAC1 (margin: PCDHA1; PCDHA2; PCDHA3; PCDHA4; PCDHA5; PCDHA6; PCDHA7; PCDHA8; PCDHA9; PCDHA10; PCDHA11; PCDHA12; PCDHA13; PCDHAC1) | 4.62 | 1.55e-04 | 2.71e-03 |
|  | 10 | 135049991 | 135050355 | PDFSTATSENSEMBLUCSC | 365 | 13 | 5455 | tss;island | 0.656817 | 0.984329 | VENTX (margin: KNDC1; UTF1; VENTX; MIR202) | 4.62 | 3.65e-06 | 2.42e-04 |
|  | 7 | 93519855 | 93520036 | PDFSTATSENSEMBLUCSC | 182 | 6 | 37091 | gene | 0.787939 | 0.218208 | GNGT1; AC002076.10; TFPI2 (margin: GNGT1; AC002076.10; TFPI2) | 4.62 | 2.16e-03 | 1.45e-02 |
|  | 19 | 15575533 | 15575685 | PDFSTATSENSEMBLUCSC | 153 | 4 | 20563 | tss | 0.283985 | 0.822829 | Not queried | 4.62 | 1.14e-01 | 1.68e-01 |
|  | 19 | 3369702 | 3369820 | PDFSTATSENSEMBLUCSC | 119 | 4 | 19714 | gene;island | 0.622067 | 0.963925 | Not queried | 4.62 | 2.86e-02 | 6.12e-02 |
|  | 6 | 118972731 | 118973353 | PDFSTATSENSEMBLUCSC | 623 | 12 | 36444 | island | 0.202432 | 0.027744 | Not queried | 4.62 | 2.42e-02 | 6.12e-02 |
|  | 1 | 53067637 | 53067965 | PDFSTATSENSEMBLUCSC | 329 | 5 | 1632 | tss;island | 0.479150 | 0.957508 | Not queried | 4.61 | 9.52e-02 | 1.53e-01 |
|  | 6 | 29323191 | 29323330 | PDFSTATSENSEMBLUCSC | 140 | 4 | 33652 | gene | 0.248442 | 0.831708 | Not queried | 4.61 | 3.43e-01 | 3.84e-01 |
|  | 17 | 66194978 | 66195485 | PDFSTATSENSEMBLUCSC | 508 | 5 | 16740 | gene | 0.609403 | 0.978534 | LOC440461 (margin: RP11-147L13.8; LOC440461) | 4.61 | 7.94e-03 | 3.19e-02 |
|  | 1 | 22351256 | 22351864 | PDFSTATSENSEMBLUCSC | 609 | 8 | 1330 | tss;island | 0.713643 | 0.049692 | Not queried | 4.61 | 4.99e-02 | 9.81e-02 |
|  | 8 | 33457226 | 33457822 | PDFSTATSENSEMBLUCSC | 597 | 8 | 40215 | island | 0.498978 | 0.965104 | DUSP26 (margin: RP11-317N12.1; DUSP26) | 4.61 | 1.55e-04 | 2.71e-03 |
|  | 3 | 45837898 | 45838338 | PDFSTATSENSEMBLUCSC | 441 | 10 | 29051 | island | 0.671566 | 0.981258 | SLC6A20 (margin: SLC6A20) | 4.61 | 1.50e-03 | 1.41e-02 |
|  | 3 | 45838037 | 45838338 | PDFSTATSENSEMBLUCSC | 302 | 8 | 28271 | tss | 0.632896 | 0.979962 | SLC6A20 (margin: SLC6A20) | 4.61 | 1.04e-02 | 3.96e-02 |
|  | 6 | 32297615 | 32297861 | PDFSTATSENSEMBLUCSC | 247 | 5 | 34101 | gene | 0.780550 | 0.973407 | C6orf10 (margin: HNRNPA1P2; C6orf10) | 4.61 | 7.94e-03 | 3.19e-02 |
|  | 16 | 53407421 | 53407808 | PDFSTATSENSEMBLUCSC | 388 | 7 | 15924 | island | 0.158443 | 0.008663 | Not queried | 4.61 | 1.75e-02 | 5.79e-02 |
|  | 5 | 153859646 | 153859973 | PDFSTATSENSEMBLUCSC | 328 | 4 | 33331 | island | 0.617541 | 0.928061 | Not queried | 4.61 | 2.86e-02 | 6.12e-02 |
|  | 15 | 41056021 | 41056369 | PDFSTATSENSEMBLUCSC | 349 | 7 | 14045 | island | 0.223257 | 0.016246 | GCHFR (margin: RP11-532F12.6; GCHFR; C15orf62; RMDN3; DNAJC17) | 4.61 | 5.83e-04 | 6.47e-03 |
|  | 6 | 32822857 | 32823116 | PDFSTATSENSEMBLUCSC | 260 | 4 | 34880 | tss | 0.167729 | 0.932011 | Not queried | 4.61 | 5.71e-02 | 1.00e-01 |
|  | 19 | 54982500 | 54982896 | PDFSTATSENSEMBLUCSC | 397 | 4 | 20224 | gene;island | 0.221413 | 0.012721 | Not queried | 4.60 | 2.86e-02 | 6.12e-02 |
|  | 16 | 15595854 | 15596423 | PDFSTATSENSEMBLUCSC | 570 | 9 | 14675 | gene | 0.395222 | 0.938668 | RP11-1021N1.1; C16orf45 (margin: RP11-1021N1.1; C16orf45) | 4.60 | 2.76e-03 | 1.78e-02 |
|  | 11 | 30607888 | 30608198 | PDFSTATSENSEMBLUCSC | 311 | 10 | 8155 | island | 0.352776 | 0.019465 | RP5-1024C24.1; MPPED2 (margin: RP5-1024C24.1; MPPED2) | 4.60 | 1.08e-05 | 4.82e-04 |
|  | 18 | 35147056 | 35147532 | PDFSTATSENSEMBLUCSC | 477 | 9 | 19478 | island | 0.666053 | 0.979735 | Not queried | 4.60 | 1.88e-02 | 6.12e-02 |
|  | 19 | 18548845 | 18549040 | PDFSTATSENSEMBLUCSC | 196 | 4 | 19935 | gene | 0.460419 | 0.031702 | Not queried | 4.60 | 2.86e-02 | 6.12e-02 |
|  | 19 | 2428350 | 2428677 | PDFSTATSENSEMBLUCSC | 328 | 4 | 19689 | gene;tss;island | 0.643145 | 0.981583 | Not queried | 4.60 | 2.86e-02 | 6.12e-02 |
|  | 7 | 45961074 | 45961569 | PDFSTATSENSEMBLUCSC | 496 | 17 | 37587 | tss;island | 0.971996 | 0.540501 | IGFBP3 (margin: IGFBP3) | 4.60 | 6.06e-03 | 3.19e-02 |
|  | 11 | 111847692 | 111847984 | PDFSTATSENSEMBLUCSC | 293 | 5 | 7695 | tss | 0.652508 | 0.033033 | Not queried | 4.60 | 3.10e-01 | 3.66e-01 |
|  | 5 | 80529067 | 80529135 | PDFSTATSENSEMBLUCSC | 69 | 4 | 32153 | tss | 0.976388 | 0.661248 | Not queried | 4.60 | 2.86e-02 | 6.12e-02 |
|  | 5 | 501863 | 502074 | PDFSTATSENSEMBLUCSC | 212 | 4 | 31510 | gene;island | 0.528996 | 0.944938 | Not queried | 4.60 | 2.86e-02 | 6.12e-02 |
|  | 10 | 49674251 | 49674530 | PDFSTATSENSEMBLUCSC | 280 | 4 | 4579 | gene;island | 0.596863 | 0.985607 | Not queried | 4.60 | 5.71e-02 | 1.00e-01 |
|  | 10 | 134918671 | 134918974 | PDFSTATSENSEMBLUCSC | 304 | 5 | 4885 | gene;island | 0.083218 | 0.813265 | GPR123 (margin: GPR123) | 4.60 | 7.94e-03 | 3.19e-02 |
|  | 2 | 134024093 | 134024618 | PDFSTATSENSEMBLUCSC | 526 | 5 | 22880 | gene;island | 0.466739 | 0.043943 | Not queried | 4.60 | 5.56e-02 | 1.00e-01 |
|  | 3 | 183948058 | 183948313 | PDFSTATSENSEMBLUCSC | 256 | 5 | 28754 | tss | 0.498710 | 0.967538 | Not queried | 4.60 | 1.51e-01 | 2.11e-01 |
|  | 1 | 7765245 | 7765564 | PDFSTATSENSEMBLUCSC | 320 | 4 | 188 | gene;island | 0.376404 | 0.883046 | Not queried | 4.60 | 3.43e-01 | 3.84e-01 |
|  | 3 | 71633826 | 71634044 | PDFSTATSENSEMBLUCSC | 219 | 4 | 28442 | tss;island | 0.070288 | 0.002188 | Not queried | 4.60 | 5.71e-02 | 1.00e-01 |
|  | 2 | 25391656 | 25391911 | PDFSTATSENSEMBLUCSC | 256 | 10 | 23231 | tss | 0.510038 | 0.969985 | POMC (margin: RP11-509E16.1; POMC) | 4.60 | 1.08e-05 | 4.82e-04 |
|  | 13 | 114875001 | 114875412 | PDFSTATSENSEMBLUCSC | 412 | 5 | 11314 | gene;island | 0.896972 | 0.385201 | RASA3; RASA3-IT1 (margin: RASA3; RASA3-IT1) | 4.60 | 7.94e-03 | 3.19e-02 |
|  | 2 | 118943676 | 118944144 | PDFSTATSENSEMBLUCSC | 469 | 5 | 24642 | island | 0.031558 | 0.440591 | Not queried | 4.60 | 9.52e-02 | 1.53e-01 |
|  | 1 | 18435619 | 18435897 | PDFSTATSENSEMBLUCSC | 279 | 4 | 270 | gene;island | 0.231124 | 0.008790 | Not queried | 4.59 | 1.14e-01 | 1.68e-01 |
|  | 11 | 134145889 | 134146324 | PDFSTATSENSEMBLUCSC | 436 | 9 | 8981 | island | 0.496692 | 0.972447 | GLB1L3 (margin: ACAD8; GLB1L3) | 4.59 | 2.88e-04 | 4.58e-03 |
|  | 6 | 168388195 | 168388388 | PDFSTATSENSEMBLUCSC | 194 | 4 | 36651 | island | 0.468447 | 0.957996 | Not queried | 4.59 | 2.86e-02 | 6.12e-02 |
|  | 16 | 56622951 | 56623549 | PDFSTATSENSEMBLUCSC | 599 | 10 | 15946 | island | 0.636101 | 0.983984 | MT3 (margin: MT3) | 4.59 | 1.08e-05 | 4.82e-04 |
|  | 12 | 2162130 | 2162491 | PDFSTATSENSEMBLUCSC | 362 | 4 | 10155 | island | 0.229417 | 0.013470 | Not queried | 4.59 | 2.86e-02 | 6.12e-02 |
|  | 4 | 114683176 | 114683375 | PDFSTATSENSEMBLUCSC | 200 | 7 | 30556 | tss;island | 0.054651 | 0.582743 | CAMK2D (margin: CAMK2D) | 4.59 | 5.83e-04 | 6.47e-03 |
|  | 13 | 98829521 | 98829666 | PDFSTATSENSEMBLUCSC | 146 | 5 | 11508 | tss | 0.623984 | 0.975697 | Not queried | 4.59 | 1.59e-02 | 5.36e-02 |
|  | 1 | 209979111 | 209979470 | PDFSTATSENSEMBLUCSC | 360 | 5 | 988 | gene | 0.043426 | 0.003595 | Not queried | 4.59 | 4.21e-01 | 4.59e-01 |
|  | 20 | 3776212 | 3776357 | PDFSTATSENSEMBLUCSC | 146 | 7 | 25568 | tss | 0.618304 | 0.063233 | CDC25B (margin: CDC25B; CENPB) | 4.59 | 1.17e-03 | 1.13e-02 |
|  | 4 | 25031951 | 25032564 | PDFSTATSENSEMBLUCSC | 614 | 9 | 30957 | island | 0.406595 | 0.010702 | LGI2 (margin: LGI2) | 4.59 | 1.23e-03 | 1.18e-02 |
|  | 6 | 32632106 | 32632338 | PDFSTATSENSEMBLUCSC | 233 | 6 | 34125 | gene;island | 0.498356 | 0.923006 | Not queried | 4.59 | 2.60e-02 | 6.12e-02 |
|  | 2 | 133175192 | 133175397 | PDFSTATSENSEMBLUCSC | 206 | 4 | 22875 | gene;island | 0.380205 | 0.971256 | Not queried | 4.58 | 5.71e-02 | 1.00e-01 |
|  | 12 | 130646022 | 130646497 | PDFSTATSENSEMBLUCSC | 476 | 6 | 10118 | tss;island | 0.108050 | 0.594564 | FZD10; FZD10-AS1 (margin: FZD10; FZD10-AS1) | 4.58 | 2.16e-03 | 1.45e-02 |
|  | 7 | 143042585 | 143042798 | PDFSTATSENSEMBLUCSC | 214 | 4 | 37253 | gene;island | 0.602386 | 0.971679 | Not queried | 4.58 | 2.86e-02 | 6.12e-02 |
|  | 7 | 98870608 | 98870835 | PDFSTATSENSEMBLUCSC | 228 | 4 | 37750 | tss | 0.215163 | 0.877077 | Not queried | 4.58 | 2.86e-02 | 6.12e-02 |
|  | 3 | 32859377 | 32859587 | PDFSTATSENSEMBLUCSC | 211 | 7 | 28968 | island | 0.550407 | 0.978075 | TRIM71 (margin: TRIM71) | 4.58 | 5.83e-04 | 6.47e-03 |
|  | 12 | 24715478 | 24715564 | PDFSTATSENSEMBLUCSC | 87 | 6 | 9605 | tss;island | 0.529605 | 0.041805 | RP11-444D3.1 (margin: RP11-444D3.1; LINC00477) | 4.58 | 2.16e-03 | 1.45e-02 |
|  | 19 | 12203029 | 12203349 | PDFSTATSENSEMBLUCSC | 321 | 5 | 21592 | island | 0.662093 | 0.987344 | Not queried | 4.58 | 1.59e-02 | 5.36e-02 |
|  | 6 | 84233917 | 84234463 | PDFSTATSENSEMBLUCSC | 547 | 8 | 34403 | gene | 0.497537 | 0.950523 | PRSS35 (margin: PRSS35) | 4.58 | 1.04e-02 | 3.96e-02 |
|  | 11 | 50227079 | 50227265 | PDFSTATSENSEMBLUCSC | 187 | 4 | 8278 | island | 0.936296 | 0.308197 | Not queried | 4.58 | 2.00e-01 | 2.55e-01 |
|  | 2 | 84743142 | 84743743 | PDFSTATSENSEMBLUCSC | 602 | 12 | 24469 | island | 0.443367 | 0.027236 | DNAH6 (margin: DNAH6) | 4.58 | 6.81e-03 | 3.19e-02 |
|  | 8 | 26371353 | 26371569 | PDFSTATSENSEMBLUCSC | 217 | 5 | 40175 | island | 0.366026 | 0.029441 | Not queried | 4.57 | 5.56e-02 | 1.00e-01 |
|  | 5 | 135415693 | 135416613 | PDFSTATSENSEMBLUCSC | 921 | 16 | 33146 | island | 0.538887 | 0.970870 | VTRNA2-1 (margin: VTRNA2-1) | 4.57 | 2.33e-08 | 5.67e-06 |
|  | 13 | 36944433 | 36944649 | PDFSTATSENSEMBLUCSC | 217 | 7 | 11395 | tss | 0.734431 | 0.984903 | SPG20OS; SPG20 (margin: SPG20OS; SPG20) | 4.57 | 4.08e-03 | 2.47e-02 |
|  | 6 | 28984234 | 28985069 | PDFSTATSENSEMBLUCSC | 836 | 24 | 35610 | island | 0.014891 | 0.325226 | (margin: ZNF311; OR2AD1P) | 4.57 | 1.25e-05 | 5.44e-04 |
|  | 19 | 281291 | 281467 | PDFSTATSENSEMBLUCSC | 177 | 4 | 19621 | gene | 0.577296 | 0.963211 | Not queried | 4.57 | 2.86e-02 | 6.12e-02 |
|  | 4 | 619162 | 619392 | PDFSTATSENSEMBLUCSC | 231 | 6 | 30723 | island | 0.257760 | 0.874224 | PDE6B (margin: PDE6B) | 4.57 | 2.16e-03 | 1.45e-02 |
|  | 2 | 19558375 | 19558569 | PDFSTATSENSEMBLUCSC | 195 | 4 | 23205 | tss | 0.472474 | 0.963104 | Not queried | 4.57 | 2.86e-02 | 6.12e-02 |
|  | 19 | 48947560 | 48947631 | PDFSTATSENSEMBLUCSC | 72 | 4 | 20124 | gene;tss;island | 0.912098 | 0.308520 | Not queried | 4.57 | 2.86e-02 | 6.12e-02 |
|  | 5 | 180632114 | 180632555 | PDFSTATSENSEMBLUCSC | 442 | 5 | 32565 | tss;island | 0.003649 | 0.020788 | Not queried | 4.57 | 1.51e-01 | 2.11e-01 |
|  | 10 | 85324700 | 85325062 | PDFSTATSENSEMBLUCSC | 363 | 5 | 5892 | island | 0.423305 | 0.970634 | Not queried | 4.57 | 9.52e-02 | 1.53e-01 |
|  | 16 | 31227272 | 31227515 | PDFSTATSENSEMBLUCSC | 244 | 4 | 14734 | gene;island | 0.545760 | 0.048844 | Not queried | 4.57 | 2.86e-02 | 6.12e-02 |
|  | 7 | 128828120 | 128828599 | PDFSTATSENSEMBLUCSC | 480 | 8 | 37891 | tss | 0.541563 | 0.966394 | SMO (margin: SMO) | 4.57 | 1.09e-03 | 1.10e-02 |
|  | 12 | 82152229 | 82152625 | PDFSTATSENSEMBLUCSC | 397 | 5 | 9229 | gene;island | 0.364491 | 0.017559 | Not queried | 4.57 | 5.56e-02 | 1.00e-01 |
|  | 10 | 30026015 | 30026143 | PDFSTATSENSEMBLUCSC | 129 | 5 | 5010 | tss;island | 0.345416 | 0.020275 | SVIL (margin: SVIL) | 4.57 | 7.94e-03 | 3.19e-02 |
|  | 10 | 88126273 | 88126306 | PDFSTATSENSEMBLUCSC | 34 | 6 | 5197 | tss | 0.562582 | 0.050001 | GRID1 (margin: GRID1) | 4.57 | 2.16e-03 | 1.45e-02 |
|  | 1 | 3664522 | 3664680 | PDFSTATSENSEMBLUCSC | 159 | 4 | 1188 | tss;island | 0.429000 | 0.958854 | Not queried | 4.56 | 2.86e-02 | 6.12e-02 |
|  | 13 | 112758379 | 112758625 | PDFSTATSENSEMBLUCSC | 247 | 4 | 11915 | island | 0.521589 | 0.957016 | Not queried | 4.56 | 2.86e-02 | 6.12e-02 |
|  | 10 | 100991959 | 100992222 | PDFSTATSENSEMBLUCSC | 264 | 4 | 4705 | gene;island | 0.460207 | 0.935718 | Not queried | 4.56 | 2.86e-02 | 6.12e-02 |
|  | 2 | 48982509 | 48982832 | PDFSTATSENSEMBLUCSC | 324 | 4 | 22674 | gene | 0.624134 | 0.973991 | Not queried | 4.56 | 2.00e-01 | 2.55e-01 |
|  | 7 | 4275634 | 4275962 | PDFSTATSENSEMBLUCSC | 329 | 4 | 36837 | gene | 0.295457 | 0.894120 | Not queried | 4.56 | 2.00e-01 | 2.55e-01 |
|  | 13 | 84455525 | 84455742 | PDFSTATSENSEMBLUCSC | 218 | 4 | 11185 | gene;island | 0.500021 | 0.041256 | Not queried | 4.56 | 2.86e-02 | 6.12e-02 |
|  | 1 | 213124368 | 213124685 | PDFSTATSENSEMBLUCSC | 318 | 5 | 1002 | gene;island | 0.197750 | 0.852924 | Not queried | 4.56 | 3.17e-02 | 6.50e-02 |
|  | 19 | 58446312 | 58446988 | PDFSTATSENSEMBLUCSC | 677 | 12 | 22487 | island | 0.929211 | 0.368589 | ZNF418 (margin: ZNF418; ZNF256) | 4.56 | 2.96e-06 | 2.02e-04 |
|  | 12 | 5936877 | 5937105 | PDFSTATSENSEMBLUCSC | 229 | 4 | 9021 | gene;island | 0.307452 | 0.871839 | Not queried | 4.56 | 2.86e-02 | 6.12e-02 |
|  | 10 | 103603722 | 103604019 | PDFSTATSENSEMBLUCSC | 298 | 5 | 5312 | tss | 0.929895 | 0.402403 | KCNIP2; C10orf76 (margin: KCNIP2; C10orf76) | 4.56 | 7.94e-03 | 3.19e-02 |
|  | 7 | 4174939 | 4175403 | PDFSTATSENSEMBLUCSC | 465 | 5 | 36830 | gene | 0.407087 | 0.861989 | SDK1 (margin: SDK1) | 4.56 | 7.94e-03 | 3.19e-02 |
|  | 11 | 134122771 | 134122914 | PDFSTATSENSEMBLUCSC | 144 | 4 | 6992 | gene;tss;island | 0.230012 | 0.011636 | Not queried | 4.56 | 2.86e-02 | 6.12e-02 |
|  | 6 | 29454557 | 29455532 | PDFSTATSENSEMBLUCSC | 976 | 15 | 33659 | gene | 0.106273 | 0.745605 | MAS1L; RPS17P1 (margin: XXbac-BPG13B8.10; MAS1LP1; MAS1L; RPS17P1) | 4.55 | 5.62e-05 | 1.53e-03 |
|  | 4 | 174452835 | 174453287 | PDFSTATSENSEMBLUCSC | 453 | 5 | 30181 | gene;island | 0.197028 | 0.831787 | Not queried | 4.55 | 9.52e-02 | 1.53e-01 |
|  | 14 | 65005951 | 65006359 | PDFSTATSENSEMBLUCSC | 409 | 10 | 12458 | tss;island | 0.015115 | 0.312320 | HSPA2; RP11-973N13.4 (margin: ZBTB1; HSPA2; PPP1R36; RP11-973N13.4; RP11-973N13.5) | 4.55 | 1.15e-02 | 4.29e-02 |
|  | 14 | 37117706 | 37117947 | PDFSTATSENSEMBLUCSC | 242 | 4 | 12789 | island | 0.958257 | 0.518647 | Not queried | 4.55 | 1.14e-01 | 1.68e-01 |
|  | 6 | 292329 | 292596 | PDFSTATSENSEMBLUCSC | 268 | 4 | 33529 | gene;island | 0.021508 | 0.338319 | Not queried | 4.55 | 2.86e-02 | 6.12e-02 |
|  | 14 | 101528123 | 101528369 | PDFSTATSENSEMBLUCSC | 247 | 8 | 12632 | tss | 0.140906 | 0.807695 | Not queried | 4.55 | 2.07e-02 | 6.12e-02 |
|  | 1 | 176998705 | 176999034 | PDFSTATSENSEMBLUCSC | 330 | 4 | 896 | gene;tss | 0.098466 | 0.610253 | Not queried | 4.55 | 1.14e-01 | 1.68e-01 |
|  | 13 | 80915447 | 80915525 | PDFSTATSENSEMBLUCSC | 79 | 4 | 11492 | tss;island | 0.229647 | 0.014833 | Not queried | 4.55 | 2.86e-02 | 6.12e-02 |
|  | 1 | 109656145 | 109656440 | PDFSTATSENSEMBLUCSC | 296 | 6 | 664 | gene;tss;island | 0.797781 | 0.078390 | Not queried | 4.55 | 6.49e-02 | 1.11e-01 |
|  | 12 | 107078410 | 107078680 | PDFSTATSENSEMBLUCSC | 271 | 5 | 9281 | gene | 0.703471 | 0.975639 | Not queried | 4.55 | 3.10e-01 | 3.66e-01 |
|  | 3 | 192444985 | 192445380 | PDFSTATSENSEMBLUCSC | 396 | 5 | 28087 | gene | 0.442110 | 0.034672 | FGF12; RNU1-20P (margin: FGF12; RNU1-20P) | 4.55 | 7.94e-03 | 3.19e-02 |
|  | 2 | 232765174 | 232765416 | PDFSTATSENSEMBLUCSC | 243 | 4 | 25189 | island | 0.681788 | 0.042360 | Not queried | 4.55 | 5.71e-02 | 1.00e-01 |
|  | 19 | 2273706 | 2274042 | PDFSTATSENSEMBLUCSC | 337 | 4 | 21276 | island | 0.203937 | 0.817605 | Not queried | 4.54 | 5.71e-02 | 1.00e-01 |
|  | 1 | 55007670 | 55008516 | PDFSTATSENSEMBLUCSC | 847 | 11 | 3299 | island | 0.013112 | 0.105780 | ACOT11 (margin: HNRNPA1P63; ACOT11) | 4.54 | 2.84e-06 | 1.96e-04 |
|  | 3 | 123813191 | 123813504 | PDFSTATSENSEMBLUCSC | 314 | 8 | 28548 | tss | 0.391695 | 0.923100 | KALRN (margin: KALRN) | 4.54 | 4.66e-03 | 2.68e-02 |
|  | 10 | 44701885 | 44702076 | PDFSTATSENSEMBLUCSC | 192 | 4 | 5705 | island | 0.381811 | 0.957709 | Not queried | 4.54 | 2.86e-02 | 6.12e-02 |
|  | 6 | 117197952 | 117198743 | PDFSTATSENSEMBLUCSC | 792 | 12 | 36434 | island | 0.496516 | 0.042537 | RFX6 (margin: RFX6) | 4.54 | 7.17e-05 | 1.92e-03 |
|  | 1 | 53904065 | 53904319 | PDFSTATSENSEMBLUCSC | 255 | 4 | 540 | gene;island | 0.601806 | 0.972136 | Not queried | 4.54 | 2.86e-02 | 6.12e-02 |
|  | 1 | 224804053 | 224804149 | PDFSTATSENSEMBLUCSC | 97 | 4 | 2278 | tss | 0.509056 | 0.029036 | Not queried | 4.54 | 2.86e-02 | 6.12e-02 |
|  | 2 | 242811830 | 242811962 | PDFSTATSENSEMBLUCSC | 133 | 4 | 25313 | island | 0.517049 | 0.972912 | Not queried | 4.54 | 5.71e-02 | 1.00e-01 |
|  | 11 | 43600032 | 43600779 | PDFSTATSENSEMBLUCSC | 748 | 8 | 8215 | island | 0.578726 | 0.086232 | Not queried | 4.54 | 6.50e-02 | 1.11e-01 |
|  | 6 | 166571782 | 166572045 | PDFSTATSENSEMBLUCSC | 264 | 5 | 34543 | gene;island | 0.721659 | 0.980093 | Not queried | 4.54 | 1.51e-01 | 2.11e-01 |
|  | 17 | 2951666 | 2951736 | PDFSTATSENSEMBLUCSC | 71 | 4 | 17850 | island | 0.572517 | 0.252945 | Not queried | 4.54 | 3.43e-01 | 3.84e-01 |
|  | 18 | 32556626 | 32557027 | PDFSTATSENSEMBLUCSC | 402 | 7 | 19231 | tss;island | 0.147140 | 0.009193 | MAPRE2 (margin: MAPRE2) | 4.53 | 1.17e-03 | 1.13e-02 |
|  | 19 | 4153364 | 4153603 | PDFSTATSENSEMBLUCSC | 240 | 4 | 20360 | tss | 0.080918 | 0.475799 | Not queried | 4.53 | 5.71e-02 | 1.00e-01 |
|  | 6 | 16238434 | 16238691 | PDFSTATSENSEMBLUCSC | 258 | 6 | 34651 | tss;island | 0.420899 | 0.044851 | Not queried | 4.53 | 6.49e-02 | 1.11e-01 |
|  | 10 | 112431719 | 112431906 | PDFSTATSENSEMBLUCSC | 188 | 4 | 4755 | gene;island | 0.016831 | 0.281977 | Not queried | 4.53 | 5.71e-02 | 1.00e-01 |
|  | 3 | 44754201 | 44754587 | PDFSTATSENSEMBLUCSC | 387 | 4 | 27742 | gene | 0.372232 | 0.968781 | Not queried | 4.53 | 3.43e-01 | 3.84e-01 |
|  | 7 | 107302255 | 107302510 | PDFSTATSENSEMBLUCSC | 256 | 7 | 37183 | gene;tss;island | 0.434857 | 0.025667 | SLC26A4; SLC26A4-AS1 (margin: SLC26A4; SLC26A4-AS1) | 4.53 | 1.17e-03 | 1.13e-02 |
|  | 19 | 53662353 | 53662723 | PDFSTATSENSEMBLUCSC | 371 | 8 | 21024 | tss | 0.857039 | 0.261367 | ZNF347; ZNF665 (margin: CTD-2245F17.2; ZNF347; ZNF665) | 4.53 | 3.11e-04 | 4.77e-03 |
|  | 6 | 36237982 | 36238410 | PDFSTATSENSEMBLUCSC | 429 | 8 | 34313 | gene | 0.101206 | 0.588040 | PNPLA1 (margin: PNPLA1) | 4.53 | 1.04e-02 | 3.96e-02 |
|  | 17 | 79503642 | 79503877 | PDFSTATSENSEMBLUCSC | 236 | 4 | 16914 | gene;island | 0.728169 | 0.983447 | Not queried | 4.53 | 2.86e-02 | 6.12e-02 |
|  | 8 | 6662963 | 6663401 | PDFSTATSENSEMBLUCSC | 439 | 4 | 40052 | island | 0.483156 | 0.871685 | Not queried | 4.53 | 2.86e-02 | 6.12e-02 |
|  | 3 | 79816794 | 79817333 | PDFSTATSENSEMBLUCSC | 540 | 7 | 29294 | island | 0.373641 | 0.841716 | Not queried | 4.53 | 1.75e-02 | 5.79e-02 |
|  | 17 | 43662488 | 43662979 | PDFSTATSENSEMBLUCSC | 492 | 6 | 18441 | island | 0.033586 | 0.411225 | DND1P1 (margin: DND1P1) | 4.53 | 2.16e-03 | 1.45e-02 |
|  | 19 | 47217237 | 47217345 | PDFSTATSENSEMBLUCSC | 109 | 4 | 20111 | gene;island | 0.028743 | 0.347973 | Not queried | 4.53 | 2.86e-02 | 6.12e-02 |
|  | 11 | 44337982 | 44338421 | PDFSTATSENSEMBLUCSC | 440 | 5 | 8226 | island | 0.431226 | 0.935887 | (margin: ALX4) | 4.53 | 7.94e-03 | 3.19e-02 |
|  | 1 | 3331987 | 3332264 | PDFSTATSENSEMBLUCSC | 278 | 6 | 125 | gene;island | 0.285106 | 0.896736 | PRDM16 (margin: PRDM16) | 4.53 | 8.66e-03 | 3.36e-02 |
|  | 11 | 110582732 | 110583036 | PDFSTATSENSEMBLUCSC | 305 | 4 | 6895 | gene;island | 0.730077 | 0.985575 | Not queried | 4.52 | 2.86e-02 | 6.12e-02 |
|  | 17 | 36719518 | 36719937 | PDFSTATSENSEMBLUCSC | 420 | 5 | 16539 | gene;island | 0.590450 | 0.969167 | Not queried | 4.52 | 3.17e-02 | 6.50e-02 |
|  | 3 | 124303268 | 124303745 | PDFSTATSENSEMBLUCSC | 478 | 5 | 27923 | gene;island | 0.408721 | 0.020713 | Not queried | 4.52 | 5.56e-02 | 1.00e-01 |
|  | 17 | 56232260 | 56232674 | PDFSTATSENSEMBLUCSC | 415 | 5 | 18594 | island | 0.015145 | 0.684786 | Not queried | 4.52 | 5.56e-02 | 1.00e-01 |
|  | 16 | 71264282 | 71264678 | PDFSTATSENSEMBLUCSC | 397 | 7 | 16073 | island | 0.945165 | 0.420245 | Not queried | 4.52 | 7.28e-02 | 1.23e-01 |
|  | 1 | 3184434 | 3184704 | PDFSTATSENSEMBLUCSC | 271 | 4 | 115 | gene;island | 0.292969 | 0.839938 | Not queried | 4.52 | 2.86e-02 | 6.12e-02 |
|  | 12 | 1940380 | 1940618 | PDFSTATSENSEMBLUCSC | 239 | 4 | 9000 | gene | 0.376358 | 0.076177 | Not queried | 4.52 | 1.14e-01 | 1.68e-01 |
|  | 1 | 156878191 | 156878573 | PDFSTATSENSEMBLUCSC | 383 | 4 | 833 | gene;island | 0.351735 | 0.845889 | Not queried | 4.52 | 2.86e-02 | 6.12e-02 |
|  | 4 | 66536186 | 66536772 | PDFSTATSENSEMBLUCSC | 587 | 7 | 30417 | tss;island | 0.780437 | 0.240590 | RP11-807H7.1; EPHA5 (margin: RP11-807H7.1; EPHA5) | 4.52 | 2.33e-03 | 1.52e-02 |
|  | 15 | 73661340 | 73662002 | PDFSTATSENSEMBLUCSC | 663 | 8 | 14288 | island | 0.413589 | 0.940603 | HCN4 (margin: HCN4) | 4.52 | 6.99e-03 | 3.19e-02 |
|  | 3 | 112931126 | 112931354 | PDFSTATSENSEMBLUCSC | 229 | 6 | 28505 | tss | 0.410678 | 0.032573 | BOC (margin: BOC) | 4.51 | 8.66e-03 | 3.36e-02 |
|  | 7 | 127225750 | 127225949 | PDFSTATSENSEMBLUCSC | 200 | 9 | 37877 | tss | 0.464902 | 0.036817 | Not queried | 4.51 | 1.90e-01 | 2.55e-01 |
|  | 7 | 89841028 | 89841217 | PDFSTATSENSEMBLUCSC | 190 | 4 | 37081 | gene | 0.006022 | 0.273679 | Not queried | 4.51 | 5.71e-02 | 1.00e-01 |
|  | 2 | 223166909 | 223167617 | PDFSTATSENSEMBLUCSC | 709 | 8 | 23050 | gene;island | 0.421325 | 0.951898 | CCDC140 (margin: CCDC140; PAX3) | 4.51 | 1.55e-04 | 2.71e-03 |
|  | 14 | 95027382 | 95027692 | PDFSTATSENSEMBLUCSC | 311 | 4 | 12584 | tss | 0.285745 | 0.907007 | Not queried | 4.51 | 5.71e-02 | 1.00e-01 |
|  | 2 | 133428422 | 133429299 | PDFSTATSENSEMBLUCSC | 878 | 10 | 24746 | island | 0.010720 | 0.108930 | LYPD1; NCKAP5 (margin: AC010974.3; LYPD1; NCKAP5) | 4.51 | 8.93e-03 | 3.45e-02 |
|  | 8 | 109095918 | 109096151 | PDFSTATSENSEMBLUCSC | 234 | 7 | 39866 | tss | 0.913593 | 0.250237 | Not queried | 4.51 | 3.18e-01 | 3.74e-01 |
|  | 12 | 124246391 | 124246525 | PDFSTATSENSEMBLUCSC | 135 | 4 | 10107 | tss;island | 0.649431 | 0.980740 | Not queried | 4.51 | 2.86e-02 | 6.12e-02 |
|  | 14 | 101350645 | 101351197 | PDFSTATSENSEMBLUCSC | 553 | 8 | 12614 | tss;island | 0.476994 | 0.945492 | Not queried | 4.51 | 4.99e-02 | 9.81e-02 |
|  | 6 | 32977400 | 32977708 | PDFSTATSENSEMBLUCSC | 309 | 4 | 34886 | tss | 0.370340 | 0.681858 | Not queried | 4.51 | 3.43e-01 | 3.84e-01 |
|  | 17 | 73874443 | 73874637 | PDFSTATSENSEMBLUCSC | 195 | 5 | 16780 | gene | 0.014639 | 0.342671 | Not queried | 4.51 | 3.17e-02 | 6.50e-02 |
|  | 12 | 119419106 | 119419331 | PDFSTATSENSEMBLUCSC | 226 | 4 | 10049 | tss;island | 0.950489 | 0.567645 | Not queried | 4.51 | 2.86e-02 | 6.12e-02 |
|  | 10 | 131576889 | 131577071 | PDFSTATSENSEMBLUCSC | 183 | 4 | 6259 | island | 0.626014 | 0.974138 | Not queried | 4.51 | 2.86e-02 | 6.12e-02 |
|  | 15 | 60294120 | 60294517 | PDFSTATSENSEMBLUCSC | 398 | 4 | 14188 | island | 0.701707 | 0.116104 | Not queried | 4.51 | 1.14e-01 | 1.68e-01 |
|  | 11 | 94245224 | 94245795 | PDFSTATSENSEMBLUCSC | 572 | 6 | 8739 | island | 0.635122 | 0.981434 | Not queried | 4.51 | 1.32e-01 | 1.89e-01 |
|  | 22 | 32555255 | 32555310 | PDFSTATSENSEMBLUCSC | 56 | 4 | 27087 | tss | 0.572634 | 0.906152 | Not queried | 4.51 | 2.86e-02 | 6.12e-02 |
|  | 1 | 40974057 | 40974239 | PDFSTATSENSEMBLUCSC | 183 | 4 | 1538 | tss;island | 0.177156 | 0.016668 | Not queried | 4.50 | 2.86e-02 | 6.12e-02 |
|  | 3 | 134369339 | 134370241 | PDFSTATSENSEMBLUCSC | 903 | 13 | 29500 | island | 0.415460 | 0.899791 | EPHB1; KY (margin: EPHB1; KY) | 4.50 | 8.65e-06 | 4.82e-04 |
|  | 1 | 7122541 | 7122970 | PDFSTATSENSEMBLUCSC | 430 | 6 | 186 | gene;island | 0.329613 | 0.915039 | Not queried | 4.50 | 6.49e-02 | 1.11e-01 |
|  | 11 | 94134646 | 94135029 | PDFSTATSENSEMBLUCSC | 384 | 8 | 7629 | tss | 0.684159 | 0.984266 | GPR83 (margin: GPR83) | 4.50 | 4.66e-03 | 2.68e-02 |
|  | 16 | 20774873 | 20775166 | PDFSTATSENSEMBLUCSC | 294 | 4 | 15122 | tss | 0.047065 | 0.622537 | Not queried | 4.50 | 2.86e-02 | 6.12e-02 |
|  | 5 | 136834155 | 136834492 | PDFSTATSENSEMBLUCSC | 338 | 6 | 31762 | gene;island | 0.611957 | 0.980899 | Not queried | 4.50 | 1.52e-02 | 5.36e-02 |
|  | 19 | 37341052 | 37341261 | PDFSTATSENSEMBLUCSC | 210 | 7 | 20731 | tss | 0.436574 | 0.020782 | ZNF345; ZNF790 (margin: ZNF345; ZNF790; Y\_RNA) | 4.50 | 2.33e-03 | 1.52e-02 |
|  | 14 | 61789466 | 61789733 | PDFSTATSENSEMBLUCSC | 268 | 5 | 12107 | gene;island | 0.011036 | 0.200907 | PRKCH; RP11-902B17.1 (margin: PRKCH; RP11-902B17.1) | 4.50 | 7.94e-03 | 3.19e-02 |
|  | 2 | 7057153 | 7057402 | PDFSTATSENSEMBLUCSC | 250 | 6 | 23168 | tss;island | 0.823153 | 0.157483 | Not queried | 4.50 | 1.32e-01 | 1.89e-01 |
|  | 10 | 679171 | 679514 | PDFSTATSENSEMBLUCSC | 344 | 5 | 4447 | gene;island | 0.835681 | 0.986000 | Not queried | 4.50 | 1.51e-01 | 2.11e-01 |
|  | 18 | 21242319 | 21242479 | PDFSTATSENSEMBLUCSC | 161 | 4 | 19074 | gene;island | 0.003378 | 0.083194 | Not queried | 4.50 | 5.71e-02 | 1.00e-01 |
|  | 1 | 1713944 | 1714271 | PDFSTATSENSEMBLUCSC | 328 | 5 | 2462 | island | 0.638812 | 0.985566 | (margin: NADK; GNB1) | 4.50 | 7.94e-03 | 3.19e-02 |
|  | 14 | 106321751 | 106321936 | PDFSTATSENSEMBLUCSC | 186 | 4 | 13246 | island | 0.214889 | 0.816298 | Not queried | 4.50 | 1.14e-01 | 1.68e-01 |
|  | 19 | 847943 | 848071 | PDFSTATSENSEMBLUCSC | 129 | 4 | 19633 | gene;island | 0.346939 | 0.872589 | Not queried | 4.49 | 5.71e-02 | 1.00e-01 |
|  | 19 | 51523550 | 51523933 | PDFSTATSENSEMBLUCSC | 384 | 5 | 20990 | tss;island | 0.057419 | 0.814084 | Not queried | 4.49 | 5.56e-02 | 1.00e-01 |
|  | 13 | 113663206 | 113663538 | PDFSTATSENSEMBLUCSC | 333 | 4 | 11268 | gene;island | 0.711928 | 0.980425 | Not queried | 4.49 | 5.71e-02 | 1.00e-01 |
|  | 19 | 13002357 | 13002535 | PDFSTATSENSEMBLUCSC | 179 | 4 | 19873 | gene;island | 0.350799 | 0.025903 | Not queried | 4.49 | 2.86e-02 | 6.12e-02 |
|  | 10 | 14050305 | 14050521 | PDFSTATSENSEMBLUCSC | 217 | 4 | 4507 | gene | 0.157680 | 0.010622 | Not queried | 4.49 | 2.86e-02 | 6.12e-02 |
|  | 5 | 167956306 | 167956581 | PDFSTATSENSEMBLUCSC | 276 | 5 | 33372 | island | 0.743405 | 0.980970 | Not queried | 4.49 | 3.17e-02 | 6.50e-02 |
|  | 1 | 3511964 | 3512243 | PDFSTATSENSEMBLUCSC | 280 | 4 | 131 | gene;island | 0.440297 | 0.945414 | Not queried | 4.48 | 2.86e-02 | 6.12e-02 |
|  | 2 | 242794854 | 242795082 | PDFSTATSENSEMBLUCSC | 229 | 5 | 23148 | gene;island | 0.691629 | 0.980015 | PDCD1 (margin: AC114730.2; PDCD1) | 4.48 | 7.94e-03 | 3.19e-02 |
|  | 3 | 183959000 | 183959171 | PDFSTATSENSEMBLUCSC | 172 | 7 | 28057 | gene;tss;island | 0.516725 | 0.925824 | EIF2B5; VWA5B2; MIR1224; ALG3 (margin: EIF2B5; VWA5B2; MIR1224; ECE2; ALG3) | 4.48 | 5.83e-04 | 6.47e-03 |
|  | 2 | 37571677 | 37571732 | PDFSTATSENSEMBLUCSC | 56 | 5 | 23306 | tss | 0.978153 | 0.662337 | QPCT (margin: QPCT) | 4.48 | 7.94e-03 | 3.19e-02 |
|  | 4 | 83483204 | 83483487 | PDFSTATSENSEMBLUCSC | 284 | 6 | 30467 | tss;island | 0.323656 | 0.016376 | Not queried | 4.48 | 2.60e-02 | 6.12e-02 |
|  | 4 | 77172617 | 77173197 | PDFSTATSENSEMBLUCSC | 581 | 10 | 31115 | island | 0.112784 | 0.005193 | FAM47E; FAM47E-STBD1 (margin: FAM47E; FAM47E-STBD1) | 4.48 | 8.93e-03 | 3.45e-02 |
|  | 5 | 142066121 | 142066398 | PDFSTATSENSEMBLUCSC | 278 | 4 | 31844 | gene;tss | 0.388822 | 0.851941 | Not queried | 4.48 | 2.86e-02 | 6.12e-02 |
|  | 2 | 119600348 | 119600627 | PDFSTATSENSEMBLUCSC | 280 | 5 | 22840 | gene;island | 0.621834 | 0.973412 | EN1 (margin: RP11-19E11.1; EN1) | 4.48 | 7.94e-03 | 3.19e-02 |
|  | 7 | 1198522 | 1198977 | PDFSTATSENSEMBLUCSC | 456 | 5 | 36752 | gene;island | 0.019098 | 0.172901 | Not queried | 4.48 | 3.17e-02 | 6.50e-02 |
|  | 3 | 100120235 | 100120961 | PDFSTATSENSEMBLUCSC | 727 | 11 | 27878 | gene;tss;island | 0.111505 | 0.010183 | LNP1; TOMM70A (margin: LNP1; TOMM70A) | 4.48 | 1.86e-03 | 1.45e-02 |
|  | 19 | 39522418 | 39523158 | PDFSTATSENSEMBLUCSC | 741 | 6 | 20024 | gene | 0.070641 | 0.006586 | Not queried | 4.48 | 2.60e-02 | 6.12e-02 |
|  | 7 | 4242769 | 4243019 | PDFSTATSENSEMBLUCSC | 251 | 4 | 36833 | gene | 0.205801 | 0.826199 | Not queried | 4.48 | 2.86e-02 | 6.12e-02 |
|  | 7 | 29603121 | 29603407 | PDFSTATSENSEMBLUCSC | 287 | 7 | 37512 | tss | 0.116620 | 0.010820 | PRR15; AC007255.8 (margin: RP11-550A18.1; PRR15; AC007255.8) | 4.48 | 6.99e-03 | 3.19e-02 |
|  | 4 | 54424280 | 54424787 | PDFSTATSENSEMBLUCSC | 508 | 5 | 30003 | gene | 0.841109 | 0.968631 | Not queried | 4.48 | 2.22e-01 | 2.76e-01 |
|  | 4 | 185942529 | 185942800 | PDFSTATSENSEMBLUCSC | 272 | 4 | 31470 | island | 0.498177 | 0.955324 | Not queried | 4.48 | 2.86e-02 | 6.12e-02 |
|  | 6 | 33160067 | 33160604 | PDFSTATSENSEMBLUCSC | 538 | 15 | 35969 | island | 0.471990 | 0.934988 | COL11A2; RXRB (margin: RNY4P10; SLC39A7; HSD17B8; COL11A2; RXRB) | 4.47 | 4.40e-05 | 1.23e-03 |
|  | 3 | 145879430 | 145879710 | PDFSTATSENSEMBLUCSC | 281 | 6 | 28647 | tss | 0.048617 | 0.541253 | PLOD2 (margin: PLOD2) | 4.47 | 2.16e-03 | 1.45e-02 |
|  | 17 | 146404 | 146926 | PDFSTATSENSEMBLUCSC | 523 | 5 | 16286 | gene | 0.426700 | 0.814951 | Not queried | 4.47 | 1.59e-02 | 5.36e-02 |
|  | 3 | 155394109 | 155394428 | PDFSTATSENSEMBLUCSC | 320 | 5 | 28004 | gene;tss | 0.530451 | 0.966149 | PLCH1 (margin: PLCH1) | 4.47 | 7.94e-03 | 3.19e-02 |
|  | 12 | 298151 | 298484 | PDFSTATSENSEMBLUCSC | 334 | 4 | 10141 | island | 0.302285 | 0.819298 | Not queried | 4.47 | 3.43e-01 | 3.84e-01 |
|  | 4 | 102712010 | 102712397 | PDFSTATSENSEMBLUCSC | 388 | 4 | 30072 | gene;island | 0.404811 | 0.977090 | Not queried | 4.47 | 5.71e-02 | 1.00e-01 |
|  | 7 | 149569715 | 149570040 | PDFSTATSENSEMBLUCSC | 326 | 8 | 37972 | tss | 0.529566 | 0.029837 | Not queried | 4.47 | 4.99e-02 | 9.81e-02 |
|  | 21 | 46131601 | 46131923 | PDFSTATSENSEMBLUCSC | 323 | 5 | 26587 | tss | 0.242882 | 0.014604 | Not queried | 4.47 | 3.17e-02 | 6.50e-02 |
|  | 12 | 53688851 | 53689090 | PDFSTATSENSEMBLUCSC | 240 | 6 | 9737 | tss;island | 0.012457 | 0.201725 | Not queried | 4.47 | 1.32e-01 | 1.89e-01 |
|  | 10 | 89263900 | 89264128 | PDFSTATSENSEMBLUCSC | 229 | 6 | 5202 | tss;island | 0.297712 | 0.784613 | Not queried | 4.47 | 2.60e-02 | 6.12e-02 |
|  | 19 | 18496756 | 18497201 | PDFSTATSENSEMBLUCSC | 446 | 8 | 21778 | island | 0.017220 | 0.190306 | GDF15; MIR3189 (margin: GDF15; MIR3189; LRRC25) | 4.47 | 1.86e-03 | 1.45e-02 |
|  | 1 | 226737766 | 226737975 | PDFSTATSENSEMBLUCSC | 210 | 4 | 1035 | gene;island | 0.438298 | 0.036343 | Not queried | 4.47 | 2.86e-02 | 6.12e-02 |
|  | 6 | 30882641 | 30882708 | PDFSTATSENSEMBLUCSC | 68 | 4 | 33837 | gene;island | 0.869775 | 0.377999 | Not queried | 4.46 | 2.86e-02 | 6.12e-02 |
|  | 17 | 71640181 | 71640369 | PDFSTATSENSEMBLUCSC | 189 | 7 | 18706 | island | 0.408269 | 0.024002 | RP11-277J6.2; SDK2 (margin: RP11-277J6.2; SDK2) | 4.46 | 4.08e-03 | 2.47e-02 |
|  | 4 | 128544255 | 128544667 | PDFSTATSENSEMBLUCSC | 413 | 4 | 31297 | island | 0.438344 | 0.881929 | Not queried | 4.46 | 2.86e-02 | 6.12e-02 |
|  | 17 | 75789279 | 75789529 | PDFSTATSENSEMBLUCSC | 251 | 5 | 18812 | island | 0.599789 | 0.973031 | Not queried | 4.46 | 1.59e-02 | 5.36e-02 |
|  | 11 | 118214762 | 118214927 | PDFSTATSENSEMBLUCSC | 166 | 4 | 7730 | tss | 0.159544 | 0.879647 | Not queried | 4.46 | 3.43e-01 | 3.84e-01 |
|  | 3 | 145968692 | 145968726 | PDFSTATSENSEMBLUCSC | 35 | 5 | 28648 | tss | 0.147861 | 0.009305 | Not queried | 4.46 | 3.17e-02 | 6.50e-02 |
|  | 2 | 240650018 | 240650458 | PDFSTATSENSEMBLUCSC | 441 | 5 | 25262 | island | 0.336230 | 0.943229 | Not queried | 4.46 | 1.59e-02 | 5.36e-02 |
|  | 1 | 39269995 | 39270310 | PDFSTATSENSEMBLUCSC | 316 | 4 | 3090 | island | 0.512908 | 0.961731 | Not queried | 4.46 | 2.86e-02 | 6.12e-02 |
|  | 7 | 27213893 | 27214383 | PDFSTATSENSEMBLUCSC | 491 | 10 | 36939 | gene;island | 0.870379 | 0.987948 | RP1-170O19.20; HOXA10 (margin: HOXA-AS4; HOXA11-AS; HOXA9; RP1-170O19.20; MIR196B; HOXA10; HOXA11; RP1-170O19.14) | 4.46 | 4.33e-05 | 1.22e-03 |
|  | 20 | 982991 | 983130 | PDFSTATSENSEMBLUCSC | 140 | 4 | 25531 | tss | 0.760116 | 0.976263 | Not queried | 4.46 | 2.86e-02 | 6.12e-02 |
|  | 8 | 39695788 | 39696062 | PDFSTATSENSEMBLUCSC | 275 | 6 | 39665 | tss | 0.646368 | 0.964748 | ADAM2 (margin: ADAM2) | 4.46 | 2.16e-03 | 1.45e-02 |
|  | 10 | 98945025 | 98946238 | PDFSTATSENSEMBLUCSC | 1214 | 15 | 5977 | island | 0.618468 | 0.051881 | Not queried | 4.45 | 4.53e-02 | 8.95e-02 |
|  | 4 | 3748154 | 3748347 | PDFSTATSENSEMBLUCSC | 194 | 4 | 30853 | island | 0.255362 | 0.876511 | Not queried | 4.45 | 2.86e-02 | 6.12e-02 |
|  | 1 | 79472532 | 79472643 | PDFSTATSENSEMBLUCSC | 112 | 4 | 1711 | tss | 0.523970 | 0.956460 | Not queried | 4.45 | 2.86e-02 | 6.12e-02 |
|  | 17 | 47308135 | 47308460 | PDFSTATSENSEMBLUCSC | 326 | 6 | 17484 | tss | 0.222319 | 0.009954 | Not queried | 4.45 | 1.52e-02 | 5.36e-02 |
|  | 7 | 108095719 | 108096041 | PDFSTATSENSEMBLUCSC | 323 | 5 | 37185 | gene;island | 0.275370 | 0.035546 | NRCAM (margin: NRCAM) | 4.45 | 7.94e-03 | 3.19e-02 |
|  | 4 | 183064851 | 183065224 | PDFSTATSENSEMBLUCSC | 374 | 4 | 30191 | gene;island | 0.139344 | 0.006765 | Not queried | 4.45 | 2.86e-02 | 6.12e-02 |
|  | 16 | 963814 | 963994 | PDFSTATSENSEMBLUCSC | 181 | 4 | 14552 | gene;island | 0.637827 | 0.979930 | Not queried | 4.45 | 2.86e-02 | 6.12e-02 |
|  | 6 | 39282316 | 39282532 | PDFSTATSENSEMBLUCSC | 217 | 4 | 34955 | tss | 0.548439 | 0.960671 | Not queried | 4.45 | 5.71e-02 | 1.00e-01 |
|  | 6 | 28493019 | 28493664 | PDFSTATSENSEMBLUCSC | 646 | 9 | 34743 | tss | 0.405613 | 0.730491 | Not queried | 4.45 | 1.42e-02 | 5.24e-02 |
|  | 1 | 248020331 | 248020436 | PDFSTATSENSEMBLUCSC | 106 | 4 | 2371 | tss | 0.333250 | 0.896031 | Not queried | 4.45 | 2.86e-02 | 6.12e-02 |
|  | 16 | 8806359 | 8806756 | PDFSTATSENSEMBLUCSC | 398 | 8 | 14652 | gene;tss | 0.358623 | 0.981840 | ABAT (margin: ABAT; RNU7-63P) | 4.45 | 1.09e-03 | 1.10e-02 |
|  | 15 | 23810163 | 23810378 | PDFSTATSENSEMBLUCSC | 216 | 5 | 13515 | tss | 0.332980 | 0.979437 | Not queried | 4.45 | 5.56e-02 | 1.00e-01 |
|  | 4 | 122854330 | 122854532 | PDFSTATSENSEMBLUCSC | 203 | 4 | 30577 | tss | 0.702410 | 0.981041 | Not queried | 4.44 | 5.71e-02 | 1.00e-01 |
|  | 1 | 156338639 | 156339286 | PDFSTATSENSEMBLUCSC | 648 | 8 | 3853 | island | 0.690837 | 0.110200 | RHBG; CCT3 (margin: RHBG; CCT3) | 4.44 | 1.55e-04 | 2.71e-03 |
|  | 17 | 30814514 | 30814814 | PDFSTATSENSEMBLUCSC | 301 | 5 | 16513 | gene;island | 0.416129 | 0.020876 | Not queried | 4.44 | 5.56e-02 | 1.00e-01 |
|  | 15 | 45406327 | 45406939 | PDFSTATSENSEMBLUCSC | 613 | 13 | 13645 | tss;island | 0.204804 | 0.848229 | DUOXA2; DUOX2 (margin: DUOXA2; DUOX2; DUOXA1) | 4.44 | 2.31e-06 | 1.96e-04 |
|  | 12 | 114843188 | 114843955 | PDFSTATSENSEMBLUCSC | 768 | 10 | 9326 | gene;island | 0.365114 | 0.850980 | TBX5 (margin: TBX5-AS1; TBX5) | 4.44 | 4.87e-04 | 6.47e-03 |
|  | 2 | 16081824 | 16082215 | PDFSTATSENSEMBLUCSC | 392 | 5 | 22582 | gene;island | 0.603122 | 0.979087 | Not queried | 4.44 | 1.59e-02 | 5.36e-02 |
|  | 14 | 101350645 | 101351026 | PDFSTATSENSEMBLUCSC | 382 | 7 | 12214 | gene | 0.507873 | 0.957217 | Not queried | 4.44 | 9.73e-02 | 1.55e-01 |
|  | 16 | 54965084 | 54965100 | PDFSTATSENSEMBLUCSC | 17 | 4 | 15238 | tss | 0.130707 | 0.007306 | Not queried | 4.44 | 2.86e-02 | 6.12e-02 |
|  | 2 | 71205052 | 71205564 | PDFSTATSENSEMBLUCSC | 513 | 6 | 23424 | tss | 0.228566 | 0.886898 | ANKRD53; AC007040.11 (margin: ANKRD53; AC007040.11; TEX261) | 4.44 | 4.33e-03 | 2.52e-02 |
|  | 19 | 12624466 | 12624832 | PDFSTATSENSEMBLUCSC | 367 | 8 | 21604 | island | 0.579203 | 0.981001 | ZNF709 (margin: CTD-3105H18.8; CTD-3105H18.10; CTD-3105H18.9; CTD-3105H18.11; PPIAP20; ZNF709; ZNF564; CTD-2192J16.20) | 4.44 | 4.66e-03 | 2.68e-02 |
|  | 10 | 100995599 | 100995821 | PDFSTATSENSEMBLUCSC | 223 | 4 | 6005 | island | 0.184166 | 0.800168 | Not queried | 4.44 | 2.86e-02 | 6.12e-02 |
|  | 10 | 133953871 | 133954211 | PDFSTATSENSEMBLUCSC | 341 | 4 | 4854 | gene;island | 0.514576 | 0.969288 | Not queried | 4.44 | 2.86e-02 | 6.12e-02 |
|  | 16 | 1480790 | 1480992 | PDFSTATSENSEMBLUCSC | 203 | 4 | 15542 | island | 0.527537 | 0.033896 | Not queried | 4.44 | 1.14e-01 | 1.68e-01 |
|  | 15 | 65203834 | 65204034 | PDFSTATSENSEMBLUCSC | 201 | 5 | 13735 | tss | 0.387776 | 0.020814 | AC069368.3; ANKDD1A (margin: AC069368.3; ANKDD1A; RN7SL348P) | 4.43 | 7.94e-03 | 3.19e-02 |
|  | 8 | 70745466 | 70745926 | PDFSTATSENSEMBLUCSC | 461 | 6 | 39754 | tss | 0.723531 | 0.149375 | RP11-159H10.3; SLCO5A1 (margin: RP11-159H10.3; SLCO5A1) | 4.43 | 8.66e-03 | 3.36e-02 |
|  | 10 | 132910868 | 132911152 | PDFSTATSENSEMBLUCSC | 285 | 4 | 4841 | gene | 0.440281 | 0.917059 | Not queried | 4.43 | 1.14e-01 | 1.68e-01 |
|  | 17 | 6679254 | 6679781 | PDFSTATSENSEMBLUCSC | 528 | 12 | 17910 | island | 0.614425 | 0.976909 | XAF1; FBXO39 (margin: XAF1; FBXO39) | 4.43 | 2.22e-05 | 9.05e-04 |
|  | 10 | 115938729 | 115939284 | PDFSTATSENSEMBLUCSC | 556 | 8 | 6151 | island | 0.959261 | 0.583104 | TDRD1 (margin: TDRD1; C10orf118; MIR2110) | 4.43 | 6.22e-04 | 6.71e-03 |
|  | 6 | 117197952 | 117198281 | PDFSTATSENSEMBLUCSC | 330 | 8 | 35194 | tss | 0.394681 | 0.064825 | RFX6 (margin: RFX6) | 4.43 | 1.04e-02 | 3.96e-02 |
|  | 11 | 14913124 | 14914012 | PDFSTATSENSEMBLUCSC | 889 | 13 | 8080 | island | 0.192657 | 0.007621 | CYP2R1 (margin: CYP2R1) | 4.43 | 2.31e-06 | 1.96e-04 |
|  | 3 | 50275394 | 50275694 | PDFSTATSENSEMBLUCSC | 301 | 5 | 27796 | gene;island | 0.008213 | 0.198314 | GNAI2; U73169.1 (margin: GNAI2; U73169.1) | 4.43 | 7.94e-03 | 3.19e-02 |
|  | 7 | 149569715 | 149570367 | PDFSTATSENSEMBLUCSC | 653 | 13 | 37268 | gene;island | 0.508493 | 0.040794 | ATP6V0E2; ATP6V0E2-AS1 (margin: ZNF862; ATP6V0E2; ATP6V0E2-AS1) | 4.43 | 7.95e-04 | 8.28e-03 |
|  | 2 | 50201382 | 50201511 | PDFSTATSENSEMBLUCSC | 130 | 5 | 22675 | gene | 0.610327 | 0.049076 | NRXN1 (margin: NRXN1) | 4.42 | 7.94e-03 | 3.19e-02 |
|  | 17 | 73084263 | 73084494 | PDFSTATSENSEMBLUCSC | 232 | 4 | 16764 | gene;island | 0.025297 | 0.334169 | Not queried | 4.42 | 2.86e-02 | 6.12e-02 |
|  | 1 | 220102277 | 220102517 | PDFSTATSENSEMBLUCSC | 241 | 5 | 2258 | tss;island | 0.629388 | 0.976514 | Not queried | 4.42 | 1.59e-02 | 5.36e-02 |
|  | 6 | 168920172 | 168920331 | PDFSTATSENSEMBLUCSC | 160 | 4 | 34561 | gene;island | 0.709707 | 0.974806 | Not queried | 4.42 | 2.86e-02 | 6.12e-02 |
|  | 12 | 113909932 | 113910034 | PDFSTATSENSEMBLUCSC | 103 | 6 | 10030 | tss;island | 0.543125 | 0.936798 | Not queried | 4.42 | 9.31e-02 | 1.53e-01 |
|  | 5 | 8457548 | 8458089 | PDFSTATSENSEMBLUCSC | 542 | 6 | 32684 | island | 0.043443 | 0.348590 | MIR4458; RP11-480D4.1; RP11-480D4.2; RP11-480D4.4 (margin: MIR4458; RP11-480D4.1; RP11-480D4.2; RP11-480D4.6; RP11-480D4.4) | 4.42 | 2.16e-03 | 1.45e-02 |
|  | 14 | 24808987 | 24809759 | PDFSTATSENSEMBLUCSC | 773 | 8 | 12747 | island | 0.557549 | 0.957387 | RIPK3 (margin: ADCY4; RP11-934B9.3; RIPK3; RNU6-802P) | 4.42 | 1.04e-02 | 3.96e-02 |
|  | 2 | 225266733 | 225266880 | PDFSTATSENSEMBLUCSC | 148 | 6 | 23925 | tss | 0.764165 | 0.980740 | Not queried | 4.42 | 4.11e-02 | 8.14e-02 |
|  | 3 | 128151940 | 128152268 | PDFSTATSENSEMBLUCSC | 329 | 4 | 29453 | island | 0.623073 | 0.971788 | Not queried | 4.42 | 2.86e-02 | 6.12e-02 |
|  | 2 | 233320486 | 233320796 | PDFSTATSENSEMBLUCSC | 311 | 4 | 23949 | tss | 0.286994 | 0.892318 | Not queried | 4.42 | 2.86e-02 | 6.12e-02 |
|  | 1 | 201708500 | 201708932 | PDFSTATSENSEMBLUCSC | 433 | 8 | 941 | gene;tss;island | 0.034164 | 0.417663 | Not queried | 4.42 | 1.05e-01 | 1.66e-01 |
|  | 10 | 7453329 | 7453594 | PDFSTATSENSEMBLUCSC | 266 | 7 | 5533 | island | 0.527821 | 0.047732 | SFMBT2 (margin: SFMBT2) | 4.42 | 1.11e-02 | 4.14e-02 |
|  | 5 | 32710456 | 32710882 | PDFSTATSENSEMBLUCSC | 427 | 6 | 32008 | tss;island | 0.390577 | 0.017690 | Not queried | 4.42 | 1.52e-02 | 5.36e-02 |
|  | 2 | 56150255 | 56150675 | PDFSTATSENSEMBLUCSC | 421 | 6 | 22691 | gene;island | 0.046656 | 0.542962 | Not queried | 4.42 | 2.60e-02 | 6.12e-02 |
|  | 10 | 95326033 | 95326409 | PDFSTATSENSEMBLUCSC | 377 | 9 | 5238 | tss;island | 0.473957 | 0.964960 | FFAR4 (margin: FFAR4) | 4.42 | 4.11e-05 | 1.17e-03 |
|  | 3 | 194406190 | 194406572 | PDFSTATSENSEMBLUCSC | 383 | 9 | 28802 | tss;island | 0.422638 | 0.054896 | FAM43A (margin: FAM43A) | 4.41 | 1.06e-02 | 4.01e-02 |
|  | 3 | 180319597 | 180320159 | PDFSTATSENSEMBLUCSC | 563 | 9 | 29684 | island | 0.025245 | 0.331409 | TTC14; RP11-496B10.3; CCDC39 (margin: TTC14; RP11-496B10.3; RP11-496B10.1; CCDC39) | 4.41 | 4.94e-04 | 6.47e-03 |
|  | 1 | 206680459 | 206680868 | PDFSTATSENSEMBLUCSC | 410 | 5 | 2212 | tss | 0.695627 | 0.966629 | Not queried | 4.41 | 2.22e-01 | 2.76e-01 |
|  | 16 | 82044738 | 82045222 | PDFSTATSENSEMBLUCSC | 485 | 9 | 16127 | island | 0.083773 | 0.619030 | Not queried | 4.41 | 2.58e-01 | 3.16e-01 |
|  | 20 | 50158996 | 50159653 | PDFSTATSENSEMBLUCSC | 658 | 6 | 25454 | gene;island | 0.817214 | 0.192707 | NFATC2 (margin: NFATC2) | 4.41 | 4.33e-03 | 2.52e-02 |
|  | 3 | 180319597 | 180319907 | PDFSTATSENSEMBLUCSC | 311 | 6 | 28735 | tss | 0.019709 | 0.309118 | Not queried | 4.41 | 1.52e-02 | 5.36e-02 |
|  | 11 | 119227592 | 119227876 | PDFSTATSENSEMBLUCSC | 285 | 4 | 6939 | gene;island | 0.213471 | 0.027695 | Not queried | 4.41 | 3.43e-01 | 3.84e-01 |
|  | 15 | 92397488 | 92397873 | PDFSTATSENSEMBLUCSC | 386 | 4 | 13485 | gene;island | 0.017244 | 0.162467 | Not queried | 4.41 | 3.43e-01 | 3.84e-01 |
|  | 7 | 103630013 | 103630093 | PDFSTATSENSEMBLUCSC | 81 | 4 | 37813 | tss;island | 0.747781 | 0.972492 | Not queried | 4.41 | 2.86e-02 | 6.12e-02 |
|  | 18 | 30352782 | 30353283 | PDFSTATSENSEMBLUCSC | 502 | 8 | 19454 | island | 0.067101 | 0.733068 | Not queried | 4.41 | 1.05e-01 | 1.66e-01 |
|  | 3 | 32859377 | 32859445 | PDFSTATSENSEMBLUCSC | 69 | 5 | 28208 | tss | 0.752612 | 0.984359 | TRIM71 (margin: TRIM71) | 4.40 | 7.94e-03 | 3.19e-02 |
|  | 6 | 32712930 | 32713188 | PDFSTATSENSEMBLUCSC | 259 | 5 | 34130 | gene | 0.099475 | 0.748634 | Not queried | 4.40 | 5.56e-02 | 1.00e-01 |
|  | 16 | 12183835 | 12184133 | PDFSTATSENSEMBLUCSC | 299 | 5 | 14669 | gene | 0.144244 | 0.879680 | Not queried | 4.40 | 2.22e-01 | 2.76e-01 |
|  | 6 | 28889357 | 28890069 | PDFSTATSENSEMBLUCSC | 713 | 9 | 33645 | gene;island | 0.205698 | 0.867240 | Not queried | 4.40 | 2.97e-01 | 3.60e-01 |
|  | 6 | 112575705 | 112576119 | PDFSTATSENSEMBLUCSC | 415 | 6 | 36423 | island | 0.482311 | 0.958969 | RP11-506B6.6; LAMA4 (margin: RP11-506B6.6; LAMA4) | 4.40 | 2.16e-03 | 1.45e-02 |
|  | 1 | 201476290 | 201476960 | PDFSTATSENSEMBLUCSC | 671 | 11 | 4077 | island | 0.019877 | 0.433694 | RP11-134G8.7; CSRP1 (margin: RP11-134G8.7; RPS10P7; CSRP1) | 4.40 | 1.04e-02 | 3.96e-02 |
|  | 10 | 102508286 | 102508633 | PDFSTATSENSEMBLUCSC | 348 | 5 | 4715 | gene;island | 0.339265 | 0.750985 | Not queried | 4.40 | 5.56e-02 | 1.00e-01 |
|  | 19 | 10397429 | 10397642 | PDFSTATSENSEMBLUCSC | 214 | 5 | 20443 | tss | 0.314858 | 0.931560 | ICAM1; ICAM4; CTD-2369P2.5; CTD-2369P2.8 (margin: ICAM1; ICAM4; ICAM5; CTD-2369P2.5; CTD-2369P2.8) | 4.40 | 7.94e-03 | 3.19e-02 |
|  | 17 | 72355995 | 72356254 | PDFSTATSENSEMBLUCSC | 260 | 4 | 16754 | gene;island | 0.462612 | 0.894880 | Not queried | 4.40 | 1.14e-01 | 1.68e-01 |
|  | 7 | 95225937 | 95226433 | PDFSTATSENSEMBLUCSC | 497 | 9 | 37730 | tss;island | 0.600613 | 0.063038 | Not queried | 4.40 | 7.70e-02 | 1.30e-01 |
|  | 10 | 31609882 | 31609960 | PDFSTATSENSEMBLUCSC | 79 | 6 | 5018 | tss | 0.082845 | 0.658260 | ZEB1; ZEB1-AS1 (margin: ZEB1; ZEB1-AS1) | 4.40 | 2.16e-03 | 1.45e-02 |
|  | 17 | 75446431 | 75446661 | PDFSTATSENSEMBLUCSC | 231 | 6 | 16808 | gene;island | 0.156153 | 0.801430 | SEPT9 (margin: SEPT9; Y\_RNA) | 4.40 | 2.16e-03 | 1.45e-02 |
|  | 12 | 52679967 | 52680245 | PDFSTATSENSEMBLUCSC | 279 | 4 | 9136 | gene | 0.591652 | 0.980627 | Not queried | 4.40 | 2.86e-02 | 6.12e-02 |
|  | 9 | 139948856 | 139949108 | PDFSTATSENSEMBLUCSC | 253 | 4 | 40857 | tss;island | 0.608594 | 0.144442 | Not queried | 4.40 | 2.86e-02 | 6.12e-02 |
|  | 6 | 32629786 | 32629955 | PDFSTATSENSEMBLUCSC | 170 | 5 | 34124 | gene;island | 0.179138 | 0.715520 | Not queried | 4.40 | 3.17e-02 | 6.50e-02 |
|  | 8 | 99076526 | 99076734 | PDFSTATSENSEMBLUCSC | 209 | 4 | 39834 | tss | 0.635310 | 0.965373 | Not queried | 4.40 | 2.86e-02 | 6.12e-02 |
|  | 1 | 40781617 | 40781907 | PDFSTATSENSEMBLUCSC | 291 | 4 | 454 | gene;island | 0.333828 | 0.889838 | Not queried | 4.40 | 2.86e-02 | 6.12e-02 |
|  | 19 | 18902117 | 18902221 | PDFSTATSENSEMBLUCSC | 105 | 4 | 20628 | tss | 0.431231 | 0.892918 | Not queried | 4.40 | 1.14e-01 | 1.68e-01 |
|  | 1 | 24645885 | 24646392 | PDFSTATSENSEMBLUCSC | 508 | 9 | 326 | gene | 0.106201 | 0.007145 | Not queried | 4.39 | 1.42e-02 | 5.24e-02 |
|  | 7 | 156796967 | 156797410 | PDFSTATSENSEMBLUCSC | 444 | 6 | 39091 | island | 0.755968 | 0.992195 | RP5-1121A15.4; MNX1 (margin: RP5-1121A15.4; RP5-1121A15.1; MNX1) | 4.39 | 8.66e-03 | 3.36e-02 |
|  | 1 | 42846740 | 42847055 | PDFSTATSENSEMBLUCSC | 316 | 4 | 464 | gene;island | 0.662887 | 0.073148 | Not queried | 4.39 | 5.71e-02 | 1.00e-01 |
|  | 12 | 40013961 | 40014292 | PDFSTATSENSEMBLUCSC | 332 | 4 | 9637 | tss | 0.229740 | 0.006753 | Not queried | 4.39 | 3.43e-01 | 3.84e-01 |
|  | 11 | 134145889 | 134146266 | PDFSTATSENSEMBLUCSC | 378 | 8 | 7832 | tss | 0.501605 | 0.974617 | GLB1L3 (margin: ACAD8; GLB1L3) | 4.39 | 1.09e-03 | 1.10e-02 |
|  | 4 | 13538730 | 13539255 | PDFSTATSENSEMBLUCSC | 526 | 7 | 30932 | island | 0.272122 | 0.881918 | Not queried | 4.39 | 5.30e-02 | 1.00e-01 |
|  | 17 | 63133186 | 63133259 | PDFSTATSENSEMBLUCSC | 74 | 4 | 17592 | tss;island | 0.292091 | 0.018093 | Not queried | 4.39 | 1.14e-01 | 1.68e-01 |
|  | 6 | 32407689 | 32407838 | PDFSTATSENSEMBLUCSC | 150 | 4 | 34112 | gene | 0.009792 | 0.516678 | Not queried | 4.39 | 1.14e-01 | 1.68e-01 |
|  | 16 | 54965084 | 54965142 | PDFSTATSENSEMBLUCSC | 59 | 5 | 15931 | island | 0.141191 | 0.007540 | IRX5; CRNDE (margin: IRX5; CTD-3032H12.2; CRNDE; CTD-3032H12.1) | 4.39 | 7.94e-03 | 3.19e-02 |
|  | 6 | 32725075 | 32725438 | PDFSTATSENSEMBLUCSC | 364 | 5 | 34131 | gene | 0.123478 | 0.762803 | Not queried | 4.39 | 1.59e-02 | 5.36e-02 |
|  | 11 | 1104295 | 1104527 | PDFSTATSENSEMBLUCSC | 233 | 4 | 7903 | island | 0.793384 | 0.985827 | Not queried | 4.39 | 2.86e-02 | 6.12e-02 |
|  | 1 | 33219580 | 33219687 | PDFSTATSENSEMBLUCSC | 108 | 4 | 398 | gene;island | 0.267839 | 0.016501 | Not queried | 4.39 | 2.86e-02 | 6.12e-02 |
|  | 20 | 35972924 | 35973098 | PDFSTATSENSEMBLUCSC | 175 | 5 | 25726 | tss;island | 0.154581 | 0.756332 | SRC (margin: SRC) | 4.39 | 7.94e-03 | 3.19e-02 |
|  | 10 | 88126089 | 88126306 | PDFSTATSENSEMBLUCSC | 218 | 8 | 5902 | island | 0.562582 | 0.054792 | GRID1 (margin: GRID1) | 4.39 | 1.55e-04 | 2.71e-03 |
|  | 17 | 75446431 | 75446592 | PDFSTATSENSEMBLUCSC | 162 | 5 | 17698 | tss | 0.208314 | 0.846268 | SEPT9 (margin: SEPT9; Y\_RNA) | 4.39 | 7.94e-03 | 3.19e-02 |
|  | 19 | 6502101 | 6502557 | PDFSTATSENSEMBLUCSC | 457 | 7 | 21433 | island | 0.640466 | 0.018679 | Not queried | 4.39 | 5.30e-02 | 1.00e-01 |
|  | 17 | 40713676 | 40714003 | PDFSTATSENSEMBLUCSC | 328 | 8 | 17365 | tss | 0.031568 | 0.397819 | RP11-400F19.8; COASY (margin: RP11-400F19.8; HSD17B1; COASY; MLX; RP11-400F19.6; PSMC3IP) | 4.39 | 6.99e-03 | 3.19e-02 |
|  | 19 | 2302783 | 2303032 | PDFSTATSENSEMBLUCSC | 250 | 4 | 19686 | gene;island | 0.509798 | 0.966818 | Not queried | 4.38 | 2.00e-01 | 2.55e-01 |
|  | 8 | 116660311 | 116660697 | PDFSTATSENSEMBLUCSC | 387 | 4 | 39403 | gene;island | 0.113462 | 0.710234 | Not queried | 4.38 | 2.86e-02 | 6.12e-02 |
|  | 20 | 2517085 | 2517231 | PDFSTATSENSEMBLUCSC | 147 | 4 | 25546 | tss | 0.581157 | 0.938734 | Not queried | 4.38 | 2.86e-02 | 6.12e-02 |
|  | 12 | 131417943 | 131418404 | PDFSTATSENSEMBLUCSC | 462 | 4 | 11011 | island | 0.182896 | 0.751299 | Not queried | 4.38 | 2.86e-02 | 6.12e-02 |
|  | 3 | 173115252 | 173116248 | PDFSTATSENSEMBLUCSC | 997 | 13 | 29671 | island | 0.545143 | 0.948845 | NLGN1 (margin: NLGN1) | 4.38 | 8.65e-06 | 4.82e-04 |
|  | 4 | 174443242 | 174443567 | PDFSTATSENSEMBLUCSC | 326 | 4 | 31427 | island | 0.575973 | 0.968510 | Not queried | 4.38 | 2.86e-02 | 6.12e-02 |
|  | 10 | 102988831 | 102988889 | PDFSTATSENSEMBLUCSC | 59 | 4 | 5305 | tss;island | 0.480944 | 0.947342 | Not queried | 4.38 | 2.86e-02 | 6.12e-02 |
|  | 7 | 4921827 | 4922196 | PDFSTATSENSEMBLUCSC | 370 | 4 | 36864 | gene;island | 0.631580 | 0.975124 | Not queried | 4.38 | 2.86e-02 | 6.12e-02 |
|  | 2 | 264120 | 264204 | PDFSTATSENSEMBLUCSC | 85 | 6 | 23152 | tss;island | 0.109763 | 0.004911 | ACP1; SH3YL1 (margin: ACP1; SH3YL1) | 4.38 | 4.33e-03 | 2.52e-02 |
|  | 20 | 21686308 | 21686728 | PDFSTATSENSEMBLUCSC | 421 | 4 | 25374 | gene | 0.427126 | 0.943985 | Not queried | 4.38 | 5.71e-02 | 1.00e-01 |
|  | 1 | 98515041 | 98515272 | PDFSTATSENSEMBLUCSC | 232 | 4 | 3515 | island | 0.519187 | 0.112431 | Not queried | 4.38 | 5.71e-02 | 1.00e-01 |
|  | 19 | 23299649 | 23300243 | PDFSTATSENSEMBLUCSC | 595 | 5 | 21844 | island | 0.543213 | 0.057817 | Not queried | 4.38 | 3.17e-02 | 6.50e-02 |
|  | 8 | 12989237 | 12989686 | PDFSTATSENSEMBLUCSC | 450 | 4 | 39211 | gene;island | 0.473208 | 0.028025 | Not queried | 4.37 | 5.71e-02 | 1.00e-01 |
|  | 8 | 56433594 | 56433717 | PDFSTATSENSEMBLUCSC | 124 | 4 | 39309 | gene | 0.265284 | 0.014873 | Not queried | 4.37 | 2.86e-02 | 6.12e-02 |
|  | 17 | 43129629 | 43129957 | PDFSTATSENSEMBLUCSC | 329 | 4 | 16625 | gene;tss;island | 0.021460 | 0.258020 | Not queried | 4.37 | 6.86e-01 | 6.99e-01 |
|  | 5 | 140562562 | 140563400 | PDFSTATSENSEMBLUCSC | 839 | 8 | 31813 | gene;island | 0.662667 | 0.977164 | PCDHB16 (margin: PCDHB7; PCDHB8; PCDHB16; PCDHB9; PCDHB10) | 4.37 | 3.11e-04 | 4.77e-03 |
|  | 3 | 142839837 | 142840240 | PDFSTATSENSEMBLUCSC | 404 | 5 | 27980 | gene;island | 0.769801 | 0.974507 | CHST2; RP11-80H8.4 (margin: RP11-80H8.3; CHST2; RP11-80H8.4) | 4.37 | 7.94e-03 | 3.19e-02 |
|  | 10 | 26726877 | 26727261 | PDFSTATSENSEMBLUCSC | 385 | 7 | 4994 | tss | 0.489592 | 0.902020 | APBB1IP (margin: APBB1IP) | 4.37 | 5.83e-04 | 6.47e-03 |
|  | 4 | 187422005 | 187422119 | PDFSTATSENSEMBLUCSC | 115 | 5 | 31481 | island | 0.704594 | 0.978871 | Not queried | 4.37 | 5.56e-02 | 1.00e-01 |
|  | 17 | 1553415 | 1553661 | PDFSTATSENSEMBLUCSC | 247 | 7 | 16990 | tss | 0.043918 | 0.431034 | Not queried | 4.37 | 1.75e-02 | 5.79e-02 |
|  | 11 | 114069864 | 114070210 | PDFSTATSENSEMBLUCSC | 347 | 6 | 6911 | gene;island | 0.589264 | 0.972092 | ZBTB16 (margin: ZBTB16; RP11-64D24.2) | 4.37 | 2.16e-03 | 1.45e-02 |
|  | 7 | 8476730 | 8477156 | PDFSTATSENSEMBLUCSC | 427 | 4 | 36890 | gene;island | 0.371241 | 0.061602 | Not queried | 4.37 | 5.71e-02 | 1.00e-01 |
|  | 14 | 101499893 | 101500080 | PDFSTATSENSEMBLUCSC | 188 | 4 | 12624 | tss | 0.474132 | 0.960745 | Not queried | 4.37 | 2.86e-02 | 6.12e-02 |
|  | 10 | 131323903 | 131324189 | PDFSTATSENSEMBLUCSC | 287 | 4 | 4824 | gene | 0.620049 | 0.978401 | Not queried | 4.36 | 2.86e-02 | 6.12e-02 |
|  | 6 | 32711889 | 32712103 | PDFSTATSENSEMBLUCSC | 215 | 4 | 34129 | gene | 0.102833 | 0.751006 | Not queried | 4.36 | 3.43e-01 | 3.84e-01 |
|  | 7 | 23221200 | 23221430 | PDFSTATSENSEMBLUCSC | 231 | 6 | 37470 | tss | 0.008393 | 0.113207 | Not queried | 4.36 | 1.52e-02 | 5.36e-02 |
|  | 3 | 183894727 | 183894778 | PDFSTATSENSEMBLUCSC | 52 | 4 | 28055 | gene;island | 0.656225 | 0.974075 | Not queried | 4.36 | 2.86e-02 | 6.12e-02 |
|  | 17 | 1082526 | 1083491 | PDFSTATSENSEMBLUCSC | 966 | 9 | 16310 | gene;island | 0.085581 | 0.009407 | AC016292.1; ABR (margin: AC016292.1; ABR) | 4.36 | 5.64e-03 | 3.18e-02 |
|  | 7 | 100493365 | 100493849 | PDFSTATSENSEMBLUCSC | 485 | 7 | 38785 | island | 0.216535 | 0.008756 | ACHE (margin: SRRT; UFSP1; ACHE; RN7SL549P) | 4.36 | 4.08e-03 | 2.47e-02 |
|  | 16 | 2801668 | 2801952 | PDFSTATSENSEMBLUCSC | 285 | 5 | 14612 | gene;tss;island | 0.678584 | 0.043903 | Not queried | 4.36 | 5.56e-02 | 1.00e-01 |
|  | 5 | 75698309 | 75698508 | PDFSTATSENSEMBLUCSC | 200 | 4 | 32133 | tss;island | 0.099160 | 0.007300 | Not queried | 4.36 | 2.86e-02 | 6.12e-02 |
|  | 14 | 89259551 | 89259748 | PDFSTATSENSEMBLUCSC | 198 | 4 | 12547 | tss;island | 0.418032 | 0.033033 | Not queried | 4.36 | 2.00e-01 | 2.55e-01 |
|  | 1 | 160068041 | 160068371 | PDFSTATSENSEMBLUCSC | 331 | 4 | 840 | gene | 0.023816 | 0.295692 | Not queried | 4.36 | 5.71e-02 | 1.00e-01 |
|  | 6 | 4775063 | 4775222 | PDFSTATSENSEMBLUCSC | 160 | 4 | 33541 | gene;island | 0.777510 | 0.990203 | Not queried | 4.36 | 2.86e-02 | 6.12e-02 |
|  | 8 | 144635260 | 144635547 | PDFSTATSENSEMBLUCSC | 288 | 9 | 39950 | tss | 0.532168 | 0.970844 | GSDMD (margin: 7SK; GSDMD; ZC3H3; RP11-661A12.5) | 4.36 | 4.11e-05 | 1.17e-03 |
|  | 4 | 73434487 | 73434856 | PDFSTATSENSEMBLUCSC | 370 | 5 | 31092 | island | 0.027031 | 0.591170 | ADAMTS3 (margin: ADAMTS3) | 4.36 | 7.94e-03 | 3.19e-02 |
|  | 11 | 14913124 | 14913620 | PDFSTATSENSEMBLUCSC | 497 | 5 | 6519 | gene | 0.233310 | 0.014899 | CYP2R1 (margin: CYP2R1) | 4.35 | 7.94e-03 | 3.19e-02 |
|  | 11 | 30607950 | 30608198 | PDFSTATSENSEMBLUCSC | 249 | 9 | 7220 | tss | 0.343962 | 0.021375 | RP5-1024C24.1; MPPED2 (margin: RP5-1024C24.1; MPPED2) | 4.35 | 4.11e-05 | 1.17e-03 |
|  | 19 | 14444267 | 14444429 | PDFSTATSENSEMBLUCSC | 163 | 4 | 21678 | island | 0.084962 | 0.644776 | Not queried | 4.35 | 3.43e-01 | 3.84e-01 |
|  | 16 | 2762569 | 2762819 | PDFSTATSENSEMBLUCSC | 251 | 4 | 14609 | gene;island | 0.660403 | 0.980000 | Not queried | 4.35 | 5.71e-02 | 1.00e-01 |
|  | 20 | 62601556 | 62601743 | PDFSTATSENSEMBLUCSC | 188 | 4 | 25907 | tss;island | 0.729104 | 0.118251 | Not queried | 4.35 | 2.86e-02 | 6.12e-02 |
|  | 16 | 1967781 | 1968232 | PDFSTATSENSEMBLUCSC | 452 | 4 | 15569 | island | 0.496989 | 0.965245 | Not queried | 4.35 | 2.86e-02 | 6.12e-02 |
|  | 12 | 23998921 | 23999045 | PDFSTATSENSEMBLUCSC | 125 | 4 | 9082 | gene | 0.459378 | 0.047899 | Not queried | 4.35 | 3.43e-01 | 3.84e-01 |
|  | 6 | 168615184 | 168615500 | PDFSTATSENSEMBLUCSC | 317 | 6 | 36661 | island | 0.437395 | 0.957606 | (margin: RP11-503C24.1; RP11-503C24.2) | 4.35 | 8.66e-03 | 3.36e-02 |
|  | 3 | 183959465 | 183959853 | PDFSTATSENSEMBLUCSC | 389 | 4 | 28058 | gene;island | 0.038750 | 0.309600 | Not queried | 4.35 | 2.86e-02 | 6.12e-02 |
|  | 7 | 69064801 | 69065031 | PDFSTATSENSEMBLUCSC | 231 | 4 | 37031 | gene;island | 0.646072 | 0.049394 | Not queried | 4.35 | 2.86e-02 | 6.12e-02 |
|  | 16 | 86411222 | 86411456 | PDFSTATSENSEMBLUCSC | 235 | 5 | 16169 | island | 0.412291 | 0.933031 | Not queried | 4.35 | 3.17e-02 | 6.50e-02 |
|  | 1 | 240986512 | 240986898 | PDFSTATSENSEMBLUCSC | 387 | 5 | 1108 | gene;island | 0.728081 | 0.976548 | Not queried | 4.35 | 1.59e-02 | 5.36e-02 |
|  | 19 | 54415702 | 54416068 | PDFSTATSENSEMBLUCSC | 367 | 6 | 21045 | tss | 0.123643 | 0.693407 | Not queried | 4.34 | 6.49e-02 | 1.11e-01 |
|  | 7 | 127744210 | 127744617 | PDFSTATSENSEMBLUCSC | 408 | 4 | 38890 | island | 0.674528 | 0.974906 | Not queried | 4.34 | 2.86e-02 | 6.12e-02 |
|  | 1 | 7740243 | 7740286 | PDFSTATSENSEMBLUCSC | 44 | 4 | 187 | gene;island | 0.284516 | 0.033291 | Not queried | 4.34 | 2.86e-02 | 6.12e-02 |
|  | 5 | 80529067 | 80529340 | PDFSTATSENSEMBLUCSC | 274 | 6 | 31666 | gene | 0.974666 | 0.663692 | CKMT2; CTC-281B15.1 (margin: RASGRF2; CKMT2; CTC-281B15.1; CTD-2248H3.1) | 4.34 | 2.16e-03 | 1.45e-02 |
|  | 17 | 80289500 | 80289701 | PDFSTATSENSEMBLUCSC | 202 | 4 | 16943 | gene;island | 0.401656 | 0.929780 | Not queried | 4.34 | 2.86e-02 | 6.12e-02 |
|  | 12 | 6420020 | 6420052 | PDFSTATSENSEMBLUCSC | 33 | 6 | 9023 | gene;tss;island | 0.938658 | 0.435392 | PLEKHG6 (margin: PLEKHG6) | 4.34 | 2.16e-03 | 1.45e-02 |
|  | 5 | 170736021 | 170736572 | PDFSTATSENSEMBLUCSC | 552 | 10 | 33379 | island | 0.760427 | 0.981002 | TLX3 (margin: RANBP17; TLX3) | 4.34 | 1.08e-05 | 4.82e-04 |
|  | 12 | 15475116 | 15475342 | PDFSTATSENSEMBLUCSC | 227 | 6 | 9591 | tss | 0.200676 | 0.804411 | PTPRO; RERG (margin: PTPRO; RERG) | 4.34 | 2.16e-03 | 1.45e-02 |
|  | 15 | 41135793 | 41135900 | PDFSTATSENSEMBLUCSC | 108 | 4 | 13597 | tss;island | 0.177580 | 0.010688 | Not queried | 4.34 | 5.71e-02 | 1.00e-01 |
|  | 15 | 78631878 | 78632194 | PDFSTATSENSEMBLUCSC | 317 | 8 | 13834 | tss;island | 0.462600 | 0.948605 | Not queried | 4.34 | 6.50e-02 | 1.11e-01 |
|  | 20 | 10200138 | 10200617 | PDFSTATSENSEMBLUCSC | 480 | 5 | 25357 | gene;island | 0.095840 | 0.006278 | Not queried | 4.34 | 9.52e-02 | 1.53e-01 |
|  | 3 | 119298195 | 119298513 | PDFSTATSENSEMBLUCSC | 319 | 8 | 28523 | tss | 0.417264 | 0.030876 | Not queried | 4.34 | 2.07e-02 | 6.12e-02 |
|  | 11 | 2481206 | 2481641 | PDFSTATSENSEMBLUCSC | 436 | 7 | 6459 | gene;tss | 0.407075 | 0.967831 | KCNQ1 (margin: KCNQ1) | 4.34 | 6.99e-03 | 3.19e-02 |
|  | 7 | 91808223 | 91808598 | PDFSTATSENSEMBLUCSC | 376 | 4 | 38655 | island | 0.025152 | 0.365183 | Not queried | 4.34 | 2.00e-01 | 2.55e-01 |
|  | 13 | 114065669 | 114066074 | PDFSTATSENSEMBLUCSC | 406 | 4 | 11963 | island | 0.070961 | 0.645784 | Not queried | 4.34 | 2.86e-02 | 6.12e-02 |
|  | 1 | 37499309 | 37499649 | PDFSTATSENSEMBLUCSC | 341 | 4 | 431 | gene;island | 0.872682 | 0.362859 | Not queried | 4.34 | 3.43e-01 | 3.84e-01 |
|  | 2 | 233320486 | 233320854 | PDFSTATSENSEMBLUCSC | 369 | 5 | 25194 | island | 0.313516 | 0.909452 | Not queried | 4.33 | 3.17e-02 | 6.50e-02 |
|  | 10 | 102986525 | 102986973 | PDFSTATSENSEMBLUCSC | 449 | 6 | 6059 | island | 0.614839 | 0.977380 | LBX1 (margin: LBX1-AS1; LBX1) | 4.33 | 8.66e-03 | 3.36e-02 |
|  | 1 | 153329781 | 153330257 | PDFSTATSENSEMBLUCSC | 477 | 6 | 1941 | tss | 0.118560 | 0.761167 | Not queried | 4.33 | 4.11e-02 | 8.14e-02 |
|  | 3 | 169683976 | 169684395 | PDFSTATSENSEMBLUCSC | 420 | 5 | 28025 | gene;tss;island | 0.022632 | 0.014131 | Not queried | 4.33 | 4.21e-01 | 4.59e-01 |
|  | 17 | 39743164 | 39743427 | PDFSTATSENSEMBLUCSC | 264 | 4 | 17341 | tss | 0.414481 | 0.923594 | Not queried | 4.33 | 2.86e-02 | 6.12e-02 |
|  | 7 | 55516724 | 55517132 | PDFSTATSENSEMBLUCSC | 409 | 5 | 38505 | island | 0.702857 | 0.968149 | VOPP1 (margin: VOPP1) | 4.33 | 7.94e-03 | 3.19e-02 |
|  | 13 | 98829311 | 98829666 | PDFSTATSENSEMBLUCSC | 356 | 8 | 11197 | gene;island | 0.669895 | 0.978884 | FARP1; RNF113B (margin: FARP1; RNF113B) | 4.33 | 6.22e-04 | 6.71e-03 |
|  | 5 | 115697214 | 115697696 | PDFSTATSENSEMBLUCSC | 483 | 5 | 33031 | island | 0.645404 | 0.973916 | COMMD10 (margin: COMMD10) | 4.33 | 7.94e-03 | 3.19e-02 |
|  | 3 | 186648175 | 186648392 | PDFSTATSENSEMBLUCSC | 218 | 4 | 28777 | tss | 0.568908 | 0.969313 | Not queried | 4.33 | 2.86e-02 | 6.12e-02 |
|  | 19 | 17392656 | 17392923 | PDFSTATSENSEMBLUCSC | 268 | 4 | 19916 | gene;island | 0.818168 | 0.989770 | Not queried | 4.33 | 2.86e-02 | 6.12e-02 |
|  | 6 | 90121670 | 90121836 | PDFSTATSENSEMBLUCSC | 167 | 4 | 34414 | gene;island | 0.383023 | 0.031934 | Not queried | 4.33 | 2.86e-02 | 6.12e-02 |
|  | 2 | 9614471 | 9614744 | PDFSTATSENSEMBLUCSC | 274 | 7 | 24056 | island | 0.049781 | 0.477131 | Not queried | 4.33 | 2.62e-02 | 6.12e-02 |
|  | 6 | 21666391 | 21666939 | PDFSTATSENSEMBLUCSC | 549 | 8 | 35486 | island | 0.477496 | 0.921616 | LINC00340 (margin: LINC00340) | 4.33 | 6.22e-04 | 6.71e-03 |
|  | 19 | 35633258 | 35633794 | PDFSTATSENSEMBLUCSC | 537 | 5 | 19981 | gene | 0.695714 | 0.024124 | Not queried | 4.33 | 1.51e-01 | 2.11e-01 |
|  | 19 | 35633258 | 35634102 | PDFSTATSENSEMBLUCSC | 845 | 7 | 20700 | tss | 0.317771 | 0.023090 | Not queried | 4.33 | 1.65e-01 | 2.28e-01 |
|  | 11 | 134262988 | 134263349 | PDFSTATSENSEMBLUCSC | 362 | 5 | 7001 | gene | 0.379972 | 0.975784 | Not queried | 4.32 | 5.56e-02 | 1.00e-01 |
|  | 13 | 53423940 | 53424127 | PDFSTATSENSEMBLUCSC | 188 | 4 | 11466 | tss;island | 0.549859 | 0.941123 | Not queried | 4.32 | 2.86e-02 | 6.12e-02 |
|  | 7 | 130125200 | 130126024 | PDFSTATSENSEMBLUCSC | 825 | 12 | 37900 | tss | 0.815743 | 0.102061 | MEST; RP11-2E11.5 (margin: MEST; MIR335; RP11-2E11.5; hsa-mir-335) | 4.32 | 2.32e-03 | 1.52e-02 |
|  | 6 | 33360495 | 33360836 | PDFSTATSENSEMBLUCSC | 342 | 5 | 34272 | gene;island | 0.083967 | 0.365374 | Not queried | 4.32 | 5.56e-02 | 1.00e-01 |
|  | 8 | 67782729 | 67783229 | PDFSTATSENSEMBLUCSC | 501 | 6 | 39748 | tss | 0.099643 | 0.434127 | Not queried | 4.32 | 1.80e-01 | 2.45e-01 |
|  | 14 | 101495604 | 101495910 | PDFSTATSENSEMBLUCSC | 307 | 6 | 12622 | tss | 0.392988 | 0.960604 | Not queried | 4.32 | 1.32e-01 | 1.89e-01 |
|  | 12 | 124246919 | 124247223 | PDFSTATSENSEMBLUCSC | 305 | 4 | 10959 | island | 0.786564 | 0.987433 | Not queried | 4.32 | 1.14e-01 | 1.68e-01 |
|  | 6 | 129478865 | 129479024 | PDFSTATSENSEMBLUCSC | 160 | 4 | 34472 | gene | 0.434540 | 0.957985 | Not queried | 4.32 | 5.71e-02 | 1.00e-01 |
|  | 1 | 6085799 | 6086652 | PDFSTATSENSEMBLUCSC | 854 | 10 | 2589 | island | 0.049736 | 0.547228 | KCNAB2 (margin: KCNAB2) | 4.32 | 7.58e-05 | 1.98e-03 |
|  | 15 | 83240550 | 83240791 | PDFSTATSENSEMBLUCSC | 242 | 7 | 13453 | gene | 0.487301 | 0.844147 | CPEB1 (margin: RP11-379H8.1; CPEB1) | 4.32 | 5.83e-04 | 6.47e-03 |
|  | 22 | 39378224 | 39378382 | PDFSTATSENSEMBLUCSC | 159 | 4 | 27148 | tss | 0.010268 | 0.183294 | Not queried | 4.32 | 2.86e-02 | 6.12e-02 |
|  | 5 | 178772203 | 178772390 | PDFSTATSENSEMBLUCSC | 188 | 4 | 33483 | island | 0.589368 | 0.961282 | Not queried | 4.31 | 2.86e-02 | 6.12e-02 |
|  | 15 | 31621629 | 31621843 | PDFSTATSENSEMBLUCSC | 215 | 4 | 13273 | gene;island | 0.485811 | 0.921952 | Not queried | 4.31 | 2.00e-01 | 2.55e-01 |
|  | 5 | 65892993 | 65893538 | PDFSTATSENSEMBLUCSC | 546 | 5 | 31635 | gene;island | 0.088883 | 0.010228 | MAST4 (margin: MAST4) | 4.31 | 7.94e-03 | 3.19e-02 |
|  | 14 | 104940229 | 104940658 | PDFSTATSENSEMBLUCSC | 430 | 5 | 13206 | island | 0.505771 | 0.971277 | TMEM179 (margin: TMEM179) | 4.31 | 7.94e-03 | 3.19e-02 |
|  | 16 | 1221825 | 1222188 | PDFSTATSENSEMBLUCSC | 364 | 4 | 14557 | gene;island | 0.515041 | 0.915718 | Not queried | 4.31 | 2.86e-02 | 6.12e-02 |
|  | 11 | 1404502 | 1404750 | PDFSTATSENSEMBLUCSC | 249 | 4 | 7920 | island | 0.344621 | 0.066397 | Not queried | 4.31 | 1.14e-01 | 1.68e-01 |
|  | 1 | 59247425 | 59247797 | PDFSTATSENSEMBLUCSC | 373 | 5 | 558 | gene;island | 0.004355 | 0.041325 | JUN (margin: RP4-794H19.2; JUN) | 4.31 | 7.94e-03 | 3.19e-02 |
|  | 5 | 1445354 | 1445593 | PDFSTATSENSEMBLUCSC | 240 | 6 | 32629 | island | 0.442529 | 0.966869 | SLC6A3 (margin: SLC6A3; LPCAT1) | 4.31 | 2.16e-03 | 1.45e-02 |
|  | 19 | 12978611 | 12978805 | PDFSTATSENSEMBLUCSC | 195 | 5 | 19871 | gene;island | 0.687689 | 0.976858 | MAST1; HOOK2 (margin: MAST1; HOOK2; AC020934.1; DNASE2) | 4.31 | 7.94e-03 | 3.19e-02 |
|  | 6 | 30881112 | 30881842 | PDFSTATSENSEMBLUCSC | 731 | 23 | 33835 | gene | 0.916063 | 0.106615 | GTF2H4; VARS2 (margin: RN7SL175P; GTF2H4; VARS2) | 4.31 | 9.28e-06 | 4.82e-04 |
|  | 20 | 57471654 | 57471844 | PDFSTATSENSEMBLUCSC | 191 | 5 | 25476 | gene | 0.640215 | 0.980491 | Not queried | 4.31 | 3.17e-02 | 6.50e-02 |
|  | 13 | 108922032 | 108922504 | PDFSTATSENSEMBLUCSC | 473 | 7 | 11224 | gene | 0.096754 | 0.656477 | TNFSF13B (margin: TNFSF13B) | 4.31 | 2.33e-03 | 1.52e-02 |
|  | 21 | 45773189 | 45773400 | PDFSTATSENSEMBLUCSC | 212 | 4 | 26584 | tss | 0.286883 | 0.717608 | Not queried | 4.31 | 2.00e-01 | 2.55e-01 |
|  | 11 | 17566011 | 17566163 | PDFSTATSENSEMBLUCSC | 153 | 6 | 7177 | tss | 0.213585 | 0.012537 | Not queried | 4.30 | 6.49e-02 | 1.11e-01 |
|  | 3 | 173115252 | 173116238 | PDFSTATSENSEMBLUCSC | 987 | 12 | 28723 | tss | 0.547077 | 0.952922 | NLGN1 (margin: NLGN1) | 4.30 | 3.33e-05 | 1.17e-03 |
|  | 5 | 170736021 | 170736277 | PDFSTATSENSEMBLUCSC | 257 | 7 | 32486 | tss | 0.775874 | 0.975876 | TLX3 (margin: RANBP17; TLX3) | 4.30 | 5.83e-04 | 6.47e-03 |
|  | 2 | 71205052 | 71205685 | PDFSTATSENSEMBLUCSC | 634 | 7 | 24391 | island | 0.389157 | 0.920247 | ANKRD53; AC007040.11 (margin: ANKRD53; AC007040.11; TEX261) | 4.30 | 2.33e-03 | 1.52e-02 |
|  | 17 | 57642628 | 57642873 | PDFSTATSENSEMBLUCSC | 246 | 8 | 17546 | tss | 0.974372 | 0.661918 | Not queried | 4.30 | 6.50e-02 | 1.11e-01 |
|  | 1 | 102462445 | 102462682 | PDFSTATSENSEMBLUCSC | 238 | 5 | 656 | gene | 0.502977 | 0.049070 | OLFM3 (margin: OLFM3) | 4.29 | 7.94e-03 | 3.19e-02 |
|  | 12 | 26348331 | 26348529 | PDFSTATSENSEMBLUCSC | 199 | 6 | 9086 | gene;island | 0.282266 | 0.021869 | SSPN (margin: SSPN) | 4.29 | 4.33e-03 | 2.52e-02 |
|  | 18 | 77193464 | 77193759 | PDFSTATSENSEMBLUCSC | 296 | 4 | 19138 | gene;island | 0.631894 | 0.957681 | Not queried | 4.29 | 2.86e-02 | 6.12e-02 |
|  | 10 | 28034669 | 28034856 | PDFSTATSENSEMBLUCSC | 188 | 7 | 5634 | island | 0.739197 | 0.977454 | RP11-360I20.2; MKX (margin: RP11-360I20.2; MKX) | 4.29 | 5.83e-04 | 6.47e-03 |
|  | 4 | 186732837 | 186733060 | PDFSTATSENSEMBLUCSC | 224 | 7 | 30212 | gene;tss | 0.755083 | 0.983242 | SORBS2 (margin: SORBS2) | 4.29 | 5.83e-04 | 6.47e-03 |
|  | 12 | 114846313 | 114846503 | PDFSTATSENSEMBLUCSC | 191 | 7 | 10033 | tss | 0.638526 | 0.969805 | TBX5-AS1; TBX5 (margin: TBX5-AS1; TBX5) | 4.29 | 5.83e-04 | 6.47e-03 |
|  | 5 | 15499895 | 15500270 | PDFSTATSENSEMBLUCSC | 376 | 4 | 31994 | tss;island | 0.493494 | 0.070427 | Not queried | 4.29 | 2.86e-02 | 6.12e-02 |
|  | 14 | 101347044 | 101347358 | PDFSTATSENSEMBLUCSC | 315 | 6 | 12210 | gene;tss;island | 0.161891 | 0.860214 | MIR431; MIR433; MIR127; RTL1 (margin: MIR493; MIR337; MIR665; MIR431; MIR433; MIR127; MIR432; MIR136; AL117190.3; RTL1) | 4.29 | 2.16e-03 | 1.45e-02 |
|  | 17 | 46805785 | 46806056 | PDFSTATSENSEMBLUCSC | 272 | 4 | 16666 | gene | 0.086457 | 0.666777 | Not queried | 4.29 | 2.86e-02 | 6.12e-02 |
|  | 15 | 28344477 | 28344730 | PDFSTATSENSEMBLUCSC | 254 | 10 | 13544 | tss | 0.129407 | 0.934950 | Not queried | 4.29 | 3.55e-02 | 7.24e-02 |
|  | 8 | 70747337 | 70747646 | PDFSTATSENSEMBLUCSC | 310 | 5 | 39756 | tss | 0.492299 | 0.013388 | Not queried | 4.29 | 1.59e-02 | 5.36e-02 |
|  | 13 | 28366427 | 28366814 | PDFSTATSENSEMBLUCSC | 388 | 9 | 11630 | island | 0.947078 | 0.497858 | Not queried | 4.29 | 1.42e-02 | 5.24e-02 |
|  | 6 | 66497586 | 66498032 | PDFSTATSENSEMBLUCSC | 447 | 4 | 36231 | island | 0.347905 | 0.951993 | Not queried | 4.29 | 1.14e-01 | 1.68e-01 |
|  | 17 | 20747058 | 20747314 | PDFSTATSENSEMBLUCSC | 257 | 4 | 18111 | island | 0.954432 | 0.584374 | Not queried | 4.29 | 3.43e-01 | 3.84e-01 |
|  | 2 | 191207820 | 191208171 | PDFSTATSENSEMBLUCSC | 352 | 4 | 23785 | tss | 0.374474 | 0.891880 | Not queried | 4.28 | 2.00e-01 | 2.55e-01 |
|  | 4 | 93224895 | 93225565 | PDFSTATSENSEMBLUCSC | 671 | 8 | 31183 | island | 0.493817 | 0.047642 | GRID2; RP11-9B6.1 (margin: GRID2; RP11-9B6.1) | 4.28 | 1.86e-03 | 1.45e-02 |
|  | 20 | 44650068 | 44650449 | PDFSTATSENSEMBLUCSC | 382 | 8 | 26212 | island | 0.743110 | 0.053422 | Not queried | 4.28 | 1.48e-02 | 5.36e-02 |
|  | 7 | 18126902 | 18127112 | PDFSTATSENSEMBLUCSC | 211 | 4 | 38280 | island | 0.482452 | 0.922915 | Not queried | 4.28 | 2.86e-02 | 6.12e-02 |
|  | 2 | 177053345 | 177053568 | PDFSTATSENSEMBLUCSC | 224 | 4 | 22940 | gene | 0.070503 | 0.566532 | Not queried | 4.28 | 2.86e-02 | 6.12e-02 |
|  | 4 | 80246713 | 80247088 | PDFSTATSENSEMBLUCSC | 376 | 4 | 30032 | gene | 0.229889 | 0.741955 | Not queried | 4.28 | 2.86e-02 | 6.12e-02 |
|  | 3 | 112931126 | 112931496 | PDFSTATSENSEMBLUCSC | 371 | 8 | 29363 | island | 0.410678 | 0.042093 | BOC (margin: BOC) | 4.28 | 1.86e-03 | 1.45e-02 |
|  | 11 | 1483099 | 1483390 | PDFSTATSENSEMBLUCSC | 292 | 4 | 7925 | island | 0.790335 | 0.985854 | Not queried | 4.28 | 2.86e-02 | 6.12e-02 |
|  | 11 | 69590282 | 69590580 | PDFSTATSENSEMBLUCSC | 299 | 7 | 7522 | tss | 0.517153 | 0.954422 | FGF4 (margin: AP001888.1; FGF4) | 4.28 | 5.83e-04 | 6.47e-03 |
|  | 10 | 23216637 | 23216847 | PDFSTATSENSEMBLUCSC | 211 | 6 | 4986 | tss | 0.647070 | 0.106642 | ARMC3 (margin: ARMC3) | 4.28 | 2.16e-03 | 1.45e-02 |
|  | 4 | 185942086 | 185942292 | PDFSTATSENSEMBLUCSC | 207 | 4 | 31469 | island | 0.441460 | 0.961796 | Not queried | 4.28 | 1.14e-01 | 1.68e-01 |
|  | 5 | 135416331 | 135416613 | PDFSTATSENSEMBLUCSC | 283 | 9 | 32310 | tss | 0.536402 | 0.954643 | VTRNA2-1 (margin: VTRNA2-1) | 4.28 | 8.23e-05 | 2.06e-03 |
|  | 5 | 123987423 | 123987747 | PDFSTATSENSEMBLUCSC | 325 | 5 | 31733 | gene;island | 0.016778 | 0.003455 | Not queried | 4.28 | 3.17e-02 | 6.50e-02 |
|  | 2 | 180725637 | 180726035 | PDFSTATSENSEMBLUCSC | 399 | 6 | 23758 | tss | 0.432116 | 0.053692 | Not queried | 4.27 | 1.32e-01 | 1.89e-01 |
|  | 5 | 1876168 | 1876397 | PDFSTATSENSEMBLUCSC | 230 | 5 | 32643 | island | 0.859674 | 0.990428 | Not queried | 4.27 | 3.17e-02 | 6.50e-02 |
|  | 10 | 133508246 | 133508866 | PDFSTATSENSEMBLUCSC | 621 | 7 | 6278 | island | 0.297049 | 0.944735 | (margin: ) | 4.27 | 5.83e-04 | 6.47e-03 |
|  | 4 | 142054660 | 142054878 | PDFSTATSENSEMBLUCSC | 219 | 8 | 30609 | tss;island | 0.137613 | 0.005399 | RNF150 (margin: RNF150) | 4.27 | 4.66e-03 | 2.68e-02 |
|  | 7 | 156741514 | 156741808 | PDFSTATSENSEMBLUCSC | 295 | 5 | 38018 | tss;island | 0.981004 | 0.664062 | Not queried | 4.27 | 3.10e-01 | 3.66e-01 |
|  | 20 | 61151391 | 61151480 | PDFSTATSENSEMBLUCSC | 90 | 6 | 25490 | gene;tss;island | 0.116732 | 0.728127 | C20orf166; MIR1-1 (margin: C20orf166; MIR1-1; MIR133A2; C20orf166-AS1) | 4.27 | 2.16e-03 | 1.45e-02 |
|  | 4 | 4868723 | 4869172 | PDFSTATSENSEMBLUCSC | 450 | 6 | 30874 | island | 0.394814 | 0.958269 | Not queried | 4.27 | 4.11e-02 | 8.14e-02 |
|  | 15 | 96868857 | 96869132 | PDFSTATSENSEMBLUCSC | 276 | 7 | 13917 | tss | 0.031723 | 0.003339 | Not queried | 4.27 | 2.62e-02 | 6.12e-02 |
|  | 2 | 150444370 | 150444834 | PDFSTATSENSEMBLUCSC | 465 | 6 | 23657 | tss | 0.021572 | 0.001954 | Not queried | 4.27 | 1.32e-01 | 1.89e-01 |
|  | 15 | 37403088 | 37403304 | PDFSTATSENSEMBLUCSC | 217 | 5 | 14015 | island | 0.423686 | 0.932819 | (margin: RP11-128A17.1; MEIS2) | 4.27 | 7.94e-03 | 3.19e-02 |
|  | 19 | 14183619 | 14183792 | PDFSTATSENSEMBLUCSC | 174 | 5 | 20537 | tss;island | 0.135269 | 0.013700 | Not queried | 4.27 | 1.59e-02 | 5.36e-02 |
|  | 22 | 38071455 | 38071607 | PDFSTATSENSEMBLUCSC | 153 | 5 | 27121 | tss | 0.014990 | 0.219301 | LGALS1 (margin: SH3BP1; PDXP; LGALS1; NOL12; RP1-37E16.12; RN7SL385P) | 4.27 | 7.94e-03 | 3.19e-02 |
|  | 5 | 133451237 | 133451625 | PDFSTATSENSEMBLUCSC | 389 | 8 | 31750 | gene;island | 0.191375 | 0.848365 | TCF7 (margin: TCF7) | 4.26 | 1.09e-03 | 1.10e-02 |
|  | 6 | 90660913 | 90661373 | PDFSTATSENSEMBLUCSC | 461 | 5 | 34416 | gene | 0.192101 | 0.725125 | Not queried | 4.26 | 1.59e-02 | 5.36e-02 |
|  | 5 | 148520669 | 148521226 | PDFSTATSENSEMBLUCSC | 558 | 11 | 33293 | island | 0.033761 | 0.506360 | ABLIM3 (margin: ABLIM3) | 4.26 | 8.33e-03 | 3.34e-02 |
|  | 4 | 40859249 | 40859344 | PDFSTATSENSEMBLUCSC | 96 | 6 | 30361 | tss | 0.967730 | 0.605984 | APBB2 (margin: APBB2; snoU13) | 4.26 | 2.16e-03 | 1.45e-02 |
|  | 14 | 85998439 | 85998669 | PDFSTATSENSEMBLUCSC | 231 | 4 | 12163 | gene;island | 0.292437 | 0.828790 | Not queried | 4.26 | 2.86e-02 | 6.12e-02 |
|  | 19 | 49925720 | 49926070 | PDFSTATSENSEMBLUCSC | 351 | 4 | 20149 | gene;island | 0.544961 | 0.957489 | Not queried | 4.26 | 1.14e-01 | 1.68e-01 |
|  | 3 | 171178943 | 171179043 | PDFSTATSENSEMBLUCSC | 101 | 4 | 28718 | tss;island | 0.117391 | 0.013234 | Not queried | 4.26 | 2.86e-02 | 6.12e-02 |
|  | 10 | 88125351 | 88125521 | PDFSTATSENSEMBLUCSC | 171 | 4 | 4660 | gene;island | 0.295342 | 0.951480 | Not queried | 4.26 | 5.71e-02 | 1.00e-01 |
|  | 1 | 22470218 | 22470343 | PDFSTATSENSEMBLUCSC | 126 | 4 | 1331 | tss;island | 0.025995 | 0.372259 | Not queried | 4.26 | 1.14e-01 | 1.68e-01 |
|  | 7 | 82073339 | 82073810 | PDFSTATSENSEMBLUCSC | 472 | 7 | 37677 | tss;island | 0.438566 | 0.026865 | CACNA2D1 (margin: CACNA2D1) | 4.26 | 5.83e-04 | 6.47e-03 |
|  | 12 | 26348331 | 26348463 | PDFSTATSENSEMBLUCSC | 133 | 5 | 9613 | tss | 0.279863 | 0.024105 | Not queried | 4.26 | 1.59e-02 | 5.36e-02 |
|  | 10 | 124907409 | 124907600 | PDFSTATSENSEMBLUCSC | 192 | 5 | 5417 | tss | 0.662513 | 0.973343 | Not queried | 4.26 | 5.56e-02 | 1.00e-01 |
|  | 5 | 1243714 | 1243949 | PDFSTATSENSEMBLUCSC | 236 | 5 | 31528 | gene;island | 0.404799 | 0.946942 | SLC6A18 (margin: SLC6A18; TERT) | 4.26 | 7.94e-03 | 3.19e-02 |
|  | 1 | 43533796 | 43534324 | PDFSTATSENSEMBLUCSC | 529 | 6 | 3157 | island | 0.955390 | 0.534578 | (margin: ) | 4.25 | 2.16e-03 | 1.45e-02 |
|  | 13 | 79168044 | 79168370 | PDFSTATSENSEMBLUCSC | 327 | 5 | 11791 | island | 0.475709 | 0.052842 | Not queried | 4.25 | 3.17e-02 | 6.50e-02 |
|  | 4 | 4389509 | 4389918 | PDFSTATSENSEMBLUCSC | 410 | 4 | 29915 | gene;island | 0.158559 | 0.745781 | Not queried | 4.25 | 2.86e-02 | 6.12e-02 |
|  | 11 | 134201447 | 134201640 | PDFSTATSENSEMBLUCSC | 194 | 6 | 7833 | tss;island | 0.319516 | 0.028015 | GLB1L2 (margin: GLB1L3; GLB1L2) | 4.25 | 4.33e-03 | 2.52e-02 |
|  | 6 | 28601269 | 28601519 | PDFSTATSENSEMBLUCSC | 251 | 14 | 35592 | island | 0.969866 | 0.626514 | RP11-373N24.2 (margin: RP11-373N24.2) | 4.25 | 4.99e-08 | 9.34e-06 |
|  | 12 | 132698353 | 132698786 | PDFSTATSENSEMBLUCSC | 434 | 4 | 9435 | gene;island | 0.152726 | 0.816945 | Not queried | 4.25 | 2.00e-01 | 2.55e-01 |
|  | 10 | 129534107 | 129534411 | PDFSTATSENSEMBLUCSC | 305 | 4 | 5435 | tss | 0.410367 | 0.869143 | Not queried | 4.25 | 2.86e-02 | 6.12e-02 |
|  | 19 | 4535211 | 4535582 | PDFSTATSENSEMBLUCSC | 372 | 8 | 20366 | tss | 0.090342 | 0.008659 | PLIN5; CTB-50L17.14; LRG1 (margin: RN7SL121P; PLIN5; CTB-50L17.14; LRG1; SEMA6B) | 4.25 | 1.04e-02 | 3.96e-02 |
|  | 2 | 207308004 | 207308375 | PDFSTATSENSEMBLUCSC | 372 | 4 | 25034 | island | 0.026717 | 0.360091 | Not queried | 4.25 | 2.86e-02 | 6.12e-02 |
|  | 6 | 166891617 | 166891959 | PDFSTATSENSEMBLUCSC | 343 | 5 | 34546 | gene | 0.740611 | 0.978701 | RPS6KA2 (margin: RPS6KA2) | 4.25 | 7.94e-03 | 3.19e-02 |
|  | 3 | 157221198 | 157221329 | PDFSTATSENSEMBLUCSC | 132 | 4 | 28010 | gene;tss | 0.326877 | 0.914054 | Not queried | 4.24 | 1.14e-01 | 1.68e-01 |
|  | 11 | 62621178 | 62621406 | PDFSTATSENSEMBLUCSC | 229 | 4 | 6649 | gene;tss;island | 0.003647 | 0.066773 | Not queried | 4.24 | 2.86e-02 | 6.12e-02 |
|  | 5 | 1225074 | 1225434 | PDFSTATSENSEMBLUCSC | 361 | 8 | 31968 | tss | 0.180399 | 0.908712 | SLC6A19; SLC6A18 (margin: SLC6A19; SLC6A18) | 4.24 | 6.99e-03 | 3.19e-02 |
|  | 5 | 113698140 | 113698506 | PDFSTATSENSEMBLUCSC | 367 | 4 | 31717 | gene | 0.918513 | 0.505017 | Not queried | 4.24 | 2.86e-02 | 6.12e-02 |
|  | 8 | 146277200 | 146277480 | PDFSTATSENSEMBLUCSC | 281 | 4 | 39999 | tss;island | 0.267992 | 0.012207 | Not queried | 4.24 | 2.86e-02 | 6.12e-02 |
|  | 17 | 41977951 | 41978218 | PDFSTATSENSEMBLUCSC | 268 | 5 | 16600 | gene;island | 0.280648 | 0.008880 | Not queried | 4.24 | 3.17e-02 | 6.50e-02 |
|  | 21 | 43431083 | 43431529 | PDFSTATSENSEMBLUCSC | 447 | 4 | 26562 | tss;island | 0.433127 | 0.049865 | Not queried | 4.24 | 3.43e-01 | 3.84e-01 |
|  | 1 | 57044733 | 57045219 | PDFSTATSENSEMBLUCSC | 487 | 5 | 552 | gene;island | 0.025611 | 0.002218 | Not queried | 4.24 | 4.21e-01 | 4.59e-01 |
|  | 15 | 56035371 | 56035616 | PDFSTATSENSEMBLUCSC | 246 | 6 | 13685 | tss;island | 0.416856 | 0.027345 | Not queried | 4.24 | 2.60e-02 | 6.12e-02 |
|  | 4 | 144621898 | 144621971 | PDFSTATSENSEMBLUCSC | 74 | 5 | 30615 | tss;island | 0.956013 | 0.489243 | RP13-578N3.3; FREM3 (margin: RP13-578N3.3; FREM3) | 4.23 | 7.94e-03 | 3.19e-02 |
|  | 12 | 85673860 | 85674694 | PDFSTATSENSEMBLUCSC | 835 | 11 | 10658 | island | 0.630367 | 0.973145 | ALX1 (margin: ALX1) | 4.23 | 5.24e-03 | 2.98e-02 |
|  | 1 | 230468464 | 230468773 | PDFSTATSENSEMBLUCSC | 310 | 5 | 1071 | gene;island | 0.274374 | 0.949278 | Not queried | 4.23 | 1.51e-01 | 2.11e-01 |
|  | 3 | 147124363 | 147124827 | PDFSTATSENSEMBLUCSC | 465 | 6 | 28651 | tss;island | 0.552796 | 0.970184 | Not queried | 4.23 | 1.52e-02 | 5.36e-02 |
|  | 4 | 164253207 | 164254230 | PDFSTATSENSEMBLUCSC | 1024 | 11 | 31400 | island | 0.054112 | 0.006540 | NPY1R (margin: NPY5R; NPY1R) | 4.23 | 5.24e-03 | 2.98e-02 |
|  | 18 | 49865711 | 49866531 | PDFSTATSENSEMBLUCSC | 821 | 17 | 19281 | tss | 0.396796 | 0.108037 | Not queried | 4.23 | 3.14e-02 | 6.50e-02 |
|  | 1 | 55504649 | 55505532 | PDFSTATSENSEMBLUCSC | 884 | 13 | 3306 | island | 0.047039 | 0.314911 | Not queried | 4.23 | 2.95e-02 | 6.30e-02 |
|  | 3 | 118753610 | 118753871 | PDFSTATSENSEMBLUCSC | 262 | 7 | 27904 | gene;island | 0.154896 | 0.007173 | IGSF11 (margin: IGSF11) | 4.23 | 2.33e-03 | 1.52e-02 |
|  | 3 | 118753705 | 118753871 | PDFSTATSENSEMBLUCSC | 167 | 5 | 28515 | tss | 0.154896 | 0.007173 | Not queried | 4.23 | 1.59e-02 | 5.36e-02 |
|  | 14 | 21769105 | 21769379 | PDFSTATSENSEMBLUCSC | 275 | 5 | 12012 | gene;island | 0.527010 | 0.018923 | Not queried | 4.23 | 3.17e-02 | 6.50e-02 |
|  | 8 | 25902284 | 25902611 | PDFSTATSENSEMBLUCSC | 328 | 6 | 40170 | island | 0.511699 | 0.087066 | EBF2 (margin: EBF2) | 4.22 | 2.16e-03 | 1.45e-02 |
|  | 19 | 54415702 | 54416165 | PDFSTATSENSEMBLUCSC | 464 | 7 | 22390 | island | 0.184685 | 0.832422 | Not queried | 4.22 | 1.65e-01 | 2.28e-01 |
|  | 6 | 30043727 | 30044259 | PDFSTATSENSEMBLUCSC | 533 | 10 | 34763 | tss | 0.044666 | 0.531996 | RNF39 (margin: ZNRD1; PPP1R11; RNF39) | 4.22 | 1.50e-03 | 1.41e-02 |
|  | 10 | 124907409 | 124907708 | PDFSTATSENSEMBLUCSC | 300 | 6 | 6217 | island | 0.698959 | 0.961622 | Not queried | 4.22 | 2.60e-02 | 6.12e-02 |
|  | 16 | 54228169 | 54228582 | PDFSTATSENSEMBLUCSC | 414 | 5 | 15929 | island | 0.807668 | 0.215833 | (margin: ) | 4.22 | 7.94e-03 | 3.19e-02 |
|  | 11 | 2596300 | 2596606 | PDFSTATSENSEMBLUCSC | 307 | 4 | 6464 | gene;island | 0.365618 | 0.914998 | Not queried | 4.22 | 5.71e-02 | 1.00e-01 |
|  | 5 | 114516141 | 114516422 | PDFSTATSENSEMBLUCSC | 282 | 6 | 33019 | island | 0.723822 | 0.978205 | Not queried | 4.22 | 1.52e-02 | 5.36e-02 |
|  | 7 | 1280040 | 1280640 | PDFSTATSENSEMBLUCSC | 601 | 5 | 38094 | island | 0.312065 | 0.898665 | (margin: UNCX) | 4.22 | 7.94e-03 | 3.19e-02 |
|  | 1 | 26233332 | 26233709 | PDFSTATSENSEMBLUCSC | 378 | 11 | 1380 | tss;island | 0.503164 | 0.952445 | STMN1; MIR3917 (margin: AL033528.1; STMN1; MIR3917) | 4.21 | 1.28e-04 | 2.71e-03 |
|  | 11 | 66034681 | 66036038 | PDFSTATSENSEMBLUCSC | 1358 | 55 | 7460 | tss | 0.292714 | 0.009635 | KLC2; RAB1B; RP11-867G23.1; RP11-867G23.2; RP11-867G23.3 (margin: KLC2; RAB1B; CNIH2; RP11-755F10.3; RP11-867G23.1; RP11-867G23.2; RP11-867G23.3; RP11-867G23.4) | 4.21 | 7.14e-06 | 4.37e-04 |
|  | 6 | 31673800 | 31673939 | PDFSTATSENSEMBLUCSC | 140 | 5 | 34830 | tss;island | 0.680691 | 0.975720 | Not queried | 4.21 | 3.10e-01 | 3.66e-01 |
|  | 11 | 71259251 | 71259439 | PDFSTATSENSEMBLUCSC | 189 | 4 | 7532 | tss | 0.395189 | 0.874369 | Not queried | 4.21 | 2.00e-01 | 2.55e-01 |
|  | 17 | 6679254 | 6679541 | PDFSTATSENSEMBLUCSC | 288 | 9 | 17053 | tss | 0.613975 | 0.976445 | XAF1; FBXO39 (margin: XAF1; FBXO39) | 4.21 | 2.88e-04 | 4.58e-03 |
|  | 1 | 201857840 | 201857955 | PDFSTATSENSEMBLUCSC | 116 | 6 | 942 | gene | 0.437680 | 0.044005 | SHISA4 (margin: IPO9; SHISA4; RP11-307B6.3; LMOD1) | 4.21 | 2.16e-03 | 1.45e-02 |
|  | 1 | 47697429 | 47697663 | PDFSTATSENSEMBLUCSC | 235 | 4 | 3232 | island | 0.613551 | 0.919866 | Not queried | 4.21 | 2.00e-01 | 2.55e-01 |
|  | 15 | 73735098 | 73735476 | PDFSTATSENSEMBLUCSC | 379 | 8 | 13782 | tss | 0.394118 | 0.930991 | C15orf60 (margin: C15orf60) | 4.20 | 6.99e-03 | 3.19e-02 |
|  | 14 | 101528123 | 101528671 | PDFSTATSENSEMBLUCSC | 549 | 11 | 13151 | island | 0.312633 | 0.826818 | MIR154; MIR496; MIR377 (margin: MIR381HG; AL132709.3; MIR487A; MIR134; MIR485; MIR323B; MIR154; MIR496; MIR377; MIR541; MIR409; MIR412; MIR369; MIR410; MIR656; MEG9; MIR382; AL132709.1) | 4.20 | 1.04e-02 | 3.96e-02 |
|  | 8 | 1921165 | 1921906 | PDFSTATSENSEMBLUCSC | 742 | 7 | 39536 | tss;island | 0.342757 | 0.008428 | Not queried | 4.20 | 2.62e-02 | 6.12e-02 |
|  | 20 | 61718852 | 61719188 | PDFSTATSENSEMBLUCSC | 337 | 5 | 26341 | island | 0.434370 | 0.975858 | Not queried | 4.20 | 9.52e-02 | 1.53e-01 |
|  | 19 | 22816896 | 22817496 | PDFSTATSENSEMBLUCSC | 601 | 10 | 21843 | island | 0.484423 | 0.018276 | ZNF492 (margin: ZNF492; AC011516.2) | 4.20 | 3.25e-04 | 4.91e-03 |
|  | 19 | 22816896 | 22817096 | PDFSTATSENSEMBLUCSC | 201 | 6 | 20664 | tss | 0.484423 | 0.018276 | ZNF492 (margin: ZNF492; AC011516.2) | 4.20 | 8.66e-03 | 3.36e-02 |
|  | 1 | 47973566 | 47973951 | PDFSTATSENSEMBLUCSC | 386 | 6 | 3242 | island | 0.233706 | 0.015451 | Not queried | 4.20 | 9.31e-02 | 1.53e-01 |
|  | 21 | 46077454 | 46077731 | PDFSTATSENSEMBLUCSC | 278 | 6 | 26459 | gene;tss | 0.495120 | 0.955067 | Not queried | 4.20 | 9.31e-02 | 1.53e-01 |
|  | 7 | 157502370 | 157502620 | PDFSTATSENSEMBLUCSC | 251 | 4 | 37340 | gene;island | 0.037606 | 0.408108 | Not queried | 4.20 | 2.00e-01 | 2.55e-01 |
|  | 10 | 50143337 | 50143517 | PDFSTATSENSEMBLUCSC | 181 | 5 | 4581 | gene | 0.312049 | 0.973627 | Not queried | 4.20 | 1.51e-01 | 2.11e-01 |
|  | 2 | 26395359 | 26395556 | PDFSTATSENSEMBLUCSC | 198 | 4 | 23237 | tss;island | 0.638027 | 0.959367 | Not queried | 4.20 | 2.86e-02 | 6.12e-02 |
|  | 2 | 12880542 | 12880961 | PDFSTATSENSEMBLUCSC | 420 | 4 | 22578 | gene | 0.420006 | 0.928828 | Not queried | 4.19 | 2.86e-02 | 6.12e-02 |
|  | 19 | 49198900 | 49199234 | PDFSTATSENSEMBLUCSC | 335 | 5 | 22230 | island | 0.321908 | 0.891192 | Not queried | 4.19 | 4.21e-01 | 4.59e-01 |
|  | 21 | 45148694 | 45149141 | PDFSTATSENSEMBLUCSC | 448 | 5 | 26447 | gene;island | 0.499797 | 0.939262 | PDXK; AP001052.9 (margin: PDXK; AP001052.9) | 4.19 | 7.94e-03 | 3.19e-02 |
|  | 11 | 85565852 | 85565989 | PDFSTATSENSEMBLUCSC | 138 | 7 | 7606 | tss;island | 0.006200 | 0.114151 | CCDC83; AP000974.1 (margin: CCDC83; AP000974.1) | 4.19 | 5.83e-04 | 6.47e-03 |
|  | 15 | 73735098 | 73735694 | PDFSTATSENSEMBLUCSC | 597 | 12 | 14289 | island | 0.463388 | 0.957353 | C15orf60 (margin: C15orf60) | 4.19 | 2.74e-04 | 4.46e-03 |
|  | 6 | 32974479 | 32974643 | PDFSTATSENSEMBLUCSC | 165 | 4 | 34175 | gene;island | 0.598851 | 0.939473 | Not queried | 4.19 | 1.14e-01 | 1.68e-01 |
|  | 5 | 169010289 | 169010600 | PDFSTATSENSEMBLUCSC | 312 | 5 | 32481 | tss | 0.020030 | 0.002805 | Not queried | 4.19 | 3.10e-01 | 3.66e-01 |
|  | 1 | 45082704 | 45083278 | PDFSTATSENSEMBLUCSC | 575 | 7 | 491 | gene;island | 0.846733 | 0.983987 | RNF220 (margin: RNF220) | 4.19 | 4.08e-03 | 2.47e-02 |
|  | 1 | 24513473 | 24513722 | PDFSTATSENSEMBLUCSC | 250 | 4 | 325 | gene | 0.040792 | 0.502631 | Not queried | 4.19 | 2.86e-02 | 6.12e-02 |
|  | 1 | 26372718 | 26373407 | PDFSTATSENSEMBLUCSC | 690 | 7 | 1381 | tss;island | 0.592055 | 0.974815 | SLC30A2 (margin: EXTL1; SLC30A2; TRIM63) | 4.19 | 6.99e-03 | 3.19e-02 |
|  | 2 | 60780415 | 60780483 | PDFSTATSENSEMBLUCSC | 69 | 4 | 22694 | gene;island | 0.269624 | 0.858503 | Not queried | 4.19 | 2.86e-02 | 6.12e-02 |
|  | 7 | 4783964 | 4784485 | PDFSTATSENSEMBLUCSC | 522 | 6 | 36845 | gene;island | 0.203448 | 0.947665 | Not queried | 4.19 | 1.80e-01 | 2.45e-01 |
|  | 11 | 3663491 | 3663842 | PDFSTATSENSEMBLUCSC | 352 | 9 | 7096 | tss | 0.320098 | 0.023018 | ART5 (margin: TRPC2; ART1; ART5) | 4.19 | 4.11e-05 | 1.17e-03 |
|  | 11 | 3663198 | 3663842 | PDFSTATSENSEMBLUCSC | 645 | 11 | 8000 | island | 0.327204 | 0.023018 | ART5 (margin: TRPC2; ART1; ART5) | 4.19 | 2.75e-04 | 4.46e-03 |
|  | 9 | 133589177 | 133589654 | PDFSTATSENSEMBLUCSC | 478 | 4 | 40961 | island | 0.004333 | 0.374221 | Not queried | 4.19 | 1.14e-01 | 1.68e-01 |
|  | 19 | 19383613 | 19383976 | PDFSTATSENSEMBLUCSC | 364 | 4 | 19949 | gene | 0.692973 | 0.972214 | Not queried | 4.19 | 2.86e-02 | 6.12e-02 |
|  | 1 | 214152709 | 214153036 | PDFSTATSENSEMBLUCSC | 328 | 5 | 4193 | island | 0.756584 | 0.971857 | Not queried | 4.18 | 1.59e-02 | 5.36e-02 |
|  | 8 | 144069457 | 144069888 | PDFSTATSENSEMBLUCSC | 432 | 6 | 39470 | gene | 0.820375 | 0.137814 | RP11-273G15.2 (margin: CDC42P3; RP11-273G15.2) | 4.18 | 4.33e-03 | 2.52e-02 |
|  | 22 | 43116932 | 43117318 | PDFSTATSENSEMBLUCSC | 387 | 8 | 27197 | tss;island | 0.138622 | 0.012595 | Not queried | 4.18 | 2.81e-02 | 6.12e-02 |
|  | 4 | 81186795 | 81187198 | PDFSTATSENSEMBLUCSC | 404 | 5 | 30461 | tss;island | 0.641858 | 0.970005 | FGF5 (margin: FGF5) | 4.18 | 7.94e-03 | 3.19e-02 |
|  | 1 | 156211409 | 156211896 | PDFSTATSENSEMBLUCSC | 488 | 10 | 2011 | tss | 0.427327 | 0.891505 | PMF1-BGLAP; PMF1; BGLAP; PAQR6 (margin: PMF1-BGLAP; PMF1; BGLAP; PAQR6; SMG5) | 4.18 | 1.05e-03 | 1.08e-02 |
|  | 17 | 17184602 | 17185060 | PDFSTATSENSEMBLUCSC | 459 | 10 | 17164 | tss | 0.029192 | 0.005195 | Not queried | 4.18 | 3.15e-01 | 3.72e-01 |
|  | 14 | 38068553 | 38069206 | PDFSTATSENSEMBLUCSC | 654 | 9 | 12798 | island | 0.918635 | 0.286835 | TTC6; FOXA1 (margin: TTC6; FOXA1) | 4.18 | 4.11e-05 | 1.17e-03 |
|  | 10 | 131665838 | 131666314 | PDFSTATSENSEMBLUCSC | 477 | 6 | 4831 | gene;island | 0.229827 | 0.929845 | Not queried | 4.18 | 4.11e-02 | 8.14e-02 |
|  | 6 | 10886946 | 10887367 | PDFSTATSENSEMBLUCSC | 422 | 9 | 35442 | island | 0.510862 | 0.974016 | Not queried | 4.18 | 1.88e-02 | 6.12e-02 |
|  | 12 | 45270207 | 45270896 | PDFSTATSENSEMBLUCSC | 690 | 18 | 9101 | gene;tss;island | 0.247954 | 0.019714 | NELL2 (margin: NELL2; Y\_RNA) | 4.18 | 8.22e-08 | 1.43e-05 |
|  | 5 | 140613740 | 140613884 | PDFSTATSENSEMBLUCSC | 145 | 5 | 32368 | tss | 0.636754 | 0.967726 | Not queried | 4.18 | 9.52e-02 | 1.53e-01 |
|  | 10 | 135020656 | 135021100 | PDFSTATSENSEMBLUCSC | 445 | 5 | 4891 | gene;island | 0.392066 | 0.920561 | KNDC1 (margin: KNDC1) | 4.17 | 7.94e-03 | 3.19e-02 |
|  | 3 | 137484220 | 137484586 | PDFSTATSENSEMBLUCSC | 367 | 4 | 29513 | island | 0.286571 | 0.880246 | Not queried | 4.17 | 2.86e-02 | 6.12e-02 |
|  | 19 | 10397429 | 10397780 | PDFSTATSENSEMBLUCSC | 352 | 6 | 21526 | island | 0.420492 | 0.937435 | Not queried | 4.17 | 4.11e-02 | 8.14e-02 |
|  | 12 | 118313657 | 118313990 | PDFSTATSENSEMBLUCSC | 334 | 5 | 9339 | gene | 0.639577 | 0.980522 | KSR2 (margin: KSR2) | 4.17 | 7.94e-03 | 3.19e-02 |
|  | 12 | 52257893 | 52258264 | PDFSTATSENSEMBLUCSC | 372 | 4 | 10431 | island | 0.509410 | 0.961723 | Not queried | 4.17 | 2.86e-02 | 6.12e-02 |
|  | 5 | 1225074 | 1225719 | PDFSTATSENSEMBLUCSC | 646 | 10 | 32616 | island | 0.253550 | 0.900277 | SLC6A19; SLC6A18 (margin: SLC6A19; SLC6A18) | 4.17 | 4.87e-04 | 6.47e-03 |
|  | 4 | 122854168 | 122854532 | PDFSTATSENSEMBLUCSC | 365 | 5 | 30118 | gene;island | 0.711815 | 0.984040 | Not queried | 4.17 | 9.52e-02 | 1.53e-01 |
|  | 16 | 1198408 | 1198900 | PDFSTATSENSEMBLUCSC | 493 | 5 | 15518 | island | 0.521614 | 0.962883 | Not queried | 4.17 | 3.10e-01 | 3.66e-01 |
|  | 20 | 61201022 | 61201271 | PDFSTATSENSEMBLUCSC | 250 | 4 | 26328 | island | 0.622393 | 0.967011 | Not queried | 4.17 | 2.86e-02 | 6.12e-02 |
|  | 10 | 103599520 | 103599757 | PDFSTATSENSEMBLUCSC | 238 | 6 | 4731 | gene;island | 0.158662 | 0.016419 | Not queried | 4.17 | 2.60e-02 | 6.12e-02 |
|  | 1 | 14075362 | 14075560 | PDFSTATSENSEMBLUCSC | 199 | 5 | 237 | gene;tss;island | 0.032876 | 0.002599 | Not queried | 4.17 | 9.52e-02 | 1.53e-01 |
|  | 2 | 27008234 | 27008453 | PDFSTATSENSEMBLUCSC | 220 | 4 | 23246 | tss;island | 0.549113 | 0.044955 | Not queried | 4.17 | 1.14e-01 | 1.68e-01 |
|  | 7 | 30029717 | 30029938 | PDFSTATSENSEMBLUCSC | 222 | 7 | 37514 | tss;island | 0.085127 | 0.543670 | AC007285.6; SCRN1 (margin: AC007285.6; SCRN1) | 4.17 | 1.11e-02 | 4.14e-02 |
|  | 5 | 140305947 | 140306458 | PDFSTATSENSEMBLUCSC | 512 | 10 | 31804 | gene;island | 0.957368 | 0.508699 | PCDHA1; PCDHA2; PCDHA3; PCDHA4; PCDHA5; PCDHA6; PCDHA7; PCDHA8; PCDHA9; PCDHA10; PCDHA11; PCDHA12; PCDHA13; PCDHAC1 (margin: PCDHA1; PCDHA2; PCDHA3; PCDHA4; PCDHA5; PCDHA6; PCDHA7; PCDHA8; PCDHA9; PCDHA10; PCDHA11; PCDHA12; PCDHA13; PCDHAC1) | 4.16 | 7.58e-05 | 1.98e-03 |
|  | 11 | 76381687 | 76382149 | PDFSTATSENSEMBLUCSC | 463 | 7 | 7577 | tss;island | 0.584332 | 0.960984 | LRRC32 (margin: AP001189.4; RP11-672A2.3; LRRC32) | 4.16 | 2.33e-03 | 1.52e-02 |
|  | 2 | 14774786 | 14775199 | PDFSTATSENSEMBLUCSC | 414 | 6 | 22579 | gene;island | 0.566965 | 0.036494 | FAM84A; AC011897.1 (margin: AC011897.2; FAM84A; AC011897.1) | 4.16 | 8.66e-03 | 3.36e-02 |
|  | 19 | 12876846 | 12877188 | PDFSTATSENSEMBLUCSC | 343 | 4 | 19866 | gene;island | 0.477601 | 0.928397 | Not queried | 4.16 | 2.86e-02 | 6.12e-02 |
|  | 17 | 7239574 | 7239821 | PDFSTATSENSEMBLUCSC | 248 | 5 | 17076 | tss | 0.145905 | 0.856305 | Not queried | 4.16 | 1.51e-01 | 2.11e-01 |
|  | 21 | 34350407 | 34350934 | PDFSTATSENSEMBLUCSC | 528 | 6 | 26641 | island | 0.420518 | 0.966922 | Not queried | 4.16 | 1.52e-02 | 5.36e-02 |
|  | 19 | 19383613 | 19384165 | PDFSTATSENSEMBLUCSC | 553 | 5 | 21810 | island | 0.626232 | 0.969111 | AC138430.4; TM6SF2 (margin: HAPLN4; AC138430.4; TM6SF2; SUGP1) | 4.16 | 7.94e-03 | 3.19e-02 |
|  | 7 | 50344242 | 50344471 | PDFSTATSENSEMBLUCSC | 230 | 4 | 38490 | island | 0.570756 | 0.975722 | Not queried | 4.16 | 5.71e-02 | 1.00e-01 |
|  | 7 | 120968877 | 120969079 | PDFSTATSENSEMBLUCSC | 203 | 7 | 37861 | tss | 0.839002 | 0.198532 | WNT16 (margin: WNT16) | 4.16 | 5.83e-04 | 6.47e-03 |
|  | 7 | 87229552 | 87230344 | PDFSTATSENSEMBLUCSC | 793 | 9 | 37072 | gene;island | 0.376842 | 0.922394 | ABCB1 (margin: ABCB1) | 4.16 | 8.23e-05 | 2.06e-03 |
|  | 10 | 38691787 | 38691801 | PDFSTATSENSEMBLUCSC | 15 | 4 | 5037 | tss;island | 0.292888 | 0.023221 | Not queried | 4.15 | 3.43e-01 | 3.84e-01 |
|  | 7 | 157561593 | 157561925 | PDFSTATSENSEMBLUCSC | 333 | 5 | 37343 | gene;island | 0.101069 | 0.658640 | Not queried | 4.15 | 2.22e-01 | 2.76e-01 |
|  | 13 | 36705446 | 36705622 | PDFSTATSENSEMBLUCSC | 177 | 5 | 11665 | island | 0.107681 | 0.011493 | Not queried | 4.15 | 3.17e-02 | 6.50e-02 |
|  | 3 | 52569026 | 52569169 | PDFSTATSENSEMBLUCSC | 144 | 5 | 28384 | tss;island | 0.950715 | 0.272962 | Not queried | 4.15 | 1.51e-01 | 2.11e-01 |
|  | 20 | 2188420 | 2188663 | PDFSTATSENSEMBLUCSC | 244 | 4 | 25942 | island | 0.506616 | 0.065888 | Not queried | 4.15 | 5.71e-02 | 1.00e-01 |
|  | 12 | 49582842 | 49583112 | PDFSTATSENSEMBLUCSC | 271 | 8 | 10388 | island | 0.128358 | 0.009218 | TUBA1C; TUBA1A (margin: TUBA1C; TUBA1A) | 4.15 | 3.11e-04 | 4.77e-03 |
|  | 15 | 90030016 | 90030410 | PDFSTATSENSEMBLUCSC | 395 | 5 | 13469 | gene;island | 0.759100 | 0.152198 | Not queried | 4.15 | 3.10e-01 | 3.66e-01 |
|  | 6 | 166907748 | 166908071 | PDFSTATSENSEMBLUCSC | 324 | 4 | 34547 | gene | 0.451024 | 0.970550 | Not queried | 4.15 | 5.71e-02 | 1.00e-01 |
|  | 14 | 106066504 | 106067201 | PDFSTATSENSEMBLUCSC | 698 | 7 | 13245 | island | 0.159108 | 0.891265 | Not queried | 4.15 | 1.75e-02 | 5.79e-02 |
|  | 11 | 132934128 | 132934554 | PDFSTATSENSEMBLUCSC | 427 | 5 | 6983 | gene;island | 0.443470 | 0.047543 | Not queried | 4.14 | 1.59e-02 | 5.36e-02 |
|  | 1 | 3028230 | 3028577 | PDFSTATSENSEMBLUCSC | 348 | 5 | 99 | gene;island | 0.429390 | 0.930222 | Not queried | 4.14 | 9.52e-02 | 1.53e-01 |
|  | 19 | 8117875 | 8117966 | PDFSTATSENSEMBLUCSC | 92 | 4 | 21475 | island | 0.605825 | 0.941859 | Not queried | 4.14 | 3.43e-01 | 3.84e-01 |
|  | 5 | 2866767 | 2866877 | PDFSTATSENSEMBLUCSC | 111 | 4 | 32661 | island | 0.062985 | 0.453529 | Not queried | 4.14 | 2.86e-02 | 6.12e-02 |
|  | 6 | 29274521 | 29275337 | PDFSTATSENSEMBLUCSC | 817 | 12 | 33651 | gene | 0.069439 | 0.621216 | Not queried | 4.14 | 1.45e-02 | 5.34e-02 |
|  | 10 | 104211296 | 104211488 | PDFSTATSENSEMBLUCSC | 193 | 6 | 6089 | island | 0.047709 | 0.261696 | Not queried | 4.14 | 4.11e-02 | 8.14e-02 |
|  | 1 | 2537547 | 2537838 | PDFSTATSENSEMBLUCSC | 292 | 6 | 92 | gene;island | 0.456664 | 0.944579 | MMEL1 (margin: MMEL1) | 4.14 | 4.33e-03 | 2.52e-02 |
|  | 1 | 159174001 | 159174162 | PDFSTATSENSEMBLUCSC | 162 | 4 | 2029 | tss | 0.019431 | 0.343001 | Not queried | 4.14 | 5.71e-02 | 1.00e-01 |
|  | 5 | 79378709 | 79378955 | PDFSTATSENSEMBLUCSC | 247 | 4 | 31662 | gene | 0.297181 | 0.031626 | Not queried | 4.14 | 2.00e-01 | 2.55e-01 |
|  | 11 | 2481857 | 2482594 | PDFSTATSENSEMBLUCSC | 738 | 15 | 7076 | tss | 0.392645 | 0.943668 | KCNQ1 (margin: KCNQ1) | 4.14 | 3.51e-06 | 2.34e-04 |
|  | 6 | 132945515 | 132945592 | PDFSTATSENSEMBLUCSC | 78 | 4 | 35229 | tss | 0.781052 | 0.982353 | Not queried | 4.14 | 2.86e-02 | 6.12e-02 |
|  | 5 | 140501321 | 140501540 | PDFSTATSENSEMBLUCSC | 220 | 7 | 32362 | tss | 0.893420 | 0.417137 | Not queried | 4.14 | 7.28e-02 | 1.23e-01 |
|  | 7 | 103969166 | 103969483 | PDFSTATSENSEMBLUCSC | 318 | 4 | 37173 | gene;island | 0.886191 | 0.369818 | Not queried | 4.14 | 2.86e-02 | 6.12e-02 |
|  | 8 | 48100081 | 48100186 | PDFSTATSENSEMBLUCSC | 106 | 4 | 40269 | island | 0.590952 | 0.030089 | Not queried | 4.14 | 5.71e-02 | 1.00e-01 |
|  | 7 | 155604780 | 155605094 | PDFSTATSENSEMBLUCSC | 315 | 7 | 39077 | island | 0.167394 | 0.839157 | Not queried | 4.14 | 3.79e-02 | 7.66e-02 |
|  | 7 | 155604999 | 155605094 | PDFSTATSENSEMBLUCSC | 96 | 5 | 38014 | tss | 0.167394 | 0.548017 | Not queried | 4.14 | 2.22e-01 | 2.76e-01 |
|  | 4 | 3671067 | 3671697 | PDFSTATSENSEMBLUCSC | 631 | 5 | 30850 | island | 0.537742 | 0.911309 | Not queried | 4.14 | 3.17e-02 | 6.50e-02 |
|  | 11 | 75139390 | 75139736 | PDFSTATSENSEMBLUCSC | 347 | 4 | 6835 | gene;island | 0.946878 | 0.501229 | Not queried | 4.14 | 2.86e-02 | 6.12e-02 |
|  | 7 | 157366933 | 157367159 | PDFSTATSENSEMBLUCSC | 227 | 5 | 37328 | gene;island | 0.037128 | 0.512099 | Not queried | 4.14 | 9.52e-02 | 1.53e-01 |
|  | 1 | 22919873 | 22920169 | PDFSTATSENSEMBLUCSC | 297 | 4 | 307 | gene;island | 0.204701 | 0.677317 | Not queried | 4.14 | 2.86e-02 | 6.12e-02 |
|  | 5 | 140613740 | 140614106 | PDFSTATSENSEMBLUCSC | 367 | 8 | 33226 | island | 0.663060 | 0.873844 | Not queried | 4.14 | 3.79e-02 | 7.66e-02 |
|  | 10 | 119295619 | 119295875 | PDFSTATSENSEMBLUCSC | 257 | 4 | 4783 | gene;island | 0.730273 | 0.971668 | Not queried | 4.14 | 5.71e-02 | 1.00e-01 |
|  | 10 | 90750169 | 90750249 | PDFSTATSENSEMBLUCSC | 81 | 4 | 5209 | tss | 0.004849 | 0.038539 | Not queried | 4.13 | 2.86e-02 | 6.12e-02 |
|  | 6 | 31589593 | 31589994 | PDFSTATSENSEMBLUCSC | 402 | 5 | 33909 | gene;tss;island | 0.002315 | 0.051136 | Not queried | 4.13 | 5.56e-02 | 1.00e-01 |
|  | 7 | 34873912 | 34874196 | PDFSTATSENSEMBLUCSC | 285 | 4 | 36962 | gene | 0.308767 | 0.875864 | Not queried | 4.13 | 1.14e-01 | 1.68e-01 |
|  | 14 | 61116782 | 61117162 | PDFSTATSENSEMBLUCSC | 381 | 5 | 12440 | tss;island | 0.918779 | 0.393239 | Not queried | 4.13 | 9.52e-02 | 1.53e-01 |
|  | 3 | 179754521 | 179754761 | PDFSTATSENSEMBLUCSC | 241 | 7 | 28734 | tss | 0.932774 | 0.395108 | PEX5L (margin: PEX5L) | 4.13 | 6.99e-03 | 3.19e-02 |
|  | 1 | 7844735 | 7845237 | PDFSTATSENSEMBLUCSC | 503 | 5 | 2627 | island | 0.960233 | 0.494924 | Not queried | 4.13 | 4.21e-01 | 4.59e-01 |
|  | 8 | 10261641 | 10262221 | PDFSTATSENSEMBLUCSC | 581 | 6 | 39195 | gene;island | 0.470947 | 0.933013 | MSRA (margin: MSRA) | 4.13 | 4.33e-03 | 2.52e-02 |
|  | 6 | 168841257 | 168841653 | PDFSTATSENSEMBLUCSC | 397 | 6 | 35349 | tss;island | 0.840534 | 0.454673 | Not queried | 4.13 | 3.10e-01 | 3.66e-01 |
|  | 10 | 130012874 | 130013154 | PDFSTATSENSEMBLUCSC | 281 | 6 | 6254 | island | 0.296979 | 0.967009 | Not queried | 4.13 | 2.60e-02 | 6.12e-02 |
|  | 6 | 33134045 | 33134325 | PDFSTATSENSEMBLUCSC | 281 | 4 | 34196 | gene | 0.241754 | 0.667746 | Not queried | 4.13 | 2.86e-02 | 6.12e-02 |
|  | 5 | 41510325 | 41510851 | PDFSTATSENSEMBLUCSC | 527 | 7 | 32754 | island | 0.401197 | 0.036869 | PLCXD3 (margin: PLCXD3) | 4.13 | 5.83e-04 | 6.47e-03 |
|  | 6 | 150463775 | 150464124 | PDFSTATSENSEMBLUCSC | 350 | 7 | 35296 | tss | 0.050007 | 0.566628 | PPP1R14C (margin: PPP1R14C) | 4.13 | 4.08e-03 | 2.47e-02 |
|  | 14 | 80328091 | 80328595 | PDFSTATSENSEMBLUCSC | 505 | 5 | 12160 | gene;island | 0.803947 | 0.968638 | Not queried | 4.13 | 9.52e-02 | 1.53e-01 |
|  | 3 | 51740741 | 51740896 | PDFSTATSENSEMBLUCSC | 156 | 4 | 28366 | tss;island | 0.788656 | 0.238758 | Not queried | 4.12 | 2.86e-02 | 6.12e-02 |
|  | 5 | 3595728 | 3595977 | PDFSTATSENSEMBLUCSC | 250 | 4 | 31977 | tss;island | 0.381898 | 0.872246 | Not queried | 4.12 | 5.71e-02 | 1.00e-01 |
|  | 6 | 156718177 | 156718546 | PDFSTATSENSEMBLUCSC | 370 | 5 | 36589 | island | 0.587032 | 0.965090 | (margin: ) | 4.12 | 7.94e-03 | 3.19e-02 |
|  | 7 | 12250161 | 12250760 | PDFSTATSENSEMBLUCSC | 600 | 7 | 37443 | tss | 0.041720 | 0.005354 | Not queried | 4.12 | 1.75e-02 | 5.79e-02 |
|  | 19 | 51017123 | 51017331 | PDFSTATSENSEMBLUCSC | 209 | 4 | 20178 | gene;island | 0.264718 | 0.810652 | Not queried | 4.12 | 2.86e-02 | 6.12e-02 |
|  | 11 | 2183708 | 2183864 | PDFSTATSENSEMBLUCSC | 157 | 4 | 7062 | tss;island | 0.623135 | 0.974323 | Not queried | 4.12 | 2.86e-02 | 6.12e-02 |
|  | 2 | 154335348 | 154335386 | PDFSTATSENSEMBLUCSC | 39 | 5 | 23667 | tss | 0.081115 | 0.004012 | AC012501.2; RPRM (margin: AC012501.2; RPRM) | 4.12 | 7.94e-03 | 3.19e-02 |
|  | 5 | 79866319 | 79866431 | PDFSTATSENSEMBLUCSC | 113 | 4 | 32151 | tss;island | 0.631683 | 0.951191 | Not queried | 4.12 | 2.86e-02 | 6.12e-02 |
|  | 10 | 28034792 | 28034856 | PDFSTATSENSEMBLUCSC | 65 | 6 | 5004 | tss | 0.720930 | 0.975219 | RP11-360I20.2; MKX (margin: RP11-360I20.2; MKX) | 4.12 | 2.16e-03 | 1.45e-02 |
|  | 6 | 147829799 | 147830055 | PDFSTATSENSEMBLUCSC | 257 | 7 | 35279 | tss | 0.724120 | 0.984634 | SAMD5 (margin: SAMD5) | 4.12 | 5.83e-04 | 6.47e-03 |
|  | 7 | 100493710 | 100493849 | PDFSTATSENSEMBLUCSC | 140 | 4 | 37795 | tss | 0.312454 | 0.035689 | Not queried | 4.12 | 2.86e-02 | 6.12e-02 |
|  | 6 | 28602543 | 28603437 | PDFSTATSENSEMBLUCSC | 895 | 35 | 35593 | island | 0.554668 | 0.977224 | RP11-373N24.2 (margin: RP11-373N24.2) | 4.11 | 5.28e-12 | 6.43e-09 |
|  | 19 | 18548845 | 18549274 | PDFSTATSENSEMBLUCSC | 430 | 8 | 21779 | island | 0.461350 | 0.044994 | ISYNA1 (margin: SSBP4; CTD-3137H5.1; ISYNA1; ELL; AC010335.1) | 4.11 | 1.86e-03 | 1.45e-02 |
|  | 7 | 48019205 | 48019713 | PDFSTATSENSEMBLUCSC | 509 | 5 | 38481 | island | 0.638865 | 0.019334 | Not queried | 4.11 | 4.21e-01 | 4.59e-01 |
|  | 8 | 144635260 | 144635610 | PDFSTATSENSEMBLUCSC | 351 | 10 | 40668 | island | 0.546792 | 0.954589 | GSDMD (margin: 7SK; GSDMD; ZC3H3; RP11-661A12.5) | 4.11 | 1.08e-05 | 4.82e-04 |
|  | 5 | 5287980 | 5288296 | PDFSTATSENSEMBLUCSC | 317 | 6 | 31552 | gene | 0.961613 | 0.357514 | Not queried | 4.11 | 2.60e-02 | 6.12e-02 |
|  | 7 | 145813413 | 145813439 | PDFSTATSENSEMBLUCSC | 27 | 6 | 37959 | tss | 0.946067 | 0.518228 | CNTNAP2 (margin: CNTNAP2) | 4.11 | 2.16e-03 | 1.45e-02 |
|  | 19 | 47217554 | 47218031 | PDFSTATSENSEMBLUCSC | 478 | 6 | 20112 | gene;island | 0.013729 | 0.206649 | PRKD2 (margin: PRKD2; MIR320E; STRN4) | 4.11 | 8.66e-03 | 3.36e-02 |
|  | 15 | 25318197 | 25318384 | PDFSTATSENSEMBLUCSC | 188 | 4 | 13523 | tss | 0.539620 | 0.068514 | Not queried | 4.11 | 2.00e-01 | 2.55e-01 |
|  | 6 | 137241923 | 137242316 | PDFSTATSENSEMBLUCSC | 394 | 5 | 35245 | tss;island | 0.795848 | 0.983993 | SLC35D3 (margin: PEX7; SLC35D3) | 4.11 | 7.94e-03 | 3.19e-02 |
|  | 17 | 46692347 | 46692592 | PDFSTATSENSEMBLUCSC | 246 | 7 | 17467 | tss | 0.930230 | 0.497616 | Not queried | 4.11 | 7.28e-02 | 1.23e-01 |
|  | 12 | 129338783 | 129338939 | PDFSTATSENSEMBLUCSC | 157 | 4 | 9411 | gene;island | 0.700001 | 0.972301 | Not queried | 4.11 | 2.86e-02 | 6.12e-02 |
|  | 8 | 144503255 | 144503586 | PDFSTATSENSEMBLUCSC | 332 | 4 | 40661 | island | 0.417825 | 0.796023 | Not queried | 4.11 | 2.86e-02 | 6.12e-02 |
|  | 6 | 28983079 | 28983178 | PDFSTATSENSEMBLUCSC | 100 | 4 | 35609 | island | 0.930261 | 0.484910 | Not queried | 4.11 | 2.86e-02 | 6.12e-02 |
|  | 10 | 61122307 | 61122716 | PDFSTATSENSEMBLUCSC | 410 | 10 | 5762 | island | 0.112351 | 0.006552 | FAM13C (margin: FAM13C) | 4.11 | 1.05e-03 | 1.08e-02 |
|  | 20 | 33146263 | 33146543 | PDFSTATSENSEMBLUCSC | 281 | 6 | 25400 | gene;island | 0.714251 | 0.975221 | MAP1LC3A; PIGU (margin: MAP1LC3A; PIGU) | 4.11 | 2.16e-03 | 1.45e-02 |
|  | 20 | 33146263 | 33146515 | PDFSTATSENSEMBLUCSC | 253 | 4 | 25693 | tss | 0.714251 | 0.975221 | Not queried | 4.11 | 2.86e-02 | 6.12e-02 |
|  | 7 | 155325552 | 155326102 | PDFSTATSENSEMBLUCSC | 551 | 6 | 37310 | gene;island | 0.554140 | 0.951147 | Not queried | 4.11 | 4.11e-02 | 8.14e-02 |
|  | 5 | 127871927 | 127872329 | PDFSTATSENSEMBLUCSC | 403 | 5 | 31738 | gene;island | 0.856779 | 0.990043 | Not queried | 4.10 | 1.59e-02 | 5.36e-02 |
|  | 1 | 27113508 | 27113753 | PDFSTATSENSEMBLUCSC | 246 | 6 | 1396 | tss;island | 0.025221 | 0.319160 | PIGV (margin: ARID1A; PIGV) | 4.10 | 2.16e-03 | 1.45e-02 |
|  | 13 | 28365587 | 28365853 | PDFSTATSENSEMBLUCSC | 267 | 4 | 11363 | tss;island | 0.944111 | 0.486200 | Not queried | 4.10 | 3.43e-01 | 3.84e-01 |
|  | 2 | 9614471 | 9614578 | PDFSTATSENSEMBLUCSC | 108 | 5 | 23173 | tss | 0.050425 | 0.477131 | Not queried | 4.10 | 1.51e-01 | 2.11e-01 |
|  | 6 | 29394438 | 29395425 | PDFSTATSENSEMBLUCSC | 988 | 12 | 33655 | gene | 0.083188 | 0.704719 | OR5V1; OR11A1 (margin: OR12D1P; OR10C1; OR5V1; OR11A1) | 4.10 | 3.71e-04 | 5.59e-03 |
|  | 5 | 148520669 | 148520934 | PDFSTATSENSEMBLUCSC | 266 | 7 | 32414 | tss | 0.121453 | 0.698751 | Not queried | 4.10 | 7.28e-02 | 1.23e-01 |
|  | 5 | 1645268 | 1645802 | PDFSTATSENSEMBLUCSC | 535 | 6 | 32638 | island | 0.472482 | 0.967476 | Not queried | 4.10 | 6.49e-02 | 1.11e-01 |
|  | 13 | 112760898 | 112761328 | PDFSTATSENSEMBLUCSC | 431 | 6 | 11916 | island | 0.566720 | 0.969552 | LINC00403; LINC00404 (margin: LINC00403; LINC00404) | 4.10 | 2.16e-03 | 1.45e-02 |
|  | 12 | 131568441 | 131568764 | PDFSTATSENSEMBLUCSC | 324 | 5 | 9424 | gene | 0.322403 | 0.984990 | Not queried | 4.10 | 5.56e-02 | 1.00e-01 |
|  | 15 | 90456248 | 90456686 | PDFSTATSENSEMBLUCSC | 439 | 5 | 13894 | tss | 0.556682 | 0.653906 | Not queried | 4.10 | 4.21e-01 | 4.59e-01 |
|  | 20 | 30060822 | 30061044 | PDFSTATSENSEMBLUCSC | 223 | 5 | 25658 | tss;island | 0.282652 | 0.870410 | DEFB124 (margin: REM1; DEFB124) | 4.10 | 7.94e-03 | 3.19e-02 |
|  | 22 | 38071455 | 38071677 | PDFSTATSENSEMBLUCSC | 223 | 7 | 27468 | island | 0.019314 | 0.225449 | LGALS1 (margin: SH3BP1; PDXP; LGALS1; NOL12; RP1-37E16.12; RN7SL385P) | 4.10 | 5.83e-04 | 6.47e-03 |
|  | 13 | 112322853 | 112323195 | PDFSTATSENSEMBLUCSC | 343 | 4 | 11902 | island | 0.684376 | 0.971983 | Not queried | 4.10 | 5.71e-02 | 1.00e-01 |
|  | 2 | 27346296 | 27346593 | PDFSTATSENSEMBLUCSC | 298 | 5 | 23255 | tss | 0.119065 | 0.012414 | Not queried | 4.10 | 9.52e-02 | 1.53e-01 |
|  | 10 | 13933996 | 13934438 | PDFSTATSENSEMBLUCSC | 443 | 5 | 4505 | gene;island | 0.557331 | 0.950449 | FRMD4A (margin: FRMD4A) | 4.10 | 7.94e-03 | 3.19e-02 |
|  | 18 | 5237527 | 5237922 | PDFSTATSENSEMBLUCSC | 396 | 5 | 19047 | gene;tss;island | 0.481615 | 0.939650 | Not queried | 4.10 | 1.51e-01 | 2.11e-01 |
|  | 1 | 50574383 | 50574591 | PDFSTATSENSEMBLUCSC | 209 | 4 | 524 | gene;tss | 0.080835 | 0.470556 | Not queried | 4.09 | 2.86e-02 | 6.12e-02 |
|  | 12 | 12849136 | 12849748 | PDFSTATSENSEMBLUCSC | 613 | 8 | 9573 | tss | 0.091486 | 0.006824 | GPR19 (margin: RP11-180M15.3; GPR19) | 4.09 | 1.04e-02 | 3.96e-02 |
|  | 17 | 79131893 | 79132083 | PDFSTATSENSEMBLUCSC | 191 | 4 | 16899 | gene;island | 0.601297 | 0.965709 | Not queried | 4.09 | 2.86e-02 | 6.12e-02 |
|  | 12 | 115134148 | 115136308 | PDFSTATSENSEMBLUCSC | 2161 | 54 | 10850 | island | 0.614152 | 0.980693 | (margin: ) | 4.09 | 1.12e-13 | 2.74e-10 |
|  | 3 | 44626492 | 44626860 | PDFSTATSENSEMBLUCSC | 369 | 4 | 27740 | gene | 0.536345 | 0.961866 | Not queried | 4.09 | 2.00e-01 | 2.55e-01 |
|  | 6 | 146349312 | 146349638 | PDFSTATSENSEMBLUCSC | 327 | 4 | 34499 | gene;island | 0.657892 | 0.053233 | Not queried | 4.09 | 3.43e-01 | 3.84e-01 |
|  | 17 | 26732836 | 26733068 | PDFSTATSENSEMBLUCSC | 233 | 4 | 16497 | gene;island | 0.316765 | 0.029050 | Not queried | 4.09 | 2.86e-02 | 6.12e-02 |
|  | 10 | 2815004 | 2815547 | PDFSTATSENSEMBLUCSC | 544 | 6 | 5502 | island | 0.373329 | 0.080611 | Not queried | 4.09 | 6.49e-02 | 1.11e-01 |
|  | 7 | 149411256 | 149411652 | PDFSTATSENSEMBLUCSC | 397 | 4 | 37970 | tss;island | 0.855219 | 0.346561 | Not queried | 4.09 | 3.43e-01 | 3.84e-01 |
|  | 6 | 108436115 | 108436519 | PDFSTATSENSEMBLUCSC | 405 | 4 | 36390 | island | 0.535820 | 0.958674 | Not queried | 4.09 | 2.86e-02 | 6.12e-02 |
|  | 10 | 119312691 | 119313075 | PDFSTATSENSEMBLUCSC | 385 | 4 | 6183 | island | 0.500578 | 0.947055 | Not queried | 4.09 | 1.14e-01 | 1.68e-01 |
|  | 6 | 32184296 | 32184498 | PDFSTATSENSEMBLUCSC | 203 | 4 | 34095 | gene | 0.472773 | 0.936729 | Not queried | 4.09 | 4.86e-01 | 5.11e-01 |
|  | 17 | 72438885 | 72439217 | PDFSTATSENSEMBLUCSC | 333 | 4 | 16757 | gene | 0.111281 | 0.492698 | Not queried | 4.09 | 2.86e-02 | 6.12e-02 |
|  | 1 | 207818395 | 207818449 | PDFSTATSENSEMBLUCSC | 55 | 4 | 2224 | tss | 0.540199 | 0.925013 | Not queried | 4.09 | 5.71e-02 | 1.00e-01 |
|  | 10 | 105212056 | 105212813 | PDFSTATSENSEMBLUCSC | 758 | 12 | 6102 | island | 0.598580 | 0.197777 | RP11-225H22.4; CALHM2; CALHM1 (margin: PDCD11; RP11-225H22.4; CALHM2; CALHM1) | 4.09 | 2.01e-04 | 3.38e-03 |
|  | 19 | 18548929 | 18549274 | PDFSTATSENSEMBLUCSC | 346 | 7 | 20619 | tss | 0.511873 | 0.047746 | ISYNA1 (margin: SSBP4; CTD-3137H5.1; ISYNA1; ELL; AC010335.1) | 4.09 | 6.99e-03 | 3.19e-02 |
|  | 1 | 165325498 | 165325833 | PDFSTATSENSEMBLUCSC | 336 | 4 | 2064 | tss;island | 0.531037 | 0.952964 | Not queried | 4.09 | 2.86e-02 | 6.12e-02 |
|  | 19 | 19971578 | 19972043 | PDFSTATSENSEMBLUCSC | 466 | 4 | 21829 | island | 0.255976 | 0.796331 | Not queried | 4.08 | 2.86e-02 | 6.12e-02 |
|  | 2 | 45237689 | 45237929 | PDFSTATSENSEMBLUCSC | 241 | 4 | 23335 | tss;island | 0.941819 | 0.492475 | Not queried | 4.08 | 2.86e-02 | 6.12e-02 |
|  | 12 | 133186599 | 133187174 | PDFSTATSENSEMBLUCSC | 576 | 5 | 11066 | island | 0.497379 | 0.984490 | Not queried | 4.08 | 9.52e-02 | 1.53e-01 |
|  | 11 | 66034681 | 66036138 | PDFSTATSENSEMBLUCSC | 1458 | 58 | 8500 | island | 0.282802 | 0.009742 | KLC2; RAB1B; RP11-867G23.1; RP11-867G23.2; RP11-867G23.3 (margin: KLC2; RAB1B; CNIH2; RP11-755F10.3; RP11-867G23.1; RP11-867G23.2; RP11-867G23.3; RP11-867G23.4) | 4.08 | 4.18e-06 | 2.75e-04 |
|  | 7 | 73062083 | 73062573 | PDFSTATSENSEMBLUCSC | 491 | 5 | 38562 | island | 0.028042 | 0.365765 | (margin: ) | 4.08 | 7.94e-03 | 3.19e-02 |
|  | 8 | 134202337 | 134202370 | PDFSTATSENSEMBLUCSC | 34 | 4 | 39921 | tss | 0.935156 | 0.316679 | Not queried | 4.08 | 2.86e-02 | 6.12e-02 |
|  | 6 | 31431002 | 31431100 | PDFSTATSENSEMBLUCSC | 99 | 4 | 33876 | gene | 0.008223 | 0.110806 | Not queried | 4.08 | 1.14e-01 | 1.68e-01 |
|  | 8 | 70745342 | 70745926 | PDFSTATSENSEMBLUCSC | 585 | 7 | 39331 | gene;island | 0.695530 | 0.168518 | Not queried | 4.08 | 5.30e-02 | 1.00e-01 |
|  | 1 | 44398868 | 44399069 | PDFSTATSENSEMBLUCSC | 202 | 4 | 3172 | island | 0.067349 | 0.493899 | Not queried | 4.08 | 2.86e-02 | 6.12e-02 |
|  | 6 | 6546777 | 6547273 | PDFSTATSENSEMBLUCSC | 497 | 5 | 33547 | gene;island | 0.497510 | 0.940942 | LY86-AS1 (margin: CNN3P1; LY86-AS1) | 4.08 | 7.94e-03 | 3.19e-02 |
|  | 15 | 69590852 | 69591357 | PDFSTATSENSEMBLUCSC | 506 | 9 | 14264 | island | 0.051520 | 0.475066 | Not queried | 4.08 | 4.00e-02 | 8.03e-02 |
|  | 22 | 39712956 | 39713086 | PDFSTATSENSEMBLUCSC | 131 | 6 | 26876 | gene;tss;island | 0.063532 | 0.530907 | RPL3; SNORD83A; SNORD43 (margin: RPL3; SNORD83B; SNORD83A; SNORD43) | 4.08 | 2.16e-03 | 1.45e-02 |
|  | 11 | 123066108 | 123066252 | PDFSTATSENSEMBLUCSC | 145 | 4 | 7777 | tss;island | 0.021309 | 0.472138 | Not queried | 4.07 | 3.43e-01 | 3.84e-01 |
|  | 20 | 30073209 | 30073576 | PDFSTATSENSEMBLUCSC | 368 | 6 | 25660 | tss;island | 0.496932 | 0.852025 | REM1; LINC00028 (margin: REM1; LINC00028; DEFB124) | 4.07 | 4.33e-03 | 2.52e-02 |
|  | 10 | 636076 | 636390 | PDFSTATSENSEMBLUCSC | 315 | 5 | 4444 | gene | 0.239941 | 0.951153 | Not queried | 4.07 | 9.52e-02 | 1.53e-01 |
|  | 1 | 23504146 | 23504632 | PDFSTATSENSEMBLUCSC | 487 | 6 | 2830 | island | 0.086265 | 0.478604 | LUZP1 (margin: LUZP1) | 4.07 | 2.16e-03 | 1.45e-02 |
|  | 4 | 96761264 | 96761796 | PDFSTATSENSEMBLUCSC | 533 | 5 | 30064 | gene | 0.561249 | 0.968082 | Not queried | 4.07 | 3.17e-02 | 6.50e-02 |
|  | 20 | 42875645 | 42876091 | PDFSTATSENSEMBLUCSC | 447 | 9 | 26180 | island | 0.579934 | 0.912764 | GDAP1L1 (margin: GDAP1L1) | 4.07 | 1.23e-03 | 1.18e-02 |
|  | 22 | 44319257 | 44319579 | PDFSTATSENSEMBLUCSC | 323 | 4 | 27207 | tss | 0.324006 | 0.007669 | Not queried | 4.07 | 2.00e-01 | 2.55e-01 |
|  | 4 | 1496822 | 1497223 | PDFSTATSENSEMBLUCSC | 402 | 5 | 30787 | island | 0.164471 | 0.635062 | Not queried | 4.06 | 9.52e-02 | 1.53e-01 |
|  | 13 | 113783975 | 113784438 | PDFSTATSENSEMBLUCSC | 464 | 4 | 11276 | gene;island | 0.746363 | 0.979242 | Not queried | 4.06 | 2.86e-02 | 6.12e-02 |
|  | 2 | 37571677 | 37571901 | PDFSTATSENSEMBLUCSC | 225 | 6 | 24223 | island | 0.973969 | 0.653280 | QPCT (margin: QPCT) | 4.06 | 2.16e-03 | 1.45e-02 |
|  | 1 | 3162066 | 3162173 | PDFSTATSENSEMBLUCSC | 108 | 4 | 112 | gene;island | 0.077926 | 0.558057 | Not queried | 4.06 | 5.71e-02 | 1.00e-01 |
|  | 12 | 40499811 | 40500139 | PDFSTATSENSEMBLUCSC | 329 | 4 | 9639 | tss | 0.109140 | 0.019461 | Not queried | 4.06 | 1.14e-01 | 1.68e-01 |
|  | 10 | 124713989 | 124714384 | PDFSTATSENSEMBLUCSC | 396 | 8 | 5412 | tss | 0.619593 | 0.153328 | PSTK; C10orf88 (margin: PSTK; C10orf88) | 4.06 | 1.04e-02 | 3.96e-02 |
|  | 7 | 2738728 | 2738961 | PDFSTATSENSEMBLUCSC | 234 | 4 | 36812 | gene;island | 0.405046 | 0.858781 | Not queried | 4.06 | 2.86e-02 | 6.12e-02 |
|  | 19 | 53636226 | 53636398 | PDFSTATSENSEMBLUCSC | 173 | 5 | 21023 | tss | 0.217989 | 0.951707 | ZNF415; ZNF347; CTD-2620I22.7 (margin: ZNF415; ZNF347; CTD-2620I22.7) | 4.06 | 7.94e-03 | 3.19e-02 |
|  | 22 | 29711916 | 29712139 | PDFSTATSENSEMBLUCSC | 224 | 6 | 27047 | tss;island | 0.696767 | 0.983027 | RASL10A (margin: GAS2L1; RASL10A; AP1B1) | 4.06 | 8.66e-03 | 3.36e-02 |
|  | 16 | 67562424 | 67562672 | PDFSTATSENSEMBLUCSC | 249 | 4 | 15305 | tss;island | 0.469622 | 0.066889 | Not queried | 4.06 | 2.86e-02 | 6.12e-02 |
|  | 7 | 45197251 | 45197346 | PDFSTATSENSEMBLUCSC | 96 | 4 | 37583 | tss | 0.064147 | 0.549092 | Not queried | 4.06 | 1.14e-01 | 1.68e-01 |
|  | 1 | 165204745 | 165205506 | PDFSTATSENSEMBLUCSC | 762 | 12 | 863 | gene;island | 0.558146 | 0.956989 | LMX1A (margin: LMX1A) | 4.06 | 1.03e-04 | 2.47e-03 |
|  | 1 | 116710227 | 116710606 | PDFSTATSENSEMBLUCSC | 380 | 4 | 3637 | island | 0.949778 | 0.562588 | Not queried | 4.06 | 2.86e-02 | 6.12e-02 |
|  | 6 | 35744188 | 35744355 | PDFSTATSENSEMBLUCSC | 168 | 4 | 34931 | tss;island | 0.540173 | 0.058383 | Not queried | 4.06 | 1.14e-01 | 1.68e-01 |
|  | 7 | 4167809 | 4168039 | PDFSTATSENSEMBLUCSC | 231 | 4 | 36828 | gene;island | 0.216039 | 0.795360 | Not queried | 4.05 | 2.86e-02 | 6.12e-02 |
|  | 17 | 71431640 | 71431877 | PDFSTATSENSEMBLUCSC | 238 | 4 | 16746 | gene;island | 0.555274 | 0.968528 | Not queried | 4.05 | 2.00e-01 | 2.55e-01 |
|  | 5 | 114516315 | 114516422 | PDFSTATSENSEMBLUCSC | 108 | 4 | 32223 | tss | 0.736075 | 0.978205 | Not queried | 4.05 | 1.14e-01 | 1.68e-01 |
|  | 11 | 132948042 | 132948244 | PDFSTATSENSEMBLUCSC | 203 | 4 | 6984 | gene;island | 0.502652 | 0.130192 | Not queried | 4.05 | 2.00e-01 | 2.55e-01 |
|  | 8 | 26481666 | 26481822 | PDFSTATSENSEMBLUCSC | 157 | 4 | 39252 | gene | 0.418186 | 0.961988 | Not queried | 4.05 | 2.86e-02 | 6.12e-02 |
|  | 17 | 54675001 | 54675231 | PDFSTATSENSEMBLUCSC | 231 | 4 | 18578 | island | 0.614140 | 0.974236 | Not queried | 4.05 | 5.71e-02 | 1.00e-01 |
|  | 10 | 26726877 | 26727318 | PDFSTATSENSEMBLUCSC | 442 | 8 | 5619 | island | 0.491990 | 0.933286 | APBB1IP (margin: APBB1IP) | 4.05 | 6.22e-04 | 6.71e-03 |
|  | 20 | 57425979 | 57428032 | PDFSTATSENSEMBLUCSC | 2054 | 66 | 25471 | gene;tss;island | 0.490360 | 0.950384 | GNAS; GNAS-AS1 (margin: GNAS; GNAS-AS1; RP1-309F20.3) | 4.05 | 3.02e-20 | 2.94e-16 |
|  | 2 | 176962658 | 176962928 | PDFSTATSENSEMBLUCSC | 271 | 4 | 24893 | island | 0.286427 | 0.036603 | Not queried | 4.05 | 4.86e-01 | 5.11e-01 |
|  | 1 | 166134182 | 166134699 | PDFSTATSENSEMBLUCSC | 518 | 6 | 868 | gene;island | 0.862911 | 0.986302 | Not queried | 4.05 | 2.60e-02 | 6.12e-02 |
|  | 12 | 79257496 | 79257853 | PDFSTATSENSEMBLUCSC | 358 | 6 | 9890 | tss | 0.284925 | 0.016648 | SYT1 (margin: SYT1) | 4.05 | 4.33e-03 | 2.52e-02 |
|  | 13 | 113652069 | 113652368 | PDFSTATSENSEMBLUCSC | 300 | 5 | 11266 | gene;island | 0.601177 | 0.954141 | MCF2L (margin: MCF2L; RP11-120K24.4; RP11-120K24.5) | 4.05 | 7.94e-03 | 3.19e-02 |
|  | 14 | 96730482 | 96730780 | PDFSTATSENSEMBLUCSC | 299 | 4 | 12199 | gene | 0.925502 | 0.449188 | Not queried | 4.05 | 1.14e-01 | 1.68e-01 |
|  | 3 | 8543014 | 8543732 | PDFSTATSENSEMBLUCSC | 719 | 10 | 28849 | island | 0.132628 | 0.007899 | LMCD1; LMCD1-AS1 (margin: LMCD1; LMCD1-AS1) | 4.05 | 8.93e-03 | 3.45e-02 |
|  | 3 | 112051808 | 112051991 | PDFSTATSENSEMBLUCSC | 184 | 4 | 29358 | island | 0.442048 | 0.871836 | Not queried | 4.04 | 2.00e-01 | 2.55e-01 |
|  | 10 | 103599617 | 103599757 | PDFSTATSENSEMBLUCSC | 141 | 5 | 5311 | tss | 0.142143 | 0.012629 | Not queried | 4.04 | 9.52e-02 | 1.53e-01 |
|  | 6 | 33091111 | 33092243 | PDFSTATSENSEMBLUCSC | 1133 | 20 | 34190 | gene | 0.247999 | 0.771632 | Not queried | 4.04 | 2.63e-02 | 6.12e-02 |
|  | 17 | 1956958 | 1958478 | PDFSTATSENSEMBLUCSC | 1521 | 45 | 17001 | tss;island | 0.423165 | 0.020299 | HIC1 (margin: DPH1; OVCA2; HIC1; RP11-667K14.4; RP11-667K14.3; MIR132; MIR212; SMG6) | 4.04 | 9.96e-11 | 6.93e-08 |
|  | 17 | 1553202 | 1553661 | PDFSTATSENSEMBLUCSC | 460 | 9 | 17822 | island | 0.029863 | 0.331256 | Not queried | 4.04 | 2.44e-02 | 6.12e-02 |
|  | 12 | 4554295 | 4554756 | PDFSTATSENSEMBLUCSC | 462 | 6 | 9016 | gene | 0.179440 | 0.793615 | FGF6 (margin: FGF6) | 4.04 | 8.66e-03 | 3.36e-02 |
|  | 17 | 63534340 | 63534688 | PDFSTATSENSEMBLUCSC | 349 | 4 | 16735 | gene;island | 0.407851 | 0.909969 | Not queried | 4.04 | 5.71e-02 | 1.00e-01 |
|  | 10 | 685962 | 686354 | PDFSTATSENSEMBLUCSC | 393 | 4 | 4448 | gene;island | 0.734418 | 0.969422 | Not queried | 4.04 | 5.71e-02 | 1.00e-01 |
|  | 7 | 18535072 | 18535499 | PDFSTATSENSEMBLUCSC | 428 | 6 | 37458 | tss | 0.597630 | 0.197771 | HDAC9 (margin: HDAC9) | 4.04 | 4.33e-03 | 2.52e-02 |
|  | 5 | 127874463 | 127874587 | PDFSTATSENSEMBLUCSC | 125 | 4 | 32259 | tss;island | 0.660617 | 0.968587 | Not queried | 4.04 | 2.86e-02 | 6.12e-02 |
|  | 5 | 38258007 | 38258451 | PDFSTATSENSEMBLUCSC | 445 | 7 | 32028 | tss | 0.596261 | 0.941103 | Not queried | 4.04 | 2.62e-02 | 6.12e-02 |
|  | 19 | 19221316 | 19221574 | PDFSTATSENSEMBLUCSC | 259 | 4 | 19946 | gene;island | 0.735305 | 0.963947 | Not queried | 4.04 | 2.86e-02 | 6.12e-02 |
|  | 4 | 154265415 | 154265635 | PDFSTATSENSEMBLUCSC | 221 | 5 | 30641 | tss;island | 0.089611 | 0.018225 | Not queried | 4.04 | 2.22e-01 | 2.76e-01 |
|  | 9 | 89763370 | 89763570 | PDFSTATSENSEMBLUCSC | 201 | 4 | 40901 | island | 0.603851 | 0.042559 | Not queried | 4.04 | 5.71e-02 | 1.00e-01 |
|  | 8 | 11560299 | 11560851 | PDFSTATSENSEMBLUCSC | 553 | 12 | 39552 | tss;island | 0.607764 | 0.964762 | GATA4 (margin: GATA4) | 4.04 | 6.56e-04 | 7.02e-03 |
|  | 7 | 1351539 | 1352002 | PDFSTATSENSEMBLUCSC | 464 | 8 | 38098 | island | 0.584811 | 0.972251 | Not queried | 4.04 | 3.79e-02 | 7.66e-02 |
|  | 13 | 112728799 | 112728964 | PDFSTATSENSEMBLUCSC | 166 | 4 | 11914 | island | 0.413969 | 0.950354 | Not queried | 4.03 | 2.86e-02 | 6.12e-02 |
|  | 10 | 25464008 | 25464513 | PDFSTATSENSEMBLUCSC | 506 | 12 | 4533 | gene;island | 0.728679 | 0.097295 | GPR158; GPR158-AS1 (margin: GPR158; GPR158-AS1) | 4.03 | 5.56e-03 | 3.16e-02 |
|  | 19 | 2289982 | 2290107 | PDFSTATSENSEMBLUCSC | 126 | 4 | 19685 | gene;island | 0.712561 | 0.970466 | Not queried | 4.03 | 2.86e-02 | 6.12e-02 |
|  | 1 | 117112616 | 117112854 | PDFSTATSENSEMBLUCSC | 239 | 4 | 709 | gene;island | 0.014090 | 0.237435 | Not queried | 4.03 | 1.14e-01 | 1.68e-01 |
|  | 17 | 72580901 | 72581081 | PDFSTATSENSEMBLUCSC | 181 | 5 | 16759 | gene | 0.185693 | 0.877480 | Not queried | 4.03 | 1.59e-02 | 5.36e-02 |
|  | 1 | 11919018 | 11919699 | PDFSTATSENSEMBLUCSC | 682 | 9 | 1252 | tss | 0.126322 | 0.034986 | NPPB (margin: NPPA-AS1; NPPA; NPPB) | 4.03 | 2.76e-03 | 1.78e-02 |
|  | 12 | 14720805 | 14721288 | PDFSTATSENSEMBLUCSC | 484 | 9 | 9584 | tss | 0.235966 | 0.927779 | Not queried | 4.03 | 5.03e-02 | 9.87e-02 |
|  | 8 | 143580770 | 143581070 | PDFSTATSENSEMBLUCSC | 301 | 4 | 39463 | gene;island | 0.685423 | 0.969365 | Not queried | 4.03 | 1.14e-01 | 1.68e-01 |
|  | 6 | 80656586 | 80656973 | PDFSTATSENSEMBLUCSC | 388 | 5 | 34399 | gene;island | 0.224159 | 0.021972 | ELOVL4 (margin: ELOVL4; GAPDHP63) | 4.03 | 7.94e-03 | 3.19e-02 |
|  | 12 | 43946284 | 43946518 | PDFSTATSENSEMBLUCSC | 235 | 4 | 9647 | tss;island | 0.548655 | 0.978071 | Not queried | 4.03 | 5.71e-02 | 1.00e-01 |
|  | 15 | 93257525 | 93258103 | PDFSTATSENSEMBLUCSC | 579 | 6 | 14474 | island | 0.360059 | 0.049119 | Not queried | 4.03 | 1.52e-02 | 5.36e-02 |
|  | 11 | 35160462 | 35160892 | PDFSTATSENSEMBLUCSC | 431 | 6 | 6583 | gene | 0.008193 | 0.086509 | CD44; RP4-607I7.1 (margin: CD44; AL356215.1; RP4-607I7.1) | 4.03 | 8.66e-03 | 3.36e-02 |
|  | 22 | 28198218 | 28198591 | PDFSTATSENSEMBLUCSC | 374 | 4 | 27038 | tss;island | 0.630793 | 0.972119 | Not queried | 4.03 | 2.86e-02 | 6.12e-02 |
|  | 15 | 81426347 | 81426820 | PDFSTATSENSEMBLUCSC | 474 | 10 | 14378 | island | 0.830843 | 0.980661 | C15orf26 (margin: C15orf26) | 4.03 | 7.25e-04 | 7.74e-03 |
|  | 15 | 81426347 | 81426610 | PDFSTATSENSEMBLUCSC | 264 | 8 | 13852 | tss | 0.831292 | 0.981286 | C15orf26 (margin: C15orf26) | 4.03 | 2.95e-03 | 1.88e-02 |
|  | 12 | 57634549 | 57634855 | PDFSTATSENSEMBLUCSC | 307 | 7 | 9814 | tss | 0.109171 | 0.017123 | NDUFA4L2 (margin: SHMT2; NDUFA4L2; STAC3; RP11-123K3.4; R3HDM2) | 4.03 | 6.99e-03 | 3.19e-02 |
|  | 1 | 68515788 | 68516713 | PDFSTATSENSEMBLUCSC | 926 | 17 | 3369 | island | 0.614361 | 0.955184 | GNG12-AS1; DIRAS3; ARL5AP3 (margin: GNG12-AS1; RP4-609E1.2; AL157407.1; DIRAS3; ARL5AP3) | 4.03 | 2.52e-04 | 4.14e-03 |
|  | 19 | 37958346 | 37958752 | PDFSTATSENSEMBLUCSC | 407 | 7 | 20738 | tss;island | 0.331619 | 0.032744 | Not queried | 4.03 | 5.30e-02 | 1.00e-01 |
|  | 12 | 131200394 | 131200855 | PDFSTATSENSEMBLUCSC | 462 | 6 | 11006 | island | 0.061165 | 0.014023 | Not queried | 4.02 | 6.49e-02 | 1.11e-01 |
|  | 1 | 43424882 | 43425412 | PDFSTATSENSEMBLUCSC | 531 | 6 | 1555 | tss | 0.150888 | 0.004992 | Not queried | 4.02 | 4.11e-02 | 8.14e-02 |
|  | 1 | 1935048 | 1935561 | PDFSTATSENSEMBLUCSC | 514 | 6 | 2467 | island | 0.521313 | 0.957298 | C1orf222 (margin: C1orf222; RP11-547D24.1) | 4.02 | 2.16e-03 | 1.45e-02 |
|  | 7 | 30029717 | 30029808 | PDFSTATSENSEMBLUCSC | 92 | 6 | 36952 | gene | 0.080215 | 0.506810 | AC007285.6; SCRN1 (margin: AC007285.6; SCRN1) | 4.02 | 2.16e-03 | 1.45e-02 |
|  | 7 | 95064691 | 95064926 | PDFSTATSENSEMBLUCSC | 236 | 6 | 37728 | tss;island | 0.367120 | 0.032842 | Not queried | 4.02 | 1.32e-01 | 1.89e-01 |
|  | 5 | 158689930 | 158690029 | PDFSTATSENSEMBLUCSC | 100 | 4 | 32462 | tss | 0.001759 | 0.005038 | Not queried | 4.02 | 8.86e-01 | 8.89e-01 |
|  | 6 | 91320964 | 91321281 | PDFSTATSENSEMBLUCSC | 318 | 4 | 36330 | island | 0.448118 | 0.070244 | Not queried | 4.02 | 2.86e-02 | 6.12e-02 |
|  | 3 | 197807842 | 197808169 | PDFSTATSENSEMBLUCSC | 328 | 5 | 28829 | tss;island | 0.215672 | 0.017711 | Not queried | 4.02 | 3.17e-02 | 6.50e-02 |
|  | 19 | 291239 | 291315 | PDFSTATSENSEMBLUCSC | 77 | 5 | 19622 | gene;tss;island | 0.137830 | 0.010572 | Not queried | 4.02 | 3.17e-02 | 6.50e-02 |
|  | 7 | 120968877 | 120969174 | PDFSTATSENSEMBLUCSC | 298 | 8 | 37203 | gene;island | 0.827127 | 0.216850 | WNT16 (margin: WNT16) | 4.02 | 1.55e-04 | 2.71e-03 |
|  | 1 | 228246632 | 228247135 | PDFSTATSENSEMBLUCSC | 504 | 4 | 1046 | gene;island | 0.831576 | 0.987893 | Not queried | 4.02 | 2.86e-02 | 6.12e-02 |
|  | 21 | 15588349 | 15588608 | PDFSTATSENSEMBLUCSC | 260 | 5 | 26607 | island | 0.332731 | 0.021603 | Not queried | 4.02 | 1.59e-02 | 5.36e-02 |
|  | 3 | 44666362 | 44666508 | PDFSTATSENSEMBLUCSC | 147 | 5 | 28258 | tss | 0.011693 | 0.047956 | Not queried | 4.02 | 2.22e-01 | 2.76e-01 |
|  | 12 | 57915555 | 57915636 | PDFSTATSENSEMBLUCSC | 82 | 4 | 9818 | tss;island | 0.983060 | 0.804007 | Not queried | 4.02 | 2.86e-02 | 6.12e-02 |
|  | 1 | 165325030 | 165325231 | PDFSTATSENSEMBLUCSC | 202 | 4 | 2063 | tss | 0.590961 | 0.969359 | Not queried | 4.02 | 5.71e-02 | 1.00e-01 |
|  | 22 | 22862802 | 22863293 | PDFSTATSENSEMBLUCSC | 492 | 5 | 26806 | gene;island | 0.765560 | 0.980733 | Not queried | 4.01 | 1.59e-02 | 5.36e-02 |
|  | 1 | 1549615 | 1549818 | PDFSTATSENSEMBLUCSC | 204 | 4 | 1157 | tss;island | 0.027035 | 0.258696 | Not queried | 4.01 | 2.86e-02 | 6.12e-02 |
|  | 12 | 109728043 | 109728241 | PDFSTATSENSEMBLUCSC | 199 | 4 | 9291 | gene;island | 0.581125 | 0.915150 | Not queried | 4.01 | 2.86e-02 | 6.12e-02 |
|  | 7 | 1660314 | 1660685 | PDFSTATSENSEMBLUCSC | 372 | 4 | 38121 | island | 0.625955 | 0.962369 | Not queried | 4.01 | 2.86e-02 | 6.12e-02 |
|  | 12 | 3600019 | 3600317 | PDFSTATSENSEMBLUCSC | 299 | 6 | 9494 | tss;island | 0.387209 | 0.825765 | Not queried | 4.01 | 6.49e-02 | 1.11e-01 |
|  | 1 | 107683532 | 107683775 | PDFSTATSENSEMBLUCSC | 244 | 5 | 658 | gene;island | 0.446481 | 0.045560 | Not queried | 4.01 | 9.52e-02 | 1.53e-01 |
|  | 12 | 7072696 | 7072891 | PDFSTATSENSEMBLUCSC | 196 | 4 | 9539 | tss | 0.077842 | 0.010780 | Not queried | 4.01 | 2.86e-02 | 6.12e-02 |
|  | 15 | 28341500 | 28341980 | PDFSTATSENSEMBLUCSC | 481 | 5 | 13265 | gene;island | 0.278556 | 0.674479 | Not queried | 4.01 | 4.21e-01 | 4.59e-01 |
|  | 7 | 43351208 | 43351604 | PDFSTATSENSEMBLUCSC | 397 | 6 | 36982 | gene | 0.729350 | 0.969319 | Not queried | 4.01 | 9.31e-02 | 1.53e-01 |
|  | 5 | 158893318 | 158893488 | PDFSTATSENSEMBLUCSC | 171 | 4 | 32463 | tss | 0.807481 | 0.985012 | Not queried | 4.01 | 2.86e-02 | 6.12e-02 |
|  | 11 | 93583672 | 93583973 | PDFSTATSENSEMBLUCSC | 302 | 6 | 7626 | tss | 0.042885 | 0.418688 | VSTM5 (margin: VSTM5) | 4.01 | 2.16e-03 | 1.45e-02 |
|  | 6 | 33171765 | 33172572 | PDFSTATSENSEMBLUCSC | 808 | 16 | 35978 | island | 0.042213 | 0.427258 | SLC39A7; HSD17B8 (margin: RNY4P10; SLC39A7; HSD17B8; MIR219-1; RING1; ZNF70P1; COL11A2; RXRB) | 4.01 | 1.94e-03 | 1.45e-02 |
|  | 8 | 140716495 | 140716766 | PDFSTATSENSEMBLUCSC | 272 | 6 | 39924 | tss;island | 0.319875 | 0.039138 | KCNK9 (margin: KCNK9) | 4.01 | 2.16e-03 | 1.45e-02 |
|  | 5 | 87974369 | 87974547 | PDFSTATSENSEMBLUCSC | 179 | 7 | 31680 | gene;tss;island | 0.816910 | 0.989267 | CTC-467M3.1; LINC00461 (margin: CTC-467M3.1; LINC00461) | 4.01 | 2.33e-03 | 1.52e-02 |
|  | 2 | 176944273 | 176944601 | PDFSTATSENSEMBLUCSC | 329 | 4 | 24887 | island | 0.275651 | 0.020602 | Not queried | 4.00 | 2.86e-02 | 6.12e-02 |
|  | 16 | 725416 | 725581 | PDFSTATSENSEMBLUCSC | 166 | 4 | 14975 | tss | 0.760835 | 0.978788 | Not queried | 4.00 | 2.86e-02 | 6.12e-02 |
|  | 19 | 41510026 | 41510285 | PDFSTATSENSEMBLUCSC | 260 | 7 | 20052 | gene | 0.701181 | 0.968617 | CYP2B6 (margin: CYP2B6) | 4.00 | 6.99e-03 | 3.19e-02 |
|  | 14 | 54421381 | 54421578 | PDFSTATSENSEMBLUCSC | 198 | 7 | 12083 | gene;tss;island | 0.612948 | 0.955923 | BMP4 (margin: MIR5580; BMP4) | 4.00 | 5.83e-04 | 6.47e-03 |
|  | 13 | 28366427 | 28366759 | PDFSTATSENSEMBLUCSC | 333 | 8 | 11364 | tss | 0.941962 | 0.489330 | Not queried | 4.00 | 3.79e-02 | 7.66e-02 |
|  | 7 | 27213966 | 27214383 | PDFSTATSENSEMBLUCSC | 418 | 8 | 37498 | tss | 0.889836 | 0.989316 | RP1-170O19.20; HOXA10 (margin: HOXA-AS4; HOXA11-AS; HOXA9; RP1-170O19.20; MIR196B; HOXA10; HOXA11; RP1-170O19.14) | 4.00 | 6.22e-04 | 6.71e-03 |
|  | 22 | 25507318 | 25507612 | PDFSTATSENSEMBLUCSC | 295 | 4 | 26818 | gene;island | 0.226949 | 0.016551 | Not queried | 4.00 | 2.86e-02 | 6.12e-02 |
|  | 17 | 7486551 | 7487593 | PDFSTATSENSEMBLUCSC | 1043 | 14 | 17959 | island | 0.222572 | 0.010021 | Not queried | 4.00 | 2.46e-01 | 3.01e-01 |
|  | 1 | 204966000 | 204966512 | PDFSTATSENSEMBLUCSC | 513 | 7 | 962 | gene | 0.498554 | 0.943485 | NFASC (margin: NFASC) | 4.00 | 4.08e-03 | 2.47e-02 |
|  | 11 | 70508022 | 70508659 | PDFSTATSENSEMBLUCSC | 638 | 10 | 7529 | tss | 0.600272 | 0.949345 | SHANK2 (margin: SHANK2) | 4.00 | 7.58e-05 | 1.98e-03 |
|  | 15 | 66790240 | 66790410 | PDFSTATSENSEMBLUCSC | 171 | 4 | 13749 | tss | 0.006183 | 0.047583 | Not queried | 4.00 | 1.14e-01 | 1.68e-01 |
|  | 7 | 73245439 | 73245752 | PDFSTATSENSEMBLUCSC | 314 | 4 | 37042 | gene;island | 0.164686 | 0.012916 | Not queried | 4.00 | 2.86e-02 | 6.12e-02 |
|  | 8 | 119963842 | 119964729 | PDFSTATSENSEMBLUCSC | 888 | 11 | 40556 | island | 0.645056 | 0.043316 | TNFRSF11B (margin: TNFRSF11B) | 4.00 | 1.28e-02 | 4.76e-02 |
|  | 1 | 27683345 | 27683501 | PDFSTATSENSEMBLUCSC | 157 | 4 | 352 | gene;island | 0.702545 | 0.971259 | Not queried | 4.00 | 2.86e-02 | 6.12e-02 |
|  | 11 | 115374969 | 115375226 | PDFSTATSENSEMBLUCSC | 258 | 4 | 6913 | gene | 0.142809 | 0.011472 | Not queried | 4.00 | 2.86e-02 | 6.12e-02 |
|  | 7 | 4264980 | 4265548 | PDFSTATSENSEMBLUCSC | 569 | 6 | 36836 | gene | 0.433500 | 0.950254 | SDK1 (margin: SDK1) | 4.00 | 2.16e-03 | 1.45e-02 |
|  | 10 | 105239085 | 105239205 | PDFSTATSENSEMBLUCSC | 121 | 4 | 5339 | tss | 0.020949 | 0.281290 | Not queried | 3.99 | 2.86e-02 | 6.12e-02 |
|  | 18 | 35147056 | 35147465 | PDFSTATSENSEMBLUCSC | 410 | 8 | 19247 | tss | 0.684345 | 0.979278 | Not queried | 3.99 | 4.99e-02 | 9.81e-02 |
|  | 18 | 49865711 | 49866548 | PDFSTATSENSEMBLUCSC | 838 | 18 | 19518 | island | 0.409230 | 0.134987 | Not queried | 3.99 | 3.42e-02 | 7.00e-02 |
|  | 1 | 151966792 | 151967023 | PDFSTATSENSEMBLUCSC | 232 | 5 | 1935 | tss;island | 0.005121 | 0.067493 | Not queried | 3.99 | 5.56e-02 | 1.00e-01 |
|  | 5 | 170210440 | 170210672 | PDFSTATSENSEMBLUCSC | 233 | 4 | 32485 | tss | 0.208952 | 0.885789 | Not queried | 3.99 | 2.86e-02 | 6.12e-02 |
|  | 6 | 70576168 | 70576388 | PDFSTATSENSEMBLUCSC | 221 | 4 | 35056 | tss | 0.657025 | 0.965560 | Not queried | 3.99 | 2.86e-02 | 6.12e-02 |
|  | 11 | 70507825 | 70508659 | PDFSTATSENSEMBLUCSC | 835 | 11 | 6793 | gene;island | 0.620673 | 0.957428 | SHANK2 (margin: SHANK2) | 3.99 | 1.98e-05 | 8.44e-04 |
|  | 12 | 85430025 | 85430336 | PDFSTATSENSEMBLUCSC | 312 | 10 | 9899 | tss | 0.027943 | 0.002919 | Not queried | 3.99 | 3.55e-02 | 7.24e-02 |
|  | 1 | 36038468 | 36038950 | PDFSTATSENSEMBLUCSC | 483 | 7 | 1485 | tss;island | 0.654418 | 0.985138 | TFAP2E; RP4-728D4.2 (margin: NCDN; TFAP2E; RP4-728D4.2) | 3.99 | 6.99e-03 | 3.19e-02 |
|  | 20 | 62209427 | 62209642 | PDFSTATSENSEMBLUCSC | 216 | 4 | 26361 | island | 0.735399 | 0.976898 | Not queried | 3.99 | 5.71e-02 | 1.00e-01 |
|  | 10 | 105344723 | 105344807 | PDFSTATSENSEMBLUCSC | 85 | 4 | 4744 | gene;island | 0.741404 | 0.975199 | Not queried | 3.99 | 2.86e-02 | 6.12e-02 |
|  | 12 | 117476704 | 117477105 | PDFSTATSENSEMBLUCSC | 402 | 4 | 10858 | island | 0.576977 | 0.964734 | Not queried | 3.99 | 5.71e-02 | 1.00e-01 |
|  | 3 | 153838788 | 153839142 | PDFSTATSENSEMBLUCSC | 355 | 7 | 28667 | tss | 0.256371 | 0.010669 | ARHGEF26; ARHGEF26-AS1 (margin: ARHGEF26; ARHGEF26-AS1) | 3.98 | 6.99e-03 | 3.19e-02 |
|  | 5 | 180258557 | 180258852 | PDFSTATSENSEMBLUCSC | 296 | 5 | 31948 | gene;island | 0.009064 | 0.067335 | Not queried | 3.98 | 9.52e-02 | 1.53e-01 |
|  | 8 | 40755380 | 40755559 | PDFSTATSENSEMBLUCSC | 180 | 5 | 39666 | tss | 0.218149 | 0.017408 | ZMAT4; RP11-465K16.1 (margin: ZMAT4; RP11-465K16.1) | 3.98 | 7.94e-03 | 3.19e-02 |
|  | 21 | 38070524 | 38071066 | PDFSTATSENSEMBLUCSC | 543 | 11 | 26538 | tss;island | 0.553452 | 0.961422 | Not queried | 3.98 | 4.00e-02 | 8.03e-02 |
|  | 17 | 7348316 | 7348490 | PDFSTATSENSEMBLUCSC | 175 | 8 | 17950 | island | 0.957797 | 0.619744 | RP11-104H15.7; FGF11; CHRNB1; RP11-104H15.8 (margin: RP11-104H15.7; TMEM102; FGF11; CHRNB1; RP11-104H15.9; RP11-104H15.8) | 3.98 | 1.55e-04 | 2.71e-03 |
|  | 17 | 7348316 | 7348399 | PDFSTATSENSEMBLUCSC | 84 | 6 | 17084 | tss | 0.957797 | 0.619744 | RP11-104H15.7; FGF11; CHRNB1; RP11-104H15.8 (margin: RP11-104H15.7; TMEM102; FGF11; CHRNB1; RP11-104H15.9; RP11-104H15.8) | 3.98 | 2.16e-03 | 1.45e-02 |
|  | 9 | 130679027 | 130679246 | PDFSTATSENSEMBLUCSC | 220 | 4 | 40785 | gene;island | 0.024727 | 0.315984 | Not queried | 3.98 | 2.00e-01 | 2.55e-01 |
|  | 4 | 15471214 | 15471377 | PDFSTATSENSEMBLUCSC | 164 | 4 | 30309 | tss | 0.248471 | 0.014460 | Not queried | 3.98 | 2.86e-02 | 6.12e-02 |
|  | 18 | 61143926 | 61143993 | PDFSTATSENSEMBLUCSC | 68 | 5 | 19312 | tss | 0.001991 | 0.025353 | SERPINB5 (margin: SERPINB5; RP11-635N19.3) | 3.98 | 7.94e-03 | 3.19e-02 |
|  | 14 | 24505537 | 24505898 | PDFSTATSENSEMBLUCSC | 362 | 7 | 12724 | island | 0.008592 | 0.339748 | DHRS4L1 (margin: DHRS4L1) | 3.98 | 1.11e-02 | 4.14e-02 |
|  | 14 | 24505537 | 24505671 | PDFSTATSENSEMBLUCSC | 135 | 5 | 12309 | tss | 0.008592 | 0.339748 | Not queried | 3.98 | 3.17e-02 | 6.50e-02 |
|  | 19 | 47217696 | 47218031 | PDFSTATSENSEMBLUCSC | 336 | 5 | 20896 | tss | 0.016369 | 0.207114 | Not queried | 3.98 | 3.17e-02 | 6.50e-02 |
|  | 2 | 124782586 | 124782831 | PDFSTATSENSEMBLUCSC | 246 | 4 | 23607 | tss | 0.952573 | 0.621926 | Not queried | 3.98 | 5.71e-02 | 1.00e-01 |
|  | 2 | 1234387 | 1234756 | PDFSTATSENSEMBLUCSC | 370 | 4 | 22527 | gene | 0.649296 | 0.981641 | Not queried | 3.98 | 5.71e-02 | 1.00e-01 |
|  | 9 | 137030463 | 137030823 | PDFSTATSENSEMBLUCSC | 361 | 5 | 40853 | tss;island | 0.459115 | 0.038731 | Not queried | 3.97 | 9.52e-02 | 1.53e-01 |
|  | 4 | 25031951 | 25032305 | PDFSTATSENSEMBLUCSC | 355 | 4 | 29969 | gene | 0.444525 | 0.040298 | Not queried | 3.97 | 5.71e-02 | 1.00e-01 |
|  | 1 | 236557473 | 236557758 | PDFSTATSENSEMBLUCSC | 286 | 4 | 2343 | tss;island | 0.199715 | 0.868930 | Not queried | 3.97 | 2.00e-01 | 2.55e-01 |
|  | 13 | 114840185 | 114840743 | PDFSTATSENSEMBLUCSC | 559 | 5 | 11312 | gene;island | 0.911415 | 0.973681 | Not queried | 3.97 | 2.22e-01 | 2.76e-01 |
|  | 1 | 46767565 | 46767971 | PDFSTATSENSEMBLUCSC | 407 | 4 | 511 | gene;island | 0.001250 | 0.017843 | Not queried | 3.97 | 2.86e-02 | 6.12e-02 |
|  | 11 | 1948933 | 1949113 | PDFSTATSENSEMBLUCSC | 181 | 5 | 6439 | gene;island | 0.649414 | 0.967303 | TNNT3 (margin: TNNT3; AC051649.1) | 3.97 | 7.94e-03 | 3.19e-02 |
|  | 7 | 82792239 | 82792411 | PDFSTATSENSEMBLUCSC | 173 | 7 | 37678 | tss | 0.146606 | 0.007479 | PCLO (margin: PCLO) | 3.97 | 2.33e-03 | 1.52e-02 |
|  | 2 | 496713 | 497417 | PDFSTATSENSEMBLUCSC | 705 | 7 | 24004 | island | 0.561086 | 0.909061 | (margin: AC093326.1) | 3.97 | 4.08e-03 | 2.47e-02 |
|  | 3 | 186648175 | 186648544 | PDFSTATSENSEMBLUCSC | 370 | 5 | 29756 | island | 0.488476 | 0.965817 | Not queried | 3.97 | 5.56e-02 | 1.00e-01 |
|  | 14 | 76044591 | 76045025 | PDFSTATSENSEMBLUCSC | 435 | 6 | 13018 | island | 0.208654 | 0.036531 | FLVCR2; AC007182.6 (margin: FLVCR2; AC007182.6) | 3.97 | 8.66e-03 | 3.36e-02 |
|  | 15 | 48470606 | 48470973 | PDFSTATSENSEMBLUCSC | 368 | 6 | 13654 | tss | 0.970828 | 0.357044 | Not queried | 3.97 | 1.32e-01 | 1.89e-01 |
|  | 14 | 105531855 | 105532252 | PDFSTATSENSEMBLUCSC | 398 | 6 | 12666 | tss | 0.262842 | 0.806144 | Not queried | 3.96 | 6.49e-02 | 1.11e-01 |
|  | 3 | 9811714 | 9811990 | PDFSTATSENSEMBLUCSC | 277 | 7 | 28130 | tss;island | 0.702927 | 0.164571 | OGG1; CAMK1 (margin: OGG1; CAMK1; TADA3) | 3.96 | 5.83e-04 | 6.47e-03 |
|  | 17 | 65040527 | 65040616 | PDFSTATSENSEMBLUCSC | 90 | 6 | 17596 | tss | 0.595661 | 0.972321 | CACNG1 (margin: CACNG4; CACNG1; RP11-74H8.1) | 3.96 | 2.16e-03 | 1.45e-02 |
|  | 4 | 48988015 | 48988321 | PDFSTATSENSEMBLUCSC | 307 | 7 | 31037 | island | 0.475123 | 0.960796 | Not queried | 3.96 | 2.62e-02 | 6.12e-02 |
|  | 1 | 3207371 | 3207588 | PDFSTATSENSEMBLUCSC | 218 | 5 | 117 | gene;island | 0.558802 | 0.960104 | PRDM16 (margin: PRDM16) | 3.96 | 7.94e-03 | 3.19e-02 |
|  | 4 | 8594324 | 8594549 | PDFSTATSENSEMBLUCSC | 226 | 4 | 30924 | island | 0.377984 | 0.028733 | Not queried | 3.96 | 2.86e-02 | 6.12e-02 |
|  | 1 | 41827960 | 41828276 | PDFSTATSENSEMBLUCSC | 317 | 4 | 3137 | island | 0.479858 | 0.137386 | Not queried | 3.96 | 4.86e-01 | 5.11e-01 |
|  | 4 | 124426923 | 124427390 | PDFSTATSENSEMBLUCSC | 468 | 5 | 31294 | island | 0.529539 | 0.126117 | Not queried | 3.96 | 5.48e-01 | 5.70e-01 |
|  | 16 | 1070957 | 1071402 | PDFSTATSENSEMBLUCSC | 446 | 5 | 15508 | island | 0.546929 | 0.937628 | Not queried | 3.96 | 1.59e-02 | 5.36e-02 |
|  | 19 | 58446312 | 58446669 | PDFSTATSENSEMBLUCSC | 358 | 4 | 20254 | gene | 0.920063 | 0.461796 | Not queried | 3.96 | 2.86e-02 | 6.12e-02 |
|  | 2 | 176948693 | 176949017 | PDFSTATSENSEMBLUCSC | 325 | 7 | 23731 | tss | 0.980766 | 0.595048 | Not queried | 3.95 | 1.65e-01 | 2.28e-01 |
|  | 6 | 112575850 | 112576119 | PDFSTATSENSEMBLUCSC | 270 | 5 | 35183 | tss | 0.468805 | 0.942590 | RP11-506B6.6; LAMA4 (margin: RP11-506B6.6; LAMA4) | 3.95 | 7.94e-03 | 3.19e-02 |
|  | 11 | 1097602 | 1098080 | PDFSTATSENSEMBLUCSC | 479 | 4 | 6405 | gene;island | 0.119653 | 0.781920 | Not queried | 3.95 | 2.00e-01 | 2.55e-01 |
|  | 12 | 14720726 | 14721288 | PDFSTATSENSEMBLUCSC | 563 | 10 | 10264 | island | 0.190021 | 0.906839 | Not queried | 3.95 | 5.24e-02 | 1.00e-01 |
|  | 2 | 238289816 | 238289897 | PDFSTATSENSEMBLUCSC | 82 | 4 | 23091 | gene | 0.683606 | 0.959030 | Not queried | 3.95 | 2.86e-02 | 6.12e-02 |
|  | 12 | 50361294 | 50361679 | PDFSTATSENSEMBLUCSC | 386 | 5 | 10408 | island | 0.511898 | 0.063512 | Not queried | 3.95 | 9.52e-02 | 1.53e-01 |
|  | 17 | 47547353 | 47547767 | PDFSTATSENSEMBLUCSC | 415 | 5 | 18523 | island | 0.308907 | 0.735374 | Not queried | 3.95 | 3.17e-02 | 6.50e-02 |
|  | 11 | 8615506 | 8616172 | PDFSTATSENSEMBLUCSC | 667 | 14 | 7126 | tss | 0.802201 | 0.975519 | STK33 (margin: STK33) | 3.95 | 1.36e-05 | 5.85e-04 |
|  | 22 | 44319257 | 44319719 | PDFSTATSENSEMBLUCSC | 463 | 6 | 27561 | island | 0.039367 | 0.004374 | Not queried | 3.95 | 2.40e-01 | 2.95e-01 |
|  | 14 | 24808987 | 24809226 | PDFSTATSENSEMBLUCSC | 240 | 4 | 12048 | gene | 0.856496 | 0.989815 | Not queried | 3.95 | 2.86e-02 | 6.12e-02 |
|  | 20 | 50159346 | 50159653 | PDFSTATSENSEMBLUCSC | 308 | 4 | 25825 | tss | 0.885019 | 0.368389 | Not queried | 3.94 | 5.71e-02 | 1.00e-01 |
|  | 2 | 19553237 | 19553546 | PDFSTATSENSEMBLUCSC | 310 | 4 | 22587 | gene;island | 0.853520 | 0.984029 | Not queried | 3.94 | 1.14e-01 | 1.68e-01 |
|  | 22 | 43830900 | 43831161 | PDFSTATSENSEMBLUCSC | 262 | 4 | 26890 | gene;island | 0.621971 | 0.977683 | Not queried | 3.94 | 2.86e-02 | 6.12e-02 |
|  | 11 | 3663198 | 3663543 | PDFSTATSENSEMBLUCSC | 346 | 6 | 6479 | gene | 0.364560 | 0.032129 | Not queried | 3.94 | 2.60e-02 | 6.12e-02 |
|  | 2 | 241938159 | 241938193 | PDFSTATSENSEMBLUCSC | 35 | 5 | 23983 | tss | 0.114432 | 0.008232 | SNED1 (margin: AC104809.3; SNED1; AC093585.6) | 3.94 | 7.94e-03 | 3.19e-02 |
|  | 2 | 241938159 | 241938436 | PDFSTATSENSEMBLUCSC | 278 | 7 | 25283 | island | 0.141209 | 0.008232 | Not queried | 3.94 | 1.75e-02 | 5.79e-02 |
|  | 22 | 39323204 | 39323648 | PDFSTATSENSEMBLUCSC | 445 | 4 | 27498 | island | 0.792264 | 0.985143 | Not queried | 3.94 | 5.71e-02 | 1.00e-01 |
|  | 10 | 46993502 | 46993586 | PDFSTATSENSEMBLUCSC | 85 | 4 | 5718 | island | 0.598475 | 0.968897 | Not queried | 3.94 | 2.86e-02 | 6.12e-02 |
|  | 11 | 61159602 | 61159837 | PDFSTATSENSEMBLUCSC | 236 | 7 | 7334 | tss;island | 0.048030 | 0.437402 | TMEM216 (margin: TMEM216; CPSF7) | 3.94 | 1.11e-02 | 4.14e-02 |
|  | 11 | 2321770 | 2322050 | PDFSTATSENSEMBLUCSC | 281 | 9 | 6453 | gene;tss | 0.599768 | 0.946469 | TSPAN32; C11orf21 (margin: TSPAN32; C11orf21) | 3.93 | 4.11e-05 | 1.17e-03 |
|  | 11 | 67417958 | 67418113 | PDFSTATSENSEMBLUCSC | 156 | 4 | 6762 | gene | 0.076209 | 0.500916 | Not queried | 3.93 | 2.00e-01 | 2.55e-01 |
|  | 10 | 47151300 | 47151589 | PDFSTATSENSEMBLUCSC | 290 | 5 | 5720 | island | 0.019695 | 0.239619 | LINC00842 (margin: LINC00842; ANXA8L1) | 3.93 | 7.94e-03 | 3.19e-02 |
|  | 19 | 19648890 | 19649054 | PDFSTATSENSEMBLUCSC | 165 | 7 | 20645 | tss | 0.283654 | 0.785204 | YJEFN3; CILP2 (margin: NDUFA13; CTC-260F20.3; YJEFN3; CILP2) | 3.93 | 5.83e-04 | 6.47e-03 |
|  | 10 | 88719848 | 88720216 | PDFSTATSENSEMBLUCSC | 369 | 4 | 4663 | gene | 0.273669 | 0.857895 | Not queried | 3.93 | 2.00e-01 | 2.55e-01 |
|  | 18 | 35145353 | 35145650 | PDFSTATSENSEMBLUCSC | 298 | 4 | 19095 | gene;island | 0.300671 | 0.031418 | Not queried | 3.93 | 2.86e-02 | 6.12e-02 |
|  | 19 | 54135129 | 54135265 | PDFSTATSENSEMBLUCSC | 137 | 4 | 21033 | tss | 0.622529 | 0.975736 | Not queried | 3.93 | 5.71e-02 | 1.00e-01 |
|  | 17 | 4500142 | 4500399 | PDFSTATSENSEMBLUCSC | 258 | 5 | 16354 | gene;island | 0.579642 | 0.953648 | Not queried | 3.93 | 1.59e-02 | 5.36e-02 |
|  | 13 | 80917058 | 80917437 | PDFSTATSENSEMBLUCSC | 380 | 5 | 11803 | island | 0.418516 | 0.033390 | Not queried | 3.93 | 3.17e-02 | 6.50e-02 |
|  | 19 | 46438607 | 46438944 | PDFSTATSENSEMBLUCSC | 338 | 4 | 22166 | island | 0.636759 | 0.223122 | Not queried | 3.93 | 2.00e-01 | 2.55e-01 |
|  | 20 | 61885236 | 61885448 | PDFSTATSENSEMBLUCSC | 213 | 7 | 25499 | gene;island | 0.415606 | 0.216425 | Not queried | 3.93 | 1.28e-01 | 1.87e-01 |
|  | 22 | 22901569 | 22901661 | PDFSTATSENSEMBLUCSC | 93 | 4 | 26807 | gene | 0.280190 | 0.014673 | Not queried | 3.93 | 2.86e-02 | 6.12e-02 |
|  | 11 | 69519088 | 69519437 | PDFSTATSENSEMBLUCSC | 350 | 8 | 8596 | island | 0.284265 | 0.673886 | FGF19 (margin: FGF19) | 3.93 | 1.09e-03 | 1.10e-02 |
|  | 12 | 72666976 | 72667707 | PDFSTATSENSEMBLUCSC | 732 | 10 | 9218 | gene;island | 0.281286 | 0.854783 | TRHDE; TRHDE-AS1 (margin: TRHDE; TRHDE-AS1) | 3.93 | 2.17e-05 | 8.94e-04 |
|  | 19 | 23941207 | 23941575 | PDFSTATSENSEMBLUCSC | 369 | 5 | 19966 | gene | 0.517815 | 0.016040 | ZNF681 (margin: RP11-255H23.2; RPSAP58; ZNF681) | 3.93 | 7.94e-03 | 3.19e-02 |
|  | 6 | 32557321 | 32557478 | PDFSTATSENSEMBLUCSC | 158 | 5 | 34123 | gene | 0.389882 | 0.905465 | Not queried | 3.93 | 1.51e-01 | 2.11e-01 |
|  | 19 | 53696569 | 53696886 | PDFSTATSENSEMBLUCSC | 318 | 7 | 22370 | island | 0.668296 | 0.965568 | Not queried | 3.93 | 5.30e-02 | 1.00e-01 |
|  | 12 | 103889516 | 103890274 | PDFSTATSENSEMBLUCSC | 759 | 11 | 10731 | island | 0.443048 | 0.046486 | C12orf42 (margin: RP11-626I20.1; C12orf42; RP11-626I20.2) | 3.93 | 1.40e-03 | 1.33e-02 |
|  | 1 | 3606550 | 3606892 | PDFSTATSENSEMBLUCSC | 343 | 4 | 137 | gene;tss;island | 0.468033 | 0.921376 | Not queried | 3.92 | 3.43e-01 | 3.84e-01 |
|  | 3 | 152552410 | 152552615 | PDFSTATSENSEMBLUCSC | 206 | 7 | 28665 | tss;island | 0.223008 | 0.017734 | P2RY1 (margin: P2RY1; RP11-38P22.2) | 3.92 | 4.08e-03 | 2.47e-02 |
|  | 4 | 1504989 | 1505559 | PDFSTATSENSEMBLUCSC | 571 | 6 | 30788 | island | 0.438145 | 0.921064 | (margin: ) | 3.92 | 4.33e-03 | 2.52e-02 |
|  | 6 | 32018906 | 32019556 | PDFSTATSENSEMBLUCSC | 651 | 5 | 34017 | gene | 0.372143 | 0.758358 | Not queried | 3.92 | 3.17e-02 | 6.50e-02 |
|  | 3 | 127347343 | 127348155 | PDFSTATSENSEMBLUCSC | 813 | 9 | 29447 | island | 0.791528 | 0.208766 | Not queried | 3.92 | 4.89e-01 | 5.15e-01 |
|  | 10 | 135039948 | 135040250 | PDFSTATSENSEMBLUCSC | 303 | 5 | 6345 | island | 0.386666 | 0.883825 | KNDC1 (margin: KNDC1; UTF1; VENTX) | 3.92 | 7.94e-03 | 3.19e-02 |
|  | 6 | 29407834 | 29408666 | PDFSTATSENSEMBLUCSC | 833 | 13 | 33656 | gene | 0.092966 | 0.786980 | Not queried | 3.92 | 3.38e-02 | 6.92e-02 |
|  | 2 | 239930734 | 239931011 | PDFSTATSENSEMBLUCSC | 278 | 4 | 25249 | island | 0.679612 | 0.980371 | Not queried | 3.92 | 5.71e-02 | 1.00e-01 |
|  | 3 | 14851743 | 14852042 | PDFSTATSENSEMBLUCSC | 300 | 4 | 28915 | island | 0.610430 | 0.963536 | Not queried | 3.92 | 2.86e-02 | 6.12e-02 |
|  | 15 | 50474069 | 50474388 | PDFSTATSENSEMBLUCSC | 320 | 7 | 13665 | tss;island | 0.839302 | 0.291634 | SLC27A2; ATP8B4 (margin: SLC27A2; ATP8B4) | 3.92 | 5.83e-04 | 6.47e-03 |
|  | 17 | 40250199 | 40250517 | PDFSTATSENSEMBLUCSC | 319 | 4 | 18326 | island | 0.646069 | 0.972706 | Not queried | 3.92 | 2.86e-02 | 6.12e-02 |
|  | 1 | 162792092 | 162792613 | PDFSTATSENSEMBLUCSC | 522 | 6 | 3921 | island | 0.469971 | 0.045689 | Not queried | 3.92 | 1.32e-01 | 1.89e-01 |
|  | 1 | 149149314 | 149149465 | PDFSTATSENSEMBLUCSC | 152 | 4 | 3705 | island | 0.318667 | 0.845754 | Not queried | 3.92 | 5.71e-02 | 1.00e-01 |
|  | 10 | 134119682 | 134120029 | PDFSTATSENSEMBLUCSC | 348 | 5 | 4861 | gene;island | 0.753249 | 0.978588 | Not queried | 3.92 | 9.52e-02 | 1.53e-01 |
|  | 1 | 181057279 | 181057597 | PDFSTATSENSEMBLUCSC | 319 | 6 | 2128 | tss;island | 0.035564 | 0.416975 | Not queried | 3.91 | 1.80e-01 | 2.45e-01 |
|  | 15 | 89921672 | 89922071 | PDFSTATSENSEMBLUCSC | 400 | 4 | 13468 | gene;island | 0.200120 | 0.007260 | Not queried | 3.91 | 3.43e-01 | 3.84e-01 |
|  | 16 | 88937115 | 88937386 | PDFSTATSENSEMBLUCSC | 272 | 5 | 16226 | island | 0.402063 | 0.884683 | (margin: TRAPPC2L; RP11-830F9.5; PABPN1L; CBFA2T3) | 3.91 | 7.94e-03 | 3.19e-02 |
|  | 1 | 109204168 | 109204325 | PDFSTATSENSEMBLUCSC | 158 | 5 | 1790 | tss;island | 0.814424 | 0.225785 | HENMT1 (margin: HENMT1) | 3.91 | 7.94e-03 | 3.19e-02 |
|  | 12 | 15475116 | 15475767 | PDFSTATSENSEMBLUCSC | 652 | 10 | 10269 | island | 0.202319 | 0.804411 | PTPRO; RERG (margin: PTPRO; RERG) | 3.91 | 7.58e-05 | 1.98e-03 |
|  | 16 | 1080388 | 1080808 | PDFSTATSENSEMBLUCSC | 421 | 4 | 15509 | island | 0.467471 | 0.846543 | Not queried | 3.91 | 1.14e-01 | 1.68e-01 |
|  | 7 | 43151725 | 43152125 | PDFSTATSENSEMBLUCSC | 401 | 6 | 37555 | tss | 0.386647 | 0.835507 | Not queried | 3.91 | 6.49e-02 | 1.11e-01 |
|  | 11 | 110583377 | 110583662 | PDFSTATSENSEMBLUCSC | 286 | 7 | 8794 | island | 0.458990 | 0.906887 | ARHGAP20 (margin: ARHGAP20) | 3.91 | 1.17e-03 | 1.13e-02 |
|  | 4 | 101111617 | 101111927 | PDFSTATSENSEMBLUCSC | 311 | 5 | 30516 | tss | 0.742314 | 0.103488 | Not queried | 3.91 | 2.22e-01 | 2.76e-01 |
|  | 1 | 112531802 | 112532085 | PDFSTATSENSEMBLUCSC | 284 | 7 | 1831 | tss | 0.598926 | 0.027494 | Not queried | 3.91 | 1.75e-02 | 5.79e-02 |
|  | 6 | 109777193 | 109777324 | PDFSTATSENSEMBLUCSC | 132 | 4 | 35164 | tss | 0.199892 | 0.029464 | Not queried | 3.91 | 2.00e-01 | 2.55e-01 |
|  | 16 | 67184918 | 67185194 | PDFSTATSENSEMBLUCSC | 277 | 6 | 15292 | tss;island | 0.672650 | 0.947783 | B3GNT9 (margin: C16orf70; FBXL8; B3GNT9; TRADD) | 3.91 | 2.16e-03 | 1.45e-02 |
|  | 3 | 119298195 | 119298585 | PDFSTATSENSEMBLUCSC | 391 | 9 | 29383 | island | 0.407000 | 0.020206 | Not queried | 3.91 | 1.42e-02 | 5.24e-02 |
|  | 12 | 2800055 | 2800500 | PDFSTATSENSEMBLUCSC | 446 | 8 | 9006 | gene;island | 0.648254 | 0.926792 | CACNA1C; CACNA1C-AS1 (margin: CACNA1C; CACNA1C-AS1) | 3.91 | 6.22e-04 | 6.71e-03 |
|  | 3 | 48647215 | 48647426 | PDFSTATSENSEMBLUCSC | 212 | 5 | 28302 | tss | 0.591924 | 0.066310 | Not queried | 3.91 | 2.22e-01 | 2.76e-01 |
|  | 2 | 81694296 | 81694608 | PDFSTATSENSEMBLUCSC | 313 | 5 | 24466 | island | 0.952177 | 0.483697 | Not queried | 3.91 | 5.56e-02 | 1.00e-01 |
|  | 19 | 19648890 | 19649144 | PDFSTATSENSEMBLUCSC | 255 | 8 | 21821 | island | 0.291595 | 0.818622 | YJEFN3; CILP2 (margin: NDUFA13; CTC-260F20.3; YJEFN3; CILP2) | 3.90 | 1.55e-04 | 2.71e-03 |
|  | 1 | 3077798 | 3078206 | PDFSTATSENSEMBLUCSC | 409 | 4 | 105 | gene;island | 0.313805 | 0.795540 | Not queried | 3.90 | 1.14e-01 | 1.68e-01 |
|  | 4 | 3873884 | 3874143 | PDFSTATSENSEMBLUCSC | 260 | 4 | 30856 | island | 0.574067 | 0.977450 | Not queried | 3.90 | 5.71e-02 | 1.00e-01 |
|  | 17 | 43324322 | 43324904 | PDFSTATSENSEMBLUCSC | 583 | 6 | 17427 | tss;island | 0.382849 | 0.035481 | Not queried | 3.90 | 3.10e-01 | 3.66e-01 |
|  | 7 | 985614 | 985839 | PDFSTATSENSEMBLUCSC | 226 | 5 | 36728 | gene;island | 0.208380 | 0.917456 | Not queried | 3.90 | 3.17e-02 | 6.50e-02 |
|  | 2 | 131010423 | 131010871 | PDFSTATSENSEMBLUCSC | 449 | 5 | 24717 | island | 0.624089 | 0.961102 | MED15P5 (margin: AC134915.1; MED15P5) | 3.90 | 7.94e-03 | 3.19e-02 |
|  | 21 | 43823604 | 43823863 | PDFSTATSENSEMBLUCSC | 260 | 5 | 26568 | tss | 0.439880 | 0.967275 | Not queried | 3.90 | 9.52e-02 | 1.53e-01 |
|  | 10 | 134095646 | 134096014 | PDFSTATSENSEMBLUCSC | 369 | 5 | 4860 | gene;island | 0.183742 | 0.769052 | STK32C (margin: STK32C) | 3.90 | 7.94e-03 | 3.19e-02 |
|  | 6 | 147829799 | 147830172 | PDFSTATSENSEMBLUCSC | 374 | 8 | 36549 | island | 0.736613 | 0.977233 | SAMD5 (margin: SAMD5) | 3.90 | 1.55e-04 | 2.71e-03 |
|  | 1 | 32827707 | 32828191 | PDFSTATSENSEMBLUCSC | 485 | 9 | 395 | gene;tss;island | 0.660730 | 0.115467 | TSSK3; FAM229A (margin: TSSK3; RP4-811H24.9; FAM229A; BSDC1) | 3.90 | 3.99e-03 | 2.47e-02 |
|  | 1 | 3100143 | 3100497 | PDFSTATSENSEMBLUCSC | 355 | 4 | 106 | gene;island | 0.719711 | 0.974088 | Not queried | 3.90 | 2.86e-02 | 6.12e-02 |
|  | 8 | 95961492 | 95961759 | PDFSTATSENSEMBLUCSC | 268 | 5 | 40464 | island | 0.008694 | 0.085002 | Not queried | 3.89 | 3.17e-02 | 6.50e-02 |
|  | 14 | 104314434 | 104314734 | PDFSTATSENSEMBLUCSC | 301 | 6 | 12654 | tss;island | 0.444986 | 0.002241 | Not queried | 3.89 | 9.31e-02 | 1.53e-01 |
|  | 15 | 71408603 | 71408914 | PDFSTATSENSEMBLUCSC | 312 | 4 | 13771 | tss;island | 0.394670 | 0.040915 | Not queried | 3.89 | 1.14e-01 | 1.68e-01 |
|  | 11 | 60869601 | 60869910 | PDFSTATSENSEMBLUCSC | 310 | 4 | 7328 | tss | 0.485481 | 0.919816 | Not queried | 3.89 | 2.86e-02 | 6.12e-02 |
|  | 4 | 174451141 | 174451564 | PDFSTATSENSEMBLUCSC | 424 | 11 | 30682 | tss;island | 0.432426 | 0.954492 | HAND2-AS1; HAND2 (margin: HAND2-AS1; HAND2) | 3.89 | 8.51e-05 | 2.07e-03 |
|  | 11 | 8615356 | 8616172 | PDFSTATSENSEMBLUCSC | 817 | 16 | 8033 | island | 0.817690 | 0.970300 | STK33 (margin: STK33) | 3.89 | 8.94e-06 | 4.82e-04 |
|  | 6 | 30458519 | 30458730 | PDFSTATSENSEMBLUCSC | 212 | 4 | 33769 | gene;island | 0.531518 | 0.936152 | Not queried | 3.89 | 5.71e-02 | 1.00e-01 |
|  | 20 | 45985495 | 45985741 | PDFSTATSENSEMBLUCSC | 247 | 7 | 25799 | tss | 0.097296 | 0.614412 | ZMYND8 (margin: RP4-569M23.2; ZMYND8) | 3.89 | 5.83e-04 | 6.47e-03 |
|  | 8 | 109095782 | 109096151 | PDFSTATSENSEMBLUCSC | 370 | 8 | 40530 | island | 0.907124 | 0.270347 | Not queried | 3.89 | 1.95e-01 | 2.55e-01 |
|  | 11 | 117389197 | 117389470 | PDFSTATSENSEMBLUCSC | 274 | 5 | 6921 | gene;island | 0.739996 | 0.951029 | Not queried | 3.89 | 5.56e-02 | 1.00e-01 |
|  | 22 | 44258572 | 44258712 | PDFSTATSENSEMBLUCSC | 141 | 5 | 27205 | tss;island | 0.726010 | 0.974596 | Not queried | 3.88 | 1.51e-01 | 2.11e-01 |
|  | 6 | 32372236 | 32372418 | PDFSTATSENSEMBLUCSC | 183 | 5 | 34110 | gene | 0.636178 | 0.961267 | Not queried | 3.88 | 3.10e-01 | 3.66e-01 |
|  | 17 | 26733294 | 26733583 | PDFSTATSENSEMBLUCSC | 290 | 6 | 17211 | tss;island | 0.359098 | 0.043655 | Not queried | 3.88 | 6.49e-02 | 1.11e-01 |
|  | 6 | 24358236 | 24358327 | PDFSTATSENSEMBLUCSC | 92 | 5 | 33590 | gene;island | 0.897507 | 0.348200 | KAAG1; DCDC2 (margin: KAAG1; RNU6-391P; DCDC2) | 3.88 | 7.94e-03 | 3.19e-02 |
|  | 1 | 44870976 | 44871634 | PDFSTATSENSEMBLUCSC | 659 | 7 | 489 | gene | 0.266203 | 0.015093 | RNF220 (margin: RNF220) | 3.88 | 2.33e-03 | 1.52e-02 |
|  | 11 | 75379432 | 75379624 | PDFSTATSENSEMBLUCSC | 193 | 7 | 8665 | island | 0.559345 | 0.941002 | MAP6 (margin: MAP6) | 3.88 | 5.83e-04 | 6.47e-03 |
|  | 5 | 178684416 | 178684658 | PDFSTATSENSEMBLUCSC | 243 | 4 | 31931 | gene;island | 0.659136 | 0.966107 | Not queried | 3.88 | 2.86e-02 | 6.12e-02 |
|  | 10 | 3109361 | 3109625 | PDFSTATSENSEMBLUCSC | 265 | 4 | 4917 | tss | 0.237771 | 0.012743 | Not queried | 3.88 | 5.71e-02 | 1.00e-01 |
|  | 10 | 25465219 | 25465503 | PDFSTATSENSEMBLUCSC | 285 | 7 | 4992 | tss | 0.363940 | 0.943761 | Not queried | 3.88 | 1.28e-01 | 1.87e-01 |
|  | 4 | 3773815 | 3773996 | PDFSTATSENSEMBLUCSC | 182 | 5 | 30855 | island | 0.476853 | 0.875236 | Not queried | 3.88 | 5.56e-02 | 1.00e-01 |
|  | 10 | 15130387 | 15130767 | PDFSTATSENSEMBLUCSC | 381 | 4 | 4511 | gene | 0.263398 | 0.032482 | Not queried | 3.88 | 2.86e-02 | 6.12e-02 |
|  | 2 | 175199259 | 175199828 | PDFSTATSENSEMBLUCSC | 570 | 12 | 24874 | island | 0.601333 | 0.976828 | SP9; AC018470.1 (margin: AC018470.4; SP9; AC018470.1) | 3.88 | 7.17e-05 | 1.92e-03 |
|  | 19 | 51017855 | 51018014 | PDFSTATSENSEMBLUCSC | 160 | 4 | 20980 | tss;island | 0.388197 | 0.819536 | Not queried | 3.88 | 2.86e-02 | 6.12e-02 |
|  | 1 | 150521958 | 150522367 | PDFSTATSENSEMBLUCSC | 410 | 4 | 740 | gene | 0.082524 | 0.433134 | Not queried | 3.88 | 3.43e-01 | 3.84e-01 |
|  | 17 | 12569089 | 12569487 | PDFSTATSENSEMBLUCSC | 399 | 11 | 18028 | island | 0.371737 | 0.861079 | MYOCD (margin: MYOCD; AC005358.3) | 3.87 | 1.90e-04 | 3.21e-03 |
|  | 17 | 12569089 | 12569204 | PDFSTATSENSEMBLUCSC | 116 | 7 | 17141 | tss | 0.579174 | 0.928174 | MYOCD (margin: MYOCD; AC005358.3) | 3.87 | 5.83e-04 | 6.47e-03 |
|  | 11 | 1229901 | 1230144 | PDFSTATSENSEMBLUCSC | 244 | 4 | 7907 | island | 0.382031 | 0.935348 | Not queried | 3.87 | 2.00e-01 | 2.55e-01 |
|  | 1 | 27480543 | 27480849 | PDFSTATSENSEMBLUCSC | 307 | 4 | 350 | gene;island | 0.492214 | 0.950620 | Not queried | 3.87 | 2.86e-02 | 6.12e-02 |
|  | 1 | 11042161 | 11042602 | PDFSTATSENSEMBLUCSC | 442 | 5 | 1240 | tss | 0.614628 | 0.958792 | Not queried | 3.87 | 9.52e-02 | 1.53e-01 |
|  | 12 | 85306323 | 85307152 | PDFSTATSENSEMBLUCSC | 830 | 13 | 10656 | island | 0.357884 | 0.945460 | SLC6A15 (margin: SLC6A15) | 3.87 | 5.23e-05 | 1.45e-03 |
|  | 7 | 4118583 | 4118868 | PDFSTATSENSEMBLUCSC | 286 | 5 | 36826 | gene | 0.393886 | 0.876023 | SDK1 (margin: SDK1) | 3.87 | 7.94e-03 | 3.19e-02 |
|  | 1 | 155948550 | 155949012 | PDFSTATSENSEMBLUCSC | 463 | 5 | 2008 | tss;island | 0.005045 | 0.081293 | Not queried | 3.87 | 3.17e-02 | 6.50e-02 |
|  | 1 | 154539974 | 154540535 | PDFSTATSENSEMBLUCSC | 562 | 8 | 3794 | island | 0.052027 | 0.564580 | Not queried | 3.87 | 8.30e-02 | 1.40e-01 |
|  | 5 | 63985282 | 63985489 | PDFSTATSENSEMBLUCSC | 208 | 4 | 32090 | tss;island | 0.459703 | 0.884288 | Not queried | 3.87 | 1.14e-01 | 1.68e-01 |
|  | 4 | 114682438 | 114682885 | PDFSTATSENSEMBLUCSC | 448 | 4 | 30106 | gene;island | 0.067015 | 0.441475 | Not queried | 3.87 | 2.86e-02 | 6.12e-02 |
|  | 11 | 313120 | 313624 | PDFSTATSENSEMBLUCSC | 505 | 6 | 7008 | tss;island | 0.280096 | 0.890860 | Not queried | 3.87 | 2.60e-02 | 6.12e-02 |
|  | 2 | 134326055 | 134326366 | PDFSTATSENSEMBLUCSC | 312 | 5 | 23641 | tss | 0.055089 | 0.454526 | Not queried | 3.87 | 9.52e-02 | 1.53e-01 |
|  | 2 | 176987161 | 176987605 | PDFSTATSENSEMBLUCSC | 445 | 8 | 24902 | island | 0.843578 | 0.986353 | HOXD9; HOXD-AS2 (margin: HOXD10; HOXD9; HOXD8; HOXD-AS2) | 3.87 | 3.11e-04 | 4.77e-03 |
|  | 10 | 23983496 | 23983538 | PDFSTATSENSEMBLUCSC | 43 | 4 | 4989 | tss | 0.724873 | 0.972042 | Not queried | 3.87 | 2.86e-02 | 6.12e-02 |
|  | 5 | 126409198 | 126409573 | PDFSTATSENSEMBLUCSC | 376 | 9 | 32255 | tss | 0.726440 | 0.083893 | Not queried | 3.87 | 1.13e-01 | 1.68e-01 |
|  | 19 | 53696623 | 53696886 | PDFSTATSENSEMBLUCSC | 264 | 6 | 21025 | tss | 0.619783 | 0.898911 | Not queried | 3.87 | 9.31e-02 | 1.53e-01 |
|  | 15 | 37180325 | 37181087 | PDFSTATSENSEMBLUCSC | 763 | 6 | 14010 | island | 0.925103 | 0.407079 | Not queried | 3.86 | 2.60e-02 | 6.12e-02 |
|  | 10 | 94452236 | 94452554 | PDFSTATSENSEMBLUCSC | 319 | 4 | 4677 | gene;island | 0.980470 | 0.722942 | Not queried | 3.86 | 1.14e-01 | 1.68e-01 |
|  | 19 | 10691765 | 10692029 | PDFSTATSENSEMBLUCSC | 265 | 6 | 19816 | gene;island | 0.037853 | 0.267735 | Not queried | 3.86 | 2.40e-01 | 2.95e-01 |
|  | 17 | 46691820 | 46692592 | PDFSTATSENSEMBLUCSC | 773 | 12 | 18490 | island | 0.847602 | 0.330640 | Not queried | 3.86 | 2.84e-02 | 6.12e-02 |
|  | 16 | 30960055 | 30960343 | PDFSTATSENSEMBLUCSC | 289 | 7 | 15199 | tss | 0.164653 | 0.024703 | Not queried | 3.86 | 1.75e-02 | 5.79e-02 |
|  | 11 | 69590113 | 69590580 | PDFSTATSENSEMBLUCSC | 468 | 8 | 8597 | island | 0.518482 | 0.955893 | FGF4 (margin: AP001888.1; FGF4) | 3.86 | 1.55e-04 | 2.71e-03 |
|  | 11 | 36422377 | 36422615 | PDFSTATSENSEMBLUCSC | 239 | 5 | 6585 | gene | 0.644785 | 0.968642 | PRR5L (margin: PRR5L) | 3.86 | 7.94e-03 | 3.19e-02 |
|  | 16 | 1226067 | 1226302 | PDFSTATSENSEMBLUCSC | 236 | 4 | 14558 | gene;island | 0.316367 | 0.770916 | Not queried | 3.86 | 2.86e-02 | 6.12e-02 |
|  | 7 | 43151725 | 43152254 | PDFSTATSENSEMBLUCSC | 530 | 7 | 38440 | island | 0.530387 | 0.838565 | Not queried | 3.86 | 9.73e-02 | 1.55e-01 |
|  | 3 | 13114343 | 13114803 | PDFSTATSENSEMBLUCSC | 461 | 6 | 28901 | island | 0.530338 | 0.954589 | IQSEC1 (margin: IQSEC1) | 3.86 | 8.66e-03 | 3.36e-02 |
|  | 10 | 131694575 | 131694856 | PDFSTATSENSEMBLUCSC | 282 | 5 | 4835 | gene;island | 0.224307 | 0.921025 | Not queried | 3.86 | 9.52e-02 | 1.53e-01 |
|  | 4 | 17513197 | 17513834 | PDFSTATSENSEMBLUCSC | 638 | 5 | 29961 | gene | 0.042031 | 0.063444 | Not queried | 3.86 | 3.10e-01 | 3.66e-01 |
|  | 5 | 131892242 | 131892523 | PDFSTATSENSEMBLUCSC | 282 | 5 | 32272 | tss | 0.008746 | 0.385057 | Not queried | 3.85 | 3.10e-01 | 3.66e-01 |
|  | 3 | 48697409 | 48697823 | PDFSTATSENSEMBLUCSC | 415 | 8 | 27769 | gene;island | 0.667706 | 0.939987 | CELSR3 (margin: RP11-148G20.1; CELSR3; NCKIPSD) | 3.85 | 4.66e-03 | 2.68e-02 |
|  | 20 | 61885236 | 61885291 | PDFSTATSENSEMBLUCSC | 56 | 6 | 25892 | tss | 0.330385 | 0.138252 | Not queried | 3.85 | 2.40e-01 | 2.95e-01 |
|  | 16 | 2879944 | 2880149 | PDFSTATSENSEMBLUCSC | 206 | 4 | 15037 | tss | 0.636407 | 0.243345 | Not queried | 3.85 | 2.86e-02 | 6.12e-02 |
|  | 10 | 104211319 | 104211488 | PDFSTATSENSEMBLUCSC | 170 | 5 | 5325 | tss | 0.081861 | 0.282847 | Not queried | 3.85 | 5.56e-02 | 1.00e-01 |
|  | 2 | 25142117 | 25142523 | PDFSTATSENSEMBLUCSC | 407 | 6 | 23228 | tss | 0.006536 | 0.279909 | Not queried | 3.85 | 1.52e-02 | 5.36e-02 |
|  | 12 | 91572142 | 91572276 | PDFSTATSENSEMBLUCSC | 135 | 4 | 9241 | gene | 0.074842 | 0.533706 | Not queried | 3.85 | 3.43e-01 | 3.84e-01 |
|  | 19 | 58280728 | 58281117 | PDFSTATSENSEMBLUCSC | 390 | 10 | 22483 | island | 0.050833 | 0.426139 | Not queried | 3.85 | 1.85e-02 | 6.12e-02 |
|  | 6 | 32942063 | 32942808 | PDFSTATSENSEMBLUCSC | 746 | 10 | 34171 | gene;island | 0.004822 | 0.058971 | BRD2; XXbac-BPG181M17.6 (margin: BRD2; BRD2-IT1; HLA-DMA; XXbac-BPG181M17.6) | 3.85 | 3.89e-03 | 2.43e-02 |
|  | 12 | 115122028 | 115122304 | PDFSTATSENSEMBLUCSC | 277 | 6 | 10036 | tss;island | 0.027717 | 0.009934 | Not queried | 3.84 | 1.32e-01 | 1.89e-01 |
|  | 3 | 196366191 | 196366669 | PDFSTATSENSEMBLUCSC | 479 | 8 | 29810 | island | 0.638609 | 0.942516 | NRROS; PIGX (margin: NRROS; PIGX; AC023797.1) | 3.84 | 2.95e-03 | 1.88e-02 |
|  | 22 | 50451054 | 50451414 | PDFSTATSENSEMBLUCSC | 361 | 5 | 27610 | island | 0.648591 | 0.142186 | Not queried | 3.84 | 3.17e-02 | 6.50e-02 |
|  | 10 | 7453452 | 7453594 | PDFSTATSENSEMBLUCSC | 143 | 6 | 4936 | tss | 0.419142 | 0.060643 | Not queried | 3.84 | 4.11e-02 | 8.14e-02 |
|  | 9 | 72873345 | 72873728 | PDFSTATSENSEMBLUCSC | 384 | 4 | 40815 | tss;island | 0.206214 | 0.004910 | Not queried | 3.84 | 4.86e-01 | 5.11e-01 |
|  | 17 | 41739130 | 41739326 | PDFSTATSENSEMBLUCSC | 197 | 4 | 17392 | tss | 0.185131 | 0.692569 | Not queried | 3.84 | 1.14e-01 | 1.68e-01 |
|  | 3 | 131245815 | 131246097 | PDFSTATSENSEMBLUCSC | 283 | 4 | 29482 | island | 0.675781 | 0.970809 | Not queried | 3.84 | 2.86e-02 | 6.12e-02 |
|  | 3 | 179754483 | 179754761 | PDFSTATSENSEMBLUCSC | 279 | 8 | 29683 | island | 0.906395 | 0.387603 | PEX5L (margin: PEX5L) | 3.84 | 1.86e-03 | 1.45e-02 |
|  | 7 | 37956554 | 37957021 | PDFSTATSENSEMBLUCSC | 468 | 9 | 37542 | tss | 0.160863 | 0.040249 | Not queried | 3.84 | 9.39e-02 | 1.53e-01 |
|  | 19 | 10381577 | 10382048 | PDFSTATSENSEMBLUCSC | 472 | 4 | 19811 | gene | 0.391693 | 0.043065 | Not queried | 3.84 | 5.71e-02 | 1.00e-01 |
|  | 10 | 42863508 | 42863594 | PDFSTATSENSEMBLUCSC | 87 | 4 | 5039 | tss | 0.265879 | 0.783226 | Not queried | 3.84 | 2.86e-02 | 6.12e-02 |
|  | 1 | 14219312 | 14219825 | PDFSTATSENSEMBLUCSC | 514 | 4 | 2711 | island | 0.383836 | 0.032825 | Not queried | 3.84 | 2.86e-02 | 6.12e-02 |
|  | 10 | 71087834 | 71088038 | PDFSTATSENSEMBLUCSC | 205 | 4 | 4613 | gene | 0.616428 | 0.954724 | Not queried | 3.84 | 2.86e-02 | 6.12e-02 |
|  | 21 | 42688468 | 42689051 | PDFSTATSENSEMBLUCSC | 584 | 9 | 26687 | island | 0.839102 | 0.986997 | FAM3B (margin: FAM3B) | 3.84 | 8.23e-05 | 2.06e-03 |
|  | 21 | 42688468 | 42688650 | PDFSTATSENSEMBLUCSC | 183 | 5 | 26555 | tss | 0.790298 | 0.986997 | FAM3B (margin: FAM3B) | 3.84 | 7.94e-03 | 3.19e-02 |
|  | 6 | 166908379 | 166908811 | PDFSTATSENSEMBLUCSC | 433 | 5 | 34548 | gene | 0.556677 | 0.966947 | Not queried | 3.84 | 3.10e-01 | 3.66e-01 |
|  | 7 | 88425077 | 88425148 | PDFSTATSENSEMBLUCSC | 72 | 5 | 37693 | tss | 0.462322 | 0.118066 | Not queried | 3.84 | 4.21e-01 | 4.59e-01 |
|  | 20 | 60795362 | 60795465 | PDFSTATSENSEMBLUCSC | 104 | 4 | 25866 | tss;island | 0.218725 | 0.016652 | Not queried | 3.84 | 2.86e-02 | 6.12e-02 |
|  | 10 | 135050619 | 135051351 | PDFSTATSENSEMBLUCSC | 733 | 25 | 5456 | tss | 0.548626 | 0.958874 | VENTX (margin: KNDC1; UTF1; VENTX; MIR202) | 3.83 | 1.86e-10 | 1.13e-07 |
|  | 2 | 124782586 | 124782885 | PDFSTATSENSEMBLUCSC | 300 | 5 | 24679 | island | 0.933456 | 0.501991 | Not queried | 3.83 | 1.59e-02 | 5.36e-02 |
|  | 7 | 75157456 | 75157765 | PDFSTATSENSEMBLUCSC | 310 | 7 | 37654 | tss | 0.007502 | 0.047012 | PMS2P3 (margin: PMS2P3; HIP1) | 3.83 | 2.33e-03 | 1.52e-02 |
|  | 2 | 237076164 | 237076497 | PDFSTATSENSEMBLUCSC | 334 | 4 | 23089 | gene;island | 0.170154 | 0.012047 | Not queried | 3.83 | 1.14e-01 | 1.68e-01 |
|  | 1 | 228473704 | 228473980 | PDFSTATSENSEMBLUCSC | 277 | 5 | 1054 | gene;island | 0.491288 | 0.961735 | OBSCN (margin: OBSCN; RP5-1139B12.2; RP5-1139B12.3; RP5-1139B12.4) | 3.83 | 7.94e-03 | 3.19e-02 |
|  | 15 | 28016437 | 28016654 | PDFSTATSENSEMBLUCSC | 218 | 4 | 13263 | gene;island | 0.539656 | 0.956361 | Not queried | 3.83 | 5.71e-02 | 1.00e-01 |
|  | 17 | 80251443 | 80251697 | PDFSTATSENSEMBLUCSC | 255 | 4 | 18982 | island | 0.433592 | 0.856823 | Not queried | 3.83 | 2.00e-01 | 2.55e-01 |
|  | 7 | 4218743 | 4219174 | PDFSTATSENSEMBLUCSC | 432 | 4 | 36832 | gene | 0.310879 | 0.803019 | Not queried | 3.83 | 5.71e-02 | 1.00e-01 |
|  | 11 | 93583509 | 93583973 | PDFSTATSENSEMBLUCSC | 465 | 8 | 8733 | island | 0.048085 | 0.390377 | VSTM5 (margin: VSTM5) | 3.83 | 1.55e-04 | 2.71e-03 |
|  | 16 | 50581707 | 50582193 | PDFSTATSENSEMBLUCSC | 487 | 5 | 15229 | tss;island | 0.097533 | 0.012639 | Not queried | 3.83 | 3.17e-02 | 6.50e-02 |
|  | 12 | 109531054 | 109531279 | PDFSTATSENSEMBLUCSC | 226 | 4 | 9288 | gene | 0.145379 | 0.005982 | Not queried | 3.83 | 1.14e-01 | 1.68e-01 |
|  | 12 | 85306648 | 85307152 | PDFSTATSENSEMBLUCSC | 505 | 9 | 9898 | tss | 0.357884 | 0.880173 | SLC6A15 (margin: SLC6A15) | 3.83 | 3.99e-03 | 2.47e-02 |
|  | 15 | 68724690 | 68725002 | PDFSTATSENSEMBLUCSC | 313 | 4 | 13759 | tss;island | 0.586666 | 0.952918 | Not queried | 3.83 | 1.14e-01 | 1.68e-01 |
|  | 5 | 1286042 | 1286354 | PDFSTATSENSEMBLUCSC | 313 | 4 | 31534 | gene;island | 0.619761 | 0.984737 | Not queried | 3.83 | 5.71e-02 | 1.00e-01 |
|  | 11 | 75379491 | 75379624 | PDFSTATSENSEMBLUCSC | 134 | 6 | 7570 | tss | 0.551989 | 0.922910 | MAP6 (margin: MAP6) | 3.82 | 2.16e-03 | 1.45e-02 |
|  | 2 | 71017846 | 71018354 | PDFSTATSENSEMBLUCSC | 509 | 5 | 23421 | tss;island | 0.765187 | 0.959199 | Not queried | 3.82 | 3.10e-01 | 3.66e-01 |
|  | 3 | 111717682 | 111718245 | PDFSTATSENSEMBLUCSC | 564 | 8 | 27893 | gene | 0.520577 | 0.957080 | TAGLN3 (margin: ABHD10; TAGLN3) | 3.82 | 1.55e-04 | 2.71e-03 |
|  | 12 | 109535320 | 109535460 | PDFSTATSENSEMBLUCSC | 141 | 6 | 9989 | tss;island | 0.271633 | 0.006166 | Not queried | 3.82 | 4.11e-02 | 8.14e-02 |
|  | 2 | 190445551 | 190446181 | PDFSTATSENSEMBLUCSC | 631 | 6 | 23779 | tss | 0.602972 | 0.973152 | Not queried | 3.82 | 2.60e-02 | 6.12e-02 |
|  | 1 | 47489195 | 47489543 | PDFSTATSENSEMBLUCSC | 349 | 5 | 3228 | island | 0.321170 | 0.927683 | Not queried | 3.82 | 5.56e-02 | 1.00e-01 |
|  | 1 | 43751270 | 43751563 | PDFSTATSENSEMBLUCSC | 294 | 8 | 1558 | tss | 0.104932 | 0.006292 | C1orf210 (margin: TMEM125; C1orf210) | 3.82 | 1.04e-02 | 3.96e-02 |
|  | 1 | 156696747 | 156697041 | PDFSTATSENSEMBLUCSC | 295 | 4 | 828 | gene;island | 0.022139 | 0.267646 | Not queried | 3.82 | 2.00e-01 | 2.55e-01 |
|  | 1 | 15250209 | 15250388 | PDFSTATSENSEMBLUCSC | 180 | 4 | 239 | gene;tss;island | 0.055459 | 0.391811 | Not queried | 3.82 | 2.00e-01 | 2.55e-01 |
|  | 10 | 135342218 | 135342620 | PDFSTATSENSEMBLUCSC | 403 | 4 | 4907 | gene;island | 0.492865 | 0.907550 | Not queried | 3.82 | 2.86e-02 | 6.12e-02 |
|  | 13 | 84456127 | 84456722 | PDFSTATSENSEMBLUCSC | 596 | 9 | 11806 | island | 0.894082 | 0.257688 | SLITRK1 (margin: SLITRK1) | 3.82 | 2.76e-03 | 1.78e-02 |
|  | 11 | 34378797 | 34379053 | PDFSTATSENSEMBLUCSC | 257 | 7 | 8201 | island | 0.173321 | 0.014300 | Not queried | 3.82 | 1.75e-02 | 5.79e-02 |
|  | 10 | 127464440 | 127464762 | PDFSTATSENSEMBLUCSC | 323 | 6 | 5430 | tss | 0.427022 | 0.872654 | Not queried | 3.82 | 2.60e-02 | 6.12e-02 |
|  | 4 | 156587884 | 156588387 | PDFSTATSENSEMBLUCSC | 504 | 8 | 30159 | gene;tss;island | 0.383802 | 0.023016 | GUCY1A3 (margin: GUCY1A3) | 3.82 | 1.55e-04 | 2.71e-03 |
|  | 5 | 17219020 | 17219239 | PDFSTATSENSEMBLUCSC | 220 | 4 | 31572 | gene;island | 0.024370 | 0.289945 | Not queried | 3.82 | 2.86e-02 | 6.12e-02 |
|  | 4 | 46126245 | 46126448 | PDFSTATSENSEMBLUCSC | 204 | 6 | 30371 | tss | 0.076432 | 0.023060 | Not queried | 3.82 | 3.10e-01 | 3.66e-01 |
|  | 20 | 33865797 | 33866020 | PDFSTATSENSEMBLUCSC | 224 | 4 | 26121 | island | 0.894240 | 0.397623 | Not queried | 3.81 | 2.86e-02 | 6.12e-02 |
|  | 1 | 220101698 | 220102043 | PDFSTATSENSEMBLUCSC | 346 | 8 | 4211 | island | 0.589472 | 0.949138 | SLC30A10 (margin: SLC30A10) | 3.81 | 6.22e-04 | 6.71e-03 |
|  | 1 | 220101698 | 220101962 | PDFSTATSENSEMBLUCSC | 265 | 6 | 1014 | gene | 0.656775 | 0.956432 | SLC30A10 (margin: SLC30A10) | 3.81 | 2.16e-03 | 1.45e-02 |
|  | 10 | 133948124 | 133948645 | PDFSTATSENSEMBLUCSC | 522 | 6 | 4851 | gene;island | 0.559883 | 0.956062 | Not queried | 3.81 | 1.32e-01 | 1.89e-01 |
|  | 6 | 30923241 | 30924004 | PDFSTATSENSEMBLUCSC | 764 | 17 | 35746 | island | 0.493822 | 0.909337 | DPCR1; HCG21 (margin: DPCR1; HCG21) | 3.81 | 9.73e-06 | 4.82e-04 |
|  | 17 | 44896147 | 44896223 | PDFSTATSENSEMBLUCSC | 77 | 6 | 17435 | tss | 0.267941 | 0.039237 | WNT3 (margin: WNT3) | 3.81 | 2.16e-03 | 1.45e-02 |
|  | 18 | 158229 | 158463 | PDFSTATSENSEMBLUCSC | 235 | 5 | 19148 | tss | 0.141552 | 0.014741 | Not queried | 3.81 | 3.10e-01 | 3.66e-01 |
|  | 1 | 156783633 | 156784152 | PDFSTATSENSEMBLUCSC | 520 | 6 | 831 | gene;island | 0.100480 | 0.550567 | Not queried | 3.81 | 4.11e-02 | 8.14e-02 |
|  | 4 | 165304842 | 165305334 | PDFSTATSENSEMBLUCSC | 493 | 7 | 30664 | tss;island | 0.329067 | 0.851615 | Not queried | 3.81 | 1.75e-02 | 5.79e-02 |
|  | 6 | 75918463 | 75918801 | PDFSTATSENSEMBLUCSC | 339 | 4 | 36267 | island | 0.360355 | 0.021327 | Not queried | 3.81 | 6.86e-01 | 6.99e-01 |
|  | 8 | 129061048 | 129061382 | PDFSTATSENSEMBLUCSC | 335 | 4 | 39431 | gene;tss | 0.972464 | 0.814956 | Not queried | 3.81 | 2.86e-02 | 6.12e-02 |
|  | 13 | 112725903 | 112726124 | PDFSTATSENSEMBLUCSC | 222 | 4 | 11913 | island | 0.689952 | 0.977743 | Not queried | 3.81 | 5.71e-02 | 1.00e-01 |
|  | 12 | 109826364 | 109826496 | PDFSTATSENSEMBLUCSC | 133 | 4 | 9991 | tss | 0.787675 | 0.981082 | Not queried | 3.81 | 1.14e-01 | 1.68e-01 |
|  | 10 | 31320775 | 31321013 | PDFSTATSENSEMBLUCSC | 239 | 7 | 5647 | island | 0.398008 | 0.005015 | Not queried | 3.81 | 1.28e-01 | 1.87e-01 |
|  | 10 | 12648032 | 12648526 | PDFSTATSENSEMBLUCSC | 495 | 4 | 4500 | gene | 0.828990 | 0.352646 | Not queried | 3.81 | 1.14e-01 | 1.68e-01 |
|  | 6 | 32179862 | 32180305 | PDFSTATSENSEMBLUCSC | 444 | 6 | 34092 | gene | 0.673926 | 0.855926 | Not queried | 3.81 | 1.32e-01 | 1.89e-01 |
|  | 11 | 32460587 | 32460980 | PDFSTATSENSEMBLUCSC | 394 | 4 | 6570 | gene;island | 0.712081 | 0.963874 | Not queried | 3.81 | 5.71e-02 | 1.00e-01 |
|  | 9 | 101705792 | 101706041 | PDFSTATSENSEMBLUCSC | 250 | 4 | 40826 | tss;island | 0.729542 | 0.972469 | Not queried | 3.81 | 2.86e-02 | 6.12e-02 |
|  | 12 | 126018024 | 126018364 | PDFSTATSENSEMBLUCSC | 341 | 5 | 9404 | gene;island | 0.301210 | 0.009211 | Not queried | 3.81 | 3.17e-02 | 6.50e-02 |
|  | 11 | 44341361 | 44341831 | PDFSTATSENSEMBLUCSC | 471 | 5 | 8227 | island | 0.771222 | 0.979388 | (margin: ALX4) | 3.81 | 7.94e-03 | 3.19e-02 |
|  | 6 | 50803820 | 50803945 | PDFSTATSENSEMBLUCSC | 126 | 4 | 34365 | gene | 0.692558 | 0.961502 | Not queried | 3.81 | 2.86e-02 | 6.12e-02 |
|  | 16 | 58059105 | 58059703 | PDFSTATSENSEMBLUCSC | 599 | 10 | 15971 | island | 0.073732 | 0.007173 | Not queried | 3.81 | 2.88e-02 | 6.16e-02 |
|  | 1 | 20692812 | 20693027 | PDFSTATSENSEMBLUCSC | 216 | 4 | 2791 | island | 0.495766 | 0.915583 | Not queried | 3.81 | 2.86e-02 | 6.12e-02 |
|  | 10 | 114043123 | 114043435 | PDFSTATSENSEMBLUCSC | 313 | 5 | 5364 | tss | 0.112513 | 0.646247 | Not queried | 3.80 | 9.52e-02 | 1.53e-01 |
|  | 6 | 32171786 | 32172151 | PDFSTATSENSEMBLUCSC | 366 | 8 | 34088 | gene | 0.173519 | 0.674541 | NOTCH4 (margin: GPSM3; NOTCH4) | 3.80 | 6.22e-04 | 6.71e-03 |
|  | 17 | 57642628 | 57643004 | PDFSTATSENSEMBLUCSC | 377 | 10 | 18611 | island | 0.957909 | 0.632673 | Not queried | 3.80 | 2.47e-01 | 3.03e-01 |
|  | 11 | 68611260 | 68611596 | PDFSTATSENSEMBLUCSC | 337 | 4 | 8579 | island | 0.311315 | 0.015443 | Not queried | 3.80 | 5.71e-02 | 1.00e-01 |
|  | 20 | 60540388 | 60540747 | PDFSTATSENSEMBLUCSC | 360 | 4 | 26309 | island | 0.382902 | 0.900562 | Not queried | 3.80 | 2.86e-02 | 6.12e-02 |
|  | 8 | 1495025 | 1495332 | PDFSTATSENSEMBLUCSC | 308 | 5 | 39166 | gene;island | 0.053843 | 0.441099 | Not queried | 3.80 | 3.10e-01 | 3.66e-01 |
|  | 15 | 79103007 | 79103221 | PDFSTATSENSEMBLUCSC | 215 | 4 | 13441 | gene;island | 0.103803 | 0.602366 | Not queried | 3.80 | 2.86e-02 | 6.12e-02 |
|  | 7 | 121784102 | 121784596 | PDFSTATSENSEMBLUCSC | 495 | 9 | 38868 | island | 0.050096 | 0.493100 | AASS (margin: AASS) | 3.80 | 2.88e-04 | 4.58e-03 |
|  | 2 | 60779189 | 60779652 | PDFSTATSENSEMBLUCSC | 464 | 5 | 22693 | gene;island | 0.296181 | 0.042204 | Not queried | 3.80 | 3.17e-02 | 6.50e-02 |
|  | 1 | 3568669 | 3568722 | PDFSTATSENSEMBLUCSC | 54 | 6 | 1183 | tss;island | 0.356578 | 0.039575 | TP73; WRAP73 (margin: TP73; WRAP73; RP5-1092A11.5) | 3.80 | 2.16e-03 | 1.45e-02 |
|  | 20 | 2781316 | 2781685 | PDFSTATSENSEMBLUCSC | 370 | 8 | 25551 | tss | 0.736049 | 0.975281 | CPXM1 (margin: CPXM1) | 3.80 | 3.11e-04 | 4.77e-03 |
|  | 2 | 26785834 | 26786046 | PDFSTATSENSEMBLUCSC | 213 | 4 | 22615 | gene;island | 0.493936 | 0.902407 | Not queried | 3.80 | 2.86e-02 | 6.12e-02 |
|  | 7 | 100081329 | 100081535 | PDFSTATSENSEMBLUCSC | 207 | 6 | 37785 | tss;island | 0.034799 | 0.006501 | Not queried | 3.80 | 6.49e-02 | 1.11e-01 |
|  | 18 | 44557635 | 44557819 | PDFSTATSENSEMBLUCSC | 185 | 5 | 19105 | gene;tss;island | 0.296902 | 0.902084 | Not queried | 3.80 | 5.56e-02 | 1.00e-01 |
|  | 17 | 29032782 | 29033187 | PDFSTATSENSEMBLUCSC | 406 | 4 | 18179 | island | 0.824730 | 0.985486 | Not queried | 3.80 | 2.86e-02 | 6.12e-02 |
|  | 15 | 29499226 | 29499526 | PDFSTATSENSEMBLUCSC | 301 | 5 | 13270 | gene | 0.694966 | 0.983560 | FAM189A1 (margin: FAM189A1) | 3.80 | 7.94e-03 | 3.19e-02 |
|  | 4 | 55093834 | 55094011 | PDFSTATSENSEMBLUCSC | 178 | 4 | 30400 | tss | 0.650024 | 0.974355 | Not queried | 3.80 | 2.86e-02 | 6.12e-02 |
|  | 1 | 165324679 | 165325231 | PDFSTATSENSEMBLUCSC | 553 | 7 | 3927 | island | 0.627666 | 0.961335 | Not queried | 3.80 | 1.75e-02 | 5.79e-02 |
|  | 15 | 90437628 | 90437891 | PDFSTATSENSEMBLUCSC | 264 | 5 | 13893 | tss | 0.144950 | 0.015416 | Not queried | 3.80 | 1.51e-01 | 2.11e-01 |
|  | 11 | 30605411 | 30605797 | PDFSTATSENSEMBLUCSC | 387 | 5 | 6551 | gene;island | 0.630225 | 0.929240 | RP5-1024C24.1; MPPED2 (margin: RP5-1024C24.1; MPPED2) | 3.80 | 7.94e-03 | 3.19e-02 |
|  | 2 | 39893078 | 39893520 | PDFSTATSENSEMBLUCSC | 443 | 6 | 22654 | gene;island | 0.782332 | 0.986842 | AC007246.3; TMEM178A (margin: AC007246.3; TMEM178A) | 3.80 | 4.33e-03 | 2.52e-02 |
|  | 7 | 42277347 | 42277410 | PDFSTATSENSEMBLUCSC | 64 | 4 | 37551 | tss;island | 0.102081 | 0.012097 | Not queried | 3.80 | 2.86e-02 | 6.12e-02 |
|  | 6 | 32363084 | 32363325 | PDFSTATSENSEMBLUCSC | 242 | 9 | 34105 | gene | 0.312437 | 0.963162 | HCG23; BTNL2 (margin: HCG23; BTNL2) | 3.79 | 7.77e-03 | 3.19e-02 |
|  | 13 | 24144483 | 24144682 | PDFSTATSENSEMBLUCSC | 200 | 4 | 11340 | tss | 0.382533 | 0.033761 | Not queried | 3.79 | 3.43e-01 | 3.84e-01 |
|  | 12 | 108297339 | 108297742 | PDFSTATSENSEMBLUCSC | 404 | 4 | 10766 | island | 0.268502 | 0.029532 | Not queried | 3.79 | 2.00e-01 | 2.55e-01 |
|  | 18 | 55019621 | 55019711 | PDFSTATSENSEMBLUCSC | 91 | 4 | 19289 | tss | 0.561990 | 0.840610 | Not queried | 3.79 | 1.14e-01 | 1.68e-01 |
|  | 16 | 2261069 | 2261237 | PDFSTATSENSEMBLUCSC | 169 | 7 | 15021 | tss | 0.603656 | 0.954671 | MLST8; RP11-304L19.8; BRICD5; PGP (margin: MLST8; RP11-304L19.8; AC009065.3; BRICD5; PGP) | 3.79 | 5.83e-04 | 6.47e-03 |
|  | 11 | 78387190 | 78387569 | PDFSTATSENSEMBLUCSC | 380 | 7 | 6846 | gene;island | 0.629494 | 0.964665 | TENM4 (margin: TENM4) | 3.79 | 6.99e-03 | 3.19e-02 |
|  | 17 | 80621636 | 80621858 | PDFSTATSENSEMBLUCSC | 223 | 4 | 16955 | gene;island | 0.735329 | 0.974871 | Not queried | 3.79 | 5.71e-02 | 1.00e-01 |
|  | 6 | 168720476 | 168720679 | PDFSTATSENSEMBLUCSC | 204 | 4 | 35348 | tss | 0.275382 | 0.833605 | Not queried | 3.79 | 2.86e-02 | 6.12e-02 |
|  | 17 | 77216389 | 77216699 | PDFSTATSENSEMBLUCSC | 311 | 4 | 16838 | gene | 0.241237 | 0.787755 | Not queried | 3.79 | 5.71e-02 | 1.00e-01 |
|  | 15 | 78556834 | 78557094 | PDFSTATSENSEMBLUCSC | 261 | 6 | 13437 | gene;tss;island | 0.079483 | 0.542311 | DNAJA4; RP11-762H8.3 (margin: DNAJA4; RP11-762H8.3) | 3.79 | 2.16e-03 | 1.45e-02 |
|  | 7 | 50132903 | 50133212 | PDFSTATSENSEMBLUCSC | 310 | 8 | 37593 | tss | 0.765775 | 0.967611 | ZPBP (margin: C7orf72; ZPBP) | 3.79 | 2.95e-03 | 1.88e-02 |
|  | 7 | 1443496 | 1443857 | PDFSTATSENSEMBLUCSC | 362 | 5 | 38102 | island | 0.102767 | 0.790829 | Not queried | 3.79 | 3.17e-02 | 6.50e-02 |
|  | 6 | 10420696 | 10421069 | PDFSTATSENSEMBLUCSC | 374 | 5 | 34626 | tss;island | 0.928177 | 0.442408 | Not queried | 3.79 | 1.51e-01 | 2.11e-01 |
|  | 11 | 67141628 | 67142030 | PDFSTATSENSEMBLUCSC | 403 | 8 | 6749 | gene;tss;island | 0.006607 | 0.098417 | AP003419.11; CLCF1 (margin: RN7SKP239; AP003419.11; CLCF1) | 3.79 | 2.95e-03 | 1.88e-02 |
|  | 19 | 4173254 | 4173482 | PDFSTATSENSEMBLUCSC | 229 | 4 | 21366 | island | 0.108310 | 0.027941 | Not queried | 3.79 | 3.43e-01 | 3.84e-01 |
|  | 9 | 139096738 | 139097029 | PDFSTATSENSEMBLUCSC | 292 | 4 | 40974 | island | 0.639064 | 0.960696 | Not queried | 3.79 | 2.86e-02 | 6.12e-02 |
|  | 6 | 168393930 | 168394198 | PDFSTATSENSEMBLUCSC | 269 | 5 | 36653 | island | 0.234588 | 0.952324 | Not queried | 3.78 | 3.10e-01 | 3.66e-01 |
|  | 8 | 142401373 | 142401867 | PDFSTATSENSEMBLUCSC | 495 | 4 | 40622 | island | 0.409639 | 0.089941 | Not queried | 3.78 | 5.71e-02 | 1.00e-01 |
|  | 14 | 53258566 | 53258684 | PDFSTATSENSEMBLUCSC | 119 | 5 | 12400 | tss;island | 0.001458 | 0.004265 | Not queried | 3.78 | 2.22e-01 | 2.76e-01 |
|  | 17 | 48585858 | 48586147 | PDFSTATSENSEMBLUCSC | 290 | 4 | 16688 | gene;island | 0.780946 | 0.980601 | Not queried | 3.78 | 2.86e-02 | 6.12e-02 |
|  | 11 | 85522279 | 85522637 | PDFSTATSENSEMBLUCSC | 359 | 8 | 7605 | tss | 0.520712 | 0.091491 | SYTL2 (margin: SYTL2) | 3.78 | 2.95e-03 | 1.88e-02 |
|  | 15 | 100882020 | 100882647 | PDFSTATSENSEMBLUCSC | 628 | 11 | 14507 | island | 0.230900 | 0.015438 | ADAMTS17 (margin: ADAMTS17; SPATA41) | 3.78 | 5.53e-04 | 6.47e-03 |
|  | 13 | 40746370 | 40746648 | PDFSTATSENSEMBLUCSC | 279 | 4 | 11685 | island | 0.066090 | 0.584340 | Not queried | 3.78 | 5.71e-02 | 1.00e-01 |
|  | 17 | 56405948 | 56406294 | PDFSTATSENSEMBLUCSC | 347 | 4 | 18598 | island | 0.254898 | 0.677550 | Not queried | 3.78 | 4.86e-01 | 5.11e-01 |
|  | 3 | 120004186 | 120004483 | PDFSTATSENSEMBLUCSC | 298 | 4 | 29388 | island | 0.827555 | 0.265090 | Not queried | 3.78 | 3.43e-01 | 3.84e-01 |
|  | 1 | 118166013 | 118166402 | PDFSTATSENSEMBLUCSC | 390 | 4 | 712 | gene | 0.707962 | 0.971272 | Not queried | 3.78 | 2.86e-02 | 6.12e-02 |
|  | 6 | 6320614 | 6320895 | PDFSTATSENSEMBLUCSC | 282 | 4 | 33546 | gene | 0.025221 | 0.240377 | Not queried | 3.78 | 1.14e-01 | 1.68e-01 |
|  | 1 | 18809531 | 18809764 | PDFSTATSENSEMBLUCSC | 234 | 4 | 272 | gene;island | 0.715894 | 0.975838 | Not queried | 3.78 | 2.86e-02 | 6.12e-02 |
|  | 8 | 24772878 | 24773268 | PDFSTATSENSEMBLUCSC | 391 | 4 | 39247 | gene;island | 0.770204 | 0.977620 | Not queried | 3.78 | 6.86e-01 | 6.99e-01 |
|  | 6 | 168120556 | 168120876 | PDFSTATSENSEMBLUCSC | 321 | 4 | 36646 | island | 0.597926 | 0.937675 | Not queried | 3.78 | 2.86e-02 | 6.12e-02 |
|  | 6 | 34524095 | 34524290 | PDFSTATSENSEMBLUCSC | 196 | 4 | 34920 | tss | 0.491985 | 0.088792 | Not queried | 3.78 | 2.00e-01 | 2.55e-01 |
|  | 19 | 42440001 | 42440198 | PDFSTATSENSEMBLUCSC | 198 | 4 | 22061 | island | 0.139690 | 0.643947 | Not queried | 3.78 | 2.00e-01 | 2.55e-01 |
|  | 12 | 53693054 | 53693453 | PDFSTATSENSEMBLUCSC | 400 | 8 | 9738 | tss | 0.028521 | 0.313038 | Not queried | 3.78 | 6.50e-02 | 1.11e-01 |
|  | 12 | 1973208 | 1973452 | PDFSTATSENSEMBLUCSC | 245 | 5 | 9001 | gene;island | 0.661413 | 0.972546 | Not queried | 3.78 | 5.56e-02 | 1.00e-01 |
|  | 19 | 11805638 | 11805966 | PDFSTATSENSEMBLUCSC | 329 | 4 | 21581 | island | 0.081684 | 0.547849 | Not queried | 3.77 | 2.86e-02 | 6.12e-02 |
|  | 2 | 190445477 | 190446181 | PDFSTATSENSEMBLUCSC | 705 | 7 | 24955 | island | 0.702217 | 0.974688 | SLC40A1 (margin: SLC40A1) | 3.77 | 1.11e-02 | 4.14e-02 |
|  | 14 | 93650515 | 93650746 | PDFSTATSENSEMBLUCSC | 232 | 4 | 12184 | gene;tss;island | 0.572920 | 0.088704 | Not queried | 3.77 | 3.43e-01 | 3.84e-01 |
|  | 2 | 240279976 | 240280294 | PDFSTATSENSEMBLUCSC | 319 | 5 | 23117 | gene | 0.986121 | 0.885821 | HDAC4 (margin: HDAC4; MIR2467) | 3.77 | 7.94e-03 | 3.19e-02 |
|  | 9 | 74061171 | 74061514 | PDFSTATSENSEMBLUCSC | 344 | 4 | 40892 | island | 0.554440 | 0.040706 | Not queried | 3.77 | 1.14e-01 | 1.68e-01 |
|  | 19 | 15618961 | 15619355 | PDFSTATSENSEMBLUCSC | 395 | 5 | 21709 | island | 0.382653 | 0.043676 | Not queried | 3.77 | 5.48e-01 | 5.70e-01 |
|  | 2 | 75147006 | 75147288 | PDFSTATSENSEMBLUCSC | 283 | 4 | 24453 | island | 0.441679 | 0.087700 | Not queried | 3.77 | 2.86e-02 | 6.12e-02 |
|  | 11 | 70563580 | 70563878 | PDFSTATSENSEMBLUCSC | 299 | 5 | 6795 | gene;island | 0.339084 | 0.942253 | Not queried | 3.77 | 1.59e-02 | 5.36e-02 |
|  | 20 | 2361431 | 2361548 | PDFSTATSENSEMBLUCSC | 118 | 5 | 25543 | tss | 0.744947 | 0.985329 | TGM6 (margin: TGM6) | 3.77 | 7.94e-03 | 3.19e-02 |
|  | 8 | 22408966 | 22409270 | PDFSTATSENSEMBLUCSC | 305 | 7 | 40134 | island | 0.881935 | 0.984110 | Not queried | 3.77 | 5.30e-02 | 1.00e-01 |
|  | 8 | 104310606 | 104310886 | PDFSTATSENSEMBLUCSC | 281 | 6 | 39857 | tss | 0.004542 | 0.036168 | FZD6; RP11-318M2.2 (margin: FZD6; RP11-318M2.2) | 3.77 | 2.16e-03 | 1.45e-02 |
|  | 17 | 73150870 | 73151149 | PDFSTATSENSEMBLUCSC | 280 | 5 | 17645 | tss | 0.007666 | 0.040763 | Not queried | 3.77 | 6.90e-01 | 7.01e-01 |
|  | 11 | 69706831 | 69706981 | PDFSTATSENSEMBLUCSC | 151 | 4 | 8600 | island | 0.653205 | 0.133048 | Not queried | 3.77 | 5.71e-02 | 1.00e-01 |
|  | 4 | 5053085 | 5053596 | PDFSTATSENSEMBLUCSC | 512 | 7 | 30877 | island | 0.486009 | 0.975514 | STK32B (margin: STK32B) | 3.77 | 1.17e-03 | 1.13e-02 |
|  | 10 | 135150367 | 135150517 | PDFSTATSENSEMBLUCSC | 151 | 6 | 6357 | island | 0.090274 | 0.741118 | Not queried | 3.77 | 6.49e-02 | 1.11e-01 |
|  | 22 | 24180995 | 24181270 | PDFSTATSENSEMBLUCSC | 276 | 6 | 27354 | island | 0.789792 | 0.983213 | DERL3 (margin: SMARCB1; DERL3) | 3.77 | 2.16e-03 | 1.45e-02 |
|  | 22 | 24181212 | 24181270 | PDFSTATSENSEMBLUCSC | 59 | 4 | 27012 | tss | 0.765273 | 0.983213 | Not queried | 3.77 | 2.86e-02 | 6.12e-02 |
|  | 1 | 166916762 | 166917267 | PDFSTATSENSEMBLUCSC | 506 | 5 | 872 | gene;island | 0.743743 | 0.953087 | Not queried | 3.77 | 9.52e-02 | 1.53e-01 |
|  | 12 | 117537264 | 117537710 | PDFSTATSENSEMBLUCSC | 447 | 6 | 10043 | tss;island | 0.461026 | 0.078764 | Not queried | 3.77 | 1.52e-02 | 5.36e-02 |
|  | 22 | 17956453 | 17956560 | PDFSTATSENSEMBLUCSC | 108 | 4 | 26943 | tss | 0.653655 | 0.962015 | Not queried | 3.77 | 2.86e-02 | 6.12e-02 |
|  | 10 | 125651370 | 125651726 | PDFSTATSENSEMBLUCSC | 357 | 8 | 6221 | island | 0.634302 | 0.962387 | CPXM2 (margin: CPXM2) | 3.76 | 6.99e-03 | 3.19e-02 |
|  | 4 | 85417312 | 85417755 | PDFSTATSENSEMBLUCSC | 444 | 4 | 30047 | gene;island | 0.767138 | 0.979739 | Not queried | 3.76 | 2.86e-02 | 6.12e-02 |
|  | 10 | 31320885 | 31321013 | PDFSTATSENSEMBLUCSC | 129 | 6 | 5014 | tss | 0.219398 | 0.004210 | Not queried | 3.76 | 1.80e-01 | 2.45e-01 |
|  | 3 | 107148807 | 107149061 | PDFSTATSENSEMBLUCSC | 255 | 4 | 29344 | island | 0.084619 | 0.025815 | Not queried | 3.76 | 2.00e-01 | 2.55e-01 |
|  | 10 | 134499266 | 134499528 | PDFSTATSENSEMBLUCSC | 263 | 5 | 4874 | gene | 0.838763 | 0.984789 | Not queried | 3.76 | 5.56e-02 | 1.00e-01 |
|  | 11 | 65640375 | 65640516 | PDFSTATSENSEMBLUCSC | 142 | 4 | 7447 | tss | 0.741270 | 0.138710 | Not queried | 3.76 | 2.00e-01 | 2.55e-01 |
|  | 17 | 77750983 | 77751636 | PDFSTATSENSEMBLUCSC | 654 | 9 | 17716 | tss;island | 0.202943 | 0.029692 | Not queried | 3.76 | 1.13e-01 | 1.68e-01 |
|  | 10 | 23983496 | 23983686 | PDFSTATSENSEMBLUCSC | 191 | 5 | 5612 | island | 0.730661 | 0.976270 | Not queried | 3.76 | 1.59e-02 | 5.36e-02 |
|  | 19 | 52035146 | 52035287 | PDFSTATSENSEMBLUCSC | 142 | 4 | 20997 | tss | 0.021277 | 0.217901 | Not queried | 3.76 | 2.86e-02 | 6.12e-02 |
|  | 10 | 61469748 | 61469980 | PDFSTATSENSEMBLUCSC | 233 | 8 | 5094 | tss | 0.596866 | 0.040963 | SLC16A9 (margin: SLC16A9) | 3.76 | 6.99e-03 | 3.19e-02 |
|  | 3 | 50341009 | 50341293 | PDFSTATSENSEMBLUCSC | 285 | 4 | 27801 | gene;tss | 0.652317 | 0.977750 | Not queried | 3.76 | 1.14e-01 | 1.68e-01 |
|  | 7 | 150778122 | 150778340 | PDFSTATSENSEMBLUCSC | 219 | 7 | 37989 | tss;island | 0.005714 | 0.234211 | Not queried | 3.76 | 2.62e-02 | 6.12e-02 |
|  | 10 | 14051636 | 14052028 | PDFSTATSENSEMBLUCSC | 393 | 6 | 4508 | gene | 0.603613 | 0.965023 | FRMD4A (margin: RP11-142M10.2; FRMD4A) | 3.76 | 4.33e-03 | 2.52e-02 |
|  | 12 | 109592348 | 109592662 | PDFSTATSENSEMBLUCSC | 315 | 5 | 9290 | gene;island | 0.340910 | 0.021374 | Not queried | 3.75 | 9.52e-02 | 1.53e-01 |
|  | 8 | 54164296 | 54164442 | PDFSTATSENSEMBLUCSC | 147 | 5 | 39701 | tss | 0.555522 | 0.971503 | Not queried | 3.75 | 5.56e-02 | 1.00e-01 |
|  | 10 | 50530557 | 50530868 | PDFSTATSENSEMBLUCSC | 312 | 4 | 4583 | gene;island | 0.305196 | 0.869957 | Not queried | 3.75 | 2.86e-02 | 6.12e-02 |
|  | 19 | 2588241 | 2588629 | PDFSTATSENSEMBLUCSC | 389 | 5 | 19695 | gene;island | 0.882797 | 0.403795 | GNG7 (margin: GNG7) | 3.75 | 7.94e-03 | 3.19e-02 |
|  | 1 | 1098900 | 1099166 | PDFSTATSENSEMBLUCSC | 267 | 4 | 2405 | island | 0.041467 | 0.004290 | Not queried | 3.75 | 1.14e-01 | 1.68e-01 |
|  | 11 | 85521839 | 85522637 | PDFSTATSENSEMBLUCSC | 799 | 12 | 8707 | island | 0.258890 | 0.044898 | SYTL2 (margin: SYTL2) | 3.75 | 2.74e-04 | 4.46e-03 |
|  | 11 | 85521839 | 85522094 | PDFSTATSENSEMBLUCSC | 256 | 4 | 6852 | gene | 0.244105 | 0.008315 | Not queried | 3.75 | 5.71e-02 | 1.00e-01 |
|  | 17 | 46795860 | 46796298 | PDFSTATSENSEMBLUCSC | 439 | 4 | 18497 | island | 0.486130 | 0.957127 | Not queried | 3.75 | 1.14e-01 | 1.68e-01 |
|  | 18 | 72124660 | 72124791 | PDFSTATSENSEMBLUCSC | 132 | 5 | 19322 | tss;island | 0.730806 | 0.168575 | Not queried | 3.75 | 2.22e-01 | 2.76e-01 |
|  | 1 | 3157287 | 3157510 | PDFSTATSENSEMBLUCSC | 224 | 4 | 111 | gene;island | 0.658052 | 0.967616 | Not queried | 3.75 | 5.71e-02 | 1.00e-01 |
|  | 12 | 111473485 | 111473809 | PDFSTATSENSEMBLUCSC | 325 | 4 | 9304 | gene;island | 0.386225 | 0.065316 | Not queried | 3.75 | 2.00e-01 | 2.55e-01 |
|  | 18 | 60987770 | 60988099 | PDFSTATSENSEMBLUCSC | 330 | 10 | 19308 | tss | 0.202407 | 0.007090 | Not queried | 3.74 | 1.47e-02 | 5.36e-02 |
|  | 6 | 30418494 | 30419612 | PDFSTATSENSEMBLUCSC | 1119 | 32 | 35680 | island | 0.463818 | 0.912848 | (margin: ) | 3.74 | 3.51e-10 | 1.71e-07 |
|  | 14 | 64195045 | 64195104 | PDFSTATSENSEMBLUCSC | 60 | 4 | 12451 | tss;island | 0.001238 | 0.010225 | Not queried | 3.74 | 1.14e-01 | 1.68e-01 |
|  | 11 | 31832710 | 31833264 | PDFSTATSENSEMBLUCSC | 555 | 11 | 6562 | gene;island | 0.752034 | 0.973280 | RCN1; PAX6 (margin: RCN1; PAX6) | 3.74 | 2.84e-06 | 1.96e-04 |
|  | 13 | 29105308 | 29105629 | PDFSTATSENSEMBLUCSC | 322 | 4 | 11639 | island | 0.606343 | 0.951645 | Not queried | 3.74 | 1.14e-01 | 1.68e-01 |
|  | 5 | 87985809 | 87986067 | PDFSTATSENSEMBLUCSC | 259 | 4 | 32949 | island | 0.769208 | 0.975339 | Not queried | 3.74 | 2.00e-01 | 2.55e-01 |
|  | 5 | 75377860 | 75378176 | PDFSTATSENSEMBLUCSC | 317 | 5 | 32132 | tss | 0.777883 | 0.968912 | Not queried | 3.74 | 3.17e-02 | 6.50e-02 |
|  | 7 | 27143046 | 27143806 | PDFSTATSENSEMBLUCSC | 761 | 12 | 37488 | tss;island | 0.959201 | 0.612951 | HOXA2; HOXA3 (margin: HOTAIRM1; HOXA-AS2; HOXA1; HOXA2; HOXA3) | 3.74 | 1.03e-04 | 2.47e-03 |
|  | 4 | 140201065 | 140201652 | PDFSTATSENSEMBLUCSC | 588 | 9 | 31313 | island | 0.515442 | 0.934237 | Not queried | 3.74 | 1.90e-01 | 2.55e-01 |
|  | 12 | 114846162 | 114846503 | PDFSTATSENSEMBLUCSC | 342 | 8 | 10841 | island | 0.585134 | 0.942764 | TBX5-AS1; TBX5 (margin: TBX5-AS1; TBX5) | 3.74 | 6.22e-04 | 6.71e-03 |
|  | 1 | 116380316 | 116380575 | PDFSTATSENSEMBLUCSC | 260 | 4 | 704 | gene;island | 0.449105 | 0.876598 | Not queried | 3.74 | 2.86e-02 | 6.12e-02 |
|  | 20 | 44650068 | 44650270 | PDFSTATSENSEMBLUCSC | 203 | 6 | 25786 | tss | 0.632974 | 0.069850 | Not queried | 3.73 | 9.31e-02 | 1.53e-01 |
|  | 19 | 17559217 | 17559666 | PDFSTATSENSEMBLUCSC | 450 | 4 | 21742 | island | 0.043145 | 0.376317 | Not queried | 3.73 | 2.86e-02 | 6.12e-02 |
|  | 5 | 77268331 | 77268888 | PDFSTATSENSEMBLUCSC | 558 | 5 | 32907 | island | 0.821990 | 0.984573 | (margin: ) | 3.73 | 7.94e-03 | 3.19e-02 |
|  | 2 | 175199259 | 175199803 | PDFSTATSENSEMBLUCSC | 545 | 11 | 23723 | tss | 0.603495 | 0.975612 | SP9; AC018470.1 (margin: AC018470.4; SP9; AC018470.1) | 3.73 | 2.75e-04 | 4.46e-03 |
|  | 10 | 115938729 | 115939024 | PDFSTATSENSEMBLUCSC | 296 | 6 | 5375 | tss | 0.957167 | 0.641491 | TDRD1 (margin: TDRD1; C10orf118; MIR2110) | 3.73 | 8.66e-03 | 3.36e-02 |
|  | 15 | 45670865 | 45671347 | PDFSTATSENSEMBLUCSC | 483 | 13 | 13333 | gene;tss;island | 0.869863 | 0.986491 | GATM (margin: GATM) | 3.73 | 2.31e-06 | 1.96e-04 |
|  | 13 | 70682019 | 70682324 | PDFSTATSENSEMBLUCSC | 306 | 4 | 11175 | gene;island | 0.601600 | 0.949120 | Not queried | 3.73 | 1.14e-01 | 1.68e-01 |
|  | 20 | 42875645 | 42875779 | PDFSTATSENSEMBLUCSC | 135 | 6 | 25756 | tss | 0.375155 | 0.880633 | GDAP1L1 (margin: GDAP1L1) | 3.73 | 2.16e-03 | 1.45e-02 |
|  | 9 | 116111377 | 116111733 | PDFSTATSENSEMBLUCSC | 357 | 5 | 40831 | tss;island | 0.179733 | 0.012931 | Not queried | 3.73 | 4.21e-01 | 4.59e-01 |
|  | 3 | 107601860 | 107602025 | PDFSTATSENSEMBLUCSC | 166 | 4 | 28486 | tss | 0.839287 | 0.984427 | Not queried | 3.73 | 2.86e-02 | 6.12e-02 |
|  | 6 | 168613133 | 168613387 | PDFSTATSENSEMBLUCSC | 255 | 4 | 36660 | island | 0.635377 | 0.896645 | Not queried | 3.73 | 1.14e-01 | 1.68e-01 |
|  | 10 | 91295317 | 91295421 | PDFSTATSENSEMBLUCSC | 105 | 6 | 5215 | tss | 0.832077 | 0.987465 | SLC16A12 (margin: SLC16A12) | 3.73 | 2.16e-03 | 1.45e-02 |
|  | 1 | 155225342 | 155225402 | PDFSTATSENSEMBLUCSC | 61 | 4 | 1989 | tss;island | 0.002679 | 0.032053 | Not queried | 3.73 | 2.86e-02 | 6.12e-02 |
|  | 21 | 17961737 | 17962124 | PDFSTATSENSEMBLUCSC | 388 | 4 | 26388 | gene;tss | 0.774148 | 0.982200 | Not queried | 3.73 | 2.86e-02 | 6.12e-02 |
|  | 4 | 979022 | 979389 | PDFSTATSENSEMBLUCSC | 368 | 6 | 29851 | gene;island | 0.861594 | 0.424200 | Not queried | 3.72 | 3.10e-01 | 3.66e-01 |
|  | 5 | 42995123 | 42995527 | PDFSTATSENSEMBLUCSC | 405 | 5 | 32763 | island | 0.603409 | 0.971426 | Not queried | 3.72 | 3.17e-02 | 6.50e-02 |
|  | 19 | 11998457 | 11998660 | PDFSTATSENSEMBLUCSC | 204 | 7 | 20484 | tss | 0.407806 | 0.868448 | ZNF69 (margin: ZNF439; ZNF69) | 3.72 | 5.83e-04 | 6.47e-03 |
|  | 6 | 39693159 | 39693479 | PDFSTATSENSEMBLUCSC | 321 | 7 | 36110 | island | 0.140376 | 0.012292 | KIF6 (margin: KIF6) | 3.72 | 4.08e-03 | 2.47e-02 |
|  | 3 | 148415443 | 148415650 | PDFSTATSENSEMBLUCSC | 208 | 4 | 28653 | tss | 0.464745 | 0.078489 | Not queried | 3.72 | 3.43e-01 | 3.84e-01 |
|  | 4 | 153859106 | 153859560 | PDFSTATSENSEMBLUCSC | 455 | 5 | 31368 | island | 0.442022 | 0.079980 | Not queried | 3.72 | 9.52e-02 | 1.53e-01 |
|  | 4 | 122301573 | 122302007 | PDFSTATSENSEMBLUCSC | 435 | 6 | 30112 | gene;island | 0.646415 | 0.967748 | QRFPR (margin: QRFPR; RP11-364P2.2) | 3.72 | 4.33e-03 | 2.52e-02 |
|  | 1 | 26394201 | 26394593 | PDFSTATSENSEMBLUCSC | 393 | 5 | 1382 | tss | 0.336558 | 0.971744 | Not queried | 3.72 | 5.56e-02 | 1.00e-01 |
|  | 5 | 63461216 | 63461654 | PDFSTATSENSEMBLUCSC | 439 | 8 | 32088 | tss | 0.665439 | 0.097552 | RNF180 (margin: RNF180) | 3.72 | 2.95e-03 | 1.88e-02 |
|  | 2 | 219824293 | 219824401 | PDFSTATSENSEMBLUCSC | 109 | 6 | 25096 | island | 0.047061 | 0.005899 | Not queried | 3.72 | 1.52e-02 | 5.36e-02 |
|  | 7 | 50132541 | 50133212 | PDFSTATSENSEMBLUCSC | 672 | 12 | 38489 | island | 0.765775 | 0.967611 | ZPBP (margin: C7orf72; ZPBP) | 3.72 | 1.83e-03 | 1.45e-02 |
|  | 14 | 103388656 | 103388859 | PDFSTATSENSEMBLUCSC | 204 | 4 | 12647 | tss | 0.532033 | 0.888142 | Not queried | 3.72 | 2.00e-01 | 2.55e-01 |
|  | 10 | 105212236 | 105212813 | PDFSTATSENSEMBLUCSC | 578 | 11 | 5338 | tss | 0.609000 | 0.347970 | RP11-225H22.4; CALHM2; CALHM1 (margin: PDCD11; RP11-225H22.4; CALHM2; CALHM1) | 3.72 | 3.94e-04 | 5.86e-03 |
|  | 10 | 60272754 | 60273225 | PDFSTATSENSEMBLUCSC | 472 | 5 | 5760 | island | 0.695523 | 0.971428 | Not queried | 3.72 | 1.51e-01 | 2.11e-01 |
|  | 3 | 27754391 | 27754675 | PDFSTATSENSEMBLUCSC | 285 | 5 | 28947 | island | 0.355507 | 0.878219 | RP11-222K16.2 (margin: RP11-222K16.2; AC098614.3; EOMES) | 3.72 | 7.94e-03 | 3.19e-02 |
|  | 12 | 8664963 | 8665133 | PDFSTATSENSEMBLUCSC | 171 | 4 | 9554 | tss | 0.273816 | 0.652481 | Not queried | 3.72 | 1.14e-01 | 1.68e-01 |
|  | 10 | 50887472 | 50887934 | PDFSTATSENSEMBLUCSC | 463 | 10 | 5738 | island | 0.497170 | 0.946012 | CHAT; C10orf53 (margin: CHAT; C10orf53) | 3.71 | 3.89e-03 | 2.43e-02 |
|  | 15 | 58357922 | 58357989 | PDFSTATSENSEMBLUCSC | 68 | 4 | 13697 | tss | 0.810456 | 0.977696 | Not queried | 3.71 | 2.86e-02 | 6.12e-02 |
|  | 17 | 4337082 | 4337202 | PDFSTATSENSEMBLUCSC | 121 | 5 | 17023 | tss | 0.358193 | 0.891281 | Not queried | 3.71 | 1.59e-02 | 5.36e-02 |
|  | 7 | 150778201 | 150778340 | PDFSTATSENSEMBLUCSC | 140 | 5 | 37286 | gene | 0.025940 | 0.356509 | FASTK; TMUB1 (margin: SLC4A2; AGAP3; RP11-148K1.12; FASTK; TMUB1) | 3.71 | 7.94e-03 | 3.19e-02 |
|  | 1 | 38470540 | 38470665 | PDFSTATSENSEMBLUCSC | 126 | 4 | 441 | gene;island | 0.006917 | 0.073035 | Not queried | 3.71 | 1.14e-01 | 1.68e-01 |
|  | 2 | 182322501 | 182322841 | PDFSTATSENSEMBLUCSC | 341 | 4 | 22952 | gene;island | 0.653466 | 0.933306 | Not queried | 3.71 | 2.86e-02 | 6.12e-02 |
|  | 2 | 223155841 | 223156136 | PDFSTATSENSEMBLUCSC | 296 | 4 | 23044 | gene;island | 0.698818 | 0.977490 | Not queried | 3.71 | 1.14e-01 | 1.68e-01 |
|  | 10 | 131761150 | 131761386 | PDFSTATSENSEMBLUCSC | 237 | 6 | 4838 | gene;island | 0.679958 | 0.962685 | EBF3 (margin: EBF3) | 3.71 | 2.16e-03 | 1.45e-02 |
|  | 12 | 93967225 | 93967711 | PDFSTATSENSEMBLUCSC | 487 | 4 | 9245 | gene;island | 0.596791 | 0.952096 | Not queried | 3.71 | 2.86e-02 | 6.12e-02 |
|  | 12 | 322514 | 322983 | PDFSTATSENSEMBLUCSC | 470 | 10 | 8991 | gene;tss | 0.337324 | 0.869478 | SLC6A12 (margin: RP11-283I3.2; SLC6A12; SLC6A13) | 3.71 | 4.87e-04 | 6.47e-03 |
|  | 5 | 41510742 | 41510851 | PDFSTATSENSEMBLUCSC | 110 | 4 | 32037 | tss | 0.327376 | 0.031025 | Not queried | 3.71 | 2.86e-02 | 6.12e-02 |
|  | 12 | 131580385 | 131580615 | PDFSTATSENSEMBLUCSC | 231 | 4 | 9425 | gene | 0.333478 | 0.907569 | Not queried | 3.71 | 2.86e-02 | 6.12e-02 |
|  | 11 | 17565744 | 17566163 | PDFSTATSENSEMBLUCSC | 420 | 10 | 8101 | island | 0.320202 | 0.029163 | Not queried | 3.71 | 1.23e-01 | 1.81e-01 |
|  | 13 | 47371267 | 47371644 | PDFSTATSENSEMBLUCSC | 378 | 11 | 11718 | island | 0.025404 | 0.361913 | Not queried | 3.71 | 1.58e-02 | 5.36e-02 |
|  | 8 | 65492528 | 65492936 | PDFSTATSENSEMBLUCSC | 409 | 10 | 40347 | island | 0.175875 | 0.738905 | BHLHE22; RP11-21C4.1 (margin: BHLHE22; RP11-21C4.5; RP11-21C4.1; CYP7B1) | 3.71 | 3.25e-04 | 4.91e-03 |
|  | 8 | 65492528 | 65492792 | PDFSTATSENSEMBLUCSC | 265 | 8 | 39736 | tss | 0.164728 | 0.738905 | BHLHE22; RP11-21C4.1 (margin: BHLHE22; RP11-21C4.5; RP11-21C4.1; CYP7B1) | 3.71 | 6.22e-04 | 6.71e-03 |
|  | 20 | 61162000 | 61162216 | PDFSTATSENSEMBLUCSC | 217 | 7 | 25491 | gene | 0.341657 | 0.968412 | C20orf166; MIR133A2 (margin: C20orf166; MIR1-1; MIR133A2; RPL7P3; WI2-87327B8.1) | 3.71 | 6.99e-03 | 3.19e-02 |
|  | 7 | 105221985 | 105222180 | PDFSTATSENSEMBLUCSC | 196 | 7 | 37819 | tss | 0.925375 | 0.476795 | Not queried | 3.71 | 2.62e-02 | 6.12e-02 |
|  | 2 | 183903743 | 183903770 | PDFSTATSENSEMBLUCSC | 28 | 4 | 23768 | tss;island | 0.123515 | 0.010397 | Not queried | 3.71 | 2.86e-02 | 6.12e-02 |
|  | 2 | 105478656 | 105479054 | PDFSTATSENSEMBLUCSC | 399 | 4 | 24579 | island | 0.811924 | 0.981727 | Not queried | 3.70 | 3.43e-01 | 3.84e-01 |
|  | 21 | 44494722 | 44495288 | PDFSTATSENSEMBLUCSC | 567 | 6 | 26443 | gene;island | 0.719849 | 0.010645 | Not queried | 3.70 | 2.60e-02 | 6.12e-02 |
|  | 22 | 19137874 | 19138267 | PDFSTATSENSEMBLUCSC | 394 | 5 | 26955 | tss;island | 0.788035 | 0.978832 | GSC2 (margin: DGCR14; GSC2) | 3.70 | 7.94e-03 | 3.19e-02 |
|  | 7 | 105221826 | 105222180 | PDFSTATSENSEMBLUCSC | 355 | 8 | 38821 | island | 0.921773 | 0.471501 | EFCAB10; YBX1P2 (margin: EFCAB10; YBX1P2) | 3.70 | 1.04e-02 | 3.96e-02 |
|  | 11 | 46354562 | 46355283 | PDFSTATSENSEMBLUCSC | 722 | 10 | 8246 | island | 0.771264 | 0.984161 | DGKZ (margin: CREB3L1; DGKZ) | 3.70 | 1.50e-03 | 1.41e-02 |
|  | 13 | 33002010 | 33002269 | PDFSTATSENSEMBLUCSC | 260 | 5 | 11124 | gene | 0.130875 | 0.011906 | N4BP2L1; RP11-298P3.4 (margin: N4BP2L1; SNORA16; RP11-298P3.4; N4BP2L2) | 3.70 | 7.94e-03 | 3.19e-02 |
|  | 10 | 128076910 | 128077080 | PDFSTATSENSEMBLUCSC | 171 | 4 | 4815 | gene;island | 0.680575 | 0.974147 | Not queried | 3.70 | 5.71e-02 | 1.00e-01 |
|  | 2 | 79740185 | 79740322 | PDFSTATSENSEMBLUCSC | 138 | 4 | 22754 | gene;island | 0.929300 | 0.479902 | Not queried | 3.70 | 2.86e-02 | 6.12e-02 |
|  | 2 | 200468445 | 200469026 | PDFSTATSENSEMBLUCSC | 582 | 5 | 24994 | island | 0.647102 | 0.936772 | Not queried | 3.70 | 5.56e-02 | 1.00e-01 |
|  | 1 | 6240267 | 6240508 | PDFSTATSENSEMBLUCSC | 242 | 5 | 1197 | tss;island | 0.424250 | 0.106140 | Not queried | 3.70 | 1.51e-01 | 2.11e-01 |
|  | 3 | 184870891 | 184871070 | PDFSTATSENSEMBLUCSC | 180 | 6 | 28765 | tss | 0.270521 | 0.069079 | Not queried | 3.70 | 4.85e-01 | 5.11e-01 |
|  | 17 | 33700745 | 33700817 | PDFSTATSENSEMBLUCSC | 73 | 6 | 17267 | tss;island | 0.107058 | 0.530248 | SLFN11 (margin: SLFN11; RP11-686D22.6) | 3.70 | 2.16e-03 | 1.45e-02 |
|  | 16 | 57318603 | 57319093 | PDFSTATSENSEMBLUCSC | 491 | 11 | 15260 | tss;island | 0.008169 | 0.066675 | PLLP (margin: PLLP) | 3.69 | 5.24e-03 | 2.98e-02 |
|  | 1 | 3056542 | 3056951 | PDFSTATSENSEMBLUCSC | 410 | 7 | 102 | gene;island | 0.133823 | 0.732067 | PRDM16 (margin: PRDM16; RP1-163G9.2) | 3.69 | 1.17e-03 | 1.13e-02 |
|  | 6 | 99290284 | 99290737 | PDFSTATSENSEMBLUCSC | 454 | 5 | 36345 | island | 0.732408 | 0.975811 | Not queried | 3.69 | 3.17e-02 | 6.50e-02 |
|  | 5 | 175223225 | 175223497 | PDFSTATSENSEMBLUCSC | 273 | 4 | 32508 | tss | 0.180351 | 0.737980 | Not queried | 3.69 | 4.86e-01 | 5.11e-01 |
|  | 6 | 168449117 | 168449422 | PDFSTATSENSEMBLUCSC | 306 | 4 | 36656 | island | 0.509370 | 0.915102 | Not queried | 3.69 | 1.14e-01 | 1.68e-01 |
|  | 8 | 41505411 | 41505696 | PDFSTATSENSEMBLUCSC | 286 | 4 | 39672 | tss;island | 0.369825 | 0.889097 | Not queried | 3.69 | 2.86e-02 | 6.12e-02 |
|  | 15 | 33602758 | 33602824 | PDFSTATSENSEMBLUCSC | 67 | 4 | 13555 | tss;island | 0.509590 | 0.857691 | Not queried | 3.69 | 1.14e-01 | 1.68e-01 |
|  | 13 | 28498091 | 28498544 | PDFSTATSENSEMBLUCSC | 454 | 6 | 11117 | gene;island | 0.317757 | 0.932476 | Not queried | 3.69 | 9.31e-02 | 1.53e-01 |
|  | 17 | 1202015 | 1202252 | PDFSTATSENSEMBLUCSC | 238 | 4 | 16312 | gene | 0.594720 | 0.880837 | Not queried | 3.69 | 1.14e-01 | 1.68e-01 |
|  | 17 | 72462164 | 72462441 | PDFSTATSENSEMBLUCSC | 278 | 4 | 17627 | tss | 0.360345 | 0.879922 | Not queried | 3.69 | 2.86e-02 | 6.12e-02 |
|  | 20 | 2781122 | 2781685 | PDFSTATSENSEMBLUCSC | 564 | 11 | 25954 | island | 0.740995 | 0.976379 | CPXM1 (margin: CPXM1) | 3.69 | 8.51e-05 | 2.07e-03 |
|  | 13 | 112721712 | 112721789 | PDFSTATSENSEMBLUCSC | 78 | 6 | 11543 | tss | 0.634855 | 0.949551 | SOX1 (margin: SOX1) | 3.68 | 2.16e-03 | 1.45e-02 |
|  | 10 | 61469599 | 61469980 | PDFSTATSENSEMBLUCSC | 382 | 9 | 5764 | island | 0.593549 | 0.049942 | SLC16A9 (margin: SLC16A9) | 3.68 | 5.64e-03 | 3.18e-02 |
|  | 7 | 4911022 | 4911224 | PDFSTATSENSEMBLUCSC | 203 | 4 | 36863 | gene | 0.805580 | 0.980472 | Not queried | 3.68 | 2.86e-02 | 6.12e-02 |
|  | 6 | 30290258 | 30290603 | PDFSTATSENSEMBLUCSC | 346 | 6 | 33756 | gene;island | 0.482915 | 0.952386 | Not queried | 3.68 | 2.60e-02 | 6.12e-02 |
|  | 4 | 42400738 | 42401242 | PDFSTATSENSEMBLUCSC | 505 | 6 | 29994 | gene;island | 0.384687 | 0.893546 | SHISA3 (margin: SHISA3; RP11-63A11.1; ATP8A1) | 3.68 | 8.66e-03 | 3.36e-02 |
|  | 11 | 76571458 | 76571610 | PDFSTATSENSEMBLUCSC | 153 | 4 | 7580 | tss;island | 0.406992 | 0.900776 | Not queried | 3.68 | 2.00e-01 | 2.55e-01 |
|  | 19 | 3869086 | 3869599 | PDFSTATSENSEMBLUCSC | 514 | 6 | 20353 | tss;island | 0.178025 | 0.807165 | Not queried | 3.68 | 4.11e-02 | 8.14e-02 |
|  | 20 | 34252918 | 34253242 | PDFSTATSENSEMBLUCSC | 325 | 6 | 25708 | tss | 0.007970 | 0.129238 | Not queried | 3.68 | 4.11e-02 | 8.14e-02 |
|  | 10 | 102821427 | 102822508 | PDFSTATSENSEMBLUCSC | 1082 | 11 | 4722 | gene;island | 0.356010 | 0.040526 | KAZALD1 (margin: KAZALD1) | 3.68 | 7.65e-04 | 8.13e-03 |
|  | 10 | 661009 | 661730 | PDFSTATSENSEMBLUCSC | 722 | 8 | 4446 | gene | 0.705071 | 0.929663 | Not queried | 3.67 | 3.79e-02 | 7.66e-02 |
|  | 7 | 4049531 | 4050114 | PDFSTATSENSEMBLUCSC | 584 | 7 | 36825 | gene | 0.568697 | 0.955493 | Not queried | 3.67 | 9.73e-02 | 1.55e-01 |
|  | 10 | 86300295 | 86300605 | PDFSTATSENSEMBLUCSC | 311 | 4 | 5898 | island | 0.197740 | 0.012484 | Not queried | 3.67 | 2.86e-02 | 6.12e-02 |
|  | 19 | 15281200 | 15281607 | PDFSTATSENSEMBLUCSC | 408 | 5 | 19900 | gene;island | 0.789726 | 0.957412 | Not queried | 3.67 | 2.22e-01 | 2.76e-01 |
|  | 19 | 2546844 | 2547067 | PDFSTATSENSEMBLUCSC | 224 | 4 | 19692 | gene;island | 0.545712 | 0.951306 | Not queried | 3.67 | 1.14e-01 | 1.68e-01 |
|  | 8 | 54164051 | 54164442 | PDFSTATSENSEMBLUCSC | 392 | 8 | 40296 | island | 0.712925 | 0.971749 | OPRK1 (margin: RP11-162D9.3; OPRK1) | 3.67 | 2.95e-03 | 1.88e-02 |
|  | 12 | 7863853 | 7864076 | PDFSTATSENSEMBLUCSC | 224 | 5 | 9550 | tss | 0.702401 | 0.971825 | Not queried | 3.67 | 3.17e-02 | 6.50e-02 |
|  | 12 | 122356316 | 122356852 | PDFSTATSENSEMBLUCSC | 537 | 6 | 10925 | island | 0.958229 | 0.637917 | PSMD9; RP11-87C12.2; WDR66 (margin: PSMD9; RP11-87C12.2; RNU7-170P; WDR66) | 3.67 | 2.16e-03 | 1.45e-02 |
|  | 7 | 1022471 | 1023156 | PDFSTATSENSEMBLUCSC | 686 | 11 | 38063 | island | 0.527375 | 0.955195 | CYP2W1 (margin: CYP2W1; COX19) | 3.67 | 8.51e-05 | 2.07e-03 |
|  | 15 | 96868857 | 96869220 | PDFSTATSENSEMBLUCSC | 364 | 8 | 14485 | island | 0.034542 | 0.003789 | Not queried | 3.67 | 6.50e-02 | 1.11e-01 |
|  | 11 | 69519128 | 69519437 | PDFSTATSENSEMBLUCSC | 310 | 7 | 7521 | tss | 0.330090 | 0.691431 | FGF19 (margin: FGF19) | 3.67 | 2.33e-03 | 1.52e-02 |
|  | 16 | 79633452 | 79633901 | PDFSTATSENSEMBLUCSC | 450 | 4 | 14851 | gene;island | 0.062782 | 0.444110 | Not queried | 3.67 | 2.86e-02 | 6.12e-02 |
|  | 2 | 177053274 | 177053568 | PDFSTATSENSEMBLUCSC | 295 | 10 | 24915 | island | 0.074999 | 0.494011 | HOXD1; HOXD-AS1 (margin: AC009336.24; HOXD1; HOXD-AS1) | 3.67 | 1.08e-05 | 4.82e-04 |
|  | 10 | 23982350 | 23982579 | PDFSTATSENSEMBLUCSC | 230 | 4 | 4988 | tss;island | 0.796257 | 0.982830 | Not queried | 3.67 | 2.86e-02 | 6.12e-02 |
|  | 10 | 93392644 | 93392839 | PDFSTATSENSEMBLUCSC | 196 | 4 | 4673 | gene | 0.549357 | 0.938287 | Not queried | 3.66 | 2.86e-02 | 6.12e-02 |
|  | 14 | 37132291 | 37132774 | PDFSTATSENSEMBLUCSC | 484 | 5 | 12067 | gene;island | 0.482204 | 0.964515 | Not queried | 3.66 | 5.56e-02 | 1.00e-01 |
|  | 4 | 3685309 | 3685875 | PDFSTATSENSEMBLUCSC | 567 | 6 | 30851 | island | 0.273373 | 0.747086 | Not queried | 3.66 | 2.60e-02 | 6.12e-02 |
|  | 1 | 207818395 | 207818493 | PDFSTATSENSEMBLUCSC | 99 | 5 | 4154 | island | 0.483733 | 0.878768 | Not queried | 3.66 | 1.59e-02 | 5.36e-02 |
|  | 11 | 67418140 | 67418405 | PDFSTATSENSEMBLUCSC | 266 | 9 | 7500 | tss | 0.073856 | 0.666909 | ACY3; AP003385.2 (margin: TBX10; ACY3; AP003385.2; ALDH3B2) | 3.66 | 7.82e-04 | 8.17e-03 |
|  | 13 | 112320825 | 112321270 | PDFSTATSENSEMBLUCSC | 446 | 4 | 11901 | island | 0.762866 | 0.981362 | Not queried | 3.66 | 1.14e-01 | 1.68e-01 |
|  | 10 | 44788627 | 44789077 | PDFSTATSENSEMBLUCSC | 451 | 5 | 5706 | island | 0.661325 | 0.957212 | Not queried | 3.66 | 1.59e-02 | 5.36e-02 |
|  | 19 | 603639 | 603853 | PDFSTATSENSEMBLUCSC | 215 | 4 | 19631 | gene;island | 0.736640 | 0.982400 | Not queried | 3.66 | 2.86e-02 | 6.12e-02 |
|  | 6 | 167136206 | 167136432 | PDFSTATSENSEMBLUCSC | 227 | 4 | 34554 | gene | 0.617826 | 0.948057 | Not queried | 3.66 | 2.00e-01 | 2.55e-01 |
|  | 19 | 18496756 | 18496953 | PDFSTATSENSEMBLUCSC | 198 | 4 | 20618 | tss | 0.011862 | 0.175537 | Not queried | 3.66 | 2.86e-02 | 6.12e-02 |
|  | 2 | 242784034 | 242784150 | PDFSTATSENSEMBLUCSC | 117 | 4 | 25307 | island | 0.743044 | 0.973362 | Not queried | 3.66 | 2.86e-02 | 6.12e-02 |
|  | 3 | 125899370 | 125900108 | PDFSTATSENSEMBLUCSC | 739 | 13 | 29427 | island | 0.771005 | 0.978300 | ALDH1L1-AS2; ALDH1L1 (margin: ALDH1L1-AS2; ALDH1L1) | 3.66 | 2.67e-05 | 1.07e-03 |
|  | 3 | 125899547 | 125900108 | PDFSTATSENSEMBLUCSC | 562 | 11 | 28556 | tss | 0.771005 | 0.978300 | ALDH1L1-AS2; ALDH1L1 (margin: ALDH1L1-AS2; ALDH1L1) | 3.66 | 5.39e-05 | 1.47e-03 |
|  | 6 | 42927940 | 42928497 | PDFSTATSENSEMBLUCSC | 558 | 22 | 34985 | tss | 0.253324 | 0.015977 | GNMT (margin: RP3-475N16.1; GNMT; RPL24P4; PEX6) | 3.66 | 2.35e-07 | 3.02e-05 |
|  | 10 | 134043618 | 134043897 | PDFSTATSENSEMBLUCSC | 280 | 5 | 4858 | gene;island | 0.658959 | 0.953866 | STK32C (margin: STK32C) | 3.65 | 7.94e-03 | 3.19e-02 |
|  | 21 | 46131464 | 46131923 | PDFSTATSENSEMBLUCSC | 460 | 6 | 26739 | island | 0.321321 | 0.015899 | Not queried | 3.65 | 2.60e-02 | 6.12e-02 |
|  | 1 | 228504281 | 228504761 | PDFSTATSENSEMBLUCSC | 481 | 5 | 1055 | gene;island | 0.615110 | 0.982724 | Not queried | 3.65 | 9.52e-02 | 1.53e-01 |
|  | 8 | 41435301 | 41435469 | PDFSTATSENSEMBLUCSC | 169 | 4 | 39670 | tss;island | 0.039627 | 0.011036 | Not queried | 3.65 | 3.43e-01 | 3.84e-01 |
|  | 7 | 101936230 | 101936527 | PDFSTATSENSEMBLUCSC | 298 | 5 | 37165 | gene;island | 0.636708 | 0.953384 | Not queried | 3.65 | 3.17e-02 | 6.50e-02 |
|  | 3 | 57742858 | 57743171 | PDFSTATSENSEMBLUCSC | 314 | 6 | 28412 | tss;island | 0.007334 | 0.117497 | Not queried | 3.65 | 4.11e-02 | 8.14e-02 |
|  | 16 | 15595854 | 15596082 | PDFSTATSENSEMBLUCSC | 229 | 6 | 15107 | tss | 0.436238 | 0.946450 | RP11-1021N1.1; C16orf45 (margin: RP11-1021N1.1; C16orf45) | 3.65 | 8.66e-03 | 3.36e-02 |
|  | 19 | 35633258 | 35634229 | PDFSTATSENSEMBLUCSC | 972 | 9 | 21898 | island | 0.397194 | 0.024124 | Not queried | 3.65 | 2.22e-01 | 2.76e-01 |
|  | 11 | 31832879 | 31833264 | PDFSTATSENSEMBLUCSC | 386 | 8 | 7223 | tss | 0.755876 | 0.974795 | RCN1; PAX6 (margin: RCN1; PAX6) | 3.65 | 1.55e-04 | 2.71e-03 |
|  | 12 | 8332808 | 8333192 | PDFSTATSENSEMBLUCSC | 385 | 5 | 9049 | gene | 0.349597 | 0.021407 | Not queried | 3.65 | 9.52e-02 | 1.53e-01 |
|  | 6 | 130031504 | 130031784 | PDFSTATSENSEMBLUCSC | 281 | 6 | 35219 | tss | 0.010701 | 0.001744 | Not queried | 3.65 | 1.32e-01 | 1.89e-01 |
|  | 6 | 154831692 | 154831780 | PDFSTATSENSEMBLUCSC | 89 | 4 | 36586 | island | 0.151013 | 0.014576 | Not queried | 3.64 | 2.86e-02 | 6.12e-02 |
|  | 3 | 196366191 | 196366618 | PDFSTATSENSEMBLUCSC | 428 | 7 | 28816 | tss | 0.613406 | 0.909359 | NRROS; PIGX (margin: NRROS; PIGX; AC023797.1) | 3.64 | 6.99e-03 | 3.19e-02 |
|  | 11 | 116451437 | 116451788 | PDFSTATSENSEMBLUCSC | 352 | 4 | 8833 | island | 0.751973 | 0.971973 | Not queried | 3.64 | 1.14e-01 | 1.68e-01 |
|  | 2 | 118942841 | 118943184 | PDFSTATSENSEMBLUCSC | 344 | 5 | 24641 | island | 0.870683 | 0.350845 | Not queried | 3.64 | 1.51e-01 | 2.11e-01 |
|  | 5 | 1879525 | 1880021 | PDFSTATSENSEMBLUCSC | 497 | 6 | 31549 | gene;island | 0.402898 | 0.962991 | Not queried | 3.64 | 2.60e-02 | 6.12e-02 |
|  | 4 | 111397134 | 111397220 | PDFSTATSENSEMBLUCSC | 87 | 5 | 30548 | tss | 0.820349 | 0.039164 | Not queried | 3.64 | 1.59e-02 | 5.36e-02 |
|  | 14 | 77737495 | 77737800 | PDFSTATSENSEMBLUCSC | 306 | 6 | 13032 | island | 0.667932 | 0.965155 | NGB (margin: TMEM63C; MIR1260A; NGB; POMT2) | 3.64 | 2.16e-03 | 1.45e-02 |
|  | 10 | 134992340 | 134992590 | PDFSTATSENSEMBLUCSC | 251 | 4 | 4888 | gene;island | 0.462644 | 0.896251 | Not queried | 3.64 | 2.86e-02 | 6.12e-02 |
|  | 8 | 24771114 | 24771645 | PDFSTATSENSEMBLUCSC | 532 | 7 | 39604 | tss;island | 0.365215 | 0.869247 | NEFM; RP11-624C23.1; GS1-72M22.1 (margin: NEFM; RP11-624C23.1; GS1-72M22.1) | 3.64 | 1.17e-03 | 1.13e-02 |
|  | 11 | 107582804 | 107582884 | PDFSTATSENSEMBLUCSC | 81 | 4 | 7664 | tss | 0.164410 | 0.557024 | Not queried | 3.64 | 2.86e-02 | 6.12e-02 |
|  | 12 | 124155451 | 124155620 | PDFSTATSENSEMBLUCSC | 170 | 5 | 10105 | tss | 0.007061 | 0.021954 | Not queried | 3.64 | 4.21e-01 | 4.59e-01 |
|  | 20 | 61162000 | 61162112 | PDFSTATSENSEMBLUCSC | 113 | 6 | 25877 | tss | 0.392522 | 0.898539 | Not queried | 3.64 | 2.60e-02 | 6.12e-02 |
|  | 15 | 25427394 | 25427509 | PDFSTATSENSEMBLUCSC | 116 | 4 | 13528 | tss | 0.067904 | 0.359274 | Not queried | 3.63 | 2.86e-02 | 6.12e-02 |
|  | 15 | 83240578 | 83240791 | PDFSTATSENSEMBLUCSC | 214 | 6 | 13857 | tss | 0.495213 | 0.875009 | CPEB1 (margin: RP11-379H8.1; CPEB1) | 3.63 | 2.16e-03 | 1.45e-02 |
|  | 1 | 115212834 | 115213259 | PDFSTATSENSEMBLUCSC | 426 | 10 | 1852 | tss | 0.005899 | 0.130886 | Not queried | 3.63 | 1.85e-02 | 6.12e-02 |
|  | 13 | 108518152 | 108518621 | PDFSTATSENSEMBLUCSC | 470 | 6 | 11220 | gene;island | 0.661948 | 0.158310 | Not queried | 3.63 | 1.52e-02 | 5.36e-02 |
|  | 1 | 50889182 | 50889510 | PDFSTATSENSEMBLUCSC | 329 | 6 | 1616 | tss | 0.678095 | 0.976904 | DMRTA2 (margin: DMRTA2) | 3.63 | 4.33e-03 | 2.52e-02 |
|  | 2 | 120281354 | 120281999 | PDFSTATSENSEMBLUCSC | 646 | 6 | 22847 | gene | 0.790774 | 0.983439 | Not queried | 3.63 | 4.11e-02 | 8.14e-02 |
|  | 10 | 91295045 | 91295421 | PDFSTATSENSEMBLUCSC | 377 | 9 | 5924 | island | 0.815173 | 0.986377 | SLC16A12 (margin: SLC16A12) | 3.63 | 4.11e-05 | 1.17e-03 |
|  | 3 | 133464463 | 133464971 | PDFSTATSENSEMBLUCSC | 509 | 6 | 28602 | tss;island | 0.552521 | 0.955996 | Not queried | 3.63 | 2.60e-02 | 6.12e-02 |
|  | 1 | 156051324 | 156051792 | PDFSTATSENSEMBLUCSC | 469 | 5 | 3843 | island | 0.579129 | 0.032878 | Not queried | 3.63 | 5.56e-02 | 1.00e-01 |
|  | 15 | 29407792 | 29408145 | PDFSTATSENSEMBLUCSC | 354 | 4 | 13268 | gene;island | 0.740141 | 0.963565 | Not queried | 3.63 | 1.14e-01 | 1.68e-01 |
|  | 5 | 36606102 | 36606446 | PDFSTATSENSEMBLUCSC | 345 | 5 | 32019 | tss | 0.572448 | 0.932628 | Not queried | 3.63 | 9.52e-02 | 1.53e-01 |
|  | 5 | 153858816 | 153859049 | PDFSTATSENSEMBLUCSC | 234 | 5 | 32449 | tss;island | 0.561021 | 0.938024 | Not queried | 3.63 | 3.10e-01 | 3.66e-01 |
|  | 16 | 49732224 | 49732623 | PDFSTATSENSEMBLUCSC | 400 | 4 | 14745 | gene;island | 0.480864 | 0.920326 | Not queried | 3.63 | 2.00e-01 | 2.55e-01 |
|  | 2 | 42068380 | 42068648 | PDFSTATSENSEMBLUCSC | 269 | 5 | 24238 | island | 0.418439 | 0.028024 | Not queried | 3.63 | 3.17e-02 | 6.50e-02 |
|  | 14 | 104690040 | 104690244 | PDFSTATSENSEMBLUCSC | 205 | 5 | 13204 | island | 0.488380 | 0.924957 | RP11-260M19.2 (margin: RP11-260M19.2) | 3.63 | 7.94e-03 | 3.19e-02 |
|  | 6 | 137817512 | 137817846 | PDFSTATSENSEMBLUCSC | 335 | 4 | 36509 | island | 0.349271 | 0.935218 | Not queried | 3.63 | 5.71e-02 | 1.00e-01 |
|  | 13 | 28562798 | 28562902 | PDFSTATSENSEMBLUCSC | 105 | 7 | 11367 | tss | 0.754448 | 0.966805 | Not queried | 3.63 | 2.09e-01 | 2.65e-01 |
|  | 2 | 65215361 | 65215753 | PDFSTATSENSEMBLUCSC | 393 | 10 | 23393 | tss;island | 0.051784 | 0.430556 | SLC1A4 (margin: SLC1A4; RNU6-548P) | 3.63 | 1.08e-05 | 4.82e-04 |
|  | 1 | 228870454 | 228870885 | PDFSTATSENSEMBLUCSC | 432 | 8 | 4305 | island | 0.586250 | 0.320565 | Not queried | 3.63 | 1.95e-01 | 2.55e-01 |
|  | 19 | 59074265 | 59074507 | PDFSTATSENSEMBLUCSC | 243 | 4 | 20265 | gene;island | 0.793998 | 0.982444 | Not queried | 3.63 | 5.71e-02 | 1.00e-01 |
|  | 1 | 205819406 | 205819609 | PDFSTATSENSEMBLUCSC | 204 | 4 | 2210 | tss | 0.803160 | 0.982420 | Not queried | 3.63 | 2.86e-02 | 6.12e-02 |
|  | 12 | 25102274 | 25102521 | PDFSTATSENSEMBLUCSC | 248 | 5 | 10290 | island | 0.738938 | 0.968373 | Not queried | 3.63 | 4.21e-01 | 4.59e-01 |
|  | 8 | 24771273 | 24771645 | PDFSTATSENSEMBLUCSC | 373 | 4 | 39245 | gene | 0.367861 | 0.845639 | Not queried | 3.63 | 2.86e-02 | 6.12e-02 |
|  | 2 | 105275613 | 105275920 | PDFSTATSENSEMBLUCSC | 308 | 4 | 24576 | island | 0.855717 | 0.976846 | Not queried | 3.63 | 5.71e-02 | 1.00e-01 |
|  | 20 | 6748710 | 6748730 | PDFSTATSENSEMBLUCSC | 21 | 4 | 25590 | tss;island | 0.530481 | 0.074994 | Not queried | 3.63 | 2.86e-02 | 6.12e-02 |
|  | 12 | 116970856 | 116971165 | PDFSTATSENSEMBLUCSC | 310 | 9 | 10037 | tss | 0.074765 | 0.006466 | Not queried | 3.63 | 2.44e-02 | 6.12e-02 |
|  | 13 | 20702922 | 20703169 | PDFSTATSENSEMBLUCSC | 248 | 4 | 11565 | island | 0.342595 | 0.080402 | Not queried | 3.62 | 3.43e-01 | 3.84e-01 |
|  | 15 | 75249787 | 75249914 | PDFSTATSENSEMBLUCSC | 128 | 6 | 13806 | tss;island | 0.191733 | 0.017345 | Not queried | 3.62 | 4.11e-02 | 8.14e-02 |
|  | 17 | 33759929 | 33759986 | PDFSTATSENSEMBLUCSC | 58 | 4 | 17269 | tss | 0.928754 | 0.995368 | Not queried | 3.62 | 2.86e-02 | 6.12e-02 |
|  | 14 | 60952405 | 60952945 | PDFSTATSENSEMBLUCSC | 541 | 6 | 12902 | island | 0.874950 | 0.969931 | Not queried | 3.62 | 2.60e-02 | 6.12e-02 |
|  | 19 | 15083795 | 15083916 | PDFSTATSENSEMBLUCSC | 122 | 5 | 20551 | tss | 0.499965 | 0.972658 | Not queried | 3.62 | 3.17e-02 | 6.50e-02 |
|  | 10 | 127464165 | 127464762 | PDFSTATSENSEMBLUCSC | 598 | 9 | 6238 | island | 0.575454 | 0.897565 | MMP21 (margin: C10orf137; MMP21) | 3.62 | 5.64e-03 | 3.18e-02 |
|  | 6 | 29523592 | 29524117 | PDFSTATSENSEMBLUCSC | 526 | 8 | 33660 | gene;island | 0.206994 | 0.680344 | OR2I1P; UBD; GABBR1 (margin: OR2I1P; UBD; GABBR1) | 3.62 | 4.66e-03 | 2.68e-02 |
|  | 14 | 101036192 | 101036470 | PDFSTATSENSEMBLUCSC | 279 | 4 | 12605 | tss;island | 0.404508 | 0.825237 | Not queried | 3.62 | 2.86e-02 | 6.12e-02 |
|  | 5 | 176170138 | 176170795 | PDFSTATSENSEMBLUCSC | 658 | 7 | 33433 | island | 0.615944 | 0.911526 | RP11-375B1.3 (margin: RP11-375B1.3; RP11-375B1.2) | 3.62 | 1.11e-02 | 4.14e-02 |
|  | 1 | 1266468 | 1266781 | PDFSTATSENSEMBLUCSC | 314 | 6 | 2426 | island | 0.348044 | 0.807535 | TAS1R3 (margin: GLTPD1; TAS1R3; CPSF3L; DVL1) | 3.62 | 2.16e-03 | 1.45e-02 |
|  | 13 | 112212075 | 112212266 | PDFSTATSENSEMBLUCSC | 192 | 4 | 11899 | island | 0.291349 | 0.835644 | Not queried | 3.62 | 2.86e-02 | 6.12e-02 |
|  | 2 | 96012586 | 96012735 | PDFSTATSENSEMBLUCSC | 150 | 4 | 23510 | tss | 0.476376 | 0.084522 | Not queried | 3.62 | 1.14e-01 | 1.68e-01 |
|  | 19 | 4304173 | 4304577 | PDFSTATSENSEMBLUCSC | 405 | 7 | 20363 | tss;island | 0.523134 | 0.018462 | Not queried | 3.62 | 3.18e-01 | 3.74e-01 |
|  | 1 | 50889128 | 50889510 | PDFSTATSENSEMBLUCSC | 383 | 7 | 3253 | island | 0.678901 | 0.977000 | DMRTA2 (margin: DMRTA2) | 3.62 | 1.17e-03 | 1.13e-02 |
|  | 16 | 959749 | 960144 | PDFSTATSENSEMBLUCSC | 396 | 4 | 14550 | gene;island | 0.803099 | 0.981274 | Not queried | 3.62 | 2.86e-02 | 6.12e-02 |
|  | 2 | 68546899 | 68547349 | PDFSTATSENSEMBLUCSC | 451 | 7 | 24363 | island | 0.330661 | 0.896915 | Not queried | 3.62 | 5.30e-02 | 1.00e-01 |
|  | 5 | 137610255 | 137610645 | PDFSTATSENSEMBLUCSC | 391 | 7 | 32317 | tss | 0.943008 | 0.673196 | Not queried | 3.62 | 9.73e-02 | 1.55e-01 |
|  | 10 | 131263962 | 131264846 | PDFSTATSENSEMBLUCSC | 885 | 12 | 5439 | tss;island | 0.516002 | 0.938225 | Not queried | 3.62 | 1.45e-02 | 5.34e-02 |
|  | 14 | 101830869 | 101831332 | PDFSTATSENSEMBLUCSC | 464 | 4 | 13155 | island | 0.535276 | 0.930187 | Not queried | 3.62 | 2.00e-01 | 2.55e-01 |
|  | 11 | 69258203 | 69258477 | PDFSTATSENSEMBLUCSC | 275 | 4 | 8587 | island | 0.561448 | 0.947528 | Not queried | 3.62 | 2.86e-02 | 6.12e-02 |
|  | 10 | 133951618 | 133951963 | PDFSTATSENSEMBLUCSC | 346 | 5 | 4853 | gene;island | 0.616284 | 0.972076 | JAKMIP3 (margin: JAKMIP3) | 3.61 | 7.94e-03 | 3.19e-02 |
|  | 1 | 212873963 | 212874153 | PDFSTATSENSEMBLUCSC | 191 | 4 | 2246 | tss;island | 0.461625 | 0.067111 | Not queried | 3.61 | 2.86e-02 | 6.12e-02 |
|  | 17 | 46691820 | 46692248 | PDFSTATSENSEMBLUCSC | 429 | 5 | 16660 | gene | 0.786946 | 0.148128 | Not queried | 3.61 | 1.51e-01 | 2.11e-01 |
|  | 19 | 41018757 | 41018880 | PDFSTATSENSEMBLUCSC | 124 | 4 | 20043 | gene;island | 0.767104 | 0.982084 | Not queried | 3.61 | 2.86e-02 | 6.12e-02 |
|  | 6 | 142409417 | 142410235 | PDFSTATSENSEMBLUCSC | 819 | 13 | 36523 | island | 0.862203 | 0.521303 | Not queried | 3.61 | 2.95e-02 | 6.30e-02 |
|  | 11 | 70385301 | 70385511 | PDFSTATSENSEMBLUCSC | 211 | 4 | 6791 | gene | 0.513720 | 0.927565 | Not queried | 3.61 | 2.86e-02 | 6.12e-02 |
|  | 10 | 102497298 | 102497666 | PDFSTATSENSEMBLUCSC | 369 | 5 | 6032 | island | 0.727077 | 0.970135 | Not queried | 3.61 | 1.51e-01 | 2.11e-01 |
|  | 3 | 192126584 | 192126996 | PDFSTATSENSEMBLUCSC | 413 | 7 | 28085 | gene;island | 0.662388 | 0.956559 | FGF12 (margin: FGF12) | 3.61 | 4.08e-03 | 2.47e-02 |
|  | 10 | 105253117 | 105253716 | PDFSTATSENSEMBLUCSC | 600 | 8 | 5340 | tss;island | 0.543481 | 0.076709 | Not queried | 3.61 | 1.05e-01 | 1.66e-01 |
|  | 8 | 145649356 | 145649678 | PDFSTATSENSEMBLUCSC | 323 | 5 | 39522 | gene;island | 0.532813 | 0.955451 | Not queried | 3.61 | 1.59e-02 | 5.36e-02 |
|  | 6 | 32073324 | 32073700 | PDFSTATSENSEMBLUCSC | 377 | 5 | 34048 | gene | 0.490440 | 0.472524 | Not queried | 3.61 | 6.90e-01 | 7.01e-01 |
|  | 19 | 6463949 | 6464214 | PDFSTATSENSEMBLUCSC | 266 | 9 | 20390 | tss | 0.315131 | 0.015143 | CRB3; SLC25A23 (margin: CRB3; SLC25A23; DENND1C) | 3.61 | 7.77e-03 | 3.19e-02 |
|  | 1 | 233462575 | 233462803 | PDFSTATSENSEMBLUCSC | 229 | 5 | 2326 | tss;island | 0.506164 | 0.167907 | Not queried | 3.61 | 2.22e-01 | 2.76e-01 |
|  | 19 | 840737 | 840950 | PDFSTATSENSEMBLUCSC | 214 | 4 | 20281 | tss | 0.284973 | 0.827509 | Not queried | 3.61 | 5.71e-02 | 1.00e-01 |
|  | 1 | 45452509 | 45452605 | PDFSTATSENSEMBLUCSC | 97 | 4 | 1578 | tss | 0.375878 | 0.041636 | Not queried | 3.61 | 2.00e-01 | 2.55e-01 |
|  | 22 | 35695066 | 35695301 | PDFSTATSENSEMBLUCSC | 236 | 4 | 27098 | tss;island | 0.971632 | 0.665160 | Not queried | 3.61 | 2.00e-01 | 2.55e-01 |
|  | 1 | 91869782 | 91870067 | PDFSTATSENSEMBLUCSC | 286 | 4 | 629 | gene;island | 0.249426 | 0.047605 | Not queried | 3.61 | 2.00e-01 | 2.55e-01 |
|  | 7 | 1748302 | 1748766 | PDFSTATSENSEMBLUCSC | 465 | 9 | 37405 | tss | 0.449636 | 0.920668 | ELFN1 (margin: ELFN1) | 3.60 | 1.65e-04 | 2.80e-03 |
|  | 4 | 24914750 | 24914970 | PDFSTATSENSEMBLUCSC | 221 | 5 | 29968 | gene;tss | 0.918332 | 0.248570 | Not queried | 3.60 | 1.51e-01 | 2.11e-01 |
|  | 2 | 289147 | 289731 | PDFSTATSENSEMBLUCSC | 585 | 6 | 23154 | tss;island | 0.674073 | 0.981288 | Not queried | 3.60 | 1.52e-02 | 5.36e-02 |
|  | 22 | 31198409 | 31198724 | PDFSTATSENSEMBLUCSC | 316 | 4 | 26838 | gene;island | 0.240314 | 0.023190 | Not queried | 3.60 | 1.14e-01 | 1.68e-01 |
|  | 17 | 27333108 | 27333617 | PDFSTATSENSEMBLUCSC | 510 | 11 | 17230 | tss | 0.478261 | 0.140620 | Not queried | 3.60 | 4.73e-02 | 9.34e-02 |
|  | 17 | 44060252 | 44060502 | PDFSTATSENSEMBLUCSC | 251 | 4 | 16642 | gene;island | 0.706973 | 0.979706 | Not queried | 3.60 | 2.86e-02 | 6.12e-02 |
|  | 17 | 43224158 | 43224582 | PDFSTATSENSEMBLUCSC | 425 | 8 | 17425 | tss;island | 0.156813 | 0.603908 | Not queried | 3.60 | 2.34e-01 | 2.90e-01 |
|  | 10 | 3374141 | 3374366 | PDFSTATSENSEMBLUCSC | 226 | 4 | 5511 | island | 0.977408 | 0.666773 | Not queried | 3.60 | 2.86e-02 | 6.12e-02 |
|  | 2 | 217236425 | 217236650 | PDFSTATSENSEMBLUCSC | 226 | 4 | 23010 | gene | 0.661567 | 0.050061 | Not queried | 3.60 | 1.14e-01 | 1.68e-01 |
|  | 10 | 135050619 | 135051581 | PDFSTATSENSEMBLUCSC | 963 | 28 | 6348 | island | 0.550924 | 0.956534 | VENTX (margin: KNDC1; UTF1; VENTX; MIR202) | 3.60 | 1.65e-09 | 6.41e-07 |
|  | 12 | 106978920 | 106979229 | PDFSTATSENSEMBLUCSC | 310 | 4 | 9280 | gene;island | 0.342229 | 0.865909 | Not queried | 3.60 | 5.71e-02 | 1.00e-01 |
|  | 10 | 52750736 | 52750930 | PDFSTATSENSEMBLUCSC | 195 | 4 | 5083 | tss | 0.858766 | 0.363413 | Not queried | 3.60 | 2.86e-02 | 6.12e-02 |
|  | 11 | 1855746 | 1856307 | PDFSTATSENSEMBLUCSC | 562 | 7 | 6432 | gene | 0.351682 | 0.781362 | SYT8 (margin: SYT8; TNNI2) | 3.60 | 6.99e-03 | 3.19e-02 |
|  | 5 | 75377755 | 75378176 | PDFSTATSENSEMBLUCSC | 422 | 6 | 32887 | island | 0.658021 | 0.962985 | Not queried | 3.60 | 1.52e-02 | 5.36e-02 |
|  | 6 | 168402227 | 168402503 | PDFSTATSENSEMBLUCSC | 277 | 4 | 36654 | island | 0.557330 | 0.934214 | Not queried | 3.60 | 5.71e-02 | 1.00e-01 |
|  | 1 | 17215834 | 17216201 | PDFSTATSENSEMBLUCSC | 368 | 4 | 2749 | island | 0.382204 | 0.911933 | Not queried | 3.60 | 2.86e-02 | 6.12e-02 |
|  | 2 | 177053274 | 177053297 | PDFSTATSENSEMBLUCSC | 24 | 6 | 23744 | tss | 0.074999 | 0.479642 | HOXD1; HOXD-AS1 (margin: AC009336.24; HOXD1; HOXD-AS1) | 3.60 | 2.16e-03 | 1.45e-02 |
|  | 12 | 54343506 | 54343835 | PDFSTATSENSEMBLUCSC | 330 | 5 | 10480 | island | 0.906257 | 0.433785 | (margin: HOXC13; HOXC12; AC012531.23; HOXC-AS5) | 3.60 | 7.94e-03 | 3.19e-02 |
|  | 14 | 101489985 | 101490253 | PDFSTATSENSEMBLUCSC | 269 | 10 | 12620 | tss | 0.434178 | 0.897905 | MIR379; MIR411; MIR299; MIR1197; MIR323A; MIR380 (margin: MIR379; MIR411; MIR299; MIR1197; MIR323A; MIR758; MIR329-1; MIR329-2; MIR494; MIR1193; MIR543; MIR495; MIR380) | 3.60 | 3.89e-03 | 2.43e-02 |
|  | 5 | 178487123 | 178487576 | PDFSTATSENSEMBLUCSC | 454 | 10 | 32548 | tss | 0.081366 | 0.485388 | ZNF354C (margin: ZNF354C) | 3.60 | 1.08e-05 | 4.82e-04 |
|  | 8 | 119964440 | 119964729 | PDFSTATSENSEMBLUCSC | 290 | 6 | 39884 | tss | 0.839115 | 0.426669 | Not queried | 3.60 | 6.49e-02 | 1.11e-01 |
|  | 2 | 26726190 | 26726691 | PDFSTATSENSEMBLUCSC | 502 | 5 | 22614 | gene;island | 0.144312 | 0.026705 | Not queried | 3.60 | 2.22e-01 | 2.76e-01 |
|  | 16 | 85670135 | 85670348 | PDFSTATSENSEMBLUCSC | 214 | 4 | 14877 | gene;island | 0.654564 | 0.985882 | Not queried | 3.60 | 5.71e-02 | 1.00e-01 |
|  | 13 | 112200641 | 112201036 | PDFSTATSENSEMBLUCSC | 396 | 6 | 11898 | island | 0.291592 | 0.747831 | Not queried | 3.60 | 4.11e-02 | 8.14e-02 |
|  | 7 | 150778724 | 150779390 | PDFSTATSENSEMBLUCSC | 667 | 14 | 37287 | gene;tss;island | 0.156903 | 0.620475 | FASTK; TMUB1 (margin: SLC4A2; AGAP3; RP11-148K1.12; FASTK; TMUB1) | 3.60 | 9.71e-04 | 1.01e-02 |
|  | 2 | 132056834 | 132057166 | PDFSTATSENSEMBLUCSC | 333 | 4 | 22870 | gene;island | 0.718726 | 0.967715 | Not queried | 3.60 | 2.86e-02 | 6.12e-02 |
|  | 19 | 4891192 | 4891471 | PDFSTATSENSEMBLUCSC | 280 | 5 | 19741 | gene;island | 0.675902 | 0.930608 | Not queried | 3.60 | 1.51e-01 | 2.11e-01 |
|  | 7 | 77648378 | 77648624 | PDFSTATSENSEMBLUCSC | 247 | 4 | 37063 | gene;island | 0.048931 | 0.419640 | Not queried | 3.60 | 2.86e-02 | 6.12e-02 |
|  | 5 | 1950701 | 1951028 | PDFSTATSENSEMBLUCSC | 328 | 4 | 32648 | island | 0.676763 | 0.936657 | Not queried | 3.60 | 5.71e-02 | 1.00e-01 |
|  | 10 | 11783794 | 11784473 | PDFSTATSENSEMBLUCSC | 680 | 10 | 5546 | island | 0.043496 | 0.353613 | Not queried | 3.59 | 1.05e-01 | 1.66e-01 |
|  | 1 | 245727127 | 245727658 | PDFSTATSENSEMBLUCSC | 532 | 6 | 1117 | gene | 0.412201 | 0.821460 | Not queried | 3.59 | 2.60e-02 | 6.12e-02 |
|  | 20 | 1093493 | 1093802 | PDFSTATSENSEMBLUCSC | 310 | 6 | 25532 | tss | 0.582069 | 0.941525 | Not queried | 3.59 | 1.52e-02 | 5.36e-02 |
|  | 14 | 53418879 | 53419111 | PDFSTATSENSEMBLUCSC | 233 | 5 | 12402 | tss;island | 0.875707 | 0.339021 | Not queried | 3.59 | 1.51e-01 | 2.11e-01 |
|  | 2 | 242930039 | 242930747 | PDFSTATSENSEMBLUCSC | 709 | 7 | 25319 | island | 0.525845 | 0.972567 | Not queried | 3.59 | 5.30e-02 | 1.00e-01 |
|  | 2 | 43019819 | 43020457 | PDFSTATSENSEMBLUCSC | 639 | 8 | 23322 | tss | 0.660483 | 0.966472 | HAAO (margin: HAAO; FTOP1) | 3.59 | 3.11e-04 | 4.77e-03 |
|  | 20 | 62716332 | 62716606 | PDFSTATSENSEMBLUCSC | 275 | 4 | 25520 | gene;tss;island | 0.652893 | 0.937392 | Not queried | 3.59 | 2.86e-02 | 6.12e-02 |
|  | 5 | 500202 | 500565 | PDFSTATSENSEMBLUCSC | 364 | 5 | 31509 | gene;island | 0.633111 | 0.877861 | Not queried | 3.59 | 1.59e-02 | 5.36e-02 |
|  | 3 | 147142102 | 147142415 | PDFSTATSENSEMBLUCSC | 314 | 5 | 29576 | island | 0.673184 | 0.968537 | Not queried | 3.59 | 9.52e-02 | 1.53e-01 |
|  | 17 | 44896017 | 44896223 | PDFSTATSENSEMBLUCSC | 207 | 8 | 18447 | island | 0.333662 | 0.051288 | WNT3 (margin: WNT3) | 3.58 | 1.55e-04 | 2.71e-03 |
|  | 19 | 8601136 | 8601423 | PDFSTATSENSEMBLUCSC | 288 | 4 | 19796 | gene;island | 0.634557 | 0.929343 | Not queried | 3.58 | 5.71e-02 | 1.00e-01 |
|  | 6 | 31324916 | 31324972 | PDFSTATSENSEMBLUCSC | 57 | 5 | 33870 | gene | 0.040061 | 0.273691 | Not queried | 3.58 | 2.22e-01 | 2.76e-01 |
|  | 2 | 219925246 | 219925330 | PDFSTATSENSEMBLUCSC | 85 | 6 | 23890 | tss | 0.825616 | 0.983765 | Not queried | 3.58 | 1.52e-02 | 5.36e-02 |
|  | 2 | 97524767 | 97525009 | PDFSTATSENSEMBLUCSC | 243 | 4 | 23525 | tss;island | 0.970909 | 0.645735 | Not queried | 3.58 | 2.86e-02 | 6.12e-02 |
|  | 11 | 65640137 | 65640516 | PDFSTATSENSEMBLUCSC | 380 | 6 | 8485 | island | 0.787199 | 0.222082 | Not queried | 3.58 | 4.11e-02 | 8.14e-02 |
|  | 1 | 2886654 | 2886868 | PDFSTATSENSEMBLUCSC | 215 | 5 | 2524 | island | 0.706185 | 0.863493 | Not queried | 3.58 | 3.17e-02 | 6.50e-02 |
|  | 5 | 75380782 | 75381070 | PDFSTATSENSEMBLUCSC | 289 | 5 | 31650 | gene;island | 0.749660 | 0.973143 | Not queried | 3.58 | 5.56e-02 | 1.00e-01 |
|  | 1 | 48459813 | 48460182 | PDFSTATSENSEMBLUCSC | 370 | 5 | 3245 | island | 0.573902 | 0.940226 | Not queried | 3.58 | 5.56e-02 | 1.00e-01 |
|  | 1 | 29138919 | 29139278 | PDFSTATSENSEMBLUCSC | 360 | 5 | 371 | gene;island | 0.741434 | 0.989245 | Not queried | 3.58 | 3.17e-02 | 6.50e-02 |
|  | 5 | 169724590 | 169724803 | PDFSTATSENSEMBLUCSC | 214 | 5 | 31889 | gene | 0.384242 | 0.895692 | LCP2 (margin: LCP2; CTB-114C7.4) | 3.58 | 7.94e-03 | 3.19e-02 |
|  | 17 | 48046663 | 48046859 | PDFSTATSENSEMBLUCSC | 197 | 4 | 16676 | gene;island | 0.445483 | 0.057123 | Not queried | 3.58 | 1.14e-01 | 1.68e-01 |
|  | 11 | 7695165 | 7695528 | PDFSTATSENSEMBLUCSC | 364 | 7 | 7121 | tss;island | 0.527611 | 0.957813 | CYB5R2 (margin: CYB5R2) | 3.58 | 1.17e-03 | 1.13e-02 |
|  | 2 | 139660149 | 139660341 | PDFSTATSENSEMBLUCSC | 193 | 4 | 24765 | island | 0.746063 | 0.915171 | Not queried | 3.58 | 1.14e-01 | 1.68e-01 |
|  | 3 | 134369339 | 134369828 | PDFSTATSENSEMBLUCSC | 490 | 4 | 27957 | gene | 0.471229 | 0.910073 | Not queried | 3.57 | 1.14e-01 | 1.68e-01 |
|  | 1 | 60392140 | 60392567 | PDFSTATSENSEMBLUCSC | 428 | 6 | 3326 | island | 0.053968 | 0.007696 | Not queried | 3.57 | 2.60e-02 | 6.12e-02 |
|  | 1 | 60392140 | 60392392 | PDFSTATSENSEMBLUCSC | 253 | 4 | 560 | gene | 0.106208 | 0.007696 | Not queried | 3.57 | 5.71e-02 | 1.00e-01 |
|  | 7 | 29603121 | 29603541 | PDFSTATSENSEMBLUCSC | 421 | 8 | 38376 | island | 0.095105 | 0.006532 | PRR15; AC007255.8 (margin: RP11-550A18.1; PRR15; AC007255.8) | 3.57 | 1.04e-02 | 3.96e-02 |
|  | 7 | 143059218 | 143059300 | PDFSTATSENSEMBLUCSC | 83 | 6 | 37254 | gene;tss;island | 0.127323 | 0.012115 | Not queried | 3.57 | 1.52e-02 | 5.36e-02 |
|  | 19 | 49993125 | 49993217 | PDFSTATSENSEMBLUCSC | 93 | 6 | 20154 | gene;tss;island | 0.115124 | 0.607747 | RPL13A; SNORD32A; SNORD33; SNORD34; SNORD35A (margin: CTD-3148I10.9; FLT3LG; RPL13A; SNORD32A; SNORD33; SNORD34; SNORD35A; RPS11; SNORD35B; hsa-mir-150; MIR150) | 3.57 | 4.33e-03 | 2.52e-02 |
|  | 17 | 68165632 | 68165759 | PDFSTATSENSEMBLUCSC | 128 | 4 | 18696 | island | 0.064213 | 0.550409 | Not queried | 3.57 | 2.86e-02 | 6.12e-02 |
|  | 1 | 3649250 | 3649971 | PDFSTATSENSEMBLUCSC | 722 | 7 | 143 | gene;island | 0.871836 | 0.968040 | Not queried | 3.57 | 7.28e-02 | 1.23e-01 |
|  | 14 | 92788914 | 92789193 | PDFSTATSENSEMBLUCSC | 280 | 4 | 12566 | tss;island | 0.260520 | 0.725328 | Not queried | 3.57 | 2.00e-01 | 2.55e-01 |
|  | 21 | 37914924 | 37915391 | PDFSTATSENSEMBLUCSC | 468 | 7 | 26418 | gene;tss | 0.646491 | 0.977323 | CLDN14 (margin: AP000695.4; CLDN14) | 3.57 | 2.33e-03 | 1.52e-02 |
|  | 12 | 114846849 | 114847164 | PDFSTATSENSEMBLUCSC | 316 | 4 | 10034 | tss;island | 0.586756 | 0.942614 | Not queried | 3.57 | 2.86e-02 | 6.12e-02 |
|  | 19 | 58280728 | 58281019 | PDFSTATSENSEMBLUCSC | 292 | 9 | 21125 | tss | 0.057824 | 0.410222 | Not queried | 3.57 | 4.00e-02 | 8.03e-02 |
|  | 10 | 95361132 | 95361386 | PDFSTATSENSEMBLUCSC | 255 | 5 | 5239 | tss;island | 0.710132 | 0.961811 | FFAR4; RBP4 (margin: FFAR4; AL356214.1; PDE6C; RBP4) | 3.57 | 7.94e-03 | 3.19e-02 |
|  | 5 | 135265753 | 135265937 | PDFSTATSENSEMBLUCSC | 185 | 5 | 32308 | tss | 0.748095 | 0.972671 | Not queried | 3.57 | 5.56e-02 | 1.00e-01 |
|  | 17 | 52763 | 52989 | PDFSTATSENSEMBLUCSC | 227 | 4 | 17784 | island | 0.601562 | 0.959270 | Not queried | 3.57 | 2.00e-01 | 2.55e-01 |
|  | 12 | 101603174 | 101603581 | PDFSTATSENSEMBLUCSC | 408 | 4 | 9266 | gene;island | 0.567816 | 0.133348 | Not queried | 3.57 | 5.71e-02 | 1.00e-01 |
|  | 21 | 33941603 | 33942312 | PDFSTATSENSEMBLUCSC | 710 | 7 | 26636 | island | 0.163686 | 0.012552 | Not queried | 3.57 | 9.73e-02 | 1.55e-01 |
|  | 5 | 37834672 | 37835168 | PDFSTATSENSEMBLUCSC | 497 | 7 | 31590 | gene;island | 0.809852 | 0.975053 | GDNF (margin: GDNF-AS1; GDNF) | 3.57 | 1.11e-02 | 4.14e-02 |
|  | 16 | 68279304 | 68279613 | PDFSTATSENSEMBLUCSC | 310 | 4 | 14812 | gene | 0.003220 | 0.006114 | Not queried | 3.57 | 3.43e-01 | 3.84e-01 |
|  | 16 | 66878426 | 66878810 | PDFSTATSENSEMBLUCSC | 385 | 9 | 15286 | tss | 0.840890 | 0.981504 | CA7; NAE1; RP11-61A14.1 (margin: CA7; NAE1; RP11-61A14.1) | 3.57 | 4.11e-05 | 1.17e-03 |
|  | 16 | 66878426 | 66879016 | PDFSTATSENSEMBLUCSC | 591 | 11 | 14783 | gene;island | 0.813533 | 0.979166 | CA7; NAE1; RP11-61A14.1 (margin: CA7; NAE1; RP11-61A14.1) | 3.57 | 2.75e-04 | 4.46e-03 |
|  | 17 | 74965015 | 74965245 | PDFSTATSENSEMBLUCSC | 231 | 4 | 18799 | island | 0.300791 | 0.897973 | Not queried | 3.56 | 2.00e-01 | 2.55e-01 |
|  | 14 | 56232650 | 56233178 | PDFSTATSENSEMBLUCSC | 529 | 7 | 12871 | island | 0.576525 | 0.935591 | RP11-813I20.2; RPL13AP3 (margin: RP11-813I20.2; RPL13AP3) | 3.56 | 4.08e-03 | 2.47e-02 |
|  | 7 | 35077598 | 35077807 | PDFSTATSENSEMBLUCSC | 210 | 9 | 38405 | island | 0.134038 | 0.006068 | DPY19L1 (margin: DPY19L1) | 3.56 | 7.77e-03 | 3.19e-02 |
|  | 20 | 30028062 | 30028292 | PDFSTATSENSEMBLUCSC | 231 | 6 | 25657 | tss | 0.242930 | 0.805620 | Not queried | 3.56 | 1.52e-02 | 5.36e-02 |
|  | 6 | 100054469 | 100054615 | PDFSTATSENSEMBLUCSC | 147 | 6 | 35137 | tss | 0.811298 | 0.985431 | PRDM13 (margin: PRDM13) | 3.56 | 2.16e-03 | 1.45e-02 |
|  | 19 | 36909326 | 36909606 | PDFSTATSENSEMBLUCSC | 281 | 4 | 21939 | island | 0.778290 | 0.985729 | Not queried | 3.56 | 5.71e-02 | 1.00e-01 |
|  | 19 | 3801294 | 3801727 | PDFSTATSENSEMBLUCSC | 434 | 4 | 19723 | gene | 0.159890 | 0.016387 | Not queried | 3.56 | 3.43e-01 | 3.84e-01 |
|  | 8 | 914335 | 914572 | PDFSTATSENSEMBLUCSC | 238 | 5 | 40009 | island | 0.946157 | 0.519069 | Not queried | 3.56 | 5.56e-02 | 1.00e-01 |
|  | 6 | 32061084 | 32061478 | PDFSTATSENSEMBLUCSC | 395 | 4 | 34044 | gene;island | 0.258518 | 0.762546 | Not queried | 3.56 | 2.00e-01 | 2.55e-01 |
|  | 19 | 46379763 | 46380134 | PDFSTATSENSEMBLUCSC | 372 | 4 | 22160 | island | 0.439305 | 0.941256 | Not queried | 3.56 | 5.71e-02 | 1.00e-01 |
|  | 7 | 6655711 | 6655973 | PDFSTATSENSEMBLUCSC | 263 | 4 | 36887 | gene;island | 0.700975 | 0.938604 | Not queried | 3.56 | 2.86e-02 | 6.12e-02 |
|  | 4 | 110223040 | 110223795 | PDFSTATSENSEMBLUCSC | 756 | 10 | 30089 | gene | 0.661326 | 0.962103 | AC004051.2; COL25A1 (margin: AC004051.2; COL25A1) | 3.56 | 5.20e-03 | 2.96e-02 |
|  | 5 | 26669610 | 26669882 | PDFSTATSENSEMBLUCSC | 273 | 4 | 32712 | island | 0.811172 | 0.336557 | Not queried | 3.56 | 2.86e-02 | 6.12e-02 |
|  | 11 | 70557445 | 70557881 | PDFSTATSENSEMBLUCSC | 437 | 6 | 6794 | gene | 0.602528 | 0.963842 | Not queried | 3.56 | 1.52e-02 | 5.36e-02 |
|  | 10 | 131671373 | 131671830 | PDFSTATSENSEMBLUCSC | 458 | 6 | 4832 | gene | 0.179253 | 0.768603 | Not queried | 3.56 | 2.60e-02 | 6.12e-02 |
|  | 17 | 72368048 | 72368401 | PDFSTATSENSEMBLUCSC | 354 | 5 | 16755 | gene;island | 0.711246 | 0.907659 | Not queried | 3.56 | 1.51e-01 | 2.11e-01 |
|  | 7 | 145813413 | 145813494 | PDFSTATSENSEMBLUCSC | 82 | 7 | 38981 | island | 0.927914 | 0.514568 | Not queried | 3.56 | 1.75e-02 | 5.79e-02 |
|  | 1 | 236511281 | 236511627 | PDFSTATSENSEMBLUCSC | 347 | 4 | 4360 | island | 0.814544 | 0.979040 | Not queried | 3.55 | 2.86e-02 | 6.12e-02 |
|  | 17 | 8661887 | 8662011 | PDFSTATSENSEMBLUCSC | 125 | 5 | 17121 | tss | 0.376481 | 0.839120 | Not queried | 3.55 | 9.52e-02 | 1.53e-01 |
|  | 11 | 123986026 | 123986094 | PDFSTATSENSEMBLUCSC | 69 | 5 | 7781 | tss | 0.001365 | 0.002690 | Not queried | 3.55 | 5.48e-01 | 5.70e-01 |
|  | 17 | 60885856 | 60886079 | PDFSTATSENSEMBLUCSC | 224 | 4 | 17562 | tss | 0.713146 | 0.582962 | Not queried | 3.55 | 4.86e-01 | 5.11e-01 |
|  | 11 | 63775071 | 63775612 | PDFSTATSENSEMBLUCSC | 542 | 5 | 6658 | gene;island | 0.736348 | 0.982606 | Not queried | 3.55 | 1.59e-02 | 5.36e-02 |
|  | 1 | 210856989 | 210857380 | PDFSTATSENSEMBLUCSC | 392 | 5 | 992 | gene;island | 0.614488 | 0.949080 | KCNH1 (margin: HHAT; KCNH1) | 3.55 | 7.94e-03 | 3.19e-02 |
|  | 7 | 122527351 | 122527522 | PDFSTATSENSEMBLUCSC | 172 | 5 | 37868 | tss;island | 0.298352 | 0.016774 | Not queried | 3.55 | 1.51e-01 | 2.11e-01 |
|  | 2 | 132152167 | 132152543 | PDFSTATSENSEMBLUCSC | 377 | 4 | 24730 | island | 0.611282 | 0.932150 | Not queried | 3.55 | 2.86e-02 | 6.12e-02 |
|  | 19 | 840737 | 841082 | PDFSTATSENSEMBLUCSC | 346 | 6 | 21178 | island | 0.314497 | 0.832261 | PRTN3 (margin: AZU1; PRTN3; ELANE) | 3.55 | 8.66e-03 | 3.36e-02 |
|  | 17 | 72244242 | 72244469 | PDFSTATSENSEMBLUCSC | 228 | 4 | 16749 | gene;tss | 0.421834 | 0.893899 | Not queried | 3.55 | 1.14e-01 | 1.68e-01 |
|  | 2 | 219824293 | 219824395 | PDFSTATSENSEMBLUCSC | 103 | 5 | 23886 | tss | 0.045047 | 0.005569 | Not queried | 3.55 | 3.17e-02 | 6.50e-02 |
|  | 12 | 2788627 | 2788847 | PDFSTATSENSEMBLUCSC | 221 | 5 | 9005 | gene;island | 0.789263 | 0.984477 | CACNA1C; CACNA1C-AS1 (margin: CACNA1C; CACNA1C-AS2; CACNA1C-AS1) | 3.55 | 7.94e-03 | 3.19e-02 |
|  | 12 | 54785171 | 54785320 | PDFSTATSENSEMBLUCSC | 150 | 4 | 9763 | tss | 0.089331 | 0.013726 | Not queried | 3.55 | 2.00e-01 | 2.55e-01 |
|  | 3 | 131080235 | 131080927 | PDFSTATSENSEMBLUCSC | 693 | 9 | 29479 | island | 0.459429 | 0.915605 | NUDT16P; RP11-933H2.4 (margin: NEK11; NUDT16P; RP11-933H2.4) | 3.54 | 1.65e-04 | 2.80e-03 |
|  | 3 | 131080235 | 131080697 | PDFSTATSENSEMBLUCSC | 463 | 7 | 28593 | tss | 0.456731 | 0.888109 | NUDT16P; RP11-933H2.4 (margin: NEK11; NUDT16P; RP11-933H2.4) | 3.54 | 1.17e-03 | 1.13e-02 |
|  | 3 | 136537607 | 136537856 | PDFSTATSENSEMBLUCSC | 250 | 8 | 28614 | tss | 0.735935 | 0.964507 | SLC35G2 (margin: SLC35G2; RP11-731C17.1) | 3.54 | 1.55e-04 | 2.71e-03 |
|  | 19 | 14542609 | 14543048 | PDFSTATSENSEMBLUCSC | 440 | 4 | 21680 | island | 0.721310 | 0.958404 | Not queried | 3.54 | 2.86e-02 | 6.12e-02 |
|  | 15 | 73735509 | 73735694 | PDFSTATSENSEMBLUCSC | 186 | 4 | 13412 | gene | 0.611776 | 0.975644 | Not queried | 3.54 | 5.71e-02 | 1.00e-01 |
|  | 1 | 3663164 | 3664084 | PDFSTATSENSEMBLUCSC | 921 | 10 | 2570 | island | 0.629121 | 0.958372 | TP73-AS1 (margin: TP73; CCDC27; TP73-AS1) | 3.54 | 1.50e-03 | 1.41e-02 |
|  | 3 | 64008007 | 64008387 | PDFSTATSENSEMBLUCSC | 381 | 5 | 27847 | gene;island | 0.537213 | 0.477915 | Not queried | 3.54 | 5.48e-01 | 5.70e-01 |
|  | 5 | 1174426 | 1174666 | PDFSTATSENSEMBLUCSC | 241 | 4 | 32611 | island | 0.027188 | 0.222636 | Not queried | 3.54 | 2.00e-01 | 2.55e-01 |
|  | 2 | 1748872 | 1749076 | PDFSTATSENSEMBLUCSC | 205 | 4 | 23158 | tss;island | 0.464124 | 0.925870 | Not queried | 3.54 | 2.00e-01 | 2.55e-01 |
|  | 17 | 7165831 | 7165909 | PDFSTATSENSEMBLUCSC | 79 | 6 | 17069 | tss;island | 0.077430 | 0.009160 | RP1-4G17.5; CLDN7 (margin: ELP5; CTDNEP1; RP1-4G17.5; CLDN7; Y\_RNA) | 3.54 | 2.16e-03 | 1.45e-02 |
|  | 11 | 2029309 | 2029688 | PDFSTATSENSEMBLUCSC | 380 | 4 | 7948 | island | 0.654497 | 0.965531 | Not queried | 3.54 | 1.14e-01 | 1.68e-01 |
|  | 7 | 1248693 | 1249199 | PDFSTATSENSEMBLUCSC | 507 | 7 | 38088 | island | 0.591919 | 0.979996 | (margin: ) | 3.54 | 1.17e-03 | 1.13e-02 |
|  | 17 | 11501999 | 11502254 | PDFSTATSENSEMBLUCSC | 256 | 4 | 16445 | gene;island | 0.476048 | 0.835779 | Not queried | 3.54 | 2.86e-02 | 6.12e-02 |
|  | 20 | 34204988 | 34205182 | PDFSTATSENSEMBLUCSC | 195 | 4 | 25407 | gene;island | 0.521621 | 0.129594 | Not queried | 3.54 | 2.86e-02 | 6.12e-02 |
|  | 6 | 33771799 | 33772017 | PDFSTATSENSEMBLUCSC | 219 | 4 | 34914 | tss | 0.221180 | 0.858714 | Not queried | 3.54 | 2.00e-01 | 2.55e-01 |
|  | 5 | 537349 | 537906 | PDFSTATSENSEMBLUCSC | 558 | 7 | 32589 | island | 0.592687 | 0.946129 | MIR4456 (margin: RP11-310P5.2; MIR4456) | 3.54 | 6.99e-03 | 3.19e-02 |
|  | 7 | 100823700 | 100823790 | PDFSTATSENSEMBLUCSC | 91 | 4 | 37800 | tss;island | 0.362367 | 0.913065 | Not queried | 3.54 | 5.71e-02 | 1.00e-01 |
|  | 6 | 24358304 | 24358327 | PDFSTATSENSEMBLUCSC | 24 | 4 | 34667 | tss | 0.874109 | 0.366622 | Not queried | 3.54 | 2.86e-02 | 6.12e-02 |
|  | 11 | 110583543 | 110583662 | PDFSTATSENSEMBLUCSC | 120 | 6 | 7679 | tss | 0.454362 | 0.903884 | ARHGAP20 (margin: ARHGAP20) | 3.54 | 4.33e-03 | 2.52e-02 |
|  | 1 | 91191583 | 91192162 | PDFSTATSENSEMBLUCSC | 580 | 6 | 3463 | island | 0.688544 | 0.976154 | (margin: BARHL2) | 3.53 | 4.33e-03 | 2.52e-02 |
|  | 6 | 32974843 | 32975360 | PDFSTATSENSEMBLUCSC | 518 | 13 | 34176 | gene;island | 0.181833 | 0.776744 | HLA-DOA (margin: HLA-DOA) | 3.53 | 7.95e-04 | 8.28e-03 |
|  | 6 | 148881126 | 148881461 | PDFSTATSENSEMBLUCSC | 336 | 4 | 36551 | island | 0.473700 | 0.089351 | Not queried | 3.53 | 3.43e-01 | 3.84e-01 |
|  | 1 | 65774857 | 65775145 | PDFSTATSENSEMBLUCSC | 289 | 5 | 569 | gene;island | 0.614254 | 0.021843 | Not queried | 3.53 | 1.51e-01 | 2.11e-01 |
|  | 10 | 44500693 | 44501318 | PDFSTATSENSEMBLUCSC | 626 | 6 | 5704 | island | 0.643482 | 0.960450 | (margin: ) | 3.53 | 8.66e-03 | 3.36e-02 |
|  | 11 | 115374969 | 115375432 | PDFSTATSENSEMBLUCSC | 464 | 8 | 8828 | island | 0.075958 | 0.018119 | Not queried | 3.53 | 1.48e-02 | 5.36e-02 |
|  | 2 | 240844772 | 240845060 | PDFSTATSENSEMBLUCSC | 289 | 4 | 25263 | island | 0.632978 | 0.965557 | Not queried | 3.53 | 5.71e-02 | 1.00e-01 |
|  | 11 | 117747689 | 117748132 | PDFSTATSENSEMBLUCSC | 444 | 6 | 6923 | gene | 0.525537 | 0.555588 | Not queried | 3.53 | 5.89e-01 | 6.11e-01 |
|  | 8 | 33424653 | 33425043 | PDFSTATSENSEMBLUCSC | 391 | 5 | 39641 | tss;island | 0.140269 | 0.015527 | Not queried | 3.53 | 3.10e-01 | 3.66e-01 |
|  | 1 | 92951355 | 92952268 | PDFSTATSENSEMBLUCSC | 914 | 12 | 636 | gene | 0.283928 | 0.019110 | Not queried | 3.53 | 1.45e-02 | 5.34e-02 |
|  | 5 | 168174689 | 168174935 | PDFSTATSENSEMBLUCSC | 247 | 4 | 31885 | gene | 0.556768 | 0.849764 | Not queried | 3.53 | 3.43e-01 | 3.84e-01 |
|  | 3 | 35680791 | 35680899 | PDFSTATSENSEMBLUCSC | 109 | 4 | 28977 | island | 0.824325 | 0.405991 | Not queried | 3.53 | 5.71e-02 | 1.00e-01 |
|  | 5 | 178017571 | 178018186 | PDFSTATSENSEMBLUCSC | 616 | 10 | 32540 | tss | 0.713695 | 0.951440 | COL23A1 (margin: COL23A1; CLK4) | 3.52 | 7.25e-04 | 7.74e-03 |
|  | 2 | 74741573 | 74741941 | PDFSTATSENSEMBLUCSC | 369 | 5 | 24443 | island | 0.366787 | 0.919492 | TLX2 (margin: LBX2-AS1; TLX2; LBX2; PCGF1; DQX1; AUP1) | 3.52 | 7.94e-03 | 3.19e-02 |
|  | 4 | 1800987 | 1801294 | PDFSTATSENSEMBLUCSC | 308 | 4 | 29876 | gene;island | 0.766981 | 0.974823 | Not queried | 3.52 | 2.86e-02 | 6.12e-02 |
|  | 1 | 8378213 | 8378538 | PDFSTATSENSEMBLUCSC | 326 | 4 | 2636 | island | 0.509980 | 0.084647 | Not queried | 3.52 | 3.43e-01 | 3.84e-01 |
|  | 1 | 36108206 | 36108233 | PDFSTATSENSEMBLUCSC | 28 | 4 | 1486 | tss;island | 0.351582 | 0.050021 | Not queried | 3.52 | 5.71e-02 | 1.00e-01 |
|  | 12 | 48398320 | 48398730 | PDFSTATSENSEMBLUCSC | 411 | 7 | 9665 | tss;island | 0.663298 | 0.959711 | COL2A1; RP1-228P16.3 (margin: RP1-228P16.4; COL2A1; RP1-228P16.3) | 3.52 | 4.08e-03 | 2.47e-02 |
|  | 4 | 3387281 | 3387765 | PDFSTATSENSEMBLUCSC | 485 | 6 | 29905 | gene | 0.277378 | 0.882827 | Not queried | 3.52 | 1.52e-02 | 5.36e-02 |
|  | 20 | 709016 | 709392 | PDFSTATSENSEMBLUCSC | 377 | 4 | 25928 | island | 0.772886 | 0.173686 | Not queried | 3.52 | 1.14e-01 | 1.68e-01 |
|  | 1 | 6265071 | 6265417 | PDFSTATSENSEMBLUCSC | 347 | 4 | 1200 | tss;island | 0.207517 | 0.015231 | Not queried | 3.52 | 3.43e-01 | 3.84e-01 |
|  | 4 | 42399699 | 42399851 | PDFSTATSENSEMBLUCSC | 153 | 6 | 30367 | tss | 0.821382 | 0.983794 | SHISA3 (margin: SHISA3; RP11-63A11.1; ATP8A1) | 3.52 | 2.16e-03 | 1.45e-02 |
|  | 10 | 98480463 | 98480474 | PDFSTATSENSEMBLUCSC | 12 | 4 | 5255 | tss;island | 0.065646 | 0.008833 | Not queried | 3.52 | 2.86e-02 | 6.12e-02 |
|  | 12 | 124788627 | 124788814 | PDFSTATSENSEMBLUCSC | 188 | 4 | 9388 | gene;island | 0.520414 | 0.931347 | Not queried | 3.52 | 5.71e-02 | 1.00e-01 |
|  | 3 | 52479069 | 52479108 | PDFSTATSENSEMBLUCSC | 40 | 4 | 28380 | tss | 0.174271 | 0.024699 | Not queried | 3.52 | 2.86e-02 | 6.12e-02 |
|  | 11 | 65222227 | 65222786 | PDFSTATSENSEMBLUCSC | 560 | 6 | 8457 | island | 0.006617 | 0.319429 | Not queried | 3.52 | 6.49e-02 | 1.11e-01 |
|  | 2 | 43019484 | 43020457 | PDFSTATSENSEMBLUCSC | 974 | 11 | 24245 | island | 0.641145 | 0.959661 | HAAO (margin: HAAO; FTOP1) | 3.51 | 1.28e-04 | 2.71e-03 |
|  | 11 | 7272653 | 7273154 | PDFSTATSENSEMBLUCSC | 502 | 8 | 7120 | tss;island | 0.949009 | 0.453788 | Not queried | 3.51 | 8.30e-02 | 1.40e-01 |
|  | 10 | 134599783 | 134600998 | PDFSTATSENSEMBLUCSC | 1216 | 30 | 5450 | tss;island | 0.849043 | 0.979283 | RP11-288G11.3; NKX6-2 (margin: INPP5A; RP11-288G11.3; NKX6-2) | 3.51 | 1.09e-10 | 7.05e-08 |
|  | 14 | 38677095 | 38677295 | PDFSTATSENSEMBLUCSC | 201 | 4 | 12801 | island | 0.435609 | 0.919618 | Not queried | 3.51 | 5.71e-02 | 1.00e-01 |
|  | 7 | 50132541 | 50132764 | PDFSTATSENSEMBLUCSC | 224 | 4 | 37010 | gene | 0.634262 | 0.970865 | Not queried | 3.51 | 3.43e-01 | 3.84e-01 |
|  | 1 | 110626868 | 110627197 | PDFSTATSENSEMBLUCSC | 330 | 5 | 3574 | island | 0.484492 | 0.925918 | Not queried | 3.51 | 1.59e-02 | 5.36e-02 |
|  | 19 | 12075601 | 12076348 | PDFSTATSENSEMBLUCSC | 748 | 11 | 21588 | island | 0.497687 | 0.840142 | ZNF763 (margin: ZNF763; CTD-2006C1.12) | 3.51 | 5.53e-04 | 6.47e-03 |
|  | 19 | 12075601 | 12075848 | PDFSTATSENSEMBLUCSC | 248 | 5 | 20486 | tss | 0.497687 | 0.884462 | Not queried | 3.51 | 3.17e-02 | 6.50e-02 |
|  | 17 | 62493555 | 62493832 | PDFSTATSENSEMBLUCSC | 278 | 4 | 17583 | tss;island | 0.020078 | 0.127873 | Not queried | 3.51 | 1.14e-01 | 1.68e-01 |
|  | 2 | 25141532 | 25142523 | PDFSTATSENSEMBLUCSC | 992 | 11 | 24135 | island | 0.065510 | 0.960411 | Not queried | 3.51 | 1.01e-01 | 1.62e-01 |
|  | 4 | 6576585 | 6576759 | PDFSTATSENSEMBLUCSC | 175 | 4 | 30280 | tss;island | 0.302847 | 0.012590 | Not queried | 3.51 | 2.00e-01 | 2.55e-01 |
|  | 6 | 152957910 | 152958489 | PDFSTATSENSEMBLUCSC | 580 | 10 | 34517 | gene | 0.257524 | 0.016874 | Not queried | 3.51 | 1.47e-02 | 5.36e-02 |
|  | 8 | 104512858 | 104513186 | PDFSTATSENSEMBLUCSC | 329 | 4 | 40521 | island | 0.286852 | 0.020953 | Not queried | 3.51 | 1.14e-01 | 1.68e-01 |
|  | 4 | 57522493 | 57523055 | PDFSTATSENSEMBLUCSC | 563 | 9 | 30010 | gene;island | 0.775855 | 0.968619 | Not queried | 3.51 | 4.00e-02 | 8.03e-02 |
|  | 13 | 112721712 | 112721950 | PDFSTATSENSEMBLUCSC | 239 | 7 | 11910 | island | 0.643848 | 0.959018 | Not queried | 3.51 | 2.62e-02 | 6.12e-02 |
|  | 6 | 167508001 | 167508226 | PDFSTATSENSEMBLUCSC | 226 | 6 | 36642 | island | 0.613736 | 0.955424 | Not queried | 3.51 | 1.52e-02 | 5.36e-02 |
|  | 12 | 49582874 | 49583112 | PDFSTATSENSEMBLUCSC | 239 | 7 | 9684 | tss | 0.091980 | 0.008150 | TUBA1C; TUBA1A (margin: TUBA1C; TUBA1A) | 3.50 | 1.17e-03 | 1.13e-02 |
|  | 12 | 54785003 | 54785320 | PDFSTATSENSEMBLUCSC | 318 | 7 | 10504 | island | 0.111177 | 0.017409 | Not queried | 3.50 | 1.75e-02 | 5.79e-02 |
|  | 6 | 30068471 | 30069319 | PDFSTATSENSEMBLUCSC | 849 | 18 | 35659 | island | 0.005996 | 0.055257 | TRIM31 (margin: TRIM31-AS1; AL669914.1; TRIM31) | 3.50 | 1.13e-04 | 2.70e-03 |
|  | 8 | 53322451 | 53322619 | PDFSTATSENSEMBLUCSC | 169 | 4 | 39697 | tss | 0.366804 | 0.751733 | Not queried | 3.50 | 3.43e-01 | 3.84e-01 |
|  | 13 | 114807633 | 114807826 | PDFSTATSENSEMBLUCSC | 194 | 4 | 11309 | gene;island | 0.365513 | 0.971912 | Not queried | 3.50 | 2.00e-01 | 2.55e-01 |
|  | 10 | 131505762 | 131505869 | PDFSTATSENSEMBLUCSC | 108 | 4 | 4830 | gene | 0.532770 | 0.971168 | Not queried | 3.50 | 3.43e-01 | 3.84e-01 |
|  | 1 | 202830475 | 202830826 | PDFSTATSENSEMBLUCSC | 352 | 8 | 2182 | tss;island | 0.154165 | 0.012097 | RP11-480I12.7; RP11-480I12.5 (margin: RP11-480I12.7; RP11-480I12.9; RP11-480I12.5) | 3.50 | 1.55e-04 | 2.71e-03 |
|  | 7 | 155601876 | 155602259 | PDFSTATSENSEMBLUCSC | 384 | 5 | 37312 | gene;island | 0.737924 | 0.980912 | Not queried | 3.50 | 9.52e-02 | 1.53e-01 |
|  | 17 | 7791882 | 7792134 | PDFSTATSENSEMBLUCSC | 253 | 5 | 17102 | tss | 0.244385 | 0.033380 | CHD3 (margin: CHD3; LSMD1) | 3.50 | 7.94e-03 | 3.19e-02 |
|  | 12 | 133010684 | 133010946 | PDFSTATSENSEMBLUCSC | 263 | 5 | 11054 | island | 0.184642 | 0.888708 | Not queried | 3.50 | 5.56e-02 | 1.00e-01 |
|  | 11 | 66314211 | 66314378 | PDFSTATSENSEMBLUCSC | 168 | 5 | 7473 | tss;island | 0.125978 | 0.635371 | ACTN3; ZDHHC24 (margin: ACTN3; ZDHHC24; CTD-3074O7.12; CTD-3074O7.2) | 3.50 | 7.94e-03 | 3.19e-02 |
|  | 10 | 50506748 | 50507092 | PDFSTATSENSEMBLUCSC | 345 | 7 | 5071 | tss | 0.541845 | 0.172163 | Not queried | 3.50 | 1.75e-02 | 5.79e-02 |
|  | 6 | 33053576 | 33054001 | PDFSTATSENSEMBLUCSC | 426 | 6 | 34187 | gene | 0.209625 | 0.603471 | HLA-DPB1 (margin: HLA-DPB1; RPL32P1; HLA-DPA1; HLA-DPA2) | 3.50 | 4.33e-03 | 2.52e-02 |
|  | 12 | 110211321 | 110211413 | PDFSTATSENSEMBLUCSC | 93 | 5 | 9995 | tss | 0.329349 | 0.912927 | FAM222A-AS1 (margin: FAM222A; FAM222A-AS1; TRPV4) | 3.50 | 7.94e-03 | 3.19e-02 |
|  | 7 | 158886098 | 158886532 | PDFSTATSENSEMBLUCSC | 435 | 6 | 37384 | gene;island | 0.611297 | 0.957890 | VIPR2 (margin: VIPR2) | 3.50 | 4.33e-03 | 2.52e-02 |
|  | 16 | 24267226 | 24267559 | PDFSTATSENSEMBLUCSC | 334 | 4 | 14697 | gene;island | 0.752818 | 0.209671 | Not queried | 3.49 | 1.14e-01 | 1.68e-01 |
|  | 12 | 49504691 | 49505053 | PDFSTATSENSEMBLUCSC | 363 | 6 | 9682 | tss | 0.008384 | 0.397149 | Not queried | 3.49 | 3.10e-01 | 3.66e-01 |
|  | 6 | 32028831 | 32029216 | PDFSTATSENSEMBLUCSC | 386 | 8 | 34025 | gene | 0.434914 | 0.924147 | TNXB (margin: TNXB) | 3.49 | 3.11e-04 | 4.77e-03 |
|  | 1 | 3059051 | 3059267 | PDFSTATSENSEMBLUCSC | 217 | 4 | 103 | gene;island | 0.221182 | 0.765027 | Not queried | 3.49 | 2.86e-02 | 6.12e-02 |
|  | 11 | 58731357 | 58731634 | PDFSTATSENSEMBLUCSC | 278 | 4 | 8299 | island | 0.484552 | 0.895630 | Not queried | 3.49 | 2.86e-02 | 6.12e-02 |
|  | 19 | 23941207 | 23942126 | PDFSTATSENSEMBLUCSC | 920 | 13 | 21848 | island | 0.409618 | 0.075129 | ZNF681 (margin: RP11-255H23.2; RPSAP58; ZNF681) | 3.49 | 1.20e-02 | 4.46e-02 |
|  | 13 | 113119625 | 113119864 | PDFSTATSENSEMBLUCSC | 240 | 4 | 11922 | island | 0.574905 | 0.926793 | Not queried | 3.49 | 2.00e-01 | 2.55e-01 |
|  | 8 | 11561283 | 11561620 | PDFSTATSENSEMBLUCSC | 338 | 9 | 39553 | tss | 0.759037 | 0.956133 | GATA4 (margin: GATA4) | 3.49 | 8.23e-05 | 2.06e-03 |
|  | 8 | 11561283 | 11561728 | PDFSTATSENSEMBLUCSC | 446 | 11 | 40087 | island | 0.781308 | 0.973173 | GATA4 (margin: GATA4) | 3.49 | 8.51e-05 | 2.07e-03 |
|  | 17 | 37784694 | 37784895 | PDFSTATSENSEMBLUCSC | 202 | 4 | 16549 | gene;island | 0.745751 | 0.978043 | Not queried | 3.49 | 5.71e-02 | 1.00e-01 |
|  | 10 | 14014419 | 14014651 | PDFSTATSENSEMBLUCSC | 233 | 5 | 4506 | gene | 0.313760 | 0.816647 | Not queried | 3.49 | 9.52e-02 | 1.53e-01 |
|  | 12 | 6419409 | 6419750 | PDFSTATSENSEMBLUCSC | 342 | 6 | 9506 | tss;island | 0.946476 | 0.545470 | PLEKHG6 (margin: PLEKHG6) | 3.49 | 2.16e-03 | 1.45e-02 |
|  | 1 | 41156730 | 41157266 | PDFSTATSENSEMBLUCSC | 537 | 8 | 457 | gene;tss;island | 0.109347 | 0.025890 | Not queried | 3.49 | 1.48e-02 | 5.36e-02 |
|  | 6 | 132931714 | 132931926 | PDFSTATSENSEMBLUCSC | 213 | 4 | 35228 | tss | 0.630168 | 0.981136 | Not queried | 3.48 | 5.71e-02 | 1.00e-01 |
|  | 4 | 74734714 | 74735101 | PDFSTATSENSEMBLUCSC | 388 | 9 | 30430 | tss | 0.170937 | 0.433298 | CXCL1 (margin: CXCL1) | 3.48 | 3.99e-03 | 2.47e-02 |
|  | 19 | 11689488 | 11689726 | PDFSTATSENSEMBLUCSC | 239 | 6 | 19846 | gene | 0.825926 | 0.981594 | ZNF627; ACP5 (margin: ZNF627; AC020947.2; ACP5) | 3.48 | 8.66e-03 | 3.36e-02 |
|  | 19 | 17791074 | 17791354 | PDFSTATSENSEMBLUCSC | 281 | 4 | 19919 | gene;island | 0.492597 | 0.156112 | Not queried | 3.48 | 4.86e-01 | 5.11e-01 |
|  | 15 | 40544855 | 40545511 | PDFSTATSENSEMBLUCSC | 657 | 11 | 13290 | gene;island | 0.794845 | 0.979413 | RP11-133K1.2; PAK6; C15orf56 (margin: RP11-133K1.2; PAK6; C15orf56) | 3.48 | 1.13e-05 | 5.02e-04 |
|  | 5 | 2642995 | 2643299 | PDFSTATSENSEMBLUCSC | 305 | 4 | 32654 | island | 0.434262 | 0.849957 | Not queried | 3.48 | 2.00e-01 | 2.55e-01 |
|  | 6 | 167763758 | 167764146 | PDFSTATSENSEMBLUCSC | 389 | 4 | 36643 | island | 0.614336 | 0.931701 | Not queried | 3.48 | 2.86e-02 | 6.12e-02 |
|  | 20 | 13200929 | 13200992 | PDFSTATSENSEMBLUCSC | 64 | 10 | 25601 | tss | 0.841141 | 0.986677 | ISM1 (margin: ISM1) | 3.48 | 1.08e-05 | 4.82e-04 |
|  | 8 | 25902400 | 25902611 | PDFSTATSENSEMBLUCSC | 212 | 5 | 39608 | tss | 0.507975 | 0.086010 | EBF2 (margin: EBF2) | 3.48 | 7.94e-03 | 3.19e-02 |
|  | 17 | 80692947 | 80693292 | PDFSTATSENSEMBLUCSC | 346 | 4 | 17780 | tss;island | 0.184246 | 0.804098 | Not queried | 3.48 | 1.14e-01 | 1.68e-01 |
|  | 17 | 37762842 | 37763088 | PDFSTATSENSEMBLUCSC | 247 | 5 | 16548 | gene;island | 0.123097 | 0.012799 | NEUROD2 (margin: NEUROD2) | 3.48 | 7.94e-03 | 3.19e-02 |
|  | 14 | 102028994 | 102029375 | PDFSTATSENSEMBLUCSC | 382 | 4 | 12215 | gene;island | 0.138963 | 0.660370 | Not queried | 3.48 | 2.86e-02 | 6.12e-02 |
|  | 15 | 69590852 | 69591194 | PDFSTATSENSEMBLUCSC | 343 | 7 | 13762 | tss | 0.079323 | 0.488697 | Not queried | 3.48 | 1.65e-01 | 2.28e-01 |
|  | 19 | 54106789 | 54106910 | PDFSTATSENSEMBLUCSC | 122 | 4 | 21032 | tss | 0.618846 | 0.906021 | Not queried | 3.48 | 5.71e-02 | 1.00e-01 |
|  | 2 | 219925065 | 219925330 | PDFSTATSENSEMBLUCSC | 266 | 7 | 25105 | island | 0.844241 | 0.982482 | IHH; MIR3131 (margin: IHH; MIR3131) | 3.48 | 6.99e-03 | 3.19e-02 |
|  | 8 | 143373411 | 143373735 | PDFSTATSENSEMBLUCSC | 325 | 4 | 39457 | gene | 0.638933 | 0.941389 | Not queried | 3.48 | 2.86e-02 | 6.12e-02 |
|  | 7 | 14028690 | 14029085 | PDFSTATSENSEMBLUCSC | 396 | 6 | 36898 | gene;island | 0.537135 | 0.079549 | ETV1 (margin: ETV1) | 3.48 | 4.33e-03 | 2.52e-02 |
|  | 6 | 168389700 | 168390347 | PDFSTATSENSEMBLUCSC | 648 | 6 | 36652 | island | 0.634189 | 0.955925 | (margin: KIF25; RP3-470B24.5; KIF25-AS1) | 3.48 | 2.16e-03 | 1.45e-02 |
|  | 1 | 26249349 | 26249725 | PDFSTATSENSEMBLUCSC | 377 | 5 | 2883 | island | 0.179940 | 0.016291 | Not queried | 3.48 | 3.17e-02 | 6.50e-02 |
|  | 1 | 186649153 | 186649985 | PDFSTATSENSEMBLUCSC | 833 | 13 | 4047 | island | 0.188892 | 0.018763 | PTGS2 (margin: PTGS2) | 3.47 | 2.97e-04 | 4.72e-03 |
|  | 5 | 1633830 | 1634218 | PDFSTATSENSEMBLUCSC | 389 | 5 | 32636 | island | 0.096762 | 0.017344 | Not queried | 3.47 | 1.59e-02 | 5.36e-02 |
|  | 6 | 32185818 | 32186244 | PDFSTATSENSEMBLUCSC | 427 | 9 | 34097 | gene | 0.239205 | 0.728843 | NOTCH4 (margin: NOTCH4) | 3.47 | 4.94e-04 | 6.47e-03 |
|  | 2 | 1846447 | 1846907 | PDFSTATSENSEMBLUCSC | 461 | 5 | 22538 | gene;island | 0.674582 | 0.957169 | Not queried | 3.47 | 3.17e-02 | 6.50e-02 |
|  | 19 | 14016603 | 14016822 | PDFSTATSENSEMBLUCSC | 220 | 7 | 19887 | gene | 0.898744 | 0.481140 | Not queried | 3.47 | 3.18e-01 | 3.74e-01 |
|  | 16 | 1041053 | 1041333 | PDFSTATSENSEMBLUCSC | 281 | 6 | 15505 | island | 0.604968 | 0.935065 | RP11-161M6.3 (margin: AC009041.2; SOX8; LMF1; RP11-161M6.2; RP11-161M6.3) | 3.47 | 8.66e-03 | 3.36e-02 |
|  | 11 | 20177746 | 20178138 | PDFSTATSENSEMBLUCSC | 393 | 4 | 8123 | island | 0.899963 | 0.979414 | Not queried | 3.47 | 2.86e-02 | 6.12e-02 |
|  | 4 | 141348862 | 141349096 | PDFSTATSENSEMBLUCSC | 235 | 7 | 30606 | tss | 0.068214 | 0.004923 | Not queried | 3.47 | 2.62e-02 | 6.12e-02 |
|  | 1 | 47489282 | 47489543 | PDFSTATSENSEMBLUCSC | 262 | 4 | 514 | gene | 0.281979 | 0.888646 | Not queried | 3.47 | 1.14e-01 | 1.68e-01 |
|  | 13 | 100643858 | 100644657 | PDFSTATSENSEMBLUCSC | 800 | 8 | 11850 | island | 0.841986 | 0.971337 | (margin: ZIC2; LINC00554) | 3.47 | 1.86e-03 | 1.45e-02 |
|  | 20 | 39995539 | 39995747 | PDFSTATSENSEMBLUCSC | 209 | 7 | 25746 | tss;island | 0.379364 | 0.938970 | EMILIN3 (margin: LPIN3; EMILIN3) | 3.47 | 4.08e-03 | 2.47e-02 |
|  | 2 | 7171869 | 7172097 | PDFSTATSENSEMBLUCSC | 229 | 4 | 22559 | gene;island | 0.246142 | 0.022593 | Not queried | 3.47 | 2.86e-02 | 6.12e-02 |
|  | 6 | 97285024 | 97285405 | PDFSTATSENSEMBLUCSC | 382 | 5 | 36335 | island | 0.141217 | 0.014368 | Not queried | 3.47 | 5.56e-02 | 1.00e-01 |
|  | 4 | 71337658 | 71337873 | PDFSTATSENSEMBLUCSC | 216 | 4 | 30016 | gene | 0.507840 | 0.830346 | Not queried | 3.47 | 3.43e-01 | 3.84e-01 |
|  | 1 | 111098150 | 111098456 | PDFSTATSENSEMBLUCSC | 307 | 5 | 3581 | island | 0.587950 | 0.940266 | Not queried | 3.47 | 1.51e-01 | 2.11e-01 |
|  | 1 | 99126913 | 99127446 | PDFSTATSENSEMBLUCSC | 534 | 6 | 3516 | island | 0.007384 | 0.032803 | Not queried | 3.47 | 6.49e-02 | 1.11e-01 |
|  | 20 | 3643863 | 3644300 | PDFSTATSENSEMBLUCSC | 438 | 6 | 25972 | island | 0.598955 | 0.894680 | Not queried | 3.47 | 1.52e-02 | 5.36e-02 |
|  | 16 | 89989024 | 89989483 | PDFSTATSENSEMBLUCSC | 460 | 4 | 15434 | tss;island | 0.009691 | 0.064476 | Not queried | 3.47 | 2.00e-01 | 2.55e-01 |
|  | 2 | 160918900 | 160919532 | PDFSTATSENSEMBLUCSC | 633 | 12 | 24813 | island | 0.066208 | 0.422863 | PLA2R1 (margin: PLA2R1) | 3.46 | 2.01e-04 | 3.38e-03 |
|  | 18 | 29598296 | 29598437 | PDFSTATSENSEMBLUCSC | 142 | 4 | 19222 | tss;island | 0.011182 | 0.111207 | Not queried | 3.46 | 2.00e-01 | 2.55e-01 |
|  | 17 | 10741502 | 10741610 | PDFSTATSENSEMBLUCSC | 109 | 4 | 17136 | tss | 0.829367 | 0.984546 | Not queried | 3.46 | 2.00e-01 | 2.55e-01 |
|  | 2 | 208890341 | 208890496 | PDFSTATSENSEMBLUCSC | 156 | 4 | 23845 | tss | 0.003905 | 0.091671 | Not queried | 3.46 | 2.00e-01 | 2.55e-01 |
|  | 17 | 74137336 | 74137522 | PDFSTATSENSEMBLUCSC | 187 | 6 | 18774 | island | 0.068583 | 0.004016 | RNF157-AS1; FOXJ1; RNF157 (margin: RNF157-AS1; FOXJ1; RNF157) | 3.46 | 8.66e-03 | 3.36e-02 |
|  | 3 | 147140754 | 147140930 | PDFSTATSENSEMBLUCSC | 177 | 4 | 29575 | island | 0.638053 | 0.967459 | Not queried | 3.46 | 2.00e-01 | 2.55e-01 |
|  | 10 | 42970987 | 42971251 | PDFSTATSENSEMBLUCSC | 265 | 4 | 4564 | gene | 0.341187 | 0.856939 | Not queried | 3.46 | 2.86e-02 | 6.12e-02 |
|  | 18 | 7567426 | 7567799 | PDFSTATSENSEMBLUCSC | 374 | 5 | 19052 | gene;island | 0.440375 | 0.930347 | PTPRM (margin: PTPRM) | 3.46 | 7.94e-03 | 3.19e-02 |
|  | 18 | 158229 | 158567 | PDFSTATSENSEMBLUCSC | 339 | 7 | 19344 | island | 0.014440 | 0.010400 | Not queried | 3.46 | 4.56e-01 | 4.96e-01 |
|  | 6 | 116937346 | 116937499 | PDFSTATSENSEMBLUCSC | 154 | 8 | 35191 | tss | 0.016578 | 0.003930 | Not queried | 3.46 | 1.48e-02 | 5.36e-02 |
|  | 5 | 131346985 | 131347773 | PDFSTATSENSEMBLUCSC | 789 | 9 | 33083 | island | 0.611266 | 0.134113 | AC034228.2; ACSL6 (margin: AC034228.3; AC034228.2; ACSL6) | 3.46 | 5.64e-03 | 3.18e-02 |
|  | 17 | 7117125 | 7117310 | PDFSTATSENSEMBLUCSC | 186 | 4 | 16383 | gene;island | 0.542709 | 0.337547 | Not queried | 3.46 | 4.86e-01 | 5.11e-01 |
|  | 22 | 25003400 | 25003552 | PDFSTATSENSEMBLUCSC | 153 | 4 | 27025 | tss | 0.440196 | 0.872743 | Not queried | 3.46 | 2.86e-02 | 6.12e-02 |
|  | 2 | 180725622 | 180726035 | PDFSTATSENSEMBLUCSC | 414 | 7 | 22951 | gene;island | 0.427694 | 0.066341 | Not queried | 3.46 | 7.28e-02 | 1.23e-01 |
|  | 19 | 2250901 | 2251067 | PDFSTATSENSEMBLUCSC | 167 | 4 | 19682 | gene;island | 0.885646 | 0.984939 | Not queried | 3.46 | 2.86e-02 | 6.12e-02 |
|  | 5 | 148928885 | 148929784 | PDFSTATSENSEMBLUCSC | 900 | 8 | 31858 | gene;island | 0.002700 | 0.016856 | Not queried | 3.46 | 1.95e-01 | 2.55e-01 |
|  | 20 | 4764077 | 4764312 | PDFSTATSENSEMBLUCSC | 236 | 5 | 25349 | gene | 0.525446 | 0.966724 | Not queried | 3.46 | 5.56e-02 | 1.00e-01 |
|  | 2 | 169658881 | 169659182 | PDFSTATSENSEMBLUCSC | 302 | 6 | 22913 | gene | 0.660799 | 0.967709 | NOSTRIN (margin: NOSTRIN) | 3.46 | 8.66e-03 | 3.36e-02 |
|  | 14 | 64319089 | 64319306 | PDFSTATSENSEMBLUCSC | 218 | 4 | 12452 | tss;island | 0.943828 | 0.464367 | Not queried | 3.46 | 3.43e-01 | 3.84e-01 |
|  | 11 | 1506936 | 1507347 | PDFSTATSENSEMBLUCSC | 412 | 6 | 6423 | gene | 0.503428 | 0.919487 | Not queried | 3.46 | 9.31e-02 | 1.53e-01 |
|  | 3 | 42947481 | 42947771 | PDFSTATSENSEMBLUCSC | 291 | 4 | 27736 | gene | 0.863760 | 0.985398 | Not queried | 3.46 | 2.86e-02 | 6.12e-02 |
|  | 2 | 60780746 | 60780809 | PDFSTATSENSEMBLUCSC | 64 | 4 | 23370 | tss;island | 0.199970 | 0.049693 | Not queried | 3.46 | 2.86e-02 | 6.12e-02 |
|  | 10 | 26506353 | 26506933 | PDFSTATSENSEMBLUCSC | 581 | 5 | 4536 | gene;island | 0.557780 | 0.953508 | Not queried | 3.46 | 3.17e-02 | 6.50e-02 |
|  | 10 | 43572065 | 43572795 | PDFSTATSENSEMBLUCSC | 731 | 9 | 5687 | island | 0.287745 | 0.017525 | RET (margin: RET) | 3.46 | 7.77e-03 | 3.19e-02 |
|  | 2 | 223162666 | 223162800 | PDFSTATSENSEMBLUCSC | 135 | 4 | 23915 | tss | 0.782999 | 0.978390 | Not queried | 3.46 | 2.86e-02 | 6.12e-02 |
|  | 6 | 30687159 | 30687707 | PDFSTATSENSEMBLUCSC | 549 | 7 | 34786 | tss;island | 0.122028 | 0.151211 | Not queried | 3.46 | 3.83e-01 | 4.27e-01 |
|  | 2 | 503672 | 503776 | PDFSTATSENSEMBLUCSC | 105 | 4 | 24006 | island | 0.846735 | 0.973957 | Not queried | 3.45 | 2.86e-02 | 6.12e-02 |
|  | 11 | 117747822 | 117748236 | PDFSTATSENSEMBLUCSC | 415 | 9 | 7723 | tss | 0.697610 | 0.855121 | Not queried | 3.45 | 1.61e-01 | 2.25e-01 |
|  | 6 | 32188695 | 32189126 | PDFSTATSENSEMBLUCSC | 432 | 6 | 34099 | gene | 0.205905 | 0.781674 | NOTCH4 (margin: NOTCH4) | 3.45 | 2.16e-03 | 1.45e-02 |
|  | 8 | 140715540 | 140715802 | PDFSTATSENSEMBLUCSC | 263 | 5 | 39923 | tss;island | 0.752739 | 0.963524 | KCNK9 (margin: KCNK9) | 3.45 | 7.94e-03 | 3.19e-02 |
|  | 20 | 982623 | 983130 | PDFSTATSENSEMBLUCSC | 508 | 8 | 25931 | island | 0.722470 | 0.960996 | RSPO4 (margin: RSPO4) | 3.45 | 1.09e-03 | 1.10e-02 |
|  | 6 | 168078772 | 168079212 | PDFSTATSENSEMBLUCSC | 441 | 5 | 36644 | island | 0.677227 | 0.957558 | Not queried | 3.45 | 5.56e-02 | 1.00e-01 |
|  | 15 | 50716270 | 50716372 | PDFSTATSENSEMBLUCSC | 103 | 4 | 13669 | tss;island | 0.003558 | 0.031841 | Not queried | 3.45 | 5.71e-02 | 1.00e-01 |
|  | 2 | 241807708 | 241808113 | PDFSTATSENSEMBLUCSC | 406 | 6 | 23982 | tss | 0.127269 | 0.519387 | Not queried | 3.45 | 1.32e-01 | 1.89e-01 |
|  | 5 | 1860135 | 1860542 | PDFSTATSENSEMBLUCSC | 408 | 4 | 32642 | island | 0.573429 | 0.943112 | Not queried | 3.45 | 3.43e-01 | 3.84e-01 |
|  | 17 | 72322388 | 72322791 | PDFSTATSENSEMBLUCSC | 404 | 4 | 16751 | gene;island | 0.857839 | 0.985101 | Not queried | 3.45 | 2.86e-02 | 6.12e-02 |
|  | 20 | 57428282 | 57428473 | PDFSTATSENSEMBLUCSC | 192 | 6 | 25472 | gene;island | 0.697752 | 0.962450 | GNAS (margin: GNAS; GNAS-AS1; RP1-309F20.3) | 3.44 | 2.16e-03 | 1.45e-02 |
|  | 6 | 32299502 | 32299676 | PDFSTATSENSEMBLUCSC | 175 | 4 | 34102 | gene | 0.619637 | 0.919435 | Not queried | 3.44 | 2.86e-02 | 6.12e-02 |
|  | 3 | 136537607 | 136537863 | PDFSTATSENSEMBLUCSC | 257 | 9 | 29508 | island | 0.735570 | 0.962306 | SLC35G2 (margin: SLC35G2; RP11-731C17.1) | 3.44 | 4.11e-05 | 1.17e-03 |
|  | 7 | 45066738 | 45067057 | PDFSTATSENSEMBLUCSC | 320 | 5 | 36997 | gene;tss | 0.572416 | 0.917158 | CCM2 (margin: CCM2) | 3.44 | 7.94e-03 | 3.19e-02 |
|  | 12 | 122687758 | 122687992 | PDFSTATSENSEMBLUCSC | 235 | 4 | 9372 | gene | 0.322854 | 0.045017 | Not queried | 3.44 | 3.43e-01 | 3.84e-01 |
|  | 19 | 45927563 | 45927696 | PDFSTATSENSEMBLUCSC | 134 | 4 | 20871 | tss;island | 0.003252 | 0.033219 | Not queried | 3.44 | 5.71e-02 | 1.00e-01 |
|  | 10 | 134842688 | 134842984 | PDFSTATSENSEMBLUCSC | 297 | 4 | 6327 | island | 0.120480 | 0.623086 | Not queried | 3.44 | 5.71e-02 | 1.00e-01 |
|  | 8 | 11471395 | 11471881 | PDFSTATSENSEMBLUCSC | 487 | 5 | 40082 | island | 0.540813 | 0.927966 | (margin: ) | 3.44 | 7.94e-03 | 3.19e-02 |
|  | 1 | 159869927 | 159870326 | PDFSTATSENSEMBLUCSC | 400 | 7 | 2032 | tss;island | 0.126561 | 0.015741 | Not queried | 3.44 | 2.62e-02 | 6.12e-02 |
|  | 2 | 27346296 | 27346938 | PDFSTATSENSEMBLUCSC | 643 | 7 | 24169 | island | 0.144886 | 0.014815 | Not queried | 3.44 | 5.30e-02 | 1.00e-01 |
|  | 2 | 242878496 | 242878811 | PDFSTATSENSEMBLUCSC | 316 | 4 | 25317 | island | 0.767047 | 0.975910 | Not queried | 3.44 | 5.71e-02 | 1.00e-01 |
|  | 10 | 42863444 | 42863594 | PDFSTATSENSEMBLUCSC | 151 | 5 | 5680 | island | 0.364125 | 0.723971 | (margin: ) | 3.44 | 7.94e-03 | 3.19e-02 |
|  | 1 | 7111184 | 7111367 | PDFSTATSENSEMBLUCSC | 184 | 4 | 185 | gene | 0.494754 | 0.889768 | Not queried | 3.44 | 2.00e-01 | 2.55e-01 |
|  | 1 | 1177886 | 1178245 | PDFSTATSENSEMBLUCSC | 360 | 5 | 23 | gene | 0.745299 | 0.969138 | FAM132A (margin: B3GALT6; SDF4; FAM132A; RP5-902P8.12; UBE2J2) | 3.44 | 7.94e-03 | 3.19e-02 |
|  | 11 | 134254573 | 134254936 | PDFSTATSENSEMBLUCSC | 364 | 6 | 6999 | gene;island | 0.639104 | 0.927893 | Not queried | 3.44 | 2.60e-02 | 6.12e-02 |
|  | 1 | 3306999 | 3307215 | PDFSTATSENSEMBLUCSC | 217 | 4 | 122 | gene;island | 0.617697 | 0.907367 | Not queried | 3.44 | 2.00e-01 | 2.55e-01 |
|  | 11 | 64512829 | 64513289 | PDFSTATSENSEMBLUCSC | 461 | 6 | 7402 | tss;island | 0.515863 | 0.911892 | RASGRP2; PYGM (margin: RASGRP2; PYGM) | 3.44 | 2.16e-03 | 1.45e-02 |
|  | 2 | 223168453 | 223168941 | PDFSTATSENSEMBLUCSC | 489 | 6 | 23051 | gene;island | 0.641455 | 0.957067 | Not queried | 3.44 | 1.80e-01 | 2.45e-01 |
|  | 17 | 29159410 | 29159713 | PDFSTATSENSEMBLUCSC | 304 | 4 | 16509 | gene;island | 0.001757 | 0.003541 | Not queried | 3.44 | 6.86e-01 | 6.99e-01 |
|  | 3 | 134513902 | 134514173 | PDFSTATSENSEMBLUCSC | 272 | 8 | 28608 | tss | 0.277481 | 0.024369 | Not queried | 3.44 | 2.81e-02 | 6.12e-02 |
|  | 3 | 134513902 | 134514472 | PDFSTATSENSEMBLUCSC | 571 | 10 | 29501 | island | 0.277481 | 0.024369 | Not queried | 3.44 | 2.32e-02 | 6.12e-02 |
|  | 21 | 28218481 | 28218959 | PDFSTATSENSEMBLUCSC | 479 | 5 | 26500 | tss;island | 0.771161 | 0.265980 | ADAMTS1 (margin: ADAMTS1) | 3.43 | 7.94e-03 | 3.19e-02 |
|  | 13 | 52768659 | 52768717 | PDFSTATSENSEMBLUCSC | 59 | 5 | 11459 | tss | 0.000518 | 0.006269 | Not queried | 3.43 | 2.22e-01 | 2.76e-01 |
|  | 2 | 171570700 | 171571151 | PDFSTATSENSEMBLUCSC | 452 | 6 | 23708 | tss;island | 0.010815 | 0.155007 | Not queried | 3.43 | 6.49e-02 | 1.11e-01 |
|  | 22 | 41956810 | 41956987 | PDFSTATSENSEMBLUCSC | 178 | 4 | 27175 | tss | 0.372169 | 0.785954 | Not queried | 3.43 | 2.86e-02 | 6.12e-02 |
|  | 16 | 967929 | 968265 | PDFSTATSENSEMBLUCSC | 337 | 6 | 14554 | gene;island | 0.797807 | 0.969398 | LMF1 (margin: LA16c-366D3.1; LMF1) | 3.43 | 8.66e-03 | 3.36e-02 |
|  | 7 | 150216872 | 150217081 | PDFSTATSENSEMBLUCSC | 210 | 4 | 37275 | gene | 0.637017 | 0.943873 | Not queried | 3.43 | 1.14e-01 | 1.68e-01 |
|  | 4 | 8412369 | 8412795 | PDFSTATSENSEMBLUCSC | 427 | 4 | 29952 | gene | 0.501253 | 0.350119 | Not queried | 3.43 | 6.86e-01 | 6.99e-01 |
|  | 1 | 111218079 | 111218287 | PDFSTATSENSEMBLUCSC | 209 | 4 | 1819 | tss;island | 0.655751 | 0.965445 | Not queried | 3.43 | 2.86e-02 | 6.12e-02 |
|  | 18 | 30352975 | 30353283 | PDFSTATSENSEMBLUCSC | 309 | 7 | 19226 | tss | 0.078836 | 0.698864 | Not queried | 3.43 | 2.09e-01 | 2.65e-01 |
|  | 20 | 57089953 | 57090317 | PDFSTATSENSEMBLUCSC | 365 | 9 | 25850 | tss | 0.825570 | 0.920394 | Not queried | 3.43 | 1.42e-02 | 5.24e-02 |
|  | 3 | 139653328 | 139653573 | PDFSTATSENSEMBLUCSC | 246 | 4 | 28634 | tss;island | 0.803459 | 0.290229 | Not queried | 3.43 | 3.43e-01 | 3.84e-01 |
|  | 17 | 72968968 | 72969066 | PDFSTATSENSEMBLUCSC | 99 | 5 | 17638 | tss;island | 0.354260 | 0.020848 | Not queried | 3.43 | 3.17e-02 | 6.50e-02 |
|  | 10 | 468051 | 468412 | PDFSTATSENSEMBLUCSC | 362 | 5 | 4439 | gene;island | 0.885399 | 0.944934 | Not queried | 3.43 | 1.51e-01 | 2.11e-01 |
|  | 7 | 4347499 | 4348107 | PDFSTATSENSEMBLUCSC | 609 | 7 | 38187 | island | 0.447791 | 0.897156 | (margin: ) | 3.43 | 5.83e-04 | 6.47e-03 |
|  | 9 | 98784231 | 98784441 | PDFSTATSENSEMBLUCSC | 211 | 4 | 40824 | tss;island | 0.816194 | 0.978698 | Not queried | 3.43 | 5.71e-02 | 1.00e-01 |
|  | 4 | 6107021 | 6107339 | PDFSTATSENSEMBLUCSC | 319 | 5 | 29925 | gene;island | 0.700225 | 0.968949 | JAKMIP1 (margin: JAKMIP1) | 3.43 | 7.94e-03 | 3.19e-02 |
|  | 3 | 71180032 | 71180071 | PDFSTATSENSEMBLUCSC | 40 | 4 | 27858 | gene | 0.845965 | 0.984165 | Not queried | 3.43 | 2.86e-02 | 6.12e-02 |
|  | 1 | 202780290 | 202780680 | PDFSTATSENSEMBLUCSC | 391 | 4 | 4092 | island | 0.023568 | 0.392369 | Not queried | 3.43 | 1.14e-01 | 1.68e-01 |
|  | 17 | 74944775 | 74944970 | PDFSTATSENSEMBLUCSC | 196 | 4 | 16801 | gene;island | 0.370483 | 0.867444 | Not queried | 3.42 | 2.86e-02 | 6.12e-02 |
|  | 5 | 121414025 | 121414425 | PDFSTATSENSEMBLUCSC | 401 | 13 | 33044 | island | 0.393905 | 0.077376 | LOX (margin: SRFBP1; LOX) | 3.42 | 8.65e-06 | 4.82e-04 |
|  | 5 | 121414067 | 121414425 | PDFSTATSENSEMBLUCSC | 359 | 11 | 32243 | tss | 0.408767 | 0.077376 | LOX (margin: SRFBP1; LOX) | 3.42 | 5.39e-05 | 1.47e-03 |
|  | 1 | 68516463 | 68516713 | PDFSTATSENSEMBLUCSC | 251 | 6 | 1686 | tss | 0.501695 | 0.871696 | Not queried | 3.42 | 9.31e-02 | 1.53e-01 |
|  | 7 | 129425330 | 129425932 | PDFSTATSENSEMBLUCSC | 603 | 6 | 38913 | island | 0.613647 | 0.951669 | RP11-738B7.1 (margin: MIR96; MIR183; RP11-738B7.1) | 3.42 | 4.33e-03 | 2.52e-02 |
|  | 1 | 234350263 | 234350515 | PDFSTATSENSEMBLUCSC | 253 | 4 | 1084 | gene;island | 0.388122 | 0.863460 | Not queried | 3.42 | 2.86e-02 | 6.12e-02 |
|  | 3 | 147136904 | 147137503 | PDFSTATSENSEMBLUCSC | 600 | 7 | 29574 | island | 0.790984 | 0.972261 | Not queried | 3.42 | 1.75e-02 | 5.79e-02 |
|  | 4 | 2964664 | 2964972 | PDFSTATSENSEMBLUCSC | 309 | 4 | 29898 | gene | 0.001983 | 0.005273 | Not queried | 3.42 | 1.14e-01 | 1.68e-01 |
|  | 21 | 40555470 | 40555523 | PDFSTATSENSEMBLUCSC | 54 | 5 | 26548 | tss | 0.000725 | 0.011963 | Not queried | 3.42 | 1.51e-01 | 2.11e-01 |
|  | 3 | 30936239 | 30936531 | PDFSTATSENSEMBLUCSC | 293 | 8 | 28201 | tss | 0.376818 | 0.054014 | GADL1 (margin: GADL1) | 3.42 | 6.99e-03 | 3.19e-02 |
|  | 10 | 95848649 | 95848841 | PDFSTATSENSEMBLUCSC | 193 | 4 | 4685 | gene | 0.055840 | 0.153456 | Not queried | 3.42 | 3.43e-01 | 3.84e-01 |
|  | 11 | 2828214 | 2828778 | PDFSTATSENSEMBLUCSC | 565 | 7 | 6467 | gene;island | 0.184933 | 0.707890 | KCNQ1 (margin: KCNQ1) | 3.42 | 1.11e-02 | 4.14e-02 |
|  | 6 | 30881112 | 30882094 | PDFSTATSENSEMBLUCSC | 983 | 28 | 34793 | tss | 0.877260 | 0.068837 | GTF2H4; VARS2 (margin: RN7SL175P; GTF2H4; VARS2) | 3.42 | 6.32e-04 | 6.81e-03 |
|  | 5 | 135364328 | 135364580 | PDFSTATSENSEMBLUCSC | 253 | 8 | 32309 | tss;island | 0.009601 | 0.046903 | Not queried | 3.42 | 8.30e-02 | 1.40e-01 |
|  | 8 | 125740429 | 125740636 | PDFSTATSENSEMBLUCSC | 208 | 4 | 39423 | gene;island | 0.075826 | 0.010781 | Not queried | 3.41 | 1.14e-01 | 1.68e-01 |
|  | 11 | 69924046 | 69924390 | PDFSTATSENSEMBLUCSC | 345 | 10 | 7524 | tss;island | 0.155892 | 0.837857 | ANO1 (margin: RNU6-1175P; ANO1; ANO1-AS2) | 3.41 | 5.20e-03 | 2.96e-02 |
|  | 2 | 241497412 | 241497846 | PDFSTATSENSEMBLUCSC | 435 | 8 | 23981 | tss;island | 0.596397 | 0.137051 | Not queried | 3.41 | 8.30e-02 | 1.40e-01 |
|  | 17 | 49242714 | 49243027 | PDFSTATSENSEMBLUCSC | 314 | 6 | 16694 | gene;tss;island | 0.949591 | 0.652972 | Not queried | 3.41 | 4.11e-02 | 8.14e-02 |
|  | 19 | 5455176 | 5455386 | PDFSTATSENSEMBLUCSC | 211 | 4 | 20376 | tss | 0.767697 | 0.942019 | Not queried | 3.41 | 2.00e-01 | 2.55e-01 |
|  | 11 | 120110544 | 120110887 | PDFSTATSENSEMBLUCSC | 344 | 9 | 7761 | tss | 0.509804 | 0.924424 | POU2F3 (margin: OAF; POU2F3; RP11-778O17.4) | 3.41 | 4.94e-04 | 6.47e-03 |
|  | 4 | 69048001 | 69048430 | PDFSTATSENSEMBLUCSC | 430 | 5 | 31082 | island | 0.605076 | 0.958318 | Not queried | 3.41 | 9.52e-02 | 1.53e-01 |
|  | 4 | 5053085 | 5053504 | PDFSTATSENSEMBLUCSC | 420 | 5 | 30274 | tss | 0.782323 | 0.974334 | Not queried | 3.41 | 1.59e-02 | 5.36e-02 |
|  | 5 | 87980882 | 87981449 | PDFSTATSENSEMBLUCSC | 568 | 7 | 32168 | tss;island | 0.844292 | 0.982850 | CTC-467M3.1; LINC00461 (margin: CTC-467M3.1; CTC-467M3.2; LINC00461; CTC-467M3.3) | 3.41 | 6.99e-03 | 3.19e-02 |
|  | 2 | 130691738 | 130691956 | PDFSTATSENSEMBLUCSC | 219 | 5 | 24713 | island | 0.239974 | 0.769133 | Not queried | 3.41 | 5.56e-02 | 1.00e-01 |
|  | 19 | 30303495 | 30303674 | PDFSTATSENSEMBLUCSC | 180 | 4 | 19969 | gene;island | 0.304412 | 0.151008 | Not queried | 3.41 | 3.43e-01 | 3.84e-01 |
|  | 11 | 120110544 | 120111025 | PDFSTATSENSEMBLUCSC | 482 | 10 | 8893 | island | 0.508810 | 0.914843 | POU2F3 (margin: OAF; POU2F3; RP11-778O17.4) | 3.41 | 3.25e-04 | 4.91e-03 |
|  | 2 | 30144376 | 30144838 | PDFSTATSENSEMBLUCSC | 463 | 7 | 24199 | island | 0.062777 | 0.008446 | Not queried | 3.41 | 3.79e-02 | 7.66e-02 |
|  | 7 | 4848683 | 4848939 | PDFSTATSENSEMBLUCSC | 257 | 4 | 36857 | gene;island | 0.761309 | 0.975823 | Not queried | 3.41 | 2.86e-02 | 6.12e-02 |
|  | 22 | 20790837 | 20791300 | PDFSTATSENSEMBLUCSC | 464 | 5 | 26798 | gene;island | 0.763928 | 0.971606 | Not queried | 3.41 | 5.56e-02 | 1.00e-01 |
|  | 14 | 96152464 | 96152706 | PDFSTATSENSEMBLUCSC | 243 | 5 | 12589 | tss | 0.165923 | 0.677683 | Not queried | 3.41 | 9.52e-02 | 1.53e-01 |
|  | 17 | 47210027 | 47210368 | PDFSTATSENSEMBLUCSC | 342 | 7 | 16672 | gene;island | 0.038473 | 0.008607 | B4GALNT2 (margin: B4GALNT2) | 3.40 | 1.11e-02 | 4.14e-02 |
|  | 19 | 48837272 | 48837598 | PDFSTATSENSEMBLUCSC | 327 | 4 | 20122 | gene;island | 0.508446 | 0.079146 | Not queried | 3.40 | 1.14e-01 | 1.68e-01 |
|  | 2 | 152956034 | 152956135 | PDFSTATSENSEMBLUCSC | 102 | 4 | 23663 | tss;island | 0.726372 | 0.207334 | Not queried | 3.40 | 1.14e-01 | 1.68e-01 |
|  | 17 | 6023675 | 6024017 | PDFSTATSENSEMBLUCSC | 343 | 4 | 16371 | gene;island | 0.574121 | 0.909900 | Not queried | 3.40 | 5.71e-02 | 1.00e-01 |
|  | 19 | 19373566 | 19373755 | PDFSTATSENSEMBLUCSC | 190 | 5 | 21809 | island | 0.682450 | 0.957964 | HAPLN4; AC138430.4; TM6SF2 (margin: NCAN; HAPLN4; AC138430.4; TM6SF2) | 3.40 | 7.94e-03 | 3.19e-02 |
|  | 7 | 23221200 | 23221679 | PDFSTATSENSEMBLUCSC | 480 | 9 | 38307 | island | 0.013193 | 0.070310 | Not queried | 3.40 | 3.15e-02 | 6.50e-02 |
|  | 1 | 19600673 | 19601069 | PDFSTATSENSEMBLUCSC | 397 | 9 | 1301 | tss;island | 0.862707 | 0.985108 | AKR7L (margin: AKR7L; AKR7A3) | 3.40 | 2.88e-04 | 4.58e-03 |
|  | 10 | 82116203 | 82116596 | PDFSTATSENSEMBLUCSC | 394 | 10 | 4654 | gene;tss;island | 0.856716 | 0.980260 | DYDC2; DYDC1 (margin: DYDC2; DYDC1) | 3.40 | 1.08e-05 | 4.82e-04 |
|  | 1 | 24645802 | 24646392 | PDFSTATSENSEMBLUCSC | 591 | 13 | 2858 | island | 0.068427 | 0.006043 | GRHL3; RP11-10N16.3 (margin: GRHL3; RP11-10N16.3) | 3.40 | 1.25e-03 | 1.19e-02 |
|  | 14 | 101491377 | 101492406 | PDFSTATSENSEMBLUCSC | 1030 | 11 | 12621 | tss | 0.534747 | 0.894535 | MIR411; MIR299; MIR1197; MIR323A; MIR758; MIR329-1; MIR329-2; MIR380 (margin: MIR379; MIR411; MIR299; MIR1197; MIR323A; MIR758; MIR329-1; MIR329-2; MIR494; MIR1193; MIR543; MIR495; MIR380) | 3.40 | 8.33e-03 | 3.34e-02 |
|  | 19 | 9846267 | 9846701 | PDFSTATSENSEMBLUCSC | 435 | 4 | 21504 | island | 0.789559 | 0.983321 | Not queried | 3.40 | 5.71e-02 | 1.00e-01 |
|  | 3 | 62360162 | 62360464 | PDFSTATSENSEMBLUCSC | 303 | 4 | 28427 | tss;island | 0.194850 | 0.719647 | Not queried | 3.40 | 5.71e-02 | 1.00e-01 |
|  | 12 | 124773870 | 124774078 | PDFSTATSENSEMBLUCSC | 209 | 4 | 9387 | gene | 0.702822 | 0.233713 | Not queried | 3.40 | 1.14e-01 | 1.68e-01 |
|  | 1 | 182922263 | 182922705 | PDFSTATSENSEMBLUCSC | 443 | 6 | 4028 | island | 0.984218 | 0.778661 | Not queried | 3.40 | 1.80e-01 | 2.45e-01 |
|  | 1 | 1266468 | 1266700 | PDFSTATSENSEMBLUCSC | 233 | 5 | 1147 | tss | 0.257530 | 0.782159 | TAS1R3 (margin: GLTPD1; TAS1R3; CPSF3L; DVL1) | 3.40 | 7.94e-03 | 3.19e-02 |
|  | 16 | 59788951 | 59789219 | PDFSTATSENSEMBLUCSC | 269 | 4 | 15984 | island | 0.194850 | 0.019356 | Not queried | 3.40 | 5.71e-02 | 1.00e-01 |
|  | 6 | 84742998 | 84743364 | PDFSTATSENSEMBLUCSC | 367 | 10 | 35099 | tss;island | 0.282185 | 0.858128 | MRAP2 (margin: RP11-51G5.1; MRAP2) | 3.39 | 2.06e-04 | 3.42e-03 |
|  | 6 | 33160847 | 33161589 | PDFSTATSENSEMBLUCSC | 743 | 22 | 34890 | tss;island | 0.637827 | 0.974676 | COL11A2; RXRB (margin: RNY4P10; SLC39A7; HSD17B8; COL11A2; RXRB) | 3.39 | 1.06e-02 | 4.01e-02 |
|  | 7 | 116139180 | 116139425 | PDFSTATSENSEMBLUCSC | 246 | 7 | 37849 | tss | 0.110220 | 0.363104 | CAV2; AC002066.1 (margin: CAV2; AC002066.1) | 3.39 | 5.83e-04 | 6.47e-03 |
|  | 5 | 41509849 | 41510116 | PDFSTATSENSEMBLUCSC | 268 | 5 | 31599 | gene;island | 0.763359 | 0.963407 | Not queried | 3.39 | 5.56e-02 | 1.00e-01 |
|  | 1 | 15127680 | 15127907 | PDFSTATSENSEMBLUCSC | 228 | 4 | 238 | gene;island | 0.713419 | 0.951432 | Not queried | 3.39 | 2.86e-02 | 6.12e-02 |
|  | 7 | 156811252 | 156811655 | PDFSTATSENSEMBLUCSC | 404 | 6 | 39093 | island | 0.568770 | 0.946034 | Not queried | 3.39 | 9.31e-02 | 1.53e-01 |
|  | 17 | 38334161 | 38334186 | PDFSTATSENSEMBLUCSC | 26 | 5 | 17326 | tss;island | 0.167223 | 0.658804 | RAPGEFL1 (margin: CASC3; RAPGEFL1) | 3.39 | 7.94e-03 | 3.19e-02 |
|  | 2 | 68546899 | 68547141 | PDFSTATSENSEMBLUCSC | 243 | 4 | 22713 | gene | 0.529991 | 0.938593 | Not queried | 3.39 | 3.43e-01 | 3.84e-01 |
|  | 13 | 98605770 | 98605892 | PDFSTATSENSEMBLUCSC | 123 | 4 | 11507 | tss | 0.771623 | 0.963997 | Not queried | 3.39 | 2.86e-02 | 6.12e-02 |
|  | 12 | 106977389 | 106977781 | PDFSTATSENSEMBLUCSC | 393 | 5 | 9279 | gene;island | 0.585268 | 0.957084 | Not queried | 3.39 | 5.56e-02 | 1.00e-01 |
|  | 15 | 62457456 | 62457681 | PDFSTATSENSEMBLUCSC | 226 | 4 | 14199 | island | 0.752702 | 0.959511 | Not queried | 3.39 | 2.86e-02 | 6.12e-02 |
|  | 10 | 134884177 | 134884645 | PDFSTATSENSEMBLUCSC | 469 | 7 | 6330 | island | 0.450698 | 0.743621 | Not queried | 3.39 | 1.75e-02 | 5.79e-02 |
|  | 17 | 77777649 | 77777947 | PDFSTATSENSEMBLUCSC | 299 | 4 | 18844 | island | 0.579217 | 0.929315 | Not queried | 3.38 | 2.86e-02 | 6.12e-02 |
|  | 6 | 29364418 | 29365287 | PDFSTATSENSEMBLUCSC | 870 | 11 | 33654 | gene | 0.185863 | 0.558223 | Not queried | 3.38 | 7.59e-02 | 1.28e-01 |
|  | 5 | 27038707 | 27038836 | PDFSTATSENSEMBLUCSC | 130 | 4 | 32003 | tss | 0.830841 | 0.314837 | Not queried | 3.38 | 3.43e-01 | 3.84e-01 |
|  | 4 | 3464653 | 3465064 | PDFSTATSENSEMBLUCSC | 412 | 10 | 30839 | island | 0.371364 | 0.028259 | DOK7 (margin: DOK7) | 3.38 | 5.20e-03 | 2.96e-02 |
|  | 16 | 49499006 | 49499385 | PDFSTATSENSEMBLUCSC | 380 | 4 | 15908 | island | 0.589920 | 0.919890 | Not queried | 3.38 | 2.86e-02 | 6.12e-02 |
|  | 10 | 97054967 | 97055439 | PDFSTATSENSEMBLUCSC | 473 | 4 | 5964 | island | 0.095249 | 0.525703 | Not queried | 3.38 | 2.86e-02 | 6.12e-02 |
|  | 15 | 45406534 | 45406939 | PDFSTATSENSEMBLUCSC | 406 | 6 | 13326 | gene | 0.413827 | 0.835745 | DUOXA2; DUOX2 (margin: DUOXA2; DUOX2; DUOXA1) | 3.38 | 2.16e-03 | 1.45e-02 |
|  | 10 | 7618453 | 7618614 | PDFSTATSENSEMBLUCSC | 162 | 4 | 4482 | gene;island | 0.640095 | 0.955324 | Not queried | 3.38 | 2.86e-02 | 6.12e-02 |
|  | 7 | 1315546 | 1315866 | PDFSTATSENSEMBLUCSC | 321 | 4 | 38096 | island | 0.553514 | 0.969828 | Not queried | 3.38 | 2.00e-01 | 2.55e-01 |
|  | 11 | 64993076 | 64993335 | PDFSTATSENSEMBLUCSC | 260 | 4 | 6704 | gene;island | 0.065990 | 0.413794 | Not queried | 3.38 | 2.86e-02 | 6.12e-02 |
|  | 1 | 2344979 | 2345475 | PDFSTATSENSEMBLUCSC | 497 | 11 | 1173 | tss;island | 0.151810 | 0.028824 | PEX10 (margin: RER1; PLCH2; PEX10) | 3.38 | 5.39e-05 | 1.47e-03 |
|  | 17 | 29719023 | 29719431 | PDFSTATSENSEMBLUCSC | 409 | 5 | 16510 | gene;island | 0.636787 | 0.967381 | RAB11FIP4 (margin: NF1; RAB11FIP4) | 3.38 | 7.94e-03 | 3.19e-02 |
|  | 8 | 124194847 | 124195192 | PDFSTATSENSEMBLUCSC | 346 | 5 | 39417 | gene | 0.012775 | 0.150218 | Not queried | 3.38 | 1.59e-02 | 5.36e-02 |
|  | 19 | 12228348 | 12228618 | PDFSTATSENSEMBLUCSC | 271 | 5 | 21593 | island | 0.661914 | 0.929699 | Not queried | 3.38 | 5.56e-02 | 1.00e-01 |
|  | 11 | 63382047 | 63382095 | PDFSTATSENSEMBLUCSC | 49 | 5 | 7376 | tss;island | 0.004598 | 0.049447 | Not queried | 3.38 | 3.17e-02 | 6.50e-02 |
|  | 6 | 50817157 | 50817450 | PDFSTATSENSEMBLUCSC | 294 | 5 | 36199 | island | 0.792926 | 0.945412 | Not queried | 3.38 | 1.51e-01 | 2.11e-01 |
|  | 20 | 61809557 | 61809841 | PDFSTATSENSEMBLUCSC | 285 | 7 | 25889 | tss | 0.964870 | 0.554190 | Not queried | 3.38 | 7.28e-02 | 1.23e-01 |
|  | 11 | 93063516 | 93064234 | PDFSTATSENSEMBLUCSC | 719 | 9 | 8727 | island | 0.571087 | 0.942023 | CCDC67 (margin: CCDC67) | 3.38 | 1.06e-02 | 4.01e-02 |
|  | 11 | 93063886 | 93064234 | PDFSTATSENSEMBLUCSC | 349 | 5 | 6859 | gene | 0.873176 | 0.981708 | Not queried | 3.38 | 9.52e-02 | 1.53e-01 |
|  | 13 | 112668130 | 112668501 | PDFSTATSENSEMBLUCSC | 372 | 6 | 11906 | island | 0.477188 | 0.919012 | Not queried | 3.38 | 1.32e-01 | 1.89e-01 |
|  | 11 | 132107419 | 132107699 | PDFSTATSENSEMBLUCSC | 281 | 6 | 6979 | gene | 0.536526 | 0.138242 | Not queried | 3.37 | 9.31e-02 | 1.53e-01 |
|  | 13 | 102104976 | 102105129 | PDFSTATSENSEMBLUCSC | 154 | 5 | 11213 | gene | 0.843664 | 0.464102 | ITGBL1 (margin: ITGBL1) | 3.37 | 7.94e-03 | 3.19e-02 |
|  | 14 | 24540174 | 24540452 | PDFSTATSENSEMBLUCSC | 279 | 4 | 12310 | tss | 0.378051 | 0.718479 | Not queried | 3.37 | 1.14e-01 | 1.68e-01 |
|  | 2 | 172949281 | 172949501 | PDFSTATSENSEMBLUCSC | 221 | 4 | 23716 | tss;island | 0.394063 | 0.053196 | Not queried | 3.37 | 2.86e-02 | 6.12e-02 |
|  | 1 | 203595048 | 203595283 | PDFSTATSENSEMBLUCSC | 236 | 4 | 2194 | tss;island | 0.160504 | 0.672621 | Not queried | 3.37 | 5.71e-02 | 1.00e-01 |
|  | 1 | 206786000 | 206786222 | PDFSTATSENSEMBLUCSC | 223 | 6 | 2215 | tss;island | 0.588928 | 0.089003 | Not queried | 3.37 | 6.49e-02 | 1.11e-01 |
|  | 16 | 2260770 | 2261237 | PDFSTATSENSEMBLUCSC | 468 | 10 | 15593 | island | 0.655808 | 0.951020 | MLST8; RP11-304L19.8; BRICD5; PGP (margin: MLST8; RP11-304L19.8; AC009065.3; BRICD5; PGP) | 3.37 | 4.87e-04 | 6.47e-03 |
|  | 2 | 219232590 | 219232875 | PDFSTATSENSEMBLUCSC | 286 | 4 | 25084 | island | 0.098898 | 0.397013 | Not queried | 3.37 | 3.43e-01 | 3.84e-01 |
|  | 22 | 32600722 | 32601274 | PDFSTATSENSEMBLUCSC | 553 | 4 | 27089 | tss | 0.093098 | 0.008934 | Not queried | 3.37 | 2.00e-01 | 2.55e-01 |
|  | 15 | 64443650 | 64443896 | PDFSTATSENSEMBLUCSC | 247 | 8 | 13727 | tss | 0.288665 | 0.018963 | Not queried | 3.37 | 3.79e-02 | 7.66e-02 |
|  | 19 | 51219975 | 51220537 | PDFSTATSENSEMBLUCSC | 563 | 10 | 22320 | island | 0.405063 | 0.883728 | SHANK1 (margin: CLEC11A; SHANK1) | 3.37 | 6.84e-03 | 3.19e-02 |
|  | 1 | 151694108 | 151694320 | PDFSTATSENSEMBLUCSC | 213 | 4 | 752 | gene | 0.763522 | 0.976853 | Not queried | 3.37 | 2.86e-02 | 6.12e-02 |
|  | 4 | 186064512 | 186064648 | PDFSTATSENSEMBLUCSC | 137 | 4 | 30208 | gene;island | 0.142772 | 0.018603 | Not queried | 3.37 | 2.86e-02 | 6.12e-02 |
|  | 19 | 17392161 | 17392438 | PDFSTATSENSEMBLUCSC | 278 | 7 | 20584 | tss;island | 0.655525 | 0.931609 | Not queried | 3.37 | 2.62e-02 | 6.12e-02 |
|  | 17 | 27346732 | 27347260 | PDFSTATSENSEMBLUCSC | 529 | 6 | 18164 | island | 0.677408 | 0.969269 | PIPOX; RP11-321A17.3 (margin: PIPOX; AC024619.2; RP11-321A17.5; RP11-321A17.3) | 3.37 | 8.66e-03 | 3.36e-02 |
|  | 3 | 27762636 | 27763266 | PDFSTATSENSEMBLUCSC | 631 | 5 | 27707 | gene;island | 0.284936 | 0.011150 | Not queried | 3.37 | 1.51e-01 | 2.11e-01 |
|  | 2 | 66664535 | 66664960 | PDFSTATSENSEMBLUCSC | 426 | 5 | 22710 | gene;island | 0.743902 | 0.958510 | Not queried | 3.37 | 1.59e-02 | 5.36e-02 |
|  | 8 | 38326209 | 38326730 | PDFSTATSENSEMBLUCSC | 522 | 8 | 40234 | island | 0.707603 | 0.498186 | Not queried | 3.36 | 5.05e-01 | 5.32e-01 |
|  | 6 | 88757302 | 88757392 | PDFSTATSENSEMBLUCSC | 91 | 5 | 35114 | tss;island | 0.759433 | 0.985862 | Not queried | 3.36 | 3.17e-02 | 6.50e-02 |
|  | 11 | 2176877 | 2177018 | PDFSTATSENSEMBLUCSC | 142 | 4 | 6451 | gene;island | 0.784248 | 0.985765 | Not queried | 3.36 | 2.86e-02 | 6.12e-02 |
|  | 19 | 53635967 | 53636398 | PDFSTATSENSEMBLUCSC | 432 | 8 | 22368 | island | 0.672489 | 0.965156 | ZNF415; ZNF347; CTD-2620I22.7 (margin: ZNF415; ZNF347; CTD-2620I22.7) | 3.36 | 1.04e-02 | 3.96e-02 |
|  | 1 | 229542868 | 229543254 | PDFSTATSENSEMBLUCSC | 387 | 4 | 4308 | island | 0.610977 | 0.966335 | Not queried | 3.36 | 3.43e-01 | 3.84e-01 |
|  | 16 | 31548640 | 31548908 | PDFSTATSENSEMBLUCSC | 269 | 4 | 15881 | island | 0.667089 | 0.963553 | Not queried | 3.36 | 5.71e-02 | 1.00e-01 |
|  | 2 | 1842860 | 1843184 | PDFSTATSENSEMBLUCSC | 325 | 6 | 22537 | gene;island | 0.913382 | 0.563821 | Not queried | 3.36 | 3.94e-01 | 4.36e-01 |
|  | 16 | 56224504 | 56224901 | PDFSTATSENSEMBLUCSC | 398 | 5 | 14755 | gene | 0.379676 | 0.033229 | Not queried | 3.36 | 3.17e-02 | 6.50e-02 |
|  | 19 | 49631042 | 49631297 | PDFSTATSENSEMBLUCSC | 256 | 4 | 20143 | gene;island | 0.466037 | 0.157896 | Not queried | 3.36 | 6.86e-01 | 6.99e-01 |
|  | 1 | 220863263 | 220863796 | PDFSTATSENSEMBLUCSC | 534 | 9 | 4217 | island | 0.466555 | 0.063608 | Not queried | 3.36 | 4.00e-02 | 8.03e-02 |
|  | 10 | 25465171 | 25465503 | PDFSTATSENSEMBLUCSC | 333 | 8 | 4534 | gene;island | 0.377546 | 0.942957 | Not queried | 3.36 | 8.30e-02 | 1.40e-01 |
|  | 13 | 52769662 | 52769951 | PDFSTATSENSEMBLUCSC | 290 | 4 | 11460 | tss;island | 0.849271 | 0.369181 | Not queried | 3.36 | 5.71e-02 | 1.00e-01 |
|  | 14 | 105635706 | 105635769 | PDFSTATSENSEMBLUCSC | 64 | 4 | 12667 | tss | 0.816906 | 0.347375 | Not queried | 3.36 | 2.86e-02 | 6.12e-02 |
|  | 5 | 122424399 | 122424706 | PDFSTATSENSEMBLUCSC | 308 | 4 | 32248 | tss;island | 0.530515 | 0.837979 | Not queried | 3.35 | 3.43e-01 | 3.84e-01 |
|  | 7 | 155255122 | 155255369 | PDFSTATSENSEMBLUCSC | 248 | 4 | 37309 | gene;island | 0.601591 | 0.957668 | Not queried | 3.35 | 2.00e-01 | 2.55e-01 |
|  | 1 | 231471991 | 231472445 | PDFSTATSENSEMBLUCSC | 455 | 5 | 1077 | gene;island | 0.080853 | 0.757278 | Not queried | 3.35 | 5.56e-02 | 1.00e-01 |
|  | 19 | 11784246 | 11784774 | PDFSTATSENSEMBLUCSC | 529 | 9 | 20479 | tss | 0.667673 | 0.957604 | Not queried | 3.35 | 1.88e-02 | 6.12e-02 |
|  | 10 | 134838014 | 134838656 | PDFSTATSENSEMBLUCSC | 643 | 7 | 6326 | island | 0.254155 | 0.575802 | Not queried | 3.35 | 7.28e-02 | 1.23e-01 |
|  | 7 | 158319029 | 158319204 | PDFSTATSENSEMBLUCSC | 176 | 4 | 37377 | gene;island | 0.583095 | 0.903709 | Not queried | 3.35 | 1.14e-01 | 1.68e-01 |
|  | 19 | 1315732 | 1316091 | PDFSTATSENSEMBLUCSC | 360 | 4 | 21215 | island | 0.476921 | 0.051480 | Not queried | 3.35 | 2.00e-01 | 2.55e-01 |
|  | 3 | 58163412 | 58163614 | PDFSTATSENSEMBLUCSC | 203 | 4 | 29233 | island | 0.155545 | 0.026239 | Not queried | 3.35 | 2.00e-01 | 2.55e-01 |
|  | 17 | 53315783 | 53316308 | PDFSTATSENSEMBLUCSC | 526 | 5 | 18574 | island | 0.037484 | 0.001024 | Not queried | 3.35 | 9.52e-02 | 1.53e-01 |
|  | 1 | 167599527 | 167599830 | PDFSTATSENSEMBLUCSC | 304 | 4 | 874 | gene | 0.829317 | 0.983812 | Not queried | 3.35 | 2.86e-02 | 6.12e-02 |
|  | 9 | 100615084 | 100615357 | PDFSTATSENSEMBLUCSC | 274 | 4 | 40825 | tss;island | 0.602240 | 0.926607 | Not queried | 3.35 | 2.86e-02 | 6.12e-02 |
|  | 1 | 14027378 | 14027581 | PDFSTATSENSEMBLUCSC | 204 | 4 | 236 | gene;island | 0.860657 | 0.978219 | Not queried | 3.35 | 1.14e-01 | 1.68e-01 |
|  | 7 | 25989524 | 25989763 | PDFSTATSENSEMBLUCSC | 240 | 4 | 38327 | island | 0.841952 | 0.984447 | Not queried | 3.35 | 3.43e-01 | 3.84e-01 |
|  | 5 | 169930841 | 169930938 | PDFSTATSENSEMBLUCSC | 98 | 6 | 32484 | tss | 0.517026 | 0.947366 | KCNIP1 (margin: KCNIP1) | 3.35 | 2.16e-03 | 1.45e-02 |
|  | 20 | 44258197 | 44258437 | PDFSTATSENSEMBLUCSC | 241 | 5 | 25431 | gene | 0.248111 | 0.050562 | Not queried | 3.35 | 9.52e-02 | 1.53e-01 |
|  | 6 | 99278991 | 99279108 | PDFSTATSENSEMBLUCSC | 118 | 4 | 36342 | island | 0.696022 | 0.952871 | Not queried | 3.35 | 2.86e-02 | 6.12e-02 |
|  | 22 | 29834388 | 29834697 | PDFSTATSENSEMBLUCSC | 310 | 5 | 26832 | gene | 0.300380 | 0.927869 | Not queried | 3.34 | 1.59e-02 | 5.36e-02 |
|  | 1 | 92951720 | 92953037 | PDFSTATSENSEMBLUCSC | 1318 | 20 | 1752 | tss | 0.544566 | 0.974289 | Not queried | 3.34 | 1.34e-01 | 1.93e-01 |
|  | 18 | 48086162 | 48086416 | PDFSTATSENSEMBLUCSC | 255 | 5 | 19276 | tss | 0.335346 | 0.029344 | Not queried | 3.34 | 3.17e-02 | 6.50e-02 |
|  | 22 | 37099037 | 37099259 | PDFSTATSENSEMBLUCSC | 223 | 4 | 27111 | tss;island | 0.255483 | 0.050763 | Not queried | 3.34 | 2.00e-01 | 2.55e-01 |
|  | 6 | 2903699 | 2904005 | PDFSTATSENSEMBLUCSC | 307 | 5 | 34593 | tss | 0.948512 | 0.451245 | Not queried | 3.34 | 9.52e-02 | 1.53e-01 |
|  | 19 | 4759746 | 4760016 | PDFSTATSENSEMBLUCSC | 271 | 4 | 21387 | island | 0.093154 | 0.018007 | Not queried | 3.34 | 2.86e-02 | 6.12e-02 |
|  | 11 | 2151676 | 2151725 | PDFSTATSENSEMBLUCSC | 50 | 4 | 6444 | gene;island | 0.646377 | 0.975231 | Not queried | 3.34 | 5.71e-02 | 1.00e-01 |
|  | 15 | 74658547 | 74658653 | PDFSTATSENSEMBLUCSC | 107 | 4 | 13422 | gene;island | 0.737050 | 0.978678 | Not queried | 3.34 | 2.00e-01 | 2.55e-01 |
|  | 5 | 122429966 | 122430821 | PDFSTATSENSEMBLUCSC | 856 | 10 | 31730 | gene;island | 0.730700 | 0.969105 | PRDM6 (margin: PRDM6; AC106786.1) | 3.34 | 8.93e-03 | 3.45e-02 |
|  | 3 | 184870685 | 184871070 | PDFSTATSENSEMBLUCSC | 386 | 8 | 29736 | island | 0.314863 | 0.075747 | Not queried | 3.34 | 1.95e-01 | 2.55e-01 |
|  | 10 | 63213104 | 63213510 | PDFSTATSENSEMBLUCSC | 407 | 7 | 5768 | island | 0.431328 | 0.901738 | Not queried | 3.34 | 2.62e-02 | 6.12e-02 |
|  | 10 | 63213339 | 63213510 | PDFSTATSENSEMBLUCSC | 172 | 5 | 5097 | tss | 0.431328 | 0.936487 | Not queried | 3.34 | 5.56e-02 | 1.00e-01 |
|  | 5 | 42756786 | 42756876 | PDFSTATSENSEMBLUCSC | 91 | 4 | 32041 | tss | 0.850568 | 0.979345 | Not queried | 3.34 | 1.14e-01 | 1.68e-01 |
|  | 22 | 22901569 | 22902237 | PDFSTATSENSEMBLUCSC | 669 | 11 | 27004 | tss;island | 0.364434 | 0.090659 | Not queried | 3.34 | 5.57e-02 | 1.00e-01 |
|  | 10 | 102987993 | 102988529 | PDFSTATSENSEMBLUCSC | 537 | 5 | 4726 | gene;tss;island | 0.792354 | 0.968236 | Not queried | 3.34 | 3.17e-02 | 6.50e-02 |
|  | 8 | 9911482 | 9911765 | PDFSTATSENSEMBLUCSC | 284 | 9 | 39546 | tss;island | 0.061125 | 0.009251 | Not queried | 3.34 | 9.39e-02 | 1.53e-01 |
|  | 1 | 3028813 | 3029070 | PDFSTATSENSEMBLUCSC | 258 | 6 | 100 | gene;island | 0.436033 | 0.870070 | Not queried | 3.34 | 2.60e-02 | 6.12e-02 |
|  | 1 | 2938646 | 2939117 | PDFSTATSENSEMBLUCSC | 472 | 5 | 94 | gene;island | 0.182318 | 0.739689 | Not queried | 3.34 | 5.56e-02 | 1.00e-01 |
|  | 16 | 1968576 | 1968709 | PDFSTATSENSEMBLUCSC | 134 | 4 | 15009 | tss;island | 0.768687 | 0.970949 | Not queried | 3.34 | 2.86e-02 | 6.12e-02 |
|  | 19 | 1449857 | 1450023 | PDFSTATSENSEMBLUCSC | 167 | 5 | 20306 | tss;island | 0.295988 | 0.033282 | APC2 (margin: RPS15; AC027307.3; APC2; CTB-25B13.12; C19orf25) | 3.34 | 7.94e-03 | 3.19e-02 |
|  | 11 | 31837104 | 31837607 | PDFSTATSENSEMBLUCSC | 504 | 7 | 6563 | gene;island | 0.686982 | 0.895013 | Not queried | 3.34 | 7.28e-02 | 1.23e-01 |
|  | 7 | 1783946 | 1784169 | PDFSTATSENSEMBLUCSC | 224 | 4 | 36766 | gene;island | 0.343624 | 0.895129 | Not queried | 3.34 | 5.71e-02 | 1.00e-01 |
|  | 17 | 75136249 | 75136439 | PDFSTATSENSEMBLUCSC | 191 | 4 | 17693 | tss;island | 0.015459 | 0.215802 | Not queried | 3.33 | 1.14e-01 | 1.68e-01 |
|  | 14 | 24837836 | 24838126 | PDFSTATSENSEMBLUCSC | 291 | 4 | 12050 | gene;island | 0.801601 | 0.970386 | Not queried | 3.33 | 5.71e-02 | 1.00e-01 |
|  | 20 | 3644097 | 3644300 | PDFSTATSENSEMBLUCSC | 204 | 4 | 25566 | tss | 0.598955 | 0.892815 | Not queried | 3.33 | 1.14e-01 | 1.68e-01 |
|  | 2 | 147344914 | 147345337 | PDFSTATSENSEMBLUCSC | 424 | 5 | 22892 | gene;island | 0.947365 | 0.647097 | Not queried | 3.33 | 9.52e-02 | 1.53e-01 |
|  | 15 | 89954560 | 89955171 | PDFSTATSENSEMBLUCSC | 612 | 6 | 14434 | island | 0.707173 | 0.493057 | Not queried | 3.33 | 3.94e-01 | 4.36e-01 |
|  | 1 | 205818956 | 205819609 | PDFSTATSENSEMBLUCSC | 654 | 8 | 4138 | island | 0.815614 | 0.984642 | PM20D1 (margin: PM20D1) | 3.33 | 4.66e-03 | 2.68e-02 |
|  | 20 | 30433233 | 30433673 | PDFSTATSENSEMBLUCSC | 441 | 5 | 26082 | island | 0.221080 | 0.732857 | Not queried | 3.33 | 1.59e-02 | 5.36e-02 |
|  | 5 | 148206173 | 148206412 | PDFSTATSENSEMBLUCSC | 240 | 4 | 31854 | gene | 0.017892 | 0.065782 | Not queried | 3.33 | 1.14e-01 | 1.68e-01 |
|  | 14 | 105173643 | 105173980 | PDFSTATSENSEMBLUCSC | 338 | 5 | 12248 | gene | 0.933862 | 0.533498 | Not queried | 3.33 | 1.51e-01 | 2.11e-01 |
|  | 1 | 66999588 | 66999813 | PDFSTATSENSEMBLUCSC | 226 | 4 | 1679 | tss | 0.607992 | 0.956067 | Not queried | 3.33 | 1.14e-01 | 1.68e-01 |
|  | 6 | 32822857 | 32823292 | PDFSTATSENSEMBLUCSC | 436 | 5 | 34157 | gene;island | 0.181257 | 0.937350 | Not queried | 3.33 | 9.52e-02 | 1.53e-01 |
|  | 16 | 1199836 | 1200097 | PDFSTATSENSEMBLUCSC | 262 | 4 | 15519 | island | 0.529215 | 0.954470 | Not queried | 3.33 | 5.71e-02 | 1.00e-01 |
|  | 6 | 84562778 | 84563098 | PDFSTATSENSEMBLUCSC | 321 | 9 | 36292 | island | 0.657288 | 0.971256 | RIPPLY2 (margin: RIPPLY2; CYB5R4) | 3.33 | 1.85e-03 | 1.45e-02 |
|  | 5 | 158531132 | 158531647 | PDFSTATSENSEMBLUCSC | 516 | 5 | 33351 | island | 0.630990 | 0.960365 | Not queried | 3.33 | 9.52e-02 | 1.53e-01 |
|  | 19 | 57988958 | 57989282 | PDFSTATSENSEMBLUCSC | 325 | 8 | 21116 | tss | 0.018452 | 0.187024 | Not queried | 3.33 | 2.07e-02 | 6.12e-02 |
|  | 11 | 1855286 | 1855665 | PDFSTATSENSEMBLUCSC | 380 | 7 | 7047 | tss | 0.016628 | 0.140630 | SYT8 (margin: SYT8; TNNI2) | 3.33 | 1.11e-02 | 4.14e-02 |
|  | 16 | 1133074 | 1133378 | PDFSTATSENSEMBLUCSC | 305 | 5 | 15515 | island | 0.070366 | 0.338469 | Not queried | 3.33 | 5.56e-02 | 1.00e-01 |
|  | 7 | 54610022 | 54610320 | PDFSTATSENSEMBLUCSC | 299 | 4 | 37017 | gene | 0.849217 | 0.363971 | Not queried | 3.32 | 2.86e-02 | 6.12e-02 |
|  | 7 | 54609587 | 54610320 | PDFSTATSENSEMBLUCSC | 734 | 12 | 38498 | island | 0.849217 | 0.338723 | Not queried | 3.32 | 2.05e-02 | 6.12e-02 |
|  | 1 | 63783375 | 63783576 | PDFSTATSENSEMBLUCSC | 202 | 4 | 3340 | island | 0.190756 | 0.023650 | Not queried | 3.32 | 1.14e-01 | 1.68e-01 |
|  | 6 | 167104601 | 167104926 | PDFSTATSENSEMBLUCSC | 326 | 5 | 34553 | gene | 0.073570 | 0.710913 | Not queried | 3.32 | 3.17e-02 | 6.50e-02 |
|  | 2 | 72374840 | 72375141 | PDFSTATSENSEMBLUCSC | 302 | 4 | 24403 | island | 0.124737 | 0.507183 | Not queried | 3.32 | 2.00e-01 | 2.55e-01 |
|  | 1 | 2560884 | 2561122 | PDFSTATSENSEMBLUCSC | 239 | 4 | 93 | gene;island | 0.285846 | 0.809740 | Not queried | 3.32 | 1.14e-01 | 1.68e-01 |
|  | 2 | 466712 | 467135 | PDFSTATSENSEMBLUCSC | 424 | 8 | 24002 | island | 0.676559 | 0.967818 | (margin: ) | 3.32 | 6.99e-03 | 3.19e-02 |
|  | 12 | 6933322 | 6933689 | PDFSTATSENSEMBLUCSC | 368 | 4 | 9034 | gene;island | 0.462403 | 0.911658 | Not queried | 3.32 | 1.14e-01 | 1.68e-01 |
|  | 17 | 38657875 | 38657949 | PDFSTATSENSEMBLUCSC | 75 | 4 | 17335 | tss | 0.019892 | 0.224246 | Not queried | 3.32 | 2.86e-02 | 6.12e-02 |
|  | 19 | 18902081 | 18902221 | PDFSTATSENSEMBLUCSC | 141 | 6 | 21794 | island | 0.821567 | 0.979304 | Not queried | 3.32 | 6.49e-02 | 1.11e-01 |
|  | 19 | 42509801 | 42510093 | PDFSTATSENSEMBLUCSC | 293 | 4 | 20063 | gene;island | 0.347697 | 0.815497 | Not queried | 3.32 | 2.86e-02 | 6.12e-02 |
|  | 4 | 42399699 | 42399858 | PDFSTATSENSEMBLUCSC | 160 | 7 | 31014 | island | 0.833178 | 0.982343 | SHISA3 (margin: SHISA3; RP11-63A11.1; ATP8A1) | 3.32 | 5.83e-04 | 6.47e-03 |
|  | 5 | 140501321 | 140501812 | PDFSTATSENSEMBLUCSC | 492 | 10 | 33211 | island | 0.891666 | 0.513373 | Not queried | 3.32 | 4.33e-02 | 8.55e-02 |
|  | 11 | 115530150 | 115530590 | PDFSTATSENSEMBLUCSC | 441 | 6 | 8829 | island | 0.268739 | 0.027701 | AP000797.3 (margin: AP000797.3) | 3.32 | 8.66e-03 | 3.36e-02 |
|  | 6 | 31508106 | 31508318 | PDFSTATSENSEMBLUCSC | 213 | 6 | 33894 | gene;island | 0.154720 | 0.550541 | DDX39B-AS1; DDX39B; ATP6V1G2-DDX39B; SNORD84 (margin: RPL15P4; MCCD1; DDX39B-AS1; NFKBIL1; DDX39B; ATP6V1G2-DDX39B; SNORD117; SNORD84; ATP6V1G2) | 3.32 | 4.33e-03 | 2.52e-02 |
|  | 7 | 121651766 | 121652024 | PDFSTATSENSEMBLUCSC | 259 | 4 | 37204 | gene | 0.141473 | 0.017481 | Not queried | 3.32 | 2.00e-01 | 2.55e-01 |
|  | 15 | 89312795 | 89313222 | PDFSTATSENSEMBLUCSC | 428 | 7 | 14421 | island | 0.575064 | 0.897596 | Not queried | 3.32 | 7.28e-02 | 1.23e-01 |
|  | 22 | 25003400 | 25003854 | PDFSTATSENSEMBLUCSC | 455 | 6 | 26816 | gene | 0.537416 | 0.903787 | Not queried | 3.32 | 1.52e-02 | 5.36e-02 |
|  | 4 | 153601329 | 153601466 | PDFSTATSENSEMBLUCSC | 138 | 5 | 30637 | tss | 0.036778 | 0.239883 | Not queried | 3.32 | 1.59e-02 | 5.36e-02 |
|  | 7 | 45197251 | 45197727 | PDFSTATSENSEMBLUCSC | 477 | 7 | 38474 | island | 0.251699 | 0.741191 | Not queried | 3.32 | 7.28e-02 | 1.23e-01 |
|  | 5 | 140180921 | 140181074 | PDFSTATSENSEMBLUCSC | 154 | 4 | 31794 | gene;island | 0.842656 | 0.566640 | Not queried | 3.31 | 1.14e-01 | 1.68e-01 |
|  | 11 | 66084599 | 66084841 | PDFSTATSENSEMBLUCSC | 243 | 4 | 7464 | tss | 0.661489 | 0.936394 | Not queried | 3.31 | 5.71e-02 | 1.00e-01 |
|  | 1 | 34630796 | 34631096 | PDFSTATSENSEMBLUCSC | 301 | 7 | 409 | gene;island | 0.247388 | 0.903478 | Not queried | 3.31 | 1.75e-02 | 5.79e-02 |
|  | 16 | 30198370 | 30198517 | PDFSTATSENSEMBLUCSC | 148 | 4 | 14718 | gene;island | 0.897973 | 0.484382 | Not queried | 3.31 | 1.14e-01 | 1.68e-01 |
|  | 11 | 93466719 | 93467095 | PDFSTATSENSEMBLUCSC | 377 | 4 | 7622 | tss | 0.778349 | 0.948314 | Not queried | 3.31 | 3.43e-01 | 3.84e-01 |
|  | 16 | 1429719 | 1430367 | PDFSTATSENSEMBLUCSC | 649 | 8 | 14993 | tss;island | 0.727343 | 0.029276 | Not queried | 3.31 | 1.05e-01 | 1.66e-01 |
|  | 2 | 121494701 | 121495233 | PDFSTATSENSEMBLUCSC | 533 | 5 | 24670 | island | 0.436235 | 0.051621 | Not queried | 3.31 | 3.17e-02 | 6.50e-02 |
|  | 12 | 102036140 | 102036563 | PDFSTATSENSEMBLUCSC | 424 | 5 | 9269 | gene;island | 0.728146 | 0.960151 | Not queried | 3.31 | 3.17e-02 | 6.50e-02 |
|  | 3 | 184104362 | 184104688 | PDFSTATSENSEMBLUCSC | 327 | 5 | 28063 | gene;island | 0.744842 | 0.975710 | Not queried | 3.31 | 3.17e-02 | 6.50e-02 |
|  | 5 | 145758586 | 145758881 | PDFSTATSENSEMBLUCSC | 296 | 5 | 33284 | island | 0.712804 | 0.960084 | Not queried | 3.31 | 1.51e-01 | 2.11e-01 |
|  | 13 | 100626995 | 100627366 | PDFSTATSENSEMBLUCSC | 372 | 4 | 11846 | island | 0.626939 | 0.962122 | Not queried | 3.31 | 5.71e-02 | 1.00e-01 |
|  | 3 | 138153127 | 138153439 | PDFSTATSENSEMBLUCSC | 313 | 9 | 29521 | island | 0.223319 | 0.700838 | ESYT3 (margin: ESYT3) | 3.31 | 2.88e-04 | 4.58e-03 |
|  | 19 | 51330265 | 51330469 | PDFSTATSENSEMBLUCSC | 205 | 4 | 20184 | gene;island | 0.845424 | 0.979493 | Not queried | 3.31 | 5.71e-02 | 1.00e-01 |
|  | 12 | 4829115 | 4829690 | PDFSTATSENSEMBLUCSC | 576 | 7 | 9501 | tss | 0.279782 | 0.727949 | Not queried | 3.31 | 2.62e-02 | 6.12e-02 |
|  | 20 | 13200685 | 13200992 | PDFSTATSENSEMBLUCSC | 308 | 12 | 26007 | island | 0.847832 | 0.985514 | ISM1 (margin: ISM1) | 3.31 | 7.40e-07 | 7.21e-05 |
|  | 1 | 47690795 | 47691301 | PDFSTATSENSEMBLUCSC | 507 | 5 | 515 | gene;island | 0.592413 | 0.959844 | Not queried | 3.31 | 2.22e-01 | 2.76e-01 |
|  | 2 | 222436947 | 222437130 | PDFSTATSENSEMBLUCSC | 184 | 8 | 25140 | island | 0.052708 | 0.007025 | EPHA4 (margin: EPHA4) | 3.31 | 1.09e-03 | 1.10e-02 |
|  | 7 | 155250210 | 155250299 | PDFSTATSENSEMBLUCSC | 90 | 4 | 38011 | tss;island | 0.168574 | 0.017906 | Not queried | 3.31 | 2.86e-02 | 6.12e-02 |
|  | 17 | 12569213 | 12569487 | PDFSTATSENSEMBLUCSC | 275 | 4 | 16447 | gene | 0.219432 | 0.675562 | Not queried | 3.31 | 5.71e-02 | 1.00e-01 |
|  | 18 | 6414958 | 6415252 | PDFSTATSENSEMBLUCSC | 295 | 9 | 19172 | tss;island | 0.527883 | 0.956500 | L3MBTL4 (margin: L3MBTL4) | 3.30 | 1.65e-04 | 2.80e-03 |
|  | 3 | 52488037 | 52488229 | PDFSTATSENSEMBLUCSC | 193 | 4 | 28381 | tss;island | 0.494931 | 0.891191 | Not queried | 3.30 | 2.86e-02 | 6.12e-02 |
|  | 1 | 214153294 | 214153645 | PDFSTATSENSEMBLUCSC | 352 | 5 | 4194 | island | 0.752320 | 0.980446 | Not queried | 3.30 | 9.52e-02 | 1.53e-01 |
|  | 6 | 164392967 | 164393860 | PDFSTATSENSEMBLUCSC | 894 | 8 | 36619 | island | 0.764633 | 0.977178 | Not queried | 3.30 | 3.79e-02 | 7.66e-02 |
|  | 17 | 29717946 | 29718306 | PDFSTATSENSEMBLUCSC | 361 | 5 | 17247 | tss;island | 0.562794 | 0.929332 | Not queried | 3.30 | 1.51e-01 | 2.11e-01 |
|  | 6 | 2903533 | 2904005 | PDFSTATSENSEMBLUCSC | 473 | 7 | 35379 | island | 0.889985 | 0.450722 | SERPINB9 (margin: SERPINB9; RP1-90J20.10) | 3.30 | 6.99e-03 | 3.19e-02 |
|  | 12 | 21927773 | 21927990 | PDFSTATSENSEMBLUCSC | 218 | 7 | 9601 | tss | 0.028987 | 0.008726 | Not queried | 3.30 | 1.28e-01 | 1.87e-01 |
|  | 10 | 3373441 | 3373770 | PDFSTATSENSEMBLUCSC | 330 | 5 | 5510 | island | 0.979402 | 0.854574 | Not queried | 3.30 | 1.59e-02 | 5.36e-02 |
|  | 8 | 42623686 | 42623946 | PDFSTATSENSEMBLUCSC | 261 | 6 | 39681 | tss | 0.064296 | 0.361564 | Not queried | 3.30 | 2.60e-02 | 6.12e-02 |
|  | 11 | 102826469 | 102826680 | PDFSTATSENSEMBLUCSC | 212 | 4 | 7650 | tss | 0.374984 | 0.839208 | Not queried | 3.30 | 2.86e-02 | 6.12e-02 |
|  | 4 | 111551642 | 111552040 | PDFSTATSENSEMBLUCSC | 399 | 4 | 30097 | gene;island | 0.760613 | 0.974237 | Not queried | 3.30 | 1.14e-01 | 1.68e-01 |
|  | 18 | 48085671 | 48085844 | PDFSTATSENSEMBLUCSC | 174 | 4 | 19275 | tss;island | 0.693500 | 0.940227 | Not queried | 3.30 | 2.86e-02 | 6.12e-02 |
|  | 15 | 90792609 | 90793056 | PDFSTATSENSEMBLUCSC | 448 | 7 | 14450 | island | 0.455505 | 0.022243 | TTLL13; RP11-697E2.6 (margin: GDPGP1; RP11-697E2.4; TTLL13; RP11-697E2.6) | 3.30 | 4.08e-03 | 2.47e-02 |
|  | 8 | 72755784 | 72756341 | PDFSTATSENSEMBLUCSC | 558 | 5 | 39335 | gene;island | 0.424857 | 0.957011 | Not queried | 3.30 | 3.17e-02 | 6.50e-02 |
|  | 8 | 70946891 | 70947218 | PDFSTATSENSEMBLUCSC | 328 | 4 | 40373 | island | 0.838238 | 0.974736 | Not queried | 3.29 | 2.00e-01 | 2.55e-01 |
|  | 4 | 3643404 | 3643959 | PDFSTATSENSEMBLUCSC | 556 | 5 | 30849 | island | 0.597779 | 0.954173 | Not queried | 3.29 | 2.22e-01 | 2.76e-01 |
|  | 2 | 242927418 | 242927773 | PDFSTATSENSEMBLUCSC | 356 | 4 | 25318 | island | 0.363643 | 0.800855 | Not queried | 3.29 | 2.86e-02 | 6.12e-02 |
|  | 7 | 150147988 | 150148073 | PDFSTATSENSEMBLUCSC | 86 | 4 | 37274 | gene | 0.331909 | 0.814460 | Not queried | 3.29 | 2.00e-01 | 2.55e-01 |
|  | 10 | 43818243 | 43818911 | PDFSTATSENSEMBLUCSC | 669 | 8 | 5693 | island | 0.462587 | 0.946942 | RP11-168L22.2 (margin: RP11-168L22.2) | 3.29 | 1.04e-02 | 3.96e-02 |
|  | 14 | 101512592 | 101512826 | PDFSTATSENSEMBLUCSC | 235 | 5 | 12627 | tss | 0.225847 | 0.931018 | Not queried | 3.29 | 1.51e-01 | 2.11e-01 |
|  | 19 | 38747079 | 38747378 | PDFSTATSENSEMBLUCSC | 300 | 10 | 21968 | island | 0.715285 | 0.980198 | Not queried | 3.29 | 2.80e-01 | 3.40e-01 |
|  | 1 | 197882003 | 197882469 | PDFSTATSENSEMBLUCSC | 467 | 4 | 930 | gene;island | 0.920395 | 0.990901 | Not queried | 3.29 | 2.86e-02 | 6.12e-02 |
|  | 5 | 177764003 | 177764408 | PDFSTATSENSEMBLUCSC | 406 | 4 | 31927 | gene;island | 0.480168 | 0.909869 | Not queried | 3.29 | 2.86e-02 | 6.12e-02 |
|  | 15 | 29213626 | 29213748 | PDFSTATSENSEMBLUCSC | 123 | 4 | 13545 | tss | 0.471098 | 0.918980 | Not queried | 3.29 | 1.14e-01 | 1.68e-01 |
|  | 5 | 179588440 | 179588838 | PDFSTATSENSEMBLUCSC | 399 | 4 | 31940 | gene;island | 0.714134 | 0.960906 | Not queried | 3.29 | 2.00e-01 | 2.55e-01 |
|  | 1 | 245848637 | 245849102 | PDFSTATSENSEMBLUCSC | 466 | 4 | 1120 | gene;island | 0.633101 | 0.941917 | Not queried | 3.29 | 2.86e-02 | 6.12e-02 |
|  | 3 | 140951194 | 140951447 | PDFSTATSENSEMBLUCSC | 254 | 4 | 27972 | gene;island | 0.105869 | 0.026642 | Not queried | 3.29 | 2.00e-01 | 2.55e-01 |
|  | 7 | 100612725 | 100612891 | PDFSTATSENSEMBLUCSC | 167 | 4 | 37796 | tss | 0.192793 | 0.590858 | Not queried | 3.29 | 2.86e-02 | 6.12e-02 |
|  | 15 | 28344273 | 28344730 | PDFSTATSENSEMBLUCSC | 458 | 13 | 13969 | island | 0.304239 | 0.940446 | OCA2 (margin: OCA2; HERC2) | 3.29 | 8.60e-03 | 3.36e-02 |
|  | 5 | 2564514 | 2564751 | PDFSTATSENSEMBLUCSC | 238 | 5 | 32653 | island | 0.804395 | 0.974510 | (margin: ) | 3.29 | 7.94e-03 | 3.19e-02 |
|  | 19 | 50931222 | 50931622 | PDFSTATSENSEMBLUCSC | 401 | 6 | 20176 | gene;island | 0.831546 | 0.983564 | Not queried | 3.29 | 2.60e-02 | 6.12e-02 |
|  | 1 | 203155938 | 203156374 | PDFSTATSENSEMBLUCSC | 437 | 4 | 2190 | tss | 0.620358 | 0.854428 | Not queried | 3.29 | 1.14e-01 | 1.68e-01 |
|  | 15 | 65648103 | 65648490 | PDFSTATSENSEMBLUCSC | 388 | 4 | 13379 | gene;island | 0.721308 | 0.969483 | Not queried | 3.29 | 2.00e-01 | 2.55e-01 |
|  | 1 | 1101302 | 1101462 | PDFSTATSENSEMBLUCSC | 161 | 4 | 1138 | tss;island | 0.614293 | 0.965582 | Not queried | 3.29 | 5.71e-02 | 1.00e-01 |
|  | 19 | 46999224 | 46999444 | PDFSTATSENSEMBLUCSC | 221 | 7 | 20893 | tss | 0.651436 | 0.958508 | AC011484.1; PPP5D1; PNMAL2 (margin: AC011484.1; PPP5D1; PNMAL2) | 3.28 | 2.33e-03 | 1.52e-02 |
|  | 8 | 22735111 | 22735478 | PDFSTATSENSEMBLUCSC | 368 | 5 | 39237 | gene | 0.621846 | 0.892841 | Not queried | 3.28 | 1.51e-01 | 2.11e-01 |
|  | 4 | 13549355 | 13549839 | PDFSTATSENSEMBLUCSC | 485 | 8 | 30934 | island | 0.467722 | 0.802319 | Not queried | 3.28 | 1.61e-01 | 2.24e-01 |
|  | 19 | 57352269 | 57352807 | PDFSTATSENSEMBLUCSC | 539 | 12 | 20243 | gene | 0.908557 | 0.462634 | Not queried | 3.28 | 5.19e-02 | 1.00e-01 |
|  | 17 | 78387621 | 78387818 | PDFSTATSENSEMBLUCSC | 198 | 4 | 16866 | gene;tss;island | 0.652594 | 0.950440 | Not queried | 3.28 | 2.00e-01 | 2.55e-01 |
|  | 11 | 93063516 | 93063692 | PDFSTATSENSEMBLUCSC | 177 | 4 | 7619 | tss | 0.364333 | 0.839783 | Not queried | 3.28 | 2.86e-02 | 6.12e-02 |
|  | 2 | 224904626 | 224904837 | PDFSTATSENSEMBLUCSC | 212 | 5 | 23924 | tss;island | 0.642087 | 0.200747 | Not queried | 3.28 | 5.56e-02 | 1.00e-01 |
|  | 7 | 150754040 | 150754283 | PDFSTATSENSEMBLUCSC | 244 | 4 | 37284 | gene;island | 0.754539 | 0.249218 | Not queried | 3.28 | 1.14e-01 | 1.68e-01 |
|  | 12 | 15475490 | 15475767 | PDFSTATSENSEMBLUCSC | 278 | 4 | 9069 | gene | 0.202319 | 0.732647 | Not queried | 3.28 | 1.14e-01 | 1.68e-01 |
|  | 12 | 57634410 | 57634855 | PDFSTATSENSEMBLUCSC | 446 | 9 | 10556 | island | 0.039965 | 0.013813 | Not queried | 3.28 | 4.00e-02 | 8.03e-02 |
|  | 14 | 96180319 | 96181044 | PDFSTATSENSEMBLUCSC | 726 | 11 | 13113 | island | 0.587336 | 0.971111 | RP11-164H13.1; TCL1A (margin: RP11-164H13.1; TCL1A) | 3.28 | 5.67e-06 | 3.54e-04 |
|  | 22 | 44708761 | 44708908 | PDFSTATSENSEMBLUCSC | 148 | 5 | 27212 | tss | 0.316470 | 0.839732 | Not queried | 3.28 | 1.59e-02 | 5.36e-02 |
|  | 7 | 150884848 | 150885103 | PDFSTATSENSEMBLUCSC | 256 | 6 | 37994 | tss | 0.287824 | 0.807405 | Not queried | 3.28 | 1.52e-02 | 5.36e-02 |
|  | 4 | 154073371 | 154074259 | PDFSTATSENSEMBLUCSC | 889 | 14 | 30639 | tss | 0.447257 | 0.023493 | TRIM2 (margin: TRIM2) | 3.28 | 2.96e-03 | 1.88e-02 |
|  | 13 | 113642457 | 113642784 | PDFSTATSENSEMBLUCSC | 328 | 6 | 11265 | gene | 0.747506 | 0.970027 | MCF2L (margin: MCF2L) | 3.27 | 2.16e-03 | 1.45e-02 |
|  | 7 | 157320088 | 157320662 | PDFSTATSENSEMBLUCSC | 575 | 7 | 39106 | island | 0.459114 | 0.857766 | Not queried | 3.27 | 5.30e-02 | 1.00e-01 |
|  | 8 | 11203882 | 11204132 | PDFSTATSENSEMBLUCSC | 251 | 4 | 39204 | gene;island | 0.704395 | 0.972583 | Not queried | 3.27 | 1.14e-01 | 1.68e-01 |
|  | 4 | 69048156 | 69048430 | PDFSTATSENSEMBLUCSC | 275 | 4 | 30015 | gene | 0.637965 | 0.956948 | Not queried | 3.27 | 3.43e-01 | 3.84e-01 |
|  | 6 | 42927940 | 42928546 | PDFSTATSENSEMBLUCSC | 607 | 25 | 36145 | island | 0.122307 | 0.014601 | GNMT (margin: RP3-475N16.1; GNMT; RPL24P4; PEX6) | 3.27 | 5.98e-08 | 1.06e-05 |
|  | 8 | 72459908 | 72460135 | PDFSTATSENSEMBLUCSC | 228 | 4 | 40379 | island | 0.703002 | 0.199584 | Not queried | 3.27 | 2.86e-02 | 6.12e-02 |
|  | 19 | 2251588 | 2252069 | PDFSTATSENSEMBLUCSC | 482 | 5 | 19683 | gene;island | 0.804004 | 0.981088 | Not queried | 3.27 | 3.17e-02 | 6.50e-02 |
|  | 1 | 86174607 | 86174929 | PDFSTATSENSEMBLUCSC | 323 | 4 | 1724 | tss;island | 0.549408 | 0.978805 | Not queried | 3.27 | 3.43e-01 | 3.84e-01 |
|  | 8 | 12523087 | 12523225 | PDFSTATSENSEMBLUCSC | 139 | 4 | 40095 | island | 0.047501 | 0.236882 | Not queried | 3.27 | 1.14e-01 | 1.68e-01 |
|  | 6 | 31324916 | 31325455 | PDFSTATSENSEMBLUCSC | 540 | 16 | 35768 | island | 0.016340 | 0.119231 | XXbac-BPG248L24.12; HLA-B (margin: XXbac-BPG248L24.12; HLA-B; DHFRP2) | 3.27 | 9.55e-03 | 3.69e-02 |
|  | 2 | 120281354 | 120282327 | PDFSTATSENSEMBLUCSC | 974 | 11 | 24657 | island | 0.865636 | 0.982762 | SCTR (margin: SCTR) | 3.27 | 1.28e-02 | 4.76e-02 |
|  | 1 | 1935289 | 1935561 | PDFSTATSENSEMBLUCSC | 273 | 4 | 1165 | tss | 0.680713 | 0.970849 | Not queried | 3.26 | 2.86e-02 | 6.12e-02 |
|  | 17 | 8028735 | 8028948 | PDFSTATSENSEMBLUCSC | 214 | 4 | 17988 | island | 0.270496 | 0.024150 | Not queried | 3.26 | 1.14e-01 | 1.68e-01 |
|  | 2 | 232395269 | 232395463 | PDFSTATSENSEMBLUCSC | 195 | 6 | 23942 | tss;island | 0.243647 | 0.033899 | Not queried | 3.26 | 6.49e-02 | 1.11e-01 |
|  | 12 | 2613703 | 2613948 | PDFSTATSENSEMBLUCSC | 246 | 5 | 9003 | gene | 0.387265 | 0.858469 | Not queried | 3.26 | 1.59e-02 | 5.36e-02 |
|  | 2 | 131046122 | 131046551 | PDFSTATSENSEMBLUCSC | 430 | 4 | 24718 | island | 0.011189 | 0.233397 | Not queried | 3.26 | 1.14e-01 | 1.68e-01 |
|  | 4 | 16085603 | 16085814 | PDFSTATSENSEMBLUCSC | 212 | 6 | 30313 | tss;island | 0.916009 | 0.974622 | Not queried | 3.26 | 1.52e-02 | 5.36e-02 |
|  | 4 | 154073371 | 154074387 | PDFSTATSENSEMBLUCSC | 1017 | 15 | 31370 | island | 0.422430 | 0.025247 | TRIM2 (margin: TRIM2) | 3.26 | 2.70e-03 | 1.76e-02 |
|  | 4 | 57522689 | 57523055 | PDFSTATSENSEMBLUCSC | 367 | 7 | 30411 | tss | 0.772290 | 0.964258 | Not queried | 3.26 | 1.28e-01 | 1.87e-01 |
|  | 1 | 3191481 | 3191883 | PDFSTATSENSEMBLUCSC | 403 | 6 | 116 | gene;island | 0.617175 | 0.959315 | PRDM16 (margin: PRDM16) | 3.26 | 8.66e-03 | 3.36e-02 |
|  | 6 | 27839751 | 27840257 | PDFSTATSENSEMBLUCSC | 507 | 10 | 35572 | island | 0.024775 | 0.000989 | Not queried | 3.26 | 4.33e-02 | 8.55e-02 |
|  | 11 | 27741916 | 27742454 | PDFSTATSENSEMBLUCSC | 539 | 9 | 6547 | gene;tss;island | 0.773527 | 0.263954 | Not queried | 3.26 | 3.87e-01 | 4.30e-01 |
|  | 15 | 93631754 | 93631991 | PDFSTATSENSEMBLUCSC | 238 | 4 | 13914 | tss | 0.386472 | 0.052684 | Not queried | 3.26 | 5.71e-02 | 1.00e-01 |
|  | 12 | 52701963 | 52702318 | PDFSTATSENSEMBLUCSC | 356 | 4 | 9137 | gene;island | 0.793508 | 0.977922 | Not queried | 3.26 | 2.86e-02 | 6.12e-02 |
|  | 1 | 11538721 | 11538865 | PDFSTATSENSEMBLUCSC | 145 | 4 | 1244 | tss;island | 0.033000 | 0.013825 | Not queried | 3.26 | 3.43e-01 | 3.84e-01 |
|  | 14 | 95234658 | 95235127 | PDFSTATSENSEMBLUCSC | 470 | 7 | 12196 | gene;island | 0.838785 | 0.980732 | Not queried | 3.26 | 2.62e-02 | 6.12e-02 |
|  | 12 | 133065724 | 133066069 | PDFSTATSENSEMBLUCSC | 346 | 16 | 10130 | tss;island | 0.565887 | 0.712790 | Not queried | 3.25 | 2.11e-02 | 6.12e-02 |
|  | 11 | 17717574 | 17717818 | PDFSTATSENSEMBLUCSC | 245 | 4 | 8102 | island | 0.707920 | 0.942065 | Not queried | 3.25 | 2.00e-01 | 2.55e-01 |
|  | 2 | 214012 | 214353 | PDFSTATSENSEMBLUCSC | 342 | 4 | 23997 | island | 0.874739 | 0.985986 | Not queried | 3.25 | 2.86e-02 | 6.12e-02 |
|  | 6 | 13925420 | 13925567 | PDFSTATSENSEMBLUCSC | 148 | 4 | 33579 | gene;island | 0.497094 | 0.108533 | Not queried | 3.25 | 2.86e-02 | 6.12e-02 |
|  | 4 | 56915651 | 56916051 | PDFSTATSENSEMBLUCSC | 401 | 4 | 31064 | island | 0.259148 | 0.025243 | Not queried | 3.25 | 5.71e-02 | 1.00e-01 |
|  | 6 | 30138178 | 30138339 | PDFSTATSENSEMBLUCSC | 162 | 4 | 33738 | gene;island | 0.723059 | 0.669624 | Not queried | 3.25 | 6.86e-01 | 6.99e-01 |
|  | 19 | 40005557 | 40005836 | PDFSTATSENSEMBLUCSC | 280 | 10 | 22001 | island | 0.532278 | 0.853407 | SELV (margin: DLL3; SELV) | 3.25 | 3.25e-04 | 4.91e-03 |
|  | 5 | 54528100 | 54528369 | PDFSTATSENSEMBLUCSC | 270 | 4 | 31615 | gene;island | 0.013400 | 0.249962 | Not queried | 3.25 | 1.14e-01 | 1.68e-01 |
|  | 12 | 99139301 | 99139867 | PDFSTATSENSEMBLUCSC | 567 | 6 | 9261 | gene;island | 0.522054 | 0.959041 | Not queried | 3.25 | 6.49e-02 | 1.11e-01 |
|  | 3 | 42814705 | 42814948 | PDFSTATSENSEMBLUCSC | 244 | 8 | 29020 | island | 0.870840 | 0.985517 | RP11-70C1.1; CCDC13; HIGD1A (margin: RP11-70C1.1; CCDC13; HIGD1A) | 3.25 | 4.66e-03 | 2.68e-02 |
|  | 16 | 31382930 | 31383136 | PDFSTATSENSEMBLUCSC | 207 | 4 | 14736 | gene;island | 0.448812 | 0.741893 | Not queried | 3.25 | 4.86e-01 | 5.11e-01 |
|  | 5 | 140514711 | 140515207 | PDFSTATSENSEMBLUCSC | 497 | 6 | 33212 | island | 0.630734 | 0.956007 | PCDHB5 (margin: PCDHB4; PCDHB5) | 3.25 | 8.66e-03 | 3.36e-02 |
|  | 9 | 136203187 | 136203387 | PDFSTATSENSEMBLUCSC | 201 | 4 | 40850 | tss;island | 0.070202 | 0.743821 | Not queried | 3.25 | 3.43e-01 | 3.84e-01 |
|  | 6 | 167275778 | 167275999 | PDFSTATSENSEMBLUCSC | 222 | 7 | 35338 | tss | 0.751792 | 0.184061 | RPS6KA2; RP11-514O12.4 (margin: RPS6KA2; RP11-514O12.4) | 3.25 | 5.83e-04 | 6.47e-03 |
|  | 11 | 64058878 | 64059057 | PDFSTATSENSEMBLUCSC | 180 | 4 | 6677 | gene | 0.958866 | 0.732679 | Not queried | 3.25 | 2.00e-01 | 2.55e-01 |
|  | 3 | 119041204 | 119041529 | PDFSTATSENSEMBLUCSC | 326 | 4 | 27907 | gene;island | 0.710362 | 0.977810 | Not queried | 3.25 | 5.71e-02 | 1.00e-01 |
|  | 8 | 65284438 | 65284519 | PDFSTATSENSEMBLUCSC | 82 | 4 | 40340 | island | 0.682680 | 0.233509 | Not queried | 3.25 | 1.14e-01 | 1.68e-01 |
|  | 4 | 812387 | 812684 | PDFSTATSENSEMBLUCSC | 298 | 4 | 29839 | gene;island | 0.620886 | 0.959167 | Not queried | 3.25 | 2.00e-01 | 2.55e-01 |
|  | 15 | 91478847 | 91479184 | PDFSTATSENSEMBLUCSC | 338 | 4 | 13480 | gene;island | 0.025610 | 0.169209 | Not queried | 3.24 | 1.14e-01 | 1.68e-01 |
|  | 2 | 132348567 | 132348901 | PDFSTATSENSEMBLUCSC | 335 | 4 | 24735 | island | 0.745272 | 0.980557 | Not queried | 3.24 | 5.71e-02 | 1.00e-01 |
|  | 21 | 34442160 | 34442674 | PDFSTATSENSEMBLUCSC | 515 | 10 | 26643 | island | 0.625716 | 0.976222 | OLIG1; AP000282.2 (margin: LINC00945; OLIG1; AP000282.2) | 3.24 | 2.88e-03 | 1.86e-02 |
|  | 15 | 100882213 | 100882647 | PDFSTATSENSEMBLUCSC | 435 | 8 | 13926 | tss | 0.159388 | 0.017360 | ADAMTS17 (margin: ADAMTS17; SPATA41) | 3.24 | 6.99e-03 | 3.19e-02 |
|  | 21 | 46056847 | 46057082 | PDFSTATSENSEMBLUCSC | 236 | 4 | 26458 | gene;tss | 0.286525 | 0.821620 | Not queried | 3.24 | 5.71e-02 | 1.00e-01 |
|  | 20 | 8000418 | 8000706 | PDFSTATSENSEMBLUCSC | 289 | 7 | 25591 | tss;island | 0.085344 | 0.011705 | Not queried | 3.24 | 2.62e-02 | 6.12e-02 |
|  | 12 | 132312702 | 132312875 | PDFSTATSENSEMBLUCSC | 174 | 7 | 10123 | tss;island | 0.036784 | 0.003237 | Not queried | 3.24 | 9.73e-02 | 1.55e-01 |
|  | 14 | 77737661 | 77737800 | PDFSTATSENSEMBLUCSC | 140 | 4 | 12529 | tss | 0.680113 | 0.955952 | Not queried | 3.24 | 2.86e-02 | 6.12e-02 |
|  | 11 | 3253581 | 3254504 | PDFSTATSENSEMBLUCSC | 924 | 15 | 7996 | island | 0.483828 | 0.943079 | AC109309.4; MRGPRE (margin: MRGPRG-AS1; AC109309.4; MRGPRE) | 3.24 | 6.57e-03 | 3.19e-02 |
|  | 17 | 79099711 | 79100287 | PDFSTATSENSEMBLUCSC | 577 | 10 | 16894 | gene;tss;island | 0.690632 | 0.978000 | MIR338; AATK; MIR657 (margin: BAIAP2; MIR338; RP11-149I9.2; AATK; MIR657; MIR1250) | 3.24 | 3.89e-03 | 2.43e-02 |
|  | 6 | 1394986 | 1395278 | PDFSTATSENSEMBLUCSC | 293 | 4 | 33532 | gene;island | 0.982708 | 0.858059 | Not queried | 3.24 | 2.86e-02 | 6.12e-02 |
|  | 3 | 138067350 | 138067623 | PDFSTATSENSEMBLUCSC | 274 | 6 | 27963 | gene;island | 0.009852 | 0.002977 | Not queried | 3.24 | 9.31e-02 | 1.53e-01 |
|  | 13 | 26623620 | 26624039 | PDFSTATSENSEMBLUCSC | 420 | 4 | 11111 | gene;island | 0.176401 | 0.769524 | Not queried | 3.23 | 1.14e-01 | 1.68e-01 |
|  | 6 | 46702683 | 46703670 | PDFSTATSENSEMBLUCSC | 988 | 19 | 36189 | island | 0.463165 | 0.930078 | PLA2G7 (margin: ANKRD66; PLA2G7) | 3.23 | 5.23e-06 | 3.42e-04 |
|  | 5 | 171057072 | 171057339 | PDFSTATSENSEMBLUCSC | 268 | 5 | 33383 | island | 0.377312 | 0.014585 | Not queried | 3.23 | 9.52e-02 | 1.53e-01 |
|  | 17 | 21280288 | 21280740 | PDFSTATSENSEMBLUCSC | 453 | 5 | 16489 | gene;island | 0.402064 | 0.781919 | KCNJ12 (margin: KCNJ12) | 3.23 | 7.94e-03 | 3.19e-02 |
|  | 17 | 76395407 | 76395530 | PDFSTATSENSEMBLUCSC | 124 | 4 | 16820 | gene;island | 0.825028 | 0.977368 | Not queried | 3.23 | 1.14e-01 | 1.68e-01 |
|  | 12 | 25102311 | 25102521 | PDFSTATSENSEMBLUCSC | 211 | 4 | 9608 | tss | 0.836931 | 0.976995 | Not queried | 3.23 | 6.86e-01 | 6.99e-01 |
|  | 19 | 30363016 | 30363526 | PDFSTATSENSEMBLUCSC | 511 | 4 | 21858 | island | 0.529591 | 0.923117 | Not queried | 3.23 | 2.00e-01 | 2.55e-01 |
|  | 12 | 29650692 | 29651041 | PDFSTATSENSEMBLUCSC | 350 | 6 | 9623 | tss | 0.331158 | 0.779569 | Not queried | 3.23 | 2.60e-02 | 6.12e-02 |
|  | 3 | 16924563 | 16925048 | PDFSTATSENSEMBLUCSC | 486 | 4 | 28926 | island | 0.741188 | 0.957161 | Not queried | 3.23 | 2.86e-02 | 6.12e-02 |
|  | 3 | 124931535 | 124931746 | PDFSTATSENSEMBLUCSC | 212 | 5 | 29419 | island | 0.101737 | 0.500125 | SLC12A8 (margin: SLC12A8) | 3.23 | 7.94e-03 | 3.19e-02 |
|  | 1 | 146543992 | 146544267 | PDFSTATSENSEMBLUCSC | 276 | 4 | 3690 | island | 0.812212 | 0.983680 | Not queried | 3.22 | 1.14e-01 | 1.68e-01 |
|  | 4 | 41753439 | 41754125 | PDFSTATSENSEMBLUCSC | 687 | 8 | 31004 | island | 0.503217 | 0.827346 | RP11-227F19.1; RP11-227F19.2 (margin: RP11-227F19.1; RP11-227F19.2; PHOX2B) | 3.22 | 1.86e-03 | 1.45e-02 |
|  | 7 | 79764176 | 79764387 | PDFSTATSENSEMBLUCSC | 212 | 4 | 37067 | gene;island | 0.011921 | 0.124498 | Not queried | 3.22 | 2.86e-02 | 6.12e-02 |
|  | 22 | 42353589 | 42353933 | PDFSTATSENSEMBLUCSC | 345 | 4 | 26885 | gene;island | 0.684350 | 0.975992 | Not queried | 3.22 | 5.71e-02 | 1.00e-01 |
|  | 16 | 29625133 | 29625259 | PDFSTATSENSEMBLUCSC | 127 | 5 | 15166 | tss;island | 0.495038 | 0.080523 | Not queried | 3.22 | 1.51e-01 | 2.11e-01 |
|  | 12 | 132689907 | 132690782 | PDFSTATSENSEMBLUCSC | 876 | 11 | 9434 | gene;island | 0.101196 | 0.757603 | GALNT9 (margin: GALNT9) | 3.22 | 1.86e-03 | 1.45e-02 |
|  | 12 | 132690643 | 132690782 | PDFSTATSENSEMBLUCSC | 140 | 5 | 10129 | tss | 0.101196 | 0.642911 | Not queried | 3.22 | 3.17e-02 | 6.50e-02 |
|  | 3 | 133614782 | 133615241 | PDFSTATSENSEMBLUCSC | 460 | 9 | 28604 | tss;island | 0.255269 | 0.017401 | Not queried | 3.22 | 7.30e-01 | 7.40e-01 |
|  | 1 | 6419606 | 6419906 | PDFSTATSENSEMBLUCSC | 301 | 4 | 170 | gene;tss | 0.546166 | 0.907559 | Not queried | 3.22 | 1.14e-01 | 1.68e-01 |
|  | 17 | 42835688 | 42836320 | PDFSTATSENSEMBLUCSC | 633 | 5 | 17415 | tss;island | 0.652232 | 0.350351 | Not queried | 3.22 | 4.21e-01 | 4.59e-01 |
|  | 3 | 42947263 | 42947771 | PDFSTATSENSEMBLUCSC | 509 | 7 | 29023 | island | 0.898288 | 0.987141 | ACKR2; KRBOX1; ZNF662 (margin: ACKR2; KRBOX1; ZNF662) | 3.22 | 5.83e-04 | 6.47e-03 |
|  | 11 | 92930739 | 92930990 | PDFSTATSENSEMBLUCSC | 252 | 4 | 6858 | gene;island | 0.071363 | 0.006040 | Not queried | 3.22 | 5.71e-02 | 1.00e-01 |
|  | 6 | 30883762 | 30884124 | PDFSTATSENSEMBLUCSC | 363 | 4 | 33839 | gene;island | 0.936824 | 0.562863 | Not queried | 3.22 | 5.71e-02 | 1.00e-01 |
|  | 21 | 15645649 | 15645988 | PDFSTATSENSEMBLUCSC | 340 | 4 | 26487 | tss | 0.615249 | 0.968320 | Not queried | 3.22 | 2.00e-01 | 2.55e-01 |
|  | 3 | 124103560 | 124103696 | PDFSTATSENSEMBLUCSC | 137 | 4 | 27922 | gene | 0.412968 | 0.070619 | Not queried | 3.22 | 2.86e-02 | 6.12e-02 |
|  | 7 | 99517136 | 99517509 | PDFSTATSENSEMBLUCSC | 374 | 12 | 38741 | island | 0.045404 | 0.337407 | TRIM4 (margin: RP4-604G5.1; TRIM4; GJC3) | 3.22 | 8.88e-06 | 4.82e-04 |
|  | 12 | 81471311 | 81471757 | PDFSTATSENSEMBLUCSC | 447 | 7 | 9894 | tss | 0.093108 | 0.017143 | ACSS3 (margin: ACSS3) | 3.22 | 5.83e-04 | 6.47e-03 |
|  | 16 | 928778 | 929101 | PDFSTATSENSEMBLUCSC | 324 | 4 | 14549 | gene;island | 0.684554 | 0.960324 | Not queried | 3.22 | 2.00e-01 | 2.55e-01 |
|  | 5 | 38445522 | 38445916 | PDFSTATSENSEMBLUCSC | 395 | 7 | 31595 | gene | 0.673289 | 0.931376 | Not queried | 3.22 | 9.73e-02 | 1.55e-01 |
|  | 16 | 89982122 | 89982618 | PDFSTATSENSEMBLUCSC | 497 | 4 | 16276 | island | 0.631782 | 0.965249 | Not queried | 3.22 | 1.14e-01 | 1.68e-01 |
|  | 1 | 149889330 | 149889364 | PDFSTATSENSEMBLUCSC | 35 | 4 | 735 | gene | 0.375039 | 0.684958 | Not queried | 3.22 | 2.00e-01 | 2.55e-01 |
|  | 1 | 228870454 | 228870867 | PDFSTATSENSEMBLUCSC | 414 | 7 | 2309 | tss | 0.883421 | 0.449404 | Not queried | 3.22 | 2.59e-01 | 3.16e-01 |
|  | 11 | 16626910 | 16627327 | PDFSTATSENSEMBLUCSC | 418 | 4 | 8087 | island | 0.268487 | 0.022683 | Not queried | 3.22 | 5.71e-02 | 1.00e-01 |
|  | 17 | 65040527 | 65040983 | PDFSTATSENSEMBLUCSC | 457 | 10 | 18677 | island | 0.666991 | 0.969173 | CACNG1 (margin: CACNG4; CACNG1; RP11-74H8.1) | 3.21 | 7.25e-04 | 7.74e-03 |
|  | 7 | 117513825 | 117514017 | PDFSTATSENSEMBLUCSC | 193 | 5 | 37857 | tss;island | 0.645561 | 0.166292 | Not queried | 3.21 | 2.22e-01 | 2.76e-01 |
|  | 19 | 17798755 | 17799384 | PDFSTATSENSEMBLUCSC | 630 | 7 | 21749 | island | 0.522305 | 0.018647 | Not queried | 3.21 | 2.62e-02 | 6.12e-02 |
|  | 19 | 17799037 | 17799384 | PDFSTATSENSEMBLUCSC | 348 | 5 | 20596 | tss | 0.373826 | 0.008485 | Not queried | 3.21 | 5.56e-02 | 1.00e-01 |
|  | 15 | 79237145 | 79237394 | PDFSTATSENSEMBLUCSC | 250 | 5 | 13443 | gene;island | 0.018169 | 0.084564 | Not queried | 3.21 | 9.52e-02 | 1.53e-01 |
|  | 6 | 26103952 | 26104165 | PDFSTATSENSEMBLUCSC | 214 | 5 | 34685 | tss | 0.010133 | 0.017287 | Not queried | 3.21 | 2.22e-01 | 2.76e-01 |
|  | 12 | 132921188 | 132921451 | PDFSTATSENSEMBLUCSC | 264 | 4 | 11044 | island | 0.431941 | 0.821413 | Not queried | 3.21 | 4.86e-01 | 5.11e-01 |
|  | 3 | 48413550 | 48413707 | PDFSTATSENSEMBLUCSC | 158 | 4 | 28296 | tss | 0.769922 | 0.974686 | Not queried | 3.21 | 2.86e-02 | 6.12e-02 |
|  | 6 | 99276697 | 99277015 | PDFSTATSENSEMBLUCSC | 319 | 4 | 36341 | island | 0.375426 | 0.873118 | Not queried | 3.21 | 2.00e-01 | 2.55e-01 |
|  | 19 | 46999055 | 46999444 | PDFSTATSENSEMBLUCSC | 390 | 10 | 22175 | island | 0.666773 | 0.963491 | AC011484.1; PPP5D1; PNMAL2 (margin: AC011484.1; PPP5D1; PNMAL2) | 3.21 | 1.15e-02 | 4.29e-02 |
|  | 5 | 169930841 | 169931363 | PDFSTATSENSEMBLUCSC | 523 | 9 | 31890 | gene;island | 0.583777 | 0.933579 | KCNIP1 (margin: KCNIP1) | 3.21 | 1.85e-03 | 1.45e-02 |
|  | 18 | 42259977 | 42260234 | PDFSTATSENSEMBLUCSC | 258 | 7 | 19249 | tss;island | 0.107152 | 0.015072 | SETBP1; RP11-456K23.1 (margin: SETBP1; RP11-456K23.1) | 3.21 | 5.83e-04 | 6.47e-03 |
|  | 4 | 69215865 | 69216111 | PDFSTATSENSEMBLUCSC | 247 | 11 | 30420 | tss | 0.004268 | 0.009481 | Not queried | 3.21 | 2.17e-01 | 2.75e-01 |
|  | 12 | 117176113 | 117176563 | PDFSTATSENSEMBLUCSC | 451 | 6 | 9329 | gene | 0.061782 | 0.006373 | Not queried | 3.21 | 1.80e-01 | 2.45e-01 |
|  | 8 | 57360586 | 57360738 | PDFSTATSENSEMBLUCSC | 153 | 5 | 39716 | tss;island | 0.540516 | 0.113186 | Not queried | 3.20 | 1.59e-02 | 5.36e-02 |
|  | 1 | 120838320 | 120838718 | PDFSTATSENSEMBLUCSC | 399 | 6 | 1878 | tss;island | 0.016563 | 0.279320 | Not queried | 3.20 | 6.49e-02 | 1.11e-01 |
|  | 10 | 109674854 | 109675284 | PDFSTATSENSEMBLUCSC | 431 | 4 | 6121 | island | 0.535506 | 0.089510 | Not queried | 3.20 | 1.14e-01 | 1.68e-01 |
|  | 4 | 2795105 | 2795501 | PDFSTATSENSEMBLUCSC | 397 | 6 | 29893 | gene;island | 0.007643 | 0.073297 | Not queried | 3.20 | 3.10e-01 | 3.66e-01 |
|  | 1 | 160039962 | 160040667 | PDFSTATSENSEMBLUCSC | 706 | 11 | 2035 | tss | 0.362764 | 0.853883 | RP11-536C5.2; KCNJ10 (margin: RP11-536C5.2; KCNJ9; KCNJ10) | 3.20 | 1.86e-03 | 1.45e-02 |
|  | 10 | 46168377 | 46168597 | PDFSTATSENSEMBLUCSC | 221 | 6 | 5061 | tss | 0.130259 | 0.019302 | Not queried | 3.20 | 2.60e-02 | 6.12e-02 |
|  | 1 | 156339011 | 156339286 | PDFSTATSENSEMBLUCSC | 276 | 4 | 822 | gene | 0.485296 | 0.087171 | Not queried | 3.20 | 2.86e-02 | 6.12e-02 |
|  | 17 | 7791882 | 7792191 | PDFSTATSENSEMBLUCSC | 310 | 6 | 16420 | gene;island | 0.254442 | 0.040153 | CHD3 (margin: CHD3; LSMD1) | 3.20 | 8.66e-03 | 3.36e-02 |
|  | 6 | 130339410 | 130339998 | PDFSTATSENSEMBLUCSC | 589 | 10 | 36475 | island | 0.112041 | 0.534391 | L3MBTL3 (margin: L3MBTL3) | 3.20 | 1.50e-03 | 1.41e-02 |
|  | 2 | 1516061 | 1516352 | PDFSTATSENSEMBLUCSC | 292 | 4 | 22532 | gene;island | 0.652940 | 0.963946 | Not queried | 3.20 | 1.14e-01 | 1.68e-01 |
|  | 1 | 179544476 | 179544765 | PDFSTATSENSEMBLUCSC | 290 | 4 | 904 | gene;island | 0.657864 | 0.963503 | Not queried | 3.20 | 5.71e-02 | 1.00e-01 |
|  | 1 | 3477168 | 3477442 | PDFSTATSENSEMBLUCSC | 275 | 4 | 130 | gene | 0.332496 | 0.742411 | Not queried | 3.20 | 2.86e-02 | 6.12e-02 |
|  | 1 | 3123338 | 3123854 | PDFSTATSENSEMBLUCSC | 517 | 4 | 108 | gene | 0.597165 | 0.892117 | Not queried | 3.20 | 5.71e-02 | 1.00e-01 |
|  | 6 | 84562778 | 84562930 | PDFSTATSENSEMBLUCSC | 153 | 7 | 35097 | tss | 0.839812 | 0.975629 | Not queried | 3.20 | 1.75e-02 | 5.79e-02 |
|  | 10 | 102415991 | 102416715 | PDFSTATSENSEMBLUCSC | 725 | 7 | 6028 | island | 0.716020 | 0.017436 | Not queried | 3.20 | 2.62e-02 | 6.12e-02 |
|  | 1 | 32930375 | 32930544 | PDFSTATSENSEMBLUCSC | 170 | 6 | 1461 | tss;island | 0.216541 | 0.021610 | Not queried | 3.20 | 3.94e-01 | 4.36e-01 |
|  | 16 | 87812658 | 87813069 | PDFSTATSENSEMBLUCSC | 412 | 4 | 16186 | island | 0.018090 | 0.094128 | Not queried | 3.20 | 2.00e-01 | 2.55e-01 |
|  | 17 | 37824461 | 37824529 | PDFSTATSENSEMBLUCSC | 69 | 4 | 18284 | island | 0.546334 | 0.934111 | Not queried | 3.20 | 2.86e-02 | 6.12e-02 |
|  | 8 | 21644953 | 21645524 | PDFSTATSENSEMBLUCSC | 572 | 6 | 39224 | gene;island | 0.833643 | 0.951462 | GFRA2 (margin: GFRA2) | 3.20 | 4.33e-03 | 2.52e-02 |
|  | 17 | 46805785 | 46806134 | PDFSTATSENSEMBLUCSC | 350 | 5 | 18503 | island | 0.095870 | 0.776043 | HOXB13 (margin: HOXB-AS5; CTD-2377D24.4; PRAC; MIR3185; HOXB13) | 3.20 | 7.94e-03 | 3.19e-02 |
|  | 6 | 134639203 | 134639482 | PDFSTATSENSEMBLUCSC | 280 | 5 | 35235 | tss | 0.485189 | 0.878407 | SGK1 (margin: SGK1) | 3.19 | 7.94e-03 | 3.19e-02 |
|  | 6 | 33132943 | 33133083 | PDFSTATSENSEMBLUCSC | 141 | 4 | 34194 | gene;island | 0.113265 | 0.302675 | Not queried | 3.19 | 6.86e-01 | 6.99e-01 |
|  | 1 | 151693838 | 151694320 | PDFSTATSENSEMBLUCSC | 483 | 6 | 3750 | island | 0.763522 | 0.957649 | Not queried | 3.19 | 4.11e-02 | 8.14e-02 |
|  | 17 | 61699734 | 61699845 | PDFSTATSENSEMBLUCSC | 112 | 6 | 18648 | island | 0.001167 | 0.009387 | Not queried | 3.19 | 2.60e-02 | 6.12e-02 |
|  | 7 | 157447022 | 157447249 | PDFSTATSENSEMBLUCSC | 228 | 4 | 37335 | gene;island | 0.868060 | 0.914643 | Not queried | 3.19 | 6.86e-01 | 6.99e-01 |
|  | 1 | 76540438 | 76540641 | PDFSTATSENSEMBLUCSC | 204 | 4 | 591 | gene;island | 0.834805 | 0.962290 | Not queried | 3.19 | 1.14e-01 | 1.68e-01 |
|  | 4 | 74734714 | 74735149 | PDFSTATSENSEMBLUCSC | 436 | 10 | 31097 | island | 0.109363 | 0.421688 | CXCL1 (margin: CXCL1) | 3.19 | 5.20e-03 | 2.96e-02 |
|  | 4 | 126237325 | 126237513 | PDFSTATSENSEMBLUCSC | 189 | 6 | 30585 | tss | 0.876200 | 0.460205 | Not queried | 3.19 | 6.49e-02 | 1.11e-01 |
|  | 1 | 1974661 | 1974941 | PDFSTATSENSEMBLUCSC | 281 | 5 | 2469 | island | 0.780554 | 0.969601 | Not queried | 3.19 | 3.17e-02 | 6.50e-02 |
|  | 6 | 28477957 | 28478579 | PDFSTATSENSEMBLUCSC | 623 | 8 | 33632 | gene | 0.396856 | 0.929941 | Not queried | 3.19 | 6.50e-02 | 1.11e-01 |
|  | 10 | 73136913 | 73137135 | PDFSTATSENSEMBLUCSC | 223 | 4 | 5828 | island | 0.013339 | 0.090040 | Not queried | 3.19 | 5.71e-02 | 1.00e-01 |
|  | 3 | 193987426 | 193987757 | PDFSTATSENSEMBLUCSC | 332 | 4 | 29785 | island | 0.885769 | 0.983569 | Not queried | 3.19 | 1.14e-01 | 1.68e-01 |
|  | 2 | 1746509 | 1746846 | PDFSTATSENSEMBLUCSC | 338 | 4 | 22536 | gene;island | 0.432208 | 0.934863 | Not queried | 3.19 | 5.71e-02 | 1.00e-01 |
|  | 10 | 99472988 | 99473224 | PDFSTATSENSEMBLUCSC | 237 | 5 | 5270 | tss;island | 0.247105 | 0.747518 | Not queried | 3.19 | 1.51e-01 | 2.11e-01 |
|  | 1 | 181382639 | 181382934 | PDFSTATSENSEMBLUCSC | 296 | 4 | 4017 | island | 0.254286 | 0.818743 | Not queried | 3.18 | 2.00e-01 | 2.55e-01 |
|  | 19 | 51220202 | 51220537 | PDFSTATSENSEMBLUCSC | 336 | 7 | 20983 | tss | 0.376948 | 0.858886 | Not queried | 3.18 | 1.75e-02 | 5.79e-02 |
|  | 1 | 24645802 | 24645853 | PDFSTATSENSEMBLUCSC | 52 | 4 | 1358 | tss | 0.052614 | 0.005616 | Not queried | 3.18 | 2.86e-02 | 6.12e-02 |
|  | 17 | 26127438 | 26127542 | PDFSTATSENSEMBLUCSC | 105 | 5 | 16492 | gene | 0.692132 | 0.968191 | Not queried | 3.18 | 1.51e-01 | 2.11e-01 |
|  | 3 | 147111120 | 147111308 | PDFSTATSENSEMBLUCSC | 189 | 4 | 27987 | gene;tss;island | 0.896931 | 0.482626 | Not queried | 3.18 | 2.86e-02 | 6.12e-02 |
|  | 12 | 7858592 | 7858848 | PDFSTATSENSEMBLUCSC | 257 | 6 | 10228 | island | 0.596732 | 0.911515 | Not queried | 3.18 | 4.11e-02 | 8.14e-02 |
|  | 19 | 19314292 | 19314415 | PDFSTATSENSEMBLUCSC | 124 | 7 | 20638 | tss | 0.062249 | 0.012101 | Not queried | 3.18 | 2.62e-02 | 6.12e-02 |
|  | 11 | 61322742 | 61323413 | PDFSTATSENSEMBLUCSC | 672 | 6 | 6630 | gene;island | 0.655293 | 0.960546 | SYT7 (margin: SYT7) | 3.18 | 8.66e-03 | 3.36e-02 |
|  | 1 | 9711632 | 9711663 | PDFSTATSENSEMBLUCSC | 32 | 4 | 1229 | tss;island | 0.627442 | 0.135710 | Not queried | 3.18 | 2.86e-02 | 6.12e-02 |
|  | 22 | 43505892 | 43506651 | PDFSTATSENSEMBLUCSC | 760 | 24 | 27200 | tss | 0.282503 | 0.031980 | BIK (margin: BIK) | 3.18 | 8.78e-03 | 3.41e-02 |
|  | 20 | 62406677 | 62406964 | PDFSTATSENSEMBLUCSC | 288 | 4 | 25512 | gene;island | 0.633460 | 0.933573 | Not queried | 3.18 | 1.14e-01 | 1.68e-01 |
|  | 15 | 76016056 | 76016306 | PDFSTATSENSEMBLUCSC | 251 | 4 | 13818 | tss | 0.445745 | 0.740273 | Not queried | 3.18 | 6.86e-01 | 6.99e-01 |
|  | 13 | 112692119 | 112692382 | PDFSTATSENSEMBLUCSC | 264 | 4 | 11907 | island | 0.208809 | 0.603081 | Not queried | 3.17 | 4.86e-01 | 5.11e-01 |
|  | 21 | 42688675 | 42689051 | PDFSTATSENSEMBLUCSC | 377 | 4 | 26429 | gene | 0.884237 | 0.986114 | Not queried | 3.17 | 2.86e-02 | 6.12e-02 |
|  | 17 | 78865087 | 78865755 | PDFSTATSENSEMBLUCSC | 669 | 7 | 16881 | gene;island | 0.585803 | 0.921721 | RPTOR (margin: RPTOR) | 3.17 | 6.99e-03 | 3.19e-02 |
|  | 9 | 140473545 | 140473875 | PDFSTATSENSEMBLUCSC | 331 | 5 | 40859 | tss;island | 0.498423 | 0.974331 | Not queried | 3.17 | 2.22e-01 | 2.76e-01 |
|  | 12 | 56040025 | 56040336 | PDFSTATSENSEMBLUCSC | 312 | 5 | 10507 | island | 0.876983 | 0.446474 | RP11-644F5.16 (margin: OR10P1; RP11-644F5.16; RP11-644F5.15) | 3.17 | 7.94e-03 | 3.19e-02 |
|  | 5 | 176307743 | 176308136 | PDFSTATSENSEMBLUCSC | 394 | 6 | 31912 | gene;island | 0.671496 | 0.948321 | Not queried | 3.17 | 6.49e-02 | 1.11e-01 |
|  | 11 | 22359188 | 22359658 | PDFSTATSENSEMBLUCSC | 471 | 7 | 7202 | tss;island | 0.124040 | 0.016456 | Not queried | 3.17 | 2.59e-01 | 3.16e-01 |
|  | 4 | 24796689 | 24797007 | PDFSTATSENSEMBLUCSC | 319 | 6 | 30327 | tss | 0.479531 | 0.977009 | Not queried | 3.17 | 1.32e-01 | 1.89e-01 |
|  | 8 | 82192519 | 82192777 | PDFSTATSENSEMBLUCSC | 259 | 7 | 39784 | tss | 0.074019 | 0.450075 | FABP5; RP11-363E6.3 (margin: FABP5; RP11-363E6.4; RP11-363E6.3) | 3.17 | 5.83e-04 | 6.47e-03 |
|  | 7 | 1370343 | 1370727 | PDFSTATSENSEMBLUCSC | 385 | 4 | 38099 | island | 0.766656 | 0.974964 | Not queried | 3.17 | 2.86e-02 | 6.12e-02 |
|  | 22 | 42373000 | 42373373 | PDFSTATSENSEMBLUCSC | 374 | 5 | 26886 | gene;island | 0.617534 | 0.099505 | Not queried | 3.17 | 9.52e-02 | 1.53e-01 |
|  | 19 | 51165207 | 51165845 | PDFSTATSENSEMBLUCSC | 639 | 6 | 20181 | gene;island | 0.721667 | 0.959028 | Not queried | 3.17 | 2.60e-02 | 6.12e-02 |
|  | 1 | 1177703 | 1178245 | PDFSTATSENSEMBLUCSC | 543 | 6 | 2417 | island | 0.753598 | 0.972845 | FAM132A (margin: B3GALT6; SDF4; FAM132A; RP5-902P8.12; UBE2J2) | 3.17 | 4.33e-03 | 2.52e-02 |
|  | 4 | 25657365 | 25657628 | PDFSTATSENSEMBLUCSC | 264 | 7 | 30963 | island | 0.795161 | 0.973242 | Not queried | 3.17 | 1.75e-02 | 5.79e-02 |
|  | 4 | 25657365 | 25657429 | PDFSTATSENSEMBLUCSC | 65 | 5 | 30334 | tss | 0.772256 | 0.973118 | Not queried | 3.17 | 9.52e-02 | 1.53e-01 |
|  | 11 | 86085623 | 86085757 | PDFSTATSENSEMBLUCSC | 135 | 4 | 7609 | tss | 0.344536 | 0.880553 | Not queried | 3.17 | 2.00e-01 | 2.55e-01 |
|  | 3 | 44666362 | 44667010 | PDFSTATSENSEMBLUCSC | 649 | 11 | 29037 | island | 0.010515 | 0.043576 | Not queried | 3.17 | 5.57e-02 | 1.00e-01 |
|  | 22 | 50451108 | 50451414 | PDFSTATSENSEMBLUCSC | 307 | 4 | 27242 | tss | 0.584137 | 0.131498 | Not queried | 3.17 | 1.14e-01 | 1.68e-01 |
|  | 13 | 99405102 | 99405269 | PDFSTATSENSEMBLUCSC | 168 | 5 | 11510 | tss;island | 0.886270 | 0.973153 | Not queried | 3.17 | 3.17e-02 | 6.50e-02 |
|  | 1 | 2828044 | 2828293 | PDFSTATSENSEMBLUCSC | 250 | 5 | 2520 | island | 0.373517 | 0.869036 | Not queried | 3.16 | 1.59e-02 | 5.36e-02 |
|  | 12 | 85305677 | 85306021 | PDFSTATSENSEMBLUCSC | 345 | 4 | 9231 | gene;island | 0.329686 | 0.782895 | Not queried | 3.16 | 3.43e-01 | 3.84e-01 |
|  | 5 | 134871645 | 134872315 | PDFSTATSENSEMBLUCSC | 671 | 18 | 32305 | tss | 0.681826 | 0.974917 | NEUROG1 (margin: NEUROG1) | 3.16 | 3.44e-05 | 1.17e-03 |
|  | 5 | 176559881 | 176560265 | PDFSTATSENSEMBLUCSC | 385 | 4 | 32522 | tss;island | 0.949679 | 0.750630 | Not queried | 3.16 | 2.86e-02 | 6.12e-02 |
|  | 6 | 32172354 | 32172871 | PDFSTATSENSEMBLUCSC | 518 | 5 | 34089 | gene | 0.143883 | 0.622686 | Not queried | 3.16 | 2.22e-01 | 2.76e-01 |
|  | 6 | 31324997 | 31325455 | PDFSTATSENSEMBLUCSC | 459 | 11 | 34807 | tss | 0.008164 | 0.078051 | Not queried | 3.16 | 3.36e-02 | 6.87e-02 |
|  | 3 | 182400091 | 182400605 | PDFSTATSENSEMBLUCSC | 515 | 6 | 29695 | island | 0.301696 | 0.016603 | Not queried | 3.16 | 9.31e-02 | 1.53e-01 |
|  | 6 | 32062160 | 32062554 | PDFSTATSENSEMBLUCSC | 395 | 5 | 34045 | gene;island | 0.530061 | 0.904281 | TNXB (margin: TNXB; ATF6B) | 3.16 | 7.94e-03 | 3.19e-02 |
|  | 10 | 13043878 | 13043986 | PDFSTATSENSEMBLUCSC | 109 | 6 | 4953 | tss;island | 0.262884 | 0.037888 | Not queried | 3.16 | 6.49e-02 | 1.11e-01 |
|  | 6 | 168720183 | 168720679 | PDFSTATSENSEMBLUCSC | 497 | 6 | 36663 | island | 0.484328 | 0.925738 | Not queried | 3.16 | 2.60e-02 | 6.12e-02 |
|  | 15 | 100533202 | 100533336 | PDFSTATSENSEMBLUCSC | 135 | 4 | 13504 | gene;island | 0.371010 | 0.746455 | Not queried | 3.16 | 2.00e-01 | 2.55e-01 |
|  | 6 | 32363875 | 32364075 | PDFSTATSENSEMBLUCSC | 201 | 6 | 34106 | gene | 0.722917 | 0.967283 | BTNL2 (margin: HCG23; BTNL2) | 3.16 | 2.16e-03 | 1.45e-02 |
|  | 10 | 134018798 | 134019008 | PDFSTATSENSEMBLUCSC | 211 | 4 | 4857 | gene;island | 0.617257 | 0.867390 | Not queried | 3.16 | 1.14e-01 | 1.68e-01 |
|  | 12 | 52626814 | 52627047 | PDFSTATSENSEMBLUCSC | 234 | 6 | 10437 | island | 0.847509 | 0.982064 | KRT7 (margin: LINC00592; KRT7; METTL7AP1; RP3-416H24.1) | 3.15 | 2.16e-03 | 1.45e-02 |
|  | 11 | 27721222 | 27721350 | PDFSTATSENSEMBLUCSC | 129 | 5 | 7211 | tss | 0.050421 | 0.203258 | Not queried | 3.15 | 9.52e-02 | 1.53e-01 |
|  | 4 | 109684077 | 109684293 | PDFSTATSENSEMBLUCSC | 217 | 4 | 31234 | island | 0.125269 | 0.016471 | Not queried | 3.15 | 3.43e-01 | 3.84e-01 |
|  | 11 | 69634240 | 69634709 | PDFSTATSENSEMBLUCSC | 470 | 11 | 7523 | tss;island | 0.572511 | 0.945047 | FGF3 (margin: FGF3) | 3.15 | 3.18e-03 | 2.01e-02 |
|  | 15 | 23085583 | 23085908 | PDFSTATSENSEMBLUCSC | 326 | 4 | 13252 | gene;island | 0.525795 | 0.124248 | Not queried | 3.15 | 2.00e-01 | 2.55e-01 |
|  | 16 | 85171054 | 85171295 | PDFSTATSENSEMBLUCSC | 242 | 4 | 16150 | island | 0.419953 | 0.876139 | Not queried | 3.15 | 2.86e-02 | 6.12e-02 |
|  | 5 | 1948875 | 1949277 | PDFSTATSENSEMBLUCSC | 403 | 5 | 32647 | island | 0.055897 | 0.380277 | RP11-259O2.1 (margin: RP11-259O2.1) | 3.15 | 7.94e-03 | 3.19e-02 |
|  | 1 | 2410885 | 2411643 | PDFSTATSENSEMBLUCSC | 759 | 9 | 85 | gene;island | 0.600498 | 0.965712 | PLCH2 (margin: PLCH2) | 3.15 | 1.23e-03 | 1.18e-02 |
|  | 6 | 46459694 | 46460003 | PDFSTATSENSEMBLUCSC | 310 | 6 | 36186 | island | 0.312415 | 0.049138 | Not queried | 3.15 | 2.60e-02 | 6.12e-02 |
|  | 1 | 8086588 | 8086959 | PDFSTATSENSEMBLUCSC | 372 | 4 | 1220 | tss;island | 0.266587 | 0.064385 | Not queried | 3.15 | 4.86e-01 | 5.11e-01 |
|  | 4 | 114213914 | 114214449 | PDFSTATSENSEMBLUCSC | 536 | 6 | 30104 | gene | 0.566672 | 0.939516 | Not queried | 3.15 | 1.32e-01 | 1.89e-01 |
|  | 20 | 35169260 | 35169609 | PDFSTATSENSEMBLUCSC | 350 | 5 | 25716 | tss;island | 0.568155 | 0.969363 | Not queried | 3.15 | 4.21e-01 | 4.59e-01 |
|  | 9 | 130700866 | 130701076 | PDFSTATSENSEMBLUCSC | 211 | 4 | 40841 | tss;island | 0.951764 | 0.638613 | Not queried | 3.15 | 1.14e-01 | 1.68e-01 |
|  | 11 | 1219189 | 1219644 | PDFSTATSENSEMBLUCSC | 456 | 5 | 7905 | island | 0.311873 | 0.635020 | Not queried | 3.14 | 3.17e-02 | 6.50e-02 |
|  | 4 | 156298166 | 156298427 | PDFSTATSENSEMBLUCSC | 262 | 7 | 30650 | tss | 0.173501 | 0.066589 | Not queried | 3.14 | 3.83e-01 | 4.27e-01 |
|  | 21 | 42219139 | 42219299 | PDFSTATSENSEMBLUCSC | 161 | 6 | 26554 | tss | 0.719594 | 0.980315 | Not queried | 3.14 | 4.11e-02 | 8.14e-02 |
|  | 10 | 119297439 | 119297716 | PDFSTATSENSEMBLUCSC | 278 | 4 | 4784 | gene;island | 0.650347 | 0.943069 | Not queried | 3.14 | 4.86e-01 | 5.11e-01 |
|  | 19 | 46580305 | 46580807 | PDFSTATSENSEMBLUCSC | 503 | 4 | 22169 | island | 0.150243 | 0.018575 | Not queried | 3.14 | 2.00e-01 | 2.55e-01 |
|  | 6 | 27064705 | 27065003 | PDFSTATSENSEMBLUCSC | 299 | 4 | 35545 | island | 0.031474 | 0.197752 | Not queried | 3.14 | 2.86e-02 | 6.12e-02 |
|  | 18 | 77230484 | 77230795 | PDFSTATSENSEMBLUCSC | 312 | 4 | 19140 | gene;island | 0.848142 | 0.972746 | Not queried | 3.14 | 2.86e-02 | 6.12e-02 |
|  | 6 | 26225258 | 26225539 | PDFSTATSENSEMBLUCSC | 282 | 5 | 35528 | island | 0.063426 | 0.458341 | Not queried | 3.14 | 3.17e-02 | 6.50e-02 |
|  | 12 | 6421437 | 6421806 | PDFSTATSENSEMBLUCSC | 370 | 6 | 9024 | gene;tss;island | 0.353606 | 0.804743 | Not queried | 3.14 | 6.49e-02 | 1.11e-01 |
|  | 5 | 11903145 | 11903441 | PDFSTATSENSEMBLUCSC | 297 | 4 | 31562 | gene;island | 0.298753 | 0.746804 | Not queried | 3.14 | 1.14e-01 | 1.68e-01 |
|  | 2 | 200320750 | 200321136 | PDFSTATSENSEMBLUCSC | 387 | 5 | 22973 | gene;island | 0.297676 | 0.015632 | Not queried | 3.13 | 1.59e-02 | 5.36e-02 |
|  | 11 | 44927766 | 44927976 | PDFSTATSENSEMBLUCSC | 211 | 4 | 6591 | gene | 0.418463 | 0.791337 | Not queried | 3.13 | 1.14e-01 | 1.68e-01 |
|  | 11 | 64039065 | 64039302 | PDFSTATSENSEMBLUCSC | 238 | 4 | 6672 | gene;island | 0.587131 | 0.128201 | Not queried | 3.13 | 2.86e-02 | 6.12e-02 |
|  | 17 | 61699734 | 61699788 | PDFSTATSENSEMBLUCSC | 55 | 5 | 17568 | tss | 0.001651 | 0.006305 | Not queried | 3.13 | 9.52e-02 | 1.53e-01 |
|  | 19 | 58011309 | 58011582 | PDFSTATSENSEMBLUCSC | 274 | 4 | 20247 | gene | 0.442551 | 0.694029 | Not queried | 3.13 | 8.86e-01 | 8.89e-01 |
|  | 5 | 82770250 | 82770619 | PDFSTATSENSEMBLUCSC | 370 | 4 | 31670 | gene;island | 0.097836 | 0.009173 | Not queried | 3.13 | 1.14e-01 | 1.68e-01 |
|  | 16 | 22386311 | 22386531 | PDFSTATSENSEMBLUCSC | 221 | 4 | 15132 | tss;island | 0.115164 | 0.012723 | Not queried | 3.13 | 3.43e-01 | 3.84e-01 |
|  | 12 | 111618925 | 111619190 | PDFSTATSENSEMBLUCSC | 266 | 5 | 9305 | gene | 0.415191 | 0.903737 | Not queried | 3.13 | 2.22e-01 | 2.76e-01 |
|  | 12 | 48399140 | 48399493 | PDFSTATSENSEMBLUCSC | 354 | 5 | 9666 | tss;island | 0.723877 | 0.957680 | Not queried | 3.13 | 9.52e-02 | 1.53e-01 |
|  | 2 | 219646316 | 219646831 | PDFSTATSENSEMBLUCSC | 516 | 5 | 25094 | island | 0.611145 | 0.966763 | Not queried | 3.13 | 3.17e-02 | 6.50e-02 |
|  | 14 | 90526639 | 90526912 | PDFSTATSENSEMBLUCSC | 274 | 4 | 12551 | tss;island | 0.685432 | 0.230713 | Not queried | 3.13 | 2.86e-02 | 6.12e-02 |
|  | 11 | 32452038 | 32452839 | PDFSTATSENSEMBLUCSC | 802 | 8 | 6566 | gene;island | 0.807060 | 0.977151 | Not queried | 3.13 | 3.79e-02 | 7.66e-02 |
|  | 22 | 36635740 | 36636055 | PDFSTATSENSEMBLUCSC | 316 | 4 | 27105 | tss | 0.019242 | 0.143643 | Not queried | 3.13 | 2.86e-02 | 6.12e-02 |
|  | 12 | 103889772 | 103890274 | PDFSTATSENSEMBLUCSC | 503 | 9 | 9952 | tss | 0.533415 | 0.053521 | C12orf42 (margin: RP11-626I20.1; C12orf42; RP11-626I20.2) | 3.13 | 7.77e-03 | 3.19e-02 |
|  | 6 | 167275684 | 167275999 | PDFSTATSENSEMBLUCSC | 316 | 9 | 36637 | island | 0.751792 | 0.184061 | RPS6KA2; RP11-514O12.4 (margin: RPS6KA2; RP11-514O12.4) | 3.13 | 3.99e-03 | 2.47e-02 |
|  | 5 | 169532686 | 169532775 | PDFSTATSENSEMBLUCSC | 90 | 6 | 32482 | tss | 0.137076 | 0.528543 | FOXI1 (margin: FOXI1) | 3.13 | 8.66e-03 | 3.36e-02 |
|  | 7 | 19184950 | 19185407 | PDFSTATSENSEMBLUCSC | 458 | 7 | 38287 | island | 0.368601 | 0.090137 | Not queried | 3.13 | 7.28e-02 | 1.23e-01 |
|  | 3 | 129407530 | 129407548 | PDFSTATSENSEMBLUCSC | 19 | 4 | 28588 | tss | 0.978272 | 0.859932 | Not queried | 3.12 | 2.86e-02 | 6.12e-02 |
|  | 6 | 99842082 | 99842368 | PDFSTATSENSEMBLUCSC | 287 | 7 | 35133 | tss | 0.040846 | 0.062001 | Not queried | 3.12 | 2.59e-01 | 3.16e-01 |
|  | 13 | 29352633 | 29353104 | PDFSTATSENSEMBLUCSC | 472 | 4 | 11643 | island | 0.589048 | 0.883962 | Not queried | 3.12 | 1.14e-01 | 1.68e-01 |
|  | 2 | 231712722 | 231712981 | PDFSTATSENSEMBLUCSC | 260 | 5 | 25175 | island | 0.318967 | 0.063295 | Not queried | 3.12 | 6.90e-01 | 7.01e-01 |
|  | 3 | 5024761 | 5024963 | PDFSTATSENSEMBLUCSC | 203 | 5 | 27644 | gene;island | 0.829536 | 0.963436 | BHLHE40 (margin: BHLHE40; BHLHE40-AS1) | 3.12 | 7.94e-03 | 3.19e-02 |
|  | 5 | 533963 | 534332 | PDFSTATSENSEMBLUCSC | 370 | 4 | 32588 | island | 0.447368 | 0.880307 | Not queried | 3.12 | 5.71e-02 | 1.00e-01 |
|  | 11 | 107798913 | 107799287 | PDFSTATSENSEMBLUCSC | 375 | 10 | 8777 | island | 0.304828 | 0.026530 | RAB39A; SLC35F2 (margin: RAB39A; SLC35F2) | 3.12 | 3.25e-04 | 4.91e-03 |
|  | 12 | 16759234 | 16759793 | PDFSTATSENSEMBLUCSC | 560 | 11 | 9073 | gene | 0.151264 | 0.023458 | Not queried | 3.12 | 1.16e-01 | 1.71e-01 |
|  | 3 | 42814752 | 42814948 | PDFSTATSENSEMBLUCSC | 197 | 7 | 28247 | tss | 0.878643 | 0.982948 | RP11-70C1.1; CCDC13; HIGD1A (margin: RP11-70C1.1; CCDC13; HIGD1A) | 3.12 | 1.11e-02 | 4.14e-02 |
|  | 21 | 46294058 | 46294356 | PDFSTATSENSEMBLUCSC | 299 | 4 | 26589 | tss;island | 0.276527 | 0.037979 | Not queried | 3.12 | 3.43e-01 | 3.84e-01 |
|  | 8 | 82024462 | 82024860 | PDFSTATSENSEMBLUCSC | 399 | 6 | 39783 | tss;island | 0.346206 | 0.300332 | Not queried | 3.12 | 4.85e-01 | 5.11e-01 |
|  | 1 | 38941882 | 38942644 | PDFSTATSENSEMBLUCSC | 763 | 7 | 3087 | island | 0.743545 | 0.976891 | Not queried | 3.12 | 2.62e-02 | 6.12e-02 |
|  | 6 | 31781814 | 31782158 | PDFSTATSENSEMBLUCSC | 345 | 4 | 33966 | gene;tss;island | 0.163850 | 0.303190 | Not queried | 3.12 | 6.86e-01 | 6.99e-01 |
|  | 17 | 76850052 | 76850277 | PDFSTATSENSEMBLUCSC | 226 | 5 | 16829 | gene;island | 0.979287 | 0.805022 | Not queried | 3.12 | 1.59e-02 | 5.36e-02 |
|  | 20 | 62318426 | 62318588 | PDFSTATSENSEMBLUCSC | 163 | 4 | 25507 | gene;island | 0.772976 | 0.963630 | Not queried | 3.12 | 2.86e-02 | 6.12e-02 |
|  | 7 | 223445 | 223896 | PDFSTATSENSEMBLUCSC | 452 | 4 | 36705 | gene;island | 0.713359 | 0.977640 | Not queried | 3.12 | 1.14e-01 | 1.68e-01 |
|  | 5 | 140743780 | 140744155 | PDFSTATSENSEMBLUCSC | 376 | 6 | 31823 | gene;island | 0.614717 | 0.946463 | PCDHGA1; PCDHGA2; PCDHGA3; PCDHGB1; PCDHGA4; PCDHGB2; PCDHGA5 (margin: PCDHGA1; PCDHGA2; PCDHGA3; PCDHGB1; PCDHGA4; PCDHGB2; PCDHGA5; PCDHGB3; PCDHGA6) | 3.12 | 8.66e-03 | 3.36e-02 |
|  | 5 | 39425374 | 39425549 | PDFSTATSENSEMBLUCSC | 176 | 6 | 32033 | tss | 0.721798 | 0.243487 | Not queried | 3.12 | 4.85e-01 | 5.11e-01 |
|  | 17 | 79412583 | 79412788 | PDFSTATSENSEMBLUCSC | 206 | 4 | 16910 | gene;island | 0.800772 | 0.977787 | Not queried | 3.12 | 5.71e-02 | 1.00e-01 |
|  | 12 | 53693054 | 53693490 | PDFSTATSENSEMBLUCSC | 437 | 10 | 10460 | island | 0.020649 | 0.178558 | Not queried | 3.12 | 1.43e-01 | 2.04e-01 |
|  | 14 | 57275166 | 57275413 | PDFSTATSENSEMBLUCSC | 248 | 4 | 12092 | gene;island | 0.421286 | 0.837208 | Not queried | 3.12 | 4.86e-01 | 5.11e-01 |
|  | 5 | 148521450 | 148521623 | PDFSTATSENSEMBLUCSC | 174 | 4 | 31856 | gene;island | 0.140516 | 0.626453 | Not queried | 3.12 | 2.86e-02 | 6.12e-02 |
|  | 6 | 35265616 | 35265759 | PDFSTATSENSEMBLUCSC | 144 | 4 | 34309 | gene | 0.220272 | 0.039957 | Not queried | 3.12 | 1.14e-01 | 1.68e-01 |
|  | 3 | 46718941 | 46719086 | PDFSTATSENSEMBLUCSC | 146 | 4 | 27752 | gene;tss | 0.763631 | 0.975632 | Not queried | 3.12 | 5.71e-02 | 1.00e-01 |
|  | 12 | 131303478 | 131303774 | PDFSTATSENSEMBLUCSC | 297 | 4 | 9419 | gene;island | 0.204238 | 0.017350 | Not queried | 3.12 | 2.86e-02 | 6.12e-02 |
|  | 12 | 49504641 | 49505053 | PDFSTATSENSEMBLUCSC | 413 | 7 | 10386 | island | 0.011716 | 0.019282 | Not queried | 3.12 | 3.18e-01 | 3.74e-01 |
|  | 3 | 35785735 | 35785958 | PDFSTATSENSEMBLUCSC | 224 | 4 | 28218 | tss | 0.039795 | 0.287259 | Not queried | 3.11 | 2.86e-02 | 6.12e-02 |
|  | 3 | 79815639 | 79815848 | PDFSTATSENSEMBLUCSC | 210 | 4 | 27863 | gene;island | 0.926523 | 0.508016 | Not queried | 3.11 | 5.71e-02 | 1.00e-01 |
|  | 11 | 43963820 | 43964150 | PDFSTATSENSEMBLUCSC | 331 | 7 | 8220 | island | 0.193499 | 0.035821 | Not queried | 3.11 | 5.35e-01 | 5.62e-01 |
|  | 2 | 33952423 | 33952673 | PDFSTATSENSEMBLUCSC | 251 | 4 | 22644 | gene;island | 0.558396 | 0.961525 | Not queried | 3.11 | 2.00e-01 | 2.55e-01 |
|  | 19 | 51293367 | 51293677 | PDFSTATSENSEMBLUCSC | 311 | 6 | 22323 | island | 0.682881 | 0.980773 | Not queried | 3.11 | 2.60e-02 | 6.12e-02 |
|  | 17 | 79430641 | 79431121 | PDFSTATSENSEMBLUCSC | 481 | 5 | 16912 | gene;island | 0.617223 | 0.929928 | Not queried | 3.11 | 1.59e-02 | 5.36e-02 |
|  | 12 | 53183773 | 53184086 | PDFSTATSENSEMBLUCSC | 314 | 5 | 9140 | gene;island | 0.898883 | 0.986093 | Not queried | 3.11 | 1.59e-02 | 5.36e-02 |
|  | 7 | 1126249 | 1126903 | PDFSTATSENSEMBLUCSC | 655 | 8 | 36748 | gene;tss;island | 0.235165 | 0.348165 | Not queried | 3.11 | 2.79e-01 | 3.38e-01 |
|  | 18 | 49868101 | 49868552 | PDFSTATSENSEMBLUCSC | 452 | 7 | 19116 | gene;island | 0.956756 | 0.684689 | Not queried | 3.11 | 1.28e-01 | 1.87e-01 |
|  | 8 | 104310606 | 104311085 | PDFSTATSENSEMBLUCSC | 480 | 8 | 40517 | island | 0.004542 | 0.036168 | Not queried | 3.11 | 1.48e-02 | 5.36e-02 |
|  | 20 | 5485245 | 5485511 | PDFSTATSENSEMBLUCSC | 267 | 7 | 25583 | tss | 0.549037 | 0.904138 | Not queried | 3.11 | 5.30e-02 | 1.00e-01 |
|  | 15 | 91642909 | 91643135 | PDFSTATSENSEMBLUCSC | 227 | 9 | 13910 | tss;island | 0.391796 | 0.727080 | SV2B (margin: SV2B) | 3.11 | 7.77e-03 | 3.19e-02 |
|  | 21 | 34774882 | 34775045 | PDFSTATSENSEMBLUCSC | 164 | 4 | 26520 | tss;island | 0.969012 | 0.842372 | Not queried | 3.11 | 2.86e-02 | 6.12e-02 |
|  | 2 | 88427581 | 88427827 | PDFSTATSENSEMBLUCSC | 247 | 4 | 23499 | tss | 0.179673 | 0.576531 | Not queried | 3.11 | 2.00e-01 | 2.55e-01 |
|  | 3 | 64430634 | 64431038 | PDFSTATSENSEMBLUCSC | 405 | 5 | 29261 | island | 0.235743 | 0.029687 | Not queried | 3.11 | 2.22e-01 | 2.76e-01 |
|  | 7 | 50628718 | 50629067 | PDFSTATSENSEMBLUCSC | 350 | 4 | 37012 | gene | 0.096547 | 0.339019 | Not queried | 3.11 | 1.14e-01 | 1.68e-01 |
|  | 17 | 6918181 | 6918952 | PDFSTATSENSEMBLUCSC | 772 | 6 | 16381 | gene | 0.008037 | 0.098821 | Not queried | 3.11 | 3.94e-01 | 4.36e-01 |
|  | 11 | 118230048 | 118230230 | PDFSTATSENSEMBLUCSC | 183 | 6 | 7731 | tss | 0.063379 | 0.298424 | Not queried | 3.11 | 3.94e-01 | 4.36e-01 |
|  | 11 | 118230048 | 118230422 | PDFSTATSENSEMBLUCSC | 375 | 10 | 8855 | island | 0.013887 | 0.015779 | Not queried | 3.11 | 3.93e-01 | 4.36e-01 |
|  | 7 | 158936508 | 158936739 | PDFSTATSENSEMBLUCSC | 232 | 4 | 37385 | gene;island | 0.885393 | 0.980923 | Not queried | 3.11 | 1.14e-01 | 1.68e-01 |
|  | 11 | 32459428 | 32459954 | PDFSTATSENSEMBLUCSC | 527 | 4 | 6569 | gene;island | 0.326881 | 0.844203 | Not queried | 3.11 | 1.14e-01 | 1.68e-01 |
|  | 16 | 23765810 | 23766116 | PDFSTATSENSEMBLUCSC | 307 | 5 | 15769 | island | 0.581058 | 0.076231 | Not queried | 3.10 | 3.17e-02 | 6.50e-02 |
|  | 21 | 47604052 | 47604361 | PDFSTATSENSEMBLUCSC | 310 | 4 | 26473 | gene;island | 0.291651 | 0.008197 | Not queried | 3.10 | 1.14e-01 | 1.68e-01 |
|  | 19 | 35800589 | 35801014 | PDFSTATSENSEMBLUCSC | 426 | 4 | 19983 | gene;island | 0.754013 | 0.399194 | Not queried | 3.10 | 1.14e-01 | 1.68e-01 |
|  | 5 | 145718392 | 145718669 | PDFSTATSENSEMBLUCSC | 278 | 6 | 33281 | island | 0.571079 | 0.932292 | Not queried | 3.10 | 1.52e-02 | 5.36e-02 |
|  | 5 | 145718392 | 145718491 | PDFSTATSENSEMBLUCSC | 100 | 4 | 32406 | tss | 0.571079 | 0.932292 | Not queried | 3.10 | 2.86e-02 | 6.12e-02 |
|  | 1 | 48449748 | 48450218 | PDFSTATSENSEMBLUCSC | 471 | 5 | 3244 | island | 0.896349 | 0.985293 | Not queried | 3.10 | 9.52e-02 | 1.53e-01 |
|  | 1 | 13910138 | 13910224 | PDFSTATSENSEMBLUCSC | 87 | 6 | 1261 | tss;island | 0.410967 | 0.858160 | PDPN (margin: PDPN) | 3.10 | 2.16e-03 | 1.45e-02 |
|  | 19 | 15122135 | 15122224 | PDFSTATSENSEMBLUCSC | 90 | 4 | 19898 | gene;island | 0.602940 | 0.933746 | Not queried | 3.10 | 2.00e-01 | 2.55e-01 |
|  | 10 | 8094431 | 8094860 | PDFSTATSENSEMBLUCSC | 430 | 6 | 4487 | gene;island | 0.928300 | 0.596975 | RP11-379F12.4; GATA3; GATA3-AS1; RP11-379F12.3 (margin: RP11-379F12.4; GATA3; GATA3-AS1; RP11-379F12.3) | 3.10 | 2.16e-03 | 1.45e-02 |
|  | 17 | 48473757 | 48474099 | PDFSTATSENSEMBLUCSC | 343 | 4 | 16686 | gene;island | 0.006973 | 0.039453 | Not queried | 3.10 | 1.14e-01 | 1.68e-01 |
|  | 18 | 77203371 | 77203667 | PDFSTATSENSEMBLUCSC | 297 | 4 | 19139 | gene;island | 0.634407 | 0.952443 | Not queried | 3.10 | 3.43e-01 | 3.84e-01 |
|  | 11 | 8290295 | 8290476 | PDFSTATSENSEMBLUCSC | 182 | 5 | 8032 | island | 0.890313 | 0.973319 | LMO1 (margin: LMO1) | 3.10 | 7.94e-03 | 3.19e-02 |
|  | 15 | 26327718 | 26328092 | PDFSTATSENSEMBLUCSC | 375 | 4 | 13962 | island | 0.022239 | 0.002326 | Not queried | 3.09 | 5.71e-02 | 1.00e-01 |
|  | 6 | 159064992 | 159065192 | PDFSTATSENSEMBLUCSC | 201 | 5 | 34530 | gene;island | 0.005664 | 0.027727 | Not queried | 3.09 | 9.52e-02 | 1.53e-01 |
|  | 1 | 186649559 | 186649985 | PDFSTATSENSEMBLUCSC | 427 | 7 | 2146 | tss | 0.224675 | 0.042449 | Not queried | 3.09 | 5.30e-02 | 1.00e-01 |
|  | 20 | 56725695 | 56725899 | PDFSTATSENSEMBLUCSC | 205 | 8 | 25847 | tss | 0.757011 | 0.962071 | C20orf85 (margin: C20orf85) | 3.09 | 4.66e-03 | 2.68e-02 |
|  | 1 | 67772896 | 67773175 | PDFSTATSENSEMBLUCSC | 280 | 8 | 3365 | island | 0.868547 | 0.981446 | IL12RB2 (margin: IL12RB2) | 3.09 | 6.99e-03 | 3.19e-02 |
|  | 11 | 2465887 | 2466141 | PDFSTATSENSEMBLUCSC | 255 | 8 | 7074 | tss;island | 0.048537 | 0.009893 | Not queried | 3.09 | 2.81e-02 | 6.12e-02 |
|  | 2 | 106959205 | 106959525 | PDFSTATSENSEMBLUCSC | 321 | 5 | 24592 | island | 0.733189 | 0.973352 | AC114755.3 (margin: AC114755.3) | 3.09 | 7.94e-03 | 3.19e-02 |
|  | 19 | 6476756 | 6477198 | PDFSTATSENSEMBLUCSC | 443 | 4 | 19763 | gene;island | 0.802447 | 0.959924 | Not queried | 3.09 | 1.14e-01 | 1.68e-01 |
|  | 5 | 1110019 | 1110315 | PDFSTATSENSEMBLUCSC | 297 | 4 | 31525 | gene;island | 0.764769 | 0.961966 | Not queried | 3.09 | 5.71e-02 | 1.00e-01 |
|  | 5 | 404678 | 404996 | PDFSTATSENSEMBLUCSC | 319 | 5 | 31501 | gene;tss | 0.828891 | 0.904399 | Not queried | 3.09 | 3.10e-01 | 3.66e-01 |
|  | 11 | 1706894 | 1707163 | PDFSTATSENSEMBLUCSC | 270 | 4 | 6428 | gene;tss | 0.351506 | 0.734420 | Not queried | 3.09 | 1.14e-01 | 1.68e-01 |
|  | 6 | 33241226 | 33241770 | PDFSTATSENSEMBLUCSC | 545 | 5 | 34245 | gene;island | 0.040175 | 0.247044 | Not queried | 3.09 | 9.52e-02 | 1.53e-01 |
|  | 1 | 26126233 | 26126588 | PDFSTATSENSEMBLUCSC | 356 | 7 | 1374 | tss | 0.509616 | 0.112106 | Not queried | 3.09 | 2.59e-01 | 3.16e-01 |
|  | 7 | 53103214 | 53103312 | PDFSTATSENSEMBLUCSC | 99 | 5 | 37600 | tss;island | 0.569286 | 0.175834 | Not queried | 3.09 | 1.59e-02 | 5.36e-02 |
|  | 7 | 157448241 | 157448659 | PDFSTATSENSEMBLUCSC | 419 | 4 | 37336 | gene;island | 0.468749 | 0.801269 | Not queried | 3.09 | 3.43e-01 | 3.84e-01 |
|  | 2 | 241389021 | 241389258 | PDFSTATSENSEMBLUCSC | 238 | 4 | 23120 | gene;island | 0.629363 | 0.978452 | Not queried | 3.09 | 4.86e-01 | 5.11e-01 |
|  | 6 | 70991156 | 70991283 | PDFSTATSENSEMBLUCSC | 128 | 4 | 34382 | gene;island | 0.224339 | 0.561860 | Not queried | 3.09 | 3.43e-01 | 3.84e-01 |
|  | 10 | 134853084 | 134853344 | PDFSTATSENSEMBLUCSC | 261 | 4 | 6328 | island | 0.172420 | 0.702596 | Not queried | 3.09 | 2.00e-01 | 2.55e-01 |
|  | 20 | 22559305 | 22559456 | PDFSTATSENSEMBLUCSC | 152 | 4 | 25632 | tss;island | 0.829858 | 0.485976 | Not queried | 3.09 | 2.00e-01 | 2.55e-01 |
|  | 4 | 13549494 | 13549839 | PDFSTATSENSEMBLUCSC | 346 | 7 | 30305 | tss | 0.445335 | 0.662277 | Not queried | 3.09 | 3.18e-01 | 3.74e-01 |
|  | 1 | 24127669 | 24128066 | PDFSTATSENSEMBLUCSC | 398 | 4 | 1349 | tss;island | 0.027413 | 0.009081 | Not queried | 3.09 | 3.43e-01 | 3.84e-01 |
|  | 2 | 209271661 | 209272057 | PDFSTATSENSEMBLUCSC | 397 | 5 | 23000 | gene | 0.773230 | 0.942661 | PTH2R (margin: PTH2R) | 3.08 | 7.94e-03 | 3.19e-02 |
|  | 17 | 80829028 | 80829309 | PDFSTATSENSEMBLUCSC | 282 | 4 | 16962 | gene;island | 0.669638 | 0.978857 | Not queried | 3.08 | 1.14e-01 | 1.68e-01 |
|  | 6 | 41339246 | 41340058 | PDFSTATSENSEMBLUCSC | 813 | 7 | 36119 | island | 0.753438 | 0.920444 | Not queried | 3.08 | 3.79e-02 | 7.66e-02 |
|  | 2 | 30144547 | 30144838 | PDFSTATSENSEMBLUCSC | 292 | 6 | 23283 | tss | 0.051554 | 0.007437 | Not queried | 3.08 | 2.60e-02 | 6.12e-02 |
|  | 8 | 25905478 | 25905811 | PDFSTATSENSEMBLUCSC | 334 | 4 | 40171 | island | 0.878542 | 0.983862 | Not queried | 3.08 | 2.86e-02 | 6.12e-02 |
|  | 2 | 45168798 | 45168924 | PDFSTATSENSEMBLUCSC | 127 | 4 | 23333 | tss;island | 0.491979 | 0.109150 | Not queried | 3.08 | 1.14e-01 | 1.68e-01 |
|  | 7 | 75157345 | 75157765 | PDFSTATSENSEMBLUCSC | 421 | 9 | 38585 | island | 0.008794 | 0.042409 | PMS2P3 (margin: PMS2P3; HIP1) | 3.08 | 7.82e-04 | 8.17e-03 |
|  | 1 | 156252714 | 156253152 | PDFSTATSENSEMBLUCSC | 439 | 6 | 819 | gene | 0.003725 | 0.017729 | Not queried | 3.08 | 1.80e-01 | 2.45e-01 |
|  | 16 | 84346722 | 84346930 | PDFSTATSENSEMBLUCSC | 209 | 4 | 14868 | gene;island | 0.552770 | 0.912999 | Not queried | 3.08 | 2.86e-02 | 6.12e-02 |
|  | 6 | 31973051 | 31973345 | PDFSTATSENSEMBLUCSC | 295 | 5 | 34859 | tss | 0.225951 | 0.520847 | Not queried | 3.08 | 5.56e-02 | 1.00e-01 |
|  | 1 | 19181040 | 19181459 | PDFSTATSENSEMBLUCSC | 420 | 6 | 276 | gene;island | 0.714712 | 0.939280 | Not queried | 3.08 | 4.11e-02 | 8.14e-02 |
|  | 3 | 136538801 | 136539113 | PDFSTATSENSEMBLUCSC | 313 | 4 | 27960 | gene;island | 0.664312 | 0.963142 | Not queried | 3.08 | 1.14e-01 | 1.68e-01 |
|  | 2 | 8168023 | 8168335 | PDFSTATSENSEMBLUCSC | 313 | 5 | 24047 | island | 0.750105 | 0.961037 | Not queried | 3.08 | 5.56e-02 | 1.00e-01 |
|  | 11 | 116694005 | 116694372 | PDFSTATSENSEMBLUCSC | 368 | 6 | 8837 | island | 0.562780 | 0.912153 | APOA4 (margin: APOC3; AP006216.5; APOA4) | 3.08 | 8.66e-03 | 3.36e-02 |
|  | 22 | 45809244 | 45810043 | PDFSTATSENSEMBLUCSC | 800 | 16 | 27223 | tss;island | 0.031412 | 0.402049 | Not queried | 3.08 | 4.26e-02 | 8.44e-02 |
|  | 10 | 52750736 | 52751341 | PDFSTATSENSEMBLUCSC | 606 | 8 | 5746 | island | 0.851085 | 0.348427 | PRKG1 (margin: PRKG1) | 3.08 | 3.11e-04 | 4.77e-03 |
|  | 6 | 24583477 | 24583848 | PDFSTATSENSEMBLUCSC | 372 | 5 | 33592 | gene | 0.539475 | 0.021966 | Not queried | 3.08 | 2.22e-01 | 2.76e-01 |
|  | 2 | 48982509 | 48982957 | PDFSTATSENSEMBLUCSC | 449 | 6 | 24290 | island | 0.794033 | 0.979658 | Not queried | 3.07 | 4.11e-02 | 8.14e-02 |
|  | 12 | 54426498 | 54427884 | PDFSTATSENSEMBLUCSC | 1387 | 17 | 9160 | gene;island | 0.066249 | 0.401070 | HOXC5; HOXC6; HOXC4; MIR615 (margin: HOXC5; HOXC6; HOXC4; AC012531.25; MIR615) | 3.07 | 7.61e-03 | 3.19e-02 |
|  | 1 | 18806517 | 18806982 | PDFSTATSENSEMBLUCSC | 466 | 4 | 1294 | tss;island | 0.190992 | 0.629857 | Not queried | 3.07 | 3.43e-01 | 3.84e-01 |
|  | 4 | 3742095 | 3742357 | PDFSTATSENSEMBLUCSC | 263 | 5 | 30852 | island | 0.517326 | 0.947119 | (margin: ) | 3.07 | 7.94e-03 | 3.19e-02 |
|  | 20 | 56725695 | 56726185 | PDFSTATSENSEMBLUCSC | 491 | 11 | 26286 | island | 0.732469 | 0.958262 | C20orf85 (margin: C20orf85) | 3.07 | 1.90e-04 | 3.21e-03 |
|  | 11 | 3253659 | 3254504 | PDFSTATSENSEMBLUCSC | 846 | 14 | 7094 | tss | 0.490052 | 0.942904 | Not queried | 3.07 | 1.62e-02 | 5.47e-02 |
|  | 1 | 159824866 | 159825173 | PDFSTATSENSEMBLUCSC | 308 | 5 | 837 | gene;island | 0.071684 | 0.614778 | Not queried | 3.07 | 1.51e-01 | 2.11e-01 |
|  | 1 | 201617847 | 201618284 | PDFSTATSENSEMBLUCSC | 438 | 6 | 940 | gene;island | 0.603554 | 0.146408 | NAV1 (margin: NAV1) | 3.07 | 4.33e-03 | 2.52e-02 |
|  | 7 | 1329387 | 1329592 | PDFSTATSENSEMBLUCSC | 206 | 5 | 38097 | island | 0.186894 | 0.591245 | Not queried | 3.07 | 1.51e-01 | 2.11e-01 |
|  | 5 | 140810051 | 140810137 | PDFSTATSENSEMBLUCSC | 87 | 5 | 32383 | tss | 0.972490 | 0.623122 | Not queried | 3.07 | 1.51e-01 | 2.11e-01 |
|  | 7 | 75931606 | 75932007 | PDFSTATSENSEMBLUCSC | 402 | 11 | 38597 | island | 0.163115 | 0.015843 | HSPB1 (margin: HSPB1) | 3.07 | 2.45e-03 | 1.59e-02 |
|  | 11 | 34378805 | 34379053 | PDFSTATSENSEMBLUCSC | 249 | 6 | 7240 | tss | 0.246415 | 0.016970 | Not queried | 3.07 | 6.49e-02 | 1.11e-01 |
|  | 4 | 111544271 | 111544463 | PDFSTATSENSEMBLUCSC | 193 | 7 | 30549 | tss | 0.050937 | 0.008848 | PITX2 (margin: RP11-380D23.2; PITX2) | 3.07 | 1.11e-02 | 4.14e-02 |
|  | 3 | 192126849 | 192126996 | PDFSTATSENSEMBLUCSC | 148 | 4 | 28790 | tss | 0.665955 | 0.919172 | Not queried | 3.07 | 2.86e-02 | 6.12e-02 |
|  | 18 | 77289084 | 77289452 | PDFSTATSENSEMBLUCSC | 369 | 4 | 19603 | island | 0.072267 | 0.311078 | Not queried | 3.07 | 2.00e-01 | 2.55e-01 |
|  | 17 | 62915551 | 62915920 | PDFSTATSENSEMBLUCSC | 370 | 4 | 17589 | tss;island | 0.017342 | 0.183224 | Not queried | 3.07 | 1.14e-01 | 1.68e-01 |
|  | 6 | 41343210 | 41343830 | PDFSTATSENSEMBLUCSC | 621 | 6 | 36120 | island | 0.909795 | 0.981433 | Not queried | 3.07 | 6.49e-02 | 1.11e-01 |
|  | 19 | 31841161 | 31841663 | PDFSTATSENSEMBLUCSC | 503 | 6 | 20674 | tss;island | 0.190935 | 0.050910 | Not queried | 3.07 | 6.49e-02 | 1.11e-01 |
|  | 12 | 4383507 | 4383770 | PDFSTATSENSEMBLUCSC | 264 | 5 | 9013 | gene;island | 0.531020 | 0.866070 | Not queried | 3.06 | 5.56e-02 | 1.00e-01 |
|  | 6 | 29690766 | 29691003 | PDFSTATSENSEMBLUCSC | 238 | 6 | 34752 | tss | 0.015279 | 0.170814 | Not queried | 3.06 | 6.49e-02 | 1.11e-01 |
|  | 14 | 101349120 | 101349373 | PDFSTATSENSEMBLUCSC | 254 | 7 | 12212 | gene;tss;island | 0.421664 | 0.875690 | Not queried | 3.06 | 2.62e-02 | 6.12e-02 |
|  | 8 | 21881998 | 21882104 | PDFSTATSENSEMBLUCSC | 107 | 4 | 39578 | tss;island | 0.760317 | 0.961477 | Not queried | 3.06 | 1.14e-01 | 1.68e-01 |
|  | 11 | 65339691 | 65339797 | PDFSTATSENSEMBLUCSC | 107 | 5 | 7434 | tss;island | 0.003847 | 0.025951 | SSSCA1; FAM89B; SSSCA1-AS1 (margin: SSSCA1; FAM89B; EHBP1L1; SSSCA1-AS1) | 3.06 | 7.94e-03 | 3.19e-02 |
|  | 19 | 35773231 | 35773479 | PDFSTATSENSEMBLUCSC | 249 | 5 | 21901 | island | 0.751326 | 0.963110 | Not queried | 3.06 | 6.90e-01 | 7.01e-01 |
|  | 14 | 77647955 | 77648283 | PDFSTATSENSEMBLUCSC | 329 | 8 | 13031 | island | 0.059544 | 0.008973 | RP11-463C8.4; TMEM63C (margin: RP11-463C8.4; TMEM63C; RP11-463C8.5) | 3.06 | 1.04e-02 | 3.96e-02 |
|  | 19 | 38308057 | 38308458 | PDFSTATSENSEMBLUCSC | 402 | 4 | 21963 | island | 0.833986 | 0.968745 | Not queried | 3.06 | 2.86e-02 | 6.12e-02 |
|  | 1 | 77747875 | 77748480 | PDFSTATSENSEMBLUCSC | 606 | 9 | 594 | gene;island | 0.454476 | 0.033711 | AK5 (margin: AK5) | 3.06 | 1.06e-02 | 4.01e-02 |
|  | 21 | 38593636 | 38593754 | PDFSTATSENSEMBLUCSC | 119 | 4 | 26678 | island | 0.013419 | 0.003084 | Not queried | 3.06 | 2.86e-02 | 6.12e-02 |
|  | 11 | 18230491 | 18230799 | PDFSTATSENSEMBLUCSC | 309 | 7 | 7181 | tss | 0.857447 | 0.979927 | Not queried | 3.06 | 5.30e-02 | 1.00e-01 |
|  | 7 | 37956276 | 37957021 | PDFSTATSENSEMBLUCSC | 746 | 12 | 38418 | island | 0.425125 | 0.062091 | Not queried | 3.06 | 8.87e-02 | 1.49e-01 |
|  | 1 | 1395945 | 1396296 | PDFSTATSENSEMBLUCSC | 352 | 7 | 37 | gene;island | 0.669091 | 0.979590 | ATAD3C (margin: ATAD3C; ATAD3B) | 3.06 | 4.08e-03 | 2.47e-02 |
|  | 7 | 4869981 | 4870162 | PDFSTATSENSEMBLUCSC | 182 | 4 | 36859 | gene | 0.461335 | 0.942266 | Not queried | 3.06 | 3.43e-01 | 3.84e-01 |
|  | 6 | 17281327 | 17281838 | PDFSTATSENSEMBLUCSC | 512 | 7 | 34653 | tss;island | 0.127645 | 0.013150 | Not queried | 3.06 | 2.09e-01 | 2.65e-01 |
|  | 1 | 34090664 | 34090941 | PDFSTATSENSEMBLUCSC | 278 | 5 | 405 | gene;island | 0.706853 | 0.895352 | Not queried | 3.06 | 1.51e-01 | 2.11e-01 |
|  | 1 | 92414722 | 92414910 | PDFSTATSENSEMBLUCSC | 189 | 4 | 1745 | tss | 0.855188 | 0.980836 | Not queried | 3.06 | 1.14e-01 | 1.68e-01 |
|  | 15 | 93632611 | 93632896 | PDFSTATSENSEMBLUCSC | 286 | 5 | 13915 | tss;island | 0.204435 | 0.697383 | Not queried | 3.06 | 3.10e-01 | 3.66e-01 |
|  | 4 | 1243849 | 1244086 | PDFSTATSENSEMBLUCSC | 238 | 7 | 30240 | tss;island | 0.419754 | 0.939414 | Not queried | 3.06 | 2.62e-02 | 6.12e-02 |
|  | 11 | 2322286 | 2323459 | PDFSTATSENSEMBLUCSC | 1174 | 23 | 6454 | gene;tss | 0.453420 | 0.904338 | TSPAN32; C11orf21 (margin: TSPAN32; C11orf21) | 3.06 | 5.58e-05 | 1.52e-03 |
|  | 10 | 7661622 | 7661796 | PDFSTATSENSEMBLUCSC | 175 | 4 | 4937 | tss | 0.588184 | 0.922754 | Not queried | 3.06 | 4.86e-01 | 5.11e-01 |
|  | 4 | 1800154 | 1800448 | PDFSTATSENSEMBLUCSC | 295 | 4 | 29875 | gene;island | 0.531461 | 0.953767 | Not queried | 3.06 | 4.86e-01 | 5.11e-01 |
|  | 6 | 160679391 | 160679811 | PDFSTATSENSEMBLUCSC | 421 | 4 | 34534 | gene | 0.689405 | 0.931297 | Not queried | 3.05 | 2.86e-02 | 6.12e-02 |
|  | 16 | 1030383 | 1030619 | PDFSTATSENSEMBLUCSC | 237 | 8 | 14986 | tss;island | 0.828697 | 0.984763 | AC009041.2; SOX8; LMF1; RP11-161M6.2 (margin: AC009041.2; SOX8; LMF1; RP11-161M6.2; RP11-161M6.3) | 3.05 | 2.95e-03 | 1.88e-02 |
|  | 10 | 50887472 | 50887632 | PDFSTATSENSEMBLUCSC | 161 | 7 | 5076 | tss | 0.527197 | 0.971182 | Not queried | 3.05 | 5.30e-02 | 1.00e-01 |
|  | 3 | 122399230 | 122399601 | PDFSTATSENSEMBLUCSC | 372 | 7 | 28540 | tss | 0.014170 | 0.150127 | PARP14 (margin: PARP14) | 3.05 | 4.08e-03 | 2.47e-02 |
|  | 2 | 128284465 | 128284730 | PDFSTATSENSEMBLUCSC | 266 | 4 | 23613 | tss;island | 0.003941 | 0.002770 | Not queried | 3.05 | 6.86e-01 | 6.99e-01 |
|  | 7 | 75443075 | 75443246 | PDFSTATSENSEMBLUCSC | 172 | 4 | 37657 | tss | 0.429045 | 0.868540 | Not queried | 3.05 | 3.43e-01 | 3.84e-01 |
|  | 6 | 167040675 | 167040954 | PDFSTATSENSEMBLUCSC | 280 | 4 | 34551 | gene;island | 0.006451 | 0.015719 | Not queried | 3.05 | 3.43e-01 | 3.84e-01 |
|  | 11 | 14993866 | 14995322 | PDFSTATSENSEMBLUCSC | 1457 | 34 | 7167 | tss | 0.575779 | 0.961273 | CALCB; CALCA (margin: CALCB; CALCA) | 3.05 | 7.89e-07 | 7.54e-05 |
|  | 4 | 1005876 | 1006278 | PDFSTATSENSEMBLUCSC | 403 | 10 | 29856 | gene;island | 0.128591 | 0.048902 | Not queried | 3.05 | 1.65e-01 | 2.29e-01 |
|  | 17 | 19436770 | 19437181 | PDFSTATSENSEMBLUCSC | 412 | 8 | 18098 | island | 0.832976 | 0.428922 | Not queried | 3.05 | 2.34e-01 | 2.90e-01 |
|  | 10 | 101282572 | 101282883 | PDFSTATSENSEMBLUCSC | 312 | 5 | 6009 | island | 0.519012 | 0.918079 | Not queried | 3.05 | 1.51e-01 | 2.11e-01 |
|  | 16 | 29912904 | 29913223 | PDFSTATSENSEMBLUCSC | 320 | 4 | 14715 | gene;island | 0.632275 | 0.931168 | Not queried | 3.05 | 5.71e-02 | 1.00e-01 |
|  | 3 | 128712993 | 128713151 | PDFSTATSENSEMBLUCSC | 159 | 5 | 28574 | tss | 0.261536 | 0.027791 | Not queried | 3.05 | 5.56e-02 | 1.00e-01 |
|  | 2 | 233390712 | 233390859 | PDFSTATSENSEMBLUCSC | 148 | 4 | 23950 | tss | 0.251407 | 0.768398 | Not queried | 3.05 | 5.71e-02 | 1.00e-01 |
|  | 12 | 132827318 | 132827617 | PDFSTATSENSEMBLUCSC | 300 | 4 | 9438 | gene;island | 0.874654 | 0.978017 | Not queried | 3.05 | 5.71e-02 | 1.00e-01 |
|  | 1 | 2984218 | 2984275 | PDFSTATSENSEMBLUCSC | 58 | 4 | 96 | gene | 0.723749 | 0.973951 | Not queried | 3.04 | 2.86e-02 | 6.12e-02 |
|  | 7 | 141673582 | 141673903 | PDFSTATSENSEMBLUCSC | 322 | 4 | 37947 | tss | 0.721239 | 0.893690 | Not queried | 3.04 | 4.86e-01 | 5.11e-01 |
|  | 19 | 855899 | 856145 | PDFSTATSENSEMBLUCSC | 247 | 4 | 19634 | gene;island | 0.319179 | 0.693755 | Not queried | 3.04 | 2.00e-01 | 2.55e-01 |
|  | 17 | 29248604 | 29248954 | PDFSTATSENSEMBLUCSC | 351 | 6 | 18183 | island | 0.595367 | 0.402916 | Not queried | 3.04 | 4.85e-01 | 5.11e-01 |
|  | 17 | 61615504 | 61615913 | PDFSTATSENSEMBLUCSC | 410 | 4 | 16728 | gene;island | 0.452990 | 0.889893 | Not queried | 3.04 | 5.71e-02 | 1.00e-01 |
|  | 5 | 1193724 | 1194126 | PDFSTATSENSEMBLUCSC | 403 | 5 | 32612 | island | 0.609846 | 0.877930 | Not queried | 3.04 | 5.56e-02 | 1.00e-01 |
|  | 8 | 10872604 | 10872819 | PDFSTATSENSEMBLUCSC | 216 | 4 | 39202 | gene;island | 0.035995 | 0.212493 | Not queried | 3.04 | 2.86e-02 | 6.12e-02 |
|  | 6 | 117802401 | 117802786 | PDFSTATSENSEMBLUCSC | 386 | 6 | 35196 | tss;island | 0.983490 | 0.915964 | Not queried | 3.04 | 3.10e-01 | 3.66e-01 |
|  | 2 | 131512651 | 131513216 | PDFSTATSENSEMBLUCSC | 566 | 12 | 23629 | tss;island | 0.582384 | 0.912881 | AMER3; AC140481.8 (margin: AMER3; AC140481.8) | 3.04 | 1.03e-04 | 2.47e-03 |
|  | 6 | 159589874 | 159590231 | PDFSTATSENSEMBLUCSC | 358 | 4 | 35319 | tss;island | 0.809522 | 0.976190 | Not queried | 3.04 | 5.71e-02 | 1.00e-01 |
|  | 11 | 47736132 | 47736463 | PDFSTATSENSEMBLUCSC | 332 | 6 | 8272 | island | 0.120114 | 0.435521 | Not queried | 3.04 | 4.11e-02 | 8.14e-02 |
|  | 17 | 33760230 | 33760527 | PDFSTATSENSEMBLUCSC | 298 | 5 | 17270 | tss | 0.804627 | 0.886063 | Not queried | 3.04 | 1.51e-01 | 2.11e-01 |
|  | 11 | 130786525 | 130786720 | PDFSTATSENSEMBLUCSC | 196 | 6 | 7821 | tss | 0.007689 | 0.060701 | Not queried | 3.04 | 3.10e-01 | 3.66e-01 |
|  | 7 | 123673549 | 123673690 | PDFSTATSENSEMBLUCSC | 142 | 4 | 37873 | tss | 0.634796 | 0.911726 | Not queried | 3.03 | 2.86e-02 | 6.12e-02 |
|  | 16 | 2770211 | 2770429 | PDFSTATSENSEMBLUCSC | 219 | 4 | 14611 | gene;island | 0.516459 | 0.150782 | Not queried | 3.03 | 2.86e-02 | 6.12e-02 |
|  | 11 | 123430575 | 123430949 | PDFSTATSENSEMBLUCSC | 375 | 4 | 6950 | gene | 0.613226 | 0.921292 | Not queried | 3.03 | 1.14e-01 | 1.68e-01 |
|  | 6 | 149772707 | 149772892 | PDFSTATSENSEMBLUCSC | 186 | 4 | 34505 | gene;island | 0.857650 | 0.977271 | Not queried | 3.03 | 2.86e-02 | 6.12e-02 |
|  | 19 | 51171640 | 51172144 | PDFSTATSENSEMBLUCSC | 505 | 5 | 20182 | gene;island | 0.845643 | 0.541729 | Not queried | 3.03 | 5.56e-02 | 1.00e-01 |
|  | 12 | 114841536 | 114842031 | PDFSTATSENSEMBLUCSC | 496 | 8 | 9325 | gene;island | 0.706190 | 0.978781 | TBX5 (margin: TBX5-AS1; TBX5) | 3.03 | 1.86e-03 | 1.45e-02 |
|  | 12 | 114841708 | 114842031 | PDFSTATSENSEMBLUCSC | 324 | 6 | 10032 | tss | 0.706190 | 0.982006 | Not queried | 3.03 | 1.52e-02 | 5.36e-02 |
|  | 12 | 117628347 | 117628589 | PDFSTATSENSEMBLUCSC | 243 | 7 | 10044 | tss | 0.094188 | 0.018580 | Not queried | 3.03 | 2.59e-01 | 3.16e-01 |
|  | 5 | 2008188 | 2008364 | PDFSTATSENSEMBLUCSC | 177 | 4 | 32650 | island | 0.535776 | 0.922647 | Not queried | 3.03 | 2.86e-02 | 6.12e-02 |
|  | 4 | 54965829 | 54966247 | PDFSTATSENSEMBLUCSC | 419 | 6 | 31051 | island | 0.561406 | 0.921350 | FIP1L1; GSX2 (margin: FIP1L1; MORF4L2P1; GSX2; AC110298.1) | 3.03 | 8.66e-03 | 3.36e-02 |
|  | 14 | 58618986 | 58619480 | PDFSTATSENSEMBLUCSC | 495 | 10 | 12420 | tss;island | 0.055845 | 0.007286 | Not queried | 3.03 | 1.47e-02 | 5.36e-02 |
|  | 8 | 37605359 | 37605978 | PDFSTATSENSEMBLUCSC | 620 | 7 | 39279 | gene | 0.906653 | 0.981575 | Not queried | 3.03 | 1.75e-02 | 5.79e-02 |
|  | 2 | 242799312 | 242799562 | PDFSTATSENSEMBLUCSC | 251 | 5 | 23149 | gene;island | 0.457884 | 0.846015 | Not queried | 3.03 | 9.52e-02 | 1.53e-01 |
|  | 16 | 89114613 | 89114919 | PDFSTATSENSEMBLUCSC | 307 | 5 | 16236 | island | 0.562019 | 0.912537 | CTD-2555A7.2 (margin: CTD-2555A7.2) | 3.03 | 7.94e-03 | 3.19e-02 |
|  | 13 | 23489817 | 23490053 | PDFSTATSENSEMBLUCSC | 237 | 4 | 11595 | island | 0.331804 | 0.016086 | Not queried | 3.03 | 2.00e-01 | 2.55e-01 |
|  | 2 | 152830505 | 152830665 | PDFSTATSENSEMBLUCSC | 161 | 5 | 22897 | gene;tss | 0.192677 | 0.616406 | Not queried | 3.03 | 1.59e-02 | 5.36e-02 |
|  | 3 | 23244051 | 23244125 | PDFSTATSENSEMBLUCSC | 75 | 4 | 28183 | tss;island | 0.782158 | 0.334159 | Not queried | 3.03 | 2.86e-02 | 6.12e-02 |
|  | 1 | 4221331 | 4221616 | PDFSTATSENSEMBLUCSC | 286 | 5 | 2582 | island | 0.383737 | 0.705566 | Not queried | 3.03 | 4.21e-01 | 4.59e-01 |
|  | 16 | 87447700 | 87448114 | PDFSTATSENSEMBLUCSC | 415 | 4 | 14890 | gene;island | 0.729892 | 0.974921 | Not queried | 3.02 | 3.43e-01 | 3.84e-01 |
|  | 12 | 132822250 | 132822693 | PDFSTATSENSEMBLUCSC | 444 | 4 | 9436 | gene;island | 0.690326 | 0.943124 | Not queried | 3.02 | 2.00e-01 | 2.55e-01 |
|  | 10 | 72238384 | 72238449 | PDFSTATSENSEMBLUCSC | 66 | 4 | 5136 | tss | 0.701532 | 0.244567 | Not queried | 3.02 | 5.71e-02 | 1.00e-01 |
|  | 4 | 41880280 | 41880747 | PDFSTATSENSEMBLUCSC | 468 | 6 | 31008 | island | 0.772500 | 0.968113 | Not queried | 3.02 | 1.32e-01 | 1.89e-01 |
|  | 8 | 104310713 | 104311085 | PDFSTATSENSEMBLUCSC | 373 | 7 | 39390 | gene | 0.005740 | 0.022281 | Not queried | 3.02 | 3.79e-02 | 7.66e-02 |
|  | 1 | 7023495 | 7023864 | PDFSTATSENSEMBLUCSC | 370 | 4 | 184 | gene;island | 0.646216 | 0.172481 | Not queried | 3.02 | 3.43e-01 | 3.84e-01 |
|  | 5 | 134871462 | 134872315 | PDFSTATSENSEMBLUCSC | 854 | 21 | 33138 | island | 0.641149 | 0.972742 | NEUROG1 (margin: NEUROG1) | 3.02 | 1.23e-05 | 5.38e-04 |
|  | 11 | 125757328 | 125757753 | PDFSTATSENSEMBLUCSC | 426 | 7 | 6960 | gene;island | 0.017704 | 0.005356 | Not queried | 3.02 | 3.18e-01 | 3.74e-01 |
|  | 20 | 61147572 | 61147679 | PDFSTATSENSEMBLUCSC | 108 | 6 | 25487 | gene;island | 0.403879 | 0.602439 | Not queried | 3.02 | 1.32e-01 | 1.89e-01 |
|  | 13 | 27131484 | 27131780 | PDFSTATSENSEMBLUCSC | 297 | 9 | 11355 | tss;island | 0.174481 | 0.011724 | WASF3 (margin: WASF3) | 3.02 | 7.82e-04 | 8.17e-03 |
|  | 2 | 242801896 | 242802192 | PDFSTATSENSEMBLUCSC | 297 | 6 | 23995 | tss;island | 0.661121 | 0.967204 | PDCD1 (margin: CXXC11; PDCD1) | 3.02 | 4.33e-03 | 2.52e-02 |
|  | 8 | 16859295 | 16859671 | PDFSTATSENSEMBLUCSC | 377 | 5 | 39214 | gene | 0.508092 | 0.864101 | Not queried | 3.02 | 3.10e-01 | 3.66e-01 |
|  | 5 | 178487123 | 178488100 | PDFSTATSENSEMBLUCSC | 978 | 14 | 33479 | island | 0.116434 | 0.521014 | ZNF354C (margin: ZNF354C) | 3.02 | 2.96e-03 | 1.88e-02 |
|  | 1 | 156217801 | 156218113 | PDFSTATSENSEMBLUCSC | 313 | 6 | 2012 | tss;island | 0.437706 | 0.813592 | Not queried | 3.02 | 1.52e-02 | 5.36e-02 |
|  | 11 | 18433500 | 18433745 | PDFSTATSENSEMBLUCSC | 246 | 6 | 7184 | tss | 0.880257 | 0.969245 | LDHC (margin: LDHA; AC084117.3; LDHC) | 3.02 | 8.66e-03 | 3.36e-02 |
|  | 22 | 50699649 | 50699947 | PDFSTATSENSEMBLUCSC | 299 | 4 | 26928 | gene;island | 0.006475 | 0.055721 | Not queried | 3.02 | 1.14e-01 | 1.68e-01 |
|  | 5 | 176314231 | 176314737 | PDFSTATSENSEMBLUCSC | 507 | 6 | 31913 | gene;island | 0.506191 | 0.905026 | HK3 (margin: UNC5A; HK3) | 3.02 | 8.66e-03 | 3.36e-02 |
|  | 10 | 49514273 | 49514546 | PDFSTATSENSEMBLUCSC | 274 | 4 | 5724 | island | 0.053826 | 0.008585 | Not queried | 3.02 | 3.43e-01 | 3.84e-01 |
|  | 5 | 52083512 | 52084055 | PDFSTATSENSEMBLUCSC | 544 | 13 | 32058 | tss;island | 0.016522 | 0.114728 | ITGA1; PELO; CTD-2288O8.1 (margin: ITGA1; PELO; CTD-2288O8.1) | 3.02 | 2.35e-03 | 1.53e-02 |
|  | 5 | 92929319 | 92929679 | PDFSTATSENSEMBLUCSC | 361 | 5 | 31690 | gene;island | 0.751177 | 0.982642 | Not queried | 3.02 | 9.52e-02 | 1.53e-01 |
|  | 6 | 33180841 | 33181565 | PDFSTATSENSEMBLUCSC | 725 | 14 | 35985 | island | 0.101880 | 0.436361 | RING1; ZNF70P1 (margin: SLC39A7; HSD17B8; MIR219-1; RING1; ZNF70P1) | 3.02 | 1.18e-03 | 1.14e-02 |
|  | 14 | 31926776 | 31926862 | PDFSTATSENSEMBLUCSC | 87 | 4 | 12342 | tss;island | 0.014033 | 0.009635 | Not queried | 3.02 | 3.43e-01 | 3.84e-01 |
|  | 2 | 233286639 | 233286870 | PDFSTATSENSEMBLUCSC | 232 | 4 | 25193 | island | 0.806165 | 0.974754 | Not queried | 3.01 | 2.86e-02 | 6.12e-02 |
|  | 17 | 79410470 | 79410752 | PDFSTATSENSEMBLUCSC | 283 | 5 | 16909 | gene;island | 0.637046 | 0.960364 | RP11-1055B8.7 (margin: RP11-1055B8.7; MIR3186) | 3.01 | 7.94e-03 | 3.19e-02 |
|  | 14 | 71107931 | 71108199 | PDFSTATSENSEMBLUCSC | 269 | 4 | 12488 | tss;island | 0.022283 | 0.006872 | Not queried | 3.01 | 8.86e-01 | 8.89e-01 |
|  | 5 | 126365232 | 126365876 | PDFSTATSENSEMBLUCSC | 645 | 6 | 31736 | gene;island | 0.109818 | 0.012095 | MARCH3 (margin: MARCH3) | 3.01 | 2.16e-03 | 1.45e-02 |
|  | 2 | 160919147 | 160919532 | PDFSTATSENSEMBLUCSC | 386 | 10 | 23684 | tss | 0.082234 | 0.430195 | PLA2R1 (margin: PLA2R1) | 3.01 | 1.50e-03 | 1.41e-02 |
|  | 16 | 30960055 | 30960483 | PDFSTATSENSEMBLUCSC | 429 | 8 | 15859 | island | 0.109417 | 0.018474 | Not queried | 3.01 | 2.81e-02 | 6.12e-02 |
|  | 4 | 174438930 | 174440103 | PDFSTATSENSEMBLUCSC | 1174 | 10 | 31426 | island | 0.633463 | 0.955764 | Not queried | 3.01 | 2.32e-02 | 6.12e-02 |
|  | 15 | 29409410 | 29409753 | PDFSTATSENSEMBLUCSC | 344 | 5 | 13269 | gene;island | 0.645339 | 0.984663 | Not queried | 3.01 | 9.52e-02 | 1.53e-01 |
|  | 3 | 8543014 | 8543508 | PDFSTATSENSEMBLUCSC | 495 | 8 | 28120 | tss | 0.073365 | 0.007899 | Not queried | 3.01 | 2.07e-02 | 6.12e-02 |
|  | 7 | 55086091 | 55086288 | PDFSTATSENSEMBLUCSC | 198 | 4 | 37603 | tss;island | 0.419492 | 0.794298 | Not queried | 3.01 | 2.86e-02 | 6.12e-02 |
|  | 22 | 46263463 | 46263834 | PDFSTATSENSEMBLUCSC | 372 | 4 | 27578 | island | 0.618233 | 0.920131 | Not queried | 3.01 | 5.71e-02 | 1.00e-01 |
|  | 22 | 19435790 | 19436004 | PDFSTATSENSEMBLUCSC | 215 | 4 | 26958 | tss;island | 0.577621 | 0.088838 | Not queried | 3.01 | 3.43e-01 | 3.84e-01 |
|  | 5 | 175299798 | 175300217 | PDFSTATSENSEMBLUCSC | 420 | 6 | 31909 | gene;island | 0.810584 | 0.309537 | Not queried | 3.01 | 6.49e-02 | 1.11e-01 |
|  | 3 | 46939978 | 46940439 | PDFSTATSENSEMBLUCSC | 462 | 5 | 27758 | gene;island | 0.850327 | 0.985406 | PTH1R (margin: PTH1R; AC109583.1) | 3.01 | 7.94e-03 | 3.19e-02 |
|  | 11 | 9113152 | 9113458 | PDFSTATSENSEMBLUCSC | 307 | 9 | 7132 | tss | 0.106663 | 0.522346 | Not queried | 3.01 | 3.15e-02 | 6.50e-02 |
|  | 6 | 30853948 | 30854233 | PDFSTATSENSEMBLUCSC | 286 | 8 | 33827 | gene;island | 0.013970 | 0.109298 | DDR1 (margin: DDR1; MIR4640; DDR1-AS1) | 3.01 | 1.04e-02 | 3.96e-02 |
|  | 6 | 83072747 | 83072781 | PDFSTATSENSEMBLUCSC | 35 | 4 | 35088 | tss;island | 0.454993 | 0.093139 | Not queried | 3.01 | 2.86e-02 | 6.12e-02 |
|  | 12 | 131443231 | 131443486 | PDFSTATSENSEMBLUCSC | 256 | 6 | 9421 | gene | 0.759767 | 0.971781 | Not queried | 3.01 | 4.11e-02 | 8.14e-02 |
|  | 5 | 140736762 | 140737111 | PDFSTATSENSEMBLUCSC | 350 | 5 | 31821 | gene;island | 0.584983 | 0.899338 | PCDHGA1; PCDHGA2; PCDHGA3; PCDHGB1; PCDHGA4 (margin: PCDHGA1; PCDHGA2; PCDHGA3; PCDHGB1; PCDHGA4; PCDHGB2; PCDHGA5) | 3.01 | 7.94e-03 | 3.19e-02 |
|  | 12 | 129028586 | 129028733 | PDFSTATSENSEMBLUCSC | 148 | 4 | 9408 | gene | 0.593181 | 0.239162 | Not queried | 3.01 | 2.86e-02 | 6.12e-02 |
|  | 1 | 202858691 | 202858826 | PDFSTATSENSEMBLUCSC | 136 | 4 | 2184 | tss;island | 0.984583 | 0.944568 | Not queried | 3.00 | 3.43e-01 | 3.84e-01 |
|  | 17 | 1627652 | 1627862 | PDFSTATSENSEMBLUCSC | 211 | 7 | 16320 | gene;tss;island | 0.279942 | 0.019005 | WDR81; RP11-961A15.1 (margin: WDR81; MIR22HG; RP11-961A15.1) | 3.00 | 1.11e-02 | 4.14e-02 |
|  | 2 | 27341795 | 27341901 | PDFSTATSENSEMBLUCSC | 107 | 4 | 22620 | gene;tss;island | 0.087807 | 0.013132 | Not queried | 3.00 | 2.00e-01 | 2.55e-01 |
|  | 17 | 76967629 | 76967825 | PDFSTATSENSEMBLUCSC | 197 | 4 | 16830 | gene | 0.627697 | 0.263061 | Not queried | 3.00 | 3.43e-01 | 3.84e-01 |
|  | 11 | 65188784 | 65189075 | PDFSTATSENSEMBLUCSC | 292 | 4 | 7428 | tss;island | 0.002243 | 0.042861 | Not queried | 3.00 | 2.00e-01 | 2.55e-01 |
|  | 1 | 231296640 | 231297103 | PDFSTATSENSEMBLUCSC | 464 | 5 | 4323 | island | 0.832683 | 0.974919 | TRIM67 (margin: TRIM67) | 3.00 | 7.94e-03 | 3.19e-02 |
|  | 20 | 327607 | 327811 | PDFSTATSENSEMBLUCSC | 205 | 4 | 25321 | gene;island | 0.002228 | 0.018031 | Not queried | 3.00 | 2.00e-01 | 2.55e-01 |
|  | 3 | 181429233 | 181429704 | PDFSTATSENSEMBLUCSC | 472 | 9 | 28042 | gene;tss;island | 0.030949 | 0.006170 | SOX2-OT; SOX2 (margin: SOX2-OT; SOX2) | 3.00 | 1.06e-02 | 4.01e-02 |
|  | 6 | 72595944 | 72596748 | PDFSTATSENSEMBLUCSC | 805 | 10 | 36245 | island | 0.636975 | 0.444484 | Not queried | 3.00 | 1.47e-02 | 5.36e-02 |
|  | 11 | 116371188 | 116371487 | PDFSTATSENSEMBLUCSC | 300 | 4 | 8832 | island | 0.730646 | 0.972805 | Not queried | 3.00 | 1.14e-01 | 1.68e-01 |
|  | 5 | 161494711 | 161494810 | PDFSTATSENSEMBLUCSC | 100 | 4 | 31881 | gene | 0.075931 | 0.039973 | Not queried | 3.00 | 4.86e-01 | 5.11e-01 |
|  | 2 | 74875227 | 74875548 | PDFSTATSENSEMBLUCSC | 322 | 8 | 23465 | tss;island | 0.881241 | 0.986365 | M1AP (margin: SEMA4F; M1AP) | 3.00 | 1.55e-04 | 2.71e-03 |
|  | 10 | 131479182 | 131479437 | PDFSTATSENSEMBLUCSC | 256 | 4 | 4829 | gene | 0.831115 | 0.976504 | Not queried | 3.00 | 2.86e-02 | 6.12e-02 |
|  | 2 | 113299370 | 113299708 | PDFSTATSENSEMBLUCSC | 339 | 4 | 24623 | island | 0.150503 | 0.004169 | Not queried | 3.00 | 4.86e-01 | 5.11e-01 |
|  | 12 | 72667326 | 72667707 | PDFSTATSENSEMBLUCSC | 382 | 7 | 9877 | tss | 0.221887 | 0.698764 | TRHDE; TRHDE-AS1 (margin: TRHDE; TRHDE-AS1) | 2.99 | 5.83e-04 | 6.47e-03 |
|  | 19 | 6463949 | 6464275 | PDFSTATSENSEMBLUCSC | 327 | 10 | 21430 | island | 0.190096 | 0.013486 | Not queried | 2.99 | 2.88e-02 | 6.16e-02 |
|  | 19 | 56151972 | 56152354 | PDFSTATSENSEMBLUCSC | 383 | 6 | 21085 | tss;island | 0.168679 | 0.034076 | Not queried | 2.99 | 1.80e-01 | 2.45e-01 |
|  | 5 | 161494274 | 161494645 | PDFSTATSENSEMBLUCSC | 372 | 6 | 32473 | tss | 0.362603 | 0.086256 | Not queried | 2.99 | 1.80e-01 | 2.45e-01 |
|  | 19 | 39894559 | 39894767 | PDFSTATSENSEMBLUCSC | 209 | 4 | 21993 | island | 0.008391 | 0.028219 | Not queried | 2.99 | 1.14e-01 | 1.68e-01 |
|  | 2 | 74741647 | 74741941 | PDFSTATSENSEMBLUCSC | 295 | 4 | 22746 | gene | 0.529217 | 0.895071 | Not queried | 2.99 | 2.86e-02 | 6.12e-02 |
|  | 6 | 10415597 | 10416090 | PDFSTATSENSEMBLUCSC | 494 | 11 | 33561 | gene;tss;island | 0.007460 | 0.035321 | Not queried | 2.99 | 5.57e-02 | 1.00e-01 |
|  | 20 | 23471627 | 23471737 | PDFSTATSENSEMBLUCSC | 111 | 4 | 25640 | tss | 0.656904 | 0.957934 | Not queried | 2.99 | 5.71e-02 | 1.00e-01 |
|  | 19 | 54041163 | 54041329 | PDFSTATSENSEMBLUCSC | 167 | 5 | 21031 | tss | 0.810397 | 0.421753 | ZNF331 (margin: ZNF331) | 2.99 | 7.94e-03 | 3.19e-02 |
|  | 1 | 39044140 | 39044452 | PDFSTATSENSEMBLUCSC | 313 | 5 | 3088 | island | 0.792008 | 0.964621 | Not queried | 2.99 | 1.51e-01 | 2.11e-01 |
|  | 17 | 4463911 | 4464075 | PDFSTATSENSEMBLUCSC | 165 | 5 | 17025 | tss | 0.122263 | 0.020986 | Not queried | 2.99 | 1.59e-02 | 5.36e-02 |
|  | 3 | 187387555 | 187388148 | PDFSTATSENSEMBLUCSC | 594 | 8 | 28077 | gene | 0.817850 | 0.450060 | Not queried | 2.99 | 2.79e-01 | 3.38e-01 |
|  | 14 | 65347138 | 65347518 | PDFSTATSENSEMBLUCSC | 381 | 4 | 12943 | island | 0.316071 | 0.033556 | Not queried | 2.99 | 2.00e-01 | 2.55e-01 |
|  | 4 | 76555547 | 76556042 | PDFSTATSENSEMBLUCSC | 496 | 11 | 31107 | island | 0.079013 | 0.449509 | CDKL2 (margin: CDKL2; G3BP2) | 2.98 | 2.84e-06 | 1.96e-04 |
|  | 3 | 153838788 | 153839287 | PDFSTATSENSEMBLUCSC | 500 | 8 | 29599 | island | 0.268477 | 0.011175 | ARHGEF26; ARHGEF26-AS1 (margin: ARHGEF26; ARHGEF26-AS1) | 2.98 | 6.99e-03 | 3.19e-02 |
|  | 18 | 7011217 | 7011463 | PDFSTATSENSEMBLUCSC | 247 | 4 | 19051 | gene;island | 0.320876 | 0.641914 | Not queried | 2.98 | 3.43e-01 | 3.84e-01 |
|  | 10 | 98802990 | 98803335 | PDFSTATSENSEMBLUCSC | 346 | 4 | 4691 | gene;island | 0.692856 | 0.983680 | Not queried | 2.98 | 1.14e-01 | 1.68e-01 |
|  | 1 | 221057558 | 221057808 | PDFSTATSENSEMBLUCSC | 251 | 5 | 1022 | gene;island | 0.704633 | 0.966047 | Not queried | 2.98 | 3.10e-01 | 3.66e-01 |
|  | 5 | 155754009 | 155754265 | PDFSTATSENSEMBLUCSC | 257 | 4 | 31871 | gene | 0.059419 | 0.388014 | Not queried | 2.98 | 3.43e-01 | 3.84e-01 |
|  | 3 | 124931627 | 124931746 | PDFSTATSENSEMBLUCSC | 120 | 4 | 28550 | tss | 0.168479 | 0.506115 | Not queried | 2.98 | 2.86e-02 | 6.12e-02 |
|  | 6 | 32634245 | 32634362 | PDFSTATSENSEMBLUCSC | 118 | 5 | 34127 | gene | 0.247845 | 0.031846 | Not queried | 2.98 | 9.52e-02 | 1.53e-01 |
|  | 22 | 50493126 | 50493212 | PDFSTATSENSEMBLUCSC | 87 | 4 | 27243 | tss | 0.816127 | 0.985445 | Not queried | 2.98 | 5.71e-02 | 1.00e-01 |
|  | 10 | 133796164 | 133796559 | PDFSTATSENSEMBLUCSC | 396 | 6 | 5444 | tss;island | 0.666428 | 0.972817 | Not queried | 2.98 | 6.49e-02 | 1.11e-01 |
|  | 15 | 73925796 | 73926058 | PDFSTATSENSEMBLUCSC | 263 | 4 | 13783 | tss;island | 0.003677 | 0.034475 | Not queried | 2.98 | 3.43e-01 | 3.84e-01 |
|  | 11 | 74459620 | 74459861 | PDFSTATSENSEMBLUCSC | 242 | 7 | 7562 | tss | 0.003658 | 0.012680 | RNF169 (margin: RNF169; RP11-864N7.1; RP11-864N7.2; AP000560.1) | 2.98 | 6.99e-03 | 3.19e-02 |
|  | 7 | 157414294 | 157414580 | PDFSTATSENSEMBLUCSC | 287 | 4 | 37333 | gene;island | 0.798286 | 0.977102 | Not queried | 2.98 | 5.71e-02 | 1.00e-01 |
|  | 20 | 55101001 | 55101089 | PDFSTATSENSEMBLUCSC | 89 | 4 | 25837 | tss | 0.571316 | 0.896945 | Not queried | 2.98 | 2.00e-01 | 2.55e-01 |
|  | 15 | 40545143 | 40545511 | PDFSTATSENSEMBLUCSC | 369 | 9 | 13585 | tss | 0.794845 | 0.968249 | RP11-133K1.2; PAK6; C15orf56 (margin: RP11-133K1.2; PAK6; C15orf56) | 2.98 | 1.65e-04 | 2.80e-03 |
|  | 2 | 66809780 | 66809985 | PDFSTATSENSEMBLUCSC | 206 | 4 | 24358 | island | 0.952557 | 0.812170 | Not queried | 2.98 | 2.00e-01 | 2.55e-01 |
|  | 2 | 50574912 | 50575098 | PDFSTATSENSEMBLUCSC | 187 | 6 | 22676 | gene;tss;island | 0.550038 | 0.114893 | Not queried | 2.98 | 2.60e-02 | 6.12e-02 |
|  | 1 | 1115378 | 1115920 | PDFSTATSENSEMBLUCSC | 543 | 5 | 19 | gene;island | 0.872427 | 0.982155 | TTLL10; TTLL10-AS1 (margin: MIR429; RP11-465B22.8; TTLL10; TTLL10-AS1) | 2.98 | 7.94e-03 | 3.19e-02 |
|  | 5 | 1282984 | 1283350 | PDFSTATSENSEMBLUCSC | 367 | 4 | 31533 | gene;island | 0.589994 | 0.924020 | Not queried | 2.98 | 5.71e-02 | 1.00e-01 |
|  | 11 | 46957758 | 46958188 | PDFSTATSENSEMBLUCSC | 431 | 8 | 7283 | tss | 0.062474 | 0.009154 | Not queried | 2.98 | 1.30e-01 | 1.89e-01 |
|  | 17 | 74533845 | 74534090 | PDFSTATSENSEMBLUCSC | 246 | 7 | 17681 | tss;island | 0.919732 | 0.982989 | PRCD; CYGB (margin: PRCD; RP11-666A8.7; CYGB; RP11-666A8.8) | 2.98 | 5.83e-04 | 6.47e-03 |
|  | 17 | 79109729 | 79110023 | PDFSTATSENSEMBLUCSC | 295 | 5 | 16897 | gene;island | 0.677922 | 0.859962 | Not queried | 2.98 | 1.51e-01 | 2.11e-01 |
|  | 17 | 79895357 | 79895584 | PDFSTATSENSEMBLUCSC | 228 | 5 | 17761 | tss;island | 0.020612 | 0.025092 | Not queried | 2.98 | 5.48e-01 | 5.70e-01 |
|  | 17 | 16394618 | 16394899 | PDFSTATSENSEMBLUCSC | 282 | 4 | 16460 | gene;island | 0.328236 | 0.070668 | Not queried | 2.98 | 3.43e-01 | 3.84e-01 |
|  | 3 | 16926159 | 16926350 | PDFSTATSENSEMBLUCSC | 192 | 5 | 28178 | tss;island | 0.138233 | 0.007823 | Not queried | 2.98 | 1.51e-01 | 2.11e-01 |
|  | 6 | 44186914 | 44187226 | PDFSTATSENSEMBLUCSC | 313 | 9 | 35007 | tss | 0.844003 | 0.345616 | RP1-302G2.5; SLC29A1 (margin: RP1-302G2.5; SLC29A1) | 2.97 | 1.65e-04 | 2.80e-03 |
|  | 5 | 1298511 | 1298965 | PDFSTATSENSEMBLUCSC | 455 | 6 | 32625 | island | 0.426645 | 0.862761 | Not queried | 2.97 | 2.40e-01 | 2.95e-01 |
|  | 1 | 15271830 | 15272326 | PDFSTATSENSEMBLUCSC | 497 | 6 | 241 | gene;tss | 0.548713 | 0.940837 | KAZN (margin: KAZN) | 2.97 | 2.16e-03 | 1.45e-02 |
|  | 11 | 1031856 | 1032316 | PDFSTATSENSEMBLUCSC | 461 | 5 | 6401 | gene;island | 0.356712 | 0.937741 | Not queried | 2.97 | 1.51e-01 | 2.11e-01 |
|  | 8 | 67090752 | 67090928 | PDFSTATSENSEMBLUCSC | 177 | 5 | 39740 | tss | 0.718212 | 0.974691 | Not queried | 2.97 | 5.56e-02 | 1.00e-01 |
|  | 19 | 54041163 | 54041398 | PDFSTATSENSEMBLUCSC | 236 | 6 | 20206 | gene;island | 0.802056 | 0.429679 | ZNF331 (margin: ZNF331) | 2.97 | 2.16e-03 | 1.45e-02 |
|  | 12 | 130961424 | 130961986 | PDFSTATSENSEMBLUCSC | 563 | 8 | 9417 | gene | 0.080575 | 0.379663 | Not queried | 2.97 | 1.95e-01 | 2.55e-01 |
|  | 3 | 44626029 | 44626860 | PDFSTATSENSEMBLUCSC | 832 | 11 | 29036 | island | 0.204365 | 0.735496 | Not queried | 2.97 | 3.36e-02 | 6.87e-02 |
|  | 13 | 95357638 | 95357832 | PDFSTATSENSEMBLUCSC | 195 | 4 | 11821 | island | 0.814607 | 0.976686 | Not queried | 2.97 | 4.86e-01 | 5.11e-01 |
|  | 1 | 205425468 | 205425944 | PDFSTATSENSEMBLUCSC | 477 | 5 | 4128 | island | 0.529758 | 0.917859 | RP11-576D8.4; LEMD1 (margin: RP11-576D8.4; LEMD1; MIR135B) | 2.97 | 7.94e-03 | 3.19e-02 |
|  | 11 | 107798913 | 107799237 | PDFSTATSENSEMBLUCSC | 325 | 9 | 7666 | tss | 0.311595 | 0.029098 | RAB39A; SLC35F2 (margin: RAB39A; SLC35F2) | 2.97 | 1.23e-03 | 1.18e-02 |
|  | 11 | 22214333 | 22214726 | PDFSTATSENSEMBLUCSC | 394 | 6 | 8132 | island | 0.687984 | 0.157139 | Not queried | 2.97 | 9.31e-02 | 1.53e-01 |
|  | 4 | 74718978 | 74719289 | PDFSTATSENSEMBLUCSC | 312 | 5 | 31096 | island | 0.915464 | 0.581288 | PF4V1 (margin: CXCL6; PPBPP1; PF4V1) | 2.97 | 7.94e-03 | 3.19e-02 |
|  | 1 | 27021704 | 27021879 | PDFSTATSENSEMBLUCSC | 176 | 5 | 1395 | tss;island | 0.009658 | 0.033626 | Not queried | 2.97 | 5.56e-02 | 1.00e-01 |
|  | 3 | 145878963 | 145879710 | PDFSTATSENSEMBLUCSC | 748 | 10 | 29562 | island | 0.030675 | 0.456042 | Not queried | 2.96 | 2.88e-02 | 6.16e-02 |
|  | 1 | 202311100 | 202311278 | PDFSTATSENSEMBLUCSC | 179 | 6 | 2178 | tss | 0.000719 | 0.003881 | Not queried | 2.96 | 3.10e-01 | 3.66e-01 |
|  | 19 | 44645459 | 44645662 | PDFSTATSENSEMBLUCSC | 204 | 5 | 20844 | tss | 0.011293 | 0.006942 | Not queried | 2.96 | 6.90e-01 | 7.01e-01 |
|  | 1 | 203055422 | 203055662 | PDFSTATSENSEMBLUCSC | 241 | 4 | 2189 | tss | 0.412102 | 0.851253 | Not queried | 2.96 | 5.71e-02 | 1.00e-01 |
|  | 19 | 21106002 | 21106053 | PDFSTATSENSEMBLUCSC | 52 | 6 | 20657 | tss | 0.752598 | 0.285301 | ZNF85 (margin: ZNF85) | 2.96 | 2.16e-03 | 1.45e-02 |
|  | 19 | 17357097 | 17357641 | PDFSTATSENSEMBLUCSC | 545 | 7 | 20582 | tss;island | 0.018448 | 0.223927 | Not queried | 2.96 | 3.18e-01 | 3.74e-01 |
|  | 2 | 69871531 | 69871759 | PDFSTATSENSEMBLUCSC | 229 | 4 | 23407 | tss;island | 0.003196 | 0.007587 | Not queried | 2.96 | 2.00e-01 | 2.55e-01 |
|  | 7 | 22894795 | 22894898 | PDFSTATSENSEMBLUCSC | 104 | 4 | 37467 | tss;island | 0.001047 | 0.024081 | Not queried | 2.96 | 4.86e-01 | 5.11e-01 |
|  | 2 | 177028606 | 177028804 | PDFSTATSENSEMBLUCSC | 199 | 5 | 24912 | island | 0.461351 | 0.890154 | Not queried | 2.96 | 1.51e-01 | 2.11e-01 |
|  | 12 | 52626814 | 52626904 | PDFSTATSENSEMBLUCSC | 91 | 5 | 9725 | tss | 0.840879 | 0.981483 | KRT7 (margin: LINC00592; KRT7; METTL7AP1; RP3-416H24.1) | 2.96 | 7.94e-03 | 3.19e-02 |
|  | 11 | 118016692 | 118017101 | PDFSTATSENSEMBLUCSC | 410 | 5 | 6925 | gene;tss;island | 0.457176 | 0.104069 | Not queried | 2.96 | 2.22e-01 | 2.76e-01 |
|  | 5 | 43000303 | 43000890 | PDFSTATSENSEMBLUCSC | 588 | 6 | 32764 | island | 0.433915 | 0.060686 | Not queried | 2.96 | 2.60e-02 | 6.12e-02 |
|  | 4 | 126237325 | 126237597 | PDFSTATSENSEMBLUCSC | 273 | 7 | 31296 | island | 0.805604 | 0.481169 | Not queried | 2.96 | 2.62e-02 | 6.12e-02 |
|  | 13 | 39260874 | 39261169 | PDFSTATSENSEMBLUCSC | 296 | 7 | 11403 | tss;island | 0.492556 | 0.085023 | Not queried | 2.96 | 7.28e-02 | 1.23e-01 |
|  | 14 | 61748243 | 61748440 | PDFSTATSENSEMBLUCSC | 198 | 4 | 12105 | gene;island | 0.097694 | 0.016006 | Not queried | 2.96 | 2.86e-02 | 6.12e-02 |
|  | 6 | 161188022 | 161188822 | PDFSTATSENSEMBLUCSC | 801 | 8 | 36615 | island | 0.670357 | 0.934106 | (margin: ) | 2.96 | 6.99e-03 | 3.19e-02 |
|  | 5 | 24644988 | 24645147 | PDFSTATSENSEMBLUCSC | 160 | 5 | 32002 | tss | 0.334124 | 0.038743 | CDH10 (margin: CDH10) | 2.96 | 7.94e-03 | 3.19e-02 |
|  | 22 | 19949873 | 19950166 | PDFSTATSENSEMBLUCSC | 294 | 7 | 26794 | gene | 0.135848 | 0.021547 | Not queried | 2.96 | 3.79e-02 | 7.66e-02 |
|  | 1 | 47010056 | 47010223 | PDFSTATSENSEMBLUCSC | 168 | 4 | 3221 | island | 0.634217 | 0.949258 | Not queried | 2.96 | 1.14e-01 | 1.68e-01 |
|  | 4 | 54965829 | 54966187 | PDFSTATSENSEMBLUCSC | 359 | 5 | 30399 | tss | 0.610516 | 0.922536 | Not queried | 2.95 | 3.17e-02 | 6.50e-02 |
|  | 1 | 91185028 | 91185441 | PDFSTATSENSEMBLUCSC | 414 | 6 | 3460 | island | 0.866283 | 0.985072 | Not queried | 2.95 | 4.11e-02 | 8.14e-02 |
|  | 6 | 35887678 | 35887853 | PDFSTATSENSEMBLUCSC | 176 | 4 | 34311 | gene;island | 0.001542 | 0.007740 | Not queried | 2.95 | 3.43e-01 | 3.84e-01 |
|  | 1 | 185703201 | 185703877 | PDFSTATSENSEMBLUCSC | 677 | 13 | 4045 | island | 0.158705 | 0.017050 | HMCN1 (margin: HMCN1) | 2.95 | 2.28e-04 | 3.75e-03 |
|  | 1 | 185703201 | 185703659 | PDFSTATSENSEMBLUCSC | 459 | 11 | 2144 | tss | 0.158705 | 0.017050 | HMCN1 (margin: HMCN1) | 2.95 | 5.53e-04 | 6.47e-03 |
|  | 1 | 184006765 | 184007101 | PDFSTATSENSEMBLUCSC | 337 | 5 | 4037 | island | 0.459329 | 0.800167 | Not queried | 2.95 | 1.51e-01 | 2.11e-01 |
|  | 2 | 223176606 | 223177008 | PDFSTATSENSEMBLUCSC | 403 | 5 | 25150 | island | 0.899745 | 0.976073 | (margin: CCDC140; AC010980.2; RP11-384O8.1) | 2.95 | 7.94e-03 | 3.19e-02 |
|  | 1 | 21996199 | 21996376 | PDFSTATSENSEMBLUCSC | 178 | 4 | 1326 | tss;island | 0.847599 | 0.962108 | Not queried | 2.95 | 5.71e-02 | 1.00e-01 |
|  | 20 | 23402051 | 23402404 | PDFSTATSENSEMBLUCSC | 354 | 10 | 26051 | island | 0.001629 | 0.004748 | NAPB (margin: NAPB) | 2.95 | 2.88e-03 | 1.86e-02 |
|  | 12 | 39299132 | 39299844 | PDFSTATSENSEMBLUCSC | 713 | 9 | 10325 | island | 0.450830 | 0.897062 | RP11-396F22.1; CPNE8 (margin: RP11-396F22.1; CPNE8; AC067735.1) | 2.95 | 4.11e-05 | 1.17e-03 |
|  | 6 | 168122607 | 168122963 | PDFSTATSENSEMBLUCSC | 357 | 6 | 36647 | island | 0.794297 | 0.982356 | Not queried | 2.95 | 5.89e-01 | 6.11e-01 |
|  | 8 | 101661767 | 101662246 | PDFSTATSENSEMBLUCSC | 480 | 9 | 40498 | island | 0.879867 | 0.978901 | SNX31 (margin: SNX31) | 2.95 | 7.82e-04 | 8.17e-03 |
|  | 11 | 1083178 | 1083509 | PDFSTATSENSEMBLUCSC | 332 | 6 | 6402 | gene;island | 0.760257 | 0.958687 | MUC2 (margin: MUC2) | 2.95 | 4.33e-03 | 2.52e-02 |
|  | 7 | 30722163 | 30722433 | PDFSTATSENSEMBLUCSC | 271 | 7 | 37520 | tss | 0.397546 | 0.894211 | Not queried | 2.95 | 2.59e-01 | 3.16e-01 |
|  | 15 | 100890168 | 100890477 | PDFSTATSENSEMBLUCSC | 310 | 8 | 13505 | gene;island | 0.035236 | 0.019183 | Not queried | 2.95 | 1.95e-01 | 2.55e-01 |
|  | 2 | 197458408 | 197458695 | PDFSTATSENSEMBLUCSC | 288 | 4 | 23795 | tss;island | 0.720769 | 0.307347 | Not queried | 2.95 | 2.00e-01 | 2.55e-01 |
|  | 15 | 68723690 | 68724065 | PDFSTATSENSEMBLUCSC | 376 | 4 | 13396 | gene;island | 0.276466 | 0.719484 | Not queried | 2.95 | 2.86e-02 | 6.12e-02 |
|  | 19 | 3801294 | 3801922 | PDFSTATSENSEMBLUCSC | 629 | 7 | 21349 | island | 0.214323 | 0.020355 | Not queried | 2.95 | 2.09e-01 | 2.65e-01 |
|  | 6 | 46703193 | 46703670 | PDFSTATSENSEMBLUCSC | 478 | 15 | 35022 | tss | 0.463165 | 0.930078 | PLA2G7 (margin: ANKRD66; PLA2G7) | 2.95 | 8.98e-05 | 2.18e-03 |
|  | 8 | 145026305 | 145026613 | PDFSTATSENSEMBLUCSC | 309 | 4 | 39503 | gene;island | 0.004998 | 0.021336 | Not queried | 2.94 | 2.00e-01 | 2.55e-01 |
|  | 19 | 46996347 | 46996868 | PDFSTATSENSEMBLUCSC | 522 | 4 | 20110 | gene;island | 0.845255 | 0.984830 | Not queried | 2.94 | 1.14e-01 | 1.68e-01 |
|  | 8 | 82192519 | 82192931 | PDFSTATSENSEMBLUCSC | 413 | 10 | 40417 | island | 0.102944 | 0.433480 | FABP5; RP11-363E6.3 (margin: FABP5; RP11-363E6.4; RP11-363E6.3) | 2.94 | 1.08e-05 | 4.82e-04 |
|  | 13 | 36429936 | 36430134 | PDFSTATSENSEMBLUCSC | 199 | 4 | 11128 | gene | 0.017633 | 0.069376 | Not queried | 2.94 | 2.86e-02 | 6.12e-02 |
|  | 6 | 32362551 | 32362744 | PDFSTATSENSEMBLUCSC | 194 | 8 | 34104 | gene | 0.464471 | 0.896732 | Not queried | 2.94 | 2.79e-01 | 3.38e-01 |
|  | 6 | 90025032 | 90025307 | PDFSTATSENSEMBLUCSC | 276 | 4 | 35118 | tss | 0.822986 | 0.977053 | Not queried | 2.94 | 2.86e-02 | 6.12e-02 |
|  | 6 | 134158982 | 134159412 | PDFSTATSENSEMBLUCSC | 431 | 5 | 34478 | gene;island | 0.028830 | 0.003571 | Not queried | 2.94 | 1.51e-01 | 2.11e-01 |
|  | 6 | 168479622 | 168480013 | PDFSTATSENSEMBLUCSC | 392 | 7 | 36658 | island | 0.604457 | 0.915151 | Not queried | 2.94 | 1.75e-02 | 5.79e-02 |
|  | 10 | 102985022 | 102985514 | PDFSTATSENSEMBLUCSC | 493 | 4 | 6058 | island | 0.319103 | 0.814345 | Not queried | 2.94 | 2.86e-02 | 6.12e-02 |
|  | 16 | 875881 | 876216 | PDFSTATSENSEMBLUCSC | 336 | 4 | 15492 | island | 0.843754 | 0.974441 | Not queried | 2.94 | 1.14e-01 | 1.68e-01 |
|  | 2 | 162272107 | 162272604 | PDFSTATSENSEMBLUCSC | 498 | 6 | 23687 | tss | 0.685290 | 0.476550 | Not queried | 2.94 | 1.32e-01 | 1.89e-01 |
|  | 17 | 58469727 | 58470033 | PDFSTATSENSEMBLUCSC | 307 | 4 | 17551 | tss;island | 0.004037 | 0.019716 | Not queried | 2.94 | 1.14e-01 | 1.68e-01 |
|  | 5 | 218988 | 219334 | PDFSTATSENSEMBLUCSC | 347 | 4 | 31499 | gene;tss;island | 0.005715 | 0.036665 | Not queried | 2.94 | 2.00e-01 | 2.55e-01 |
|  | 7 | 112758734 | 112758954 | PDFSTATSENSEMBLUCSC | 221 | 6 | 37846 | tss | 0.009114 | 0.004368 | Not queried | 2.94 | 2.40e-01 | 2.95e-01 |
|  | 6 | 169026928 | 169027218 | PDFSTATSENSEMBLUCSC | 291 | 5 | 34565 | gene | 0.861789 | 0.971820 | SMOC2 (margin: SMOC2) | 2.94 | 7.94e-03 | 3.19e-02 |
|  | 19 | 51774377 | 51774666 | PDFSTATSENSEMBLUCSC | 290 | 5 | 22339 | island | 0.341178 | 0.927398 | Not queried | 2.94 | 2.22e-01 | 2.76e-01 |
|  | 11 | 116694114 | 116694372 | PDFSTATSENSEMBLUCSC | 259 | 5 | 7712 | tss | 0.494769 | 0.895943 | Not queried | 2.94 | 3.17e-02 | 6.50e-02 |
|  | 8 | 122654023 | 122654328 | PDFSTATSENSEMBLUCSC | 306 | 4 | 39415 | gene;tss;island | 0.005704 | 0.001604 | Not queried | 2.94 | 1.14e-01 | 1.68e-01 |
|  | 3 | 44626029 | 44626453 | PDFSTATSENSEMBLUCSC | 425 | 7 | 28257 | tss | 0.196329 | 0.667641 | Not queried | 2.94 | 1.28e-01 | 1.87e-01 |
|  | 17 | 6735063 | 6735316 | PDFSTATSENSEMBLUCSC | 254 | 8 | 17054 | tss | 0.139902 | 0.020322 | TEKT1 (margin: TEKT1) | 2.94 | 4.66e-03 | 2.68e-02 |
|  | 4 | 1052372 | 1052717 | PDFSTATSENSEMBLUCSC | 346 | 4 | 30766 | island | 0.522362 | 0.789158 | Not queried | 2.94 | 2.00e-01 | 2.55e-01 |
|  | 16 | 84328104 | 84328391 | PDFSTATSENSEMBLUCSC | 288 | 6 | 15392 | tss;island | 0.139590 | 0.599424 | WFDC1; RP11-558A11.2 (margin: WFDC1; RP11-558A11.2) | 2.94 | 8.66e-03 | 3.36e-02 |
|  | 8 | 145008909 | 145009406 | PDFSTATSENSEMBLUCSC | 498 | 5 | 39497 | gene;island | 0.857230 | 0.953334 | Not queried | 2.94 | 3.17e-02 | 6.50e-02 |
|  | 8 | 110987530 | 110988444 | PDFSTATSENSEMBLUCSC | 915 | 9 | 39875 | tss;island | 0.243737 | 0.011428 | Not queried | 2.94 | 1.42e-02 | 5.24e-02 |
|  | 6 | 33156779 | 33157222 | PDFSTATSENSEMBLUCSC | 444 | 10 | 34213 | gene;island | 0.377141 | 0.835403 | COL11A2 (margin: RNY4P10; SLC39A7; COL11A2; RXRB) | 2.94 | 8.93e-03 | 3.45e-02 |
|  | 17 | 51899837 | 51900115 | PDFSTATSENSEMBLUCSC | 279 | 6 | 17516 | tss | 0.459505 | 0.083000 | Not queried | 2.93 | 4.11e-02 | 8.14e-02 |
|  | 2 | 27256009 | 27256285 | PDFSTATSENSEMBLUCSC | 277 | 4 | 22619 | gene;island | 0.001410 | 0.005097 | Not queried | 2.93 | 3.43e-01 | 3.84e-01 |
|  | 2 | 228736089 | 228736324 | PDFSTATSENSEMBLUCSC | 236 | 6 | 23932 | tss | 0.902142 | 0.979305 | Not queried | 2.93 | 6.49e-02 | 1.11e-01 |
|  | 13 | 20735819 | 20736342 | PDFSTATSENSEMBLUCSC | 524 | 8 | 11328 | tss;island | 0.809150 | 0.962418 | Not queried | 2.93 | 1.05e-01 | 1.66e-01 |
|  | 4 | 4250464 | 4250746 | PDFSTATSENSEMBLUCSC | 283 | 4 | 30265 | tss;island | 0.039294 | 0.118115 | Not queried | 2.93 | 8.86e-01 | 8.89e-01 |
|  | 15 | 72104228 | 72104417 | PDFSTATSENSEMBLUCSC | 190 | 4 | 13404 | gene;island | 0.694352 | 0.941076 | Not queried | 2.93 | 3.43e-01 | 3.84e-01 |
|  | 3 | 87040269 | 87040739 | PDFSTATSENSEMBLUCSC | 471 | 8 | 28451 | tss | 0.005646 | 0.024906 | Not queried | 2.93 | 1.05e-01 | 1.66e-01 |
|  | 17 | 73150420 | 73151149 | PDFSTATSENSEMBLUCSC | 730 | 8 | 18741 | island | 0.005002 | 0.023394 | Not queried | 2.93 | 3.82e-01 | 4.27e-01 |
|  | 1 | 917642 | 917892 | PDFSTATSENSEMBLUCSC | 251 | 5 | 1136 | tss;island | 0.330632 | 0.790162 | Not queried | 2.93 | 9.52e-02 | 1.53e-01 |
|  | 15 | 45479977 | 45480196 | PDFSTATSENSEMBLUCSC | 220 | 4 | 13330 | gene;island | 0.348530 | 0.030802 | Not queried | 2.93 | 1.14e-01 | 1.68e-01 |
|  | 11 | 113953462 | 113953838 | PDFSTATSENSEMBLUCSC | 377 | 4 | 6910 | gene;island | 0.536027 | 0.950693 | Not queried | 2.93 | 2.00e-01 | 2.55e-01 |
|  | 7 | 4308120 | 4308449 | PDFSTATSENSEMBLUCSC | 330 | 6 | 36839 | gene;island | 0.399663 | 0.763200 | Not queried | 2.93 | 1.32e-01 | 1.89e-01 |
|  | 1 | 84543369 | 84543558 | PDFSTATSENSEMBLUCSC | 190 | 5 | 1712 | tss;island | 0.026224 | 0.010765 | Not queried | 2.93 | 2.22e-01 | 2.76e-01 |
|  | 3 | 2139977 | 2140256 | PDFSTATSENSEMBLUCSC | 280 | 4 | 28832 | island | 0.911913 | 0.580331 | Not queried | 2.93 | 1.14e-01 | 1.68e-01 |
|  | 1 | 45672383 | 45672848 | PDFSTATSENSEMBLUCSC | 466 | 8 | 1581 | tss;island | 0.065808 | 0.006034 | Not queried | 2.93 | 2.81e-02 | 6.12e-02 |
|  | 8 | 102504447 | 102504859 | PDFSTATSENSEMBLUCSC | 413 | 9 | 40506 | island | 0.043483 | 0.005669 | GRHL2; KB-1562D12.1 (margin: GRHL2; KB-1562D12.1) | 2.93 | 3.99e-03 | 2.47e-02 |
|  | 8 | 102504447 | 102504666 | PDFSTATSENSEMBLUCSC | 220 | 7 | 39850 | tss | 0.043483 | 0.005669 | Not queried | 2.93 | 1.75e-02 | 5.79e-02 |
|  | 11 | 67777618 | 67777952 | PDFSTATSENSEMBLUCSC | 335 | 8 | 6766 | gene | 0.015284 | 0.111954 | ALDH3B1 (margin: ALDH3B1; UNC93B1) | 2.93 | 3.11e-04 | 4.77e-03 |
|  | 3 | 30936070 | 30936531 | PDFSTATSENSEMBLUCSC | 462 | 9 | 28958 | island | 0.327686 | 0.047588 | Not queried | 2.92 | 1.42e-02 | 5.24e-02 |
|  | 6 | 26240307 | 26240579 | PDFSTATSENSEMBLUCSC | 273 | 5 | 34699 | tss;island | 0.976786 | 0.775857 | HIST1H4F (margin: HIST1H2APS3; HIST1H4F; HIST1H2BH; HIST1H1D; HIST1H4G; HIST1H3F) | 2.92 | 7.94e-03 | 3.19e-02 |
|  | 17 | 41277694 | 41279022 | PDFSTATSENSEMBLUCSC | 1329 | 28 | 16595 | gene | 0.009157 | 0.137068 | NBR2; BRCA1 (margin: NBR2; BRCA1) | 2.92 | 3.69e-03 | 2.33e-02 |
|  | 9 | 137392877 | 137393288 | PDFSTATSENSEMBLUCSC | 412 | 5 | 40970 | island | 0.537157 | 0.896836 | Not queried | 2.92 | 9.52e-02 | 1.53e-01 |
|  | 5 | 140568075 | 140568535 | PDFSTATSENSEMBLUCSC | 461 | 4 | 31814 | gene;island | 0.683263 | 0.960320 | Not queried | 2.92 | 2.00e-01 | 2.55e-01 |
|  | 20 | 51588780 | 51588860 | PDFSTATSENSEMBLUCSC | 81 | 4 | 25828 | tss;island | 0.106926 | 0.013647 | Not queried | 2.92 | 2.86e-02 | 6.12e-02 |
|  | 1 | 15480555 | 15480853 | PDFSTATSENSEMBLUCSC | 299 | 4 | 244 | gene;island | 0.038148 | 0.302025 | Not queried | 2.92 | 5.71e-02 | 1.00e-01 |
|  | 20 | 33460474 | 33460585 | PDFSTATSENSEMBLUCSC | 112 | 4 | 25402 | gene | 0.059174 | 0.012107 | Not queried | 2.92 | 2.86e-02 | 6.12e-02 |
|  | 6 | 31554829 | 31555016 | PDFSTATSENSEMBLUCSC | 188 | 4 | 33907 | gene | 0.564924 | 0.876907 | Not queried | 2.92 | 2.00e-01 | 2.55e-01 |
|  | 7 | 121784351 | 121784596 | PDFSTATSENSEMBLUCSC | 246 | 7 | 37864 | tss | 0.074548 | 0.378571 | AASS (margin: AASS) | 2.92 | 4.08e-03 | 2.47e-02 |
|  | 14 | 74705942 | 74706336 | PDFSTATSENSEMBLUCSC | 395 | 8 | 12997 | island | 0.622617 | 0.876807 | VSX2 (margin: VSX2) | 2.92 | 1.55e-04 | 2.71e-03 |
|  | 14 | 74705942 | 74706113 | PDFSTATSENSEMBLUCSC | 172 | 6 | 12505 | tss | 0.630797 | 0.862092 | VSX2 (margin: VSX2) | 2.92 | 2.16e-03 | 1.45e-02 |
|  | 5 | 1037853 | 1038282 | PDFSTATSENSEMBLUCSC | 430 | 4 | 31517 | gene;island | 0.745105 | 0.957027 | Not queried | 2.92 | 4.86e-01 | 5.11e-01 |
|  | 11 | 69061244 | 69061473 | PDFSTATSENSEMBLUCSC | 230 | 4 | 7518 | tss | 0.029171 | 0.198400 | Not queried | 2.92 | 2.00e-01 | 2.55e-01 |
|  | 4 | 170678860 | 170679082 | PDFSTATSENSEMBLUCSC | 223 | 5 | 30176 | gene | 0.002755 | 0.004880 | Not queried | 2.92 | 3.10e-01 | 3.66e-01 |
|  | 1 | 34632573 | 34632658 | PDFSTATSENSEMBLUCSC | 86 | 4 | 1476 | tss;island | 0.589213 | 0.863574 | Not queried | 2.92 | 1.14e-01 | 1.68e-01 |
|  | 7 | 39453532 | 39453855 | PDFSTATSENSEMBLUCSC | 324 | 5 | 36973 | gene;island | 0.254434 | 0.907001 | Not queried | 2.92 | 9.52e-02 | 1.53e-01 |
|  | 1 | 63791922 | 63792239 | PDFSTATSENSEMBLUCSC | 318 | 6 | 3343 | island | 0.949661 | 0.547664 | Not queried | 2.92 | 6.49e-02 | 1.11e-01 |
|  | 20 | 46415306 | 46415859 | PDFSTATSENSEMBLUCSC | 554 | 12 | 25802 | tss;island | 0.061332 | 0.348200 | Not queried | 2.92 | 1.01e-01 | 1.60e-01 |
|  | 4 | 174451954 | 174452159 | PDFSTATSENSEMBLUCSC | 206 | 4 | 30180 | gene;tss;island | 0.797344 | 0.973396 | Not queried | 2.92 | 3.43e-01 | 3.84e-01 |
|  | 6 | 27258460 | 27258767 | PDFSTATSENSEMBLUCSC | 308 | 6 | 35552 | island | 0.113293 | 0.460821 | Not queried | 2.92 | 1.80e-01 | 2.45e-01 |
|  | 1 | 156626749 | 156627099 | PDFSTATSENSEMBLUCSC | 351 | 4 | 826 | gene;island | 0.954496 | 0.688444 | Not queried | 2.92 | 2.86e-02 | 6.12e-02 |
|  | 12 | 111181439 | 111181819 | PDFSTATSENSEMBLUCSC | 381 | 4 | 10010 | tss;island | 0.775494 | 0.132505 | Not queried | 2.92 | 6.86e-01 | 6.99e-01 |
|  | 13 | 23471764 | 23471968 | PDFSTATSENSEMBLUCSC | 205 | 4 | 11594 | island | 0.713507 | 0.958358 | Not queried | 2.91 | 3.43e-01 | 3.84e-01 |
|  | 15 | 25430614 | 25430671 | PDFSTATSENSEMBLUCSC | 58 | 4 | 13530 | tss | 0.132428 | 0.618460 | Not queried | 2.91 | 5.71e-02 | 1.00e-01 |
|  | 5 | 118603742 | 118603938 | PDFSTATSENSEMBLUCSC | 197 | 5 | 32236 | tss;island | 0.038421 | 0.008623 | TNFAIP8 (margin: TNFAIP8; AC027320.1) | 2.91 | 7.94e-03 | 3.19e-02 |
|  | 20 | 26188919 | 26188997 | PDFSTATSENSEMBLUCSC | 79 | 6 | 25655 | tss | 0.857208 | 0.972512 | Not queried | 2.91 | 2.60e-02 | 6.12e-02 |
|  | 1 | 91176405 | 91176810 | PDFSTATSENSEMBLUCSC | 406 | 5 | 3456 | island | 0.441499 | 0.855413 | Not queried | 2.91 | 5.56e-02 | 1.00e-01 |
|  | 4 | 4866047 | 4866192 | PDFSTATSENSEMBLUCSC | 146 | 4 | 30871 | island | 0.731681 | 0.964039 | Not queried | 2.91 | 2.00e-01 | 2.55e-01 |
|  | 13 | 36919674 | 36919960 | PDFSTATSENSEMBLUCSC | 287 | 5 | 11130 | gene;island | 0.916728 | 0.988508 | SPG20OS; SPG20 (margin: SPG20OS; AL139377.1; SPG20) | 2.91 | 7.94e-03 | 3.19e-02 |
|  | 11 | 10316295 | 10316555 | PDFSTATSENSEMBLUCSC | 261 | 5 | 7141 | tss;island | 0.011192 | 0.145631 | Not queried | 2.91 | 3.17e-02 | 6.50e-02 |
|  | 9 | 79521082 | 79521532 | PDFSTATSENSEMBLUCSC | 451 | 5 | 40816 | tss;island | 0.191780 | 0.025821 | Not queried | 2.91 | 9.52e-02 | 1.53e-01 |
|  | 7 | 157412628 | 157413057 | PDFSTATSENSEMBLUCSC | 430 | 5 | 37332 | gene;island | 0.502822 | 0.855607 | Not queried | 2.91 | 5.56e-02 | 1.00e-01 |
|  | 10 | 97802849 | 97803058 | PDFSTATSENSEMBLUCSC | 210 | 10 | 5252 | tss;island | 0.581687 | 0.094440 | Not queried | 2.91 | 2.32e-02 | 6.12e-02 |
|  | 7 | 53254947 | 53255225 | PDFSTATSENSEMBLUCSC | 279 | 5 | 38497 | island | 0.319900 | 0.056303 | Not queried | 2.91 | 9.52e-02 | 1.53e-01 |
|  | 7 | 1278142 | 1278508 | PDFSTATSENSEMBLUCSC | 367 | 4 | 38093 | island | 0.794627 | 0.979850 | Not queried | 2.91 | 2.00e-01 | 2.55e-01 |
|  | 1 | 53925952 | 53926227 | PDFSTATSENSEMBLUCSC | 276 | 4 | 542 | gene;island | 0.682720 | 0.939447 | Not queried | 2.91 | 1.14e-01 | 1.68e-01 |
|  | 1 | 182361258 | 182361557 | PDFSTATSENSEMBLUCSC | 300 | 7 | 2131 | tss;island | 0.311984 | 0.725665 | GLUL (margin: GLUL; TEDDM1) | 2.91 | 1.17e-03 | 1.13e-02 |
|  | 14 | 100111183 | 100111392 | PDFSTATSENSEMBLUCSC | 210 | 8 | 12599 | tss;island | 0.819104 | 0.943878 | HHIPL1 (margin: HHIPL1) | 2.91 | 1.04e-02 | 3.96e-02 |
|  | 10 | 49892741 | 49893026 | PDFSTATSENSEMBLUCSC | 286 | 8 | 5068 | tss | 0.564010 | 0.921854 | WDFY4 (margin: WDFY4) | 2.91 | 1.86e-03 | 1.45e-02 |
|  | 22 | 23484001 | 23484600 | PDFSTATSENSEMBLUCSC | 600 | 11 | 27347 | island | 0.079306 | 0.417617 | Not queried | 2.91 | 5.57e-02 | 1.00e-01 |
|  | 22 | 23484241 | 23484600 | PDFSTATSENSEMBLUCSC | 360 | 9 | 27006 | tss | 0.079306 | 0.417617 | Not queried | 2.91 | 6.25e-02 | 1.09e-01 |
|  | 6 | 29425885 | 29426132 | PDFSTATSENSEMBLUCSC | 248 | 5 | 34747 | tss | 0.958270 | 0.990409 | Not queried | 2.90 | 9.52e-02 | 1.53e-01 |
|  | 16 | 67282824 | 67283238 | PDFSTATSENSEMBLUCSC | 415 | 5 | 16015 | island | 0.149156 | 0.028324 | Not queried | 2.90 | 3.10e-01 | 3.66e-01 |
|  | 2 | 233561679 | 233561917 | PDFSTATSENSEMBLUCSC | 239 | 6 | 23954 | tss | 0.005047 | 0.040640 | Not queried | 2.90 | 1.32e-01 | 1.89e-01 |
|  | 2 | 105484357 | 105484823 | PDFSTATSENSEMBLUCSC | 467 | 4 | 24580 | island | 0.815668 | 0.965510 | Not queried | 2.90 | 2.86e-02 | 6.12e-02 |
|  | 20 | 46130755 | 46131144 | PDFSTATSENSEMBLUCSC | 390 | 5 | 25441 | gene | 0.001358 | 0.009660 | Not queried | 2.90 | 9.52e-02 | 1.53e-01 |
|  | 7 | 157809235 | 157809596 | PDFSTATSENSEMBLUCSC | 362 | 6 | 37357 | gene | 0.353605 | 0.793015 | Not queried | 2.90 | 1.32e-01 | 1.89e-01 |
|  | 12 | 117628187 | 117628589 | PDFSTATSENSEMBLUCSC | 403 | 8 | 10863 | island | 0.066307 | 0.023010 | Not queried | 2.90 | 3.28e-01 | 3.84e-01 |
|  | 6 | 44186914 | 44187674 | PDFSTATSENSEMBLUCSC | 761 | 12 | 36171 | island | 0.866913 | 0.360541 | RP1-302G2.5; SLC29A1 (margin: RP1-302G2.5; SLC29A1) | 2.90 | 3.33e-05 | 1.17e-03 |
|  | 3 | 5021225 | 5021469 | PDFSTATSENSEMBLUCSC | 245 | 4 | 27643 | gene;island | 0.001982 | 0.007818 | Not queried | 2.90 | 3.43e-01 | 3.84e-01 |
|  | 1 | 161067636 | 161068138 | PDFSTATSENSEMBLUCSC | 503 | 9 | 2045 | tss | 0.124026 | 0.004083 | Not queried | 2.90 | 4.00e-02 | 8.03e-02 |
|  | 11 | 123396039 | 123396505 | PDFSTATSENSEMBLUCSC | 467 | 6 | 7778 | tss | 0.685552 | 0.419966 | Not queried | 2.90 | 2.40e-01 | 2.95e-01 |
|  | 18 | 51750159 | 51750371 | PDFSTATSENSEMBLUCSC | 213 | 4 | 19117 | gene;island | 0.000814 | 0.005182 | Not queried | 2.90 | 1.14e-01 | 1.68e-01 |
|  | 21 | 47394354 | 47394659 | PDFSTATSENSEMBLUCSC | 306 | 4 | 26763 | island | 0.453415 | 0.854307 | Not queried | 2.90 | 2.86e-02 | 6.12e-02 |
|  | 4 | 5891833 | 5892350 | PDFSTATSENSEMBLUCSC | 518 | 6 | 29923 | gene;island | 0.657121 | 0.929742 | Not queried | 2.90 | 9.31e-02 | 1.53e-01 |
|  | 17 | 72931569 | 72931857 | PDFSTATSENSEMBLUCSC | 289 | 10 | 17637 | tss | 0.514874 | 0.859491 | OTOP2; OTOP3 (margin: OTOP2; OTOP3) | 2.90 | 7.58e-05 | 1.98e-03 |
|  | 6 | 32632565 | 32633163 | PDFSTATSENSEMBLUCSC | 599 | 22 | 34126 | gene;island | 0.537709 | 0.936018 | HLA-DQB1 (margin: HLA-DQB1-AS1; HLA-DQB1; XXbac-BPG254F23.6) | 2.90 | 1.03e-05 | 4.82e-04 |
|  | 6 | 136871881 | 136872119 | PDFSTATSENSEMBLUCSC | 239 | 8 | 35242 | tss | 0.038935 | 0.005458 | Not queried | 2.90 | 2.07e-02 | 6.12e-02 |
|  | 1 | 79472282 | 79472643 | PDFSTATSENSEMBLUCSC | 362 | 9 | 3403 | island | 0.828666 | 0.972788 | Not queried | 2.90 | 7.70e-02 | 1.30e-01 |
|  | 1 | 9129487 | 9129791 | PDFSTATSENSEMBLUCSC | 305 | 4 | 199 | gene;island | 0.801774 | 0.972363 | Not queried | 2.90 | 1.14e-01 | 1.68e-01 |
|  | 15 | 96875591 | 96875830 | PDFSTATSENSEMBLUCSC | 240 | 8 | 13493 | gene;tss;island | 0.003604 | 0.029395 | Not queried | 2.90 | 1.30e-01 | 1.89e-01 |
|  | 16 | 86543519 | 86543567 | PDFSTATSENSEMBLUCSC | 49 | 5 | 15401 | tss;island | 0.027597 | 0.008003 | Not queried | 2.90 | 2.22e-01 | 2.76e-01 |
|  | 18 | 10588980 | 10589360 | PDFSTATSENSEMBLUCSC | 381 | 5 | 19392 | island | 0.020195 | 0.003033 | Not queried | 2.90 | 4.21e-01 | 4.59e-01 |
|  | 19 | 12997997 | 12998135 | PDFSTATSENSEMBLUCSC | 139 | 4 | 21632 | island | 0.644873 | 0.930272 | Not queried | 2.89 | 2.86e-02 | 6.12e-02 |
|  | 13 | 46189883 | 46190198 | PDFSTATSENSEMBLUCSC | 316 | 8 | 11423 | tss | 0.485625 | 0.929263 | Not queried | 2.89 | 4.99e-02 | 9.81e-02 |
|  | 2 | 217497890 | 217497949 | PDFSTATSENSEMBLUCSC | 60 | 4 | 23870 | tss;island | 0.606988 | 0.182284 | Not queried | 2.89 | 2.86e-02 | 6.12e-02 |
|  | 13 | 114837593 | 114837807 | PDFSTATSENSEMBLUCSC | 215 | 4 | 11311 | gene;island | 0.719320 | 0.929630 | Not queried | 2.89 | 3.43e-01 | 3.84e-01 |
|  | 16 | 1100552 | 1100847 | PDFSTATSENSEMBLUCSC | 296 | 4 | 15511 | island | 0.629317 | 0.920171 | Not queried | 2.89 | 2.86e-02 | 6.12e-02 |
|  | 1 | 145477137 | 145477534 | PDFSTATSENSEMBLUCSC | 398 | 6 | 726 | gene | 0.437924 | 0.022308 | Not queried | 2.89 | 9.31e-02 | 1.53e-01 |
|  | 8 | 141468467 | 141468933 | PDFSTATSENSEMBLUCSC | 467 | 7 | 39925 | tss;island | 0.967154 | 0.555204 | Not queried | 2.89 | 1.28e-01 | 1.87e-01 |
|  | 19 | 12075882 | 12076348 | PDFSTATSENSEMBLUCSC | 467 | 6 | 19851 | gene | 0.379190 | 0.779209 | Not queried | 2.89 | 2.60e-02 | 6.12e-02 |
|  | 17 | 1811181 | 1811625 | PDFSTATSENSEMBLUCSC | 445 | 5 | 17833 | island | 0.831830 | 0.971403 | (margin: RPA1) | 2.89 | 7.94e-03 | 3.19e-02 |
|  | 17 | 57915665 | 57915773 | PDFSTATSENSEMBLUCSC | 109 | 4 | 16715 | gene | 0.002677 | 0.015476 | Not queried | 2.89 | 2.86e-02 | 6.12e-02 |
|  | 17 | 48133890 | 48134226 | PDFSTATSENSEMBLUCSC | 337 | 4 | 16679 | gene;island | 0.003331 | 0.013169 | Not queried | 2.89 | 2.00e-01 | 2.55e-01 |
|  | 3 | 160167646 | 160167990 | PDFSTATSENSEMBLUCSC | 345 | 10 | 28694 | tss | 0.323923 | 0.023470 | Not queried | 2.89 | 2.32e-02 | 6.12e-02 |
|  | 15 | 63334355 | 63335016 | PDFSTATSENSEMBLUCSC | 662 | 10 | 14201 | island | 0.114948 | 0.522886 | Not queried | 2.89 | 1.43e-01 | 2.04e-01 |
|  | 17 | 67323709 | 67323953 | PDFSTATSENSEMBLUCSC | 245 | 4 | 17609 | tss;island | 0.489059 | 0.774406 | Not queried | 2.89 | 4.86e-01 | 5.11e-01 |
|  | 14 | 78432211 | 78432599 | PDFSTATSENSEMBLUCSC | 389 | 4 | 13044 | island | 0.583049 | 0.912997 | Not queried | 2.89 | 2.86e-02 | 6.12e-02 |
|  | 1 | 46668822 | 46668882 | PDFSTATSENSEMBLUCSC | 61 | 4 | 509 | gene;tss;island | 0.850407 | 0.977336 | Not queried | 2.89 | 5.71e-02 | 1.00e-01 |
|  | 3 | 138153127 | 138153381 | PDFSTATSENSEMBLUCSC | 255 | 8 | 28623 | tss | 0.213739 | 0.663162 | ESYT3 (margin: ESYT3) | 2.89 | 1.55e-04 | 2.71e-03 |
|  | 5 | 32709859 | 32710231 | PDFSTATSENSEMBLUCSC | 373 | 5 | 32720 | island | 0.091448 | 0.016364 | Not queried | 2.89 | 9.52e-02 | 1.53e-01 |
|  | 7 | 8483249 | 8483710 | PDFSTATSENSEMBLUCSC | 462 | 4 | 36893 | gene;island | 0.631741 | 0.914279 | Not queried | 2.88 | 3.43e-01 | 3.84e-01 |
|  | 3 | 133392794 | 133393240 | PDFSTATSENSEMBLUCSC | 447 | 4 | 29490 | island | 0.863110 | 0.956258 | Not queried | 2.88 | 2.00e-01 | 2.55e-01 |
|  | 1 | 6259688 | 6259905 | PDFSTATSENSEMBLUCSC | 218 | 4 | 1198 | tss | 0.004796 | 0.000769 | Not queried | 2.88 | 1.14e-01 | 1.68e-01 |
|  | 16 | 10275934 | 10276119 | PDFSTATSENSEMBLUCSC | 186 | 4 | 15086 | tss | 0.095417 | 0.429192 | Not queried | 2.88 | 2.86e-02 | 6.12e-02 |
|  | 6 | 32977138 | 32977708 | PDFSTATSENSEMBLUCSC | 571 | 9 | 35956 | island | 0.478213 | 0.871062 | Not queried | 2.88 | 1.61e-01 | 2.25e-01 |
|  | 10 | 27793255 | 27793713 | PDFSTATSENSEMBLUCSC | 459 | 4 | 4541 | gene | 0.017502 | 0.083129 | Not queried | 2.88 | 4.86e-01 | 5.11e-01 |
|  | 7 | 37955508 | 37956018 | PDFSTATSENSEMBLUCSC | 511 | 6 | 36969 | gene;island | 0.835898 | 0.976265 | Not queried | 2.88 | 1.52e-02 | 5.36e-02 |
|  | 19 | 1401576 | 1401781 | PDFSTATSENSEMBLUCSC | 206 | 4 | 20304 | tss | 0.971608 | 0.764929 | Not queried | 2.88 | 2.00e-01 | 2.55e-01 |
|  | 1 | 156211409 | 156212005 | PDFSTATSENSEMBLUCSC | 597 | 11 | 3848 | island | 0.448341 | 0.893082 | PMF1-BGLAP; PMF1; BGLAP; PAQR6 (margin: PMF1-BGLAP; PMF1; BGLAP; PAQR6; SMG5) | 2.88 | 7.65e-04 | 8.13e-03 |
|  | 16 | 68544012 | 68544404 | PDFSTATSENSEMBLUCSC | 393 | 4 | 16047 | island | 0.887334 | 0.480696 | Not queried | 2.88 | 2.00e-01 | 2.55e-01 |
|  | 1 | 179711859 | 179712361 | PDFSTATSENSEMBLUCSC | 503 | 10 | 4001 | island | 0.775828 | 0.961744 | RP11-12M5.1; FAM163A (margin: RP11-12M5.1; FAM163A) | 2.88 | 7.58e-05 | 1.98e-03 |
|  | 14 | 96180576 | 96181044 | PDFSTATSENSEMBLUCSC | 469 | 9 | 12590 | tss | 0.728216 | 0.977181 | RP11-164H13.1; TCL1A (margin: RP11-164H13.1; TCL1A) | 2.88 | 8.23e-05 | 2.06e-03 |
|  | 3 | 129982835 | 129983089 | PDFSTATSENSEMBLUCSC | 255 | 4 | 27946 | gene;island | 0.552920 | 0.898243 | Not queried | 2.88 | 2.00e-01 | 2.55e-01 |
|  | 8 | 61086176 | 61086268 | PDFSTATSENSEMBLUCSC | 93 | 4 | 40328 | island | 0.288290 | 0.839958 | Not queried | 2.88 | 3.43e-01 | 3.84e-01 |
|  | 15 | 23932620 | 23933079 | PDFSTATSENSEMBLUCSC | 460 | 5 | 13516 | tss;island | 0.380069 | 0.792497 | Not queried | 2.88 | 3.17e-02 | 6.50e-02 |
|  | 8 | 43147211 | 43147581 | PDFSTATSENSEMBLUCSC | 371 | 6 | 39687 | tss | 0.625566 | 0.965638 | Not queried | 2.88 | 2.60e-02 | 6.12e-02 |
|  | 11 | 30038615 | 30038797 | PDFSTATSENSEMBLUCSC | 183 | 9 | 7218 | tss;island | 0.870632 | 0.514975 | KCNA4 (margin: KCNA4) | 2.88 | 1.23e-03 | 1.18e-02 |
|  | 10 | 38692145 | 38692317 | PDFSTATSENSEMBLUCSC | 173 | 4 | 5038 | tss;island | 0.130696 | 0.020894 | Not queried | 2.88 | 2.00e-01 | 2.55e-01 |
|  | 1 | 70035080 | 70035479 | PDFSTATSENSEMBLUCSC | 400 | 4 | 3376 | island | 0.335647 | 0.608925 | Not queried | 2.87 | 3.43e-01 | 3.84e-01 |
|  | 17 | 46827458 | 46827626 | PDFSTATSENSEMBLUCSC | 169 | 4 | 18507 | island | 0.791450 | 0.960197 | Not queried | 2.87 | 2.86e-02 | 6.12e-02 |
|  | 10 | 46168220 | 46168597 | PDFSTATSENSEMBLUCSC | 378 | 8 | 5715 | island | 0.170297 | 0.036859 | Not queried | 2.87 | 8.30e-02 | 1.40e-01 |
|  | 16 | 57023022 | 57023398 | PDFSTATSENSEMBLUCSC | 377 | 7 | 15256 | tss | 0.024810 | 0.135094 | Not queried | 2.87 | 7.10e-01 | 7.20e-01 |
|  | 1 | 78511140 | 78511488 | PDFSTATSENSEMBLUCSC | 349 | 7 | 1710 | tss | 0.918831 | 0.980894 | GIPC2; RP11-386I14.2 (margin: GIPC2; RP11-386I14.2) | 2.87 | 6.99e-03 | 3.19e-02 |
|  | 5 | 3188066 | 3188374 | PDFSTATSENSEMBLUCSC | 309 | 5 | 32662 | island | 0.497376 | 0.136905 | Not queried | 2.87 | 1.59e-02 | 5.36e-02 |
|  | 21 | 42792127 | 42792516 | PDFSTATSENSEMBLUCSC | 390 | 4 | 26557 | tss | 0.002471 | 0.009780 | Not queried | 2.87 | 2.00e-01 | 2.55e-01 |
|  | 19 | 19050971 | 19051482 | PDFSTATSENSEMBLUCSC | 512 | 8 | 19944 | gene;tss;island | 0.013192 | 0.083671 | AC005932.1; HOMER3 (margin: DDX49; AC005932.1; AC002985.3; HOMER3) | 2.87 | 2.95e-03 | 1.88e-02 |
|  | 11 | 9112989 | 9113458 | PDFSTATSENSEMBLUCSC | 470 | 11 | 8040 | island | 0.118825 | 0.522346 | Not queried | 2.87 | 5.57e-02 | 1.00e-01 |
|  | 6 | 122931154 | 122931308 | PDFSTATSENSEMBLUCSC | 155 | 8 | 35206 | tss | 0.112124 | 0.014630 | PKIB (margin: PKIB) | 2.87 | 1.09e-03 | 1.10e-02 |
|  | 15 | 89910632 | 89910860 | PDFSTATSENSEMBLUCSC | 229 | 4 | 13886 | tss;island | 0.642602 | 0.909855 | Not queried | 2.87 | 2.86e-02 | 6.12e-02 |
|  | 1 | 2324205 | 2324396 | PDFSTATSENSEMBLUCSC | 192 | 4 | 81 | gene;tss;island | 0.259793 | 0.764193 | Not queried | 2.87 | 1.14e-01 | 1.68e-01 |
|  | 7 | 54609587 | 54609968 | PDFSTATSENSEMBLUCSC | 382 | 8 | 37601 | tss | 0.698685 | 0.329021 | Not queried | 2.87 | 2.34e-01 | 2.90e-01 |
|  | 10 | 54074743 | 54075082 | PDFSTATSENSEMBLUCSC | 340 | 4 | 4594 | gene;island | 0.067612 | 0.011150 | Not queried | 2.87 | 5.71e-02 | 1.00e-01 |
|  | 10 | 131591484 | 131591629 | PDFSTATSENSEMBLUCSC | 146 | 4 | 6260 | island | 0.575913 | 0.946191 | Not queried | 2.87 | 1.14e-01 | 1.68e-01 |
|  | 8 | 2046752 | 2047010 | PDFSTATSENSEMBLUCSC | 259 | 4 | 39188 | gene;island | 0.566640 | 0.891521 | Not queried | 2.87 | 2.86e-02 | 6.12e-02 |
|  | 3 | 49170599 | 49170849 | PDFSTATSENSEMBLUCSC | 251 | 5 | 28320 | tss | 0.023803 | 0.008127 | Not queried | 2.87 | 2.22e-01 | 2.76e-01 |
|  | 14 | 29243173 | 29243762 | PDFSTATSENSEMBLUCSC | 590 | 7 | 12053 | gene;island | 0.773593 | 0.429594 | Not queried | 2.87 | 1.28e-01 | 1.87e-01 |
|  | 1 | 209848306 | 209848577 | PDFSTATSENSEMBLUCSC | 272 | 5 | 2231 | tss;island | 0.858003 | 0.968932 | Not queried | 2.87 | 1.51e-01 | 2.11e-01 |
|  | 1 | 999599 | 999888 | PDFSTATSENSEMBLUCSC | 290 | 5 | 2398 | island | 0.012772 | 0.042281 | Not queried | 2.87 | 9.52e-02 | 1.53e-01 |
|  | 13 | 27333803 | 27334259 | PDFSTATSENSEMBLUCSC | 457 | 4 | 11113 | gene;island | 0.230853 | 0.531303 | Not queried | 2.86 | 2.00e-01 | 2.55e-01 |
|  | 14 | 97208114 | 97208333 | PDFSTATSENSEMBLUCSC | 220 | 4 | 13120 | island | 0.486203 | 0.653722 | Not queried | 2.86 | 6.86e-01 | 6.99e-01 |
|  | 6 | 32977138 | 32977349 | PDFSTATSENSEMBLUCSC | 212 | 5 | 34179 | gene | 0.478213 | 0.871062 | Not queried | 2.86 | 3.10e-01 | 3.66e-01 |
|  | 4 | 94748617 | 94748860 | PDFSTATSENSEMBLUCSC | 244 | 4 | 30500 | tss;island | 0.606142 | 0.911649 | Not queried | 2.86 | 2.86e-02 | 6.12e-02 |
|  | 2 | 106015518 | 106015869 | PDFSTATSENSEMBLUCSC | 352 | 9 | 22815 | gene;island | 0.055999 | 0.038706 | Not queried | 2.86 | 3.40e-01 | 3.84e-01 |
|  | 7 | 63667212 | 63667572 | PDFSTATSENSEMBLUCSC | 361 | 6 | 37614 | tss | 0.186329 | 0.757532 | Not queried | 2.86 | 1.32e-01 | 1.89e-01 |
|  | 1 | 240160972 | 240161447 | PDFSTATSENSEMBLUCSC | 476 | 5 | 4372 | island | 0.780514 | 0.956484 | Not queried | 2.86 | 9.52e-02 | 1.53e-01 |
|  | 17 | 8230130 | 8230940 | PDFSTATSENSEMBLUCSC | 811 | 7 | 18001 | island | 0.816762 | 0.971844 | SNORA69 (margin: ARHGEF15; SNORA69; RP11-849F2.4; AC135178.7) | 2.86 | 5.83e-04 | 6.47e-03 |
|  | 4 | 26030463 | 26030691 | PDFSTATSENSEMBLUCSC | 229 | 4 | 30965 | island | 0.634382 | 0.925637 | Not queried | 2.86 | 2.00e-01 | 2.55e-01 |
|  | 7 | 4169306 | 4169752 | PDFSTATSENSEMBLUCSC | 447 | 6 | 36829 | gene;island | 0.722540 | 0.973057 | Not queried | 2.86 | 6.49e-02 | 1.11e-01 |
|  | 2 | 54682758 | 54683048 | PDFSTATSENSEMBLUCSC | 291 | 4 | 23356 | tss;island | 0.522654 | 0.147446 | Not queried | 2.86 | 2.00e-01 | 2.55e-01 |
|  | 7 | 155283738 | 155284062 | PDFSTATSENSEMBLUCSC | 325 | 4 | 39070 | island | 0.610928 | 0.938358 | Not queried | 2.86 | 2.00e-01 | 2.55e-01 |
|  | 3 | 27410970 | 27411065 | PDFSTATSENSEMBLUCSC | 96 | 4 | 28195 | tss | 0.049296 | 0.296986 | Not queried | 2.86 | 2.00e-01 | 2.55e-01 |
|  | 6 | 167031273 | 167031411 | PDFSTATSENSEMBLUCSC | 139 | 4 | 34549 | gene;island | 0.914024 | 0.980503 | Not queried | 2.86 | 2.00e-01 | 2.55e-01 |
|  | 3 | 128712882 | 128713151 | PDFSTATSENSEMBLUCSC | 270 | 6 | 29461 | island | 0.273163 | 0.032696 | Not queried | 2.86 | 2.60e-02 | 6.12e-02 |
|  | 16 | 2581570 | 2581833 | PDFSTATSENSEMBLUCSC | 264 | 4 | 15030 | tss | 0.681612 | 0.947596 | Not queried | 2.86 | 4.86e-01 | 5.11e-01 |
|  | 10 | 128152075 | 128152247 | PDFSTATSENSEMBLUCSC | 173 | 4 | 4816 | gene | 0.147306 | 0.550458 | Not queried | 2.86 | 1.14e-01 | 1.68e-01 |
|  | 15 | 25436114 | 25436246 | PDFSTATSENSEMBLUCSC | 133 | 4 | 13532 | tss | 0.493238 | 0.679079 | Not queried | 2.86 | 6.86e-01 | 6.99e-01 |
|  | 19 | 58662188 | 58662330 | PDFSTATSENSEMBLUCSC | 143 | 4 | 21135 | tss;island | 0.273479 | 0.023393 | Not queried | 2.85 | 2.00e-01 | 2.55e-01 |
|  | 13 | 32605187 | 32605406 | PDFSTATSENSEMBLUCSC | 220 | 7 | 11381 | tss;island | 0.358917 | 0.059821 | Not queried | 2.85 | 3.79e-02 | 7.66e-02 |
|  | 13 | 25745946 | 25746644 | PDFSTATSENSEMBLUCSC | 699 | 11 | 11350 | tss | 0.657428 | 0.917557 | AMER2; RP11-165I9.4 (margin: RP11-165I9.8; AMER2; RP11-165I9.4) | 2.85 | 4.10e-03 | 2.48e-02 |
|  | 12 | 56882314 | 56882693 | PDFSTATSENSEMBLUCSC | 380 | 7 | 9800 | tss;island | 0.273908 | 0.739180 | Not queried | 2.85 | 1.28e-01 | 1.87e-01 |
|  | 17 | 35293930 | 35294230 | PDFSTATSENSEMBLUCSC | 301 | 4 | 17283 | tss;island | 0.080258 | 0.007533 | Not queried | 2.85 | 2.00e-01 | 2.55e-01 |
|  | 16 | 62069806 | 62070365 | PDFSTATSENSEMBLUCSC | 560 | 11 | 15985 | island | 0.855866 | 0.387991 | CDH8 (margin: CDH8) | 2.85 | 1.28e-02 | 4.76e-02 |
|  | 16 | 62070072 | 62070365 | PDFSTATSENSEMBLUCSC | 294 | 9 | 15278 | tss | 0.905400 | 0.424157 | Not queried | 2.85 | 3.15e-02 | 6.50e-02 |
|  | 11 | 86383182 | 86383585 | PDFSTATSENSEMBLUCSC | 404 | 12 | 6854 | gene | 0.796912 | 0.961128 | ME3 (margin: ME3) | 2.85 | 7.40e-07 | 7.21e-05 |
|  | 17 | 3848156 | 3848506 | PDFSTATSENSEMBLUCSC | 351 | 4 | 16352 | gene;island | 0.310430 | 0.698580 | Not queried | 2.85 | 1.14e-01 | 1.68e-01 |
|  | 10 | 18689378 | 18689503 | PDFSTATSENSEMBLUCSC | 126 | 5 | 4523 | gene;tss | 0.074061 | 0.010042 | Not queried | 2.85 | 3.10e-01 | 3.66e-01 |
|  | 6 | 87646738 | 87647154 | PDFSTATSENSEMBLUCSC | 417 | 11 | 36305 | island | 0.426752 | 0.041357 | HTR1E (margin: HTR1E) | 2.85 | 6.63e-03 | 3.19e-02 |
|  | 11 | 1483731 | 1483973 | PDFSTATSENSEMBLUCSC | 243 | 4 | 7926 | island | 0.759625 | 0.974485 | Not queried | 2.85 | 3.43e-01 | 3.84e-01 |
|  | 11 | 63438586 | 63439065 | PDFSTATSENSEMBLUCSC | 480 | 6 | 6653 | gene | 0.040391 | 0.009700 | Not queried | 2.85 | 1.52e-02 | 5.36e-02 |
|  | 22 | 38218945 | 38219336 | PDFSTATSENSEMBLUCSC | 392 | 4 | 27126 | tss | 0.362164 | 0.855991 | Not queried | 2.85 | 5.71e-02 | 1.00e-01 |
|  | 5 | 135265753 | 135266135 | PDFSTATSENSEMBLUCSC | 383 | 6 | 33142 | island | 0.749417 | 0.977520 | Not queried | 2.85 | 9.31e-02 | 1.53e-01 |
|  | 1 | 223538174 | 223538379 | PDFSTATSENSEMBLUCSC | 206 | 4 | 2271 | tss;island | 0.834828 | 0.541279 | Not queried | 2.85 | 2.86e-02 | 6.12e-02 |
|  | 17 | 21415244 | 21415527 | PDFSTATSENSEMBLUCSC | 284 | 4 | 18123 | island | 0.633762 | 0.240326 | Not queried | 2.85 | 3.43e-01 | 3.84e-01 |
|  | 1 | 166136206 | 166136939 | PDFSTATSENSEMBLUCSC | 734 | 8 | 2070 | tss;island | 0.267629 | 0.014660 | Not queried | 2.85 | 2.79e-01 | 3.38e-01 |
|  | 1 | 200004529 | 200004757 | PDFSTATSENSEMBLUCSC | 229 | 5 | 933 | gene;island | 0.683844 | 0.946193 | Not queried | 2.84 | 3.17e-02 | 6.50e-02 |
|  | 11 | 77850393 | 77850650 | PDFSTATSENSEMBLUCSC | 258 | 4 | 6844 | gene | 0.000553 | 0.004562 | Not queried | 2.84 | 2.86e-02 | 6.12e-02 |
|  | 3 | 38691248 | 38691506 | PDFSTATSENSEMBLUCSC | 259 | 4 | 28230 | tss;island | 0.377685 | 0.045511 | Not queried | 2.84 | 1.14e-01 | 1.68e-01 |
|  | 19 | 55887704 | 55888156 | PDFSTATSENSEMBLUCSC | 453 | 8 | 21077 | tss;island | 0.262517 | 0.773670 | Not queried | 2.84 | 1.48e-02 | 5.36e-02 |
|  | 13 | 36788667 | 36789025 | PDFSTATSENSEMBLUCSC | 359 | 8 | 11667 | island | 0.648314 | 0.972219 | SOHLH2; CCDC169-SOHLH2 (margin: SOHLH2; CCDC169-SOHLH2) | 2.84 | 6.99e-03 | 3.19e-02 |
|  | 4 | 154605087 | 154605276 | PDFSTATSENSEMBLUCSC | 190 | 4 | 30642 | tss | 0.522035 | 0.153130 | Not queried | 2.84 | 2.86e-02 | 6.12e-02 |
|  | 5 | 72530105 | 72530245 | PDFSTATSENSEMBLUCSC | 141 | 4 | 32867 | island | 0.667762 | 0.929728 | Not queried | 2.84 | 2.86e-02 | 6.12e-02 |
|  | 13 | 100607772 | 100608447 | PDFSTATSENSEMBLUCSC | 676 | 5 | 11844 | island | 0.668042 | 0.979175 | Not queried | 2.84 | 3.17e-02 | 6.50e-02 |
|  | 2 | 220378996 | 220379260 | PDFSTATSENSEMBLUCSC | 265 | 9 | 23036 | gene;island | 0.555074 | 0.950989 | ASIC4; AC053503.11 (margin: GMPPA; ASIC4; AC053503.11) | 2.84 | 1.06e-02 | 4.01e-02 |
|  | 6 | 29760856 | 29761075 | PDFSTATSENSEMBLUCSC | 220 | 7 | 34755 | tss | 0.925862 | 0.623490 | Not queried | 2.84 | 5.30e-02 | 1.00e-01 |
|  | 1 | 3213431 | 3213790 | PDFSTATSENSEMBLUCSC | 360 | 4 | 118 | gene | 0.505259 | 0.817328 | Not queried | 2.84 | 2.00e-01 | 2.55e-01 |
|  | 1 | 55504649 | 55505189 | PDFSTATSENSEMBLUCSC | 541 | 10 | 1651 | tss | 0.061511 | 0.234296 | Not queried | 2.84 | 1.43e-01 | 2.04e-01 |
|  | 1 | 161008811 | 161008977 | PDFSTATSENSEMBLUCSC | 167 | 4 | 2042 | tss | 0.009666 | 0.010680 | Not queried | 2.84 | 8.86e-01 | 8.89e-01 |
|  | 19 | 54876728 | 54876795 | PDFSTATSENSEMBLUCSC | 68 | 4 | 21060 | tss | 0.834090 | 0.951600 | Not queried | 2.84 | 5.71e-02 | 1.00e-01 |
|  | 17 | 3375469 | 3375601 | PDFSTATSENSEMBLUCSC | 133 | 5 | 17011 | tss;island | 0.601517 | 0.943964 | Not queried | 2.84 | 1.59e-02 | 5.36e-02 |
|  | 11 | 58673453 | 58673615 | PDFSTATSENSEMBLUCSC | 163 | 4 | 8298 | island | 0.175057 | 0.766981 | Not queried | 2.84 | 1.14e-01 | 1.68e-01 |
|  | 3 | 107646471 | 107647202 | PDFSTATSENSEMBLUCSC | 732 | 7 | 27887 | gene;island | 0.026011 | 0.009888 | Not queried | 2.84 | 3.79e-02 | 7.66e-02 |
|  | 13 | 51640297 | 51640442 | PDFSTATSENSEMBLUCSC | 146 | 4 | 11450 | tss | 0.684655 | 0.914196 | Not queried | 2.84 | 2.00e-01 | 2.55e-01 |
|  | 5 | 145724352 | 145724643 | PDFSTATSENSEMBLUCSC | 292 | 5 | 33283 | island | 0.591560 | 0.945777 | Not queried | 2.84 | 1.59e-02 | 5.36e-02 |
|  | 8 | 65489863 | 65489939 | PDFSTATSENSEMBLUCSC | 77 | 5 | 39734 | tss;island | 0.546337 | 0.927886 | RP11-21C4.1 (margin: BHLHE22; RP11-21C4.5; RP11-21C4.1; CYP7B1) | 2.83 | 7.94e-03 | 3.19e-02 |
|  | 10 | 6244514 | 6244686 | PDFSTATSENSEMBLUCSC | 173 | 4 | 4480 | gene;tss;island | 0.098359 | 0.026518 | Not queried | 2.83 | 3.43e-01 | 3.84e-01 |
|  | 1 | 16346120 | 16346434 | PDFSTATSENSEMBLUCSC | 315 | 4 | 1279 | tss | 0.567010 | 0.810192 | Not queried | 2.83 | 2.00e-01 | 2.55e-01 |
|  | 22 | 38218945 | 38219400 | PDFSTATSENSEMBLUCSC | 456 | 5 | 27475 | island | 0.415024 | 0.837109 | Not queried | 2.83 | 3.17e-02 | 6.50e-02 |
|  | 7 | 96653617 | 96654198 | PDFSTATSENSEMBLUCSC | 582 | 9 | 38696 | island | 0.551820 | 0.119323 | DLX5 (margin: DLX6-AS1; DLX5) | 2.83 | 1.06e-02 | 4.01e-02 |
|  | 6 | 30076659 | 30076980 | PDFSTATSENSEMBLUCSC | 322 | 4 | 33723 | gene | 0.883890 | 0.523750 | Not queried | 2.83 | 2.00e-01 | 2.55e-01 |
|  | 17 | 36105064 | 36105517 | PDFSTATSENSEMBLUCSC | 454 | 7 | 18250 | island | 0.619792 | 0.972102 | RP11-115K3.1; HNF1B (margin: RP11-115K3.1; HNF1B) | 2.83 | 1.11e-02 | 4.14e-02 |
|  | 5 | 168195173 | 168195460 | PDFSTATSENSEMBLUCSC | 288 | 4 | 31886 | gene | 0.370050 | 0.817153 | Not queried | 2.83 | 2.00e-01 | 2.55e-01 |
|  | 22 | 42062693 | 42063028 | PDFSTATSENSEMBLUCSC | 336 | 4 | 27526 | island | 0.005333 | 0.026494 | Not queried | 2.83 | 2.86e-02 | 6.12e-02 |
|  | 16 | 820050 | 820212 | PDFSTATSENSEMBLUCSC | 163 | 4 | 14546 | gene | 0.859626 | 0.977415 | Not queried | 2.83 | 5.71e-02 | 1.00e-01 |
|  | 7 | 124404244 | 124404472 | PDFSTATSENSEMBLUCSC | 229 | 4 | 37207 | gene;island | 0.586703 | 0.891342 | Not queried | 2.83 | 2.00e-01 | 2.55e-01 |
|  | 19 | 1725535 | 1725758 | PDFSTATSENSEMBLUCSC | 224 | 4 | 21238 | island | 0.307696 | 0.075813 | Not queried | 2.83 | 4.86e-01 | 5.11e-01 |
|  | 1 | 160039847 | 160040667 | PDFSTATSENSEMBLUCSC | 821 | 13 | 3887 | island | 0.389606 | 0.853883 | RP11-536C5.2; KCNJ10 (margin: RP11-536C5.2; KCNJ9; KCNJ10) | 2.83 | 6.28e-04 | 6.77e-03 |
|  | 7 | 157260817 | 157261163 | PDFSTATSENSEMBLUCSC | 347 | 4 | 39103 | island | 0.519352 | 0.808995 | Not queried | 2.83 | 2.00e-01 | 2.55e-01 |
|  | 3 | 115377592 | 115377939 | PDFSTATSENSEMBLUCSC | 348 | 4 | 27901 | gene;island | 0.718268 | 0.962611 | Not queried | 2.83 | 4.86e-01 | 5.11e-01 |
|  | 13 | 114926584 | 114927035 | PDFSTATSENSEMBLUCSC | 452 | 4 | 11998 | island | 0.691844 | 0.972105 | Not queried | 2.83 | 1.14e-01 | 1.68e-01 |
|  | 15 | 44116991 | 44117354 | PDFSTATSENSEMBLUCSC | 364 | 6 | 13635 | tss | 0.498162 | 0.301765 | Not queried | 2.83 | 3.94e-01 | 4.36e-01 |
|  | 12 | 8332640 | 8333192 | PDFSTATSENSEMBLUCSC | 553 | 9 | 10233 | island | 0.162468 | 0.019755 | Not queried | 2.83 | 1.88e-02 | 6.12e-02 |
|  | 2 | 158453600 | 158454401 | PDFSTATSENSEMBLUCSC | 802 | 9 | 22903 | gene;island | 0.341089 | 0.805723 | Not queried | 2.83 | 3.87e-01 | 4.30e-01 |
|  | 10 | 27547536 | 27547965 | PDFSTATSENSEMBLUCSC | 430 | 6 | 5628 | island | 0.799525 | 0.977925 | ARMC4P1; LRRC37A6P (margin: RP11-85G18.6; ARMC4P1; LRRC37A6P) | 2.83 | 2.16e-03 | 1.45e-02 |
|  | 11 | 117947459 | 117947657 | PDFSTATSENSEMBLUCSC | 199 | 5 | 7725 | tss | 0.010782 | 0.082491 | Not queried | 2.83 | 1.51e-01 | 2.11e-01 |
|  | 5 | 50674642 | 50675192 | PDFSTATSENSEMBLUCSC | 551 | 6 | 32787 | island | 0.772187 | 0.430492 | Not queried | 2.83 | 1.52e-02 | 5.36e-02 |
|  | 16 | 332515 | 332963 | PDFSTATSENSEMBLUCSC | 449 | 6 | 14525 | gene | 0.726268 | 0.948096 | Not queried | 2.83 | 2.40e-01 | 2.95e-01 |
|  | 19 | 46526333 | 46526675 | PDFSTATSENSEMBLUCSC | 343 | 7 | 20889 | tss | 0.600238 | 0.940981 | CCDC61; PGLYRP1 (margin: CCDC61; MIR769; CTC-344H19.4; PGLYRP1) | 2.83 | 4.08e-03 | 2.47e-02 |
|  | 12 | 568798 | 569221 | PDFSTATSENSEMBLUCSC | 424 | 5 | 9479 | tss;island | 0.050082 | 0.005752 | Not queried | 2.83 | 5.56e-02 | 1.00e-01 |
|  | 11 | 78780700 | 78780983 | PDFSTATSENSEMBLUCSC | 284 | 4 | 6848 | gene;island | 0.892445 | 0.981709 | Not queried | 2.82 | 2.86e-02 | 6.12e-02 |
|  | 17 | 36103036 | 36103289 | PDFSTATSENSEMBLUCSC | 254 | 4 | 16538 | gene;island | 0.845032 | 0.977537 | Not queried | 2.82 | 2.00e-01 | 2.55e-01 |
|  | 15 | 57598511 | 57599225 | PDFSTATSENSEMBLUCSC | 715 | 8 | 13364 | gene;island | 0.284750 | 0.034095 | Not queried | 2.82 | 3.79e-02 | 7.66e-02 |
|  | 1 | 3104999 | 3105326 | PDFSTATSENSEMBLUCSC | 328 | 5 | 107 | gene;island | 0.682747 | 0.947706 | PRDM16 (margin: PRDM16) | 2.82 | 7.94e-03 | 3.19e-02 |
|  | 11 | 115630531 | 115631762 | PDFSTATSENSEMBLUCSC | 1232 | 14 | 8831 | island | 0.655795 | 0.971369 | AP000797.3; LINC00900 (margin: AP000797.3; LINC00900) | 2.82 | 1.31e-04 | 2.71e-03 |
|  | 17 | 58677051 | 58677246 | PDFSTATSENSEMBLUCSC | 196 | 4 | 17554 | tss;island | 0.006470 | 0.025826 | Not queried | 2.82 | 2.00e-01 | 2.55e-01 |
|  | 11 | 14993378 | 14995322 | PDFSTATSENSEMBLUCSC | 1945 | 40 | 8081 | island | 0.551579 | 0.954245 | CALCB; CALCA (margin: CALCB; CALCA) | 2.82 | 5.34e-08 | 9.64e-06 |
|  | 5 | 172665707 | 172666228 | PDFSTATSENSEMBLUCSC | 522 | 4 | 33409 | island | 0.270100 | 0.606684 | Not queried | 2.82 | 2.00e-01 | 2.55e-01 |
|  | 11 | 27722037 | 27722066 | PDFSTATSENSEMBLUCSC | 30 | 4 | 6545 | gene;tss;island | 0.010934 | 0.042594 | Not queried | 2.82 | 3.43e-01 | 3.84e-01 |
|  | 17 | 47113407 | 47113596 | PDFSTATSENSEMBLUCSC | 190 | 4 | 16671 | gene;island | 0.161963 | 0.019328 | Not queried | 2.82 | 1.14e-01 | 1.68e-01 |
|  | 3 | 44596178 | 44596770 | PDFSTATSENSEMBLUCSC | 593 | 9 | 29035 | island | 0.882707 | 0.981091 | Not queried | 2.82 | 1.42e-02 | 5.24e-02 |
|  | 7 | 22705331 | 22705607 | PDFSTATSENSEMBLUCSC | 277 | 4 | 38301 | island | 0.865435 | 0.974428 | Not queried | 2.82 | 8.86e-01 | 8.89e-01 |
|  | 7 | 155603792 | 155604091 | PDFSTATSENSEMBLUCSC | 300 | 4 | 37313 | gene;island | 0.254464 | 0.613679 | Not queried | 2.82 | 2.00e-01 | 2.55e-01 |
|  | 1 | 33648648 | 33648819 | PDFSTATSENSEMBLUCSC | 172 | 4 | 1470 | tss;island | 0.775033 | 0.438524 | Not queried | 2.82 | 1.14e-01 | 1.68e-01 |
|  | 12 | 131438523 | 131438788 | PDFSTATSENSEMBLUCSC | 266 | 6 | 9420 | gene | 0.114244 | 0.532822 | Not queried | 2.82 | 1.52e-02 | 5.36e-02 |
|  | 7 | 92464881 | 92465342 | PDFSTATSENSEMBLUCSC | 462 | 8 | 37089 | gene;island | 0.010133 | 0.071381 | AC002454.1; CDK6 (margin: AC002454.1; CDK6) | 2.82 | 1.86e-03 | 1.45e-02 |
|  | 1 | 12123262 | 12123339 | PDFSTATSENSEMBLUCSC | 78 | 4 | 1257 | tss | 0.626779 | 0.936921 | Not queried | 2.82 | 5.71e-02 | 1.00e-01 |
|  | 8 | 25909171 | 25909599 | PDFSTATSENSEMBLUCSC | 429 | 6 | 40172 | island | 0.585711 | 0.977495 | Not queried | 2.81 | 9.31e-02 | 1.53e-01 |
|  | 14 | 56584485 | 56584987 | PDFSTATSENSEMBLUCSC | 503 | 5 | 12416 | tss | 0.008470 | 0.006655 | Not queried | 2.81 | 6.90e-01 | 7.01e-01 |
|  | 17 | 59940709 | 59941132 | PDFSTATSENSEMBLUCSC | 424 | 8 | 18633 | island | 0.003587 | 0.021337 | Not queried | 2.81 | 2.81e-02 | 6.12e-02 |
|  | 1 | 248854887 | 248855545 | PDFSTATSENSEMBLUCSC | 659 | 6 | 4423 | island | 0.787912 | 0.857775 | Not queried | 2.81 | 2.40e-01 | 2.95e-01 |
|  | 5 | 493262 | 493893 | PDFSTATSENSEMBLUCSC | 632 | 6 | 31507 | gene;island | 0.507410 | 0.815436 | Not queried | 2.81 | 2.60e-02 | 6.12e-02 |
|  | 5 | 177697400 | 177697669 | PDFSTATSENSEMBLUCSC | 270 | 4 | 31926 | gene | 0.441898 | 0.766654 | Not queried | 2.81 | 2.00e-01 | 2.55e-01 |
|  | 17 | 41150487 | 41150871 | PDFSTATSENSEMBLUCSC | 385 | 4 | 16593 | gene | 0.004604 | 0.013868 | Not queried | 2.81 | 5.71e-02 | 1.00e-01 |
|  | 15 | 37391424 | 37391721 | PDFSTATSENSEMBLUCSC | 298 | 4 | 13284 | gene;tss;island | 0.006747 | 0.119870 | Not queried | 2.81 | 2.00e-01 | 2.55e-01 |
|  | 5 | 1553051 | 1553224 | PDFSTATSENSEMBLUCSC | 174 | 4 | 32634 | island | 0.727235 | 0.870320 | Not queried | 2.81 | 3.43e-01 | 3.84e-01 |
|  | 5 | 72415687 | 72416275 | PDFSTATSENSEMBLUCSC | 589 | 9 | 32120 | tss | 0.168007 | 0.446097 | Not queried | 2.81 | 1.90e-01 | 2.55e-01 |
|  | 11 | 86085623 | 86086101 | PDFSTATSENSEMBLUCSC | 479 | 7 | 8711 | island | 0.744010 | 0.923703 | Not queried | 2.81 | 1.28e-01 | 1.87e-01 |
|  | 5 | 75469826 | 75470039 | PDFSTATSENSEMBLUCSC | 214 | 4 | 31651 | gene;island | 0.489094 | 0.847987 | Not queried | 2.81 | 2.86e-02 | 6.12e-02 |
|  | 4 | 190905801 | 190906123 | PDFSTATSENSEMBLUCSC | 323 | 7 | 31495 | island | 0.811592 | 0.968240 | Not queried | 2.81 | 1.28e-01 | 1.87e-01 |
|  | 11 | 31845148 | 31845639 | PDFSTATSENSEMBLUCSC | 492 | 5 | 8168 | island | 0.738291 | 0.904927 | Not queried | 2.81 | 2.22e-01 | 2.76e-01 |
|  | 12 | 133526451 | 133526768 | PDFSTATSENSEMBLUCSC | 318 | 4 | 9470 | gene | 0.982040 | 0.797362 | Not queried | 2.81 | 3.43e-01 | 3.84e-01 |
|  | 7 | 42275813 | 42276004 | PDFSTATSENSEMBLUCSC | 192 | 4 | 36977 | gene;island | 0.083657 | 0.016341 | Not queried | 2.81 | 3.43e-01 | 3.84e-01 |
|  | 7 | 116139180 | 116139849 | PDFSTATSENSEMBLUCSC | 670 | 11 | 38850 | island | 0.040383 | 0.289594 | Not queried | 2.81 | 4.00e-02 | 8.03e-02 |
|  | 17 | 12692246 | 12692692 | PDFSTATSENSEMBLUCSC | 447 | 6 | 17142 | tss;island | 0.047806 | 0.006021 | Not queried | 2.81 | 6.99e-01 | 7.09e-01 |
|  | 7 | 19157296 | 19157420 | PDFSTATSENSEMBLUCSC | 125 | 4 | 37459 | tss | 0.245555 | 0.590390 | Not queried | 2.80 | 2.86e-02 | 6.12e-02 |
|  | 11 | 68451699 | 68451858 | PDFSTATSENSEMBLUCSC | 160 | 6 | 7510 | tss;island | 0.103501 | 0.383691 | Not queried | 2.80 | 4.11e-02 | 8.14e-02 |
|  | 17 | 77397067 | 77397758 | PDFSTATSENSEMBLUCSC | 692 | 6 | 16841 | gene | 0.156965 | 0.053564 | Not queried | 2.80 | 6.99e-01 | 7.09e-01 |
|  | 17 | 79128885 | 79129078 | PDFSTATSENSEMBLUCSC | 194 | 5 | 16898 | gene;island | 0.846458 | 0.984457 | Not queried | 2.80 | 2.22e-01 | 2.76e-01 |
|  | 19 | 13616871 | 13617094 | PDFSTATSENSEMBLUCSC | 224 | 4 | 19882 | gene;island | 0.826577 | 0.975036 | Not queried | 2.80 | 2.86e-02 | 6.12e-02 |
|  | 7 | 27170241 | 27170394 | PDFSTATSENSEMBLUCSC | 154 | 4 | 36933 | gene | 0.043967 | 0.010824 | Not queried | 2.80 | 2.00e-01 | 2.55e-01 |
|  | 12 | 128245397 | 128245800 | PDFSTATSENSEMBLUCSC | 404 | 6 | 10987 | island | 0.706798 | 0.187339 | Not queried | 2.80 | 9.31e-02 | 1.53e-01 |
|  | 7 | 50518148 | 50518647 | PDFSTATSENSEMBLUCSC | 500 | 7 | 37594 | tss | 0.733310 | 0.361188 | Not queried | 2.80 | 3.18e-01 | 3.74e-01 |
|  | 16 | 1060367 | 1060765 | PDFSTATSENSEMBLUCSC | 399 | 5 | 15506 | island | 0.243770 | 0.665776 | Not queried | 2.80 | 9.52e-02 | 1.53e-01 |
|  | 2 | 209271164 | 209272057 | PDFSTATSENSEMBLUCSC | 894 | 10 | 25050 | island | 0.796132 | 0.968239 | PTH2R (margin: PTH2R) | 2.80 | 2.06e-04 | 3.42e-03 |
|  | 7 | 158854447 | 158854856 | PDFSTATSENSEMBLUCSC | 410 | 4 | 37383 | gene;island | 0.887173 | 0.946987 | Not queried | 2.80 | 3.43e-01 | 3.84e-01 |
|  | 14 | 70038717 | 70038949 | PDFSTATSENSEMBLUCSC | 233 | 4 | 12482 | tss;island | 0.355616 | 0.693898 | Not queried | 2.80 | 1.14e-01 | 1.68e-01 |
|  | 2 | 239140340 | 239141043 | PDFSTATSENSEMBLUCSC | 704 | 9 | 23097 | gene | 0.558933 | 0.934166 | AC096574.4; AC016757.3 (margin: AC096574.4; AC016757.3; HES6; PER2) | 2.79 | 7.77e-03 | 3.19e-02 |
|  | 6 | 24357720 | 24357985 | PDFSTATSENSEMBLUCSC | 266 | 4 | 33589 | gene;island | 0.832237 | 0.421823 | Not queried | 2.79 | 2.86e-02 | 6.12e-02 |
|  | 4 | 74719046 | 74719289 | PDFSTATSENSEMBLUCSC | 244 | 4 | 30022 | gene | 0.876259 | 0.568520 | Not queried | 2.79 | 2.86e-02 | 6.12e-02 |
|  | 2 | 228736089 | 228736459 | PDFSTATSENSEMBLUCSC | 371 | 9 | 25169 | island | 0.848077 | 0.975393 | Not queried | 2.79 | 3.15e-02 | 6.50e-02 |
|  | 11 | 94965364 | 94965610 | PDFSTATSENSEMBLUCSC | 247 | 4 | 7635 | tss;island | 0.934865 | 0.988870 | Not queried | 2.79 | 2.86e-02 | 6.12e-02 |
|  | 6 | 28481660 | 28482191 | PDFSTATSENSEMBLUCSC | 532 | 6 | 33634 | gene | 0.333429 | 0.770260 | Not queried | 2.79 | 3.10e-01 | 3.66e-01 |
|  | 21 | 34442160 | 34442377 | PDFSTATSENSEMBLUCSC | 218 | 7 | 26517 | tss | 0.837944 | 0.972830 | Not queried | 2.79 | 3.79e-02 | 7.66e-02 |
|  | 2 | 182549353 | 182549604 | PDFSTATSENSEMBLUCSC | 252 | 5 | 24939 | island | 0.658845 | 0.966423 | Not queried | 2.79 | 5.56e-02 | 1.00e-01 |
|  | 2 | 16080501 | 16080760 | PDFSTATSENSEMBLUCSC | 260 | 5 | 22581 | gene;island | 0.046338 | 0.011217 | Not queried | 2.79 | 4.21e-01 | 4.59e-01 |
|  | 11 | 14993378 | 14993818 | PDFSTATSENSEMBLUCSC | 441 | 6 | 6520 | gene | 0.452193 | 0.786722 | Not queried | 2.79 | 4.11e-02 | 8.14e-02 |
|  | 6 | 148663353 | 148663680 | PDFSTATSENSEMBLUCSC | 328 | 10 | 35280 | tss | 0.046244 | 0.271113 | SASH1 (margin: SASH1) | 2.79 | 1.15e-02 | 4.29e-02 |
|  | 7 | 138483046 | 138483314 | PDFSTATSENSEMBLUCSC | 269 | 4 | 37238 | gene;tss | 0.643334 | 0.918937 | Not queried | 2.79 | 1.14e-01 | 1.68e-01 |
|  | 2 | 232260116 | 232260305 | PDFSTATSENSEMBLUCSC | 190 | 4 | 23939 | tss;island | 0.871866 | 0.982601 | Not queried | 2.79 | 5.71e-02 | 1.00e-01 |
|  | 3 | 68981852 | 68982098 | PDFSTATSENSEMBLUCSC | 247 | 9 | 28437 | tss;island | 0.752155 | 0.392432 | FAM19A4 (margin: FAM19A4) | 2.79 | 8.23e-05 | 2.06e-03 |
|  | 1 | 2984218 | 2984869 | PDFSTATSENSEMBLUCSC | 652 | 10 | 2528 | island | 0.884607 | 0.974694 | PRDM16; LINC00982 (margin: PRDM16; LINC00982; AL008733.1) | 2.79 | 2.06e-04 | 3.42e-03 |
|  | 15 | 85177026 | 85177473 | PDFSTATSENSEMBLUCSC | 448 | 4 | 13461 | gene;island | 0.299156 | 0.049526 | Not queried | 2.79 | 2.00e-01 | 2.55e-01 |
|  | 17 | 81047627 | 81047941 | PDFSTATSENSEMBLUCSC | 315 | 4 | 16976 | gene;island | 0.092761 | 0.512150 | Not queried | 2.79 | 1.14e-01 | 1.68e-01 |
|  | 10 | 88024553 | 88024673 | PDFSTATSENSEMBLUCSC | 121 | 6 | 5196 | tss | 0.539943 | 0.917576 | Not queried | 2.79 | 1.52e-02 | 5.36e-02 |
|  | 19 | 8495671 | 8495870 | PDFSTATSENSEMBLUCSC | 200 | 4 | 19794 | gene;island | 0.656531 | 0.923396 | Not queried | 2.79 | 2.00e-01 | 2.55e-01 |
|  | 10 | 1245261 | 1245586 | PDFSTATSENSEMBLUCSC | 326 | 4 | 4458 | gene | 0.425394 | 0.682682 | Not queried | 2.79 | 4.86e-01 | 5.11e-01 |
|  | 13 | 25745768 | 25746644 | PDFSTATSENSEMBLUCSC | 877 | 13 | 11610 | island | 0.681769 | 0.947584 | Not queried | 2.79 | 1.40e-02 | 5.20e-02 |
|  | 18 | 59992157 | 59992501 | PDFSTATSENSEMBLUCSC | 345 | 7 | 19303 | tss;island | 0.390757 | 0.016162 | Not queried | 2.78 | 7.28e-02 | 1.23e-01 |
|  | 11 | 74459620 | 74460020 | PDFSTATSENSEMBLUCSC | 401 | 8 | 8654 | island | 0.003771 | 0.013792 | Not queried | 2.78 | 2.07e-02 | 6.12e-02 |
|  | 10 | 88391658 | 88392088 | PDFSTATSENSEMBLUCSC | 431 | 4 | 5907 | island | 0.854542 | 0.977976 | Not queried | 2.78 | 5.71e-02 | 1.00e-01 |
|  | 19 | 3687751 | 3688176 | PDFSTATSENSEMBLUCSC | 426 | 5 | 19721 | gene;island | 0.915567 | 0.593627 | Not queried | 2.78 | 5.48e-01 | 5.70e-01 |
|  | 4 | 2278261 | 2278594 | PDFSTATSENSEMBLUCSC | 334 | 5 | 29886 | gene;island | 0.860167 | 0.963601 | Not queried | 2.78 | 5.56e-02 | 1.00e-01 |
|  | 17 | 1083183 | 1083491 | PDFSTATSENSEMBLUCSC | 309 | 4 | 16985 | tss | 0.060693 | 0.007173 | Not queried | 2.78 | 3.43e-01 | 3.84e-01 |
|  | 6 | 33158503 | 33158597 | PDFSTATSENSEMBLUCSC | 95 | 4 | 34214 | gene;island | 0.739763 | 0.979263 | Not queried | 2.78 | 1.14e-01 | 1.68e-01 |
|  | 20 | 34252616 | 34253242 | PDFSTATSENSEMBLUCSC | 627 | 9 | 26128 | island | 0.004542 | 0.014862 | Not queried | 2.78 | 2.97e-01 | 3.60e-01 |
|  | 1 | 101702519 | 101702782 | PDFSTATSENSEMBLUCSC | 264 | 5 | 654 | gene;island | 0.323418 | 0.765093 | S1PR1; RP4-575N6.4 (margin: S1PR1; RP4-575N6.5; snoU13; RP4-575N6.4) | 2.78 | 7.94e-03 | 3.19e-02 |
|  | 22 | 43505892 | 43506952 | PDFSTATSENSEMBLUCSC | 1061 | 27 | 27551 | island | 0.234504 | 0.027424 | BIK (margin: BIK) | 2.78 | 1.25e-02 | 4.66e-02 |
|  | 15 | 40762807 | 40763199 | PDFSTATSENSEMBLUCSC | 393 | 6 | 13589 | tss | 0.016800 | 0.104441 | Not queried | 2.78 | 6.49e-02 | 1.11e-01 |
|  | 6 | 168106055 | 168106326 | PDFSTATSENSEMBLUCSC | 272 | 4 | 36645 | island | 0.755576 | 0.973727 | Not queried | 2.78 | 1.14e-01 | 1.68e-01 |
|  | 5 | 1253833 | 1254048 | PDFSTATSENSEMBLUCSC | 216 | 4 | 31529 | gene;island | 0.493863 | 0.952672 | Not queried | 2.78 | 5.71e-02 | 1.00e-01 |
|  | 10 | 85985296 | 85985448 | PDFSTATSENSEMBLUCSC | 153 | 4 | 5193 | tss | 0.684078 | 0.349046 | Not queried | 2.78 | 6.86e-01 | 6.99e-01 |
|  | 15 | 25429176 | 25429408 | PDFSTATSENSEMBLUCSC | 233 | 5 | 13529 | tss | 0.063141 | 0.368727 | Not queried | 2.77 | 1.59e-02 | 5.36e-02 |
|  | 4 | 183370445 | 183370703 | PDFSTATSENSEMBLUCSC | 259 | 4 | 30192 | gene;island | 0.119698 | 0.012600 | Not queried | 2.77 | 1.14e-01 | 1.68e-01 |
|  | 3 | 39230030 | 39230320 | PDFSTATSENSEMBLUCSC | 291 | 4 | 27724 | gene | 0.391433 | 0.887831 | Not queried | 2.77 | 3.43e-01 | 3.84e-01 |
|  | 1 | 221051758 | 221052143 | PDFSTATSENSEMBLUCSC | 386 | 4 | 2263 | tss;island | 0.775803 | 0.967533 | Not queried | 2.77 | 2.00e-01 | 2.55e-01 |
|  | 19 | 40006192 | 40006625 | PDFSTATSENSEMBLUCSC | 434 | 4 | 20029 | gene;island | 0.478825 | 0.855417 | Not queried | 2.77 | 1.14e-01 | 1.68e-01 |
|  | 17 | 42084376 | 42084953 | PDFSTATSENSEMBLUCSC | 578 | 5 | 16602 | gene;island | 0.019242 | 0.011039 | Not queried | 2.77 | 6.90e-01 | 7.01e-01 |
|  | 8 | 49834354 | 49834643 | PDFSTATSENSEMBLUCSC | 290 | 4 | 39693 | tss;island | 0.039479 | 0.350606 | Not queried | 2.77 | 2.00e-01 | 2.55e-01 |
|  | 19 | 7267351 | 7267638 | PDFSTATSENSEMBLUCSC | 288 | 4 | 19775 | gene;island | 0.881193 | 0.983494 | Not queried | 2.77 | 5.71e-02 | 1.00e-01 |
|  | 6 | 19804711 | 19805404 | PDFSTATSENSEMBLUCSC | 694 | 7 | 35483 | island | 0.018793 | 0.008470 | Not queried | 2.77 | 3.18e-01 | 3.74e-01 |
|  | 6 | 33145396 | 33145571 | PDFSTATSENSEMBLUCSC | 176 | 4 | 34206 | gene | 0.631620 | 0.922759 | Not queried | 2.77 | 1.14e-01 | 1.68e-01 |
|  | 11 | 63803781 | 63804119 | PDFSTATSENSEMBLUCSC | 339 | 6 | 6659 | gene;island | 0.500129 | 0.908768 | RP11-21A7A.2; MACROD1 (margin: RP11-21A7A.2; MACROD1) | 2.77 | 8.66e-03 | 3.36e-02 |
|  | 19 | 55962948 | 55963317 | PDFSTATSENSEMBLUCSC | 370 | 4 | 22425 | island | 0.174768 | 0.686184 | Not queried | 2.77 | 5.71e-02 | 1.00e-01 |
|  | 16 | 12997467 | 12997684 | PDFSTATSENSEMBLUCSC | 218 | 4 | 14673 | gene;island | 0.883422 | 0.980967 | Not queried | 2.77 | 2.00e-01 | 2.55e-01 |
|  | 3 | 138679347 | 138679578 | PDFSTATSENSEMBLUCSC | 232 | 4 | 29531 | island | 0.851560 | 0.977753 | Not queried | 2.77 | 2.86e-02 | 6.12e-02 |
|  | 6 | 32063394 | 32064258 | PDFSTATSENSEMBLUCSC | 865 | 28 | 34046 | gene;island | 0.783652 | 0.981094 | TNXB; ATF6B (margin: TNXB; ATF6B) | 2.77 | 2.28e-06 | 1.96e-04 |
|  | 21 | 46912386 | 46912545 | PDFSTATSENSEMBLUCSC | 160 | 4 | 26470 | gene;island | 0.840001 | 0.981971 | Not queried | 2.77 | 1.14e-01 | 1.68e-01 |
|  | 17 | 79393463 | 79393718 | PDFSTATSENSEMBLUCSC | 256 | 5 | 16908 | gene;island | 0.516805 | 0.912538 | RP11-1055B8.7 (margin: RP11-1055B8.7) | 2.77 | 7.94e-03 | 3.19e-02 |
|  | 1 | 2483719 | 2483933 | PDFSTATSENSEMBLUCSC | 215 | 5 | 90 | gene | 0.787593 | 0.983749 | Not queried | 2.77 | 9.52e-02 | 1.53e-01 |
|  | 7 | 123673331 | 123673690 | PDFSTATSENSEMBLUCSC | 360 | 6 | 38879 | island | 0.634796 | 0.911726 | Not queried | 2.77 | 4.11e-02 | 8.14e-02 |
|  | 5 | 145722132 | 145722517 | PDFSTATSENSEMBLUCSC | 386 | 5 | 33282 | island | 0.768275 | 0.933756 | Not queried | 2.77 | 3.17e-02 | 6.50e-02 |
|  | 15 | 40633194 | 40633416 | PDFSTATSENSEMBLUCSC | 223 | 4 | 13586 | tss | 0.010749 | 0.026294 | Not queried | 2.77 | 4.86e-01 | 5.11e-01 |
|  | 6 | 137815539 | 137815725 | PDFSTATSENSEMBLUCSC | 187 | 6 | 35249 | tss;island | 0.500194 | 0.882738 | Not queried | 2.77 | 4.11e-02 | 8.14e-02 |
|  | 12 | 108991991 | 108992265 | PDFSTATSENSEMBLUCSC | 275 | 5 | 9984 | tss | 0.453928 | 0.888702 | Not queried | 2.77 | 1.51e-01 | 2.11e-01 |
|  | 16 | 84029457 | 84029838 | PDFSTATSENSEMBLUCSC | 382 | 5 | 14862 | gene;island | 0.890214 | 0.545649 | Not queried | 2.77 | 9.52e-02 | 1.53e-01 |
|  | 5 | 80256371 | 80256738 | PDFSTATSENSEMBLUCSC | 368 | 5 | 32925 | island | 0.777217 | 0.972353 | RASGRF2; CTC-459I6.1 (margin: RASGRF2; CTC-459I6.1) | 2.77 | 7.94e-03 | 3.19e-02 |
|  | 19 | 10381355 | 10382048 | PDFSTATSENSEMBLUCSC | 694 | 10 | 21525 | island | 0.501347 | 0.156403 | Not queried | 2.76 | 4.33e-02 | 8.55e-02 |
|  | 1 | 65730339 | 65730409 | PDFSTATSENSEMBLUCSC | 71 | 4 | 1676 | tss | 0.187166 | 0.531856 | Not queried | 2.76 | 2.86e-02 | 6.12e-02 |
|  | 5 | 92956494 | 92957098 | PDFSTATSENSEMBLUCSC | 605 | 9 | 32177 | tss | 0.038638 | 0.004885 | Not queried | 2.76 | 6.25e-02 | 1.09e-01 |
|  | 10 | 102590168 | 102590401 | PDFSTATSENSEMBLUCSC | 234 | 4 | 6036 | island | 0.727680 | 0.933062 | Not queried | 2.76 | 1.14e-01 | 1.68e-01 |
|  | 20 | 10199210 | 10199536 | PDFSTATSENSEMBLUCSC | 327 | 8 | 25999 | island | 0.041065 | 0.005844 | SNAP25; SNAP25-AS1 (margin: SNAP25; SNAP25-AS1) | 2.76 | 2.95e-03 | 1.88e-02 |
|  | 1 | 15256054 | 15256222 | PDFSTATSENSEMBLUCSC | 169 | 5 | 240 | gene;tss | 0.180590 | 0.599183 | Not queried | 2.76 | 1.59e-02 | 5.36e-02 |
|  | 13 | 111773936 | 111774127 | PDFSTATSENSEMBLUCSC | 192 | 4 | 11242 | gene | 0.412055 | 0.904481 | Not queried | 2.76 | 1.14e-01 | 1.68e-01 |
|  | 5 | 172482723 | 172482832 | PDFSTATSENSEMBLUCSC | 110 | 4 | 32500 | tss;island | 0.061321 | 0.008845 | Not queried | 2.76 | 2.86e-02 | 6.12e-02 |
|  | 19 | 41054942 | 41055297 | PDFSTATSENSEMBLUCSC | 356 | 4 | 20045 | gene;island | 0.511353 | 0.030759 | Not queried | 2.76 | 3.43e-01 | 3.84e-01 |
|  | 18 | 61637105 | 61637441 | PDFSTATSENSEMBLUCSC | 337 | 4 | 19558 | island | 0.004985 | 0.027470 | Not queried | 2.76 | 2.86e-02 | 6.12e-02 |
|  | 18 | 4454189 | 4454548 | PDFSTATSENSEMBLUCSC | 360 | 4 | 19367 | island | 0.937988 | 0.587200 | Not queried | 2.76 | 2.00e-01 | 2.55e-01 |
|  | 3 | 50541011 | 50541195 | PDFSTATSENSEMBLUCSC | 185 | 6 | 28358 | tss;island | 0.050653 | 0.006641 | Not queried | 2.76 | 4.11e-02 | 8.14e-02 |
|  | 8 | 114447055 | 114447871 | PDFSTATSENSEMBLUCSC | 817 | 6 | 39402 | gene;island | 0.193143 | 0.019306 | Not queried | 2.76 | 9.31e-02 | 1.53e-01 |
|  | 10 | 118386951 | 118387518 | PDFSTATSENSEMBLUCSC | 568 | 5 | 4776 | gene;island | 0.850996 | 0.973405 | Not queried | 2.76 | 5.56e-02 | 1.00e-01 |
|  | 4 | 174429263 | 174429649 | PDFSTATSENSEMBLUCSC | 387 | 7 | 31425 | island | 0.625699 | 0.966340 | Not queried | 2.76 | 1.28e-01 | 1.87e-01 |
|  | 7 | 4875911 | 4876228 | PDFSTATSENSEMBLUCSC | 318 | 4 | 36861 | gene;island | 0.822621 | 0.960231 | Not queried | 2.76 | 3.43e-01 | 3.84e-01 |
|  | 19 | 3825141 | 3825630 | PDFSTATSENSEMBLUCSC | 490 | 5 | 19724 | gene;island | 0.625203 | 0.948003 | Not queried | 2.76 | 9.52e-02 | 1.53e-01 |
|  | 22 | 48977177 | 48977448 | PDFSTATSENSEMBLUCSC | 272 | 4 | 26919 | gene;island | 0.429464 | 0.939567 | Not queried | 2.76 | 1.14e-01 | 1.68e-01 |
|  | 7 | 35292896 | 35293309 | PDFSTATSENSEMBLUCSC | 414 | 6 | 36963 | gene;island | 0.687405 | 0.919239 | TBX20 (margin: TBX20; AC009531.2) | 2.76 | 8.66e-03 | 3.36e-02 |
|  | 20 | 33880266 | 33880370 | PDFSTATSENSEMBLUCSC | 105 | 4 | 25704 | tss | 0.386872 | 0.775304 | Not queried | 2.75 | 2.86e-02 | 6.12e-02 |
|  | 11 | 113143664 | 113143807 | PDFSTATSENSEMBLUCSC | 144 | 4 | 6908 | gene | 0.741825 | 0.956531 | Not queried | 2.75 | 2.00e-01 | 2.55e-01 |
|  | 10 | 133644553 | 133644760 | PDFSTATSENSEMBLUCSC | 208 | 4 | 6279 | island | 0.153250 | 0.612645 | Not queried | 2.75 | 2.86e-02 | 6.12e-02 |
|  | 11 | 101454676 | 101454996 | PDFSTATSENSEMBLUCSC | 321 | 7 | 7643 | tss | 0.557563 | 0.157584 | Not queried | 2.75 | 2.09e-01 | 2.65e-01 |
|  | 17 | 1933006 | 1933418 | PDFSTATSENSEMBLUCSC | 413 | 9 | 16998 | tss | 0.015959 | 0.018487 | Not queried | 2.75 | 1.90e-01 | 2.55e-01 |
|  | 1 | 33115703 | 33116178 | PDFSTATSENSEMBLUCSC | 476 | 4 | 397 | gene | 0.017899 | 0.002789 | Not queried | 2.75 | 4.86e-01 | 5.11e-01 |
|  | 5 | 92956474 | 92957098 | PDFSTATSENSEMBLUCSC | 625 | 10 | 31691 | gene;island | 0.032760 | 0.004747 | Not queried | 2.75 | 1.05e-01 | 1.66e-01 |
|  | 1 | 158119395 | 158119673 | PDFSTATSENSEMBLUCSC | 279 | 5 | 3877 | island | 0.099997 | 0.427186 | Not queried | 2.75 | 3.17e-02 | 6.50e-02 |
|  | 19 | 19932565 | 19932702 | PDFSTATSENSEMBLUCSC | 138 | 6 | 20653 | tss | 0.491275 | 0.849045 | Not queried | 2.75 | 2.60e-02 | 6.12e-02 |
|  | 6 | 27101633 | 27101941 | PDFSTATSENSEMBLUCSC | 309 | 4 | 34712 | tss;island | 0.001703 | 0.008110 | Not queried | 2.75 | 1.14e-01 | 1.68e-01 |
|  | 15 | 28272148 | 28272656 | PDFSTATSENSEMBLUCSC | 509 | 4 | 13264 | gene | 0.780003 | 0.977985 | Not queried | 2.75 | 6.86e-01 | 6.99e-01 |
|  | 11 | 1854174 | 1854368 | PDFSTATSENSEMBLUCSC | 195 | 4 | 7046 | tss | 0.976913 | 0.756894 | Not queried | 2.75 | 5.71e-02 | 1.00e-01 |
|  | 7 | 157444235 | 157444501 | PDFSTATSENSEMBLUCSC | 267 | 5 | 37334 | gene;island | 0.434725 | 0.822038 | Not queried | 2.75 | 1.51e-01 | 2.11e-01 |
|  | 16 | 71496259 | 71496385 | PDFSTATSENSEMBLUCSC | 127 | 4 | 15346 | tss | 0.048375 | 0.334056 | Not queried | 2.75 | 2.00e-01 | 2.55e-01 |
|  | 13 | 113992056 | 113992391 | PDFSTATSENSEMBLUCSC | 336 | 4 | 11283 | gene | 0.702579 | 0.967975 | Not queried | 2.75 | 5.71e-02 | 1.00e-01 |
|  | 5 | 140588139 | 140588424 | PDFSTATSENSEMBLUCSC | 286 | 5 | 33224 | island | 0.488998 | 0.154653 | Not queried | 2.75 | 3.10e-01 | 3.66e-01 |
|  | 2 | 45029543 | 45030072 | PDFSTATSENSEMBLUCSC | 530 | 6 | 24257 | island | 0.888153 | 0.974396 | Not queried | 2.74 | 2.60e-02 | 6.12e-02 |
|  | 1 | 220863263 | 220863572 | PDFSTATSENSEMBLUCSC | 310 | 7 | 2261 | tss | 0.731541 | 0.174319 | Not queried | 2.74 | 1.28e-01 | 1.87e-01 |
|  | 19 | 58193071 | 58193426 | PDFSTATSENSEMBLUCSC | 356 | 10 | 22479 | island | 0.051022 | 0.008976 | Not queried | 2.74 | 2.32e-02 | 6.12e-02 |
|  | 19 | 58193071 | 58193344 | PDFSTATSENSEMBLUCSC | 274 | 8 | 21123 | tss | 0.061369 | 0.012965 | Not queried | 2.74 | 6.50e-02 | 1.11e-01 |
|  | 6 | 118241243 | 118241659 | PDFSTATSENSEMBLUCSC | 417 | 5 | 34456 | gene;island | 0.532364 | 0.789843 | Not queried | 2.74 | 2.22e-01 | 2.76e-01 |
|  | 1 | 94147461 | 94147831 | PDFSTATSENSEMBLUCSC | 371 | 4 | 1759 | tss;island | 0.006320 | 0.006010 | Not queried | 2.74 | 6.86e-01 | 6.99e-01 |
|  | 22 | 48027641 | 48027921 | PDFSTATSENSEMBLUCSC | 281 | 4 | 27599 | island | 0.065860 | 0.013105 | Not queried | 2.74 | 3.43e-01 | 3.84e-01 |
|  | 6 | 108496716 | 108497141 | PDFSTATSENSEMBLUCSC | 426 | 4 | 34441 | gene;island | 0.798856 | 0.940453 | Not queried | 2.74 | 5.71e-02 | 1.00e-01 |
|  | 22 | 22901830 | 22902237 | PDFSTATSENSEMBLUCSC | 408 | 6 | 26808 | gene | 0.559238 | 0.324438 | Not queried | 2.74 | 1.32e-01 | 1.89e-01 |
|  | 5 | 54281198 | 54281362 | PDFSTATSENSEMBLUCSC | 165 | 4 | 31614 | gene | 0.361815 | 0.047811 | Not queried | 2.74 | 5.71e-02 | 1.00e-01 |
|  | 2 | 66667039 | 66667101 | PDFSTATSENSEMBLUCSC | 63 | 5 | 22711 | gene | 0.912496 | 0.981233 | Not queried | 2.74 | 1.59e-02 | 5.36e-02 |
|  | 2 | 169658881 | 169659047 | PDFSTATSENSEMBLUCSC | 167 | 4 | 23699 | tss | 0.655520 | 0.968376 | Not queried | 2.74 | 5.71e-02 | 1.00e-01 |
|  | 7 | 99517192 | 99517509 | PDFSTATSENSEMBLUCSC | 318 | 10 | 37762 | tss | 0.056448 | 0.337407 | TRIM4 (margin: RP4-604G5.1; TRIM4; GJC3) | 2.74 | 1.30e-04 | 2.71e-03 |
|  | 7 | 134832770 | 134833299 | PDFSTATSENSEMBLUCSC | 530 | 7 | 37235 | gene | 0.088925 | 0.373062 | Not queried | 2.74 | 1.65e-01 | 2.28e-01 |
|  | 4 | 7395388 | 7395612 | PDFSTATSENSEMBLUCSC | 225 | 4 | 29936 | gene | 0.966498 | 0.878662 | Not queried | 2.74 | 3.43e-01 | 3.84e-01 |
|  | 1 | 39281613 | 39282003 | PDFSTATSENSEMBLUCSC | 391 | 4 | 3091 | island | 0.709887 | 0.921323 | Not queried | 2.74 | 2.00e-01 | 2.55e-01 |
|  | 8 | 136469480 | 136469780 | PDFSTATSENSEMBLUCSC | 301 | 4 | 40603 | island | 0.058970 | 0.007164 | Not queried | 2.74 | 1.14e-01 | 1.68e-01 |
|  | 6 | 33085470 | 33085800 | PDFSTATSENSEMBLUCSC | 331 | 8 | 34189 | gene;island | 0.429535 | 0.517611 | Not queried | 2.74 | 6.45e-01 | 6.69e-01 |
|  | 3 | 69788259 | 69788442 | PDFSTATSENSEMBLUCSC | 184 | 4 | 28441 | tss;island | 0.001837 | 0.006854 | Not queried | 2.74 | 3.43e-01 | 3.84e-01 |
|  | 17 | 56832804 | 56833425 | PDFSTATSENSEMBLUCSC | 622 | 13 | 18605 | island | 0.805132 | 0.375615 | PPM1E (margin: PPM1E) | 2.74 | 1.29e-05 | 5.58e-04 |
|  | 17 | 56832804 | 56833201 | PDFSTATSENSEMBLUCSC | 398 | 11 | 17540 | tss | 0.807239 | 0.383988 | PPM1E (margin: PPM1E) | 2.74 | 8.51e-05 | 2.07e-03 |
|  | 1 | 114471711 | 114471974 | PDFSTATSENSEMBLUCSC | 264 | 9 | 1847 | tss | 0.003018 | 0.024771 | HIPK1; RP5-1073O3.7 (margin: HIPK1; RP5-1073O3.7) | 2.74 | 1.06e-02 | 4.01e-02 |
|  | 10 | 8096669 | 8097354 | PDFSTATSENSEMBLUCSC | 686 | 6 | 4489 | gene | 0.059849 | 0.009440 | Not queried | 2.73 | 2.60e-02 | 6.12e-02 |
|  | 5 | 138534130 | 138534431 | PDFSTATSENSEMBLUCSC | 302 | 7 | 32327 | tss | 0.003946 | 0.053291 | Not queried | 2.73 | 2.09e-01 | 2.65e-01 |
|  | 4 | 21950567 | 21951046 | PDFSTATSENSEMBLUCSC | 480 | 6 | 30325 | tss;island | 0.853916 | 0.467618 | Not queried | 2.73 | 1.52e-02 | 5.36e-02 |
|  | 7 | 127225619 | 127225949 | PDFSTATSENSEMBLUCSC | 331 | 10 | 38885 | island | 0.272378 | 0.027657 | Not queried | 2.73 | 2.80e-01 | 3.40e-01 |
|  | 1 | 150947442 | 150947589 | PDFSTATSENSEMBLUCSC | 148 | 4 | 1915 | tss | 0.488766 | 0.063242 | Not queried | 2.73 | 3.43e-01 | 3.84e-01 |
|  | 7 | 117512803 | 117513101 | PDFSTATSENSEMBLUCSC | 299 | 4 | 37201 | gene;island | 0.642776 | 0.957973 | Not queried | 2.73 | 1.14e-01 | 1.68e-01 |
|  | 7 | 89840708 | 89841217 | PDFSTATSENSEMBLUCSC | 510 | 12 | 38646 | island | 0.136122 | 0.552131 | Not queried | 2.73 | 1.01e-01 | 1.60e-01 |
|  | 3 | 127056837 | 127057149 | PDFSTATSENSEMBLUCSC | 313 | 5 | 29442 | island | 0.873087 | 0.984161 | Not queried | 2.73 | 1.59e-02 | 5.36e-02 |
|  | 19 | 46174395 | 46174565 | PDFSTATSENSEMBLUCSC | 171 | 4 | 20104 | gene;island | 0.820536 | 0.437846 | Not queried | 2.73 | 2.00e-01 | 2.55e-01 |
|  | 19 | 1248287 | 1248507 | PDFSTATSENSEMBLUCSC | 221 | 5 | 20296 | tss | 0.008620 | 0.450955 | Not queried | 2.73 | 2.22e-01 | 2.76e-01 |
|  | 3 | 3842527 | 3842717 | PDFSTATSENSEMBLUCSC | 191 | 4 | 27639 | gene;island | 0.755977 | 0.955391 | Not queried | 2.73 | 5.71e-02 | 1.00e-01 |
|  | 6 | 26021768 | 26021843 | PDFSTATSENSEMBLUCSC | 76 | 4 | 34679 | tss | 0.004114 | 0.001224 | Not queried | 2.73 | 2.00e-01 | 2.55e-01 |
|  | 22 | 51038379 | 51038723 | PDFSTATSENSEMBLUCSC | 345 | 4 | 27262 | tss;island | 0.456082 | 0.268695 | Not queried | 2.73 | 3.43e-01 | 3.84e-01 |
|  | 12 | 119772354 | 119772577 | PDFSTATSENSEMBLUCSC | 224 | 5 | 10874 | island | 0.488178 | 0.695690 | Not queried | 2.73 | 3.10e-01 | 3.66e-01 |
|  | 19 | 52872916 | 52873125 | PDFSTATSENSEMBLUCSC | 210 | 6 | 21012 | tss | 0.462996 | 0.707911 | Not queried | 2.73 | 2.40e-01 | 2.95e-01 |
|  | 2 | 220070926 | 220071103 | PDFSTATSENSEMBLUCSC | 178 | 4 | 23894 | tss;island | 0.254810 | 0.043129 | Not queried | 2.73 | 2.00e-01 | 2.55e-01 |
|  | 7 | 56183885 | 56184217 | PDFSTATSENSEMBLUCSC | 333 | 4 | 38512 | island | 0.604974 | 0.876904 | Not queried | 2.73 | 1.14e-01 | 1.68e-01 |
|  | 1 | 102462806 | 102462877 | PDFSTATSENSEMBLUCSC | 72 | 4 | 1784 | tss | 0.432864 | 0.138501 | Not queried | 2.73 | 1.14e-01 | 1.68e-01 |
|  | 17 | 47210027 | 47210318 | PDFSTATSENSEMBLUCSC | 292 | 6 | 17482 | tss | 0.034342 | 0.006052 | Not queried | 2.73 | 2.60e-02 | 6.12e-02 |
|  | 2 | 176987161 | 176987411 | PDFSTATSENSEMBLUCSC | 251 | 5 | 23737 | tss | 0.842313 | 0.985631 | Not queried | 2.73 | 1.59e-02 | 5.36e-02 |
|  | 14 | 36983404 | 36983949 | PDFSTATSENSEMBLUCSC | 546 | 7 | 12357 | tss;island | 0.825406 | 0.879464 | Not queried | 2.73 | 2.09e-01 | 2.65e-01 |
|  | 11 | 3181446 | 3182119 | PDFSTATSENSEMBLUCSC | 674 | 6 | 6477 | gene;island | 0.843256 | 0.978179 | Not queried | 2.73 | 2.60e-02 | 6.12e-02 |
|  | 20 | 39321346 | 39321729 | PDFSTATSENSEMBLUCSC | 384 | 4 | 26164 | island | 0.312828 | 0.601460 | Not queried | 2.72 | 6.86e-01 | 6.99e-01 |
|  | 1 | 1309161 | 1309641 | PDFSTATSENSEMBLUCSC | 481 | 4 | 33 | gene;island | 0.479913 | 0.825467 | Not queried | 2.72 | 2.00e-01 | 2.55e-01 |
|  | 2 | 176947764 | 176948105 | PDFSTATSENSEMBLUCSC | 342 | 4 | 22934 | gene;island | 0.619895 | 0.912391 | Not queried | 2.72 | 2.00e-01 | 2.55e-01 |
|  | 5 | 77591420 | 77591744 | PDFSTATSENSEMBLUCSC | 325 | 4 | 32141 | tss;island | 0.068242 | 0.008474 | Not queried | 2.72 | 5.71e-02 | 1.00e-01 |
|  | 1 | 29213106 | 29213367 | PDFSTATSENSEMBLUCSC | 262 | 4 | 1437 | tss;island | 0.820208 | 0.314813 | Not queried | 2.72 | 2.00e-01 | 2.55e-01 |
|  | 20 | 57089933 | 57090317 | PDFSTATSENSEMBLUCSC | 385 | 10 | 26291 | island | 0.726587 | 0.919282 | APCDD1L-AS1; APCDD1L (margin: APCDD1L-AS1; APCDD1L) | 2.72 | 1.15e-02 | 4.29e-02 |
|  | 11 | 77122839 | 77123047 | PDFSTATSENSEMBLUCSC | 209 | 4 | 6840 | gene;island | 0.011840 | 0.146344 | Not queried | 2.72 | 3.43e-01 | 3.84e-01 |
|  | 5 | 43396802 | 43397288 | PDFSTATSENSEMBLUCSC | 487 | 7 | 31603 | gene;island | 0.817542 | 0.980732 | CCL28 (margin: CCL28) | 2.72 | 6.99e-03 | 3.19e-02 |
|  | 19 | 3480363 | 3480672 | PDFSTATSENSEMBLUCSC | 310 | 5 | 21330 | island | 0.509345 | 0.853835 | Not queried | 2.72 | 1.59e-02 | 5.36e-02 |
|  | 16 | 85878940 | 85879145 | PDFSTATSENSEMBLUCSC | 206 | 4 | 16166 | island | 0.593938 | 0.980653 | Not queried | 2.72 | 2.00e-01 | 2.55e-01 |
|  | 20 | 61583537 | 61583987 | PDFSTATSENSEMBLUCSC | 451 | 8 | 25886 | tss | 0.663194 | 0.947722 | SLC17A9 (margin: GID8; SLC17A9) | 2.72 | 6.99e-03 | 3.19e-02 |
|  | 11 | 70257035 | 70257280 | PDFSTATSENSEMBLUCSC | 246 | 4 | 6789 | gene;island | 0.924240 | 0.985266 | Not queried | 2.72 | 2.00e-01 | 2.55e-01 |
|  | 12 | 1609245 | 1609549 | PDFSTATSENSEMBLUCSC | 305 | 8 | 9483 | tss | 0.665197 | 0.944500 | Not queried | 2.72 | 2.81e-02 | 6.12e-02 |
|  | 19 | 31847893 | 31848310 | PDFSTATSENSEMBLUCSC | 418 | 6 | 21863 | island | 0.761975 | 0.941861 | Not queried | 2.72 | 1.32e-01 | 1.89e-01 |
|  | 6 | 33147499 | 33147659 | PDFSTATSENSEMBLUCSC | 161 | 4 | 34208 | gene | 0.411130 | 0.891542 | Not queried | 2.72 | 1.14e-01 | 1.68e-01 |
|  | 11 | 66084469 | 66084841 | PDFSTATSENSEMBLUCSC | 373 | 5 | 8506 | island | 0.671798 | 0.903782 | Not queried | 2.72 | 3.17e-02 | 6.50e-02 |
|  | 5 | 138533871 | 138534431 | PDFSTATSENSEMBLUCSC | 561 | 9 | 33164 | island | 0.003946 | 0.025152 | Not queried | 2.72 | 1.36e-01 | 1.94e-01 |
|  | 6 | 153452724 | 153452954 | PDFSTATSENSEMBLUCSC | 231 | 4 | 35310 | tss;island | 0.925456 | 0.729663 | Not queried | 2.72 | 3.43e-01 | 3.84e-01 |
|  | 21 | 43736736 | 43737065 | PDFSTATSENSEMBLUCSC | 330 | 4 | 26566 | tss | 0.602359 | 0.936635 | Not queried | 2.72 | 1.14e-01 | 1.68e-01 |
|  | 20 | 23402196 | 23402404 | PDFSTATSENSEMBLUCSC | 209 | 8 | 25639 | tss | 0.001903 | 0.004748 | Not queried | 2.72 | 2.07e-02 | 6.12e-02 |
|  | 10 | 126138593 | 126138879 | PDFSTATSENSEMBLUCSC | 287 | 6 | 5422 | tss | 0.107762 | 0.463447 | RP13-238F13.3; NKX1-2 (margin: RP13-238F13.3; LHPP; RP13-238F13.5; NKX1-2) | 2.72 | 2.16e-03 | 1.45e-02 |
|  | 1 | 161695659 | 161696302 | PDFSTATSENSEMBLUCSC | 644 | 7 | 854 | gene;island | 0.143581 | 0.025693 | Not queried | 2.72 | 1.65e-01 | 2.28e-01 |
|  | 1 | 50893469 | 50893984 | PDFSTATSENSEMBLUCSC | 516 | 5 | 3254 | island | 0.731121 | 0.942620 | Not queried | 2.72 | 2.22e-01 | 2.76e-01 |
|  | 11 | 45928841 | 45928988 | PDFSTATSENSEMBLUCSC | 148 | 6 | 7271 | tss | 0.189718 | 0.505821 | Not queried | 2.72 | 2.60e-02 | 6.12e-02 |
|  | 1 | 143467660 | 143467856 | PDFSTATSENSEMBLUCSC | 197 | 4 | 3670 | island | 0.319540 | 0.099883 | Not queried | 2.72 | 2.86e-02 | 6.12e-02 |
|  | 17 | 50236344 | 50236765 | PDFSTATSENSEMBLUCSC | 422 | 4 | 16697 | gene;tss;island | 0.863997 | 0.202450 | Not queried | 2.72 | 5.71e-02 | 1.00e-01 |
|  | 7 | 114561804 | 114562063 | PDFSTATSENSEMBLUCSC | 260 | 5 | 37847 | tss;island | 0.972742 | 0.758808 | Not queried | 2.71 | 3.10e-01 | 3.66e-01 |
|  | 14 | 31677219 | 31677716 | PDFSTATSENSEMBLUCSC | 498 | 5 | 12341 | tss;island | 0.004450 | 0.013825 | Not queried | 2.71 | 1.51e-01 | 2.11e-01 |
|  | 22 | 19949873 | 19950040 | PDFSTATSENSEMBLUCSC | 168 | 6 | 26970 | tss | 0.140734 | 0.032224 | Not queried | 2.71 | 9.31e-02 | 1.53e-01 |
|  | 3 | 127347343 | 127347978 | PDFSTATSENSEMBLUCSC | 636 | 8 | 28565 | tss | 0.795664 | 0.528482 | Not queried | 2.71 | 7.21e-01 | 7.30e-01 |
|  | 3 | 62861544 | 62861925 | PDFSTATSENSEMBLUCSC | 382 | 4 | 28429 | tss;island | 0.878066 | 0.378801 | Not queried | 2.71 | 2.00e-01 | 2.55e-01 |
|  | 17 | 3438857 | 3439372 | PDFSTATSENSEMBLUCSC | 516 | 7 | 16335 | gene;island | 0.901378 | 0.988272 | TRPV3 (margin: TRPV3) | 2.71 | 6.99e-03 | 3.19e-02 |
|  | 1 | 109656708 | 109657035 | PDFSTATSENSEMBLUCSC | 328 | 5 | 665 | gene;tss;island | 0.357968 | 0.048760 | KIAA1324; C1orf194 (margin: KIAA1324; RP5-1065J22.4; C1orf194) | 2.71 | 7.94e-03 | 3.19e-02 |
|  | 3 | 33138872 | 33139098 | PDFSTATSENSEMBLUCSC | 227 | 4 | 28210 | tss;island | 0.021979 | 0.007535 | Not queried | 2.71 | 4.86e-01 | 5.11e-01 |
|  | 4 | 187065417 | 187065955 | PDFSTATSENSEMBLUCSC | 539 | 9 | 30704 | tss;island | 0.128732 | 0.021175 | Not queried | 2.71 | 1.13e-01 | 1.68e-01 |
|  | 7 | 26191458 | 26191726 | PDFSTATSENSEMBLUCSC | 269 | 6 | 37481 | tss;island | 0.068728 | 0.486789 | Not queried | 2.71 | 1.32e-01 | 1.89e-01 |
|  | 19 | 10698020 | 10698098 | PDFSTATSENSEMBLUCSC | 79 | 4 | 20453 | tss | 0.056371 | 0.007134 | Not queried | 2.71 | 1.14e-01 | 1.68e-01 |
|  | 11 | 129245558 | 129245744 | PDFSTATSENSEMBLUCSC | 187 | 7 | 7811 | tss | 0.271556 | 0.030298 | Not queried | 2.71 | 1.28e-01 | 1.87e-01 |
|  | 11 | 129245558 | 129245988 | PDFSTATSENSEMBLUCSC | 431 | 9 | 8947 | island | 0.031381 | 0.027901 | Not queried | 2.71 | 2.58e-01 | 3.16e-01 |
|  | 13 | 102104573 | 102105129 | PDFSTATSENSEMBLUCSC | 557 | 9 | 11856 | island | 0.904815 | 0.529329 | Not queried | 2.71 | 2.44e-02 | 6.12e-02 |
|  | 12 | 105723904 | 105724534 | PDFSTATSENSEMBLUCSC | 631 | 8 | 10745 | island | 0.348538 | 0.077615 | Not queried | 2.71 | 8.30e-02 | 1.40e-01 |
|  | 12 | 105723904 | 105724410 | PDFSTATSENSEMBLUCSC | 507 | 6 | 9965 | tss | 0.579802 | 0.206541 | Not queried | 2.71 | 1.32e-01 | 1.89e-01 |
|  | 2 | 219827660 | 219827930 | PDFSTATSENSEMBLUCSC | 271 | 4 | 25097 | island | 0.040543 | 0.224691 | Not queried | 2.71 | 3.43e-01 | 3.84e-01 |
|  | 10 | 125732604 | 125732842 | PDFSTATSENSEMBLUCSC | 239 | 4 | 6222 | island | 0.757979 | 0.963306 | Not queried | 2.71 | 1.14e-01 | 1.68e-01 |
|  | 2 | 55450577 | 55450874 | PDFSTATSENSEMBLUCSC | 298 | 4 | 22686 | gene;island | 0.113958 | 0.474700 | Not queried | 2.71 | 2.86e-02 | 6.12e-02 |
|  | 16 | 28550525 | 28550637 | PDFSTATSENSEMBLUCSC | 113 | 4 | 15155 | tss | 0.485186 | 0.795486 | Not queried | 2.71 | 3.43e-01 | 3.84e-01 |
|  | 11 | 111383515 | 111383668 | PDFSTATSENSEMBLUCSC | 154 | 6 | 7685 | tss;island | 0.804070 | 0.964092 | Not queried | 2.70 | 2.60e-02 | 6.12e-02 |
|  | 2 | 242844601 | 242844943 | PDFSTATSENSEMBLUCSC | 343 | 4 | 25315 | island | 0.728644 | 0.950038 | Not queried | 2.70 | 2.86e-02 | 6.12e-02 |
|  | 1 | 19970204 | 19970337 | PDFSTATSENSEMBLUCSC | 134 | 4 | 284 | gene;tss;island | 0.915619 | 0.985015 | Not queried | 2.70 | 2.86e-02 | 6.12e-02 |
|  | 5 | 178017260 | 178018186 | PDFSTATSENSEMBLUCSC | 927 | 13 | 33471 | island | 0.679628 | 0.926302 | COL23A1 (margin: COL23A1; CLK4) | 2.70 | 1.20e-02 | 4.46e-02 |
|  | 6 | 167507447 | 167507762 | PDFSTATSENSEMBLUCSC | 316 | 4 | 36641 | island | 0.587254 | 0.918404 | Not queried | 2.70 | 2.86e-02 | 6.12e-02 |
|  | 17 | 35289351 | 35289875 | PDFSTATSENSEMBLUCSC | 525 | 5 | 18234 | island | 0.859511 | 0.718961 | Not queried | 2.70 | 2.22e-01 | 2.76e-01 |
|  | 5 | 112824614 | 112824920 | PDFSTATSENSEMBLUCSC | 307 | 6 | 32219 | tss | 0.021510 | 0.188178 | Not queried | 2.70 | 1.80e-01 | 2.45e-01 |
|  | 19 | 41531634 | 41531976 | PDFSTATSENSEMBLUCSC | 343 | 4 | 22044 | island | 0.725018 | 0.961257 | Not queried | 2.70 | 2.86e-02 | 6.12e-02 |
|  | 17 | 79304498 | 79304683 | PDFSTATSENSEMBLUCSC | 186 | 7 | 17737 | tss | 0.910714 | 0.650869 | TMEM105 (margin: TMEM105) | 2.70 | 5.83e-04 | 6.47e-03 |
|  | 2 | 132431211 | 132431520 | PDFSTATSENSEMBLUCSC | 310 | 4 | 24736 | island | 0.331445 | 0.114740 | Not queried | 2.70 | 2.00e-01 | 2.55e-01 |
|  | 19 | 40732691 | 40732902 | PDFSTATSENSEMBLUCSC | 212 | 8 | 20784 | tss | 0.416655 | 0.097025 | CNTD2 (margin: MAP3K10; TTC9B; CNTD2; AKT2) | 2.70 | 1.55e-04 | 2.71e-03 |
|  | 11 | 32421514 | 32421845 | PDFSTATSENSEMBLUCSC | 332 | 6 | 6564 | gene | 0.793069 | 0.951952 | Not queried | 2.70 | 2.60e-02 | 6.12e-02 |
|  | 19 | 45737483 | 45737880 | PDFSTATSENSEMBLUCSC | 398 | 8 | 20864 | tss;island | 0.924767 | 0.989190 | Not queried | 2.70 | 6.50e-02 | 1.11e-01 |
|  | 19 | 4607057 | 4607324 | PDFSTATSENSEMBLUCSC | 268 | 5 | 21382 | island | 0.811563 | 0.906537 | Not queried | 2.70 | 1.51e-01 | 2.11e-01 |
|  | 1 | 20988073 | 20988237 | PDFSTATSENSEMBLUCSC | 165 | 5 | 1317 | tss;island | 0.040222 | 0.004421 | Not queried | 2.70 | 5.56e-02 | 1.00e-01 |
|  | 19 | 21860753 | 21860916 | PDFSTATSENSEMBLUCSC | 164 | 4 | 21837 | island | 0.336386 | 0.849429 | Not queried | 2.70 | 1.14e-01 | 1.68e-01 |
|  | 7 | 72722923 | 72723152 | PDFSTATSENSEMBLUCSC | 230 | 5 | 37631 | tss | 0.005949 | 0.002788 | Not queried | 2.70 | 5.48e-01 | 5.70e-01 |
|  | 4 | 185872527 | 185872977 | PDFSTATSENSEMBLUCSC | 451 | 4 | 31464 | island | 0.797463 | 0.970231 | Not queried | 2.70 | 2.00e-01 | 2.55e-01 |
|  | 19 | 590409 | 590541 | PDFSTATSENSEMBLUCSC | 133 | 4 | 19630 | gene;island | 0.512671 | 0.046077 | Not queried | 2.70 | 1.14e-01 | 1.68e-01 |
|  | 1 | 209979111 | 209979779 | PDFSTATSENSEMBLUCSC | 669 | 13 | 4167 | island | 0.056728 | 0.009393 | IRF6 (margin: IRF6) | 2.70 | 3.49e-03 | 2.20e-02 |
|  | 1 | 3182620 | 3183165 | PDFSTATSENSEMBLUCSC | 546 | 5 | 114 | gene;island | 0.300629 | 0.953284 | Not queried | 2.70 | 9.52e-02 | 1.53e-01 |
|  | 16 | 10275767 | 10276119 | PDFSTATSENSEMBLUCSC | 353 | 5 | 14658 | gene;island | 0.119076 | 0.464712 | GRIN2A (margin: GRIN2A) | 2.69 | 7.94e-03 | 3.19e-02 |
|  | 12 | 16757954 | 16758267 | PDFSTATSENSEMBLUCSC | 314 | 6 | 9071 | gene | 0.908230 | 0.981542 | Not queried | 2.69 | 1.52e-02 | 5.36e-02 |
|  | 7 | 44365289 | 44365627 | PDFSTATSENSEMBLUCSC | 339 | 8 | 37567 | tss;island | 0.154695 | 0.032935 | CAMK2B (margin: CAMK2B) | 2.69 | 6.99e-03 | 3.19e-02 |
|  | 5 | 1416793 | 1417003 | PDFSTATSENSEMBLUCSC | 211 | 4 | 31538 | gene;island | 0.742504 | 0.947141 | Not queried | 2.69 | 5.71e-02 | 1.00e-01 |
|  | 14 | 61188077 | 61188431 | PDFSTATSENSEMBLUCSC | 355 | 4 | 12104 | gene;island | 0.072840 | 0.324641 | Not queried | 2.69 | 2.00e-01 | 2.55e-01 |
|  | 11 | 3173868 | 3174128 | PDFSTATSENSEMBLUCSC | 261 | 5 | 6475 | gene | 0.898867 | 0.946468 | Not queried | 2.69 | 5.56e-02 | 1.00e-01 |
|  | 17 | 7153814 | 7154245 | PDFSTATSENSEMBLUCSC | 432 | 4 | 16387 | gene;island | 0.022527 | 0.203168 | Not queried | 2.69 | 4.86e-01 | 5.11e-01 |
|  | 16 | 49311698 | 49312033 | PDFSTATSENSEMBLUCSC | 336 | 6 | 15906 | island | 0.626798 | 0.886666 | Not queried | 2.69 | 3.94e-01 | 4.36e-01 |
|  | 1 | 66999588 | 66999929 | PDFSTATSENSEMBLUCSC | 342 | 5 | 3360 | island | 0.801534 | 0.976606 | Not queried | 2.69 | 5.56e-02 | 1.00e-01 |
|  | 1 | 2984245 | 2984869 | PDFSTATSENSEMBLUCSC | 625 | 9 | 1178 | tss | 0.904245 | 0.974739 | PRDM16; LINC00982 (margin: PRDM16; LINC00982; AL008733.1) | 2.69 | 7.82e-04 | 8.17e-03 |
|  | 2 | 118616155 | 118616576 | PDFSTATSENSEMBLUCSC | 422 | 5 | 24638 | island | 0.603215 | 0.760444 | Not queried | 2.69 | 4.21e-01 | 4.59e-01 |
|  | 8 | 101661918 | 101662246 | PDFSTATSENSEMBLUCSC | 329 | 7 | 39844 | tss | 0.895678 | 0.975119 | SNX31 (margin: SNX31) | 2.69 | 1.11e-02 | 4.14e-02 |
|  | 5 | 2754753 | 2755158 | PDFSTATSENSEMBLUCSC | 406 | 4 | 31551 | gene;island | 0.902421 | 0.984013 | Not queried | 2.69 | 5.71e-02 | 1.00e-01 |
|  | 5 | 112824497 | 112824920 | PDFSTATSENSEMBLUCSC | 424 | 7 | 33013 | island | 0.023441 | 0.137809 | Not queried | 2.69 | 1.65e-01 | 2.28e-01 |
|  | 12 | 125399699 | 125399964 | PDFSTATSENSEMBLUCSC | 266 | 5 | 10110 | tss;island | 0.000769 | 0.012120 | Not queried | 2.69 | 9.52e-02 | 1.53e-01 |
|  | 3 | 33260732 | 33261204 | PDFSTATSENSEMBLUCSC | 473 | 7 | 28212 | tss;island | 0.908936 | 0.973646 | Not queried | 2.69 | 3.83e-01 | 4.27e-01 |
|  | 12 | 68041257 | 68041428 | PDFSTATSENSEMBLUCSC | 172 | 4 | 9852 | tss;island | 0.398536 | 0.721634 | Not queried | 2.69 | 2.00e-01 | 2.55e-01 |
|  | 7 | 93204959 | 93205295 | PDFSTATSENSEMBLUCSC | 337 | 11 | 37714 | tss;island | 0.025972 | 0.154391 | CALCR (margin: CALCR) | 2.69 | 1.90e-04 | 3.21e-03 |
|  | 14 | 70655686 | 70656116 | PDFSTATSENSEMBLUCSC | 431 | 9 | 12976 | island | 0.620561 | 0.207382 | RP11-486O13.2; SLC8A3 (margin: RP11-486O13.2; SLC8A3) | 2.69 | 1.85e-03 | 1.45e-02 |
|  | 1 | 146556239 | 146556859 | PDFSTATSENSEMBLUCSC | 621 | 5 | 3691 | island | 0.111727 | 0.450950 | NBPF13P; RP11-325P15.2 (margin: RNVU1-8; NBPF13P; RP11-325P15.2) | 2.69 | 7.94e-03 | 3.19e-02 |
|  | 10 | 124713766 | 124714384 | PDFSTATSENSEMBLUCSC | 619 | 10 | 6212 | island | 0.594491 | 0.036307 | Not queried | 2.69 | 4.33e-02 | 8.55e-02 |
|  | 2 | 220363628 | 220363862 | PDFSTATSENSEMBLUCSC | 235 | 4 | 23035 | gene | 0.002467 | 0.008453 | Not queried | 2.69 | 3.43e-01 | 3.84e-01 |
|  | 13 | 36872048 | 36872346 | PDFSTATSENSEMBLUCSC | 299 | 8 | 11393 | tss | 0.439499 | 0.731273 | SOHLH2; CCDC169-SOHLH2; CCDC169 (margin: SOHLH2; CCDC169-SOHLH2; CCDC169; SPG20) | 2.68 | 2.95e-03 | 1.88e-02 |
|  | 12 | 132905611 | 132906010 | PDFSTATSENSEMBLUCSC | 400 | 4 | 11042 | island | 0.910510 | 0.596554 | Not queried | 2.68 | 3.43e-01 | 3.84e-01 |
|  | 8 | 11565277 | 11565725 | PDFSTATSENSEMBLUCSC | 449 | 4 | 39205 | gene;island | 0.590079 | 0.917899 | Not queried | 2.68 | 1.14e-01 | 1.68e-01 |
|  | 12 | 52300911 | 52301163 | PDFSTATSENSEMBLUCSC | 253 | 4 | 9721 | tss | 0.748623 | 0.935111 | Not queried | 2.68 | 2.00e-01 | 2.55e-01 |
|  | 17 | 77903985 | 77904396 | PDFSTATSENSEMBLUCSC | 412 | 5 | 18847 | island | 0.647114 | 0.917438 | Not queried | 2.68 | 1.59e-02 | 5.36e-02 |
|  | 6 | 31831340 | 31831890 | PDFSTATSENSEMBLUCSC | 551 | 16 | 33975 | gene;tss;island | 0.083374 | 0.011966 | NEU1; SLC44A4 (margin: NEU1; SLC44A4) | 2.68 | 7.70e-04 | 8.17e-03 |
|  | 18 | 20735537 | 20735693 | PDFSTATSENSEMBLUCSC | 157 | 4 | 19071 | gene;tss | 0.833186 | 0.364506 | Not queried | 2.68 | 3.43e-01 | 3.84e-01 |
|  | 11 | 49579997 | 49580074 | PDFSTATSENSEMBLUCSC | 78 | 4 | 7295 | tss | 0.901028 | 0.620890 | Not queried | 2.68 | 2.86e-02 | 6.12e-02 |
|  | 12 | 52627272 | 52627576 | PDFSTATSENSEMBLUCSC | 305 | 4 | 9135 | gene;island | 0.831182 | 0.976333 | Not queried | 2.68 | 1.14e-01 | 1.68e-01 |
|  | 7 | 96654425 | 96654572 | PDFSTATSENSEMBLUCSC | 148 | 4 | 37736 | tss;island | 0.153439 | 0.528440 | Not queried | 2.68 | 5.71e-02 | 1.00e-01 |
|  | 1 | 32230048 | 32230251 | PDFSTATSENSEMBLUCSC | 204 | 5 | 1450 | tss;island | 0.087014 | 0.011767 | Not queried | 2.68 | 2.22e-01 | 2.76e-01 |
|  | 10 | 88728073 | 88728361 | PDFSTATSENSEMBLUCSC | 289 | 6 | 5909 | island | 0.056204 | 0.012410 | Not queried | 2.68 | 2.60e-02 | 6.12e-02 |
|  | 6 | 100054469 | 100055173 | PDFSTATSENSEMBLUCSC | 705 | 11 | 36357 | island | 0.787069 | 0.981094 | Not queried | 2.68 | 1.92e-02 | 6.12e-02 |
|  | 6 | 33036464 | 33036717 | PDFSTATSENSEMBLUCSC | 254 | 6 | 34180 | gene | 0.509406 | 0.871870 | Not queried | 2.68 | 2.60e-02 | 6.12e-02 |
|  | 2 | 186603398 | 186603639 | PDFSTATSENSEMBLUCSC | 242 | 4 | 24947 | island | 0.893364 | 0.597742 | Not queried | 2.68 | 2.86e-02 | 6.12e-02 |
|  | 13 | 109779684 | 109779925 | PDFSTATSENSEMBLUCSC | 242 | 4 | 11225 | gene;island | 0.848589 | 0.976773 | Not queried | 2.68 | 1.14e-01 | 1.68e-01 |
|  | 13 | 114793125 | 114793396 | PDFSTATSENSEMBLUCSC | 272 | 4 | 11308 | gene | 0.702727 | 0.934294 | Not queried | 2.68 | 2.86e-02 | 6.12e-02 |
|  | 1 | 112531263 | 112532085 | PDFSTATSENSEMBLUCSC | 823 | 11 | 3599 | island | 0.728895 | 0.321802 | Not queried | 2.68 | 4.00e-02 | 8.03e-02 |
|  | 15 | 90437601 | 90437891 | PDFSTATSENSEMBLUCSC | 291 | 6 | 14443 | island | 0.098837 | 0.016639 | Not queried | 2.68 | 2.40e-01 | 2.95e-01 |
|  | 12 | 4554295 | 4554971 | PDFSTATSENSEMBLUCSC | 677 | 12 | 10175 | island | 0.434427 | 0.849569 | Not queried | 2.68 | 3.32e-02 | 6.81e-02 |
|  | 1 | 214170114 | 214170376 | PDFSTATSENSEMBLUCSC | 263 | 5 | 1005 | gene | 0.827730 | 0.960563 | Not queried | 2.68 | 2.22e-01 | 2.76e-01 |
|  | 16 | 85845626 | 85845936 | PDFSTATSENSEMBLUCSC | 311 | 4 | 16165 | island | 0.520121 | 0.876182 | Not queried | 2.68 | 2.86e-02 | 6.12e-02 |
|  | 8 | 71316578 | 71316869 | PDFSTATSENSEMBLUCSC | 292 | 5 | 39759 | tss;island | 0.003019 | 0.004369 | Not queried | 2.68 | 4.21e-01 | 4.59e-01 |
|  | 12 | 125002940 | 125003558 | PDFSTATSENSEMBLUCSC | 619 | 7 | 9401 | gene;island | 0.005245 | 0.085439 | Not queried | 2.68 | 5.30e-02 | 1.00e-01 |
|  | 2 | 24162256 | 24162667 | PDFSTATSENSEMBLUCSC | 412 | 5 | 23219 | tss;island | 0.503227 | 0.922705 | Not queried | 2.68 | 9.52e-02 | 1.53e-01 |
|  | 19 | 46974886 | 46975180 | PDFSTATSENSEMBLUCSC | 295 | 7 | 20892 | tss;island | 0.826160 | 0.979507 | Not queried | 2.68 | 1.75e-02 | 5.79e-02 |
|  | 1 | 223899685 | 223899998 | PDFSTATSENSEMBLUCSC | 314 | 7 | 1025 | gene;tss;island | 0.381015 | 0.941892 | Not queried | 2.67 | 5.35e-01 | 5.62e-01 |
|  | 2 | 220110328 | 220110550 | PDFSTATSENSEMBLUCSC | 223 | 4 | 23028 | gene;tss;island | 0.016515 | 0.027938 | Not queried | 2.67 | 4.86e-01 | 5.11e-01 |
|  | 1 | 42618891 | 42619087 | PDFSTATSENSEMBLUCSC | 197 | 4 | 1546 | tss | 0.521764 | 0.892507 | Not queried | 2.67 | 2.00e-01 | 2.55e-01 |
|  | 19 | 54385215 | 54385441 | PDFSTATSENSEMBLUCSC | 227 | 7 | 21044 | tss;island | 0.068374 | 0.023829 | Not queried | 2.67 | 9.73e-02 | 1.55e-01 |
|  | 12 | 100967016 | 100967381 | PDFSTATSENSEMBLUCSC | 366 | 6 | 9942 | tss | 0.184485 | 0.037757 | Not queried | 2.67 | 1.80e-01 | 2.45e-01 |
|  | 6 | 13274151 | 13274354 | PDFSTATSENSEMBLUCSC | 204 | 5 | 33575 | gene | 0.470133 | 0.972590 | Not queried | 2.67 | 3.17e-02 | 6.50e-02 |
|  | 1 | 47695138 | 47695475 | PDFSTATSENSEMBLUCSC | 338 | 4 | 3230 | island | 0.822323 | 0.953445 | Not queried | 2.67 | 3.43e-01 | 3.84e-01 |
|  | 18 | 76782568 | 76782895 | PDFSTATSENSEMBLUCSC | 328 | 5 | 19594 | island | 0.953823 | 0.983153 | Not queried | 2.67 | 3.10e-01 | 3.66e-01 |
|  | 20 | 26188908 | 26188997 | PDFSTATSENSEMBLUCSC | 90 | 7 | 26067 | island | 0.897927 | 0.975242 | Not queried | 2.67 | 7.28e-02 | 1.23e-01 |
|  | 20 | 21000809 | 21001266 | PDFSTATSENSEMBLUCSC | 458 | 4 | 26037 | island | 0.837531 | 0.962682 | Not queried | 2.67 | 2.00e-01 | 2.55e-01 |
|  | 11 | 2871400 | 2871992 | PDFSTATSENSEMBLUCSC | 593 | 9 | 7973 | island | 0.542421 | 0.928233 | Not queried | 2.67 | 3.15e-02 | 6.50e-02 |
|  | 7 | 75931606 | 75931843 | PDFSTATSENSEMBLUCSC | 238 | 8 | 37662 | tss | 0.161966 | 0.027326 | Not queried | 2.67 | 2.81e-02 | 6.12e-02 |
|  | 11 | 17373103 | 17373302 | PDFSTATSENSEMBLUCSC | 200 | 6 | 7174 | tss;island | 0.098264 | 0.018108 | NUCB2; NCR3LG1 (margin: NUCB2; NCR3LG1) | 2.67 | 4.33e-03 | 2.52e-02 |
|  | 6 | 130339410 | 130339725 | PDFSTATSENSEMBLUCSC | 316 | 7 | 35221 | tss | 0.162389 | 0.539565 | Not queried | 2.67 | 2.62e-02 | 6.12e-02 |
|  | 6 | 31276088 | 31276212 | PDFSTATSENSEMBLUCSC | 125 | 5 | 35763 | island | 0.388840 | 0.246310 | Not queried | 2.67 | 9.52e-02 | 1.53e-01 |
|  | 1 | 47696212 | 47696788 | PDFSTATSENSEMBLUCSC | 577 | 6 | 1607 | tss;island | 0.855688 | 0.978024 | RP1-18D14.7; TAL1 (margin: RP1-18D14.7; TAL1) | 2.67 | 4.33e-03 | 2.52e-02 |
|  | 14 | 94088174 | 94088604 | PDFSTATSENSEMBLUCSC | 431 | 4 | 12191 | gene | 0.608496 | 0.956615 | Not queried | 2.67 | 4.86e-01 | 5.11e-01 |
|  | 12 | 133532874 | 133533354 | PDFSTATSENSEMBLUCSC | 481 | 7 | 10135 | tss;island | 0.013801 | 0.203317 | CHFR; ZNF605 (margin: CHFR; ZNF605) | 2.67 | 6.99e-03 | 3.19e-02 |
|  | 18 | 76552701 | 76552971 | PDFSTATSENSEMBLUCSC | 271 | 4 | 19592 | island | 0.661925 | 0.946500 | Not queried | 2.67 | 2.86e-02 | 6.12e-02 |
|  | 8 | 101118377 | 101118548 | PDFSTATSENSEMBLUCSC | 172 | 4 | 39839 | tss;island | 0.872319 | 0.972457 | Not queried | 2.67 | 2.86e-02 | 6.12e-02 |
|  | 17 | 59940826 | 59941132 | PDFSTATSENSEMBLUCSC | 307 | 7 | 17559 | tss | 0.003655 | 0.021909 | Not queried | 2.66 | 9.73e-02 | 1.55e-01 |
|  | 7 | 63767753 | 63768139 | PDFSTATSENSEMBLUCSC | 387 | 4 | 38524 | island | 0.806412 | 0.398690 | Not queried | 2.66 | 2.00e-01 | 2.55e-01 |
|  | 13 | 112547341 | 112547653 | PDFSTATSENSEMBLUCSC | 313 | 4 | 11903 | island | 0.475732 | 0.913242 | Not queried | 2.66 | 5.71e-02 | 1.00e-01 |
|  | 1 | 78511140 | 78512129 | PDFSTATSENSEMBLUCSC | 990 | 12 | 3402 | island | 0.860338 | 0.980520 | GIPC2; RP11-386I14.2 (margin: GIPC2; RP11-386I14.2) | 2.66 | 3.71e-04 | 5.59e-03 |
|  | 8 | 27468981 | 27469338 | PDFSTATSENSEMBLUCSC | 358 | 5 | 39257 | gene;tss;island | 0.820086 | 0.969287 | CLU (margin: CLU) | 2.66 | 7.94e-03 | 3.19e-02 |
|  | 14 | 101526485 | 101527144 | PDFSTATSENSEMBLUCSC | 660 | 10 | 12631 | tss | 0.600021 | 0.900026 | MIR154; MIR496; MIR377 (margin: MIR381HG; MIR544A; MIR655; AL132709.3; MIR487A; MIR134; MIR485; MIR323B; MIR154; MIR496; MIR377; MIR541; MIR409; MIR412; MIR369; MIR410; MIR656; MEG9; MIR382) | 2.66 | 1.15e-02 | 4.29e-02 |
|  | 16 | 332515 | 333104 | PDFSTATSENSEMBLUCSC | 590 | 7 | 14964 | tss | 0.606092 | 0.924156 | Not queried | 2.66 | 1.65e-01 | 2.28e-01 |
|  | 14 | 96505296 | 96505874 | PDFSTATSENSEMBLUCSC | 579 | 12 | 13114 | island | 0.130796 | 0.018927 | C14orf132 (margin: C14orf132) | 2.66 | 4.96e-04 | 6.47e-03 |
|  | 12 | 106460568 | 106461007 | PDFSTATSENSEMBLUCSC | 440 | 5 | 9276 | gene;island | 0.781594 | 0.925289 | Not queried | 2.66 | 9.52e-02 | 1.53e-01 |
|  | 12 | 57588031 | 57588350 | PDFSTATSENSEMBLUCSC | 320 | 8 | 9187 | gene | 0.970962 | 0.806002 | LRP1; MIR1228 (margin: LRP1; MIR1228) | 2.66 | 1.86e-03 | 1.45e-02 |
|  | 11 | 126173516 | 126173637 | PDFSTATSENSEMBLUCSC | 122 | 6 | 7799 | tss | 0.009987 | 0.017093 | Not queried | 2.66 | 3.10e-01 | 3.66e-01 |
|  | 2 | 164592477 | 164592571 | PDFSTATSENSEMBLUCSC | 95 | 4 | 24828 | island | 0.063226 | 0.008723 | Not queried | 2.66 | 2.86e-02 | 6.12e-02 |
|  | 6 | 33037413 | 33037690 | PDFSTATSENSEMBLUCSC | 278 | 6 | 34181 | gene | 0.440156 | 0.891974 | Not queried | 2.66 | 2.40e-01 | 2.95e-01 |
|  | 8 | 68864769 | 68864964 | PDFSTATSENSEMBLUCSC | 196 | 4 | 39328 | gene;island | 0.701390 | 0.936550 | Not queried | 2.66 | 2.00e-01 | 2.55e-01 |
|  | 1 | 6515580 | 6515748 | PDFSTATSENSEMBLUCSC | 169 | 4 | 173 | gene;island | 0.836531 | 0.972105 | Not queried | 2.66 | 2.00e-01 | 2.55e-01 |
|  | 4 | 6695203 | 6695475 | PDFSTATSENSEMBLUCSC | 273 | 6 | 30283 | tss | 0.155733 | 0.536683 | Not queried | 2.66 | 4.11e-02 | 8.14e-02 |
|  | 17 | 22191704 | 22191979 | PDFSTATSENSEMBLUCSC | 276 | 4 | 18131 | island | 0.370385 | 0.799719 | Not queried | 2.66 | 1.14e-01 | 1.68e-01 |
|  | 4 | 1005876 | 1006240 | PDFSTATSENSEMBLUCSC | 365 | 8 | 30236 | tss | 0.041565 | 0.038465 | Not queried | 2.66 | 2.79e-01 | 3.38e-01 |
|  | 11 | 68437736 | 68438018 | PDFSTATSENSEMBLUCSC | 283 | 4 | 8575 | island | 0.749936 | 0.938712 | Not queried | 2.66 | 4.86e-01 | 5.11e-01 |
|  | 3 | 42947263 | 42947565 | PDFSTATSENSEMBLUCSC | 303 | 5 | 28250 | tss | 0.907377 | 0.987141 | ACKR2; KRBOX1; ZNF662 (margin: ACKR2; KRBOX1; ZNF662) | 2.66 | 7.94e-03 | 3.19e-02 |
|  | 17 | 46824727 | 46825371 | PDFSTATSENSEMBLUCSC | 645 | 5 | 18506 | island | 0.805762 | 0.979614 | Not queried | 2.66 | 4.21e-01 | 4.59e-01 |
|  | 20 | 5451661 | 5451815 | PDFSTATSENSEMBLUCSC | 155 | 6 | 25582 | tss | 0.439669 | 0.881636 | Not queried | 2.66 | 6.49e-02 | 1.11e-01 |
|  | 14 | 92414151 | 92414425 | PDFSTATSENSEMBLUCSC | 275 | 5 | 12562 | tss;island | 0.223156 | 0.494940 | Not queried | 2.66 | 5.56e-02 | 1.00e-01 |
|  | 3 | 50263508 | 50263894 | PDFSTATSENSEMBLUCSC | 387 | 5 | 29140 | island | 0.197770 | 0.025393 | Not queried | 2.66 | 4.21e-01 | 4.59e-01 |
|  | 4 | 76555772 | 76556042 | PDFSTATSENSEMBLUCSC | 271 | 9 | 30439 | tss | 0.115174 | 0.453354 | CDKL2 (margin: CDKL2; G3BP2) | 2.66 | 4.11e-05 | 1.17e-03 |
|  | 12 | 49740639 | 49740996 | PDFSTATSENSEMBLUCSC | 358 | 10 | 9687 | tss | 0.085685 | 0.017948 | DNAJC22 (margin: DNAJC22; C1QL4) | 2.65 | 6.84e-03 | 3.19e-02 |
|  | 12 | 49740639 | 49741245 | PDFSTATSENSEMBLUCSC | 607 | 12 | 10392 | island | 0.085685 | 0.021227 | DNAJC22 (margin: DNAJC22; C1QL4) | 2.65 | 8.29e-03 | 3.33e-02 |
|  | 13 | 112630296 | 112630795 | PDFSTATSENSEMBLUCSC | 500 | 7 | 11905 | island | 0.756268 | 0.967465 | Not queried | 2.65 | 2.62e-02 | 6.12e-02 |
|  | 6 | 32010178 | 32010333 | PDFSTATSENSEMBLUCSC | 156 | 4 | 34011 | gene | 0.876686 | 0.977382 | Not queried | 2.65 | 3.43e-01 | 3.84e-01 |
|  | 20 | 35503832 | 35504553 | PDFSTATSENSEMBLUCSC | 722 | 8 | 25721 | tss | 0.413950 | 0.796278 | Not queried | 2.65 | 6.50e-02 | 1.11e-01 |
|  | 5 | 169532686 | 169533120 | PDFSTATSENSEMBLUCSC | 435 | 8 | 33376 | island | 0.170298 | 0.636744 | FOXI1 (margin: FOXI1) | 2.65 | 4.66e-03 | 2.68e-02 |
|  | 8 | 145560531 | 145560718 | PDFSTATSENSEMBLUCSC | 188 | 4 | 39977 | tss;island | 0.243943 | 0.030327 | Not queried | 2.65 | 1.14e-01 | 1.68e-01 |
|  | 1 | 45792540 | 45792733 | PDFSTATSENSEMBLUCSC | 194 | 7 | 3198 | island | 0.375137 | 0.113030 | HPDL (margin: HPDL; MUTYH) | 2.65 | 5.83e-04 | 6.47e-03 |
|  | 20 | 23969237 | 23969432 | PDFSTATSENSEMBLUCSC | 196 | 4 | 26053 | island | 0.739434 | 0.949984 | Not queried | 2.65 | 5.71e-02 | 1.00e-01 |
|  | 3 | 44666543 | 44667010 | PDFSTATSENSEMBLUCSC | 468 | 6 | 27741 | gene | 0.006616 | 0.026475 | Not queried | 2.65 | 1.80e-01 | 2.45e-01 |
|  | 4 | 105411974 | 105412536 | PDFSTATSENSEMBLUCSC | 563 | 8 | 31218 | island | 0.135523 | 0.027622 | AC093628.1; AC004053.1; CXXC4 (margin: AC093628.1; AC004053.1; CXXC4) | 2.65 | 1.55e-04 | 2.71e-03 |
|  | 4 | 105412467 | 105412536 | PDFSTATSENSEMBLUCSC | 70 | 4 | 30528 | tss | 0.151092 | 0.030342 | Not queried | 2.65 | 2.86e-02 | 6.12e-02 |
|  | 3 | 40428713 | 40429014 | PDFSTATSENSEMBLUCSC | 302 | 4 | 27727 | gene | 0.048461 | 0.211150 | Not queried | 2.65 | 1.14e-01 | 1.68e-01 |
|  | 8 | 1906312 | 1906498 | PDFSTATSENSEMBLUCSC | 187 | 4 | 39186 | gene | 0.921801 | 0.986258 | Not queried | 2.65 | 1.14e-01 | 1.68e-01 |
|  | 6 | 26183508 | 26183899 | PDFSTATSENSEMBLUCSC | 392 | 9 | 34691 | tss | 0.153976 | 0.453806 | Not queried | 2.65 | 7.70e-02 | 1.30e-01 |
|  | 1 | 177150789 | 177151164 | PDFSTATSENSEMBLUCSC | 376 | 5 | 898 | gene;island | 0.329806 | 0.064670 | FAM5B (margin: FAM5B; AL021398.1) | 2.65 | 7.94e-03 | 3.19e-02 |
|  | 2 | 216301341 | 216301766 | PDFSTATSENSEMBLUCSC | 426 | 5 | 23863 | tss;island | 0.022223 | 0.079236 | Not queried | 2.65 | 4.21e-01 | 4.59e-01 |
|  | 3 | 43147571 | 43148002 | PDFSTATSENSEMBLUCSC | 432 | 8 | 28251 | tss | 0.025360 | 0.010563 | Not queried | 2.65 | 1.30e-01 | 1.89e-01 |
|  | 17 | 74928552 | 74928863 | PDFSTATSENSEMBLUCSC | 312 | 6 | 16800 | gene;island | 0.626013 | 0.970291 | Not queried | 2.65 | 1.52e-02 | 5.36e-02 |
|  | 17 | 36105117 | 36105517 | PDFSTATSENSEMBLUCSC | 401 | 6 | 17291 | tss | 0.743959 | 0.972753 | Not queried | 2.65 | 1.52e-02 | 5.36e-02 |
|  | 1 | 150947403 | 150947589 | PDFSTATSENSEMBLUCSC | 187 | 5 | 3731 | island | 0.432390 | 0.031262 | Not queried | 2.65 | 3.10e-01 | 3.66e-01 |
|  | 10 | 134698483 | 134698937 | PDFSTATSENSEMBLUCSC | 455 | 4 | 6316 | island | 0.380649 | 0.812741 | Not queried | 2.65 | 2.00e-01 | 2.55e-01 |
|  | 6 | 39693228 | 39693479 | PDFSTATSENSEMBLUCSC | 252 | 6 | 34957 | tss | 0.127515 | 0.019166 | Not queried | 2.65 | 1.52e-02 | 5.36e-02 |
|  | 15 | 74528522 | 74528606 | PDFSTATSENSEMBLUCSC | 85 | 4 | 13791 | tss | 0.497474 | 0.874010 | Not queried | 2.64 | 2.86e-02 | 6.12e-02 |
|  | 8 | 53477881 | 53478102 | PDFSTATSENSEMBLUCSC | 222 | 5 | 40293 | island | 0.888336 | 0.973680 | Not queried | 2.64 | 5.56e-02 | 1.00e-01 |
|  | 3 | 25469392 | 25469720 | PDFSTATSENSEMBLUCSC | 329 | 8 | 28190 | tss | 0.155647 | 0.547067 | RARB (margin: RARB) | 2.64 | 2.95e-03 | 1.88e-02 |
|  | 20 | 2796729 | 2796872 | PDFSTATSENSEMBLUCSC | 144 | 4 | 25552 | tss | 0.758961 | 0.930432 | Not queried | 2.64 | 3.43e-01 | 3.84e-01 |
|  | 12 | 105478345 | 105478590 | PDFSTATSENSEMBLUCSC | 246 | 7 | 9962 | tss | 0.021696 | 0.026252 | Not queried | 2.64 | 5.35e-01 | 5.62e-01 |
|  | 2 | 216973806 | 216973988 | PDFSTATSENSEMBLUCSC | 183 | 5 | 23866 | tss | 0.004415 | 0.003063 | Not queried | 2.64 | 3.10e-01 | 3.66e-01 |
|  | 16 | 3142911 | 3143250 | PDFSTATSENSEMBLUCSC | 340 | 6 | 15051 | tss | 0.751604 | 0.960120 | Not queried | 2.64 | 2.60e-02 | 6.12e-02 |
|  | 16 | 3142645 | 3143250 | PDFSTATSENSEMBLUCSC | 606 | 8 | 15636 | island | 0.751604 | 0.939732 | Not queried | 2.64 | 8.30e-02 | 1.40e-01 |
|  | 2 | 239755455 | 239755703 | PDFSTATSENSEMBLUCSC | 249 | 4 | 23976 | tss;island | 0.806350 | 0.974900 | Not queried | 2.64 | 5.71e-02 | 1.00e-01 |
|  | 17 | 26574231 | 26574551 | PDFSTATSENSEMBLUCSC | 321 | 5 | 18136 | island | 0.466875 | 0.844119 | Not queried | 2.64 | 1.59e-02 | 5.36e-02 |
|  | 15 | 52860530 | 52861112 | PDFSTATSENSEMBLUCSC | 583 | 7 | 13356 | gene;island | 0.006875 | 0.010426 | Not queried | 2.64 | 2.09e-01 | 2.65e-01 |
|  | 8 | 145162905 | 145163136 | PDFSTATSENSEMBLUCSC | 232 | 4 | 39511 | gene;island | 0.848796 | 0.979814 | Not queried | 2.64 | 5.71e-02 | 1.00e-01 |
|  | 5 | 1270598 | 1271100 | PDFSTATSENSEMBLUCSC | 503 | 5 | 31532 | gene;island | 0.860012 | 0.923038 | Not queried | 2.64 | 1.51e-01 | 2.11e-01 |
|  | 3 | 185911519 | 185911885 | PDFSTATSENSEMBLUCSC | 367 | 4 | 28072 | gene;island | 0.850586 | 0.975968 | Not queried | 2.64 | 2.00e-01 | 2.55e-01 |
|  | 6 | 133561614 | 133562494 | PDFSTATSENSEMBLUCSC | 881 | 38 | 36486 | island | 0.829707 | 0.971963 | EYA4 (margin: EYA4) | 2.64 | 1.52e-08 | 3.90e-06 |
|  | 1 | 1104257 | 1104366 | PDFSTATSENSEMBLUCSC | 110 | 4 | 1141 | tss | 0.884503 | 0.978834 | Not queried | 2.64 | 2.86e-02 | 6.12e-02 |
|  | 7 | 149629 | 150084 | PDFSTATSENSEMBLUCSC | 456 | 5 | 38029 | island | 0.317432 | 0.029611 | Not queried | 2.64 | 4.21e-01 | 4.59e-01 |
|  | 6 | 30173936 | 30174261 | PDFSTATSENSEMBLUCSC | 326 | 7 | 33750 | gene | 0.762220 | 0.953680 | Not queried | 2.64 | 7.28e-02 | 1.23e-01 |
|  | 5 | 16617290 | 16617587 | PDFSTATSENSEMBLUCSC | 298 | 4 | 31996 | tss;island | 0.571807 | 0.103018 | Not queried | 2.64 | 6.86e-01 | 6.99e-01 |
|  | 10 | 81664323 | 81664698 | PDFSTATSENSEMBLUCSC | 376 | 5 | 5884 | island | 0.211412 | 0.534895 | Not queried | 2.64 | 9.52e-02 | 1.53e-01 |
|  | 7 | 155249398 | 155249941 | PDFSTATSENSEMBLUCSC | 544 | 6 | 38010 | tss;island | 0.135396 | 0.072237 | Not queried | 2.64 | 1.80e-01 | 2.45e-01 |
|  | 1 | 232941468 | 232941775 | PDFSTATSENSEMBLUCSC | 308 | 4 | 1081 | gene;island | 0.722951 | 0.954277 | Not queried | 2.64 | 5.71e-02 | 1.00e-01 |
|  | 11 | 111895234 | 111895485 | PDFSTATSENSEMBLUCSC | 252 | 6 | 7696 | tss | 0.672027 | 0.205522 | Not queried | 2.64 | 4.85e-01 | 5.11e-01 |
|  | 11 | 134257429 | 134257744 | PDFSTATSENSEMBLUCSC | 316 | 4 | 7000 | gene;island | 0.858794 | 0.951807 | Not queried | 2.64 | 2.86e-02 | 6.12e-02 |
|  | 1 | 226410871 | 226411270 | PDFSTATSENSEMBLUCSC | 400 | 8 | 2288 | tss;island | 0.730834 | 0.975939 | Not queried | 2.63 | 2.07e-02 | 6.12e-02 |
|  | 19 | 55417361 | 55417500 | PDFSTATSENSEMBLUCSC | 140 | 5 | 21066 | tss | 0.873737 | 0.920772 | Not queried | 2.63 | 9.52e-02 | 1.53e-01 |
|  | 5 | 130330787 | 130331091 | PDFSTATSENSEMBLUCSC | 305 | 4 | 33076 | island | 0.559336 | 0.895767 | Not queried | 2.63 | 3.43e-01 | 3.84e-01 |
|  | 18 | 10471657 | 10471866 | PDFSTATSENSEMBLUCSC | 210 | 4 | 19054 | gene;island | 0.459578 | 0.855875 | Not queried | 2.63 | 2.00e-01 | 2.55e-01 |
|  | 4 | 41875470 | 41875875 | PDFSTATSENSEMBLUCSC | 406 | 4 | 31007 | island | 0.757347 | 0.960821 | Not queried | 2.63 | 5.71e-02 | 1.00e-01 |
|  | 2 | 152146125 | 152146478 | PDFSTATSENSEMBLUCSC | 354 | 4 | 24782 | island | 0.003691 | 0.014450 | Not queried | 2.63 | 3.43e-01 | 3.84e-01 |
|  | 4 | 154680808 | 154681323 | PDFSTATSENSEMBLUCSC | 516 | 6 | 30155 | gene;island | 0.885930 | 0.975538 | RNF175 (margin: RNF175) | 2.63 | 2.16e-03 | 1.45e-02 |
|  | 1 | 110081561 | 110081965 | PDFSTATSENSEMBLUCSC | 405 | 4 | 1806 | tss | 0.562527 | 0.938508 | Not queried | 2.63 | 1.14e-01 | 1.68e-01 |
|  | 1 | 208132388 | 208132787 | PDFSTATSENSEMBLUCSC | 400 | 4 | 4158 | island | 0.864916 | 0.971700 | Not queried | 2.63 | 2.86e-02 | 6.12e-02 |
|  | 19 | 13985408 | 13985587 | PDFSTATSENSEMBLUCSC | 180 | 4 | 20531 | tss | 0.945584 | 0.650802 | Not queried | 2.63 | 1.14e-01 | 1.68e-01 |
|  | 6 | 33264834 | 33265605 | PDFSTATSENSEMBLUCSC | 772 | 14 | 34256 | gene;island | 0.132319 | 0.023403 | Not queried | 2.63 | 3.50e-02 | 7.16e-02 |
|  | 16 | 31146999 | 31147054 | PDFSTATSENSEMBLUCSC | 56 | 4 | 14732 | gene | 0.059456 | 0.009440 | Not queried | 2.63 | 2.86e-02 | 6.12e-02 |
|  | 7 | 100728343 | 100728731 | PDFSTATSENSEMBLUCSC | 389 | 6 | 37797 | tss | 0.008401 | 0.045619 | Not queried | 2.63 | 1.32e-01 | 1.89e-01 |
|  | 17 | 3827633 | 3828052 | PDFSTATSENSEMBLUCSC | 420 | 5 | 16350 | gene;island | 0.925787 | 0.971282 | Not queried | 2.63 | 3.17e-02 | 6.50e-02 |
|  | 15 | 51200672 | 51200847 | PDFSTATSENSEMBLUCSC | 176 | 7 | 13672 | tss;island | 0.006294 | 0.014120 | Not queried | 2.63 | 1.28e-01 | 1.87e-01 |
|  | 12 | 57637268 | 57637690 | PDFSTATSENSEMBLUCSC | 423 | 5 | 9192 | gene | 0.014109 | 0.006680 | Not queried | 2.63 | 4.21e-01 | 4.59e-01 |
|  | 17 | 33814163 | 33814596 | PDFSTATSENSEMBLUCSC | 434 | 5 | 16521 | gene;island | 0.055092 | 0.263949 | Not queried | 2.63 | 3.10e-01 | 3.66e-01 |
|  | 16 | 89033692 | 89034292 | PDFSTATSENSEMBLUCSC | 601 | 7 | 14926 | gene;island | 0.626041 | 0.919590 | Not queried | 2.63 | 2.62e-02 | 6.12e-02 |
|  | 15 | 58357874 | 58357989 | PDFSTATSENSEMBLUCSC | 116 | 7 | 14175 | island | 0.800824 | 0.963230 | Not queried | 2.63 | 2.62e-02 | 6.12e-02 |
|  | 17 | 54230419 | 54230793 | PDFSTATSENSEMBLUCSC | 375 | 5 | 17520 | tss | 0.098361 | 0.420683 | Not queried | 2.63 | 5.56e-02 | 1.00e-01 |
|  | 8 | 142139654 | 142140021 | PDFSTATSENSEMBLUCSC | 368 | 4 | 39445 | gene;island | 0.091160 | 0.408371 | Not queried | 2.62 | 2.86e-02 | 6.12e-02 |
|  | 1 | 111148858 | 111149307 | PDFSTATSENSEMBLUCSC | 450 | 5 | 1817 | tss;island | 0.485011 | 0.921941 | Not queried | 2.62 | 9.52e-02 | 1.53e-01 |
|  | 18 | 74691097 | 74691423 | PDFSTATSENSEMBLUCSC | 327 | 4 | 19131 | gene;island | 0.852763 | 0.985356 | Not queried | 2.62 | 5.71e-02 | 1.00e-01 |
|  | 5 | 177018929 | 177019065 | PDFSTATSENSEMBLUCSC | 137 | 6 | 32534 | tss;island | 0.029714 | 0.006429 | Not queried | 2.62 | 3.94e-01 | 4.36e-01 |
|  | 4 | 8430380 | 8430496 | PDFSTATSENSEMBLUCSC | 117 | 5 | 29953 | gene;island | 0.010574 | 0.023429 | Not queried | 2.62 | 1.51e-01 | 2.11e-01 |
|  | 10 | 102279690 | 102279954 | PDFSTATSENSEMBLUCSC | 265 | 7 | 5294 | tss;island | 0.989507 | 0.955458 | SEC31B; NDUFB8 (margin: HIF1AN; SEC31B; NDUFB8) | 2.62 | 1.11e-02 | 4.14e-02 |
|  | 20 | 13279761 | 13280201 | PDFSTATSENSEMBLUCSC | 441 | 6 | 25359 | gene;island | 0.802654 | 0.945217 | Not queried | 2.62 | 1.32e-01 | 1.89e-01 |
|  | 19 | 33764284 | 33764550 | PDFSTATSENSEMBLUCSC | 267 | 4 | 21873 | island | 0.540879 | 0.900603 | Not queried | 2.62 | 1.14e-01 | 1.68e-01 |
|  | 13 | 112172244 | 112172440 | PDFSTATSENSEMBLUCSC | 197 | 4 | 11897 | island | 0.694966 | 0.919772 | Not queried | 2.62 | 3.43e-01 | 3.84e-01 |
|  | 1 | 21044031 | 21044207 | PDFSTATSENSEMBLUCSC | 177 | 4 | 294 | gene | 0.805450 | 0.416234 | Not queried | 2.62 | 2.86e-02 | 6.12e-02 |
|  | 1 | 21044031 | 21044327 | PDFSTATSENSEMBLUCSC | 297 | 6 | 2801 | island | 0.764047 | 0.213441 | Not queried | 2.62 | 6.49e-02 | 1.11e-01 |
|  | 5 | 2112109 | 2112561 | PDFSTATSENSEMBLUCSC | 453 | 7 | 32652 | island | 0.640088 | 0.934477 | (margin: ) | 2.62 | 4.08e-03 | 2.47e-02 |
|  | 15 | 40762807 | 40763522 | PDFSTATSENSEMBLUCSC | 716 | 10 | 14038 | island | 0.010622 | 0.053347 | CHST14 (margin: BAHD1; CHST14; RP11-64K12.8; RP11-64K12.1) | 2.62 | 3.89e-03 | 2.43e-02 |
|  | 7 | 25018440 | 25018595 | PDFSTATSENSEMBLUCSC | 156 | 4 | 36923 | gene;island | 0.787426 | 0.878909 | Not queried | 2.62 | 4.86e-01 | 5.11e-01 |
|  | 6 | 29618205 | 29618382 | PDFSTATSENSEMBLUCSC | 178 | 4 | 35622 | island | 0.020418 | 0.095583 | Not queried | 2.62 | 2.00e-01 | 2.55e-01 |
|  | 21 | 30671675 | 30671710 | PDFSTATSENSEMBLUCSC | 36 | 5 | 26504 | tss | 0.005272 | 0.012037 | Not queried | 2.62 | 1.51e-01 | 2.11e-01 |
|  | 17 | 171006 | 171434 | PDFSTATSENSEMBLUCSC | 429 | 5 | 16290 | gene;island | 0.373762 | 0.954997 | Not queried | 2.62 | 2.22e-01 | 2.76e-01 |
|  | 17 | 79920395 | 79920755 | PDFSTATSENSEMBLUCSC | 361 | 5 | 18962 | island | 0.703586 | 0.933557 | Not queried | 2.61 | 2.22e-01 | 2.76e-01 |
|  | 12 | 102090980 | 102091387 | PDFSTATSENSEMBLUCSC | 408 | 9 | 9946 | tss;island | 0.041049 | 0.016300 | Not queried | 2.61 | 1.88e-02 | 6.12e-02 |
|  | 7 | 148984536 | 148984719 | PDFSTATSENSEMBLUCSC | 184 | 4 | 37265 | gene;island | 0.981142 | 0.804291 | Not queried | 2.61 | 1.14e-01 | 1.68e-01 |
|  | 10 | 27149660 | 27149860 | PDFSTATSENSEMBLUCSC | 201 | 4 | 4538 | gene | 0.000275 | 0.003214 | Not queried | 2.61 | 5.71e-02 | 1.00e-01 |
|  | 14 | 105830509 | 105830859 | PDFSTATSENSEMBLUCSC | 351 | 5 | 12262 | gene;island | 0.825938 | 0.979490 | Not queried | 2.61 | 3.17e-02 | 6.50e-02 |
|  | 15 | 91839949 | 91840307 | PDFSTATSENSEMBLUCSC | 359 | 4 | 13484 | gene;island | 0.516985 | 0.862989 | Not queried | 2.61 | 1.14e-01 | 1.68e-01 |
|  | 5 | 95297792 | 95298307 | PDFSTATSENSEMBLUCSC | 516 | 7 | 32188 | tss | 0.074980 | 0.925923 | Not queried | 2.61 | 2.59e-01 | 3.16e-01 |
|  | 20 | 35580195 | 35580555 | PDFSTATSENSEMBLUCSC | 361 | 9 | 25722 | tss | 0.009571 | 0.093263 | Not queried | 2.61 | 4.00e-02 | 8.03e-02 |
|  | 1 | 2771846 | 2772363 | PDFSTATSENSEMBLUCSC | 518 | 5 | 2518 | island | 0.588704 | 0.836024 | Not queried | 2.61 | 3.17e-02 | 6.50e-02 |
|  | 5 | 140552347 | 140552600 | PDFSTATSENSEMBLUCSC | 254 | 4 | 31811 | gene | 0.334869 | 0.583075 | Not queried | 2.61 | 4.86e-01 | 5.11e-01 |
|  | 1 | 156405249 | 156405495 | PDFSTATSENSEMBLUCSC | 247 | 4 | 3856 | island | 0.715912 | 0.920874 | Not queried | 2.61 | 3.43e-01 | 3.84e-01 |
|  | 11 | 926062 | 926250 | PDFSTATSENSEMBLUCSC | 189 | 4 | 6397 | gene;island | 0.002262 | 0.009205 | Not queried | 2.61 | 2.00e-01 | 2.55e-01 |
|  | 4 | 46392253 | 46392657 | PDFSTATSENSEMBLUCSC | 405 | 6 | 30373 | tss;island | 0.968699 | 0.708708 | Not queried | 2.61 | 6.49e-02 | 1.11e-01 |
|  | 20 | 43538745 | 43538936 | PDFSTATSENSEMBLUCSC | 192 | 4 | 25426 | gene | 0.004090 | 0.012423 | Not queried | 2.61 | 2.00e-01 | 2.55e-01 |
|  | 1 | 11795746 | 11796079 | PDFSTATSENSEMBLUCSC | 334 | 10 | 1249 | tss | 0.009704 | 0.061019 | Not queried | 2.61 | 2.80e-01 | 3.40e-01 |
|  | 16 | 67979437 | 67979792 | PDFSTATSENSEMBLUCSC | 356 | 4 | 14809 | gene | 0.611029 | 0.950735 | Not queried | 2.61 | 1.14e-01 | 1.68e-01 |
|  | 13 | 48876603 | 48876950 | PDFSTATSENSEMBLUCSC | 348 | 5 | 11434 | tss;island | 0.877652 | 0.973221 | Not queried | 2.61 | 3.10e-01 | 3.66e-01 |
|  | 11 | 2323801 | 2323938 | PDFSTATSENSEMBLUCSC | 138 | 4 | 6455 | gene;tss | 0.781367 | 0.955860 | Not queried | 2.61 | 2.86e-02 | 6.12e-02 |
|  | 6 | 133561614 | 133562492 | PDFSTATSENSEMBLUCSC | 879 | 37 | 35231 | tss | 0.813407 | 0.970430 | EYA4 (margin: EYA4) | 2.61 | 2.76e-08 | 6.41e-06 |
|  | 8 | 142183507 | 142183860 | PDFSTATSENSEMBLUCSC | 354 | 4 | 39447 | gene;island | 0.847999 | 0.505063 | Not queried | 2.61 | 1.14e-01 | 1.68e-01 |
|  | 8 | 50823124 | 50823541 | PDFSTATSENSEMBLUCSC | 418 | 6 | 39694 | tss;island | 0.316957 | 0.253398 | Not queried | 2.60 | 3.94e-01 | 4.36e-01 |
|  | 16 | 56224504 | 56225059 | PDFSTATSENSEMBLUCSC | 556 | 6 | 15242 | tss;island | 0.342652 | 0.025898 | Not queried | 2.60 | 9.31e-02 | 1.53e-01 |
|  | 19 | 10046864 | 10047420 | PDFSTATSENSEMBLUCSC | 557 | 8 | 21511 | island | 0.096399 | 0.021051 | OLFM2 (margin: OLFM2) | 2.60 | 3.11e-04 | 4.77e-03 |
|  | 11 | 113746456 | 113746831 | PDFSTATSENSEMBLUCSC | 376 | 5 | 7706 | tss;island | 0.003742 | 0.013874 | Not queried | 2.60 | 9.52e-02 | 1.53e-01 |
|  | 3 | 138739807 | 138740022 | PDFSTATSENSEMBLUCSC | 216 | 6 | 28628 | tss | 0.701327 | 0.931399 | Not queried | 2.60 | 2.60e-02 | 6.12e-02 |
|  | 7 | 144533232 | 144533471 | PDFSTATSENSEMBLUCSC | 240 | 7 | 37957 | tss | 0.008151 | 0.098818 | Not queried | 2.60 | 1.65e-01 | 2.28e-01 |
|  | 1 | 245843825 | 245844133 | PDFSTATSENSEMBLUCSC | 309 | 4 | 1119 | gene | 0.804008 | 0.972885 | Not queried | 2.60 | 2.86e-02 | 6.12e-02 |
|  | 19 | 11689488 | 11689997 | PDFSTATSENSEMBLUCSC | 510 | 12 | 20477 | tss;island | 0.876010 | 0.981594 | ZNF627; ACP5 (margin: ZNF627; AC020947.2; ACP5) | 2.60 | 1.11e-03 | 1.12e-02 |
|  | 5 | 178450457 | 178450763 | PDFSTATSENSEMBLUCSC | 307 | 6 | 32547 | tss | 0.074159 | 0.305924 | ZNF879 (margin: ZNF879) | 2.60 | 2.16e-03 | 1.45e-02 |
|  | 10 | 61122358 | 61122716 | PDFSTATSENSEMBLUCSC | 359 | 8 | 5093 | tss | 0.071865 | 0.006552 | FAM13C (margin: FAM13C) | 2.60 | 1.04e-02 | 3.96e-02 |
|  | 8 | 1404885 | 1405138 | PDFSTATSENSEMBLUCSC | 254 | 4 | 40025 | island | 0.476749 | 0.863656 | Not queried | 2.60 | 2.00e-01 | 2.55e-01 |
|  | 16 | 50875140 | 50875496 | PDFSTATSENSEMBLUCSC | 357 | 4 | 15915 | island | 0.577326 | 0.938418 | Not queried | 2.60 | 1.14e-01 | 1.68e-01 |
|  | 7 | 145813915 | 145814306 | PDFSTATSENSEMBLUCSC | 392 | 5 | 37257 | gene;island | 0.836235 | 0.525692 | Not queried | 2.60 | 9.52e-02 | 1.53e-01 |
|  | 20 | 37434158 | 37434552 | PDFSTATSENSEMBLUCSC | 395 | 8 | 26161 | island | 0.748051 | 0.947198 | Not queried | 2.60 | 1.48e-02 | 5.36e-02 |
|  | 12 | 52300911 | 52301307 | PDFSTATSENSEMBLUCSC | 397 | 5 | 10432 | island | 0.776614 | 0.939776 | Not queried | 2.60 | 2.22e-01 | 2.76e-01 |
|  | 16 | 56622951 | 56623215 | PDFSTATSENSEMBLUCSC | 265 | 6 | 15246 | tss | 0.908559 | 0.983984 | MT3 (margin: MT3) | 2.60 | 2.16e-03 | 1.45e-02 |
|  | 1 | 45792557 | 45792733 | PDFSTATSENSEMBLUCSC | 177 | 5 | 501 | gene | 0.434480 | 0.116702 | HPDL (margin: HPDL; MUTYH) | 2.60 | 7.94e-03 | 3.19e-02 |
|  | 22 | 17489112 | 17489367 | PDFSTATSENSEMBLUCSC | 256 | 7 | 26938 | tss;island | 0.864131 | 0.971027 | Not queried | 2.60 | 9.73e-02 | 1.55e-01 |
|  | 11 | 70666452 | 70666748 | PDFSTATSENSEMBLUCSC | 297 | 4 | 6796 | gene;island | 0.807880 | 0.958335 | Not queried | 2.60 | 2.86e-02 | 6.12e-02 |
|  | 7 | 1451136 | 1451332 | PDFSTATSENSEMBLUCSC | 197 | 4 | 38103 | island | 0.763421 | 0.987371 | Not queried | 2.59 | 4.86e-01 | 5.11e-01 |
|  | 1 | 242688081 | 242688320 | PDFSTATSENSEMBLUCSC | 240 | 4 | 2351 | tss;island | 0.358789 | 0.754004 | Not queried | 2.59 | 1.14e-01 | 1.68e-01 |
|  | 11 | 17410182 | 17410554 | PDFSTATSENSEMBLUCSC | 373 | 4 | 6523 | gene;island | 0.100229 | 0.049643 | Not queried | 2.59 | 6.86e-01 | 6.99e-01 |
|  | 14 | 96505296 | 96505636 | PDFSTATSENSEMBLUCSC | 341 | 9 | 12591 | tss | 0.124201 | 0.022925 | C14orf132 (margin: C14orf132) | 2.59 | 1.06e-02 | 4.01e-02 |
|  | 2 | 80531483 | 80531898 | PDFSTATSENSEMBLUCSC | 416 | 10 | 22756 | gene;island | 0.828147 | 0.453306 | CTNNA2; LRRTM1 (margin: CTNNA2; LRRTM1) | 2.59 | 2.06e-04 | 3.42e-03 |
|  | 1 | 247681102 | 247681931 | PDFSTATSENSEMBLUCSC | 830 | 8 | 4418 | island | 0.621045 | 0.873868 | Not queried | 2.59 | 1.05e-01 | 1.66e-01 |
|  | 11 | 1154976 | 1155278 | PDFSTATSENSEMBLUCSC | 303 | 4 | 7904 | island | 0.706579 | 0.955441 | Not queried | 2.59 | 4.86e-01 | 5.11e-01 |
|  | 5 | 126852955 | 126853253 | PDFSTATSENSEMBLUCSC | 299 | 10 | 32257 | tss | 0.065713 | 0.011242 | Not queried | 2.59 | 3.55e-02 | 7.24e-02 |
|  | 7 | 26904342 | 26904713 | PDFSTATSENSEMBLUCSC | 372 | 9 | 37485 | tss | 0.004477 | 0.001627 | Not queried | 2.59 | 5.03e-02 | 9.87e-02 |
|  | 19 | 1355910 | 1356315 | PDFSTATSENSEMBLUCSC | 406 | 9 | 20301 | tss | 0.944647 | 0.713477 | Not queried | 2.59 | 6.25e-02 | 1.09e-01 |
|  | 8 | 55370192 | 55370434 | PDFSTATSENSEMBLUCSC | 243 | 8 | 39706 | tss | 0.776897 | 0.962341 | SOX17 (margin: SOX17) | 2.59 | 1.04e-02 | 3.96e-02 |
|  | 6 | 168630047 | 168630308 | PDFSTATSENSEMBLUCSC | 262 | 4 | 36662 | island | 0.605911 | 0.956141 | Not queried | 2.59 | 2.00e-01 | 2.55e-01 |
|  | 10 | 134729942 | 134730258 | PDFSTATSENSEMBLUCSC | 317 | 5 | 6317 | island | 0.319742 | 0.922501 | Not queried | 2.59 | 2.22e-01 | 2.76e-01 |
|  | 10 | 52499393 | 52499571 | PDFSTATSENSEMBLUCSC | 179 | 4 | 5082 | tss | 0.059288 | 0.413797 | Not queried | 2.59 | 2.00e-01 | 2.55e-01 |
|  | 14 | 56232985 | 56233178 | PDFSTATSENSEMBLUCSC | 194 | 4 | 12090 | gene | 0.671875 | 0.963171 | Not queried | 2.59 | 5.71e-02 | 1.00e-01 |
|  | 17 | 15847977 | 15848221 | PDFSTATSENSEMBLUCSC | 245 | 7 | 17152 | tss | 0.541954 | 0.017800 | Not queried | 2.59 | 3.79e-02 | 7.66e-02 |
|  | 22 | 43045525 | 43045736 | PDFSTATSENSEMBLUCSC | 212 | 4 | 27196 | tss;island | 0.010560 | 0.222020 | Not queried | 2.59 | 4.86e-01 | 5.11e-01 |
|  | 16 | 11761803 | 11762212 | PDFSTATSENSEMBLUCSC | 410 | 7 | 15094 | tss;island | 0.526579 | 0.156743 | Not queried | 2.59 | 5.35e-01 | 5.62e-01 |
|  | 2 | 201980504 | 201980910 | PDFSTATSENSEMBLUCSC | 407 | 8 | 25007 | island | 0.042311 | 0.272257 | Not queried | 2.59 | 1.95e-01 | 2.55e-01 |
|  | 19 | 52872916 | 52873624 | PDFSTATSENSEMBLUCSC | 709 | 10 | 22356 | island | 0.572366 | 0.831416 | Not queried | 2.59 | 1.23e-01 | 1.81e-01 |
|  | 1 | 27709575 | 27709791 | PDFSTATSENSEMBLUCSC | 217 | 6 | 354 | gene | 0.328824 | 0.101602 | Not queried | 2.59 | 3.10e-01 | 3.66e-01 |
|  | 17 | 15165803 | 15166153 | PDFSTATSENSEMBLUCSC | 351 | 8 | 16454 | gene;island | 0.094144 | 0.028922 | Not queried | 2.59 | 2.07e-02 | 6.12e-02 |
|  | 1 | 11561497 | 11562134 | PDFSTATSENSEMBLUCSC | 638 | 6 | 221 | gene;island | 0.899349 | 0.981292 | Not queried | 2.59 | 6.49e-02 | 1.11e-01 |
|  | 15 | 50716705 | 50716929 | PDFSTATSENSEMBLUCSC | 225 | 4 | 13347 | gene;island | 0.007320 | 0.007939 | Not queried | 2.59 | 8.86e-01 | 8.89e-01 |
|  | 13 | 36705475 | 36705622 | PDFSTATSENSEMBLUCSC | 148 | 4 | 11391 | tss | 0.121104 | 0.041590 | Not queried | 2.59 | 2.86e-02 | 6.12e-02 |
|  | 19 | 56158714 | 56158885 | PDFSTATSENSEMBLUCSC | 172 | 4 | 21087 | tss;island | 0.726931 | 0.978767 | Not queried | 2.59 | 1.14e-01 | 1.68e-01 |
|  | 5 | 139839 | 140289 | PDFSTATSENSEMBLUCSC | 451 | 7 | 31953 | tss | 0.391257 | 0.862808 | Not queried | 2.59 | 5.30e-02 | 1.00e-01 |
|  | 2 | 163695776 | 163696187 | PDFSTATSENSEMBLUCSC | 412 | 6 | 23690 | tss | 0.798993 | 0.943515 | Not queried | 2.58 | 2.60e-02 | 6.12e-02 |
|  | 11 | 126455596 | 126456119 | PDFSTATSENSEMBLUCSC | 524 | 6 | 6963 | gene | 0.458123 | 0.848083 | Not queried | 2.58 | 2.40e-01 | 2.95e-01 |
|  | 2 | 240168836 | 240169280 | PDFSTATSENSEMBLUCSC | 445 | 6 | 23111 | gene | 0.875321 | 0.981412 | Not queried | 2.58 | 9.31e-02 | 1.53e-01 |
|  | 12 | 54426498 | 54426772 | PDFSTATSENSEMBLUCSC | 275 | 8 | 9755 | tss | 0.004090 | 0.087706 | Not queried | 2.58 | 4.99e-02 | 9.81e-02 |
|  | 5 | 131281008 | 131281574 | PDFSTATSENSEMBLUCSC | 567 | 6 | 33082 | island | 0.840572 | 0.982099 | Not queried | 2.58 | 6.49e-02 | 1.11e-01 |
|  | 1 | 26232706 | 26233035 | PDFSTATSENSEMBLUCSC | 330 | 7 | 335 | gene;tss;island | 0.000728 | 0.004859 | Not queried | 2.58 | 9.73e-02 | 1.55e-01 |
|  | 2 | 201980504 | 201980867 | PDFSTATSENSEMBLUCSC | 364 | 7 | 23814 | tss | 0.075914 | 0.515227 | Not queried | 2.58 | 2.09e-01 | 2.65e-01 |
|  | 2 | 70313163 | 70313947 | PDFSTATSENSEMBLUCSC | 785 | 7 | 23412 | tss;island | 0.262773 | 0.016659 | Not queried | 2.58 | 5.35e-01 | 5.62e-01 |
|  | 10 | 85997177 | 85997517 | PDFSTATSENSEMBLUCSC | 341 | 4 | 4657 | gene;island | 0.563974 | 0.940226 | Not queried | 2.58 | 3.43e-01 | 3.84e-01 |
|  | 8 | 90996948 | 90997072 | PDFSTATSENSEMBLUCSC | 125 | 4 | 39805 | tss;island | 0.000641 | 0.003118 | Not queried | 2.58 | 3.43e-01 | 3.84e-01 |
|  | 5 | 1093580 | 1093797 | PDFSTATSENSEMBLUCSC | 218 | 4 | 31523 | gene;island | 0.718334 | 0.953818 | Not queried | 2.58 | 1.14e-01 | 1.68e-01 |
|  | 13 | 114886700 | 114886883 | PDFSTATSENSEMBLUCSC | 184 | 4 | 11317 | gene | 0.713507 | 0.933586 | Not queried | 2.58 | 2.86e-02 | 6.12e-02 |
|  | 5 | 83678796 | 83679364 | PDFSTATSENSEMBLUCSC | 569 | 6 | 31671 | gene;island | 0.473075 | 0.806874 | Not queried | 2.58 | 1.32e-01 | 1.89e-01 |
|  | 6 | 78176473 | 78176847 | PDFSTATSENSEMBLUCSC | 375 | 6 | 36270 | island | 0.489068 | 0.876843 | Not queried | 2.58 | 1.32e-01 | 1.89e-01 |
|  | 1 | 38231746 | 38231980 | PDFSTATSENSEMBLUCSC | 235 | 4 | 1508 | tss;island | 0.192626 | 0.054444 | Not queried | 2.58 | 3.43e-01 | 3.84e-01 |
|  | 15 | 69744390 | 69745085 | PDFSTATSENSEMBLUCSC | 696 | 12 | 13764 | tss | 0.372612 | 0.719692 | Not queried | 2.58 | 1.01e-01 | 1.60e-01 |
|  | 8 | 53478023 | 53478102 | PDFSTATSENSEMBLUCSC | 80 | 4 | 39698 | tss | 0.860593 | 0.968229 | Not queried | 2.58 | 1.14e-01 | 1.68e-01 |
|  | 22 | 44287876 | 44288155 | PDFSTATSENSEMBLUCSC | 280 | 6 | 27206 | tss | 0.721752 | 0.900001 | Not queried | 2.58 | 1.80e-01 | 2.45e-01 |
|  | 19 | 44529541 | 44529822 | PDFSTATSENSEMBLUCSC | 282 | 4 | 20080 | gene | 0.001608 | 0.019215 | Not queried | 2.58 | 1.14e-01 | 1.68e-01 |
|  | 11 | 116706372 | 116706626 | PDFSTATSENSEMBLUCSC | 255 | 4 | 8838 | island | 0.961763 | 0.733873 | Not queried | 2.58 | 2.00e-01 | 2.55e-01 |
|  | 1 | 230492737 | 230493133 | PDFSTATSENSEMBLUCSC | 397 | 5 | 1072 | gene;island | 0.896691 | 0.981021 | Not queried | 2.58 | 3.10e-01 | 3.66e-01 |
|  | 1 | 115300693 | 115300877 | PDFSTATSENSEMBLUCSC | 185 | 5 | 1853 | tss | 0.001732 | 0.009736 | Not queried | 2.58 | 4.21e-01 | 4.59e-01 |
|  | 1 | 179545091 | 179545458 | PDFSTATSENSEMBLUCSC | 368 | 8 | 2118 | tss | 0.474838 | 0.872089 | Not queried | 2.58 | 8.30e-02 | 1.40e-01 |
|  | 11 | 32454718 | 32455735 | PDFSTATSENSEMBLUCSC | 1018 | 12 | 6567 | gene;island | 0.566199 | 0.958000 | WT1-AS; WT1 (margin: WT1-AS; WT1) | 2.57 | 2.32e-03 | 1.52e-02 |
|  | 7 | 31726494 | 31726704 | PDFSTATSENSEMBLUCSC | 211 | 5 | 37523 | tss | 0.495387 | 0.853892 | Not queried | 2.57 | 2.22e-01 | 2.76e-01 |
|  | 10 | 134955530 | 134955748 | PDFSTATSENSEMBLUCSC | 219 | 4 | 6336 | island | 0.310274 | 0.869506 | Not queried | 2.57 | 3.43e-01 | 3.84e-01 |
|  | 7 | 19185139 | 19185407 | PDFSTATSENSEMBLUCSC | 269 | 5 | 37461 | tss | 0.150202 | 0.035204 | Not queried | 2.57 | 3.17e-02 | 6.50e-02 |
|  | 10 | 133000130 | 133000471 | PDFSTATSENSEMBLUCSC | 342 | 6 | 4843 | gene | 0.587696 | 0.926286 | Not queried | 2.57 | 6.49e-02 | 1.11e-01 |
|  | 14 | 60097124 | 60097536 | PDFSTATSENSEMBLUCSC | 413 | 5 | 12099 | gene;island | 0.909581 | 0.549894 | Not queried | 2.57 | 3.10e-01 | 3.66e-01 |
|  | 11 | 107461585 | 107461899 | PDFSTATSENSEMBLUCSC | 315 | 7 | 7663 | tss;island | 0.060436 | 0.009817 | Not queried | 2.57 | 5.30e-02 | 1.00e-01 |
|  | 2 | 223161771 | 223162128 | PDFSTATSENSEMBLUCSC | 358 | 4 | 23046 | gene;tss;island | 0.888308 | 0.985099 | Not queried | 2.57 | 2.86e-02 | 6.12e-02 |
|  | 1 | 200011684 | 200011988 | PDFSTATSENSEMBLUCSC | 305 | 6 | 934 | gene;island | 0.913987 | 0.977029 | NR5A2 (margin: NR5A2; RNU6-570P) | 2.57 | 4.33e-03 | 2.52e-02 |
|  | 9 | 117267242 | 117267672 | PDFSTATSENSEMBLUCSC | 431 | 5 | 40780 | gene;island | 0.045653 | 0.003913 | Not queried | 2.57 | 3.10e-01 | 3.66e-01 |
|  | 7 | 157561010 | 157561161 | PDFSTATSENSEMBLUCSC | 152 | 4 | 37342 | gene;island | 0.552898 | 0.870981 | Not queried | 2.57 | 6.86e-01 | 6.99e-01 |
|  | 3 | 141496874 | 141496998 | PDFSTATSENSEMBLUCSC | 125 | 5 | 28639 | tss | 0.813737 | 0.948347 | Not queried | 2.57 | 5.56e-02 | 1.00e-01 |
|  | 17 | 68128754 | 68128923 | PDFSTATSENSEMBLUCSC | 170 | 4 | 16743 | gene | 0.506567 | 0.077505 | Not queried | 2.57 | 2.00e-01 | 2.55e-01 |
|  | 20 | 10199210 | 10199473 | PDFSTATSENSEMBLUCSC | 264 | 7 | 25595 | tss | 0.040608 | 0.007249 | SNAP25; SNAP25-AS1 (margin: SNAP25; SNAP25-AS1) | 2.57 | 1.11e-02 | 4.14e-02 |
|  | 19 | 40732518 | 40732902 | PDFSTATSENSEMBLUCSC | 385 | 9 | 22017 | island | 0.410154 | 0.098825 | CNTD2 (margin: MAP3K10; TTC9B; CNTD2; AKT2) | 2.57 | 5.64e-03 | 3.18e-02 |
|  | 17 | 79869748 | 79870056 | PDFSTATSENSEMBLUCSC | 309 | 6 | 17756 | tss;island | 0.769827 | 0.965807 | Not queried | 2.57 | 2.40e-01 | 2.95e-01 |
|  | 8 | 144462007 | 144462235 | PDFSTATSENSEMBLUCSC | 229 | 4 | 39475 | gene;island | 0.368151 | 0.890055 | Not queried | 2.57 | 1.14e-01 | 1.68e-01 |
|  | 20 | 62367698 | 62367998 | PDFSTATSENSEMBLUCSC | 301 | 7 | 26370 | island | 0.615767 | 0.957137 | Not queried | 2.57 | 5.30e-02 | 1.00e-01 |
|  | 1 | 112532802 | 112533260 | PDFSTATSENSEMBLUCSC | 459 | 7 | 1832 | tss;island | 0.286717 | 0.070050 | Not queried | 2.57 | 9.73e-02 | 1.55e-01 |
|  | 1 | 114471711 | 114472148 | PDFSTATSENSEMBLUCSC | 438 | 11 | 3619 | island | 0.005606 | 0.029867 | HIPK1; RP5-1073O3.7 (margin: HIPK1; RP5-1073O3.7) | 2.57 | 1.04e-02 | 3.96e-02 |
|  | 14 | 102829788 | 102829918 | PDFSTATSENSEMBLUCSC | 131 | 4 | 12232 | gene;tss;island | 0.004895 | 0.029998 | Not queried | 2.57 | 3.43e-01 | 3.84e-01 |
|  | 1 | 87617710 | 87617972 | PDFSTATSENSEMBLUCSC | 263 | 4 | 614 | gene;island | 0.880442 | 0.975195 | Not queried | 2.57 | 2.86e-02 | 6.12e-02 |
|  | 13 | 95364510 | 95364675 | PDFSTATSENSEMBLUCSC | 166 | 6 | 11500 | tss | 0.622218 | 0.937076 | Not queried | 2.56 | 1.52e-02 | 5.36e-02 |
|  | 8 | 142139002 | 142139341 | PDFSTATSENSEMBLUCSC | 340 | 4 | 39444 | gene;island | 0.037989 | 0.218024 | Not queried | 2.56 | 2.00e-01 | 2.55e-01 |
|  | 2 | 24232603 | 24232974 | PDFSTATSENSEMBLUCSC | 372 | 11 | 24125 | island | 0.918705 | 0.984519 | MFSD2B (margin: UBXN2A; MFSD2B) | 2.56 | 5.53e-04 | 6.47e-03 |
|  | 7 | 155623690 | 155624002 | PDFSTATSENSEMBLUCSC | 313 | 4 | 39078 | island | 0.644038 | 0.845619 | Not queried | 2.56 | 2.86e-02 | 6.12e-02 |
|  | 22 | 32599511 | 32599648 | PDFSTATSENSEMBLUCSC | 138 | 5 | 26846 | gene;tss | 0.937073 | 0.736597 | Not queried | 2.56 | 1.51e-01 | 2.11e-01 |
|  | 7 | 100318080 | 100318321 | PDFSTATSENSEMBLUCSC | 242 | 8 | 37791 | tss;island | 0.839135 | 0.982373 | Not queried | 2.56 | 4.99e-02 | 9.81e-02 |
|  | 2 | 40739661 | 40740063 | PDFSTATSENSEMBLUCSC | 403 | 4 | 23318 | tss | 0.346489 | 0.861040 | Not queried | 2.56 | 3.43e-01 | 3.84e-01 |
|  | 2 | 176948544 | 176949017 | PDFSTATSENSEMBLUCSC | 474 | 9 | 24889 | island | 0.936457 | 0.595048 | Not queried | 2.56 | 2.58e-01 | 3.16e-01 |
|  | 12 | 111664582 | 111664785 | PDFSTATSENSEMBLUCSC | 204 | 6 | 9306 | gene | 0.599579 | 0.914546 | Not queried | 2.56 | 1.80e-01 | 2.45e-01 |
|  | 11 | 111895234 | 111895577 | PDFSTATSENSEMBLUCSC | 344 | 7 | 8809 | island | 0.556402 | 0.176075 | Not queried | 2.56 | 5.35e-01 | 5.62e-01 |
|  | 20 | 2633258 | 2633362 | PDFSTATSENSEMBLUCSC | 105 | 4 | 25334 | gene | 0.020471 | 0.034276 | Not queried | 2.56 | 4.86e-01 | 5.11e-01 |
|  | 7 | 93519220 | 93519621 | PDFSTATSENSEMBLUCSC | 402 | 4 | 37090 | gene;island | 0.965572 | 0.731861 | Not queried | 2.56 | 3.43e-01 | 3.84e-01 |
|  | 11 | 2920052 | 2920819 | PDFSTATSENSEMBLUCSC | 768 | 16 | 6469 | gene;tss;island | 0.394485 | 0.799497 | SLC22A18; SLC22A18AS (margin: SLC22A18; SLC22A18AS) | 2.56 | 3.92e-03 | 2.46e-02 |
|  | 8 | 38239693 | 38239917 | PDFSTATSENSEMBLUCSC | 225 | 4 | 40232 | island | 0.013372 | 0.214497 | Not queried | 2.56 | 3.43e-01 | 3.84e-01 |
|  | 20 | 31261784 | 31261994 | PDFSTATSENSEMBLUCSC | 211 | 4 | 25675 | tss | 0.190382 | 0.600445 | Not queried | 2.56 | 2.86e-02 | 6.12e-02 |
|  | 6 | 69345836 | 69346248 | PDFSTATSENSEMBLUCSC | 413 | 5 | 34379 | gene;island | 0.480994 | 0.834948 | BAI3; RP3-525N10.2 (margin: BAI3; RP3-525N10.2) | 2.56 | 7.94e-03 | 3.19e-02 |
|  | 12 | 113013048 | 113013494 | PDFSTATSENSEMBLUCSC | 447 | 5 | 10822 | island | 0.728935 | 0.948909 | Not queried | 2.56 | 5.56e-02 | 1.00e-01 |
|  | 6 | 32370520 | 32370879 | PDFSTATSENSEMBLUCSC | 360 | 7 | 34108 | gene | 0.168923 | 0.838551 | Not queried | 2.56 | 1.28e-01 | 1.87e-01 |
|  | 5 | 95768997 | 95769079 | PDFSTATSENSEMBLUCSC | 83 | 6 | 32189 | tss | 0.966235 | 0.835113 | Not queried | 2.56 | 9.31e-02 | 1.53e-01 |
|  | 1 | 235811811 | 235812280 | PDFSTATSENSEMBLUCSC | 470 | 6 | 1092 | gene;island | 0.831307 | 0.960236 | Not queried | 2.56 | 1.80e-01 | 2.45e-01 |
|  | 1 | 180201002 | 180201319 | PDFSTATSENSEMBLUCSC | 318 | 4 | 905 | gene;island | 0.708938 | 0.428945 | Not queried | 2.56 | 6.86e-01 | 6.99e-01 |
|  | 6 | 32076079 | 32076592 | PDFSTATSENSEMBLUCSC | 514 | 9 | 34049 | gene | 0.042925 | 0.321134 | Not queried | 2.56 | 3.15e-02 | 6.50e-02 |
|  | 2 | 24232603 | 24232891 | PDFSTATSENSEMBLUCSC | 289 | 10 | 23220 | tss | 0.923183 | 0.985503 | MFSD2B (margin: UBXN2A; MFSD2B) | 2.56 | 1.50e-03 | 1.41e-02 |
|  | 5 | 174905244 | 174905420 | PDFSTATSENSEMBLUCSC | 177 | 7 | 32506 | tss | 0.038702 | 0.021468 | Not queried | 2.55 | 4.56e-01 | 4.96e-01 |
|  | 7 | 21583226 | 21583623 | PDFSTATSENSEMBLUCSC | 398 | 4 | 36911 | gene;island | 0.656547 | 0.145071 | Not queried | 2.55 | 1.14e-01 | 1.68e-01 |
|  | 2 | 96012586 | 96012803 | PDFSTATSENSEMBLUCSC | 218 | 5 | 22776 | gene;island | 0.476285 | 0.134074 | Not queried | 2.55 | 9.52e-02 | 1.53e-01 |
|  | 10 | 93334974 | 93335196 | PDFSTATSENSEMBLUCSC | 223 | 4 | 4672 | gene | 0.388946 | 0.843978 | Not queried | 2.55 | 2.86e-02 | 6.12e-02 |
|  | 19 | 35940425 | 35940593 | PDFSTATSENSEMBLUCSC | 169 | 4 | 20703 | tss | 0.024897 | 0.004709 | Not queried | 2.55 | 2.00e-01 | 2.55e-01 |
|  | 1 | 202311052 | 202311278 | PDFSTATSENSEMBLUCSC | 227 | 7 | 4087 | island | 0.001279 | 0.004755 | Not queried | 2.55 | 4.56e-01 | 4.96e-01 |
|  | 7 | 63505584 | 63505811 | PDFSTATSENSEMBLUCSC | 228 | 6 | 37613 | tss | 0.066351 | 0.322079 | Not queried | 2.55 | 1.32e-01 | 1.89e-01 |
|  | 14 | 65016517 | 65016602 | PDFSTATSENSEMBLUCSC | 86 | 5 | 12460 | tss;island | 0.927390 | 0.638439 | PPP1R36 (margin: HSPA2; PPP1R36; RP11-973N13.4; RP11-973N13.3) | 2.55 | 7.94e-03 | 3.19e-02 |
|  | 5 | 143584811 | 143585107 | PDFSTATSENSEMBLUCSC | 297 | 4 | 31848 | gene;island | 0.398142 | 0.085856 | Not queried | 2.55 | 5.71e-02 | 1.00e-01 |
|  | 14 | 29247148 | 29247705 | PDFSTATSENSEMBLUCSC | 558 | 6 | 12054 | gene;island | 0.639601 | 0.105351 | Not queried | 2.55 | 1.80e-01 | 2.45e-01 |
|  | 1 | 209979487 | 209979779 | PDFSTATSENSEMBLUCSC | 293 | 8 | 2233 | tss | 0.074692 | 0.010071 | IRF6 (margin: IRF6) | 2.55 | 6.99e-03 | 3.19e-02 |
|  | 12 | 7341580 | 7341666 | PDFSTATSENSEMBLUCSC | 87 | 4 | 9547 | tss;island | 0.013819 | 0.025191 | Not queried | 2.55 | 4.86e-01 | 5.11e-01 |
|  | 12 | 132923339 | 132923603 | PDFSTATSENSEMBLUCSC | 265 | 4 | 11045 | island | 0.112114 | 0.491580 | Not queried | 2.55 | 2.00e-01 | 2.55e-01 |
|  | 14 | 102780478 | 102781012 | PDFSTATSENSEMBLUCSC | 535 | 5 | 13175 | island | 0.226669 | 0.040208 | Not queried | 2.55 | 5.48e-01 | 5.70e-01 |
|  | 1 | 1104257 | 1104526 | PDFSTATSENSEMBLUCSC | 270 | 7 | 2410 | island | 0.849008 | 0.968696 | Not queried | 2.55 | 1.75e-02 | 5.79e-02 |
|  | 12 | 14926572 | 14927245 | PDFSTATSENSEMBLUCSC | 674 | 9 | 9586 | tss | 0.146521 | 0.015512 | Not queried | 2.55 | 7.70e-02 | 1.30e-01 |
|  | 11 | 20617937 | 20618399 | PDFSTATSENSEMBLUCSC | 463 | 8 | 8128 | island | 0.831424 | 0.973566 | Not queried | 2.55 | 1.61e-01 | 2.24e-01 |
|  | 7 | 127881269 | 127881440 | PDFSTATSENSEMBLUCSC | 172 | 5 | 38892 | island | 0.900391 | 0.985438 | Not queried | 2.55 | 9.52e-02 | 1.53e-01 |
|  | 5 | 158533621 | 158533942 | PDFSTATSENSEMBLUCSC | 322 | 5 | 33352 | island | 0.795748 | 0.028619 | Not queried | 2.55 | 5.56e-02 | 1.00e-01 |
|  | 6 | 30655870 | 30656094 | PDFSTATSENSEMBLUCSC | 225 | 5 | 33807 | gene | 0.389841 | 0.675428 | Not queried | 2.55 | 3.10e-01 | 3.66e-01 |
|  | 15 | 23932370 | 23932412 | PDFSTATSENSEMBLUCSC | 43 | 4 | 13254 | gene;island | 0.818403 | 0.965199 | Not queried | 2.55 | 2.86e-02 | 6.12e-02 |
|  | 13 | 113622539 | 113622750 | PDFSTATSENSEMBLUCSC | 212 | 7 | 11545 | tss;island | 0.161351 | 0.011291 | Not queried | 2.55 | 7.10e-01 | 7.20e-01 |
|  | 8 | 41518004 | 41518139 | PDFSTATSENSEMBLUCSC | 136 | 4 | 39289 | gene | 0.633177 | 0.974157 | Not queried | 2.55 | 3.43e-01 | 3.84e-01 |
|  | 22 | 29426511 | 29426832 | PDFSTATSENSEMBLUCSC | 322 | 4 | 26829 | gene;island | 0.391319 | 0.123663 | Not queried | 2.55 | 1.14e-01 | 1.68e-01 |
|  | 12 | 8332640 | 8332802 | PDFSTATSENSEMBLUCSC | 163 | 4 | 9553 | tss | 0.094785 | 0.014894 | Not queried | 2.55 | 2.00e-01 | 2.55e-01 |
|  | 14 | 85996101 | 85996499 | PDFSTATSENSEMBLUCSC | 399 | 10 | 13053 | island | 0.217010 | 0.580131 | RP11-497E19.2; FLRT2; RP11-497E19.1 (margin: RP11-497E19.2; FLRT2; RP11-497E19.1) | 2.55 | 2.09e-03 | 1.45e-02 |
|  | 2 | 71787431 | 71787827 | PDFSTATSENSEMBLUCSC | 397 | 6 | 22732 | gene;island | 0.831600 | 0.506440 | Not queried | 2.55 | 3.94e-01 | 4.36e-01 |
|  | 20 | 49347891 | 49347936 | PDFSTATSENSEMBLUCSC | 46 | 5 | 25820 | tss;island | 0.000100 | 0.002917 | Not queried | 2.54 | 4.21e-01 | 4.59e-01 |
|  | 12 | 58012960 | 58013687 | PDFSTATSENSEMBLUCSC | 728 | 20 | 9822 | tss;island | 0.714889 | 0.966286 | ARHGEF25; SLC26A10; AC025165.8 (margin: DTX3; ARHGEF25; SLC26A10; AC025165.8; B4GALNT1) | 2.54 | 3.45e-06 | 2.32e-04 |
|  | 16 | 27413459 | 27413904 | PDFSTATSENSEMBLUCSC | 446 | 5 | 15151 | tss | 0.264284 | 0.792414 | Not queried | 2.54 | 9.52e-02 | 1.53e-01 |
|  | 14 | 57263993 | 57264287 | PDFSTATSENSEMBLUCSC | 295 | 4 | 12875 | island | 0.789957 | 0.939728 | Not queried | 2.54 | 1.14e-01 | 1.68e-01 |
|  | 14 | 32670315 | 32670656 | PDFSTATSENSEMBLUCSC | 342 | 4 | 12770 | island | 0.004897 | 0.001142 | Not queried | 2.54 | 2.86e-02 | 6.12e-02 |
|  | 2 | 107503547 | 107503834 | PDFSTATSENSEMBLUCSC | 288 | 6 | 23560 | tss;island | 0.878822 | 0.543868 | Not queried | 2.54 | 4.11e-02 | 8.14e-02 |
|  | 3 | 169490563 | 169490789 | PDFSTATSENSEMBLUCSC | 227 | 5 | 28708 | tss | 0.000585 | 0.005416 | Not queried | 2.54 | 1.51e-01 | 2.11e-01 |
|  | 1 | 156646293 | 156646746 | PDFSTATSENSEMBLUCSC | 454 | 5 | 827 | gene;island | 0.931531 | 0.984801 | NES (margin: NES; RP11-66D17.3) | 2.54 | 7.94e-03 | 3.19e-02 |
|  | 1 | 28906210 | 28906538 | PDFSTATSENSEMBLUCSC | 329 | 5 | 368 | gene;tss;island | 0.146838 | 0.562055 | Not queried | 2.54 | 5.56e-02 | 1.00e-01 |
|  | 10 | 62704070 | 62704447 | PDFSTATSENSEMBLUCSC | 378 | 9 | 4599 | gene;tss;island | 0.441602 | 0.048025 | Not queried | 2.54 | 2.44e-02 | 6.12e-02 |
|  | 15 | 52043486 | 52043572 | PDFSTATSENSEMBLUCSC | 87 | 5 | 13353 | gene | 0.217279 | 0.021117 | Not queried | 2.54 | 3.17e-02 | 6.50e-02 |
|  | 6 | 106959535 | 106959651 | PDFSTATSENSEMBLUCSC | 117 | 6 | 35151 | tss;island | 0.015386 | 0.077110 | AIM1 (margin: AIM1) | 2.54 | 4.33e-03 | 2.52e-02 |
|  | 13 | 114321214 | 114321595 | PDFSTATSENSEMBLUCSC | 382 | 6 | 11555 | tss | 0.552101 | 0.885255 | Not queried | 2.54 | 9.31e-02 | 1.53e-01 |
|  | 1 | 110230252 | 110230633 | PDFSTATSENSEMBLUCSC | 382 | 5 | 3570 | island | 0.858014 | 0.957222 | Not queried | 2.54 | 1.59e-02 | 5.36e-02 |
|  | 7 | 1708566 | 1708892 | PDFSTATSENSEMBLUCSC | 327 | 4 | 38122 | island | 0.272187 | 0.706359 | Not queried | 2.54 | 1.14e-01 | 1.68e-01 |
|  | 2 | 98852704 | 98852938 | PDFSTATSENSEMBLUCSC | 235 | 5 | 22799 | gene | 0.873587 | 0.979889 | Not queried | 2.54 | 2.22e-01 | 2.76e-01 |
|  | 12 | 69202458 | 69202718 | PDFSTATSENSEMBLUCSC | 261 | 6 | 9858 | tss | 0.001162 | 0.003041 | Not queried | 2.54 | 1.32e-01 | 1.89e-01 |
|  | 7 | 89840708 | 89841037 | PDFSTATSENSEMBLUCSC | 330 | 10 | 37696 | tss | 0.334847 | 0.636007 | Not queried | 2.54 | 1.65e-01 | 2.29e-01 |
|  | 9 | 32384244 | 32384458 | PDFSTATSENSEMBLUCSC | 215 | 4 | 40806 | tss;island | 0.025851 | 0.008041 | Not queried | 2.54 | 1.14e-01 | 1.68e-01 |
|  | 1 | 3663164 | 3663832 | PDFSTATSENSEMBLUCSC | 669 | 6 | 144 | gene | 0.849878 | 0.968663 | Not queried | 2.54 | 2.60e-02 | 6.12e-02 |
|  | 11 | 35441558 | 35441900 | PDFSTATSENSEMBLUCSC | 343 | 5 | 7246 | tss;island | 0.574492 | 0.903238 | Not queried | 2.54 | 5.56e-02 | 1.00e-01 |
|  | 15 | 42289618 | 42289939 | PDFSTATSENSEMBLUCSC | 322 | 4 | 13306 | gene | 0.767534 | 0.328482 | Not queried | 2.54 | 1.14e-01 | 1.68e-01 |
|  | 20 | 61583537 | 61584159 | PDFSTATSENSEMBLUCSC | 623 | 11 | 26338 | island | 0.749194 | 0.956542 | SLC17A9 (margin: GID8; SLC17A9) | 2.54 | 1.86e-03 | 1.45e-02 |
|  | 7 | 43153023 | 43153392 | PDFSTATSENSEMBLUCSC | 370 | 5 | 36980 | gene;island | 0.408330 | 0.110814 | Not queried | 2.54 | 5.56e-02 | 1.00e-01 |
|  | 5 | 140186484 | 140186983 | PDFSTATSENSEMBLUCSC | 500 | 6 | 31795 | gene;island | 0.750971 | 0.900830 | Not queried | 2.54 | 9.31e-02 | 1.53e-01 |
|  | 11 | 19367501 | 19368277 | PDFSTATSENSEMBLUCSC | 777 | 8 | 8120 | island | 0.683496 | 0.940554 | Not queried | 2.53 | 2.07e-02 | 6.12e-02 |
|  | 3 | 126006749 | 126006903 | PDFSTATSENSEMBLUCSC | 155 | 4 | 29428 | island | 0.872134 | 0.976149 | Not queried | 2.53 | 2.86e-02 | 6.12e-02 |
|  | 6 | 26183508 | 26184245 | PDFSTATSENSEMBLUCSC | 738 | 12 | 35521 | island | 0.158193 | 0.459467 | HIST1H2BE (margin: HIST1H2BD; HIST1H2BE; HIST1H1PS1; HIST1H4D) | 2.53 | 8.29e-03 | 3.33e-02 |
|  | 5 | 61601529 | 61601647 | PDFSTATSENSEMBLUCSC | 119 | 4 | 32084 | tss;island | 0.310830 | 0.075149 | Not queried | 2.53 | 3.43e-01 | 3.84e-01 |
|  | 7 | 152591099 | 152592038 | PDFSTATSENSEMBLUCSC | 940 | 10 | 39052 | island | 0.583517 | 0.845599 | Not queried | 2.53 | 1.65e-01 | 2.29e-01 |
|  | 7 | 151136940 | 151137192 | PDFSTATSENSEMBLUCSC | 253 | 5 | 39040 | island | 0.862262 | 0.946307 | RP4-555L14.4; CRYGN (margin: RP4-555L14.4; CRYGN; MIR3907) | 2.53 | 7.94e-03 | 3.19e-02 |
|  | 12 | 7073179 | 7073247 | PDFSTATSENSEMBLUCSC | 69 | 4 | 9540 | tss | 0.164788 | 0.031518 | Not queried | 2.53 | 2.86e-02 | 6.12e-02 |
|  | 6 | 33156164 | 33156463 | PDFSTATSENSEMBLUCSC | 300 | 5 | 34212 | gene;island | 0.788590 | 0.950210 | Not queried | 2.53 | 1.51e-01 | 2.11e-01 |
|  | 11 | 82444798 | 82445170 | PDFSTATSENSEMBLUCSC | 373 | 5 | 8698 | island | 0.220388 | 0.598881 | Not queried | 2.53 | 1.51e-01 | 2.11e-01 |
|  | 13 | 113120945 | 113121280 | PDFSTATSENSEMBLUCSC | 336 | 4 | 11923 | island | 0.877757 | 0.951087 | Not queried | 2.53 | 2.00e-01 | 2.55e-01 |
|  | 11 | 77532174 | 77532226 | PDFSTATSENSEMBLUCSC | 53 | 4 | 7587 | tss;island | 0.007776 | 0.001688 | Not queried | 2.53 | 6.86e-01 | 6.99e-01 |
|  | 2 | 26569718 | 26570144 | PDFSTATSENSEMBLUCSC | 427 | 8 | 23242 | tss | 0.003638 | 0.025906 | Not queried | 2.53 | 3.79e-02 | 7.66e-02 |
|  | 13 | 111672375 | 111672726 | PDFSTATSENSEMBLUCSC | 352 | 4 | 11894 | island | 0.695749 | 0.946130 | Not queried | 2.53 | 2.00e-01 | 2.55e-01 |
|  | 11 | 128712280 | 128712697 | PDFSTATSENSEMBLUCSC | 418 | 6 | 6968 | gene | 0.381791 | 0.717226 | Not queried | 2.53 | 2.40e-01 | 2.95e-01 |
|  | 6 | 32086755 | 32087190 | PDFSTATSENSEMBLUCSC | 436 | 12 | 34053 | gene | 0.018130 | 0.163901 | Not queried | 2.53 | 2.42e-02 | 6.12e-02 |
|  | 16 | 58497714 | 58497815 | PDFSTATSENSEMBLUCSC | 102 | 4 | 14771 | gene;tss;island | 0.133770 | 0.426487 | Not queried | 2.53 | 5.71e-02 | 1.00e-01 |
|  | 6 | 32178603 | 32178775 | PDFSTATSENSEMBLUCSC | 173 | 4 | 34091 | gene | 0.662141 | 0.927563 | Not queried | 2.53 | 2.86e-02 | 6.12e-02 |
|  | 2 | 7017571 | 7017787 | PDFSTATSENSEMBLUCSC | 217 | 4 | 23167 | tss | 0.005081 | 0.027403 | Not queried | 2.52 | 4.86e-01 | 5.11e-01 |
|  | 10 | 82168043 | 82168295 | PDFSTATSENSEMBLUCSC | 253 | 6 | 5890 | island | 0.036516 | 0.006279 | FAM213A (margin: FAM213A) | 2.52 | 8.66e-03 | 3.36e-02 |
|  | 3 | 71833543 | 71833907 | PDFSTATSENSEMBLUCSC | 365 | 5 | 27860 | gene;island | 0.819900 | 0.965387 | PROK2 (margin: PROK2) | 2.52 | 7.94e-03 | 3.19e-02 |
|  | 22 | 36682843 | 36682870 | PDFSTATSENSEMBLUCSC | 28 | 4 | 26857 | gene | 0.777524 | 0.419344 | Not queried | 2.52 | 2.86e-02 | 6.12e-02 |
|  | 20 | 62367698 | 62367961 | PDFSTATSENSEMBLUCSC | 264 | 6 | 25902 | tss | 0.765154 | 0.970116 | Not queried | 2.52 | 6.49e-02 | 1.11e-01 |
|  | 14 | 101521409 | 101521809 | PDFSTATSENSEMBLUCSC | 401 | 10 | 12629 | tss | 0.495308 | 0.865412 | MIR134; MIR485; MIR323B; MIR382 (margin: MIR1185-2; MIR381HG; MIR381; MIR487B; MIR539; MIR889; MIR544A; MIR655; AL132709.3; MIR487A; MIR134; MIR485; MIR323B; MIR154; MIR496; MIR377; MIR541; MIR409; MIR412; MIR369; MIR410; MIR656; MIR382) | 2.52 | 4.87e-04 | 6.47e-03 |
|  | 3 | 49057661 | 49058586 | PDFSTATSENSEMBLUCSC | 926 | 16 | 27776 | gene;tss;island | 0.005954 | 0.039678 | NDUFAF3; DALRD3; MIR425; MIR191 (margin: WDR6; NDUFAF3; RP13-131K19.6; DALRD3; MIR425; MIR191; IMPDH2; QRICH1) | 2.52 | 6.61e-03 | 3.19e-02 |
|  | 17 | 80275498 | 80276134 | PDFSTATSENSEMBLUCSC | 637 | 7 | 17772 | tss | 0.671926 | 0.873004 | Not queried | 2.52 | 7.28e-02 | 1.23e-01 |
|  | 15 | 61521674 | 61522112 | PDFSTATSENSEMBLUCSC | 439 | 7 | 13713 | tss;island | 0.410639 | 0.671252 | Not queried | 2.52 | 3.83e-01 | 4.27e-01 |
|  | 11 | 128562003 | 128562408 | PDFSTATSENSEMBLUCSC | 406 | 7 | 7802 | tss;island | 0.560086 | 0.546443 | Not queried | 2.52 | 3.18e-01 | 3.74e-01 |
|  | 16 | 57570661 | 57570819 | PDFSTATSENSEMBLUCSC | 159 | 4 | 15265 | tss;island | 0.394609 | 0.099986 | Not queried | 2.52 | 5.71e-02 | 1.00e-01 |
|  | 2 | 241075901 | 241076777 | PDFSTATSENSEMBLUCSC | 877 | 25 | 23979 | tss;island | 0.466682 | 0.048560 | Not queried | 2.52 | 5.68e-02 | 1.00e-01 |
|  | 11 | 128712399 | 128712697 | PDFSTATSENSEMBLUCSC | 299 | 5 | 7804 | tss | 0.390465 | 0.837060 | Not queried | 2.52 | 2.22e-01 | 2.76e-01 |
|  | 5 | 524679 | 524941 | PDFSTATSENSEMBLUCSC | 263 | 4 | 31960 | tss | 0.206478 | 0.528976 | Not queried | 2.52 | 2.00e-01 | 2.55e-01 |
|  | 6 | 100903561 | 100904055 | PDFSTATSENSEMBLUCSC | 495 | 7 | 34431 | gene;island | 0.825648 | 0.972845 | Not queried | 2.52 | 1.65e-01 | 2.28e-01 |
|  | 2 | 223163573 | 223163809 | PDFSTATSENSEMBLUCSC | 237 | 5 | 23048 | gene;island | 0.914014 | 0.980121 | Not queried | 2.52 | 1.51e-01 | 2.11e-01 |
|  | 13 | 19755954 | 19756318 | PDFSTATSENSEMBLUCSC | 365 | 8 | 11322 | tss | 0.758194 | 0.901127 | Not queried | 2.52 | 1.48e-02 | 5.36e-02 |
|  | 3 | 183145817 | 183146204 | PDFSTATSENSEMBLUCSC | 388 | 6 | 29703 | island | 0.284184 | 0.071856 | Not queried | 2.51 | 2.60e-02 | 6.12e-02 |
|  | 3 | 183145870 | 183146204 | PDFSTATSENSEMBLUCSC | 335 | 4 | 28745 | tss | 0.236282 | 0.028225 | Not queried | 2.51 | 1.14e-01 | 1.68e-01 |
|  | 1 | 40782713 | 40783264 | PDFSTATSENSEMBLUCSC | 552 | 10 | 3120 | island | 0.896823 | 0.978132 | COL9A2 (margin: COL9A2) | 2.51 | 7.58e-05 | 1.98e-03 |
|  | 14 | 65569751 | 65570008 | PDFSTATSENSEMBLUCSC | 258 | 4 | 12467 | tss;island | 0.002357 | 0.001105 | Not queried | 2.51 | 3.43e-01 | 3.84e-01 |
|  | 14 | 76044591 | 76044828 | PDFSTATSENSEMBLUCSC | 238 | 4 | 12520 | tss | 0.132291 | 0.036531 | Not queried | 2.51 | 5.71e-02 | 1.00e-01 |
|  | 19 | 52900882 | 52900988 | PDFSTATSENSEMBLUCSC | 107 | 8 | 21013 | tss | 0.587496 | 0.876330 | Not queried | 2.51 | 4.99e-02 | 9.81e-02 |
|  | 20 | 31755609 | 31755943 | PDFSTATSENSEMBLUCSC | 335 | 4 | 25679 | tss | 0.635980 | 0.967910 | Not queried | 2.51 | 1.14e-01 | 1.68e-01 |
|  | 6 | 87646738 | 87646986 | PDFSTATSENSEMBLUCSC | 249 | 8 | 35106 | tss | 0.468065 | 0.047948 | Not queried | 2.51 | 4.99e-02 | 9.81e-02 |
|  | 7 | 96643454 | 96643821 | PDFSTATSENSEMBLUCSC | 368 | 5 | 37734 | tss;island | 0.060983 | 0.357131 | Not queried | 2.51 | 4.21e-01 | 4.59e-01 |
|  | 15 | 43531947 | 43532243 | PDFSTATSENSEMBLUCSC | 297 | 4 | 13314 | gene | 0.829522 | 0.969173 | Not queried | 2.51 | 2.00e-01 | 2.55e-01 |
|  | 15 | 52264002 | 52264396 | PDFSTATSENSEMBLUCSC | 395 | 8 | 13675 | tss | 0.003484 | 0.015065 | Not queried | 2.51 | 2.81e-02 | 6.12e-02 |
|  | 17 | 66097276 | 66097529 | PDFSTATSENSEMBLUCSC | 254 | 5 | 17602 | tss;island | 0.282100 | 0.031266 | Not queried | 2.51 | 1.51e-01 | 2.11e-01 |
|  | 17 | 79133095 | 79133388 | PDFSTATSENSEMBLUCSC | 294 | 4 | 16900 | gene;island | 0.744785 | 0.950405 | Not queried | 2.51 | 2.86e-02 | 6.12e-02 |
|  | 12 | 8025517 | 8025646 | PDFSTATSENSEMBLUCSC | 130 | 6 | 9551 | tss | 0.689092 | 0.904208 | Not queried | 2.51 | 1.80e-01 | 2.45e-01 |
|  | 19 | 3942158 | 3942330 | PDFSTATSENSEMBLUCSC | 173 | 4 | 19725 | gene;island | 0.931188 | 0.979350 | Not queried | 2.51 | 2.00e-01 | 2.55e-01 |
|  | 15 | 26109204 | 26109614 | PDFSTATSENSEMBLUCSC | 411 | 12 | 13540 | tss;island | 0.536286 | 0.917160 | Not queried | 2.51 | 1.45e-02 | 5.34e-02 |
|  | 2 | 220222978 | 220223283 | PDFSTATSENSEMBLUCSC | 306 | 4 | 25121 | island | 0.745965 | 0.949269 | Not queried | 2.51 | 2.86e-02 | 6.12e-02 |
|  | 13 | 46961583 | 46961897 | PDFSTATSENSEMBLUCSC | 315 | 10 | 11714 | island | 0.108088 | 0.011084 | Not queried | 2.51 | 1.85e-02 | 6.12e-02 |
|  | 1 | 68698376 | 68698718 | PDFSTATSENSEMBLUCSC | 343 | 7 | 1688 | tss;island | 0.113913 | 0.649492 | Not queried | 2.51 | 7.28e-02 | 1.23e-01 |
|  | 3 | 156878512 | 156878622 | PDFSTATSENSEMBLUCSC | 111 | 4 | 28678 | tss;island | 0.000131 | 0.000752 | Not queried | 2.51 | 2.86e-02 | 6.12e-02 |
|  | 11 | 32458656 | 32459057 | PDFSTATSENSEMBLUCSC | 402 | 5 | 6568 | gene;island | 0.336981 | 0.860130 | Not queried | 2.51 | 3.10e-01 | 3.66e-01 |
|  | 13 | 96743492 | 96743895 | PDFSTATSENSEMBLUCSC | 404 | 5 | 11195 | gene;island | 0.477226 | 0.298939 | Not queried | 2.50 | 9.52e-02 | 1.53e-01 |
|  | 22 | 50585197 | 50585427 | PDFSTATSENSEMBLUCSC | 231 | 8 | 26924 | gene;tss | 0.069048 | 0.282473 | Not queried | 2.50 | 1.05e-01 | 1.66e-01 |
|  | 8 | 48650511 | 48650745 | PDFSTATSENSEMBLUCSC | 235 | 4 | 40272 | island | 0.021971 | 0.032712 | Not queried | 2.50 | 3.43e-01 | 3.84e-01 |
|  | 3 | 183993341 | 183994242 | PDFSTATSENSEMBLUCSC | 902 | 12 | 28060 | gene;island | 0.793076 | 0.979036 | Not queried | 2.50 | 2.05e-02 | 6.12e-02 |
|  | 6 | 27486764 | 27487169 | PDFSTATSENSEMBLUCSC | 406 | 5 | 35559 | island | 0.001863 | 0.009998 | Not queried | 2.50 | 3.10e-01 | 3.66e-01 |
|  | 19 | 49133421 | 49133845 | PDFSTATSENSEMBLUCSC | 425 | 4 | 20133 | gene;island | 0.915954 | 0.989035 | Not queried | 2.50 | 1.14e-01 | 1.68e-01 |
|  | 11 | 299545 | 299668 | PDFSTATSENSEMBLUCSC | 124 | 5 | 7007 | tss | 0.479665 | 0.906709 | Not queried | 2.50 | 9.52e-02 | 1.53e-01 |
|  | 4 | 828098 | 828281 | PDFSTATSENSEMBLUCSC | 184 | 4 | 30738 | island | 0.747192 | 0.946872 | Not queried | 2.50 | 2.86e-02 | 6.12e-02 |
|  | 11 | 32354958 | 32355509 | PDFSTATSENSEMBLUCSC | 552 | 6 | 8174 | island | 0.782083 | 0.974499 | Not queried | 2.50 | 1.52e-02 | 5.36e-02 |
|  | 6 | 136172553 | 136172818 | PDFSTATSENSEMBLUCSC | 266 | 5 | 35239 | tss | 0.011150 | 0.001766 | Not queried | 2.50 | 2.22e-01 | 2.76e-01 |
|  | 4 | 105411974 | 105412299 | PDFSTATSENSEMBLUCSC | 326 | 4 | 30079 | gene | 0.089329 | 0.016011 | Not queried | 2.50 | 2.86e-02 | 6.12e-02 |
|  | 4 | 8207058 | 8207325 | PDFSTATSENSEMBLUCSC | 268 | 4 | 29949 | gene | 0.613638 | 0.861887 | Not queried | 2.50 | 4.86e-01 | 5.11e-01 |
|  | 15 | 75135717 | 75136007 | PDFSTATSENSEMBLUCSC | 291 | 4 | 13803 | tss;island | 0.134622 | 0.012604 | Not queried | 2.50 | 4.86e-01 | 5.11e-01 |
|  | 19 | 19314215 | 19314415 | PDFSTATSENSEMBLUCSC | 201 | 8 | 21807 | island | 0.057299 | 0.011634 | Not queried | 2.50 | 2.07e-02 | 6.12e-02 |
|  | 22 | 30642820 | 30643063 | PDFSTATSENSEMBLUCSC | 244 | 4 | 27056 | tss | 0.039728 | 0.286128 | Not queried | 2.50 | 2.00e-01 | 2.55e-01 |
|  | 13 | 114214248 | 114214740 | PDFSTATSENSEMBLUCSC | 493 | 5 | 11971 | island | 0.765412 | 0.977014 | Not queried | 2.50 | 2.22e-01 | 2.76e-01 |
|  | 11 | 14665335 | 14665558 | PDFSTATSENSEMBLUCSC | 224 | 4 | 6517 | gene;tss;island | 0.272306 | 0.075309 | Not queried | 2.50 | 2.86e-02 | 6.12e-02 |
|  | 13 | 28371375 | 28371682 | PDFSTATSENSEMBLUCSC | 308 | 4 | 11631 | island | 0.604929 | 0.964614 | Not queried | 2.50 | 5.71e-02 | 1.00e-01 |
|  | 1 | 158150581 | 158151363 | PDFSTATSENSEMBLUCSC | 783 | 8 | 835 | gene;island | 0.656690 | 0.913823 | Not queried | 2.50 | 4.99e-02 | 9.81e-02 |
|  | 4 | 1642054 | 1642257 | PDFSTATSENSEMBLUCSC | 204 | 4 | 29873 | gene;island | 0.778672 | 0.941236 | Not queried | 2.50 | 5.71e-02 | 1.00e-01 |
|  | 1 | 10719929 | 10720466 | PDFSTATSENSEMBLUCSC | 538 | 5 | 214 | gene;island | 0.932737 | 0.985922 | Not queried | 2.49 | 5.56e-02 | 1.00e-01 |
|  | 7 | 1018384 | 1018603 | PDFSTATSENSEMBLUCSC | 220 | 4 | 38062 | island | 0.715064 | 0.969678 | Not queried | 2.49 | 1.14e-01 | 1.68e-01 |
|  | 16 | 67678593 | 67678846 | PDFSTATSENSEMBLUCSC | 254 | 5 | 15307 | tss;island | 0.891240 | 0.974688 | RLTPR (margin: CTCF; RLTPR) | 2.49 | 7.94e-03 | 3.19e-02 |
|  | 17 | 59488988 | 59489098 | PDFSTATSENSEMBLUCSC | 111 | 4 | 17557 | tss | 0.023317 | 0.150525 | Not queried | 2.49 | 1.14e-01 | 1.68e-01 |
|  | 11 | 78673073 | 78673290 | PDFSTATSENSEMBLUCSC | 218 | 4 | 6847 | gene;island | 0.830796 | 0.985142 | Not queried | 2.49 | 1.14e-01 | 1.68e-01 |
|  | 17 | 56297193 | 56297515 | PDFSTATSENSEMBLUCSC | 323 | 4 | 17534 | tss;island | 0.479647 | 0.851594 | Not queried | 2.49 | 4.86e-01 | 5.11e-01 |
|  | 17 | 26574231 | 26574374 | PDFSTATSENSEMBLUCSC | 144 | 4 | 17206 | tss | 0.500236 | 0.859464 | Not queried | 2.49 | 2.86e-02 | 6.12e-02 |
|  | 6 | 32728786 | 32730299 | PDFSTATSENSEMBLUCSC | 1514 | 40 | 34132 | gene;island | 0.744104 | 0.943447 | HLA-DQB2 (margin: MIR3135B; HLA-DQB2) | 2.49 | 3.94e-04 | 5.86e-03 |
|  | 1 | 21044685 | 21044945 | PDFSTATSENSEMBLUCSC | 261 | 6 | 1318 | tss;island | 0.904914 | 0.602566 | Not queried | 2.49 | 2.60e-02 | 6.12e-02 |
|  | 22 | 19746984 | 19747140 | PDFSTATSENSEMBLUCSC | 157 | 4 | 26789 | gene;island | 0.870946 | 0.975937 | Not queried | 2.49 | 5.71e-02 | 1.00e-01 |
|  | 8 | 72756656 | 72757004 | PDFSTATSENSEMBLUCSC | 349 | 8 | 40383 | island | 0.663860 | 0.899784 | Not queried | 2.49 | 3.79e-02 | 7.66e-02 |
|  | 21 | 42792127 | 42792703 | PDFSTATSENSEMBLUCSC | 577 | 6 | 26688 | island | 0.003705 | 0.017576 | Not queried | 2.49 | 1.80e-01 | 2.45e-01 |
|  | 19 | 31210456 | 31210714 | PDFSTATSENSEMBLUCSC | 259 | 4 | 21861 | island | 0.111819 | 0.046905 | Not queried | 2.49 | 3.43e-01 | 3.84e-01 |
|  | 1 | 179711859 | 179712281 | PDFSTATSENSEMBLUCSC | 423 | 8 | 2120 | tss | 0.767768 | 0.946841 | RP11-12M5.1; FAM163A (margin: RP11-12M5.1; FAM163A) | 2.49 | 1.09e-03 | 1.10e-02 |
|  | 6 | 108396139 | 108396498 | PDFSTATSENSEMBLUCSC | 360 | 4 | 35157 | tss;island | 0.149285 | 0.010071 | Not queried | 2.49 | 4.86e-01 | 5.11e-01 |
|  | 19 | 12946231 | 12946465 | PDFSTATSENSEMBLUCSC | 235 | 6 | 20515 | tss | 0.350878 | 0.112208 | MAST1; HOOK2; RTBDN (margin: CTD-2265O21.3; MAST1; HOOK2; RTBDN) | 2.49 | 8.66e-03 | 3.36e-02 |
|  | 3 | 46506104 | 46506374 | PDFSTATSENSEMBLUCSC | 271 | 4 | 27749 | gene | 0.891147 | 0.950181 | Not queried | 2.49 | 2.00e-01 | 2.55e-01 |
|  | 6 | 35265308 | 35265568 | PDFSTATSENSEMBLUCSC | 261 | 9 | 34928 | tss | 0.114174 | 0.037034 | Not queried | 2.49 | 3.87e-01 | 4.30e-01 |
|  | 3 | 12837740 | 12838266 | PDFSTATSENSEMBLUCSC | 527 | 8 | 28894 | island | 0.710264 | 0.924459 | Not queried | 2.49 | 2.07e-02 | 6.12e-02 |
|  | 1 | 110672769 | 110673319 | PDFSTATSENSEMBLUCSC | 551 | 6 | 3575 | island | 0.690694 | 0.931158 | Not queried | 2.49 | 1.80e-01 | 2.45e-01 |
|  | 22 | 43820888 | 43821227 | PDFSTATSENSEMBLUCSC | 340 | 4 | 26889 | gene;island | 0.904616 | 0.981229 | Not queried | 2.49 | 5.71e-02 | 1.00e-01 |
|  | 22 | 50528179 | 50528333 | PDFSTATSENSEMBLUCSC | 155 | 7 | 27246 | tss;island | 0.860227 | 0.978178 | Not queried | 2.49 | 1.28e-01 | 1.87e-01 |
|  | 7 | 12250161 | 12250938 | PDFSTATSENSEMBLUCSC | 778 | 9 | 38265 | island | 0.030966 | 0.008734 | Not queried | 2.49 | 2.44e-02 | 6.12e-02 |
|  | 5 | 180486604 | 180486879 | PDFSTATSENSEMBLUCSC | 276 | 4 | 31949 | gene;island | 0.827234 | 0.936758 | Not queried | 2.49 | 5.71e-02 | 1.00e-01 |
|  | 18 | 42530692 | 42531132 | PDFSTATSENSEMBLUCSC | 441 | 8 | 19096 | gene | 0.163333 | 0.444016 | Not queried | 2.48 | 8.30e-02 | 1.40e-01 |
|  | 3 | 111717534 | 111717969 | PDFSTATSENSEMBLUCSC | 436 | 7 | 28498 | tss | 0.548037 | 0.827476 | TAGLN3 (margin: ABHD10; TAGLN3) | 2.48 | 5.83e-04 | 6.47e-03 |
|  | 16 | 2765620 | 2765900 | PDFSTATSENSEMBLUCSC | 281 | 5 | 14610 | gene;island | 0.887716 | 0.977965 | Not queried | 2.48 | 9.52e-02 | 1.53e-01 |
|  | 11 | 18230491 | 18231021 | PDFSTATSENSEMBLUCSC | 531 | 10 | 8107 | island | 0.823231 | 0.970257 | Not queried | 2.48 | 1.47e-02 | 5.36e-02 |
|  | 14 | 105154557 | 105155327 | PDFSTATSENSEMBLUCSC | 771 | 7 | 12660 | tss;island | 0.017051 | 0.247909 | Not queried | 2.48 | 2.62e-02 | 6.12e-02 |
|  | 11 | 118977782 | 118978076 | PDFSTATSENSEMBLUCSC | 295 | 6 | 7750 | tss | 0.011463 | 0.050309 | Not queried | 2.48 | 3.94e-01 | 4.36e-01 |
|  | 10 | 129704949 | 129705415 | PDFSTATSENSEMBLUCSC | 467 | 6 | 6250 | island | 0.022893 | 0.097007 | Not queried | 2.48 | 9.31e-02 | 1.53e-01 |
|  | 20 | 982623 | 982831 | PDFSTATSENSEMBLUCSC | 209 | 4 | 25326 | gene | 0.613216 | 0.908165 | Not queried | 2.48 | 5.71e-02 | 1.00e-01 |
|  | 10 | 88024529 | 88024673 | PDFSTATSENSEMBLUCSC | 145 | 7 | 4659 | gene;island | 0.591541 | 0.920639 | Not queried | 2.48 | 3.79e-02 | 7.66e-02 |
|  | 17 | 79520615 | 79521089 | PDFSTATSENSEMBLUCSC | 475 | 5 | 18930 | island | 0.069736 | 0.294673 | Not queried | 2.48 | 4.21e-01 | 4.59e-01 |
|  | 6 | 164758059 | 164758516 | PDFSTATSENSEMBLUCSC | 458 | 4 | 36620 | island | 0.714058 | 0.979400 | Not queried | 2.48 | 3.43e-01 | 3.84e-01 |
|  | 6 | 32526263 | 32526414 | PDFSTATSENSEMBLUCSC | 152 | 5 | 34118 | gene | 0.904651 | 0.436287 | Not queried | 2.48 | 5.56e-02 | 1.00e-01 |
|  | 14 | 24685281 | 24685421 | PDFSTATSENSEMBLUCSC | 141 | 4 | 12321 | tss | 0.011601 | 0.016055 | Not queried | 2.48 | 2.00e-01 | 2.55e-01 |
|  | 6 | 99292665 | 99293069 | PDFSTATSENSEMBLUCSC | 405 | 5 | 36348 | island | 0.682080 | 0.309753 | Not queried | 2.48 | 2.22e-01 | 2.76e-01 |
|  | 19 | 10047197 | 10047420 | PDFSTATSENSEMBLUCSC | 224 | 5 | 20434 | tss | 0.112250 | 0.021144 | Not queried | 2.48 | 1.59e-02 | 5.36e-02 |
|  | 4 | 47033126 | 47033274 | PDFSTATSENSEMBLUCSC | 149 | 5 | 30376 | tss | 0.131773 | 0.457391 | Not queried | 2.48 | 3.10e-01 | 3.66e-01 |
|  | 1 | 31845923 | 31846110 | PDFSTATSENSEMBLUCSC | 188 | 6 | 1445 | tss | 0.899896 | 0.973886 | FABP3 (margin: ZCCHC17; FABP3) | 2.48 | 2.16e-03 | 1.45e-02 |
|  | 1 | 2387670 | 2388073 | PDFSTATSENSEMBLUCSC | 404 | 5 | 2502 | island | 0.780167 | 0.927372 | Not queried | 2.48 | 5.56e-02 | 1.00e-01 |
|  | 8 | 88886051 | 88886432 | PDFSTATSENSEMBLUCSC | 382 | 9 | 40440 | island | 0.840976 | 0.980040 | Not queried | 2.48 | 1.42e-02 | 5.24e-02 |
|  | 1 | 113216774 | 113217043 | PDFSTATSENSEMBLUCSC | 270 | 5 | 1837 | tss | 0.011643 | 0.065822 | Not queried | 2.48 | 5.56e-02 | 1.00e-01 |
|  | 16 | 89686618 | 89686913 | PDFSTATSENSEMBLUCSC | 296 | 4 | 15428 | tss | 0.349023 | 0.752738 | Not queried | 2.48 | 5.71e-02 | 1.00e-01 |
|  | 16 | 27120945 | 27121267 | PDFSTATSENSEMBLUCSC | 323 | 4 | 15778 | island | 0.221955 | 0.100883 | Not queried | 2.48 | 6.86e-01 | 6.99e-01 |
|  | 22 | 25758455 | 25758749 | PDFSTATSENSEMBLUCSC | 295 | 5 | 26821 | gene;island | 0.849553 | 0.730853 | Not queried | 2.48 | 4.21e-01 | 4.59e-01 |
|  | 8 | 85096835 | 85097056 | PDFSTATSENSEMBLUCSC | 222 | 5 | 39789 | tss | 0.703274 | 0.444129 | RALYL (margin: RALYL) | 2.48 | 7.94e-03 | 3.19e-02 |
|  | 11 | 27721088 | 27721350 | PDFSTATSENSEMBLUCSC | 263 | 6 | 6544 | gene;island | 0.073547 | 0.217273 | Not queried | 2.47 | 1.32e-01 | 1.89e-01 |
|  | 18 | 77631625 | 77631756 | PDFSTATSENSEMBLUCSC | 132 | 4 | 19143 | gene;island | 0.715065 | 0.920083 | Not queried | 2.47 | 2.86e-02 | 6.12e-02 |
|  | 13 | 114786825 | 114786904 | PDFSTATSENSEMBLUCSC | 80 | 4 | 11307 | gene;island | 0.907439 | 0.981377 | Not queried | 2.47 | 1.14e-01 | 1.68e-01 |
|  | 3 | 119528956 | 119529290 | PDFSTATSENSEMBLUCSC | 335 | 4 | 27909 | gene;island | 0.952292 | 0.607030 | Not queried | 2.47 | 1.14e-01 | 1.68e-01 |
|  | 10 | 122739002 | 122739544 | PDFSTATSENSEMBLUCSC | 543 | 5 | 6199 | island | 0.481597 | 0.092143 | Not queried | 2.47 | 1.51e-01 | 2.11e-01 |
|  | 4 | 1762881 | 1763211 | PDFSTATSENSEMBLUCSC | 331 | 4 | 30798 | island | 0.567769 | 0.813981 | Not queried | 2.47 | 4.86e-01 | 5.11e-01 |
|  | 6 | 130686592 | 130686865 | PDFSTATSENSEMBLUCSC | 274 | 5 | 36477 | island | 0.880337 | 0.981155 | TMEM200A; SAMD3 (margin: TMEM200A; SAMD3) | 2.47 | 7.94e-03 | 3.19e-02 |
|  | 6 | 391664 | 391743 | PDFSTATSENSEMBLUCSC | 80 | 4 | 34586 | tss | 0.218170 | 0.653120 | Not queried | 2.47 | 2.00e-01 | 2.55e-01 |
|  | 6 | 30951084 | 30951394 | PDFSTATSENSEMBLUCSC | 311 | 4 | 34796 | tss | 0.775445 | 0.951999 | Not queried | 2.47 | 2.86e-02 | 6.12e-02 |
|  | 8 | 2078050 | 2078387 | PDFSTATSENSEMBLUCSC | 338 | 4 | 39189 | gene;island | 0.935257 | 0.668028 | Not queried | 2.47 | 5.71e-02 | 1.00e-01 |
|  | 8 | 143459774 | 143460209 | PDFSTATSENSEMBLUCSC | 436 | 6 | 39462 | gene | 0.863331 | 0.967512 | TSNARE1 (margin: TSNARE1) | 2.47 | 4.33e-03 | 2.52e-02 |
|  | 21 | 33031960 | 33032168 | PDFSTATSENSEMBLUCSC | 209 | 4 | 26397 | gene | 0.004230 | 0.009936 | Not queried | 2.47 | 2.00e-01 | 2.55e-01 |
|  | 17 | 46806445 | 46806935 | PDFSTATSENSEMBLUCSC | 491 | 6 | 17472 | tss;island | 0.674826 | 0.901872 | Not queried | 2.47 | 1.80e-01 | 2.45e-01 |
|  | 1 | 205537752 | 205537877 | PDFSTATSENSEMBLUCSC | 126 | 4 | 2206 | tss;island | 0.858957 | 0.520532 | Not queried | 2.47 | 5.71e-02 | 1.00e-01 |
|  | 12 | 70083090 | 70083213 | PDFSTATSENSEMBLUCSC | 124 | 5 | 9212 | gene;tss | 0.055595 | 0.164312 | Not queried | 2.47 | 3.17e-02 | 6.50e-02 |
|  | 5 | 76939333 | 76939558 | PDFSTATSENSEMBLUCSC | 226 | 4 | 32904 | island | 0.643105 | 0.944889 | Not queried | 2.47 | 5.71e-02 | 1.00e-01 |
|  | 15 | 52263485 | 52264396 | PDFSTATSENSEMBLUCSC | 912 | 13 | 14145 | island | 0.003701 | 0.012777 | Not queried | 2.47 | 1.40e-02 | 5.20e-02 |
|  | 1 | 4192648 | 4193192 | PDFSTATSENSEMBLUCSC | 545 | 5 | 2581 | island | 0.408510 | 0.779257 | Not queried | 2.47 | 9.52e-02 | 1.53e-01 |
|  | 17 | 35290307 | 35291580 | PDFSTATSENSEMBLUCSC | 1274 | 12 | 18235 | island | 0.896785 | 0.980525 | RP11-445F12.1 (margin: LHX1; RP11-445F12.1; RP11-445F12.2) | 2.47 | 1.11e-03 | 1.12e-02 |
|  | 8 | 97507561 | 97507666 | PDFSTATSENSEMBLUCSC | 106 | 4 | 39369 | gene;island | 0.197378 | 0.033969 | Not queried | 2.47 | 2.00e-01 | 2.55e-01 |
|  | 1 | 184943512 | 184943630 | PDFSTATSENSEMBLUCSC | 119 | 4 | 921 | gene;island | 0.008061 | 0.024645 | Not queried | 2.47 | 1.14e-01 | 1.68e-01 |
|  | 5 | 140764302 | 140764748 | PDFSTATSENSEMBLUCSC | 447 | 4 | 31826 | gene;island | 0.461934 | 0.837913 | Not queried | 2.47 | 1.14e-01 | 1.68e-01 |
|  | 19 | 12476027 | 12476363 | PDFSTATSENSEMBLUCSC | 337 | 4 | 19854 | gene | 0.180401 | 0.496068 | Not queried | 2.47 | 3.43e-01 | 3.84e-01 |
|  | 2 | 74757141 | 74757221 | PDFSTATSENSEMBLUCSC | 81 | 5 | 22749 | gene;tss;island | 0.002273 | 0.005313 | Not queried | 2.47 | 1.59e-02 | 5.36e-02 |
|  | 10 | 57387511 | 57387715 | PDFSTATSENSEMBLUCSC | 205 | 4 | 5753 | island | 0.697752 | 0.941199 | Not queried | 2.47 | 3.43e-01 | 3.84e-01 |
|  | 2 | 27505396 | 27505593 | PDFSTATSENSEMBLUCSC | 198 | 5 | 23262 | tss | 0.552309 | 0.918981 | Not queried | 2.47 | 1.59e-02 | 5.36e-02 |
|  | 11 | 82444946 | 82445170 | PDFSTATSENSEMBLUCSC | 225 | 4 | 7594 | tss | 0.409663 | 0.681533 | Not queried | 2.47 | 2.00e-01 | 2.55e-01 |
|  | 10 | 126138415 | 126138879 | PDFSTATSENSEMBLUCSC | 465 | 7 | 6228 | island | 0.147673 | 0.487684 | RP13-238F13.3; NKX1-2 (margin: RP13-238F13.3; LHPP; RP13-238F13.5; NKX1-2) | 2.47 | 5.83e-04 | 6.47e-03 |
|  | 1 | 75593804 | 75594066 | PDFSTATSENSEMBLUCSC | 263 | 5 | 1698 | tss | 0.830079 | 0.948784 | Not queried | 2.46 | 3.17e-02 | 6.50e-02 |
|  | 10 | 43278214 | 43278349 | PDFSTATSENSEMBLUCSC | 136 | 4 | 4566 | gene;island | 0.013198 | 0.082596 | Not queried | 2.46 | 2.00e-01 | 2.55e-01 |
|  | 10 | 103052161 | 103052605 | PDFSTATSENSEMBLUCSC | 445 | 5 | 6066 | island | 0.353245 | 0.035106 | Not queried | 2.46 | 2.22e-01 | 2.76e-01 |
|  | 17 | 41131966 | 41132169 | PDFSTATSENSEMBLUCSC | 204 | 5 | 16591 | gene;tss;island | 0.035875 | 0.022602 | Not queried | 2.46 | 8.41e-01 | 8.48e-01 |
|  | 11 | 68748461 | 68748584 | PDFSTATSENSEMBLUCSC | 124 | 4 | 7514 | tss | 0.232508 | 0.538686 | Not queried | 2.46 | 3.43e-01 | 3.84e-01 |
|  | 20 | 61041274 | 61041765 | PDFSTATSENSEMBLUCSC | 492 | 5 | 25486 | gene;island | 0.427169 | 0.885369 | Not queried | 2.46 | 1.59e-02 | 5.36e-02 |
|  | 16 | 85517170 | 85517684 | PDFSTATSENSEMBLUCSC | 515 | 7 | 16156 | island | 0.873342 | 0.970839 | Not queried | 2.46 | 1.28e-01 | 1.87e-01 |
|  | 13 | 37004536 | 37005582 | PDFSTATSENSEMBLUCSC | 1047 | 29 | 11396 | tss;island | 0.745720 | 0.934621 | CCNA1 (margin: CCNA1) | 2.46 | 2.32e-04 | 3.81e-03 |
|  | 1 | 8938701 | 8938754 | PDFSTATSENSEMBLUCSC | 54 | 4 | 198 | gene | 0.010389 | 0.003610 | Not queried | 2.46 | 4.86e-01 | 5.11e-01 |
|  | 5 | 122372722 | 122372942 | PDFSTATSENSEMBLUCSC | 221 | 5 | 32247 | tss;island | 0.004917 | 0.005239 | Not queried | 2.46 | 6.90e-01 | 7.01e-01 |
|  | 4 | 123747447 | 123747750 | PDFSTATSENSEMBLUCSC | 304 | 7 | 30581 | tss;island | 0.919190 | 0.979003 | FGF2; AC021205.1 (margin: FGF2; AC021205.1; RP11-170N16.1) | 2.46 | 6.99e-03 | 3.19e-02 |
|  | 13 | 47371412 | 47371644 | PDFSTATSENSEMBLUCSC | 233 | 8 | 11428 | tss | 0.086462 | 0.342683 | Not queried | 2.46 | 1.61e-01 | 2.24e-01 |
|  | 10 | 1256339 | 1256487 | PDFSTATSENSEMBLUCSC | 149 | 4 | 5492 | island | 0.093632 | 0.336580 | Not queried | 2.46 | 2.86e-02 | 6.12e-02 |
|  | 10 | 1256339 | 1256651 | PDFSTATSENSEMBLUCSC | 313 | 6 | 4459 | gene | 0.147398 | 0.402399 | Not queried | 2.46 | 1.32e-01 | 1.89e-01 |
|  | 15 | 63481323 | 63481580 | PDFSTATSENSEMBLUCSC | 258 | 5 | 13720 | tss;island | 0.013995 | 0.009607 | Not queried | 2.46 | 3.10e-01 | 3.66e-01 |
|  | 2 | 241640149 | 241640444 | PDFSTATSENSEMBLUCSC | 296 | 4 | 25278 | island | 0.820793 | 0.958119 | Not queried | 2.46 | 2.00e-01 | 2.55e-01 |
|  | 6 | 31475762 | 31475984 | PDFSTATSENSEMBLUCSC | 223 | 4 | 33885 | gene | 0.960141 | 0.743883 | Not queried | 2.46 | 1.14e-01 | 1.68e-01 |
|  | 4 | 145568182 | 145568399 | PDFSTATSENSEMBLUCSC | 218 | 4 | 30143 | gene;island | 0.713918 | 0.302836 | Not queried | 2.46 | 5.71e-02 | 1.00e-01 |
|  | 6 | 18264719 | 18264854 | PDFSTATSENSEMBLUCSC | 136 | 4 | 35480 | island | 0.004219 | 0.018288 | Not queried | 2.46 | 3.43e-01 | 3.84e-01 |
|  | 17 | 52977769 | 52978035 | PDFSTATSENSEMBLUCSC | 267 | 7 | 17517 | tss | 0.162646 | 0.047716 | Not queried | 2.46 | 7.28e-02 | 1.23e-01 |
|  | 18 | 10414355 | 10414556 | PDFSTATSENSEMBLUCSC | 202 | 4 | 19389 | island | 0.904387 | 0.976800 | Not queried | 2.46 | 2.00e-01 | 2.55e-01 |
|  | 12 | 21590246 | 21590536 | PDFSTATSENSEMBLUCSC | 291 | 6 | 9598 | tss | 0.011532 | 0.006846 | Not queried | 2.46 | 4.85e-01 | 5.11e-01 |
|  | 12 | 57588031 | 57588243 | PDFSTATSENSEMBLUCSC | 213 | 7 | 9811 | tss | 0.972239 | 0.832512 | LRP1; MIR1228 (margin: LRP1; MIR1228) | 2.46 | 2.33e-03 | 1.52e-02 |
|  | 19 | 2717293 | 2717615 | PDFSTATSENSEMBLUCSC | 323 | 4 | 19700 | gene;island | 0.771956 | 0.943476 | Not queried | 2.46 | 3.43e-01 | 3.84e-01 |
|  | 4 | 154605087 | 154605468 | PDFSTATSENSEMBLUCSC | 382 | 5 | 31375 | island | 0.536941 | 0.157156 | (margin: RP11-153M7.3) | 2.46 | 7.94e-03 | 3.19e-02 |
|  | 6 | 76022192 | 76022556 | PDFSTATSENSEMBLUCSC | 365 | 6 | 34395 | gene | 0.768296 | 0.964394 | Not queried | 2.46 | 1.52e-02 | 5.36e-02 |
|  | 22 | 24823389 | 24823519 | PDFSTATSENSEMBLUCSC | 131 | 5 | 27019 | tss | 0.866551 | 0.921285 | Not queried | 2.45 | 3.10e-01 | 3.66e-01 |
|  | 5 | 125800764 | 125800916 | PDFSTATSENSEMBLUCSC | 153 | 4 | 31734 | gene | 0.970034 | 0.904235 | Not queried | 2.45 | 5.71e-02 | 1.00e-01 |
|  | 1 | 1875780 | 1876015 | PDFSTATSENSEMBLUCSC | 236 | 4 | 2465 | island | 0.426812 | 0.071226 | Not queried | 2.45 | 6.86e-01 | 6.99e-01 |
|  | 2 | 192746039 | 192746213 | PDFSTATSENSEMBLUCSC | 175 | 4 | 24969 | island | 0.959605 | 0.825038 | Not queried | 2.45 | 3.43e-01 | 3.84e-01 |
|  | 3 | 108836693 | 108836912 | PDFSTATSENSEMBLUCSC | 220 | 4 | 27889 | gene | 0.468318 | 0.855150 | Not queried | 2.45 | 4.86e-01 | 5.11e-01 |
|  | 15 | 32322525 | 32323114 | PDFSTATSENSEMBLUCSC | 590 | 8 | 13987 | island | 0.114177 | 0.026220 | Not queried | 2.45 | 2.81e-02 | 6.12e-02 |
|  | 15 | 32322829 | 32323114 | PDFSTATSENSEMBLUCSC | 286 | 6 | 13275 | gene | 0.114177 | 0.022429 | Not queried | 2.45 | 4.11e-02 | 8.14e-02 |
|  | 14 | 101531403 | 101532352 | PDFSTATSENSEMBLUCSC | 950 | 20 | 12634 | tss;island | 0.565064 | 0.922784 | MIR541; MIR409; MIR412; MIR369; MIR410; MIR656 (margin: MIR134; MIR485; MIR323B; MIR154; MIR496; MIR377; MIR541; MIR409; MIR412; MIR369; MIR410; MIR656; MEG9; MIR382; AL132709.1) | 2.45 | 5.13e-03 | 2.94e-02 |
|  | 17 | 46831306 | 46831742 | PDFSTATSENSEMBLUCSC | 437 | 4 | 18508 | island | 0.656214 | 0.964285 | Not queried | 2.45 | 1.14e-01 | 1.68e-01 |
|  | 4 | 81106240 | 81106414 | PDFSTATSENSEMBLUCSC | 175 | 6 | 30459 | tss | 0.683401 | 0.865791 | Not queried | 2.45 | 2.40e-01 | 2.95e-01 |
|  | 2 | 26624823 | 26625047 | PDFSTATSENSEMBLUCSC | 225 | 4 | 22613 | gene | 0.082945 | 0.278946 | Not queried | 2.45 | 2.86e-02 | 6.12e-02 |
|  | 13 | 114861689 | 114862026 | PDFSTATSENSEMBLUCSC | 338 | 5 | 11313 | gene;island | 0.935967 | 0.988766 | Not queried | 2.45 | 1.59e-02 | 5.36e-02 |
|  | 7 | 157345452 | 157345827 | PDFSTATSENSEMBLUCSC | 376 | 6 | 37325 | gene;island | 0.387797 | 0.397990 | Not queried | 2.45 | 4.85e-01 | 5.11e-01 |
|  | 19 | 46806907 | 46807660 | PDFSTATSENSEMBLUCSC | 754 | 6 | 20109 | gene;island | 0.851323 | 0.980401 | Not queried | 2.45 | 2.40e-01 | 2.95e-01 |
|  | 15 | 90319420 | 90319680 | PDFSTATSENSEMBLUCSC | 261 | 5 | 14442 | island | 0.934200 | 0.953533 | Not queried | 2.45 | 2.22e-01 | 2.76e-01 |
|  | 17 | 34062219 | 34062573 | PDFSTATSENSEMBLUCSC | 355 | 5 | 16524 | gene;island | 0.750877 | 0.979933 | Not queried | 2.45 | 9.52e-02 | 1.53e-01 |
|  | 6 | 100055662 | 100056127 | PDFSTATSENSEMBLUCSC | 466 | 5 | 34428 | gene;island | 0.726936 | 0.935962 | Not queried | 2.45 | 2.22e-01 | 2.76e-01 |
|  | 1 | 231176985 | 231177201 | PDFSTATSENSEMBLUCSC | 217 | 4 | 2320 | tss;island | 0.937835 | 0.785996 | Not queried | 2.45 | 2.00e-01 | 2.55e-01 |
|  | 10 | 104178670 | 104179054 | PDFSTATSENSEMBLUCSC | 385 | 8 | 5322 | tss;island | 0.294991 | 0.661310 | Not queried | 2.45 | 1.30e-01 | 1.89e-01 |
|  | 4 | 111544235 | 111544463 | PDFSTATSENSEMBLUCSC | 229 | 8 | 30095 | gene;island | 0.038634 | 0.007945 | PITX2 (margin: RP11-380D23.2; PITX2) | 2.45 | 6.99e-03 | 3.19e-02 |
|  | 13 | 113724113 | 113724548 | PDFSTATSENSEMBLUCSC | 436 | 4 | 11273 | gene;island | 0.626029 | 0.889020 | Not queried | 2.45 | 1.14e-01 | 1.68e-01 |
|  | 10 | 105036611 | 105036863 | PDFSTATSENSEMBLUCSC | 253 | 7 | 5334 | tss;island | 0.949957 | 0.986519 | INA (margin: INA) | 2.45 | 6.99e-03 | 3.19e-02 |
|  | 5 | 145713607 | 145713882 | PDFSTATSENSEMBLUCSC | 276 | 6 | 33279 | island | 0.668750 | 0.933420 | Not queried | 2.45 | 9.31e-02 | 1.53e-01 |
|  | 10 | 73497514 | 73497772 | PDFSTATSENSEMBLUCSC | 259 | 4 | 4623 | gene | 0.686858 | 0.882873 | Not queried | 2.44 | 2.00e-01 | 2.55e-01 |
|  | 1 | 203595809 | 203596190 | PDFSTATSENSEMBLUCSC | 382 | 6 | 4107 | island | 0.002284 | 0.008674 | Not queried | 2.44 | 4.11e-02 | 8.14e-02 |
|  | 10 | 108924366 | 108924560 | PDFSTATSENSEMBLUCSC | 195 | 4 | 5351 | tss;island | 0.939502 | 0.739645 | Not queried | 2.44 | 2.86e-02 | 6.12e-02 |
|  | 19 | 23386650 | 23387133 | PDFSTATSENSEMBLUCSC | 484 | 5 | 21845 | island | 0.603953 | 0.895596 | Not queried | 2.44 | 3.17e-02 | 6.50e-02 |
|  | 2 | 233390712 | 233390969 | PDFSTATSENSEMBLUCSC | 258 | 5 | 25198 | island | 0.337722 | 0.802097 | Not queried | 2.44 | 5.56e-02 | 1.00e-01 |
|  | 19 | 36236496 | 36236615 | PDFSTATSENSEMBLUCSC | 120 | 4 | 19991 | gene | 0.001141 | 0.003617 | Not queried | 2.44 | 3.43e-01 | 3.84e-01 |
|  | 2 | 177028606 | 177028714 | PDFSTATSENSEMBLUCSC | 109 | 4 | 23742 | tss | 0.485963 | 0.869678 | Not queried | 2.44 | 3.43e-01 | 3.84e-01 |
|  | 3 | 169530268 | 169530621 | PDFSTATSENSEMBLUCSC | 354 | 6 | 29650 | island | 0.597743 | 0.911782 | LRRC34 (margin: LRRIQ4; LRRC34) | 2.44 | 2.16e-03 | 1.45e-02 |
|  | 13 | 25620833 | 25621118 | PDFSTATSENSEMBLUCSC | 286 | 5 | 11608 | island | 0.405677 | 0.941150 | Not queried | 2.44 | 2.22e-01 | 2.76e-01 |
|  | 18 | 21719263 | 21719433 | PDFSTATSENSEMBLUCSC | 171 | 5 | 19210 | tss | 0.015348 | 0.078189 | Not queried | 2.44 | 1.59e-02 | 5.36e-02 |
|  | 8 | 41655673 | 41656079 | PDFSTATSENSEMBLUCSC | 407 | 6 | 39294 | gene;tss;island | 0.615229 | 0.841377 | Not queried | 2.44 | 2.60e-02 | 6.12e-02 |
|  | 4 | 57410776 | 57411219 | PDFSTATSENSEMBLUCSC | 444 | 5 | 31069 | island | 0.371883 | 0.112490 | THEGL (margin: THEGL) | 2.44 | 7.94e-03 | 3.19e-02 |
|  | 16 | 8768488 | 8768700 | PDFSTATSENSEMBLUCSC | 213 | 4 | 14651 | gene | 0.033525 | 0.007967 | Not queried | 2.44 | 3.43e-01 | 3.84e-01 |
|  | 10 | 130339134 | 130339927 | PDFSTATSENSEMBLUCSC | 794 | 12 | 6256 | island | 0.886813 | 0.030842 | (margin: ) | 2.44 | 1.43e-03 | 1.36e-02 |
|  | 11 | 125036088 | 125036420 | PDFSTATSENSEMBLUCSC | 333 | 4 | 6957 | gene;island | 0.726163 | 0.965793 | Not queried | 2.44 | 2.00e-01 | 2.55e-01 |
|  | 14 | 77732283 | 77732641 | PDFSTATSENSEMBLUCSC | 359 | 5 | 12150 | gene | 0.540035 | 0.848896 | Not queried | 2.44 | 4.21e-01 | 4.59e-01 |
|  | 7 | 140103545 | 140103783 | PDFSTATSENSEMBLUCSC | 239 | 7 | 37937 | tss | 0.119697 | 0.029026 | Not queried | 2.44 | 9.73e-02 | 1.55e-01 |
|  | 4 | 155662217 | 155662515 | PDFSTATSENSEMBLUCSC | 299 | 4 | 31385 | island | 0.509841 | 0.882384 | Not queried | 2.44 | 1.00e+00 | 1.00e+00 |
|  | 14 | 104165052 | 104165475 | PDFSTATSENSEMBLUCSC | 424 | 5 | 12239 | gene;island | 0.884001 | 0.580394 | Not queried | 2.44 | 3.17e-02 | 6.50e-02 |
|  | 2 | 198669113 | 198669339 | PDFSTATSENSEMBLUCSC | 227 | 6 | 23804 | tss;island | 0.122686 | 0.014745 | PLCL1 (margin: PLCL1) | 2.44 | 4.33e-03 | 2.52e-02 |
|  | 1 | 35258435 | 35258933 | PDFSTATSENSEMBLUCSC | 499 | 4 | 3025 | island | 0.821436 | 0.974197 | Not queried | 2.44 | 1.14e-01 | 1.68e-01 |
|  | 19 | 17633880 | 17634172 | PDFSTATSENSEMBLUCSC | 293 | 5 | 21746 | island | 0.478793 | 0.950208 | Not queried | 2.44 | 5.56e-02 | 1.00e-01 |
|  | 17 | 14206774 | 14207036 | PDFSTATSENSEMBLUCSC | 263 | 7 | 16450 | gene;tss;island | 0.113784 | 0.658516 | Not queried | 2.44 | 7.28e-02 | 1.23e-01 |
|  | 12 | 65562903 | 65563329 | PDFSTATSENSEMBLUCSC | 427 | 7 | 9844 | tss;island | 0.086237 | 0.006467 | Not queried | 2.44 | 2.62e-02 | 6.12e-02 |
|  | 17 | 46655164 | 46655736 | PDFSTATSENSEMBLUCSC | 573 | 11 | 16653 | gene | 0.843091 | 0.977841 | HOXB-AS3; HOXB3; HOXB4; MIR10A (margin: HOXB-AS3; HOXB3; HOXB4; MIR10A) | 2.44 | 1.28e-04 | 2.71e-03 |
|  | 6 | 29623646 | 29623992 | PDFSTATSENSEMBLUCSC | 347 | 4 | 34751 | tss | 0.627948 | 0.934057 | Not queried | 2.44 | 2.00e-01 | 2.55e-01 |
|  | 20 | 55100881 | 55101089 | PDFSTATSENSEMBLUCSC | 209 | 5 | 25464 | gene | 0.569028 | 0.883050 | Not queried | 2.44 | 1.51e-01 | 2.11e-01 |
|  | 14 | 58711355 | 58711570 | PDFSTATSENSEMBLUCSC | 216 | 4 | 12422 | tss | 0.001295 | 0.006291 | Not queried | 2.43 | 3.43e-01 | 3.84e-01 |
|  | 10 | 50816917 | 50817306 | PDFSTATSENSEMBLUCSC | 390 | 6 | 5073 | tss;island | 0.515771 | 0.800510 | Not queried | 2.43 | 2.60e-02 | 6.12e-02 |
|  | 14 | 23844876 | 23845091 | PDFSTATSENSEMBLUCSC | 216 | 4 | 12030 | gene;tss | 0.803385 | 0.947277 | Not queried | 2.43 | 2.86e-02 | 6.12e-02 |
|  | 19 | 450940 | 451320 | PDFSTATSENSEMBLUCSC | 381 | 4 | 19627 | gene;island | 0.840838 | 0.951171 | Not queried | 2.43 | 2.86e-02 | 6.12e-02 |
|  | 10 | 52750995 | 52751341 | PDFSTATSENSEMBLUCSC | 347 | 4 | 4589 | gene | 0.744505 | 0.334099 | Not queried | 2.43 | 5.71e-02 | 1.00e-01 |
|  | 6 | 391664 | 392131 | PDFSTATSENSEMBLUCSC | 468 | 7 | 35362 | island | 0.832657 | 0.943732 | Not queried | 2.43 | 1.28e-01 | 1.87e-01 |
|  | 7 | 150210742 | 150211152 | PDFSTATSENSEMBLUCSC | 411 | 5 | 37980 | tss | 0.362019 | 0.830101 | Not queried | 2.43 | 3.10e-01 | 3.66e-01 |
|  | 2 | 223162666 | 223163326 | PDFSTATSENSEMBLUCSC | 661 | 9 | 23047 | gene;island | 0.810193 | 0.979164 | CCDC140; PAX3 (margin: CCDC140; PAX3) | 2.43 | 3.99e-03 | 2.47e-02 |
|  | 10 | 129704949 | 129705218 | PDFSTATSENSEMBLUCSC | 270 | 5 | 5437 | tss | 0.022932 | 0.103247 | Not queried | 2.43 | 3.17e-02 | 6.50e-02 |
|  | 19 | 17137685 | 17137714 | PDFSTATSENSEMBLUCSC | 30 | 4 | 20578 | tss | 0.873414 | 0.974108 | Not queried | 2.43 | 2.86e-02 | 6.12e-02 |
|  | 6 | 33171765 | 33172398 | PDFSTATSENSEMBLUCSC | 634 | 10 | 34894 | tss | 0.073821 | 0.407606 | Not queried | 2.43 | 1.05e-01 | 1.66e-01 |
|  | 6 | 31507701 | 31507879 | PDFSTATSENSEMBLUCSC | 179 | 4 | 33893 | gene;island | 0.492097 | 0.875416 | Not queried | 2.43 | 3.43e-01 | 3.84e-01 |
|  | 13 | 95364333 | 95364675 | PDFSTATSENSEMBLUCSC | 343 | 7 | 11822 | island | 0.619329 | 0.908187 | SOX21-AS1; SOX21 (margin: SOX21-AS1; LINC00391; SOX21) | 2.43 | 6.99e-03 | 3.19e-02 |
|  | 13 | 25338113 | 25338289 | PDFSTATSENSEMBLUCSC | 177 | 6 | 11347 | tss | 0.809222 | 0.966066 | Not queried | 2.43 | 2.40e-01 | 2.95e-01 |
|  | 1 | 40782991 | 40783264 | PDFSTATSENSEMBLUCSC | 274 | 7 | 1535 | tss | 0.900811 | 0.977509 | COL9A2 (margin: COL9A2) | 2.43 | 4.08e-03 | 2.47e-02 |
|  | 4 | 2303638 | 2303860 | PDFSTATSENSEMBLUCSC | 223 | 5 | 29887 | gene;island | 0.803740 | 0.956245 | Not queried | 2.43 | 2.22e-01 | 2.76e-01 |
|  | 14 | 45356607 | 45357076 | PDFSTATSENSEMBLUCSC | 470 | 5 | 12810 | island | 0.719000 | 0.921213 | Not queried | 2.43 | 5.56e-02 | 1.00e-01 |
|  | 3 | 123987591 | 123988013 | PDFSTATSENSEMBLUCSC | 423 | 6 | 27921 | gene;island | 0.537687 | 0.888231 | Not queried | 2.43 | 1.80e-01 | 2.45e-01 |
|  | 1 | 11822116 | 11822362 | PDFSTATSENSEMBLUCSC | 247 | 4 | 2688 | island | 0.703923 | 0.311437 | Not queried | 2.43 | 1.14e-01 | 1.68e-01 |
|  | 10 | 102893925 | 102894639 | PDFSTATSENSEMBLUCSC | 715 | 8 | 4724 | gene;island | 0.901336 | 0.977732 | TLX1; RP11-31L23.3 (margin: HUG1; TLX1; TLX1NB; RP11-31L23.3) | 2.43 | 1.09e-03 | 1.10e-02 |
|  | 1 | 236304799 | 236305169 | PDFSTATSENSEMBLUCSC | 371 | 5 | 2340 | tss;island | 0.073175 | 0.006843 | Not queried | 2.43 | 4.21e-01 | 4.59e-01 |
|  | 1 | 55464530 | 55464927 | PDFSTATSENSEMBLUCSC | 398 | 5 | 3305 | island | 0.715312 | 0.878508 | Not queried | 2.43 | 4.21e-01 | 4.59e-01 |
|  | 20 | 33879989 | 33880370 | PDFSTATSENSEMBLUCSC | 382 | 8 | 26123 | island | 0.607092 | 0.889516 | Not queried | 2.42 | 1.05e-01 | 1.66e-01 |
|  | 2 | 239139911 | 239141043 | PDFSTATSENSEMBLUCSC | 1133 | 17 | 23972 | tss;island | 0.764399 | 0.969619 | AC096574.4; AC016757.3 (margin: AC096574.4; AC016757.3; HES6; PER2) | 2.42 | 1.00e-03 | 1.04e-02 |
|  | 11 | 3177622 | 3178322 | PDFSTATSENSEMBLUCSC | 701 | 6 | 6476 | gene;island | 0.896637 | 0.973982 | Not queried | 2.42 | 4.11e-02 | 8.14e-02 |
|  | 6 | 41168941 | 41169171 | PDFSTATSENSEMBLUCSC | 231 | 6 | 34967 | tss | 0.598398 | 0.921756 | Not queried | 2.42 | 6.49e-02 | 1.11e-01 |
|  | 2 | 68546467 | 68546688 | PDFSTATSENSEMBLUCSC | 222 | 4 | 22712 | gene;island | 0.869972 | 0.976448 | Not queried | 2.42 | 2.86e-02 | 6.12e-02 |
|  | 1 | 113008799 | 113009082 | PDFSTATSENSEMBLUCSC | 284 | 7 | 1834 | tss;island | 0.065004 | 0.009950 | Not queried | 2.42 | 7.28e-02 | 1.23e-01 |
|  | 6 | 31702632 | 31702915 | PDFSTATSENSEMBLUCSC | 284 | 5 | 33948 | gene | 0.003025 | 0.043434 | Not queried | 2.42 | 9.52e-02 | 1.53e-01 |
|  | 20 | 61810348 | 61810902 | PDFSTATSENSEMBLUCSC | 555 | 5 | 26344 | island | 0.108586 | 0.014517 | Not queried | 2.42 | 1.51e-01 | 2.11e-01 |
|  | 6 | 26021916 | 26022273 | PDFSTATSENSEMBLUCSC | 358 | 5 | 33596 | gene | 0.001276 | 0.006024 | Not queried | 2.42 | 9.52e-02 | 1.53e-01 |
|  | 2 | 162273011 | 162273648 | PDFSTATSENSEMBLUCSC | 638 | 7 | 22909 | gene;island | 0.919284 | 0.970969 | Not queried | 2.42 | 7.28e-02 | 1.23e-01 |
|  | 10 | 7621729 | 7622106 | PDFSTATSENSEMBLUCSC | 378 | 4 | 4483 | gene;island | 0.556380 | 0.842933 | Not queried | 2.42 | 2.00e-01 | 2.55e-01 |
|  | 17 | 15847977 | 15848264 | PDFSTATSENSEMBLUCSC | 288 | 9 | 18045 | island | 0.052174 | 0.016739 | ADORA2B (margin: ADORA2B) | 2.42 | 1.06e-02 | 4.01e-02 |
|  | 12 | 47473761 | 47474262 | PDFSTATSENSEMBLUCSC | 502 | 10 | 9660 | tss;island | 0.013394 | 0.043782 | Not queried | 2.42 | 2.88e-02 | 6.16e-02 |
|  | 12 | 32112763 | 32113101 | PDFSTATSENSEMBLUCSC | 339 | 6 | 9093 | gene;island | 0.011966 | 0.001191 | Not queried | 2.42 | 4.85e-01 | 5.11e-01 |
|  | 8 | 67686767 | 67687060 | PDFSTATSENSEMBLUCSC | 294 | 5 | 39325 | gene;tss;island | 0.040988 | 0.010314 | Not queried | 2.42 | 4.21e-01 | 4.59e-01 |
|  | 2 | 583115 | 583384 | PDFSTATSENSEMBLUCSC | 270 | 5 | 24007 | island | 0.898456 | 0.972862 | Not queried | 2.42 | 9.52e-02 | 1.53e-01 |
|  | 3 | 51749782 | 51749959 | PDFSTATSENSEMBLUCSC | 178 | 4 | 27815 | gene;island | 0.668286 | 0.959601 | Not queried | 2.42 | 4.86e-01 | 5.11e-01 |
|  | 16 | 810643 | 811078 | PDFSTATSENSEMBLUCSC | 436 | 8 | 14982 | tss | 0.660655 | 0.895724 | Not queried | 2.42 | 1.48e-02 | 5.36e-02 |
|  | 8 | 56436393 | 56436771 | PDFSTATSENSEMBLUCSC | 379 | 5 | 39310 | gene | 0.707527 | 0.915988 | Not queried | 2.42 | 5.48e-01 | 5.70e-01 |
|  | 11 | 46957758 | 46958558 | PDFSTATSENSEMBLUCSC | 801 | 12 | 8257 | island | 0.062474 | 0.012675 | Not queried | 2.42 | 2.42e-02 | 6.12e-02 |
|  | 16 | 25228569 | 25228709 | PDFSTATSENSEMBLUCSC | 141 | 4 | 14699 | gene | 0.893435 | 0.978319 | Not queried | 2.42 | 2.86e-02 | 6.12e-02 |
|  | 1 | 179545073 | 179545458 | PDFSTATSENSEMBLUCSC | 386 | 9 | 3998 | island | 0.537881 | 0.873873 | Not queried | 2.42 | 9.39e-02 | 1.53e-01 |
|  | 2 | 26624450 | 26625047 | PDFSTATSENSEMBLUCSC | 598 | 11 | 24152 | island | 0.086053 | 0.315768 | Not queried | 2.42 | 2.33e-02 | 6.12e-02 |
|  | 4 | 55093612 | 55094011 | PDFSTATSENSEMBLUCSC | 400 | 6 | 31054 | island | 0.650024 | 0.972106 | Not queried | 2.42 | 6.49e-02 | 1.11e-01 |
|  | 2 | 80531500 | 80531898 | PDFSTATSENSEMBLUCSC | 399 | 9 | 23473 | tss | 0.805138 | 0.459509 | CTNNA2; LRRTM1 (margin: CTNNA2; LRRTM1) | 2.42 | 7.82e-04 | 8.17e-03 |
|  | 3 | 16555288 | 16555620 | PDFSTATSENSEMBLUCSC | 333 | 9 | 28177 | tss;island | 0.036910 | 0.130651 | Not queried | 2.42 | 6.25e-02 | 1.09e-01 |
|  | 1 | 17765068 | 17765394 | PDFSTATSENSEMBLUCSC | 327 | 4 | 1292 | tss | 0.002045 | 0.004738 | Not queried | 2.42 | 2.86e-02 | 6.12e-02 |
|  | 2 | 73871782 | 73871942 | PDFSTATSENSEMBLUCSC | 161 | 5 | 23442 | tss | 0.265212 | 0.603076 | Not queried | 2.41 | 2.22e-01 | 2.76e-01 |
|  | 8 | 27850215 | 27850423 | PDFSTATSENSEMBLUCSC | 209 | 6 | 39619 | tss | 0.764693 | 0.458623 | Not queried | 2.41 | 1.52e-02 | 5.36e-02 |
|  | 2 | 54342584 | 54342961 | PDFSTATSENSEMBLUCSC | 378 | 4 | 22682 | gene;island | 0.002986 | 0.009183 | Not queried | 2.41 | 2.00e-01 | 2.55e-01 |
|  | 10 | 45455245 | 45455500 | PDFSTATSENSEMBLUCSC | 256 | 4 | 4573 | gene | 0.340630 | 0.035349 | Not queried | 2.41 | 6.86e-01 | 6.99e-01 |
|  | 11 | 64059307 | 64059594 | PDFSTATSENSEMBLUCSC | 288 | 4 | 6678 | gene;island | 0.868817 | 0.469281 | Not queried | 2.41 | 2.86e-02 | 6.12e-02 |
|  | 11 | 45827260 | 45827763 | PDFSTATSENSEMBLUCSC | 504 | 7 | 6594 | gene;island | 0.527270 | 0.884936 | Not queried | 2.41 | 3.79e-02 | 7.66e-02 |
|  | 2 | 132152814 | 132152960 | PDFSTATSENSEMBLUCSC | 147 | 4 | 24731 | island | 0.732616 | 0.927327 | Not queried | 2.41 | 2.00e-01 | 2.55e-01 |
|  | 14 | 105126275 | 105126697 | PDFSTATSENSEMBLUCSC | 423 | 5 | 13212 | island | 0.358511 | 0.930279 | Not queried | 2.41 | 3.17e-02 | 6.50e-02 |
|  | 19 | 52391234 | 52391789 | PDFSTATSENSEMBLUCSC | 556 | 10 | 20999 | tss | 0.964228 | 0.793461 | CTC-429C10.2; ZNF577; ZNF649 (margin: CTC-429C10.2; ZNF577; ZNF649) | 2.41 | 5.20e-03 | 2.96e-02 |
|  | 6 | 160679391 | 160680213 | PDFSTATSENSEMBLUCSC | 823 | 12 | 36612 | island | 0.527601 | 0.912152 | SLC22A2 (margin: SLC22A2) | 2.41 | 1.00e-02 | 3.88e-02 |
|  | 15 | 59225861 | 59225903 | PDFSTATSENSEMBLUCSC | 43 | 4 | 13701 | tss | 0.010044 | 0.002732 | Not queried | 2.41 | 4.86e-01 | 5.11e-01 |
|  | 13 | 81229232 | 81229541 | PDFSTATSENSEMBLUCSC | 310 | 4 | 11804 | island | 0.444503 | 0.778334 | Not queried | 2.41 | 2.86e-02 | 6.12e-02 |
|  | 1 | 158147446 | 158147849 | PDFSTATSENSEMBLUCSC | 404 | 4 | 3878 | island | 0.376540 | 0.690618 | Not queried | 2.41 | 4.86e-01 | 5.11e-01 |
|  | 6 | 97285662 | 97285948 | PDFSTATSENSEMBLUCSC | 287 | 5 | 35128 | tss;island | 0.638912 | 0.065842 | Not queried | 2.41 | 2.22e-01 | 2.76e-01 |
|  | 19 | 1848742 | 1848907 | PDFSTATSENSEMBLUCSC | 166 | 4 | 20314 | tss;island | 0.003086 | 0.003426 | Not queried | 2.41 | 4.86e-01 | 5.11e-01 |
|  | 15 | 37110774 | 37110854 | PDFSTATSENSEMBLUCSC | 81 | 4 | 13568 | tss | 0.984305 | 0.917946 | Not queried | 2.41 | 1.14e-01 | 1.68e-01 |
|  | 11 | 113947036 | 113947359 | PDFSTATSENSEMBLUCSC | 324 | 4 | 6909 | gene | 0.864834 | 0.660567 | Not queried | 2.41 | 6.86e-01 | 6.99e-01 |
|  | 2 | 217236425 | 217237043 | PDFSTATSENSEMBLUCSC | 619 | 14 | 25072 | island | 0.246139 | 0.052272 | MARCH4 (margin: MARCH4) | 2.41 | 6.74e-03 | 3.19e-02 |
|  | 1 | 40137913 | 40138052 | PDFSTATSENSEMBLUCSC | 140 | 4 | 1525 | tss;island | 0.823856 | 0.722859 | Not queried | 2.41 | 6.86e-01 | 6.99e-01 |
|  | 11 | 75115376 | 75115584 | PDFSTATSENSEMBLUCSC | 209 | 4 | 6834 | gene | 0.653293 | 0.914670 | Not queried | 2.41 | 3.43e-01 | 3.84e-01 |
|  | 2 | 161127463 | 161127884 | PDFSTATSENSEMBLUCSC | 422 | 5 | 24815 | island | 0.871204 | 0.977775 | Not queried | 2.41 | 9.52e-02 | 1.53e-01 |
|  | 7 | 63505584 | 63505871 | PDFSTATSENSEMBLUCSC | 288 | 7 | 38522 | island | 0.073573 | 0.295061 | Not queried | 2.41 | 1.28e-01 | 1.87e-01 |
|  | 1 | 201476502 | 201476960 | PDFSTATSENSEMBLUCSC | 459 | 6 | 2168 | tss | 0.198922 | 0.520749 | Not queried | 2.41 | 3.10e-01 | 3.66e-01 |
|  | 13 | 113637695 | 113637903 | PDFSTATSENSEMBLUCSC | 209 | 4 | 11264 | gene;island | 0.275002 | 0.252169 | Not queried | 2.41 | 6.86e-01 | 6.99e-01 |
|  | 5 | 50678451 | 50678928 | PDFSTATSENSEMBLUCSC | 478 | 9 | 32057 | tss;island | 0.576094 | 0.526045 | Not queried | 2.41 | 7.96e-01 | 8.06e-01 |
|  | 5 | 110062384 | 110062473 | PDFSTATSENSEMBLUCSC | 90 | 4 | 32204 | tss | 0.011293 | 0.004637 | Not queried | 2.40 | 2.86e-02 | 6.12e-02 |
|  | 11 | 32457124 | 32457386 | PDFSTATSENSEMBLUCSC | 263 | 7 | 7226 | tss;island | 0.816882 | 0.962981 | WT1-AS; WT1 (margin: WT1-AS; WT1) | 2.40 | 1.11e-02 | 4.14e-02 |
|  | 6 | 49518347 | 49518627 | PDFSTATSENSEMBLUCSC | 281 | 5 | 34363 | gene;island | 0.052299 | 0.010456 | C6orf141 (margin: C6orf141) | 2.40 | 7.94e-03 | 3.19e-02 |
|  | 22 | 44287772 | 44288155 | PDFSTATSENSEMBLUCSC | 384 | 8 | 27560 | island | 0.583469 | 0.863510 | Not queried | 2.40 | 8.30e-02 | 1.40e-01 |
|  | 5 | 76934327 | 76934696 | PDFSTATSENSEMBLUCSC | 370 | 6 | 32902 | island | 0.822764 | 0.963071 | Not queried | 2.40 | 1.80e-01 | 2.45e-01 |
|  | 2 | 242808427 | 242808907 | PDFSTATSENSEMBLUCSC | 481 | 5 | 25312 | island | 0.483445 | 0.904697 | (margin: CXXC11; PDCD1) | 2.40 | 7.94e-03 | 3.19e-02 |
|  | 3 | 40518669 | 40518798 | PDFSTATSENSEMBLUCSC | 130 | 4 | 27728 | gene | 0.002185 | 0.008546 | Not queried | 2.40 | 2.00e-01 | 2.55e-01 |
|  | 5 | 72415687 | 72416469 | PDFSTATSENSEMBLUCSC | 783 | 10 | 32865 | island | 0.167905 | 0.428167 | Not queried | 2.40 | 2.80e-01 | 3.40e-01 |
|  | 19 | 51293367 | 51293654 | PDFSTATSENSEMBLUCSC | 288 | 5 | 20985 | tss | 0.784016 | 0.985401 | Not queried | 2.40 | 9.52e-02 | 1.53e-01 |
|  | 5 | 135188170 | 135188477 | PDFSTATSENSEMBLUCSC | 308 | 4 | 31759 | gene;island | 0.710565 | 0.916923 | Not queried | 2.40 | 3.43e-01 | 3.84e-01 |
|  | 6 | 126277704 | 126277793 | PDFSTATSENSEMBLUCSC | 90 | 4 | 35212 | tss | 0.004037 | 0.002261 | Not queried | 2.40 | 4.86e-01 | 5.11e-01 |
|  | 15 | 80543353 | 80543685 | PDFSTATSENSEMBLUCSC | 333 | 5 | 14374 | island | 0.821580 | 0.870443 | Not queried | 2.40 | 8.41e-01 | 8.48e-01 |
|  | 11 | 30344200 | 30344599 | PDFSTATSENSEMBLUCSC | 400 | 8 | 7219 | tss | 0.007447 | 0.012934 | Not queried | 2.40 | 2.79e-01 | 3.38e-01 |
|  | 3 | 13974286 | 13974800 | PDFSTATSENSEMBLUCSC | 515 | 4 | 28909 | island | 0.867787 | 0.969789 | Not queried | 2.40 | 5.71e-02 | 1.00e-01 |
|  | 8 | 143596671 | 143596947 | PDFSTATSENSEMBLUCSC | 277 | 4 | 39464 | gene | 0.550539 | 0.862127 | Not queried | 2.40 | 2.86e-02 | 6.12e-02 |
|  | 4 | 126236006 | 126236438 | PDFSTATSENSEMBLUCSC | 433 | 4 | 31295 | island | 0.036561 | 0.015073 | Not queried | 2.40 | 3.43e-01 | 3.84e-01 |
|  | 7 | 4210949 | 4211755 | PDFSTATSENSEMBLUCSC | 807 | 8 | 36831 | gene | 0.704586 | 0.919150 | SDK1 (margin: SDK1) | 2.40 | 1.86e-03 | 1.45e-02 |
|  | 6 | 33384179 | 33384537 | PDFSTATSENSEMBLUCSC | 359 | 7 | 34281 | gene;island | 0.542617 | 0.908577 | Not queried | 2.40 | 5.30e-02 | 1.00e-01 |
|  | 5 | 32585221 | 32585549 | PDFSTATSENSEMBLUCSC | 329 | 6 | 32007 | tss | 0.003296 | 0.007099 | Not queried | 2.40 | 5.89e-01 | 6.11e-01 |
|  | 10 | 131697669 | 131697980 | PDFSTATSENSEMBLUCSC | 312 | 4 | 4836 | gene;island | 0.727174 | 0.896625 | Not queried | 2.40 | 1.14e-01 | 1.68e-01 |
|  | 7 | 115850376 | 115850450 | PDFSTATSENSEMBLUCSC | 75 | 5 | 37848 | tss;island | 0.009799 | 0.050344 | TES (margin: TES) | 2.40 | 7.94e-03 | 3.19e-02 |
|  | 11 | 118230307 | 118230422 | PDFSTATSENSEMBLUCSC | 116 | 4 | 6928 | gene | 0.004804 | 0.006779 | Not queried | 2.40 | 4.86e-01 | 5.11e-01 |
|  | 1 | 228604037 | 228604566 | PDFSTATSENSEMBLUCSC | 530 | 5 | 1061 | gene | 0.839075 | 0.956829 | Not queried | 2.40 | 5.56e-02 | 1.00e-01 |
|  | 10 | 124402673 | 124403063 | PDFSTATSENSEMBLUCSC | 391 | 5 | 4798 | gene;island | 0.667475 | 0.903451 | Not queried | 2.40 | 1.51e-01 | 2.11e-01 |
|  | 11 | 69516939 | 69517471 | PDFSTATSENSEMBLUCSC | 533 | 4 | 6783 | gene;island | 0.779879 | 0.961925 | Not queried | 2.40 | 1.14e-01 | 1.68e-01 |
|  | 6 | 32027379 | 32027666 | PDFSTATSENSEMBLUCSC | 288 | 7 | 34024 | gene | 0.610213 | 0.855032 | Not queried | 2.39 | 7.28e-02 | 1.23e-01 |
|  | 8 | 41167660 | 41168481 | PDFSTATSENSEMBLUCSC | 822 | 12 | 39668 | tss;island | 0.618888 | 0.926521 | SFRP1 (margin: RNU6-895P; SFRP1) | 2.39 | 1.11e-03 | 1.12e-02 |
|  | 14 | 23771758 | 23772235 | PDFSTATSENSEMBLUCSC | 478 | 9 | 12714 | island | 0.036463 | 0.005852 | Not queried | 2.39 | 9.39e-02 | 1.53e-01 |
|  | 3 | 138739722 | 138740022 | PDFSTATSENSEMBLUCSC | 301 | 7 | 29533 | island | 0.748323 | 0.955366 | Not queried | 2.39 | 1.75e-02 | 5.79e-02 |
|  | 5 | 180542154 | 180542623 | PDFSTATSENSEMBLUCSC | 470 | 8 | 33515 | island | 0.083442 | 0.020585 | Not queried | 2.39 | 4.99e-02 | 9.81e-02 |
|  | 3 | 140813769 | 140814133 | PDFSTATSENSEMBLUCSC | 365 | 4 | 27971 | gene;island | 0.902820 | 0.977278 | Not queried | 2.39 | 2.00e-01 | 2.55e-01 |
|  | 2 | 241569557 | 241569846 | PDFSTATSENSEMBLUCSC | 290 | 4 | 23124 | gene;island | 0.538882 | 0.892860 | Not queried | 2.39 | 5.71e-02 | 1.00e-01 |
|  | 8 | 75227148 | 75227355 | PDFSTATSENSEMBLUCSC | 208 | 4 | 39338 | gene;island | 0.874557 | 0.983611 | Not queried | 2.39 | 2.00e-01 | 2.55e-01 |
|  | 17 | 7083015 | 7083519 | PDFSTATSENSEMBLUCSC | 505 | 5 | 17061 | tss | 0.493107 | 0.835651 | Not queried | 2.39 | 1.59e-02 | 5.36e-02 |
|  | 8 | 8085496 | 8085544 | PDFSTATSENSEMBLUCSC | 49 | 4 | 39540 | tss;island | 0.016475 | 0.003704 | Not queried | 2.39 | 3.43e-01 | 3.84e-01 |
|  | 10 | 106399877 | 106400824 | PDFSTATSENSEMBLUCSC | 948 | 10 | 5350 | tss | 0.887640 | 0.968255 | Not queried | 2.39 | 1.23e-01 | 1.81e-01 |
|  | 15 | 40763244 | 40763522 | PDFSTATSENSEMBLUCSC | 279 | 4 | 13294 | gene | 0.009139 | 0.038723 | Not queried | 2.39 | 2.86e-02 | 6.12e-02 |
|  | 12 | 53613892 | 53614284 | PDFSTATSENSEMBLUCSC | 393 | 7 | 9148 | gene;island | 0.796755 | 0.960686 | RARG (margin: RARG) | 2.39 | 6.99e-03 | 3.19e-02 |
|  | 6 | 74063982 | 74064594 | PDFSTATSENSEMBLUCSC | 613 | 8 | 35066 | tss;island | 0.752954 | 0.912211 | Not queried | 2.39 | 2.79e-01 | 3.38e-01 |
|  | 12 | 30907191 | 30907599 | PDFSTATSENSEMBLUCSC | 409 | 6 | 10307 | island | 0.006646 | 0.070960 | Not queried | 2.39 | 4.85e-01 | 5.11e-01 |
|  | 19 | 10381355 | 10381498 | PDFSTATSENSEMBLUCSC | 144 | 6 | 20442 | tss | 0.582839 | 0.279304 | Not queried | 2.39 | 3.10e-01 | 3.66e-01 |
|  | 1 | 153030514 | 153030751 | PDFSTATSENSEMBLUCSC | 238 | 4 | 1939 | tss | 0.823633 | 0.508661 | Not queried | 2.39 | 6.86e-01 | 6.99e-01 |
|  | 14 | 101520369 | 101520662 | PDFSTATSENSEMBLUCSC | 294 | 4 | 12628 | tss | 0.900286 | 0.960811 | Not queried | 2.38 | 2.00e-01 | 2.55e-01 |
|  | 7 | 158362729 | 158362966 | PDFSTATSENSEMBLUCSC | 238 | 4 | 37378 | gene;island | 0.863371 | 0.970523 | Not queried | 2.38 | 5.71e-02 | 1.00e-01 |
|  | 4 | 146100881 | 146101151 | PDFSTATSENSEMBLUCSC | 271 | 4 | 30618 | tss | 0.000990 | 0.004212 | Not queried | 2.38 | 3.43e-01 | 3.84e-01 |
|  | 8 | 109799518 | 109799909 | PDFSTATSENSEMBLUCSC | 392 | 5 | 40534 | island | 0.567192 | 0.129559 | TMEM74 (margin: TMEM74) | 2.38 | 7.94e-03 | 3.19e-02 |
|  | 16 | 34407715 | 34408014 | PDFSTATSENSEMBLUCSC | 300 | 5 | 15892 | island | 0.644105 | 0.852070 | Not queried | 2.38 | 3.17e-02 | 6.50e-02 |
|  | 5 | 95768915 | 95769079 | PDFSTATSENSEMBLUCSC | 165 | 7 | 32974 | island | 0.965725 | 0.811087 | Not queried | 2.38 | 7.28e-02 | 1.23e-01 |
|  | 1 | 223566127 | 223566794 | PDFSTATSENSEMBLUCSC | 668 | 9 | 4237 | island | 0.667493 | 0.201927 | Not queried | 2.38 | 1.61e-01 | 2.25e-01 |
|  | 7 | 111202666 | 111202755 | PDFSTATSENSEMBLUCSC | 90 | 4 | 37839 | tss;island | 0.006178 | 0.013515 | Not queried | 2.38 | 4.86e-01 | 5.11e-01 |
|  | 17 | 80275401 | 80276134 | PDFSTATSENSEMBLUCSC | 734 | 8 | 18983 | island | 0.682340 | 0.894009 | Not queried | 2.38 | 3.79e-02 | 7.66e-02 |
|  | 6 | 26021768 | 26022273 | PDFSTATSENSEMBLUCSC | 506 | 9 | 35506 | island | 0.001736 | 0.003865 | Not queried | 2.38 | 1.00e+00 | 1.00e+00 |
|  | 2 | 2193511 | 2193665 | PDFSTATSENSEMBLUCSC | 155 | 4 | 22542 | gene;island | 0.495915 | 0.806146 | Not queried | 2.38 | 2.00e-01 | 2.55e-01 |
|  | 2 | 232651086 | 232651151 | PDFSTATSENSEMBLUCSC | 66 | 6 | 23945 | tss | 0.000990 | 0.005601 | Not queried | 2.38 | 1.80e-01 | 2.45e-01 |
|  | 19 | 56915403 | 56915658 | PDFSTATSENSEMBLUCSC | 256 | 6 | 21097 | tss | 0.850588 | 0.507661 | Not queried | 2.38 | 4.11e-02 | 8.14e-02 |
|  | 5 | 110408740 | 110408997 | PDFSTATSENSEMBLUCSC | 258 | 7 | 32206 | tss | 0.126511 | 0.026740 | Not queried | 2.38 | 5.30e-02 | 1.00e-01 |
|  | 7 | 1809087 | 1809455 | PDFSTATSENSEMBLUCSC | 369 | 6 | 38125 | island | 0.925084 | 0.981250 | Not queried | 2.38 | 4.11e-02 | 8.14e-02 |
|  | 1 | 113051685 | 113052167 | PDFSTATSENSEMBLUCSC | 483 | 6 | 691 | gene;island | 0.615342 | 0.874311 | WNT2B (margin: WNT2B) | 2.38 | 8.66e-03 | 3.36e-02 |
|  | 3 | 35785735 | 35786030 | PDFSTATSENSEMBLUCSC | 296 | 5 | 27713 | gene | 0.048490 | 0.269319 | Not queried | 2.38 | 1.51e-01 | 2.11e-01 |
|  | 16 | 77822083 | 77822500 | PDFSTATSENSEMBLUCSC | 418 | 9 | 16111 | island | 0.416252 | 0.157042 | Not queried | 2.38 | 2.44e-02 | 6.12e-02 |
|  | 14 | 74892986 | 74893183 | PDFSTATSENSEMBLUCSC | 198 | 5 | 12506 | tss;island | 0.965885 | 0.190936 | Not queried | 2.38 | 1.51e-01 | 2.11e-01 |
|  | 3 | 11034279 | 11034501 | PDFSTATSENSEMBLUCSC | 223 | 4 | 28880 | island | 0.806059 | 0.953053 | Not queried | 2.38 | 2.86e-02 | 6.12e-02 |
|  | 14 | 101498206 | 101498317 | PDFSTATSENSEMBLUCSC | 112 | 5 | 12623 | tss | 0.604000 | 0.965795 | Not queried | 2.38 | 3.10e-01 | 3.66e-01 |
|  | 1 | 146649635 | 146649851 | PDFSTATSENSEMBLUCSC | 217 | 4 | 729 | gene;island | 0.911094 | 0.690461 | Not queried | 2.38 | 2.86e-02 | 6.12e-02 |
|  | 19 | 52599190 | 52599329 | PDFSTATSENSEMBLUCSC | 140 | 5 | 21006 | tss | 0.014947 | 0.004160 | Not queried | 2.37 | 4.21e-01 | 4.59e-01 |
|  | 16 | 27414272 | 27414536 | PDFSTATSENSEMBLUCSC | 265 | 4 | 14702 | gene | 0.693813 | 0.934085 | Not queried | 2.37 | 1.14e-01 | 1.68e-01 |
|  | 1 | 32281665 | 32281812 | PDFSTATSENSEMBLUCSC | 148 | 4 | 1451 | tss | 0.280338 | 0.083884 | Not queried | 2.37 | 5.71e-02 | 1.00e-01 |
|  | 12 | 110271439 | 110271625 | PDFSTATSENSEMBLUCSC | 187 | 4 | 9996 | tss;island | 0.869427 | 0.501297 | Not queried | 2.37 | 5.71e-02 | 1.00e-01 |
|  | 1 | 241587493 | 241587783 | PDFSTATSENSEMBLUCSC | 291 | 4 | 4377 | island | 0.685005 | 0.944791 | Not queried | 2.37 | 2.86e-02 | 6.12e-02 |
|  | 12 | 16506202 | 16506341 | PDFSTATSENSEMBLUCSC | 140 | 4 | 9070 | gene;tss | 0.433631 | 0.812780 | Not queried | 2.37 | 2.86e-02 | 6.12e-02 |
|  | 11 | 61584319 | 61584773 | PDFSTATSENSEMBLUCSC | 455 | 8 | 8335 | island | 0.507546 | 0.163056 | FADS2; FADS1; MIR1908 (margin: FADS2; FADS1; MIR1908) | 2.37 | 1.86e-03 | 1.45e-02 |
|  | 11 | 61584616 | 61584773 | PDFSTATSENSEMBLUCSC | 158 | 6 | 7341 | tss | 0.520046 | 0.163056 | FADS2; FADS1; MIR1908 (margin: FADS2; FADS1; MIR1908) | 2.37 | 8.66e-03 | 3.36e-02 |
|  | 12 | 96184787 | 96184965 | PDFSTATSENSEMBLUCSC | 179 | 4 | 9926 | tss;island | 0.150958 | 0.506159 | Not queried | 2.37 | 2.86e-02 | 6.12e-02 |
|  | 2 | 129659633 | 129659946 | PDFSTATSENSEMBLUCSC | 314 | 4 | 24711 | island | 0.548219 | 0.850246 | Not queried | 2.37 | 2.00e-01 | 2.55e-01 |
|  | 1 | 4832352 | 4832593 | PDFSTATSENSEMBLUCSC | 242 | 5 | 153 | gene;island | 0.607104 | 0.846877 | Not queried | 2.37 | 2.22e-01 | 2.76e-01 |
|  | 19 | 20012036 | 20012489 | PDFSTATSENSEMBLUCSC | 454 | 4 | 19957 | gene;island | 0.832734 | 0.498200 | Not queried | 2.37 | 2.86e-02 | 6.12e-02 |
|  | 16 | 1542910 | 1543288 | PDFSTATSENSEMBLUCSC | 379 | 6 | 14996 | tss;island | 0.099289 | 0.014085 | Not queried | 2.37 | 4.85e-01 | 5.11e-01 |
|  | 3 | 9745369 | 9745610 | PDFSTATSENSEMBLUCSC | 242 | 4 | 28859 | island | 0.692742 | 0.947943 | Not queried | 2.37 | 1.14e-01 | 1.68e-01 |
|  | 9 | 4792285 | 4792649 | PDFSTATSENSEMBLUCSC | 365 | 4 | 40802 | tss;island | 0.062542 | 0.009451 | Not queried | 2.37 | 4.86e-01 | 5.11e-01 |
|  | 21 | 46407926 | 46408142 | PDFSTATSENSEMBLUCSC | 217 | 4 | 26746 | island | 0.670150 | 0.914695 | Not queried | 2.37 | 5.71e-02 | 1.00e-01 |
|  | 16 | 22926347 | 22926625 | PDFSTATSENSEMBLUCSC | 279 | 4 | 14691 | gene;island | 0.392117 | 0.766704 | Not queried | 2.37 | 2.00e-01 | 2.55e-01 |
|  | 3 | 44596178 | 44596684 | PDFSTATSENSEMBLUCSC | 507 | 8 | 28256 | tss | 0.883628 | 0.980205 | Not queried | 2.37 | 4.99e-02 | 9.81e-02 |
|  | 8 | 118532625 | 118532951 | PDFSTATSENSEMBLUCSC | 327 | 8 | 39883 | tss | 0.021373 | 0.004570 | Not queried | 2.37 | 4.99e-02 | 9.81e-02 |
|  | 19 | 1028379 | 1028780 | PDFSTATSENSEMBLUCSC | 402 | 4 | 19640 | gene;island | 0.031689 | 0.133569 | Not queried | 2.37 | 2.86e-02 | 6.12e-02 |
|  | 17 | 38501397 | 38501773 | PDFSTATSENSEMBLUCSC | 377 | 4 | 16564 | gene;island | 0.844775 | 0.968901 | Not queried | 2.37 | 2.86e-02 | 6.12e-02 |
|  | 12 | 109162390 | 109162748 | PDFSTATSENSEMBLUCSC | 359 | 6 | 10773 | island | 0.917289 | 0.759432 | Not queried | 2.36 | 1.32e-01 | 1.89e-01 |
|  | 17 | 42462958 | 42463277 | PDFSTATSENSEMBLUCSC | 320 | 4 | 16618 | gene;island | 0.297955 | 0.022214 | Not queried | 2.36 | 4.86e-01 | 5.11e-01 |
|  | 19 | 52873173 | 52873624 | PDFSTATSENSEMBLUCSC | 452 | 4 | 20194 | gene | 0.585081 | 0.878271 | Not queried | 2.36 | 4.86e-01 | 5.11e-01 |
|  | 15 | 26108612 | 26108948 | PDFSTATSENSEMBLUCSC | 337 | 12 | 13539 | tss;island | 0.751221 | 0.956845 | RP11-2C7.1; ATP10A (margin: RP11-2C7.1; ATP10A) | 2.36 | 4.51e-03 | 2.62e-02 |
|  | 3 | 189838996 | 189839473 | PDFSTATSENSEMBLUCSC | 478 | 7 | 28081 | gene;tss;island | 0.966171 | 0.893625 | Not queried | 2.36 | 3.83e-01 | 4.27e-01 |
|  | 21 | 39047670 | 39047966 | PDFSTATSENSEMBLUCSC | 297 | 5 | 26424 | gene;island | 0.091675 | 0.023619 | Not queried | 2.36 | 5.56e-02 | 1.00e-01 |
|  | 3 | 42846907 | 42847116 | PDFSTATSENSEMBLUCSC | 210 | 4 | 28249 | tss;island | 0.982134 | 0.838730 | Not queried | 2.36 | 1.14e-01 | 1.68e-01 |
|  | 4 | 80994236 | 80994605 | PDFSTATSENSEMBLUCSC | 370 | 6 | 31129 | island | 0.005399 | 0.051204 | Not queried | 2.36 | 2.60e-02 | 6.12e-02 |
|  | 1 | 38412519 | 38412711 | PDFSTATSENSEMBLUCSC | 193 | 4 | 439 | gene | 0.826338 | 0.345797 | Not queried | 2.36 | 2.86e-02 | 6.12e-02 |
|  | 6 | 32054441 | 32055738 | PDFSTATSENSEMBLUCSC | 1298 | 29 | 34040 | gene;island | 0.539209 | 0.815643 | Not queried | 2.36 | 1.73e-02 | 5.79e-02 |
|  | 11 | 76858648 | 76859056 | PDFSTATSENSEMBLUCSC | 409 | 6 | 6839 | gene;island | 0.559804 | 0.942231 | Not queried | 2.36 | 2.60e-02 | 6.12e-02 |
|  | 19 | 375746 | 376152 | PDFSTATSENSEMBLUCSC | 407 | 6 | 21158 | island | 0.693673 | 0.953763 | Not queried | 2.36 | 9.31e-02 | 1.53e-01 |
|  | 19 | 375746 | 375995 | PDFSTATSENSEMBLUCSC | 250 | 4 | 19624 | gene | 0.693673 | 0.937336 | Not queried | 2.36 | 1.14e-01 | 1.68e-01 |
|  | 13 | 77461056 | 77461426 | PDFSTATSENSEMBLUCSC | 371 | 7 | 11482 | tss;island | 0.970667 | 0.861453 | Not queried | 2.36 | 5.30e-02 | 1.00e-01 |
|  | 3 | 183993341 | 183993726 | PDFSTATSENSEMBLUCSC | 386 | 8 | 28757 | tss | 0.833746 | 0.977044 | Not queried | 2.36 | 8.30e-02 | 1.40e-01 |
|  | 19 | 3358827 | 3359204 | PDFSTATSENSEMBLUCSC | 378 | 4 | 20342 | tss;island | 0.423487 | 0.094219 | Not queried | 2.36 | 4.86e-01 | 5.11e-01 |
|  | 19 | 11450511 | 11450739 | PDFSTATSENSEMBLUCSC | 229 | 5 | 20467 | tss;island | 0.416151 | 0.069559 | Not queried | 2.36 | 8.41e-01 | 8.48e-01 |
|  | 16 | 56666323 | 56666640 | PDFSTATSENSEMBLUCSC | 318 | 5 | 15950 | island | 0.883516 | 0.987750 | Not queried | 2.36 | 1.59e-02 | 5.36e-02 |
|  | 19 | 52900882 | 52901431 | PDFSTATSENSEMBLUCSC | 550 | 12 | 22357 | island | 0.679487 | 0.876330 | Not queried | 2.36 | 2.42e-02 | 6.12e-02 |
|  | 22 | 39709951 | 39710068 | PDFSTATSENSEMBLUCSC | 118 | 4 | 27151 | tss | 0.745248 | 0.938787 | Not queried | 2.36 | 2.86e-02 | 6.12e-02 |
|  | 10 | 17271929 | 17272219 | PDFSTATSENSEMBLUCSC | 291 | 5 | 4519 | gene;island | 0.137148 | 0.015925 | Not queried | 2.36 | 1.51e-01 | 2.11e-01 |
|  | 7 | 1022841 | 1023156 | PDFSTATSENSEMBLUCSC | 316 | 5 | 36730 | gene | 0.629349 | 0.853517 | Not queried | 2.36 | 5.56e-02 | 1.00e-01 |
|  | 3 | 4508494 | 4508875 | PDFSTATSENSEMBLUCSC | 382 | 4 | 27642 | gene | 0.000389 | 0.002192 | Not queried | 2.36 | 4.86e-01 | 5.11e-01 |
|  | 7 | 157697057 | 157697628 | PDFSTATSENSEMBLUCSC | 572 | 5 | 37353 | gene;island | 0.324887 | 0.722060 | Not queried | 2.36 | 9.52e-02 | 1.53e-01 |
|  | 12 | 7261691 | 7262137 | PDFSTATSENSEMBLUCSC | 447 | 11 | 9045 | gene | 0.005833 | 0.073448 | Not queried | 2.36 | 1.58e-02 | 5.36e-02 |
|  | 17 | 72357992 | 72358370 | PDFSTATSENSEMBLUCSC | 379 | 5 | 17624 | tss;island | 0.314482 | 0.681140 | BTBD17 (margin: KIF19; GPR142; AC103809.2; BTBD17) | 2.35 | 7.94e-03 | 3.19e-02 |
|  | 6 | 32025416 | 32025657 | PDFSTATSENSEMBLUCSC | 242 | 4 | 34021 | gene | 0.591582 | 0.807080 | Not queried | 2.35 | 1.14e-01 | 1.68e-01 |
|  | 12 | 132939657 | 132939992 | PDFSTATSENSEMBLUCSC | 336 | 4 | 11047 | island | 0.668783 | 0.913242 | Not queried | 2.35 | 2.00e-01 | 2.55e-01 |
|  | 6 | 32551749 | 32552670 | PDFSTATSENSEMBLUCSC | 922 | 17 | 34122 | gene;island | 0.844318 | 0.411100 | HLA-DRB1 (margin: HLA-DRB1) | 2.35 | 8.51e-03 | 3.36e-02 |
|  | 2 | 173600969 | 173601150 | PDFSTATSENSEMBLUCSC | 182 | 5 | 22927 | gene;tss;island | 0.412792 | 0.556544 | Not queried | 2.35 | 1.51e-01 | 2.11e-01 |
|  | 18 | 5238137 | 5238310 | PDFSTATSENSEMBLUCSC | 174 | 5 | 19048 | gene;tss;island | 0.501018 | 0.825076 | Not queried | 2.35 | 2.22e-01 | 2.76e-01 |
|  | 16 | 55866757 | 55867072 | PDFSTATSENSEMBLUCSC | 316 | 5 | 14754 | gene;island | 0.737341 | 0.816734 | Not queried | 2.35 | 3.10e-01 | 3.66e-01 |
|  | 1 | 2084246 | 2084595 | PDFSTATSENSEMBLUCSC | 350 | 5 | 65 | gene;island | 0.891892 | 0.986600 | Not queried | 2.35 | 1.51e-01 | 2.11e-01 |
|  | 6 | 100050480 | 100051116 | PDFSTATSENSEMBLUCSC | 637 | 8 | 36356 | island | 0.888308 | 0.965139 | (margin: PRDM13) | 2.35 | 6.99e-03 | 3.19e-02 |
|  | 17 | 28902652 | 28903005 | PDFSTATSENSEMBLUCSC | 354 | 4 | 17242 | tss;island | 0.511150 | 0.851452 | Not queried | 2.35 | 2.00e-01 | 2.55e-01 |
|  | 5 | 137610127 | 137610645 | PDFSTATSENSEMBLUCSC | 519 | 9 | 33154 | island | 0.894722 | 0.656925 | Not queried | 2.35 | 2.44e-02 | 6.12e-02 |
|  | 15 | 25307169 | 25307422 | PDFSTATSENSEMBLUCSC | 254 | 4 | 13522 | tss | 0.523229 | 0.224010 | Not queried | 2.35 | 1.14e-01 | 1.68e-01 |
|  | 22 | 35937058 | 35937319 | PDFSTATSENSEMBLUCSC | 262 | 6 | 27100 | tss | 0.392418 | 0.108019 | Not queried | 2.35 | 2.60e-02 | 6.12e-02 |
|  | 19 | 49939549 | 49940062 | PDFSTATSENSEMBLUCSC | 514 | 6 | 20150 | gene;island | 0.813368 | 0.959638 | Not queried | 2.35 | 6.49e-02 | 1.11e-01 |
|  | 10 | 72141375 | 72141625 | PDFSTATSENSEMBLUCSC | 251 | 4 | 4618 | gene;island | 0.968237 | 0.825758 | Not queried | 2.35 | 2.86e-02 | 6.12e-02 |
|  | 1 | 38099874 | 38100467 | PDFSTATSENSEMBLUCSC | 594 | 6 | 435 | gene;island | 0.388534 | 0.183707 | Not queried | 2.35 | 3.94e-01 | 4.36e-01 |
|  | 8 | 67090581 | 67090928 | PDFSTATSENSEMBLUCSC | 348 | 6 | 40352 | island | 0.778118 | 0.978451 | Not queried | 2.35 | 4.11e-02 | 8.14e-02 |
|  | 3 | 137728692 | 137728997 | PDFSTATSENSEMBLUCSC | 306 | 8 | 28617 | tss | 0.772346 | 0.974377 | Not queried | 2.35 | 3.79e-02 | 7.66e-02 |
|  | 10 | 106399877 | 106400880 | PDFSTATSENSEMBLUCSC | 1004 | 11 | 6119 | island | 0.898621 | 0.971220 | Not queried | 2.35 | 1.33e-01 | 1.91e-01 |
|  | 12 | 53472792 | 53473136 | PDFSTATSENSEMBLUCSC | 345 | 4 | 9145 | gene | 0.017657 | 0.067477 | Not queried | 2.35 | 3.43e-01 | 3.84e-01 |
|  | 2 | 26624450 | 26624767 | PDFSTATSENSEMBLUCSC | 318 | 7 | 23243 | tss | 0.112885 | 0.315768 | Not queried | 2.35 | 2.09e-01 | 2.65e-01 |
|  | 1 | 26735846 | 26736154 | PDFSTATSENSEMBLUCSC | 309 | 7 | 1391 | tss;island | 0.529261 | 0.850148 | Not queried | 2.35 | 3.79e-02 | 7.66e-02 |
|  | 3 | 122514443 | 122514814 | PDFSTATSENSEMBLUCSC | 372 | 4 | 27917 | gene;island | 0.333099 | 0.701121 | Not queried | 2.34 | 1.14e-01 | 1.68e-01 |
|  | 7 | 107300939 | 107301048 | PDFSTATSENSEMBLUCSC | 110 | 5 | 37829 | tss | 0.643495 | 0.898955 | Not queried | 2.34 | 3.10e-01 | 3.66e-01 |
|  | 7 | 1067951 | 1068244 | PDFSTATSENSEMBLUCSC | 294 | 5 | 36737 | gene;island | 0.005075 | 0.011899 | Not queried | 2.34 | 3.17e-02 | 6.50e-02 |
|  | 11 | 57364841 | 57365088 | PDFSTATSENSEMBLUCSC | 248 | 6 | 7304 | tss | 0.852588 | 0.983383 | Not queried | 2.34 | 6.49e-02 | 1.11e-01 |
|  | 17 | 48911983 | 48912556 | PDFSTATSENSEMBLUCSC | 574 | 12 | 17508 | tss | 0.843804 | 0.954189 | WFIKKN2 (margin: WFIKKN2; RP11-506D12.5) | 2.34 | 1.03e-04 | 2.47e-03 |
|  | 17 | 72269945 | 72270201 | PDFSTATSENSEMBLUCSC | 257 | 5 | 17622 | tss;island | 0.699374 | 0.973258 | Not queried | 2.34 | 3.17e-02 | 6.50e-02 |
|  | 17 | 78010264 | 78010476 | PDFSTATSENSEMBLUCSC | 213 | 7 | 17718 | tss;island | 0.016967 | 0.006662 | Not queried | 2.34 | 3.18e-01 | 3.74e-01 |
|  | 10 | 135061171 | 135061360 | PDFSTATSENSEMBLUCSC | 190 | 6 | 5457 | tss | 0.706822 | 0.930049 | Not queried | 2.34 | 1.32e-01 | 1.89e-01 |
|  | 19 | 54290737 | 54291194 | PDFSTATSENSEMBLUCSC | 458 | 11 | 21039 | tss | 0.936821 | 0.974567 | Not queried | 2.34 | 7.59e-02 | 1.28e-01 |
|  | 13 | 114067889 | 114068181 | PDFSTATSENSEMBLUCSC | 293 | 4 | 11964 | island | 0.629216 | 0.899290 | Not queried | 2.34 | 2.00e-01 | 2.55e-01 |
|  | 19 | 29704201 | 29704446 | PDFSTATSENSEMBLUCSC | 246 | 8 | 20669 | tss | 0.035385 | 0.006666 | Not queried | 2.34 | 3.28e-01 | 3.84e-01 |
|  | 1 | 6284844 | 6285107 | PDFSTATSENSEMBLUCSC | 264 | 4 | 164 | gene | 0.763690 | 0.977539 | Not queried | 2.34 | 4.86e-01 | 5.11e-01 |
|  | 17 | 72208213 | 72208504 | PDFSTATSENSEMBLUCSC | 292 | 4 | 16748 | gene;tss;island | 0.569203 | 0.941399 | Not queried | 2.34 | 2.00e-01 | 2.55e-01 |
|  | 2 | 102091195 | 102091321 | PDFSTATSENSEMBLUCSC | 127 | 6 | 23548 | tss | 0.767366 | 0.937072 | RFX8 (margin: AC092570.2; RFX8) | 2.34 | 2.16e-03 | 1.45e-02 |
|  | 10 | 135160697 | 135160792 | PDFSTATSENSEMBLUCSC | 96 | 5 | 5462 | tss | 0.315362 | 0.903350 | Not queried | 2.34 | 3.17e-02 | 6.50e-02 |
|  | 16 | 216450 | 216757 | PDFSTATSENSEMBLUCSC | 308 | 4 | 14523 | gene;island | 0.623945 | 0.918988 | Not queried | 2.34 | 2.00e-01 | 2.55e-01 |
|  | 5 | 63257554 | 63257941 | PDFSTATSENSEMBLUCSC | 388 | 9 | 32087 | tss | 0.796042 | 0.460244 | Not queried | 2.34 | 5.03e-02 | 9.87e-02 |
|  | 2 | 211090117 | 211090487 | PDFSTATSENSEMBLUCSC | 371 | 8 | 25059 | island | 0.905094 | 0.629453 | Not queried | 2.34 | 1.30e-01 | 1.89e-01 |
|  | 2 | 211090247 | 211090487 | PDFSTATSENSEMBLUCSC | 241 | 6 | 23855 | tss | 0.905094 | 0.724860 | Not queried | 2.34 | 1.80e-01 | 2.45e-01 |
|  | 4 | 156680007 | 156680518 | PDFSTATSENSEMBLUCSC | 512 | 7 | 31392 | island | 0.837231 | 0.448121 | GUCY1B3 (margin: GUCY1B3) | 2.34 | 5.83e-04 | 6.47e-03 |
|  | 17 | 45810359 | 45810615 | PDFSTATSENSEMBLUCSC | 257 | 9 | 18459 | island | 0.860557 | 0.947988 | Not queried | 2.34 | 1.42e-02 | 5.24e-02 |
|  | 16 | 77822083 | 77822441 | PDFSTATSENSEMBLUCSC | 359 | 8 | 15371 | tss | 0.390815 | 0.163744 | Not queried | 2.34 | 6.50e-02 | 1.11e-01 |
|  | 17 | 50237205 | 50237844 | PDFSTATSENSEMBLUCSC | 640 | 9 | 17515 | tss;island | 0.540805 | 0.315583 | Not queried | 2.34 | 1.90e-01 | 2.55e-01 |
|  | 13 | 39611764 | 39612086 | PDFSTATSENSEMBLUCSC | 323 | 4 | 11141 | gene | 0.000929 | 0.006774 | Not queried | 2.34 | 2.86e-02 | 6.12e-02 |
|  | 21 | 14410329 | 14410566 | PDFSTATSENSEMBLUCSC | 238 | 4 | 26604 | island | 0.369764 | 0.785795 | Not queried | 2.34 | 2.86e-02 | 6.12e-02 |
|  | 14 | 65171673 | 65172195 | PDFSTATSENSEMBLUCSC | 523 | 5 | 12112 | gene;island | 0.005996 | 0.030843 | Not queried | 2.33 | 9.52e-02 | 1.53e-01 |
|  | 19 | 4785298 | 4785605 | PDFSTATSENSEMBLUCSC | 308 | 4 | 21388 | island | 0.698525 | 0.945356 | Not queried | 2.33 | 1.14e-01 | 1.68e-01 |
|  | 6 | 32805142 | 32806489 | PDFSTATSENSEMBLUCSC | 1348 | 25 | 34146 | gene | 0.011911 | 0.065753 | TAP2 (margin: XXbac-BPG246D15.8; PSMB9; TAP2; PSMB8; TAP1) | 2.33 | 6.49e-04 | 6.98e-03 |
|  | 14 | 70655803 | 70656116 | PDFSTATSENSEMBLUCSC | 314 | 8 | 12485 | tss | 0.568964 | 0.207406 | RP11-486O13.2; SLC8A3 (margin: RP11-486O13.2; SLC8A3) | 2.33 | 6.99e-03 | 3.19e-02 |
|  | 10 | 132912784 | 132913255 | PDFSTATSENSEMBLUCSC | 472 | 5 | 4842 | gene | 0.323202 | 0.453905 | Not queried | 2.33 | 3.10e-01 | 3.66e-01 |
|  | 10 | 43866966 | 43867071 | PDFSTATSENSEMBLUCSC | 106 | 5 | 5046 | tss | 0.461032 | 0.814038 | FXYD4 (margin: FXYD4) | 2.33 | 7.94e-03 | 3.19e-02 |
|  | 11 | 130786230 | 130786720 | PDFSTATSENSEMBLUCSC | 491 | 8 | 8964 | island | 0.011778 | 0.078259 | Not queried | 2.33 | 2.34e-01 | 2.90e-01 |
|  | 10 | 100993553 | 100994143 | PDFSTATSENSEMBLUCSC | 591 | 11 | 4706 | gene;island | 0.868307 | 0.959582 | HPSE2 (margin: HPSE2) | 2.33 | 1.28e-02 | 4.76e-02 |
|  | 10 | 134188633 | 134188873 | PDFSTATSENSEMBLUCSC | 241 | 5 | 4863 | gene | 0.948087 | 0.702287 | Not queried | 2.33 | 8.41e-01 | 8.48e-01 |
|  | 14 | 85996101 | 85996393 | PDFSTATSENSEMBLUCSC | 293 | 8 | 12542 | tss | 0.175518 | 0.487793 | RP11-497E19.2; FLRT2; RP11-497E19.1 (margin: RP11-497E19.2; FLRT2; RP11-497E19.1) | 2.33 | 2.95e-03 | 1.88e-02 |
|  | 11 | 94276895 | 94277190 | PDFSTATSENSEMBLUCSC | 296 | 6 | 8740 | island | 0.230827 | 0.560404 | Not queried | 2.33 | 3.94e-01 | 4.36e-01 |
|  | 19 | 38210837 | 38210932 | PDFSTATSENSEMBLUCSC | 96 | 4 | 20744 | tss | 0.051593 | 0.006234 | Not queried | 2.33 | 1.14e-01 | 1.68e-01 |
|  | 12 | 6603150 | 6603428 | PDFSTATSENSEMBLUCSC | 279 | 5 | 9514 | tss;island | 0.003168 | 0.012563 | Not queried | 2.33 | 4.21e-01 | 4.59e-01 |
|  | 19 | 12951996 | 12952280 | PDFSTATSENSEMBLUCSC | 285 | 4 | 19869 | gene;island | 0.590372 | 0.887647 | Not queried | 2.33 | 3.43e-01 | 3.84e-01 |
|  | 18 | 10525737 | 10525850 | PDFSTATSENSEMBLUCSC | 114 | 5 | 19180 | tss | 0.002968 | 0.009295 | Not queried | 2.33 | 5.48e-01 | 5.70e-01 |
|  | 3 | 132756721 | 132757106 | PDFSTATSENSEMBLUCSC | 386 | 9 | 28599 | tss | 0.927082 | 0.980595 | TMEM108; RP11-469L4.1 (margin: TMEM108; RP11-469L4.1) | 2.33 | 1.06e-02 | 4.01e-02 |
|  | 17 | 29297380 | 29297873 | PDFSTATSENSEMBLUCSC | 494 | 14 | 17245 | tss;island | 0.019012 | 0.003139 | Not queried | 2.33 | 1.86e-02 | 6.12e-02 |
|  | 7 | 140097579 | 140098259 | PDFSTATSENSEMBLUCSC | 681 | 6 | 37246 | gene | 0.004886 | 0.051649 | Not queried | 2.33 | 6.49e-02 | 1.11e-01 |
|  | 10 | 49482723 | 49482871 | PDFSTATSENSEMBLUCSC | 149 | 4 | 5066 | tss | 0.692445 | 0.944847 | Not queried | 2.33 | 2.00e-01 | 2.55e-01 |
|  | 5 | 41870746 | 41871189 | PDFSTATSENSEMBLUCSC | 444 | 10 | 32755 | island | 0.275205 | 0.552981 | Not queried | 2.33 | 1.05e-01 | 1.66e-01 |
|  | 3 | 69133862 | 69134087 | PDFSTATSENSEMBLUCSC | 226 | 4 | 28440 | tss | 0.000174 | 0.002255 | Not queried | 2.33 | 2.00e-01 | 2.55e-01 |
|  | 22 | 24373134 | 24373618 | PDFSTATSENSEMBLUCSC | 485 | 4 | 26811 | gene | 0.080469 | 0.020279 | Not queried | 2.33 | 3.43e-01 | 3.84e-01 |
|  | 15 | 49255364 | 49255563 | PDFSTATSENSEMBLUCSC | 200 | 4 | 13339 | gene | 0.304660 | 0.073907 | Not queried | 2.33 | 2.00e-01 | 2.55e-01 |
|  | 6 | 33288180 | 33289280 | PDFSTATSENSEMBLUCSC | 1101 | 36 | 34268 | gene;island | 0.786933 | 0.901894 | DAXX (margin: TAPBP; ZBTB22; DAXX) | 2.33 | 1.64e-03 | 1.45e-02 |
|  | 13 | 52585597 | 52585813 | PDFSTATSENSEMBLUCSC | 217 | 7 | 11455 | tss;island | 0.053447 | 0.013892 | Not queried | 2.33 | 4.56e-01 | 4.96e-01 |
|  | 16 | 77469538 | 77469710 | PDFSTATSENSEMBLUCSC | 173 | 5 | 15369 | tss;island | 0.272443 | 0.041833 | RP11-449J10.1; ADAMTS18; AC025284.1 (margin: RP11-449J10.1; ADAMTS18; AC025284.1) | 2.33 | 7.94e-03 | 3.19e-02 |
|  | 12 | 5018715 | 5019091 | PDFSTATSENSEMBLUCSC | 377 | 10 | 10180 | island | 0.362880 | 0.726877 | Not queried | 2.33 | 1.90e-01 | 2.55e-01 |
|  | 3 | 180547422 | 180547932 | PDFSTATSENSEMBLUCSC | 511 | 4 | 29686 | island | 0.671397 | 0.897445 | Not queried | 2.33 | 2.86e-02 | 6.12e-02 |
|  | 22 | 24890690 | 24890833 | PDFSTATSENSEMBLUCSC | 144 | 7 | 27020 | tss;island | 0.943322 | 0.986471 | Not queried | 2.33 | 1.75e-02 | 5.79e-02 |
|  | 2 | 27373014 | 27373412 | PDFSTATSENSEMBLUCSC | 399 | 5 | 22621 | gene;island | 0.904336 | 0.966642 | Not queried | 2.33 | 3.10e-01 | 3.66e-01 |
|  | 6 | 31146124 | 31146583 | PDFSTATSENSEMBLUCSC | 460 | 5 | 34802 | tss;island | 0.978486 | 0.887875 | Not queried | 2.32 | 5.56e-02 | 1.00e-01 |
|  | 1 | 231297516 | 231297737 | PDFSTATSENSEMBLUCSC | 222 | 4 | 2321 | tss;island | 0.561523 | 0.854620 | Not queried | 2.32 | 2.00e-01 | 2.55e-01 |
|  | 1 | 75198211 | 75198841 | PDFSTATSENSEMBLUCSC | 631 | 7 | 1697 | tss | 0.011702 | 0.004805 | Not queried | 2.32 | 5.35e-01 | 5.62e-01 |
|  | 7 | 150756392 | 150756773 | PDFSTATSENSEMBLUCSC | 382 | 11 | 39023 | island | 0.011084 | 0.024674 | Not queried | 2.32 | 1.51e-01 | 2.11e-01 |
|  | 6 | 3227556 | 3227981 | PDFSTATSENSEMBLUCSC | 426 | 5 | 35385 | island | 0.473211 | 0.144015 | Not queried | 2.32 | 3.17e-02 | 6.50e-02 |
|  | 6 | 3227556 | 3227949 | PDFSTATSENSEMBLUCSC | 394 | 4 | 33536 | gene | 0.512297 | 0.147070 | Not queried | 2.32 | 5.71e-02 | 1.00e-01 |
|  | 17 | 55938748 | 55939089 | PDFSTATSENSEMBLUCSC | 342 | 4 | 18587 | island | 0.934965 | 0.746468 | Not queried | 2.32 | 2.86e-02 | 6.12e-02 |
|  | 16 | 29196472 | 29196699 | PDFSTATSENSEMBLUCSC | 228 | 4 | 15800 | island | 0.740903 | 0.897830 | Not queried | 2.32 | 3.43e-01 | 3.84e-01 |
|  | 15 | 89943283 | 89943836 | PDFSTATSENSEMBLUCSC | 554 | 5 | 14432 | island | 0.858817 | 0.427111 | Not queried | 2.32 | 3.17e-02 | 6.50e-02 |
|  | 18 | 67069959 | 67070461 | PDFSTATSENSEMBLUCSC | 503 | 6 | 19123 | gene;island | 0.625752 | 0.155834 | Not queried | 2.32 | 9.31e-02 | 1.53e-01 |
|  | 19 | 2278451 | 2278847 | PDFSTATSENSEMBLUCSC | 397 | 5 | 19684 | gene;island | 0.786272 | 0.968875 | Not queried | 2.32 | 3.17e-02 | 6.50e-02 |
|  | 3 | 27771667 | 27772201 | PDFSTATSENSEMBLUCSC | 535 | 7 | 28954 | island | 0.527949 | 0.828216 | Not queried | 2.32 | 5.30e-02 | 1.00e-01 |
|  | 17 | 8093380 | 8093504 | PDFSTATSENSEMBLUCSC | 125 | 4 | 16428 | gene | 0.002725 | 0.010075 | Not queried | 2.32 | 3.43e-01 | 3.84e-01 |
|  | 1 | 229569877 | 229570288 | PDFSTATSENSEMBLUCSC | 412 | 11 | 2311 | tss | 0.768394 | 0.924758 | ACTA1 (margin: RP5-1068B5.1; ACTA1; RP5-1068B5.3; NUP133) | 2.32 | 1.86e-03 | 1.45e-02 |
|  | 14 | 105616988 | 105617308 | PDFSTATSENSEMBLUCSC | 321 | 4 | 12256 | gene;island | 0.930010 | 0.745199 | Not queried | 2.32 | 2.00e-01 | 2.55e-01 |
|  | 15 | 89951787 | 89952299 | PDFSTATSENSEMBLUCSC | 513 | 6 | 14433 | island | 0.848655 | 0.958536 | Not queried | 2.32 | 1.52e-02 | 5.36e-02 |
|  | 17 | 34838488 | 34838831 | PDFSTATSENSEMBLUCSC | 344 | 4 | 18226 | island | 0.764946 | 0.937933 | Not queried | 2.32 | 3.43e-01 | 3.84e-01 |
|  | 11 | 118135094 | 118135407 | PDFSTATSENSEMBLUCSC | 314 | 7 | 7729 | tss | 0.003254 | 0.022989 | Not queried | 2.32 | 5.30e-02 | 1.00e-01 |
|  | 6 | 168936486 | 168936684 | PDFSTATSENSEMBLUCSC | 199 | 4 | 34562 | gene;island | 0.858748 | 0.964284 | Not queried | 2.32 | 1.14e-01 | 1.68e-01 |
|  | 3 | 55522611 | 55522716 | PDFSTATSENSEMBLUCSC | 106 | 4 | 28403 | tss | 0.286495 | 0.021167 | Not queried | 2.32 | 4.86e-01 | 5.11e-01 |
|  | 8 | 144457677 | 144458069 | PDFSTATSENSEMBLUCSC | 393 | 5 | 39474 | gene;island | 0.541181 | 0.870810 | RHPN1 (margin: RHPN1; RHPN1-AS1) | 2.32 | 7.94e-03 | 3.19e-02 |
|  | 6 | 111888540 | 111888840 | PDFSTATSENSEMBLUCSC | 301 | 6 | 35177 | tss | 0.749697 | 0.959940 | Not queried | 2.32 | 9.31e-02 | 1.53e-01 |
|  | 2 | 26569594 | 26570144 | PDFSTATSENSEMBLUCSC | 551 | 9 | 22612 | gene;island | 0.003775 | 0.029950 | Not queried | 2.32 | 6.25e-02 | 1.09e-01 |
|  | 5 | 524503 | 524941 | PDFSTATSENSEMBLUCSC | 439 | 5 | 32587 | island | 0.229484 | 0.530177 | Not queried | 2.32 | 3.10e-01 | 3.66e-01 |
|  | 5 | 44388799 | 44389062 | PDFSTATSENSEMBLUCSC | 264 | 6 | 32052 | tss | 0.524540 | 0.751847 | Not queried | 2.31 | 6.49e-02 | 1.11e-01 |
|  | 2 | 132439861 | 132440298 | PDFSTATSENSEMBLUCSC | 438 | 7 | 24737 | island | 0.052640 | 0.004959 | Not queried | 2.31 | 7.28e-02 | 1.23e-01 |
|  | 3 | 38080635 | 38080925 | PDFSTATSENSEMBLUCSC | 291 | 5 | 28986 | island | 0.875534 | 0.969784 | Not queried | 2.31 | 3.17e-02 | 6.50e-02 |
|  | 6 | 43149356 | 43149737 | PDFSTATSENSEMBLUCSC | 382 | 4 | 34994 | tss;island | 0.713854 | 0.415692 | Not queried | 2.31 | 4.86e-01 | 5.11e-01 |
|  | 4 | 39368001 | 39368212 | PDFSTATSENSEMBLUCSC | 212 | 6 | 30349 | tss | 0.042975 | 0.005282 | Not queried | 2.31 | 6.99e-01 | 7.09e-01 |
|  | 12 | 69202458 | 69202838 | PDFSTATSENSEMBLUCSC | 381 | 7 | 9211 | gene;island | 0.001544 | 0.003246 | Not queried | 2.31 | 2.59e-01 | 3.16e-01 |
|  | 13 | 31191332 | 31191824 | PDFSTATSENSEMBLUCSC | 493 | 8 | 11375 | tss;island | 0.001371 | 0.002629 | Not queried | 2.31 | 6.45e-01 | 6.69e-01 |
|  | 19 | 46526100 | 46526675 | PDFSTATSENSEMBLUCSC | 576 | 10 | 22168 | island | 0.639634 | 0.949675 | Not queried | 2.31 | 1.85e-02 | 6.12e-02 |
|  | 1 | 160771696 | 160771900 | PDFSTATSENSEMBLUCSC | 205 | 4 | 845 | gene;island | 0.825812 | 0.949234 | Not queried | 2.31 | 2.86e-02 | 6.12e-02 |
|  | 6 | 72298242 | 72298704 | PDFSTATSENSEMBLUCSC | 463 | 5 | 36244 | island | 0.771206 | 0.931367 | Not queried | 2.31 | 2.22e-01 | 2.76e-01 |
|  | 20 | 25129126 | 25129562 | PDFSTATSENSEMBLUCSC | 437 | 10 | 25647 | tss;island | 0.680225 | 0.882408 | Not queried | 2.31 | 1.85e-02 | 6.12e-02 |
|  | 1 | 235813272 | 235813452 | PDFSTATSENSEMBLUCSC | 181 | 4 | 1093 | gene;island | 0.017874 | 0.007502 | Not queried | 2.31 | 4.86e-01 | 5.11e-01 |
|  | 10 | 102505266 | 102505365 | PDFSTATSENSEMBLUCSC | 100 | 6 | 5297 | tss | 0.068078 | 0.016337 | Not queried | 2.31 | 9.31e-02 | 1.53e-01 |
|  | 14 | 69726197 | 69727092 | PDFSTATSENSEMBLUCSC | 896 | 11 | 12965 | island | 0.631683 | 0.559828 | Not queried | 2.31 | 3.00e-01 | 3.63e-01 |
|  | 1 | 149859456 | 149859690 | PDFSTATSENSEMBLUCSC | 235 | 5 | 1899 | tss;island | 0.000591 | 0.003498 | Not queried | 2.31 | 1.51e-01 | 2.11e-01 |
|  | 1 | 155827015 | 155827082 | PDFSTATSENSEMBLUCSC | 68 | 4 | 2004 | tss | 0.002352 | 0.009748 | Not queried | 2.31 | 2.00e-01 | 2.55e-01 |
|  | 17 | 44061117 | 44061467 | PDFSTATSENSEMBLUCSC | 351 | 4 | 16643 | gene;island | 0.770055 | 0.972032 | Not queried | 2.31 | 1.14e-01 | 1.68e-01 |
|  | 13 | 112712424 | 112712795 | PDFSTATSENSEMBLUCSC | 372 | 4 | 11908 | island | 0.755794 | 0.939226 | Not queried | 2.31 | 1.14e-01 | 1.68e-01 |
|  | 12 | 6756671 | 6756815 | PDFSTATSENSEMBLUCSC | 145 | 4 | 9519 | tss | 0.657345 | 0.399022 | Not queried | 2.31 | 3.43e-01 | 3.84e-01 |
|  | 4 | 128983279 | 128983807 | PDFSTATSENSEMBLUCSC | 529 | 4 | 30126 | gene;island | 0.016305 | 0.017372 | Not queried | 2.30 | 8.86e-01 | 8.89e-01 |
|  | 11 | 62783361 | 62783573 | PDFSTATSENSEMBLUCSC | 213 | 7 | 7374 | tss | 0.752979 | 0.881236 | Not queried | 2.30 | 5.30e-02 | 1.00e-01 |
|  | 7 | 157216599 | 157216893 | PDFSTATSENSEMBLUCSC | 295 | 4 | 39102 | island | 0.750924 | 0.961857 | Not queried | 2.30 | 5.71e-02 | 1.00e-01 |
|  | 8 | 109799518 | 109799756 | PDFSTATSENSEMBLUCSC | 239 | 4 | 39397 | gene | 0.579637 | 0.196583 | Not queried | 2.30 | 2.86e-02 | 6.12e-02 |
|  | 6 | 33094069 | 33094339 | PDFSTATSENSEMBLUCSC | 271 | 4 | 34191 | gene | 0.447700 | 0.532477 | Not queried | 2.30 | 4.86e-01 | 5.11e-01 |
|  | 8 | 37797680 | 37797828 | PDFSTATSENSEMBLUCSC | 149 | 5 | 39649 | tss | 0.760893 | 0.954822 | Not queried | 2.30 | 1.51e-01 | 2.11e-01 |
|  | 16 | 30934656 | 30934727 | PDFSTATSENSEMBLUCSC | 72 | 5 | 15198 | tss;island | 0.002049 | 0.006712 | Not queried | 2.30 | 2.22e-01 | 2.76e-01 |
|  | 2 | 222437040 | 222437130 | PDFSTATSENSEMBLUCSC | 91 | 6 | 23913 | tss | 0.043454 | 0.009279 | Not queried | 2.30 | 1.52e-02 | 5.36e-02 |
|  | 7 | 2556962 | 2557466 | PDFSTATSENSEMBLUCSC | 505 | 8 | 36802 | gene;tss;island | 0.564502 | 0.820074 | LFNG (margin: LFNG; MIR4648) | 2.30 | 1.04e-02 | 3.96e-02 |
|  | 1 | 1148973 | 1149211 | PDFSTATSENSEMBLUCSC | 239 | 4 | 21 | gene;island | 0.659872 | 0.931937 | Not queried | 2.30 | 1.14e-01 | 1.68e-01 |
|  | 18 | 28682229 | 28682497 | PDFSTATSENSEMBLUCSC | 269 | 4 | 19447 | island | 0.006897 | 0.012515 | Not queried | 2.30 | 3.43e-01 | 3.84e-01 |
|  | 17 | 80668611 | 80668944 | PDFSTATSENSEMBLUCSC | 334 | 5 | 19006 | island | 0.696083 | 0.886419 | (margin: FN3KRP; RP11-388C12.1) | 2.30 | 7.94e-03 | 3.19e-02 |
|  | 4 | 40751717 | 40751845 | PDFSTATSENSEMBLUCSC | 129 | 4 | 30360 | tss | 0.229501 | 0.056309 | Not queried | 2.30 | 2.00e-01 | 2.55e-01 |
|  | 14 | 100258973 | 100259352 | PDFSTATSENSEMBLUCSC | 380 | 6 | 12601 | tss;island | 0.985413 | 0.884920 | Not queried | 2.30 | 2.60e-02 | 6.12e-02 |
|  | 17 | 7255461 | 7255736 | PDFSTATSENSEMBLUCSC | 276 | 4 | 16392 | gene;island | 0.004184 | 0.024143 | Not queried | 2.30 | 5.71e-02 | 1.00e-01 |
|  | 6 | 168479622 | 168479787 | PDFSTATSENSEMBLUCSC | 166 | 4 | 34560 | gene | 0.657180 | 0.923762 | Not queried | 2.30 | 2.00e-01 | 2.55e-01 |
|  | 19 | 15121204 | 15121596 | PDFSTATSENSEMBLUCSC | 393 | 9 | 21691 | island | 0.878001 | 0.972697 | Not queried | 2.30 | 4.00e-02 | 8.03e-02 |
|  | 19 | 15121204 | 15121531 | PDFSTATSENSEMBLUCSC | 328 | 7 | 20552 | tss | 0.878001 | 0.972697 | Not queried | 2.30 | 7.28e-02 | 1.23e-01 |
|  | 3 | 32509018 | 32509543 | PDFSTATSENSEMBLUCSC | 526 | 9 | 28964 | island | 0.895794 | 0.271255 | Not queried | 2.30 | 3.40e-01 | 3.84e-01 |
|  | 2 | 162272107 | 162272687 | PDFSTATSENSEMBLUCSC | 581 | 7 | 24821 | island | 0.689846 | 0.558048 | Not queried | 2.30 | 2.09e-01 | 2.65e-01 |
|  | 16 | 21289750 | 21289947 | PDFSTATSENSEMBLUCSC | 198 | 6 | 15127 | tss | 0.071879 | 0.019875 | Not queried | 2.30 | 4.11e-02 | 8.14e-02 |
|  | 3 | 132756721 | 132757973 | PDFSTATSENSEMBLUCSC | 1253 | 16 | 29487 | island | 0.782673 | 0.970122 | Not queried | 2.29 | 2.11e-02 | 6.12e-02 |
|  | 21 | 44312851 | 44313047 | PDFSTATSENSEMBLUCSC | 197 | 4 | 26571 | tss;island | 0.025321 | 0.002931 | Not queried | 2.29 | 2.00e-01 | 2.55e-01 |
|  | 12 | 39299501 | 39299844 | PDFSTATSENSEMBLUCSC | 344 | 6 | 9636 | tss | 0.453800 | 0.760164 | RP11-396F22.1; CPNE8 (margin: RP11-396F22.1; CPNE8; AC067735.1) | 2.29 | 2.16e-03 | 1.45e-02 |
|  | 4 | 113432595 | 113432911 | PDFSTATSENSEMBLUCSC | 317 | 4 | 31255 | island | 0.798188 | 0.951951 | Not queried | 2.29 | 1.14e-01 | 1.68e-01 |
|  | 19 | 12949372 | 12949727 | PDFSTATSENSEMBLUCSC | 356 | 5 | 19868 | gene;island | 0.203697 | 0.082537 | Not queried | 2.29 | 5.56e-02 | 1.00e-01 |
|  | 7 | 22539908 | 22540154 | PDFSTATSENSEMBLUCSC | 247 | 5 | 37465 | tss | 0.498757 | 0.185695 | Not queried | 2.29 | 3.17e-02 | 6.50e-02 |
|  | 5 | 149792330 | 149792515 | PDFSTATSENSEMBLUCSC | 186 | 6 | 32429 | tss | 0.371342 | 0.752338 | Not queried | 2.29 | 1.32e-01 | 1.89e-01 |
|  | 19 | 22605151 | 22605234 | PDFSTATSENSEMBLUCSC | 84 | 4 | 20663 | tss | 0.499739 | 0.225457 | Not queried | 2.29 | 2.86e-02 | 6.12e-02 |
|  | 16 | 72042591 | 72042925 | PDFSTATSENSEMBLUCSC | 335 | 5 | 16081 | island | 0.000456 | 0.005193 | Not queried | 2.29 | 3.17e-02 | 6.50e-02 |
|  | 14 | 65381157 | 65381464 | PDFSTATSENSEMBLUCSC | 308 | 6 | 12116 | gene | 0.001697 | 0.004318 | Not queried | 2.29 | 1.80e-01 | 2.45e-01 |
|  | 13 | 113739251 | 113739577 | PDFSTATSENSEMBLUCSC | 327 | 4 | 11275 | gene;island | 0.603860 | 0.897591 | Not queried | 2.29 | 2.86e-02 | 6.12e-02 |
|  | 8 | 53852030 | 53852446 | PDFSTATSENSEMBLUCSC | 417 | 7 | 39700 | tss;island | 0.775513 | 0.964093 | Not queried | 2.29 | 7.28e-02 | 1.23e-01 |
|  | 6 | 33133329 | 33133752 | PDFSTATSENSEMBLUCSC | 424 | 9 | 34195 | gene | 0.368527 | 0.726795 | COL11A2 (margin: COL11A2) | 2.29 | 7.77e-03 | 3.19e-02 |
|  | 15 | 74494781 | 74494909 | PDFSTATSENSEMBLUCSC | 129 | 5 | 13420 | gene | 0.602113 | 0.840038 | RP11-60L3.1; STRA6 (margin: RP11-60L3.1; STRA6) | 2.29 | 7.94e-03 | 3.19e-02 |
|  | 7 | 121945445 | 121945743 | PDFSTATSENSEMBLUCSC | 299 | 4 | 37866 | tss;island | 0.397662 | 0.723486 | Not queried | 2.29 | 1.14e-01 | 1.68e-01 |
|  | 11 | 64067685 | 64067858 | PDFSTATSENSEMBLUCSC | 174 | 6 | 7394 | tss | 0.676240 | 0.972853 | Not queried | 2.29 | 2.60e-02 | 6.12e-02 |
|  | 4 | 3464653 | 3465004 | PDFSTATSENSEMBLUCSC | 352 | 9 | 30262 | tss | 0.409553 | 0.030037 | DOK7 (margin: DOK7) | 2.29 | 5.64e-03 | 3.18e-02 |
|  | 2 | 175870204 | 175870490 | PDFSTATSENSEMBLUCSC | 287 | 7 | 23726 | tss;island | 0.011921 | 0.010694 | Not queried | 2.29 | 2.59e-01 | 3.16e-01 |
|  | 19 | 50553682 | 50554067 | PDFSTATSENSEMBLUCSC | 386 | 7 | 22299 | island | 0.053922 | 0.018078 | Not queried | 2.29 | 2.62e-02 | 6.12e-02 |
|  | 13 | 33780205 | 33780362 | PDFSTATSENSEMBLUCSC | 158 | 4 | 11386 | tss | 0.979705 | 0.896050 | Not queried | 2.29 | 5.71e-02 | 1.00e-01 |
|  | 1 | 75595919 | 75596336 | PDFSTATSENSEMBLUCSC | 418 | 4 | 586 | gene;island | 0.749477 | 0.934043 | Not queried | 2.29 | 2.00e-01 | 2.55e-01 |
|  | 18 | 21719263 | 21719568 | PDFSTATSENSEMBLUCSC | 306 | 7 | 19077 | gene;island | 0.024001 | 0.087327 | Not queried | 2.29 | 9.73e-02 | 1.55e-01 |
|  | 12 | 70759665 | 70760024 | PDFSTATSENSEMBLUCSC | 360 | 6 | 9867 | tss | 0.022967 | 0.010898 | Not queried | 2.29 | 1.80e-01 | 2.45e-01 |
|  | 7 | 156111254 | 156111445 | PDFSTATSENSEMBLUCSC | 192 | 4 | 39083 | island | 0.325070 | 0.724834 | Not queried | 2.29 | 2.00e-01 | 2.55e-01 |
|  | 15 | 93633145 | 93633408 | PDFSTATSENSEMBLUCSC | 264 | 5 | 13916 | tss;island | 0.892966 | 0.978872 | Not queried | 2.29 | 9.52e-02 | 1.53e-01 |
|  | 12 | 113495386 | 113495839 | PDFSTATSENSEMBLUCSC | 454 | 5 | 10824 | island | 0.555105 | 0.832691 | Not queried | 2.29 | 1.51e-01 | 2.11e-01 |
|  | 12 | 91573135 | 91573372 | PDFSTATSENSEMBLUCSC | 238 | 4 | 9242 | gene;tss | 0.253330 | 0.612367 | Not queried | 2.29 | 3.43e-01 | 3.84e-01 |
|  | 11 | 130029478 | 130029615 | PDFSTATSENSEMBLUCSC | 138 | 4 | 7817 | tss | 0.057189 | 0.017115 | Not queried | 2.29 | 5.71e-02 | 1.00e-01 |
|  | 12 | 57636980 | 57637690 | PDFSTATSENSEMBLUCSC | 711 | 7 | 10557 | island | 0.014109 | 0.006680 | Not queried | 2.28 | 3.83e-01 | 4.27e-01 |
|  | 16 | 74331461 | 74331660 | PDFSTATSENSEMBLUCSC | 200 | 5 | 14840 | gene;island | 0.022205 | 0.046273 | Not queried | 2.28 | 1.51e-01 | 2.11e-01 |
|  | 6 | 32975590 | 32976131 | PDFSTATSENSEMBLUCSC | 542 | 15 | 34177 | gene;island | 0.885332 | 0.912780 | Not queried | 2.28 | 6.75e-02 | 1.15e-01 |
|  | 1 | 222790952 | 222791172 | PDFSTATSENSEMBLUCSC | 221 | 4 | 2267 | tss;island | 0.092226 | 0.026112 | Not queried | 2.28 | 6.86e-01 | 6.99e-01 |
|  | 6 | 62996119 | 62996299 | PDFSTATSENSEMBLUCSC | 181 | 6 | 35052 | tss | 0.854085 | 0.461255 | Not queried | 2.28 | 9.31e-02 | 1.53e-01 |
|  | 11 | 60609242 | 60609414 | PDFSTATSENSEMBLUCSC | 173 | 6 | 7322 | tss | 0.018190 | 0.047195 | Not queried | 2.28 | 4.85e-01 | 5.11e-01 |
|  | 7 | 94285270 | 94286955 | PDFSTATSENSEMBLUCSC | 1686 | 61 | 37093 | gene;tss;island | 0.816427 | 0.441327 | PEG10; SGCE (margin: PEG10; SGCE) | 2.28 | 3.43e-12 | 5.17e-09 |
|  | 3 | 98451002 | 98451398 | PDFSTATSENSEMBLUCSC | 397 | 5 | 28461 | tss;island | 0.104523 | 0.024945 | Not queried | 2.28 | 5.56e-02 | 1.00e-01 |
|  | 17 | 17399667 | 17400111 | PDFSTATSENSEMBLUCSC | 445 | 9 | 18066 | island | 0.022560 | 0.140404 | Not queried | 2.28 | 1.88e-02 | 6.12e-02 |
|  | 19 | 37157776 | 37157995 | PDFSTATSENSEMBLUCSC | 220 | 8 | 20729 | tss | 0.030593 | 0.005540 | Not queried | 2.28 | 1.48e-02 | 5.36e-02 |
|  | 11 | 64703415 | 64703818 | PDFSTATSENSEMBLUCSC | 404 | 5 | 7409 | tss | 0.866784 | 0.518011 | Not queried | 2.28 | 8.41e-01 | 8.48e-01 |
|  | 10 | 93392644 | 93393509 | PDFSTATSENSEMBLUCSC | 866 | 14 | 5932 | island | 0.504912 | 0.901141 | PPP1R3C (margin: PPP1R3C) | 2.28 | 5.23e-04 | 6.47e-03 |
|  | 2 | 237072673 | 237072998 | PDFSTATSENSEMBLUCSC | 326 | 4 | 25223 | island | 0.868269 | 0.974451 | Not queried | 2.28 | 2.00e-01 | 2.55e-01 |
|  | 14 | 57274550 | 57274900 | PDFSTATSENSEMBLUCSC | 351 | 5 | 12091 | gene;island | 0.785900 | 0.962208 | Not queried | 2.28 | 5.56e-02 | 1.00e-01 |
|  | 6 | 118228382 | 118228650 | PDFSTATSENSEMBLUCSC | 269 | 6 | 35199 | tss;island | 0.691474 | 0.318317 | SLC35F1 (margin: SLC35F1) | 2.28 | 2.16e-03 | 1.45e-02 |
|  | 17 | 57184330 | 57184624 | PDFSTATSENSEMBLUCSC | 295 | 9 | 17541 | tss | 0.007384 | 0.021756 | AC099850.1; TRIM37 (margin: AC099850.1; TRIM37; SKA2) | 2.28 | 3.99e-03 | 2.47e-02 |
|  | 7 | 26903972 | 26904713 | PDFSTATSENSEMBLUCSC | 742 | 13 | 38333 | island | 0.019891 | 0.002245 | Not queried | 2.28 | 6.42e-02 | 1.11e-01 |
|  | 2 | 242497960 | 242498098 | PDFSTATSENSEMBLUCSC | 139 | 4 | 23991 | tss;island | 0.281245 | 0.033572 | Not queried | 2.28 | 3.43e-01 | 3.84e-01 |
|  | 12 | 129337674 | 129337910 | PDFSTATSENSEMBLUCSC | 237 | 5 | 10116 | tss;island | 0.730018 | 0.925775 | Not queried | 2.28 | 2.22e-01 | 2.76e-01 |
|  | 6 | 30881112 | 30882384 | PDFSTATSENSEMBLUCSC | 1273 | 39 | 35740 | island | 0.441796 | 0.049736 | GTF2H4; VARS2 (margin: RN7SL175P; GTF2H4; VARS2) | 2.27 | 1.16e-02 | 4.31e-02 |
|  | 8 | 9791214 | 9791557 | PDFSTATSENSEMBLUCSC | 344 | 4 | 40067 | island | 0.127037 | 0.028375 | Not queried | 2.27 | 5.71e-02 | 1.00e-01 |
|  | 15 | 50647554 | 50647856 | PDFSTATSENSEMBLUCSC | 303 | 10 | 13346 | gene;tss;island | 0.007036 | 0.012586 | Not queried | 2.27 | 2.88e-02 | 6.16e-02 |
|  | 11 | 63753020 | 63753251 | PDFSTATSENSEMBLUCSC | 232 | 6 | 7382 | tss | 0.579004 | 0.852400 | Not queried | 2.27 | 4.11e-02 | 8.14e-02 |
|  | 1 | 12123262 | 12124114 | PDFSTATSENSEMBLUCSC | 853 | 10 | 2698 | island | 0.626779 | 0.868370 | TNFRSF8 (margin: TNFRSF8) | 2.27 | 6.84e-03 | 3.19e-02 |
|  | 1 | 12123437 | 12124114 | PDFSTATSENSEMBLUCSC | 678 | 6 | 231 | gene | 0.629530 | 0.789698 | Not queried | 2.27 | 1.80e-01 | 2.45e-01 |
|  | 18 | 55019621 | 55019843 | PDFSTATSENSEMBLUCSC | 223 | 5 | 19531 | island | 0.616659 | 0.885592 | Not queried | 2.27 | 2.22e-01 | 2.76e-01 |
|  | 19 | 2613894 | 2614177 | PDFSTATSENSEMBLUCSC | 284 | 6 | 19698 | gene;island | 0.043255 | 0.052173 | Not queried | 2.27 | 5.89e-01 | 6.11e-01 |
|  | 12 | 6982806 | 6983111 | PDFSTATSENSEMBLUCSC | 306 | 5 | 9531 | tss;island | 0.015777 | 0.072496 | Not queried | 2.27 | 4.21e-01 | 4.59e-01 |
|  | 5 | 1084425 | 1084670 | PDFSTATSENSEMBLUCSC | 246 | 4 | 31520 | gene;island | 0.773931 | 0.940740 | Not queried | 2.27 | 1.14e-01 | 1.68e-01 |
|  | 3 | 62861055 | 62861311 | PDFSTATSENSEMBLUCSC | 257 | 7 | 29252 | island | 0.952785 | 0.580665 | Not queried | 2.27 | 9.73e-02 | 1.55e-01 |
|  | 19 | 3062808 | 3062986 | PDFSTATSENSEMBLUCSC | 179 | 4 | 20339 | tss;island | 0.037515 | 0.200481 | Not queried | 2.27 | 3.43e-01 | 3.84e-01 |
|  | 16 | 21289539 | 21289947 | PDFSTATSENSEMBLUCSC | 409 | 8 | 14689 | gene;island | 0.047734 | 0.019300 | Not queried | 2.27 | 4.99e-02 | 9.81e-02 |
|  | 17 | 59534637 | 59534998 | PDFSTATSENSEMBLUCSC | 362 | 5 | 16720 | gene;island | 0.985086 | 0.835342 | Not queried | 2.27 | 5.48e-01 | 5.70e-01 |
|  | 4 | 113739249 | 113739379 | PDFSTATSENSEMBLUCSC | 131 | 5 | 30102 | gene | 0.044811 | 0.185027 | Not queried | 2.27 | 9.52e-02 | 1.53e-01 |
|  | 19 | 21646329 | 21646781 | PDFSTATSENSEMBLUCSC | 453 | 5 | 21834 | island | 0.870430 | 0.979348 | Not queried | 2.27 | 5.56e-02 | 1.00e-01 |
|  | 13 | 114760892 | 114761326 | PDFSTATSENSEMBLUCSC | 435 | 4 | 11302 | gene;island | 0.627077 | 0.912614 | Not queried | 2.27 | 1.14e-01 | 1.68e-01 |
|  | 11 | 101918304 | 101918710 | PDFSTATSENSEMBLUCSC | 407 | 4 | 6876 | gene | 0.285852 | 0.546618 | Not queried | 2.27 | 4.86e-01 | 5.11e-01 |
|  | 7 | 157690609 | 157690769 | PDFSTATSENSEMBLUCSC | 161 | 4 | 37350 | gene;island | 0.650514 | 0.906199 | Not queried | 2.27 | 2.00e-01 | 2.55e-01 |
|  | 5 | 157078573 | 157078881 | PDFSTATSENSEMBLUCSC | 309 | 4 | 31875 | gene;island | 0.617743 | 0.921580 | Not queried | 2.27 | 1.14e-01 | 1.68e-01 |
|  | 4 | 111558988 | 111559134 | PDFSTATSENSEMBLUCSC | 147 | 4 | 30550 | tss;island | 0.774222 | 0.978243 | Not queried | 2.27 | 4.86e-01 | 5.11e-01 |
|  | 6 | 32634245 | 32634508 | PDFSTATSENSEMBLUCSC | 264 | 6 | 35922 | island | 0.203997 | 0.028910 | Not queried | 2.27 | 2.40e-01 | 2.95e-01 |
|  | 15 | 68127656 | 68128261 | PDFSTATSENSEMBLUCSC | 606 | 5 | 14253 | island | 0.889136 | 0.981669 | Not queried | 2.27 | 5.56e-02 | 1.00e-01 |
|  | 1 | 217311511 | 217311608 | PDFSTATSENSEMBLUCSC | 98 | 4 | 2255 | tss;island | 0.818419 | 0.979201 | Not queried | 2.27 | 1.14e-01 | 1.68e-01 |
|  | 2 | 176993017 | 176993320 | PDFSTATSENSEMBLUCSC | 304 | 6 | 23738 | tss;island | 0.721058 | 0.956796 | Not queried | 2.27 | 6.49e-02 | 1.11e-01 |
|  | 6 | 33135753 | 33136061 | PDFSTATSENSEMBLUCSC | 309 | 7 | 34198 | gene | 0.558704 | 0.787015 | COL11A2 (margin: COL11A2) | 2.27 | 1.11e-02 | 4.14e-02 |
|  | 2 | 223163725 | 223163809 | PDFSTATSENSEMBLUCSC | 85 | 4 | 23916 | tss | 0.927883 | 0.981574 | Not queried | 2.27 | 1.14e-01 | 1.68e-01 |
|  | 1 | 6304272 | 6304499 | PDFSTATSENSEMBLUCSC | 228 | 4 | 165 | gene | 0.778740 | 0.957539 | Not queried | 2.27 | 2.00e-01 | 2.55e-01 |
|  | 3 | 169530417 | 169530621 | PDFSTATSENSEMBLUCSC | 205 | 5 | 28709 | tss | 0.577273 | 0.906356 | LRRC34 (margin: LRRIQ4; LRRC34) | 2.27 | 7.94e-03 | 3.19e-02 |
|  | 20 | 42983920 | 42984394 | PDFSTATSENSEMBLUCSC | 475 | 9 | 25758 | tss | 0.679949 | 0.898892 | Not queried | 2.26 | 6.25e-02 | 1.09e-01 |
|  | 5 | 174905244 | 174905618 | PDFSTATSENSEMBLUCSC | 375 | 8 | 33419 | island | 0.035262 | 0.017942 | Not queried | 2.26 | 4.42e-01 | 4.81e-01 |
|  | 13 | 48611439 | 48611687 | PDFSTATSENSEMBLUCSC | 249 | 9 | 11431 | tss | 0.000626 | 0.003124 | Not queried | 2.26 | 1.13e-01 | 1.68e-01 |
|  | 2 | 173099800 | 173100242 | PDFSTATSENSEMBLUCSC | 443 | 4 | 24864 | island | 0.982609 | 0.873515 | Not queried | 2.26 | 2.00e-01 | 2.55e-01 |
|  | 7 | 73241729 | 73242028 | PDFSTATSENSEMBLUCSC | 300 | 4 | 38570 | island | 0.101712 | 0.013627 | Not queried | 2.26 | 2.00e-01 | 2.55e-01 |
|  | 1 | 35395541 | 35395837 | PDFSTATSENSEMBLUCSC | 297 | 4 | 3030 | island | 0.699909 | 0.851871 | Not queried | 2.26 | 6.86e-01 | 6.99e-01 |
|  | 6 | 22569543 | 22569665 | PDFSTATSENSEMBLUCSC | 123 | 6 | 34665 | tss | 0.914672 | 0.973490 | Not queried | 2.26 | 4.11e-02 | 8.14e-02 |
|  | 1 | 229569608 | 229570288 | PDFSTATSENSEMBLUCSC | 681 | 14 | 4310 | island | 0.774712 | 0.930603 | ACTA1 (margin: RP5-1068B5.1; ACTA1; RP5-1068B5.3; NUP133) | 2.26 | 1.43e-03 | 1.36e-02 |
|  | 13 | 37453429 | 37454023 | PDFSTATSENSEMBLUCSC | 595 | 7 | 11135 | gene | 0.441856 | 0.918422 | Not queried | 2.26 | 7.28e-02 | 1.23e-01 |
|  | 21 | 14982080 | 14982363 | PDFSTATSENSEMBLUCSC | 284 | 4 | 26485 | tss | 0.417152 | 0.622696 | Not queried | 2.26 | 4.86e-01 | 5.11e-01 |
|  | 4 | 141294522 | 141294796 | PDFSTATSENSEMBLUCSC | 275 | 9 | 30137 | gene;island | 0.015921 | 0.004892 | Not queried | 2.26 | 6.25e-02 | 1.09e-01 |
|  | 19 | 44455140 | 44455605 | PDFSTATSENSEMBLUCSC | 466 | 10 | 22098 | island | 0.000759 | 0.012313 | Not queried | 2.26 | 1.43e-01 | 2.04e-01 |
|  | 7 | 150756392 | 150756600 | PDFSTATSENSEMBLUCSC | 209 | 10 | 37988 | tss | 0.011204 | 0.028822 | Not queried | 2.26 | 1.43e-01 | 2.04e-01 |
|  | 11 | 61283865 | 61284134 | PDFSTATSENSEMBLUCSC | 270 | 4 | 6629 | gene;island | 0.688316 | 0.894034 | Not queried | 2.26 | 3.43e-01 | 3.84e-01 |
|  | 2 | 169642729 | 169643037 | PDFSTATSENSEMBLUCSC | 309 | 5 | 23698 | tss | 0.510592 | 0.894784 | Not queried | 2.26 | 3.10e-01 | 3.66e-01 |
|  | 16 | 89146961 | 89147306 | PDFSTATSENSEMBLUCSC | 346 | 4 | 16237 | island | 0.347469 | 0.764605 | Not queried | 2.26 | 1.14e-01 | 1.68e-01 |
|  | 16 | 67515103 | 67515344 | PDFSTATSENSEMBLUCSC | 242 | 4 | 15304 | tss | 0.001755 | 0.006902 | Not queried | 2.26 | 2.00e-01 | 2.55e-01 |
|  | 21 | 43491213 | 43491424 | PDFSTATSENSEMBLUCSC | 212 | 5 | 26563 | tss | 0.554775 | 0.802893 | Not queried | 2.26 | 1.59e-02 | 5.36e-02 |
|  | 1 | 27668325 | 27668445 | PDFSTATSENSEMBLUCSC | 121 | 4 | 1408 | tss | 0.133871 | 0.031877 | Not queried | 2.26 | 1.14e-01 | 1.68e-01 |
|  | 4 | 146100596 | 146101151 | PDFSTATSENSEMBLUCSC | 556 | 7 | 31342 | island | 0.001091 | 0.004342 | Not queried | 2.26 | 1.28e-01 | 1.87e-01 |
|  | 6 | 133563764 | 133564066 | PDFSTATSENSEMBLUCSC | 303 | 4 | 34477 | gene;island | 0.696958 | 0.928223 | Not queried | 2.26 | 5.71e-02 | 1.00e-01 |
|  | 10 | 3500383 | 3500727 | PDFSTATSENSEMBLUCSC | 345 | 4 | 5512 | island | 0.822768 | 0.507067 | Not queried | 2.25 | 2.86e-02 | 6.12e-02 |
|  | 5 | 111092845 | 111093945 | PDFSTATSENSEMBLUCSC | 1101 | 15 | 31710 | gene;tss;island | 0.064976 | 0.023718 | Not queried | 2.25 | 4.08e-02 | 8.14e-02 |
|  | 19 | 1304806 | 1305252 | PDFSTATSENSEMBLUCSC | 447 | 4 | 21212 | island | 0.806657 | 0.930135 | Not queried | 2.25 | 5.71e-02 | 1.00e-01 |
|  | 5 | 37834909 | 37835168 | PDFSTATSENSEMBLUCSC | 260 | 5 | 32025 | tss | 0.833745 | 0.959506 | Not queried | 2.25 | 9.52e-02 | 1.53e-01 |
|  | 3 | 50487955 | 50488230 | PDFSTATSENSEMBLUCSC | 276 | 4 | 27810 | gene | 0.855173 | 0.970135 | Not queried | 2.25 | 1.14e-01 | 1.68e-01 |
|  | 11 | 118781633 | 118781978 | PDFSTATSENSEMBLUCSC | 346 | 7 | 7741 | tss | 0.015251 | 0.137586 | Not queried | 2.25 | 1.75e-02 | 5.79e-02 |
|  | 12 | 7055452 | 7056008 | PDFSTATSENSEMBLUCSC | 557 | 7 | 10221 | island | 0.066742 | 0.022265 | C12orf57; PTPN6; U47924.31 (margin: ATN1; C12orf57; RNU7-1; PTPN6; U47924.31) | 2.25 | 4.08e-03 | 2.47e-02 |
|  | 12 | 7055452 | 7055683 | PDFSTATSENSEMBLUCSC | 232 | 5 | 9537 | tss | 0.052604 | 0.022540 | Not queried | 2.25 | 5.56e-02 | 1.00e-01 |
|  | 8 | 142437128 | 142437236 | PDFSTATSENSEMBLUCSC | 109 | 4 | 39453 | gene;island | 0.796884 | 0.964029 | Not queried | 2.25 | 3.43e-01 | 3.84e-01 |
|  | 2 | 45176863 | 45177243 | PDFSTATSENSEMBLUCSC | 381 | 4 | 24264 | island | 0.645321 | 0.937876 | Not queried | 2.25 | 3.43e-01 | 3.84e-01 |
|  | 10 | 7661611 | 7661796 | PDFSTATSENSEMBLUCSC | 186 | 5 | 4484 | gene | 0.786813 | 0.897924 | Not queried | 2.25 | 4.21e-01 | 4.59e-01 |
|  | 19 | 51925128 | 51925472 | PDFSTATSENSEMBLUCSC | 345 | 4 | 22341 | island | 0.204137 | 0.108727 | Not queried | 2.25 | 4.86e-01 | 5.11e-01 |
|  | 10 | 99789825 | 99790205 | PDFSTATSENSEMBLUCSC | 381 | 4 | 4703 | gene;island | 0.752264 | 0.852168 | Not queried | 2.25 | 6.86e-01 | 6.99e-01 |
|  | 17 | 57184151 | 57184624 | PDFSTATSENSEMBLUCSC | 474 | 12 | 18606 | island | 0.007404 | 0.021810 | AC099850.1; TRIM37 (margin: AC099850.1; TRIM37; SKA2) | 2.25 | 5.56e-03 | 3.16e-02 |
|  | 3 | 64211155 | 64211659 | PDFSTATSENSEMBLUCSC | 505 | 5 | 28433 | tss | 0.699769 | 0.806278 | Not queried | 2.25 | 5.48e-01 | 5.70e-01 |
|  | 16 | 966362 | 966659 | PDFSTATSENSEMBLUCSC | 298 | 4 | 14553 | gene;island | 0.819231 | 0.971114 | Not queried | 2.25 | 1.14e-01 | 1.68e-01 |
|  | 5 | 14581404 | 14581826 | PDFSTATSENSEMBLUCSC | 423 | 8 | 31992 | tss | 0.508679 | 0.211921 | Not queried | 2.25 | 2.34e-01 | 2.90e-01 |
|  | 3 | 39851641 | 39851931 | PDFSTATSENSEMBLUCSC | 291 | 4 | 27726 | gene;island | 0.661790 | 0.429235 | Not queried | 2.25 | 3.43e-01 | 3.84e-01 |
|  | 2 | 11809681 | 11810183 | PDFSTATSENSEMBLUCSC | 503 | 5 | 22574 | gene | 0.636249 | 0.172288 | Not queried | 2.25 | 5.48e-01 | 5.70e-01 |
|  | 7 | 158380884 | 158381220 | PDFSTATSENSEMBLUCSC | 337 | 4 | 38021 | tss;island | 0.600113 | 0.847888 | Not queried | 2.25 | 2.00e-01 | 2.55e-01 |
|  | 16 | 67219152 | 67219218 | PDFSTATSENSEMBLUCSC | 67 | 4 | 14792 | gene;tss;island | 0.741678 | 0.943390 | Not queried | 2.25 | 1.14e-01 | 1.68e-01 |
|  | 16 | 22308466 | 22308661 | PDFSTATSENSEMBLUCSC | 196 | 5 | 15131 | tss;island | 0.002672 | 0.011671 | Not queried | 2.25 | 2.22e-01 | 2.76e-01 |
|  | 7 | 5183992 | 5184295 | PDFSTATSENSEMBLUCSC | 304 | 4 | 38212 | island | 0.474555 | 0.807479 | Not queried | 2.25 | 1.14e-01 | 1.68e-01 |
|  | 6 | 37225949 | 37226214 | PDFSTATSENSEMBLUCSC | 266 | 5 | 34321 | gene;tss;island | 0.000770 | 0.004145 | Not queried | 2.25 | 4.21e-01 | 4.59e-01 |
|  | 16 | 83171068 | 83171314 | PDFSTATSENSEMBLUCSC | 247 | 4 | 14860 | gene | 0.039238 | 0.117220 | Not queried | 2.25 | 2.86e-02 | 6.12e-02 |
|  | 17 | 7258337 | 7258501 | PDFSTATSENSEMBLUCSC | 165 | 4 | 17940 | island | 0.472430 | 0.726930 | Not queried | 2.25 | 2.86e-02 | 6.12e-02 |
|  | 11 | 93394605 | 93394809 | PDFSTATSENSEMBLUCSC | 205 | 8 | 7621 | tss | 0.013287 | 0.007021 | Not queried | 2.25 | 3.28e-01 | 3.84e-01 |
|  | 11 | 93394605 | 93394910 | PDFSTATSENSEMBLUCSC | 306 | 10 | 8729 | island | 0.008758 | 0.007021 | Not queried | 2.25 | 4.81e-01 | 5.11e-01 |
|  | 3 | 62305003 | 62305285 | PDFSTATSENSEMBLUCSC | 283 | 5 | 28425 | tss;island | 0.560357 | 0.211827 | Not queried | 2.25 | 1.59e-02 | 5.36e-02 |
|  | 5 | 176520024 | 176520502 | PDFSTATSENSEMBLUCSC | 479 | 5 | 31916 | gene;island | 0.925098 | 0.984471 | Not queried | 2.25 | 5.56e-02 | 1.00e-01 |
|  | 14 | 57261601 | 57262177 | PDFSTATSENSEMBLUCSC | 577 | 5 | 12874 | island | 0.574637 | 0.942532 | Not queried | 2.25 | 3.10e-01 | 3.66e-01 |
|  | 17 | 46035183 | 46035343 | PDFSTATSENSEMBLUCSC | 161 | 4 | 17448 | tss | 0.037437 | 0.006345 | Not queried | 2.25 | 2.00e-01 | 2.55e-01 |
|  | 3 | 110789300 | 110789788 | PDFSTATSENSEMBLUCSC | 489 | 4 | 29352 | island | 0.523407 | 0.796429 | Not queried | 2.25 | 2.00e-01 | 2.55e-01 |
|  | 11 | 133994642 | 133995182 | PDFSTATSENSEMBLUCSC | 541 | 5 | 6989 | gene;island | 0.364658 | 0.944677 | Not queried | 2.25 | 1.51e-01 | 2.11e-01 |
|  | 1 | 111813509 | 111813852 | PDFSTATSENSEMBLUCSC | 344 | 4 | 3590 | island | 0.630830 | 0.927324 | Not queried | 2.24 | 1.14e-01 | 1.68e-01 |
|  | 6 | 30698584 | 30698987 | PDFSTATSENSEMBLUCSC | 404 | 11 | 33818 | gene | 0.258745 | 0.124124 | Not queried | 2.24 | 1.58e-02 | 5.36e-02 |
|  | 1 | 107682504 | 107682963 | PDFSTATSENSEMBLUCSC | 460 | 6 | 3537 | island | 0.353124 | 0.719032 | NTNG1 (margin: NTNG1) | 2.24 | 2.16e-03 | 1.45e-02 |
|  | 11 | 57508416 | 57509018 | PDFSTATSENSEMBLUCSC | 603 | 9 | 8293 | island | 0.336491 | 0.069017 | Not queried | 2.24 | 4.36e-01 | 4.76e-01 |
|  | 11 | 57508416 | 57508677 | PDFSTATSENSEMBLUCSC | 262 | 7 | 7309 | tss | 0.415026 | 0.395464 | Not queried | 2.24 | 5.35e-01 | 5.62e-01 |
|  | 3 | 75668627 | 75669109 | PDFSTATSENSEMBLUCSC | 483 | 4 | 29288 | island | 0.811894 | 0.966840 | Not queried | 2.24 | 5.71e-02 | 1.00e-01 |
|  | 5 | 178450457 | 178451176 | PDFSTATSENSEMBLUCSC | 720 | 9 | 33478 | island | 0.076743 | 0.309135 | ZNF879 (margin: ZNF879) | 2.24 | 3.99e-03 | 2.47e-02 |
|  | 3 | 16645732 | 16646058 | PDFSTATSENSEMBLUCSC | 327 | 4 | 27694 | gene;island | 0.902147 | 0.980250 | Not queried | 2.24 | 6.86e-01 | 6.99e-01 |
|  | 14 | 53620162 | 53620401 | PDFSTATSENSEMBLUCSC | 240 | 5 | 12403 | tss;island | 0.035873 | 0.183450 | Not queried | 2.24 | 2.22e-01 | 2.76e-01 |
|  | 10 | 101287609 | 101287846 | PDFSTATSENSEMBLUCSC | 238 | 5 | 6010 | island | 0.225175 | 0.843943 | Not queried | 2.24 | 4.21e-01 | 4.59e-01 |
|  | 10 | 18429643 | 18429760 | PDFSTATSENSEMBLUCSC | 118 | 6 | 4520 | gene;island | 0.733489 | 0.424320 | CACNB2 (margin: CACNB2) | 2.24 | 2.16e-03 | 1.45e-02 |
|  | 10 | 18429643 | 18429703 | PDFSTATSENSEMBLUCSC | 61 | 4 | 4972 | tss | 0.733489 | 0.378535 | Not queried | 2.24 | 2.86e-02 | 6.12e-02 |
|  | 3 | 194014481 | 194014928 | PDFSTATSENSEMBLUCSC | 448 | 6 | 29786 | island | 0.634651 | 0.946039 | Not queried | 2.24 | 6.49e-02 | 1.11e-01 |
|  | 7 | 102036828 | 102036991 | PDFSTATSENSEMBLUCSC | 164 | 4 | 37166 | gene | 0.003391 | 0.009430 | Not queried | 2.24 | 3.43e-01 | 3.84e-01 |
|  | 15 | 89764999 | 89765204 | PDFSTATSENSEMBLUCSC | 206 | 4 | 13884 | tss | 0.783105 | 0.923225 | Not queried | 2.24 | 1.14e-01 | 1.68e-01 |
|  | 10 | 118900324 | 118900492 | PDFSTATSENSEMBLUCSC | 169 | 4 | 6173 | island | 0.862183 | 0.979834 | Not queried | 2.24 | 2.00e-01 | 2.55e-01 |
|  | 8 | 72756812 | 72757004 | PDFSTATSENSEMBLUCSC | 193 | 7 | 39763 | tss | 0.785134 | 0.839415 | Not queried | 2.24 | 9.73e-02 | 1.55e-01 |
|  | 13 | 26625273 | 26625396 | PDFSTATSENSEMBLUCSC | 124 | 6 | 11352 | tss | 0.763754 | 0.394472 | Not queried | 2.24 | 9.31e-02 | 1.53e-01 |
|  | 1 | 34631324 | 34631613 | PDFSTATSENSEMBLUCSC | 290 | 4 | 1475 | tss;island | 0.389985 | 0.805358 | Not queried | 2.24 | 2.00e-01 | 2.55e-01 |
|  | 21 | 32931935 | 32932267 | PDFSTATSENSEMBLUCSC | 333 | 4 | 26505 | tss;island | 0.286206 | 0.050126 | Not queried | 2.24 | 1.14e-01 | 1.68e-01 |
|  | 7 | 124405366 | 124405760 | PDFSTATSENSEMBLUCSC | 395 | 6 | 38881 | island | 0.114591 | 0.034478 | Not queried | 2.24 | 2.60e-02 | 6.12e-02 |
|  | 1 | 153650884 | 153651325 | PDFSTATSENSEMBLUCSC | 442 | 10 | 3767 | island | 0.092802 | 0.449420 | NPR1 (margin: NPR1; ILF2) | 2.24 | 1.50e-03 | 1.41e-02 |
|  | 2 | 106681831 | 106681998 | PDFSTATSENSEMBLUCSC | 168 | 5 | 23556 | tss;island | 0.596412 | 0.910384 | Not queried | 2.24 | 1.51e-01 | 2.11e-01 |
|  | 11 | 89867637 | 89867809 | PDFSTATSENSEMBLUCSC | 173 | 5 | 7615 | tss | 0.708158 | 0.346774 | Not queried | 2.24 | 2.22e-01 | 2.76e-01 |
|  | 10 | 118956639 | 118956998 | PDFSTATSENSEMBLUCSC | 360 | 7 | 5390 | tss | 0.556312 | 0.801196 | Not queried | 2.24 | 2.62e-02 | 6.12e-02 |
|  | 5 | 66124395 | 66124801 | PDFSTATSENSEMBLUCSC | 407 | 7 | 31636 | gene | 0.865848 | 0.890382 | Not queried | 2.24 | 3.18e-01 | 3.74e-01 |
|  | 7 | 73038859 | 73039108 | PDFSTATSENSEMBLUCSC | 250 | 8 | 38561 | island | 0.113049 | 0.018195 | Not queried | 2.24 | 3.28e-01 | 3.84e-01 |
|  | 16 | 48530553 | 48530953 | PDFSTATSENSEMBLUCSC | 401 | 4 | 15904 | island | 0.914938 | 0.980431 | Not queried | 2.24 | 2.00e-01 | 2.55e-01 |
|  | 8 | 48675647 | 48676092 | PDFSTATSENSEMBLUCSC | 446 | 7 | 40273 | island | 0.892919 | 0.970268 | (margin: PRKDC) | 2.24 | 5.83e-04 | 6.47e-03 |
|  | 17 | 16593426 | 16593613 | PDFSTATSENSEMBLUCSC | 188 | 6 | 17161 | tss;island | 0.905840 | 0.688504 | Not queried | 2.23 | 1.32e-01 | 1.89e-01 |
|  | 20 | 42143080 | 42143502 | PDFSTATSENSEMBLUCSC | 423 | 7 | 25421 | gene | 0.933865 | 0.734805 | Not queried | 2.23 | 2.09e-01 | 2.65e-01 |
|  | 12 | 5018715 | 5019066 | PDFSTATSENSEMBLUCSC | 352 | 9 | 9502 | tss | 0.374879 | 0.698491 | Not queried | 2.23 | 2.97e-01 | 3.60e-01 |
|  | 17 | 72931569 | 72932048 | PDFSTATSENSEMBLUCSC | 480 | 12 | 18731 | island | 0.527000 | 0.861809 | OTOP2; OTOP3 (margin: OTOP2; OTOP3) | 2.23 | 2.01e-04 | 3.38e-03 |
|  | 12 | 49487944 | 49488311 | PDFSTATSENSEMBLUCSC | 368 | 4 | 9116 | gene;island | 0.464339 | 0.682237 | Not queried | 2.23 | 2.00e-01 | 2.55e-01 |
|  | 5 | 178651074 | 178651519 | PDFSTATSENSEMBLUCSC | 446 | 4 | 31930 | gene;island | 0.869688 | 0.969619 | Not queried | 2.23 | 5.71e-02 | 1.00e-01 |
|  | 4 | 1404128 | 1404607 | PDFSTATSENSEMBLUCSC | 480 | 4 | 30785 | island | 0.502355 | 0.169834 | Not queried | 2.23 | 3.43e-01 | 3.84e-01 |
|  | 6 | 167032331 | 167032502 | PDFSTATSENSEMBLUCSC | 172 | 4 | 34550 | gene;island | 0.694574 | 0.970582 | Not queried | 2.23 | 1.14e-01 | 1.68e-01 |
|  | 19 | 52391078 | 52391789 | PDFSTATSENSEMBLUCSC | 712 | 13 | 22344 | island | 0.968067 | 0.836599 | CTC-429C10.2; ZNF577; ZNF649 (margin: CTC-429C10.2; ZNF577; ZNF649) | 2.23 | 3.49e-03 | 2.20e-02 |
|  | 10 | 119000927 | 119001590 | PDFSTATSENSEMBLUCSC | 664 | 8 | 4778 | gene;island | 0.773323 | 0.411506 | SLC18A2; RP11-501J20.5 (margin: SLC18A2; RP11-501J20.5) | 2.23 | 1.09e-03 | 1.10e-02 |
|  | 8 | 63997977 | 63998463 | PDFSTATSENSEMBLUCSC | 487 | 5 | 39320 | gene | 0.366436 | 0.109755 | Not queried | 2.23 | 5.56e-02 | 1.00e-01 |
|  | 7 | 94536541 | 94536787 | PDFSTATSENSEMBLUCSC | 247 | 4 | 37722 | tss | 0.560939 | 0.847936 | Not queried | 2.23 | 4.86e-01 | 5.11e-01 |
|  | 3 | 186080015 | 186080359 | PDFSTATSENSEMBLUCSC | 345 | 8 | 29749 | island | 0.685560 | 0.939454 | Not queried | 2.23 | 2.81e-02 | 6.12e-02 |
|  | 6 | 31430641 | 31430954 | PDFSTATSENSEMBLUCSC | 314 | 11 | 34809 | tss | 0.007878 | 0.026531 | Not queried | 2.23 | 4.00e-02 | 8.03e-02 |
|  | 8 | 37593814 | 37594173 | PDFSTATSENSEMBLUCSC | 360 | 10 | 39645 | tss | 0.041096 | 0.018677 | Not queried | 2.23 | 2.32e-02 | 6.12e-02 |
|  | 7 | 134464242 | 134464411 | PDFSTATSENSEMBLUCSC | 170 | 4 | 37231 | gene | 0.082181 | 0.279125 | Not queried | 2.23 | 5.71e-02 | 1.00e-01 |
|  | 7 | 107204135 | 107204399 | PDFSTATSENSEMBLUCSC | 265 | 6 | 37825 | tss | 0.003782 | 0.018966 | Not queried | 2.23 | 4.11e-02 | 8.14e-02 |
|  | 7 | 107204135 | 107204720 | PDFSTATSENSEMBLUCSC | 586 | 8 | 37179 | gene;island | 0.003782 | 0.011112 | Not queried | 2.23 | 8.30e-02 | 1.40e-01 |
|  | 11 | 119227020 | 119227328 | PDFSTATSENSEMBLUCSC | 309 | 4 | 6938 | gene;island | 0.053602 | 0.013029 | Not queried | 2.23 | 2.00e-01 | 2.55e-01 |
|  | 17 | 6347867 | 6348200 | PDFSTATSENSEMBLUCSC | 334 | 6 | 16372 | gene | 0.252749 | 0.031690 | Not queried | 2.22 | 5.89e-01 | 6.11e-01 |
|  | 5 | 114632606 | 114632793 | PDFSTATSENSEMBLUCSC | 188 | 6 | 32226 | tss;island | 0.055623 | 0.019637 | Not queried | 2.22 | 1.32e-01 | 1.89e-01 |
|  | 22 | 45598886 | 45599149 | PDFSTATSENSEMBLUCSC | 264 | 4 | 26900 | gene;island | 0.893354 | 0.965042 | Not queried | 2.22 | 2.86e-02 | 6.12e-02 |
|  | 7 | 99595095 | 99595458 | PDFSTATSENSEMBLUCSC | 364 | 5 | 38742 | island | 0.640301 | 0.872295 | Not queried | 2.22 | 5.56e-02 | 1.00e-01 |
|  | 6 | 166722053 | 166722248 | PDFSTATSENSEMBLUCSC | 196 | 6 | 35335 | tss;island | 0.813898 | 0.951375 | Not queried | 2.22 | 4.11e-02 | 8.14e-02 |
|  | 11 | 124767840 | 124768015 | PDFSTATSENSEMBLUCSC | 176 | 5 | 7787 | tss | 0.977445 | 0.881685 | Not queried | 2.22 | 4.21e-01 | 4.59e-01 |
|  | 16 | 30406122 | 30406533 | PDFSTATSENSEMBLUCSC | 412 | 5 | 15183 | tss;island | 0.364095 | 0.074548 | Not queried | 2.22 | 6.90e-01 | 7.01e-01 |
|  | 17 | 56355299 | 56355431 | PDFSTATSENSEMBLUCSC | 133 | 4 | 16709 | gene;island | 0.801644 | 0.977858 | Not queried | 2.22 | 6.86e-01 | 6.99e-01 |
|  | 15 | 48623100 | 48623205 | PDFSTATSENSEMBLUCSC | 106 | 4 | 13655 | tss;island | 0.002951 | 0.004136 | Not queried | 2.22 | 6.86e-01 | 6.99e-01 |
|  | 17 | 73642404 | 73642628 | PDFSTATSENSEMBLUCSC | 225 | 6 | 17658 | tss | 0.747180 | 0.946208 | Not queried | 2.22 | 6.49e-02 | 1.11e-01 |
|  | 19 | 52839603 | 52840042 | PDFSTATSENSEMBLUCSC | 440 | 5 | 20193 | gene | 0.869347 | 0.588343 | Not queried | 2.22 | 1.59e-02 | 5.36e-02 |
|  | 5 | 178986620 | 178986906 | PDFSTATSENSEMBLUCSC | 287 | 5 | 31933 | gene;island | 0.048224 | 0.004943 | Not queried | 2.22 | 3.17e-02 | 6.50e-02 |
|  | 13 | 36788776 | 36789025 | PDFSTATSENSEMBLUCSC | 250 | 6 | 11392 | tss | 0.860980 | 0.972219 | Not queried | 2.22 | 1.52e-02 | 5.36e-02 |
|  | 8 | 37824306 | 37824529 | PDFSTATSENSEMBLUCSC | 224 | 4 | 39650 | tss;island | 0.544293 | 0.689184 | Not queried | 2.22 | 3.43e-01 | 3.84e-01 |
|  | 11 | 20620680 | 20620920 | PDFSTATSENSEMBLUCSC | 241 | 6 | 7199 | tss | 0.780060 | 0.895337 | Not queried | 2.22 | 5.89e-01 | 6.11e-01 |
|  | 1 | 205091232 | 205091505 | PDFSTATSENSEMBLUCSC | 274 | 8 | 2201 | tss | 0.001747 | 0.007471 | Not queried | 2.22 | 1.30e-01 | 1.89e-01 |
|  | 12 | 54475409 | 54475673 | PDFSTATSENSEMBLUCSC | 265 | 4 | 9164 | gene;island | 0.555454 | 0.808543 | Not queried | 2.22 | 2.86e-02 | 6.12e-02 |
|  | 6 | 169653905 | 169654035 | PDFSTATSENSEMBLUCSC | 131 | 7 | 34567 | gene | 0.746400 | 0.936860 | Not queried | 2.22 | 9.73e-02 | 1.55e-01 |
|  | 5 | 497397 | 497639 | PDFSTATSENSEMBLUCSC | 243 | 4 | 31508 | gene;island | 0.184360 | 0.578201 | Not queried | 2.22 | 5.71e-02 | 1.00e-01 |
|  | 13 | 100547728 | 100548078 | PDFSTATSENSEMBLUCSC | 351 | 5 | 11208 | gene;island | 0.920417 | 0.981869 | Not queried | 2.22 | 5.56e-02 | 1.00e-01 |
|  | 2 | 130345044 | 130345405 | PDFSTATSENSEMBLUCSC | 362 | 4 | 24712 | island | 0.776107 | 0.948608 | Not queried | 2.21 | 1.14e-01 | 1.68e-01 |
|  | 3 | 38206610 | 38206939 | PDFSTATSENSEMBLUCSC | 330 | 6 | 28227 | tss | 0.003774 | 0.005539 | Not queried | 2.21 | 1.80e-01 | 2.45e-01 |
|  | 5 | 693476 | 693638 | PDFSTATSENSEMBLUCSC | 163 | 4 | 32593 | island | 0.019404 | 0.003382 | Not queried | 2.21 | 3.43e-01 | 3.84e-01 |
|  | 15 | 74421523 | 74421584 | PDFSTATSENSEMBLUCSC | 62 | 4 | 13415 | gene | 0.872571 | 0.573417 | Not queried | 2.21 | 5.71e-02 | 1.00e-01 |
|  | 4 | 26492153 | 26492488 | PDFSTATSENSEMBLUCSC | 336 | 6 | 30337 | tss | 0.251101 | 0.594699 | Not queried | 2.21 | 4.85e-01 | 5.11e-01 |
|  | 1 | 167090972 | 167091161 | PDFSTATSENSEMBLUCSC | 190 | 4 | 873 | gene;island | 0.858678 | 0.958106 | Not queried | 2.21 | 2.00e-01 | 2.55e-01 |
|  | 3 | 99594893 | 99595254 | PDFSTATSENSEMBLUCSC | 362 | 6 | 27876 | gene;island | 0.149446 | 0.072860 | Not queried | 2.21 | 4.85e-01 | 5.11e-01 |
|  | 1 | 2398837 | 2399054 | PDFSTATSENSEMBLUCSC | 218 | 4 | 2504 | island | 0.277950 | 0.088265 | Not queried | 2.21 | 6.86e-01 | 6.99e-01 |
|  | 7 | 98245716 | 98246006 | PDFSTATSENSEMBLUCSC | 291 | 4 | 37747 | tss;island | 0.976246 | 0.872322 | Not queried | 2.21 | 3.43e-01 | 3.84e-01 |
|  | 4 | 40752692 | 40752947 | PDFSTATSENSEMBLUCSC | 256 | 4 | 29988 | gene;island | 0.523815 | 0.135735 | Not queried | 2.21 | 3.43e-01 | 3.84e-01 |
|  | 6 | 36410485 | 36410534 | PDFSTATSENSEMBLUCSC | 50 | 6 | 34937 | tss | 0.005317 | 0.016503 | Not queried | 2.21 | 2.60e-02 | 6.12e-02 |
|  | 1 | 33115703 | 33116289 | PDFSTATSENSEMBLUCSC | 587 | 5 | 1462 | tss;island | 0.014915 | 0.004746 | Not queried | 2.21 | 4.21e-01 | 4.59e-01 |
|  | 15 | 50979137 | 50979486 | PDFSTATSENSEMBLUCSC | 350 | 6 | 13670 | tss;island | 0.014115 | 0.036465 | RN7SL354P; TRPM7 (margin: RN7SL354P; TRPM7) | 2.21 | 4.33e-03 | 2.52e-02 |
|  | 7 | 569642 | 570041 | PDFSTATSENSEMBLUCSC | 400 | 5 | 38036 | island | 0.537994 | 0.726383 | Not queried | 2.21 | 3.10e-01 | 3.66e-01 |
|  | 12 | 119772354 | 119772421 | PDFSTATSENSEMBLUCSC | 68 | 4 | 10050 | tss | 0.327936 | 0.663480 | Not queried | 2.21 | 2.00e-01 | 2.55e-01 |
|  | 2 | 68882144 | 68882627 | PDFSTATSENSEMBLUCSC | 484 | 6 | 22716 | gene;island | 0.655450 | 0.846668 | Not queried | 2.21 | 1.80e-01 | 2.45e-01 |
|  | 4 | 156680207 | 156680518 | PDFSTATSENSEMBLUCSC | 312 | 4 | 30160 | gene | 0.813493 | 0.486365 | Not queried | 2.21 | 2.86e-02 | 6.12e-02 |
|  | 19 | 12946093 | 12946465 | PDFSTATSENSEMBLUCSC | 373 | 8 | 21625 | island | 0.350878 | 0.170168 | Not queried | 2.21 | 1.48e-02 | 5.36e-02 |
|  | 1 | 247694271 | 247694557 | PDFSTATSENSEMBLUCSC | 287 | 5 | 1127 | gene;tss;island | 0.914779 | 0.980201 | GCSAML; OR2C3 (margin: GCSAML; GCSAML-AS1; OR2C3) | 2.21 | 7.94e-03 | 3.19e-02 |
|  | 5 | 115152326 | 115152494 | PDFSTATSENSEMBLUCSC | 169 | 8 | 33027 | island | 0.848483 | 0.976807 | Not queried | 2.21 | 1.05e-01 | 1.66e-01 |
|  | 5 | 115152413 | 115152494 | PDFSTATSENSEMBLUCSC | 82 | 6 | 32230 | tss | 0.872611 | 0.976807 | Not queried | 2.21 | 3.10e-01 | 3.66e-01 |
|  | 11 | 85338771 | 85338971 | PDFSTATSENSEMBLUCSC | 201 | 4 | 7602 | tss;island | 0.004892 | 0.018551 | Not queried | 2.20 | 3.43e-01 | 3.84e-01 |
|  | 11 | 75111078 | 75111455 | PDFSTATSENSEMBLUCSC | 378 | 4 | 6833 | gene;island | 0.002481 | 0.008889 | Not queried | 2.20 | 2.00e-01 | 2.55e-01 |
|  | 12 | 130526916 | 130527212 | PDFSTATSENSEMBLUCSC | 297 | 8 | 10117 | tss | 0.221538 | 0.525592 | Not queried | 2.20 | 8.30e-02 | 1.40e-01 |
|  | 2 | 233322928 | 233324053 | PDFSTATSENSEMBLUCSC | 1126 | 10 | 23063 | gene;island | 0.526468 | 0.880602 | Not queried | 2.20 | 4.33e-02 | 8.55e-02 |
|  | 15 | 25513277 | 25513654 | PDFSTATSENSEMBLUCSC | 378 | 4 | 13536 | tss | 0.022901 | 0.049708 | Not queried | 2.20 | 6.86e-01 | 6.99e-01 |
|  | 1 | 6484616 | 6484980 | PDFSTATSENSEMBLUCSC | 365 | 4 | 2605 | island | 0.369578 | 0.176076 | Not queried | 2.20 | 3.43e-01 | 3.84e-01 |
|  | 16 | 31884589 | 31884761 | PDFSTATSENSEMBLUCSC | 173 | 4 | 15217 | tss;island | 0.941474 | 0.712363 | Not queried | 2.20 | 4.86e-01 | 5.11e-01 |
|  | 1 | 50886782 | 50886969 | PDFSTATSENSEMBLUCSC | 188 | 4 | 525 | gene;island | 0.834144 | 0.960209 | Not queried | 2.20 | 3.43e-01 | 3.84e-01 |
|  | 10 | 71811927 | 71812332 | PDFSTATSENSEMBLUCSC | 406 | 9 | 5129 | tss;island | 0.337988 | 0.079215 | Not queried | 2.20 | 5.03e-02 | 9.87e-02 |
|  | 10 | 15130387 | 15131036 | PDFSTATSENSEMBLUCSC | 650 | 10 | 5567 | island | 0.140784 | 0.008964 | DCLRE1CP1; ACBD7 (margin: RPP38; DCLRE1CP1; ACBD7; GAPDHP45; C10orf111) | 2.20 | 3.89e-03 | 2.43e-02 |
|  | 12 | 130526797 | 130527212 | PDFSTATSENSEMBLUCSC | 416 | 9 | 10997 | island | 0.287690 | 0.557008 | Not queried | 2.20 | 4.00e-02 | 8.03e-02 |
|  | 9 | 140115806 | 140116137 | PDFSTATSENSEMBLUCSC | 332 | 4 | 40858 | tss;island | 0.931503 | 0.742205 | Not queried | 2.20 | 5.71e-02 | 1.00e-01 |
|  | 13 | 100637113 | 100637258 | PDFSTATSENSEMBLUCSC | 146 | 4 | 11210 | gene;island | 0.910852 | 0.984640 | Not queried | 2.20 | 1.14e-01 | 1.68e-01 |
|  | 7 | 4153707 | 4153919 | PDFSTATSENSEMBLUCSC | 213 | 5 | 36827 | gene;island | 0.540472 | 0.884885 | Not queried | 2.20 | 5.56e-02 | 1.00e-01 |
|  | 2 | 1358513 | 1358730 | PDFSTATSENSEMBLUCSC | 218 | 4 | 22529 | gene | 0.720656 | 0.948092 | Not queried | 2.20 | 1.14e-01 | 1.68e-01 |
|  | 8 | 786658 | 787298 | PDFSTATSENSEMBLUCSC | 641 | 6 | 40006 | island | 0.735584 | 0.899049 | Not queried | 2.20 | 4.11e-02 | 8.14e-02 |
|  | 10 | 38691306 | 38691563 | PDFSTATSENSEMBLUCSC | 258 | 4 | 4561 | gene;island | 0.265736 | 0.069450 | Not queried | 2.20 | 2.00e-01 | 2.55e-01 |
|  | 17 | 1933006 | 1933471 | PDFSTATSENSEMBLUCSC | 466 | 10 | 17836 | island | 0.011162 | 0.016385 | Not queried | 2.20 | 1.65e-01 | 2.29e-01 |
|  | 1 | 99470550 | 99470967 | PDFSTATSENSEMBLUCSC | 418 | 7 | 1772 | tss;island | 0.906145 | 0.350320 | Not queried | 2.20 | 2.09e-01 | 2.65e-01 |
|  | 1 | 12538341 | 12538678 | PDFSTATSENSEMBLUCSC | 338 | 4 | 233 | gene | 0.003983 | 0.014721 | Not queried | 2.20 | 2.00e-01 | 2.55e-01 |
|  | 8 | 142984321 | 142984613 | PDFSTATSENSEMBLUCSC | 293 | 4 | 40625 | island | 0.732704 | 0.908343 | Not queried | 2.20 | 1.14e-01 | 1.68e-01 |
|  | 2 | 87088796 | 87088843 | PDFSTATSENSEMBLUCSC | 48 | 4 | 22773 | gene;island | 0.615567 | 0.917793 | Not queried | 2.20 | 2.00e-01 | 2.55e-01 |
|  | 6 | 30882994 | 30883203 | PDFSTATSENSEMBLUCSC | 210 | 5 | 33838 | gene;island | 0.954247 | 0.626257 | Not queried | 2.20 | 3.17e-02 | 6.50e-02 |
|  | 16 | 74641227 | 74641419 | PDFSTATSENSEMBLUCSC | 193 | 4 | 15354 | tss;island | 0.046522 | 0.021728 | Not queried | 2.20 | 4.86e-01 | 5.11e-01 |
|  | 2 | 242874841 | 242874987 | PDFSTATSENSEMBLUCSC | 147 | 4 | 25316 | island | 0.804095 | 0.966595 | Not queried | 2.20 | 1.14e-01 | 1.68e-01 |
|  | 11 | 86383182 | 86383940 | PDFSTATSENSEMBLUCSC | 759 | 20 | 8712 | island | 0.838721 | 0.963529 | ME3 (margin: ME3) | 2.20 | 2.90e-05 | 1.16e-03 |
|  | 11 | 130029478 | 130029684 | PDFSTATSENSEMBLUCSC | 207 | 5 | 8957 | island | 0.045989 | 0.017904 | Not queried | 2.20 | 1.51e-01 | 2.11e-01 |
|  | 17 | 57970021 | 57970287 | PDFSTATSENSEMBLUCSC | 267 | 6 | 16716 | gene | 0.001461 | 0.008115 | Not queried | 2.20 | 5.89e-01 | 6.11e-01 |
|  | 3 | 47620963 | 47621127 | PDFSTATSENSEMBLUCSC | 165 | 4 | 28289 | tss;island | 0.913949 | 0.556857 | Not queried | 2.20 | 1.14e-01 | 1.68e-01 |
|  | 14 | 35873967 | 35874353 | PDFSTATSENSEMBLUCSC | 387 | 7 | 12353 | tss;island | 0.002667 | 0.007799 | Not queried | 2.20 | 5.30e-02 | 1.00e-01 |
|  | 5 | 1212434 | 1212641 | PDFSTATSENSEMBLUCSC | 208 | 5 | 31526 | gene;island | 0.899383 | 0.970390 | SLC6A19 (margin: SLC6A19) | 2.20 | 7.94e-03 | 3.19e-02 |
|  | 4 | 166795260 | 166795913 | PDFSTATSENSEMBLUCSC | 654 | 9 | 30173 | gene;island | 0.475516 | 0.799892 | TLL1 (margin: TLL1) | 2.20 | 2.88e-04 | 4.58e-03 |
|  | 2 | 128433058 | 128433507 | PDFSTATSENSEMBLUCSC | 450 | 6 | 22863 | gene;tss;island | 0.208315 | 0.542582 | Not queried | 2.20 | 1.80e-01 | 2.45e-01 |
|  | 14 | 95240143 | 95240560 | PDFSTATSENSEMBLUCSC | 418 | 5 | 13108 | island | 0.862396 | 0.966350 | Not queried | 2.20 | 4.21e-01 | 4.59e-01 |
|  | 5 | 177412586 | 177412718 | PDFSTATSENSEMBLUCSC | 133 | 4 | 33462 | island | 0.728142 | 0.953736 | Not queried | 2.20 | 5.71e-02 | 1.00e-01 |
|  | 10 | 105037349 | 105037796 | PDFSTATSENSEMBLUCSC | 448 | 4 | 4740 | gene;island | 0.785956 | 0.982359 | Not queried | 2.20 | 2.00e-01 | 2.55e-01 |
|  | 19 | 55038263 | 55038476 | PDFSTATSENSEMBLUCSC | 214 | 4 | 22411 | island | 0.622712 | 0.879547 | Not queried | 2.20 | 5.71e-02 | 1.00e-01 |
|  | 11 | 14280742 | 14281053 | PDFSTATSENSEMBLUCSC | 312 | 4 | 6512 | gene;island | 0.967079 | 0.856817 | Not queried | 2.20 | 2.00e-01 | 2.55e-01 |
|  | 7 | 157691177 | 157691684 | PDFSTATSENSEMBLUCSC | 508 | 6 | 37351 | gene;island | 0.126412 | 0.456013 | Not queried | 2.20 | 2.40e-01 | 2.95e-01 |
|  | 12 | 130918514 | 130918994 | PDFSTATSENSEMBLUCSC | 481 | 5 | 9414 | gene | 0.403938 | 0.683915 | Not queried | 2.19 | 3.10e-01 | 3.66e-01 |
|  | 6 | 99291343 | 99291677 | PDFSTATSENSEMBLUCSC | 335 | 4 | 36346 | island | 0.586592 | 0.959332 | Not queried | 2.19 | 2.00e-01 | 2.55e-01 |
|  | 6 | 32190281 | 32190556 | PDFSTATSENSEMBLUCSC | 276 | 6 | 34100 | gene | 0.716652 | 0.903109 | Not queried | 2.19 | 6.49e-02 | 1.11e-01 |
|  | 19 | 11530021 | 11530065 | PDFSTATSENSEMBLUCSC | 45 | 4 | 20471 | tss | 0.944749 | 0.984149 | Not queried | 2.19 | 2.86e-02 | 6.12e-02 |
|  | 17 | 41910734 | 41910968 | PDFSTATSENSEMBLUCSC | 235 | 5 | 17394 | tss;island | 0.055648 | 0.008682 | Not queried | 2.19 | 5.48e-01 | 5.70e-01 |
|  | 20 | 62290277 | 62290499 | PDFSTATSENSEMBLUCSC | 223 | 4 | 25505 | gene;island | 0.021400 | 0.164679 | Not queried | 2.19 | 3.43e-01 | 3.84e-01 |
|  | 4 | 150999899 | 151000235 | PDFSTATSENSEMBLUCSC | 337 | 8 | 31361 | island | 0.028790 | 0.005907 | Not queried | 2.19 | 1.61e-01 | 2.24e-01 |
|  | 20 | 31331814 | 31332353 | PDFSTATSENSEMBLUCSC | 540 | 6 | 25676 | tss;island | 0.383538 | 0.197682 | Not queried | 2.19 | 2.40e-01 | 2.95e-01 |
|  | 13 | 26625089 | 26625396 | PDFSTATSENSEMBLUCSC | 308 | 7 | 11616 | island | 0.771714 | 0.403887 | Not queried | 2.19 | 2.09e-01 | 2.65e-01 |
|  | 21 | 33104864 | 33105032 | PDFSTATSENSEMBLUCSC | 169 | 4 | 26507 | tss;island | 0.003040 | 0.023642 | Not queried | 2.19 | 1.14e-01 | 1.68e-01 |
|  | 1 | 167599371 | 167599830 | PDFSTATSENSEMBLUCSC | 460 | 5 | 3947 | island | 0.882381 | 0.982408 | RCSD1; RP3-455J7.4 (margin: RCSD1; RP3-455J7.4) | 2.19 | 7.94e-03 | 3.19e-02 |
|  | 4 | 298681 | 299370 | PDFSTATSENSEMBLUCSC | 690 | 9 | 30715 | island | 0.642593 | 0.427964 | Not queried | 2.19 | 7.70e-02 | 1.30e-01 |
|  | 14 | 23564610 | 23564699 | PDFSTATSENSEMBLUCSC | 90 | 5 | 12295 | tss | 0.005924 | 0.015433 | Not queried | 2.19 | 2.22e-01 | 2.76e-01 |
|  | 17 | 61042718 | 61043072 | PDFSTATSENSEMBLUCSC | 355 | 4 | 18639 | island | 0.920405 | 0.793011 | Not queried | 2.19 | 2.86e-02 | 6.12e-02 |
|  | 2 | 27886066 | 27886333 | PDFSTATSENSEMBLUCSC | 268 | 5 | 23275 | tss | 0.002517 | 0.009673 | Not queried | 2.19 | 1.51e-01 | 2.11e-01 |
|  | 19 | 21688263 | 21688905 | PDFSTATSENSEMBLUCSC | 643 | 9 | 21836 | island | 0.269789 | 0.067796 | Not queried | 2.19 | 6.25e-02 | 1.09e-01 |
|  | 10 | 42970618 | 42971251 | PDFSTATSENSEMBLUCSC | 634 | 10 | 5681 | island | 0.385019 | 0.822617 | Not queried | 2.19 | 1.47e-02 | 5.36e-02 |
|  | 7 | 1083072 | 1083473 | PDFSTATSENSEMBLUCSC | 402 | 4 | 36740 | gene;island | 0.384234 | 0.846590 | Not queried | 2.19 | 5.71e-02 | 1.00e-01 |
|  | 3 | 129407365 | 129407548 | PDFSTATSENSEMBLUCSC | 184 | 5 | 27943 | gene | 0.970907 | 0.877302 | TMCC1 (margin: TMCC1) | 2.19 | 7.94e-03 | 3.19e-02 |
|  | 7 | 158264663 | 158264935 | PDFSTATSENSEMBLUCSC | 273 | 5 | 37374 | gene | 0.459292 | 0.872971 | Not queried | 2.19 | 9.52e-02 | 1.53e-01 |
|  | 11 | 72492171 | 72492538 | PDFSTATSENSEMBLUCSC | 368 | 4 | 6818 | gene;island | 0.094693 | 0.027249 | Not queried | 2.19 | 6.86e-01 | 6.99e-01 |
|  | 5 | 37835506 | 37836392 | PDFSTATSENSEMBLUCSC | 887 | 11 | 31591 | gene;tss;island | 0.931966 | 0.988828 | GDNF (margin: GDNF-AS1; GDNF) | 2.19 | 1.86e-03 | 1.45e-02 |
|  | 15 | 38857079 | 38857474 | PDFSTATSENSEMBLUCSC | 396 | 7 | 13575 | tss;island | 0.043429 | 0.173197 | Not queried | 2.19 | 5.35e-01 | 5.62e-01 |
|  | 17 | 72306008 | 72306215 | PDFSTATSENSEMBLUCSC | 208 | 4 | 16750 | gene | 0.396245 | 0.773660 | Not queried | 2.18 | 2.86e-02 | 6.12e-02 |
|  | 19 | 3029503 | 3029785 | PDFSTATSENSEMBLUCSC | 283 | 4 | 19702 | gene;tss;island | 0.449557 | 0.082368 | Not queried | 2.18 | 3.43e-01 | 3.84e-01 |
|  | 19 | 49926586 | 49927309 | PDFSTATSENSEMBLUCSC | 724 | 7 | 22264 | island | 0.585047 | 0.875260 | Not queried | 2.18 | 2.62e-02 | 6.12e-02 |
|  | 1 | 2259065 | 2259411 | PDFSTATSENSEMBLUCSC | 347 | 5 | 74 | gene;island | 0.866137 | 0.960595 | Not queried | 2.18 | 3.17e-02 | 6.50e-02 |
|  | 1 | 153919171 | 153919388 | PDFSTATSENSEMBLUCSC | 218 | 6 | 1955 | tss;island | 0.006709 | 0.016918 | Not queried | 2.18 | 1.52e-02 | 5.36e-02 |
|  | 5 | 140018644 | 140018944 | PDFSTATSENSEMBLUCSC | 301 | 9 | 32344 | tss | 0.004355 | 0.006279 | Not queried | 2.18 | 8.63e-01 | 8.70e-01 |
|  | 6 | 31794278 | 31794519 | PDFSTATSENSEMBLUCSC | 242 | 4 | 34840 | tss;island | 0.083732 | 0.304780 | Not queried | 2.18 | 4.86e-01 | 5.11e-01 |
|  | 2 | 209010890 | 209011053 | PDFSTATSENSEMBLUCSC | 164 | 5 | 23847 | tss | 0.861333 | 0.894536 | Not queried | 2.18 | 6.90e-01 | 7.01e-01 |
|  | 16 | 72042693 | 72042925 | PDFSTATSENSEMBLUCSC | 233 | 4 | 14834 | gene | 0.001249 | 0.005428 | Not queried | 2.18 | 1.14e-01 | 1.68e-01 |
|  | 18 | 35064900 | 35065386 | PDFSTATSENSEMBLUCSC | 487 | 4 | 19094 | gene;island | 0.733719 | 0.924546 | Not queried | 2.18 | 2.00e-01 | 2.55e-01 |
|  | 17 | 6616644 | 6617192 | PDFSTATSENSEMBLUCSC | 549 | 8 | 17909 | island | 0.867168 | 0.963489 | SLC13A5 (margin: SLC13A5) | 2.18 | 4.66e-03 | 2.68e-02 |
|  | 17 | 6616799 | 6617192 | PDFSTATSENSEMBLUCSC | 394 | 6 | 17051 | tss | 0.867168 | 0.956172 | Not queried | 2.18 | 1.52e-02 | 5.36e-02 |
|  | 10 | 112836471 | 112836785 | PDFSTATSENSEMBLUCSC | 315 | 7 | 5363 | tss;island | 0.075634 | 0.020133 | Not queried | 2.18 | 1.65e-01 | 2.28e-01 |
|  | 4 | 81307586 | 81307951 | PDFSTATSENSEMBLUCSC | 366 | 4 | 30037 | gene;island | 0.917216 | 0.726084 | Not queried | 2.18 | 2.00e-01 | 2.55e-01 |
|  | 19 | 50015511 | 50015532 | PDFSTATSENSEMBLUCSC | 22 | 4 | 20953 | tss;island | 0.450780 | 0.813654 | Not queried | 2.18 | 1.14e-01 | 1.68e-01 |
|  | 5 | 83017000 | 83017184 | PDFSTATSENSEMBLUCSC | 185 | 4 | 32160 | tss;island | 0.960640 | 0.989932 | Not queried | 2.18 | 5.71e-02 | 1.00e-01 |
|  | 4 | 175135048 | 175135997 | PDFSTATSENSEMBLUCSC | 950 | 9 | 31432 | island | 0.537164 | 0.851299 | Not queried | 2.18 | 6.25e-02 | 1.09e-01 |
|  | 6 | 32908466 | 32908794 | PDFSTATSENSEMBLUCSC | 329 | 6 | 34163 | gene | 0.009984 | 0.045460 | HLA-DMB; XXbac-BPG181M17.5 (margin: HLA-DMB; AL645941.1; XXbac-BPG181M17.5; HLA-DMA) | 2.18 | 4.33e-03 | 2.52e-02 |
|  | 12 | 91348999 | 91349273 | PDFSTATSENSEMBLUCSC | 275 | 7 | 9911 | tss;island | 0.804358 | 0.958496 | Not queried | 2.18 | 5.30e-02 | 1.00e-01 |
|  | 12 | 52241719 | 52242166 | PDFSTATSENSEMBLUCSC | 448 | 4 | 10430 | island | 0.476287 | 0.226226 | Not queried | 2.18 | 1.14e-01 | 1.68e-01 |
|  | 4 | 148401661 | 148402128 | PDFSTATSENSEMBLUCSC | 468 | 6 | 31354 | island | 0.714696 | 0.954705 | Not queried | 2.18 | 9.31e-02 | 1.53e-01 |
|  | 17 | 46507549 | 46507895 | PDFSTATSENSEMBLUCSC | 347 | 8 | 18472 | island | 0.823462 | 0.956090 | SKAP1 (margin: SKAP1) | 2.18 | 1.04e-02 | 3.96e-02 |
|  | 6 | 33143721 | 33143807 | PDFSTATSENSEMBLUCSC | 87 | 4 | 34204 | gene | 0.160799 | 0.567398 | Not queried | 2.18 | 2.00e-01 | 2.55e-01 |
|  | 1 | 201252200 | 201252734 | PDFSTATSENSEMBLUCSC | 535 | 8 | 4074 | island | 0.148940 | 0.027640 | Not queried | 2.18 | 3.79e-02 | 7.66e-02 |
|  | 1 | 201252200 | 201252510 | PDFSTATSENSEMBLUCSC | 311 | 6 | 2165 | tss | 0.117103 | 0.024533 | Not queried | 2.18 | 4.11e-02 | 8.14e-02 |
|  | 19 | 17861850 | 17862210 | PDFSTATSENSEMBLUCSC | 361 | 6 | 20599 | tss | 0.756593 | 0.971954 | Not queried | 2.18 | 3.10e-01 | 3.66e-01 |
|  | 21 | 46812996 | 46813298 | PDFSTATSENSEMBLUCSC | 303 | 4 | 26753 | island | 0.787791 | 0.942339 | Not queried | 2.18 | 1.14e-01 | 1.68e-01 |
|  | 4 | 1107495 | 1107720 | PDFSTATSENSEMBLUCSC | 226 | 5 | 30767 | island | 0.811457 | 0.980581 | Not queried | 2.18 | 9.52e-02 | 1.53e-01 |
|  | 16 | 82660596 | 82660873 | PDFSTATSENSEMBLUCSC | 278 | 5 | 14858 | gene | 0.184664 | 0.582719 | CDH13 (margin: CDH13) | 2.18 | 7.94e-03 | 3.19e-02 |
|  | 1 | 221052350 | 221052886 | PDFSTATSENSEMBLUCSC | 537 | 8 | 4221 | island | 0.346654 | 0.168880 | Not queried | 2.17 | 2.34e-01 | 2.90e-01 |
|  | 19 | 53073242 | 53073420 | PDFSTATSENSEMBLUCSC | 179 | 7 | 21016 | tss | 0.834115 | 0.477259 | ZNF701 (margin: ZNF808; ZNF701; RPL39P34; CTD-3099C6.7) | 2.17 | 4.08e-03 | 2.47e-02 |
|  | 2 | 55509105 | 55509779 | PDFSTATSENSEMBLUCSC | 675 | 8 | 24309 | island | 0.023315 | 0.080915 | Not queried | 2.17 | 1.30e-01 | 1.89e-01 |
|  | 8 | 37605552 | 37605978 | PDFSTATSENSEMBLUCSC | 427 | 5 | 39646 | tss | 0.930747 | 0.974906 | Not queried | 2.17 | 9.52e-02 | 1.53e-01 |
|  | 7 | 8480586 | 8481036 | PDFSTATSENSEMBLUCSC | 451 | 4 | 36891 | gene;island | 0.454835 | 0.679924 | Not queried | 2.17 | 6.86e-01 | 6.99e-01 |
|  | 6 | 41410759 | 41411128 | PDFSTATSENSEMBLUCSC | 370 | 4 | 36121 | island | 0.517588 | 0.253162 | Not queried | 2.17 | 2.86e-02 | 6.12e-02 |
|  | 22 | 39759848 | 39760165 | PDFSTATSENSEMBLUCSC | 318 | 7 | 27156 | tss | 0.863518 | 0.543612 | Not queried | 2.17 | 9.73e-02 | 1.55e-01 |
|  | 5 | 140778342 | 140778546 | PDFSTATSENSEMBLUCSC | 205 | 4 | 31830 | gene;island | 0.808343 | 0.978654 | Not queried | 2.17 | 1.14e-01 | 1.68e-01 |
|  | 16 | 66584061 | 66584358 | PDFSTATSENSEMBLUCSC | 298 | 4 | 15987 | island | 0.003787 | 0.023353 | Not queried | 2.17 | 3.43e-01 | 3.84e-01 |
|  | 20 | 3149249 | 3149495 | PDFSTATSENSEMBLUCSC | 247 | 6 | 25560 | tss;island | 0.690347 | 0.943638 | Not queried | 2.17 | 2.60e-02 | 6.12e-02 |
|  | 10 | 1228893 | 1229080 | PDFSTATSENSEMBLUCSC | 188 | 4 | 4457 | gene;island | 0.822054 | 0.964665 | Not queried | 2.17 | 2.86e-02 | 6.12e-02 |
|  | 11 | 60282712 | 60282864 | PDFSTATSENSEMBLUCSC | 153 | 6 | 7320 | tss | 0.670091 | 0.924770 | Not queried | 2.17 | 9.31e-02 | 1.53e-01 |
|  | 17 | 9808600 | 9808824 | PDFSTATSENSEMBLUCSC | 225 | 4 | 18019 | island | 0.294687 | 0.819899 | Not queried | 2.17 | 2.00e-01 | 2.55e-01 |
|  | 14 | 103388656 | 103389022 | PDFSTATSENSEMBLUCSC | 367 | 6 | 13180 | island | 0.781003 | 0.940857 | Not queried | 2.17 | 1.80e-01 | 2.45e-01 |
|  | 7 | 38350921 | 38351226 | PDFSTATSENSEMBLUCSC | 306 | 5 | 38422 | island | 0.828103 | 0.517927 | Not queried | 2.17 | 2.22e-01 | 2.76e-01 |
|  | 11 | 64067685 | 64067868 | PDFSTATSENSEMBLUCSC | 184 | 7 | 8403 | island | 0.782029 | 0.972317 | Not queried | 2.17 | 2.62e-02 | 6.12e-02 |
|  | 19 | 53496664 | 53497048 | PDFSTATSENSEMBLUCSC | 385 | 8 | 22367 | island | 0.712900 | 0.329570 | Not queried | 2.17 | 2.81e-02 | 6.12e-02 |
|  | 22 | 39759848 | 39760267 | PDFSTATSENSEMBLUCSC | 420 | 8 | 26878 | gene | 0.826071 | 0.507876 | Not queried | 2.17 | 1.30e-01 | 1.89e-01 |
|  | 19 | 41283407 | 41283667 | PDFSTATSENSEMBLUCSC | 261 | 4 | 20799 | tss;island | 0.001695 | 0.006895 | Not queried | 2.17 | 4.86e-01 | 5.11e-01 |
|  | 1 | 165910516 | 165910942 | PDFSTATSENSEMBLUCSC | 427 | 6 | 3934 | island | 0.756424 | 0.932449 | Not queried | 2.17 | 2.60e-02 | 6.12e-02 |
|  | 15 | 40674878 | 40674910 | PDFSTATSENSEMBLUCSC | 33 | 4 | 13588 | tss | 0.459974 | 0.265740 | Not queried | 2.17 | 3.43e-01 | 3.84e-01 |
|  | 2 | 139537745 | 139537845 | PDFSTATSENSEMBLUCSC | 101 | 4 | 24764 | island | 0.851410 | 0.527546 | Not queried | 2.17 | 2.86e-02 | 6.12e-02 |
|  | 16 | 57832005 | 57832309 | PDFSTATSENSEMBLUCSC | 305 | 4 | 15267 | tss;island | 0.480535 | 0.053006 | Not queried | 2.17 | 6.86e-01 | 6.99e-01 |
|  | 20 | 32319621 | 32319743 | PDFSTATSENSEMBLUCSC | 123 | 5 | 25686 | tss;island | 0.015826 | 0.006470 | Not queried | 2.17 | 4.21e-01 | 4.59e-01 |
|  | 16 | 84681507 | 84681729 | PDFSTATSENSEMBLUCSC | 223 | 4 | 15393 | tss;island | 0.915660 | 0.705096 | Not queried | 2.17 | 5.71e-02 | 1.00e-01 |
|  | 10 | 71333152 | 71333216 | PDFSTATSENSEMBLUCSC | 65 | 5 | 5126 | tss | 0.361784 | 0.094450 | NEUROG3 (margin: RP11-343J3.2; NEUROG3) | 2.17 | 7.94e-03 | 3.19e-02 |
|  | 10 | 99790438 | 99790910 | PDFSTATSENSEMBLUCSC | 473 | 5 | 6000 | island | 0.872728 | 0.973013 | CRTAC1 (margin: CRTAC1) | 2.17 | 7.94e-03 | 3.19e-02 |
|  | 12 | 130184591 | 130185016 | PDFSTATSENSEMBLUCSC | 426 | 4 | 9412 | gene;island | 0.470044 | 0.092859 | Not queried | 2.17 | 2.00e-01 | 2.55e-01 |
|  | 7 | 4030274 | 4030736 | PDFSTATSENSEMBLUCSC | 463 | 5 | 36824 | gene | 0.377377 | 0.909103 | Not queried | 2.17 | 5.56e-02 | 1.00e-01 |
|  | 17 | 17399718 | 17400111 | PDFSTATSENSEMBLUCSC | 394 | 7 | 17166 | tss | 0.009610 | 0.121437 | Not queried | 2.17 | 9.73e-02 | 1.55e-01 |
|  | 1 | 206729034 | 206729258 | PDFSTATSENSEMBLUCSC | 225 | 4 | 971 | gene;tss;island | 0.962952 | 0.849375 | Not queried | 2.16 | 2.00e-01 | 2.55e-01 |
|  | 2 | 11750735 | 11751071 | PDFSTATSENSEMBLUCSC | 337 | 4 | 22572 | gene;island | 0.915930 | 0.981859 | Not queried | 2.16 | 2.00e-01 | 2.55e-01 |
|  | 2 | 133426129 | 133426418 | PDFSTATSENSEMBLUCSC | 290 | 4 | 22878 | gene;island | 0.707330 | 0.888506 | Not queried | 2.16 | 2.86e-02 | 6.12e-02 |
|  | 15 | 52472201 | 52472389 | PDFSTATSENSEMBLUCSC | 189 | 4 | 13678 | tss | 0.013808 | 0.086654 | Not queried | 2.16 | 3.43e-01 | 3.84e-01 |
|  | 6 | 32410247 | 32410459 | PDFSTATSENSEMBLUCSC | 213 | 6 | 34113 | gene | 0.567911 | 0.879707 | Not queried | 2.16 | 4.85e-01 | 5.11e-01 |
|  | 6 | 26285766 | 26286262 | PDFSTATSENSEMBLUCSC | 497 | 9 | 34705 | tss | 0.002160 | 0.008856 | Not queried | 2.16 | 5.03e-02 | 9.87e-02 |
|  | 8 | 144601734 | 144601883 | PDFSTATSENSEMBLUCSC | 150 | 5 | 39480 | gene;island | 0.811810 | 0.976075 | Not queried | 2.16 | 5.56e-02 | 1.00e-01 |
|  | 19 | 38042009 | 38042154 | PDFSTATSENSEMBLUCSC | 146 | 5 | 20741 | tss;island | 0.353149 | 0.857792 | Not queried | 2.16 | 2.22e-01 | 2.76e-01 |
|  | 1 | 113261711 | 113261894 | PDFSTATSENSEMBLUCSC | 184 | 4 | 1840 | tss | 0.925042 | 0.763853 | Not queried | 2.16 | 3.43e-01 | 3.84e-01 |
|  | 6 | 169013150 | 169013766 | PDFSTATSENSEMBLUCSC | 617 | 7 | 34563 | gene | 0.838253 | 0.956806 | Not queried | 2.16 | 5.30e-02 | 1.00e-01 |
|  | 10 | 50747167 | 50747328 | PDFSTATSENSEMBLUCSC | 162 | 4 | 5072 | tss | 0.002523 | 0.008311 | Not queried | 2.16 | 6.86e-01 | 6.99e-01 |
|  | 6 | 32811884 | 32812708 | PDFSTATSENSEMBLUCSC | 825 | 32 | 34877 | tss | 0.007803 | 0.061036 | XXbac-BPG246D15.8; PSMB9; PSMB8; TAP1 (margin: XXbac-BPG246D15.8; PSMB9; TAP2; PSMB8; TAP1) | 2.16 | 5.20e-05 | 1.45e-03 |
|  | 19 | 49926748 | 49927309 | PDFSTATSENSEMBLUCSC | 562 | 6 | 20944 | tss | 0.596011 | 0.902024 | Not queried | 2.16 | 4.11e-02 | 8.14e-02 |
|  | 6 | 32057053 | 32057341 | PDFSTATSENSEMBLUCSC | 289 | 4 | 34042 | gene;island | 0.552124 | 0.800856 | Not queried | 2.16 | 6.86e-01 | 6.99e-01 |
|  | 8 | 145536028 | 145536348 | PDFSTATSENSEMBLUCSC | 321 | 4 | 39516 | gene | 0.788803 | 0.954563 | Not queried | 2.16 | 5.71e-02 | 1.00e-01 |
|  | 17 | 79374181 | 79374741 | PDFSTATSENSEMBLUCSC | 561 | 6 | 16906 | gene;island | 0.906444 | 0.986114 | Not queried | 2.16 | 1.52e-02 | 5.36e-02 |
|  | 10 | 64565642 | 64565772 | PDFSTATSENSEMBLUCSC | 131 | 4 | 4601 | gene;island | 0.007496 | 0.036053 | Not queried | 2.16 | 2.00e-01 | 2.55e-01 |
|  | 4 | 155547985 | 155548273 | PDFSTATSENSEMBLUCSC | 289 | 5 | 31383 | island | 0.147137 | 0.434336 | LRAT (margin: LRAT) | 2.16 | 7.94e-03 | 3.19e-02 |
|  | 3 | 56590940 | 56591125 | PDFSTATSENSEMBLUCSC | 186 | 5 | 28405 | tss | 0.001604 | 0.005145 | Not queried | 2.16 | 1.51e-01 | 2.11e-01 |
|  | 1 | 241520598 | 241520941 | PDFSTATSENSEMBLUCSC | 344 | 6 | 2349 | tss;island | 0.704181 | 0.946040 | Not queried | 2.16 | 1.32e-01 | 1.89e-01 |
|  | 5 | 140740387 | 140740607 | PDFSTATSENSEMBLUCSC | 221 | 4 | 31822 | gene;island | 0.683811 | 0.846348 | Not queried | 2.16 | 3.43e-01 | 3.84e-01 |
|  | 17 | 1028673 | 1028931 | PDFSTATSENSEMBLUCSC | 259 | 4 | 16309 | gene;island | 0.912694 | 0.978144 | Not queried | 2.16 | 1.14e-01 | 1.68e-01 |
|  | 6 | 30124647 | 30124942 | PDFSTATSENSEMBLUCSC | 296 | 7 | 33732 | gene | 0.978108 | 0.886455 | Not queried | 2.16 | 5.30e-02 | 1.00e-01 |
|  | 17 | 27332879 | 27333617 | PDFSTATSENSEMBLUCSC | 739 | 13 | 18163 | island | 0.511047 | 0.250518 | Not queried | 2.16 | 2.21e-02 | 6.12e-02 |
|  | 1 | 6304199 | 6304499 | PDFSTATSENSEMBLUCSC | 301 | 7 | 2599 | island | 0.797416 | 0.969913 | Not queried | 2.16 | 7.28e-02 | 1.23e-01 |
|  | 8 | 142452378 | 142452509 | PDFSTATSENSEMBLUCSC | 132 | 4 | 39454 | gene;island | 0.926641 | 0.982133 | Not queried | 2.16 | 2.00e-01 | 2.55e-01 |
|  | 1 | 55464653 | 55464927 | PDFSTATSENSEMBLUCSC | 275 | 4 | 551 | gene | 0.817204 | 0.890605 | Not queried | 2.16 | 6.86e-01 | 6.99e-01 |
|  | 19 | 7911120 | 7911362 | PDFSTATSENSEMBLUCSC | 243 | 5 | 20409 | tss | 0.775381 | 0.922051 | Not queried | 2.15 | 9.52e-02 | 1.53e-01 |
|  | 4 | 5021203 | 5021328 | PDFSTATSENSEMBLUCSC | 126 | 5 | 30272 | tss | 0.642237 | 0.942385 | CYTL1 (margin: CYTL1) | 2.15 | 7.94e-03 | 3.19e-02 |
|  | 12 | 14926572 | 14927542 | PDFSTATSENSEMBLUCSC | 971 | 13 | 10266 | island | 0.021060 | 0.014106 | Not queried | 2.15 | 1.69e-01 | 2.33e-01 |
|  | 17 | 77811499 | 77812240 | PDFSTATSENSEMBLUCSC | 742 | 6 | 16844 | gene;island | 0.025035 | 0.069435 | Not queried | 2.15 | 1.32e-01 | 1.89e-01 |
|  | 3 | 50374929 | 50375735 | PDFSTATSENSEMBLUCSC | 807 | 22 | 28351 | tss | 0.640294 | 0.049067 | Not queried | 2.15 | 4.40e-02 | 8.69e-02 |
|  | 4 | 41869086 | 41869611 | PDFSTATSENSEMBLUCSC | 526 | 7 | 31006 | island | 0.831710 | 0.956388 | Not queried | 2.15 | 9.73e-02 | 1.55e-01 |
|  | 6 | 26271716 | 26271827 | PDFSTATSENSEMBLUCSC | 112 | 4 | 34702 | tss | 0.001746 | 0.007561 | Not queried | 2.15 | 4.86e-01 | 5.11e-01 |
|  | 12 | 123319894 | 123320057 | PDFSTATSENSEMBLUCSC | 164 | 5 | 10934 | island | 0.003909 | 0.011312 | Not queried | 2.15 | 4.21e-01 | 4.59e-01 |
|  | 17 | 8089904 | 8090248 | PDFSTATSENSEMBLUCSC | 345 | 4 | 17994 | island | 0.001516 | 0.004062 | Not queried | 2.15 | 4.86e-01 | 5.11e-01 |
|  | 11 | 299390 | 299668 | PDFSTATSENSEMBLUCSC | 279 | 8 | 7838 | island | 0.645449 | 0.903712 | Not queried | 2.15 | 1.05e-01 | 1.66e-01 |
|  | 22 | 19712623 | 19712750 | PDFSTATSENSEMBLUCSC | 128 | 4 | 27302 | island | 0.753193 | 0.897978 | Not queried | 2.15 | 1.14e-01 | 1.68e-01 |
|  | 7 | 153749736 | 153749759 | PDFSTATSENSEMBLUCSC | 24 | 4 | 37302 | gene;tss;island | 0.831703 | 0.572515 | Not queried | 2.15 | 2.86e-02 | 6.12e-02 |
|  | 21 | 47064079 | 47064462 | PDFSTATSENSEMBLUCSC | 384 | 4 | 26762 | island | 0.415885 | 0.146944 | Not queried | 2.15 | 3.43e-01 | 3.84e-01 |
|  | 11 | 94883242 | 94883516 | PDFSTATSENSEMBLUCSC | 275 | 4 | 8746 | island | 0.970098 | 0.733881 | Not queried | 2.15 | 2.00e-01 | 2.55e-01 |
|  | 18 | 3261971 | 3262147 | PDFSTATSENSEMBLUCSC | 177 | 6 | 19160 | tss;island | 0.002662 | 0.010595 | Not queried | 2.15 | 1.32e-01 | 1.89e-01 |
|  | 16 | 419800 | 420230 | PDFSTATSENSEMBLUCSC | 431 | 5 | 14528 | gene;island | 0.591267 | 0.705768 | Not queried | 2.15 | 2.22e-01 | 2.76e-01 |
|  | 6 | 31238751 | 31239411 | PDFSTATSENSEMBLUCSC | 661 | 19 | 33866 | gene;island | 0.060114 | 0.416874 | Not queried | 2.15 | 2.15e-02 | 6.12e-02 |
|  | 10 | 71333037 | 71333216 | PDFSTATSENSEMBLUCSC | 180 | 6 | 5806 | island | 0.364495 | 0.103550 | Not queried | 2.15 | 6.49e-02 | 1.11e-01 |
|  | 1 | 74663927 | 74664160 | PDFSTATSENSEMBLUCSC | 234 | 4 | 584 | gene | 0.003279 | 0.012499 | Not queried | 2.15 | 2.00e-01 | 2.55e-01 |
|  | 19 | 11784246 | 11785337 | PDFSTATSENSEMBLUCSC | 1092 | 15 | 21580 | island | 0.690615 | 0.957947 | ZNF833P (margin: ZNF833P; CTC-499B15.4; HNRNPA1P10) | 2.15 | 4.94e-03 | 2.83e-02 |
|  | 11 | 119187772 | 119187879 | PDFSTATSENSEMBLUCSC | 108 | 5 | 8877 | island | 0.230179 | 0.054278 | Not queried | 2.15 | 4.21e-01 | 4.59e-01 |
|  | 10 | 5488225 | 5488535 | PDFSTATSENSEMBLUCSC | 311 | 7 | 4924 | tss | 0.008638 | 0.047671 | Not queried | 2.15 | 1.65e-01 | 2.28e-01 |
|  | 10 | 124895638 | 124895886 | PDFSTATSENSEMBLUCSC | 249 | 4 | 4802 | gene | 0.910786 | 0.978803 | Not queried | 2.15 | 1.14e-01 | 1.68e-01 |
|  | 19 | 40503048 | 40503358 | PDFSTATSENSEMBLUCSC | 311 | 4 | 20032 | gene | 0.497680 | 0.065756 | Not queried | 2.15 | 2.00e-01 | 2.55e-01 |
|  | 11 | 75046147 | 75046354 | PDFSTATSENSEMBLUCSC | 208 | 4 | 6832 | gene | 0.970852 | 0.887707 | Not queried | 2.15 | 5.71e-02 | 1.00e-01 |
|  | 20 | 52824106 | 52824483 | PDFSTATSENSEMBLUCSC | 378 | 8 | 25830 | tss | 0.014208 | 0.040791 | Not queried | 2.15 | 1.95e-01 | 2.55e-01 |
|  | 3 | 10547179 | 10547228 | PDFSTATSENSEMBLUCSC | 50 | 4 | 27663 | gene | 0.464040 | 0.174939 | Not queried | 2.15 | 2.86e-02 | 6.12e-02 |
|  | 3 | 195539297 | 195539463 | PDFSTATSENSEMBLUCSC | 167 | 5 | 28806 | tss | 0.924206 | 0.642073 | Not queried | 2.14 | 5.56e-02 | 1.00e-01 |
|  | 6 | 78173200 | 78173408 | PDFSTATSENSEMBLUCSC | 209 | 5 | 35078 | tss;island | 0.701024 | 0.948793 | Not queried | 2.14 | 5.56e-02 | 1.00e-01 |
|  | 13 | 114914537 | 114914682 | PDFSTATSENSEMBLUCSC | 146 | 4 | 11997 | island | 0.591902 | 0.884500 | Not queried | 2.14 | 3.43e-01 | 3.84e-01 |
|  | 4 | 186741788 | 186742139 | PDFSTATSENSEMBLUCSC | 352 | 5 | 30213 | gene | 0.929905 | 0.979111 | Not queried | 2.14 | 2.22e-01 | 2.76e-01 |
|  | 3 | 14597418 | 14597729 | PDFSTATSENSEMBLUCSC | 312 | 4 | 28913 | island | 0.077694 | 0.088279 | Not queried | 2.14 | 8.86e-01 | 8.89e-01 |
|  | 14 | 67708177 | 67708475 | PDFSTATSENSEMBLUCSC | 299 | 4 | 12117 | gene;island | 0.001528 | 0.004825 | Not queried | 2.14 | 2.00e-01 | 2.55e-01 |
|  | 14 | 51135030 | 51135466 | PDFSTATSENSEMBLUCSC | 437 | 7 | 12389 | tss | 0.002687 | 0.011630 | Not queried | 2.14 | 2.59e-01 | 3.16e-01 |
|  | 2 | 97303175 | 97303412 | PDFSTATSENSEMBLUCSC | 238 | 4 | 22783 | gene;island | 0.028582 | 0.125584 | Not queried | 2.14 | 5.71e-02 | 1.00e-01 |
|  | 3 | 178978849 | 178979013 | PDFSTATSENSEMBLUCSC | 165 | 5 | 28036 | gene;tss;island | 0.133275 | 0.019909 | Not queried | 2.14 | 1.51e-01 | 2.11e-01 |
|  | 10 | 102976327 | 102977342 | PDFSTATSENSEMBLUCSC | 1016 | 11 | 6056 | island | 0.880302 | 0.974015 | (margin: LBX1) | 2.14 | 1.40e-03 | 1.33e-02 |
|  | 14 | 23589066 | 23589419 | PDFSTATSENSEMBLUCSC | 354 | 4 | 12297 | tss | 0.656930 | 0.911027 | Not queried | 2.14 | 2.00e-01 | 2.55e-01 |
|  | 19 | 48897863 | 48898160 | PDFSTATSENSEMBLUCSC | 298 | 4 | 22214 | island | 0.325661 | 0.058255 | Not queried | 2.14 | 5.71e-02 | 1.00e-01 |
|  | 5 | 126366464 | 126366741 | PDFSTATSENSEMBLUCSC | 278 | 6 | 32254 | tss;island | 0.011412 | 0.005392 | Not queried | 2.14 | 2.40e-01 | 2.95e-01 |
|  | 6 | 31628050 | 31628440 | PDFSTATSENSEMBLUCSC | 391 | 15 | 33929 | gene | 0.002256 | 0.020221 | C6orf47-AS1; C6orf47; GPANK1 (margin: APOM; C6orf47-AS1; Y\_RNA; CSNK2B; CSNK2B-LY6G5B-1181; LY6G5B; BAG6; C6orf47; GPANK1) | 2.14 | 1.67e-03 | 1.45e-02 |
|  | 11 | 278394 | 278538 | PDFSTATSENSEMBLUCSC | 145 | 6 | 7006 | tss;island | 0.707090 | 0.958371 | Not queried | 2.14 | 9.31e-02 | 1.53e-01 |
|  | 7 | 42533001 | 42533414 | PDFSTATSENSEMBLUCSC | 414 | 5 | 38437 | island | 0.143560 | 0.042474 | Not queried | 2.14 | 2.22e-01 | 2.76e-01 |
|  | 5 | 3590820 | 3591171 | PDFSTATSENSEMBLUCSC | 352 | 4 | 32665 | island | 0.725497 | 0.435351 | Not queried | 2.14 | 3.43e-01 | 3.84e-01 |
|  | 1 | 17026786 | 17027073 | PDFSTATSENSEMBLUCSC | 288 | 4 | 261 | gene;island | 0.367893 | 0.633713 | Not queried | 2.14 | 5.71e-02 | 1.00e-01 |
|  | 5 | 1218507 | 1218653 | PDFSTATSENSEMBLUCSC | 147 | 4 | 31527 | gene;island | 0.777655 | 0.964607 | Not queried | 2.14 | 1.14e-01 | 1.68e-01 |
|  | 3 | 27410627 | 27411065 | PDFSTATSENSEMBLUCSC | 439 | 6 | 28946 | island | 0.071664 | 0.302942 | Not queried | 2.14 | 2.60e-02 | 6.12e-02 |
|  | 10 | 50821031 | 50821161 | PDFSTATSENSEMBLUCSC | 131 | 5 | 4585 | gene;tss;island | 0.010616 | 0.162093 | Not queried | 2.14 | 1.51e-01 | 2.11e-01 |
|  | 16 | 15489614 | 15489968 | PDFSTATSENSEMBLUCSC | 355 | 4 | 14674 | gene | 0.808080 | 0.933410 | Not queried | 2.13 | 6.86e-01 | 6.99e-01 |
|  | 3 | 10805956 | 10806569 | PDFSTATSENSEMBLUCSC | 614 | 11 | 28152 | tss | 0.754395 | 0.941563 | LINC00606 (margin: AC018495.3; LINC00606) | 2.13 | 1.40e-03 | 1.33e-02 |
|  | 17 | 34068044 | 34068571 | PDFSTATSENSEMBLUCSC | 528 | 4 | 16525 | gene;island | 0.525074 | 0.819124 | Not queried | 2.13 | 2.86e-02 | 6.12e-02 |
|  | 19 | 44455140 | 44455383 | PDFSTATSENSEMBLUCSC | 244 | 8 | 20837 | tss | 0.000759 | 0.012313 | Not queried | 2.13 | 1.61e-01 | 2.24e-01 |
|  | 22 | 39640835 | 39641154 | PDFSTATSENSEMBLUCSC | 320 | 7 | 27500 | island | 0.107085 | 0.027388 | PDGFB (margin: PDGFB) | 2.13 | 4.08e-03 | 2.47e-02 |
|  | 1 | 86622395 | 86622737 | PDFSTATSENSEMBLUCSC | 343 | 6 | 1725 | tss;island | 0.914811 | 0.962850 | Not queried | 2.13 | 1.32e-01 | 1.89e-01 |
|  | 16 | 6533549 | 6533789 | PDFSTATSENSEMBLUCSC | 241 | 4 | 14648 | gene;island | 0.196367 | 0.294013 | Not queried | 2.13 | 1.00e+00 | 1.00e+00 |
|  | 5 | 149493008 | 149493258 | PDFSTATSENSEMBLUCSC | 251 | 6 | 32424 | tss | 0.228379 | 0.817459 | Not queried | 2.13 | 9.31e-02 | 1.53e-01 |
|  | 10 | 134944446 | 134945099 | PDFSTATSENSEMBLUCSC | 654 | 6 | 4886 | gene;island | 0.219524 | 0.573079 | Not queried | 2.13 | 9.31e-02 | 1.53e-01 |
|  | 19 | 48216390 | 48216502 | PDFSTATSENSEMBLUCSC | 113 | 5 | 20908 | tss | 0.003416 | 0.008562 | Not queried | 2.13 | 1.51e-01 | 2.11e-01 |
|  | 14 | 54908185 | 54908353 | PDFSTATSENSEMBLUCSC | 169 | 6 | 12406 | tss | 0.002905 | 0.004137 | Not queried | 2.13 | 3.94e-01 | 4.36e-01 |
|  | 3 | 62861071 | 62861311 | PDFSTATSENSEMBLUCSC | 241 | 6 | 28428 | tss | 0.948421 | 0.567431 | Not queried | 2.13 | 1.32e-01 | 1.89e-01 |
|  | 16 | 979898 | 980146 | PDFSTATSENSEMBLUCSC | 249 | 4 | 14555 | gene;island | 0.623654 | 0.951495 | Not queried | 2.13 | 1.14e-01 | 1.68e-01 |
|  | 1 | 149858164 | 149858558 | PDFSTATSENSEMBLUCSC | 395 | 10 | 1898 | tss;island | 0.001759 | 0.008444 | Not queried | 2.13 | 8.92e-02 | 1.50e-01 |
|  | 12 | 40019768 | 40019919 | PDFSTATSENSEMBLUCSC | 152 | 4 | 9638 | tss | 0.921760 | 0.977839 | Not queried | 2.13 | 2.00e-01 | 2.55e-01 |
|  | 12 | 133179887 | 133180238 | PDFSTATSENSEMBLUCSC | 352 | 4 | 11065 | island | 0.693231 | 0.934068 | Not queried | 2.13 | 5.71e-02 | 1.00e-01 |
|  | 4 | 1027885 | 1028067 | PDFSTATSENSEMBLUCSC | 183 | 4 | 30760 | island | 0.909912 | 0.961686 | Not queried | 2.13 | 2.00e-01 | 2.55e-01 |
|  | 2 | 210010124 | 210010325 | PDFSTATSENSEMBLUCSC | 202 | 4 | 25051 | island | 0.544672 | 0.943293 | Not queried | 2.13 | 2.00e-01 | 2.55e-01 |
|  | 11 | 537287 | 537496 | PDFSTATSENSEMBLUCSC | 210 | 7 | 7015 | tss | 0.028408 | 0.012169 | Not queried | 2.13 | 1.28e-01 | 1.87e-01 |
|  | 3 | 46506104 | 46506554 | PDFSTATSENSEMBLUCSC | 451 | 10 | 29054 | island | 0.645979 | 0.865424 | Not queried | 2.13 | 8.92e-02 | 1.50e-01 |
|  | 3 | 46506404 | 46506554 | PDFSTATSENSEMBLUCSC | 151 | 6 | 28275 | tss | 0.520148 | 0.825696 | Not queried | 2.13 | 1.80e-01 | 2.45e-01 |
|  | 15 | 74218418 | 74218921 | PDFSTATSENSEMBLUCSC | 504 | 11 | 14292 | island | 0.803470 | 0.946542 | LOXL1; LOXL1-AS1 (margin: LOXL1; LOXL1-AS1) | 2.13 | 5.39e-05 | 1.47e-03 |
|  | 3 | 53529144 | 53529655 | PDFSTATSENSEMBLUCSC | 512 | 5 | 27829 | gene;island | 0.554989 | 0.081547 | Not queried | 2.13 | 1.51e-01 | 2.11e-01 |
|  | 7 | 153584582 | 153584873 | PDFSTATSENSEMBLUCSC | 292 | 6 | 37301 | gene | 0.964449 | 0.798693 | Not queried | 2.13 | 4.11e-02 | 8.14e-02 |
|  | 5 | 140753415 | 140753570 | PDFSTATSENSEMBLUCSC | 156 | 7 | 32377 | tss | 0.878023 | 0.965090 | Not queried | 2.13 | 1.28e-01 | 1.87e-01 |
|  | 5 | 157098338 | 157098555 | PDFSTATSENSEMBLUCSC | 218 | 6 | 32457 | tss | 0.896274 | 0.975185 | C5orf52; SOX30 (margin: C5orf52; SOX30; AC008694.3) | 2.13 | 8.66e-03 | 3.36e-02 |
|  | 5 | 157098338 | 157098619 | PDFSTATSENSEMBLUCSC | 282 | 7 | 33346 | island | 0.906247 | 0.978656 | C5orf52; SOX30 (margin: C5orf52; SOX30; AC008694.3) | 2.13 | 1.11e-02 | 4.14e-02 |
|  | 11 | 1221398 | 1221812 | PDFSTATSENSEMBLUCSC | 415 | 4 | 7906 | island | 0.359245 | 0.748667 | Not queried | 2.13 | 2.00e-01 | 2.55e-01 |
|  | 5 | 122434178 | 122434544 | PDFSTATSENSEMBLUCSC | 367 | 5 | 31731 | gene;island | 0.861130 | 0.968772 | Not queried | 2.13 | 2.22e-01 | 2.76e-01 |
|  | 12 | 6756377 | 6756815 | PDFSTATSENSEMBLUCSC | 439 | 6 | 10202 | island | 0.654980 | 0.352259 | Not queried | 2.13 | 6.49e-02 | 1.11e-01 |
|  | 7 | 100728343 | 100728792 | PDFSTATSENSEMBLUCSC | 450 | 7 | 38787 | island | 0.009002 | 0.042947 | Not queried | 2.13 | 7.28e-02 | 1.23e-01 |
|  | 1 | 115124302 | 115124491 | PDFSTATSENSEMBLUCSC | 190 | 7 | 1851 | tss | 0.004025 | 0.009512 | Not queried | 2.13 | 9.73e-02 | 1.55e-01 |
|  | 3 | 42543933 | 42544101 | PDFSTATSENSEMBLUCSC | 169 | 6 | 28243 | tss | 0.002526 | 0.007000 | Not queried | 2.13 | 1.80e-01 | 2.45e-01 |
|  | 12 | 8025394 | 8025646 | PDFSTATSENSEMBLUCSC | 253 | 7 | 10229 | island | 0.812173 | 0.949157 | Not queried | 2.13 | 1.28e-01 | 1.87e-01 |
|  | 5 | 149535646 | 149535765 | PDFSTATSENSEMBLUCSC | 120 | 4 | 32425 | tss | 0.570954 | 0.854948 | Not queried | 2.12 | 2.86e-02 | 6.12e-02 |
|  | 6 | 148663353 | 148663964 | PDFSTATSENSEMBLUCSC | 612 | 12 | 36550 | island | 0.044607 | 0.215989 | Not queried | 2.12 | 4.49e-02 | 8.87e-02 |
|  | 10 | 1517083 | 1517340 | PDFSTATSENSEMBLUCSC | 258 | 5 | 4467 | gene;island | 0.503546 | 0.607526 | Not queried | 2.12 | 5.48e-01 | 5.70e-01 |
|  | 7 | 99097871 | 99098103 | PDFSTATSENSEMBLUCSC | 233 | 7 | 38735 | island | 0.001615 | 0.009026 | Not queried | 2.12 | 9.73e-02 | 1.55e-01 |
|  | 10 | 72201417 | 72201741 | PDFSTATSENSEMBLUCSC | 325 | 7 | 5819 | island | 0.643156 | 0.841335 | Not queried | 2.12 | 1.28e-01 | 1.87e-01 |
|  | 8 | 2003810 | 2004050 | PDFSTATSENSEMBLUCSC | 241 | 5 | 39187 | gene;island | 0.832709 | 0.951500 | Not queried | 2.12 | 1.51e-01 | 2.11e-01 |
|  | 8 | 144852570 | 144853338 | PDFSTATSENSEMBLUCSC | 769 | 7 | 40687 | island | 0.847318 | 0.952405 | Not queried | 2.12 | 2.09e-01 | 2.65e-01 |
|  | 16 | 85319859 | 85320156 | PDFSTATSENSEMBLUCSC | 298 | 5 | 16152 | island | 0.606719 | 0.956479 | Not queried | 2.12 | 5.56e-02 | 1.00e-01 |
|  | 8 | 79427993 | 79428725 | PDFSTATSENSEMBLUCSC | 733 | 8 | 40406 | island | 0.221891 | 0.662897 | PKIA; RP11-594N15.2 (margin: PKIA; RP11-594N15.2) | 2.12 | 4.66e-03 | 2.68e-02 |
|  | 1 | 51425958 | 51426199 | PDFSTATSENSEMBLUCSC | 242 | 7 | 1617 | tss | 0.004808 | 0.000707 | Not queried | 2.12 | 2.59e-01 | 3.16e-01 |
|  | 11 | 71823834 | 71824165 | PDFSTATSENSEMBLUCSC | 332 | 8 | 7538 | tss | 0.005324 | 0.021190 | Not queried | 2.12 | 2.07e-02 | 6.12e-02 |
|  | 15 | 56286067 | 56286336 | PDFSTATSENSEMBLUCSC | 270 | 4 | 13686 | tss;island | 0.002518 | 0.013802 | Not queried | 2.12 | 3.43e-01 | 3.84e-01 |
|  | 1 | 16345293 | 16345491 | PDFSTATSENSEMBLUCSC | 199 | 4 | 1278 | tss | 0.616009 | 0.901205 | Not queried | 2.12 | 1.14e-01 | 1.68e-01 |
|  | 8 | 72987836 | 72988022 | PDFSTATSENSEMBLUCSC | 187 | 7 | 39765 | tss | 0.679745 | 0.506738 | Not queried | 2.12 | 7.28e-02 | 1.23e-01 |
|  | 2 | 232791732 | 232792015 | PDFSTATSENSEMBLUCSC | 284 | 5 | 23947 | tss;island | 0.863408 | 0.972700 | Not queried | 2.12 | 3.17e-02 | 6.50e-02 |
|  | 17 | 15165908 | 15166153 | PDFSTATSENSEMBLUCSC | 246 | 6 | 17149 | tss | 0.068811 | 0.015919 | RP11-849N15.1; PMP22 (margin: RP11-849N15.1; PMP22; MIR4731; AC005703.3) | 2.12 | 4.33e-03 | 2.52e-02 |
|  | 12 | 113592319 | 113592619 | PDFSTATSENSEMBLUCSC | 301 | 4 | 9321 | gene;island | 0.815650 | 0.972788 | Not queried | 2.11 | 3.43e-01 | 3.84e-01 |
|  | 17 | 76227227 | 76227681 | PDFSTATSENSEMBLUCSC | 455 | 7 | 18820 | island | 0.749918 | 0.859301 | Not queried | 2.11 | 1.65e-01 | 2.28e-01 |
|  | 1 | 202858335 | 202858430 | PDFSTATSENSEMBLUCSC | 96 | 6 | 2183 | tss | 0.001989 | 0.010282 | Not queried | 2.11 | 3.10e-01 | 3.66e-01 |
|  | 8 | 145700190 | 145700268 | PDFSTATSENSEMBLUCSC | 79 | 4 | 39525 | gene;island | 0.870456 | 0.959452 | Not queried | 2.11 | 2.00e-01 | 2.55e-01 |
|  | 14 | 106857188 | 106857655 | PDFSTATSENSEMBLUCSC | 468 | 4 | 13247 | island | 0.914229 | 0.971333 | Not queried | 2.11 | 1.14e-01 | 1.68e-01 |
|  | 19 | 51568260 | 51568523 | PDFSTATSENSEMBLUCSC | 264 | 4 | 22337 | island | 0.012094 | 0.037363 | Not queried | 2.11 | 1.14e-01 | 1.68e-01 |
|  | 16 | 56651283 | 56651456 | PDFSTATSENSEMBLUCSC | 174 | 4 | 15948 | island | 0.942655 | 0.980112 | Not queried | 2.11 | 5.71e-02 | 1.00e-01 |
|  | 6 | 44695195 | 44695536 | PDFSTATSENSEMBLUCSC | 342 | 5 | 36180 | island | 0.723883 | 0.951454 | Not queried | 2.11 | 3.17e-02 | 6.50e-02 |
|  | 1 | 26503623 | 26503979 | PDFSTATSENSEMBLUCSC | 357 | 8 | 1385 | tss | 0.110500 | 0.019926 | Not queried | 2.11 | 2.07e-02 | 6.12e-02 |
|  | 2 | 85839322 | 85839722 | PDFSTATSENSEMBLUCSC | 401 | 5 | 23486 | tss | 0.043256 | 0.293882 | Not queried | 2.11 | 1.51e-01 | 2.11e-01 |
|  | 19 | 35329860 | 35330243 | PDFSTATSENSEMBLUCSC | 384 | 4 | 21892 | island | 0.431695 | 0.797973 | Not queried | 2.11 | 2.00e-01 | 2.55e-01 |
|  | 6 | 31466722 | 31466859 | PDFSTATSENSEMBLUCSC | 138 | 5 | 33880 | gene;island | 0.490627 | 0.152863 | Not queried | 2.11 | 3.17e-02 | 6.50e-02 |
|  | 17 | 58754402 | 58755141 | PDFSTATSENSEMBLUCSC | 740 | 11 | 17555 | tss | 0.005184 | 0.012834 | Not queried | 2.11 | 2.81e-02 | 6.12e-02 |
|  | 2 | 27165540 | 27165781 | PDFSTATSENSEMBLUCSC | 242 | 4 | 22618 | gene | 0.324498 | 0.121411 | Not queried | 2.11 | 6.86e-01 | 6.99e-01 |
|  | 11 | 117747689 | 117748236 | PDFSTATSENSEMBLUCSC | 548 | 10 | 8852 | island | 0.658372 | 0.826358 | Not queried | 2.11 | 2.47e-01 | 3.03e-01 |
|  | 9 | 33001657 | 33002021 | PDFSTATSENSEMBLUCSC | 365 | 4 | 40807 | tss | 0.096971 | 0.010903 | Not queried | 2.11 | 8.86e-01 | 8.89e-01 |
|  | 7 | 27224092 | 27225897 | PDFSTATSENSEMBLUCSC | 1806 | 34 | 37499 | tss;island | 0.809211 | 0.952166 | HOXA11-AS; HOXA11; RP1-170O19.14 (margin: HOXA11-AS; RP1-170O19.20; HOXA10; HOXA11; RP1-170O19.14; HOXA13) | 2.11 | 3.93e-05 | 1.17e-03 |
|  | 6 | 29555799 | 29556415 | PDFSTATSENSEMBLUCSC | 617 | 8 | 33662 | gene | 0.229268 | 0.605849 | Not queried | 2.11 | 4.99e-02 | 9.81e-02 |
|  | 10 | 102729046 | 102729112 | PDFSTATSENSEMBLUCSC | 67 | 4 | 6038 | island | 0.121087 | 0.022592 | Not queried | 2.11 | 2.00e-01 | 2.55e-01 |
|  | 19 | 19626251 | 19626605 | PDFSTATSENSEMBLUCSC | 355 | 6 | 20642 | tss;island | 0.110193 | 0.390094 | Not queried | 2.11 | 1.32e-01 | 1.89e-01 |
|  | 1 | 27286944 | 27287541 | PDFSTATSENSEMBLUCSC | 598 | 6 | 1402 | tss | 0.022519 | 0.012606 | Not queried | 2.11 | 3.10e-01 | 3.66e-01 |
|  | 12 | 66582582 | 66582805 | PDFSTATSENSEMBLUCSC | 224 | 4 | 9848 | tss;island | 0.794467 | 0.964195 | Not queried | 2.11 | 5.71e-02 | 1.00e-01 |
|  | 14 | 77767644 | 77767800 | PDFSTATSENSEMBLUCSC | 157 | 4 | 12151 | gene | 0.832473 | 0.490316 | Not queried | 2.11 | 3.43e-01 | 3.84e-01 |
|  | 11 | 73881872 | 73882018 | PDFSTATSENSEMBLUCSC | 147 | 4 | 6825 | gene | 0.010601 | 0.026041 | Not queried | 2.11 | 6.86e-01 | 6.99e-01 |
|  | 8 | 63161579 | 63161676 | PDFSTATSENSEMBLUCSC | 98 | 5 | 39318 | gene;island | 0.813229 | 0.496320 | NKAIN3 (margin: NKAIN3) | 2.11 | 7.94e-03 | 3.19e-02 |
|  | 14 | 101192190 | 101193038 | PDFSTATSENSEMBLUCSC | 849 | 16 | 12607 | tss;island | 0.889818 | 0.967475 | DLK1 (margin: DLK1) | 2.11 | 6.53e-04 | 7.01e-03 |
|  | 2 | 180726247 | 180726697 | PDFSTATSENSEMBLUCSC | 451 | 8 | 23759 | tss;island | 0.520547 | 0.271477 | Not queried | 2.11 | 8.30e-02 | 1.40e-01 |
|  | 1 | 27961746 | 27962037 | PDFSTATSENSEMBLUCSC | 292 | 5 | 1416 | tss | 0.829915 | 0.821788 | Not queried | 2.11 | 6.90e-01 | 7.01e-01 |
|  | 14 | 45722617 | 45722745 | PDFSTATSENSEMBLUCSC | 129 | 6 | 12374 | tss | 0.004928 | 0.022172 | Not queried | 2.11 | 1.32e-01 | 1.89e-01 |
|  | 8 | 95653174 | 95653325 | PDFSTATSENSEMBLUCSC | 152 | 4 | 39818 | tss | 0.083737 | 0.031490 | Not queried | 2.11 | 5.71e-02 | 1.00e-01 |
|  | 15 | 79164541 | 79164980 | PDFSTATSENSEMBLUCSC | 440 | 4 | 13839 | tss;island | 0.925660 | 0.743142 | Not queried | 2.10 | 3.43e-01 | 3.84e-01 |
|  | 3 | 50374670 | 50375735 | PDFSTATSENSEMBLUCSC | 1066 | 25 | 27805 | gene;island | 0.505652 | 0.024569 | Not queried | 2.10 | 1.21e-01 | 1.78e-01 |
|  | 2 | 84517738 | 84517782 | PDFSTATSENSEMBLUCSC | 45 | 5 | 23474 | tss | 0.963425 | 0.670194 | Not queried | 2.10 | 3.17e-02 | 6.50e-02 |
|  | 2 | 84517738 | 84517950 | PDFSTATSENSEMBLUCSC | 213 | 7 | 24467 | island | 0.938745 | 0.670194 | Not queried | 2.10 | 5.30e-02 | 1.00e-01 |
|  | 5 | 1076587 | 1076892 | PDFSTATSENSEMBLUCSC | 306 | 5 | 31518 | gene;island | 0.732620 | 0.921229 | Not queried | 2.10 | 5.56e-02 | 1.00e-01 |
|  | 17 | 7589251 | 7589491 | PDFSTATSENSEMBLUCSC | 241 | 6 | 16409 | gene;tss;island | 0.002869 | 0.006488 | Not queried | 2.10 | 1.32e-01 | 1.89e-01 |
|  | 10 | 3823758 | 3824016 | PDFSTATSENSEMBLUCSC | 259 | 5 | 4475 | gene;island | 0.899679 | 0.981222 | KLF6 (margin: KLF6) | 2.10 | 7.94e-03 | 3.19e-02 |
|  | 1 | 66258022 | 66258134 | PDFSTATSENSEMBLUCSC | 113 | 4 | 1678 | tss;island | 0.792165 | 0.976773 | Not queried | 2.10 | 2.00e-01 | 2.55e-01 |
|  | 7 | 27291346 | 27291695 | PDFSTATSENSEMBLUCSC | 350 | 4 | 38367 | island | 0.418807 | 0.862642 | Not queried | 2.10 | 2.00e-01 | 2.55e-01 |
|  | 6 | 99790960 | 99791250 | PDFSTATSENSEMBLUCSC | 291 | 4 | 34422 | gene | 0.780759 | 0.953590 | Not queried | 2.10 | 2.86e-02 | 6.12e-02 |
|  | 1 | 90228963 | 90229095 | PDFSTATSENSEMBLUCSC | 133 | 4 | 3451 | island | 0.234000 | 0.472252 | Not queried | 2.10 | 2.00e-01 | 2.55e-01 |
|  | 13 | 46961656 | 46961897 | PDFSTATSENSEMBLUCSC | 242 | 9 | 11426 | tss | 0.107405 | 0.011957 | Not queried | 2.10 | 6.25e-02 | 1.09e-01 |
|  | 22 | 50708924 | 50709221 | PDFSTATSENSEMBLUCSC | 298 | 5 | 27253 | tss;island | 0.012956 | 0.010196 | Not queried | 2.10 | 5.48e-01 | 5.70e-01 |
|  | 19 | 35531639 | 35532097 | PDFSTATSENSEMBLUCSC | 459 | 5 | 19979 | gene;island | 0.716053 | 0.257760 | Not queried | 2.10 | 1.51e-01 | 2.11e-01 |
|  | 8 | 143333074 | 143333834 | PDFSTATSENSEMBLUCSC | 761 | 7 | 39455 | gene;island | 0.835721 | 0.956057 | Not queried | 2.10 | 1.65e-01 | 2.28e-01 |
|  | 3 | 55508379 | 55508686 | PDFSTATSENSEMBLUCSC | 308 | 4 | 27837 | gene;island | 0.806693 | 0.974855 | Not queried | 2.10 | 2.00e-01 | 2.55e-01 |
|  | 7 | 73894573 | 73895278 | PDFSTATSENSEMBLUCSC | 706 | 7 | 37044 | gene;island | 0.790658 | 0.948969 | GTF2IRD1 (margin: GTF2IRD1; RNA5SP233) | 2.10 | 5.83e-04 | 6.47e-03 |
|  | 6 | 31847922 | 31848099 | PDFSTATSENSEMBLUCSC | 178 | 4 | 33980 | gene;tss | 0.980060 | 0.893442 | Not queried | 2.10 | 2.86e-02 | 6.12e-02 |
|  | 22 | 36877697 | 36877737 | PDFSTATSENSEMBLUCSC | 41 | 4 | 27108 | tss | 0.004733 | 0.001796 | Not queried | 2.10 | 3.43e-01 | 3.84e-01 |
|  | 4 | 74864596 | 74864725 | PDFSTATSENSEMBLUCSC | 130 | 4 | 30432 | tss;island | 0.972511 | 0.911226 | Not queried | 2.10 | 3.43e-01 | 3.84e-01 |
|  | 19 | 49198900 | 49199217 | PDFSTATSENSEMBLUCSC | 318 | 4 | 20923 | tss | 0.646808 | 0.928195 | Not queried | 2.10 | 6.86e-01 | 6.99e-01 |
|  | 19 | 1355910 | 1356444 | PDFSTATSENSEMBLUCSC | 535 | 10 | 19652 | gene;island | 0.965568 | 0.725283 | Not queried | 2.10 | 5.24e-02 | 1.00e-01 |
|  | 1 | 67217673 | 67218275 | PDFSTATSENSEMBLUCSC | 603 | 12 | 3361 | island | 0.722334 | 0.957048 | Not queried | 2.10 | 7.80e-02 | 1.32e-01 |
|  | 1 | 67217673 | 67218135 | PDFSTATSENSEMBLUCSC | 463 | 10 | 1680 | tss | 0.722334 | 0.957048 | Not queried | 2.10 | 8.92e-02 | 1.50e-01 |
|  | 18 | 23713407 | 23714084 | PDFSTATSENSEMBLUCSC | 678 | 10 | 19440 | island | 0.674434 | 0.977345 | Not queried | 2.10 | 1.05e-01 | 1.66e-01 |
|  | 18 | 23713407 | 23713741 | PDFSTATSENSEMBLUCSC | 335 | 6 | 19215 | tss | 0.674434 | 0.933631 | Not queried | 2.10 | 3.10e-01 | 3.66e-01 |
|  | 12 | 48166716 | 48166813 | PDFSTATSENSEMBLUCSC | 98 | 4 | 9663 | tss | 0.028174 | 0.007043 | Not queried | 2.10 | 1.14e-01 | 1.68e-01 |
|  | 17 | 33775295 | 33776116 | PDFSTATSENSEMBLUCSC | 822 | 12 | 18214 | island | 0.648494 | 0.953507 | SLFN13 (margin: SLFN13; RP11-1094M14.7) | 2.10 | 2.91e-03 | 1.88e-02 |
|  | 6 | 33169581 | 33169731 | PDFSTATSENSEMBLUCSC | 151 | 4 | 34222 | gene;tss;island | 0.933814 | 0.803490 | Not queried | 2.10 | 1.14e-01 | 1.68e-01 |
|  | 4 | 69215675 | 69216111 | PDFSTATSENSEMBLUCSC | 437 | 14 | 31083 | island | 0.004988 | 0.008300 | Not queried | 2.10 | 3.76e-01 | 4.21e-01 |
|  | 4 | 7070632 | 7070879 | PDFSTATSENSEMBLUCSC | 248 | 4 | 30291 | tss;island | 0.401168 | 0.130547 | Not queried | 2.10 | 6.86e-01 | 6.99e-01 |
|  | 1 | 183385831 | 183386237 | PDFSTATSENSEMBLUCSC | 407 | 4 | 915 | gene;island | 0.768985 | 0.944014 | Not queried | 2.10 | 3.43e-01 | 3.84e-01 |
|  | 7 | 4838902 | 4839249 | PDFSTATSENSEMBLUCSC | 348 | 5 | 36856 | gene | 0.955621 | 0.978821 | Not queried | 2.10 | 5.56e-02 | 1.00e-01 |
|  | 11 | 118781408 | 118781978 | PDFSTATSENSEMBLUCSC | 571 | 10 | 8862 | island | 0.015991 | 0.084819 | Not queried | 2.10 | 2.88e-02 | 6.16e-02 |
|  | 6 | 31707744 | 31708299 | PDFSTATSENSEMBLUCSC | 556 | 19 | 33949 | gene | 0.074162 | 0.025138 | Not queried | 2.10 | 1.68e-02 | 5.66e-02 |
|  | 5 | 63257499 | 63257941 | PDFSTATSENSEMBLUCSC | 443 | 10 | 32827 | island | 0.850736 | 0.489936 | Not queried | 2.10 | 2.88e-02 | 6.16e-02 |
|  | 5 | 72594461 | 72594851 | PDFSTATSENSEMBLUCSC | 391 | 4 | 32868 | island | 0.977532 | 0.887669 | Not queried | 2.10 | 6.86e-01 | 6.99e-01 |
|  | 22 | 30868060 | 30868226 | PDFSTATSENSEMBLUCSC | 167 | 4 | 27063 | tss | 0.576016 | 0.817592 | Not queried | 2.09 | 4.86e-01 | 5.11e-01 |
|  | 7 | 150652948 | 150653195 | PDFSTATSENSEMBLUCSC | 248 | 5 | 37984 | tss | 0.876624 | 0.976113 | Not queried | 2.09 | 2.22e-01 | 2.76e-01 |
|  | 10 | 133058857 | 133058988 | PDFSTATSENSEMBLUCSC | 132 | 4 | 4844 | gene;island | 0.436563 | 0.653055 | Not queried | 2.09 | 3.43e-01 | 3.84e-01 |
|  | 2 | 102090791 | 102091321 | PDFSTATSENSEMBLUCSC | 531 | 11 | 24571 | island | 0.706319 | 0.892861 | Not queried | 2.09 | 3.32e-01 | 3.84e-01 |
|  | 11 | 63997306 | 63997740 | PDFSTATSENSEMBLUCSC | 435 | 7 | 7387 | tss;island | 0.359116 | 0.147188 | Not queried | 2.09 | 3.18e-01 | 3.74e-01 |
|  | 8 | 124780287 | 124780424 | PDFSTATSENSEMBLUCSC | 138 | 4 | 39902 | tss;island | 0.034500 | 0.007108 | Not queried | 2.09 | 3.43e-01 | 3.84e-01 |
|  | 7 | 107300939 | 107301288 | PDFSTATSENSEMBLUCSC | 350 | 7 | 37182 | gene;island | 0.643495 | 0.898955 | Not queried | 2.09 | 1.28e-01 | 1.87e-01 |
|  | 8 | 95653174 | 95653368 | PDFSTATSENSEMBLUCSC | 195 | 5 | 40460 | island | 0.081542 | 0.021799 | Not queried | 2.09 | 2.22e-01 | 2.76e-01 |
|  | 5 | 74349644 | 74350214 | PDFSTATSENSEMBLUCSC | 571 | 5 | 32882 | island | 0.934651 | 0.977661 | Not queried | 2.09 | 3.10e-01 | 3.66e-01 |
|  | 1 | 33816020 | 33816159 | PDFSTATSENSEMBLUCSC | 140 | 5 | 404 | gene;tss;island | 0.039281 | 0.006741 | Not queried | 2.09 | 3.17e-02 | 6.50e-02 |
|  | 19 | 17633880 | 17634108 | PDFSTATSENSEMBLUCSC | 229 | 4 | 20594 | tss | 0.562261 | 0.890015 | Not queried | 2.09 | 2.00e-01 | 2.55e-01 |
|  | 14 | 61119122 | 61119419 | PDFSTATSENSEMBLUCSC | 298 | 4 | 12909 | island | 0.883670 | 0.974374 | Not queried | 2.09 | 5.71e-02 | 1.00e-01 |
|  | 7 | 23720292 | 23720838 | PDFSTATSENSEMBLUCSC | 547 | 6 | 36920 | gene;island | 0.616283 | 0.309860 | FAM221A; AC006026.13 (margin: FAM221A; AC006026.1; AC006026.13) | 2.09 | 2.16e-03 | 1.45e-02 |
|  | 10 | 70319998 | 70320073 | PDFSTATSENSEMBLUCSC | 76 | 5 | 5114 | tss | 0.059154 | 0.025897 | Not queried | 2.09 | 9.52e-02 | 1.53e-01 |
|  | 11 | 17565744 | 17565885 | PDFSTATSENSEMBLUCSC | 142 | 4 | 6525 | gene | 0.615152 | 0.364672 | Not queried | 2.09 | 4.86e-01 | 5.11e-01 |
|  | 7 | 19157634 | 19158747 | PDFSTATSENSEMBLUCSC | 1114 | 22 | 37460 | tss;island | 0.749993 | 0.481984 | Not queried | 2.09 | 3.69e-02 | 7.52e-02 |
|  | 5 | 56110761 | 56110881 | PDFSTATSENSEMBLUCSC | 121 | 5 | 32072 | tss | 0.001198 | 0.004640 | Not queried | 2.09 | 5.48e-01 | 5.70e-01 |
|  | 6 | 32139504 | 32139828 | PDFSTATSENSEMBLUCSC | 325 | 4 | 34069 | gene | 0.739282 | 0.976749 | Not queried | 2.09 | 3.43e-01 | 3.84e-01 |
|  | 2 | 54952078 | 54952487 | PDFSTATSENSEMBLUCSC | 410 | 5 | 24304 | island | 0.758773 | 0.437226 | Not queried | 2.08 | 3.17e-02 | 6.50e-02 |
|  | 3 | 147077127 | 147077774 | PDFSTATSENSEMBLUCSC | 648 | 9 | 29565 | island | 0.584780 | 0.886516 | Not queried | 2.08 | 4.00e-02 | 8.03e-02 |
|  | 11 | 118436807 | 118437133 | PDFSTATSENSEMBLUCSC | 327 | 9 | 7735 | tss | 0.005899 | 0.002597 | Not queried | 2.08 | 7.96e-01 | 8.06e-01 |
|  | 12 | 109729478 | 109729809 | PDFSTATSENSEMBLUCSC | 332 | 4 | 9292 | gene;island | 0.080986 | 0.267895 | Not queried | 2.08 | 4.86e-01 | 5.11e-01 |
|  | 5 | 72598865 | 72599228 | PDFSTATSENSEMBLUCSC | 364 | 4 | 32871 | island | 0.817339 | 0.979132 | Not queried | 2.08 | 1.14e-01 | 1.68e-01 |
|  | 11 | 82612407 | 82612887 | PDFSTATSENSEMBLUCSC | 481 | 10 | 7595 | tss;island | 0.003101 | 0.008224 | Not queried | 2.08 | 6.30e-02 | 1.10e-01 |
|  | 17 | 79495268 | 79495348 | PDFSTATSENSEMBLUCSC | 81 | 5 | 17740 | tss | 0.379580 | 0.682061 | RP13-766D20.2; FSCN2 (margin: RP13-766D20.2; FSCN2; ACTG1; C17orf70) | 2.08 | 7.94e-03 | 3.19e-02 |
|  | 17 | 79495268 | 79495664 | PDFSTATSENSEMBLUCSC | 397 | 7 | 18927 | island | 0.384087 | 0.682061 | Not queried | 2.08 | 7.28e-02 | 1.23e-01 |
|  | 19 | 50789808 | 50789973 | PDFSTATSENSEMBLUCSC | 166 | 4 | 20172 | gene;island | 0.924406 | 0.981635 | Not queried | 2.08 | 2.86e-02 | 6.12e-02 |
|  | 14 | 100842311 | 100843067 | PDFSTATSENSEMBLUCSC | 757 | 9 | 12207 | gene;tss;island | 0.003238 | 0.005670 | Not queried | 2.08 | 6.25e-02 | 1.09e-01 |
|  | 1 | 92545580 | 92545768 | PDFSTATSENSEMBLUCSC | 189 | 4 | 1747 | tss | 0.097018 | 0.198170 | Not queried | 2.08 | 2.00e-01 | 2.55e-01 |
|  | 15 | 51387571 | 51387921 | PDFSTATSENSEMBLUCSC | 351 | 5 | 13349 | gene;island | 0.956137 | 0.820569 | Not queried | 2.08 | 9.52e-02 | 1.53e-01 |
|  | 3 | 71113948 | 71114335 | PDFSTATSENSEMBLUCSC | 388 | 5 | 27857 | gene;island | 0.296817 | 0.034962 | Not queried | 2.08 | 1.59e-02 | 5.36e-02 |
|  | 11 | 93822022 | 93822294 | PDFSTATSENSEMBLUCSC | 273 | 4 | 6863 | gene;island | 0.857786 | 0.979286 | Not queried | 2.08 | 1.14e-01 | 1.68e-01 |
|  | 2 | 132919375 | 132919865 | PDFSTATSENSEMBLUCSC | 491 | 6 | 22873 | gene | 0.534105 | 0.826178 | Not queried | 2.08 | 2.60e-02 | 6.12e-02 |
|  | 17 | 7909776 | 7910040 | PDFSTATSENSEMBLUCSC | 265 | 4 | 16423 | gene;island | 0.908922 | 0.982378 | Not queried | 2.08 | 2.00e-01 | 2.55e-01 |
|  | 17 | 28903436 | 28903809 | PDFSTATSENSEMBLUCSC | 374 | 5 | 18178 | island | 0.696153 | 0.850824 | Not queried | 2.08 | 5.48e-01 | 5.70e-01 |
|  | 10 | 1451381 | 1451648 | PDFSTATSENSEMBLUCSC | 268 | 5 | 4464 | gene | 0.857581 | 0.962395 | Not queried | 2.08 | 9.52e-02 | 1.53e-01 |
|  | 2 | 176961552 | 176961907 | PDFSTATSENSEMBLUCSC | 356 | 4 | 24892 | island | 0.039866 | 0.008807 | Not queried | 2.08 | 1.14e-01 | 1.68e-01 |
|  | 8 | 41519026 | 41519399 | PDFSTATSENSEMBLUCSC | 374 | 4 | 39290 | gene;tss | 0.803794 | 0.969788 | Not queried | 2.08 | 5.71e-02 | 1.00e-01 |
|  | 6 | 10695206 | 10695389 | PDFSTATSENSEMBLUCSC | 184 | 5 | 33565 | gene | 0.001930 | 0.008032 | Not queried | 2.07 | 4.21e-01 | 4.59e-01 |
|  | 7 | 150755072 | 150755981 | PDFSTATSENSEMBLUCSC | 910 | 20 | 37987 | tss | 0.012987 | 0.050783 | SLC4A2; CDK5 (margin: ABCB8; ASIC3; SLC4A2; CDK5) | 2.07 | 5.62e-03 | 3.18e-02 |
|  | 20 | 33460474 | 33460865 | PDFSTATSENSEMBLUCSC | 392 | 12 | 26116 | island | 0.025629 | 0.009457 | Not queried | 2.07 | 1.73e-02 | 5.79e-02 |
|  | 1 | 183774114 | 183774526 | PDFSTATSENSEMBLUCSC | 413 | 4 | 919 | gene;island | 0.083873 | 0.025796 | Not queried | 2.07 | 2.00e-01 | 2.55e-01 |
|  | 4 | 122872996 | 122873181 | PDFSTATSENSEMBLUCSC | 186 | 4 | 30578 | tss | 0.719465 | 0.443024 | Not queried | 2.07 | 2.86e-02 | 6.12e-02 |
|  | 7 | 155166205 | 155166619 | PDFSTATSENSEMBLUCSC | 415 | 5 | 39063 | island | 0.935959 | 0.869024 | Not queried | 2.07 | 5.48e-01 | 5.70e-01 |
|  | 19 | 12444541 | 12444910 | PDFSTATSENSEMBLUCSC | 370 | 8 | 20493 | tss | 0.012658 | 0.007504 | Not queried | 2.07 | 3.82e-01 | 4.27e-01 |
|  | 16 | 75150744 | 75150880 | PDFSTATSENSEMBLUCSC | 137 | 6 | 15358 | tss | 0.515697 | 0.221894 | Not queried | 2.07 | 1.80e-01 | 2.45e-01 |
|  | 12 | 108955167 | 108955348 | PDFSTATSENSEMBLUCSC | 182 | 6 | 9982 | tss;island | 0.000418 | 0.000918 | Not queried | 2.07 | 3.94e-01 | 4.36e-01 |
|  | 19 | 58864846 | 58864929 | PDFSTATSENSEMBLUCSC | 84 | 4 | 20261 | gene;island | 0.833576 | 0.962690 | Not queried | 2.07 | 2.86e-02 | 6.12e-02 |
|  | 12 | 121890163 | 121890503 | PDFSTATSENSEMBLUCSC | 341 | 4 | 9368 | gene;island | 0.185316 | 0.487416 | Not queried | 2.07 | 1.14e-01 | 1.68e-01 |
|  | 17 | 45949677 | 45949878 | PDFSTATSENSEMBLUCSC | 202 | 5 | 18464 | island | 0.916531 | 0.970023 | Not queried | 2.07 | 1.51e-01 | 2.11e-01 |
|  | 11 | 73498584 | 73499134 | PDFSTATSENSEMBLUCSC | 551 | 5 | 8644 | island | 0.008289 | 0.023421 | Not queried | 2.07 | 4.21e-01 | 4.59e-01 |
|  | 12 | 128899735 | 128899917 | PDFSTATSENSEMBLUCSC | 183 | 4 | 9407 | gene;island | 0.465174 | 0.172306 | Not queried | 2.07 | 2.86e-02 | 6.12e-02 |
|  | 13 | 24152779 | 24153266 | PDFSTATSENSEMBLUCSC | 488 | 4 | 11102 | gene;tss;island | 0.078840 | 0.016406 | Not queried | 2.07 | 4.86e-01 | 5.11e-01 |
|  | 10 | 37413610 | 37413799 | PDFSTATSENSEMBLUCSC | 190 | 4 | 5030 | tss;island | 0.638854 | 0.921497 | Not queried | 2.07 | 5.71e-02 | 1.00e-01 |
|  | 13 | 48611439 | 48612050 | PDFSTATSENSEMBLUCSC | 612 | 13 | 11720 | island | 0.001576 | 0.003124 | Not queried | 2.07 | 1.39e-01 | 1.99e-01 |
|  | 17 | 47074576 | 47074713 | PDFSTATSENSEMBLUCSC | 138 | 7 | 17480 | tss | 0.237513 | 0.065864 | Not queried | 2.07 | 2.59e-01 | 3.16e-01 |
|  | 17 | 30770932 | 30771552 | PDFSTATSENSEMBLUCSC | 621 | 8 | 18195 | island | 0.023744 | 0.012888 | Not queried | 2.07 | 3.79e-02 | 7.66e-02 |
|  | 19 | 51326980 | 51327477 | PDFSTATSENSEMBLUCSC | 498 | 4 | 22326 | island | 0.568365 | 0.674451 | Not queried | 2.07 | 8.86e-01 | 8.89e-01 |
|  | 10 | 135270766 | 135271052 | PDFSTATSENSEMBLUCSC | 287 | 5 | 4905 | gene;island | 0.805803 | 0.944272 | Not queried | 2.07 | 3.17e-02 | 6.50e-02 |
|  | 12 | 54089240 | 54089497 | PDFSTATSENSEMBLUCSC | 258 | 4 | 10471 | island | 0.906256 | 0.976626 | Not queried | 2.07 | 1.14e-01 | 1.68e-01 |
|  | 15 | 52472025 | 52472389 | PDFSTATSENSEMBLUCSC | 365 | 5 | 13355 | gene;island | 0.011515 | 0.054003 | Not queried | 2.07 | 5.48e-01 | 5.70e-01 |
|  | 2 | 220174201 | 220174251 | PDFSTATSENSEMBLUCSC | 51 | 4 | 23904 | tss;island | 0.912310 | 0.981270 | Not queried | 2.07 | 2.86e-02 | 6.12e-02 |
|  | 1 | 192777870 | 192778160 | PDFSTATSENSEMBLUCSC | 291 | 5 | 2148 | tss | 0.004023 | 0.003791 | Not queried | 2.07 | 4.21e-01 | 4.59e-01 |
|  | 11 | 130298544 | 130298880 | PDFSTATSENSEMBLUCSC | 337 | 8 | 7819 | tss | 0.295774 | 0.642631 | Not queried | 2.07 | 1.30e-01 | 1.89e-01 |
|  | 7 | 157694404 | 157694745 | PDFSTATSENSEMBLUCSC | 342 | 4 | 37352 | gene;island | 0.560800 | 0.822376 | Not queried | 2.07 | 2.86e-02 | 6.12e-02 |
|  | 1 | 219347021 | 219347458 | PDFSTATSENSEMBLUCSC | 438 | 7 | 4210 | island | 0.015673 | 0.003780 | Not queried | 2.07 | 9.73e-02 | 1.55e-01 |
|  | 1 | 219347252 | 219347458 | PDFSTATSENSEMBLUCSC | 207 | 5 | 1013 | gene | 0.020986 | 0.008949 | Not queried | 2.07 | 2.22e-01 | 2.76e-01 |
|  | 17 | 56736571 | 56736918 | PDFSTATSENSEMBLUCSC | 348 | 5 | 16710 | gene;island | 0.006625 | 0.019069 | Not queried | 2.07 | 8.41e-01 | 8.48e-01 |
|  | 20 | 37353032 | 37353096 | PDFSTATSENSEMBLUCSC | 65 | 4 | 25738 | tss | 0.900316 | 0.978562 | Not queried | 2.07 | 2.86e-02 | 6.12e-02 |
|  | 20 | 29611652 | 29611924 | PDFSTATSENSEMBLUCSC | 273 | 8 | 26069 | island | 0.591716 | 0.237872 | FRG1B (margin: FRG1B) | 2.07 | 6.22e-04 | 6.71e-03 |
|  | 8 | 72757787 | 72758095 | PDFSTATSENSEMBLUCSC | 309 | 4 | 39764 | tss;island | 0.646231 | 0.606416 | Not queried | 2.06 | 8.86e-01 | 8.89e-01 |
|  | 13 | 22033395 | 22033473 | PDFSTATSENSEMBLUCSC | 79 | 4 | 11590 | island | 0.032316 | 0.023049 | Not queried | 2.06 | 8.86e-01 | 8.89e-01 |
|  | 7 | 99097886 | 99098103 | PDFSTATSENSEMBLUCSC | 218 | 6 | 37757 | tss | 0.001941 | 0.009273 | Not queried | 2.06 | 1.80e-01 | 2.45e-01 |
|  | 17 | 7227499 | 7227737 | PDFSTATSENSEMBLUCSC | 239 | 4 | 16391 | gene;island | 0.556391 | 0.849085 | Not queried | 2.06 | 2.00e-01 | 2.55e-01 |
|  | 22 | 38073049 | 38073472 | PDFSTATSENSEMBLUCSC | 424 | 4 | 26862 | gene;island | 0.016223 | 0.061522 | Not queried | 2.06 | 3.43e-01 | 3.84e-01 |
|  | 6 | 161695210 | 161695561 | PDFSTATSENSEMBLUCSC | 352 | 6 | 35328 | tss | 0.014290 | 0.268873 | Not queried | 2.06 | 4.85e-01 | 5.11e-01 |
|  | 4 | 89618324 | 89619573 | PDFSTATSENSEMBLUCSC | 1250 | 20 | 30055 | gene | 0.916552 | 0.727714 | HERC3; NAP1L5 (margin: HERC3; FAM13A-AS1; NAP1L5) | 2.06 | 1.77e-03 | 1.45e-02 |
|  | 11 | 65601167 | 65601427 | PDFSTATSENSEMBLUCSC | 261 | 10 | 8481 | island | 0.817298 | 0.968632 | SNX32; CFL1 (margin: SNX32; CFL1) | 2.06 | 5.20e-03 | 2.96e-02 |
|  | 2 | 85838641 | 85839722 | PDFSTATSENSEMBLUCSC | 1082 | 9 | 24485 | island | 0.013555 | 0.086809 | Not queried | 2.06 | 6.25e-02 | 1.09e-01 |
|  | 1 | 156261200 | 156261403 | PDFSTATSENSEMBLUCSC | 204 | 5 | 820 | gene;island | 0.865515 | 0.963143 | TMEM79; C1orf85 (margin: TMEM79; SMG5; C1orf85; VHLL) | 2.06 | 7.94e-03 | 3.19e-02 |
|  | 17 | 41277006 | 41278906 | PDFSTATSENSEMBLUCSC | 1901 | 44 | 17383 | tss | 0.004738 | 0.014715 | Not queried | 2.06 | 1.38e-02 | 5.12e-02 |
|  | 5 | 176784318 | 176784719 | PDFSTATSENSEMBLUCSC | 402 | 6 | 32525 | tss | 0.343664 | 0.811456 | Not queried | 2.06 | 1.80e-01 | 2.45e-01 |
|  | 20 | 44519396 | 44519697 | PDFSTATSENSEMBLUCSC | 302 | 4 | 25435 | gene;island | 0.133101 | 0.022975 | Not queried | 2.06 | 6.86e-01 | 6.99e-01 |
|  | 11 | 58672702 | 58673167 | PDFSTATSENSEMBLUCSC | 466 | 5 | 8297 | island | 0.647497 | 0.858206 | Not queried | 2.06 | 1.51e-01 | 2.11e-01 |
|  | 7 | 44279049 | 44279273 | PDFSTATSENSEMBLUCSC | 225 | 4 | 36992 | gene;island | 0.470612 | 0.817328 | Not queried | 2.06 | 4.86e-01 | 5.11e-01 |
|  | 2 | 97405817 | 97406019 | PDFSTATSENSEMBLUCSC | 203 | 6 | 23522 | tss | 0.000274 | 0.001027 | Not queried | 2.06 | 4.11e-02 | 8.14e-02 |
|  | 8 | 86350278 | 86350592 | PDFSTATSENSEMBLUCSC | 315 | 6 | 39794 | tss;island | 0.800759 | 0.493979 | Not queried | 2.06 | 9.31e-02 | 1.53e-01 |
|  | 8 | 30240920 | 30241661 | PDFSTATSENSEMBLUCSC | 742 | 8 | 39631 | tss;island | 0.526127 | 0.128883 | Not queried | 2.06 | 3.82e-01 | 4.27e-01 |
|  | 11 | 2593857 | 2594153 | PDFSTATSENSEMBLUCSC | 297 | 4 | 6463 | gene;island | 0.511427 | 0.858342 | Not queried | 2.06 | 2.00e-01 | 2.55e-01 |
|  | 11 | 71823750 | 71824165 | PDFSTATSENSEMBLUCSC | 416 | 9 | 8627 | island | 0.003960 | 0.016467 | Not queried | 2.06 | 5.03e-02 | 9.87e-02 |
|  | 15 | 67417607 | 67417964 | PDFSTATSENSEMBLUCSC | 358 | 6 | 13392 | gene;tss | 0.036307 | 0.117475 | Not queried | 2.05 | 5.89e-01 | 6.11e-01 |
|  | 12 | 13256639 | 13256869 | PDFSTATSENSEMBLUCSC | 231 | 4 | 9581 | tss;island | 0.851906 | 0.970822 | Not queried | 2.05 | 2.00e-01 | 2.55e-01 |
|  | 22 | 32767005 | 32767162 | PDFSTATSENSEMBLUCSC | 158 | 4 | 27091 | tss | 0.660429 | 0.932250 | Not queried | 2.05 | 4.86e-01 | 5.11e-01 |
|  | 3 | 149530133 | 149530380 | PDFSTATSENSEMBLUCSC | 248 | 7 | 28659 | tss;island | 0.009858 | 0.019784 | Not queried | 2.05 | 2.09e-01 | 2.65e-01 |
|  | 2 | 163200302 | 163200576 | PDFSTATSENSEMBLUCSC | 275 | 5 | 23689 | tss | 0.084000 | 0.025314 | Not queried | 2.05 | 3.10e-01 | 3.66e-01 |
|  | 12 | 49371987 | 49372180 | PDFSTATSENSEMBLUCSC | 194 | 7 | 9679 | tss | 0.230877 | 0.472849 | Not queried | 2.05 | 1.75e-02 | 5.79e-02 |
|  | 3 | 42977777 | 42978248 | PDFSTATSENSEMBLUCSC | 472 | 8 | 29024 | island | 0.562840 | 0.947051 | Not queried | 2.05 | 1.95e-01 | 2.55e-01 |
|  | 3 | 64547108 | 64547373 | PDFSTATSENSEMBLUCSC | 266 | 5 | 27849 | gene | 0.793113 | 0.475992 | Not queried | 2.05 | 1.59e-02 | 5.36e-02 |
|  | 8 | 27347861 | 27348671 | PDFSTATSENSEMBLUCSC | 811 | 7 | 40181 | island | 0.602422 | 0.598784 | Not queried | 2.05 | 6.20e-01 | 6.43e-01 |
|  | 1 | 116380946 | 116381082 | PDFSTATSENSEMBLUCSC | 137 | 4 | 705 | gene;island | 0.269837 | 0.622637 | Not queried | 2.05 | 2.86e-02 | 6.12e-02 |
|  | 11 | 74660429 | 74660536 | PDFSTATSENSEMBLUCSC | 108 | 4 | 6830 | gene | 0.007716 | 0.015739 | Not queried | 2.05 | 4.86e-01 | 5.11e-01 |
|  | 14 | 106938234 | 106938621 | PDFSTATSENSEMBLUCSC | 388 | 10 | 13248 | island | 0.870637 | 0.966609 | LINC00221 (margin: LINC00221; IGHV3-43; IGHVII-43-1; IGHVIII-44; IGHVIV-44-1; IGHVII-44-2) | 2.05 | 5.20e-03 | 2.96e-02 |
|  | 11 | 76839129 | 76839217 | PDFSTATSENSEMBLUCSC | 89 | 4 | 7583 | tss | 0.816286 | 0.596694 | Not queried | 2.05 | 4.86e-01 | 5.11e-01 |
|  | 10 | 51784543 | 51784888 | PDFSTATSENSEMBLUCSC | 346 | 4 | 5742 | island | 0.599175 | 0.872804 | Not queried | 2.05 | 2.00e-01 | 2.55e-01 |
|  | 7 | 99686291 | 99686457 | PDFSTATSENSEMBLUCSC | 167 | 9 | 37767 | tss;island | 0.012040 | 0.020776 | Not queried | 2.05 | 2.58e-01 | 3.16e-01 |
|  | 19 | 3971119 | 3971417 | PDFSTATSENSEMBLUCSC | 299 | 5 | 21357 | island | 0.004871 | 0.013584 | Not queried | 2.05 | 4.21e-01 | 4.59e-01 |
|  | 3 | 50243021 | 50243323 | PDFSTATSENSEMBLUCSC | 303 | 4 | 27795 | gene;island | 0.586167 | 0.501927 | Not queried | 2.05 | 6.86e-01 | 6.99e-01 |
|  | 17 | 79317768 | 79318146 | PDFSTATSENSEMBLUCSC | 379 | 4 | 18914 | island | 0.074128 | 0.012300 | Not queried | 2.05 | 1.14e-01 | 1.68e-01 |
|  | 10 | 45496476 | 45496710 | PDFSTATSENSEMBLUCSC | 235 | 5 | 5058 | tss | 0.169611 | 0.080981 | Not queried | 2.05 | 2.22e-01 | 2.76e-01 |
|  | 16 | 67876973 | 67877413 | PDFSTATSENSEMBLUCSC | 441 | 4 | 14803 | gene;island | 0.689655 | 0.398024 | Not queried | 2.05 | 6.86e-01 | 6.99e-01 |
|  | 10 | 93646991 | 93647300 | PDFSTATSENSEMBLUCSC | 310 | 4 | 5934 | island | 0.269633 | 0.601237 | Not queried | 2.05 | 2.86e-02 | 6.12e-02 |
|  | 4 | 80247171 | 80247281 | PDFSTATSENSEMBLUCSC | 111 | 5 | 30456 | tss | 0.886046 | 0.976266 | NAA11 (margin: NAA11) | 2.05 | 7.94e-03 | 3.19e-02 |
|  | 8 | 29210580 | 29210800 | PDFSTATSENSEMBLUCSC | 221 | 4 | 40202 | island | 0.910688 | 0.977201 | Not queried | 2.05 | 3.43e-01 | 3.84e-01 |
|  | 11 | 63753020 | 63753598 | PDFSTATSENSEMBLUCSC | 579 | 10 | 8379 | island | 0.541299 | 0.817869 | Not queried | 2.05 | 2.18e-01 | 2.76e-01 |
|  | 1 | 45451992 | 45452605 | PDFSTATSENSEMBLUCSC | 614 | 8 | 3193 | island | 0.087904 | 0.022513 | Not queried | 2.05 | 4.42e-01 | 4.81e-01 |
|  | 5 | 37838290 | 37838871 | PDFSTATSENSEMBLUCSC | 582 | 6 | 31592 | gene;island | 0.242032 | 0.585885 | Not queried | 2.05 | 3.94e-01 | 4.36e-01 |
|  | 14 | 67982146 | 67982198 | PDFSTATSENSEMBLUCSC | 53 | 6 | 12471 | tss;island | 0.004209 | 0.003742 | Not queried | 2.05 | 1.80e-01 | 2.45e-01 |
|  | 3 | 123752393 | 123752573 | PDFSTATSENSEMBLUCSC | 181 | 4 | 29414 | island | 0.068983 | 0.024090 | Not queried | 2.04 | 3.43e-01 | 3.84e-01 |
|  | 22 | 19701734 | 19701871 | PDFSTATSENSEMBLUCSC | 138 | 4 | 26963 | tss;island | 0.376214 | 0.603244 | Not queried | 2.04 | 3.43e-01 | 3.84e-01 |
|  | 7 | 25897148 | 25897907 | PDFSTATSENSEMBLUCSC | 760 | 8 | 38324 | island | 0.286760 | 0.046493 | Not queried | 2.04 | 5.05e-01 | 5.32e-01 |
|  | 20 | 47835750 | 47835804 | PDFSTATSENSEMBLUCSC | 55 | 4 | 25805 | tss | 0.004369 | 0.011080 | Not queried | 2.04 | 2.86e-02 | 6.12e-02 |
|  | 12 | 56843287 | 56843596 | PDFSTATSENSEMBLUCSC | 310 | 7 | 9798 | tss | 0.002133 | 0.009169 | Not queried | 2.04 | 4.56e-01 | 4.96e-01 |
|  | 19 | 5838729 | 5838999 | PDFSTATSENSEMBLUCSC | 271 | 7 | 19757 | gene | 0.662902 | 0.963688 | Not queried | 2.04 | 7.28e-02 | 1.23e-01 |
|  | 5 | 140213783 | 140213924 | PDFSTATSENSEMBLUCSC | 142 | 5 | 31798 | gene;tss;island | 0.276456 | 0.818427 | Not queried | 2.04 | 9.52e-02 | 1.53e-01 |
|  | 4 | 74847646 | 74848016 | PDFSTATSENSEMBLUCSC | 371 | 8 | 31099 | island | 0.774410 | 0.557629 | Not queried | 2.04 | 3.28e-01 | 3.84e-01 |
|  | 2 | 45159894 | 45160554 | PDFSTATSENSEMBLUCSC | 661 | 8 | 24260 | island | 0.808178 | 0.965447 | Not queried | 2.04 | 1.05e-01 | 1.66e-01 |
|  | 2 | 165698673 | 165698949 | PDFSTATSENSEMBLUCSC | 277 | 4 | 23693 | tss;island | 0.003280 | 0.012057 | Not queried | 2.04 | 2.00e-01 | 2.55e-01 |
|  | 7 | 27225058 | 27225897 | PDFSTATSENSEMBLUCSC | 840 | 23 | 36942 | gene | 0.833675 | 0.959871 | HOXA11-AS; HOXA11; RP1-170O19.14 (margin: HOXA11-AS; RP1-170O19.20; HOXA10; HOXA11; RP1-170O19.14; HOXA13) | 2.04 | 1.01e-06 | 9.49e-05 |
|  | 6 | 30582174 | 30582416 | PDFSTATSENSEMBLUCSC | 243 | 5 | 33785 | gene;island | 0.561928 | 0.894464 | Not queried | 2.04 | 2.22e-01 | 2.76e-01 |
|  | 20 | 46414866 | 46414999 | PDFSTATSENSEMBLUCSC | 134 | 5 | 25801 | tss | 0.047954 | 0.171546 | Not queried | 2.04 | 3.10e-01 | 3.66e-01 |
|  | 17 | 60885491 | 60886079 | PDFSTATSENSEMBLUCSC | 589 | 7 | 18638 | island | 0.093786 | 0.041673 | Not queried | 2.04 | 3.18e-01 | 3.74e-01 |
|  | 12 | 117319785 | 117320316 | PDFSTATSENSEMBLUCSC | 532 | 7 | 10041 | tss;island | 0.771713 | 0.453923 | Not queried | 2.04 | 1.28e-01 | 1.87e-01 |
|  | 2 | 85553759 | 85554135 | PDFSTATSENSEMBLUCSC | 377 | 5 | 22761 | gene;island | 0.074682 | 0.323650 | Not queried | 2.04 | 2.22e-01 | 2.76e-01 |
|  | 14 | 21270850 | 21271355 | PDFSTATSENSEMBLUCSC | 506 | 6 | 12278 | tss | 0.924397 | 0.980462 | Not queried | 2.04 | 4.11e-02 | 8.14e-02 |
|  | 12 | 106974832 | 106975246 | PDFSTATSENSEMBLUCSC | 415 | 5 | 10751 | island | 0.814524 | 0.949161 | Not queried | 2.04 | 9.52e-02 | 1.53e-01 |
|  | 12 | 7261841 | 7262137 | PDFSTATSENSEMBLUCSC | 297 | 9 | 9545 | tss | 0.003981 | 0.028844 | Not queried | 2.03 | 5.03e-02 | 9.87e-02 |
|  | 18 | 47018095 | 47018265 | PDFSTATSENSEMBLUCSC | 171 | 4 | 19110 | gene;tss;island | 0.005055 | 0.009116 | Not queried | 2.03 | 3.43e-01 | 3.84e-01 |
|  | 1 | 3435887 | 3436219 | PDFSTATSENSEMBLUCSC | 333 | 5 | 128 | gene;island | 0.696388 | 0.950082 | Not queried | 2.03 | 1.51e-01 | 2.11e-01 |
|  | 19 | 58545001 | 58545333 | PDFSTATSENSEMBLUCSC | 333 | 8 | 21131 | tss;island | 0.975146 | 0.894480 | Not queried | 2.03 | 1.05e-01 | 1.66e-01 |
|  | 10 | 11047271 | 11047586 | PDFSTATSENSEMBLUCSC | 316 | 6 | 4491 | gene | 0.665388 | 0.349649 | Not queried | 2.03 | 9.31e-02 | 1.53e-01 |
|  | 10 | 82168043 | 82168153 | PDFSTATSENSEMBLUCSC | 111 | 5 | 5189 | tss | 0.034565 | 0.008010 | Not queried | 2.03 | 3.17e-02 | 6.50e-02 |
|  | 10 | 124906421 | 124906930 | PDFSTATSENSEMBLUCSC | 510 | 6 | 5416 | tss;island | 0.432435 | 0.793763 | Not queried | 2.03 | 6.49e-02 | 1.11e-01 |
|  | 7 | 150864885 | 150865291 | PDFSTATSENSEMBLUCSC | 407 | 10 | 37993 | tss;island | 0.140529 | 0.032386 | GBX1 (margin: GBX1; ASB10) | 2.03 | 1.15e-02 | 4.29e-02 |
|  | 2 | 154727907 | 154728308 | PDFSTATSENSEMBLUCSC | 402 | 7 | 23670 | tss | 0.785151 | 0.956244 | Not queried | 2.03 | 5.30e-02 | 1.00e-01 |
|  | 2 | 105488809 | 105488932 | PDFSTATSENSEMBLUCSC | 124 | 4 | 24581 | island | 0.309343 | 0.102787 | Not queried | 2.03 | 2.86e-02 | 6.12e-02 |
|  | 1 | 161059429 | 161059654 | PDFSTATSENSEMBLUCSC | 226 | 4 | 2044 | tss | 0.164723 | 0.044934 | Not queried | 2.03 | 3.43e-01 | 3.84e-01 |
|  | 4 | 79472605 | 79472939 | PDFSTATSENSEMBLUCSC | 335 | 5 | 31127 | island | 0.031050 | 0.077206 | Not queried | 2.03 | 6.90e-01 | 7.01e-01 |
|  | 6 | 64281439 | 64281886 | PDFSTATSENSEMBLUCSC | 448 | 8 | 35054 | tss | 0.003302 | 0.025565 | Not queried | 2.03 | 8.30e-02 | 1.40e-01 |
|  | 6 | 33133329 | 33133675 | PDFSTATSENSEMBLUCSC | 347 | 8 | 35964 | island | 0.406394 | 0.735657 | Not queried | 2.03 | 2.07e-02 | 6.12e-02 |
|  | 1 | 26605913 | 26606110 | PDFSTATSENSEMBLUCSC | 198 | 4 | 1388 | tss;island | 0.347705 | 0.687118 | Not queried | 2.03 | 5.71e-02 | 1.00e-01 |
|  | 3 | 12837740 | 12838136 | PDFSTATSENSEMBLUCSC | 397 | 7 | 28161 | tss | 0.680865 | 0.878524 | Not queried | 2.03 | 1.75e-02 | 5.79e-02 |
|  | 7 | 100034139 | 100034454 | PDFSTATSENSEMBLUCSC | 316 | 6 | 37782 | tss | 0.004694 | 0.012314 | Not queried | 2.03 | 3.94e-01 | 4.36e-01 |
|  | 20 | 36889208 | 36889389 | PDFSTATSENSEMBLUCSC | 182 | 7 | 25732 | tss | 0.819947 | 0.934596 | Not queried | 2.03 | 9.73e-02 | 1.55e-01 |
|  | 7 | 127033361 | 127033695 | PDFSTATSENSEMBLUCSC | 335 | 4 | 37876 | tss;island | 0.146951 | 0.025084 | Not queried | 2.03 | 3.43e-01 | 3.84e-01 |
|  | 11 | 102187898 | 102188180 | PDFSTATSENSEMBLUCSC | 283 | 7 | 7647 | tss | 0.001843 | 0.008428 | Not queried | 2.03 | 1.28e-01 | 1.87e-01 |
|  | 19 | 17448797 | 17449202 | PDFSTATSENSEMBLUCSC | 406 | 4 | 19917 | gene;island | 0.182698 | 0.046611 | Not queried | 2.03 | 4.86e-01 | 5.11e-01 |
|  | 1 | 153650884 | 153651147 | PDFSTATSENSEMBLUCSC | 264 | 7 | 1950 | tss | 0.127992 | 0.372358 | Not queried | 2.03 | 5.30e-02 | 1.00e-01 |
|  | 8 | 22960461 | 22960619 | PDFSTATSENSEMBLUCSC | 159 | 4 | 39239 | gene | 0.845295 | 0.978469 | Not queried | 2.03 | 5.71e-02 | 1.00e-01 |
|  | 5 | 139422525 | 139422829 | PDFSTATSENSEMBLUCSC | 305 | 4 | 31783 | gene | 0.057616 | 0.018553 | Not queried | 2.03 | 5.71e-02 | 1.00e-01 |
|  | 1 | 154475068 | 154475269 | PDFSTATSENSEMBLUCSC | 202 | 4 | 779 | gene;tss;island | 0.940904 | 0.987412 | Not queried | 2.03 | 5.71e-02 | 1.00e-01 |
|  | 21 | 45713510 | 45713719 | PDFSTATSENSEMBLUCSC | 210 | 4 | 26454 | gene;island | 0.902866 | 0.966303 | Not queried | 2.03 | 3.43e-01 | 3.84e-01 |
|  | 5 | 173738161 | 173738479 | PDFSTATSENSEMBLUCSC | 319 | 4 | 33415 | island | 0.285480 | 0.607141 | Not queried | 2.03 | 4.86e-01 | 5.11e-01 |
|  | 1 | 11795746 | 11796297 | PDFSTATSENSEMBLUCSC | 552 | 13 | 2687 | island | 0.008406 | 0.013087 | Not queried | 2.03 | 2.04e-01 | 2.59e-01 |
|  | 11 | 3187271 | 3188016 | PDFSTATSENSEMBLUCSC | 746 | 24 | 7092 | tss;island | 0.814123 | 0.934281 | OSBPL5 (margin: OSBPL5) | 2.03 | 7.11e-05 | 1.91e-03 |
|  | 6 | 169951905 | 169952299 | PDFSTATSENSEMBLUCSC | 395 | 4 | 34569 | gene | 0.967829 | 0.988114 | Not queried | 2.03 | 1.14e-01 | 1.68e-01 |
|  | 4 | 41936852 | 41937393 | PDFSTATSENSEMBLUCSC | 542 | 8 | 31011 | island | 0.012196 | 0.036938 | Not queried | 2.03 | 4.99e-02 | 9.81e-02 |
|  | 6 | 30122947 | 30123226 | PDFSTATSENSEMBLUCSC | 280 | 5 | 33730 | gene | 0.165577 | 0.653741 | Not queried | 2.02 | 5.48e-01 | 5.70e-01 |
|  | 6 | 31801526 | 31801807 | PDFSTATSENSEMBLUCSC | 282 | 5 | 34842 | tss;island | 0.906923 | 0.978506 | Not queried | 2.02 | 9.52e-02 | 1.53e-01 |
|  | 4 | 106816089 | 106816564 | PDFSTATSENSEMBLUCSC | 476 | 5 | 30532 | tss;island | 0.053472 | 0.015545 | Not queried | 2.02 | 1.51e-01 | 2.11e-01 |
|  | 12 | 52579502 | 52579837 | PDFSTATSENSEMBLUCSC | 336 | 4 | 9134 | gene | 0.837867 | 0.937097 | Not queried | 2.02 | 2.86e-02 | 6.12e-02 |
|  | 7 | 150147536 | 150147927 | PDFSTATSENSEMBLUCSC | 392 | 6 | 37979 | tss | 0.600374 | 0.807049 | Not queried | 2.02 | 9.31e-02 | 1.53e-01 |
|  | 13 | 46425866 | 46426263 | PDFSTATSENSEMBLUCSC | 398 | 9 | 11424 | tss | 0.820308 | 0.982753 | Not queried | 2.02 | 2.22e-01 | 2.76e-01 |
|  | 19 | 58038573 | 58038689 | PDFSTATSENSEMBLUCSC | 117 | 6 | 21118 | tss | 0.661445 | 0.356010 | ZNF549 (margin: ZNF773; ZNF549; ZNF550) | 2.02 | 2.16e-03 | 1.45e-02 |
|  | 8 | 58055876 | 58056175 | PDFSTATSENSEMBLUCSC | 300 | 4 | 40319 | island | 0.889450 | 0.962600 | Not queried | 2.02 | 4.86e-01 | 5.11e-01 |
|  | 19 | 7197021 | 7197397 | PDFSTATSENSEMBLUCSC | 377 | 4 | 19773 | gene;island | 0.186379 | 0.500054 | Not queried | 2.02 | 3.43e-01 | 3.84e-01 |
|  | 19 | 40791312 | 40791701 | PDFSTATSENSEMBLUCSC | 390 | 8 | 20785 | tss;island | 0.004502 | 0.013995 | Not queried | 2.02 | 2.34e-01 | 2.90e-01 |
|  | 11 | 1474386 | 1474841 | PDFSTATSENSEMBLUCSC | 456 | 5 | 6421 | gene;island | 0.254102 | 0.615822 | Not queried | 2.02 | 2.22e-01 | 2.76e-01 |
|  | 19 | 11465809 | 11466020 | PDFSTATSENSEMBLUCSC | 212 | 4 | 20469 | tss | 0.010972 | 0.025954 | Not queried | 2.02 | 6.86e-01 | 6.99e-01 |
|  | 1 | 91870362 | 91870450 | PDFSTATSENSEMBLUCSC | 89 | 4 | 3472 | island | 0.390289 | 0.123399 | Not queried | 2.02 | 3.43e-01 | 3.84e-01 |
|  | 3 | 42543933 | 42544131 | PDFSTATSENSEMBLUCSC | 199 | 7 | 29013 | island | 0.003578 | 0.008362 | Not queried | 2.02 | 2.59e-01 | 3.16e-01 |
|  | 5 | 63802491 | 63802993 | PDFSTATSENSEMBLUCSC | 503 | 5 | 31628 | gene | 0.621221 | 0.321884 | Not queried | 2.02 | 8.41e-01 | 8.48e-01 |
|  | 3 | 25469860 | 25469991 | PDFSTATSENSEMBLUCSC | 132 | 6 | 27705 | gene | 0.622443 | 0.916741 | Not queried | 2.02 | 2.40e-01 | 2.95e-01 |
|  | 2 | 213400697 | 213401333 | PDFSTATSENSEMBLUCSC | 637 | 5 | 23005 | gene;island | 0.832168 | 0.528477 | Not queried | 2.02 | 5.48e-01 | 5.70e-01 |
|  | 20 | 44657637 | 44657812 | PDFSTATSENSEMBLUCSC | 176 | 5 | 25787 | tss | 0.905406 | 0.951658 | Not queried | 2.02 | 2.22e-01 | 2.76e-01 |
|  | 17 | 46507690 | 46507895 | PDFSTATSENSEMBLUCSC | 206 | 7 | 17455 | tss | 0.791682 | 0.935175 | Not queried | 2.01 | 2.62e-02 | 6.12e-02 |
|  | 17 | 43222106 | 43222258 | PDFSTATSENSEMBLUCSC | 153 | 4 | 18426 | island | 0.819976 | 0.915065 | Not queried | 2.01 | 5.71e-02 | 1.00e-01 |
|  | 2 | 131185333 | 131185700 | PDFSTATSENSEMBLUCSC | 368 | 6 | 24720 | island | 0.871543 | 0.716327 | Not queried | 2.01 | 2.60e-02 | 6.12e-02 |
|  | 12 | 123464511 | 123464823 | PDFSTATSENSEMBLUCSC | 313 | 5 | 10095 | tss | 0.081475 | 0.167295 | Not queried | 2.01 | 2.22e-01 | 2.76e-01 |
|  | 16 | 67197084 | 67197186 | PDFSTATSENSEMBLUCSC | 103 | 5 | 14790 | gene;tss;island | 0.852980 | 0.980058 | Not queried | 2.01 | 9.52e-02 | 1.53e-01 |
|  | 4 | 37246688 | 37247022 | PDFSTATSENSEMBLUCSC | 335 | 4 | 30973 | island | 0.837796 | 0.923075 | Not queried | 2.01 | 2.00e-01 | 2.55e-01 |
|  | 5 | 76507050 | 76507680 | PDFSTATSENSEMBLUCSC | 631 | 5 | 31654 | gene;island | 0.639026 | 0.939224 | Not queried | 2.01 | 5.56e-02 | 1.00e-01 |
|  | 14 | 50052765 | 50053030 | PDFSTATSENSEMBLUCSC | 266 | 4 | 12076 | gene | 0.003267 | 0.006266 | Not queried | 2.01 | 2.00e-01 | 2.55e-01 |
|  | 6 | 29910051 | 29910292 | PDFSTATSENSEMBLUCSC | 242 | 9 | 34759 | tss | 0.026947 | 0.292075 | Not queried | 2.01 | 2.97e-01 | 3.60e-01 |
|  | 7 | 23636744 | 23636910 | PDFSTATSENSEMBLUCSC | 167 | 5 | 37474 | tss | 0.023707 | 0.018266 | Not queried | 2.01 | 6.90e-01 | 7.01e-01 |
|  | 19 | 53141620 | 53141793 | PDFSTATSENSEMBLUCSC | 174 | 4 | 20198 | gene;island | 0.040655 | 0.024919 | Not queried | 2.01 | 6.86e-01 | 6.99e-01 |
|  | 15 | 45815087 | 45815405 | PDFSTATSENSEMBLUCSC | 319 | 7 | 13334 | gene;tss;island | 0.046648 | 0.011735 | HMGN2P46; SLC30A4 (margin: RP11-519G16.3; HMGN2P46; SLC30A4) | 2.01 | 1.11e-02 | 4.14e-02 |
|  | 4 | 81187601 | 81187906 | PDFSTATSENSEMBLUCSC | 306 | 5 | 31136 | island | 0.521121 | 0.212256 | FGF5 (margin: FGF5) | 2.01 | 7.94e-03 | 3.19e-02 |
|  | 17 | 4544925 | 4545157 | PDFSTATSENSEMBLUCSC | 233 | 5 | 17873 | island | 0.903767 | 0.972708 | ALOX15 (margin: ALOX15) | 2.01 | 7.94e-03 | 3.19e-02 |
|  | 3 | 160167317 | 160167990 | PDFSTATSENSEMBLUCSC | 674 | 14 | 29631 | island | 0.226536 | 0.015480 | Not queried | 2.01 | 3.95e-02 | 7.97e-02 |
|  | 16 | 850240 | 850713 | PDFSTATSENSEMBLUCSC | 474 | 6 | 14548 | gene | 0.106227 | 0.019205 | Not queried | 2.01 | 1.80e-01 | 2.45e-01 |
|  | 8 | 140744200 | 140744488 | PDFSTATSENSEMBLUCSC | 289 | 5 | 39436 | gene;island | 0.937628 | 0.983775 | Not queried | 2.01 | 3.10e-01 | 3.66e-01 |
|  | 11 | 2017362 | 2018247 | PDFSTATSENSEMBLUCSC | 886 | 13 | 6443 | gene;island | 0.591597 | 0.937674 | H19 (margin: MRPL23; AC051649.6; MRPL23-AS1; H19) | 2.01 | 7.95e-04 | 8.28e-03 |
|  | 5 | 143978290 | 143978420 | PDFSTATSENSEMBLUCSC | 131 | 4 | 33275 | island | 0.606882 | 0.317538 | Not queried | 2.01 | 1.14e-01 | 1.68e-01 |
|  | 20 | 62153067 | 62153431 | PDFSTATSENSEMBLUCSC | 365 | 4 | 25502 | gene;island | 0.907612 | 0.615861 | Not queried | 2.01 | 1.14e-01 | 1.68e-01 |
|  | 19 | 15590328 | 15590570 | PDFSTATSENSEMBLUCSC | 243 | 4 | 20564 | tss | 0.523380 | 0.247893 | Not queried | 2.01 | 6.86e-01 | 6.99e-01 |
|  | 4 | 647194 | 647480 | PDFSTATSENSEMBLUCSC | 287 | 4 | 29830 | gene;island | 0.474947 | 0.844313 | Not queried | 2.01 | 2.00e-01 | 2.55e-01 |
|  | 7 | 28725358 | 28725676 | PDFSTATSENSEMBLUCSC | 319 | 5 | 36946 | gene;tss | 0.014305 | 0.003667 | Not queried | 2.01 | 3.10e-01 | 3.66e-01 |
|  | 10 | 15761192 | 15761657 | PDFSTATSENSEMBLUCSC | 466 | 4 | 4514 | gene | 0.813172 | 0.949496 | Not queried | 2.01 | 5.71e-02 | 1.00e-01 |
|  | 12 | 71003625 | 71003964 | PDFSTATSENSEMBLUCSC | 340 | 7 | 9868 | tss | 0.241343 | 0.561063 | Not queried | 2.01 | 2.09e-01 | 2.65e-01 |
|  | 2 | 239139911 | 239140296 | PDFSTATSENSEMBLUCSC | 386 | 7 | 23096 | gene | 0.937505 | 0.983479 | AC096574.4; AC016757.3 (margin: AC096574.4; AC016757.3; HES6) | 2.01 | 2.33e-03 | 1.52e-02 |
|  | 2 | 128422179 | 128422717 | PDFSTATSENSEMBLUCSC | 539 | 8 | 23614 | tss | 0.863115 | 0.953905 | LIMS2 (margin: GPR17; LIMS2) | 2.01 | 1.04e-02 | 3.96e-02 |
|  | 15 | 68117903 | 68118127 | PDFSTATSENSEMBLUCSC | 225 | 4 | 14252 | island | 0.461181 | 0.212343 | Not queried | 2.00 | 1.14e-01 | 1.68e-01 |
|  | 19 | 37157565 | 37157995 | PDFSTATSENSEMBLUCSC | 431 | 11 | 21944 | island | 0.039552 | 0.005840 | ZNF461 (margin: ZNF461) | 2.00 | 4.10e-03 | 2.48e-02 |
|  | 6 | 70992907 | 70993107 | PDFSTATSENSEMBLUCSC | 201 | 4 | 34383 | gene;island | 0.790373 | 0.341140 | Not queried | 2.00 | 1.14e-01 | 1.68e-01 |
|  | 5 | 1295737 | 1296007 | PDFSTATSENSEMBLUCSC | 271 | 5 | 31969 | tss;island | 0.875634 | 0.969996 | Not queried | 2.00 | 1.51e-01 | 2.11e-01 |
|  | 19 | 13985408 | 13985688 | PDFSTATSENSEMBLUCSC | 281 | 5 | 21661 | island | 0.938767 | 0.777777 | Not queried | 2.00 | 2.22e-01 | 2.76e-01 |
|  | 14 | 23503928 | 23504423 | PDFSTATSENSEMBLUCSC | 496 | 6 | 12022 | gene | 0.001472 | 0.005018 | Not queried | 2.00 | 9.31e-02 | 1.53e-01 |
|  | 7 | 102105124 | 102105344 | PDFSTATSENSEMBLUCSC | 221 | 6 | 37804 | tss;island | 0.003612 | 0.007466 | Not queried | 2.00 | 2.40e-01 | 2.95e-01 |
|  | 11 | 1036714 | 1036938 | PDFSTATSENSEMBLUCSC | 225 | 6 | 7036 | tss | 0.098237 | 0.384009 | MUC6 (margin: MUC6) | 2.00 | 8.66e-03 | 3.36e-02 |
|  | 2 | 132919534 | 132919865 | PDFSTATSENSEMBLUCSC | 332 | 5 | 23636 | tss | 0.567272 | 0.800301 | Not queried | 2.00 | 9.52e-02 | 1.53e-01 |
|  | 2 | 47150 | 47452 | PDFSTATSENSEMBLUCSC | 303 | 4 | 23151 | tss;island | 0.002206 | 0.008374 | Not queried | 2.00 | 3.43e-01 | 3.84e-01 |
|  | 18 | 21033072 | 21033426 | PDFSTATSENSEMBLUCSC | 355 | 4 | 19073 | gene;island | 0.005336 | 0.016823 | Not queried | 2.00 | 3.43e-01 | 3.84e-01 |
|  | 13 | 112984478 | 112984976 | PDFSTATSENSEMBLUCSC | 499 | 5 | 11919 | island | 0.805527 | 0.920599 | Not queried | 2.00 | 4.21e-01 | 4.59e-01 |
|  | 5 | 176326365 | 176326619 | PDFSTATSENSEMBLUCSC | 255 | 4 | 32519 | tss | 0.615379 | 0.801128 | Not queried | 2.00 | 3.43e-01 | 3.84e-01 |
|  | 6 | 30709428 | 30709924 | PDFSTATSENSEMBLUCSC | 497 | 5 | 33822 | gene;island | 0.444591 | 0.777246 | Not queried | 2.00 | 9.52e-02 | 1.53e-01 |
|  | 4 | 41694736 | 41694847 | PDFSTATSENSEMBLUCSC | 112 | 4 | 29992 | gene | 0.485474 | 0.788755 | Not queried | 2.00 | 6.86e-01 | 6.99e-01 |
|  | 17 | 46114615 | 46114933 | PDFSTATSENSEMBLUCSC | 319 | 6 | 17450 | tss | 0.181848 | 0.048458 | Not queried | 2.00 | 1.80e-01 | 2.45e-01 |
|  | 6 | 24403225 | 24403712 | PDFSTATSENSEMBLUCSC | 488 | 5 | 33591 | gene | 0.003919 | 0.015366 | Not queried | 2.00 | 3.10e-01 | 3.66e-01 |
|  | 1 | 6663269 | 6663664 | PDFSTATSENSEMBLUCSC | 396 | 5 | 1212 | tss;island | 0.580910 | 0.847225 | Not queried | 2.00 | 5.48e-01 | 5.70e-01 |
|  | 16 | 81254010 | 81254209 | PDFSTATSENSEMBLUCSC | 200 | 4 | 15380 | tss | 0.938256 | 0.773910 | Not queried | 2.00 | 2.00e-01 | 2.55e-01 |
|  | 2 | 3063115 | 3063671 | PDFSTATSENSEMBLUCSC | 557 | 9 | 24025 | island | 0.381819 | 0.883266 | Not queried | 2.00 | 1.88e-02 | 6.12e-02 |
|  | 8 | 69244152 | 69244734 | PDFSTATSENSEMBLUCSC | 583 | 6 | 40369 | island | 0.625440 | 0.881983 | Not queried | 2.00 | 2.40e-01 | 2.95e-01 |
|  | 4 | 53727714 | 53728038 | PDFSTATSENSEMBLUCSC | 325 | 5 | 30393 | tss;island | 0.948418 | 0.897214 | Not queried | 2.00 | 5.48e-01 | 5.70e-01 |
|  | 15 | 80696264 | 80696529 | PDFSTATSENSEMBLUCSC | 266 | 4 | 13850 | tss;island | 0.488169 | 0.710172 | Not queried | 2.00 | 1.00e+00 | 1.00e+00 |
|  | 5 | 140753415 | 140753924 | PDFSTATSENSEMBLUCSC | 510 | 10 | 31825 | gene;island | 0.876774 | 0.956341 | Not queried | 2.00 | 1.05e-01 | 1.66e-01 |
|  | 5 | 172664995 | 172665149 | PDFSTATSENSEMBLUCSC | 155 | 4 | 33408 | island | 0.756676 | 0.512977 | Not queried | 2.00 | 3.43e-01 | 3.84e-01 |
|  | 5 | 10442103 | 10442612 | PDFSTATSENSEMBLUCSC | 510 | 5 | 31561 | gene;island | 0.129155 | 0.047008 | Not queried | 2.00 | 4.21e-01 | 4.59e-01 |
|  | 21 | 38362632 | 38362754 | PDFSTATSENSEMBLUCSC | 123 | 5 | 26540 | tss;island | 0.007101 | 0.044167 | Not queried | 2.00 | 2.22e-01 | 2.76e-01 |
|  | 6 | 146919854 | 146920295 | PDFSTATSENSEMBLUCSC | 442 | 10 | 36545 | island | 0.265691 | 0.018965 | Not queried | 2.00 | 1.47e-02 | 5.36e-02 |
|  | 6 | 146919854 | 146920129 | PDFSTATSENSEMBLUCSC | 276 | 8 | 35277 | tss | 0.275897 | 0.024129 | Not queried | 2.00 | 6.50e-02 | 1.11e-01 |
|  | 5 | 140201153 | 140201746 | PDFSTATSENSEMBLUCSC | 594 | 5 | 31796 | gene;island | 0.884266 | 0.643851 | Not queried | 2.00 | 2.22e-01 | 2.76e-01 |
|  | 7 | 154003215 | 154003309 | PDFSTATSENSEMBLUCSC | 95 | 4 | 37303 | gene;island | 0.564440 | 0.254019 | Not queried | 2.00 | 3.43e-01 | 3.84e-01 |
|  | 1 | 3824232 | 3824447 | PDFSTATSENSEMBLUCSC | 216 | 4 | 151 | gene;island | 0.851477 | 0.971588 | Not queried | 2.00 | 1.14e-01 | 1.68e-01 |
|  | 21 | 43198283 | 43198797 | PDFSTATSENSEMBLUCSC | 515 | 6 | 26694 | island | 0.010290 | 0.009938 | Not queried | 2.00 | 6.99e-01 | 7.09e-01 |
|  | 16 | 22012456 | 22012743 | PDFSTATSENSEMBLUCSC | 288 | 7 | 15129 | tss | 0.007257 | 0.033263 | Not queried | 2.00 | 2.62e-02 | 6.12e-02 |
|  | 8 | 37593814 | 37594251 | PDFSTATSENSEMBLUCSC | 438 | 11 | 40219 | island | 0.039883 | 0.022659 | Not queried | 2.00 | 4.00e-02 | 8.03e-02 |
|  | 6 | 31088188 | 31088434 | PDFSTATSENSEMBLUCSC | 247 | 5 | 33850 | gene | 0.929932 | 0.740114 | Not queried | 1.99 | 5.56e-02 | 1.00e-01 |
|  | 17 | 47074576 | 47074883 | PDFSTATSENSEMBLUCSC | 308 | 8 | 18516 | island | 0.347907 | 0.070822 | Not queried | 1.99 | 1.95e-01 | 2.55e-01 |
|  | 22 | 37823517 | 37823656 | PDFSTATSENSEMBLUCSC | 140 | 4 | 27116 | tss;island | 0.366939 | 0.101419 | Not queried | 1.99 | 2.00e-01 | 2.55e-01 |
|  | 19 | 42437245 | 42437425 | PDFSTATSENSEMBLUCSC | 181 | 4 | 22060 | island | 0.919383 | 0.978807 | Not queried | 1.99 | 2.86e-02 | 6.12e-02 |
|  | 16 | 1014818 | 1015103 | PDFSTATSENSEMBLUCSC | 286 | 4 | 14556 | gene;island | 0.914355 | 0.982299 | Not queried | 1.99 | 2.86e-02 | 6.12e-02 |
|  | 2 | 74357527 | 74357872 | PDFSTATSENSEMBLUCSC | 346 | 4 | 24425 | island | 0.922362 | 0.627296 | Not queried | 1.99 | 2.00e-01 | 2.55e-01 |
|  | 5 | 114961180 | 114961559 | PDFSTATSENSEMBLUCSC | 380 | 4 | 31722 | gene;island | 0.001304 | 0.005049 | Not queried | 1.99 | 1.14e-01 | 1.68e-01 |
|  | 3 | 119379414 | 119379792 | PDFSTATSENSEMBLUCSC | 379 | 4 | 28524 | tss | 0.773346 | 0.911034 | Not queried | 1.99 | 3.43e-01 | 3.84e-01 |
|  | 11 | 49914985 | 49915371 | PDFSTATSENSEMBLUCSC | 387 | 4 | 8277 | island | 0.637967 | 0.295246 | Not queried | 1.99 | 5.71e-02 | 1.00e-01 |
|  | 4 | 57773149 | 57773436 | PDFSTATSENSEMBLUCSC | 288 | 5 | 30412 | tss;island | 0.648188 | 0.316648 | Not queried | 1.99 | 2.22e-01 | 2.76e-01 |
|  | 7 | 149322723 | 149323101 | PDFSTATSENSEMBLUCSC | 379 | 5 | 37969 | tss;island | 0.019150 | 0.002839 | Not queried | 1.99 | 3.10e-01 | 3.66e-01 |
|  | 7 | 157791721 | 157792100 | PDFSTATSENSEMBLUCSC | 380 | 4 | 37356 | gene | 0.865799 | 0.969160 | Not queried | 1.99 | 1.14e-01 | 1.68e-01 |
|  | 4 | 155253864 | 155254602 | PDFSTATSENSEMBLUCSC | 739 | 6 | 30156 | gene;island | 0.614175 | 0.875970 | Not queried | 1.99 | 9.31e-02 | 1.53e-01 |
|  | 18 | 32557882 | 32558112 | PDFSTATSENSEMBLUCSC | 231 | 4 | 19087 | gene;tss;island | 0.116346 | 0.177744 | Not queried | 1.99 | 4.86e-01 | 5.11e-01 |
|  | 4 | 54931064 | 54931210 | PDFSTATSENSEMBLUCSC | 147 | 4 | 30398 | tss;island | 0.002824 | 0.001567 | Not queried | 1.99 | 1.00e+00 | 1.00e+00 |
|  | 1 | 180204221 | 180204499 | PDFSTATSENSEMBLUCSC | 279 | 4 | 906 | gene;island | 0.806079 | 0.933026 | Not queried | 1.99 | 4.86e-01 | 5.11e-01 |
|  | 20 | 55107833 | 55108228 | PDFSTATSENSEMBLUCSC | 396 | 7 | 25838 | tss | 0.732104 | 0.951307 | Not queried | 1.99 | 7.28e-02 | 1.23e-01 |
|  | 1 | 39957387 | 39957535 | PDFSTATSENSEMBLUCSC | 149 | 5 | 447 | gene | 0.109831 | 0.022850 | Not queried | 1.99 | 3.17e-02 | 6.50e-02 |
|  | 6 | 26457928 | 26458121 | PDFSTATSENSEMBLUCSC | 194 | 5 | 34706 | tss | 0.000962 | 0.018513 | Not queried | 1.99 | 3.10e-01 | 3.66e-01 |
|  | 1 | 228604037 | 228604595 | PDFSTATSENSEMBLUCSC | 559 | 6 | 4296 | island | 0.883793 | 0.962260 | Not queried | 1.99 | 2.60e-02 | 6.12e-02 |
|  | 22 | 42828411 | 42828516 | PDFSTATSENSEMBLUCSC | 106 | 5 | 27190 | tss | 0.935702 | 0.982992 | Not queried | 1.99 | 1.59e-02 | 5.36e-02 |
|  | 2 | 85766104 | 85766511 | PDFSTATSENSEMBLUCSC | 408 | 6 | 24480 | island | 0.004324 | 0.011785 | Not queried | 1.99 | 2.40e-01 | 2.95e-01 |
|  | 5 | 161274307 | 161275138 | PDFSTATSENSEMBLUCSC | 832 | 9 | 31879 | gene;tss | 0.678532 | 0.287127 | Not queried | 1.99 | 1.36e-01 | 1.94e-01 |
|  | 6 | 32025886 | 32026350 | PDFSTATSENSEMBLUCSC | 465 | 10 | 34022 | gene | 0.677187 | 0.948425 | Not queried | 1.99 | 1.47e-02 | 5.36e-02 |
|  | 1 | 34642000 | 34642519 | PDFSTATSENSEMBLUCSC | 520 | 8 | 1477 | tss | 0.867860 | 0.966249 | Not queried | 1.99 | 1.05e-01 | 1.66e-01 |
|  | 6 | 117584596 | 117584815 | PDFSTATSENSEMBLUCSC | 220 | 4 | 36435 | island | 0.646857 | 0.905132 | Not queried | 1.99 | 6.86e-01 | 6.99e-01 |
|  | 2 | 238777587 | 238777806 | PDFSTATSENSEMBLUCSC | 220 | 4 | 23093 | gene | 0.838169 | 0.971821 | Not queried | 1.99 | 3.43e-01 | 3.84e-01 |
|  | 8 | 122651271 | 122651575 | PDFSTATSENSEMBLUCSC | 305 | 8 | 39413 | gene;tss;island | 0.299848 | 0.606112 | Not queried | 1.99 | 8.30e-02 | 1.40e-01 |
|  | 19 | 3671903 | 3672411 | PDFSTATSENSEMBLUCSC | 509 | 4 | 19719 | gene;island | 0.537345 | 0.788373 | Not queried | 1.99 | 3.43e-01 | 3.84e-01 |
|  | 17 | 6947288 | 6947464 | PDFSTATSENSEMBLUCSC | 177 | 8 | 17060 | tss | 0.852850 | 0.915995 | SLC16A11 (margin: SLC16A13; SLC16A11) | 1.99 | 6.22e-04 | 6.71e-03 |
|  | 13 | 28491326 | 28491975 | PDFSTATSENSEMBLUCSC | 650 | 7 | 11632 | island | 0.749220 | 0.956558 | Not queried | 1.99 | 1.28e-01 | 1.87e-01 |
|  | 3 | 169490563 | 169491284 | PDFSTATSENSEMBLUCSC | 722 | 9 | 29649 | island | 0.005121 | 0.009245 | Not queried | 1.99 | 1.90e-01 | 2.55e-01 |
|  | 12 | 22094963 | 22095477 | PDFSTATSENSEMBLUCSC | 515 | 5 | 10282 | island | 0.844000 | 0.980598 | Not queried | 1.99 | 3.17e-02 | 6.50e-02 |
|  | 17 | 79905152 | 79905319 | PDFSTATSENSEMBLUCSC | 168 | 7 | 17762 | tss | 0.769454 | 0.970782 | Not queried | 1.99 | 9.73e-02 | 1.55e-01 |
|  | 7 | 4243286 | 4243734 | PDFSTATSENSEMBLUCSC | 449 | 5 | 36834 | gene | 0.663425 | 0.835293 | Not queried | 1.99 | 9.52e-02 | 1.53e-01 |
|  | 1 | 2014244 | 2014628 | PDFSTATSENSEMBLUCSC | 385 | 4 | 58 | gene | 0.810748 | 0.906375 | Not queried | 1.99 | 1.14e-01 | 1.68e-01 |
|  | 7 | 1641705 | 1641922 | PDFSTATSENSEMBLUCSC | 218 | 4 | 38120 | island | 0.337641 | 0.667706 | Not queried | 1.99 | 2.86e-02 | 6.12e-02 |
|  | 6 | 169242769 | 169243075 | PDFSTATSENSEMBLUCSC | 307 | 4 | 36668 | island | 0.613553 | 0.904819 | Not queried | 1.99 | 3.43e-01 | 3.84e-01 |
|  | 1 | 152595842 | 152596177 | PDFSTATSENSEMBLUCSC | 336 | 5 | 1938 | tss | 0.786318 | 0.935041 | Not queried | 1.99 | 2.22e-01 | 2.76e-01 |
|  | 15 | 25068564 | 25068790 | PDFSTATSENSEMBLUCSC | 227 | 7 | 13518 | tss | 0.827490 | 0.526454 | Not queried | 1.99 | 2.59e-01 | 3.16e-01 |
|  | 6 | 134209935 | 134210307 | PDFSTATSENSEMBLUCSC | 373 | 9 | 36489 | island | 0.391856 | 0.750164 | Not queried | 1.99 | 1.61e-01 | 2.25e-01 |
|  | 17 | 21367002 | 21367564 | PDFSTATSENSEMBLUCSC | 563 | 5 | 18122 | island | 0.795881 | 0.949245 | Not queried | 1.99 | 1.51e-01 | 2.11e-01 |
|  | 1 | 46951291 | 46951683 | PDFSTATSENSEMBLUCSC | 393 | 6 | 3219 | island | 0.936853 | 0.981450 | (margin: LINC00505) | 1.99 | 2.16e-03 | 1.45e-02 |
|  | 1 | 149856997 | 149857491 | PDFSTATSENSEMBLUCSC | 495 | 7 | 733 | gene;island | 0.001209 | 0.005357 | Not queried | 1.98 | 9.73e-02 | 1.55e-01 |
|  | 6 | 33165404 | 33165899 | PDFSTATSENSEMBLUCSC | 496 | 9 | 34219 | gene;island | 0.861448 | 0.475988 | Not queried | 1.98 | 1.61e-01 | 2.25e-01 |
|  | 22 | 46731652 | 46731750 | PDFSTATSENSEMBLUCSC | 99 | 4 | 26912 | gene;island | 0.002812 | 0.003636 | Not queried | 1.98 | 3.43e-01 | 3.84e-01 |
|  | 6 | 122931154 | 122931702 | PDFSTATSENSEMBLUCSC | 549 | 11 | 34463 | gene;island | 0.073470 | 0.019806 | PKIB (margin: PKIB) | 1.98 | 3.94e-04 | 5.86e-03 |
|  | 1 | 6052593 | 6052770 | PDFSTATSENSEMBLUCSC | 178 | 4 | 1195 | tss | 0.003452 | 0.006101 | Not queried | 1.98 | 4.86e-01 | 5.11e-01 |
|  | 11 | 76381044 | 76381236 | PDFSTATSENSEMBLUCSC | 193 | 6 | 7576 | tss | 0.549168 | 0.789045 | Not queried | 1.98 | 4.85e-01 | 5.11e-01 |
|  | 7 | 95115026 | 95115246 | PDFSTATSENSEMBLUCSC | 221 | 5 | 37729 | tss | 0.950614 | 0.869864 | Not queried | 1.98 | 4.21e-01 | 4.59e-01 |
|  | 3 | 169781404 | 169781717 | PDFSTATSENSEMBLUCSC | 314 | 5 | 28027 | gene | 0.909268 | 0.971464 | Not queried | 1.98 | 1.51e-01 | 2.11e-01 |
|  | 4 | 110223040 | 110224288 | PDFSTATSENSEMBLUCSC | 1249 | 20 | 31235 | island | 0.754867 | 0.672070 | Not queried | 1.98 | 7.79e-01 | 7.88e-01 |
|  | 7 | 157593864 | 157594102 | PDFSTATSENSEMBLUCSC | 239 | 4 | 37345 | gene;island | 0.766212 | 0.953199 | Not queried | 1.98 | 3.43e-01 | 3.84e-01 |
|  | 11 | 132864104 | 132864357 | PDFSTATSENSEMBLUCSC | 254 | 4 | 6982 | gene | 0.609134 | 0.350557 | Not queried | 1.98 | 8.86e-01 | 8.89e-01 |
|  | 1 | 181074635 | 181074948 | PDFSTATSENSEMBLUCSC | 314 | 4 | 4015 | island | 0.242890 | 0.038521 | Not queried | 1.98 | 8.86e-01 | 8.89e-01 |
|  | 11 | 2018088 | 2018247 | PDFSTATSENSEMBLUCSC | 160 | 7 | 7053 | tss | 0.694402 | 0.915457 | Not queried | 1.98 | 5.30e-02 | 1.00e-01 |
|  | 12 | 247224 | 247459 | PDFSTATSENSEMBLUCSC | 236 | 4 | 8990 | gene;island | 0.740212 | 0.921665 | Not queried | 1.98 | 1.14e-01 | 1.68e-01 |
|  | 12 | 4382980 | 4383281 | PDFSTATSENSEMBLUCSC | 302 | 5 | 9012 | gene | 0.362302 | 0.060271 | Not queried | 1.98 | 5.56e-02 | 1.00e-01 |
|  | 11 | 74442547 | 74442900 | PDFSTATSENSEMBLUCSC | 354 | 4 | 7561 | tss;island | 0.810570 | 0.954758 | Not queried | 1.98 | 1.14e-01 | 1.68e-01 |
|  | 19 | 53496835 | 53497048 | PDFSTATSENSEMBLUCSC | 214 | 5 | 21022 | tss | 0.640105 | 0.289895 | Not queried | 1.98 | 1.51e-01 | 2.11e-01 |
|  | 19 | 12848092 | 12848303 | PDFSTATSENSEMBLUCSC | 212 | 5 | 20509 | tss | 0.000556 | 0.003761 | Not queried | 1.98 | 5.48e-01 | 5.70e-01 |
|  | 5 | 140734543 | 140734758 | PDFSTATSENSEMBLUCSC | 216 | 4 | 32375 | tss | 0.579866 | 0.920874 | Not queried | 1.98 | 4.86e-01 | 5.11e-01 |
|  | 11 | 2597528 | 2597838 | PDFSTATSENSEMBLUCSC | 311 | 4 | 6465 | gene;island | 0.756816 | 0.976189 | Not queried | 1.98 | 1.14e-01 | 1.68e-01 |
|  | 19 | 47617042 | 47617301 | PDFSTATSENSEMBLUCSC | 260 | 7 | 20902 | tss;island | 0.002949 | 0.014567 | Not queried | 1.98 | 1.65e-01 | 2.28e-01 |
|  | 11 | 134582289 | 134582508 | PDFSTATSENSEMBLUCSC | 220 | 4 | 8989 | island | 0.597923 | 0.879241 | Not queried | 1.98 | 3.43e-01 | 3.84e-01 |
|  | 11 | 115375341 | 115375432 | PDFSTATSENSEMBLUCSC | 92 | 4 | 7709 | tss | 0.037371 | 0.019003 | Not queried | 1.98 | 3.43e-01 | 3.84e-01 |
|  | 7 | 153584416 | 153584873 | PDFSTATSENSEMBLUCSC | 458 | 7 | 39053 | island | 0.965573 | 0.805050 | Not queried | 1.98 | 1.75e-02 | 5.79e-02 |
|  | 5 | 82767280 | 82767450 | PDFSTATSENSEMBLUCSC | 171 | 4 | 32159 | tss | 0.902201 | 0.970622 | Not queried | 1.97 | 5.71e-02 | 1.00e-01 |
|  | 1 | 45242073 | 45242356 | PDFSTATSENSEMBLUCSC | 284 | 4 | 496 | gene;tss;island | 0.008883 | 0.020413 | Not queried | 1.97 | 4.86e-01 | 5.11e-01 |
|  | 19 | 58459097 | 58459358 | PDFSTATSENSEMBLUCSC | 262 | 8 | 21130 | tss | 0.028321 | 0.007081 | Not queried | 1.97 | 1.48e-02 | 5.36e-02 |
|  | 17 | 57970442 | 57970656 | PDFSTATSENSEMBLUCSC | 215 | 4 | 16717 | gene | 0.004765 | 0.020286 | Not queried | 1.97 | 3.43e-01 | 3.84e-01 |
|  | 17 | 52977769 | 52978209 | PDFSTATSENSEMBLUCSC | 441 | 9 | 18572 | island | 0.053379 | 0.040296 | Not queried | 1.97 | 1.61e-01 | 2.25e-01 |
|  | 11 | 16628281 | 16628944 | PDFSTATSENSEMBLUCSC | 664 | 6 | 8089 | island | 0.382543 | 0.919096 | Not queried | 1.97 | 1.32e-01 | 1.89e-01 |
|  | 10 | 90750169 | 90750580 | PDFSTATSENSEMBLUCSC | 412 | 7 | 4669 | gene;island | 0.007729 | 0.035683 | FAS; ACTA2 (margin: FAS; ACTA2) | 1.97 | 1.11e-02 | 4.14e-02 |
|  | 11 | 124735089 | 124735227 | PDFSTATSENSEMBLUCSC | 139 | 7 | 7786 | tss | 0.909641 | 0.972381 | Not queried | 1.97 | 1.28e-01 | 1.87e-01 |
|  | 2 | 162283434 | 162284206 | PDFSTATSENSEMBLUCSC | 773 | 8 | 24823 | island | 0.902938 | 0.976036 | TBR1; AC009487.5; RP11-334E15.2 (margin: TBR1; AC009487.5; RP11-334E15.2; AC009487.4) | 1.97 | 1.09e-03 | 1.10e-02 |
|  | 4 | 74847758 | 74848016 | PDFSTATSENSEMBLUCSC | 259 | 6 | 30431 | tss | 0.691967 | 0.557629 | Not queried | 1.97 | 5.89e-01 | 6.11e-01 |
|  | 12 | 12418943 | 12419209 | PDFSTATSENSEMBLUCSC | 267 | 4 | 9056 | gene;island | 0.044257 | 0.202643 | Not queried | 1.97 | 5.71e-02 | 1.00e-01 |
|  | 15 | 66795595 | 66795844 | PDFSTATSENSEMBLUCSC | 250 | 4 | 13389 | gene;tss | 0.229591 | 0.522873 | Not queried | 1.97 | 2.00e-01 | 2.55e-01 |
|  | 2 | 223177287 | 223177785 | PDFSTATSENSEMBLUCSC | 499 | 7 | 25151 | island | 0.651622 | 0.880061 | Not queried | 1.97 | 3.83e-01 | 4.27e-01 |
|  | 14 | 23815798 | 23816109 | PDFSTATSENSEMBLUCSC | 312 | 5 | 12028 | gene;island | 0.592850 | 0.924736 | Not queried | 1.97 | 5.56e-02 | 1.00e-01 |
|  | 1 | 89357317 | 89357426 | PDFSTATSENSEMBLUCSC | 110 | 4 | 1736 | tss | 0.000606 | 0.007726 | Not queried | 1.97 | 8.86e-01 | 8.89e-01 |
|  | 7 | 42267269 | 42267747 | PDFSTATSENSEMBLUCSC | 479 | 7 | 36976 | gene;island | 0.918732 | 0.973505 | Not queried | 1.97 | 9.73e-02 | 1.55e-01 |
|  | 2 | 217236783 | 217237043 | PDFSTATSENSEMBLUCSC | 261 | 10 | 23867 | tss | 0.118474 | 0.052272 | Not queried | 1.97 | 4.33e-02 | 8.55e-02 |
|  | 5 | 41870796 | 41871189 | PDFSTATSENSEMBLUCSC | 394 | 9 | 32038 | tss | 0.301881 | 0.587567 | Not queried | 1.97 | 1.61e-01 | 2.25e-01 |
|  | 20 | 48552575 | 48552853 | PDFSTATSENSEMBLUCSC | 279 | 7 | 25813 | tss;island | 0.016507 | 0.027413 | Not queried | 1.97 | 9.02e-01 | 9.05e-01 |
|  | 11 | 35547074 | 35547311 | PDFSTATSENSEMBLUCSC | 238 | 4 | 8209 | island | 0.504774 | 0.218561 | Not queried | 1.97 | 1.14e-01 | 1.68e-01 |
|  | 1 | 63795636 | 63795970 | PDFSTATSENSEMBLUCSC | 335 | 5 | 3344 | island | 0.569922 | 0.920874 | Not queried | 1.97 | 1.51e-01 | 2.11e-01 |
|  | 15 | 41221376 | 41221546 | PDFSTATSENSEMBLUCSC | 171 | 4 | 14053 | island | 0.056599 | 0.013618 | Not queried | 1.97 | 2.00e-01 | 2.55e-01 |
|  | 6 | 134209935 | 134210257 | PDFSTATSENSEMBLUCSC | 323 | 6 | 35232 | tss | 0.617137 | 0.851921 | Not queried | 1.97 | 4.85e-01 | 5.11e-01 |
|  | 1 | 95698827 | 95699097 | PDFSTATSENSEMBLUCSC | 271 | 5 | 1770 | tss;island | 0.760771 | 0.982917 | Not queried | 1.97 | 2.22e-01 | 2.76e-01 |
|  | 7 | 96653617 | 96654132 | PDFSTATSENSEMBLUCSC | 516 | 5 | 37107 | gene | 0.469791 | 0.212163 | Not queried | 1.97 | 3.10e-01 | 3.66e-01 |
|  | 10 | 102440587 | 102440894 | PDFSTATSENSEMBLUCSC | 308 | 4 | 6030 | island | 0.056806 | 0.023393 | Not queried | 1.97 | 4.86e-01 | 5.11e-01 |
|  | 15 | 25841831 | 25842111 | PDFSTATSENSEMBLUCSC | 281 | 4 | 13956 | island | 0.673539 | 0.953517 | Not queried | 1.97 | 2.00e-01 | 2.55e-01 |
|  | 21 | 36042170 | 36042464 | PDFSTATSENSEMBLUCSC | 295 | 4 | 26411 | gene;island | 0.706281 | 0.887830 | Not queried | 1.97 | 1.14e-01 | 1.68e-01 |
|  | 17 | 48585470 | 48585645 | PDFSTATSENSEMBLUCSC | 176 | 4 | 17501 | tss;island | 0.901358 | 0.980926 | Not queried | 1.97 | 2.86e-02 | 6.12e-02 |
|  | 6 | 106533925 | 106534146 | PDFSTATSENSEMBLUCSC | 222 | 6 | 35147 | tss | 0.009918 | 0.054253 | Not queried | 1.97 | 2.60e-02 | 6.12e-02 |
|  | 15 | 42447840 | 42448259 | PDFSTATSENSEMBLUCSC | 420 | 5 | 13308 | gene | 0.825343 | 0.640152 | Not queried | 1.97 | 5.48e-01 | 5.70e-01 |
|  | 2 | 145277596 | 145277852 | PDFSTATSENSEMBLUCSC | 257 | 9 | 22891 | gene;island | 0.183263 | 0.083278 | Not queried | 1.97 | 7.70e-02 | 1.30e-01 |
|  | 14 | 97499908 | 97500345 | PDFSTATSENSEMBLUCSC | 438 | 4 | 13122 | island | 0.677380 | 0.924494 | Not queried | 1.97 | 6.86e-01 | 6.99e-01 |
|  | 6 | 34725116 | 34725359 | PDFSTATSENSEMBLUCSC | 244 | 5 | 34305 | gene;island | 0.002125 | 0.009213 | Not queried | 1.97 | 4.21e-01 | 4.59e-01 |
|  | 12 | 120933726 | 120933893 | PDFSTATSENSEMBLUCSC | 168 | 7 | 9354 | gene;island | 0.004209 | 0.013671 | Not queried | 1.97 | 2.59e-01 | 3.16e-01 |
|  | 5 | 115911760 | 115912383 | PDFSTATSENSEMBLUCSC | 624 | 6 | 33033 | island | 0.563613 | 0.198471 | Not queried | 1.97 | 1.80e-01 | 2.45e-01 |
|  | 12 | 54393966 | 54394212 | PDFSTATSENSEMBLUCSC | 247 | 5 | 9157 | gene;island | 0.797168 | 0.510284 | Not queried | 1.97 | 1.59e-02 | 5.36e-02 |
|  | 19 | 11784955 | 11785337 | PDFSTATSENSEMBLUCSC | 383 | 6 | 19848 | gene | 0.895321 | 0.966397 | Not queried | 1.97 | 1.80e-01 | 2.45e-01 |
|  | 2 | 220282914 | 220283214 | PDFSTATSENSEMBLUCSC | 301 | 6 | 25123 | island | 0.894393 | 0.958235 | Not queried | 1.97 | 1.80e-01 | 2.45e-01 |
|  | 7 | 97736036 | 97736138 | PDFSTATSENSEMBLUCSC | 103 | 4 | 37742 | tss;island | 0.002372 | 0.008760 | Not queried | 1.97 | 2.86e-02 | 6.12e-02 |
|  | 1 | 247275735 | 247276096 | PDFSTATSENSEMBLUCSC | 362 | 5 | 2367 | tss | 0.762899 | 0.337959 | Not queried | 1.96 | 2.22e-01 | 2.76e-01 |
|  | 11 | 2907670 | 2908471 | PDFSTATSENSEMBLUCSC | 802 | 14 | 7081 | tss;island | 0.891219 | 0.972002 | CDKN1C; SLC22A18AS (margin: CDKN1C; SLC22A18AS) | 1.96 | 1.22e-02 | 4.56e-02 |
|  | 2 | 208031734 | 208031995 | PDFSTATSENSEMBLUCSC | 262 | 5 | 23841 | tss;island | 0.022862 | 0.034858 | Not queried | 1.96 | 1.51e-01 | 2.11e-01 |
|  | 2 | 27072271 | 27072564 | PDFSTATSENSEMBLUCSC | 294 | 4 | 22617 | gene;island | 0.761033 | 0.944847 | Not queried | 1.96 | 2.00e-01 | 2.55e-01 |
|  | 14 | 35761633 | 35761922 | PDFSTATSENSEMBLUCSC | 290 | 4 | 12064 | gene | 0.004011 | 0.010207 | Not queried | 1.96 | 1.14e-01 | 1.68e-01 |
|  | 7 | 102105124 | 102105256 | PDFSTATSENSEMBLUCSC | 133 | 5 | 37168 | gene | 0.005071 | 0.010567 | Not queried | 1.96 | 2.22e-01 | 2.76e-01 |
|  | 4 | 8345155 | 8345588 | PDFSTATSENSEMBLUCSC | 434 | 4 | 30919 | island | 0.959539 | 0.876537 | Not queried | 1.96 | 1.14e-01 | 1.68e-01 |
|  | 5 | 56206110 | 56206289 | PDFSTATSENSEMBLUCSC | 180 | 4 | 31621 | gene;island | 0.007873 | 0.015097 | Not queried | 1.96 | 3.43e-01 | 3.84e-01 |
|  | 8 | 25898191 | 25898539 | PDFSTATSENSEMBLUCSC | 349 | 4 | 39250 | gene;island | 0.939343 | 0.979251 | Not queried | 1.96 | 5.71e-02 | 1.00e-01 |
|  | 4 | 174427388 | 174428191 | PDFSTATSENSEMBLUCSC | 804 | 9 | 31424 | island | 0.639794 | 0.849239 | Not queried | 1.96 | 2.58e-01 | 3.16e-01 |
|  | 14 | 90722653 | 90722861 | PDFSTATSENSEMBLUCSC | 209 | 5 | 12553 | tss | 0.001946 | 0.025244 | Not queried | 1.96 | 5.48e-01 | 5.70e-01 |
|  | 11 | 60414615 | 60415111 | PDFSTATSENSEMBLUCSC | 497 | 5 | 6621 | gene;island | 0.806400 | 0.934736 | Not queried | 1.96 | 2.22e-01 | 2.76e-01 |
|  | 16 | 57496324 | 57496545 | PDFSTATSENSEMBLUCSC | 222 | 5 | 15263 | tss | 0.006663 | 0.026015 | Not queried | 1.96 | 2.22e-01 | 2.76e-01 |
|  | 21 | 46367939 | 46368313 | PDFSTATSENSEMBLUCSC | 375 | 5 | 26462 | gene;island | 0.920680 | 0.988530 | Not queried | 1.96 | 2.22e-01 | 2.76e-01 |
|  | 19 | 46800338 | 46800602 | PDFSTATSENSEMBLUCSC | 265 | 4 | 20108 | gene | 0.943143 | 0.983105 | Not queried | 1.96 | 1.14e-01 | 1.68e-01 |
|  | 20 | 19998368 | 19998558 | PDFSTATSENSEMBLUCSC | 191 | 6 | 25370 | gene;island | 0.016641 | 0.058570 | Not queried | 1.96 | 6.49e-02 | 1.11e-01 |
|  | 8 | 43131260 | 43131656 | PDFSTATSENSEMBLUCSC | 397 | 5 | 40267 | island | 0.923149 | 0.976531 | Not queried | 1.96 | 1.51e-01 | 2.11e-01 |
|  | 19 | 48216390 | 48216613 | PDFSTATSENSEMBLUCSC | 224 | 6 | 22200 | island | 0.003034 | 0.007527 | Not queried | 1.96 | 3.10e-01 | 3.66e-01 |
|  | 8 | 143359879 | 143360290 | PDFSTATSENSEMBLUCSC | 412 | 4 | 39456 | gene;island | 0.673847 | 0.927074 | Not queried | 1.96 | 3.43e-01 | 3.84e-01 |
|  | 19 | 53073242 | 53074012 | PDFSTATSENSEMBLUCSC | 771 | 11 | 22360 | island | 0.772057 | 0.438828 | ZNF701 (margin: ZNF808; ZNF701; RPL39P34; CTD-3099C6.7) | 1.96 | 2.75e-04 | 4.46e-03 |
|  | 11 | 27015656 | 27015991 | PDFSTATSENSEMBLUCSC | 336 | 5 | 6541 | gene | 0.635424 | 0.505029 | Not queried | 1.96 | 4.21e-01 | 4.59e-01 |
|  | 11 | 67777618 | 67777770 | PDFSTATSENSEMBLUCSC | 153 | 6 | 7503 | tss | 0.021765 | 0.087019 | ALDH3B1 (margin: ALDH3B1; UNC93B1) | 1.96 | 4.33e-03 | 2.52e-02 |
|  | 3 | 4534791 | 4535021 | PDFSTATSENSEMBLUCSC | 231 | 7 | 28117 | tss | 0.001777 | 0.007499 | Not queried | 1.96 | 7.28e-02 | 1.23e-01 |
|  | 17 | 61627082 | 61627772 | PDFSTATSENSEMBLUCSC | 691 | 9 | 17565 | tss | 0.007888 | 0.012486 | Not queried | 1.96 | 2.58e-01 | 3.16e-01 |
|  | 1 | 42921582 | 42921899 | PDFSTATSENSEMBLUCSC | 318 | 5 | 465 | gene | 0.064031 | 0.020326 | Not queried | 1.96 | 5.48e-01 | 5.70e-01 |
|  | 11 | 58830191 | 58830610 | PDFSTATSENSEMBLUCSC | 420 | 4 | 8300 | island | 0.890685 | 0.969501 | Not queried | 1.96 | 4.86e-01 | 5.11e-01 |
|  | 5 | 5034274 | 5034397 | PDFSTATSENSEMBLUCSC | 124 | 5 | 31978 | tss | 0.514007 | 0.214474 | Not queried | 1.95 | 8.41e-01 | 8.48e-01 |
|  | 12 | 107974186 | 107974612 | PDFSTATSENSEMBLUCSC | 427 | 8 | 9976 | tss | 0.718749 | 0.716590 | Not queried | 1.95 | 4.42e-01 | 4.81e-01 |
|  | 12 | 123464511 | 123464932 | PDFSTATSENSEMBLUCSC | 422 | 6 | 10941 | island | 0.051859 | 0.122625 | Not queried | 1.95 | 3.94e-01 | 4.36e-01 |
|  | 12 | 57943724 | 57944059 | PDFSTATSENSEMBLUCSC | 336 | 4 | 10564 | island | 0.779740 | 0.477983 | Not queried | 1.95 | 1.14e-01 | 1.68e-01 |
|  | 5 | 1077533 | 1077845 | PDFSTATSENSEMBLUCSC | 313 | 4 | 31519 | gene;island | 0.819032 | 0.942580 | Not queried | 1.95 | 1.14e-01 | 1.68e-01 |
|  | 3 | 21447391 | 21447667 | PDFSTATSENSEMBLUCSC | 277 | 4 | 27699 | gene | 0.773540 | 0.445558 | Not queried | 1.95 | 2.00e-01 | 2.55e-01 |
|  | 15 | 74218418 | 74218780 | PDFSTATSENSEMBLUCSC | 363 | 9 | 13785 | tss | 0.881955 | 0.967210 | LOXL1; LOXL1-AS1 (margin: LOXL1; LOXL1-AS1) | 1.95 | 7.82e-04 | 8.17e-03 |
|  | 4 | 10686394 | 10686610 | PDFSTATSENSEMBLUCSC | 217 | 4 | 30301 | tss | 0.473553 | 0.722361 | Not queried | 1.95 | 4.86e-01 | 5.11e-01 |
|  | 4 | 100867933 | 100868318 | PDFSTATSENSEMBLUCSC | 386 | 4 | 30513 | tss | 0.004176 | 0.014133 | Not queried | 1.95 | 2.00e-01 | 2.55e-01 |
|  | 20 | 46130313 | 46131144 | PDFSTATSENSEMBLUCSC | 832 | 13 | 26223 | island | 0.005908 | 0.010315 | Not queried | 1.95 | 1.01e-01 | 1.62e-01 |
|  | 14 | 53173891 | 53174235 | PDFSTATSENSEMBLUCSC | 345 | 5 | 12845 | island | 0.003031 | 0.011678 | Not queried | 1.95 | 9.52e-02 | 1.53e-01 |
|  | 8 | 68864012 | 68864549 | PDFSTATSENSEMBLUCSC | 538 | 9 | 39751 | tss;island | 0.818099 | 0.941209 | Not queried | 1.95 | 2.44e-02 | 6.12e-02 |
|  | 5 | 39425138 | 39425549 | PDFSTATSENSEMBLUCSC | 412 | 8 | 32748 | island | 0.273785 | 0.048509 | Not queried | 1.95 | 5.74e-01 | 5.97e-01 |
|  | 6 | 53213948 | 53214258 | PDFSTATSENSEMBLUCSC | 311 | 8 | 35039 | tss | 0.192090 | 0.041878 | Not queried | 1.95 | 6.50e-02 | 1.11e-01 |
|  | 16 | 28270490 | 28270754 | PDFSTATSENSEMBLUCSC | 265 | 4 | 15785 | island | 0.847795 | 0.965833 | Not queried | 1.95 | 8.86e-01 | 8.89e-01 |
|  | 19 | 836606 | 836898 | PDFSTATSENSEMBLUCSC | 293 | 4 | 21177 | island | 0.760377 | 0.920477 | Not queried | 1.95 | 3.43e-01 | 3.84e-01 |
|  | 19 | 21950196 | 21950478 | PDFSTATSENSEMBLUCSC | 283 | 6 | 21839 | island | 0.331729 | 0.115527 | Not queried | 1.95 | 3.94e-01 | 4.36e-01 |
|  | 10 | 15130785 | 15131036 | PDFSTATSENSEMBLUCSC | 252 | 6 | 4961 | tss | 0.079129 | 0.006912 | Not queried | 1.95 | 1.52e-02 | 5.36e-02 |
|  | 7 | 45927735 | 45928326 | PDFSTATSENSEMBLUCSC | 592 | 8 | 38477 | island | 0.722504 | 0.935542 | Not queried | 1.95 | 3.79e-02 | 7.66e-02 |
|  | 13 | 113729242 | 113729446 | PDFSTATSENSEMBLUCSC | 205 | 4 | 11274 | gene;island | 0.857213 | 0.968003 | Not queried | 1.95 | 2.86e-02 | 6.12e-02 |
|  | 16 | 75032477 | 75032853 | PDFSTATSENSEMBLUCSC | 377 | 6 | 15357 | tss;island | 0.013189 | 0.022159 | Not queried | 1.95 | 1.80e-01 | 2.45e-01 |
|  | 1 | 166845374 | 166845621 | PDFSTATSENSEMBLUCSC | 248 | 4 | 870 | gene | 0.008565 | 0.016833 | Not queried | 1.95 | 4.86e-01 | 5.11e-01 |
|  | 6 | 86352429 | 86352639 | PDFSTATSENSEMBLUCSC | 211 | 5 | 34406 | gene;tss;island | 0.082851 | 0.017686 | Not queried | 1.95 | 9.52e-02 | 1.53e-01 |
|  | 5 | 50265272 | 50265767 | PDFSTATSENSEMBLUCSC | 496 | 5 | 32786 | island | 0.941585 | 0.745111 | Not queried | 1.95 | 5.56e-02 | 1.00e-01 |
|  | 11 | 31823141 | 31823282 | PDFSTATSENSEMBLUCSC | 142 | 4 | 6558 | gene;island | 0.707700 | 0.969221 | Not queried | 1.95 | 4.86e-01 | 5.11e-01 |
|  | 6 | 166582159 | 166582585 | PDFSTATSENSEMBLUCSC | 427 | 9 | 35334 | tss;island | 0.849003 | 0.957262 | Not queried | 1.95 | 1.90e-01 | 2.55e-01 |
|  | 18 | 72265164 | 72265361 | PDFSTATSENSEMBLUCSC | 198 | 8 | 19325 | tss | 0.001047 | 0.003553 | Not queried | 1.95 | 4.42e-01 | 4.81e-01 |
|  | 6 | 56112392 | 56112696 | PDFSTATSENSEMBLUCSC | 305 | 8 | 35044 | tss | 0.261109 | 0.411116 | Not queried | 1.95 | 1.48e-02 | 5.36e-02 |
|  | 8 | 1960217 | 1960466 | PDFSTATSENSEMBLUCSC | 250 | 4 | 40046 | island | 0.340676 | 0.139241 | Not queried | 1.95 | 6.86e-01 | 6.99e-01 |
|  | 20 | 61147572 | 61147652 | PDFSTATSENSEMBLUCSC | 81 | 5 | 25873 | tss | 0.415745 | 0.699681 | Not queried | 1.94 | 9.52e-02 | 1.53e-01 |
|  | 17 | 60704382 | 60704698 | PDFSTATSENSEMBLUCSC | 317 | 7 | 17561 | tss;island | 0.096908 | 0.225622 | Not queried | 1.94 | 1.28e-01 | 1.87e-01 |
|  | 6 | 31322577 | 31322996 | PDFSTATSENSEMBLUCSC | 420 | 5 | 33867 | gene;island | 0.985101 | 0.854994 | Not queried | 1.94 | 1.51e-01 | 2.11e-01 |
|  | 1 | 9258678 | 9258836 | PDFSTATSENSEMBLUCSC | 159 | 4 | 2647 | island | 0.899697 | 0.967212 | Not queried | 1.94 | 2.86e-02 | 6.12e-02 |
|  | 18 | 47013750 | 47014260 | PDFSTATSENSEMBLUCSC | 511 | 6 | 19269 | tss;island | 0.047607 | 0.080460 | Not queried | 1.94 | 6.99e-01 | 7.09e-01 |
|  | 15 | 74659901 | 74660566 | PDFSTATSENSEMBLUCSC | 666 | 7 | 13792 | tss;island | 0.489115 | 0.745669 | CTD-2311M21.3; CYP11A1 (margin: CTD-2311M21.2; CTD-2311M21.3; CYP11A1) | 1.94 | 6.99e-03 | 3.19e-02 |
|  | 7 | 158279694 | 158279891 | PDFSTATSENSEMBLUCSC | 198 | 4 | 37376 | gene;island | 0.659258 | 0.876177 | Not queried | 1.94 | 4.86e-01 | 5.11e-01 |
|  | 3 | 4534791 | 4535154 | PDFSTATSENSEMBLUCSC | 364 | 9 | 28839 | island | 0.002169 | 0.007499 | Not queried | 1.94 | 7.70e-02 | 1.30e-01 |
|  | 8 | 91803657 | 91803805 | PDFSTATSENSEMBLUCSC | 149 | 5 | 39807 | tss;island | 0.471228 | 0.715245 | Not queried | 1.94 | 3.17e-02 | 6.50e-02 |
|  | 10 | 99790593 | 99790910 | PDFSTATSENSEMBLUCSC | 318 | 4 | 5275 | tss | 0.886570 | 0.965942 | Not queried | 1.94 | 2.86e-02 | 6.12e-02 |
|  | 2 | 233321600 | 233321900 | PDFSTATSENSEMBLUCSC | 301 | 4 | 23062 | gene;island | 0.872984 | 0.956795 | Not queried | 1.94 | 1.14e-01 | 1.68e-01 |
|  | 19 | 42806146 | 42806265 | PDFSTATSENSEMBLUCSC | 120 | 7 | 20823 | tss | 0.018073 | 0.002286 | Not queried | 1.94 | 2.59e-01 | 3.16e-01 |
|  | 13 | 25254440 | 25254512 | PDFSTATSENSEMBLUCSC | 73 | 4 | 11346 | tss;island | 0.068068 | 0.021325 | Not queried | 1.94 | 3.43e-01 | 3.84e-01 |
|  | 17 | 58754402 | 58755368 | PDFSTATSENSEMBLUCSC | 967 | 15 | 18626 | island | 0.007939 | 0.013310 | Not queried | 1.94 | 2.64e-02 | 6.12e-02 |
|  | 17 | 76498808 | 76499012 | PDFSTATSENSEMBLUCSC | 205 | 4 | 16822 | gene;island | 0.919507 | 0.750917 | Not queried | 1.94 | 5.71e-02 | 1.00e-01 |
|  | 12 | 104359248 | 104359478 | PDFSTATSENSEMBLUCSC | 231 | 5 | 9955 | tss | 0.002206 | 0.006130 | Not queried | 1.94 | 1.51e-01 | 2.11e-01 |
|  | 17 | 19314299 | 19314408 | PDFSTATSENSEMBLUCSC | 110 | 5 | 17191 | tss | 0.941222 | 0.656935 | Not queried | 1.94 | 3.10e-01 | 3.66e-01 |
|  | 17 | 26577563 | 26577877 | PDFSTATSENSEMBLUCSC | 315 | 5 | 18137 | island | 0.732079 | 0.972992 | Not queried | 1.93 | 3.10e-01 | 3.66e-01 |
|  | 2 | 219906245 | 219906468 | PDFSTATSENSEMBLUCSC | 224 | 7 | 23889 | tss | 0.815646 | 0.475130 | Not queried | 1.93 | 2.62e-02 | 6.12e-02 |
|  | 19 | 47925344 | 47925618 | PDFSTATSENSEMBLUCSC | 275 | 5 | 22194 | island | 0.066043 | 0.214766 | Not queried | 1.93 | 3.10e-01 | 3.66e-01 |
|  | 11 | 124735089 | 124735554 | PDFSTATSENSEMBLUCSC | 466 | 10 | 8917 | island | 0.508687 | 0.776328 | Not queried | 1.93 | 1.05e-01 | 1.66e-01 |
|  | 11 | 129234117 | 129234377 | PDFSTATSENSEMBLUCSC | 261 | 6 | 8946 | island | 0.004961 | 0.006841 | Not queried | 1.93 | 3.94e-01 | 4.36e-01 |
|  | 14 | 74111075 | 74111498 | PDFSTATSENSEMBLUCSC | 424 | 6 | 12498 | tss | 0.007976 | 0.006474 | Not queried | 1.93 | 9.37e-01 | 9.41e-01 |
|  | 1 | 113216774 | 113217181 | PDFSTATSENSEMBLUCSC | 408 | 6 | 3606 | island | 0.009846 | 0.050244 | Not queried | 1.93 | 9.31e-02 | 1.53e-01 |
|  | 13 | 112275633 | 112276127 | PDFSTATSENSEMBLUCSC | 495 | 5 | 11900 | island | 0.760701 | 0.930556 | Not queried | 1.93 | 4.21e-01 | 4.59e-01 |
|  | 10 | 124578028 | 124578544 | PDFSTATSENSEMBLUCSC | 517 | 5 | 6210 | island | 0.779749 | 0.911821 | Not queried | 1.93 | 3.10e-01 | 3.66e-01 |
|  | 18 | 56338220 | 56338298 | PDFSTATSENSEMBLUCSC | 79 | 4 | 19294 | tss;island | 0.002865 | 0.010200 | Not queried | 1.93 | 2.00e-01 | 2.55e-01 |
|  | 10 | 71892694 | 71892787 | PDFSTATSENSEMBLUCSC | 94 | 6 | 5130 | tss | 0.007496 | 0.041461 | Not queried | 1.93 | 2.40e-01 | 2.95e-01 |
|  | 6 | 160783785 | 160784044 | PDFSTATSENSEMBLUCSC | 260 | 4 | 34535 | gene;island | 0.588742 | 0.829564 | Not queried | 1.93 | 2.86e-02 | 6.12e-02 |
|  | 17 | 73775612 | 73776120 | PDFSTATSENSEMBLUCSC | 509 | 12 | 18762 | island | 0.002767 | 0.005078 | Not queried | 1.93 | 1.60e-01 | 2.23e-01 |
|  | 3 | 21792857 | 21792991 | PDFSTATSENSEMBLUCSC | 135 | 4 | 28182 | tss | 0.154227 | 0.050312 | Not queried | 1.93 | 3.43e-01 | 3.84e-01 |
|  | 12 | 48577720 | 48578223 | PDFSTATSENSEMBLUCSC | 504 | 4 | 9111 | gene;island | 0.159674 | 0.420766 | Not queried | 1.93 | 4.86e-01 | 5.11e-01 |
|  | 20 | 19998368 | 19998460 | PDFSTATSENSEMBLUCSC | 93 | 5 | 25623 | tss | 0.015690 | 0.057576 | Not queried | 1.93 | 9.52e-02 | 1.53e-01 |
|  | 6 | 31439037 | 31439148 | PDFSTATSENSEMBLUCSC | 112 | 4 | 33878 | gene | 0.924533 | 0.686905 | Not queried | 1.93 | 1.14e-01 | 1.68e-01 |
|  | 12 | 133657535 | 133657810 | PDFSTATSENSEMBLUCSC | 276 | 4 | 9471 | gene;island | 0.005833 | 0.033944 | Not queried | 1.93 | 3.43e-01 | 3.84e-01 |
|  | 5 | 1087095 | 1087341 | PDFSTATSENSEMBLUCSC | 247 | 4 | 31521 | gene;island | 0.928065 | 0.973653 | Not queried | 1.93 | 2.00e-01 | 2.55e-01 |
|  | 22 | 45403493 | 45403893 | PDFSTATSENSEMBLUCSC | 401 | 5 | 26897 | gene;island | 0.409105 | 0.022114 | Not queried | 1.93 | 1.51e-01 | 2.11e-01 |
|  | 5 | 140857474 | 140858107 | PDFSTATSENSEMBLUCSC | 634 | 8 | 31838 | gene;island | 0.440037 | 0.101438 | Not queried | 1.93 | 3.28e-01 | 3.84e-01 |
|  | 6 | 31631638 | 31631801 | PDFSTATSENSEMBLUCSC | 164 | 7 | 33931 | gene;island | 0.877012 | 0.961965 | Not queried | 1.93 | 1.65e-01 | 2.28e-01 |
|  | 8 | 144462502 | 144462851 | PDFSTATSENSEMBLUCSC | 350 | 5 | 39476 | gene;island | 0.791849 | 0.945005 | Not queried | 1.93 | 3.10e-01 | 3.66e-01 |
|  | 1 | 166958221 | 166958583 | PDFSTATSENSEMBLUCSC | 363 | 11 | 3943 | island | 0.932543 | 0.972308 | Not queried | 1.93 | 1.16e-01 | 1.71e-01 |
|  | 7 | 156812806 | 156813602 | PDFSTATSENSEMBLUCSC | 797 | 8 | 39094 | island | 0.900079 | 0.984305 | Not queried | 1.93 | 2.81e-02 | 6.12e-02 |
|  | 19 | 54495183 | 54495525 | PDFSTATSENSEMBLUCSC | 343 | 6 | 21047 | tss;island | 0.610465 | 0.848276 | Not queried | 1.93 | 2.40e-01 | 2.95e-01 |
|  | 10 | 90343166 | 90343435 | PDFSTATSENSEMBLUCSC | 270 | 8 | 5206 | tss;island | 0.010092 | 0.034737 | Not queried | 1.93 | 4.99e-02 | 9.81e-02 |
|  | 6 | 31548340 | 31549276 | PDFSTATSENSEMBLUCSC | 937 | 21 | 33905 | gene | 0.392589 | 0.227258 | Not queried | 1.93 | 6.98e-02 | 1.19e-01 |
|  | 11 | 788406 | 788714 | PDFSTATSENSEMBLUCSC | 309 | 4 | 6390 | gene;island | 0.707944 | 0.909295 | Not queried | 1.93 | 2.00e-01 | 2.55e-01 |
|  | 20 | 23549455 | 23549525 | PDFSTATSENSEMBLUCSC | 71 | 5 | 25641 | tss | 0.560438 | 0.860033 | Not queried | 1.93 | 3.17e-02 | 6.50e-02 |
|  | 11 | 94771024 | 94771514 | PDFSTATSENSEMBLUCSC | 491 | 4 | 8745 | island | 0.898830 | 0.975884 | Not queried | 1.92 | 2.00e-01 | 2.55e-01 |
|  | 7 | 158750244 | 158750607 | PDFSTATSENSEMBLUCSC | 364 | 4 | 39157 | island | 0.808805 | 0.539062 | Not queried | 1.92 | 3.43e-01 | 3.84e-01 |
|  | 5 | 76925380 | 76925623 | PDFSTATSENSEMBLUCSC | 244 | 4 | 31655 | gene;island | 0.865271 | 0.978692 | Not queried | 1.92 | 4.86e-01 | 5.11e-01 |
|  | 3 | 4508494 | 4509421 | PDFSTATSENSEMBLUCSC | 928 | 10 | 28838 | island | 0.010155 | 0.018870 | Not queried | 1.92 | 5.79e-01 | 6.02e-01 |
|  | 3 | 54157557 | 54157900 | PDFSTATSENSEMBLUCSC | 344 | 5 | 27835 | gene;island | 0.607187 | 0.759875 | Not queried | 1.92 | 3.10e-01 | 3.66e-01 |
|  | 19 | 41106155 | 41106351 | PDFSTATSENSEMBLUCSC | 197 | 4 | 20046 | gene;tss;island | 0.570322 | 0.412469 | Not queried | 1.92 | 1.00e+00 | 1.00e+00 |
|  | 14 | 58893672 | 58893942 | PDFSTATSENSEMBLUCSC | 271 | 4 | 12097 | gene;tss;island | 0.001014 | 0.003105 | Not queried | 1.92 | 3.43e-01 | 3.84e-01 |
|  | 13 | 25861182 | 25861655 | PDFSTATSENSEMBLUCSC | 474 | 5 | 11108 | gene | 0.007323 | 0.006784 | Not queried | 1.92 | 4.21e-01 | 4.59e-01 |
|  | 17 | 65040739 | 65040983 | PDFSTATSENSEMBLUCSC | 245 | 4 | 16736 | gene | 0.814054 | 0.952412 | Not queried | 1.92 | 3.43e-01 | 3.84e-01 |
|  | 1 | 2391121 | 2391347 | PDFSTATSENSEMBLUCSC | 227 | 4 | 2503 | island | 0.650049 | 0.887301 | Not queried | 1.92 | 4.86e-01 | 5.11e-01 |
|  | 17 | 1880884 | 1881333 | PDFSTATSENSEMBLUCSC | 450 | 6 | 16325 | gene;island | 0.890602 | 0.972658 | Not queried | 1.92 | 4.11e-02 | 8.14e-02 |
|  | 12 | 64845451 | 64845926 | PDFSTATSENSEMBLUCSC | 476 | 5 | 9842 | tss | 0.005303 | 0.005431 | Not queried | 1.92 | 4.21e-01 | 4.59e-01 |
|  | 16 | 680872 | 681057 | PDFSTATSENSEMBLUCSC | 186 | 4 | 15465 | island | 0.555359 | 0.842859 | Not queried | 1.92 | 2.86e-02 | 6.12e-02 |
|  | 1 | 155826844 | 155827082 | PDFSTATSENSEMBLUCSC | 239 | 6 | 3834 | island | 0.002352 | 0.006713 | Not queried | 1.92 | 3.94e-01 | 4.36e-01 |
|  | 2 | 237120069 | 237120313 | PDFSTATSENSEMBLUCSC | 245 | 4 | 23090 | gene;island | 0.885598 | 0.973940 | Not queried | 1.92 | 3.43e-01 | 3.84e-01 |
|  | 10 | 131763676 | 131763929 | PDFSTATSENSEMBLUCSC | 254 | 4 | 6267 | island | 0.499973 | 0.816309 | Not queried | 1.92 | 2.00e-01 | 2.55e-01 |
|  | 2 | 241858058 | 241858448 | PDFSTATSENSEMBLUCSC | 391 | 4 | 25281 | island | 0.662865 | 0.926180 | Not queried | 1.92 | 5.71e-02 | 1.00e-01 |
|  | 7 | 155090783 | 155091023 | PDFSTATSENSEMBLUCSC | 241 | 4 | 37307 | gene;island | 0.003545 | 0.027683 | Not queried | 1.92 | 1.14e-01 | 1.68e-01 |
|  | 2 | 127414802 | 127415063 | PDFSTATSENSEMBLUCSC | 262 | 4 | 22854 | gene;island | 0.727377 | 0.862271 | Not queried | 1.92 | 1.14e-01 | 1.68e-01 |
|  | 11 | 71639806 | 71640085 | PDFSTATSENSEMBLUCSC | 280 | 4 | 6805 | gene | 0.002169 | 0.003291 | Not queried | 1.92 | 6.86e-01 | 6.99e-01 |
|  | 12 | 54867429 | 54867653 | PDFSTATSENSEMBLUCSC | 225 | 4 | 9764 | tss | 0.684374 | 0.941338 | Not queried | 1.92 | 3.43e-01 | 3.84e-01 |
|  | 19 | 58838208 | 58838374 | PDFSTATSENSEMBLUCSC | 167 | 5 | 21138 | tss | 0.002610 | 0.012791 | Not queried | 1.92 | 5.56e-02 | 1.00e-01 |
|  | 3 | 38179815 | 38180116 | PDFSTATSENSEMBLUCSC | 302 | 8 | 28226 | tss;island | 0.210620 | 0.183691 | Not queried | 1.92 | 2.79e-01 | 3.38e-01 |
|  | 12 | 34319734 | 34320121 | PDFSTATSENSEMBLUCSC | 388 | 5 | 10321 | island | 0.876444 | 0.934885 | Not queried | 1.92 | 2.22e-01 | 2.76e-01 |
|  | 6 | 31324227 | 31324684 | PDFSTATSENSEMBLUCSC | 458 | 13 | 33869 | gene;island | 0.045498 | 0.250892 | XXbac-BPG248L24.12; HLA-B (margin: XXbac-BPG248L24.12; HLA-B; DHFRP2) | 1.92 | 7.24e-03 | 3.19e-02 |
|  | 6 | 31628050 | 31629199 | PDFSTATSENSEMBLUCSC | 1150 | 25 | 35813 | island | 0.005318 | 0.027250 | C6orf47-AS1; Y\_RNA; C6orf47; GPANK1 (margin: APOM; C6orf47-AS1; Y\_RNA; CSNK2B; CSNK2B-LY6G5B-1181; LY6G5B; BAG6; C6orf47; GPANK1) | 1.92 | 4.53e-03 | 2.63e-02 |
|  | 19 | 12807371 | 12807673 | PDFSTATSENSEMBLUCSC | 303 | 10 | 21614 | island | 0.008077 | 0.014600 | Not queried | 1.92 | 2.88e-02 | 6.16e-02 |
|  | 19 | 12807494 | 12807673 | PDFSTATSENSEMBLUCSC | 180 | 8 | 20505 | tss | 0.008077 | 0.013415 | Not queried | 1.92 | 8.30e-02 | 1.40e-01 |
|  | 16 | 27215034 | 27215281 | PDFSTATSENSEMBLUCSC | 248 | 5 | 15149 | tss | 0.002793 | 0.004336 | Not queried | 1.92 | 2.22e-01 | 2.76e-01 |
|  | 16 | 850240 | 850880 | PDFSTATSENSEMBLUCSC | 641 | 8 | 15489 | island | 0.106227 | 0.019205 | Not queried | 1.91 | 1.95e-01 | 2.55e-01 |
|  | 17 | 62084205 | 62084359 | PDFSTATSENSEMBLUCSC | 155 | 5 | 16731 | gene | 0.703058 | 0.899215 | Not queried | 1.91 | 2.22e-01 | 2.76e-01 |
|  | 6 | 17102310 | 17102678 | PDFSTATSENSEMBLUCSC | 369 | 6 | 35473 | island | 0.660293 | 0.363502 | STMND1 (margin: STMND1) | 1.91 | 8.66e-03 | 3.36e-02 |
|  | 2 | 173600362 | 173600676 | PDFSTATSENSEMBLUCSC | 315 | 4 | 22926 | gene;island | 0.064340 | 0.022944 | Not queried | 1.91 | 3.43e-01 | 3.84e-01 |
|  | 14 | 106938234 | 106938451 | PDFSTATSENSEMBLUCSC | 218 | 7 | 12676 | tss | 0.882420 | 0.965784 | Not queried | 1.91 | 1.75e-02 | 5.79e-02 |
|  | 10 | 118083701 | 118083921 | PDFSTATSENSEMBLUCSC | 221 | 5 | 5384 | tss | 0.908619 | 0.979866 | Not queried | 1.91 | 3.17e-02 | 6.50e-02 |
|  | 22 | 17178585 | 17178751 | PDFSTATSENSEMBLUCSC | 167 | 4 | 27268 | island | 0.825840 | 0.944181 | Not queried | 1.91 | 2.00e-01 | 2.55e-01 |
|  | 4 | 40751717 | 40752151 | PDFSTATSENSEMBLUCSC | 435 | 6 | 30997 | island | 0.088011 | 0.046577 | Not queried | 1.91 | 3.94e-01 | 4.36e-01 |
|  | 15 | 55881075 | 55881246 | PDFSTATSENSEMBLUCSC | 172 | 6 | 13684 | tss | 0.595192 | 0.882230 | Not queried | 1.91 | 4.11e-02 | 8.14e-02 |
|  | 1 | 2266138 | 2266591 | PDFSTATSENSEMBLUCSC | 454 | 5 | 75 | gene;island | 0.869323 | 0.939727 | Not queried | 1.91 | 3.10e-01 | 3.66e-01 |
|  | 17 | 46802740 | 46803008 | PDFSTATSENSEMBLUCSC | 269 | 4 | 16664 | gene;island | 0.938336 | 0.983496 | Not queried | 1.91 | 4.86e-01 | 5.11e-01 |
|  | 17 | 41277006 | 41279022 | PDFSTATSENSEMBLUCSC | 2017 | 45 | 18364 | island | 0.004818 | 0.016976 | Not queried | 1.91 | 1.60e-02 | 5.41e-02 |
|  | 19 | 7911120 | 7911551 | PDFSTATSENSEMBLUCSC | 432 | 6 | 19786 | gene;island | 0.802821 | 0.939416 | Not queried | 1.91 | 1.32e-01 | 1.89e-01 |
|  | 8 | 144242029 | 144242448 | PDFSTATSENSEMBLUCSC | 420 | 6 | 39944 | tss;island | 0.336533 | 0.616651 | Not queried | 1.91 | 1.32e-01 | 1.89e-01 |
|  | 7 | 88424985 | 88425148 | PDFSTATSENSEMBLUCSC | 164 | 6 | 37079 | gene | 0.285805 | 0.118957 | Not queried | 1.91 | 5.89e-01 | 6.11e-01 |
|  | 22 | 21321253 | 21321434 | PDFSTATSENSEMBLUCSC | 182 | 4 | 26985 | tss | 0.701649 | 0.908292 | Not queried | 1.91 | 2.00e-01 | 2.55e-01 |
|  | 16 | 3062349 | 3062426 | PDFSTATSENSEMBLUCSC | 78 | 4 | 15043 | tss | 0.578463 | 0.870137 | Not queried | 1.91 | 5.71e-02 | 1.00e-01 |
|  | 12 | 70759665 | 70760124 | PDFSTATSENSEMBLUCSC | 460 | 7 | 10621 | island | 0.017241 | 0.012907 | Not queried | 1.91 | 2.59e-01 | 3.16e-01 |
|  | 20 | 48599526 | 48599682 | PDFSTATSENSEMBLUCSC | 157 | 4 | 25450 | gene | 0.141243 | 0.037088 | Not queried | 1.91 | 4.86e-01 | 5.11e-01 |
|  | 1 | 101704441 | 101704656 | PDFSTATSENSEMBLUCSC | 216 | 4 | 655 | gene;island | 0.068883 | 0.047573 | Not queried | 1.91 | 1.00e+00 | 1.00e+00 |
|  | 1 | 23521272 | 23521417 | PDFSTATSENSEMBLUCSC | 146 | 4 | 1336 | tss | 0.624213 | 0.868201 | Not queried | 1.91 | 2.00e-01 | 2.55e-01 |
|  | 15 | 89786496 | 89787113 | PDFSTATSENSEMBLUCSC | 618 | 6 | 13885 | tss | 0.076143 | 0.450156 | Not queried | 1.91 | 4.85e-01 | 5.11e-01 |
|  | 2 | 74729100 | 74729710 | PDFSTATSENSEMBLUCSC | 611 | 7 | 22744 | gene;tss;island | 0.338660 | 0.632066 | Not queried | 1.91 | 1.28e-01 | 1.87e-01 |
|  | 20 | 43743835 | 43744110 | PDFSTATSENSEMBLUCSC | 276 | 5 | 25769 | tss | 0.565854 | 0.798578 | Not queried | 1.91 | 4.21e-01 | 4.59e-01 |
|  | 6 | 33739406 | 33739653 | PDFSTATSENSEMBLUCSC | 248 | 4 | 34298 | gene;island | 0.878351 | 0.969005 | Not queried | 1.91 | 2.86e-02 | 6.12e-02 |
|  | 17 | 4545074 | 4545157 | PDFSTATSENSEMBLUCSC | 84 | 4 | 17027 | tss | 0.905006 | 0.973997 | Not queried | 1.91 | 2.86e-02 | 6.12e-02 |
|  | 17 | 36102254 | 36102740 | PDFSTATSENSEMBLUCSC | 487 | 4 | 16537 | gene;island | 0.531664 | 0.647251 | Not queried | 1.91 | 1.00e+00 | 1.00e+00 |
|  | 12 | 131323926 | 131324319 | PDFSTATSENSEMBLUCSC | 394 | 6 | 10120 | tss;island | 0.041922 | 0.039703 | Not queried | 1.91 | 6.99e-01 | 7.09e-01 |
|  | 18 | 23713838 | 23714084 | PDFSTATSENSEMBLUCSC | 247 | 4 | 19079 | gene | 0.782536 | 0.977345 | Not queried | 1.91 | 4.86e-01 | 5.11e-01 |
|  | 16 | 3062349 | 3062975 | PDFSTATSENSEMBLUCSC | 627 | 8 | 15628 | island | 0.578463 | 0.870137 | Not queried | 1.91 | 2.81e-02 | 6.12e-02 |
|  | 12 | 124870112 | 124870394 | PDFSTATSENSEMBLUCSC | 283 | 4 | 9393 | gene;island | 0.787159 | 0.938981 | Not queried | 1.91 | 1.14e-01 | 1.68e-01 |
|  | 6 | 8102871 | 8103092 | PDFSTATSENSEMBLUCSC | 222 | 8 | 34619 | tss | 0.047189 | 0.005865 | Not queried | 1.91 | 1.30e-01 | 1.89e-01 |
|  | 17 | 900031 | 900491 | PDFSTATSENSEMBLUCSC | 461 | 11 | 17804 | island | 0.003136 | 0.007915 | Not queried | 1.91 | 4.00e-02 | 8.03e-02 |
|  | 8 | 21966036 | 21966491 | PDFSTATSENSEMBLUCSC | 456 | 4 | 39227 | gene;island | 0.203084 | 0.410527 | Not queried | 1.91 | 8.86e-01 | 8.89e-01 |
|  | 22 | 42372359 | 42372667 | PDFSTATSENSEMBLUCSC | 309 | 4 | 27185 | tss;island | 0.177458 | 0.028922 | Not queried | 1.91 | 3.43e-01 | 3.84e-01 |
|  | 19 | 12848092 | 12848676 | PDFSTATSENSEMBLUCSC | 585 | 10 | 21617 | island | 0.001736 | 0.006754 | Not queried | 1.91 | 1.90e-01 | 2.55e-01 |
|  | 4 | 2964664 | 2965392 | PDFSTATSENSEMBLUCSC | 729 | 11 | 30257 | tss;island | 0.003392 | 0.008742 | Not queried | 1.91 | 1.71e-01 | 2.37e-01 |
|  | 19 | 16999576 | 16999800 | PDFSTATSENSEMBLUCSC | 225 | 6 | 20577 | tss | 0.800589 | 0.935841 | Not queried | 1.91 | 6.49e-02 | 1.11e-01 |
|  | 4 | 15376072 | 15376170 | PDFSTATSENSEMBLUCSC | 99 | 6 | 30308 | tss | 0.962428 | 0.790231 | Not queried | 1.90 | 2.40e-01 | 2.95e-01 |
|  | 3 | 120314841 | 120315121 | PDFSTATSENSEMBLUCSC | 281 | 4 | 28529 | tss | 0.004785 | 0.009858 | Not queried | 1.90 | 2.00e-01 | 2.55e-01 |
|  | 5 | 159343148 | 159343549 | PDFSTATSENSEMBLUCSC | 402 | 6 | 32464 | tss;island | 0.387860 | 0.068350 | Not queried | 1.90 | 1.32e-01 | 1.89e-01 |
|  | 16 | 75150456 | 75150880 | PDFSTATSENSEMBLUCSC | 425 | 8 | 16096 | island | 0.493140 | 0.221894 | Not queried | 1.90 | 2.79e-01 | 3.38e-01 |
|  | 1 | 45243311 | 45243518 | PDFSTATSENSEMBLUCSC | 208 | 4 | 497 | gene;tss;island | 0.477588 | 0.757429 | Not queried | 1.90 | 5.71e-02 | 1.00e-01 |
|  | 14 | 38064490 | 38064532 | PDFSTATSENSEMBLUCSC | 43 | 4 | 12362 | tss | 0.004966 | 0.019021 | Not queried | 1.90 | 4.86e-01 | 5.11e-01 |
|  | 1 | 236767889 | 236768366 | PDFSTATSENSEMBLUCSC | 478 | 8 | 2345 | tss | 0.037760 | 0.010702 | Not queried | 1.90 | 1.61e-01 | 2.24e-01 |
|  | 17 | 907643 | 907886 | PDFSTATSENSEMBLUCSC | 244 | 4 | 16302 | gene;island | 0.945439 | 0.852389 | Not queried | 1.90 | 1.14e-01 | 1.68e-01 |
|  | 8 | 145729106 | 145729537 | PDFSTATSENSEMBLUCSC | 432 | 7 | 40736 | island | 0.789032 | 0.917673 | Not queried | 1.90 | 1.65e-01 | 2.28e-01 |
|  | 1 | 7844775 | 7845237 | PDFSTATSENSEMBLUCSC | 463 | 4 | 189 | gene | 0.965311 | 0.731177 | Not queried | 1.90 | 6.86e-01 | 6.99e-01 |
|  | 14 | 50052765 | 50053293 | PDFSTATSENSEMBLUCSC | 529 | 8 | 12818 | island | 0.004665 | 0.011467 | Not queried | 1.90 | 2.34e-01 | 2.90e-01 |
|  | 1 | 232940990 | 232941253 | PDFSTATSENSEMBLUCSC | 264 | 4 | 1080 | gene;island | 0.537266 | 0.265789 | Not queried | 1.90 | 3.43e-01 | 3.84e-01 |
|  | 22 | 35937058 | 35937531 | PDFSTATSENSEMBLUCSC | 474 | 8 | 27448 | island | 0.425888 | 0.135021 | Not queried | 1.90 | 8.30e-02 | 1.40e-01 |
|  | 16 | 51189801 | 51190201 | PDFSTATSENSEMBLUCSC | 401 | 5 | 15921 | island | 0.585349 | 0.949617 | Not queried | 1.90 | 5.48e-01 | 5.70e-01 |
|  | 2 | 27593896 | 27594204 | PDFSTATSENSEMBLUCSC | 309 | 4 | 22627 | gene;tss;island | 0.015705 | 0.081170 | Not queried | 1.90 | 3.43e-01 | 3.84e-01 |
|  | 1 | 217310887 | 217311177 | PDFSTATSENSEMBLUCSC | 291 | 6 | 4205 | island | 0.830333 | 0.953397 | Not queried | 1.90 | 6.49e-02 | 1.11e-01 |
|  | 10 | 28822348 | 28822500 | PDFSTATSENSEMBLUCSC | 153 | 5 | 4545 | gene;island | 0.005052 | 0.003635 | Not queried | 1.90 | 4.21e-01 | 4.59e-01 |
|  | 17 | 5026393 | 5026840 | PDFSTATSENSEMBLUCSC | 448 | 8 | 17890 | island | 0.525581 | 0.827533 | Not queried | 1.90 | 3.79e-02 | 7.66e-02 |
|  | 12 | 22199200 | 22199588 | PDFSTATSENSEMBLUCSC | 389 | 5 | 9081 | gene;island | 0.004918 | 0.013090 | Not queried | 1.90 | 3.10e-01 | 3.66e-01 |
|  | 3 | 154146398 | 154146871 | PDFSTATSENSEMBLUCSC | 474 | 5 | 28002 | gene;island | 0.511902 | 0.791621 | Not queried | 1.90 | 3.17e-02 | 6.50e-02 |
|  | 7 | 134574947 | 134575524 | PDFSTATSENSEMBLUCSC | 578 | 9 | 37232 | gene;tss | 0.980046 | 0.942558 | Not queried | 1.90 | 2.97e-01 | 3.60e-01 |
|  | 2 | 20190016 | 20190349 | PDFSTATSENSEMBLUCSC | 334 | 9 | 23207 | tss | 0.025558 | 0.008710 | Not queried | 1.90 | 1.36e-01 | 1.94e-01 |
|  | 18 | 4455337 | 4455862 | PDFSTATSENSEMBLUCSC | 526 | 5 | 19368 | island | 0.822889 | 0.397958 | Not queried | 1.90 | 5.48e-01 | 5.70e-01 |
|  | 8 | 55370192 | 55370579 | PDFSTATSENSEMBLUCSC | 388 | 10 | 40302 | island | 0.862163 | 0.972374 | SOX17 (margin: SOX17) | 1.90 | 1.50e-03 | 1.41e-02 |
|  | 1 | 54665230 | 54665711 | PDFSTATSENSEMBLUCSC | 482 | 7 | 546 | gene | 0.050589 | 0.006929 | Not queried | 1.90 | 1.65e-01 | 2.28e-01 |
|  | 12 | 95467350 | 95467612 | PDFSTATSENSEMBLUCSC | 263 | 6 | 10684 | island | 0.001247 | 0.003210 | Not queried | 1.90 | 2.40e-01 | 2.95e-01 |
|  | 3 | 50383167 | 50383485 | PDFSTATSENSEMBLUCSC | 319 | 7 | 28354 | tss | 0.315713 | 0.490518 | ZMYND10-AS1; ZMYND10; NPRL2 (margin: ZMYND10-AS1; CYB561D2; XXcos-LUCA11.5; RASSF1; ZMYND10; NPRL2; TMEM115) | 1.90 | 6.99e-03 | 3.19e-02 |
|  | 7 | 29234786 | 29235180 | PDFSTATSENSEMBLUCSC | 395 | 5 | 36948 | gene;island | 0.321794 | 0.066950 | Not queried | 1.90 | 1.51e-01 | 2.11e-01 |
|  | 19 | 12886455 | 12886624 | PDFSTATSENSEMBLUCSC | 170 | 4 | 20510 | tss | 0.004515 | 0.008790 | Not queried | 1.90 | 3.43e-01 | 3.84e-01 |
|  | 17 | 42977377 | 42977550 | PDFSTATSENSEMBLUCSC | 174 | 4 | 16620 | gene;tss;island | 0.122043 | 0.019613 | Not queried | 1.90 | 3.43e-01 | 3.84e-01 |
|  | 2 | 124782117 | 124782263 | PDFSTATSENSEMBLUCSC | 147 | 4 | 23606 | tss;island | 0.851547 | 0.608125 | Not queried | 1.90 | 3.43e-01 | 3.84e-01 |
|  | 6 | 32187918 | 32188404 | PDFSTATSENSEMBLUCSC | 487 | 5 | 34098 | gene | 0.231089 | 0.536047 | NOTCH4 (margin: NOTCH4) | 1.90 | 7.94e-03 | 3.19e-02 |
|  | 10 | 121484846 | 121485184 | PDFSTATSENSEMBLUCSC | 339 | 6 | 5402 | tss;island | 0.088248 | 0.046838 | Not queried | 1.90 | 1.32e-01 | 1.89e-01 |
|  | 11 | 70458719 | 70459053 | PDFSTATSENSEMBLUCSC | 335 | 5 | 6792 | gene | 0.636255 | 0.881608 | Not queried | 1.90 | 9.52e-02 | 1.53e-01 |
|  | 3 | 98452812 | 98453086 | PDFSTATSENSEMBLUCSC | 275 | 4 | 27873 | gene;island | 0.595537 | 0.224397 | Not queried | 1.90 | 1.14e-01 | 1.68e-01 |
|  | 11 | 68181087 | 68181383 | PDFSTATSENSEMBLUCSC | 297 | 4 | 6774 | gene;island | 0.981356 | 0.887894 | Not queried | 1.89 | 3.43e-01 | 3.84e-01 |
|  | 4 | 7512016 | 7512498 | PDFSTATSENSEMBLUCSC | 483 | 5 | 29938 | gene;island | 0.976703 | 0.865784 | Not queried | 1.89 | 4.21e-01 | 4.59e-01 |
|  | 12 | 6898682 | 6898975 | PDFSTATSENSEMBLUCSC | 294 | 4 | 9033 | gene | 0.511384 | 0.773180 | Not queried | 1.89 | 2.00e-01 | 2.55e-01 |
|  | 17 | 46800992 | 46801314 | PDFSTATSENSEMBLUCSC | 323 | 4 | 16663 | gene;tss;island | 0.895179 | 0.974561 | Not queried | 1.89 | 3.43e-01 | 3.84e-01 |
|  | 11 | 60623415 | 60623918 | PDFSTATSENSEMBLUCSC | 504 | 7 | 8314 | island | 0.584662 | 0.857171 | PTGDR2 (margin: CCDC86; ZP1; RP11-804A23.4; PTGDR2) | 1.89 | 1.17e-03 | 1.13e-02 |
|  | 3 | 350503 | 351003 | PDFSTATSENSEMBLUCSC | 501 | 6 | 27637 | gene;island | 0.394908 | 0.312326 | Not queried | 1.89 | 4.85e-01 | 5.11e-01 |
|  | 17 | 57970021 | 57970656 | PDFSTATSENSEMBLUCSC | 636 | 13 | 17549 | tss;island | 0.004333 | 0.015163 | Not queried | 1.89 | 1.25e-01 | 1.84e-01 |
|  | 20 | 2489835 | 2490109 | PDFSTATSENSEMBLUCSC | 275 | 8 | 25545 | tss;island | 0.006895 | 0.009384 | Not queried | 1.89 | 5.74e-01 | 5.97e-01 |
|  | 4 | 48271072 | 48271149 | PDFSTATSENSEMBLUCSC | 78 | 4 | 29998 | gene;island | 0.007484 | 0.028959 | Not queried | 1.89 | 1.14e-01 | 1.68e-01 |
|  | 4 | 90759046 | 90759203 | PDFSTATSENSEMBLUCSC | 158 | 4 | 30059 | gene;tss;island | 0.338242 | 0.482433 | Not queried | 1.89 | 1.00e+00 | 1.00e+00 |
|  | 3 | 129694392 | 129694683 | PDFSTATSENSEMBLUCSC | 292 | 4 | 27945 | gene;island | 0.818660 | 0.949160 | Not queried | 1.89 | 2.00e-01 | 2.55e-01 |
|  | 17 | 17740275 | 17740468 | PDFSTATSENSEMBLUCSC | 194 | 6 | 18073 | island | 0.000948 | 0.009560 | Not queried | 1.89 | 1.80e-01 | 2.45e-01 |
|  | 14 | 52455805 | 52456142 | PDFSTATSENSEMBLUCSC | 338 | 4 | 12394 | tss;island | 0.002979 | 0.010325 | Not queried | 1.89 | 6.86e-01 | 6.99e-01 |
|  | 12 | 95467511 | 95467612 | PDFSTATSENSEMBLUCSC | 102 | 5 | 9921 | tss | 0.001818 | 0.003888 | Not queried | 1.89 | 4.21e-01 | 4.59e-01 |
|  | 12 | 132982231 | 132982483 | PDFSTATSENSEMBLUCSC | 253 | 4 | 11050 | island | 0.232173 | 0.373091 | Not queried | 1.89 | 1.14e-01 | 1.68e-01 |
|  | 5 | 154320467 | 154320616 | PDFSTATSENSEMBLUCSC | 150 | 5 | 32453 | tss | 0.003833 | 0.013991 | Not queried | 1.89 | 6.90e-01 | 7.01e-01 |
|  | 19 | 39694993 | 39695237 | PDFSTATSENSEMBLUCSC | 245 | 5 | 20767 | tss | 0.559224 | 0.886514 | Not queried | 1.89 | 2.22e-01 | 2.76e-01 |
|  | 1 | 65731689 | 65731886 | PDFSTATSENSEMBLUCSC | 198 | 4 | 568 | gene;island | 0.934765 | 0.991387 | Not queried | 1.89 | 1.14e-01 | 1.68e-01 |
|  | 1 | 108023249 | 108023482 | PDFSTATSENSEMBLUCSC | 234 | 5 | 660 | gene;island | 0.592858 | 0.959179 | Not queried | 1.89 | 9.52e-02 | 1.53e-01 |
|  | 12 | 75785089 | 75785295 | PDFSTATSENSEMBLUCSC | 207 | 5 | 9222 | gene;island | 0.007058 | 0.002517 | Not queried | 1.89 | 3.10e-01 | 3.66e-01 |
|  | 4 | 47033126 | 47033634 | PDFSTATSENSEMBLUCSC | 509 | 8 | 31022 | island | 0.345526 | 0.389158 | Not queried | 1.89 | 9.59e-01 | 9.62e-01 |
|  | 17 | 73840822 | 73840989 | PDFSTATSENSEMBLUCSC | 168 | 4 | 17663 | tss | 0.004845 | 0.014593 | Not queried | 1.89 | 2.86e-02 | 6.12e-02 |
|  | 7 | 150974273 | 150974554 | PDFSTATSENSEMBLUCSC | 282 | 7 | 37999 | tss;island | 0.010603 | 0.038098 | Not queried | 1.89 | 5.30e-02 | 1.00e-01 |
|  | 7 | 106808956 | 106809317 | PDFSTATSENSEMBLUCSC | 362 | 4 | 37824 | tss;island | 0.004657 | 0.016172 | Not queried | 1.89 | 2.00e-01 | 2.55e-01 |
|  | 1 | 1142132 | 1142405 | PDFSTATSENSEMBLUCSC | 274 | 4 | 1144 | tss | 0.598105 | 0.215208 | Not queried | 1.89 | 5.71e-02 | 1.00e-01 |
|  | 2 | 200524002 | 200524259 | PDFSTATSENSEMBLUCSC | 258 | 4 | 24995 | island | 0.857197 | 0.647063 | Not queried | 1.89 | 3.43e-01 | 3.84e-01 |
|  | 6 | 74405447 | 74406189 | PDFSTATSENSEMBLUCSC | 743 | 7 | 36262 | island | 0.005381 | 0.009407 | Not queried | 1.89 | 3.18e-01 | 3.74e-01 |
|  | 11 | 67210033 | 67210282 | PDFSTATSENSEMBLUCSC | 250 | 5 | 6757 | gene | 0.980012 | 0.800274 | Not queried | 1.89 | 9.52e-02 | 1.53e-01 |
|  | 5 | 480033 | 480412 | PDFSTATSENSEMBLUCSC | 380 | 4 | 31506 | gene;island | 0.839441 | 0.955089 | Not queried | 1.89 | 2.00e-01 | 2.55e-01 |
|  | 14 | 53173926 | 53174235 | PDFSTATSENSEMBLUCSC | 310 | 4 | 12081 | gene | 0.003583 | 0.011693 | Not queried | 1.88 | 2.00e-01 | 2.55e-01 |
|  | 11 | 44331905 | 44333192 | PDFSTATSENSEMBLUCSC | 1288 | 40 | 7261 | tss;island | 0.795993 | 0.961162 | ALX4 (margin: ALX4) | 1.88 | 6.54e-05 | 1.76e-03 |
|  | 20 | 2731102 | 2731560 | PDFSTATSENSEMBLUCSC | 459 | 4 | 25337 | gene;island | 0.601739 | 0.210111 | Not queried | 1.88 | 1.14e-01 | 1.68e-01 |
|  | 5 | 112312093 | 112312406 | PDFSTATSENSEMBLUCSC | 314 | 6 | 32217 | tss | 0.095070 | 0.019232 | Not queried | 1.88 | 3.10e-01 | 3.66e-01 |
|  | 13 | 33780078 | 33780362 | PDFSTATSENSEMBLUCSC | 285 | 5 | 11125 | gene | 0.979961 | 0.949998 | Not queried | 1.88 | 1.59e-02 | 5.36e-02 |
|  | 13 | 113705742 | 113706204 | PDFSTATSENSEMBLUCSC | 463 | 4 | 11272 | gene;island | 0.790831 | 0.969855 | Not queried | 1.88 | 2.00e-01 | 2.55e-01 |
|  | 3 | 64670459 | 64670640 | PDFSTATSENSEMBLUCSC | 182 | 4 | 27850 | gene;island | 0.678614 | 0.524334 | Not queried | 1.88 | 4.86e-01 | 5.11e-01 |
|  | 2 | 176986261 | 176986659 | PDFSTATSENSEMBLUCSC | 399 | 4 | 23736 | tss;island | 0.883533 | 0.970968 | Not queried | 1.88 | 1.14e-01 | 1.68e-01 |
|  | 8 | 79470784 | 79471036 | PDFSTATSENSEMBLUCSC | 253 | 4 | 39347 | gene | 0.267203 | 0.566137 | Not queried | 1.88 | 2.86e-02 | 6.12e-02 |
|  | 7 | 111202222 | 111202457 | PDFSTATSENSEMBLUCSC | 236 | 5 | 38841 | island | 0.009260 | 0.030641 | Not queried | 1.88 | 4.21e-01 | 4.59e-01 |
|  | 17 | 21356245 | 21356372 | PDFSTATSENSEMBLUCSC | 128 | 4 | 18121 | island | 0.590942 | 0.805478 | Not queried | 1.88 | 2.00e-01 | 2.55e-01 |
|  | 1 | 155178215 | 155178670 | PDFSTATSENSEMBLUCSC | 456 | 6 | 1986 | tss;island | 0.008496 | 0.036663 | Not queried | 1.88 | 1.80e-01 | 2.45e-01 |
|  | 2 | 223290012 | 223290515 | PDFSTATSENSEMBLUCSC | 504 | 4 | 23053 | gene;island | 0.724648 | 0.935619 | Not queried | 1.88 | 5.71e-02 | 1.00e-01 |
|  | 6 | 31657467 | 31657855 | PDFSTATSENSEMBLUCSC | 389 | 4 | 33938 | gene | 0.916484 | 0.745422 | Not queried | 1.88 | 4.86e-01 | 5.11e-01 |
|  | 6 | 31547917 | 31549276 | PDFSTATSENSEMBLUCSC | 1360 | 26 | 35788 | island | 0.581186 | 0.316044 | Not queried | 1.88 | 1.23e-01 | 1.80e-01 |
|  | 1 | 154965419 | 154965572 | PDFSTATSENSEMBLUCSC | 154 | 4 | 787 | gene;tss | 0.945062 | 0.811640 | Not queried | 1.88 | 2.00e-01 | 2.55e-01 |
|  | 15 | 74420511 | 74420890 | PDFSTATSENSEMBLUCSC | 380 | 4 | 13414 | gene;tss;island | 0.528262 | 0.312087 | Not queried | 1.88 | 4.86e-01 | 5.11e-01 |
|  | 21 | 46875142 | 46875433 | PDFSTATSENSEMBLUCSC | 292 | 5 | 26468 | gene;island | 0.496439 | 0.230320 | Not queried | 1.88 | 5.56e-02 | 1.00e-01 |
|  | 12 | 132900274 | 132901088 | PDFSTATSENSEMBLUCSC | 815 | 7 | 9448 | gene;island | 0.493860 | 0.880565 | Not queried | 1.88 | 3.79e-02 | 7.66e-02 |
|  | 17 | 16472670 | 16472866 | PDFSTATSENSEMBLUCSC | 197 | 7 | 17160 | tss;island | 0.100486 | 0.058675 | Not queried | 1.88 | 2.09e-01 | 2.65e-01 |
|  | 2 | 55458963 | 55459287 | PDFSTATSENSEMBLUCSC | 325 | 5 | 22687 | gene;tss | 0.000469 | 0.000509 | Not queried | 1.88 | 8.41e-01 | 8.48e-01 |
|  | 1 | 228509380 | 228509762 | PDFSTATSENSEMBLUCSC | 383 | 5 | 1056 | gene;island | 0.728444 | 0.975234 | Not queried | 1.88 | 3.10e-01 | 3.66e-01 |
|  | 19 | 18337107 | 18337365 | PDFSTATSENSEMBLUCSC | 259 | 5 | 19932 | gene;island | 0.334948 | 0.525916 | Not queried | 1.88 | 3.10e-01 | 3.66e-01 |
|  | 10 | 71892593 | 71892787 | PDFSTATSENSEMBLUCSC | 195 | 7 | 5812 | island | 0.005611 | 0.034531 | Not queried | 1.88 | 2.09e-01 | 2.65e-01 |
|  | 17 | 1837588 | 1837848 | PDFSTATSENSEMBLUCSC | 261 | 6 | 17834 | island | 0.855053 | 0.964621 | Not queried | 1.88 | 1.52e-02 | 5.36e-02 |
|  | 5 | 154320467 | 154320678 | PDFSTATSENSEMBLUCSC | 212 | 7 | 33338 | island | 0.005929 | 0.013991 | Not queried | 1.88 | 5.35e-01 | 5.62e-01 |
|  | 10 | 65733092 | 65733575 | PDFSTATSENSEMBLUCSC | 484 | 4 | 5780 | island | 0.575013 | 0.827743 | Not queried | 1.88 | 3.43e-01 | 3.84e-01 |
|  | 2 | 55920477 | 55920932 | PDFSTATSENSEMBLUCSC | 456 | 5 | 22690 | gene | 0.002549 | 0.003667 | Not queried | 1.88 | 4.21e-01 | 4.59e-01 |
|  | 15 | 85197162 | 85197409 | PDFSTATSENSEMBLUCSC | 248 | 4 | 13462 | gene;island | 0.004406 | 0.012665 | Not queried | 1.88 | 2.00e-01 | 2.55e-01 |
|  | 12 | 57569768 | 57569940 | PDFSTATSENSEMBLUCSC | 173 | 4 | 9183 | gene;island | 0.036082 | 0.062225 | Not queried | 1.88 | 6.86e-01 | 6.99e-01 |
|  | 9 | 94186882 | 94187181 | PDFSTATSENSEMBLUCSC | 300 | 4 | 40820 | tss;island | 0.099417 | 0.054200 | Not queried | 1.88 | 6.86e-01 | 6.99e-01 |
|  | 19 | 1940412 | 1940875 | PDFSTATSENSEMBLUCSC | 464 | 6 | 20316 | tss;island | 0.028126 | 0.065647 | Not queried | 1.88 | 6.49e-02 | 1.11e-01 |
|  | 12 | 104443925 | 104444136 | PDFSTATSENSEMBLUCSC | 212 | 8 | 9956 | tss | 0.744378 | 0.461256 | Not queried | 1.88 | 3.28e-01 | 3.84e-01 |
|  | 16 | 89634447 | 89634715 | PDFSTATSENSEMBLUCSC | 269 | 4 | 16265 | island | 0.760446 | 0.931001 | Not queried | 1.88 | 5.71e-02 | 1.00e-01 |
|  | 10 | 695844 | 696063 | PDFSTATSENSEMBLUCSC | 220 | 6 | 4449 | gene | 0.886661 | 0.977087 | Not queried | 1.88 | 1.80e-01 | 2.45e-01 |
|  | 19 | 49496473 | 49496578 | PDFSTATSENSEMBLUCSC | 106 | 4 | 20142 | gene | 0.004809 | 0.010593 | Not queried | 1.87 | 2.00e-01 | 2.55e-01 |
|  | 8 | 1094484 | 1094904 | PDFSTATSENSEMBLUCSC | 421 | 6 | 40012 | island | 0.554453 | 0.757834 | CTD-2281E23.2 (margin: ERICH1-AS1; CTD-2281E23.2) | 1.87 | 8.66e-03 | 3.36e-02 |
|  | 2 | 25896721 | 25896925 | PDFSTATSENSEMBLUCSC | 205 | 4 | 23234 | tss;island | 0.001410 | 0.006319 | Not queried | 1.87 | 3.43e-01 | 3.84e-01 |
|  | 19 | 57631118 | 57631478 | PDFSTATSENSEMBLUCSC | 361 | 9 | 21105 | tss;island | 0.819841 | 0.956883 | Not queried | 1.87 | 2.22e-01 | 2.76e-01 |
|  | 10 | 11059290 | 11059733 | PDFSTATSENSEMBLUCSC | 444 | 10 | 4492 | gene;tss;island | 0.854168 | 0.951807 | Not queried | 1.87 | 3.15e-01 | 3.72e-01 |
|  | 17 | 37331279 | 37331768 | PDFSTATSENSEMBLUCSC | 490 | 6 | 16546 | gene;island | 0.814161 | 0.226219 | Not queried | 1.87 | 3.10e-01 | 3.66e-01 |
|  | 8 | 832917 | 833183 | PDFSTATSENSEMBLUCSC | 267 | 4 | 40007 | island | 0.191455 | 0.479603 | Not queried | 1.87 | 3.43e-01 | 3.84e-01 |
|  | 19 | 5832033 | 5832343 | PDFSTATSENSEMBLUCSC | 311 | 4 | 19756 | gene;island | 0.832584 | 0.946464 | Not queried | 1.87 | 2.86e-02 | 6.12e-02 |
|  | 14 | 60632234 | 60632532 | PDFSTATSENSEMBLUCSC | 299 | 4 | 12436 | tss | 0.019669 | 0.048263 | Not queried | 1.87 | 4.86e-01 | 5.11e-01 |
|  | 16 | 834043 | 834510 | PDFSTATSENSEMBLUCSC | 468 | 4 | 15486 | island | 0.728114 | 0.935189 | Not queried | 1.87 | 4.86e-01 | 5.11e-01 |
|  | 1 | 206223453 | 206223719 | PDFSTATSENSEMBLUCSC | 267 | 5 | 2211 | tss;island | 0.209365 | 0.439092 | Not queried | 1.87 | 2.22e-01 | 2.76e-01 |
|  | 6 | 31762353 | 31762901 | PDFSTATSENSEMBLUCSC | 549 | 15 | 33958 | gene;island | 0.009670 | 0.031706 | VARS (margin: VARS; LSM2) | 1.87 | 5.78e-04 | 6.47e-03 |
|  | 14 | 24605550 | 24605782 | PDFSTATSENSEMBLUCSC | 233 | 4 | 12036 | gene;island | 0.013795 | 0.029776 | Not queried | 1.87 | 3.43e-01 | 3.84e-01 |
|  | 14 | 102553207 | 102553431 | PDFSTATSENSEMBLUCSC | 225 | 4 | 12225 | gene;island | 0.003082 | 0.005840 | Not queried | 1.87 | 2.00e-01 | 2.55e-01 |
|  | 6 | 56911139 | 56911257 | PDFSTATSENSEMBLUCSC | 119 | 4 | 35047 | tss | 0.004790 | 0.007962 | Not queried | 1.87 | 4.86e-01 | 5.11e-01 |
|  | 14 | 60337474 | 60337864 | PDFSTATSENSEMBLUCSC | 391 | 10 | 12896 | island | 0.066616 | 0.016111 | Not queried | 1.87 | 2.32e-02 | 6.12e-02 |
|  | 1 | 34642000 | 34642899 | PDFSTATSENSEMBLUCSC | 900 | 13 | 410 | gene;island | 0.852364 | 0.956590 | Not queried | 1.87 | 6.42e-02 | 1.11e-01 |
|  | 7 | 92158172 | 92158437 | PDFSTATSENSEMBLUCSC | 266 | 4 | 37085 | gene | 0.002004 | 0.006566 | Not queried | 1.87 | 4.86e-01 | 5.11e-01 |
|  | 10 | 98273714 | 98273924 | PDFSTATSENSEMBLUCSC | 211 | 5 | 5254 | tss | 0.107115 | 0.026565 | Not queried | 1.87 | 3.10e-01 | 3.66e-01 |
|  | 1 | 236925843 | 236925965 | PDFSTATSENSEMBLUCSC | 123 | 4 | 1101 | gene | 0.842613 | 0.987643 | Not queried | 1.87 | 1.14e-01 | 1.68e-01 |
|  | 17 | 44896748 | 44897024 | PDFSTATSENSEMBLUCSC | 277 | 4 | 17436 | tss;island | 0.549593 | 0.522531 | Not queried | 1.87 | 6.86e-01 | 6.99e-01 |
|  | 20 | 61002595 | 61002866 | PDFSTATSENSEMBLUCSC | 272 | 6 | 25870 | tss | 0.073387 | 0.017343 | Not queried | 1.87 | 4.85e-01 | 5.11e-01 |
|  | 19 | 38886554 | 38886789 | PDFSTATSENSEMBLUCSC | 236 | 4 | 20016 | gene;island | 0.893418 | 0.969040 | Not queried | 1.87 | 2.86e-02 | 6.12e-02 |
|  | 20 | 43029468 | 43029997 | PDFSTATSENSEMBLUCSC | 530 | 8 | 25424 | gene | 0.619427 | 0.951182 | Not queried | 1.87 | 1.95e-01 | 2.55e-01 |
|  | 17 | 2907895 | 2908369 | PDFSTATSENSEMBLUCSC | 475 | 5 | 16334 | gene | 0.776391 | 0.462040 | Not queried | 1.87 | 9.52e-02 | 1.53e-01 |
|  | 1 | 85514554 | 85514708 | PDFSTATSENSEMBLUCSC | 155 | 4 | 1720 | tss;island | 0.014058 | 0.027105 | Not queried | 1.87 | 3.43e-01 | 3.84e-01 |
|  | 8 | 82543035 | 82543431 | PDFSTATSENSEMBLUCSC | 397 | 5 | 40419 | island | 0.427095 | 0.680612 | Not queried | 1.87 | 1.51e-01 | 2.11e-01 |
|  | 10 | 73156377 | 73156858 | PDFSTATSENSEMBLUCSC | 482 | 6 | 5829 | island | 0.829644 | 0.960819 | Not queried | 1.87 | 6.49e-02 | 1.11e-01 |
|  | 22 | 24115096 | 24115568 | PDFSTATSENSEMBLUCSC | 473 | 4 | 26809 | gene | 0.151782 | 0.235307 | Not queried | 1.87 | 4.86e-01 | 5.11e-01 |
|  | 4 | 2251938 | 2252266 | PDFSTATSENSEMBLUCSC | 329 | 4 | 29883 | gene | 0.859478 | 0.948269 | Not queried | 1.87 | 2.00e-01 | 2.55e-01 |
|  | 6 | 100060917 | 100061504 | PDFSTATSENSEMBLUCSC | 588 | 6 | 34429 | gene;island | 0.833692 | 0.955045 | Not queried | 1.87 | 4.85e-01 | 5.11e-01 |
|  | 5 | 1666974 | 1667258 | PDFSTATSENSEMBLUCSC | 285 | 4 | 32639 | island | 0.445263 | 0.124783 | Not queried | 1.87 | 4.86e-01 | 5.11e-01 |
|  | 2 | 97202260 | 97202435 | PDFSTATSENSEMBLUCSC | 176 | 4 | 23519 | tss | 0.006339 | 0.025999 | Not queried | 1.87 | 6.86e-01 | 6.99e-01 |
|  | 10 | 45470023 | 45470562 | PDFSTATSENSEMBLUCSC | 540 | 6 | 4574 | gene;island | 0.303327 | 0.048959 | Not queried | 1.87 | 3.94e-01 | 4.36e-01 |
|  | 11 | 60623504 | 60623918 | PDFSTATSENSEMBLUCSC | 415 | 6 | 7323 | tss | 0.598316 | 0.873791 | PTGDR2 (margin: CCDC86; ZP1; RP11-804A23.4; PTGDR2) | 1.87 | 4.33e-03 | 2.52e-02 |
|  | 17 | 73642404 | 73642773 | PDFSTATSENSEMBLUCSC | 370 | 7 | 16773 | gene | 0.761533 | 0.944274 | Not queried | 1.86 | 9.73e-02 | 1.55e-01 |
|  | 13 | 46038825 | 46038994 | PDFSTATSENSEMBLUCSC | 170 | 6 | 11422 | tss | 0.000878 | 0.001794 | Not queried | 1.86 | 4.85e-01 | 5.11e-01 |
|  | 7 | 157667762 | 157668141 | PDFSTATSENSEMBLUCSC | 380 | 5 | 37348 | gene;island | 0.837916 | 0.329071 | Not queried | 1.86 | 4.21e-01 | 4.59e-01 |
|  | 22 | 36806414 | 36806655 | PDFSTATSENSEMBLUCSC | 242 | 4 | 27452 | island | 0.127490 | 0.062654 | Not queried | 1.86 | 6.86e-01 | 6.99e-01 |
|  | 17 | 74721824 | 74722097 | PDFSTATSENSEMBLUCSC | 274 | 6 | 16793 | gene;tss;island | 0.013475 | 0.031746 | Not queried | 1.86 | 4.85e-01 | 5.11e-01 |
|  | 1 | 75593804 | 75594261 | PDFSTATSENSEMBLUCSC | 458 | 6 | 3385 | island | 0.807084 | 0.921870 | Not queried | 1.86 | 1.52e-02 | 5.36e-02 |
|  | 6 | 29759718 | 29760164 | PDFSTATSENSEMBLUCSC | 447 | 14 | 33693 | gene;island | 0.126507 | 0.362549 | HLA-V; HCG4 (margin: HLA-V; HLA-P; HCG4; RPL7AP7) | 1.86 | 7.87e-03 | 3.19e-02 |
|  | 5 | 95297236 | 95298307 | PDFSTATSENSEMBLUCSC | 1072 | 12 | 32972 | island | 0.071180 | 0.352907 | Not queried | 1.86 | 1.98e-01 | 2.55e-01 |
|  | 12 | 81110706 | 81111012 | PDFSTATSENSEMBLUCSC | 307 | 5 | 10648 | island | 0.785644 | 0.959437 | Not queried | 1.86 | 1.51e-01 | 2.11e-01 |
|  | 6 | 32489553 | 32489991 | PDFSTATSENSEMBLUCSC | 439 | 6 | 34115 | gene;island | 0.510344 | 0.251210 | Not queried | 1.86 | 6.49e-02 | 1.11e-01 |
|  | 12 | 4384688 | 4384890 | PDFSTATSENSEMBLUCSC | 203 | 5 | 9014 | gene;island | 0.791290 | 0.431372 | Not queried | 1.86 | 5.48e-01 | 5.70e-01 |
|  | 16 | 1493343 | 1493859 | PDFSTATSENSEMBLUCSC | 517 | 5 | 14571 | gene;island | 0.695236 | 0.956758 | Not queried | 1.86 | 3.10e-01 | 3.66e-01 |
|  | 10 | 72201484 | 72201741 | PDFSTATSENSEMBLUCSC | 258 | 6 | 5135 | tss | 0.664011 | 0.835376 | Not queried | 1.86 | 2.40e-01 | 2.95e-01 |
|  | 6 | 12957651 | 12958080 | PDFSTATSENSEMBLUCSC | 430 | 5 | 33573 | gene | 0.025692 | 0.068104 | Not queried | 1.86 | 1.51e-01 | 2.11e-01 |
|  | 1 | 227746111 | 227746453 | PDFSTATSENSEMBLUCSC | 343 | 4 | 4269 | island | 0.874548 | 0.941867 | Not queried | 1.86 | 5.71e-02 | 1.00e-01 |
|  | 5 | 138211043 | 138211184 | PDFSTATSENSEMBLUCSC | 142 | 6 | 31771 | gene | 0.914765 | 0.975115 | Not queried | 1.86 | 9.31e-02 | 1.53e-01 |
|  | 4 | 186131234 | 186131285 | PDFSTATSENSEMBLUCSC | 52 | 4 | 31473 | island | 0.117373 | 0.321530 | Not queried | 1.86 | 2.86e-02 | 6.12e-02 |
|  | 13 | 21900392 | 21900591 | PDFSTATSENSEMBLUCSC | 200 | 4 | 11589 | island | 0.791557 | 0.659895 | Not queried | 1.86 | 4.86e-01 | 5.11e-01 |
|  | 6 | 27858279 | 27858637 | PDFSTATSENSEMBLUCSC | 359 | 4 | 35575 | island | 0.050807 | 0.014425 | Not queried | 1.86 | 2.86e-02 | 6.12e-02 |
|  | 7 | 99063876 | 99064011 | PDFSTATSENSEMBLUCSC | 136 | 4 | 37755 | tss | 0.003774 | 0.012363 | Not queried | 1.86 | 3.43e-01 | 3.84e-01 |
|  | 3 | 48646722 | 48647426 | PDFSTATSENSEMBLUCSC | 705 | 8 | 29091 | island | 0.063488 | 0.028488 | Not queried | 1.86 | 4.42e-01 | 4.81e-01 |
|  | 12 | 372049 | 372259 | PDFSTATSENSEMBLUCSC | 211 | 5 | 9475 | tss | 0.104957 | 0.641646 | Not queried | 1.86 | 4.21e-01 | 4.59e-01 |
|  | 7 | 57324417 | 57324924 | PDFSTATSENSEMBLUCSC | 508 | 5 | 38518 | island | 0.679258 | 0.343581 | Not queried | 1.86 | 3.17e-02 | 6.50e-02 |
|  | 16 | 80966033 | 80966491 | PDFSTATSENSEMBLUCSC | 459 | 6 | 16118 | island | 0.848212 | 0.583217 | (margin: ) | 1.86 | 2.16e-03 | 1.45e-02 |
|  | 19 | 39785679 | 39786136 | PDFSTATSENSEMBLUCSC | 458 | 4 | 20768 | tss | 0.824100 | 0.973910 | Not queried | 1.86 | 2.00e-01 | 2.55e-01 |
|  | 8 | 56791576 | 56791882 | PDFSTATSENSEMBLUCSC | 307 | 5 | 39709 | tss;island | 0.562339 | 0.261974 | Not queried | 1.86 | 1.59e-02 | 5.36e-02 |
|  | 1 | 54204112 | 54204517 | PDFSTATSENSEMBLUCSC | 406 | 5 | 3287 | island | 0.850534 | 0.953715 | Not queried | 1.86 | 4.21e-01 | 4.59e-01 |
|  | 11 | 71791511 | 71791903 | PDFSTATSENSEMBLUCSC | 393 | 11 | 6810 | gene;tss;island | 0.001444 | 0.004479 | Not queried | 1.86 | 6.52e-01 | 6.76e-01 |
|  | 1 | 91182128 | 91182305 | PDFSTATSENSEMBLUCSC | 178 | 4 | 624 | gene;island | 0.912769 | 0.896744 | Not queried | 1.86 | 6.86e-01 | 6.99e-01 |
|  | 2 | 128422113 | 128422717 | PDFSTATSENSEMBLUCSC | 605 | 10 | 22862 | gene;island | 0.863115 | 0.952667 | LIMS2 (margin: GPR17; LIMS2) | 1.86 | 2.88e-03 | 1.86e-02 |
|  | 2 | 97405364 | 97406019 | PDFSTATSENSEMBLUCSC | 656 | 11 | 24532 | island | 0.000593 | 0.001376 | Not queried | 1.86 | 1.33e-01 | 1.91e-01 |
|  | 16 | 88832476 | 88832700 | PDFSTATSENSEMBLUCSC | 225 | 6 | 14918 | gene | 0.544271 | 0.919682 | Not queried | 1.85 | 6.49e-02 | 1.11e-01 |
|  | 13 | 113301636 | 113301782 | PDFSTATSENSEMBLUCSC | 147 | 4 | 11248 | gene;island | 0.775496 | 0.877120 | Not queried | 1.85 | 4.86e-01 | 5.11e-01 |
|  | 3 | 11058836 | 11059050 | PDFSTATSENSEMBLUCSC | 215 | 6 | 27665 | gene;island | 0.498676 | 0.922653 | Not queried | 1.85 | 4.11e-02 | 8.14e-02 |
|  | 7 | 27224092 | 27224795 | PDFSTATSENSEMBLUCSC | 704 | 8 | 36941 | gene | 0.024098 | 0.095667 | Not queried | 1.85 | 5.05e-01 | 5.32e-01 |
|  | 7 | 30722114 | 30722433 | PDFSTATSENSEMBLUCSC | 320 | 8 | 38389 | island | 0.659704 | 0.909142 | Not queried | 1.85 | 3.28e-01 | 3.84e-01 |
|  | 18 | 21852313 | 21852436 | PDFSTATSENSEMBLUCSC | 124 | 5 | 19211 | tss | 0.008073 | 0.015835 | Not queried | 1.85 | 2.22e-01 | 2.76e-01 |
|  | 6 | 27832728 | 27833095 | PDFSTATSENSEMBLUCSC | 368 | 8 | 34725 | tss | 0.001560 | 0.004269 | Not queried | 1.85 | 2.34e-01 | 2.90e-01 |
|  | 3 | 184529753 | 184529876 | PDFSTATSENSEMBLUCSC | 124 | 5 | 28764 | tss | 0.005755 | 0.003394 | Not queried | 1.85 | 4.21e-01 | 4.59e-01 |
|  | 3 | 112013079 | 112013357 | PDFSTATSENSEMBLUCSC | 279 | 4 | 28501 | tss | 0.712850 | 0.733338 | Not queried | 1.85 | 6.86e-01 | 6.99e-01 |
|  | 6 | 110501281 | 110501372 | PDFSTATSENSEMBLUCSC | 92 | 5 | 35167 | tss;island | 0.003253 | 0.005681 | Not queried | 1.85 | 2.22e-01 | 2.76e-01 |
|  | 8 | 93107604 | 93107744 | PDFSTATSENSEMBLUCSC | 141 | 5 | 39809 | tss | 0.554023 | 0.256120 | Not queried | 1.85 | 5.48e-01 | 5.70e-01 |
|  | 8 | 25901017 | 25901422 | PDFSTATSENSEMBLUCSC | 406 | 4 | 39251 | gene;island | 0.914226 | 0.972583 | Not queried | 1.85 | 2.86e-02 | 6.12e-02 |
|  | 19 | 30096869 | 30097075 | PDFSTATSENSEMBLUCSC | 207 | 4 | 20670 | tss | 0.003095 | 0.005455 | Not queried | 1.85 | 1.14e-01 | 1.68e-01 |
|  | 10 | 133850441 | 133850760 | PDFSTATSENSEMBLUCSC | 320 | 5 | 6284 | island | 0.442322 | 0.210485 | Not queried | 1.85 | 6.90e-01 | 7.01e-01 |
|  | 1 | 27709575 | 27710270 | PDFSTATSENSEMBLUCSC | 696 | 17 | 2919 | island | 0.133056 | 0.014759 | Not queried | 1.85 | 1.40e-01 | 2.00e-01 |
|  | 17 | 46655164 | 46656093 | PDFSTATSENSEMBLUCSC | 930 | 18 | 18480 | island | 0.861904 | 0.973590 | HOXB-AS3; HOXB3; HOXB4; MIR10A (margin: HOXB-AS3; HOXB3; HOXB4; MIR10A) | 1.85 | 2.39e-05 | 9.70e-04 |
|  | 12 | 4140624 | 4141193 | PDFSTATSENSEMBLUCSC | 570 | 6 | 10168 | island | 0.016469 | 0.198532 | Not queried | 1.85 | 1.32e-01 | 1.89e-01 |
|  | 22 | 33195343 | 33195688 | PDFSTATSENSEMBLUCSC | 346 | 6 | 26849 | gene;tss;island | 0.946976 | 0.978808 | Not queried | 1.85 | 1.80e-01 | 2.45e-01 |
|  | 8 | 41733475 | 41733931 | PDFSTATSENSEMBLUCSC | 457 | 5 | 39295 | gene;island | 0.745349 | 0.900355 | Not queried | 1.85 | 2.22e-01 | 2.76e-01 |
|  | 17 | 9728972 | 9729337 | PDFSTATSENSEMBLUCSC | 366 | 8 | 17131 | tss | 0.630880 | 0.779803 | GLP2R (margin: GLP2R; RP11-477N12.3) | 1.85 | 6.99e-03 | 3.19e-02 |
|  | 20 | 37434158 | 37434262 | PDFSTATSENSEMBLUCSC | 105 | 6 | 25740 | tss | 0.748051 | 0.947198 | Not queried | 1.85 | 6.49e-02 | 1.11e-01 |
|  | 11 | 74870268 | 74870741 | PDFSTATSENSEMBLUCSC | 474 | 5 | 6831 | gene;tss | 0.908726 | 0.939846 | Not queried | 1.85 | 5.48e-01 | 5.70e-01 |
|  | 2 | 219524425 | 219524576 | PDFSTATSENSEMBLUCSC | 152 | 4 | 23020 | gene | 0.005688 | 0.011140 | Not queried | 1.85 | 3.43e-01 | 3.84e-01 |
|  | 15 | 37782885 | 37783172 | PDFSTATSENSEMBLUCSC | 288 | 6 | 14016 | island | 0.901118 | 0.612573 | Not queried | 1.85 | 2.40e-01 | 2.95e-01 |
|  | 6 | 33398522 | 33399151 | PDFSTATSENSEMBLUCSC | 630 | 9 | 34290 | gene;island | 0.755727 | 0.240346 | Not queried | 1.85 | 2.58e-01 | 3.16e-01 |
|  | 1 | 207626214 | 207626535 | PDFSTATSENSEMBLUCSC | 322 | 4 | 2221 | tss;island | 0.473791 | 0.754420 | Not queried | 1.85 | 5.71e-02 | 1.00e-01 |
|  | 2 | 133173433 | 133173718 | PDFSTATSENSEMBLUCSC | 286 | 4 | 23638 | tss;island | 0.008380 | 0.028961 | Not queried | 1.85 | 3.43e-01 | 3.84e-01 |
|  | 6 | 53213606 | 53214258 | PDFSTATSENSEMBLUCSC | 653 | 12 | 36213 | island | 0.050779 | 0.031718 | Not queried | 1.85 | 2.19e-01 | 2.76e-01 |
|  | 22 | 32341378 | 32341780 | PDFSTATSENSEMBLUCSC | 403 | 9 | 27086 | tss | 0.004487 | 0.005639 | Not queried | 1.85 | 3.40e-01 | 3.84e-01 |
|  | 2 | 127865197 | 127865431 | PDFSTATSENSEMBLUCSC | 235 | 5 | 23609 | tss;island | 0.055313 | 0.326975 | Not queried | 1.85 | 2.22e-01 | 2.76e-01 |
|  | 12 | 62586552 | 62586892 | PDFSTATSENSEMBLUCSC | 341 | 6 | 9832 | tss | 0.025935 | 0.002636 | Not queried | 1.85 | 3.10e-01 | 3.66e-01 |
|  | 21 | 47404109 | 47404268 | PDFSTATSENSEMBLUCSC | 160 | 4 | 26472 | gene;island | 0.794034 | 0.926471 | Not queried | 1.84 | 2.00e-01 | 2.55e-01 |
|  | 7 | 49812836 | 49813111 | PDFSTATSENSEMBLUCSC | 276 | 7 | 37592 | tss;island | 0.906993 | 0.978739 | Not queried | 1.84 | 2.62e-02 | 6.12e-02 |
|  | 15 | 99548859 | 99549047 | PDFSTATSENSEMBLUCSC | 189 | 4 | 13501 | gene | 0.797889 | 0.970155 | Not queried | 1.84 | 1.14e-01 | 1.68e-01 |
|  | 20 | 2308707 | 2308988 | PDFSTATSENSEMBLUCSC | 282 | 4 | 25331 | gene;island | 0.799030 | 0.924632 | Not queried | 1.84 | 4.86e-01 | 5.11e-01 |
|  | 10 | 93392870 | 93393509 | PDFSTATSENSEMBLUCSC | 640 | 10 | 5223 | tss | 0.504912 | 0.842422 | PPP1R3C (margin: PPP1R3C) | 1.84 | 6.84e-03 | 3.19e-02 |
|  | 8 | 77589908 | 77590513 | PDFSTATSENSEMBLUCSC | 606 | 5 | 39341 | gene;island | 0.890911 | 0.694451 | Not queried | 1.84 | 5.48e-01 | 5.70e-01 |
|  | 18 | 21718735 | 21718979 | PDFSTATSENSEMBLUCSC | 245 | 10 | 19209 | tss;island | 0.012389 | 0.020388 | Not queried | 1.84 | 1.23e-01 | 1.81e-01 |
|  | 8 | 85096835 | 85097429 | PDFSTATSENSEMBLUCSC | 595 | 9 | 39352 | gene;island | 0.703274 | 0.467170 | Not queried | 1.84 | 5.03e-02 | 9.87e-02 |
|  | 10 | 131812411 | 131812878 | PDFSTATSENSEMBLUCSC | 468 | 6 | 6269 | island | 0.639172 | 0.956093 | Not queried | 1.84 | 6.49e-02 | 1.11e-01 |
|  | 2 | 175546916 | 175547126 | PDFSTATSENSEMBLUCSC | 211 | 4 | 22931 | gene;island | 0.340429 | 0.145220 | Not queried | 1.84 | 2.86e-02 | 6.12e-02 |
|  | 8 | 72274546 | 72274615 | PDFSTATSENSEMBLUCSC | 70 | 4 | 39762 | tss | 0.092384 | 0.044325 | Not queried | 1.84 | 6.86e-01 | 6.99e-01 |
|  | 7 | 100033938 | 100034454 | PDFSTATSENSEMBLUCSC | 517 | 8 | 38760 | island | 0.006642 | 0.019561 | Not queried | 1.84 | 2.79e-01 | 3.38e-01 |
|  | 4 | 189580331 | 189580798 | PDFSTATSENSEMBLUCSC | 468 | 5 | 31492 | island | 0.926504 | 0.978876 | Not queried | 1.84 | 8.41e-01 | 8.48e-01 |
|  | 14 | 21152202 | 21152358 | PDFSTATSENSEMBLUCSC | 157 | 4 | 12277 | tss | 0.011391 | 0.028208 | Not queried | 1.84 | 3.43e-01 | 3.84e-01 |
|  | 19 | 49944488 | 49944964 | PDFSTATSENSEMBLUCSC | 477 | 8 | 22266 | island | 0.084231 | 0.029944 | Not queried | 1.84 | 6.50e-02 | 1.11e-01 |
|  | 17 | 58156134 | 58156411 | PDFSTATSENSEMBLUCSC | 278 | 5 | 18616 | island | 0.004027 | 0.008897 | Not queried | 1.84 | 6.90e-01 | 7.01e-01 |
|  | 16 | 77468277 | 77468486 | PDFSTATSENSEMBLUCSC | 210 | 4 | 14848 | gene;island | 0.910015 | 0.650785 | Not queried | 1.84 | 1.14e-01 | 1.68e-01 |
|  | 6 | 37321643 | 37322018 | PDFSTATSENSEMBLUCSC | 376 | 6 | 36095 | island | 0.010341 | 0.029618 | Not queried | 1.84 | 2.40e-01 | 2.95e-01 |
|  | 11 | 46958255 | 46958558 | PDFSTATSENSEMBLUCSC | 304 | 4 | 6603 | gene | 0.092967 | 0.026402 | Not queried | 1.84 | 1.14e-01 | 1.68e-01 |
|  | 2 | 6121909 | 6122107 | PDFSTATSENSEMBLUCSC | 199 | 4 | 23165 | tss | 0.493768 | 0.247184 | Not queried | 1.84 | 2.86e-02 | 6.12e-02 |
|  | 1 | 204951031 | 204951336 | PDFSTATSENSEMBLUCSC | 306 | 4 | 961 | gene;island | 0.371982 | 0.839825 | Not queried | 1.84 | 2.00e-01 | 2.55e-01 |
|  | 17 | 58603287 | 58603815 | PDFSTATSENSEMBLUCSC | 529 | 11 | 18623 | island | 0.005256 | 0.018745 | Not queried | 1.84 | 1.01e-01 | 1.62e-01 |
|  | 5 | 43042108 | 43042233 | PDFSTATSENSEMBLUCSC | 126 | 4 | 32044 | tss | 0.006411 | 0.025110 | Not queried | 1.84 | 1.14e-01 | 1.68e-01 |
|  | 7 | 99680278 | 99680324 | PDFSTATSENSEMBLUCSC | 47 | 5 | 37766 | tss;island | 0.887611 | 0.980652 | Not queried | 1.84 | 3.17e-02 | 6.50e-02 |
|  | 15 | 83316640 | 83316911 | PDFSTATSENSEMBLUCSC | 272 | 4 | 14382 | island | 0.377104 | 0.530925 | Not queried | 1.84 | 4.86e-01 | 5.11e-01 |
|  | 5 | 140165938 | 140166213 | PDFSTATSENSEMBLUCSC | 276 | 4 | 31793 | gene;island | 0.674262 | 0.916788 | Not queried | 1.84 | 4.86e-01 | 5.11e-01 |
|  | 11 | 86383241 | 86383940 | PDFSTATSENSEMBLUCSC | 700 | 18 | 7610 | tss | 0.848051 | 0.967069 | ME3 (margin: ME3) | 1.84 | 2.11e-04 | 3.49e-03 |
|  | 3 | 186080023 | 186080359 | PDFSTATSENSEMBLUCSC | 337 | 7 | 28772 | tss | 0.718346 | 0.964279 | Not queried | 1.84 | 5.30e-02 | 1.00e-01 |
|  | 6 | 33142936 | 33143356 | PDFSTATSENSEMBLUCSC | 421 | 7 | 34203 | gene | 0.514600 | 0.759016 | Not queried | 1.84 | 3.83e-01 | 4.27e-01 |
|  | 6 | 152957910 | 152958664 | PDFSTATSENSEMBLUCSC | 755 | 15 | 36580 | island | 0.289092 | 0.053810 | Not queried | 1.84 | 5.39e-01 | 5.66e-01 |
|  | 6 | 27100370 | 27101279 | PDFSTATSENSEMBLUCSC | 910 | 13 | 34711 | tss;island | 0.000467 | 0.001460 | Not queried | 1.84 | 1.13e-01 | 1.68e-01 |
|  | 13 | 28674720 | 28675056 | PDFSTATSENSEMBLUCSC | 337 | 5 | 11637 | island | 0.420426 | 0.244445 | Not queried | 1.84 | 5.56e-02 | 1.00e-01 |
|  | 2 | 127413363 | 127413530 | PDFSTATSENSEMBLUCSC | 168 | 5 | 23608 | tss;island | 0.813154 | 0.946030 | Not queried | 1.84 | 3.10e-01 | 3.66e-01 |
|  | 12 | 132851783 | 132852053 | PDFSTATSENSEMBLUCSC | 271 | 4 | 9440 | gene;island | 0.240709 | 0.392041 | Not queried | 1.84 | 2.00e-01 | 2.55e-01 |
|  | 10 | 99532138 | 99532410 | PDFSTATSENSEMBLUCSC | 273 | 6 | 5273 | tss;island | 0.573650 | 0.814644 | Not queried | 1.84 | 4.11e-02 | 8.14e-02 |
|  | 2 | 119605761 | 119606746 | PDFSTATSENSEMBLUCSC | 986 | 10 | 23590 | tss;island | 0.750120 | 0.896964 | Not queried | 1.83 | 1.90e-01 | 2.55e-01 |
|  | 5 | 153863417 | 153863826 | PDFSTATSENSEMBLUCSC | 410 | 4 | 33333 | island | 0.606356 | 0.879899 | Not queried | 1.83 | 4.86e-01 | 5.11e-01 |
|  | 22 | 43547171 | 43547448 | PDFSTATSENSEMBLUCSC | 278 | 6 | 27202 | tss | 0.245863 | 0.027359 | Not queried | 1.83 | 4.85e-01 | 5.11e-01 |
|  | 2 | 196933546 | 196933901 | PDFSTATSENSEMBLUCSC | 356 | 8 | 23792 | tss | 0.087261 | 0.018468 | Not queried | 1.83 | 1.61e-01 | 2.24e-01 |
|  | 17 | 81042782 | 81043176 | PDFSTATSENSEMBLUCSC | 395 | 4 | 16975 | gene;island | 0.937568 | 0.978378 | Not queried | 1.83 | 2.00e-01 | 2.55e-01 |
|  | 17 | 1619840 | 1620044 | PDFSTATSENSEMBLUCSC | 205 | 4 | 16319 | gene | 0.003237 | 0.004718 | Not queried | 1.83 | 3.43e-01 | 3.84e-01 |
|  | 12 | 105478329 | 105478590 | PDFSTATSENSEMBLUCSC | 262 | 8 | 10742 | island | 0.024864 | 0.031131 | Not queried | 1.83 | 5.74e-01 | 5.97e-01 |
|  | 6 | 119669112 | 119669372 | PDFSTATSENSEMBLUCSC | 261 | 4 | 34459 | gene;island | 0.204212 | 0.223891 | Not queried | 1.83 | 8.86e-01 | 8.89e-01 |
|  | 19 | 12848324 | 12848676 | PDFSTATSENSEMBLUCSC | 353 | 5 | 19864 | gene | 0.002191 | 0.009747 | Not queried | 1.83 | 2.22e-01 | 2.76e-01 |
|  | 10 | 123356041 | 123356336 | PDFSTATSENSEMBLUCSC | 296 | 5 | 4795 | gene;island | 0.909282 | 0.952947 | Not queried | 1.83 | 1.51e-01 | 2.11e-01 |
|  | 14 | 76819107 | 76819459 | PDFSTATSENSEMBLUCSC | 353 | 5 | 13023 | island | 0.312755 | 0.534010 | Not queried | 1.83 | 9.52e-02 | 1.53e-01 |
|  | 1 | 151042743 | 151043263 | PDFSTATSENSEMBLUCSC | 521 | 10 | 3738 | island | 0.002570 | 0.009005 | Not queried | 1.83 | 2.80e-01 | 3.40e-01 |
|  | 15 | 25492314 | 25492469 | PDFSTATSENSEMBLUCSC | 156 | 5 | 13535 | tss | 0.529652 | 0.643089 | Not queried | 1.83 | 3.10e-01 | 3.66e-01 |
|  | 1 | 39956641 | 39956807 | PDFSTATSENSEMBLUCSC | 167 | 4 | 1522 | tss;island | 0.459420 | 0.225538 | Not queried | 1.83 | 2.00e-01 | 2.55e-01 |
|  | 14 | 105444384 | 105445253 | PDFSTATSENSEMBLUCSC | 870 | 10 | 13224 | island | 0.064936 | 0.032397 | Not queried | 1.83 | 1.65e-01 | 2.29e-01 |
|  | 14 | 105444724 | 105445253 | PDFSTATSENSEMBLUCSC | 530 | 8 | 12663 | tss | 0.104564 | 0.034299 | Not queried | 1.83 | 2.79e-01 | 3.38e-01 |
|  | 20 | 45313109 | 45313390 | PDFSTATSENSEMBLUCSC | 282 | 6 | 25440 | gene;tss | 0.623599 | 0.881495 | Not queried | 1.83 | 1.52e-02 | 5.36e-02 |
|  | 14 | 90722653 | 90722978 | PDFSTATSENSEMBLUCSC | 326 | 6 | 13072 | island | 0.002432 | 0.016994 | Not queried | 1.83 | 3.10e-01 | 3.66e-01 |
|  | 17 | 18684650 | 18685008 | PDFSTATSENSEMBLUCSC | 359 | 4 | 16475 | gene | 0.003249 | 0.008658 | Not queried | 1.83 | 1.14e-01 | 1.68e-01 |
|  | 19 | 18699118 | 18699444 | PDFSTATSENSEMBLUCSC | 327 | 9 | 20623 | tss | 0.243007 | 0.105332 | Not queried | 1.83 | 3.87e-01 | 4.30e-01 |
|  | 19 | 49944817 | 49944964 | PDFSTATSENSEMBLUCSC | 148 | 5 | 20945 | tss | 0.035551 | 0.017190 | Not queried | 1.83 | 9.52e-02 | 1.53e-01 |
|  | 5 | 72715731 | 72716122 | PDFSTATSENSEMBLUCSC | 392 | 5 | 32872 | island | 0.919866 | 0.978064 | Not queried | 1.83 | 5.48e-01 | 5.70e-01 |
|  | 8 | 133573739 | 133574073 | PDFSTATSENSEMBLUCSC | 335 | 5 | 39919 | tss | 0.016560 | 0.086227 | Not queried | 1.83 | 5.48e-01 | 5.70e-01 |
|  | 5 | 130500979 | 130501199 | PDFSTATSENSEMBLUCSC | 221 | 4 | 33077 | island | 0.000478 | 0.001908 | Not queried | 1.83 | 2.00e-01 | 2.55e-01 |
|  | 6 | 31464699 | 31465720 | PDFSTATSENSEMBLUCSC | 1022 | 19 | 34810 | tss | 0.025271 | 0.103527 | Not queried | 1.83 | 1.72e-01 | 2.37e-01 |
|  | 1 | 1102276 | 1102446 | PDFSTATSENSEMBLUCSC | 171 | 5 | 1139 | tss;island | 0.921516 | 0.981498 | Not queried | 1.83 | 1.59e-02 | 5.36e-02 |
|  | 13 | 28712342 | 28712670 | PDFSTATSENSEMBLUCSC | 329 | 4 | 11118 | gene;island | 0.005436 | 0.016025 | Not queried | 1.83 | 1.14e-01 | 1.68e-01 |
|  | 10 | 21783445 | 21783930 | PDFSTATSENSEMBLUCSC | 486 | 5 | 4525 | gene | 0.605772 | 0.982628 | Not queried | 1.83 | 3.10e-01 | 3.66e-01 |
|  | 19 | 37742739 | 37742956 | PDFSTATSENSEMBLUCSC | 218 | 4 | 21951 | island | 0.218250 | 0.514791 | Not queried | 1.83 | 2.86e-02 | 6.12e-02 |
|  | 6 | 14211743 | 14212256 | PDFSTATSENSEMBLUCSC | 514 | 5 | 35464 | island | 0.823357 | 0.515129 | Not queried | 1.83 | 8.41e-01 | 8.48e-01 |
|  | 7 | 157982161 | 157982589 | PDFSTATSENSEMBLUCSC | 429 | 5 | 37361 | gene;island | 0.049569 | 0.203953 | Not queried | 1.83 | 1.51e-01 | 2.11e-01 |
|  | 11 | 368351 | 369192 | PDFSTATSENSEMBLUCSC | 842 | 24 | 7009 | tss | 0.351758 | 0.134187 | B4GALNT4 (margin: B4GALNT4) | 1.83 | 9.36e-03 | 3.61e-02 |
|  | 4 | 187984412 | 187984718 | PDFSTATSENSEMBLUCSC | 307 | 4 | 31488 | island | 0.557301 | 0.764329 | Not queried | 1.83 | 5.71e-02 | 1.00e-01 |
|  | 22 | 20861940 | 20862326 | PDFSTATSENSEMBLUCSC | 387 | 6 | 26799 | gene | 0.003374 | 0.006485 | Not queried | 1.82 | 9.31e-02 | 1.53e-01 |
|  | 4 | 117520432 | 117520758 | PDFSTATSENSEMBLUCSC | 327 | 4 | 31263 | island | 0.754177 | 0.964481 | Not queried | 1.82 | 4.86e-01 | 5.11e-01 |
|  | 2 | 234296642 | 234297039 | PDFSTATSENSEMBLUCSC | 398 | 4 | 23073 | gene | 0.936807 | 0.980186 | Not queried | 1.82 | 1.14e-01 | 1.68e-01 |
|  | 10 | 8095121 | 8095363 | PDFSTATSENSEMBLUCSC | 243 | 4 | 4488 | gene | 0.368771 | 0.115714 | Not queried | 1.82 | 6.86e-01 | 6.99e-01 |
|  | 19 | 7562249 | 7562555 | PDFSTATSENSEMBLUCSC | 307 | 4 | 21450 | island | 0.763174 | 0.704157 | Not queried | 1.82 | 6.86e-01 | 6.99e-01 |
|  | 20 | 41818574 | 41818905 | PDFSTATSENSEMBLUCSC | 332 | 5 | 25748 | tss;island | 0.895644 | 0.967923 | Not queried | 1.82 | 3.17e-02 | 6.50e-02 |
|  | 13 | 100641646 | 100642106 | PDFSTATSENSEMBLUCSC | 461 | 6 | 11849 | island | 0.929146 | 0.977717 | (margin: ZIC2; LINC00554) | 1.82 | 2.16e-03 | 1.45e-02 |
|  | 14 | 60558421 | 60558592 | PDFSTATSENSEMBLUCSC | 172 | 5 | 12435 | tss | 0.002125 | 0.007292 | Not queried | 1.82 | 4.21e-01 | 4.59e-01 |
|  | 19 | 708469 | 708709 | PDFSTATSENSEMBLUCSC | 241 | 5 | 20278 | tss;island | 0.664630 | 0.436814 | Not queried | 1.82 | 4.21e-01 | 4.59e-01 |
|  | 12 | 4382878 | 4383281 | PDFSTATSENSEMBLUCSC | 404 | 6 | 10171 | island | 0.264336 | 0.057297 | Not queried | 1.82 | 1.32e-01 | 1.89e-01 |
|  | 1 | 22974053 | 22974243 | PDFSTATSENSEMBLUCSC | 191 | 5 | 308 | gene;island | 0.875088 | 0.958076 | Not queried | 1.82 | 3.10e-01 | 3.66e-01 |
|  | 2 | 136743411 | 136743698 | PDFSTATSENSEMBLUCSC | 288 | 5 | 23648 | tss;island | 0.630913 | 0.313948 | Not queried | 1.82 | 3.10e-01 | 3.66e-01 |
|  | 7 | 2739969 | 2740362 | PDFSTATSENSEMBLUCSC | 394 | 4 | 36813 | gene;island | 0.852459 | 0.968517 | Not queried | 1.82 | 6.86e-01 | 6.99e-01 |
|  | 19 | 58892495 | 58892618 | PDFSTATSENSEMBLUCSC | 124 | 5 | 21140 | tss;island | 0.000844 | 0.003559 | Not queried | 1.82 | 3.10e-01 | 3.66e-01 |
|  | 6 | 101846650 | 101846805 | PDFSTATSENSEMBLUCSC | 156 | 7 | 35143 | tss | 0.956468 | 0.982650 | Not queried | 1.82 | 7.28e-02 | 1.23e-01 |
|  | 1 | 1108820 | 1109244 | PDFSTATSENSEMBLUCSC | 425 | 6 | 1142 | tss | 0.924906 | 0.963249 | Not queried | 1.82 | 6.49e-02 | 1.11e-01 |
|  | 7 | 150754936 | 150755981 | PDFSTATSENSEMBLUCSC | 1046 | 23 | 39022 | island | 0.013311 | 0.047650 | SLC4A2; CDK5 (margin: ABCB8; ASIC3; SLC4A2; CDK5) | 1.82 | 3.35e-03 | 2.12e-02 |
|  | 6 | 5995766 | 5996657 | PDFSTATSENSEMBLUCSC | 892 | 8 | 35403 | island | 0.839664 | 0.772159 | Not queried | 1.82 | 6.45e-01 | 6.69e-01 |
|  | 1 | 42618041 | 42618506 | PDFSTATSENSEMBLUCSC | 466 | 4 | 1545 | tss | 0.551033 | 0.796376 | Not queried | 1.82 | 3.43e-01 | 3.84e-01 |
|  | 22 | 39640959 | 39641154 | PDFSTATSENSEMBLUCSC | 196 | 6 | 27150 | tss | 0.101303 | 0.027173 | PDGFB (margin: PDGFB) | 1.82 | 4.33e-03 | 2.52e-02 |
|  | 7 | 99149191 | 99149896 | PDFSTATSENSEMBLUCSC | 706 | 10 | 38737 | island | 0.021019 | 0.041426 | Not queried | 1.82 | 1.90e-01 | 2.55e-01 |
|  | 13 | 114220436 | 114220724 | PDFSTATSENSEMBLUCSC | 289 | 4 | 11972 | island | 0.934815 | 0.976890 | Not queried | 1.82 | 2.86e-02 | 6.12e-02 |
|  | 7 | 5085252 | 5085745 | PDFSTATSENSEMBLUCSC | 494 | 9 | 38210 | island | 0.006183 | 0.016231 | Not queried | 1.82 | 1.61e-01 | 2.25e-01 |
|  | 7 | 5085252 | 5085485 | PDFSTATSENSEMBLUCSC | 234 | 7 | 37419 | tss | 0.006183 | 0.016231 | Not queried | 1.82 | 1.65e-01 | 2.28e-01 |
|  | 14 | 70346151 | 70346477 | PDFSTATSENSEMBLUCSC | 327 | 5 | 12127 | gene | 0.797379 | 0.935126 | SMOC1 (margin: SMOC1; RPL7AP6) | 1.82 | 7.94e-03 | 3.19e-02 |
|  | 13 | 44544993 | 44545268 | PDFSTATSENSEMBLUCSC | 276 | 4 | 11702 | island | 0.808134 | 0.955234 | Not queried | 1.82 | 4.86e-01 | 5.11e-01 |
|  | 3 | 158518888 | 158519176 | PDFSTATSENSEMBLUCSC | 289 | 4 | 28687 | tss;island | 0.656044 | 0.931306 | Not queried | 1.82 | 3.43e-01 | 3.84e-01 |
|  | 11 | 76813828 | 76814245 | PDFSTATSENSEMBLUCSC | 418 | 4 | 6838 | gene;island | 0.738148 | 0.932407 | Not queried | 1.82 | 1.14e-01 | 1.68e-01 |
|  | 7 | 96647882 | 96648205 | PDFSTATSENSEMBLUCSC | 324 | 4 | 38693 | island | 0.703667 | 0.870714 | Not queried | 1.82 | 4.86e-01 | 5.11e-01 |
|  | 19 | 49109809 | 49109961 | PDFSTATSENSEMBLUCSC | 153 | 4 | 20130 | gene;tss;island | 0.857178 | 0.962259 | Not queried | 1.82 | 4.86e-01 | 5.11e-01 |
|  | 2 | 220491676 | 220492186 | PDFSTATSENSEMBLUCSC | 511 | 5 | 23912 | tss;island | 0.510414 | 0.832040 | Not queried | 1.82 | 5.48e-01 | 5.70e-01 |
|  | 6 | 168418276 | 168418527 | PDFSTATSENSEMBLUCSC | 252 | 7 | 35346 | tss | 0.233342 | 0.047794 | Not queried | 1.82 | 1.65e-01 | 2.28e-01 |
|  | 2 | 132202485 | 132202537 | PDFSTATSENSEMBLUCSC | 53 | 5 | 23633 | tss | 0.915657 | 0.757681 | Not queried | 1.81 | 5.48e-01 | 5.70e-01 |
|  | 20 | 52790589 | 52790733 | PDFSTATSENSEMBLUCSC | 145 | 4 | 25829 | tss;island | 0.363262 | 0.113318 | Not queried | 1.81 | 3.43e-01 | 3.84e-01 |
|  | 7 | 27170241 | 27171528 | PDFSTATSENSEMBLUCSC | 1288 | 22 | 38345 | island | 0.197600 | 0.022770 | Not queried | 1.81 | 4.40e-02 | 8.69e-02 |
|  | 4 | 184908254 | 184909018 | PDFSTATSENSEMBLUCSC | 765 | 9 | 30196 | gene | 0.023337 | 0.162270 | Not queried | 1.81 | 2.97e-01 | 3.60e-01 |
|  | 15 | 25958878 | 25959239 | PDFSTATSENSEMBLUCSC | 362 | 5 | 13258 | gene;island | 0.804856 | 0.918193 | Not queried | 1.81 | 4.21e-01 | 4.59e-01 |
|  | 7 | 56031952 | 56032122 | PDFSTATSENSEMBLUCSC | 171 | 6 | 37606 | tss;island | 0.034393 | 0.010456 | GBAS (margin: MRPS17; GBAS) | 1.81 | 8.66e-03 | 3.36e-02 |
|  | 19 | 10426722 | 10426933 | PDFSTATSENSEMBLUCSC | 212 | 4 | 20445 | tss;island | 0.009758 | 0.022092 | Not queried | 1.81 | 3.43e-01 | 3.84e-01 |
|  | 18 | 77793182 | 77793554 | PDFSTATSENSEMBLUCSC | 373 | 5 | 19340 | tss;island | 0.072474 | 0.007404 | Not queried | 1.81 | 5.48e-01 | 5.70e-01 |
|  | 12 | 1754980 | 1755322 | PDFSTATSENSEMBLUCSC | 343 | 5 | 8999 | gene;island | 0.928063 | 0.980717 | Not queried | 1.81 | 3.10e-01 | 3.66e-01 |
|  | 3 | 52091484 | 52091829 | PDFSTATSENSEMBLUCSC | 346 | 4 | 28371 | tss;island | 0.006912 | 0.015051 | Not queried | 1.81 | 6.86e-01 | 6.99e-01 |
|  | 15 | 44116616 | 44117354 | PDFSTATSENSEMBLUCSC | 739 | 10 | 14094 | island | 0.012457 | 0.018832 | Not queried | 1.81 | 7.96e-01 | 8.06e-01 |
|  | 1 | 112938339 | 112938731 | PDFSTATSENSEMBLUCSC | 393 | 6 | 1833 | tss;island | 0.005011 | 0.007635 | Not queried | 1.81 | 2.40e-01 | 2.95e-01 |
|  | 1 | 154474344 | 154474530 | PDFSTATSENSEMBLUCSC | 187 | 4 | 778 | gene;tss;island | 0.365605 | 0.667005 | Not queried | 1.81 | 3.43e-01 | 3.84e-01 |
|  | 1 | 115259226 | 115259668 | PDFSTATSENSEMBLUCSC | 443 | 4 | 701 | gene | 0.004767 | 0.013489 | Not queried | 1.81 | 6.86e-01 | 6.99e-01 |
|  | 19 | 39694477 | 39695237 | PDFSTATSENSEMBLUCSC | 761 | 9 | 21990 | island | 0.928189 | 0.974284 | Not queried | 1.81 | 1.36e-01 | 1.94e-01 |
|  | 1 | 68512539 | 68513063 | PDFSTATSENSEMBLUCSC | 525 | 8 | 573 | gene;island | 0.673881 | 0.944159 | Not queried | 1.81 | 1.95e-01 | 2.55e-01 |
|  | 4 | 146403866 | 146404134 | PDFSTATSENSEMBLUCSC | 269 | 8 | 30146 | gene;island | 0.000917 | 0.002229 | Not queried | 1.81 | 2.34e-01 | 2.90e-01 |
|  | 14 | 24801616 | 24802150 | PDFSTATSENSEMBLUCSC | 535 | 4 | 12047 | gene;island | 0.828112 | 0.944812 | Not queried | 1.81 | 3.43e-01 | 3.84e-01 |
|  | 16 | 56701994 | 56702158 | PDFSTATSENSEMBLUCSC | 165 | 5 | 15251 | tss | 0.718370 | 0.960824 | Not queried | 1.81 | 9.52e-02 | 1.53e-01 |
|  | 18 | 21852118 | 21852436 | PDFSTATSENSEMBLUCSC | 319 | 6 | 19078 | gene;island | 0.008367 | 0.013997 | Not queried | 1.81 | 1.80e-01 | 2.45e-01 |
|  | 1 | 205818956 | 205819251 | PDFSTATSENSEMBLUCSC | 296 | 4 | 970 | gene | 0.889339 | 0.987265 | Not queried | 1.81 | 1.14e-01 | 1.68e-01 |
|  | 15 | 37110422 | 37110854 | PDFSTATSENSEMBLUCSC | 433 | 7 | 14008 | island | 0.977494 | 0.901277 | Not queried | 1.81 | 2.09e-01 | 2.65e-01 |
|  | 1 | 78511600 | 78512129 | PDFSTATSENSEMBLUCSC | 530 | 5 | 595 | gene | 0.816752 | 0.967666 | Not queried | 1.81 | 3.17e-02 | 6.50e-02 |
|  | 8 | 23423734 | 23423960 | PDFSTATSENSEMBLUCSC | 227 | 4 | 39243 | gene | 0.958099 | 0.982808 | Not queried | 1.81 | 3.43e-01 | 3.84e-01 |
|  | 8 | 1132050 | 1132189 | PDFSTATSENSEMBLUCSC | 140 | 4 | 40016 | island | 0.100734 | 0.339641 | Not queried | 1.81 | 1.14e-01 | 1.68e-01 |
|  | 22 | 45680498 | 45680844 | PDFSTATSENSEMBLUCSC | 347 | 9 | 27220 | tss | 0.752770 | 0.491985 | Not queried | 1.81 | 1.88e-02 | 6.12e-02 |
|  | 1 | 151031286 | 151031323 | PDFSTATSENSEMBLUCSC | 38 | 4 | 747 | gene;tss;island | 0.006464 | 0.009220 | Not queried | 1.81 | 4.86e-01 | 5.11e-01 |
|  | 10 | 50747084 | 50747328 | PDFSTATSENSEMBLUCSC | 245 | 5 | 5734 | island | 0.003404 | 0.011847 | Not queried | 1.81 | 5.48e-01 | 5.70e-01 |
|  | 13 | 21100191 | 21100460 | PDFSTATSENSEMBLUCSC | 270 | 4 | 11332 | tss;island | 0.002403 | 0.005575 | Not queried | 1.81 | 1.14e-01 | 1.68e-01 |
|  | 14 | 75389066 | 75389280 | PDFSTATSENSEMBLUCSC | 215 | 4 | 13009 | island | 0.790603 | 0.600998 | Not queried | 1.81 | 3.43e-01 | 3.84e-01 |
|  | 18 | 44676656 | 44676764 | PDFSTATSENSEMBLUCSC | 109 | 4 | 19107 | gene | 0.002164 | 0.003197 | Not queried | 1.81 | 1.14e-01 | 1.68e-01 |
|  | 16 | 2892518 | 2892994 | PDFSTATSENSEMBLUCSC | 477 | 8 | 15620 | island | 0.974454 | 0.855248 | Not queried | 1.81 | 3.79e-02 | 7.66e-02 |
|  | 1 | 155231766 | 155232061 | PDFSTATSENSEMBLUCSC | 296 | 6 | 796 | gene | 0.009901 | 0.009871 | Not queried | 1.81 | 2.40e-01 | 2.95e-01 |
|  | 2 | 25016078 | 25016199 | PDFSTATSENSEMBLUCSC | 122 | 5 | 22599 | gene | 0.003534 | 0.008343 | Not queried | 1.81 | 4.21e-01 | 4.59e-01 |
|  | 14 | 24682679 | 24682916 | PDFSTATSENSEMBLUCSC | 238 | 4 | 12039 | gene;island | 0.005271 | 0.010950 | Not queried | 1.81 | 4.86e-01 | 5.11e-01 |
|  | 19 | 11545949 | 11546473 | PDFSTATSENSEMBLUCSC | 525 | 10 | 20473 | tss;island | 0.003471 | 0.006332 | Not queried | 1.80 | 7.53e-02 | 1.27e-01 |
|  | 8 | 109455254 | 109455575 | PDFSTATSENSEMBLUCSC | 322 | 5 | 39868 | tss;island | 0.004779 | 0.012894 | Not queried | 1.80 | 9.52e-02 | 1.53e-01 |
|  | 8 | 42010142 | 42010433 | PDFSTATSENSEMBLUCSC | 292 | 8 | 39676 | tss | 0.059180 | 0.008099 | Not queried | 1.80 | 1.30e-01 | 1.89e-01 |
|  | 6 | 31477847 | 31478432 | PDFSTATSENSEMBLUCSC | 586 | 7 | 33887 | gene | 0.773553 | 0.435389 | Not queried | 1.80 | 1.65e-01 | 2.28e-01 |
|  | 11 | 1036677 | 1036938 | PDFSTATSENSEMBLUCSC | 262 | 8 | 7897 | island | 0.153004 | 0.411969 | Not queried | 1.80 | 3.79e-02 | 7.66e-02 |
|  | 19 | 55866246 | 55866412 | PDFSTATSENSEMBLUCSC | 167 | 7 | 21076 | tss | 0.214826 | 0.552122 | Not queried | 1.80 | 2.59e-01 | 3.16e-01 |
|  | 3 | 8810980 | 8811279 | PDFSTATSENSEMBLUCSC | 300 | 4 | 27647 | gene | 0.081818 | 0.188510 | Not queried | 1.80 | 3.43e-01 | 3.84e-01 |
|  | 5 | 32585221 | 32585895 | PDFSTATSENSEMBLUCSC | 675 | 10 | 32718 | island | 0.002353 | 0.006062 | Not queried | 1.80 | 3.93e-01 | 4.36e-01 |
|  | 2 | 209271164 | 209271505 | PDFSTATSENSEMBLUCSC | 342 | 5 | 23850 | tss | 0.892352 | 0.977309 | PTH2R (margin: PTH2R) | 1.80 | 7.94e-03 | 3.19e-02 |
|  | 4 | 38869436 | 38869779 | PDFSTATSENSEMBLUCSC | 344 | 8 | 29979 | gene | 0.009505 | 0.040178 | Not queried | 1.80 | 1.95e-01 | 2.55e-01 |
|  | 17 | 58218643 | 58218965 | PDFSTATSENSEMBLUCSC | 323 | 4 | 18619 | island | 0.504368 | 0.780985 | Not queried | 1.80 | 2.00e-01 | 2.55e-01 |
|  | 17 | 15686294 | 15686473 | PDFSTATSENSEMBLUCSC | 180 | 4 | 18043 | island | 0.929459 | 0.981481 | Not queried | 1.80 | 1.14e-01 | 1.68e-01 |
|  | 6 | 13924355 | 13924708 | PDFSTATSENSEMBLUCSC | 354 | 6 | 34647 | tss;island | 0.557942 | 0.789993 | Not queried | 1.80 | 4.85e-01 | 5.11e-01 |
|  | 8 | 65711274 | 65711658 | PDFSTATSENSEMBLUCSC | 385 | 9 | 40349 | island | 0.498841 | 0.790515 | Not queried | 1.80 | 3.15e-02 | 6.50e-02 |
|  | 5 | 140566303 | 140566767 | PDFSTATSENSEMBLUCSC | 465 | 5 | 32365 | tss;island | 0.302648 | 0.444782 | Not queried | 1.80 | 5.48e-01 | 5.70e-01 |
|  | 5 | 134783075 | 134783273 | PDFSTATSENSEMBLUCSC | 199 | 4 | 32304 | tss | 0.458227 | 0.796166 | Not queried | 1.80 | 5.71e-02 | 1.00e-01 |
|  | 3 | 131221830 | 131222106 | PDFSTATSENSEMBLUCSC | 277 | 7 | 28595 | tss | 0.002181 | 0.004433 | Not queried | 1.80 | 3.83e-01 | 4.27e-01 |
|  | 7 | 86974950 | 86975244 | PDFSTATSENSEMBLUCSC | 295 | 6 | 37071 | gene | 0.005469 | 0.010533 | Not queried | 1.80 | 2.40e-01 | 2.95e-01 |
|  | 1 | 144533778 | 144533951 | PDFSTATSENSEMBLUCSC | 174 | 4 | 3672 | island | 0.320351 | 0.633612 | Not queried | 1.80 | 2.86e-02 | 6.12e-02 |
|  | 6 | 26216541 | 26217212 | PDFSTATSENSEMBLUCSC | 672 | 8 | 34696 | tss;island | 0.000274 | 0.000597 | Not queried | 1.80 | 5.05e-01 | 5.32e-01 |
|  | 6 | 31795003 | 31795436 | PDFSTATSENSEMBLUCSC | 434 | 14 | 34841 | tss | 0.002602 | 0.006997 | Not queried | 1.80 | 3.06e-01 | 3.66e-01 |
|  | 1 | 228466481 | 228466971 | PDFSTATSENSEMBLUCSC | 491 | 5 | 1053 | gene;island | 0.802832 | 0.955499 | Not queried | 1.80 | 9.52e-02 | 1.53e-01 |
|  | 5 | 76011127 | 76011428 | PDFSTATSENSEMBLUCSC | 302 | 5 | 32135 | tss;island | 0.780338 | 0.919938 | Not queried | 1.80 | 4.21e-01 | 4.59e-01 |
|  | 20 | 61051317 | 61052060 | PDFSTATSENSEMBLUCSC | 744 | 18 | 25872 | tss;island | 0.721073 | 0.921595 | RP13-379O24.3; GATA5 (margin: RP13-379O24.3; GATA5) | 1.80 | 1.32e-04 | 2.71e-03 |
|  | 10 | 50886878 | 50887212 | PDFSTATSENSEMBLUCSC | 335 | 5 | 5075 | tss;island | 0.651013 | 0.702711 | Not queried | 1.80 | 4.21e-01 | 4.59e-01 |
|  | 1 | 1959442 | 1959832 | PDFSTATSENSEMBLUCSC | 391 | 6 | 52 | gene;island | 0.847438 | 0.964843 | Not queried | 1.80 | 9.31e-02 | 1.53e-01 |
|  | 1 | 26202137 | 26202601 | PDFSTATSENSEMBLUCSC | 465 | 5 | 2880 | island | 0.009806 | 0.038214 | Not queried | 1.80 | 1.51e-01 | 2.11e-01 |
|  | 6 | 31927895 | 31928257 | PDFSTATSENSEMBLUCSC | 363 | 5 | 34855 | tss | 0.967005 | 0.917797 | Not queried | 1.80 | 5.56e-02 | 1.00e-01 |
|  | 1 | 111743020 | 111743537 | PDFSTATSENSEMBLUCSC | 518 | 7 | 3588 | island | 0.827784 | 0.965186 | Not queried | 1.80 | 1.65e-01 | 2.28e-01 |
|  | 1 | 240656217 | 240656737 | PDFSTATSENSEMBLUCSC | 521 | 6 | 1106 | gene;island | 0.746048 | 0.916058 | Not queried | 1.80 | 9.31e-02 | 1.53e-01 |
|  | 6 | 32817662 | 32818477 | PDFSTATSENSEMBLUCSC | 816 | 12 | 34154 | gene;island | 0.985170 | 0.948747 | PSMB9; TAP1 (margin: XXbac-BPG246D15.8; PSMB9; TAP2; PSMB8; TAP1) | 1.80 | 1.83e-03 | 1.45e-02 |
|  | 5 | 14581404 | 14582133 | PDFSTATSENSEMBLUCSC | 730 | 10 | 32697 | island | 0.226007 | 0.095072 | Not queried | 1.80 | 2.80e-01 | 3.40e-01 |
|  | 5 | 127872767 | 127873711 | PDFSTATSENSEMBLUCSC | 945 | 7 | 31739 | gene;island | 0.605503 | 0.924335 | Not queried | 1.79 | 4.56e-01 | 4.96e-01 |
|  | 4 | 11430353 | 11430908 | PDFSTATSENSEMBLUCSC | 556 | 9 | 30928 | island | 0.010810 | 0.042721 | HS3ST1 (margin: HS3ST1) | 1.79 | 2.76e-03 | 1.78e-02 |
|  | 4 | 11430552 | 11430908 | PDFSTATSENSEMBLUCSC | 357 | 7 | 30302 | tss | 0.010992 | 0.051810 | HS3ST1 (margin: HS3ST1) | 1.79 | 4.08e-03 | 2.47e-02 |
|  | 11 | 134147143 | 134147634 | PDFSTATSENSEMBLUCSC | 492 | 4 | 6994 | gene;island | 0.902980 | 0.973807 | Not queried | 1.79 | 4.86e-01 | 5.11e-01 |
|  | 8 | 41504884 | 41505042 | PDFSTATSENSEMBLUCSC | 159 | 5 | 39671 | tss | 0.612834 | 0.862125 | NKX6-3 (margin: NKX6-3; ANK1) | 1.79 | 7.94e-03 | 3.19e-02 |
|  | 8 | 41504566 | 41505042 | PDFSTATSENSEMBLUCSC | 477 | 7 | 40247 | island | 0.612834 | 0.892720 | NKX6-3 (margin: NKX6-3; ANK1) | 1.79 | 6.99e-03 | 3.19e-02 |
|  | 14 | 70345898 | 70346477 | PDFSTATSENSEMBLUCSC | 580 | 10 | 12974 | island | 0.695844 | 0.874666 | SMOC1 (margin: SMOC1; RPL7AP6) | 1.79 | 8.93e-03 | 3.45e-02 |
|  | 1 | 6051551 | 6051640 | PDFSTATSENSEMBLUCSC | 90 | 4 | 158 | gene;island | 0.152846 | 0.027558 | Not queried | 1.79 | 6.86e-01 | 6.99e-01 |
|  | 19 | 1248287 | 1248689 | PDFSTATSENSEMBLUCSC | 403 | 6 | 21206 | island | 0.009291 | 0.234843 | Not queried | 1.79 | 1.32e-01 | 1.89e-01 |
|  | 7 | 73867797 | 73868174 | PDFSTATSENSEMBLUCSC | 378 | 10 | 37649 | tss;island | 0.008884 | 0.079752 | Not queried | 1.79 | 2.47e-01 | 3.03e-01 |
|  | 1 | 155145515 | 155145786 | PDFSTATSENSEMBLUCSC | 272 | 6 | 794 | gene | 0.002264 | 0.004784 | Not queried | 1.79 | 3.10e-01 | 3.66e-01 |
|  | 14 | 57283942 | 57284319 | PDFSTATSENSEMBLUCSC | 378 | 4 | 12093 | gene;island | 0.713025 | 0.923373 | Not queried | 1.79 | 2.00e-01 | 2.55e-01 |
|  | 16 | 28943019 | 28943232 | PDFSTATSENSEMBLUCSC | 214 | 4 | 15161 | tss | 0.752630 | 0.900423 | Not queried | 1.79 | 1.14e-01 | 1.68e-01 |
|  | 6 | 34984836 | 34984953 | PDFSTATSENSEMBLUCSC | 118 | 4 | 34308 | gene | 0.934696 | 0.980998 | Not queried | 1.79 | 2.00e-01 | 2.55e-01 |
|  | 11 | 94502349 | 94502879 | PDFSTATSENSEMBLUCSC | 531 | 8 | 6869 | gene;island | 0.022442 | 0.026537 | Not queried | 1.79 | 5.74e-01 | 5.97e-01 |
|  | 6 | 31165567 | 31166174 | PDFSTATSENSEMBLUCSC | 608 | 20 | 33863 | gene | 0.008354 | 0.025829 | Not queried | 1.79 | 2.45e-02 | 6.12e-02 |
|  | 19 | 57078727 | 57078794 | PDFSTATSENSEMBLUCSC | 68 | 5 | 21101 | tss;island | 0.967724 | 0.898924 | Not queried | 1.79 | 1.51e-01 | 2.11e-01 |
|  | 19 | 54927528 | 54928185 | PDFSTATSENSEMBLUCSC | 658 | 5 | 20221 | gene;island | 0.507868 | 0.760311 | Not queried | 1.79 | 4.21e-01 | 4.59e-01 |
|  | 5 | 92916862 | 92917125 | PDFSTATSENSEMBLUCSC | 264 | 4 | 32959 | island | 0.009272 | 0.002322 | Not queried | 1.79 | 2.00e-01 | 2.55e-01 |
|  | 10 | 27703247 | 27703547 | PDFSTATSENSEMBLUCSC | 301 | 8 | 5630 | island | 0.537681 | 0.897353 | Not queried | 1.79 | 1.30e-01 | 1.89e-01 |
|  | 10 | 27703329 | 27703547 | PDFSTATSENSEMBLUCSC | 219 | 6 | 5002 | tss | 0.749252 | 0.968204 | Not queried | 1.79 | 2.40e-01 | 2.95e-01 |
|  | 2 | 11774142 | 11774474 | PDFSTATSENSEMBLUCSC | 333 | 5 | 22573 | gene;island | 0.827890 | 0.959288 | Not queried | 1.79 | 5.56e-02 | 1.00e-01 |
|  | 8 | 10928985 | 10929210 | PDFSTATSENSEMBLUCSC | 226 | 4 | 39203 | gene;island | 0.364686 | 0.658350 | Not queried | 1.79 | 5.71e-02 | 1.00e-01 |
|  | 17 | 40575462 | 40575582 | PDFSTATSENSEMBLUCSC | 121 | 6 | 17362 | tss | 0.010260 | 0.022280 | Not queried | 1.79 | 3.94e-01 | 4.36e-01 |
|  | 5 | 110074217 | 110074725 | PDFSTATSENSEMBLUCSC | 509 | 10 | 32205 | tss | 0.004491 | 0.013248 | Not queried | 1.79 | 1.23e-01 | 1.81e-01 |
|  | 1 | 27693782 | 27693942 | PDFSTATSENSEMBLUCSC | 161 | 4 | 1410 | tss;island | 0.451005 | 0.718239 | Not queried | 1.79 | 4.86e-01 | 5.11e-01 |
|  | 17 | 19483367 | 19483721 | PDFSTATSENSEMBLUCSC | 355 | 5 | 18100 | island | 0.926587 | 0.977364 | Not queried | 1.79 | 5.56e-02 | 1.00e-01 |
|  | 1 | 39284267 | 39284609 | PDFSTATSENSEMBLUCSC | 343 | 4 | 3092 | island | 0.549281 | 0.247015 | Not queried | 1.79 | 6.86e-01 | 6.99e-01 |
|  | 8 | 41386345 | 41386637 | PDFSTATSENSEMBLUCSC | 293 | 5 | 39669 | tss | 0.016080 | 0.017892 | Not queried | 1.79 | 6.90e-01 | 7.01e-01 |
|  | 7 | 2742274 | 2742484 | PDFSTATSENSEMBLUCSC | 211 | 5 | 36814 | gene;island | 0.898792 | 0.950247 | Not queried | 1.79 | 3.17e-02 | 6.50e-02 |
|  | 14 | 31343146 | 31343427 | PDFSTATSENSEMBLUCSC | 282 | 4 | 12338 | tss;island | 0.979812 | 0.901781 | Not queried | 1.79 | 2.00e-01 | 2.55e-01 |
|  | 1 | 150980086 | 150980411 | PDFSTATSENSEMBLUCSC | 326 | 4 | 744 | gene;tss;island | 0.531621 | 0.781548 | Not queried | 1.79 | 4.86e-01 | 5.11e-01 |
|  | 15 | 53097649 | 53097840 | PDFSTATSENSEMBLUCSC | 192 | 4 | 14154 | island | 0.801149 | 0.978388 | Not queried | 1.79 | 3.43e-01 | 3.84e-01 |
|  | 20 | 30458612 | 30458745 | PDFSTATSENSEMBLUCSC | 134 | 5 | 25385 | gene;tss;island | 0.598584 | 0.778600 | Not queried | 1.79 | 1.59e-02 | 5.36e-02 |
|  | 20 | 33577997 | 33578276 | PDFSTATSENSEMBLUCSC | 280 | 5 | 25403 | gene | 0.695290 | 0.910471 | Not queried | 1.79 | 4.21e-01 | 4.59e-01 |
|  | 5 | 140792511 | 140792992 | PDFSTATSENSEMBLUCSC | 482 | 8 | 31832 | gene;island | 0.561608 | 0.189501 | Not queried | 1.79 | 7.21e-01 | 7.30e-01 |
|  | 11 | 33758121 | 33758529 | PDFSTATSENSEMBLUCSC | 409 | 7 | 7235 | tss | 0.010522 | 0.003982 | Not queried | 1.79 | 1.65e-01 | 2.28e-01 |
|  | 16 | 28996270 | 28996362 | PDFSTATSENSEMBLUCSC | 93 | 4 | 15165 | tss | 0.894140 | 0.956525 | Not queried | 1.79 | 3.43e-01 | 3.84e-01 |
|  | 20 | 25129126 | 25129400 | PDFSTATSENSEMBLUCSC | 275 | 4 | 25378 | gene | 0.714991 | 0.930001 | Not queried | 1.79 | 5.71e-02 | 1.00e-01 |
|  | 18 | 77160030 | 77160396 | PDFSTATSENSEMBLUCSC | 367 | 7 | 19136 | gene;island | 0.072825 | 0.029681 | Not queried | 1.79 | 1.28e-01 | 1.87e-01 |
|  | 4 | 165898825 | 165898967 | PDFSTATSENSEMBLUCSC | 143 | 6 | 30666 | tss | 0.751949 | 0.942220 | Not queried | 1.79 | 3.94e-01 | 4.36e-01 |
|  | 3 | 33155133 | 33155642 | PDFSTATSENSEMBLUCSC | 510 | 11 | 28971 | island | 0.004627 | 0.008050 | Not queried | 1.79 | 3.00e-01 | 3.63e-01 |
|  | 3 | 33155133 | 33155354 | PDFSTATSENSEMBLUCSC | 222 | 9 | 28211 | tss | 0.004627 | 0.008050 | Not queried | 1.79 | 3.87e-01 | 4.30e-01 |
|  | 10 | 5060291 | 5060566 | PDFSTATSENSEMBLUCSC | 276 | 4 | 4921 | tss | 0.971503 | 0.888734 | Not queried | 1.79 | 2.00e-01 | 2.55e-01 |
|  | 6 | 149285724 | 149285963 | PDFSTATSENSEMBLUCSC | 240 | 4 | 34503 | gene | 0.967333 | 0.982544 | Not queried | 1.79 | 2.00e-01 | 2.55e-01 |
|  | 15 | 49338748 | 49339070 | PDFSTATSENSEMBLUCSC | 323 | 5 | 13661 | tss | 0.032090 | 0.051934 | Not queried | 1.78 | 1.00e+00 | 1.00e+00 |
|  | 12 | 62860029 | 62860470 | PDFSTATSENSEMBLUCSC | 442 | 8 | 9834 | tss;island | 0.027022 | 0.003092 | Not queried | 1.78 | 1.05e-01 | 1.66e-01 |
|  | 19 | 13985513 | 13985688 | PDFSTATSENSEMBLUCSC | 176 | 4 | 19885 | gene | 0.858711 | 0.806718 | Not queried | 1.78 | 6.86e-01 | 6.99e-01 |
|  | 2 | 128458240 | 128458556 | PDFSTATSENSEMBLUCSC | 317 | 6 | 23616 | tss | 0.151203 | 0.350302 | Not queried | 1.78 | 3.10e-01 | 3.66e-01 |
|  | 21 | 46875142 | 46875417 | PDFSTATSENSEMBLUCSC | 276 | 4 | 26596 | tss | 0.576278 | 0.285944 | Not queried | 1.78 | 1.14e-01 | 1.68e-01 |
|  | 8 | 33580365 | 33580539 | PDFSTATSENSEMBLUCSC | 175 | 4 | 40216 | island | 0.355133 | 0.614647 | Not queried | 1.78 | 2.00e-01 | 2.55e-01 |
|  | 19 | 17829972 | 17830251 | PDFSTATSENSEMBLUCSC | 280 | 7 | 20597 | tss | 0.010836 | 0.021489 | Not queried | 1.78 | 2.59e-01 | 3.16e-01 |
|  | 7 | 150211761 | 150211939 | PDFSTATSENSEMBLUCSC | 179 | 5 | 37981 | tss | 0.466256 | 0.631862 | Not queried | 1.78 | 1.51e-01 | 2.11e-01 |
|  | 16 | 68770944 | 68771182 | PDFSTATSENSEMBLUCSC | 239 | 6 | 15323 | tss | 0.068209 | 0.030980 | Not queried | 1.78 | 1.80e-01 | 2.45e-01 |
|  | 8 | 54507118 | 54507532 | PDFSTATSENSEMBLUCSC | 415 | 5 | 40297 | island | 0.965845 | 0.984632 | Not queried | 1.78 | 1.59e-02 | 5.36e-02 |
|  | 3 | 196754322 | 196754806 | PDFSTATSENSEMBLUCSC | 485 | 5 | 28102 | gene;island | 0.943024 | 0.979666 | Not queried | 1.78 | 1.59e-02 | 5.36e-02 |
|  | 17 | 80023822 | 80024326 | PDFSTATSENSEMBLUCSC | 505 | 8 | 17767 | tss;island | 0.031303 | 0.138451 | Not queried | 1.78 | 3.82e-01 | 4.27e-01 |
|  | 16 | 88729471 | 88729968 | PDFSTATSENSEMBLUCSC | 498 | 10 | 15411 | tss;island | 0.003766 | 0.006365 | Not queried | 1.78 | 7.39e-01 | 7.49e-01 |
|  | 11 | 64360724 | 64361020 | PDFSTATSENSEMBLUCSC | 297 | 6 | 6690 | gene | 0.573100 | 0.839890 | SLC22A12 (margin: SLC22A12) | 1.78 | 2.16e-03 | 1.45e-02 |
|  | 1 | 203320386 | 203320541 | PDFSTATSENSEMBLUCSC | 156 | 4 | 2192 | tss | 0.527345 | 0.855293 | Not queried | 1.78 | 2.00e-01 | 2.55e-01 |
|  | 8 | 143407432 | 143407817 | PDFSTATSENSEMBLUCSC | 386 | 4 | 39460 | gene;island | 0.861601 | 0.945846 | Not queried | 1.78 | 2.00e-01 | 2.55e-01 |
|  | 2 | 224866411 | 224866636 | PDFSTATSENSEMBLUCSC | 226 | 6 | 23054 | gene | 0.944206 | 0.981469 | Not queried | 1.78 | 3.10e-01 | 3.66e-01 |
|  | 1 | 107682674 | 107682963 | PDFSTATSENSEMBLUCSC | 290 | 4 | 657 | gene | 0.487396 | 0.758303 | Not queried | 1.78 | 2.86e-02 | 6.12e-02 |
|  | 10 | 133955398 | 133955570 | PDFSTATSENSEMBLUCSC | 173 | 5 | 4855 | gene;island | 0.956966 | 0.987005 | Not queried | 1.78 | 5.48e-01 | 5.70e-01 |
|  | 17 | 66243994 | 66244127 | PDFSTATSENSEMBLUCSC | 134 | 7 | 17605 | tss | 0.002156 | 0.006302 | Not queried | 1.78 | 9.73e-02 | 1.55e-01 |
|  | 17 | 66243994 | 66244249 | PDFSTATSENSEMBLUCSC | 256 | 9 | 18689 | island | 0.002156 | 0.005391 | Not queried | 1.78 | 1.61e-01 | 2.25e-01 |
|  | 2 | 105857809 | 105858511 | PDFSTATSENSEMBLUCSC | 703 | 11 | 24584 | island | 0.751188 | 0.953297 | Not queried | 1.78 | 1.33e-01 | 1.91e-01 |
|  | 14 | 102771635 | 102771717 | PDFSTATSENSEMBLUCSC | 83 | 5 | 12642 | tss | 0.008439 | 0.020418 | Not queried | 1.78 | 2.22e-01 | 2.76e-01 |
|  | 16 | 88803803 | 88804051 | PDFSTATSENSEMBLUCSC | 249 | 4 | 14914 | gene;island | 0.026334 | 0.158627 | Not queried | 1.78 | 1.14e-01 | 1.68e-01 |
|  | 6 | 144329672 | 144330345 | PDFSTATSENSEMBLUCSC | 674 | 15 | 35269 | tss | 0.929844 | 0.776605 | Not queried | 1.78 | 2.67e-01 | 3.25e-01 |
|  | 11 | 2019079 | 2019167 | PDFSTATSENSEMBLUCSC | 89 | 5 | 7054 | tss;island | 0.280163 | 0.051042 | Not queried | 1.78 | 5.56e-02 | 1.00e-01 |
|  | 14 | 24685205 | 24685421 | PDFSTATSENSEMBLUCSC | 217 | 6 | 12737 | island | 0.011894 | 0.016055 | Not queried | 1.78 | 1.80e-01 | 2.45e-01 |
|  | 11 | 66059064 | 66059164 | PDFSTATSENSEMBLUCSC | 101 | 4 | 7463 | tss;island | 0.814945 | 0.606125 | Not queried | 1.78 | 3.43e-01 | 3.84e-01 |
|  | 16 | 86542659 | 86543092 | PDFSTATSENSEMBLUCSC | 434 | 10 | 15400 | tss;island | 0.411848 | 0.264777 | Not queried | 1.78 | 4.33e-02 | 8.55e-02 |
|  | 5 | 32709071 | 32709396 | PDFSTATSENSEMBLUCSC | 326 | 4 | 32719 | island | 0.838755 | 0.951214 | Not queried | 1.78 | 1.14e-01 | 1.68e-01 |
|  | 4 | 155413392 | 155413789 | PDFSTATSENSEMBLUCSC | 398 | 4 | 30644 | tss;island | 0.915108 | 0.972064 | Not queried | 1.78 | 3.43e-01 | 3.84e-01 |
|  | 2 | 55921045 | 55921125 | PDFSTATSENSEMBLUCSC | 81 | 6 | 23364 | tss | 0.002730 | 0.004592 | Not queried | 1.78 | 5.89e-01 | 6.11e-01 |
|  | 8 | 25314954 | 25315063 | PDFSTATSENSEMBLUCSC | 110 | 4 | 39249 | gene;island | 0.009306 | 0.003585 | Not queried | 1.78 | 3.43e-01 | 3.84e-01 |
|  | 14 | 91720173 | 91720696 | PDFSTATSENSEMBLUCSC | 524 | 6 | 13078 | island | 0.890411 | 0.742231 | Not queried | 1.78 | 1.80e-01 | 2.45e-01 |
|  | 20 | 61446962 | 61448325 | PDFSTATSENSEMBLUCSC | 1364 | 42 | 25882 | tss;island | 0.564441 | 0.877416 | OGFR; COL9A3 (margin: OGFR; COL9A3; OGFR-AS1) | 1.77 | 5.73e-06 | 3.56e-04 |
|  | 12 | 27396706 | 27397070 | PDFSTATSENSEMBLUCSC | 365 | 8 | 9619 | tss | 0.223095 | 0.378059 | Not queried | 1.77 | 4.42e-01 | 4.81e-01 |
|  | 16 | 57125654 | 57125836 | PDFSTATSENSEMBLUCSC | 183 | 5 | 15257 | tss;island | 0.261133 | 0.133986 | Not queried | 1.77 | 8.41e-01 | 8.48e-01 |
|  | 12 | 54392734 | 54393725 | PDFSTATSENSEMBLUCSC | 992 | 16 | 9751 | tss;island | 0.880733 | 0.652767 | HOXC5; HOXC6; HOXC9; HOXC-AS1 (margin: HOXC10; HOXC5; HOXC6; MIR196A2; HOXC9; HOXC8; HOXC-AS2; HOXC-AS1) | 1.77 | 6.53e-04 | 7.01e-03 |
|  | 7 | 157567895 | 157568392 | PDFSTATSENSEMBLUCSC | 498 | 6 | 37344 | gene;island | 0.488398 | 0.669187 | Not queried | 1.77 | 9.31e-02 | 1.53e-01 |
|  | 6 | 168476553 | 168476860 | PDFSTATSENSEMBLUCSC | 308 | 7 | 35347 | tss | 0.923110 | 0.976150 | Not queried | 1.77 | 1.28e-01 | 1.87e-01 |
|  | 19 | 58458572 | 58459358 | PDFSTATSENSEMBLUCSC | 787 | 12 | 22488 | island | 0.046628 | 0.010942 | Not queried | 1.77 | 1.45e-02 | 5.34e-02 |
|  | 19 | 58458572 | 58458979 | PDFSTATSENSEMBLUCSC | 408 | 4 | 20255 | gene | 0.082095 | 0.019607 | Not queried | 1.77 | 3.43e-01 | 3.84e-01 |
|  | 15 | 98503714 | 98503952 | PDFSTATSENSEMBLUCSC | 239 | 11 | 14493 | island | 0.052348 | 0.009412 | Not queried | 1.77 | 4.73e-02 | 9.34e-02 |
|  | 17 | 47583654 | 47583920 | PDFSTATSENSEMBLUCSC | 267 | 4 | 16673 | gene;island | 0.938336 | 0.975942 | Not queried | 1.77 | 2.00e-01 | 2.55e-01 |
|  | 7 | 151107257 | 151107400 | PDFSTATSENSEMBLUCSC | 144 | 4 | 38001 | tss;island | 0.822165 | 0.567722 | Not queried | 1.77 | 2.86e-02 | 6.12e-02 |
|  | 6 | 33256078 | 33257141 | PDFSTATSENSEMBLUCSC | 1064 | 30 | 34251 | gene | 0.005604 | 0.013445 | Not queried | 1.77 | 3.32e-02 | 6.79e-02 |
|  | 5 | 174162510 | 174162907 | PDFSTATSENSEMBLUCSC | 398 | 4 | 33417 | island | 0.664152 | 0.349701 | Not queried | 1.77 | 5.71e-02 | 1.00e-01 |
|  | 12 | 56522077 | 56522453 | PDFSTATSENSEMBLUCSC | 377 | 4 | 9173 | gene | 0.004433 | 0.011204 | Not queried | 1.77 | 4.86e-01 | 5.11e-01 |
|  | 22 | 45680498 | 45681036 | PDFSTATSENSEMBLUCSC | 539 | 12 | 27573 | island | 0.762491 | 0.522119 | UPK3A (margin: CTA-268H5.9; UPK3A) | 1.77 | 5.56e-03 | 3.16e-02 |
|  | 3 | 128294970 | 128295141 | PDFSTATSENSEMBLUCSC | 172 | 6 | 28570 | tss | 0.623581 | 0.803028 | Not queried | 1.77 | 3.10e-01 | 3.66e-01 |
|  | 14 | 70345898 | 70346082 | PDFSTATSENSEMBLUCSC | 185 | 5 | 12484 | tss | 0.474127 | 0.749908 | Not queried | 1.77 | 9.52e-02 | 1.53e-01 |
|  | 10 | 134672412 | 134672917 | PDFSTATSENSEMBLUCSC | 506 | 8 | 6314 | island | 0.923513 | 0.969474 | Not queried | 1.77 | 8.30e-02 | 1.40e-01 |
|  | 15 | 52121542 | 52121944 | PDFSTATSENSEMBLUCSC | 403 | 5 | 14144 | island | 0.023445 | 0.074329 | Not queried | 1.77 | 3.10e-01 | 3.66e-01 |
|  | 21 | 40555395 | 40555523 | PDFSTATSENSEMBLUCSC | 129 | 8 | 26682 | island | 0.000514 | 0.005479 | Not queried | 1.77 | 2.34e-01 | 2.90e-01 |
|  | 6 | 15246396 | 15246613 | PDFSTATSENSEMBLUCSC | 218 | 4 | 35466 | island | 0.005509 | 0.005969 | Not queried | 1.77 | 6.86e-01 | 6.99e-01 |
|  | 12 | 48551185 | 48551594 | PDFSTATSENSEMBLUCSC | 410 | 9 | 10365 | island | 0.005765 | 0.021349 | Not queried | 1.77 | 1.13e-01 | 1.68e-01 |
|  | 12 | 48551378 | 48551594 | PDFSTATSENSEMBLUCSC | 217 | 7 | 9669 | tss | 0.005765 | 0.021349 | Not queried | 1.77 | 1.28e-01 | 1.87e-01 |
|  | 11 | 1096283 | 1096455 | PDFSTATSENSEMBLUCSC | 173 | 4 | 6404 | gene;island | 0.823549 | 0.959746 | Not queried | 1.77 | 4.86e-01 | 5.11e-01 |
|  | 8 | 117950244 | 117950504 | PDFSTATSENSEMBLUCSC | 261 | 7 | 40553 | island | 0.497997 | 0.097937 | Not queried | 1.77 | 7.28e-02 | 1.23e-01 |
|  | 3 | 160879441 | 160879721 | PDFSTATSENSEMBLUCSC | 281 | 5 | 29636 | island | 0.926494 | 0.987133 | Not queried | 1.77 | 2.22e-01 | 2.76e-01 |
|  | 8 | 6692785 | 6693125 | PDFSTATSENSEMBLUCSC | 341 | 4 | 39191 | gene | 0.664302 | 0.812576 | Not queried | 1.77 | 2.00e-01 | 2.55e-01 |
|  | 4 | 146403866 | 146403948 | PDFSTATSENSEMBLUCSC | 83 | 5 | 30620 | tss | 0.002715 | 0.002888 | Not queried | 1.77 | 2.22e-01 | 2.76e-01 |
|  | 7 | 50861418 | 50862266 | PDFSTATSENSEMBLUCSC | 849 | 20 | 37598 | tss;island | 0.655393 | 0.929866 | GRB10 (margin: GRB10) | 1.77 | 3.89e-03 | 2.43e-02 |
|  | 19 | 21688519 | 21688905 | PDFSTATSENSEMBLUCSC | 387 | 6 | 19960 | gene | 0.331254 | 0.242825 | Not queried | 1.77 | 3.10e-01 | 3.66e-01 |
|  | 1 | 9086460 | 9086648 | PDFSTATSENSEMBLUCSC | 189 | 4 | 1226 | tss | 0.769609 | 0.938775 | Not queried | 1.77 | 3.43e-01 | 3.84e-01 |
|  | 8 | 140716970 | 140717372 | PDFSTATSENSEMBLUCSC | 403 | 6 | 40606 | island | 0.535499 | 0.296434 | Not queried | 1.77 | 3.94e-01 | 4.36e-01 |
|  | 10 | 129845375 | 129845766 | PDFSTATSENSEMBLUCSC | 392 | 6 | 5438 | tss | 0.088373 | 0.246684 | PTPRE (margin: PTPRE) | 1.77 | 2.16e-03 | 1.45e-02 |
|  | 10 | 129845375 | 129846082 | PDFSTATSENSEMBLUCSC | 708 | 8 | 4821 | gene | 0.089666 | 0.246684 | PTPRE (margin: PTPRE) | 1.77 | 1.04e-02 | 3.96e-02 |
|  | 19 | 38924045 | 38924436 | PDFSTATSENSEMBLUCSC | 392 | 10 | 21976 | island | 0.339987 | 0.317368 | Not queried | 1.77 | 2.80e-01 | 3.40e-01 |
|  | 12 | 132972609 | 132972940 | PDFSTATSENSEMBLUCSC | 332 | 4 | 11048 | island | 0.750911 | 0.881201 | Not queried | 1.76 | 4.86e-01 | 5.11e-01 |
|  | 11 | 2153991 | 2154432 | PDFSTATSENSEMBLUCSC | 442 | 7 | 6445 | gene;island | 0.256416 | 0.737467 | Not queried | 1.76 | 5.30e-02 | 1.00e-01 |
|  | 19 | 36485938 | 36485972 | PDFSTATSENSEMBLUCSC | 35 | 4 | 20719 | tss | 0.006203 | 0.003693 | Not queried | 1.76 | 3.43e-01 | 3.84e-01 |
|  | 16 | 15188232 | 15188395 | PDFSTATSENSEMBLUCSC | 164 | 7 | 15106 | tss;island | 0.011941 | 0.028146 | Not queried | 1.76 | 9.73e-02 | 1.55e-01 |
|  | 2 | 89065319 | 89065605 | PDFSTATSENSEMBLUCSC | 287 | 7 | 24507 | island | 0.712816 | 0.400667 | Not queried | 1.76 | 9.73e-02 | 1.55e-01 |
|  | 14 | 101291068 | 101292392 | PDFSTATSENSEMBLUCSC | 1325 | 22 | 12608 | tss;island | 0.817094 | 0.942019 | MEG3 (margin: MEG3; MIR2392; AL117190.2; RP11-123M6.2) | 1.76 | 1.69e-03 | 1.45e-02 |
|  | 8 | 70983567 | 70983760 | PDFSTATSENSEMBLUCSC | 194 | 4 | 39757 | tss | 0.944628 | 0.982272 | Not queried | 1.76 | 2.86e-02 | 6.12e-02 |
|  | 15 | 96876248 | 96876486 | PDFSTATSENSEMBLUCSC | 239 | 5 | 13494 | gene;tss;island | 0.001837 | 0.007472 | Not queried | 1.76 | 8.41e-01 | 8.48e-01 |
|  | 20 | 61870691 | 61871067 | PDFSTATSENSEMBLUCSC | 377 | 4 | 25498 | gene;island | 0.807788 | 0.933790 | Not queried | 1.76 | 2.86e-02 | 6.12e-02 |
|  | 17 | 19881077 | 19881329 | PDFSTATSENSEMBLUCSC | 253 | 4 | 18107 | island | 0.002622 | 0.009184 | Not queried | 1.76 | 4.86e-01 | 5.11e-01 |
|  | 16 | 82045140 | 82045222 | PDFSTATSENSEMBLUCSC | 83 | 4 | 15383 | tss | 0.807976 | 0.560302 | Not queried | 1.76 | 3.43e-01 | 3.84e-01 |
|  | 22 | 25615317 | 25615557 | PDFSTATSENSEMBLUCSC | 241 | 8 | 27029 | tss | 0.878100 | 0.949882 | Not queried | 1.76 | 1.61e-01 | 2.24e-01 |
|  | 2 | 306606 | 307058 | PDFSTATSENSEMBLUCSC | 453 | 4 | 24001 | island | 0.875850 | 0.943514 | Not queried | 1.76 | 1.14e-01 | 1.68e-01 |
|  | 1 | 43738485 | 43738828 | PDFSTATSENSEMBLUCSC | 344 | 5 | 474 | gene;island | 0.835145 | 0.949415 | Not queried | 1.76 | 9.52e-02 | 1.53e-01 |
|  | 7 | 116139477 | 116139849 | PDFSTATSENSEMBLUCSC | 373 | 4 | 37193 | gene | 0.007362 | 0.015409 | Not queried | 1.76 | 3.43e-01 | 3.84e-01 |
|  | 7 | 97841334 | 97841513 | PDFSTATSENSEMBLUCSC | 180 | 5 | 37743 | tss | 0.641434 | 0.940165 | Not queried | 1.76 | 4.21e-01 | 4.59e-01 |
|  | 4 | 24981801 | 24982173 | PDFSTATSENSEMBLUCSC | 373 | 6 | 30329 | tss | 0.048909 | 0.023065 | Not queried | 1.76 | 4.85e-01 | 5.11e-01 |
|  | 19 | 1438017 | 1438306 | PDFSTATSENSEMBLUCSC | 290 | 10 | 20305 | tss | 0.023556 | 0.129185 | Not queried | 1.76 | 3.53e-01 | 3.95e-01 |
|  | 11 | 43963820 | 43964098 | PDFSTATSENSEMBLUCSC | 279 | 6 | 7258 | tss | 0.258387 | 0.145403 | Not queried | 1.76 | 8.18e-01 | 8.27e-01 |
|  | 8 | 50822168 | 50822565 | PDFSTATSENSEMBLUCSC | 398 | 5 | 40289 | island | 0.880585 | 0.824854 | Not queried | 1.76 | 8.41e-01 | 8.48e-01 |
|  | 19 | 44905782 | 44905980 | PDFSTATSENSEMBLUCSC | 199 | 7 | 20848 | tss | 0.873949 | 0.946644 | Not queried | 1.76 | 3.83e-01 | 4.27e-01 |
|  | 4 | 169240001 | 169240081 | PDFSTATSENSEMBLUCSC | 81 | 4 | 30671 | tss | 0.003092 | 0.012986 | Not queried | 1.76 | 2.00e-01 | 2.55e-01 |
|  | 3 | 150421865 | 150422143 | PDFSTATSENSEMBLUCSC | 279 | 5 | 28662 | tss | 0.025085 | 0.008698 | Not queried | 1.76 | 2.22e-01 | 2.76e-01 |
|  | 12 | 129309117 | 129309376 | PDFSTATSENSEMBLUCSC | 260 | 5 | 10115 | tss;island | 0.008333 | 0.027821 | Not queried | 1.76 | 2.22e-01 | 2.76e-01 |
|  | 8 | 141057285 | 141057460 | PDFSTATSENSEMBLUCSC | 176 | 5 | 39438 | gene;island | 0.971694 | 0.977010 | Not queried | 1.76 | 4.21e-01 | 4.59e-01 |
|  | 7 | 91763850 | 91764605 | PDFSTATSENSEMBLUCSC | 756 | 16 | 37703 | tss | 0.005990 | 0.013030 | Not queried | 1.76 | 1.38e-01 | 1.98e-01 |
|  | 7 | 11871837 | 11872073 | PDFSTATSENSEMBLUCSC | 237 | 7 | 37442 | tss | 0.808892 | 0.852922 | Not queried | 1.76 | 7.10e-01 | 7.20e-01 |
|  | 1 | 2918373 | 2918752 | PDFSTATSENSEMBLUCSC | 380 | 5 | 2525 | island | 0.289722 | 0.362119 | Not queried | 1.76 | 3.10e-01 | 3.66e-01 |
|  | 14 | 23772058 | 23772235 | PDFSTATSENSEMBLUCSC | 178 | 7 | 12300 | tss | 0.051449 | 0.011751 | Not queried | 1.76 | 1.28e-01 | 1.87e-01 |
|  | 4 | 81951951 | 81952099 | PDFSTATSENSEMBLUCSC | 149 | 6 | 30463 | tss;island | 0.881207 | 0.949057 | Not queried | 1.76 | 1.80e-01 | 2.45e-01 |
|  | 1 | 8878173 | 8878227 | PDFSTATSENSEMBLUCSC | 55 | 4 | 1223 | tss;island | 0.002323 | 0.006972 | Not queried | 1.75 | 1.14e-01 | 1.68e-01 |
|  | 19 | 15838756 | 15839006 | PDFSTATSENSEMBLUCSC | 251 | 4 | 21710 | island | 0.215678 | 0.644091 | Not queried | 1.75 | 1.14e-01 | 1.68e-01 |
|  | 1 | 155231766 | 155232379 | PDFSTATSENSEMBLUCSC | 614 | 13 | 3820 | island | 0.009103 | 0.010384 | Not queried | 1.75 | 2.87e-01 | 3.48e-01 |
|  | 1 | 155232227 | 155232379 | PDFSTATSENSEMBLUCSC | 153 | 7 | 1990 | tss | 0.009103 | 0.015756 | Not queried | 1.75 | 6.20e-01 | 6.43e-01 |
|  | 14 | 51562486 | 51562646 | PDFSTATSENSEMBLUCSC | 161 | 5 | 12391 | tss | 0.713053 | 0.453761 | Not queried | 1.75 | 6.90e-01 | 7.01e-01 |
|  | 4 | 166299786 | 166300051 | PDFSTATSENSEMBLUCSC | 266 | 6 | 30670 | tss;island | 0.022630 | 0.011173 | Not queried | 1.75 | 1.00e+00 | 1.00e+00 |
|  | 2 | 74681787 | 74682183 | PDFSTATSENSEMBLUCSC | 397 | 10 | 23455 | tss | 0.007313 | 0.004605 | Not queried | 1.75 | 3.53e-01 | 3.95e-01 |
|  | 2 | 23840537 | 23840763 | PDFSTATSENSEMBLUCSC | 227 | 4 | 22594 | gene | 0.891265 | 0.947608 | Not queried | 1.75 | 4.86e-01 | 5.11e-01 |
|  | 7 | 158220708 | 158221076 | PDFSTATSENSEMBLUCSC | 369 | 5 | 37370 | gene;island | 0.749323 | 0.906287 | Not queried | 1.75 | 3.10e-01 | 3.66e-01 |
|  | 10 | 11783794 | 11784253 | PDFSTATSENSEMBLUCSC | 460 | 8 | 4945 | tss | 0.097348 | 0.353613 | Not queried | 1.75 | 3.28e-01 | 3.84e-01 |
|  | 17 | 42988784 | 42989323 | PDFSTATSENSEMBLUCSC | 540 | 7 | 16621 | gene;island | 0.939897 | 0.547549 | Not queried | 1.75 | 3.18e-01 | 3.74e-01 |
|  | 20 | 33759605 | 33760017 | PDFSTATSENSEMBLUCSC | 413 | 7 | 26120 | island | 0.007290 | 0.053327 | Not queried | 1.75 | 7.28e-02 | 1.23e-01 |
|  | 11 | 67183148 | 67183616 | PDFSTATSENSEMBLUCSC | 469 | 5 | 6753 | gene | 0.595186 | 0.497259 | Not queried | 1.75 | 4.21e-01 | 4.59e-01 |
|  | 6 | 42016671 | 42016713 | PDFSTATSENSEMBLUCSC | 43 | 4 | 34977 | tss;island | 0.006771 | 0.003902 | Not queried | 1.75 | 3.43e-01 | 3.84e-01 |
|  | 3 | 122399230 | 122400006 | PDFSTATSENSEMBLUCSC | 777 | 10 | 29403 | island | 0.006572 | 0.088987 | Not queried | 1.75 | 3.55e-02 | 7.24e-02 |
|  | 8 | 1051510 | 1051814 | PDFSTATSENSEMBLUCSC | 305 | 5 | 40011 | island | 0.306272 | 0.083847 | Not queried | 1.75 | 2.22e-01 | 2.76e-01 |
|  | 20 | 61637360 | 61637856 | PDFSTATSENSEMBLUCSC | 497 | 4 | 25496 | gene;island | 0.925868 | 0.966368 | Not queried | 1.75 | 3.43e-01 | 3.84e-01 |
|  | 11 | 1873757 | 1874093 | PDFSTATSENSEMBLUCSC | 337 | 5 | 7049 | tss | 0.477243 | 0.777183 | Not queried | 1.75 | 4.21e-01 | 4.59e-01 |
|  | 20 | 3065343 | 3065582 | PDFSTATSENSEMBLUCSC | 240 | 7 | 25960 | island | 0.664774 | 0.904728 | Not queried | 1.75 | 9.73e-02 | 1.55e-01 |
|  | 4 | 4867387 | 4867638 | PDFSTATSENSEMBLUCSC | 252 | 4 | 30873 | island | 0.909018 | 0.960319 | Not queried | 1.75 | 4.86e-01 | 5.11e-01 |
|  | 19 | 51670251 | 51670552 | PDFSTATSENSEMBLUCSC | 302 | 5 | 20994 | tss | 0.181582 | 0.536312 | Not queried | 1.75 | 1.51e-01 | 2.11e-01 |
|  | 4 | 109541739 | 109542144 | PDFSTATSENSEMBLUCSC | 406 | 6 | 30088 | gene | 0.002362 | 0.005386 | Not queried | 1.75 | 1.80e-01 | 2.45e-01 |
|  | 1 | 894056 | 894257 | PDFSTATSENSEMBLUCSC | 202 | 4 | 5 | gene;island | 0.020623 | 0.004170 | Not queried | 1.75 | 3.43e-01 | 3.84e-01 |
|  | 6 | 26537736 | 26537980 | PDFSTATSENSEMBLUCSC | 245 | 4 | 34707 | tss;island | 0.004926 | 0.016424 | Not queried | 1.75 | 1.14e-01 | 1.68e-01 |
|  | 7 | 42277807 | 42278096 | PDFSTATSENSEMBLUCSC | 290 | 6 | 37552 | tss;island | 0.957508 | 0.856648 | Not queried | 1.75 | 1.32e-01 | 1.89e-01 |
|  | 19 | 54215458 | 54215577 | PDFSTATSENSEMBLUCSC | 120 | 4 | 21036 | tss | 0.768945 | 0.857909 | Not queried | 1.75 | 3.43e-01 | 3.84e-01 |
|  | 12 | 127212034 | 127212528 | PDFSTATSENSEMBLUCSC | 495 | 4 | 10985 | island | 0.351293 | 0.103566 | Not queried | 1.75 | 1.14e-01 | 1.68e-01 |
|  | 15 | 63334355 | 63334761 | PDFSTATSENSEMBLUCSC | 407 | 8 | 13717 | tss | 0.256022 | 0.522886 | Not queried | 1.75 | 4.42e-01 | 4.81e-01 |
|  | 6 | 168476501 | 168476860 | PDFSTATSENSEMBLUCSC | 360 | 8 | 34559 | gene;island | 0.890863 | 0.969132 | Not queried | 1.75 | 1.30e-01 | 1.89e-01 |
|  | 3 | 77087684 | 77087994 | PDFSTATSENSEMBLUCSC | 311 | 4 | 29291 | island | 0.090623 | 0.021656 | Not queried | 1.75 | 3.43e-01 | 3.84e-01 |
|  | 3 | 37033625 | 37034787 | PDFSTATSENSEMBLUCSC | 1163 | 22 | 27716 | gene | 0.004070 | 0.017113 | MLH1; EPM2AIP1 (margin: MLH1; RP11-129K12.4; RP11-129K12.3; EPM2AIP1) | 1.75 | 9.18e-03 | 3.55e-02 |
|  | 8 | 118532625 | 118533030 | PDFSTATSENSEMBLUCSC | 406 | 10 | 40554 | island | 0.016503 | 0.005951 | Not queried | 1.74 | 7.53e-02 | 1.27e-01 |
|  | 6 | 32805142 | 32806699 | PDFSTATSENSEMBLUCSC | 1558 | 31 | 35928 | island | 0.013101 | 0.037644 | TAP2; PSMB8 (margin: XXbac-BPG246D15.8; PSMB9; TAP2; PSMB8; TAP1) | 1.74 | 9.55e-04 | 9.93e-03 |
|  | 14 | 45553114 | 45553636 | PDFSTATSENSEMBLUCSC | 523 | 5 | 12813 | island | 0.005071 | 0.015567 | Not queried | 1.74 | 3.10e-01 | 3.66e-01 |
|  | 5 | 141302906 | 141303293 | PDFSTATSENSEMBLUCSC | 388 | 9 | 32392 | tss | 0.005866 | 0.014138 | Not queried | 1.74 | 2.22e-01 | 2.76e-01 |
|  | 7 | 140714508 | 140715277 | PDFSTATSENSEMBLUCSC | 770 | 13 | 37943 | tss | 0.019267 | 0.004321 | Not queried | 1.74 | 1.40e-02 | 5.20e-02 |
|  | 19 | 3398706 | 3399015 | PDFSTATSENSEMBLUCSC | 310 | 5 | 19715 | gene;island | 0.648761 | 0.868664 | Not queried | 1.74 | 3.10e-01 | 3.66e-01 |
|  | 1 | 234367145 | 234367586 | PDFSTATSENSEMBLUCSC | 442 | 5 | 1085 | gene;island | 0.694115 | 0.826440 | Not queried | 1.74 | 6.90e-01 | 7.01e-01 |
|  | 11 | 14664598 | 14665070 | PDFSTATSENSEMBLUCSC | 473 | 7 | 6516 | gene;tss;island | 0.699095 | 0.636437 | Not queried | 1.74 | 1.00e+00 | 1.00e+00 |
|  | 12 | 58011764 | 58011875 | PDFSTATSENSEMBLUCSC | 112 | 4 | 10567 | island | 0.576247 | 0.860574 | Not queried | 1.74 | 4.86e-01 | 5.11e-01 |
|  | 10 | 421981 | 422393 | PDFSTATSENSEMBLUCSC | 413 | 5 | 4436 | gene;island | 0.921622 | 0.978282 | Not queried | 1.74 | 2.22e-01 | 2.76e-01 |
|  | 8 | 77585222 | 77585448 | PDFSTATSENSEMBLUCSC | 227 | 4 | 39340 | gene | 0.811727 | 0.330656 | Not queried | 1.74 | 3.43e-01 | 3.84e-01 |
|  | 4 | 186317760 | 186317907 | PDFSTATSENSEMBLUCSC | 148 | 4 | 31474 | island | 0.007773 | 0.006206 | Not queried | 1.74 | 3.43e-01 | 3.84e-01 |
|  | 2 | 175190821 | 175191069 | PDFSTATSENSEMBLUCSC | 249 | 4 | 24871 | island | 0.850681 | 0.936659 | Not queried | 1.74 | 2.00e-01 | 2.55e-01 |
|  | 12 | 120875539 | 120875978 | PDFSTATSENSEMBLUCSC | 440 | 10 | 10890 | island | 0.004731 | 0.011065 | Not queried | 1.74 | 1.23e-01 | 1.81e-01 |
|  | 11 | 118938260 | 118938475 | PDFSTATSENSEMBLUCSC | 216 | 7 | 7747 | tss | 0.005482 | 0.006509 | Not queried | 1.74 | 4.56e-01 | 4.96e-01 |
|  | 21 | 45926600 | 45926753 | PDFSTATSENSEMBLUCSC | 154 | 4 | 26457 | gene;island | 0.925849 | 0.972536 | Not queried | 1.74 | 3.43e-01 | 3.84e-01 |
|  | 1 | 53527412 | 53527693 | PDFSTATSENSEMBLUCSC | 282 | 6 | 1635 | tss;island | 0.835352 | 0.935889 | Not queried | 1.74 | 1.80e-01 | 2.45e-01 |
|  | 6 | 163149167 | 163149453 | PDFSTATSENSEMBLUCSC | 287 | 4 | 34540 | gene;tss;island | 0.058353 | 0.341503 | Not queried | 1.74 | 2.00e-01 | 2.55e-01 |
|  | 4 | 55095148 | 55095261 | PDFSTATSENSEMBLUCSC | 114 | 4 | 30401 | tss | 0.533222 | 0.838394 | Not queried | 1.74 | 2.00e-01 | 2.55e-01 |
|  | 1 | 119543883 | 119544329 | PDFSTATSENSEMBLUCSC | 447 | 5 | 3661 | island | 0.891883 | 0.980407 | Not queried | 1.74 | 9.52e-02 | 1.53e-01 |
|  | 5 | 138628911 | 138629210 | PDFSTATSENSEMBLUCSC | 300 | 8 | 31772 | gene;tss;island | 0.014005 | 0.015924 | Not queried | 1.74 | 5.05e-01 | 5.32e-01 |
|  | 4 | 206112 | 206562 | PDFSTATSENSEMBLUCSC | 451 | 8 | 30714 | island | 0.298576 | 0.617480 | Not queried | 1.74 | 2.34e-01 | 2.90e-01 |
|  | 12 | 132637734 | 132638107 | PDFSTATSENSEMBLUCSC | 374 | 4 | 11025 | island | 0.949357 | 0.982612 | Not queried | 1.74 | 3.43e-01 | 3.84e-01 |
|  | 19 | 5455176 | 5455638 | PDFSTATSENSEMBLUCSC | 463 | 8 | 21404 | island | 0.880365 | 0.980690 | Not queried | 1.74 | 2.79e-01 | 3.38e-01 |
|  | 8 | 11555178 | 11555548 | PDFSTATSENSEMBLUCSC | 371 | 4 | 40084 | island | 0.960025 | 0.970775 | Not queried | 1.74 | 4.86e-01 | 5.11e-01 |
|  | 11 | 20408536 | 20408972 | PDFSTATSENSEMBLUCSC | 437 | 8 | 7197 | tss;island | 0.000787 | 0.002159 | Not queried | 1.74 | 3.82e-01 | 4.27e-01 |
|  | 7 | 141250684 | 141251045 | PDFSTATSENSEMBLUCSC | 362 | 6 | 37944 | tss | 0.034939 | 0.191294 | Not queried | 1.74 | 1.32e-01 | 1.89e-01 |
|  | 19 | 45754942 | 45755260 | PDFSTATSENSEMBLUCSC | 319 | 4 | 20090 | gene | 0.012558 | 0.043342 | Not queried | 1.74 | 3.43e-01 | 3.84e-01 |
|  | 7 | 4901628 | 4901798 | PDFSTATSENSEMBLUCSC | 171 | 4 | 37417 | tss | 0.911271 | 0.967623 | Not queried | 1.74 | 1.14e-01 | 1.68e-01 |
|  | 15 | 78632670 | 78632861 | PDFSTATSENSEMBLUCSC | 192 | 5 | 13438 | gene | 0.817057 | 0.659640 | Not queried | 1.74 | 4.21e-01 | 4.59e-01 |
|  | 15 | 74421739 | 74421935 | PDFSTATSENSEMBLUCSC | 197 | 7 | 13416 | gene | 0.932629 | 0.533969 | Not queried | 1.74 | 1.75e-02 | 5.79e-02 |
|  | 15 | 25425326 | 25425615 | PDFSTATSENSEMBLUCSC | 290 | 4 | 13527 | tss | 0.178627 | 0.326534 | Not queried | 1.74 | 3.43e-01 | 3.84e-01 |
|  | 3 | 56717625 | 56717804 | PDFSTATSENSEMBLUCSC | 180 | 6 | 28406 | tss;island | 0.923132 | 0.584721 | Not queried | 1.73 | 1.80e-01 | 2.45e-01 |
|  | 11 | 116643733 | 116643957 | PDFSTATSENSEMBLUCSC | 225 | 7 | 7710 | tss | 0.001613 | 0.007679 | Not queried | 1.73 | 3.18e-01 | 3.74e-01 |
|  | 10 | 73848817 | 73849167 | PDFSTATSENSEMBLUCSC | 351 | 8 | 5146 | tss | 0.960925 | 0.842020 | Not queried | 1.73 | 4.99e-02 | 9.81e-02 |
|  | 20 | 62461159 | 62461539 | PDFSTATSENSEMBLUCSC | 381 | 4 | 26373 | island | 0.435548 | 0.388484 | Not queried | 1.73 | 6.86e-01 | 6.99e-01 |
|  | 6 | 29716108 | 29716804 | PDFSTATSENSEMBLUCSC | 697 | 21 | 33692 | gene | 0.007354 | 0.015074 | Not queried | 1.73 | 5.21e-02 | 1.00e-01 |
|  | 7 | 26438234 | 26438411 | PDFSTATSENSEMBLUCSC | 178 | 5 | 38332 | island | 0.001211 | 0.004182 | Not queried | 1.73 | 4.21e-01 | 4.59e-01 |
|  | 2 | 106886503 | 106886729 | PDFSTATSENSEMBLUCSC | 227 | 4 | 24591 | island | 0.593524 | 0.256295 | Not queried | 1.73 | 2.00e-01 | 2.55e-01 |
|  | 15 | 93631578 | 93631991 | PDFSTATSENSEMBLUCSC | 414 | 6 | 13491 | gene;island | 0.220184 | 0.053667 | RGMA (margin: RGMA) | 1.73 | 8.66e-03 | 3.36e-02 |
|  | 20 | 48530309 | 48530654 | PDFSTATSENSEMBLUCSC | 346 | 4 | 25449 | gene;tss;island | 0.934359 | 0.907971 | Not queried | 1.73 | 6.86e-01 | 6.99e-01 |
|  | 10 | 73848764 | 73849167 | PDFSTATSENSEMBLUCSC | 404 | 9 | 5836 | island | 0.971743 | 0.852465 | Not queried | 1.73 | 3.15e-02 | 6.50e-02 |
|  | 4 | 987108 | 987672 | PDFSTATSENSEMBLUCSC | 565 | 6 | 29854 | gene;island | 0.914637 | 0.767673 | Not queried | 1.73 | 4.11e-02 | 8.14e-02 |
|  | 17 | 3867794 | 3868216 | PDFSTATSENSEMBLUCSC | 423 | 7 | 17019 | tss;island | 0.019988 | 0.005992 | Not queried | 1.73 | 7.28e-02 | 1.23e-01 |
|  | 12 | 4381435 | 4382425 | PDFSTATSENSEMBLUCSC | 991 | 30 | 9497 | tss;island | 0.585297 | 0.294531 | Not queried | 1.73 | 2.74e-02 | 6.12e-02 |
|  | 1 | 156215418 | 156215727 | PDFSTATSENSEMBLUCSC | 310 | 4 | 818 | gene;island | 0.794204 | 0.931338 | Not queried | 1.73 | 2.00e-01 | 2.55e-01 |
|  | 14 | 21151021 | 21151223 | PDFSTATSENSEMBLUCSC | 203 | 4 | 12275 | tss;island | 0.009534 | 0.002373 | Not queried | 1.73 | 3.43e-01 | 3.84e-01 |
|  | 6 | 90597340 | 90597591 | PDFSTATSENSEMBLUCSC | 252 | 4 | 36326 | island | 0.873255 | 0.974938 | Not queried | 1.73 | 2.00e-01 | 2.55e-01 |
|  | 6 | 75911716 | 75912132 | PDFSTATSENSEMBLUCSC | 417 | 4 | 34393 | gene;island | 0.370485 | 0.683905 | Not queried | 1.73 | 2.86e-02 | 6.12e-02 |
|  | 16 | 3068315 | 3068707 | PDFSTATSENSEMBLUCSC | 393 | 8 | 15044 | tss | 0.906561 | 0.959044 | Not queried | 1.73 | 3.79e-02 | 7.66e-02 |
|  | 18 | 44236847 | 44237151 | PDFSTATSENSEMBLUCSC | 305 | 8 | 19490 | island | 0.597307 | 0.855913 | Not queried | 1.73 | 1.61e-01 | 2.24e-01 |
|  | 18 | 44237002 | 44237151 | PDFSTATSENSEMBLUCSC | 150 | 6 | 19258 | tss | 0.597307 | 0.799539 | Not queried | 1.73 | 2.40e-01 | 2.95e-01 |
|  | 7 | 148936572 | 148936732 | PDFSTATSENSEMBLUCSC | 161 | 6 | 37966 | tss | 0.010118 | 0.026354 | Not queried | 1.73 | 1.80e-01 | 2.45e-01 |
|  | 16 | 70680320 | 70680406 | PDFSTATSENSEMBLUCSC | 87 | 4 | 15342 | tss | 0.810401 | 0.928377 | Not queried | 1.73 | 3.43e-01 | 3.84e-01 |
|  | 17 | 11501503 | 11501663 | PDFSTATSENSEMBLUCSC | 161 | 8 | 17137 | tss;island | 0.492326 | 0.751384 | Not queried | 1.73 | 1.05e-01 | 1.66e-01 |
|  | 10 | 7709639 | 7709962 | PDFSTATSENSEMBLUCSC | 324 | 4 | 4939 | tss | 0.850633 | 0.952333 | Not queried | 1.73 | 6.86e-01 | 6.99e-01 |
|  | 7 | 116164422 | 116164836 | PDFSTATSENSEMBLUCSC | 415 | 11 | 37850 | tss | 0.004297 | 0.015748 | Not queried | 1.73 | 7.59e-02 | 1.28e-01 |
|  | 5 | 180633615 | 180633891 | PDFSTATSENSEMBLUCSC | 277 | 4 | 33520 | island | 0.012781 | 0.034116 | Not queried | 1.73 | 2.00e-01 | 2.55e-01 |
|  | 5 | 33936171 | 33936462 | PDFSTATSENSEMBLUCSC | 292 | 10 | 32010 | tss | 0.855788 | 0.601518 | Not queried | 1.73 | 3.55e-02 | 7.24e-02 |
|  | 6 | 32005786 | 32006079 | PDFSTATSENSEMBLUCSC | 294 | 4 | 34860 | tss | 0.467811 | 0.792976 | Not queried | 1.73 | 2.00e-01 | 2.55e-01 |
|  | 1 | 118727658 | 118728226 | PDFSTATSENSEMBLUCSC | 569 | 10 | 3655 | island | 0.186722 | 0.348446 | Not queried | 1.73 | 1.05e-01 | 1.66e-01 |
|  | 12 | 16759509 | 16759793 | PDFSTATSENSEMBLUCSC | 285 | 9 | 9596 | tss | 0.124676 | 0.025867 | Not queried | 1.73 | 4.36e-01 | 4.76e-01 |
|  | 17 | 78388520 | 78388593 | PDFSTATSENSEMBLUCSC | 74 | 4 | 16867 | gene;tss;island | 0.086141 | 0.043899 | Not queried | 1.73 | 2.86e-02 | 6.12e-02 |
|  | 17 | 78157897 | 78158248 | PDFSTATSENSEMBLUCSC | 352 | 4 | 16858 | gene;island | 0.867704 | 0.959341 | Not queried | 1.73 | 3.43e-01 | 3.84e-01 |
|  | 2 | 73340007 | 73340489 | PDFSTATSENSEMBLUCSC | 483 | 5 | 24410 | island | 0.002360 | 0.007758 | Not queried | 1.73 | 2.22e-01 | 2.76e-01 |
|  | 5 | 176449842 | 176450179 | PDFSTATSENSEMBLUCSC | 338 | 4 | 31914 | gene | 0.002208 | 0.006697 | Not queried | 1.72 | 5.71e-02 | 1.00e-01 |
|  | 1 | 200992283 | 200992788 | PDFSTATSENSEMBLUCSC | 506 | 7 | 937 | gene | 0.017021 | 0.013150 | Not queried | 1.72 | 1.00e+00 | 1.00e+00 |
|  | 11 | 112831501 | 112832249 | PDFSTATSENSEMBLUCSC | 749 | 12 | 8813 | island | 0.255839 | 0.071461 | Not queried | 1.72 | 8.87e-02 | 1.49e-01 |
|  | 8 | 42010142 | 42010494 | PDFSTATSENSEMBLUCSC | 353 | 9 | 40255 | island | 0.034296 | 0.008144 | Not queried | 1.72 | 1.13e-01 | 1.68e-01 |
|  | 10 | 46090406 | 46090807 | PDFSTATSENSEMBLUCSC | 402 | 7 | 5060 | tss | 0.007193 | 0.004691 | Not queried | 1.72 | 6.20e-01 | 6.43e-01 |
|  | 6 | 86158972 | 86159426 | PDFSTATSENSEMBLUCSC | 455 | 7 | 36300 | island | 0.963464 | 0.986926 | Not queried | 1.72 | 1.65e-01 | 2.28e-01 |
|  | 1 | 55317098 | 55317306 | PDFSTATSENSEMBLUCSC | 209 | 4 | 550 | gene | 0.909899 | 0.971943 | Not queried | 1.72 | 2.86e-02 | 6.12e-02 |
|  | 7 | 111202389 | 111202457 | PDFSTATSENSEMBLUCSC | 69 | 4 | 37838 | tss | 0.008107 | 0.020734 | Not queried | 1.72 | 6.86e-01 | 6.99e-01 |
|  | 3 | 126398077 | 126398357 | PDFSTATSENSEMBLUCSC | 281 | 4 | 29436 | island | 0.150506 | 0.034424 | Not queried | 1.72 | 4.86e-01 | 5.11e-01 |
|  | 2 | 60812505 | 60812753 | PDFSTATSENSEMBLUCSC | 249 | 4 | 24322 | island | 0.903655 | 0.965371 | Not queried | 1.72 | 1.14e-01 | 1.68e-01 |
|  | 1 | 32410413 | 32410678 | PDFSTATSENSEMBLUCSC | 266 | 4 | 2979 | island | 0.781338 | 0.952781 | Not queried | 1.72 | 3.43e-01 | 3.84e-01 |
|  | 15 | 41228521 | 41228736 | PDFSTATSENSEMBLUCSC | 216 | 5 | 13300 | gene;island | 0.917785 | 0.965277 | Not queried | 1.72 | 1.51e-01 | 2.11e-01 |
|  | 1 | 28974730 | 28975001 | PDFSTATSENSEMBLUCSC | 272 | 4 | 1434 | tss | 0.000804 | 0.003561 | Not queried | 1.72 | 2.00e-01 | 2.55e-01 |
|  | 2 | 232651086 | 232651190 | PDFSTATSENSEMBLUCSC | 105 | 7 | 25188 | island | 0.001691 | 0.007657 | Not queried | 1.72 | 1.65e-01 | 2.28e-01 |
|  | 19 | 36359211 | 36359556 | PDFSTATSENSEMBLUCSC | 346 | 8 | 21922 | island | 0.052215 | 0.012986 | Not queried | 1.72 | 2.34e-01 | 2.90e-01 |
|  | 19 | 36359211 | 36359327 | PDFSTATSENSEMBLUCSC | 117 | 6 | 20717 | tss | 0.086377 | 0.024084 | Not queried | 1.72 | 3.10e-01 | 3.66e-01 |
|  | 14 | 101349632 | 101350094 | PDFSTATSENSEMBLUCSC | 463 | 4 | 12213 | gene;tss;island | 0.914296 | 0.969389 | Not queried | 1.72 | 5.71e-02 | 1.00e-01 |
|  | 15 | 69744390 | 69745373 | PDFSTATSENSEMBLUCSC | 984 | 14 | 14266 | island | 0.189224 | 0.309392 | Not queried | 1.72 | 1.94e-01 | 2.55e-01 |
|  | 8 | 92082016 | 92082417 | PDFSTATSENSEMBLUCSC | 402 | 7 | 39808 | tss | 0.002179 | 0.007254 | Not queried | 1.72 | 3.83e-01 | 4.27e-01 |
|  | 10 | 118429481 | 118429952 | PDFSTATSENSEMBLUCSC | 472 | 7 | 5386 | tss | 0.642477 | 0.445147 | Not queried | 1.72 | 2.09e-01 | 2.65e-01 |
|  | 4 | 186391758 | 186391968 | PDFSTATSENSEMBLUCSC | 211 | 4 | 30209 | gene;island | 0.018726 | 0.016380 | Not queried | 1.72 | 1.00e+00 | 1.00e+00 |
|  | 2 | 233351525 | 233351921 | PDFSTATSENSEMBLUCSC | 397 | 5 | 23064 | gene;island | 0.801547 | 0.981549 | Not queried | 1.72 | 9.52e-02 | 1.53e-01 |
|  | 5 | 110408740 | 110409196 | PDFSTATSENSEMBLUCSC | 457 | 8 | 31708 | gene;island | 0.125901 | 0.040800 | Not queried | 1.72 | 1.61e-01 | 2.24e-01 |
|  | 11 | 71954982 | 71955599 | PDFSTATSENSEMBLUCSC | 618 | 11 | 8629 | island | 0.967640 | 0.975914 | Not queried | 1.72 | 1.93e-01 | 2.55e-01 |
|  | 11 | 2292361 | 2293309 | PDFSTATSENSEMBLUCSC | 949 | 32 | 7064 | tss;island | 0.760247 | 0.505816 | ASCL2 (margin: ASCL2) | 1.72 | 1.90e-09 | 6.85e-07 |
|  | 1 | 161736000 | 161736072 | PDFSTATSENSEMBLUCSC | 73 | 5 | 2055 | tss | 0.000523 | 0.001927 | Not queried | 1.72 | 9.52e-02 | 1.53e-01 |
|  | 3 | 49203799 | 49203975 | PDFSTATSENSEMBLUCSC | 177 | 5 | 28322 | tss | 0.141217 | 0.047894 | Not queried | 1.72 | 6.90e-01 | 7.01e-01 |
|  | 13 | 20797356 | 20797693 | PDFSTATSENSEMBLUCSC | 338 | 5 | 11095 | gene | 0.946947 | 0.818962 | Not queried | 1.72 | 1.51e-01 | 2.11e-01 |
|  | 5 | 140514811 | 140515207 | PDFSTATSENSEMBLUCSC | 397 | 5 | 31808 | gene | 0.647923 | 0.947791 | Not queried | 1.72 | 3.17e-02 | 6.50e-02 |
|  | 2 | 86564885 | 86565390 | PDFSTATSENSEMBLUCSC | 506 | 9 | 23493 | tss;island | 0.082388 | 0.026821 | Not queried | 1.72 | 2.58e-01 | 3.16e-01 |
|  | 13 | 51417686 | 51418221 | PDFSTATSENSEMBLUCSC | 536 | 10 | 11745 | island | 0.865364 | 0.976597 | Not queried | 1.72 | 1.05e-01 | 1.66e-01 |
|  | 13 | 51417923 | 51418221 | PDFSTATSENSEMBLUCSC | 299 | 8 | 11448 | tss | 0.906035 | 0.979972 | Not queried | 1.72 | 1.30e-01 | 1.89e-01 |
|  | 18 | 61089798 | 61090076 | PDFSTATSENSEMBLUCSC | 279 | 6 | 19311 | tss | 0.002489 | 0.009406 | Not queried | 1.72 | 2.40e-01 | 2.95e-01 |
|  | 15 | 75493819 | 75494077 | PDFSTATSENSEMBLUCSC | 259 | 7 | 13809 | tss;island | 0.001977 | 0.005796 | Not queried | 1.72 | 3.83e-01 | 4.27e-01 |
|  | 7 | 91763737 | 91764605 | PDFSTATSENSEMBLUCSC | 869 | 17 | 38654 | island | 0.006769 | 0.015776 | Not queried | 1.72 | 1.22e-01 | 1.80e-01 |
|  | 17 | 6947032 | 6947464 | PDFSTATSENSEMBLUCSC | 433 | 10 | 17918 | island | 0.842418 | 0.915213 | SLC16A11 (margin: SLC16A13; SLC16A11) | 1.72 | 3.89e-03 | 2.43e-02 |
|  | 22 | 40766469 | 40766549 | PDFSTATSENSEMBLUCSC | 81 | 6 | 27162 | tss | 0.024769 | 0.018498 | Not queried | 1.72 | 4.85e-01 | 5.11e-01 |
|  | 14 | 91283255 | 91283606 | PDFSTATSENSEMBLUCSC | 352 | 4 | 12556 | tss;island | 0.302992 | 0.559634 | Not queried | 1.72 | 2.00e-01 | 2.55e-01 |
|  | 1 | 100816943 | 100817041 | PDFSTATSENSEMBLUCSC | 99 | 4 | 1779 | tss;island | 0.010831 | 0.002929 | Not queried | 1.72 | 2.00e-01 | 2.55e-01 |
|  | 2 | 131862302 | 131862633 | PDFSTATSENSEMBLUCSC | 332 | 6 | 24728 | island | 0.003318 | 0.004235 | Not queried | 1.72 | 3.10e-01 | 3.66e-01 |
|  | 1 | 36915704 | 36916039 | PDFSTATSENSEMBLUCSC | 336 | 6 | 430 | gene | 0.023183 | 0.110684 | Not queried | 1.71 | 2.40e-01 | 2.95e-01 |
|  | 5 | 2752139 | 2752278 | PDFSTATSENSEMBLUCSC | 140 | 4 | 31976 | tss;island | 0.319510 | 0.086149 | Not queried | 1.71 | 1.14e-01 | 1.68e-01 |
|  | 19 | 51231557 | 51232007 | PDFSTATSENSEMBLUCSC | 451 | 5 | 22322 | island | 0.929759 | 0.968777 | Not queried | 1.71 | 1.59e-02 | 5.36e-02 |
|  | 13 | 33002010 | 33002820 | PDFSTATSENSEMBLUCSC | 811 | 14 | 11659 | island | 0.122541 | 0.050613 | Not queried | 1.71 | 2.10e-01 | 2.66e-01 |
|  | 16 | 202311 | 202542 | PDFSTATSENSEMBLUCSC | 232 | 4 | 14961 | tss;island | 0.530164 | 0.859921 | Not queried | 1.71 | 2.00e-01 | 2.55e-01 |
|  | 12 | 132855060 | 132855381 | PDFSTATSENSEMBLUCSC | 322 | 4 | 9441 | gene;island | 0.578929 | 0.797489 | Not queried | 1.71 | 2.00e-01 | 2.55e-01 |
|  | 7 | 45927735 | 45927947 | PDFSTATSENSEMBLUCSC | 213 | 5 | 37586 | tss | 0.648548 | 0.905155 | Not queried | 1.71 | 5.56e-02 | 1.00e-01 |
|  | 19 | 11529133 | 11530065 | PDFSTATSENSEMBLUCSC | 933 | 11 | 21568 | island | 0.861221 | 0.893234 | Not queried | 1.71 | 3.32e-01 | 3.84e-01 |
|  | 1 | 205038785 | 205039083 | PDFSTATSENSEMBLUCSC | 299 | 4 | 963 | gene;island | 0.835517 | 0.940861 | Not queried | 1.71 | 3.43e-01 | 3.84e-01 |
|  | 19 | 34012884 | 34013247 | PDFSTATSENSEMBLUCSC | 364 | 7 | 20685 | tss | 0.029907 | 0.029297 | Not queried | 1.71 | 6.20e-01 | 6.43e-01 |
|  | 11 | 124631981 | 124632552 | PDFSTATSENSEMBLUCSC | 572 | 6 | 8915 | island | 0.065482 | 0.020359 | Not queried | 1.71 | 3.94e-01 | 4.36e-01 |
|  | 18 | 76160108 | 76160443 | PDFSTATSENSEMBLUCSC | 336 | 5 | 19590 | island | 0.129330 | 0.076511 | Not queried | 1.71 | 5.56e-02 | 1.00e-01 |
|  | 13 | 36871646 | 36872346 | PDFSTATSENSEMBLUCSC | 701 | 14 | 11668 | island | 0.557836 | 0.670678 | SOHLH2; CCDC169-SOHLH2; CCDC169 (margin: SOHLH2; CCDC169-SOHLH2; CCDC169; SPG20) | 1.71 | 5.76e-03 | 3.19e-02 |
|  | 6 | 34856704 | 34857281 | PDFSTATSENSEMBLUCSC | 578 | 7 | 34925 | tss | 0.004288 | 0.008005 | Not queried | 1.71 | 3.83e-01 | 4.27e-01 |
|  | 6 | 34856704 | 34857663 | PDFSTATSENSEMBLUCSC | 960 | 9 | 36068 | island | 0.004644 | 0.008005 | Not queried | 1.71 | 3.87e-01 | 4.30e-01 |
|  | 6 | 33135269 | 33135453 | PDFSTATSENSEMBLUCSC | 185 | 4 | 34197 | gene | 0.544965 | 0.792137 | Not queried | 1.71 | 1.14e-01 | 1.68e-01 |
|  | 8 | 21899862 | 21900337 | PDFSTATSENSEMBLUCSC | 476 | 6 | 39579 | tss | 0.342245 | 0.572138 | Not queried | 1.71 | 6.49e-02 | 1.11e-01 |
|  | 10 | 49482695 | 49482871 | PDFSTATSENSEMBLUCSC | 177 | 5 | 4578 | gene | 0.757383 | 0.965259 | Not queried | 1.71 | 2.22e-01 | 2.76e-01 |
|  | 20 | 29611652 | 29611810 | PDFSTATSENSEMBLUCSC | 159 | 5 | 25656 | tss | 0.571479 | 0.289562 | Not queried | 1.71 | 1.59e-02 | 5.36e-02 |
|  | 12 | 82153109 | 82153464 | PDFSTATSENSEMBLUCSC | 356 | 7 | 9895 | tss | 0.602722 | 0.853483 | Not queried | 1.71 | 8.05e-01 | 8.14e-01 |
|  | 10 | 7708934 | 7709073 | PDFSTATSENSEMBLUCSC | 140 | 4 | 4938 | tss | 0.045599 | 0.137261 | Not queried | 1.71 | 2.00e-01 | 2.55e-01 |
|  | 19 | 17970314 | 17970713 | PDFSTATSENSEMBLUCSC | 400 | 10 | 20603 | tss | 0.002803 | 0.006331 | Not queried | 1.71 | 1.05e-01 | 1.66e-01 |
|  | 17 | 5402883 | 5403516 | PDFSTATSENSEMBLUCSC | 634 | 6 | 16368 | gene;island | 0.941190 | 0.836688 | Not queried | 1.71 | 2.40e-01 | 2.95e-01 |
|  | 10 | 652260 | 652597 | PDFSTATSENSEMBLUCSC | 338 | 4 | 4445 | gene;island | 0.772964 | 0.974721 | Not queried | 1.71 | 3.43e-01 | 3.84e-01 |
|  | 6 | 31513571 | 31513886 | PDFSTATSENSEMBLUCSC | 316 | 4 | 33897 | gene;tss;island | 0.573093 | 0.812908 | Not queried | 1.71 | 4.86e-01 | 5.11e-01 |
|  | 10 | 38739819 | 38740029 | PDFSTATSENSEMBLUCSC | 211 | 4 | 4562 | gene;island | 0.871653 | 0.953207 | Not queried | 1.71 | 4.86e-01 | 5.11e-01 |
|  | 18 | 10525737 | 10525881 | PDFSTATSENSEMBLUCSC | 145 | 6 | 19391 | island | 0.003705 | 0.009373 | Not queried | 1.71 | 5.89e-01 | 6.11e-01 |
|  | 1 | 3634811 | 3635210 | PDFSTATSENSEMBLUCSC | 400 | 6 | 142 | gene;island | 0.882698 | 0.965819 | TP73 (margin: TP73; RP5-1092A11.2) | 1.71 | 8.66e-03 | 3.36e-02 |
|  | 17 | 73083632 | 73083887 | PDFSTATSENSEMBLUCSC | 256 | 6 | 17642 | tss;island | 0.011459 | 0.044304 | Not queried | 1.71 | 2.40e-01 | 2.95e-01 |
|  | 3 | 157821919 | 157822072 | PDFSTATSENSEMBLUCSC | 154 | 4 | 28011 | gene;island | 0.956986 | 0.982605 | Not queried | 1.71 | 5.71e-02 | 1.00e-01 |
|  | 5 | 167719478 | 167719742 | PDFSTATSENSEMBLUCSC | 265 | 4 | 31884 | gene;island | 0.010876 | 0.033600 | Not queried | 1.71 | 1.14e-01 | 1.68e-01 |
|  | 6 | 30655197 | 30656094 | PDFSTATSENSEMBLUCSC | 898 | 15 | 34780 | tss;island | 0.023201 | 0.127346 | Not queried | 1.71 | 5.12e-01 | 5.39e-01 |
|  | 5 | 122434853 | 122435202 | PDFSTATSENSEMBLUCSC | 350 | 4 | 31732 | gene;island | 0.327085 | 0.626442 | Not queried | 1.71 | 2.86e-02 | 6.12e-02 |
|  | 2 | 85132797 | 85133262 | PDFSTATSENSEMBLUCSC | 466 | 4 | 22760 | gene | 0.003509 | 0.013925 | Not queried | 1.71 | 3.43e-01 | 3.84e-01 |
|  | 1 | 88928168 | 88928839 | PDFSTATSENSEMBLUCSC | 672 | 6 | 3443 | island | 0.553855 | 0.385840 | Not queried | 1.71 | 5.89e-01 | 6.11e-01 |
|  | 1 | 34098656 | 34099118 | PDFSTATSENSEMBLUCSC | 463 | 4 | 406 | gene;island | 0.052027 | 0.016081 | Not queried | 1.71 | 6.86e-01 | 6.99e-01 |
|  | 6 | 33147086 | 33147259 | PDFSTATSENSEMBLUCSC | 174 | 4 | 34207 | gene | 0.471033 | 0.820645 | Not queried | 1.71 | 1.14e-01 | 1.68e-01 |
|  | 11 | 45868398 | 45868501 | PDFSTATSENSEMBLUCSC | 104 | 4 | 7268 | tss;island | 0.950442 | 0.912326 | Not queried | 1.70 | 6.86e-01 | 6.99e-01 |
|  | 22 | 45073031 | 45073165 | PDFSTATSENSEMBLUCSC | 135 | 5 | 26893 | gene | 0.856474 | 0.955941 | Not queried | 1.70 | 3.10e-01 | 3.66e-01 |
|  | 13 | 38443634 | 38444227 | PDFSTATSENSEMBLUCSC | 594 | 9 | 11678 | island | 0.689535 | 0.435036 | Not queried | 1.70 | 7.70e-02 | 1.30e-01 |
|  | 19 | 36485938 | 36486149 | PDFSTATSENSEMBLUCSC | 212 | 6 | 21930 | island | 0.005449 | 0.003655 | Not queried | 1.70 | 3.94e-01 | 4.36e-01 |
|  | 1 | 2058230 | 2059086 | PDFSTATSENSEMBLUCSC | 857 | 6 | 62 | gene | 0.918789 | 0.969282 | PRKCZ (margin: PRKCZ) | 1.70 | 8.66e-03 | 3.36e-02 |
|  | 16 | 66400320 | 66400411 | PDFSTATSENSEMBLUCSC | 92 | 4 | 15279 | tss | 0.869705 | 0.953961 | Not queried | 1.70 | 1.14e-01 | 1.68e-01 |
|  | 12 | 64615825 | 64616026 | PDFSTATSENSEMBLUCSC | 202 | 4 | 9202 | gene | 0.005485 | 0.015612 | Not queried | 1.70 | 3.43e-01 | 3.84e-01 |
|  | 12 | 120524653 | 120525338 | PDFSTATSENSEMBLUCSC | 686 | 6 | 9344 | gene;island | 0.008826 | 0.028866 | Not queried | 1.70 | 4.85e-01 | 5.11e-01 |
|  | 2 | 99771423 | 99771627 | PDFSTATSENSEMBLUCSC | 205 | 4 | 22806 | gene | 0.001739 | 0.005035 | Not queried | 1.70 | 6.86e-01 | 6.99e-01 |
|  | 22 | 50608033 | 50608598 | PDFSTATSENSEMBLUCSC | 566 | 6 | 27248 | tss;island | 0.452806 | 0.100915 | Not queried | 1.70 | 5.89e-01 | 6.11e-01 |
|  | 1 | 1103225 | 1103312 | PDFSTATSENSEMBLUCSC | 88 | 4 | 1140 | tss;island | 0.839631 | 0.958243 | Not queried | 1.70 | 5.71e-02 | 1.00e-01 |
|  | 6 | 74405564 | 74406189 | PDFSTATSENSEMBLUCSC | 626 | 6 | 34391 | gene | 0.005465 | 0.012299 | Not queried | 1.70 | 3.10e-01 | 3.66e-01 |
|  | 16 | 88717374 | 88717989 | PDFSTATSENSEMBLUCSC | 616 | 13 | 16212 | island | 0.617213 | 0.239391 | CYBA; MVD (margin: IL17C; SNAI3-AS1; CYBA; MVD) | 1.70 | 2.87e-03 | 1.85e-02 |
|  | 22 | 25159879 | 25160098 | PDFSTATSENSEMBLUCSC | 220 | 4 | 26817 | gene;tss;island | 0.812436 | 0.967302 | Not queried | 1.70 | 3.43e-01 | 3.84e-01 |
|  | 5 | 135170000 | 135170456 | PDFSTATSENSEMBLUCSC | 457 | 8 | 33140 | island | 0.322032 | 0.078838 | Not queried | 1.70 | 1.30e-01 | 1.89e-01 |
|  | 6 | 17988404 | 17988960 | PDFSTATSENSEMBLUCSC | 557 | 9 | 34658 | tss;island | 0.025495 | 0.088219 | Not queried | 1.70 | 5.46e-01 | 5.70e-01 |
|  | 12 | 119212160 | 119212319 | PDFSTATSENSEMBLUCSC | 160 | 4 | 10871 | island | 0.639505 | 0.418666 | Not queried | 1.70 | 3.43e-01 | 3.84e-01 |
|  | 5 | 121187445 | 121187598 | PDFSTATSENSEMBLUCSC | 154 | 5 | 32241 | tss;island | 0.793646 | 0.912312 | Not queried | 1.70 | 1.51e-01 | 2.11e-01 |
|  | 6 | 25726884 | 25727291 | PDFSTATSENSEMBLUCSC | 408 | 9 | 34675 | tss;island | 0.837687 | 0.974419 | Not queried | 1.70 | 6.25e-02 | 1.09e-01 |
|  | 19 | 18699118 | 18699631 | PDFSTATSENSEMBLUCSC | 514 | 11 | 21785 | island | 0.205161 | 0.105332 | Not queried | 1.70 | 4.78e-01 | 5.11e-01 |
|  | 6 | 33265886 | 33266089 | PDFSTATSENSEMBLUCSC | 204 | 6 | 34257 | gene;island | 0.005770 | 0.019790 | Not queried | 1.70 | 1.80e-01 | 2.45e-01 |
|  | 3 | 125239063 | 125239413 | PDFSTATSENSEMBLUCSC | 351 | 8 | 28552 | tss | 0.011761 | 0.007857 | Not queried | 1.70 | 1.95e-01 | 2.55e-01 |
|  | 3 | 129147230 | 129147846 | PDFSTATSENSEMBLUCSC | 617 | 9 | 29469 | island | 0.041376 | 0.017441 | Not queried | 1.70 | 2.22e-01 | 2.76e-01 |
|  | 17 | 39680217 | 39680662 | PDFSTATSENSEMBLUCSC | 446 | 4 | 16570 | gene;island | 0.773204 | 0.487306 | Not queried | 1.70 | 2.86e-02 | 6.12e-02 |
|  | 19 | 58838208 | 58838697 | PDFSTATSENSEMBLUCSC | 490 | 9 | 22503 | island | 0.002610 | 0.009764 | Not queried | 1.70 | 5.03e-02 | 9.87e-02 |
|  | 12 | 110841169 | 110841516 | PDFSTATSENSEMBLUCSC | 348 | 4 | 9300 | gene | 0.003428 | 0.009125 | Not queried | 1.70 | 2.00e-01 | 2.55e-01 |
|  | 19 | 39805850 | 39806102 | PDFSTATSENSEMBLUCSC | 253 | 4 | 21991 | island | 0.827918 | 0.964766 | Not queried | 1.70 | 3.43e-01 | 3.84e-01 |
|  | 18 | 43684326 | 43684385 | PDFSTATSENSEMBLUCSC | 60 | 4 | 19101 | gene | 0.004776 | 0.015450 | Not queried | 1.70 | 6.86e-01 | 6.99e-01 |
|  | 11 | 111944731 | 111944894 | PDFSTATSENSEMBLUCSC | 164 | 5 | 6904 | gene | 0.001656 | 0.003426 | Not queried | 1.70 | 3.10e-01 | 3.66e-01 |
|  | 7 | 92861488 | 92861612 | PDFSTATSENSEMBLUCSC | 125 | 7 | 37712 | tss | 0.006171 | 0.013989 | Not queried | 1.70 | 3.79e-02 | 7.66e-02 |
|  | 5 | 158689930 | 158690246 | PDFSTATSENSEMBLUCSC | 317 | 6 | 33356 | island | 0.002348 | 0.005109 | Not queried | 1.70 | 3.10e-01 | 3.66e-01 |
|  | 1 | 43282382 | 43282720 | PDFSTATSENSEMBLUCSC | 339 | 8 | 1553 | tss | 0.017952 | 0.005646 | Not queried | 1.69 | 2.34e-01 | 2.90e-01 |
|  | 7 | 14030127 | 14030482 | PDFSTATSENSEMBLUCSC | 356 | 5 | 36900 | gene;tss;island | 0.010044 | 0.026710 | ETV1 (margin: ETV1) | 1.69 | 7.94e-03 | 3.19e-02 |
|  | 15 | 55880894 | 55881246 | PDFSTATSENSEMBLUCSC | 353 | 9 | 14160 | island | 0.578985 | 0.877919 | Not queried | 1.69 | 1.88e-02 | 6.12e-02 |
|  | 18 | 44676656 | 44677236 | PDFSTATSENSEMBLUCSC | 581 | 10 | 19496 | island | 0.001741 | 0.003197 | Not queried | 1.69 | 2.32e-02 | 6.12e-02 |
|  | 18 | 44676959 | 44677236 | PDFSTATSENSEMBLUCSC | 278 | 6 | 19263 | tss | 0.001713 | 0.005520 | Not queried | 1.69 | 3.10e-01 | 3.66e-01 |
|  | 10 | 133937893 | 133938266 | PDFSTATSENSEMBLUCSC | 374 | 5 | 4848 | gene;island | 0.955898 | 0.985940 | Not queried | 1.69 | 3.10e-01 | 3.66e-01 |
|  | 4 | 141294522 | 141294594 | PDFSTATSENSEMBLUCSC | 73 | 5 | 30605 | tss | 0.015921 | 0.008994 | Not queried | 1.69 | 1.51e-01 | 2.11e-01 |
|  | 3 | 147109426 | 147109784 | PDFSTATSENSEMBLUCSC | 359 | 4 | 27985 | gene;island | 0.612212 | 0.752462 | Not queried | 1.69 | 6.86e-01 | 6.99e-01 |
|  | 19 | 41870097 | 41870343 | PDFSTATSENSEMBLUCSC | 247 | 8 | 20808 | tss | 0.001808 | 0.005922 | Not queried | 1.69 | 6.50e-02 | 1.11e-01 |
|  | 2 | 61371880 | 61372226 | PDFSTATSENSEMBLUCSC | 347 | 7 | 23377 | tss | 0.413036 | 0.670610 | KIAA1841; C2orf74; AC016747.3 (margin: KIAA1841; C2orf74; AC016747.3; RP11-493E12.1) | 1.69 | 1.17e-03 | 1.13e-02 |
|  | 1 | 6188946 | 6189304 | PDFSTATSENSEMBLUCSC | 359 | 5 | 161 | gene;island | 0.870164 | 0.958844 | Not queried | 1.69 | 2.22e-01 | 2.76e-01 |
|  | 11 | 7041752 | 7041987 | PDFSTATSENSEMBLUCSC | 236 | 6 | 6495 | gene | 0.633432 | 0.774921 | Not queried | 1.69 | 3.10e-01 | 3.66e-01 |
|  | 7 | 77669697 | 77670191 | PDFSTATSENSEMBLUCSC | 495 | 5 | 37064 | gene;island | 0.353804 | 0.638775 | Not queried | 1.69 | 5.48e-01 | 5.70e-01 |
|  | 3 | 20227789 | 20227940 | PDFSTATSENSEMBLUCSC | 152 | 6 | 28181 | tss | 0.006240 | 0.018571 | Not queried | 1.69 | 3.10e-01 | 3.66e-01 |
|  | 5 | 9546335 | 9546404 | PDFSTATSENSEMBLUCSC | 70 | 4 | 31985 | tss;island | 0.094481 | 0.025826 | Not queried | 1.69 | 2.00e-01 | 2.55e-01 |
|  | 1 | 156721823 | 156722410 | PDFSTATSENSEMBLUCSC | 588 | 12 | 2023 | tss;island | 0.003930 | 0.009418 | Not queried | 1.69 | 2.19e-01 | 2.76e-01 |
|  | 6 | 169018751 | 169018877 | PDFSTATSENSEMBLUCSC | 127 | 4 | 34564 | gene | 0.860643 | 0.915756 | Not queried | 1.69 | 2.86e-02 | 6.12e-02 |
|  | 6 | 26156234 | 26156990 | PDFSTATSENSEMBLUCSC | 757 | 12 | 35518 | island | 0.005972 | 0.012273 | Not queried | 1.69 | 1.98e-01 | 2.55e-01 |
|  | 1 | 221052350 | 221052729 | PDFSTATSENSEMBLUCSC | 380 | 7 | 2264 | tss | 0.249631 | 0.287061 | Not queried | 1.69 | 4.56e-01 | 4.96e-01 |
|  | 2 | 128283239 | 128283815 | PDFSTATSENSEMBLUCSC | 577 | 5 | 22859 | gene;island | 0.003396 | 0.009362 | Not queried | 1.69 | 2.22e-01 | 2.76e-01 |
|  | 6 | 37400664 | 37400863 | PDFSTATSENSEMBLUCSC | 200 | 4 | 34947 | tss | 0.205202 | 0.300135 | Not queried | 1.69 | 3.43e-01 | 3.84e-01 |
|  | 11 | 2193041 | 2193293 | PDFSTATSENSEMBLUCSC | 253 | 4 | 7063 | tss | 0.785584 | 0.921039 | Not queried | 1.69 | 3.43e-01 | 3.84e-01 |
|  | 1 | 8484004 | 8484417 | PDFSTATSENSEMBLUCSC | 414 | 8 | 196 | gene;tss;island | 0.028071 | 0.046828 | Not queried | 1.69 | 3.82e-01 | 4.27e-01 |
|  | 6 | 31237664 | 31238036 | PDFSTATSENSEMBLUCSC | 373 | 5 | 33865 | gene;island | 0.827989 | 0.731412 | Not queried | 1.69 | 5.56e-02 | 1.00e-01 |
|  | 15 | 52030519 | 52030746 | PDFSTATSENSEMBLUCSC | 228 | 4 | 13352 | gene;tss;island | 0.942390 | 0.824669 | Not queried | 1.69 | 1.14e-01 | 1.68e-01 |
|  | 5 | 140235435 | 140235607 | PDFSTATSENSEMBLUCSC | 173 | 5 | 32355 | tss | 0.945946 | 0.982464 | Not queried | 1.69 | 3.10e-01 | 3.66e-01 |
|  | 6 | 85473171 | 85473344 | PDFSTATSENSEMBLUCSC | 174 | 4 | 34404 | gene;island | 0.867511 | 0.958331 | Not queried | 1.69 | 3.43e-01 | 3.84e-01 |
|  | 19 | 12444273 | 12444910 | PDFSTATSENSEMBLUCSC | 638 | 11 | 21599 | island | 0.010458 | 0.007973 | Not queried | 1.69 | 3.65e-01 | 4.09e-01 |
|  | 19 | 1818466 | 1818886 | PDFSTATSENSEMBLUCSC | 421 | 4 | 19663 | gene;island | 0.934213 | 0.978380 | Not queried | 1.69 | 1.14e-01 | 1.68e-01 |
|  | 5 | 173315549 | 173315978 | PDFSTATSENSEMBLUCSC | 430 | 5 | 31906 | gene;island | 0.040033 | 0.011379 | Not queried | 1.69 | 3.17e-02 | 6.50e-02 |
|  | 6 | 137366322 | 137366545 | PDFSTATSENSEMBLUCSC | 224 | 7 | 35247 | tss | 0.952320 | 0.880205 | Not queried | 1.69 | 5.30e-02 | 1.00e-01 |
|  | 4 | 113332969 | 113333269 | PDFSTATSENSEMBLUCSC | 301 | 6 | 30099 | gene;island | 0.975708 | 0.912887 | Not queried | 1.69 | 9.31e-02 | 1.53e-01 |
|  | 16 | 64984626 | 64984977 | PDFSTATSENSEMBLUCSC | 352 | 5 | 14777 | gene;island | 0.531890 | 0.265861 | Not queried | 1.69 | 5.56e-02 | 1.00e-01 |
|  | 1 | 229567914 | 229568467 | PDFSTATSENSEMBLUCSC | 554 | 5 | 1064 | gene;island | 0.832464 | 0.960341 | Not queried | 1.69 | 2.22e-01 | 2.76e-01 |
|  | 22 | 47370132 | 47370342 | PDFSTATSENSEMBLUCSC | 211 | 4 | 26917 | gene;island | 0.815564 | 0.947731 | Not queried | 1.69 | 1.14e-01 | 1.68e-01 |
|  | 6 | 19692011 | 19692418 | PDFSTATSENSEMBLUCSC | 408 | 6 | 35482 | island | 0.677859 | 0.760222 | Not queried | 1.68 | 3.94e-01 | 4.36e-01 |
|  | 1 | 53924998 | 53925368 | PDFSTATSENSEMBLUCSC | 371 | 6 | 3285 | island | 0.919930 | 0.980866 | Not queried | 1.68 | 1.52e-02 | 5.36e-02 |
|  | 1 | 149857049 | 149857491 | PDFSTATSENSEMBLUCSC | 443 | 6 | 1897 | tss | 0.001488 | 0.005728 | Not queried | 1.68 | 1.80e-01 | 2.45e-01 |
|  | 3 | 38206610 | 38207121 | PDFSTATSENSEMBLUCSC | 512 | 8 | 28989 | island | 0.004762 | 0.008909 | Not queried | 1.68 | 2.79e-01 | 3.38e-01 |
|  | 5 | 172660796 | 172661132 | PDFSTATSENSEMBLUCSC | 337 | 4 | 31903 | gene;island | 0.802836 | 0.949615 | Not queried | 1.68 | 6.86e-01 | 6.99e-01 |
|  | 8 | 144399946 | 144400386 | PDFSTATSENSEMBLUCSC | 441 | 4 | 39472 | gene;island | 0.691179 | 0.960875 | Not queried | 1.68 | 2.00e-01 | 2.55e-01 |
|  | 6 | 126277704 | 126277910 | PDFSTATSENSEMBLUCSC | 207 | 5 | 36464 | island | 0.006161 | 0.002264 | Not queried | 1.68 | 4.21e-01 | 4.59e-01 |
|  | 6 | 32184829 | 32185340 | PDFSTATSENSEMBLUCSC | 512 | 9 | 34096 | gene | 0.384211 | 0.762019 | Not queried | 1.68 | 4.00e-02 | 8.03e-02 |
|  | 12 | 85673200 | 85673347 | PDFSTATSENSEMBLUCSC | 148 | 4 | 9900 | tss;island | 0.865707 | 0.969768 | Not queried | 1.68 | 4.86e-01 | 5.11e-01 |
|  | 2 | 201170790 | 201170828 | PDFSTATSENSEMBLUCSC | 39 | 5 | 22978 | gene;tss;island | 0.158485 | 0.028280 | Not queried | 1.68 | 9.52e-02 | 1.53e-01 |
|  | 1 | 9005474 | 9005910 | PDFSTATSENSEMBLUCSC | 437 | 5 | 1225 | tss | 0.807997 | 0.952675 | Not queried | 1.68 | 5.56e-02 | 1.00e-01 |
|  | 19 | 4909175 | 4909600 | PDFSTATSENSEMBLUCSC | 426 | 11 | 20375 | tss;island | 0.005976 | 0.008128 | Not queried | 1.68 | 7.97e-01 | 8.06e-01 |
|  | 20 | 61732467 | 61732608 | PDFSTATSENSEMBLUCSC | 142 | 4 | 25497 | gene;tss;island | 0.216590 | 0.427518 | Not queried | 1.68 | 2.00e-01 | 2.55e-01 |
|  | 15 | 73976229 | 73976560 | PDFSTATSENSEMBLUCSC | 332 | 5 | 13784 | tss | 0.010983 | 0.052865 | Not queried | 1.68 | 9.52e-02 | 1.53e-01 |
|  | 10 | 132873657 | 132873903 | PDFSTATSENSEMBLUCSC | 247 | 5 | 6275 | island | 0.802249 | 0.957794 | Not queried | 1.68 | 5.56e-02 | 1.00e-01 |
|  | 1 | 151042743 | 151043024 | PDFSTATSENSEMBLUCSC | 282 | 8 | 1921 | tss | 0.001863 | 0.008378 | Not queried | 1.68 | 3.82e-01 | 4.27e-01 |
|  | 12 | 120875539 | 120875898 | PDFSTATSENSEMBLUCSC | 360 | 9 | 10058 | tss | 0.003884 | 0.010062 | Not queried | 1.68 | 9.39e-02 | 1.53e-01 |
|  | 1 | 17765043 | 17765394 | PDFSTATSENSEMBLUCSC | 352 | 6 | 268 | gene;island | 0.002045 | 0.004468 | Not queried | 1.68 | 6.49e-02 | 1.11e-01 |
|  | 11 | 62538951 | 62539386 | PDFSTATSENSEMBLUCSC | 436 | 5 | 6645 | gene | 0.007807 | 0.024140 | Not queried | 1.68 | 3.17e-02 | 6.50e-02 |
|  | 20 | 43935222 | 43935471 | PDFSTATSENSEMBLUCSC | 250 | 8 | 25770 | tss | 0.754717 | 0.459612 | Not queried | 1.68 | 3.28e-01 | 3.84e-01 |
|  | 1 | 203830556 | 203830697 | PDFSTATSENSEMBLUCSC | 142 | 4 | 2196 | tss | 0.007138 | 0.013174 | Not queried | 1.68 | 4.86e-01 | 5.11e-01 |
|  | 2 | 128458240 | 128458845 | PDFSTATSENSEMBLUCSC | 606 | 8 | 24700 | island | 0.061868 | 0.278453 | Not queried | 1.68 | 2.34e-01 | 2.90e-01 |
|  | 19 | 12948848 | 12949060 | PDFSTATSENSEMBLUCSC | 213 | 4 | 20516 | tss;island | 0.347834 | 0.500926 | Not queried | 1.68 | 6.86e-01 | 6.99e-01 |
|  | 21 | 27011436 | 27011482 | PDFSTATSENSEMBLUCSC | 47 | 4 | 26497 | tss;island | 0.196970 | 0.057407 | Not queried | 1.68 | 2.00e-01 | 2.55e-01 |
|  | 10 | 85954312 | 85954336 | PDFSTATSENSEMBLUCSC | 25 | 4 | 5192 | tss;island | 0.312694 | 0.596907 | Not queried | 1.68 | 2.86e-02 | 6.12e-02 |
|  | 6 | 30951917 | 30952175 | PDFSTATSENSEMBLUCSC | 259 | 4 | 33844 | gene | 0.777713 | 0.955432 | Not queried | 1.68 | 4.86e-01 | 5.11e-01 |
|  | 11 | 76380921 | 76381236 | PDFSTATSENSEMBLUCSC | 316 | 8 | 6837 | gene;island | 0.544272 | 0.761167 | Not queried | 1.68 | 3.28e-01 | 3.84e-01 |
|  | 6 | 28553927 | 28555079 | PDFSTATSENSEMBLUCSC | 1153 | 20 | 33639 | gene | 0.009866 | 0.026636 | Not queried | 1.68 | 1.08e-01 | 1.68e-01 |
|  | 17 | 42393858 | 42394004 | PDFSTATSENSEMBLUCSC | 147 | 4 | 16615 | gene;island | 0.563367 | 0.890227 | Not queried | 1.68 | 1.14e-01 | 1.68e-01 |
|  | 19 | 58111307 | 58111769 | PDFSTATSENSEMBLUCSC | 463 | 6 | 20248 | gene | 0.123620 | 0.331804 | Not queried | 1.68 | 2.40e-01 | 2.95e-01 |
|  | 12 | 7023195 | 7023352 | PDFSTATSENSEMBLUCSC | 158 | 4 | 9039 | gene | 0.910264 | 0.827626 | Not queried | 1.68 | 4.86e-01 | 5.11e-01 |
|  | 1 | 150534021 | 150534265 | PDFSTATSENSEMBLUCSC | 245 | 5 | 3725 | island | 0.006509 | 0.020778 | Not queried | 1.68 | 2.22e-01 | 2.76e-01 |
|  | 2 | 1286216 | 1286641 | PDFSTATSENSEMBLUCSC | 426 | 5 | 22528 | gene;island | 0.744578 | 0.665376 | Not queried | 1.68 | 1.00e+00 | 1.00e+00 |
|  | 7 | 1286023 | 1286386 | PDFSTATSENSEMBLUCSC | 364 | 4 | 38095 | island | 0.484564 | 0.660297 | Not queried | 1.68 | 1.00e+00 | 1.00e+00 |
|  | 6 | 32808504 | 32808918 | PDFSTATSENSEMBLUCSC | 415 | 7 | 34147 | gene;island | 0.961676 | 0.854296 | Not queried | 1.68 | 2.62e-02 | 6.12e-02 |
|  | 19 | 54641515 | 54641696 | PDFSTATSENSEMBLUCSC | 182 | 4 | 20217 | gene | 0.004085 | 0.012964 | Not queried | 1.68 | 5.71e-02 | 1.00e-01 |
|  | 6 | 30710200 | 30710447 | PDFSTATSENSEMBLUCSC | 248 | 5 | 33823 | gene | 0.003736 | 0.009932 | Not queried | 1.68 | 4.21e-01 | 4.59e-01 |
|  | 6 | 37400664 | 37400926 | PDFSTATSENSEMBLUCSC | 263 | 5 | 36097 | island | 0.091106 | 0.124810 | Not queried | 1.68 | 4.21e-01 | 4.59e-01 |
|  | 7 | 75831232 | 75831678 | PDFSTATSENSEMBLUCSC | 447 | 5 | 37051 | gene | 0.002819 | 0.004037 | Not queried | 1.68 | 8.41e-01 | 8.48e-01 |
|  | 10 | 70319998 | 70320213 | PDFSTATSENSEMBLUCSC | 216 | 6 | 5794 | island | 0.069631 | 0.026178 | Not queried | 1.67 | 2.40e-01 | 2.95e-01 |
|  | 11 | 442018 | 442380 | PDFSTATSENSEMBLUCSC | 363 | 6 | 7012 | tss | 0.357916 | 0.125337 | Not queried | 1.67 | 8.18e-01 | 8.27e-01 |
|  | 17 | 27312855 | 27313254 | PDFSTATSENSEMBLUCSC | 400 | 6 | 16504 | gene;island | 0.627177 | 0.793582 | Not queried | 1.67 | 6.49e-02 | 1.11e-01 |
|  | 5 | 137549033 | 137549144 | PDFSTATSENSEMBLUCSC | 112 | 6 | 32316 | tss | 0.001072 | 0.003482 | Not queried | 1.67 | 4.85e-01 | 5.11e-01 |
|  | 12 | 130821453 | 130821607 | PDFSTATSENSEMBLUCSC | 155 | 4 | 10119 | tss;island | 0.953830 | 0.743210 | Not queried | 1.67 | 3.43e-01 | 3.84e-01 |
|  | 1 | 21835945 | 21836326 | PDFSTATSENSEMBLUCSC | 382 | 4 | 297 | gene;island | 0.849917 | 0.920367 | Not queried | 1.67 | 8.86e-01 | 8.89e-01 |
|  | 2 | 176988203 | 176988580 | PDFSTATSENSEMBLUCSC | 378 | 5 | 22937 | gene;island | 0.900902 | 0.971590 | Not queried | 1.67 | 3.10e-01 | 3.66e-01 |
|  | 10 | 102802634 | 102803042 | PDFSTATSENSEMBLUCSC | 409 | 4 | 6047 | island | 0.004845 | 0.012075 | Not queried | 1.67 | 3.43e-01 | 3.84e-01 |
|  | 1 | 92414722 | 92415145 | PDFSTATSENSEMBLUCSC | 424 | 6 | 3479 | island | 0.855188 | 0.980836 | Not queried | 1.67 | 9.31e-02 | 1.53e-01 |
|  | 10 | 118429452 | 118429952 | PDFSTATSENSEMBLUCSC | 501 | 8 | 6165 | island | 0.697414 | 0.466836 | Not queried | 1.67 | 2.79e-01 | 3.38e-01 |
|  | 17 | 62207299 | 62207789 | PDFSTATSENSEMBLUCSC | 491 | 7 | 18655 | island | 0.002142 | 0.006881 | Not queried | 1.67 | 3.18e-01 | 3.74e-01 |
|  | 17 | 62207504 | 62207789 | PDFSTATSENSEMBLUCSC | 286 | 5 | 17579 | tss | 0.002142 | 0.006881 | Not queried | 1.67 | 4.21e-01 | 4.59e-01 |
|  | 6 | 31830721 | 31830947 | PDFSTATSENSEMBLUCSC | 227 | 8 | 34845 | tss | 0.003638 | 0.007362 | Not queried | 1.67 | 6.50e-02 | 1.11e-01 |
|  | 7 | 148036314 | 148036740 | PDFSTATSENSEMBLUCSC | 427 | 7 | 37258 | gene;island | 0.421817 | 0.016247 | Not queried | 1.67 | 2.59e-01 | 3.16e-01 |
|  | 10 | 100227419 | 100227782 | PDFSTATSENSEMBLUCSC | 364 | 4 | 4704 | gene;island | 0.018565 | 0.004057 | Not queried | 1.67 | 4.86e-01 | 5.11e-01 |
|  | 2 | 219264573 | 219264807 | PDFSTATSENSEMBLUCSC | 235 | 5 | 23017 | gene;tss;island | 0.008972 | 0.024066 | Not queried | 1.67 | 9.52e-02 | 1.53e-01 |
|  | 17 | 41909601 | 41909716 | PDFSTATSENSEMBLUCSC | 116 | 4 | 16599 | gene;island | 0.922507 | 0.755639 | Not queried | 1.67 | 1.14e-01 | 1.68e-01 |
|  | 10 | 46090241 | 46090807 | PDFSTATSENSEMBLUCSC | 567 | 8 | 5714 | island | 0.008853 | 0.011040 | Not queried | 1.67 | 7.21e-01 | 7.30e-01 |
|  | 16 | 70557093 | 70557485 | PDFSTATSENSEMBLUCSC | 393 | 6 | 15341 | tss;island | 0.003113 | 0.008725 | Not queried | 1.67 | 1.32e-01 | 1.89e-01 |
|  | 5 | 177580860 | 177581072 | PDFSTATSENSEMBLUCSC | 213 | 7 | 33464 | island | 0.005385 | 0.012703 | Not queried | 1.67 | 9.73e-02 | 1.55e-01 |
|  | 2 | 86850757 | 86850888 | PDFSTATSENSEMBLUCSC | 132 | 4 | 22772 | gene | 0.003655 | 0.009205 | Not queried | 1.67 | 6.86e-01 | 6.99e-01 |
|  | 2 | 241905733 | 241905910 | PDFSTATSENSEMBLUCSC | 178 | 5 | 25282 | island | 0.842491 | 0.919368 | Not queried | 1.67 | 1.59e-02 | 5.36e-02 |
|  | 19 | 57701880 | 57702809 | PDFSTATSENSEMBLUCSC | 930 | 19 | 21106 | tss | 0.919824 | 0.761205 | Not queried | 1.67 | 8.40e-01 | 8.48e-01 |
|  | 10 | 13748985 | 13749625 | PDFSTATSENSEMBLUCSC | 641 | 9 | 4504 | gene | 0.012359 | 0.001343 | Not queried | 1.67 | 3.15e-02 | 6.50e-02 |
|  | 2 | 10444432 | 10445016 | PDFSTATSENSEMBLUCSC | 585 | 6 | 22566 | gene;island | 0.550924 | 0.825779 | Not queried | 1.67 | 1.32e-01 | 1.89e-01 |
|  | 1 | 200992902 | 200993311 | PDFSTATSENSEMBLUCSC | 410 | 5 | 2163 | tss | 0.654724 | 0.136345 | Not queried | 1.67 | 5.48e-01 | 5.70e-01 |
|  | 4 | 85422998 | 85423302 | PDFSTATSENSEMBLUCSC | 305 | 4 | 31162 | island | 0.738617 | 0.869339 | Not queried | 1.67 | 3.43e-01 | 3.84e-01 |
|  | 6 | 85484287 | 85484863 | PDFSTATSENSEMBLUCSC | 577 | 5 | 36299 | island | 0.906856 | 0.946554 | Not queried | 1.67 | 3.10e-01 | 3.66e-01 |
|  | 7 | 102715121 | 102715232 | PDFSTATSENSEMBLUCSC | 112 | 8 | 37170 | gene;tss;island | 0.008521 | 0.031597 | Not queried | 1.67 | 2.81e-02 | 6.12e-02 |
|  | 17 | 46800444 | 46800680 | PDFSTATSENSEMBLUCSC | 237 | 4 | 17470 | tss;island | 0.899968 | 0.981528 | Not queried | 1.67 | 3.43e-01 | 3.84e-01 |
|  | 16 | 31117067 | 31117318 | PDFSTATSENSEMBLUCSC | 252 | 4 | 15868 | island | 0.017719 | 0.073663 | Not queried | 1.67 | 2.00e-01 | 2.55e-01 |
|  | 17 | 14207530 | 14207968 | PDFSTATSENSEMBLUCSC | 439 | 5 | 16451 | gene;island | 0.915811 | 0.971828 | Not queried | 1.67 | 5.56e-02 | 1.00e-01 |
|  | 15 | 99409194 | 99409506 | PDFSTATSENSEMBLUCSC | 313 | 4 | 13497 | gene | 0.056770 | 0.018027 | Not queried | 1.67 | 2.86e-02 | 6.12e-02 |
|  | 20 | 39945503 | 39946125 | PDFSTATSENSEMBLUCSC | 623 | 5 | 26168 | island | 0.009828 | 0.019725 | Not queried | 1.67 | 5.48e-01 | 5.70e-01 |
|  | 8 | 97171668 | 97172012 | PDFSTATSENSEMBLUCSC | 345 | 4 | 39367 | gene;island | 0.893362 | 0.966313 | Not queried | 1.67 | 4.86e-01 | 5.11e-01 |
|  | 8 | 145729106 | 145729373 | PDFSTATSENSEMBLUCSC | 268 | 6 | 39987 | tss | 0.803658 | 0.932464 | Not queried | 1.67 | 1.80e-01 | 2.45e-01 |
|  | 4 | 17782853 | 17783502 | PDFSTATSENSEMBLUCSC | 650 | 11 | 30949 | island | 0.834711 | 0.605013 | FAM184B (margin: FAM184B) | 1.67 | 1.28e-02 | 4.76e-02 |
|  | 3 | 57204106 | 57204530 | PDFSTATSENSEMBLUCSC | 425 | 7 | 29223 | island | 0.013663 | 0.004840 | Not queried | 1.66 | 2.59e-01 | 3.16e-01 |
|  | 2 | 74648700 | 74649460 | PDFSTATSENSEMBLUCSC | 761 | 8 | 24430 | island | 0.003456 | 0.006518 | Not queried | 1.66 | 2.34e-01 | 2.90e-01 |
|  | 8 | 144120335 | 144120648 | PDFSTATSENSEMBLUCSC | 314 | 5 | 39943 | tss | 0.307493 | 0.286367 | Not queried | 1.66 | 5.48e-01 | 5.70e-01 |
|  | 15 | 83317281 | 83317632 | PDFSTATSENSEMBLUCSC | 352 | 5 | 13858 | tss;island | 0.941671 | 0.965658 | Not queried | 1.66 | 5.48e-01 | 5.70e-01 |
|  | 21 | 37852479 | 37852822 | PDFSTATSENSEMBLUCSC | 344 | 4 | 26536 | tss | 0.909109 | 0.803430 | Not queried | 1.66 | 2.00e-01 | 2.55e-01 |
|  | 14 | 65380577 | 65381464 | PDFSTATSENSEMBLUCSC | 888 | 15 | 12944 | island | 0.002824 | 0.005800 | Not queried | 1.66 | 2.67e-01 | 3.25e-01 |
|  | 6 | 32048449 | 32048679 | PDFSTATSENSEMBLUCSC | 231 | 4 | 34035 | gene;island | 0.904518 | 0.732454 | Not queried | 1.66 | 4.86e-01 | 5.11e-01 |
|  | 6 | 31865522 | 31866286 | PDFSTATSENSEMBLUCSC | 765 | 26 | 34849 | tss;island | 0.003202 | 0.010547 | C2; EHMT2; ZBTB12 (margin: C2; EHMT2; ZBTB12) | 1.66 | 5.18e-04 | 6.47e-03 |
|  | 1 | 111888719 | 111889159 | PDFSTATSENSEMBLUCSC | 441 | 8 | 1823 | tss | 0.072979 | 0.019086 | PIFO (margin: PIFO; CHIAP3) | 1.66 | 1.04e-02 | 3.96e-02 |
|  | 16 | 3306524 | 3307037 | PDFSTATSENSEMBLUCSC | 514 | 4 | 15643 | island | 0.533645 | 0.787061 | Not queried | 1.66 | 4.86e-01 | 5.11e-01 |
|  | 2 | 241065401 | 241065850 | PDFSTATSENSEMBLUCSC | 450 | 5 | 25266 | island | 0.970289 | 0.990039 | Not queried | 1.66 | 1.51e-01 | 2.11e-01 |
|  | 16 | 2581414 | 2581833 | PDFSTATSENSEMBLUCSC | 420 | 5 | 15608 | island | 0.878542 | 0.913427 | Not queried | 1.66 | 4.21e-01 | 4.59e-01 |
|  | 19 | 38183253 | 38183456 | PDFSTATSENSEMBLUCSC | 204 | 6 | 20743 | tss | 0.936413 | 0.967609 | Not queried | 1.66 | 1.32e-01 | 1.89e-01 |
|  | 10 | 43572065 | 43572467 | PDFSTATSENSEMBLUCSC | 403 | 7 | 5044 | tss | 0.178708 | 0.017525 | Not queried | 1.66 | 3.79e-02 | 7.66e-02 |
|  | 6 | 32976805 | 32976897 | PDFSTATSENSEMBLUCSC | 93 | 4 | 34178 | gene;island | 0.499754 | 0.777563 | Not queried | 1.66 | 2.86e-02 | 6.12e-02 |
|  | 10 | 23479993 | 23480316 | PDFSTATSENSEMBLUCSC | 324 | 6 | 4987 | tss;island | 0.716119 | 0.939627 | Not queried | 1.66 | 3.10e-01 | 3.66e-01 |
|  | 8 | 6664028 | 6664281 | PDFSTATSENSEMBLUCSC | 254 | 4 | 40053 | island | 0.802604 | 0.835588 | Not queried | 1.66 | 8.86e-01 | 8.89e-01 |
|  | 10 | 123355268 | 123355748 | PDFSTATSENSEMBLUCSC | 481 | 4 | 4794 | gene;island | 0.874629 | 0.956753 | Not queried | 1.66 | 2.00e-01 | 2.55e-01 |
|  | 15 | 91427821 | 91428056 | PDFSTATSENSEMBLUCSC | 236 | 5 | 13478 | gene;tss;island | 0.909605 | 0.969604 | Not queried | 1.66 | 1.59e-02 | 5.36e-02 |
|  | 4 | 185939339 | 185940173 | PDFSTATSENSEMBLUCSC | 835 | 12 | 31467 | island | 0.438027 | 0.635310 | Not queried | 1.66 | 4.78e-01 | 5.11e-01 |
|  | 14 | 23425770 | 23426164 | PDFSTATSENSEMBLUCSC | 395 | 6 | 12020 | gene;island | 0.002287 | 0.018500 | Not queried | 1.66 | 1.80e-01 | 2.45e-01 |
|  | 2 | 150176762 | 150177277 | PDFSTATSENSEMBLUCSC | 516 | 6 | 24779 | island | 0.897342 | 0.962331 | Not queried | 1.66 | 2.60e-02 | 6.12e-02 |
|  | 2 | 201980838 | 201980910 | PDFSTATSENSEMBLUCSC | 73 | 4 | 22985 | gene | 0.003461 | 0.010422 | Not queried | 1.66 | 2.00e-01 | 2.55e-01 |
|  | 1 | 2003864 | 2004346 | PDFSTATSENSEMBLUCSC | 483 | 5 | 56 | gene;tss;island | 0.959479 | 0.986036 | Not queried | 1.66 | 2.22e-01 | 2.76e-01 |
|  | 13 | 20356921 | 20357223 | PDFSTATSENSEMBLUCSC | 303 | 5 | 11561 | island | 0.003822 | 0.010770 | Not queried | 1.66 | 2.22e-01 | 2.76e-01 |
|  | 6 | 44310228 | 44310350 | PDFSTATSENSEMBLUCSC | 123 | 6 | 35013 | tss | 0.758090 | 0.883138 | Not queried | 1.66 | 6.49e-02 | 1.11e-01 |
|  | 13 | 20735139 | 20735532 | PDFSTATSENSEMBLUCSC | 394 | 8 | 11569 | island | 0.012428 | 0.035620 | Not queried | 1.66 | 1.61e-01 | 2.24e-01 |
|  | 5 | 149887422 | 149887497 | PDFSTATSENSEMBLUCSC | 76 | 4 | 32431 | tss | 0.841353 | 0.567069 | Not queried | 1.66 | 1.14e-01 | 1.68e-01 |
|  | 1 | 101701734 | 101702062 | PDFSTATSENSEMBLUCSC | 329 | 5 | 1783 | tss;island | 0.022908 | 0.087071 | Not queried | 1.66 | 4.21e-01 | 4.59e-01 |
|  | 2 | 176936377 | 176936581 | PDFSTATSENSEMBLUCSC | 205 | 4 | 24885 | island | 0.922549 | 0.968306 | Not queried | 1.66 | 3.43e-01 | 3.84e-01 |
|  | 8 | 107782513 | 107782734 | PDFSTATSENSEMBLUCSC | 222 | 5 | 39864 | tss | 0.651925 | 0.854063 | Not queried | 1.66 | 9.52e-02 | 1.53e-01 |
|  | 18 | 76740088 | 76740284 | PDFSTATSENSEMBLUCSC | 197 | 7 | 19593 | island | 0.950974 | 0.987203 | SALL3; RP11-849I19.1 (margin: SALL3; RP11-849I19.1) | 1.66 | 2.33e-03 | 1.52e-02 |
|  | 3 | 159756648 | 159757071 | PDFSTATSENSEMBLUCSC | 424 | 6 | 29626 | island | 0.751566 | 0.892692 | Not queried | 1.66 | 3.10e-01 | 3.66e-01 |
|  | 8 | 72470700 | 72471153 | PDFSTATSENSEMBLUCSC | 454 | 4 | 40381 | island | 0.733193 | 0.636665 | Not queried | 1.66 | 6.86e-01 | 6.99e-01 |
|  | 22 | 42896105 | 42896573 | PDFSTATSENSEMBLUCSC | 469 | 6 | 27191 | tss | 0.700385 | 0.904744 | SERHL (margin: SERHL; RRP7A) | 1.65 | 8.66e-03 | 3.36e-02 |
|  | 3 | 173302142 | 173302602 | PDFSTATSENSEMBLUCSC | 461 | 5 | 28034 | gene | 0.436570 | 0.751829 | Not queried | 1.65 | 3.10e-01 | 3.66e-01 |
|  | 2 | 182850059 | 182850441 | PDFSTATSENSEMBLUCSC | 383 | 4 | 23764 | tss | 0.708114 | 0.861497 | Not queried | 1.65 | 3.43e-01 | 3.84e-01 |
|  | 7 | 111846693 | 111846915 | PDFSTATSENSEMBLUCSC | 223 | 5 | 37189 | gene | 0.002867 | 0.003221 | Not queried | 1.65 | 2.22e-01 | 2.76e-01 |
|  | 14 | 50101902 | 50102006 | PDFSTATSENSEMBLUCSC | 105 | 8 | 12821 | island | 0.003927 | 0.009958 | Not queried | 1.65 | 2.79e-01 | 3.38e-01 |
|  | 14 | 50101952 | 50102006 | PDFSTATSENSEMBLUCSC | 55 | 6 | 12379 | tss | 0.003927 | 0.009958 | Not queried | 1.65 | 4.85e-01 | 5.11e-01 |
|  | 3 | 10436197 | 10436869 | PDFSTATSENSEMBLUCSC | 673 | 9 | 27662 | gene | 0.669275 | 0.886978 | Not queried | 1.65 | 6.25e-02 | 1.09e-01 |
|  | 1 | 16399560 | 16400060 | PDFSTATSENSEMBLUCSC | 501 | 5 | 258 | gene;island | 0.073701 | 0.041966 | Not queried | 1.65 | 5.48e-01 | 5.70e-01 |
|  | 2 | 121279886 | 121280106 | PDFSTATSENSEMBLUCSC | 221 | 4 | 24667 | island | 0.841392 | 0.935277 | Not queried | 1.65 | 3.43e-01 | 3.84e-01 |
|  | 16 | 14403004 | 14403138 | PDFSTATSENSEMBLUCSC | 135 | 4 | 15102 | tss | 0.983366 | 0.925336 | Not queried | 1.65 | 2.00e-01 | 2.55e-01 |
|  | 11 | 70858237 | 70858695 | PDFSTATSENSEMBLUCSC | 459 | 6 | 6799 | gene | 0.817023 | 0.943812 | Not queried | 1.65 | 9.31e-02 | 1.53e-01 |
|  | 8 | 28748202 | 28748679 | PDFSTATSENSEMBLUCSC | 478 | 5 | 39266 | gene;tss;island | 0.002995 | 0.011647 | Not queried | 1.65 | 1.00e+00 | 1.00e+00 |
|  | 6 | 166711138 | 166711488 | PDFSTATSENSEMBLUCSC | 351 | 5 | 36629 | island | 0.634202 | 0.862749 | Not queried | 1.65 | 4.21e-01 | 4.59e-01 |
|  | 5 | 141348202 | 141348550 | PDFSTATSENSEMBLUCSC | 349 | 10 | 31841 | gene;tss;island | 0.009410 | 0.004858 | Not queried | 1.65 | 4.36e-01 | 4.76e-01 |
|  | 5 | 88179867 | 88180330 | PDFSTATSENSEMBLUCSC | 464 | 6 | 31683 | gene;tss;island | 0.654113 | 0.397112 | MEF2C-AS1; MEF2C (margin: MEF2C-AS1; MEF2C) | 1.65 | 2.16e-03 | 1.45e-02 |
|  | 20 | 42574332 | 42574362 | PDFSTATSENSEMBLUCSC | 31 | 4 | 25423 | gene;tss | 0.834338 | 0.694598 | Not queried | 1.65 | 3.43e-01 | 3.84e-01 |
|  | 1 | 92495720 | 92495877 | PDFSTATSENSEMBLUCSC | 158 | 4 | 632 | gene;island | 0.028209 | 0.011085 | Not queried | 1.65 | 2.86e-02 | 6.12e-02 |
|  | 7 | 4901337 | 4901798 | PDFSTATSENSEMBLUCSC | 462 | 7 | 36862 | gene;island | 0.881810 | 0.973266 | Not queried | 1.65 | 1.75e-02 | 5.79e-02 |
|  | 4 | 40337262 | 40337443 | PDFSTATSENSEMBLUCSC | 182 | 4 | 30357 | tss | 0.933248 | 0.836298 | Not queried | 1.65 | 4.86e-01 | 5.11e-01 |
|  | 2 | 133014663 | 133014805 | PDFSTATSENSEMBLUCSC | 143 | 6 | 23637 | tss | 0.639625 | 0.841808 | Not queried | 1.65 | 6.49e-02 | 1.11e-01 |
|  | 10 | 134411295 | 134411480 | PDFSTATSENSEMBLUCSC | 186 | 4 | 4868 | gene;island | 0.917174 | 0.964689 | Not queried | 1.65 | 3.43e-01 | 3.84e-01 |
|  | 16 | 3068014 | 3068707 | PDFSTATSENSEMBLUCSC | 694 | 11 | 15629 | island | 0.911663 | 0.970124 | TNFRSF12A; CLDN6 (margin: LA16c-380H5.5; CLDN9; TNFRSF12A; THOC6; CLDN6; HCFC1R1; CCDC64B) | 1.65 | 1.28e-02 | 4.76e-02 |
|  | 4 | 17783205 | 17783502 | PDFSTATSENSEMBLUCSC | 298 | 8 | 30318 | tss | 0.922789 | 0.617961 | Not queried | 1.65 | 8.30e-02 | 1.40e-01 |
|  | 18 | 76740088 | 76740262 | PDFSTATSENSEMBLUCSC | 175 | 6 | 19332 | tss | 0.957786 | 0.987872 | SALL3; RP11-849I19.1 (margin: SALL3; RP11-849I19.1) | 1.65 | 2.16e-03 | 1.45e-02 |
|  | 21 | 33244742 | 33245114 | PDFSTATSENSEMBLUCSC | 373 | 5 | 26508 | tss;island | 0.495301 | 0.109055 | Not queried | 1.65 | 4.21e-01 | 4.59e-01 |
|  | 11 | 2293552 | 2293665 | PDFSTATSENSEMBLUCSC | 114 | 6 | 7065 | tss;island | 0.874190 | 0.952409 | ASCL2 (margin: ASCL2) | 1.65 | 2.16e-03 | 1.45e-02 |
|  | 3 | 101546486 | 101546814 | PDFSTATSENSEMBLUCSC | 329 | 5 | 28481 | tss | 0.000273 | 0.001168 | Not queried | 1.65 | 3.10e-01 | 3.66e-01 |
|  | 12 | 75601711 | 75601928 | PDFSTATSENSEMBLUCSC | 218 | 4 | 9219 | gene;island | 0.820544 | 0.953394 | Not queried | 1.65 | 2.00e-01 | 2.55e-01 |
|  | 8 | 145691582 | 145691776 | PDFSTATSENSEMBLUCSC | 195 | 4 | 39985 | tss;island | 0.003974 | 0.001954 | Not queried | 1.65 | 4.86e-01 | 5.11e-01 |
|  | 5 | 72251326 | 72251508 | PDFSTATSENSEMBLUCSC | 183 | 4 | 32119 | tss;island | 0.008554 | 0.049501 | Not queried | 1.65 | 1.14e-01 | 1.68e-01 |
|  | 17 | 15635376 | 15635468 | PDFSTATSENSEMBLUCSC | 93 | 5 | 17151 | tss | 0.779391 | 0.865437 | Not queried | 1.65 | 1.51e-01 | 2.11e-01 |
|  | 17 | 21495693 | 21495791 | PDFSTATSENSEMBLUCSC | 99 | 4 | 18126 | island | 0.748301 | 0.906960 | Not queried | 1.65 | 4.86e-01 | 5.11e-01 |
|  | 17 | 618140 | 618545 | PDFSTATSENSEMBLUCSC | 406 | 7 | 16980 | tss | 0.003181 | 0.007872 | Not queried | 1.65 | 3.18e-01 | 3.74e-01 |
|  | 2 | 231851704 | 231852154 | PDFSTATSENSEMBLUCSC | 451 | 5 | 25176 | island | 0.201396 | 0.042562 | Not queried | 1.65 | 5.48e-01 | 5.70e-01 |
|  | 8 | 23571979 | 23572375 | PDFSTATSENSEMBLUCSC | 397 | 5 | 40158 | island | 0.821810 | 0.953515 | Not queried | 1.65 | 9.52e-02 | 1.53e-01 |
|  | 2 | 230933630 | 230933934 | PDFSTATSENSEMBLUCSC | 305 | 4 | 23936 | tss | 0.333688 | 0.599855 | Not queried | 1.65 | 4.86e-01 | 5.11e-01 |
|  | 13 | 95248547 | 95248888 | PDFSTATSENSEMBLUCSC | 342 | 6 | 11498 | tss | 0.114395 | 0.012774 | Not queried | 1.65 | 3.10e-01 | 3.66e-01 |
|  | 1 | 3230250 | 3230647 | PDFSTATSENSEMBLUCSC | 398 | 5 | 119 | gene | 0.629700 | 0.925316 | Not queried | 1.65 | 1.51e-01 | 2.11e-01 |
|  | 13 | 37393368 | 37393523 | PDFSTATSENSEMBLUCSC | 156 | 4 | 11134 | gene | 0.011803 | 0.016211 | Not queried | 1.65 | 4.86e-01 | 5.11e-01 |
|  | 7 | 27219224 | 27219704 | PDFSTATSENSEMBLUCSC | 481 | 4 | 36940 | gene;island | 0.259958 | 0.364608 | Not queried | 1.65 | 4.86e-01 | 5.11e-01 |
|  | 17 | 8213195 | 8213522 | PDFSTATSENSEMBLUCSC | 328 | 4 | 17117 | tss | 0.457975 | 0.862633 | Not queried | 1.65 | 1.14e-01 | 1.68e-01 |
|  | 5 | 176900792 | 176900842 | PDFSTATSENSEMBLUCSC | 51 | 5 | 32529 | tss | 0.029008 | 0.016431 | Not queried | 1.65 | 3.10e-01 | 3.66e-01 |
|  | 1 | 3459949 | 3460467 | PDFSTATSENSEMBLUCSC | 519 | 4 | 129 | gene;island | 0.465018 | 0.698733 | Not queried | 1.65 | 6.86e-01 | 6.99e-01 |
|  | 19 | 5622997 | 5623257 | PDFSTATSENSEMBLUCSC | 261 | 11 | 20378 | tss;island | 0.001808 | 0.006627 | Not queried | 1.65 | 7.59e-02 | 1.28e-01 |
|  | 12 | 48099724 | 48099889 | PDFSTATSENSEMBLUCSC | 166 | 4 | 10352 | island | 0.003890 | 0.006157 | Not queried | 1.65 | 3.43e-01 | 3.84e-01 |
|  | 14 | 50778832 | 50779169 | PDFSTATSENSEMBLUCSC | 338 | 6 | 12386 | tss;island | 0.004501 | 0.004628 | Not queried | 1.65 | 1.80e-01 | 2.45e-01 |
|  | 21 | 42880448 | 42880714 | PDFSTATSENSEMBLUCSC | 267 | 5 | 26559 | tss;island | 0.825451 | 0.676599 | Not queried | 1.65 | 8.41e-01 | 8.48e-01 |
|  | 10 | 11344086 | 11344395 | PDFSTATSENSEMBLUCSC | 310 | 4 | 4494 | gene | 0.567035 | 0.870701 | Not queried | 1.65 | 3.43e-01 | 3.84e-01 |
|  | 17 | 45810359 | 45810512 | PDFSTATSENSEMBLUCSC | 154 | 7 | 17444 | tss | 0.887348 | 0.945785 | Not queried | 1.64 | 9.73e-02 | 1.55e-01 |
|  | 2 | 74753691 | 74753946 | PDFSTATSENSEMBLUCSC | 256 | 4 | 23462 | tss;island | 0.887836 | 0.961807 | Not queried | 1.64 | 2.00e-01 | 2.55e-01 |
|  | 10 | 72648543 | 72648693 | PDFSTATSENSEMBLUCSC | 151 | 4 | 5140 | tss | 0.026237 | 0.013922 | Not queried | 1.64 | 2.00e-01 | 2.55e-01 |
|  | 1 | 53905612 | 53905934 | PDFSTATSENSEMBLUCSC | 323 | 6 | 3284 | island | 0.937444 | 0.973791 | Not queried | 1.64 | 3.94e-01 | 4.36e-01 |
|  | 10 | 134404873 | 134405566 | PDFSTATSENSEMBLUCSC | 694 | 8 | 4867 | gene | 0.899069 | 0.764748 | Not queried | 1.64 | 7.21e-01 | 7.30e-01 |
|  | 19 | 22193293 | 22193724 | PDFSTATSENSEMBLUCSC | 432 | 5 | 19963 | gene | 0.389915 | 0.673977 | Not queried | 1.64 | 3.17e-02 | 6.50e-02 |
|  | 19 | 22193293 | 22193911 | PDFSTATSENSEMBLUCSC | 619 | 7 | 21841 | island | 0.539255 | 0.673977 | Not queried | 1.64 | 3.79e-02 | 7.66e-02 |
|  | 16 | 57405979 | 57406353 | PDFSTATSENSEMBLUCSC | 375 | 5 | 15261 | tss | 0.736595 | 0.937655 | Not queried | 1.64 | 5.48e-01 | 5.70e-01 |
|  | 15 | 66274614 | 66274894 | PDFSTATSENSEMBLUCSC | 281 | 5 | 13385 | gene;island | 0.291147 | 0.080876 | Not queried | 1.64 | 9.52e-02 | 1.53e-01 |
|  | 22 | 18632404 | 18632618 | PDFSTATSENSEMBLUCSC | 215 | 5 | 26949 | tss | 0.032089 | 0.078292 | Not queried | 1.64 | 1.51e-01 | 2.11e-01 |
|  | 1 | 6390473 | 6390830 | PDFSTATSENSEMBLUCSC | 358 | 5 | 169 | gene | 0.977643 | 0.982638 | Not queried | 1.64 | 3.10e-01 | 3.66e-01 |
|  | 6 | 53412803 | 53413189 | PDFSTATSENSEMBLUCSC | 387 | 5 | 36215 | island | 0.003518 | 0.008170 | Not queried | 1.64 | 2.22e-01 | 2.76e-01 |
|  | 12 | 133263907 | 133264322 | PDFSTATSENSEMBLUCSC | 416 | 7 | 10132 | tss;island | 0.006784 | 0.008717 | Not queried | 1.64 | 3.18e-01 | 3.74e-01 |
|  | 2 | 170220063 | 170220388 | PDFSTATSENSEMBLUCSC | 326 | 4 | 23701 | tss;island | 0.433845 | 0.260903 | Not queried | 1.64 | 4.86e-01 | 5.11e-01 |
|  | 22 | 38851318 | 38851366 | PDFSTATSENSEMBLUCSC | 49 | 6 | 27139 | tss;island | 0.731022 | 0.537697 | Not queried | 1.64 | 1.80e-01 | 2.45e-01 |
|  | 8 | 95566223 | 95566670 | PDFSTATSENSEMBLUCSC | 448 | 4 | 39817 | tss;island | 0.001714 | 0.001889 | Not queried | 1.64 | 8.86e-01 | 8.89e-01 |
|  | 11 | 77348873 | 77349090 | PDFSTATSENSEMBLUCSC | 218 | 7 | 7586 | tss | 0.005858 | 0.004119 | Not queried | 1.64 | 1.00e+00 | 1.00e+00 |
|  | 17 | 77704741 | 77704772 | PDFSTATSENSEMBLUCSC | 32 | 4 | 17715 | tss | 0.857494 | 0.935139 | Not queried | 1.64 | 2.86e-02 | 6.12e-02 |
|  | 1 | 60280716 | 60280868 | PDFSTATSENSEMBLUCSC | 153 | 4 | 559 | gene;island | 0.074081 | 0.021064 | Not queried | 1.64 | 6.86e-01 | 6.99e-01 |
|  | 14 | 71373858 | 71373980 | PDFSTATSENSEMBLUCSC | 123 | 4 | 12490 | tss;island | 0.012474 | 0.053677 | Not queried | 1.64 | 5.71e-02 | 1.00e-01 |
|  | 18 | 54318615 | 54318940 | PDFSTATSENSEMBLUCSC | 326 | 4 | 19119 | gene | 0.010457 | 0.016155 | Not queried | 1.64 | 4.86e-01 | 5.11e-01 |
|  | 7 | 44259684 | 44259913 | PDFSTATSENSEMBLUCSC | 230 | 4 | 36991 | gene;island | 0.861077 | 0.977705 | Not queried | 1.64 | 1.14e-01 | 1.68e-01 |
|  | 6 | 31782675 | 31782761 | PDFSTATSENSEMBLUCSC | 87 | 4 | 33967 | gene | 0.024483 | 0.001843 | Not queried | 1.64 | 3.43e-01 | 3.84e-01 |
|  | 17 | 74524852 | 74525125 | PDFSTATSENSEMBLUCSC | 274 | 4 | 16792 | gene | 0.966030 | 0.943941 | Not queried | 1.64 | 4.86e-01 | 5.11e-01 |
|  | 8 | 1615950 | 1616925 | PDFSTATSENSEMBLUCSC | 976 | 10 | 39171 | gene;island | 0.326504 | 0.623556 | Not queried | 1.64 | 5.24e-02 | 1.00e-01 |
|  | 17 | 76794506 | 76795028 | PDFSTATSENSEMBLUCSC | 523 | 5 | 16826 | gene;island | 0.888424 | 0.967307 | Not queried | 1.64 | 5.56e-02 | 1.00e-01 |
|  | 10 | 50339928 | 50340173 | PDFSTATSENSEMBLUCSC | 246 | 4 | 4582 | gene;island | 0.586784 | 0.801171 | Not queried | 1.64 | 1.14e-01 | 1.68e-01 |
|  | 10 | 105362388 | 105362799 | PDFSTATSENSEMBLUCSC | 412 | 4 | 4745 | gene;island | 0.965927 | 0.821287 | Not queried | 1.64 | 5.71e-02 | 1.00e-01 |
|  | 2 | 136742688 | 136743046 | PDFSTATSENSEMBLUCSC | 359 | 4 | 22885 | gene;island | 0.003944 | 0.009624 | Not queried | 1.64 | 2.00e-01 | 2.55e-01 |
|  | 21 | 30671675 | 30671891 | PDFSTATSENSEMBLUCSC | 217 | 6 | 26395 | gene;island | 0.008422 | 0.012423 | Not queried | 1.64 | 1.80e-01 | 2.45e-01 |
|  | 2 | 108602784 | 108603244 | PDFSTATSENSEMBLUCSC | 461 | 9 | 24599 | island | 0.930502 | 0.961406 | Not queried | 1.64 | 4.00e-02 | 8.03e-02 |
|  | 16 | 69364538 | 69364643 | PDFSTATSENSEMBLUCSC | 106 | 4 | 15330 | tss | 0.002341 | 0.004238 | Not queried | 1.64 | 3.43e-01 | 3.84e-01 |
|  | 14 | 93813495 | 93813777 | PDFSTATSENSEMBLUCSC | 283 | 5 | 12190 | gene;island | 0.770486 | 0.968865 | Not queried | 1.64 | 6.90e-01 | 7.01e-01 |
|  | 2 | 154727907 | 154728468 | PDFSTATSENSEMBLUCSC | 562 | 8 | 24795 | island | 0.823022 | 0.951313 | Not queried | 1.64 | 3.28e-01 | 3.84e-01 |
|  | 20 | 36888790 | 36889389 | PDFSTATSENSEMBLUCSC | 600 | 10 | 26153 | island | 0.733754 | 0.923669 | Not queried | 1.64 | 6.30e-02 | 1.10e-01 |
|  | 3 | 197517855 | 197518017 | PDFSTATSENSEMBLUCSC | 163 | 8 | 28826 | tss | 0.007224 | 0.007621 | Not queried | 1.64 | 7.21e-01 | 7.30e-01 |
|  | 3 | 197517855 | 197518344 | PDFSTATSENSEMBLUCSC | 490 | 10 | 29822 | island | 0.005469 | 0.004183 | Not queried | 1.64 | 8.53e-01 | 8.60e-01 |
|  | 13 | 30424821 | 30424932 | PDFSTATSENSEMBLUCSC | 112 | 4 | 11372 | tss;island | 0.000375 | 0.001903 | Not queried | 1.64 | 2.00e-01 | 2.55e-01 |
|  | 17 | 46667587 | 46667812 | PDFSTATSENSEMBLUCSC | 226 | 6 | 17462 | tss;island | 0.981247 | 0.867884 | Not queried | 1.64 | 9.31e-02 | 1.53e-01 |
|  | 7 | 19147041 | 19147487 | PDFSTATSENSEMBLUCSC | 447 | 5 | 38282 | island | 0.659617 | 0.346310 | Not queried | 1.64 | 3.17e-02 | 6.50e-02 |
|  | 12 | 107348689 | 107349191 | PDFSTATSENSEMBLUCSC | 503 | 7 | 9971 | tss;island | 0.836930 | 0.978256 | Not queried | 1.63 | 5.35e-01 | 5.62e-01 |
|  | 17 | 17109651 | 17110353 | PDFSTATSENSEMBLUCSC | 703 | 12 | 17162 | tss | 0.035406 | 0.003688 | Not queried | 1.63 | 1.43e-01 | 2.04e-01 |
|  | 19 | 40909474 | 40909817 | PDFSTATSENSEMBLUCSC | 344 | 4 | 20041 | gene;island | 0.012946 | 0.012247 | Not queried | 1.63 | 6.86e-01 | 6.99e-01 |
|  | 19 | 47742558 | 47742961 | PDFSTATSENSEMBLUCSC | 404 | 4 | 22189 | island | 0.807753 | 0.582617 | Not queried | 1.63 | 2.86e-02 | 6.12e-02 |
|  | 20 | 2821390 | 2821485 | PDFSTATSENSEMBLUCSC | 96 | 4 | 25340 | gene | 0.008549 | 0.009776 | Not queried | 1.63 | 6.86e-01 | 6.99e-01 |
|  | 3 | 10547179 | 10547584 | PDFSTATSENSEMBLUCSC | 406 | 8 | 28878 | island | 0.235205 | 0.172759 | Not queried | 1.63 | 9.59e-01 | 9.62e-01 |
|  | 6 | 12290322 | 12290473 | PDFSTATSENSEMBLUCSC | 152 | 6 | 34640 | tss | 0.001273 | 0.002347 | Not queried | 1.63 | 1.80e-01 | 2.45e-01 |
|  | 3 | 50540137 | 50540261 | PDFSTATSENSEMBLUCSC | 125 | 4 | 27811 | gene;island | 0.228284 | 0.491507 | Not queried | 1.63 | 1.14e-01 | 1.68e-01 |
|  | 3 | 135684439 | 135684468 | PDFSTATSENSEMBLUCSC | 30 | 4 | 28609 | tss;island | 0.006119 | 0.010621 | Not queried | 1.63 | 3.43e-01 | 3.84e-01 |
|  | 10 | 134735893 | 134736248 | PDFSTATSENSEMBLUCSC | 356 | 5 | 6318 | island | 0.936916 | 0.956990 | Not queried | 1.63 | 3.10e-01 | 3.66e-01 |
|  | 6 | 36390970 | 36391494 | PDFSTATSENSEMBLUCSC | 525 | 6 | 34314 | gene;island | 0.003545 | 0.014626 | Not queried | 1.63 | 1.52e-02 | 5.36e-02 |
|  | 6 | 33047944 | 33048919 | PDFSTATSENSEMBLUCSC | 976 | 25 | 34186 | gene;island | 0.884545 | 0.955398 | HLA-DPB1; RPL32P1; HLA-DPA1 (margin: HLA-DPB1; RPL32P1; HLA-DPA1; HLA-DPA2) | 1.63 | 7.99e-03 | 3.21e-02 |
|  | 1 | 158083159 | 158083569 | PDFSTATSENSEMBLUCSC | 411 | 6 | 3876 | island | 0.319420 | 0.622104 | (margin: ) | 1.63 | 2.16e-03 | 1.45e-02 |
|  | 17 | 185102 | 185263 | PDFSTATSENSEMBLUCSC | 162 | 4 | 16292 | gene;island | 0.933582 | 0.978357 | Not queried | 1.63 | 2.00e-01 | 2.55e-01 |
|  | 1 | 153283628 | 153283994 | PDFSTATSENSEMBLUCSC | 367 | 4 | 1940 | tss | 0.812172 | 0.877291 | Not queried | 1.63 | 2.00e-01 | 2.55e-01 |
|  | 10 | 23481385 | 23481786 | PDFSTATSENSEMBLUCSC | 402 | 6 | 5610 | island | 0.924136 | 0.954931 | Not queried | 1.63 | 3.10e-01 | 3.66e-01 |
|  | 19 | 13030096 | 13030354 | PDFSTATSENSEMBLUCSC | 259 | 6 | 20519 | tss | 0.020367 | 0.005742 | Not queried | 1.63 | 1.32e-01 | 1.89e-01 |
|  | 8 | 1248861 | 1249215 | PDFSTATSENSEMBLUCSC | 355 | 5 | 40021 | island | 0.034663 | 0.217227 | Not queried | 1.63 | 3.10e-01 | 3.66e-01 |
|  | 7 | 34118464 | 34118935 | PDFSTATSENSEMBLUCSC | 472 | 5 | 36961 | gene;island | 0.499446 | 0.586992 | Not queried | 1.63 | 3.10e-01 | 3.66e-01 |
|  | 4 | 41361623 | 41362397 | PDFSTATSENSEMBLUCSC | 775 | 9 | 30363 | tss;island | 0.755138 | 0.911577 | Not queried | 1.63 | 2.58e-01 | 3.16e-01 |
|  | 19 | 56915403 | 56915879 | PDFSTATSENSEMBLUCSC | 477 | 9 | 20237 | gene;island | 0.832609 | 0.555784 | Not queried | 1.63 | 2.22e-01 | 2.76e-01 |
|  | 12 | 59313279 | 59313517 | PDFSTATSENSEMBLUCSC | 239 | 4 | 9198 | gene;island | 0.002437 | 0.006145 | Not queried | 1.63 | 1.14e-01 | 1.68e-01 |
|  | 12 | 56109593 | 56109799 | PDFSTATSENSEMBLUCSC | 207 | 5 | 9768 | tss | 0.462789 | 0.217896 | Not queried | 1.63 | 4.21e-01 | 4.59e-01 |
|  | 1 | 41978691 | 41979124 | PDFSTATSENSEMBLUCSC | 434 | 5 | 461 | gene;island | 0.918751 | 0.972216 | Not queried | 1.63 | 9.52e-02 | 1.53e-01 |
|  | 8 | 140643009 | 140643434 | PDFSTATSENSEMBLUCSC | 426 | 4 | 39435 | gene | 0.507397 | 0.312461 | Not queried | 1.63 | 3.43e-01 | 3.84e-01 |
|  | 7 | 100612725 | 100612937 | PDFSTATSENSEMBLUCSC | 213 | 5 | 38786 | island | 0.312677 | 0.601729 | Not queried | 1.63 | 9.52e-02 | 1.53e-01 |
|  | 8 | 52321814 | 52322341 | PDFSTATSENSEMBLUCSC | 528 | 7 | 39303 | gene;island | 0.973037 | 0.924023 | Not queried | 1.63 | 3.83e-01 | 4.27e-01 |
|  | 6 | 101846650 | 101847058 | PDFSTATSENSEMBLUCSC | 409 | 12 | 36370 | island | 0.920327 | 0.977213 | Not queried | 1.63 | 2.42e-02 | 6.12e-02 |
|  | 5 | 87963342 | 87963592 | PDFSTATSENSEMBLUCSC | 251 | 4 | 31676 | gene | 0.859457 | 0.904738 | Not queried | 1.63 | 6.86e-01 | 6.99e-01 |
|  | 20 | 17511826 | 17512294 | PDFSTATSENSEMBLUCSC | 469 | 5 | 25365 | gene;island | 0.248626 | 0.046561 | Not queried | 1.63 | 4.21e-01 | 4.59e-01 |
|  | 19 | 17970314 | 17971045 | PDFSTATSENSEMBLUCSC | 732 | 13 | 21758 | island | 0.002669 | 0.004271 | Not queried | 1.63 | 1.25e-01 | 1.84e-01 |
|  | 16 | 56701868 | 56702158 | PDFSTATSENSEMBLUCSC | 291 | 7 | 15954 | island | 0.718370 | 0.951389 | Not queried | 1.63 | 5.30e-02 | 1.00e-01 |
|  | 13 | 112720171 | 112720565 | PDFSTATSENSEMBLUCSC | 395 | 6 | 11909 | island | 0.849787 | 0.965045 | Not queried | 1.63 | 1.80e-01 | 2.45e-01 |
|  | 4 | 80329388 | 80329633 | PDFSTATSENSEMBLUCSC | 246 | 8 | 30457 | tss | 0.907463 | 0.973383 | Not queried | 1.63 | 1.30e-01 | 1.89e-01 |
|  | 11 | 71320513 | 71320766 | PDFSTATSENSEMBLUCSC | 254 | 4 | 8622 | island | 0.790808 | 0.897271 | Not queried | 1.62 | 4.86e-01 | 5.11e-01 |
|  | 7 | 148823215 | 148823355 | PDFSTATSENSEMBLUCSC | 141 | 4 | 37261 | gene;tss;island | 0.003645 | 0.006048 | Not queried | 1.62 | 8.86e-01 | 8.89e-01 |
|  | 5 | 140235435 | 140236081 | PDFSTATSENSEMBLUCSC | 647 | 9 | 31800 | gene;island | 0.725997 | 0.888589 | Not queried | 1.62 | 3.40e-01 | 3.84e-01 |
|  | 7 | 16684321 | 16684906 | PDFSTATSENSEMBLUCSC | 586 | 6 | 36904 | gene;tss;island | 0.002933 | 0.004498 | Not queried | 1.62 | 3.10e-01 | 3.66e-01 |
|  | 6 | 33256078 | 33258581 | PDFSTATSENSEMBLUCSC | 2504 | 57 | 34899 | tss;island | 0.007424 | 0.022272 | PFDN6; WDR46; RGL2 (margin: RPS18; B3GALT4; PFDN6; WDR46; RGL2; TAPBP) | 1.62 | 6.98e-03 | 3.19e-02 |
|  | 9 | 131965232 | 131965635 | PDFSTATSENSEMBLUCSC | 404 | 4 | 40957 | island | 0.022520 | 0.093826 | Not queried | 1.62 | 2.00e-01 | 2.55e-01 |
|  | 18 | 596703 | 596943 | PDFSTATSENSEMBLUCSC | 241 | 5 | 19152 | tss | 0.009763 | 0.006743 | Not queried | 1.62 | 6.90e-01 | 7.01e-01 |
|  | 3 | 30647378 | 30648098 | PDFSTATSENSEMBLUCSC | 721 | 13 | 28957 | island | 0.008056 | 0.014111 | Not queried | 1.62 | 9.08e-02 | 1.52e-01 |
|  | 3 | 30647378 | 30647988 | PDFSTATSENSEMBLUCSC | 611 | 11 | 28200 | tss | 0.011726 | 0.024285 | Not queried | 1.62 | 1.33e-01 | 1.91e-01 |
|  | 2 | 54557836 | 54557974 | PDFSTATSENSEMBLUCSC | 139 | 6 | 23355 | tss | 0.002200 | 0.008522 | Not queried | 1.62 | 2.40e-01 | 2.95e-01 |
|  | 6 | 8436218 | 8436466 | PDFSTATSENSEMBLUCSC | 249 | 4 | 34621 | tss;island | 0.073938 | 0.018387 | Not queried | 1.62 | 3.43e-01 | 3.84e-01 |
|  | 4 | 6988644 | 6988785 | PDFSTATSENSEMBLUCSC | 142 | 5 | 29930 | gene;tss;island | 0.005919 | 0.015420 | Not queried | 1.62 | 3.10e-01 | 3.66e-01 |
|  | 1 | 64058883 | 64059052 | PDFSTATSENSEMBLUCSC | 170 | 4 | 3346 | island | 0.006242 | 0.033919 | Not queried | 1.62 | 4.86e-01 | 5.11e-01 |
|  | 11 | 16632311 | 16632724 | PDFSTATSENSEMBLUCSC | 414 | 4 | 8091 | island | 0.790176 | 0.920778 | Not queried | 1.62 | 8.86e-01 | 8.89e-01 |
|  | 2 | 133014640 | 133014805 | PDFSTATSENSEMBLUCSC | 166 | 7 | 22874 | gene;island | 0.725325 | 0.868615 | Not queried | 1.62 | 9.73e-02 | 1.55e-01 |
|  | 22 | 36648692 | 36649077 | PDFSTATSENSEMBLUCSC | 386 | 4 | 27106 | tss | 0.558519 | 0.920027 | Not queried | 1.62 | 4.86e-01 | 5.11e-01 |
|  | 12 | 123459803 | 123459832 | PDFSTATSENSEMBLUCSC | 30 | 4 | 10094 | tss | 0.004068 | 0.010397 | Not queried | 1.62 | 2.00e-01 | 2.55e-01 |
|  | 5 | 93953866 | 93954303 | PDFSTATSENSEMBLUCSC | 438 | 9 | 31694 | gene | 0.001493 | 0.005937 | Not queried | 1.62 | 3.87e-01 | 4.30e-01 |
|  | 2 | 127864762 | 127864964 | PDFSTATSENSEMBLUCSC | 203 | 5 | 24685 | island | 0.007317 | 0.016834 | Not queried | 1.62 | 4.21e-01 | 4.59e-01 |
|  | 1 | 111217691 | 111217785 | PDFSTATSENSEMBLUCSC | 95 | 4 | 1818 | tss | 0.904995 | 0.958788 | Not queried | 1.62 | 1.14e-01 | 1.68e-01 |
|  | 7 | 105752848 | 105752987 | PDFSTATSENSEMBLUCSC | 140 | 6 | 37178 | gene | 0.002490 | 0.005305 | Not queried | 1.62 | 4.85e-01 | 5.11e-01 |
|  | 6 | 32143923 | 32144245 | PDFSTATSENSEMBLUCSC | 323 | 4 | 34867 | tss | 0.017715 | 0.072473 | Not queried | 1.62 | 2.00e-01 | 2.55e-01 |
|  | 6 | 112408804 | 112409181 | PDFSTATSENSEMBLUCSC | 378 | 10 | 35182 | tss | 0.010747 | 0.005204 | Not queried | 1.62 | 2.80e-01 | 3.40e-01 |
|  | 14 | 51706677 | 51706820 | PDFSTATSENSEMBLUCSC | 144 | 4 | 12392 | tss | 0.003025 | 0.003723 | Not queried | 1.62 | 4.86e-01 | 5.11e-01 |
|  | 2 | 3691145 | 3691651 | PDFSTATSENSEMBLUCSC | 507 | 6 | 22556 | gene;island | 0.585235 | 0.931855 | Not queried | 1.62 | 1.80e-01 | 2.45e-01 |
|  | 2 | 196933518 | 196933901 | PDFSTATSENSEMBLUCSC | 384 | 9 | 24973 | island | 0.080816 | 0.017889 | Not queried | 1.62 | 1.36e-01 | 1.94e-01 |
|  | 18 | 19284713 | 19285051 | PDFSTATSENSEMBLUCSC | 339 | 10 | 19419 | island | 0.005867 | 0.007607 | Not queried | 1.62 | 4.36e-01 | 4.76e-01 |
|  | 3 | 154797508 | 154798079 | PDFSTATSENSEMBLUCSC | 572 | 12 | 28003 | gene | 0.906019 | 0.978051 | Not queried | 1.62 | 2.42e-02 | 6.12e-02 |
|  | 19 | 45445437 | 45445491 | PDFSTATSENSEMBLUCSC | 55 | 4 | 20857 | tss | 0.585149 | 0.855559 | Not queried | 1.62 | 5.71e-02 | 1.00e-01 |
|  | 1 | 149871404 | 149871658 | PDFSTATSENSEMBLUCSC | 255 | 4 | 734 | gene;island | 0.130514 | 0.023900 | Not queried | 1.62 | 4.86e-01 | 5.11e-01 |
|  | 20 | 62738197 | 62738358 | PDFSTATSENSEMBLUCSC | 162 | 5 | 25914 | tss | 0.475034 | 0.767220 | Not queried | 1.62 | 5.56e-02 | 1.00e-01 |
|  | 8 | 72987744 | 72988022 | PDFSTATSENSEMBLUCSC | 279 | 10 | 40385 | island | 0.738809 | 0.545789 | Not queried | 1.62 | 1.90e-01 | 2.55e-01 |
|  | 1 | 203734396 | 203734559 | PDFSTATSENSEMBLUCSC | 164 | 4 | 954 | gene | 0.908527 | 0.590042 | Not queried | 1.62 | 4.86e-01 | 5.11e-01 |
|  | 4 | 100574455 | 100574653 | PDFSTATSENSEMBLUCSC | 199 | 4 | 31199 | island | 0.856463 | 0.979401 | Not queried | 1.62 | 6.86e-01 | 6.99e-01 |
|  | 4 | 143767608 | 143767672 | PDFSTATSENSEMBLUCSC | 65 | 6 | 30612 | tss | 0.028565 | 0.006249 | Not queried | 1.62 | 2.40e-01 | 2.95e-01 |
|  | 21 | 15038314 | 15038699 | PDFSTATSENSEMBLUCSC | 386 | 4 | 26605 | island | 0.659107 | 0.867487 | Not queried | 1.62 | 3.43e-01 | 3.84e-01 |
|  | 22 | 32341191 | 32341780 | PDFSTATSENSEMBLUCSC | 590 | 11 | 26844 | gene;island | 0.004487 | 0.005639 | Not queried | 1.62 | 2.43e-01 | 2.98e-01 |
|  | 6 | 30231639 | 30232015 | PDFSTATSENSEMBLUCSC | 377 | 5 | 33755 | gene;island | 0.207680 | 0.573170 | Not queried | 1.62 | 2.22e-01 | 2.76e-01 |
|  | 10 | 52499393 | 52500187 | PDFSTATSENSEMBLUCSC | 795 | 9 | 5745 | island | 0.035271 | 0.070728 | Not queried | 1.62 | 3.87e-01 | 4.30e-01 |
|  | 11 | 64051699 | 64051814 | PDFSTATSENSEMBLUCSC | 116 | 6 | 6673 | gene;tss;island | 0.254871 | 0.105239 | Not queried | 1.62 | 3.10e-01 | 3.66e-01 |
|  | 19 | 45873596 | 45873826 | PDFSTATSENSEMBLUCSC | 231 | 4 | 20093 | gene | 0.001199 | 0.003757 | Not queried | 1.62 | 3.43e-01 | 3.84e-01 |
|  | 12 | 5603131 | 5603261 | PDFSTATSENSEMBLUCSC | 131 | 6 | 9019 | gene;tss;island | 0.665523 | 0.859521 | Not queried | 1.62 | 2.40e-01 | 2.95e-01 |
|  | 3 | 64008736 | 64008893 | PDFSTATSENSEMBLUCSC | 158 | 4 | 27848 | gene;island | 0.016682 | 0.078549 | Not queried | 1.62 | 2.00e-01 | 2.55e-01 |
|  | 1 | 2716877 | 2717193 | PDFSTATSENSEMBLUCSC | 317 | 4 | 2517 | island | 0.778466 | 0.911378 | Not queried | 1.62 | 2.00e-01 | 2.55e-01 |
|  | 20 | 52824106 | 52824603 | PDFSTATSENSEMBLUCSC | 498 | 9 | 26266 | island | 0.015431 | 0.035353 | Not queried | 1.62 | 2.97e-01 | 3.60e-01 |
|  | 21 | 36041334 | 36041683 | PDFSTATSENSEMBLUCSC | 350 | 7 | 26531 | tss | 0.877198 | 0.974724 | Not queried | 1.62 | 7.28e-02 | 1.23e-01 |
|  | 1 | 145507220 | 145507616 | PDFSTATSENSEMBLUCSC | 397 | 10 | 1886 | tss | 0.006992 | 0.010434 | Not queried | 1.62 | 3.15e-01 | 3.72e-01 |
|  | 21 | 45811363 | 45811549 | PDFSTATSENSEMBLUCSC | 187 | 4 | 26456 | gene;island | 0.881607 | 0.964534 | Not queried | 1.62 | 5.71e-02 | 1.00e-01 |
|  | 1 | 173639044 | 173639135 | PDFSTATSENSEMBLUCSC | 92 | 4 | 2097 | tss;island | 0.925571 | 0.981695 | Not queried | 1.62 | 1.14e-01 | 1.68e-01 |
|  | 19 | 35630106 | 35630474 | PDFSTATSENSEMBLUCSC | 369 | 7 | 19980 | gene;island | 0.744149 | 0.865645 | Not queried | 1.61 | 9.73e-02 | 1.55e-01 |
|  | 12 | 75603068 | 75603748 | PDFSTATSENSEMBLUCSC | 681 | 7 | 10632 | island | 0.756785 | 0.544338 | Not queried | 1.61 | 2.09e-01 | 2.65e-01 |
|  | 12 | 75603068 | 75603480 | PDFSTATSENSEMBLUCSC | 413 | 5 | 9220 | gene | 0.887266 | 0.544338 | Not queried | 1.61 | 3.10e-01 | 3.66e-01 |
|  | 16 | 29796620 | 29796798 | PDFSTATSENSEMBLUCSC | 179 | 4 | 15805 | island | 0.961605 | 0.988781 | Not queried | 1.61 | 5.71e-02 | 1.00e-01 |
|  | 10 | 21814313 | 21814538 | PDFSTATSENSEMBLUCSC | 226 | 4 | 4528 | gene | 0.043120 | 0.015232 | Not queried | 1.61 | 2.00e-01 | 2.55e-01 |
|  | 19 | 41858816 | 41859304 | PDFSTATSENSEMBLUCSC | 489 | 4 | 20057 | gene;island | 0.003134 | 0.007162 | Not queried | 1.61 | 4.86e-01 | 5.11e-01 |
|  | 15 | 37179603 | 37179779 | PDFSTATSENSEMBLUCSC | 177 | 4 | 13569 | tss;island | 0.114585 | 0.270143 | Not queried | 1.61 | 3.43e-01 | 3.84e-01 |
|  | 6 | 27100831 | 27101279 | PDFSTATSENSEMBLUCSC | 449 | 5 | 33616 | gene | 0.000640 | 0.002535 | Not queried | 1.61 | 4.21e-01 | 4.59e-01 |
|  | 1 | 65468273 | 65468921 | PDFSTATSENSEMBLUCSC | 649 | 7 | 3350 | island | 0.008810 | 0.025593 | Not queried | 1.61 | 3.83e-01 | 4.27e-01 |
|  | 4 | 646293 | 646577 | PDFSTATSENSEMBLUCSC | 285 | 5 | 29829 | gene;tss;island | 0.954717 | 0.981895 | Not queried | 1.61 | 4.21e-01 | 4.59e-01 |
|  | 17 | 1587547 | 1588142 | PDFSTATSENSEMBLUCSC | 596 | 6 | 16317 | gene | 0.005718 | 0.008800 | Not queried | 1.61 | 3.94e-01 | 4.36e-01 |
|  | 13 | 26828738 | 26828880 | PDFSTATSENSEMBLUCSC | 143 | 4 | 11620 | island | 0.001787 | 0.005319 | Not queried | 1.61 | 2.00e-01 | 2.55e-01 |
|  | 6 | 1384491 | 1384780 | PDFSTATSENSEMBLUCSC | 290 | 4 | 35366 | island | 0.986481 | 0.956699 | Not queried | 1.61 | 3.43e-01 | 3.84e-01 |
|  | 3 | 45077649 | 45078075 | PDFSTATSENSEMBLUCSC | 427 | 6 | 29046 | island | 0.889184 | 0.969490 | Not queried | 1.61 | 2.40e-01 | 2.95e-01 |
|  | 17 | 47287410 | 47287577 | PDFSTATSENSEMBLUCSC | 168 | 6 | 17483 | tss | 0.757309 | 0.935892 | Not queried | 1.61 | 1.32e-01 | 1.89e-01 |
|  | 11 | 17498236 | 17498619 | PDFSTATSENSEMBLUCSC | 384 | 9 | 8100 | island | 0.668127 | 0.601743 | Not queried | 1.61 | 3.87e-01 | 4.30e-01 |
|  | 21 | 15755931 | 15756122 | PDFSTATSENSEMBLUCSC | 192 | 5 | 26489 | tss;island | 0.002073 | 0.000982 | Not queried | 1.61 | 4.21e-01 | 4.59e-01 |
|  | 3 | 50382952 | 50383485 | PDFSTATSENSEMBLUCSC | 534 | 11 | 29154 | island | 0.315713 | 0.522278 | ZMYND10-AS1; ZMYND10; NPRL2 (margin: ZMYND10-AS1; CYB561D2; XXcos-LUCA11.5; RASSF1; ZMYND10; NPRL2; TMEM115) | 1.61 | 3.18e-03 | 2.01e-02 |
|  | 11 | 119293601 | 119293869 | PDFSTATSENSEMBLUCSC | 269 | 5 | 6941 | gene;island | 0.904420 | 0.972402 | Not queried | 1.61 | 1.51e-01 | 2.11e-01 |
|  | 14 | 101175970 | 101176310 | PDFSTATSENSEMBLUCSC | 341 | 4 | 13141 | island | 0.795459 | 0.949367 | Not queried | 1.61 | 2.00e-01 | 2.55e-01 |
|  | 4 | 46911291 | 46911550 | PDFSTATSENSEMBLUCSC | 260 | 5 | 30374 | tss | 0.445488 | 0.721015 | Not queried | 1.61 | 6.90e-01 | 7.01e-01 |
|  | 7 | 114055074 | 114055210 | PDFSTATSENSEMBLUCSC | 137 | 6 | 37192 | gene | 0.046276 | 0.117392 | Not queried | 1.61 | 6.49e-02 | 1.11e-01 |
|  | 8 | 55533655 | 55534103 | PDFSTATSENSEMBLUCSC | 449 | 7 | 39306 | gene;island | 0.890082 | 0.668262 | Not queried | 1.61 | 3.18e-01 | 3.74e-01 |
|  | 12 | 132990805 | 132991277 | PDFSTATSENSEMBLUCSC | 473 | 5 | 11051 | island | 0.109672 | 0.484552 | Not queried | 1.61 | 5.48e-01 | 5.70e-01 |
|  | 5 | 2755820 | 2756113 | PDFSTATSENSEMBLUCSC | 294 | 4 | 32659 | island | 0.891045 | 0.967795 | Not queried | 1.61 | 5.71e-02 | 1.00e-01 |
|  | 13 | 28674876 | 28675056 | PDFSTATSENSEMBLUCSC | 181 | 4 | 11368 | tss | 0.400694 | 0.206764 | Not queried | 1.61 | 2.00e-01 | 2.55e-01 |
|  | 1 | 230777685 | 230777875 | PDFSTATSENSEMBLUCSC | 191 | 4 | 2316 | tss;island | 0.940459 | 0.684861 | Not queried | 1.61 | 4.86e-01 | 5.11e-01 |
|  | 6 | 31543169 | 31543300 | PDFSTATSENSEMBLUCSC | 132 | 4 | 34819 | tss | 0.609738 | 0.819136 | Not queried | 1.61 | 2.00e-01 | 2.55e-01 |
|  | 5 | 138211071 | 138211184 | PDFSTATSENSEMBLUCSC | 114 | 5 | 32326 | tss | 0.908848 | 0.969303 | Not queried | 1.61 | 3.10e-01 | 3.66e-01 |
|  | 10 | 102996197 | 102996571 | PDFSTATSENSEMBLUCSC | 375 | 6 | 4727 | gene;island | 0.908782 | 0.951796 | Not queried | 1.61 | 2.40e-01 | 2.95e-01 |
|  | 12 | 20521087 | 20522177 | PDFSTATSENSEMBLUCSC | 1091 | 10 | 9597 | tss | 0.465529 | 0.200070 | Not queried | 1.61 | 1.43e-01 | 2.04e-01 |
|  | 5 | 162887193 | 162887539 | PDFSTATSENSEMBLUCSC | 347 | 12 | 32475 | tss;island | 0.002419 | 0.005932 | Not queried | 1.61 | 2.42e-01 | 2.96e-01 |
|  | 11 | 67407037 | 67407224 | PDFSTATSENSEMBLUCSC | 188 | 5 | 7499 | tss | 0.769044 | 0.910449 | Not queried | 1.61 | 5.56e-02 | 1.00e-01 |
|  | 13 | 20735236 | 20735532 | PDFSTATSENSEMBLUCSC | 297 | 7 | 11327 | tss | 0.019509 | 0.045742 | Not queried | 1.61 | 2.09e-01 | 2.65e-01 |
|  | 16 | 1587610 | 1587842 | PDFSTATSENSEMBLUCSC | 233 | 4 | 14575 | gene;island | 0.901729 | 0.969131 | Not queried | 1.61 | 4.86e-01 | 5.11e-01 |
|  | 6 | 31914952 | 31915198 | PDFSTATSENSEMBLUCSC | 247 | 8 | 33996 | gene | 0.978659 | 0.934543 | C2; CFB (margin: C2; CFB; SKIV2L; NELFE; MIR1236) | 1.61 | 1.04e-02 | 3.96e-02 |
|  | 10 | 21783344 | 21783930 | PDFSTATSENSEMBLUCSC | 587 | 6 | 5589 | island | 0.790568 | 0.982148 | Not queried | 1.61 | 3.10e-01 | 3.66e-01 |
|  | 6 | 137366125 | 137366545 | PDFSTATSENSEMBLUCSC | 421 | 8 | 36506 | island | 0.946474 | 0.867210 | Not queried | 1.60 | 8.30e-02 | 1.40e-01 |
|  | 7 | 96651915 | 96652481 | PDFSTATSENSEMBLUCSC | 567 | 10 | 37106 | gene;island | 0.680481 | 0.910360 | Not queried | 1.60 | 5.29e-01 | 5.56e-01 |
|  | 2 | 119916431 | 119916686 | PDFSTATSENSEMBLUCSC | 256 | 7 | 24651 | island | 0.930728 | 0.975997 | Not queried | 1.60 | 9.73e-02 | 1.55e-01 |
|  | 11 | 57424284 | 57424534 | PDFSTATSENSEMBLUCSC | 251 | 4 | 7305 | tss;island | 0.669674 | 0.636111 | Not queried | 1.60 | 6.86e-01 | 6.99e-01 |
|  | 5 | 523430 | 523794 | PDFSTATSENSEMBLUCSC | 365 | 4 | 31511 | gene;island | 0.422008 | 0.048469 | Not queried | 1.60 | 6.86e-01 | 6.99e-01 |
|  | 19 | 45281006 | 45281287 | PDFSTATSENSEMBLUCSC | 282 | 5 | 22113 | island | 0.215214 | 0.386964 | Not queried | 1.60 | 5.56e-02 | 1.00e-01 |
|  | 7 | 89874187 | 89874597 | PDFSTATSENSEMBLUCSC | 411 | 8 | 38647 | island | 0.007243 | 0.018876 | Not queried | 1.60 | 3.82e-01 | 4.27e-01 |
|  | 11 | 65601167 | 65601385 | PDFSTATSENSEMBLUCSC | 219 | 8 | 7443 | tss | 0.837246 | 0.964857 | Not queried | 1.60 | 3.79e-02 | 7.66e-02 |
|  | 4 | 3526676 | 3526865 | PDFSTATSENSEMBLUCSC | 190 | 4 | 29912 | gene;island | 0.979294 | 0.921618 | Not queried | 1.60 | 2.00e-01 | 2.55e-01 |
|  | 14 | 65380577 | 65381118 | PDFSTATSENSEMBLUCSC | 542 | 9 | 12463 | tss | 0.004350 | 0.006216 | Not queried | 1.60 | 5.46e-01 | 5.70e-01 |
|  | 16 | 30710278 | 30710486 | PDFSTATSENSEMBLUCSC | 209 | 4 | 15844 | island | 0.002060 | 0.003224 | Not queried | 1.60 | 4.86e-01 | 5.11e-01 |
|  | 1 | 203242409 | 203242638 | PDFSTATSENSEMBLUCSC | 230 | 4 | 4101 | island | 0.102262 | 0.282690 | Not queried | 1.60 | 3.43e-01 | 3.84e-01 |
|  | 2 | 27665017 | 27665150 | PDFSTATSENSEMBLUCSC | 134 | 6 | 23270 | tss | 0.150152 | 0.053680 | Not queried | 1.60 | 4.85e-01 | 5.11e-01 |
|  | 7 | 35293753 | 35293892 | PDFSTATSENSEMBLUCSC | 140 | 5 | 37535 | tss;island | 0.718913 | 0.927380 | Not queried | 1.60 | 3.10e-01 | 3.66e-01 |
|  | 12 | 57881856 | 57882021 | PDFSTATSENSEMBLUCSC | 166 | 4 | 9193 | gene | 0.005193 | 0.006253 | Not queried | 1.60 | 4.86e-01 | 5.11e-01 |
|  | 17 | 7982510 | 7983254 | PDFSTATSENSEMBLUCSC | 745 | 9 | 16424 | gene;island | 0.683543 | 0.834364 | Not queried | 1.60 | 3.40e-01 | 3.84e-01 |
|  | 6 | 32798417 | 32798510 | PDFSTATSENSEMBLUCSC | 94 | 4 | 34140 | gene | 0.979595 | 0.886643 | Not queried | 1.60 | 3.43e-01 | 3.84e-01 |
|  | 7 | 87849017 | 87849201 | PDFSTATSENSEMBLUCSC | 185 | 4 | 37077 | gene;island | 0.004722 | 0.024824 | Not queried | 1.60 | 6.86e-01 | 6.99e-01 |
|  | 7 | 155915373 | 155915712 | PDFSTATSENSEMBLUCSC | 340 | 4 | 39082 | island | 0.517280 | 0.729745 | Not queried | 1.60 | 4.86e-01 | 5.11e-01 |
|  | 19 | 984141 | 984170 | PDFSTATSENSEMBLUCSC | 30 | 4 | 20287 | tss;island | 0.002313 | 0.005772 | Not queried | 1.60 | 3.43e-01 | 3.84e-01 |
|  | 17 | 33288564 | 33288597 | PDFSTATSENSEMBLUCSC | 34 | 4 | 16518 | gene | 0.002158 | 0.003328 | Not queried | 1.60 | 4.86e-01 | 5.11e-01 |
|  | 10 | 37414506 | 37414802 | PDFSTATSENSEMBLUCSC | 297 | 7 | 5668 | island | 0.936628 | 0.966178 | Not queried | 1.60 | 7.28e-02 | 1.23e-01 |
|  | 8 | 141599141 | 141599436 | PDFSTATSENSEMBLUCSC | 296 | 5 | 39443 | gene | 0.229456 | 0.385626 | Not queried | 1.60 | 5.48e-01 | 5.70e-01 |
|  | 10 | 72238384 | 72238844 | PDFSTATSENSEMBLUCSC | 461 | 7 | 5820 | island | 0.723386 | 0.464238 | Not queried | 1.60 | 2.62e-02 | 6.12e-02 |
|  | 10 | 100028236 | 100028499 | PDFSTATSENSEMBLUCSC | 264 | 4 | 5276 | tss;island | 0.804311 | 0.920871 | Not queried | 1.60 | 2.00e-01 | 2.55e-01 |
|  | 10 | 102505266 | 102505483 | PDFSTATSENSEMBLUCSC | 218 | 7 | 6034 | island | 0.052217 | 0.017147 | Not queried | 1.60 | 1.28e-01 | 1.87e-01 |
|  | 10 | 88022641 | 88023243 | PDFSTATSENSEMBLUCSC | 603 | 7 | 4658 | gene;island | 0.868978 | 0.975621 | Not queried | 1.60 | 1.65e-01 | 2.28e-01 |
|  | 17 | 18908220 | 18908324 | PDFSTATSENSEMBLUCSC | 105 | 4 | 16477 | gene;tss;island | 0.001957 | 0.004864 | Not queried | 1.60 | 4.86e-01 | 5.11e-01 |
|  | 1 | 17247530 | 17248100 | PDFSTATSENSEMBLUCSC | 571 | 5 | 1287 | tss | 0.831983 | 0.937921 | Not queried | 1.60 | 2.22e-01 | 2.76e-01 |
|  | 7 | 129422915 | 129423354 | PDFSTATSENSEMBLUCSC | 440 | 4 | 38912 | island | 0.881655 | 0.955960 | Not queried | 1.60 | 1.14e-01 | 1.68e-01 |
|  | 17 | 57232291 | 57232584 | PDFSTATSENSEMBLUCSC | 294 | 5 | 16712 | gene;tss;island | 0.001696 | 0.008953 | Not queried | 1.60 | 5.48e-01 | 5.70e-01 |
|  | 16 | 14397576 | 14397892 | PDFSTATSENSEMBLUCSC | 317 | 6 | 15721 | island | 0.005521 | 0.016117 | Not queried | 1.60 | 1.32e-01 | 1.89e-01 |
|  | 4 | 77172861 | 77173197 | PDFSTATSENSEMBLUCSC | 337 | 4 | 30027 | gene | 0.303367 | 0.035031 | Not queried | 1.60 | 2.00e-01 | 2.55e-01 |
|  | 17 | 76227227 | 76227386 | PDFSTATSENSEMBLUCSC | 160 | 4 | 17702 | tss | 0.611273 | 0.844745 | Not queried | 1.60 | 3.43e-01 | 3.84e-01 |
|  | 19 | 11564442 | 11564539 | PDFSTATSENSEMBLUCSC | 98 | 4 | 19842 | gene;island | 0.806880 | 0.935482 | Not queried | 1.60 | 3.43e-01 | 3.84e-01 |
|  | 13 | 99174341 | 99174609 | PDFSTATSENSEMBLUCSC | 269 | 5 | 11509 | tss | 0.878742 | 0.983557 | Not queried | 1.60 | 1.51e-01 | 2.11e-01 |
|  | 5 | 151304312 | 151304559 | PDFSTATSENSEMBLUCSC | 248 | 8 | 33323 | island | 0.573609 | 0.145937 | Not queried | 1.60 | 2.34e-01 | 2.90e-01 |
|  | 1 | 204042123 | 204042338 | PDFSTATSENSEMBLUCSC | 216 | 4 | 4112 | island | 0.007324 | 0.019533 | Not queried | 1.60 | 3.43e-01 | 3.84e-01 |
|  | 21 | 35831871 | 35832364 | PDFSTATSENSEMBLUCSC | 494 | 10 | 26410 | gene;island | 0.954807 | 0.978775 | Not queried | 1.60 | 3.55e-02 | 7.24e-02 |
|  | 21 | 36041334 | 36041699 | PDFSTATSENSEMBLUCSC | 366 | 8 | 26662 | island | 0.885115 | 0.968745 | Not queried | 1.60 | 3.79e-02 | 7.66e-02 |
|  | 8 | 23563859 | 23564294 | PDFSTATSENSEMBLUCSC | 436 | 9 | 40156 | island | 0.954125 | 0.982543 | Not queried | 1.60 | 1.42e-02 | 5.24e-02 |
|  | 5 | 176810904 | 176811305 | PDFSTATSENSEMBLUCSC | 402 | 6 | 32526 | tss | 0.572733 | 0.803830 | Not queried | 1.60 | 2.40e-01 | 2.95e-01 |
|  | 17 | 80408768 | 80408928 | PDFSTATSENSEMBLUCSC | 161 | 7 | 17775 | tss;island | 0.003421 | 0.004973 | Not queried | 1.60 | 3.83e-01 | 4.27e-01 |
|  | 1 | 27319836 | 27320132 | PDFSTATSENSEMBLUCSC | 297 | 6 | 1403 | tss | 0.005797 | 0.003510 | Not queried | 1.60 | 4.85e-01 | 5.11e-01 |
|  | 14 | 77228508 | 77228753 | PDFSTATSENSEMBLUCSC | 246 | 4 | 12149 | gene;island | 0.531719 | 0.688989 | Not queried | 1.60 | 6.86e-01 | 6.99e-01 |
|  | 20 | 33104052 | 33104198 | PDFSTATSENSEMBLUCSC | 147 | 5 | 25692 | tss | 0.001670 | 0.004228 | Not queried | 1.60 | 2.22e-01 | 2.76e-01 |
|  | 16 | 31724470 | 31724534 | PDFSTATSENSEMBLUCSC | 65 | 4 | 15216 | tss | 0.005586 | 0.017026 | Not queried | 1.59 | 1.14e-01 | 1.68e-01 |
|  | 2 | 241974858 | 241975140 | PDFSTATSENSEMBLUCSC | 283 | 4 | 23127 | gene;island | 0.917501 | 0.971122 | Not queried | 1.59 | 1.14e-01 | 1.68e-01 |
|  | 2 | 145281942 | 145282262 | PDFSTATSENSEMBLUCSC | 321 | 4 | 24771 | island | 0.480507 | 0.777666 | Not queried | 1.59 | 6.86e-01 | 6.99e-01 |
|  | 19 | 58220295 | 58220516 | PDFSTATSENSEMBLUCSC | 222 | 4 | 20249 | gene | 0.951825 | 0.984203 | Not queried | 1.59 | 2.86e-02 | 6.12e-02 |
|  | 12 | 95397187 | 95397489 | PDFSTATSENSEMBLUCSC | 303 | 5 | 9251 | gene | 0.003323 | 0.011292 | Not queried | 1.59 | 4.21e-01 | 4.59e-01 |
|  | 16 | 55542668 | 55542751 | PDFSTATSENSEMBLUCSC | 84 | 4 | 15240 | tss | 0.927359 | 0.822116 | Not queried | 1.59 | 1.14e-01 | 1.68e-01 |
|  | 19 | 53073577 | 53074012 | PDFSTATSENSEMBLUCSC | 436 | 4 | 20197 | gene | 0.629361 | 0.387764 | Not queried | 1.59 | 5.71e-02 | 1.00e-01 |
|  | 2 | 60781524 | 60781869 | PDFSTATSENSEMBLUCSC | 346 | 5 | 23371 | tss;island | 0.063162 | 0.019348 | Not queried | 1.59 | 3.17e-02 | 6.50e-02 |
|  | 6 | 127835990 | 127836159 | PDFSTATSENSEMBLUCSC | 170 | 4 | 34471 | gene;island | 0.906798 | 0.970095 | Not queried | 1.59 | 2.00e-01 | 2.55e-01 |
|  | 16 | 88569083 | 88569490 | PDFSTATSENSEMBLUCSC | 408 | 5 | 14906 | gene;island | 0.797040 | 0.914454 | Not queried | 1.59 | 1.51e-01 | 2.11e-01 |
|  | 3 | 147131069 | 147131332 | PDFSTATSENSEMBLUCSC | 264 | 4 | 27990 | gene;island | 0.868093 | 0.948973 | Not queried | 1.59 | 1.14e-01 | 1.68e-01 |
|  | 19 | 39694477 | 39694831 | PDFSTATSENSEMBLUCSC | 355 | 4 | 20026 | gene | 0.948327 | 0.981935 | Not queried | 1.59 | 3.43e-01 | 3.84e-01 |
|  | 22 | 31608022 | 31608245 | PDFSTATSENSEMBLUCSC | 224 | 4 | 27077 | tss | 0.009695 | 0.017853 | Not queried | 1.59 | 3.43e-01 | 3.84e-01 |
|  | 12 | 52281810 | 52282079 | PDFSTATSENSEMBLUCSC | 270 | 4 | 9133 | gene | 0.868290 | 0.944286 | Not queried | 1.59 | 2.00e-01 | 2.55e-01 |
|  | 22 | 38054505 | 38054892 | PDFSTATSENSEMBLUCSC | 388 | 6 | 27466 | island | 0.010105 | 0.010233 | Not queried | 1.59 | 3.10e-01 | 3.66e-01 |
|  | 1 | 75596724 | 75596930 | PDFSTATSENSEMBLUCSC | 207 | 4 | 587 | gene;island | 0.642953 | 0.846285 | Not queried | 1.59 | 4.86e-01 | 5.11e-01 |
|  | 20 | 44657637 | 44657948 | PDFSTATSENSEMBLUCSC | 312 | 7 | 25438 | gene;island | 0.929185 | 0.972639 | Not queried | 1.59 | 1.65e-01 | 2.28e-01 |
|  | 14 | 75330608 | 75330798 | PDFSTATSENSEMBLUCSC | 191 | 4 | 12511 | tss | 0.722437 | 0.856764 | Not queried | 1.59 | 2.86e-02 | 6.12e-02 |
|  | 6 | 64281439 | 64281996 | PDFSTATSENSEMBLUCSC | 558 | 9 | 36230 | island | 0.002048 | 0.023333 | Not queried | 1.59 | 1.13e-01 | 1.68e-01 |
|  | 11 | 57414401 | 57414626 | PDFSTATSENSEMBLUCSC | 226 | 4 | 6615 | gene;island | 0.706324 | 0.787103 | Not queried | 1.59 | 4.86e-01 | 5.11e-01 |
|  | 22 | 36635583 | 36635882 | PDFSTATSENSEMBLUCSC | 300 | 5 | 26855 | gene | 0.022905 | 0.065519 | Not queried | 1.59 | 5.56e-02 | 1.00e-01 |
|  | 22 | 37678548 | 37678791 | PDFSTATSENSEMBLUCSC | 244 | 4 | 26861 | gene | 0.420698 | 0.574974 | Not queried | 1.59 | 8.86e-01 | 8.89e-01 |
|  | 14 | 81421297 | 81421770 | PDFSTATSENSEMBLUCSC | 474 | 7 | 12539 | tss;island | 0.182753 | 0.023289 | Not queried | 1.59 | 8.05e-01 | 8.14e-01 |
|  | 11 | 64546243 | 64546534 | PDFSTATSENSEMBLUCSC | 292 | 8 | 7403 | tss | 0.005384 | 0.002474 | Not queried | 1.59 | 3.82e-01 | 4.27e-01 |
|  | 2 | 177021702 | 177022285 | PDFSTATSENSEMBLUCSC | 584 | 7 | 24910 | island | 0.904429 | 0.941987 | Not queried | 1.59 | 2.59e-01 | 3.16e-01 |
|  | 5 | 140719008 | 140719302 | PDFSTATSENSEMBLUCSC | 295 | 4 | 31817 | gene;island | 0.946606 | 0.984193 | Not queried | 1.59 | 2.00e-01 | 2.55e-01 |
|  | 11 | 75525637 | 75526167 | PDFSTATSENSEMBLUCSC | 531 | 9 | 7572 | tss | 0.002498 | 0.016255 | Not queried | 1.59 | 2.22e-01 | 2.76e-01 |
|  | 5 | 125936116 | 125936553 | PDFSTATSENSEMBLUCSC | 438 | 6 | 32252 | tss | 0.199457 | 0.061146 | Not queried | 1.59 | 5.89e-01 | 6.11e-01 |
|  | 11 | 65409760 | 65410126 | PDFSTATSENSEMBLUCSC | 367 | 4 | 6715 | gene;island | 0.981476 | 0.931660 | Not queried | 1.59 | 5.71e-02 | 1.00e-01 |
|  | 1 | 45205136 | 45205518 | PDFSTATSENSEMBLUCSC | 383 | 9 | 3187 | island | 0.002886 | 0.003194 | Not queried | 1.59 | 6.66e-01 | 6.91e-01 |
|  | 17 | 56064295 | 56064928 | PDFSTATSENSEMBLUCSC | 634 | 7 | 16706 | gene;island | 0.002788 | 0.016666 | Not queried | 1.59 | 5.30e-02 | 1.00e-01 |
|  | 11 | 47399813 | 47400113 | PDFSTATSENSEMBLUCSC | 301 | 6 | 6607 | gene | 0.933806 | 0.981843 | Not queried | 1.59 | 4.85e-01 | 5.11e-01 |
|  | 11 | 111385338 | 111385496 | PDFSTATSENSEMBLUCSC | 159 | 5 | 7687 | tss | 0.860905 | 0.969463 | Not queried | 1.59 | 2.22e-01 | 2.76e-01 |
|  | 3 | 9291125 | 9291310 | PDFSTATSENSEMBLUCSC | 186 | 5 | 27652 | gene | 0.005857 | 0.017366 | Not queried | 1.59 | 5.48e-01 | 5.70e-01 |
|  | 5 | 36607333 | 36607417 | PDFSTATSENSEMBLUCSC | 85 | 4 | 31587 | gene;tss | 0.037332 | 0.016106 | Not queried | 1.59 | 3.43e-01 | 3.84e-01 |
|  | 17 | 9694619 | 9695137 | PDFSTATSENSEMBLUCSC | 519 | 6 | 17130 | tss | 0.658277 | 0.934034 | Not queried | 1.59 | 6.49e-02 | 1.11e-01 |
|  | 20 | 10655288 | 10655644 | PDFSTATSENSEMBLUCSC | 357 | 6 | 25599 | tss;island | 0.005560 | 0.015423 | Not queried | 1.59 | 4.11e-02 | 8.14e-02 |
|  | 21 | 45208971 | 45209230 | PDFSTATSENSEMBLUCSC | 260 | 4 | 26578 | tss;island | 0.012168 | 0.016492 | Not queried | 1.59 | 6.86e-01 | 6.99e-01 |
|  | 22 | 24891129 | 24891452 | PDFSTATSENSEMBLUCSC | 324 | 8 | 27021 | tss;island | 0.959683 | 0.980263 | Not queried | 1.59 | 1.05e-01 | 1.66e-01 |
|  | 7 | 27170412 | 27171528 | PDFSTATSENSEMBLUCSC | 1117 | 18 | 37491 | tss | 0.274178 | 0.067622 | Not queried | 1.59 | 1.26e-01 | 1.85e-01 |
|  | 20 | 30225325 | 30226046 | PDFSTATSENSEMBLUCSC | 722 | 9 | 26077 | island | 0.746062 | 0.948032 | Not queried | 1.59 | 6.25e-02 | 1.09e-01 |
|  | 15 | 22646692 | 22647092 | PDFSTATSENSEMBLUCSC | 401 | 4 | 13938 | island | 0.607413 | 0.828578 | Not queried | 1.59 | 3.43e-01 | 3.84e-01 |
|  | 2 | 43454447 | 43455103 | PDFSTATSENSEMBLUCSC | 657 | 21 | 22658 | gene | 0.021105 | 0.034570 | Not queried | 1.59 | 6.22e-02 | 1.09e-01 |
|  | 1 | 58715499 | 58715747 | PDFSTATSENSEMBLUCSC | 249 | 6 | 556 | gene;island | 0.740334 | 0.973233 | Not queried | 1.58 | 3.10e-01 | 3.66e-01 |
|  | 20 | 33104052 | 33104576 | PDFSTATSENSEMBLUCSC | 525 | 10 | 26110 | island | 0.004229 | 0.009301 | Not queried | 1.58 | 1.05e-01 | 1.66e-01 |
|  | 2 | 232790974 | 232791126 | PDFSTATSENSEMBLUCSC | 153 | 6 | 23946 | tss;island | 0.076627 | 0.035396 | Not queried | 1.58 | 9.31e-02 | 1.53e-01 |
|  | 3 | 13590221 | 13590450 | PDFSTATSENSEMBLUCSC | 230 | 8 | 28165 | tss;island | 0.907088 | 0.968247 | Not queried | 1.58 | 3.79e-02 | 7.66e-02 |
|  | 22 | 29168272 | 29168647 | PDFSTATSENSEMBLUCSC | 376 | 8 | 27041 | tss | 0.007693 | 0.022465 | Not queried | 1.58 | 5.74e-01 | 5.97e-01 |
|  | 16 | 3414983 | 3415311 | PDFSTATSENSEMBLUCSC | 329 | 5 | 15647 | island | 0.269898 | 0.477481 | Not queried | 1.58 | 2.22e-01 | 2.76e-01 |
|  | 1 | 53925109 | 53925368 | PDFSTATSENSEMBLUCSC | 260 | 4 | 541 | gene | 0.919930 | 0.972214 | Not queried | 1.58 | 2.00e-01 | 2.55e-01 |
|  | 18 | 33529997 | 33530560 | PDFSTATSENSEMBLUCSC | 564 | 6 | 19466 | island | 0.147758 | 0.472489 | Not queried | 1.58 | 5.89e-01 | 6.11e-01 |
|  | 14 | 54908007 | 54908353 | PDFSTATSENSEMBLUCSC | 347 | 9 | 12856 | island | 0.003137 | 0.006380 | Not queried | 1.58 | 3.87e-01 | 4.30e-01 |
|  | 1 | 13881331 | 13881656 | PDFSTATSENSEMBLUCSC | 326 | 5 | 2704 | island | 0.912999 | 0.970913 | Not queried | 1.58 | 3.10e-01 | 3.66e-01 |
|  | 10 | 30638275 | 30638503 | PDFSTATSENSEMBLUCSC | 229 | 5 | 5011 | tss | 0.008735 | 0.027256 | Not queried | 1.58 | 6.90e-01 | 7.01e-01 |
|  | 11 | 20620680 | 20621109 | PDFSTATSENSEMBLUCSC | 430 | 7 | 8130 | island | 0.740047 | 0.844440 | Not queried | 1.58 | 6.20e-01 | 6.43e-01 |
|  | 2 | 3683030 | 3683283 | PDFSTATSENSEMBLUCSC | 254 | 4 | 22554 | gene;island | 0.568060 | 0.842490 | Not queried | 1.58 | 1.14e-01 | 1.68e-01 |
|  | 17 | 77311577 | 77311812 | PDFSTATSENSEMBLUCSC | 236 | 4 | 16839 | gene | 0.412710 | 0.564727 | Not queried | 1.58 | 2.00e-01 | 2.55e-01 |
|  | 17 | 46799479 | 46800028 | PDFSTATSENSEMBLUCSC | 550 | 9 | 17469 | tss;island | 0.801052 | 0.968458 | Not queried | 1.58 | 1.88e-02 | 6.12e-02 |
|  | 17 | 6917614 | 6918952 | PDFSTATSENSEMBLUCSC | 1339 | 13 | 17915 | island | 0.002761 | 0.008714 | Not queried | 1.58 | 3.36e-01 | 3.84e-01 |
|  | 17 | 43506497 | 43507153 | PDFSTATSENSEMBLUCSC | 657 | 7 | 18438 | island | 0.890035 | 0.973373 | Not queried | 1.58 | 9.73e-02 | 1.55e-01 |
|  | 14 | 37116121 | 37116408 | PDFSTATSENSEMBLUCSC | 288 | 4 | 12788 | island | 0.902092 | 0.965118 | Not queried | 1.58 | 2.00e-01 | 2.55e-01 |
|  | 22 | 17602561 | 17602681 | PDFSTATSENSEMBLUCSC | 121 | 4 | 26941 | tss;island | 0.731390 | 0.518742 | Not queried | 1.58 | 3.43e-01 | 3.84e-01 |
|  | 17 | 46755422 | 46755786 | PDFSTATSENSEMBLUCSC | 365 | 4 | 18496 | island | 0.735246 | 0.632223 | Not queried | 1.58 | 3.43e-01 | 3.84e-01 |
|  | 14 | 51562381 | 51562646 | PDFSTATSENSEMBLUCSC | 266 | 6 | 12837 | island | 0.598049 | 0.439113 | Not queried | 1.58 | 3.94e-01 | 4.36e-01 |
|  | 1 | 145507220 | 145507663 | PDFSTATSENSEMBLUCSC | 444 | 12 | 3682 | island | 0.007902 | 0.019044 | Not queried | 1.58 | 3.47e-01 | 3.89e-01 |
|  | 2 | 201936469 | 201936709 | PDFSTATSENSEMBLUCSC | 241 | 7 | 22984 | gene | 0.001780 | 0.003264 | Not queried | 1.58 | 1.65e-01 | 2.28e-01 |
|  | 17 | 21030030 | 21030370 | PDFSTATSENSEMBLUCSC | 341 | 8 | 18115 | island | 0.002423 | 0.006923 | Not queried | 1.58 | 2.79e-01 | 3.38e-01 |
|  | 6 | 11537706 | 11538071 | PDFSTATSENSEMBLUCSC | 366 | 4 | 34637 | tss;island | 0.061548 | 0.053031 | Not queried | 1.58 | 4.86e-01 | 5.11e-01 |
|  | 20 | 61885926 | 61886313 | PDFSTATSENSEMBLUCSC | 388 | 6 | 25893 | tss | 0.739374 | 0.917317 | Not queried | 1.58 | 3.10e-01 | 3.66e-01 |
|  | 19 | 19651867 | 19652066 | PDFSTATSENSEMBLUCSC | 200 | 4 | 19955 | gene;island | 0.944251 | 0.982150 | Not queried | 1.58 | 2.86e-02 | 6.12e-02 |
|  | 3 | 49314370 | 49314920 | PDFSTATSENSEMBLUCSC | 551 | 11 | 29113 | island | 0.948245 | 0.981949 | C3orf62; USP4 (margin: Y\_RNA; MIR4271; C3orf62; USP4) | 1.58 | 7.65e-04 | 8.13e-03 |
|  | 3 | 49314539 | 49314920 | PDFSTATSENSEMBLUCSC | 382 | 9 | 28325 | tss | 0.952964 | 0.981949 | C3orf62; USP4 (margin: Y\_RNA; MIR4271; C3orf62; USP4) | 1.58 | 3.99e-03 | 2.47e-02 |
|  | 1 | 153581494 | 153581793 | PDFSTATSENSEMBLUCSC | 300 | 5 | 761 | gene | 0.014530 | 0.031614 | Not queried | 1.58 | 2.22e-01 | 2.76e-01 |
|  | 20 | 48599292 | 48599682 | PDFSTATSENSEMBLUCSC | 391 | 8 | 26242 | island | 0.060961 | 0.032334 | Not queried | 1.58 | 3.82e-01 | 4.27e-01 |
|  | 7 | 156433068 | 156433342 | PDFSTATSENSEMBLUCSC | 275 | 5 | 37314 | gene | 0.007850 | 0.007967 | Not queried | 1.58 | 8.41e-01 | 8.48e-01 |
|  | 18 | 77160030 | 77160221 | PDFSTATSENSEMBLUCSC | 192 | 6 | 19334 | tss | 0.067299 | 0.032846 | Not queried | 1.58 | 3.10e-01 | 3.66e-01 |
|  | 14 | 58666496 | 58666796 | PDFSTATSENSEMBLUCSC | 301 | 4 | 12421 | tss | 0.003454 | 0.004185 | Not queried | 1.58 | 8.86e-01 | 8.89e-01 |
|  | 11 | 13983705 | 13984067 | PDFSTATSENSEMBLUCSC | 363 | 10 | 8072 | island | 0.939476 | 0.977085 | Not queried | 1.58 | 8.92e-02 | 1.50e-01 |
|  | 18 | 57364722 | 57364872 | PDFSTATSENSEMBLUCSC | 151 | 4 | 19299 | tss | 0.083622 | 0.033196 | Not queried | 1.58 | 3.43e-01 | 3.84e-01 |
|  | 22 | 50919817 | 50919961 | PDFSTATSENSEMBLUCSC | 145 | 5 | 27255 | tss | 0.043071 | 0.157581 | Not queried | 1.58 | 5.48e-01 | 5.70e-01 |
|  | 19 | 55866087 | 55866412 | PDFSTATSENSEMBLUCSC | 326 | 9 | 22419 | island | 0.126114 | 0.388056 | Not queried | 1.58 | 1.90e-01 | 2.55e-01 |
|  | 19 | 55866305 | 55866412 | PDFSTATSENSEMBLUCSC | 108 | 5 | 20228 | gene | 0.261296 | 0.552122 | Not queried | 1.58 | 6.90e-01 | 7.01e-01 |
|  | 6 | 33257475 | 33258581 | PDFSTATSENSEMBLUCSC | 1107 | 25 | 34252 | gene | 0.012145 | 0.043629 | Not queried | 1.58 | 8.78e-02 | 1.48e-01 |
|  | 6 | 34393975 | 34394098 | PDFSTATSENSEMBLUCSC | 124 | 7 | 34918 | tss | 0.000282 | 0.001605 | Not queried | 1.58 | 1.28e-01 | 1.87e-01 |
|  | 3 | 9791448 | 9791615 | PDFSTATSENSEMBLUCSC | 168 | 4 | 28129 | tss | 0.010430 | 0.004902 | Not queried | 1.57 | 6.86e-01 | 6.99e-01 |
|  | 16 | 3085571 | 3085982 | PDFSTATSENSEMBLUCSC | 412 | 4 | 15048 | tss | 0.565728 | 0.710767 | Not queried | 1.57 | 3.43e-01 | 3.84e-01 |
|  | 6 | 111279493 | 111279703 | PDFSTATSENSEMBLUCSC | 211 | 9 | 35173 | tss | 0.028243 | 0.124880 | Not queried | 1.57 | 4.00e-02 | 8.03e-02 |
|  | 19 | 49259215 | 49259452 | PDFSTATSENSEMBLUCSC | 238 | 4 | 20927 | tss;island | 0.852007 | 0.909751 | Not queried | 1.57 | 4.86e-01 | 5.11e-01 |
|  | 8 | 74205400 | 74205666 | PDFSTATSENSEMBLUCSC | 267 | 4 | 39336 | gene;island | 0.004473 | 0.016892 | Not queried | 1.57 | 1.14e-01 | 1.68e-01 |
|  | 10 | 104535792 | 104536121 | PDFSTATSENSEMBLUCSC | 330 | 9 | 4738 | gene | 0.871398 | 0.953473 | Not queried | 1.57 | 9.39e-02 | 1.53e-01 |
|  | 1 | 152008878 | 152009496 | PDFSTATSENSEMBLUCSC | 619 | 8 | 757 | gene | 0.007079 | 0.011305 | Not queried | 1.57 | 6.50e-02 | 1.11e-01 |
|  | 11 | 73693792 | 73694157 | PDFSTATSENSEMBLUCSC | 366 | 6 | 8646 | island | 0.046255 | 0.010886 | Not queried | 1.57 | 3.10e-01 | 3.66e-01 |
|  | 17 | 15902501 | 15903290 | PDFSTATSENSEMBLUCSC | 790 | 11 | 16456 | gene;island | 0.004441 | 0.006991 | Not queried | 1.57 | 6.99e-01 | 7.09e-01 |
|  | 3 | 49314595 | 49314920 | PDFSTATSENSEMBLUCSC | 326 | 8 | 27779 | gene | 0.956234 | 0.984618 | C3orf62; USP4 (margin: Y\_RNA; MIR4271; C3orf62; USP4) | 1.57 | 1.04e-02 | 3.96e-02 |
|  | 17 | 4510572 | 4510827 | PDFSTATSENSEMBLUCSC | 256 | 5 | 16355 | gene;island | 0.837063 | 0.967761 | Not queried | 1.57 | 2.22e-01 | 2.76e-01 |
|  | 10 | 94455543 | 94455895 | PDFSTATSENSEMBLUCSC | 353 | 4 | 5946 | island | 0.934835 | 0.982279 | Not queried | 1.57 | 2.00e-01 | 2.55e-01 |
|  | 10 | 18940291 | 18940404 | PDFSTATSENSEMBLUCSC | 114 | 4 | 4524 | gene;island | 0.001071 | 0.002632 | Not queried | 1.57 | 2.00e-01 | 2.55e-01 |
|  | 2 | 44065259 | 44065893 | PDFSTATSENSEMBLUCSC | 635 | 13 | 22661 | gene | 0.769590 | 0.888537 | ABCG8; ABCG5 (margin: ABCG8; ABCG5) | 1.57 | 4.22e-03 | 2.52e-02 |
|  | 2 | 44065259 | 44066294 | PDFSTATSENSEMBLUCSC | 1036 | 19 | 23329 | tss | 0.769590 | 0.888537 | ABCG8; ABCG5 (margin: ABCG8; ABCG5) | 1.57 | 1.19e-02 | 4.42e-02 |
|  | 1 | 228395660 | 228395858 | PDFSTATSENSEMBLUCSC | 199 | 4 | 2303 | tss | 0.837800 | 0.889721 | Not queried | 1.57 | 4.86e-01 | 5.11e-01 |
|  | 8 | 86350889 | 86351018 | PDFSTATSENSEMBLUCSC | 130 | 5 | 39795 | tss | 0.685549 | 0.428133 | CA3 (margin: CA3; RP11-317J10.2) | 1.57 | 7.94e-03 | 3.19e-02 |
|  | 2 | 240031454 | 240031700 | PDFSTATSENSEMBLUCSC | 247 | 4 | 23105 | gene;island | 0.968639 | 0.896847 | Not queried | 1.57 | 2.00e-01 | 2.55e-01 |
|  | 6 | 32145071 | 32145830 | PDFSTATSENSEMBLUCSC | 760 | 22 | 34072 | gene | 0.004197 | 0.009005 | Not queried | 1.57 | 1.95e-01 | 2.55e-01 |
|  | 10 | 119786 | 120151 | PDFSTATSENSEMBLUCSC | 366 | 4 | 5467 | island | 0.150940 | 0.195764 | Not queried | 1.57 | 3.43e-01 | 3.84e-01 |
|  | 7 | 27281216 | 27281964 | PDFSTATSENSEMBLUCSC | 749 | 14 | 37500 | tss;island | 0.889069 | 0.948642 | Not queried | 1.57 | 1.94e-01 | 2.55e-01 |
|  | 20 | 4795836 | 4796176 | PDFSTATSENSEMBLUCSC | 341 | 7 | 25350 | gene;tss | 0.853318 | 0.942759 | Not queried | 1.57 | 9.73e-02 | 1.55e-01 |
|  | 14 | 88792913 | 88793311 | PDFSTATSENSEMBLUCSC | 399 | 8 | 13057 | island | 0.297270 | 0.524384 | KCNK10 (margin: KCNK10) | 1.57 | 4.66e-03 | 2.68e-02 |
|  | 7 | 157849615 | 157849794 | PDFSTATSENSEMBLUCSC | 180 | 5 | 37358 | gene | 0.625497 | 0.351531 | Not queried | 1.57 | 4.21e-01 | 4.59e-01 |
|  | 22 | 24823389 | 24823554 | PDFSTATSENSEMBLUCSC | 166 | 6 | 27361 | island | 0.904184 | 0.938730 | Not queried | 1.57 | 3.10e-01 | 3.66e-01 |
|  | 3 | 62362660 | 62363248 | PDFSTATSENSEMBLUCSC | 589 | 5 | 29250 | island | 0.863326 | 0.971135 | Not queried | 1.57 | 5.56e-02 | 1.00e-01 |
|  | 6 | 35656590 | 35656906 | PDFSTATSENSEMBLUCSC | 317 | 4 | 34310 | gene;island | 0.001776 | 0.009040 | Not queried | 1.57 | 4.86e-01 | 5.11e-01 |
|  | 3 | 183353051 | 183353393 | PDFSTATSENSEMBLUCSC | 343 | 5 | 28747 | tss | 0.002903 | 0.004262 | Not queried | 1.57 | 8.41e-01 | 8.48e-01 |
|  | 11 | 3818695 | 3818872 | PDFSTATSENSEMBLUCSC | 178 | 6 | 6481 | gene | 0.003864 | 0.007582 | Not queried | 1.57 | 4.85e-01 | 5.11e-01 |
|  | 13 | 46626913 | 46627203 | PDFSTATSENSEMBLUCSC | 291 | 9 | 11425 | tss | 0.002587 | 0.009303 | Not queried | 1.57 | 2.58e-01 | 3.16e-01 |
|  | 6 | 26271193 | 26271827 | PDFSTATSENSEMBLUCSC | 635 | 9 | 35532 | island | 0.008351 | 0.026594 | Not queried | 1.57 | 3.87e-01 | 4.30e-01 |
|  | 17 | 77375461 | 77375842 | PDFSTATSENSEMBLUCSC | 382 | 5 | 16840 | gene | 0.437448 | 0.429271 | Not queried | 1.57 | 6.90e-01 | 7.01e-01 |
|  | 2 | 178417539 | 178418053 | PDFSTATSENSEMBLUCSC | 515 | 11 | 23749 | tss | 0.003843 | 0.007468 | Not queried | 1.57 | 3.36e-02 | 6.87e-02 |
|  | 20 | 61569198 | 61569335 | PDFSTATSENSEMBLUCSC | 138 | 5 | 25885 | tss;island | 0.003819 | 0.005350 | Not queried | 1.57 | 6.90e-01 | 7.01e-01 |
|  | 1 | 3566701 | 3568245 | PDFSTATSENSEMBLUCSC | 1545 | 34 | 1182 | tss | 0.840915 | 0.970906 | TP73; WRAP73 (margin: TP73; WRAP73; RP5-1092A11.5) | 1.57 | 7.07e-03 | 3.19e-02 |
|  | 1 | 207627941 | 207628182 | PDFSTATSENSEMBLUCSC | 242 | 4 | 977 | gene;island | 0.569218 | 0.309213 | Not queried | 1.57 | 1.14e-01 | 1.68e-01 |
|  | 7 | 907797 | 908060 | PDFSTATSENSEMBLUCSC | 264 | 4 | 36724 | gene;island | 0.964663 | 0.902173 | Not queried | 1.57 | 2.00e-01 | 2.55e-01 |
|  | 3 | 183892577 | 183892606 | PDFSTATSENSEMBLUCSC | 30 | 6 | 28752 | tss | 0.003565 | 0.008311 | Not queried | 1.57 | 3.94e-01 | 4.36e-01 |
|  | 3 | 152552877 | 152553204 | PDFSTATSENSEMBLUCSC | 328 | 4 | 27998 | gene;island | 0.180874 | 0.336203 | Not queried | 1.57 | 6.86e-01 | 6.99e-01 |
|  | 19 | 11517079 | 11517436 | PDFSTATSENSEMBLUCSC | 358 | 5 | 19838 | gene;island | 0.683070 | 0.492668 | Not queried | 1.57 | 5.56e-02 | 1.00e-01 |
|  | 16 | 23521605 | 23521983 | PDFSTATSENSEMBLUCSC | 379 | 5 | 15763 | island | 0.007268 | 0.002362 | Not queried | 1.57 | 2.22e-01 | 2.76e-01 |
|  | 11 | 6280988 | 6281383 | PDFSTATSENSEMBLUCSC | 396 | 6 | 6487 | gene | 0.191311 | 0.481736 | Not queried | 1.56 | 8.18e-01 | 8.27e-01 |
|  | 12 | 72079550 | 72080251 | PDFSTATSENSEMBLUCSC | 702 | 7 | 10625 | island | 0.002262 | 0.005452 | Not queried | 1.56 | 6.20e-01 | 6.43e-01 |
|  | 6 | 36561383 | 36562081 | PDFSTATSENSEMBLUCSC | 699 | 9 | 34939 | tss | 0.003302 | 0.010964 | Not queried | 1.56 | 1.36e-01 | 1.94e-01 |
|  | 12 | 108985685 | 108986019 | PDFSTATSENSEMBLUCSC | 335 | 4 | 9286 | gene | 0.855260 | 0.942433 | Not queried | 1.56 | 6.86e-01 | 6.99e-01 |
|  | 1 | 145575570 | 145575814 | PDFSTATSENSEMBLUCSC | 245 | 5 | 1889 | tss;island | 0.005595 | 0.013971 | Not queried | 1.56 | 2.22e-01 | 2.76e-01 |
|  | 3 | 52828583 | 52828680 | PDFSTATSENSEMBLUCSC | 98 | 4 | 28393 | tss | 0.637981 | 0.909750 | Not queried | 1.56 | 4.86e-01 | 5.11e-01 |
|  | 14 | 88788343 | 88788672 | PDFSTATSENSEMBLUCSC | 330 | 6 | 12165 | gene;island | 0.191286 | 0.338478 | Not queried | 1.56 | 5.89e-01 | 6.11e-01 |
|  | 21 | 41238940 | 41239323 | PDFSTATSENSEMBLUCSC | 384 | 4 | 26553 | tss | 0.008915 | 0.078978 | Not queried | 1.56 | 4.86e-01 | 5.11e-01 |
|  | 3 | 69812798 | 69812973 | PDFSTATSENSEMBLUCSC | 176 | 4 | 27855 | gene | 0.675676 | 0.770298 | Not queried | 1.56 | 8.86e-01 | 8.89e-01 |
|  | 7 | 102036598 | 102036991 | PDFSTATSENSEMBLUCSC | 394 | 8 | 38801 | island | 0.005350 | 0.016182 | Not queried | 1.56 | 2.34e-01 | 2.90e-01 |
|  | 17 | 46620300 | 46620460 | PDFSTATSENSEMBLUCSC | 161 | 5 | 16650 | gene;island | 0.657589 | 0.858554 | Not queried | 1.56 | 1.51e-01 | 2.11e-01 |
|  | 17 | 46894191 | 46894465 | PDFSTATSENSEMBLUCSC | 275 | 4 | 16668 | gene | 0.013067 | 0.009728 | Not queried | 1.56 | 6.86e-01 | 6.99e-01 |
|  | 4 | 987303 | 987672 | PDFSTATSENSEMBLUCSC | 370 | 5 | 30234 | tss | 0.897001 | 0.749334 | Not queried | 1.56 | 9.52e-02 | 1.53e-01 |
|  | 8 | 21771059 | 21771668 | PDFSTATSENSEMBLUCSC | 610 | 7 | 40119 | island | 0.938884 | 0.962875 | Not queried | 1.56 | 1.65e-01 | 2.28e-01 |
|  | 1 | 101361710 | 101362002 | PDFSTATSENSEMBLUCSC | 293 | 4 | 653 | gene;island | 0.001297 | 0.000710 | Not queried | 1.56 | 2.00e-01 | 2.55e-01 |
|  | 13 | 25861182 | 25861849 | PDFSTATSENSEMBLUCSC | 668 | 6 | 11611 | island | 0.004999 | 0.006382 | Not queried | 1.56 | 4.85e-01 | 5.11e-01 |
|  | 7 | 100183965 | 100184356 | PDFSTATSENSEMBLUCSC | 392 | 5 | 37143 | gene | 0.005979 | 0.006857 | Not queried | 1.56 | 6.90e-01 | 7.01e-01 |
|  | 6 | 169224886 | 169225041 | PDFSTATSENSEMBLUCSC | 156 | 4 | 36667 | island | 0.874435 | 0.940274 | Not queried | 1.56 | 1.14e-01 | 1.68e-01 |
|  | 6 | 36842476 | 36842933 | PDFSTATSENSEMBLUCSC | 458 | 8 | 36088 | island | 0.007169 | 0.008743 | Not queried | 1.56 | 5.74e-01 | 5.97e-01 |
|  | 4 | 8588716 | 8588929 | PDFSTATSENSEMBLUCSC | 214 | 5 | 29955 | gene;island | 0.809373 | 0.544304 | Not queried | 1.56 | 5.48e-01 | 5.70e-01 |
|  | 6 | 35772606 | 35772999 | PDFSTATSENSEMBLUCSC | 394 | 8 | 34932 | tss;island | 0.037235 | 0.008246 | Not queried | 1.56 | 3.28e-01 | 3.84e-01 |
|  | 6 | 32021154 | 32021500 | PDFSTATSENSEMBLUCSC | 347 | 5 | 34019 | gene | 0.451919 | 0.697922 | Not queried | 1.56 | 3.10e-01 | 3.66e-01 |
|  | 1 | 151138249 | 151138408 | PDFSTATSENSEMBLUCSC | 160 | 5 | 749 | gene | 0.011749 | 0.026489 | Not queried | 1.56 | 9.52e-02 | 1.53e-01 |
|  | 8 | 12869510 | 12869722 | PDFSTATSENSEMBLUCSC | 213 | 5 | 39210 | gene;tss | 0.451446 | 0.958986 | Not queried | 1.56 | 2.22e-01 | 2.76e-01 |
|  | 17 | 17109570 | 17110353 | PDFSTATSENSEMBLUCSC | 784 | 14 | 18060 | island | 0.011392 | 0.002609 | Not queried | 1.56 | 2.27e-01 | 2.82e-01 |
|  | 5 | 172044853 | 172045075 | PDFSTATSENSEMBLUCSC | 223 | 4 | 33392 | island | 0.759068 | 0.912420 | Not queried | 1.56 | 3.43e-01 | 3.84e-01 |
|  | 13 | 96204493 | 96204917 | PDFSTATSENSEMBLUCSC | 425 | 9 | 11502 | tss | 0.904283 | 0.975433 | Not queried | 1.56 | 1.61e-01 | 2.25e-01 |
|  | 11 | 60609242 | 60610004 | PDFSTATSENSEMBLUCSC | 763 | 11 | 8313 | island | 0.015838 | 0.024587 | Not queried | 1.56 | 3.00e-01 | 3.63e-01 |
|  | 20 | 56323934 | 56324226 | PDFSTATSENSEMBLUCSC | 293 | 4 | 26285 | island | 0.060787 | 0.109939 | Not queried | 1.56 | 3.43e-01 | 3.84e-01 |
|  | 12 | 108954266 | 108954532 | PDFSTATSENSEMBLUCSC | 267 | 4 | 9285 | gene;island | 0.004978 | 0.013297 | Not queried | 1.56 | 2.00e-01 | 2.55e-01 |
|  | 6 | 7389798 | 7389903 | PDFSTATSENSEMBLUCSC | 106 | 7 | 33551 | gene | 0.005414 | 0.016493 | Not queried | 1.56 | 1.65e-01 | 2.28e-01 |
|  | 10 | 327132 | 327612 | PDFSTATSENSEMBLUCSC | 481 | 6 | 4431 | gene | 0.878643 | 0.975846 | Not queried | 1.56 | 3.10e-01 | 3.66e-01 |
|  | 14 | 67707306 | 67707462 | PDFSTATSENSEMBLUCSC | 157 | 4 | 12468 | tss;island | 0.004190 | 0.006989 | Not queried | 1.56 | 6.86e-01 | 6.99e-01 |
|  | 6 | 117086931 | 117087238 | PDFSTATSENSEMBLUCSC | 308 | 7 | 35193 | tss | 0.378455 | 0.515854 | Not queried | 1.56 | 3.18e-01 | 3.74e-01 |
|  | 19 | 291986 | 292498 | PDFSTATSENSEMBLUCSC | 513 | 12 | 20270 | tss;island | 0.925731 | 0.973256 | PPAP2C (margin: VN2R11P; PPAP2C) | 1.56 | 4.96e-04 | 6.47e-03 |
|  | 1 | 228657381 | 228657762 | PDFSTATSENSEMBLUCSC | 382 | 4 | 4303 | island | 0.780479 | 0.960555 | Not queried | 1.56 | 4.86e-01 | 5.11e-01 |
|  | 14 | 45722289 | 45722745 | PDFSTATSENSEMBLUCSC | 457 | 9 | 12816 | island | 0.006336 | 0.014281 | Not queried | 1.56 | 3.15e-02 | 6.50e-02 |
|  | 2 | 201753570 | 201753996 | PDFSTATSENSEMBLUCSC | 427 | 6 | 22981 | gene | 0.009210 | 0.021713 | Not queried | 1.56 | 2.40e-01 | 2.95e-01 |
|  | 2 | 158114069 | 158114300 | PDFSTATSENSEMBLUCSC | 232 | 4 | 23676 | tss | 0.400630 | 0.654733 | Not queried | 1.56 | 2.86e-02 | 6.12e-02 |
|  | 8 | 124219425 | 124219725 | PDFSTATSENSEMBLUCSC | 301 | 4 | 39419 | gene;island | 0.974053 | 0.901481 | Not queried | 1.56 | 2.00e-01 | 2.55e-01 |
|  | 1 | 10489962 | 10490667 | PDFSTATSENSEMBLUCSC | 706 | 11 | 1237 | tss;island | 0.440360 | 0.119813 | Not queried | 1.56 | 4.38e-01 | 4.78e-01 |
|  | 11 | 109292789 | 109293216 | PDFSTATSENSEMBLUCSC | 428 | 7 | 8787 | island | 0.230259 | 0.514832 | Not queried | 1.56 | 9.73e-02 | 1.55e-01 |
|  | 3 | 170136159 | 170136539 | PDFSTATSENSEMBLUCSC | 381 | 9 | 28714 | tss;island | 0.939784 | 0.972163 | Not queried | 1.56 | 1.61e-01 | 2.25e-01 |
|  | 17 | 16120515 | 16120890 | PDFSTATSENSEMBLUCSC | 376 | 4 | 16457 | gene | 0.003018 | 0.008996 | Not queried | 1.56 | 4.86e-01 | 5.11e-01 |
|  | 1 | 990293 | 990557 | PDFSTATSENSEMBLUCSC | 265 | 4 | 11 | gene;island | 0.946739 | 0.985131 | Not queried | 1.56 | 5.71e-02 | 1.00e-01 |
|  | 18 | 2654819 | 2655213 | PDFSTATSENSEMBLUCSC | 395 | 6 | 19158 | tss;island | 0.005195 | 0.023520 | Not queried | 1.56 | 3.94e-01 | 4.36e-01 |
|  | 6 | 99272871 | 99273830 | PDFSTATSENSEMBLUCSC | 960 | 12 | 36340 | island | 0.647898 | 0.825195 | Not queried | 1.55 | 2.91e-01 | 3.53e-01 |
|  | 6 | 1601229 | 1601802 | PDFSTATSENSEMBLUCSC | 574 | 5 | 35371 | island | 0.984320 | 0.958745 | Not queried | 1.55 | 5.56e-02 | 1.00e-01 |
|  | 6 | 33215649 | 33215868 | PDFSTATSENSEMBLUCSC | 220 | 6 | 35986 | island | 0.979741 | 0.950363 | Not queried | 1.55 | 1.32e-01 | 1.89e-01 |
|  | 2 | 54557836 | 54558089 | PDFSTATSENSEMBLUCSC | 254 | 7 | 24300 | island | 0.002419 | 0.009017 | Not queried | 1.55 | 2.09e-01 | 2.65e-01 |
|  | 1 | 109234629 | 109235208 | PDFSTATSENSEMBLUCSC | 580 | 11 | 3544 | island | 0.003207 | 0.007542 | Not queried | 1.55 | 1.16e-01 | 1.71e-01 |
|  | 17 | 900031 | 900325 | PDFSTATSENSEMBLUCSC | 295 | 10 | 16984 | tss | 0.003148 | 0.006811 | Not queried | 1.55 | 8.92e-02 | 1.50e-01 |
|  | 6 | 32920567 | 32920880 | PDFSTATSENSEMBLUCSC | 314 | 5 | 34166 | gene | 0.531097 | 0.782041 | Not queried | 1.55 | 4.21e-01 | 4.59e-01 |
|  | 6 | 29817963 | 29818300 | PDFSTATSENSEMBLUCSC | 338 | 8 | 35638 | island | 0.601095 | 0.937310 | Not queried | 1.55 | 1.30e-01 | 1.89e-01 |
|  | 1 | 70385100 | 70385450 | PDFSTATSENSEMBLUCSC | 351 | 5 | 577 | gene;island | 0.811656 | 0.965330 | Not queried | 1.55 | 2.22e-01 | 2.76e-01 |
|  | 4 | 121844174 | 121844401 | PDFSTATSENSEMBLUCSC | 228 | 7 | 30569 | tss;island | 0.446199 | 0.256758 | Not queried | 1.55 | 2.59e-01 | 3.16e-01 |
|  | 19 | 3982566 | 3983219 | PDFSTATSENSEMBLUCSC | 654 | 10 | 19728 | gene;island | 0.847620 | 0.969111 | Not queried | 1.55 | 3.55e-02 | 7.24e-02 |
|  | 10 | 6020330 | 6020585 | PDFSTATSENSEMBLUCSC | 256 | 4 | 4930 | tss;island | 0.847825 | 0.965213 | Not queried | 1.55 | 2.00e-01 | 2.55e-01 |
|  | 11 | 1862020 | 1862477 | PDFSTATSENSEMBLUCSC | 458 | 4 | 6434 | gene | 0.927666 | 0.967917 | Not queried | 1.55 | 1.14e-01 | 1.68e-01 |
|  | 7 | 86781331 | 86781855 | PDFSTATSENSEMBLUCSC | 525 | 10 | 37682 | tss | 0.012967 | 0.017787 | Not queried | 1.55 | 1.05e-01 | 1.66e-01 |
|  | 3 | 113666360 | 113666584 | PDFSTATSENSEMBLUCSC | 225 | 4 | 28511 | tss;island | 0.005509 | 0.011542 | Not queried | 1.55 | 2.00e-01 | 2.55e-01 |
|  | 6 | 105400884 | 105401339 | PDFSTATSENSEMBLUCSC | 456 | 5 | 36373 | island | 0.975780 | 0.984065 | Not queried | 1.55 | 4.21e-01 | 4.59e-01 |
|  | 17 | 5026540 | 5026840 | PDFSTATSENSEMBLUCSC | 301 | 7 | 17041 | tss | 0.532723 | 0.812758 | Not queried | 1.55 | 9.73e-02 | 1.55e-01 |
|  | 19 | 58838420 | 58838697 | PDFSTATSENSEMBLUCSC | 278 | 4 | 20260 | gene | 0.001852 | 0.006175 | Not queried | 1.55 | 4.86e-01 | 5.11e-01 |
|  | 16 | 19124925 | 19125206 | PDFSTATSENSEMBLUCSC | 282 | 7 | 15113 | tss;island | 0.007850 | 0.019692 | Not queried | 1.55 | 2.09e-01 | 2.65e-01 |
|  | 17 | 61613061 | 61613356 | PDFSTATSENSEMBLUCSC | 296 | 5 | 16727 | gene;island | 0.777508 | 0.972111 | Not queried | 1.55 | 2.22e-01 | 2.76e-01 |
|  | 5 | 168271613 | 168272051 | PDFSTATSENSEMBLUCSC | 439 | 7 | 31887 | gene | 0.925736 | 0.967648 | Not queried | 1.55 | 2.09e-01 | 2.65e-01 |
|  | 6 | 33169975 | 33170452 | PDFSTATSENSEMBLUCSC | 478 | 6 | 34223 | gene;island | 0.892781 | 0.761202 | Not queried | 1.55 | 2.60e-02 | 6.12e-02 |
|  | 3 | 45635931 | 45636386 | PDFSTATSENSEMBLUCSC | 456 | 7 | 29048 | island | 0.039722 | 0.152138 | Not queried | 1.55 | 2.09e-01 | 2.65e-01 |
|  | 6 | 170531180 | 170531435 | PDFSTATSENSEMBLUCSC | 256 | 5 | 36682 | island | 0.974032 | 0.862581 | Not queried | 1.55 | 2.22e-01 | 2.76e-01 |
|  | 2 | 183943388 | 183943551 | PDFSTATSENSEMBLUCSC | 164 | 5 | 22954 | gene | 0.000722 | 0.003245 | Not queried | 1.55 | 6.90e-01 | 7.01e-01 |
|  | 1 | 15735880 | 15736208 | PDFSTATSENSEMBLUCSC | 329 | 6 | 1270 | tss;island | 0.023918 | 0.009802 | Not queried | 1.55 | 2.40e-01 | 2.95e-01 |
|  | 3 | 98376390 | 98376574 | PDFSTATSENSEMBLUCSC | 185 | 4 | 29316 | island | 0.606582 | 0.271284 | Not queried | 1.55 | 1.14e-01 | 1.68e-01 |
|  | 7 | 55433949 | 55434429 | PDFSTATSENSEMBLUCSC | 481 | 4 | 37019 | gene;island | 0.056465 | 0.008462 | Not queried | 1.55 | 3.43e-01 | 3.84e-01 |
|  | 19 | 45281140 | 45281287 | PDFSTATSENSEMBLUCSC | 148 | 4 | 20084 | gene | 0.238936 | 0.411202 | Not queried | 1.55 | 2.86e-02 | 6.12e-02 |
|  | 7 | 157368881 | 157369158 | PDFSTATSENSEMBLUCSC | 278 | 6 | 37329 | gene;island | 0.760337 | 0.883745 | Not queried | 1.55 | 3.10e-01 | 3.66e-01 |
|  | 16 | 838502 | 838782 | PDFSTATSENSEMBLUCSC | 281 | 4 | 14984 | tss;island | 0.002768 | 0.011006 | Not queried | 1.55 | 3.43e-01 | 3.84e-01 |
|  | 16 | 85157762 | 85158047 | PDFSTATSENSEMBLUCSC | 286 | 5 | 16149 | island | 0.753255 | 0.855164 | Not queried | 1.55 | 3.10e-01 | 3.66e-01 |
|  | 12 | 40499623 | 40500139 | PDFSTATSENSEMBLUCSC | 517 | 5 | 10326 | island | 0.137493 | 0.033771 | Not queried | 1.55 | 1.51e-01 | 2.11e-01 |
|  | 10 | 102896376 | 102896640 | PDFSTATSENSEMBLUCSC | 265 | 4 | 4725 | gene;island | 0.941738 | 0.979577 | Not queried | 1.55 | 1.14e-01 | 1.68e-01 |
|  | 6 | 30104552 | 30105198 | PDFSTATSENSEMBLUCSC | 647 | 11 | 33726 | gene | 0.361077 | 0.766818 | Not queried | 1.55 | 2.70e-01 | 3.29e-01 |
|  | 16 | 3085468 | 3085982 | PDFSTATSENSEMBLUCSC | 515 | 6 | 15634 | island | 0.780259 | 0.850966 | Not queried | 1.54 | 4.85e-01 | 5.11e-01 |
|  | 15 | 37393009 | 37393408 | PDFSTATSENSEMBLUCSC | 400 | 5 | 13285 | gene | 0.007326 | 0.035025 | Not queried | 1.54 | 9.52e-02 | 1.53e-01 |
|  | 6 | 170919283 | 170919448 | PDFSTATSENSEMBLUCSC | 166 | 4 | 36700 | island | 0.792635 | 0.961690 | Not queried | 1.54 | 4.86e-01 | 5.11e-01 |
|  | 10 | 118609145 | 118609402 | PDFSTATSENSEMBLUCSC | 258 | 4 | 6167 | island | 0.480462 | 0.269082 | Not queried | 1.54 | 3.43e-01 | 3.84e-01 |
|  | 19 | 42806146 | 42806556 | PDFSTATSENSEMBLUCSC | 411 | 10 | 20067 | gene;island | 0.007872 | 0.002458 | Not queried | 1.54 | 6.30e-02 | 1.10e-01 |
|  | 18 | 56435611 | 56435982 | PDFSTATSENSEMBLUCSC | 372 | 4 | 19540 | island | 0.008115 | 0.004211 | Not queried | 1.54 | 6.86e-01 | 6.99e-01 |
|  | 5 | 10441490 | 10441903 | PDFSTATSENSEMBLUCSC | 414 | 8 | 31989 | tss;island | 0.009377 | 0.007133 | Not queried | 1.54 | 6.45e-01 | 6.69e-01 |
|  | 2 | 45159434 | 45159663 | PDFSTATSENSEMBLUCSC | 230 | 4 | 24259 | island | 0.731768 | 0.891269 | Not queried | 1.54 | 4.86e-01 | 5.11e-01 |
|  | 1 | 2273591 | 2273958 | PDFSTATSENSEMBLUCSC | 368 | 4 | 76 | gene;island | 0.888813 | 0.961313 | Not queried | 1.54 | 2.00e-01 | 2.55e-01 |
|  | 5 | 87898489 | 87898688 | PDFSTATSENSEMBLUCSC | 200 | 4 | 31674 | gene;island | 0.350415 | 0.251419 | Not queried | 1.54 | 6.86e-01 | 6.99e-01 |
|  | 8 | 86350889 | 86351195 | PDFSTATSENSEMBLUCSC | 307 | 7 | 40432 | island | 0.685549 | 0.509735 | Not queried | 1.54 | 1.75e-02 | 5.79e-02 |
|  | 17 | 48422933 | 48423282 | PDFSTATSENSEMBLUCSC | 350 | 7 | 17496 | tss;island | 0.011213 | 0.011454 | Not queried | 1.54 | 8.05e-01 | 8.14e-01 |
|  | 12 | 2986348 | 2987138 | PDFSTATSENSEMBLUCSC | 791 | 9 | 9492 | tss;island | 0.010283 | 0.012818 | Not queried | 1.54 | 6.66e-01 | 6.91e-01 |
|  | 1 | 67772896 | 67773044 | PDFSTATSENSEMBLUCSC | 149 | 5 | 1683 | tss | 0.889273 | 0.980620 | Not queried | 1.54 | 1.51e-01 | 2.11e-01 |
|  | 10 | 112289853 | 112290331 | PDFSTATSENSEMBLUCSC | 479 | 7 | 6130 | island | 0.012876 | 0.026131 | Not queried | 1.54 | 7.28e-02 | 1.23e-01 |
|  | 1 | 24151609 | 24151885 | PDFSTATSENSEMBLUCSC | 277 | 4 | 322 | gene | 0.003620 | 0.005358 | Not queried | 1.54 | 1.14e-01 | 1.68e-01 |
|  | 13 | 114085424 | 114085732 | PDFSTATSENSEMBLUCSC | 309 | 5 | 11285 | gene;island | 0.934589 | 0.968014 | Not queried | 1.54 | 1.51e-01 | 2.11e-01 |
|  | 15 | 33360195 | 33360485 | PDFSTATSENSEMBLUCSC | 291 | 6 | 13554 | tss | 0.029608 | 0.008467 | Not queried | 1.54 | 3.94e-01 | 4.36e-01 |
|  | 22 | 42949526 | 42949829 | PDFSTATSENSEMBLUCSC | 304 | 8 | 27193 | tss | 0.120503 | 0.009583 | Not queried | 1.54 | 1.30e-01 | 1.89e-01 |
|  | 11 | 60282712 | 60282972 | PDFSTATSENSEMBLUCSC | 261 | 9 | 8310 | island | 0.803711 | 0.928468 | Not queried | 1.54 | 1.61e-01 | 2.25e-01 |
|  | 7 | 119912636 | 119912900 | PDFSTATSENSEMBLUCSC | 265 | 5 | 37859 | tss;island | 0.884833 | 0.458601 | Not queried | 1.54 | 1.00e+00 | 1.00e+00 |
|  | 22 | 26986233 | 26986419 | PDFSTATSENSEMBLUCSC | 187 | 5 | 27036 | tss;island | 0.008438 | 0.011473 | Not queried | 1.54 | 6.90e-01 | 7.01e-01 |
|  | 3 | 46742865 | 46743199 | PDFSTATSENSEMBLUCSC | 335 | 4 | 27754 | gene | 0.868036 | 0.910083 | Not queried | 1.54 | 6.86e-01 | 6.99e-01 |
|  | 17 | 202588 | 202759 | PDFSTATSENSEMBLUCSC | 172 | 4 | 16978 | tss;island | 0.644545 | 0.446315 | Not queried | 1.54 | 6.86e-01 | 6.99e-01 |
|  | 2 | 241395181 | 241395351 | PDFSTATSENSEMBLUCSC | 171 | 4 | 23121 | gene;tss;island | 0.348718 | 0.186850 | Not queried | 1.54 | 2.86e-02 | 6.12e-02 |
|  | 12 | 6832737 | 6833223 | PDFSTATSENSEMBLUCSC | 487 | 10 | 9524 | tss;island | 0.002078 | 0.009329 | Not queried | 1.54 | 3.55e-02 | 7.24e-02 |
|  | 6 | 32819858 | 32821742 | PDFSTATSENSEMBLUCSC | 1885 | 54 | 34155 | gene | 0.013181 | 0.032161 | PSMB9; TAP1 (margin: XXbac-BPG246D15.8; PSMB9; PSMB8; TAP1) | 1.54 | 6.31e-03 | 3.19e-02 |
|  | 6 | 26285232 | 26286262 | PDFSTATSENSEMBLUCSC | 1031 | 14 | 35535 | island | 0.002457 | 0.008870 | Not queried | 1.54 | 3.10e-02 | 6.50e-02 |
|  | 10 | 83636072 | 83636325 | PDFSTATSENSEMBLUCSC | 254 | 4 | 4656 | gene;tss;island | 0.526849 | 0.206167 | Not queried | 1.54 | 3.43e-01 | 3.84e-01 |
|  | 3 | 181413810 | 181414237 | PDFSTATSENSEMBLUCSC | 428 | 6 | 28040 | gene;island | 0.917847 | 0.975027 | Not queried | 1.54 | 4.11e-02 | 8.14e-02 |
|  | 19 | 13029889 | 13030354 | PDFSTATSENSEMBLUCSC | 466 | 8 | 21635 | island | 0.020367 | 0.005742 | Not queried | 1.54 | 2.79e-01 | 3.38e-01 |
|  | 10 | 1094976 | 1095235 | PDFSTATSENSEMBLUCSC | 260 | 7 | 5485 | island | 0.001799 | 0.005965 | Not queried | 1.54 | 2.59e-01 | 3.16e-01 |
|  | 10 | 1095069 | 1095235 | PDFSTATSENSEMBLUCSC | 167 | 5 | 4912 | tss | 0.001799 | 0.005965 | Not queried | 1.54 | 3.10e-01 | 3.66e-01 |
|  | 15 | 36871420 | 36871800 | PDFSTATSENSEMBLUCSC | 381 | 5 | 13567 | tss | 0.027957 | 0.008950 | Not queried | 1.54 | 2.22e-01 | 2.76e-01 |
|  | 12 | 48500072 | 48500318 | PDFSTATSENSEMBLUCSC | 247 | 5 | 9110 | gene;tss;island | 0.001085 | 0.000851 | Not queried | 1.54 | 8.41e-01 | 8.48e-01 |
|  | 14 | 101198681 | 101198811 | PDFSTATSENSEMBLUCSC | 131 | 4 | 12208 | gene;island | 0.948736 | 0.977647 | Not queried | 1.54 | 3.43e-01 | 3.84e-01 |
|  | 22 | 29168272 | 29168792 | PDFSTATSENSEMBLUCSC | 521 | 11 | 27386 | island | 0.003917 | 0.008368 | Not queried | 1.54 | 5.19e-01 | 5.46e-01 |
|  | 6 | 33130918 | 33132727 | PDFSTATSENSEMBLUCSC | 1810 | 30 | 34193 | gene;island | 0.498822 | 0.730910 | COL11A2 (margin: COL11A2) | 1.54 | 4.76e-03 | 2.73e-02 |
|  | 5 | 140529499 | 140529819 | PDFSTATSENSEMBLUCSC | 321 | 6 | 32363 | tss;island | 0.175789 | 0.381369 | Not queried | 1.54 | 3.94e-01 | 4.36e-01 |
|  | 12 | 48166716 | 48167095 | PDFSTATSENSEMBLUCSC | 380 | 6 | 10355 | island | 0.035159 | 0.007120 | Not queried | 1.54 | 9.31e-02 | 1.53e-01 |
|  | 19 | 34662636 | 34663206 | PDFSTATSENSEMBLUCSC | 571 | 7 | 20690 | tss;island | 0.005867 | 0.019429 | Not queried | 1.54 | 1.65e-01 | 2.28e-01 |
|  | 8 | 110374294 | 110374675 | PDFSTATSENSEMBLUCSC | 382 | 6 | 39870 | tss | 0.779789 | 0.814464 | Not queried | 1.53 | 3.94e-01 | 4.36e-01 |
|  | 8 | 110374294 | 110374866 | PDFSTATSENSEMBLUCSC | 573 | 8 | 40536 | island | 0.769279 | 0.768827 | Not queried | 1.53 | 4.42e-01 | 4.81e-01 |
|  | 3 | 44726891 | 44727195 | PDFSTATSENSEMBLUCSC | 305 | 5 | 29039 | island | 0.966436 | 0.980533 | Not queried | 1.53 | 2.22e-01 | 2.76e-01 |
|  | 19 | 20011538 | 20011770 | PDFSTATSENSEMBLUCSC | 233 | 4 | 21830 | island | 0.281466 | 0.404211 | Not queried | 1.53 | 8.86e-01 | 8.89e-01 |
|  | 12 | 64845451 | 64845936 | PDFSTATSENSEMBLUCSC | 486 | 6 | 10595 | island | 0.006702 | 0.011172 | Not queried | 1.53 | 4.85e-01 | 5.11e-01 |
|  | 16 | 88717464 | 88717989 | PDFSTATSENSEMBLUCSC | 526 | 11 | 15410 | tss | 0.595808 | 0.228342 | CYBA; MVD (margin: IL17C; SNAI3-AS1; CYBA; MVD) | 1.53 | 1.04e-02 | 3.96e-02 |
|  | 7 | 27197455 | 27198429 | PDFSTATSENSEMBLUCSC | 975 | 10 | 38354 | island | 0.897687 | 0.985365 | HOXA-AS3; RP1-170O19.21; HOXA7 (margin: HOXA-AS3; RP1-170O19.21; HOXA-AS4; HOXA3; HOXA6; HOXA7; HOXA9; RP1-170O19.20; MIR196B; HOXA10) | 1.53 | 1.15e-02 | 4.29e-02 |
|  | 15 | 35047023 | 35047411 | PDFSTATSENSEMBLUCSC | 389 | 6 | 13564 | tss;island | 0.347182 | 0.535874 | Not queried | 1.53 | 3.94e-01 | 4.36e-01 |
|  | 17 | 33569972 | 33570047 | PDFSTATSENSEMBLUCSC | 76 | 7 | 17266 | tss | 0.005710 | 0.002578 | Not queried | 1.53 | 1.65e-01 | 2.28e-01 |
|  | 19 | 11849777 | 11850013 | PDFSTATSENSEMBLUCSC | 237 | 7 | 20480 | tss | 0.002378 | 0.005258 | Not queried | 1.53 | 1.65e-01 | 2.28e-01 |
|  | 14 | 93260599 | 93260913 | PDFSTATSENSEMBLUCSC | 315 | 4 | 13092 | island | 0.006469 | 0.018895 | Not queried | 1.53 | 4.86e-01 | 5.11e-01 |
|  | 14 | 23563715 | 23564120 | PDFSTATSENSEMBLUCSC | 406 | 4 | 12025 | gene;tss;island | 0.002764 | 0.006516 | Not queried | 1.53 | 4.86e-01 | 5.11e-01 |
|  | 5 | 851101 | 851458 | PDFSTATSENSEMBLUCSC | 358 | 6 | 31962 | tss;island | 0.423259 | 0.754231 | Not queried | 1.53 | 2.60e-02 | 6.12e-02 |
|  | 17 | 58227181 | 58227342 | PDFSTATSENSEMBLUCSC | 162 | 4 | 18620 | island | 0.732266 | 0.861681 | Not queried | 1.53 | 2.00e-01 | 2.55e-01 |
|  | 10 | 43697817 | 43698141 | PDFSTATSENSEMBLUCSC | 325 | 5 | 4568 | gene;island | 0.289106 | 0.145800 | Not queried | 1.53 | 3.10e-01 | 3.66e-01 |
|  | 11 | 777775 | 778018 | PDFSTATSENSEMBLUCSC | 244 | 4 | 7026 | tss;island | 0.005455 | 0.011484 | Not queried | 1.53 | 6.86e-01 | 6.99e-01 |
|  | 18 | 44337664 | 44338147 | PDFSTATSENSEMBLUCSC | 484 | 6 | 19259 | tss;island | 0.917144 | 0.978672 | Not queried | 1.53 | 9.31e-02 | 1.53e-01 |
|  | 6 | 43655346 | 43655489 | PDFSTATSENSEMBLUCSC | 144 | 4 | 34353 | gene | 0.090207 | 0.012908 | Not queried | 1.53 | 3.43e-01 | 3.84e-01 |
|  | 1 | 110880366 | 110880555 | PDFSTATSENSEMBLUCSC | 190 | 4 | 3578 | island | 0.003025 | 0.005942 | Not queried | 1.53 | 3.43e-01 | 3.84e-01 |
|  | 16 | 56691551 | 56691849 | PDFSTATSENSEMBLUCSC | 299 | 7 | 15250 | tss | 0.020208 | 0.007629 | Not queried | 1.53 | 3.18e-01 | 3.74e-01 |
|  | 8 | 143761503 | 143761811 | PDFSTATSENSEMBLUCSC | 309 | 6 | 39932 | tss | 0.092710 | 0.099884 | Not queried | 1.53 | 4.85e-01 | 5.11e-01 |
|  | 19 | 16999576 | 16999953 | PDFSTATSENSEMBLUCSC | 378 | 7 | 21726 | island | 0.828084 | 0.920960 | Not queried | 1.53 | 1.28e-01 | 1.87e-01 |
|  | 16 | 3074170 | 3074537 | PDFSTATSENSEMBLUCSC | 368 | 10 | 14621 | gene;island | 0.002740 | 0.006157 | Not queried | 1.53 | 7.53e-02 | 1.27e-01 |
|  | 16 | 3074294 | 3074537 | PDFSTATSENSEMBLUCSC | 244 | 8 | 15047 | tss | 0.002740 | 0.006157 | Not queried | 1.53 | 1.95e-01 | 2.55e-01 |
|  | 6 | 27832728 | 27833555 | PDFSTATSENSEMBLUCSC | 828 | 13 | 35570 | island | 0.002108 | 0.004270 | Not queried | 1.53 | 2.04e-01 | 2.59e-01 |
|  | 6 | 27833121 | 27833555 | PDFSTATSENSEMBLUCSC | 435 | 5 | 33622 | gene | 0.005106 | 0.007038 | Not queried | 1.53 | 4.21e-01 | 4.59e-01 |
|  | 6 | 135501674 | 135501969 | PDFSTATSENSEMBLUCSC | 296 | 4 | 35237 | tss;island | 0.011519 | 0.005831 | Not queried | 1.53 | 6.86e-01 | 6.99e-01 |
|  | 4 | 72897565 | 72897868 | PDFSTATSENSEMBLUCSC | 304 | 5 | 30019 | gene;island | 0.778552 | 0.552389 | Not queried | 1.53 | 9.52e-02 | 1.53e-01 |
|  | 14 | 102605829 | 102606318 | PDFSTATSENSEMBLUCSC | 490 | 8 | 12227 | gene;tss;island | 0.007156 | 0.012433 | Not queried | 1.53 | 1.61e-01 | 2.24e-01 |
|  | 7 | 99698799 | 99699161 | PDFSTATSENSEMBLUCSC | 363 | 5 | 37136 | gene;tss;island | 0.003618 | 0.008022 | Not queried | 1.53 | 2.22e-01 | 2.76e-01 |
|  | 10 | 133449506 | 133449715 | PDFSTATSENSEMBLUCSC | 210 | 4 | 6277 | island | 0.718371 | 0.863626 | Not queried | 1.53 | 2.00e-01 | 2.55e-01 |
|  | 1 | 33367304 | 33367416 | PDFSTATSENSEMBLUCSC | 113 | 4 | 1466 | tss;island | 0.142255 | 0.050869 | Not queried | 1.53 | 6.86e-01 | 6.99e-01 |
|  | 19 | 55173482 | 55173527 | PDFSTATSENSEMBLUCSC | 46 | 4 | 21064 | tss | 0.680996 | 0.874692 | Not queried | 1.53 | 2.00e-01 | 2.55e-01 |
|  | 5 | 71014736 | 71014918 | PDFSTATSENSEMBLUCSC | 183 | 7 | 32115 | tss;island | 0.502300 | 0.788064 | Not queried | 1.53 | 1.28e-01 | 1.87e-01 |
|  | 3 | 192127327 | 192127457 | PDFSTATSENSEMBLUCSC | 131 | 4 | 28086 | gene;tss;island | 0.702353 | 0.470440 | Not queried | 1.53 | 5.71e-02 | 1.00e-01 |
|  | 6 | 32032689 | 32033006 | PDFSTATSENSEMBLUCSC | 318 | 10 | 34028 | gene | 0.808840 | 0.900988 | Not queried | 1.53 | 5.29e-01 | 5.56e-01 |
|  | 3 | 132757267 | 132757973 | PDFSTATSENSEMBLUCSC | 707 | 7 | 27952 | gene | 0.648963 | 0.818469 | Not queried | 1.53 | 2.59e-01 | 3.16e-01 |
|  | 10 | 37414506 | 37414768 | PDFSTATSENSEMBLUCSC | 263 | 6 | 5031 | tss | 0.862485 | 0.963592 | Not queried | 1.53 | 1.32e-01 | 1.89e-01 |
|  | 17 | 6796745 | 6797119 | PDFSTATSENSEMBLUCSC | 375 | 5 | 16378 | gene;island | 0.878551 | 0.954190 | Not queried | 1.53 | 1.51e-01 | 2.11e-01 |
|  | 8 | 143859143 | 143859410 | PDFSTATSENSEMBLUCSC | 268 | 5 | 39469 | gene;tss;island | 0.940767 | 0.982508 | LYNX1 (margin: RP11-706C16.7; RP11-706C16.8; LYNX1; LY6D) | 1.53 | 7.94e-03 | 3.19e-02 |
|  | 12 | 104359248 | 104359732 | PDFSTATSENSEMBLUCSC | 485 | 8 | 10734 | island | 0.002736 | 0.006530 | Not queried | 1.53 | 2.79e-01 | 3.38e-01 |
|  | 19 | 56739731 | 56739868 | PDFSTATSENSEMBLUCSC | 138 | 6 | 21094 | tss | 0.951986 | 0.983973 | Not queried | 1.53 | 1.52e-02 | 5.36e-02 |
|  | 6 | 44191036 | 44191312 | PDFSTATSENSEMBLUCSC | 277 | 8 | 35008 | tss | 0.009537 | 0.008881 | Not queried | 1.53 | 5.05e-01 | 5.32e-01 |
|  | 6 | 74103959 | 74104137 | PDFSTATSENSEMBLUCSC | 179 | 4 | 35069 | tss;island | 0.988716 | 0.960676 | Not queried | 1.53 | 6.86e-01 | 6.99e-01 |
|  | 15 | 25301783 | 25301950 | PDFSTATSENSEMBLUCSC | 168 | 4 | 13521 | tss | 0.879104 | 0.698009 | Not queried | 1.53 | 4.86e-01 | 5.11e-01 |
|  | 6 | 32044104 | 32044496 | PDFSTATSENSEMBLUCSC | 393 | 8 | 34033 | gene;island | 0.680519 | 0.939403 | Not queried | 1.52 | 4.42e-01 | 4.81e-01 |
|  | 11 | 75946680 | 75946986 | PDFSTATSENSEMBLUCSC | 307 | 6 | 8669 | island | 0.003143 | 0.006270 | Not queried | 1.52 | 4.85e-01 | 5.11e-01 |
|  | 1 | 65209468 | 65209572 | PDFSTATSENSEMBLUCSC | 105 | 4 | 1673 | tss;island | 0.967472 | 0.956127 | Not queried | 1.52 | 6.86e-01 | 6.99e-01 |
|  | 4 | 8442223 | 8442666 | PDFSTATSENSEMBLUCSC | 444 | 10 | 30921 | island | 0.005559 | 0.009598 | Not queried | 1.52 | 1.85e-02 | 6.12e-02 |
|  | 10 | 90639684 | 90640067 | PDFSTATSENSEMBLUCSC | 384 | 10 | 5917 | island | 0.005656 | 0.008857 | Not queried | 1.52 | 1.05e-01 | 1.66e-01 |
|  | 10 | 90639684 | 90640001 | PDFSTATSENSEMBLUCSC | 318 | 8 | 5207 | tss | 0.006692 | 0.010836 | Not queried | 1.52 | 1.95e-01 | 2.55e-01 |
|  | 12 | 120703729 | 120704006 | PDFSTATSENSEMBLUCSC | 278 | 4 | 10056 | tss;island | 0.248490 | 0.525995 | Not queried | 1.52 | 6.86e-01 | 6.99e-01 |
|  | 15 | 95869918 | 95870475 | PDFSTATSENSEMBLUCSC | 558 | 9 | 14484 | island | 0.867446 | 0.956379 | CTD-2536I1.1 (margin: CTD-2536I1.1) | 1.52 | 7.77e-03 | 3.19e-02 |
|  | 5 | 142077712 | 142078020 | PDFSTATSENSEMBLUCSC | 309 | 4 | 32400 | tss | 0.435568 | 0.789938 | Not queried | 1.52 | 4.86e-01 | 5.11e-01 |
|  | 20 | 33680648 | 33681223 | PDFSTATSENSEMBLUCSC | 576 | 11 | 25700 | tss | 0.007824 | 0.012397 | Not queried | 1.52 | 2.70e-01 | 3.29e-01 |
|  | 7 | 106505688 | 106505914 | PDFSTATSENSEMBLUCSC | 227 | 4 | 37822 | tss | 0.915775 | 0.969722 | Not queried | 1.52 | 4.86e-01 | 5.11e-01 |
|  | 7 | 89783435 | 89783976 | PDFSTATSENSEMBLUCSC | 542 | 10 | 38645 | island | 0.334559 | 0.041239 | Not queried | 1.52 | 2.32e-02 | 6.12e-02 |
|  | 1 | 150529664 | 150529891 | PDFSTATSENSEMBLUCSC | 228 | 5 | 741 | gene;island | 0.972930 | 0.918250 | Not queried | 1.52 | 2.22e-01 | 2.76e-01 |
|  | 7 | 104653783 | 104654258 | PDFSTATSENSEMBLUCSC | 476 | 5 | 37174 | gene;tss;island | 0.004489 | 0.014425 | Not queried | 1.52 | 3.10e-01 | 3.66e-01 |
|  | 12 | 120933726 | 120933845 | PDFSTATSENSEMBLUCSC | 120 | 6 | 10061 | tss | 0.008784 | 0.018224 | Not queried | 1.52 | 3.94e-01 | 4.36e-01 |
|  | 6 | 100054723 | 100055173 | PDFSTATSENSEMBLUCSC | 451 | 5 | 34427 | gene | 0.748517 | 0.950293 | Not queried | 1.52 | 5.48e-01 | 5.70e-01 |
|  | 7 | 44530236 | 44530730 | PDFSTATSENSEMBLUCSC | 495 | 11 | 38462 | island | 0.002862 | 0.006415 | Not queried | 1.52 | 1.71e-01 | 2.37e-01 |
|  | 18 | 3451447 | 3451564 | PDFSTATSENSEMBLUCSC | 118 | 7 | 19164 | tss | 0.003732 | 0.003985 | Not queried | 1.52 | 3.18e-01 | 3.74e-01 |
|  | 2 | 11809681 | 11810683 | PDFSTATSENSEMBLUCSC | 1003 | 12 | 24082 | island | 0.812279 | 0.460233 | Not queried | 1.52 | 1.43e-01 | 2.04e-01 |
|  | 7 | 139168305 | 139168804 | PDFSTATSENSEMBLUCSC | 500 | 9 | 38954 | island | 0.111230 | 0.021051 | Not queried | 1.52 | 1.90e-01 | 2.55e-01 |
|  | 12 | 49259714 | 49259997 | PDFSTATSENSEMBLUCSC | 284 | 5 | 9675 | tss | 0.029551 | 0.009569 | Not queried | 1.52 | 4.21e-01 | 4.59e-01 |
|  | 14 | 47120795 | 47121002 | PDFSTATSENSEMBLUCSC | 208 | 4 | 12075 | gene | 0.619615 | 0.845246 | Not queried | 1.52 | 6.86e-01 | 6.99e-01 |
|  | 12 | 52828203 | 52828633 | PDFSTATSENSEMBLUCSC | 431 | 5 | 9727 | tss | 0.822751 | 0.944188 | Not queried | 1.52 | 2.22e-01 | 2.76e-01 |
|  | 4 | 155337722 | 155338166 | PDFSTATSENSEMBLUCSC | 445 | 5 | 30157 | gene;island | 0.109724 | 0.048576 | Not queried | 1.52 | 4.21e-01 | 4.59e-01 |
|  | 12 | 128752040 | 128752356 | PDFSTATSENSEMBLUCSC | 317 | 4 | 9405 | gene;island | 0.969992 | 0.847510 | Not queried | 1.52 | 3.43e-01 | 3.84e-01 |
|  | 8 | 109455852 | 109456248 | PDFSTATSENSEMBLUCSC | 397 | 5 | 39396 | gene | 0.003725 | 0.010344 | Not queried | 1.52 | 3.10e-01 | 3.66e-01 |
|  | 19 | 3036221 | 3036484 | PDFSTATSENSEMBLUCSC | 264 | 4 | 19703 | gene;island | 0.004606 | 0.008892 | Not queried | 1.52 | 3.43e-01 | 3.84e-01 |
|  | 17 | 21743878 | 21744126 | PDFSTATSENSEMBLUCSC | 249 | 4 | 18128 | island | 0.667002 | 0.787985 | Not queried | 1.52 | 4.86e-01 | 5.11e-01 |
|  | 6 | 31165290 | 31166174 | PDFSTATSENSEMBLUCSC | 885 | 29 | 35756 | island | 0.008159 | 0.019397 | HCG27; XXbac-BPG299F13.17; XXbac-BPG299F13.14 (margin: HCG27; XXbac-BPG299F13.17; XXbac-BPG299F13.14) | 1.52 | 8.68e-03 | 3.37e-02 |
|  | 17 | 19280802 | 19281001 | PDFSTATSENSEMBLUCSC | 200 | 4 | 17188 | tss;island | 0.003231 | 0.010115 | Not queried | 1.52 | 1.14e-01 | 1.68e-01 |
|  | 6 | 29716108 | 29717656 | PDFSTATSENSEMBLUCSC | 1549 | 40 | 35625 | island | 0.033169 | 0.052111 | Not queried | 1.52 | 1.62e-01 | 2.26e-01 |
|  | 16 | 31119484 | 31119650 | PDFSTATSENSEMBLUCSC | 167 | 4 | 15206 | tss;island | 0.008296 | 0.008988 | Not queried | 1.52 | 8.86e-01 | 8.89e-01 |
|  | 5 | 139422525 | 139423077 | PDFSTATSENSEMBLUCSC | 553 | 8 | 33181 | island | 0.057616 | 0.016185 | Not queried | 1.52 | 1.05e-01 | 1.66e-01 |
|  | 15 | 65578830 | 65579557 | PDFSTATSENSEMBLUCSC | 728 | 10 | 14230 | island | 0.003013 | 0.007241 | Not queried | 1.52 | 4.36e-01 | 4.76e-01 |
|  | 17 | 8021572 | 8022155 | PDFSTATSENSEMBLUCSC | 584 | 8 | 16425 | gene | 0.002742 | 0.007597 | Not queried | 1.52 | 1.30e-01 | 1.89e-01 |
|  | 20 | 30062970 | 30063249 | PDFSTATSENSEMBLUCSC | 280 | 8 | 26071 | island | 0.550111 | 0.728374 | Not queried | 1.52 | 1.30e-01 | 1.89e-01 |
|  | 2 | 91777099 | 91777482 | PDFSTATSENSEMBLUCSC | 384 | 4 | 24508 | island | 0.549578 | 0.662718 | Not queried | 1.52 | 4.86e-01 | 5.11e-01 |
|  | 1 | 32930832 | 32931323 | PDFSTATSENSEMBLUCSC | 492 | 5 | 396 | gene;island | 0.437170 | 0.418544 | Not queried | 1.52 | 4.21e-01 | 4.59e-01 |
|  | 6 | 32158153 | 32158513 | PDFSTATSENSEMBLUCSC | 361 | 9 | 34870 | tss | 0.009005 | 0.038497 | Not queried | 1.52 | 2.22e-01 | 2.76e-01 |
|  | 16 | 29674903 | 29675214 | PDFSTATSENSEMBLUCSC | 312 | 5 | 14710 | gene;island | 0.661186 | 0.888961 | Not queried | 1.52 | 1.00e+00 | 1.00e+00 |
|  | 18 | 33077969 | 33078285 | PDFSTATSENSEMBLUCSC | 317 | 8 | 19238 | tss | 0.002122 | 0.004929 | Not queried | 1.52 | 3.82e-01 | 4.27e-01 |
|  | 11 | 2156282 | 2156699 | PDFSTATSENSEMBLUCSC | 418 | 6 | 6446 | gene;tss;island | 0.771093 | 0.940829 | Not queried | 1.51 | 1.32e-01 | 1.89e-01 |
|  | 5 | 41925698 | 41925854 | PDFSTATSENSEMBLUCSC | 157 | 4 | 31600 | gene;island | 0.002409 | 0.009173 | Not queried | 1.51 | 6.86e-01 | 6.99e-01 |
|  | 2 | 136876652 | 136877039 | PDFSTATSENSEMBLUCSC | 388 | 5 | 23650 | tss;island | 0.453684 | 0.226879 | Not queried | 1.51 | 3.17e-02 | 6.50e-02 |
|  | 17 | 79045526 | 79045877 | PDFSTATSENSEMBLUCSC | 352 | 5 | 16891 | gene;island | 0.858069 | 0.967106 | Not queried | 1.51 | 3.10e-01 | 3.66e-01 |
|  | 12 | 54385275 | 54385625 | PDFSTATSENSEMBLUCSC | 351 | 5 | 10487 | island | 0.839393 | 0.936979 | Not queried | 1.51 | 9.52e-02 | 1.53e-01 |
|  | 1 | 1242730 | 1243003 | PDFSTATSENSEMBLUCSC | 274 | 6 | 28 | gene;tss;island | 0.023892 | 0.009150 | Not queried | 1.51 | 2.40e-01 | 2.95e-01 |
|  | 2 | 66659348 | 66659590 | PDFSTATSENSEMBLUCSC | 243 | 4 | 24355 | island | 0.906343 | 0.843292 | Not queried | 1.51 | 6.86e-01 | 6.99e-01 |
|  | 3 | 189348936 | 189349129 | PDFSTATSENSEMBLUCSC | 194 | 4 | 28782 | tss | 0.967742 | 0.956473 | Not queried | 1.51 | 6.86e-01 | 6.99e-01 |
|  | 1 | 150266158 | 150266242 | PDFSTATSENSEMBLUCSC | 85 | 4 | 1906 | tss | 0.008054 | 0.019641 | Not queried | 1.51 | 4.86e-01 | 5.11e-01 |
|  | 17 | 48649219 | 48649370 | PDFSTATSENSEMBLUCSC | 152 | 4 | 16690 | gene;island | 0.710885 | 0.884438 | Not queried | 1.51 | 4.86e-01 | 5.11e-01 |
|  | 1 | 29460817 | 29461070 | PDFSTATSENSEMBLUCSC | 254 | 4 | 2959 | island | 0.837209 | 0.947189 | Not queried | 1.51 | 5.71e-02 | 1.00e-01 |
|  | 14 | 45366330 | 45366564 | PDFSTATSENSEMBLUCSC | 235 | 6 | 12811 | island | 0.002559 | 0.008417 | Not queried | 1.51 | 2.40e-01 | 2.95e-01 |
|  | 19 | 52452317 | 52452528 | PDFSTATSENSEMBLUCSC | 212 | 4 | 22345 | island | 0.961872 | 0.894413 | Not queried | 1.51 | 3.43e-01 | 3.84e-01 |
|  | 15 | 57025816 | 57026064 | PDFSTATSENSEMBLUCSC | 249 | 5 | 13691 | tss;island | 0.682889 | 0.221646 | Not queried | 1.51 | 5.48e-01 | 5.70e-01 |
|  | 3 | 8810980 | 8811758 | PDFSTATSENSEMBLUCSC | 779 | 8 | 28851 | island | 0.201739 | 0.639219 | Not queried | 1.51 | 7.21e-01 | 7.30e-01 |
|  | 18 | 61144150 | 61144339 | PDFSTATSENSEMBLUCSC | 190 | 4 | 19121 | gene | 0.007739 | 0.058914 | Not queried | 1.51 | 3.43e-01 | 3.84e-01 |
|  | 9 | 136243334 | 136243698 | PDFSTATSENSEMBLUCSC | 365 | 4 | 40791 | gene | 0.323309 | 0.140348 | Not queried | 1.51 | 2.00e-01 | 2.55e-01 |
|  | 9 | 136243027 | 136243698 | PDFSTATSENSEMBLUCSC | 672 | 6 | 40851 | tss;island | 0.170441 | 0.037237 | Not queried | 1.51 | 4.85e-01 | 5.11e-01 |
|  | 1 | 47909931 | 47910239 | PDFSTATSENSEMBLUCSC | 309 | 4 | 3240 | island | 0.874371 | 0.950552 | Not queried | 1.51 | 2.86e-02 | 6.12e-02 |
|  | 7 | 155167283 | 155167844 | PDFSTATSENSEMBLUCSC | 562 | 5 | 39064 | island | 0.916504 | 0.901230 | Not queried | 1.51 | 5.48e-01 | 5.70e-01 |
|  | 6 | 31370412 | 31371353 | PDFSTATSENSEMBLUCSC | 942 | 24 | 34808 | tss | 0.047045 | 0.433852 | Not queried | 1.51 | 2.71e-02 | 6.12e-02 |
|  | 2 | 55509105 | 55509334 | PDFSTATSENSEMBLUCSC | 230 | 5 | 23362 | tss | 0.030882 | 0.102996 | Not queried | 1.51 | 3.10e-01 | 3.66e-01 |
|  | 14 | 64009971 | 64010226 | PDFSTATSENSEMBLUCSC | 256 | 4 | 12922 | island | 0.002506 | 0.002935 | Not queried | 1.51 | 1.00e+00 | 1.00e+00 |
|  | 6 | 32825040 | 32825148 | PDFSTATSENSEMBLUCSC | 109 | 6 | 34158 | gene;island | 0.982013 | 0.926379 | Not queried | 1.51 | 1.52e-02 | 5.36e-02 |
|  | 5 | 1800069 | 1800184 | PDFSTATSENSEMBLUCSC | 116 | 5 | 31972 | tss;island | 0.004436 | 0.008402 | Not queried | 1.51 | 6.90e-01 | 7.01e-01 |
|  | 6 | 34393779 | 34394098 | PDFSTATSENSEMBLUCSC | 320 | 8 | 36059 | island | 0.000716 | 0.002005 | Not queried | 1.51 | 2.34e-01 | 2.90e-01 |
|  | 16 | 19421817 | 19421979 | PDFSTATSENSEMBLUCSC | 163 | 5 | 15115 | tss;island | 0.022881 | 0.062209 | Not queried | 1.51 | 2.22e-01 | 2.76e-01 |
|  | 20 | 54919037 | 54919410 | PDFSTATSENSEMBLUCSC | 374 | 6 | 26271 | island | 0.345158 | 0.176788 | Not queried | 1.51 | 1.80e-01 | 2.45e-01 |
|  | 6 | 31323253 | 31324024 | PDFSTATSENSEMBLUCSC | 772 | 11 | 33868 | gene;island | 0.490398 | 0.848552 | Not queried | 1.51 | 1.71e-01 | 2.37e-01 |
|  | 2 | 119916486 | 119916686 | PDFSTATSENSEMBLUCSC | 201 | 6 | 23591 | tss | 0.925480 | 0.954911 | Not queried | 1.51 | 2.40e-01 | 2.95e-01 |
|  | 2 | 105461096 | 105461392 | PDFSTATSENSEMBLUCSC | 297 | 4 | 24577 | island | 0.618223 | 0.802471 | Not queried | 1.51 | 8.86e-01 | 8.89e-01 |
|  | 6 | 166868856 | 166869078 | PDFSTATSENSEMBLUCSC | 223 | 4 | 34544 | gene;island | 0.768101 | 0.982123 | Not queried | 1.51 | 2.00e-01 | 2.55e-01 |
|  | 19 | 5536532 | 5536919 | PDFSTATSENSEMBLUCSC | 388 | 4 | 21406 | island | 0.802702 | 0.906722 | Not queried | 1.51 | 1.14e-01 | 1.68e-01 |
|  | 12 | 115120789 | 115121489 | PDFSTATSENSEMBLUCSC | 701 | 8 | 9327 | gene;island | 0.038448 | 0.016557 | Not queried | 1.51 | 8.30e-02 | 1.40e-01 |
|  | 1 | 2850512 | 2850951 | PDFSTATSENSEMBLUCSC | 440 | 5 | 2522 | island | 0.564526 | 0.860231 | Not queried | 1.51 | 9.52e-02 | 1.53e-01 |
|  | 17 | 26732120 | 26732374 | PDFSTATSENSEMBLUCSC | 255 | 5 | 16496 | gene;island | 0.841537 | 0.947631 | Not queried | 1.51 | 5.56e-02 | 1.00e-01 |
|  | 2 | 173291908 | 173292277 | PDFSTATSENSEMBLUCSC | 370 | 9 | 23719 | tss | 0.006574 | 0.007013 | Not queried | 1.51 | 9.31e-01 | 9.35e-01 |
|  | 7 | 120577 | 120801 | PDFSTATSENSEMBLUCSC | 225 | 5 | 38028 | island | 0.527227 | 0.767321 | Not queried | 1.51 | 2.22e-01 | 2.76e-01 |
|  | 15 | 71146679 | 71146902 | PDFSTATSENSEMBLUCSC | 224 | 5 | 13768 | tss;island | 0.025204 | 0.137223 | Not queried | 1.51 | 4.21e-01 | 4.59e-01 |
|  | 11 | 116700436 | 116700556 | PDFSTATSENSEMBLUCSC | 121 | 4 | 7713 | tss | 0.742118 | 0.845905 | Not queried | 1.50 | 1.14e-01 | 1.68e-01 |
|  | 11 | 118754280 | 118754535 | PDFSTATSENSEMBLUCSC | 256 | 5 | 7739 | tss | 0.911250 | 0.979423 | Not queried | 1.50 | 6.90e-01 | 7.01e-01 |
|  | 11 | 62572592 | 62572907 | PDFSTATSENSEMBLUCSC | 316 | 4 | 6648 | gene | 0.001354 | 0.004192 | Not queried | 1.50 | 4.86e-01 | 5.11e-01 |
|  | 1 | 65991461 | 65991765 | PDFSTATSENSEMBLUCSC | 305 | 4 | 571 | gene;island | 0.949539 | 0.976143 | Not queried | 1.50 | 3.43e-01 | 3.84e-01 |
|  | 1 | 109288968 | 109289295 | PDFSTATSENSEMBLUCSC | 328 | 7 | 3545 | island | 0.016743 | 0.010593 | Not queried | 1.50 | 3.18e-01 | 3.74e-01 |
|  | 1 | 150521190 | 150522367 | PDFSTATSENSEMBLUCSC | 1178 | 13 | 3723 | island | 0.019207 | 0.101772 | Not queried | 1.50 | 1.69e-01 | 2.33e-01 |
|  | 2 | 96067986 | 96068411 | PDFSTATSENSEMBLUCSC | 426 | 4 | 23511 | tss | 0.050732 | 0.026065 | Not queried | 1.50 | 6.86e-01 | 6.99e-01 |
|  | 7 | 1095416 | 1095720 | PDFSTATSENSEMBLUCSC | 305 | 6 | 36742 | gene;island | 0.782493 | 0.898929 | Not queried | 1.50 | 1.80e-01 | 2.45e-01 |
|  | 11 | 6704667 | 6704982 | PDFSTATSENSEMBLUCSC | 316 | 10 | 7117 | tss | 0.002321 | 0.004981 | Not queried | 1.50 | 1.23e-01 | 1.81e-01 |
|  | 8 | 69243285 | 69243904 | PDFSTATSENSEMBLUCSC | 620 | 7 | 40368 | island | 0.665932 | 0.518738 | Not queried | 1.50 | 1.28e-01 | 1.87e-01 |
|  | 19 | 1313407 | 1313642 | PDFSTATSENSEMBLUCSC | 236 | 4 | 21214 | island | 0.145402 | 0.203561 | Not queried | 1.50 | 3.43e-01 | 3.84e-01 |
|  | 16 | 14397576 | 14397766 | PDFSTATSENSEMBLUCSC | 191 | 5 | 15101 | tss | 0.006665 | 0.016490 | Not queried | 1.50 | 2.22e-01 | 2.76e-01 |
|  | 1 | 247614654 | 247614943 | PDFSTATSENSEMBLUCSC | 290 | 5 | 1126 | gene;island | 0.496808 | 0.647616 | Not queried | 1.50 | 5.48e-01 | 5.70e-01 |
|  | 1 | 156182812 | 156183160 | PDFSTATSENSEMBLUCSC | 349 | 4 | 817 | gene | 0.004449 | 0.011145 | Not queried | 1.50 | 2.00e-01 | 2.55e-01 |
|  | 5 | 140723331 | 140723583 | PDFSTATSENSEMBLUCSC | 253 | 6 | 32373 | tss | 0.865212 | 0.936584 | Not queried | 1.50 | 4.85e-01 | 5.11e-01 |
|  | 1 | 27286832 | 27287541 | PDFSTATSENSEMBLUCSC | 710 | 7 | 2907 | island | 0.018735 | 0.012516 | Not queried | 1.50 | 2.59e-01 | 3.16e-01 |
|  | 6 | 37322624 | 37322835 | PDFSTATSENSEMBLUCSC | 212 | 5 | 34322 | gene;island | 0.007286 | 0.016969 | Not queried | 1.50 | 1.51e-01 | 2.11e-01 |
|  | 1 | 201123560 | 201124117 | PDFSTATSENSEMBLUCSC | 558 | 9 | 4072 | island | 0.004537 | 0.116463 | Not queried | 1.50 | 4.36e-01 | 4.76e-01 |
|  | 11 | 61659326 | 61659518 | PDFSTATSENSEMBLUCSC | 193 | 4 | 7342 | tss;island | 0.342810 | 0.100744 | Not queried | 1.50 | 2.00e-01 | 2.55e-01 |
|  | 13 | 21140606 | 21140776 | PDFSTATSENSEMBLUCSC | 171 | 4 | 11333 | tss;island | 0.009006 | 0.005378 | Not queried | 1.50 | 6.86e-01 | 6.99e-01 |
|  | 4 | 2936973 | 2937381 | PDFSTATSENSEMBLUCSC | 409 | 6 | 30256 | tss;island | 0.011413 | 0.031438 | Not queried | 1.50 | 1.80e-01 | 2.45e-01 |
|  | 6 | 32810551 | 32810833 | PDFSTATSENSEMBLUCSC | 283 | 5 | 34150 | gene;island | 0.969074 | 0.929476 | Not queried | 1.50 | 8.41e-01 | 8.48e-01 |
|  | 4 | 68411154 | 68411531 | PDFSTATSENSEMBLUCSC | 378 | 10 | 31080 | island | 0.002961 | 0.008554 | Not queried | 1.50 | 3.15e-01 | 3.72e-01 |
|  | 18 | 3451447 | 3451750 | PDFSTATSENSEMBLUCSC | 304 | 9 | 19042 | gene;island | 0.003732 | 0.003985 | Not queried | 1.50 | 2.97e-01 | 3.60e-01 |
|  | 14 | 89029552 | 89029605 | PDFSTATSENSEMBLUCSC | 54 | 5 | 12546 | tss | 0.010860 | 0.004413 | Not queried | 1.50 | 2.22e-01 | 2.76e-01 |
|  | 4 | 82393069 | 82393369 | PDFSTATSENSEMBLUCSC | 301 | 8 | 30464 | tss | 0.064561 | 0.023399 | RASGEF1B (margin: RASGEF1B) | 1.50 | 6.99e-03 | 3.19e-02 |
|  | 11 | 44330731 | 44331108 | PDFSTATSENSEMBLUCSC | 378 | 4 | 6590 | gene;island | 0.925318 | 0.983341 | Not queried | 1.50 | 4.86e-01 | 5.11e-01 |
|  | 19 | 58220295 | 58220837 | PDFSTATSENSEMBLUCSC | 543 | 10 | 22480 | island | 0.951825 | 0.985694 | AC003006.7; ZNF154 (margin: AC003006.7; ZNF154; ZNF671) | 1.50 | 2.09e-03 | 1.45e-02 |
|  | 4 | 121992988 | 121993378 | PDFSTATSENSEMBLUCSC | 391 | 5 | 30111 | gene;island | 0.884990 | 0.847060 | Not queried | 1.50 | 5.48e-01 | 5.70e-01 |
|  | 13 | 42846072 | 42846375 | PDFSTATSENSEMBLUCSC | 304 | 5 | 11695 | island | 0.004999 | 0.013419 | Not queried | 1.50 | 3.10e-01 | 3.66e-01 |
|  | 17 | 58603597 | 58603815 | PDFSTATSENSEMBLUCSC | 219 | 8 | 17553 | tss | 0.004358 | 0.012279 | Not queried | 1.50 | 3.28e-01 | 3.84e-01 |
|  | 13 | 41635698 | 41636195 | PDFSTATSENSEMBLUCSC | 498 | 4 | 11145 | gene | 0.004064 | 0.011820 | Not queried | 1.50 | 4.86e-01 | 5.11e-01 |
|  | 8 | 120219902 | 120220131 | PDFSTATSENSEMBLUCSC | 230 | 7 | 39885 | tss;island | 0.004960 | 0.040010 | Not queried | 1.50 | 1.65e-01 | 2.28e-01 |
|  | 19 | 54466187 | 54466724 | PDFSTATSENSEMBLUCSC | 538 | 6 | 22391 | island | 0.782738 | 0.960974 | Not queried | 1.50 | 4.85e-01 | 5.11e-01 |
|  | 5 | 49736988 | 49737492 | PDFSTATSENSEMBLUCSC | 505 | 7 | 32784 | island | 0.012437 | 0.140258 | Not queried | 1.50 | 2.59e-01 | 3.16e-01 |
|  | 1 | 160336475 | 160336712 | PDFSTATSENSEMBLUCSC | 238 | 5 | 2039 | tss | 0.653865 | 0.607374 | Not queried | 1.50 | 6.90e-01 | 7.01e-01 |
|  | 1 | 91190005 | 91190620 | PDFSTATSENSEMBLUCSC | 616 | 7 | 3461 | island | 0.965645 | 0.980168 | Not queried | 1.50 | 1.65e-01 | 2.28e-01 |
|  | 12 | 110149962 | 110150261 | PDFSTATSENSEMBLUCSC | 300 | 4 | 10783 | island | 0.782017 | 0.896608 | Not queried | 1.50 | 4.86e-01 | 5.11e-01 |
|  | 8 | 42234724 | 42234975 | PDFSTATSENSEMBLUCSC | 252 | 5 | 39678 | tss | 0.934859 | 0.960173 | Not queried | 1.50 | 4.21e-01 | 4.59e-01 |
|  | 6 | 170190914 | 170191082 | PDFSTATSENSEMBLUCSC | 169 | 4 | 34580 | gene;island | 0.628336 | 0.930166 | Not queried | 1.50 | 3.43e-01 | 3.84e-01 |
|  | 1 | 115396277 | 115396629 | PDFSTATSENSEMBLUCSC | 353 | 4 | 1856 | tss;island | 0.808959 | 0.926340 | Not queried | 1.50 | 5.71e-02 | 1.00e-01 |
|  | 16 | 70557093 | 70557429 | PDFSTATSENSEMBLUCSC | 337 | 5 | 14828 | gene | 0.003242 | 0.009044 | Not queried | 1.50 | 2.22e-01 | 2.76e-01 |
|  | 8 | 88886301 | 88886432 | PDFSTATSENSEMBLUCSC | 132 | 6 | 39801 | tss | 0.837280 | 0.981794 | Not queried | 1.50 | 1.32e-01 | 1.89e-01 |
|  | 4 | 169401687 | 169401797 | PDFSTATSENSEMBLUCSC | 111 | 4 | 30672 | tss | 0.001287 | 0.006132 | Not queried | 1.50 | 6.86e-01 | 6.99e-01 |
|  | 2 | 152118408 | 152118835 | PDFSTATSENSEMBLUCSC | 428 | 8 | 23658 | tss;island | 0.008023 | 0.021334 | Not queried | 1.49 | 4.42e-01 | 4.81e-01 |
|  | 19 | 49977222 | 49977483 | PDFSTATSENSEMBLUCSC | 262 | 7 | 20947 | tss | 0.008161 | 0.019813 | Not queried | 1.49 | 3.83e-01 | 4.27e-01 |
|  | 3 | 194117018 | 194117403 | PDFSTATSENSEMBLUCSC | 386 | 4 | 28090 | gene;island | 0.583068 | 0.886885 | Not queried | 1.49 | 1.14e-01 | 1.68e-01 |
|  | 10 | 13141779 | 13141924 | PDFSTATSENSEMBLUCSC | 146 | 6 | 4954 | tss;island | 0.007367 | 0.021906 | Not queried | 1.49 | 6.49e-02 | 1.11e-01 |
|  | 10 | 102881114 | 102881545 | PDFSTATSENSEMBLUCSC | 432 | 6 | 4723 | gene;island | 0.741141 | 0.928514 | Not queried | 1.49 | 1.32e-01 | 1.89e-01 |
|  | 6 | 117086850 | 117087238 | PDFSTATSENSEMBLUCSC | 389 | 8 | 36433 | island | 0.492648 | 0.548793 | Not queried | 1.49 | 5.74e-01 | 5.97e-01 |
|  | 11 | 117800233 | 117800571 | PDFSTATSENSEMBLUCSC | 339 | 5 | 7724 | tss | 0.022266 | 0.019252 | Not queried | 1.49 | 8.41e-01 | 8.48e-01 |
|  | 4 | 109093158 | 109093430 | PDFSTATSENSEMBLUCSC | 273 | 5 | 30086 | gene;island | 0.591064 | 0.761322 | Not queried | 1.49 | 9.52e-02 | 1.53e-01 |
|  | 1 | 247802703 | 247803166 | PDFSTATSENSEMBLUCSC | 464 | 7 | 4420 | island | 0.552823 | 0.796758 | Not queried | 1.49 | 8.05e-01 | 8.14e-01 |
|  | 7 | 100209537 | 100210097 | PDFSTATSENSEMBLUCSC | 561 | 13 | 37788 | tss | 0.005597 | 0.018624 | Not queried | 1.49 | 9.08e-02 | 1.52e-01 |
|  | 3 | 186334993 | 186335202 | PDFSTATSENSEMBLUCSC | 210 | 4 | 28074 | gene | 0.004439 | 0.024511 | Not queried | 1.49 | 3.43e-01 | 3.84e-01 |
|  | 1 | 95392925 | 95393138 | PDFSTATSENSEMBLUCSC | 214 | 4 | 1767 | tss;island | 0.776528 | 0.972847 | Not queried | 1.49 | 2.00e-01 | 2.55e-01 |
|  | 7 | 56019372 | 56019579 | PDFSTATSENSEMBLUCSC | 208 | 8 | 37605 | tss;island | 0.004617 | 0.003698 | Not queried | 1.49 | 2.34e-01 | 2.90e-01 |
|  | 3 | 52273291 | 52273525 | PDFSTATSENSEMBLUCSC | 235 | 6 | 28374 | tss;island | 0.001519 | 0.005800 | Not queried | 1.49 | 3.94e-01 | 4.36e-01 |
|  | 12 | 53491483 | 53491850 | PDFSTATSENSEMBLUCSC | 368 | 4 | 9146 | gene;island | 0.009477 | 0.013454 | Not queried | 1.49 | 1.00e+00 | 1.00e+00 |
|  | 4 | 55095282 | 55095529 | PDFSTATSENSEMBLUCSC | 248 | 4 | 30004 | gene | 0.658378 | 0.495462 | Not queried | 1.49 | 6.86e-01 | 6.99e-01 |
|  | 16 | 56691551 | 56692077 | PDFSTATSENSEMBLUCSC | 527 | 10 | 15953 | island | 0.036332 | 0.017888 | Not queried | 1.49 | 3.53e-01 | 3.95e-01 |
|  | 22 | 45664617 | 45664817 | PDFSTATSENSEMBLUCSC | 201 | 4 | 27572 | island | 0.022014 | 0.007215 | Not queried | 1.49 | 6.86e-01 | 6.99e-01 |
|  | 19 | 12792705 | 12792793 | PDFSTATSENSEMBLUCSC | 89 | 5 | 20504 | tss;island | 0.002359 | 0.008057 | Not queried | 1.49 | 3.10e-01 | 3.66e-01 |
|  | 1 | 43317080 | 43317191 | PDFSTATSENSEMBLUCSC | 112 | 4 | 470 | gene | 0.948976 | 0.985958 | Not queried | 1.49 | 2.00e-01 | 2.55e-01 |
|  | 1 | 115322961 | 115323269 | PDFSTATSENSEMBLUCSC | 309 | 4 | 702 | gene | 0.004562 | 0.015304 | Not queried | 1.49 | 2.00e-01 | 2.55e-01 |
|  | 6 | 30908579 | 30908739 | PDFSTATSENSEMBLUCSC | 161 | 4 | 34795 | tss | 0.634850 | 0.770994 | Not queried | 1.49 | 6.86e-01 | 6.99e-01 |
|  | 20 | 14318152 | 14318410 | PDFSTATSENSEMBLUCSC | 259 | 6 | 25362 | gene | 0.009160 | 0.005385 | Not queried | 1.49 | 4.85e-01 | 5.11e-01 |
|  | 18 | 5544031 | 5544237 | PDFSTATSENSEMBLUCSC | 207 | 8 | 19171 | tss | 0.884738 | 0.655106 | Not queried | 1.49 | 2.79e-01 | 3.38e-01 |
|  | 11 | 33061388 | 33061907 | PDFSTATSENSEMBLUCSC | 520 | 5 | 6573 | gene;island | 0.006266 | 0.018513 | Not queried | 1.49 | 6.90e-01 | 7.01e-01 |
|  | 16 | 2892766 | 2892994 | PDFSTATSENSEMBLUCSC | 229 | 6 | 15038 | tss | 0.941023 | 0.905370 | Not queried | 1.49 | 3.94e-01 | 4.36e-01 |
|  | 5 | 176827671 | 176827833 | PDFSTATSENSEMBLUCSC | 163 | 4 | 32527 | tss;island | 0.933160 | 0.983911 | Not queried | 1.49 | 3.43e-01 | 3.84e-01 |
|  | 21 | 44869682 | 44869885 | PDFSTATSENSEMBLUCSC | 204 | 5 | 26575 | tss | 0.953840 | 0.898941 | Not queried | 1.49 | 1.51e-01 | 2.11e-01 |
|  | 13 | 52703328 | 52703615 | PDFSTATSENSEMBLUCSC | 288 | 7 | 11457 | tss | 0.171092 | 0.090519 | Not queried | 1.49 | 1.65e-01 | 2.28e-01 |
|  | 12 | 99037846 | 99038577 | PDFSTATSENSEMBLUCSC | 732 | 14 | 9260 | gene | 0.004704 | 0.011105 | Not queried | 1.49 | 5.56e-02 | 1.00e-01 |
|  | 17 | 26926457 | 26926868 | PDFSTATSENSEMBLUCSC | 412 | 6 | 17214 | tss;island | 0.009756 | 0.035091 | Not queried | 1.49 | 1.32e-01 | 1.89e-01 |
|  | 1 | 36235406 | 36235538 | PDFSTATSENSEMBLUCSC | 133 | 5 | 423 | gene | 0.007364 | 0.009141 | Not queried | 1.49 | 6.90e-01 | 7.01e-01 |
|  | 19 | 2543602 | 2543877 | PDFSTATSENSEMBLUCSC | 276 | 5 | 19691 | gene;island | 0.001886 | 0.016673 | Not queried | 1.49 | 2.22e-01 | 2.76e-01 |
|  | 12 | 100594586 | 100594894 | PDFSTATSENSEMBLUCSC | 309 | 5 | 9264 | gene | 0.004047 | 0.009401 | Not queried | 1.49 | 3.10e-01 | 3.66e-01 |
|  | 6 | 34123215 | 34123609 | PDFSTATSENSEMBLUCSC | 395 | 5 | 36051 | island | 0.890997 | 0.957464 | Not queried | 1.49 | 3.10e-01 | 3.66e-01 |
|  | 6 | 10434761 | 10434975 | PDFSTATSENSEMBLUCSC | 215 | 4 | 33563 | gene | 0.519103 | 0.860124 | Not queried | 1.49 | 1.14e-01 | 1.68e-01 |
|  | 2 | 27274131 | 27274487 | PDFSTATSENSEMBLUCSC | 357 | 7 | 23250 | tss | 0.006358 | 0.013837 | Not queried | 1.49 | 2.59e-01 | 3.16e-01 |
|  | 13 | 79181285 | 79181613 | PDFSTATSENSEMBLUCSC | 329 | 4 | 11795 | island | 0.821925 | 0.921568 | Not queried | 1.49 | 4.86e-01 | 5.11e-01 |
|  | 7 | 32111001 | 32111453 | PDFSTATSENSEMBLUCSC | 453 | 7 | 37524 | tss | 0.760658 | 0.444398 | Not queried | 1.49 | 1.28e-01 | 1.87e-01 |
|  | 12 | 100660633 | 100660829 | PDFSTATSENSEMBLUCSC | 197 | 5 | 9265 | gene | 0.004050 | 0.008876 | Not queried | 1.49 | 1.51e-01 | 2.11e-01 |
|  | 1 | 3620638 | 3621048 | PDFSTATSENSEMBLUCSC | 411 | 5 | 140 | gene;island | 0.825024 | 0.945494 | Not queried | 1.49 | 2.22e-01 | 2.76e-01 |
|  | 19 | 18723491 | 18723569 | PDFSTATSENSEMBLUCSC | 79 | 4 | 20625 | tss;island | 0.385409 | 0.109117 | Not queried | 1.49 | 2.00e-01 | 2.55e-01 |
|  | 17 | 37894014 | 37894413 | PDFSTATSENSEMBLUCSC | 400 | 7 | 17316 | tss | 0.007595 | 0.005168 | Not queried | 1.49 | 4.56e-01 | 4.96e-01 |
|  | 3 | 160823231 | 160823505 | PDFSTATSENSEMBLUCSC | 275 | 7 | 28697 | tss;island | 0.629692 | 0.452414 | Not queried | 1.49 | 5.30e-02 | 1.00e-01 |
|  | 12 | 99039011 | 99039295 | PDFSTATSENSEMBLUCSC | 285 | 6 | 9937 | tss;island | 0.006440 | 0.023594 | Not queried | 1.49 | 5.89e-01 | 6.11e-01 |
|  | 10 | 5488225 | 5488628 | PDFSTATSENSEMBLUCSC | 404 | 8 | 4477 | gene;island | 0.015767 | 0.061159 | Not queried | 1.48 | 1.61e-01 | 2.24e-01 |
|  | 7 | 15726348 | 15726821 | PDFSTATSENSEMBLUCSC | 474 | 7 | 37450 | tss | 0.870108 | 0.600191 | Not queried | 1.48 | 4.56e-01 | 4.96e-01 |
|  | 11 | 46383031 | 46383209 | PDFSTATSENSEMBLUCSC | 179 | 4 | 6599 | gene | 0.899909 | 0.775645 | Not queried | 1.48 | 2.00e-01 | 2.55e-01 |
|  | 7 | 128809100 | 128809442 | PDFSTATSENSEMBLUCSC | 343 | 5 | 38906 | island | 0.909393 | 0.964072 | TSPAN33; RP11-286H14.6 (margin: TSPAN33; Y\_RNA; RP11-286H14.6) | 1.48 | 7.94e-03 | 3.19e-02 |
|  | 7 | 152456579 | 152456815 | PDFSTATSENSEMBLUCSC | 237 | 5 | 38007 | tss;island | 0.006698 | 0.006125 | Not queried | 1.48 | 6.90e-01 | 7.01e-01 |
|  | 17 | 48502923 | 48503406 | PDFSTATSENSEMBLUCSC | 484 | 5 | 17499 | tss;island | 0.472598 | 0.216503 | Not queried | 1.48 | 4.21e-01 | 4.59e-01 |
|  | 18 | 56886915 | 56887351 | PDFSTATSENSEMBLUCSC | 437 | 9 | 19298 | tss | 0.382638 | 0.860368 | Not queried | 1.48 | 2.22e-01 | 2.76e-01 |
|  | 17 | 21496125 | 21496299 | PDFSTATSENSEMBLUCSC | 175 | 4 | 18127 | island | 0.635321 | 0.908015 | Not queried | 1.48 | 3.43e-01 | 3.84e-01 |
|  | 18 | 24130267 | 24130633 | PDFSTATSENSEMBLUCSC | 367 | 4 | 19081 | gene;tss;island | 0.154349 | 0.335176 | Not queried | 1.48 | 3.43e-01 | 3.84e-01 |
|  | 3 | 111852156 | 111852325 | PDFSTATSENSEMBLUCSC | 170 | 4 | 28500 | tss | 0.275788 | 0.434184 | Not queried | 1.48 | 4.86e-01 | 5.11e-01 |
|  | 15 | 65579119 | 65579557 | PDFSTATSENSEMBLUCSC | 439 | 7 | 13742 | tss | 0.002911 | 0.003053 | Not queried | 1.48 | 7.10e-01 | 7.20e-01 |
|  | 4 | 119200017 | 119200372 | PDFSTATSENSEMBLUCSC | 356 | 6 | 30107 | gene | 0.004075 | 0.008327 | Not queried | 1.48 | 4.85e-01 | 5.11e-01 |
|  | 16 | 76269043 | 76269409 | PDFSTATSENSEMBLUCSC | 367 | 5 | 16106 | island | 0.631712 | 0.383487 | Not queried | 1.48 | 1.51e-01 | 2.11e-01 |
|  | 2 | 23913769 | 23914666 | PDFSTATSENSEMBLUCSC | 898 | 8 | 22597 | gene;island | 0.872237 | 0.971660 | Not queried | 1.48 | 1.05e-01 | 1.66e-01 |
|  | 15 | 91643376 | 91643742 | PDFSTATSENSEMBLUCSC | 367 | 5 | 13483 | gene;island | 0.346964 | 0.535032 | Not queried | 1.48 | 2.22e-01 | 2.76e-01 |
|  | 6 | 150067738 | 150068017 | PDFSTATSENSEMBLUCSC | 280 | 4 | 35287 | tss | 0.076781 | 0.007191 | Not queried | 1.48 | 6.86e-01 | 6.99e-01 |
|  | 7 | 150076338 | 150076646 | PDFSTATSENSEMBLUCSC | 309 | 5 | 39010 | island | 0.002093 | 0.003796 | Not queried | 1.48 | 4.21e-01 | 4.59e-01 |
|  | 10 | 131356942 | 131357256 | PDFSTATSENSEMBLUCSC | 315 | 6 | 4827 | gene | 0.847430 | 0.946731 | Not queried | 1.48 | 1.32e-01 | 1.89e-01 |
|  | 7 | 64254469 | 64254733 | PDFSTATSENSEMBLUCSC | 265 | 6 | 37616 | tss | 0.015325 | 0.015956 | Not queried | 1.48 | 9.37e-01 | 9.41e-01 |
|  | 11 | 73693896 | 73694157 | PDFSTATSENSEMBLUCSC | 262 | 5 | 7554 | tss | 0.028385 | 0.008649 | Not queried | 1.48 | 5.48e-01 | 5.70e-01 |
|  | 2 | 46925970 | 46926272 | PDFSTATSENSEMBLUCSC | 303 | 5 | 23339 | tss | 0.001906 | 0.005212 | Not queried | 1.48 | 4.21e-01 | 4.59e-01 |
|  | 5 | 1100096 | 1100544 | PDFSTATSENSEMBLUCSC | 449 | 4 | 31524 | gene;island | 0.810829 | 0.979832 | Not queried | 1.48 | 1.14e-01 | 1.68e-01 |
|  | 19 | 47363757 | 47363949 | PDFSTATSENSEMBLUCSC | 193 | 5 | 22185 | island | 0.004769 | 0.007821 | Not queried | 1.48 | 3.10e-01 | 3.66e-01 |
|  | 5 | 958808 | 959141 | PDFSTATSENSEMBLUCSC | 334 | 4 | 32599 | island | 0.135902 | 0.299944 | Not queried | 1.48 | 4.86e-01 | 5.11e-01 |
|  | 1 | 11918807 | 11919699 | PDFSTATSENSEMBLUCSC | 893 | 11 | 2691 | island | 0.258020 | 0.041351 | Not queried | 1.48 | 4.00e-02 | 8.03e-02 |
|  | 7 | 151106022 | 151106245 | PDFSTATSENSEMBLUCSC | 224 | 4 | 37292 | gene;island | 0.852322 | 0.931349 | Not queried | 1.48 | 4.86e-01 | 5.11e-01 |
|  | 19 | 59084023 | 59084242 | PDFSTATSENSEMBLUCSC | 220 | 4 | 20266 | gene;island | 0.014392 | 0.014432 | Not queried | 1.48 | 8.86e-01 | 8.89e-01 |
|  | 1 | 153700021 | 153700146 | PDFSTATSENSEMBLUCSC | 126 | 4 | 1951 | tss;island | 0.050963 | 0.008287 | Not queried | 1.48 | 6.86e-01 | 6.99e-01 |
|  | 12 | 31744043 | 31744436 | PDFSTATSENSEMBLUCSC | 394 | 4 | 9627 | tss | 0.293276 | 0.091557 | Not queried | 1.48 | 6.86e-01 | 6.99e-01 |
|  | 14 | 45603667 | 45603864 | PDFSTATSENSEMBLUCSC | 198 | 6 | 12372 | tss;island | 0.000950 | 0.004469 | Not queried | 1.48 | 1.32e-01 | 1.89e-01 |
|  | 10 | 35894012 | 35894430 | PDFSTATSENSEMBLUCSC | 419 | 8 | 5665 | island | 0.517599 | 0.677482 | Not queried | 1.48 | 1.48e-02 | 5.36e-02 |
|  | 16 | 3493336 | 3493440 | PDFSTATSENSEMBLUCSC | 105 | 4 | 14627 | gene | 0.224998 | 0.474176 | Not queried | 1.48 | 2.86e-02 | 6.12e-02 |
|  | 12 | 90102477 | 90102578 | PDFSTATSENSEMBLUCSC | 102 | 4 | 9910 | tss;island | 0.009815 | 0.016324 | Not queried | 1.48 | 3.43e-01 | 3.84e-01 |
|  | 13 | 114889950 | 114890566 | PDFSTATSENSEMBLUCSC | 617 | 7 | 11318 | gene | 0.882448 | 0.981569 | Not queried | 1.48 | 3.18e-01 | 3.74e-01 |
|  | 18 | 48724096 | 48724403 | PDFSTATSENSEMBLUCSC | 308 | 6 | 19280 | tss | 0.004585 | 0.010874 | Not queried | 1.48 | 3.94e-01 | 4.36e-01 |
|  | 4 | 149363678 | 149363912 | PDFSTATSENSEMBLUCSC | 235 | 5 | 30629 | tss | 0.002119 | 0.004815 | Not queried | 1.48 | 4.21e-01 | 4.59e-01 |
|  | 2 | 240062051 | 240062353 | PDFSTATSENSEMBLUCSC | 303 | 4 | 23107 | gene;island | 0.955303 | 0.978437 | Not queried | 1.48 | 5.71e-02 | 1.00e-01 |
|  | 17 | 27224801 | 27225046 | PDFSTATSENSEMBLUCSC | 246 | 7 | 17227 | tss | 0.136567 | 0.031275 | Not queried | 1.48 | 3.83e-01 | 4.27e-01 |
|  | 6 | 30954872 | 30955142 | PDFSTATSENSEMBLUCSC | 271 | 4 | 33845 | gene | 0.252390 | 0.458331 | Not queried | 1.48 | 2.86e-02 | 6.12e-02 |
|  | 20 | 40247136 | 40247245 | PDFSTATSENSEMBLUCSC | 110 | 6 | 25747 | tss | 0.003302 | 0.008671 | Not queried | 1.48 | 1.32e-01 | 1.89e-01 |
|  | 15 | 78914083 | 78914439 | PDFSTATSENSEMBLUCSC | 357 | 4 | 13837 | tss;island | 0.914229 | 0.971116 | Not queried | 1.48 | 2.86e-02 | 6.12e-02 |
|  | 17 | 5342543 | 5342830 | PDFSTATSENSEMBLUCSC | 288 | 7 | 17045 | tss | 0.009972 | 0.011564 | Not queried | 1.48 | 6.20e-01 | 6.43e-01 |
|  | 7 | 32110988 | 32111453 | PDFSTATSENSEMBLUCSC | 466 | 8 | 38393 | island | 0.655083 | 0.476204 | Not queried | 1.48 | 1.30e-01 | 1.89e-01 |
|  | 6 | 86158972 | 86159251 | PDFSTATSENSEMBLUCSC | 280 | 6 | 35102 | tss | 0.966540 | 0.989008 | Not queried | 1.48 | 9.31e-02 | 1.53e-01 |
|  | 16 | 25077868 | 25078439 | PDFSTATSENSEMBLUCSC | 572 | 5 | 15775 | island | 0.060636 | 0.024896 | Not queried | 1.48 | 3.10e-01 | 3.66e-01 |
|  | 1 | 223566127 | 223566709 | PDFSTATSENSEMBLUCSC | 583 | 7 | 2272 | tss | 0.674505 | 0.427565 | Not queried | 1.47 | 3.83e-01 | 4.27e-01 |
|  | 5 | 140723331 | 140723899 | PDFSTATSENSEMBLUCSC | 569 | 9 | 31818 | gene;island | 0.929598 | 0.942015 | Not queried | 1.47 | 2.58e-01 | 3.16e-01 |
|  | 7 | 158216857 | 158217285 | PDFSTATSENSEMBLUCSC | 429 | 4 | 37368 | gene;island | 0.836033 | 0.947909 | Not queried | 1.47 | 4.86e-01 | 5.11e-01 |
|  | 12 | 74564847 | 74565116 | PDFSTATSENSEMBLUCSC | 270 | 4 | 10629 | island | 0.822918 | 0.943218 | Not queried | 1.47 | 1.14e-01 | 1.68e-01 |
|  | 1 | 1114912 | 1115070 | PDFSTATSENSEMBLUCSC | 159 | 4 | 1143 | tss | 0.946762 | 0.976236 | Not queried | 1.47 | 5.71e-02 | 1.00e-01 |
|  | 2 | 96990762 | 96991327 | PDFSTATSENSEMBLUCSC | 566 | 9 | 23515 | tss;island | 0.862754 | 0.947999 | Not queried | 1.47 | 1.90e-01 | 2.55e-01 |
|  | 8 | 70984199 | 70984559 | PDFSTATSENSEMBLUCSC | 361 | 4 | 39758 | tss;island | 0.918195 | 0.953787 | Not queried | 1.47 | 2.86e-02 | 6.12e-02 |
|  | 3 | 56590940 | 56591241 | PDFSTATSENSEMBLUCSC | 302 | 7 | 29220 | island | 0.002072 | 0.005702 | Not queried | 1.47 | 3.83e-01 | 4.27e-01 |
|  | 21 | 39644216 | 39644354 | PDFSTATSENSEMBLUCSC | 139 | 4 | 26426 | gene;tss | 0.149666 | 0.360685 | Not queried | 1.47 | 2.00e-01 | 2.55e-01 |
|  | 5 | 140473857 | 140474184 | PDFSTATSENSEMBLUCSC | 328 | 6 | 32361 | tss | 0.274339 | 0.531897 | Not queried | 1.47 | 9.31e-02 | 1.53e-01 |
|  | 8 | 77594595 | 77595027 | PDFSTATSENSEMBLUCSC | 433 | 4 | 39343 | gene;island | 0.730720 | 0.390163 | Not queried | 1.47 | 3.43e-01 | 3.84e-01 |
|  | 19 | 10697690 | 10698098 | PDFSTATSENSEMBLUCSC | 409 | 7 | 21540 | island | 0.022705 | 0.003552 | Not queried | 1.47 | 3.18e-01 | 3.74e-01 |
|  | 1 | 155145515 | 155145926 | PDFSTATSENSEMBLUCSC | 412 | 10 | 1984 | tss;island | 0.004345 | 0.012096 | Not queried | 1.47 | 1.65e-01 | 2.29e-01 |
|  | 19 | 49457665 | 49458001 | PDFSTATSENSEMBLUCSC | 337 | 5 | 20933 | tss;island | 0.043795 | 0.074984 | Not queried | 1.47 | 4.21e-01 | 4.59e-01 |
|  | 17 | 12921270 | 12921796 | PDFSTATSENSEMBLUCSC | 527 | 10 | 18030 | island | 0.002417 | 0.005569 | Not queried | 1.47 | 1.47e-02 | 5.36e-02 |
|  | 11 | 68780866 | 68780984 | PDFSTATSENSEMBLUCSC | 119 | 4 | 7515 | tss;island | 0.836083 | 0.596789 | Not queried | 1.47 | 5.71e-02 | 1.00e-01 |
|  | 2 | 207506480 | 207506675 | PDFSTATSENSEMBLUCSC | 196 | 4 | 23837 | tss;island | 0.428439 | 0.725234 | Not queried | 1.47 | 2.00e-01 | 2.55e-01 |
|  | 4 | 15704393 | 15704550 | PDFSTATSENSEMBLUCSC | 158 | 4 | 30311 | tss | 0.779380 | 0.941244 | Not queried | 1.47 | 3.43e-01 | 3.84e-01 |
|  | 1 | 154947991 | 154948305 | PDFSTATSENSEMBLUCSC | 315 | 4 | 786 | gene;tss;island | 0.003574 | 0.006661 | Not queried | 1.47 | 8.86e-01 | 8.89e-01 |
|  | 11 | 113258210 | 113258455 | PDFSTATSENSEMBLUCSC | 246 | 6 | 7703 | tss;island | 0.861555 | 0.929038 | Not queried | 1.47 | 3.10e-01 | 3.66e-01 |
|  | 17 | 58042144 | 58042493 | PDFSTATSENSEMBLUCSC | 350 | 11 | 17550 | tss | 0.006685 | 0.017253 | Not queried | 1.47 | 3.36e-02 | 6.87e-02 |
|  | 18 | 67955861 | 67955960 | PDFSTATSENSEMBLUCSC | 100 | 4 | 19317 | tss;island | 0.012068 | 0.031593 | Not queried | 1.47 | 4.86e-01 | 5.11e-01 |
|  | 4 | 186877497 | 186877778 | PDFSTATSENSEMBLUCSC | 282 | 5 | 30214 | gene | 0.824893 | 0.243397 | Not queried | 1.47 | 5.56e-02 | 1.00e-01 |
|  | 19 | 4639662 | 4640159 | PDFSTATSENSEMBLUCSC | 498 | 5 | 19736 | gene | 0.114504 | 0.501355 | Not queried | 1.47 | 3.10e-01 | 3.66e-01 |
|  | 8 | 65286244 | 65286632 | PDFSTATSENSEMBLUCSC | 389 | 7 | 40341 | island | 0.577932 | 0.101640 | Not queried | 1.47 | 1.28e-01 | 1.87e-01 |
|  | 8 | 11536778 | 11537112 | PDFSTATSENSEMBLUCSC | 335 | 4 | 40083 | island | 0.826534 | 0.942468 | Not queried | 1.47 | 4.86e-01 | 5.11e-01 |
|  | 1 | 184943911 | 184944288 | PDFSTATSENSEMBLUCSC | 378 | 4 | 2139 | tss;island | 0.800207 | 0.610944 | Not queried | 1.47 | 6.86e-01 | 6.99e-01 |
|  | 16 | 3493336 | 3494155 | PDFSTATSENSEMBLUCSC | 820 | 11 | 15056 | tss;island | 0.294576 | 0.527597 | LA16c-306E5.2; NAA60; ZNF597 (margin: LA16c-306E5.2; NAA60; ZNF597) | 1.47 | 5.67e-06 | 3.54e-04 |
|  | 20 | 30225325 | 30225685 | PDFSTATSENSEMBLUCSC | 361 | 6 | 25664 | tss | 0.760631 | 0.951800 | Not queried | 1.47 | 6.49e-02 | 1.11e-01 |
|  | 14 | 69951720 | 69952104 | PDFSTATSENSEMBLUCSC | 385 | 5 | 12125 | gene;island | 0.532893 | 0.576740 | Not queried | 1.47 | 3.10e-01 | 3.66e-01 |
|  | 6 | 32141584 | 32142236 | PDFSTATSENSEMBLUCSC | 653 | 10 | 34070 | gene | 0.975824 | 0.942879 | Not queried | 1.47 | 3.93e-01 | 4.36e-01 |
|  | 1 | 220864119 | 220864321 | PDFSTATSENSEMBLUCSC | 203 | 4 | 1019 | gene;island | 0.698575 | 0.430874 | Not queried | 1.47 | 3.43e-01 | 3.84e-01 |
|  | 11 | 12695341 | 12695578 | PDFSTATSENSEMBLUCSC | 238 | 4 | 7155 | tss;island | 0.034074 | 0.014802 | Not queried | 1.47 | 4.86e-01 | 5.11e-01 |
|  | 20 | 61493177 | 61493341 | PDFSTATSENSEMBLUCSC | 165 | 9 | 25883 | tss;island | 0.002551 | 0.004725 | Not queried | 1.47 | 3.40e-01 | 3.84e-01 |
|  | 21 | 44394280 | 44394503 | PDFSTATSENSEMBLUCSC | 224 | 4 | 26572 | tss;island | 0.006134 | 0.018338 | Not queried | 1.47 | 2.00e-01 | 2.55e-01 |
|  | 16 | 88933068 | 88933359 | PDFSTATSENSEMBLUCSC | 292 | 4 | 15417 | tss;island | 0.664711 | 0.810009 | Not queried | 1.47 | 2.86e-02 | 6.12e-02 |
|  | 4 | 25677759 | 25678113 | PDFSTATSENSEMBLUCSC | 355 | 5 | 29971 | gene;island | 0.627632 | 0.932561 | Not queried | 1.47 | 1.51e-01 | 2.11e-01 |
|  | 1 | 39957129 | 39957535 | PDFSTATSENSEMBLUCSC | 407 | 10 | 3101 | island | 0.026031 | 0.020420 | Not queried | 1.47 | 3.15e-01 | 3.72e-01 |
|  | 6 | 28553927 | 28555929 | PDFSTATSENSEMBLUCSC | 2003 | 37 | 35589 | island | 0.005694 | 0.018788 | Not queried | 1.47 | 7.80e-02 | 1.32e-01 |
|  | 6 | 28317687 | 28317796 | PDFSTATSENSEMBLUCSC | 110 | 4 | 33631 | gene | 0.011215 | 0.016014 | Not queried | 1.47 | 2.00e-01 | 2.55e-01 |
|  | 3 | 49577229 | 49577663 | PDFSTATSENSEMBLUCSC | 435 | 4 | 29121 | island | 0.003870 | 0.012608 | Not queried | 1.47 | 3.43e-01 | 3.84e-01 |
|  | 13 | 95201227 | 95201535 | PDFSTATSENSEMBLUCSC | 309 | 4 | 11818 | island | 0.029167 | 0.053894 | Not queried | 1.47 | 3.43e-01 | 3.84e-01 |
|  | 6 | 31927895 | 31928395 | PDFSTATSENSEMBLUCSC | 501 | 6 | 34001 | gene;island | 0.973156 | 0.923892 | Not queried | 1.47 | 6.49e-02 | 1.11e-01 |
|  | 6 | 13814875 | 13815054 | PDFSTATSENSEMBLUCSC | 180 | 5 | 34646 | tss;island | 0.006510 | 0.011358 | Not queried | 1.46 | 6.90e-01 | 7.01e-01 |
|  | 11 | 118938260 | 118938596 | PDFSTATSENSEMBLUCSC | 337 | 8 | 8869 | island | 0.004308 | 0.005689 | Not queried | 1.46 | 5.74e-01 | 5.97e-01 |
|  | 19 | 17982599 | 17982930 | PDFSTATSENSEMBLUCSC | 332 | 9 | 21759 | island | 0.195642 | 0.386477 | Not queried | 1.46 | 1.13e-01 | 1.68e-01 |
|  | 14 | 38064456 | 38064532 | PDFSTATSENSEMBLUCSC | 77 | 5 | 12796 | island | 0.007921 | 0.027928 | Not queried | 1.46 | 5.48e-01 | 5.70e-01 |
|  | 16 | 3192260 | 3192668 | PDFSTATSENSEMBLUCSC | 409 | 4 | 14623 | gene;island | 0.938993 | 0.983751 | Not queried | 1.46 | 4.86e-01 | 5.11e-01 |
|  | 5 | 112196734 | 112196944 | PDFSTATSENSEMBLUCSC | 211 | 4 | 32215 | tss | 0.007028 | 0.004115 | Not queried | 1.46 | 4.86e-01 | 5.11e-01 |
|  | 6 | 25732093 | 25732239 | PDFSTATSENSEMBLUCSC | 147 | 4 | 35501 | island | 0.805328 | 0.914469 | Not queried | 1.46 | 6.86e-01 | 6.99e-01 |
|  | 2 | 37572102 | 37572423 | PDFSTATSENSEMBLUCSC | 322 | 4 | 22649 | gene;island | 0.470090 | 0.707853 | Not queried | 1.46 | 2.00e-01 | 2.55e-01 |
|  | 20 | 33680135 | 33681223 | PDFSTATSENSEMBLUCSC | 1089 | 15 | 26118 | island | 0.007824 | 0.012397 | Not queried | 1.46 | 3.05e-01 | 3.66e-01 |
|  | 14 | 102414421 | 102414739 | PDFSTATSENSEMBLUCSC | 319 | 4 | 13165 | island | 0.008030 | 0.016896 | Not queried | 1.46 | 3.43e-01 | 3.84e-01 |
|  | 14 | 23510934 | 23511306 | PDFSTATSENSEMBLUCSC | 373 | 4 | 12293 | tss | 0.494118 | 0.764694 | Not queried | 1.46 | 3.43e-01 | 3.84e-01 |
|  | 8 | 42911287 | 42911370 | PDFSTATSENSEMBLUCSC | 84 | 5 | 39684 | tss | 0.000167 | 0.001558 | Not queried | 1.46 | 3.17e-02 | 6.50e-02 |
|  | 17 | 41476044 | 41476317 | PDFSTATSENSEMBLUCSC | 274 | 6 | 17389 | tss | 0.002984 | 0.004981 | Not queried | 1.46 | 3.10e-01 | 3.66e-01 |
|  | 6 | 32078398 | 32078624 | PDFSTATSENSEMBLUCSC | 227 | 8 | 34862 | tss | 0.193422 | 0.486042 | Not queried | 1.46 | 2.07e-02 | 6.12e-02 |
|  | 11 | 1092135 | 1092321 | PDFSTATSENSEMBLUCSC | 187 | 6 | 6403 | gene;island | 0.968176 | 0.980408 | Not queried | 1.46 | 3.10e-01 | 3.66e-01 |
|  | 16 | 71928811 | 71929079 | PDFSTATSENSEMBLUCSC | 269 | 5 | 15351 | tss;island | 0.004252 | 0.006156 | Not queried | 1.46 | 2.22e-01 | 2.76e-01 |
|  | 5 | 176755199 | 176755542 | PDFSTATSENSEMBLUCSC | 344 | 4 | 33445 | island | 0.766806 | 0.976455 | Not queried | 1.46 | 2.00e-01 | 2.55e-01 |
|  | 2 | 18059103 | 18059200 | PDFSTATSENSEMBLUCSC | 98 | 4 | 23203 | tss;island | 0.949677 | 0.982803 | Not queried | 1.46 | 5.71e-02 | 1.00e-01 |
|  | 2 | 1732172 | 1732454 | PDFSTATSENSEMBLUCSC | 283 | 4 | 22535 | gene | 0.823370 | 0.942774 | Not queried | 1.46 | 1.14e-01 | 1.68e-01 |
|  | 4 | 187810446 | 187810783 | PDFSTATSENSEMBLUCSC | 338 | 5 | 31487 | island | 0.930975 | 0.977395 | Not queried | 1.46 | 1.00e+00 | 1.00e+00 |
|  | 3 | 40657083 | 40657497 | PDFSTATSENSEMBLUCSC | 415 | 6 | 29008 | island | 0.556602 | 0.753588 | Not queried | 1.46 | 1.32e-01 | 1.89e-01 |
|  | 6 | 31895464 | 31895598 | PDFSTATSENSEMBLUCSC | 135 | 4 | 33994 | gene | 0.933098 | 0.731645 | Not queried | 1.46 | 1.14e-01 | 1.68e-01 |
|  | 2 | 220363572 | 220363862 | PDFSTATSENSEMBLUCSC | 291 | 7 | 25127 | island | 0.004809 | 0.012054 | Not queried | 1.46 | 3.18e-01 | 3.74e-01 |
|  | 14 | 89029552 | 89029717 | PDFSTATSENSEMBLUCSC | 166 | 6 | 12168 | gene;island | 0.010953 | 0.004937 | Not queried | 1.46 | 3.94e-01 | 4.36e-01 |
|  | 10 | 46971222 | 46971658 | PDFSTATSENSEMBLUCSC | 437 | 4 | 5062 | tss;island | 0.930626 | 0.973574 | Not queried | 1.46 | 4.86e-01 | 5.11e-01 |
|  | 13 | 45992551 | 45992673 | PDFSTATSENSEMBLUCSC | 123 | 6 | 11421 | tss | 0.006912 | 0.003551 | Not queried | 1.46 | 3.10e-01 | 3.66e-01 |
|  | 20 | 43160245 | 43160301 | PDFSTATSENSEMBLUCSC | 57 | 4 | 25762 | tss;island | 0.023292 | 0.068843 | Not queried | 1.46 | 2.00e-01 | 2.55e-01 |
|  | 6 | 127840328 | 127840755 | PDFSTATSENSEMBLUCSC | 428 | 10 | 36473 | island | 0.897538 | 0.740147 | Not queried | 1.46 | 4.81e-01 | 5.11e-01 |
|  | 6 | 127840517 | 127840755 | PDFSTATSENSEMBLUCSC | 239 | 8 | 35217 | tss | 0.897538 | 0.720623 | Not queried | 1.46 | 5.05e-01 | 5.32e-01 |
|  | 6 | 33561181 | 33561449 | PDFSTATSENSEMBLUCSC | 269 | 7 | 34910 | tss | 0.971292 | 0.933673 | LINC00336 (margin: GGNBP1; LINC00336) | 1.46 | 4.08e-03 | 2.47e-02 |
|  | 6 | 36842801 | 36842933 | PDFSTATSENSEMBLUCSC | 133 | 5 | 34942 | tss | 0.011986 | 0.011323 | Not queried | 1.46 | 1.00e+00 | 1.00e+00 |
|  | 7 | 1121657 | 1121929 | PDFSTATSENSEMBLUCSC | 273 | 4 | 36747 | gene;island | 0.901129 | 0.969289 | Not queried | 1.46 | 4.86e-01 | 5.11e-01 |
|  | 7 | 99679295 | 99679490 | PDFSTATSENSEMBLUCSC | 196 | 6 | 38745 | island | 0.009691 | 0.012836 | Not queried | 1.46 | 4.85e-01 | 5.11e-01 |
|  | 17 | 38375119 | 38375510 | PDFSTATSENSEMBLUCSC | 392 | 7 | 17327 | tss;island | 0.046463 | 0.006945 | Not queried | 1.46 | 7.10e-01 | 7.20e-01 |
|  | 16 | 9185438 | 9185541 | PDFSTATSENSEMBLUCSC | 104 | 5 | 15691 | island | 0.001345 | 0.005936 | Not queried | 1.46 | 2.22e-01 | 2.76e-01 |
|  | 7 | 142583555 | 142583836 | PDFSTATSENSEMBLUCSC | 282 | 4 | 37948 | tss | 0.015905 | 0.107629 | Not queried | 1.46 | 4.86e-01 | 5.11e-01 |
|  | 6 | 33560953 | 33561449 | PDFSTATSENSEMBLUCSC | 497 | 9 | 36042 | island | 0.978411 | 0.950541 | LINC00336 (margin: GGNBP1; LINC00336) | 1.46 | 7.77e-03 | 3.19e-02 |
|  | 14 | 67826350 | 67826504 | PDFSTATSENSEMBLUCSC | 155 | 4 | 12118 | gene;tss;island | 0.015286 | 0.029209 | Not queried | 1.46 | 3.43e-01 | 3.84e-01 |
|  | 7 | 99149732 | 99149896 | PDFSTATSENSEMBLUCSC | 165 | 5 | 37759 | tss | 0.006614 | 0.015744 | Not queried | 1.46 | 2.22e-01 | 2.76e-01 |
|  | 7 | 148936572 | 148936883 | PDFSTATSENSEMBLUCSC | 312 | 7 | 38993 | island | 0.007997 | 0.015570 | Not queried | 1.46 | 3.83e-01 | 4.27e-01 |
|  | 6 | 32809263 | 32809657 | PDFSTATSENSEMBLUCSC | 395 | 10 | 34148 | gene;island | 0.982997 | 0.951179 | PSMB8 (margin: XXbac-BPG246D15.8; PSMB9; TAP2; PSMB8; TAP1) | 1.46 | 2.17e-05 | 8.94e-04 |
|  | 8 | 86157735 | 86157935 | PDFSTATSENSEMBLUCSC | 201 | 4 | 39353 | gene | 0.007663 | 0.017493 | Not queried | 1.46 | 4.86e-01 | 5.11e-01 |
|  | 11 | 45825356 | 45825579 | PDFSTATSENSEMBLUCSC | 224 | 8 | 7266 | tss;island | 0.976164 | 0.941903 | Not queried | 1.46 | 1.61e-01 | 2.24e-01 |
|  | 8 | 124428540 | 124428910 | PDFSTATSENSEMBLUCSC | 371 | 6 | 39900 | tss;island | 0.004170 | 0.005953 | Not queried | 1.46 | 3.10e-01 | 3.66e-01 |
|  | 14 | 55657967 | 55658290 | PDFSTATSENSEMBLUCSC | 324 | 4 | 12087 | gene | 0.003176 | 0.013117 | Not queried | 1.45 | 4.86e-01 | 5.11e-01 |
|  | 11 | 111741914 | 111742291 | PDFSTATSENSEMBLUCSC | 378 | 7 | 6900 | gene | 0.001606 | 0.005352 | Not queried | 1.45 | 1.65e-01 | 2.28e-01 |
|  | 5 | 150227510 | 150227845 | PDFSTATSENSEMBLUCSC | 336 | 4 | 31865 | gene | 0.872627 | 0.925673 | Not queried | 1.45 | 1.14e-01 | 1.68e-01 |
|  | 20 | 22567724 | 22568116 | PDFSTATSENSEMBLUCSC | 393 | 4 | 26046 | island | 0.806817 | 0.911872 | Not queried | 1.45 | 2.00e-01 | 2.55e-01 |
|  | 12 | 10766016 | 10766485 | PDFSTATSENSEMBLUCSC | 470 | 12 | 10240 | island | 0.005584 | 0.013738 | Not queried | 1.45 | 1.98e-01 | 2.55e-01 |
|  | 12 | 10766264 | 10766485 | PDFSTATSENSEMBLUCSC | 222 | 8 | 9561 | tss | 0.003012 | 0.008936 | Not queried | 1.45 | 2.34e-01 | 2.90e-01 |
|  | 11 | 66206319 | 66206505 | PDFSTATSENSEMBLUCSC | 187 | 5 | 7468 | tss | 0.007436 | 0.023251 | Not queried | 1.45 | 9.52e-02 | 1.53e-01 |
|  | 7 | 116594421 | 116594693 | PDFSTATSENSEMBLUCSC | 273 | 6 | 37199 | gene;tss;island | 0.002489 | 0.004124 | Not queried | 1.45 | 2.40e-01 | 2.95e-01 |
|  | 17 | 20946875 | 20947103 | PDFSTATSENSEMBLUCSC | 229 | 4 | 17199 | tss;island | 0.006508 | 0.004758 | Not queried | 1.45 | 8.86e-01 | 8.89e-01 |
|  | 1 | 151371944 | 151372335 | PDFSTATSENSEMBLUCSC | 392 | 5 | 3745 | island | 0.000876 | 0.001619 | Not queried | 1.45 | 8.41e-01 | 8.48e-01 |
|  | 3 | 112738642 | 112738800 | PDFSTATSENSEMBLUCSC | 159 | 7 | 28504 | tss | 0.067811 | 0.022117 | Not queried | 1.45 | 5.35e-01 | 5.62e-01 |
|  | 17 | 66254884 | 66255444 | PDFSTATSENSEMBLUCSC | 561 | 8 | 18690 | island | 0.343958 | 0.598750 | Not queried | 1.45 | 3.79e-02 | 7.66e-02 |
|  | 17 | 66254884 | 66255265 | PDFSTATSENSEMBLUCSC | 382 | 6 | 17606 | tss | 0.343958 | 0.579935 | Not queried | 1.45 | 1.32e-01 | 1.89e-01 |
|  | 3 | 118864587 | 118864895 | PDFSTATSENSEMBLUCSC | 309 | 7 | 27905 | gene | 0.974058 | 0.972102 | Not queried | 1.45 | 5.35e-01 | 5.62e-01 |
|  | 3 | 52008487 | 52009002 | PDFSTATSENSEMBLUCSC | 516 | 11 | 28369 | tss;island | 0.245858 | 0.498183 | Not queried | 1.45 | 7.48e-01 | 7.57e-01 |
|  | 3 | 9027157 | 9027576 | PDFSTATSENSEMBLUCSC | 420 | 4 | 27649 | gene;island | 0.875470 | 0.950851 | Not queried | 1.45 | 3.43e-01 | 3.84e-01 |
|  | 1 | 203830556 | 203831055 | PDFSTATSENSEMBLUCSC | 500 | 8 | 4111 | island | 0.004000 | 0.007199 | Not queried | 1.45 | 3.82e-01 | 4.27e-01 |
|  | 19 | 49375485 | 49375640 | PDFSTATSENSEMBLUCSC | 156 | 4 | 20929 | tss | 0.012486 | 0.030690 | Not queried | 1.45 | 8.86e-01 | 8.89e-01 |
|  | 15 | 89089914 | 89090195 | PDFSTATSENSEMBLUCSC | 282 | 4 | 13880 | tss | 0.011982 | 0.002934 | Not queried | 1.45 | 8.86e-01 | 8.89e-01 |
|  | 10 | 130010134 | 130010447 | PDFSTATSENSEMBLUCSC | 314 | 4 | 6253 | island | 0.231625 | 0.084599 | Not queried | 1.45 | 6.86e-01 | 6.99e-01 |
|  | 19 | 35630106 | 35630388 | PDFSTATSENSEMBLUCSC | 283 | 6 | 20699 | tss | 0.767839 | 0.880814 | Not queried | 1.45 | 1.80e-01 | 2.45e-01 |
|  | 7 | 19157193 | 19157420 | PDFSTATSENSEMBLUCSC | 228 | 6 | 38283 | island | 0.304460 | 0.614377 | Not queried | 1.45 | 4.11e-02 | 8.14e-02 |
|  | 14 | 54976234 | 54976570 | PDFSTATSENSEMBLUCSC | 337 | 8 | 12408 | tss | 0.004687 | 0.010352 | Not queried | 1.45 | 2.07e-02 | 6.12e-02 |
|  | 2 | 24346215 | 24346267 | PDFSTATSENSEMBLUCSC | 53 | 4 | 23224 | tss;island | 0.002123 | 0.004832 | Not queried | 1.45 | 3.43e-01 | 3.84e-01 |
|  | 1 | 240255377 | 240255691 | PDFSTATSENSEMBLUCSC | 315 | 4 | 1105 | gene;island | 0.684150 | 0.886910 | Not queried | 1.45 | 3.43e-01 | 3.84e-01 |
|  | 1 | 8385896 | 8386089 | PDFSTATSENSEMBLUCSC | 194 | 4 | 194 | gene;island | 0.972188 | 0.989628 | Not queried | 1.45 | 4.86e-01 | 5.11e-01 |
|  | 6 | 32146466 | 32146779 | PDFSTATSENSEMBLUCSC | 314 | 7 | 34073 | gene;tss | 0.019107 | 0.029879 | Not queried | 1.45 | 3.83e-01 | 4.27e-01 |
|  | 10 | 50970428 | 50970649 | PDFSTATSENSEMBLUCSC | 222 | 8 | 5077 | tss | 0.023137 | 0.014777 | Not queried | 1.45 | 2.34e-01 | 2.90e-01 |
|  | 7 | 102985332 | 102985587 | PDFSTATSENSEMBLUCSC | 256 | 4 | 37812 | tss | 0.247025 | 0.183946 | Not queried | 1.45 | 1.00e+00 | 1.00e+00 |
|  | 20 | 61427684 | 61427693 | PDFSTATSENSEMBLUCSC | 10 | 4 | 25880 | tss;island | 0.011078 | 0.007799 | Not queried | 1.45 | 6.86e-01 | 6.99e-01 |
|  | 7 | 80548695 | 80548752 | PDFSTATSENSEMBLUCSC | 58 | 5 | 37675 | tss;island | 0.002520 | 0.008834 | Not queried | 1.45 | 2.22e-01 | 2.76e-01 |
|  | 17 | 35305905 | 35306063 | PDFSTATSENSEMBLUCSC | 159 | 6 | 17285 | tss;island | 0.014071 | 0.012822 | Not queried | 1.45 | 5.89e-01 | 6.11e-01 |
|  | 14 | 101292643 | 101293090 | PDFSTATSENSEMBLUCSC | 448 | 5 | 12209 | gene;island | 0.947274 | 0.967561 | Not queried | 1.45 | 4.21e-01 | 4.59e-01 |
|  | 20 | 48770442 | 48770800 | PDFSTATSENSEMBLUCSC | 359 | 7 | 25817 | tss;island | 0.009387 | 0.010891 | Not queried | 1.45 | 7.10e-01 | 7.20e-01 |
|  | 16 | 82660206 | 82660873 | PDFSTATSENSEMBLUCSC | 668 | 14 | 16129 | island | 0.195635 | 0.433019 | CDH13 (margin: CDH13) | 1.45 | 1.43e-03 | 1.36e-02 |
|  | 15 | 93609305 | 93609551 | PDFSTATSENSEMBLUCSC | 247 | 4 | 13488 | gene;island | 0.774511 | 0.907092 | Not queried | 1.45 | 3.43e-01 | 3.84e-01 |
|  | 2 | 31361593 | 31361818 | PDFSTATSENSEMBLUCSC | 226 | 8 | 23287 | tss;island | 0.182775 | 0.036935 | Not queried | 1.45 | 3.79e-02 | 7.66e-02 |
|  | 15 | 67458370 | 67458665 | PDFSTATSENSEMBLUCSC | 296 | 4 | 13393 | gene | 0.954934 | 0.976638 | Not queried | 1.45 | 2.00e-01 | 2.55e-01 |
|  | 7 | 100860599 | 100861373 | PDFSTATSENSEMBLUCSC | 775 | 15 | 37153 | gene;tss;island | 0.012742 | 0.029350 | Not queried | 1.45 | 1.06e-01 | 1.68e-01 |
|  | 6 | 118972731 | 118972834 | PDFSTATSENSEMBLUCSC | 104 | 4 | 34457 | gene | 0.020020 | 0.016356 | Not queried | 1.45 | 3.43e-01 | 3.84e-01 |
|  | 6 | 31797821 | 31798138 | PDFSTATSENSEMBLUCSC | 318 | 5 | 35848 | island | 0.001747 | 0.010227 | Not queried | 1.45 | 5.48e-01 | 5.70e-01 |
|  | 7 | 100209784 | 100210343 | PDFSTATSENSEMBLUCSC | 560 | 10 | 37145 | gene | 0.010567 | 0.015997 | Not queried | 1.45 | 1.65e-01 | 2.29e-01 |
|  | 11 | 116643266 | 116643957 | PDFSTATSENSEMBLUCSC | 692 | 12 | 8834 | island | 0.003375 | 0.006923 | Not queried | 1.45 | 1.01e-01 | 1.60e-01 |
|  | 19 | 13044570 | 13045039 | PDFSTATSENSEMBLUCSC | 470 | 9 | 20520 | tss | 0.009194 | 0.023301 | Not queried | 1.45 | 4.36e-01 | 4.76e-01 |
|  | 15 | 99057732 | 99058098 | PDFSTATSENSEMBLUCSC | 367 | 4 | 13922 | tss | 0.804072 | 0.951343 | Not queried | 1.45 | 3.43e-01 | 3.84e-01 |
|  | 3 | 49977118 | 49977428 | PDFSTATSENSEMBLUCSC | 311 | 11 | 28341 | tss | 0.009778 | 0.028499 | Not queried | 1.45 | 2.43e-01 | 2.98e-01 |
|  | 12 | 127765195 | 127765627 | PDFSTATSENSEMBLUCSC | 433 | 4 | 10986 | island | 0.872859 | 0.708281 | Not queried | 1.45 | 4.86e-01 | 5.11e-01 |
|  | 1 | 152008878 | 152009772 | PDFSTATSENSEMBLUCSC | 895 | 13 | 3758 | island | 0.007729 | 0.014112 | Not queried | 1.45 | 9.08e-02 | 1.52e-01 |
|  | 1 | 152009578 | 152009772 | PDFSTATSENSEMBLUCSC | 195 | 5 | 1936 | tss | 0.027999 | 0.109833 | Not queried | 1.45 | 1.51e-01 | 2.11e-01 |
|  | 2 | 95537044 | 95537475 | PDFSTATSENSEMBLUCSC | 432 | 8 | 24510 | island | 0.539630 | 0.375706 | Not queried | 1.45 | 2.34e-01 | 2.90e-01 |
|  | 14 | 54976234 | 54976718 | PDFSTATSENSEMBLUCSC | 485 | 10 | 12858 | island | 0.004687 | 0.010315 | CGRRF1 (margin: CGRRF1) | 1.45 | 1.15e-02 | 4.29e-02 |
|  | 5 | 140734543 | 140735027 | PDFSTATSENSEMBLUCSC | 485 | 6 | 31820 | gene;island | 0.648280 | 0.917514 | Not queried | 1.45 | 3.10e-01 | 3.66e-01 |
|  | 17 | 63119174 | 63119618 | PDFSTATSENSEMBLUCSC | 445 | 5 | 18671 | island | 0.002663 | 0.005574 | Not queried | 1.45 | 5.48e-01 | 5.70e-01 |
|  | 1 | 11724057 | 11724943 | PDFSTATSENSEMBLUCSC | 887 | 10 | 2683 | island | 0.008705 | 0.024099 | Not queried | 1.45 | 2.47e-01 | 3.03e-01 |
|  | 16 | 731772 | 732216 | PDFSTATSENSEMBLUCSC | 445 | 4 | 14537 | gene;island | 0.605916 | 0.842642 | Not queried | 1.45 | 6.86e-01 | 6.99e-01 |
|  | 4 | 84376750 | 84376998 | PDFSTATSENSEMBLUCSC | 249 | 5 | 30045 | gene | 0.005104 | 0.009093 | Not queried | 1.45 | 3.10e-01 | 3.66e-01 |
|  | 2 | 10182878 | 10183271 | PDFSTATSENSEMBLUCSC | 394 | 7 | 23176 | tss;island | 0.838889 | 0.621128 | Not queried | 1.45 | 3.83e-01 | 4.27e-01 |
|  | 1 | 23696021 | 23696209 | PDFSTATSENSEMBLUCSC | 189 | 4 | 312 | gene;tss;island | 0.003799 | 0.009035 | Not queried | 1.44 | 1.14e-01 | 1.68e-01 |
|  | 13 | 36045201 | 36045352 | PDFSTATSENSEMBLUCSC | 152 | 4 | 11126 | gene;island | 0.757978 | 0.873516 | Not queried | 1.44 | 4.86e-01 | 5.11e-01 |
|  | 2 | 131673771 | 131674221 | PDFSTATSENSEMBLUCSC | 451 | 7 | 23631 | tss | 0.964805 | 0.910574 | Not queried | 1.44 | 3.79e-02 | 7.66e-02 |
|  | 2 | 97001274 | 97001430 | PDFSTATSENSEMBLUCSC | 157 | 6 | 23516 | tss;island | 0.004657 | 0.011157 | Not queried | 1.44 | 9.31e-02 | 1.53e-01 |
|  | 16 | 2054079 | 2054287 | PDFSTATSENSEMBLUCSC | 209 | 4 | 14594 | gene;island | 0.578979 | 0.468173 | Not queried | 1.44 | 4.86e-01 | 5.11e-01 |
|  | 19 | 3880535 | 3880670 | PDFSTATSENSEMBLUCSC | 136 | 4 | 21353 | island | 0.529957 | 0.701559 | Not queried | 1.44 | 2.00e-01 | 2.55e-01 |
|  | 3 | 124774855 | 124775112 | PDFSTATSENSEMBLUCSC | 258 | 5 | 28549 | tss;island | 0.007201 | 0.010905 | Not queried | 1.44 | 3.10e-01 | 3.66e-01 |
|  | 3 | 46792023 | 46792462 | PDFSTATSENSEMBLUCSC | 440 | 5 | 29060 | island | 0.928336 | 0.958036 | Not queried | 1.44 | 5.48e-01 | 5.70e-01 |
|  | 17 | 39890994 | 39891458 | PDFSTATSENSEMBLUCSC | 465 | 7 | 17343 | tss;island | 0.157808 | 0.024316 | Not queried | 1.44 | 2.09e-01 | 2.65e-01 |
|  | 6 | 20401608 | 20402153 | PDFSTATSENSEMBLUCSC | 546 | 11 | 35484 | island | 0.004675 | 0.009729 | Not queried | 1.44 | 2.43e-01 | 2.98e-01 |
|  | 6 | 3118573 | 3118938 | PDFSTATSENSEMBLUCSC | 366 | 8 | 35382 | island | 0.003170 | 0.006564 | Not queried | 1.44 | 1.30e-01 | 1.89e-01 |
|  | 11 | 330872 | 331179 | PDFSTATSENSEMBLUCSC | 308 | 4 | 7843 | island | 0.946082 | 0.983471 | Not queried | 1.44 | 3.43e-01 | 3.84e-01 |
|  | 19 | 11465809 | 11466145 | PDFSTATSENSEMBLUCSC | 337 | 5 | 21563 | island | 0.016969 | 0.028100 | Not queried | 1.44 | 5.48e-01 | 5.70e-01 |
|  | 8 | 11986813 | 11986933 | PDFSTATSENSEMBLUCSC | 121 | 4 | 39209 | gene;tss | 0.484143 | 0.646236 | Not queried | 1.44 | 3.43e-01 | 3.84e-01 |
|  | 6 | 31845413 | 31845879 | PDFSTATSENSEMBLUCSC | 467 | 6 | 33979 | gene | 0.600919 | 0.490374 | Not queried | 1.44 | 5.89e-01 | 6.11e-01 |
|  | 19 | 56739573 | 56739868 | PDFSTATSENSEMBLUCSC | 296 | 7 | 22441 | island | 0.946041 | 0.980968 | Not queried | 1.44 | 2.62e-02 | 6.12e-02 |
|  | 17 | 40428759 | 40428970 | PDFSTATSENSEMBLUCSC | 212 | 4 | 17360 | tss;island | 0.015354 | 0.044566 | Not queried | 1.44 | 4.86e-01 | 5.11e-01 |
|  | 3 | 101443535 | 101443992 | PDFSTATSENSEMBLUCSC | 458 | 5 | 27881 | gene | 0.001850 | 0.007744 | Not queried | 1.44 | 4.21e-01 | 4.59e-01 |
|  | 5 | 33936171 | 33936752 | PDFSTATSENSEMBLUCSC | 582 | 13 | 32723 | island | 0.840452 | 0.605810 | Not queried | 1.44 | 5.68e-02 | 1.00e-01 |
|  | 2 | 105858730 | 105859083 | PDFSTATSENSEMBLUCSC | 354 | 4 | 22813 | gene;island | 0.779296 | 0.937454 | Not queried | 1.44 | 6.86e-01 | 6.99e-01 |
|  | 13 | 31479935 | 31480304 | PDFSTATSENSEMBLUCSC | 370 | 6 | 11376 | tss | 0.458845 | 0.623494 | Not queried | 1.44 | 3.94e-01 | 4.36e-01 |
|  | 7 | 28220498 | 28220645 | PDFSTATSENSEMBLUCSC | 148 | 8 | 37503 | tss;island | 0.042547 | 0.166574 | Not queried | 1.44 | 3.28e-01 | 3.84e-01 |
|  | 17 | 37821447 | 37821536 | PDFSTATSENSEMBLUCSC | 90 | 4 | 17312 | tss | 0.815790 | 0.918144 | Not queried | 1.44 | 5.71e-02 | 1.00e-01 |
|  | 19 | 56347722 | 56347927 | PDFSTATSENSEMBLUCSC | 206 | 5 | 20234 | gene;tss | 0.708123 | 0.922232 | Not queried | 1.44 | 8.41e-01 | 8.48e-01 |
|  | 7 | 158379132 | 158379348 | PDFSTATSENSEMBLUCSC | 217 | 4 | 37379 | gene;island | 0.860837 | 0.974159 | Not queried | 1.44 | 1.14e-01 | 1.68e-01 |
|  | 8 | 80942618 | 80942820 | PDFSTATSENSEMBLUCSC | 203 | 5 | 39781 | tss;island | 0.000693 | 0.002311 | Not queried | 1.44 | 3.10e-01 | 3.66e-01 |
|  | 14 | 51134997 | 51135466 | PDFSTATSENSEMBLUCSC | 470 | 8 | 12833 | island | 0.003324 | 0.011199 | Not queried | 1.44 | 1.95e-01 | 2.55e-01 |
|  | 4 | 20702045 | 20702377 | PDFSTATSENSEMBLUCSC | 333 | 4 | 29964 | gene | 0.002368 | 0.005489 | Not queried | 1.44 | 6.86e-01 | 6.99e-01 |
|  | 17 | 78074910 | 78074979 | PDFSTATSENSEMBLUCSC | 70 | 4 | 17719 | tss;island | 0.004878 | 0.004761 | Not queried | 1.44 | 4.86e-01 | 5.11e-01 |
|  | 18 | 24765473 | 24765781 | PDFSTATSENSEMBLUCSC | 309 | 5 | 19219 | tss;island | 0.682842 | 0.424222 | Not queried | 1.44 | 2.22e-01 | 2.76e-01 |
|  | 3 | 11314015 | 11314335 | PDFSTATSENSEMBLUCSC | 321 | 4 | 27667 | gene | 0.002471 | 0.009773 | Not queried | 1.44 | 3.43e-01 | 3.84e-01 |
|  | 10 | 72163475 | 72163814 | PDFSTATSENSEMBLUCSC | 340 | 5 | 5134 | tss | 0.012166 | 0.009896 | Not queried | 1.44 | 6.90e-01 | 7.01e-01 |
|  | 10 | 31609200 | 31609531 | PDFSTATSENSEMBLUCSC | 332 | 4 | 4553 | gene;tss;island | 0.277835 | 0.442358 | Not queried | 1.44 | 4.86e-01 | 5.11e-01 |
|  | 4 | 37828046 | 37828114 | PDFSTATSENSEMBLUCSC | 69 | 5 | 30343 | tss;island | 0.004704 | 0.009699 | Not queried | 1.44 | 1.51e-01 | 2.11e-01 |
|  | 12 | 56511647 | 56511953 | PDFSTATSENSEMBLUCSC | 307 | 8 | 9784 | tss | 0.004145 | 0.007093 | RPL41; ZC3H10; ESYT1; RP11-603J24.6; RP11-603J24.5 (margin: RP11-603J24.9; PA2G4; RPL41; ZC3H10; ESYT1; RP11-603J24.17; RP11-603J24.6; RP11-603J24.5) | 1.44 | 4.66e-03 | 2.68e-02 |
|  | 16 | 89686618 | 89687052 | PDFSTATSENSEMBLUCSC | 435 | 5 | 14947 | gene | 0.397790 | 0.715865 | Not queried | 1.44 | 5.56e-02 | 1.00e-01 |
|  | 6 | 31628639 | 31629199 | PDFSTATSENSEMBLUCSC | 561 | 10 | 34826 | tss | 0.007889 | 0.034044 | Not queried | 1.44 | 3.93e-01 | 4.36e-01 |
|  | 6 | 32178017 | 32178298 | PDFSTATSENSEMBLUCSC | 282 | 5 | 34090 | gene | 0.863788 | 0.870567 | Not queried | 1.44 | 5.48e-01 | 5.70e-01 |
|  | 19 | 47968997 | 47969574 | PDFSTATSENSEMBLUCSC | 578 | 6 | 20116 | gene;island | 0.737361 | 0.882024 | Not queried | 1.44 | 6.49e-02 | 1.11e-01 |
|  | 16 | 81040594 | 81040735 | PDFSTATSENSEMBLUCSC | 142 | 6 | 14853 | gene;tss;island | 0.005232 | 0.017072 | Not queried | 1.44 | 4.11e-02 | 8.14e-02 |
|  | 3 | 101498198 | 101498279 | PDFSTATSENSEMBLUCSC | 82 | 4 | 28480 | tss | 0.002150 | 0.004330 | Not queried | 1.44 | 2.00e-01 | 2.55e-01 |
|  | 13 | 38923912 | 38924138 | PDFSTATSENSEMBLUCSC | 227 | 5 | 11679 | island | 0.002986 | 0.002479 | Not queried | 1.44 | 5.48e-01 | 5.70e-01 |
|  | 1 | 160312929 | 160313057 | PDFSTATSENSEMBLUCSC | 129 | 5 | 2038 | tss | 0.008011 | 0.004404 | Not queried | 1.43 | 2.22e-01 | 2.76e-01 |
|  | 3 | 128967945 | 128968412 | PDFSTATSENSEMBLUCSC | 468 | 8 | 28580 | tss | 0.017903 | 0.010378 | Not queried | 1.43 | 1.30e-01 | 1.89e-01 |
|  | 19 | 12035674 | 12035877 | PDFSTATSENSEMBLUCSC | 204 | 5 | 20485 | tss | 0.037912 | 0.054994 | Not queried | 1.43 | 3.10e-01 | 3.66e-01 |
|  | 12 | 113229582 | 113230065 | PDFSTATSENSEMBLUCSC | 484 | 8 | 9317 | gene | 0.521734 | 0.232473 | Not queried | 1.43 | 4.42e-01 | 4.81e-01 |
|  | 19 | 42463610 | 42463824 | PDFSTATSENSEMBLUCSC | 215 | 5 | 20817 | tss | 0.005102 | 0.004963 | Not queried | 1.43 | 6.90e-01 | 7.01e-01 |
|  | 2 | 73298805 | 73299297 | PDFSTATSENSEMBLUCSC | 493 | 10 | 24409 | island | 0.007195 | 0.014326 | Not queried | 1.43 | 4.81e-01 | 5.11e-01 |
|  | 2 | 27665507 | 27665711 | PDFSTATSENSEMBLUCSC | 205 | 4 | 22630 | gene;island | 0.313732 | 0.144482 | Not queried | 1.43 | 3.43e-01 | 3.84e-01 |
|  | 2 | 45870771 | 45871073 | PDFSTATSENSEMBLUCSC | 303 | 4 | 24272 | island | 0.036585 | 0.121820 | Not queried | 1.43 | 3.43e-01 | 3.84e-01 |
|  | 7 | 100472263 | 100472765 | PDFSTATSENSEMBLUCSC | 503 | 7 | 38781 | island | 0.002348 | 0.003511 | Not queried | 1.43 | 6.20e-01 | 6.43e-01 |
|  | 7 | 100472263 | 100472566 | PDFSTATSENSEMBLUCSC | 304 | 5 | 37793 | tss | 0.005764 | 0.006523 | Not queried | 1.43 | 1.00e+00 | 1.00e+00 |
|  | 19 | 54466297 | 54466724 | PDFSTATSENSEMBLUCSC | 428 | 5 | 20212 | gene | 0.794886 | 0.963785 | Not queried | 1.43 | 8.41e-01 | 8.48e-01 |
|  | 10 | 127584967 | 127585090 | PDFSTATSENSEMBLUCSC | 124 | 4 | 5432 | tss | 0.261731 | 0.071888 | Not queried | 1.43 | 6.86e-01 | 6.99e-01 |
|  | 2 | 11796786 | 11797017 | PDFSTATSENSEMBLUCSC | 232 | 4 | 24081 | island | 0.056910 | 0.103583 | Not queried | 1.43 | 4.86e-01 | 5.11e-01 |
|  | 12 | 2921865 | 2922039 | PDFSTATSENSEMBLUCSC | 175 | 4 | 9008 | gene | 0.000843 | 0.002211 | Not queried | 1.43 | 4.86e-01 | 5.11e-01 |
|  | 20 | 61885850 | 61886313 | PDFSTATSENSEMBLUCSC | 464 | 7 | 25500 | gene;island | 0.937727 | 0.944110 | Not queried | 1.43 | 2.59e-01 | 3.16e-01 |
|  | 1 | 53905709 | 53905934 | PDFSTATSENSEMBLUCSC | 226 | 5 | 1638 | tss | 0.937555 | 0.971657 | Not queried | 1.43 | 6.90e-01 | 7.01e-01 |
|  | 2 | 51254021 | 51254170 | PDFSTATSENSEMBLUCSC | 150 | 4 | 22677 | gene;island | 0.273846 | 0.355217 | Not queried | 1.43 | 3.43e-01 | 3.84e-01 |
|  | 18 | 24128506 | 24128625 | PDFSTATSENSEMBLUCSC | 120 | 4 | 19217 | tss | 0.003005 | 0.010048 | Not queried | 1.43 | 5.71e-02 | 1.00e-01 |
|  | 14 | 23315028 | 23315264 | PDFSTATSENSEMBLUCSC | 237 | 4 | 12017 | gene;island | 0.981022 | 0.926120 | Not queried | 1.43 | 4.86e-01 | 5.11e-01 |
|  | 12 | 105379893 | 105379979 | PDFSTATSENSEMBLUCSC | 87 | 4 | 9961 | tss | 0.001592 | 0.004307 | Not queried | 1.43 | 4.86e-01 | 5.11e-01 |
|  | 2 | 219920195 | 219920517 | PDFSTATSENSEMBLUCSC | 323 | 4 | 23024 | gene;island | 0.696880 | 0.871209 | Not queried | 1.43 | 4.86e-01 | 5.11e-01 |
|  | 6 | 31543540 | 31543686 | PDFSTATSENSEMBLUCSC | 147 | 8 | 33902 | gene | 0.714859 | 0.808773 | Not queried | 1.43 | 3.79e-02 | 7.66e-02 |
|  | 14 | 70014548 | 70014974 | PDFSTATSENSEMBLUCSC | 427 | 4 | 12969 | island | 0.706474 | 0.912658 | Not queried | 1.43 | 3.43e-01 | 3.84e-01 |
|  | 12 | 63195695 | 63195954 | PDFSTATSENSEMBLUCSC | 260 | 4 | 9200 | gene;island | 0.962591 | 0.977656 | Not queried | 1.43 | 2.00e-01 | 2.55e-01 |
|  | 3 | 15642818 | 15643125 | PDFSTATSENSEMBLUCSC | 308 | 8 | 27693 | gene | 0.004232 | 0.010712 | Not queried | 1.43 | 3.28e-01 | 3.84e-01 |
|  | 15 | 90808551 | 90808880 | PDFSTATSENSEMBLUCSC | 330 | 7 | 13899 | tss | 0.002125 | 0.005002 | Not queried | 1.43 | 7.10e-01 | 7.20e-01 |
|  | 3 | 50273532 | 50273895 | PDFSTATSENSEMBLUCSC | 364 | 4 | 29141 | island | 0.006814 | 0.007640 | Not queried | 1.43 | 2.00e-01 | 2.55e-01 |
|  | 6 | 11094066 | 11094291 | PDFSTATSENSEMBLUCSC | 226 | 7 | 35444 | island | 0.007209 | 0.008876 | Not queried | 1.43 | 3.83e-01 | 4.27e-01 |
|  | 4 | 111397332 | 111398146 | PDFSTATSENSEMBLUCSC | 815 | 8 | 30093 | gene | 0.783155 | 0.863756 | Not queried | 1.43 | 7.98e-01 | 8.08e-01 |
|  | 10 | 29834018 | 29834266 | PDFSTATSENSEMBLUCSC | 249 | 4 | 4547 | gene | 0.984759 | 0.951343 | Not queried | 1.43 | 4.86e-01 | 5.11e-01 |
|  | 2 | 155554688 | 155555414 | PDFSTATSENSEMBLUCSC | 727 | 9 | 24797 | island | 0.659901 | 0.807683 | Not queried | 1.43 | 3.40e-01 | 3.84e-01 |
|  | 10 | 1444456 | 1444808 | PDFSTATSENSEMBLUCSC | 353 | 6 | 4463 | gene | 0.915128 | 0.965200 | Not queried | 1.43 | 4.85e-01 | 5.11e-01 |
|  | 11 | 15959856 | 15960076 | PDFSTATSENSEMBLUCSC | 221 | 4 | 8085 | island | 0.872488 | 0.950940 | Not queried | 1.43 | 3.43e-01 | 3.84e-01 |
|  | 10 | 45719751 | 45720134 | PDFSTATSENSEMBLUCSC | 384 | 5 | 5711 | island | 0.945274 | 0.971226 | Not queried | 1.43 | 2.22e-01 | 2.76e-01 |
|  | 8 | 143695822 | 143696144 | PDFSTATSENSEMBLUCSC | 323 | 7 | 40638 | island | 0.071766 | 0.018227 | Not queried | 1.43 | 1.65e-01 | 2.28e-01 |
|  | 1 | 202927535 | 202927977 | PDFSTATSENSEMBLUCSC | 443 | 5 | 2186 | tss | 0.022933 | 0.013856 | Not queried | 1.43 | 6.90e-01 | 7.01e-01 |
|  | 5 | 77146197 | 77146318 | PDFSTATSENSEMBLUCSC | 122 | 4 | 32906 | island | 0.970736 | 0.982448 | Not queried | 1.43 | 6.86e-01 | 6.99e-01 |
|  | 11 | 119187849 | 119187879 | PDFSTATSENSEMBLUCSC | 31 | 4 | 7753 | tss | 0.117311 | 0.028780 | Not queried | 1.43 | 6.86e-01 | 6.99e-01 |
|  | 7 | 83824255 | 83824481 | PDFSTATSENSEMBLUCSC | 227 | 7 | 37679 | tss | 0.014877 | 0.100027 | SEMA3A (margin: SEMA3A) | 1.43 | 4.08e-03 | 2.47e-02 |
|  | 6 | 32064491 | 32065211 | PDFSTATSENSEMBLUCSC | 721 | 29 | 34047 | gene;island | 0.809616 | 0.892447 | TNXB; ATF6B (margin: TNXB; ATF6B) | 1.43 | 5.87e-03 | 3.19e-02 |
|  | 3 | 87039536 | 87040082 | PDFSTATSENSEMBLUCSC | 547 | 6 | 27866 | gene | 0.099769 | 0.073201 | Not queried | 1.43 | 3.10e-01 | 3.66e-01 |
|  | 11 | 66639308 | 66639578 | PDFSTATSENSEMBLUCSC | 271 | 7 | 6741 | gene | 0.951181 | 0.912568 | Not queried | 1.43 | 1.28e-01 | 1.87e-01 |
|  | 6 | 160211355 | 160211577 | PDFSTATSENSEMBLUCSC | 223 | 6 | 35323 | tss;island | 0.003011 | 0.002822 | Not queried | 1.43 | 6.99e-01 | 7.09e-01 |
|  | 21 | 44588552 | 44589128 | PDFSTATSENSEMBLUCSC | 577 | 9 | 26574 | tss | 0.547322 | 0.791191 | Not queried | 1.43 | 5.03e-02 | 9.87e-02 |
|  | 7 | 112120730 | 112121036 | PDFSTATSENSEMBLUCSC | 307 | 7 | 37842 | tss | 0.966349 | 0.876093 | Not queried | 1.43 | 2.59e-01 | 3.16e-01 |
|  | 15 | 72612556 | 72613029 | PDFSTATSENSEMBLUCSC | 474 | 7 | 13775 | tss;island | 0.039421 | 0.020908 | Not queried | 1.43 | 9.73e-02 | 1.55e-01 |
|  | 14 | 24769112 | 24769404 | PDFSTATSENSEMBLUCSC | 293 | 7 | 12044 | gene | 0.003065 | 0.006533 | Not queried | 1.43 | 3.18e-01 | 3.74e-01 |
|  | 6 | 26103952 | 26104560 | PDFSTATSENSEMBLUCSC | 609 | 9 | 35514 | island | 0.010133 | 0.017287 | Not queried | 1.43 | 1.61e-01 | 2.25e-01 |
|  | 6 | 32814921 | 32815091 | PDFSTATSENSEMBLUCSC | 171 | 6 | 34153 | gene;island | 0.983150 | 0.960269 | Not queried | 1.43 | 2.40e-01 | 2.95e-01 |
|  | 11 | 1785407 | 1785804 | PDFSTATSENSEMBLUCSC | 398 | 11 | 7045 | tss;island | 0.026635 | 0.010576 | Not queried | 1.43 | 4.78e-01 | 5.11e-01 |
|  | 6 | 110299835 | 110300233 | PDFSTATSENSEMBLUCSC | 399 | 7 | 35166 | tss | 0.715066 | 0.338576 | Not queried | 1.43 | 7.10e-01 | 7.20e-01 |
|  | 16 | 2059559 | 2060056 | PDFSTATSENSEMBLUCSC | 498 | 7 | 15581 | island | 0.001365 | 0.002749 | Not queried | 1.43 | 1.65e-01 | 2.28e-01 |
|  | 18 | 3411743 | 3411996 | PDFSTATSENSEMBLUCSC | 254 | 4 | 19162 | tss | 0.680071 | 0.867504 | Not queried | 1.43 | 4.86e-01 | 5.11e-01 |
|  | 2 | 47403888 | 47404009 | PDFSTATSENSEMBLUCSC | 122 | 6 | 23342 | tss | 0.002854 | 0.006901 | Not queried | 1.43 | 3.10e-01 | 3.66e-01 |
|  | 17 | 32483560 | 32484259 | PDFSTATSENSEMBLUCSC | 700 | 13 | 18200 | island | 0.399099 | 0.656245 | Not queried | 1.43 | 2.95e-02 | 6.30e-02 |
|  | 17 | 32483882 | 32484259 | PDFSTATSENSEMBLUCSC | 378 | 10 | 17258 | tss | 0.447078 | 0.659902 | Not queried | 1.43 | 1.43e-01 | 2.04e-01 |
|  | 14 | 50863043 | 50863307 | PDFSTATSENSEMBLUCSC | 265 | 4 | 12387 | tss;island | 0.002466 | 0.019982 | Not queried | 1.43 | 4.86e-01 | 5.11e-01 |
|  | 15 | 64679818 | 64680295 | PDFSTATSENSEMBLUCSC | 478 | 6 | 14215 | island | 0.001035 | 0.009412 | Not queried | 1.43 | 3.94e-01 | 4.36e-01 |
|  | 5 | 600817 | 601208 | PDFSTATSENSEMBLUCSC | 392 | 5 | 32590 | island | 0.824224 | 0.973809 | Not queried | 1.43 | 3.10e-01 | 3.66e-01 |
|  | 11 | 110167556 | 110167871 | PDFSTATSENSEMBLUCSC | 316 | 11 | 7677 | tss;island | 0.004226 | 0.005075 | Not queried | 1.43 | 1.93e-01 | 2.55e-01 |
|  | 6 | 167051518 | 167051781 | PDFSTATSENSEMBLUCSC | 264 | 5 | 34552 | gene | 0.730964 | 0.959846 | Not queried | 1.43 | 3.10e-01 | 3.66e-01 |
|  | 17 | 48042605 | 48043063 | PDFSTATSENSEMBLUCSC | 459 | 4 | 18533 | island | 0.937406 | 0.976569 | Not queried | 1.43 | 2.00e-01 | 2.55e-01 |
|  | 6 | 31545252 | 31545473 | PDFSTATSENSEMBLUCSC | 222 | 5 | 33903 | gene;island | 0.860512 | 0.872620 | Not queried | 1.43 | 4.21e-01 | 4.59e-01 |
|  | 16 | 22217246 | 22217694 | PDFSTATSENSEMBLUCSC | 449 | 10 | 15753 | island | 0.010698 | 0.023972 | Not queried | 1.43 | 1.05e-01 | 1.66e-01 |
|  | 14 | 64805389 | 64805944 | PDFSTATSENSEMBLUCSC | 556 | 5 | 12453 | tss;island | 0.014114 | 0.007675 | Not queried | 1.42 | 5.48e-01 | 5.70e-01 |
|  | 3 | 129261768 | 129262055 | PDFSTATSENSEMBLUCSC | 288 | 4 | 28587 | tss | 0.642549 | 0.863041 | Not queried | 1.42 | 2.00e-01 | 2.55e-01 |
|  | 12 | 89748726 | 89749142 | PDFSTATSENSEMBLUCSC | 417 | 9 | 10666 | island | 0.831324 | 0.976583 | Not queried | 1.42 | 3.15e-02 | 6.50e-02 |
|  | 8 | 19796299 | 19796591 | PDFSTATSENSEMBLUCSC | 293 | 6 | 40114 | island | 0.123288 | 0.449386 | Not queried | 1.42 | 1.80e-01 | 2.45e-01 |
|  | 12 | 48722474 | 48722754 | PDFSTATSENSEMBLUCSC | 281 | 4 | 9670 | tss;island | 0.684682 | 0.913970 | Not queried | 1.42 | 2.00e-01 | 2.55e-01 |
|  | 12 | 107168153 | 107168633 | PDFSTATSENSEMBLUCSC | 481 | 5 | 10755 | island | 0.003596 | 0.002576 | Not queried | 1.42 | 1.00e+00 | 1.00e+00 |
|  | 1 | 91301461 | 91301731 | PDFSTATSENSEMBLUCSC | 271 | 4 | 3467 | island | 0.969042 | 0.987803 | Not queried | 1.42 | 1.14e-01 | 1.68e-01 |
|  | 15 | 75073839 | 75074068 | PDFSTATSENSEMBLUCSC | 230 | 4 | 13801 | tss;island | 0.350093 | 0.136332 | Not queried | 1.42 | 4.86e-01 | 5.11e-01 |
|  | 12 | 66524311 | 66524943 | PDFSTATSENSEMBLUCSC | 633 | 10 | 10602 | island | 0.003943 | 0.009354 | Not queried | 1.42 | 3.93e-01 | 4.36e-01 |
|  | 2 | 27665017 | 27665306 | PDFSTATSENSEMBLUCSC | 290 | 7 | 24185 | island | 0.174255 | 0.069187 | Not queried | 1.42 | 5.35e-01 | 5.62e-01 |
|  | 7 | 100209537 | 100210343 | PDFSTATSENSEMBLUCSC | 807 | 15 | 38771 | island | 0.005597 | 0.013370 | Not queried | 1.42 | 8.14e-02 | 1.38e-01 |
|  | 6 | 38607933 | 38608228 | PDFSTATSENSEMBLUCSC | 296 | 7 | 34950 | tss | 0.081491 | 0.082340 | Not queried | 1.42 | 5.35e-01 | 5.62e-01 |
|  | 14 | 105190158 | 105190590 | PDFSTATSENSEMBLUCSC | 433 | 6 | 13216 | island | 0.004432 | 0.011683 | Not queried | 1.42 | 9.31e-02 | 1.53e-01 |
|  | 6 | 28220064 | 28220258 | PDFSTATSENSEMBLUCSC | 195 | 6 | 34737 | tss;island | 0.002470 | 0.009982 | Not queried | 1.42 | 2.40e-01 | 2.95e-01 |
|  | 14 | 23057890 | 23058425 | PDFSTATSENSEMBLUCSC | 536 | 8 | 12698 | island | 0.002171 | 0.004975 | Not queried | 1.42 | 5.05e-01 | 5.32e-01 |
|  | 20 | 30795553 | 30795610 | PDFSTATSENSEMBLUCSC | 58 | 6 | 25673 | tss;island | 0.004629 | 0.002672 | Not queried | 1.42 | 3.94e-01 | 4.36e-01 |
|  | 10 | 60094511 | 60094732 | PDFSTATSENSEMBLUCSC | 222 | 6 | 5090 | tss | 0.009370 | 0.016094 | Not queried | 1.42 | 5.89e-01 | 6.11e-01 |
|  | 7 | 140714412 | 140715277 | PDFSTATSENSEMBLUCSC | 866 | 15 | 38966 | island | 0.014721 | 0.005428 | Not queried | 1.42 | 4.53e-02 | 8.95e-02 |
|  | 1 | 169863438 | 169863532 | PDFSTATSENSEMBLUCSC | 95 | 4 | 2088 | tss;island | 0.004491 | 0.007471 | Not queried | 1.42 | 6.86e-01 | 6.99e-01 |
|  | 16 | 68278871 | 68279613 | PDFSTATSENSEMBLUCSC | 743 | 10 | 16042 | island | 0.005425 | 0.011268 | Not queried | 1.42 | 1.43e-01 | 2.04e-01 |
|  | 11 | 32449163 | 32450244 | PDFSTATSENSEMBLUCSC | 1082 | 8 | 6565 | gene;island | 0.892452 | 0.931574 | Not queried | 1.42 | 1.30e-01 | 1.89e-01 |
|  | 2 | 95537044 | 95537210 | PDFSTATSENSEMBLUCSC | 167 | 5 | 23506 | tss | 0.559444 | 0.415981 | Not queried | 1.42 | 6.90e-01 | 7.01e-01 |
|  | 17 | 7218821 | 7219025 | PDFSTATSENSEMBLUCSC | 205 | 6 | 17074 | tss | 0.020194 | 0.065104 | Not queried | 1.42 | 4.11e-02 | 8.14e-02 |
|  | 19 | 6481819 | 6482169 | PDFSTATSENSEMBLUCSC | 351 | 8 | 20391 | tss | 0.749700 | 0.891795 | Not queried | 1.42 | 6.50e-02 | 1.11e-01 |
|  | 22 | 38794898 | 38795061 | PDFSTATSENSEMBLUCSC | 164 | 8 | 27487 | island | 0.007845 | 0.021366 | Not queried | 1.42 | 2.79e-01 | 3.38e-01 |
|  | 22 | 38794936 | 38795061 | PDFSTATSENSEMBLUCSC | 126 | 6 | 27138 | tss | 0.007578 | 0.024876 | Not queried | 1.42 | 4.85e-01 | 5.11e-01 |
|  | 5 | 169659618 | 169659950 | PDFSTATSENSEMBLUCSC | 333 | 10 | 33377 | island | 0.911544 | 0.978889 | C5orf58 (margin: C5orf58) | 1.42 | 1.15e-02 | 4.29e-02 |
|  | 11 | 71639070 | 71640085 | PDFSTATSENSEMBLUCSC | 1016 | 15 | 7533 | tss | 0.003533 | 0.004704 | Not queried | 1.42 | 1.87e-01 | 2.55e-01 |
|  | 1 | 146644318 | 146644500 | PDFSTATSENSEMBLUCSC | 183 | 6 | 1892 | tss;island | 0.009417 | 0.016318 | Not queried | 1.42 | 2.40e-01 | 2.95e-01 |
|  | 3 | 15642818 | 15643321 | PDFSTATSENSEMBLUCSC | 504 | 13 | 28175 | tss;island | 0.004381 | 0.009608 | Not queried | 1.42 | 3.36e-01 | 3.84e-01 |
|  | 13 | 95655083 | 95655579 | PDFSTATSENSEMBLUCSC | 497 | 5 | 11824 | island | 0.930946 | 0.970803 | Not queried | 1.42 | 1.51e-01 | 2.11e-01 |
|  | 2 | 46707626 | 46707844 | PDFSTATSENSEMBLUCSC | 219 | 4 | 22667 | gene;island | 0.959932 | 0.929049 | Not queried | 1.42 | 3.43e-01 | 3.84e-01 |
|  | 8 | 59323544 | 59323842 | PDFSTATSENSEMBLUCSC | 299 | 4 | 40322 | island | 0.022373 | 0.035377 | Not queried | 1.42 | 6.86e-01 | 6.99e-01 |
|  | 17 | 48171802 | 48172203 | PDFSTATSENSEMBLUCSC | 402 | 7 | 17491 | tss;island | 0.004083 | 0.008736 | Not queried | 1.42 | 9.73e-02 | 1.55e-01 |
|  | 11 | 66488919 | 66489022 | PDFSTATSENSEMBLUCSC | 104 | 4 | 7479 | tss | 0.958194 | 0.857630 | Not queried | 1.42 | 3.43e-01 | 3.84e-01 |
|  | 12 | 56101328 | 56102252 | PDFSTATSENSEMBLUCSC | 925 | 11 | 9167 | gene;island | 0.135222 | 0.014966 | Not queried | 1.42 | 2.81e-02 | 6.12e-02 |
|  | 19 | 36365514 | 36365800 | PDFSTATSENSEMBLUCSC | 287 | 4 | 19995 | gene;island | 0.805202 | 0.893496 | Not queried | 1.42 | 4.86e-01 | 5.11e-01 |
|  | 6 | 21593717 | 21594038 | PDFSTATSENSEMBLUCSC | 322 | 9 | 35485 | island | 0.080800 | 0.012556 | Not queried | 1.42 | 3.15e-02 | 6.50e-02 |
|  | 2 | 74681787 | 74682200 | PDFSTATSENSEMBLUCSC | 414 | 11 | 24433 | island | 0.006785 | 0.004274 | Not queried | 1.42 | 3.65e-01 | 4.09e-01 |
|  | 11 | 62622834 | 62622991 | PDFSTATSENSEMBLUCSC | 158 | 7 | 6650 | gene;tss;island | 0.000476 | 0.003636 | Not queried | 1.42 | 7.10e-01 | 7.20e-01 |
|  | 11 | 73588074 | 73588532 | PDFSTATSENSEMBLUCSC | 459 | 5 | 6824 | gene | 0.009801 | 0.015572 | Not queried | 1.42 | 3.10e-01 | 3.66e-01 |
|  | 11 | 118436523 | 118436778 | PDFSTATSENSEMBLUCSC | 256 | 8 | 6930 | gene | 0.003216 | 0.004547 | Not queried | 1.42 | 4.99e-02 | 9.81e-02 |
|  | 4 | 141264393 | 141264721 | PDFSTATSENSEMBLUCSC | 329 | 5 | 30136 | gene | 0.075001 | 0.151878 | Not queried | 1.42 | 5.48e-01 | 5.70e-01 |
|  | 1 | 17338409 | 17338766 | PDFSTATSENSEMBLUCSC | 358 | 7 | 2754 | island | 0.054861 | 0.030103 | Not queried | 1.42 | 1.00e+00 | 1.00e+00 |
|  | 14 | 50319474 | 50319966 | PDFSTATSENSEMBLUCSC | 493 | 11 | 12825 | island | 0.002282 | 0.002206 | Not queried | 1.42 | 1.00e+00 | 1.00e+00 |
|  | 5 | 94955575 | 94956175 | PDFSTATSENSEMBLUCSC | 601 | 8 | 32969 | island | 0.959027 | 0.984183 | Not queried | 1.42 | 6.50e-02 | 1.11e-01 |
|  | 17 | 73775869 | 73776120 | PDFSTATSENSEMBLUCSC | 252 | 9 | 17661 | tss | 0.001347 | 0.002325 | Not queried | 1.42 | 2.97e-01 | 3.60e-01 |
|  | 6 | 123317124 | 123317569 | PDFSTATSENSEMBLUCSC | 446 | 8 | 35207 | tss | 0.802116 | 0.964803 | Not queried | 1.42 | 2.79e-01 | 3.38e-01 |
|  | 20 | 60119508 | 60119664 | PDFSTATSENSEMBLUCSC | 157 | 4 | 25481 | gene;island | 0.460642 | 0.585159 | Not queried | 1.42 | 6.86e-01 | 6.99e-01 |
|  | 22 | 42469976 | 42470223 | PDFSTATSENSEMBLUCSC | 248 | 6 | 27187 | tss;island | 0.783864 | 0.829373 | Not queried | 1.42 | 8.18e-01 | 8.27e-01 |
|  | 5 | 43602595 | 43602919 | PDFSTATSENSEMBLUCSC | 325 | 5 | 32050 | tss;island | 0.002772 | 0.007359 | Not queried | 1.42 | 9.52e-02 | 1.53e-01 |
|  | 4 | 150999899 | 151000014 | PDFSTATSENSEMBLUCSC | 116 | 6 | 30631 | tss | 0.014000 | 0.004194 | Not queried | 1.42 | 3.94e-01 | 4.36e-01 |
|  | 7 | 86781331 | 86781902 | PDFSTATSENSEMBLUCSC | 572 | 11 | 38630 | island | 0.013340 | 0.019124 | Not queried | 1.42 | 8.79e-02 | 1.48e-01 |
|  | 7 | 1269693 | 1270134 | PDFSTATSENSEMBLUCSC | 442 | 4 | 38090 | island | 0.555756 | 0.769410 | Not queried | 1.42 | 8.86e-01 | 8.89e-01 |
|  | 7 | 2968316 | 2968627 | PDFSTATSENSEMBLUCSC | 312 | 5 | 36820 | gene;island | 0.882711 | 0.835839 | Not queried | 1.42 | 6.90e-01 | 7.01e-01 |
|  | 10 | 118380175 | 118380396 | PDFSTATSENSEMBLUCSC | 222 | 4 | 5385 | tss | 0.932753 | 0.981057 | Not queried | 1.42 | 3.43e-01 | 3.84e-01 |
|  | 6 | 136871667 | 136872119 | PDFSTATSENSEMBLUCSC | 453 | 10 | 36501 | island | 0.025467 | 0.005458 | Not queried | 1.42 | 5.24e-02 | 1.00e-01 |
|  | 16 | 30569726 | 30569978 | PDFSTATSENSEMBLUCSC | 253 | 8 | 15187 | tss;island | 0.004633 | 0.014838 | Not queried | 1.41 | 2.79e-01 | 3.38e-01 |
|  | 3 | 176915062 | 176915282 | PDFSTATSENSEMBLUCSC | 221 | 4 | 28724 | tss;island | 0.049858 | 0.067890 | Not queried | 1.41 | 4.86e-01 | 5.11e-01 |
|  | 3 | 6902824 | 6903327 | PDFSTATSENSEMBLUCSC | 504 | 5 | 27645 | gene | 0.874736 | 0.825271 | Not queried | 1.41 | 2.22e-01 | 2.76e-01 |
|  | 14 | 102027514 | 102027797 | PDFSTATSENSEMBLUCSC | 284 | 6 | 12636 | tss;island | 0.686037 | 0.454886 | DIO3; DIO3OS (margin: DIO3; DIO3OS) | 1.41 | 2.16e-03 | 1.45e-02 |
|  | 19 | 41882232 | 41882327 | PDFSTATSENSEMBLUCSC | 96 | 4 | 20059 | gene;tss;island | 0.532251 | 0.295046 | Not queried | 1.41 | 3.43e-01 | 3.84e-01 |
|  | 12 | 69080752 | 69081084 | PDFSTATSENSEMBLUCSC | 333 | 4 | 9209 | gene | 0.003041 | 0.005667 | Not queried | 1.41 | 1.14e-01 | 1.68e-01 |
|  | 10 | 72200612 | 72201008 | PDFSTATSENSEMBLUCSC | 397 | 4 | 4619 | gene;island | 0.905236 | 0.985963 | Not queried | 1.41 | 3.43e-01 | 3.84e-01 |
|  | 1 | 65730339 | 65730494 | PDFSTATSENSEMBLUCSC | 156 | 6 | 3353 | island | 0.276178 | 0.649463 | Not queried | 1.41 | 6.49e-02 | 1.11e-01 |
|  | 12 | 77272832 | 77273469 | PDFSTATSENSEMBLUCSC | 638 | 8 | 9887 | tss | 0.007443 | 0.009500 | Not queried | 1.41 | 1.05e-01 | 1.66e-01 |
|  | 14 | 56045827 | 56046098 | PDFSTATSENSEMBLUCSC | 272 | 4 | 12089 | gene;tss;island | 0.103777 | 0.042470 | Not queried | 1.41 | 4.86e-01 | 5.11e-01 |
|  | 7 | 48495072 | 48495333 | PDFSTATSENSEMBLUCSC | 262 | 4 | 37008 | gene;island | 0.663863 | 0.885276 | Not queried | 1.41 | 3.43e-01 | 3.84e-01 |
|  | 4 | 41646293 | 41646672 | PDFSTATSENSEMBLUCSC | 380 | 4 | 29991 | gene;island | 0.936315 | 0.955888 | Not queried | 1.41 | 4.86e-01 | 5.11e-01 |
|  | 12 | 69080414 | 69081084 | PDFSTATSENSEMBLUCSC | 671 | 11 | 10610 | island | 0.003152 | 0.006031 | Not queried | 1.41 | 1.16e-01 | 1.71e-01 |
|  | 17 | 7905636 | 7905928 | PDFSTATSENSEMBLUCSC | 293 | 4 | 17105 | tss | 0.654836 | 0.894413 | Not queried | 1.41 | 5.71e-02 | 1.00e-01 |
|  | 14 | 59655074 | 59655429 | PDFSTATSENSEMBLUCSC | 356 | 11 | 12891 | island | 0.007643 | 0.012104 | Not queried | 1.41 | 8.79e-02 | 1.48e-01 |
|  | 17 | 42081810 | 42082036 | PDFSTATSENSEMBLUCSC | 227 | 8 | 17397 | tss;island | 0.894103 | 0.955062 | Not queried | 1.41 | 6.50e-02 | 1.11e-01 |
|  | 18 | 48723744 | 48724403 | PDFSTATSENSEMBLUCSC | 660 | 9 | 19517 | island | 0.004760 | 0.009941 | Not queried | 1.41 | 4.36e-01 | 4.76e-01 |
|  | 1 | 234735904 | 234736253 | PDFSTATSENSEMBLUCSC | 350 | 4 | 4339 | island | 0.011397 | 0.056984 | Not queried | 1.41 | 8.86e-01 | 8.89e-01 |
|  | 11 | 66627194 | 66627706 | PDFSTATSENSEMBLUCSC | 513 | 5 | 6739 | gene;island | 0.741077 | 0.886301 | Not queried | 1.41 | 2.22e-01 | 2.76e-01 |
|  | 12 | 38710408 | 38710700 | PDFSTATSENSEMBLUCSC | 293 | 9 | 10324 | island | 0.004625 | 0.006003 | Not queried | 1.41 | 1.36e-01 | 1.94e-01 |
|  | 20 | 3713341 | 3713584 | PDFSTATSENSEMBLUCSC | 244 | 4 | 25973 | island | 0.881390 | 0.957281 | Not queried | 1.41 | 2.00e-01 | 2.55e-01 |
|  | 21 | 34602027 | 34602157 | PDFSTATSENSEMBLUCSC | 131 | 5 | 26518 | tss;island | 0.005508 | 0.002398 | Not queried | 1.41 | 8.41e-01 | 8.48e-01 |
|  | 2 | 86850757 | 86851071 | PDFSTATSENSEMBLUCSC | 315 | 7 | 24496 | island | 0.002370 | 0.008569 | Not queried | 1.41 | 6.20e-01 | 6.43e-01 |
|  | 18 | 56886915 | 56887410 | PDFSTATSENSEMBLUCSC | 496 | 11 | 19543 | island | 0.388792 | 0.860368 | Not queried | 1.41 | 4.38e-01 | 4.78e-01 |
|  | 1 | 160991154 | 160991224 | PDFSTATSENSEMBLUCSC | 71 | 4 | 2041 | tss | 0.002814 | 0.007366 | Not queried | 1.41 | 2.00e-01 | 2.55e-01 |
|  | 17 | 56609534 | 56609687 | PDFSTATSENSEMBLUCSC | 154 | 5 | 17538 | tss;island | 0.418260 | 0.260230 | Not queried | 1.41 | 3.10e-01 | 3.66e-01 |
|  | 2 | 127783082 | 127783370 | PDFSTATSENSEMBLUCSC | 289 | 4 | 24683 | island | 0.400400 | 0.632465 | Not queried | 1.41 | 3.43e-01 | 3.84e-01 |
|  | 5 | 137514611 | 137515009 | PDFSTATSENSEMBLUCSC | 399 | 5 | 31766 | gene;tss;island | 0.001103 | 0.004184 | Not queried | 1.41 | 1.51e-01 | 2.11e-01 |
|  | 1 | 151881785 | 151882406 | PDFSTATSENSEMBLUCSC | 622 | 7 | 3756 | island | 0.006093 | 0.016204 | Not queried | 1.41 | 3.83e-01 | 4.27e-01 |
|  | 2 | 74375133 | 74375213 | PDFSTATSENSEMBLUCSC | 81 | 6 | 23448 | tss;island | 0.005915 | 0.007489 | Not queried | 1.41 | 1.00e+00 | 1.00e+00 |
|  | 17 | 9862752 | 9863081 | PDFSTATSENSEMBLUCSC | 330 | 9 | 16439 | gene | 0.006399 | 0.014235 | Not queried | 1.41 | 1.00e+00 | 1.00e+00 |
|  | 12 | 130953464 | 130953814 | PDFSTATSENSEMBLUCSC | 351 | 8 | 9416 | gene;island | 0.896007 | 0.943992 | Not queried | 1.41 | 4.42e-01 | 4.81e-01 |
|  | 1 | 14026054 | 14026618 | PDFSTATSENSEMBLUCSC | 565 | 9 | 1263 | tss | 0.204508 | 0.023411 | Not queried | 1.41 | 3.40e-01 | 3.84e-01 |
|  | 11 | 115372874 | 115373119 | PDFSTATSENSEMBLUCSC | 246 | 4 | 6912 | gene;island | 0.776110 | 0.955380 | Not queried | 1.41 | 4.86e-01 | 5.11e-01 |
|  | 1 | 85725329 | 85725654 | PDFSTATSENSEMBLUCSC | 326 | 4 | 3422 | island | 0.011388 | 0.011093 | Not queried | 1.41 | 8.86e-01 | 8.89e-01 |
|  | 1 | 156571296 | 156571598 | PDFSTATSENSEMBLUCSC | 303 | 9 | 2019 | tss | 0.005205 | 0.011807 | Not queried | 1.41 | 1.36e-01 | 1.94e-01 |
|  | 5 | 146614298 | 146614559 | PDFSTATSENSEMBLUCSC | 262 | 9 | 32409 | tss | 0.080635 | 0.027502 | Not queried | 1.41 | 5.03e-02 | 9.87e-02 |
|  | 5 | 146614298 | 146614719 | PDFSTATSENSEMBLUCSC | 422 | 11 | 33287 | island | 0.080635 | 0.033282 | Not queried | 1.41 | 8.79e-02 | 1.48e-01 |
|  | 14 | 93651022 | 93651169 | PDFSTATSENSEMBLUCSC | 148 | 5 | 12185 | gene;tss;island | 0.006768 | 0.006928 | Not queried | 1.41 | 6.90e-01 | 7.01e-01 |
|  | 16 | 776416 | 776918 | PDFSTATSENSEMBLUCSC | 503 | 8 | 14978 | tss;island | 0.116525 | 0.046584 | Not queried | 1.41 | 3.79e-02 | 7.66e-02 |
|  | 1 | 205197460 | 205197876 | PDFSTATSENSEMBLUCSC | 417 | 4 | 965 | gene;island | 0.020260 | 0.035589 | Not queried | 1.41 | 3.43e-01 | 3.84e-01 |
|  | 16 | 3062597 | 3062975 | PDFSTATSENSEMBLUCSC | 379 | 4 | 14619 | gene | 0.564515 | 0.863463 | Not queried | 1.40 | 3.43e-01 | 3.84e-01 |
|  | 3 | 50382952 | 50383134 | PDFSTATSENSEMBLUCSC | 183 | 4 | 27807 | gene | 0.313176 | 0.544387 | Not queried | 1.40 | 3.43e-01 | 3.84e-01 |
|  | 19 | 3982603 | 3983219 | PDFSTATSENSEMBLUCSC | 617 | 9 | 20356 | tss | 0.944673 | 0.971057 | Not queried | 1.40 | 7.70e-02 | 1.30e-01 |
|  | 12 | 117484306 | 117484586 | PDFSTATSENSEMBLUCSC | 281 | 4 | 9335 | gene;island | 0.635029 | 0.817275 | Not queried | 1.40 | 4.86e-01 | 5.11e-01 |
|  | 7 | 87936225 | 87936414 | PDFSTATSENSEMBLUCSC | 190 | 5 | 37691 | tss | 0.152422 | 0.310262 | Not queried | 1.40 | 3.17e-02 | 6.50e-02 |
|  | 4 | 164394686 | 164395287 | PDFSTATSENSEMBLUCSC | 602 | 8 | 31402 | island | 0.967994 | 0.976326 | Not queried | 1.40 | 2.34e-01 | 2.90e-01 |
|  | 11 | 102323937 | 102324112 | PDFSTATSENSEMBLUCSC | 176 | 4 | 7649 | tss;island | 0.012640 | 0.026539 | Not queried | 1.40 | 4.86e-01 | 5.11e-01 |
|  | 11 | 118868622 | 118868795 | PDFSTATSENSEMBLUCSC | 174 | 8 | 7743 | tss | 0.002085 | 0.004955 | Not queried | 1.40 | 2.34e-01 | 2.90e-01 |
|  | 6 | 37673335 | 37673863 | PDFSTATSENSEMBLUCSC | 529 | 6 | 36101 | island | 0.807776 | 0.895861 | Not queried | 1.40 | 5.89e-01 | 6.11e-01 |
|  | 5 | 82767280 | 82767617 | PDFSTATSENSEMBLUCSC | 338 | 6 | 32931 | island | 0.896247 | 0.963317 | Not queried | 1.40 | 2.60e-02 | 6.12e-02 |
|  | 17 | 79876118 | 79876287 | PDFSTATSENSEMBLUCSC | 170 | 5 | 17757 | tss | 0.012748 | 0.010345 | Not queried | 1.40 | 1.00e+00 | 1.00e+00 |
|  | 2 | 177134241 | 177134497 | PDFSTATSENSEMBLUCSC | 257 | 4 | 22941 | gene | 0.004966 | 0.008591 | Not queried | 1.40 | 4.86e-01 | 5.11e-01 |
|  | 11 | 71955299 | 71955599 | PDFSTATSENSEMBLUCSC | 301 | 8 | 7540 | tss | 0.971807 | 0.985969 | Not queried | 1.40 | 2.34e-01 | 2.90e-01 |
|  | 8 | 144790317 | 144790410 | PDFSTATSENSEMBLUCSC | 94 | 4 | 39958 | tss | 0.943124 | 0.978015 | Not queried | 1.40 | 4.86e-01 | 5.11e-01 |
|  | 6 | 111888446 | 111888840 | PDFSTATSENSEMBLUCSC | 395 | 7 | 34446 | gene | 0.796031 | 0.967808 | Not queried | 1.40 | 2.09e-01 | 2.65e-01 |
|  | 17 | 6917614 | 6918055 | PDFSTATSENSEMBLUCSC | 442 | 7 | 17058 | tss | 0.002693 | 0.004095 | Not queried | 1.40 | 7.10e-01 | 7.20e-01 |
|  | 10 | 73157611 | 73158072 | PDFSTATSENSEMBLUCSC | 462 | 5 | 4621 | gene;island | 0.600023 | 0.720146 | Not queried | 1.40 | 2.22e-01 | 2.76e-01 |
|  | 11 | 6261411 | 6261798 | PDFSTATSENSEMBLUCSC | 388 | 5 | 6486 | gene;island | 0.563958 | 0.822576 | Not queried | 1.40 | 9.52e-02 | 1.53e-01 |
|  | 8 | 97657072 | 97657432 | PDFSTATSENSEMBLUCSC | 361 | 10 | 39828 | tss | 0.301878 | 0.109176 | Not queried | 1.40 | 2.47e-01 | 3.03e-01 |
|  | 7 | 960115 | 960528 | PDFSTATSENSEMBLUCSC | 414 | 5 | 36727 | gene;island | 0.929270 | 0.981490 | Not queried | 1.40 | 4.21e-01 | 4.59e-01 |
|  | 7 | 75677153 | 75677249 | PDFSTATSENSEMBLUCSC | 97 | 6 | 37049 | gene | 0.008835 | 0.021007 | Not queried | 1.40 | 1.32e-01 | 1.89e-01 |
|  | 5 | 95159365 | 95159639 | PDFSTATSENSEMBLUCSC | 275 | 4 | 32187 | tss | 0.607846 | 0.744634 | Not queried | 1.40 | 5.71e-02 | 1.00e-01 |
|  | 2 | 58468575 | 58468651 | PDFSTATSENSEMBLUCSC | 77 | 4 | 23369 | tss | 0.003625 | 0.011935 | Not queried | 1.40 | 6.86e-01 | 6.99e-01 |
|  | 14 | 36278484 | 36278684 | PDFSTATSENSEMBLUCSC | 201 | 8 | 12355 | tss;island | 0.001544 | 0.003825 | Not queried | 1.40 | 3.28e-01 | 3.84e-01 |
|  | 12 | 510474 | 510772 | PDFSTATSENSEMBLUCSC | 299 | 9 | 8993 | gene;island | 0.006788 | 0.019490 | Not queried | 1.40 | 2.58e-01 | 3.16e-01 |
|  | 3 | 45635931 | 45636225 | PDFSTATSENSEMBLUCSC | 295 | 6 | 28268 | tss | 0.033445 | 0.128628 | Not queried | 1.40 | 3.10e-01 | 3.66e-01 |
